# Molecular Subtypes Based on Cuproptosis-Related Genes and Tumor Microenvironment Infiltration Characterization in Colorectal Cancer

**DOI:** 10.1155/2022/5034092

**Published:** 2022-10-11

**Authors:** Hao Huang, Zhiping Long, Yilin Xie, Pei Qin, Lei Kuang, Xi Li, Yang Zhao, Xing Zhang, Longkun Yang, Wancheng Ma, Xiang Xiao, Yu Liu, Xizhuo Sun, Ming Zhang, Fan Wang, Dongsheng Hu, Fulan Hu

**Affiliations:** ^1^Department of General Practice, The Affiliated Luohu Hospital of Shenzhen University Health Science Center, Shenzhen, Guangdong, China; ^2^Department of Epidemiology, Public Health School of Harbin Medical University, Harbin, Heilongjiang, China; ^3^Department of Epidemiology and Health Statistics, Shenzhen University Health Science Center, Shenzhen, Guangdong, China; ^4^Department of Epidemiology and Biostatistics, College of Public Health, Zhengzhou University, Zhengzhou, Henan, China; ^5^Department of Epidemiology and Health Statistics, Fujian Provincial Key Laboratory of Environment Factors and Cancer, School of Public Health, Fujian Medical University, Fuzhou, China; ^6^Luohu Center for Chronic Disease Control, Shenzhen, China; ^7^Hunan Cancer Hospital/The Affiliated Cancer Hospital of Xiangya School of Medicine, Central South University, Changsha, China

## Abstract

Recent studies have demonstrated the biological significance of cuproptosis modification, a newly discovered programmed cell death, in tumor progression. Nonetheless, the potential role of cuproptosis-related genes (CRGs) in the immune landscape and tumor microenvironment (TME) formation of colorectal cancer (CRC) remains unknown. We comprehensively assessed cuproptosis modification patterns of 1339 CRC samples based on 27 CRGs and systematically analyzed the correlation of these patterns with TME. The CRG-score was constructed to quantify cuproptosis characteristics by LASSO and multivariate Cox regression methods, and its predictive capability was validated in an independent cohort. We identified three distinct cuproptosis modification patterns in CRC. The TME immune cell infiltration demonstrated immune heterogeneity among these three subtypes. Enrichment for multiple metabolism signatures was pronounced in cluster A. Cluster C was significantly correlated with the signaling pathways of immune activation-related, resulting in poor prognoses. Cluster B with mixed features possibly represents a transition phenotype or intratumoral heterogeneity. Then, based on constructed eight-gene CRG-score, we found that the signature could predict the disease-free survival of CRC patients, and the low CRG-score was related to increased neoantigen load, immunity activation, and microsatellite instability-high (MSI-H). Additionally, we observed significant correlations of the CRG-score with the cancer stem cell index and chemotherapeutic drug susceptibility. This study demonstrated that cuproptosis was correlated with tumor progression, prognosis, and TME. Our findings may improve the understanding of CRGs in TME infiltration characterization of CRC patients and contribute to guiding more effective clinical therapeutic strategies.

## 1. Introduction

Colorectal cancer (CRC) is the third most frequent malignancy and the second biggest cause of cancer-related mortality in the world [1], which has very limited treatment options still, despite the vast amount of research undertaken. Targeted therapies for solid tumors, such as tyrosine kinase inhibitors and immunotherapy, have shown little benefit in CRC [2] (except for BRAF V600E-mutated tumors [3]). The gene expression profiles of CRC have strong clinical relevance. The characteristics of immune cells and stromal cells infiltrating the tumor microenvironment [4, 5], combined with different subtypes of microenvironment-related signals and inherent signals of cancer cells, have always shown predictive value [6, 7]. In recent clinical trials, combining the molecular and histological characteristics of tumors to guide treatment has improved the prognosis of CRC patients [8–10]. The most widely used risk assessment tools are the current molecular subtype of consensus (CMS) and the tumor lymph node metastasis staging system (TNM). However, the current robust classification system has limitations in predicting this highly heterogeneous disease [11–13]. Therefore, more factors related to prognosis need to be considered. And the implementation of molecular classification in the clinical decision-making of CRC is crucial to solving various clinical problems in CRC progression.

Cuproptosis is a novel form of programmed cell death along with copper accumulation, protein lipoylation, and mitochondrial respiration [14]. Cuproptosis differs from other forms of cell death, such as apoptosis, necrosis, autophagy, and ferroptosis, in terms of molecular process. Copper binding causes a hazardous increase of function in lipoylated tricarboxylic acid (TCA) cycle proteins, excess copper increases lipoylated protein aggregation and instability of Fe-S cluster proteins, resulting in proteotoxic stress and cell death. As major regulators of cuproptosis, FDX1 and protein lipoylation play important roles in this process. Copper ionophores are extremely sensitive to cells conducting mitochondrial respiration, which is explained by their high quantities of lipoylated TCA enzymes. CRC tumor cells demonstrate aberrant mitochondrial metabolism as a result of active oncogene and loss of tumor suppressor gene [15], and in the tumor microenvironment (TME), aerobic glycolysis is widely observed in activated immune cells, to support biosynthetic demands [16]. The TME is now well recognized as playing a critical role in carcinogenesis and cancer development. Mitochondria serve as an intriguing target for cancer therapy since they govern the bioenergetics, biosynthesis, and signaling organelles of cancer cells [17, 18]. Cuproptosis, a kind of programmed cell death that varies from apoptosis, is likely to offer fresh hope for CRC tumor therapy. Despite this, little is known about the involvement of cuproptosis-related genes (CRGs) in prognosis and the tumor microenvironment. As a result, a thorough understanding of the features of TME immune cell infiltration mediated by numerous CRGs should help researchers better understand the underlying mechanism of CRC carcinogenesis and predict immunotherapy response.

The goal of this study is to evaluate the prognostic value and numerous functions of CRGs in the TME in a systematic way. According to The Cancer Genome Atlas (TCGA) and Gene Expression Omnibus (GEO) datasets, 1339 CRC samples were divided into three cuproptosis subtypes based on 27 CRGs, and the differences in survival and immune cell infiltration of these subtypes were explored. Patients were subsequently divided into two gene subtypes based on three cuproptosis subtypes' differentially expressed genes (DEGs). Additionally, a CRG-score was established to quantify the cuproptosis characteristics of a single tumor. The findings revealed that the CRG-score is a powerful prognostic marker.

## 2. Materials and Methods

### 2.1. Data Sources

Figure S1 in the Supplementary Material online depicts the research design used in this investigation. The public databases GEO (https://www.ncbi.nlm.nih.gov/geo/) and TCGA (https://portal.gdc.cancer.gov/) were used to get open CRC gene expression datasets with clinical information and survival outcomes. For the following analyses, a total of six datasets were obtained, containing five datasets from the GEO datasets (GSE17536, GSE39582, GSE17537, GEO161158, and GSE38832) and one from the TCGA CRC dataset (Table 1). Background correction and normalizing of CEL data received from GEO were performed using Robust Multichip Average with quantile normalization. For normalized counts, the FPKM format of RNA-seq data from the TCGA cohort was transformed to log_2_(TPM+1). GSE17536, GSE17537, GSE39582, GSE38832, and TCGA CRC datasets were pooled, and a batch correction was conducted using the ComBat method. Subsequent analyses involved a total of 1339 CRC patients. Age, sex, TNM stage, BRAF mutation, KRAS mutation, mismatch repair (MMR) status, follow-up period, and survival status were incorporated among the clinical factors. The open-source R/Bioconductor programs were used to handle and analyze all of the data.

### 2.2. Consensus Clustering and Pathway Enrichment Analysis of CRGs

Twenty-seven CRGs were retrieved from the research of Tsvetkov et al. with a false discovery rate (FDR) < 0.05 as a filter condition [14]. Tables S1 and S2 provide all of the information on these genes. Unsupervised clustering analysis was utilized to categorize CRC patients into different molecular subgroups based on CRG expression using the “ConsensusClusterPlus” package in the R studio program. A consensus clustering approach was performed to estimate the number of clusters [19]. Furthermore, the hallmark gene sets (c2.cp.kegg.v7.4.symbols) were also obtained from the Molecular Signatures Database (MSigDB) database, and the activity of signaling pathways was further accurately assessed by a gene set variation analysis (GSVA) algorithm in each subtype. The possible relationship between subtypes and disease-free survival (DFS) was investigated using Kaplan–Meier survival curves.

### 2.3. CRC Immune Landscape

CIBERSORT (https://cibersort.stanford.edu/) was used to examine the relative number of 22 immune cells in 1339 CRC samples to better understand their immunological features. We assessed the quantity of distinct immune-infiltrating cell subtypes in each sample based on the gene expression matrix of 1339 samples and the supplied gene expression feature set of 22 immune cell subtypes. The differences in the percentage of immune cells were analyzed using the Wilcoxon rank-sum test in each subtype. We also further observed the differential expression levels of *PD-1*, *PD-L1*, and *CTLA4* among these subtypes.

### 2.4. Construction and Validation of CRG-Score Based on DEGs

With a fold − change > 1.5 and an adjusted *P* value of 0.05 as filter conditions, the DEGs among the cuproptosis clusters were discovered by the limma R package. Following that, the functional annotation of the DEGs was done with the “clusterProfiler” software, and then Gene Ontology (GO) and Kyoto Encyclopedia of Genes and Genomes (KEGG) enrichment analysis were performed to clarify the biological function of these DEGs. The findings of unsupervised cluster analysis were utilized to identify possible gene categories after detecting prognosis-associated DEGs using univariate Cox regression. Finally, CRC patients were allocated in a 1 : 1 ratio to the training (*n* = 670) and testing (*n* = 669) groups, and a CRG-score related to cuproptosis was created. To reduce the overfitting of recurring characteristics and restrict the range of genes predicting DFS, LASSO Cox regression was used with the R “glmnet” package. The penalty parameter's optimal values were established using the 10-fold cross-validation approach. Following that, the CRG-score was calculated using a multivariate Cox regression analysis.

(1) $$\begin{array}{c} \text{CRG}\_\text{score}=\overset{n}{\underset{i=1}{\sum }}\text{C}\text{oefi}*\text{Expri}, \end{array}$$

where Expri expressed the signature genes and the multivariate Cox regression calculated the Coefi coefficient. The patients were separated into low CRG-score and high CRG-score groups using the median CRG-score as the cut-off value, and then Kaplan–Meier survival analysis was performed.

### 2.5. TME, Mutation, and Drug Susceptibility Analysis Based on CRG-Score

The TME differences between the low- and high-risk groups were investigated using the relative proportions of 22 kinds of immune cells identified and determined using CIBERSORT. Each CRC patient's tumor mutation burden (TMB) score was calculated in high- and low-risk groups. Meanwhile, based on maftools approach, a waterfall plot was created to show the number of variations and mutation distribution of the most often mutated genes in each sample. We also observed the links between the two risk groups and microsatellite instability (MSI), as well as cancer stem cells (CSC). The difference in semi-inhibitory concentration (IC50) values of common CRC chemotherapeutic medicines between the low and high CRG-score groups was calculated using the ‘pRRophetic' software.

### 2.6. Statistical Analyses

The random stratified sampling was carried out using R's sample function. Meanwhile, the pheatmap and maftools packages in R were used to characterize the DEGs heatmap and mutation landscape, respectively. One-way ANOVA and Kruskal-Wallis tests were used to determine the significance of differences between three or more groups. The Wilcoxon test was utilized to compare the differences between the two groups. The correlation coefficients were calculated by the Spearman and distance correlation analysis. The log-rank tests and Kaplan–Meier survival analysis were created using the survminer R program. A *P* value of less than 0.05 was considered statistically significant for all two-sided statistical *P* values. R version 4.1.1 was used for all statistical analyses, and no default settings were utilized in any of the investigations.

## 3. Results

### 3.1. Genetic Mutation Landscape of CRGs in CRC

**Figure 1:**
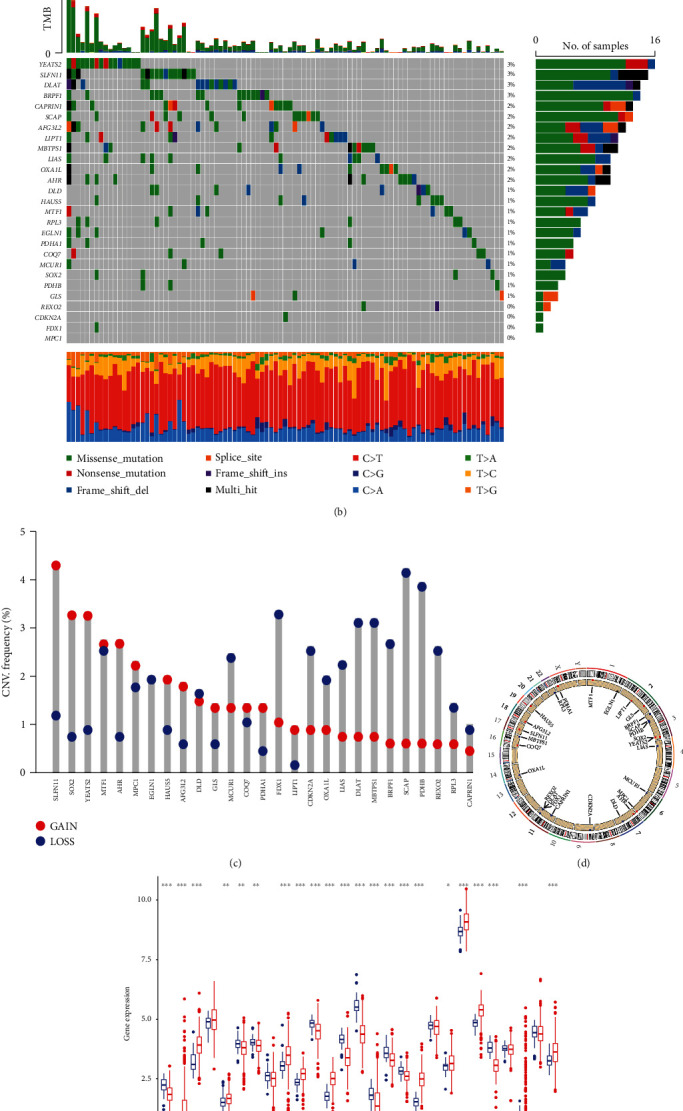
Multiomics analyses of CRGs in TCGA CRC data. (a) Whole-genome CRISPR-Cas9 positive selection screen using two copper ionophores (Cu-DDC and elesclomol-copper) in cells. Overlapping hits with a false discovery rate (FDR) value < 0.05 were analyzed. (b) Mutation frequencies of 27 CRGs in 535 patients with CRC from TCGA cohort. (c) Frequencies of CNV gain, loss, and non-CNV among CRGs. (d) Locations of CNV alterations in CRGs on 23 chromosomes. (e) Expression levels of 27 CRGs between normal and CRC tissues. Red gene names present CNV gains, blue gene names represent CNV loss, and black gene names represent CNV constant. CRGs: cuproptosis-related genes; CRC: colorectal cancer; TCGA: The Cancer Genome Atlas; CNV: copy number variant.

Based on the study by Tsvetkov et al., we first identified 27 CRGs through a whole-genome CRISPR-Cas9 positive selection screen using two copper ionophores (Cu-DDC and Elesclomol-copper) in cells (Figure 1(a)). Then, we analyzed the mutational landscape of the CRGs in the TCGA-CRC datasets. As depicted in Figure 1(b) 95 of 535 (17.76%) CRC samples presented genetic mutations among the 27 CRGs. Furthermore, we explored the mutational incidence of copy number variation (CNV), indicating that 27 CRGs demonstrated evident CNV alterations (Figure 1(c)). Among them, *SLFN11*, *SOX2*, *YEAST2*, *AHR*, *MPC1*, *HAUS5*, *AFG3L2*, *COQ7*, *LIPT1*, *PDHA1*, and *GLS* had widespread CNV increases, while *MCUR1*, *OXAL1*, *LIAS*, *MBTPS1*, *BRPF1*, *REXO2*, *RPL3*, *CAPRIN1*, *FDX1*, *CDKN2A*, *DLAT*, *PDHB*, and *SCAP* showed CNV decreases.  Figure 1(d) displayed the site of CNV alterations of 27 CRGs on chromosomes. We also analyzed the expression levels of CRGs in CRC and normal tissues and discovered that most CRG expression levels were linked to CNV changes (Figure 1(e)), and CRGs with CNV gains, such as *YEATS2* and *AHR*, were significantly elevated in CRC samples.

### 3.2. Identification of Cuproptosis Subtypes in CRC

**Figure 2:**
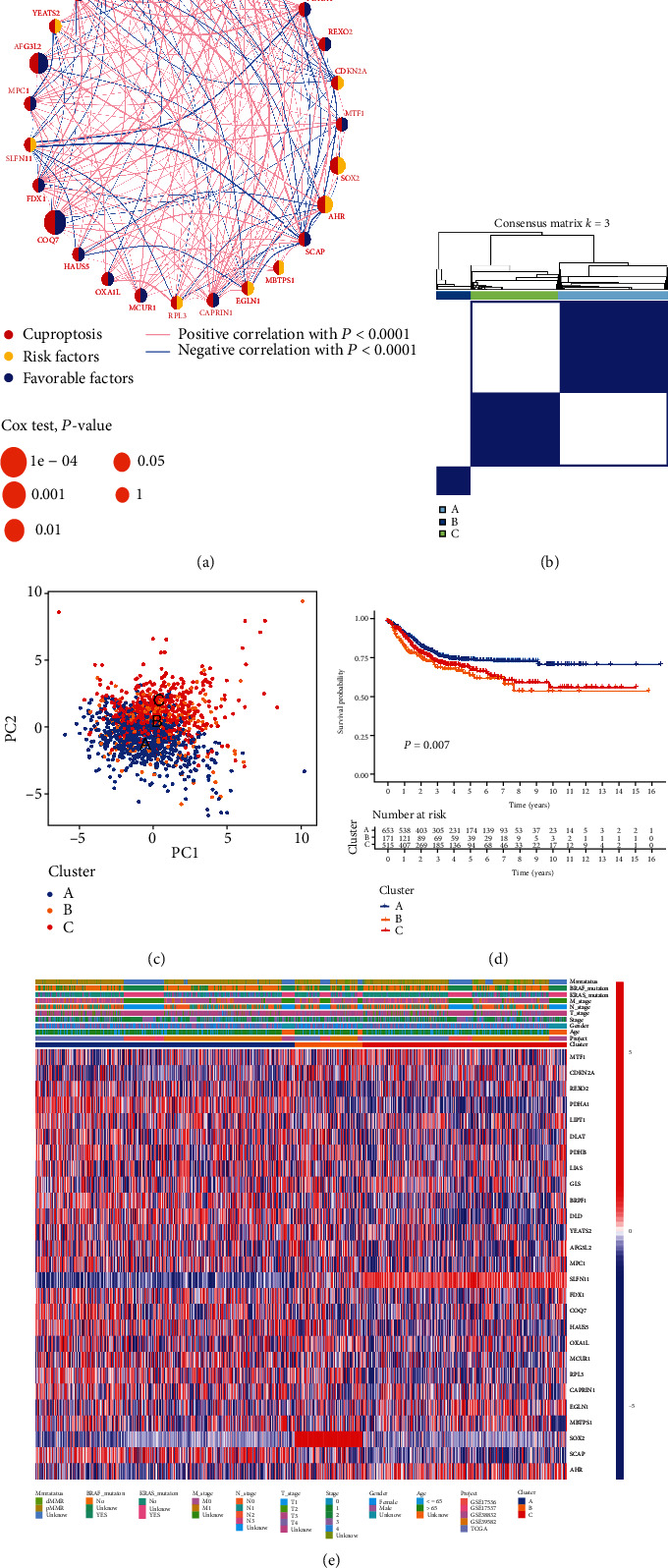
The clinicopathological and biological characteristics in distinct CRG subtypes. (a) The Interaction analysis among CRGs in CRC. The line of the CRGs represents their interaction and the line thickness indicates the strength of the correlation between genes. Blue and pink represent negative and positive correlations, respectively. (b) Consensus analysis defining three CRGs clusters (*k* = 3) and correlation area. (c) The PCA analysis among three CRG cluster subtypes. (d) The Kaplan–Meier survival analysis for CRC patients of three subtypes associated with DFS. (e) Heatmap analysis in clinicopathologic features and expression levels of CRGs among three distinct subtypes. CRG: cuproptosis-related genes; CRC: colorectal cancer; PCA: principal components analysis; DFS: disease-free survival.

This study enlisted 1339 CRC patients from five suitable cohorts (TCGA-CRC, GSE39582, GSE17536, GSE17537, and GSE38832) for the following analysis. With Kaplan–Meier survival analysis and univariate Cox regression, the prognostic values of 27 CRGs in CRC patients were discovered (Table S3). The 27 CRGs were then assembled into a network map, allowing for a thorough examination of the genes' interactions and interconnections, as well as their influence on the prognosis of CRC patients (Figure 2(a)). We further conducted unsupervised clustering on 1339 CRC samples by the consensus clustering algorithm and identified three subtypes, including clusters A (*n* = 653), B (*n* = 171), and C (*n* = 515) (Figures 2(b) and 2(c), S2, and Table S4). Prognostic analysis for the three distinct phenotypes showed obviously survival advantage in the A subtype and survival inferiority in the B and C subtypes (Figure 2(d)). We also analyzed the genomic expression and clinicopathological factors across three clusters, as shown in Figure 2(e), and some genes like *PDHA1*, *AHR*, *SOX2*, and *SLFN11* showed significant differential expression levels, but none of the clinicopathological features were significantly different.

### 3.3. Characteristics of the TME in Different Subtypes

**Figure 3:**
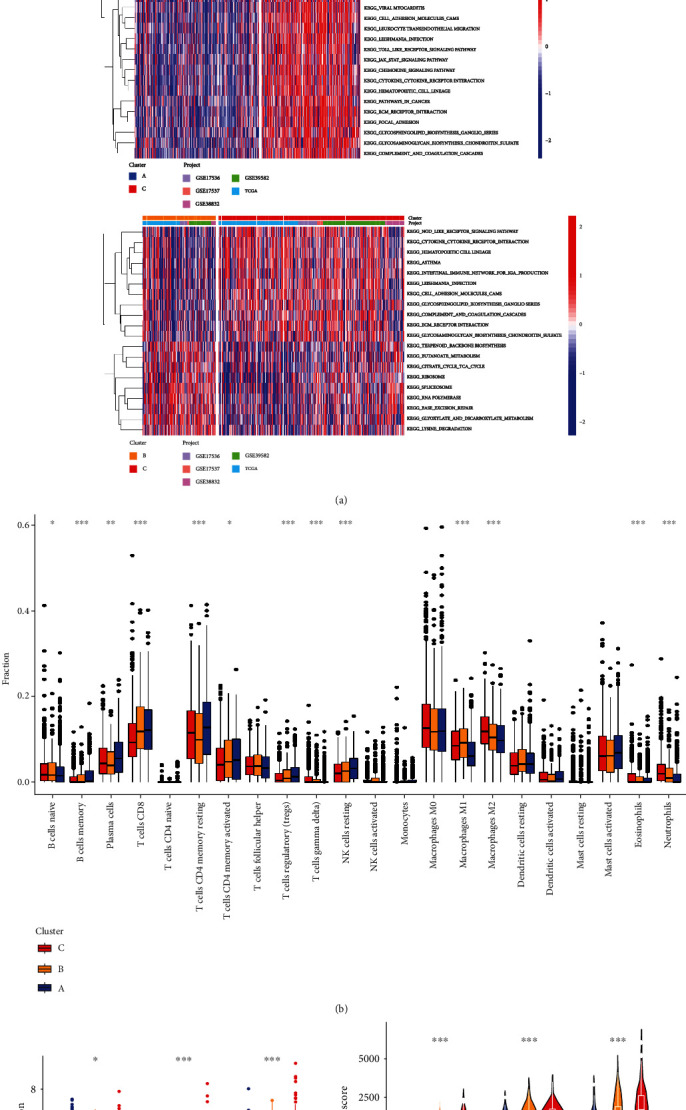
Correlations of tumor immune microenvironment with three CRG subtypes. (a) GSVA of biological pathways among three distinct subtypes in five datasets, where red and blue represent activated and blue inhibited pathways, respectively. (b) Abundance of 22 infiltrating immune cell types in three CRG subtypes. (c) Expression levels of PD-1, CTLA4, and PD-L1 among three CRG subtypes. (d) Difference of TME score among three CRG subtypes. CRG: cuproptosis-related genes; GSVA: gene set variation analysis; TME: tumor microenvironment.

We used GSVA enrichment analysis on the three subtypes to observe whether there were any changes in biological behavior between them (Figure 3(a) and Table S5). Cluster A and cluster B, cluster A and cluster C, and cluster B and cluster C were compared in terms of enrichment analysis, respectively. We observed that enrichment for multiple metabolism signatures was pronounced in cluster A, including pyruvate metabolism, pentose phosphate pathway, and citrate cycle TCA cycle. In contrast, cluster C showed enrichment in terms of pathways associated with immune activation, such as the activated JAK-STAT signaling pathway, TOLL/NOD-like receptor signaling pathways, and antigen processing and presentation. Following TME cell infiltration investigations (Table S6), as shown in Figures 3(b) and 3(a), significant enrichment difference in most immune cells was observed among the three clusters. In cluster A, significant immune-infiltrating cells were plasma cells, T cells CD8, T cells CD4 memory resting and activated, and NK cells resting. Cluster C was enriched with macrophages, neutrophils, and eosinophils. Cluster B had an immune cell infiltration level that was halfway between clusters A and C. Similarly, *CTLA4*, *PD-1*, and *PD-L1* expression levels were found to be higher in cluster C (cluster C vs. cluster A), when three critical immunological checkpoints were examined (Figure 3(c)). We utilized the ESTIMATE method to get TME scores (stromal score, immune score, and estimate score) in the different clusters, and TME scores could evaluate the amount of immunological and stromal components in TME. CRC patients in cluster C had higher TME scores, according to the findings (Figure 3(d)). Up to a point, the higher TME scores in cluster C can indicate tumors in cluster C patients have higher interactions between stromal cells and immune cells.

### 3.4. Development and Validation of a Gene Signature Based on Cuproptosis-Related Clusters

**Figure 4:**
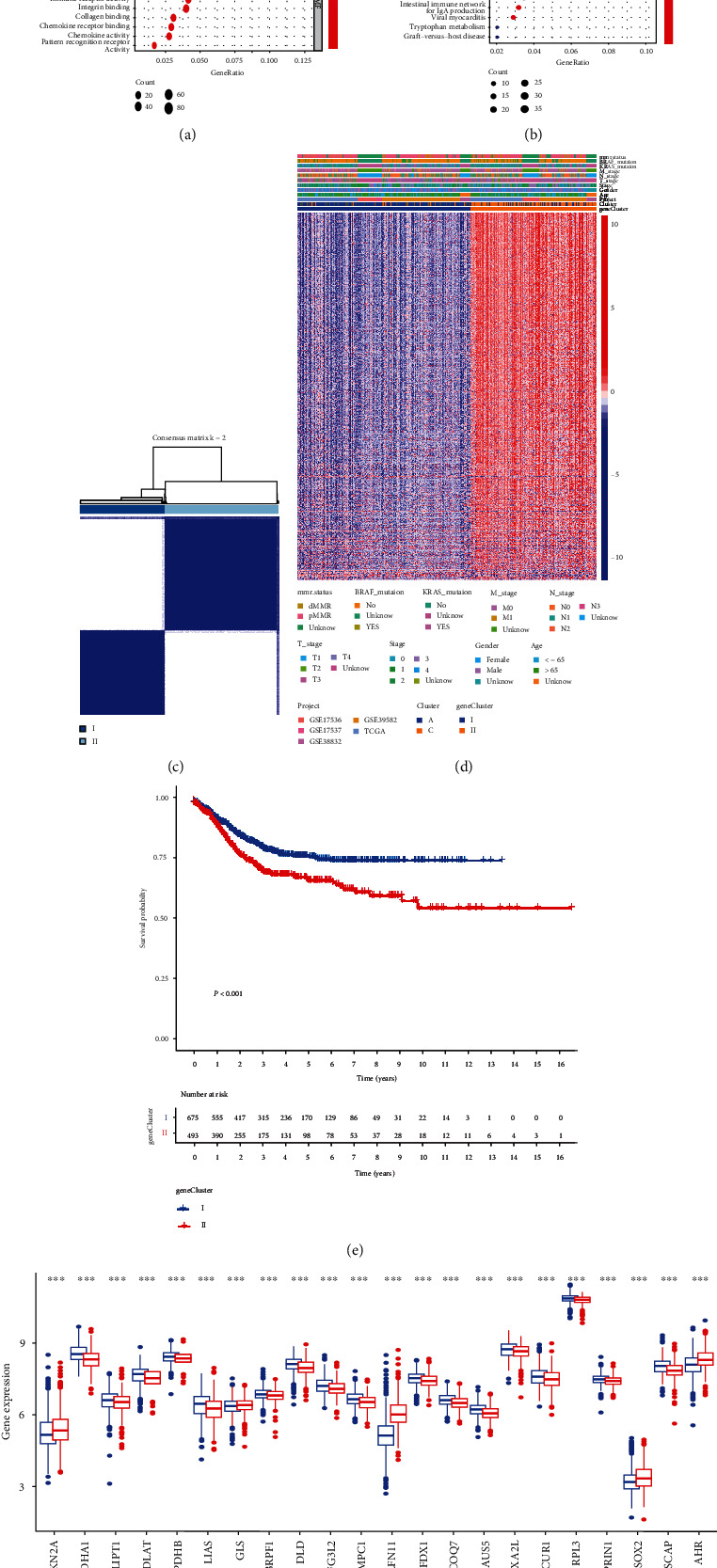
Identification of CRG-related gene subtypes. GO (a) and KEGG (b) enrichment analyses of DEGs between cuproptosis clusters A and C. (c) Consensus analysis defining two gene clusters (*k* = 2) and correlation area. (d) Heatmap analysis in clinicopathologic features and expression levels of CRGs between two distinct gene subtypes. (e) The Kaplan–Meier survival analysis for CRC patients of two gene subtypes associated with DFS. (f) Differential expression levels of 27 CRGs between two gene subtypes. GO: Gene Ontology; KEGG: Kyoto Encyclopedia of Genes and Genomes; DEGs, differentially expressed genes; CRG, cuproptosis-related genes; DFS: disease-free survival.

We identified the DEGs between clusters A and C (*P* < 0.05) in order to further investigate the probable biological role of the cuproptosis subtypes in CRC, and 702 genes were retrieved. Then, using the “clusterProfile” package, we performed GO and KEGG enrichment analysis and discovered that these DEGs are strongly correlated with immune biological processes (Figures 4(a) and 4(b) and Table S7). Following that, 428 prognosis-related genes were identified using univariate Cox regression analysis for these 702 cuproptosis subtype-related genes. Then, based on the 428 prognosis-related DEGs, a consensus clustering technique was used to divide patients into two genomic subgroups, which were dubbed gene clusters I and II, respectively (Figure 4(c), S3, and Table S8). On the basis of DEG expression levels, we discovered that the two gene subtypes could be distinguished considerably (Figure 4(d)). Furthermore, CRC patients in gene cluster II had a worse DFS than those in gene cluster I, according to the survival analysis (Figure 4(e)). Meanwhile, we found the expression levels of the majority of CRGs (22/27) were significantly different between the two gene subtypes (Figure 4(f)).

**Figure 5:**
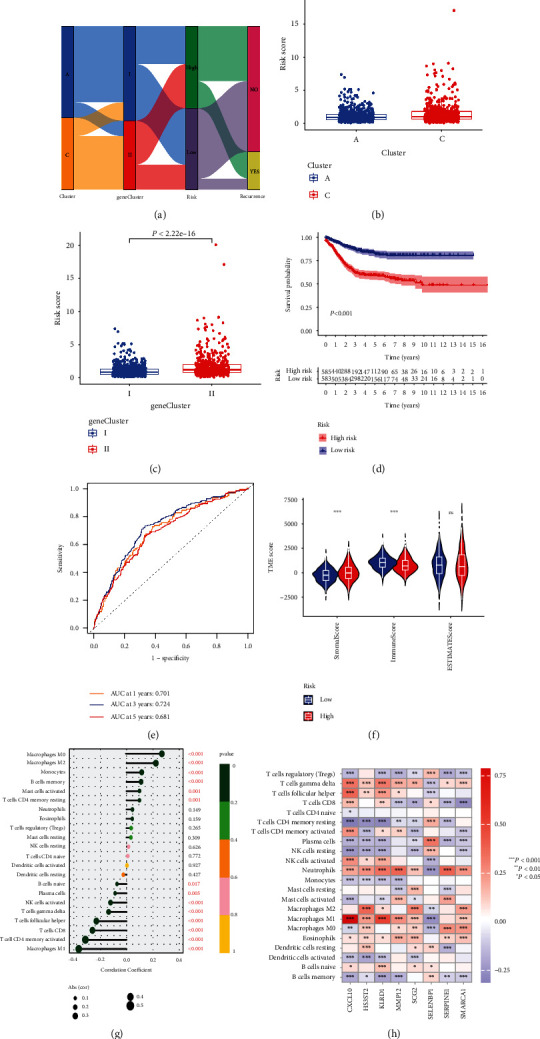
Construction, validation, and evaluation of CRG-score model. (a) Alluvial diagram of subtype distributions in groups with different CRG-scores and DFS. (b) Differences in CRG-scores between cuproptosis clusters A and C. (c) Differences in CRG-scores between two gene subtypes. (d) Kaplan–Meier curves analysis for DFS of CRC patients between high-risk and low-risk groups in the training and testing sets. (e) ROC curves of the CRG-scores to demonstrate the sensitivity and specificity in predicting the DFS of CRC patients from training and testing sets. (f) Differences in TME score between two gene subtypes. (g) Ranked dot and scatter plots showing the correlation between CRG-score and 22 infiltrating immune cell types. (h) Correlations between the abundance of immune cells and eight genes from the CRG-score model.

To establish a model that could quantify each CRC patient, we assigned randomly CRC patients to a training cohort (*n* = 670) or a testing cohort (*n* = 669) at a ratio of 1 : 1. Then eight of the 428 DEGs were retained by application of LASSO regression and multivariate Cox regression models (Figure S4 and Table S9). We used these eight genes to build a cuproptosis-related signature score which we named the “CRG − score” = (−0:469 × expressionlevel of KLRD1) + (0:281 × expression level of SMARCA1) + (0:185 × expression level of SCG2) + (0:263 × expression level of SERPINE1) + (0:227 × expression level of HS3ST2) + (−0:230 × expression level of CXCL10) + (−0:217 × expression level of SELENBP1) + (−0:148 × expression level of MMP12) (Table S10). We divided CRC patients into high and low CRG-score groups with the cut-off value of the median. We further observed molecular subtype distributions with different CRG-score and DFS outcomes in Figure 5(a), and found that the low- and high-risk CRG-score was significantly differential in the cuproptosis clusters and gene clusters (Figures 5(b) and 5(c)).

Then, in the training and testing sets, we sought to establish the possible predictive value of each CRC patient's CRG-score (Figures S5 and S6). The Kaplan–Meier survival curves in two datasets demonstrated that CRC patients with low risk had a considerably better disease-free survival than those with high risk (log-rank test, *P* < 0.001; Figure 5(d)). Furthermore, for 1-, 3-, and 5-year survival rates, the AUC values of CRG-score were 0.701, 0.724, and 0.687, respectively (Figure 5(e)). To validate the applicability and generalizability of the CRG-score, we further verified this score in an independent cohort with a total of 191 CRC patients (GSE161158) and obtained the same results (Figure S7). We further included CRG-score as an effective indicator and other clinical characteristics in univariate and multivariate Cox regression analysis and found that CRG-score could be recognized as an independent factor that predicts the prognosis of CRC patients (Table 2). Additionally, we also created a predictive nomogram based on the CRG-score and clinicopathological parameters to predict the 1-, 3-, and 5-year DFS status of each patient in the training and testing sets, and calibration plots revealed that the constructed nomogram performed similarly to an ideal model (Figures S8a, S8b, S8d, and S8e). Meanwhile, in the training and testing sets, we also evaluated the prediction accuracy of the CRG-score with clinicopathological markers, and the findings revealed that our signature's AUC was better than that of other clinical criteria (AUC = 0.723) (Figures S8(c) and S8(f)).

### 3.5. Correlation of CRG-Score with TME, TMB, MSI, and CSC Index and Drug Susceptibility

We discovered that CRG-score might play an essential role in clinical prediction based on these findings. Then we observed whether the CRG-score may be used as a guide for treatment, particularly immunotherapy. With different CRG scores, we looked at TME cell infiltration. First, when the CRG-score grew, the immuneScore from ESTIMATE analysis decreased, but the stromalScore had the reverse impact (Figure 5(f)). Furthermore, immunological activation-related cells such as T cells CD8, NK cells, and macrophages M1 had significant negative correlations with CRG-score (*P* < 0.001), but M2 macrophages had a positive connection with CRG-score owing to polarization (*P* < 0.001) (Figure 5(g)). Furthermore, we discovered that the majority of immune invading cells were highly associated with the eight CRG-score genes (Figure 5(h)).

**Figure 6:**
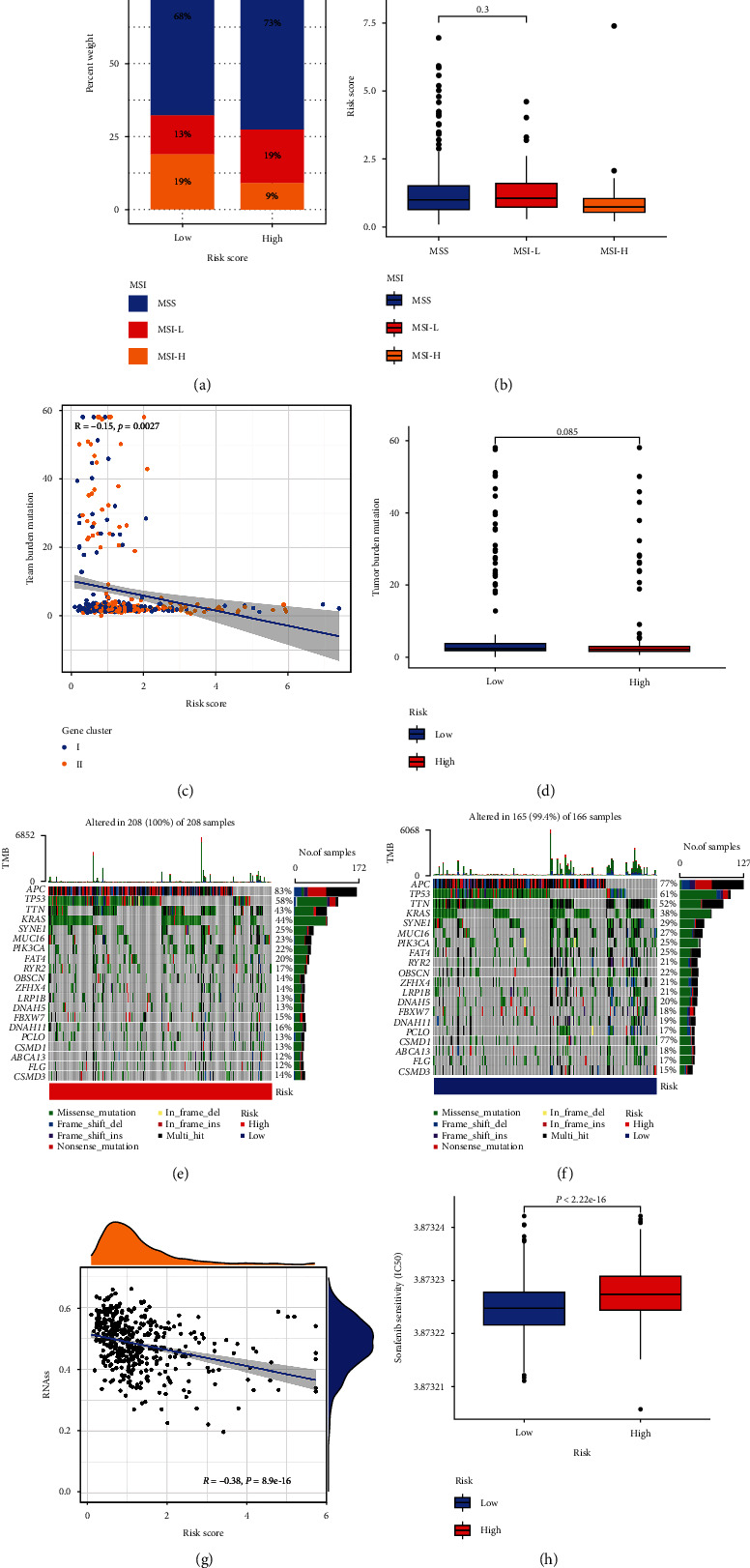
Comprehensive analysis of the CRG-score in CRC. (a) and (b) Relationships between CRG-score and MSI. (c) The correlation of the CRG-score with TMB between two gene subtypes. (d) TMB levels between the high and low-risk groups. The waterfall plot of somatic mutation features established with high (e) and low (f) CRG-scores. Each column represented an individual patient and the number on the right indicated the mutation frequency in each gene. (g) Correlation between CRG-score and stem cell index. (h) Correlation between CRG-score and chemotherapeutic sensitivity. CRG: cuproptosis-related genes; TMB: Tumor mutation burden.

TMB and MSI were important predictors of tumor immune response and played an indispensable role in carcinogenesis and progression. As a result, we investigated the function of MSI and TMB in the CRG-score in greater depth. Low CRG-score was connected with MSI-H status, whereas the high CRG-score was associated with microsatellite stable (MSS) status, according to correlation studies (Figures 6(a) and 6(b)). The Spearman correlation analysis revealed a weak correlation between CRG-score and TMB (*R* = −0.15) (Figure 6(c)), and no significant difference in TMB between the low-risk and high-risk groups (Figure 6(d)). As a result, we looked at the changes in somatic mutation distribution in the CRG-score signature. We observed the top 20 somatic mutation genes with high mutational frequencies in TCGA CRC cohort and found no significant changes in TMB or mutation types between the high and low CRG-score groups (Figures 6(e) and 6(f)).

In addition, CRG-score was shown to be inversely linked with the CSC index in Figure 6(g), showing that CRC cells with a lower CRG-score exhibited more unique stem cell features and a lower degree of cell differentiation (*R* = −0.38, *P* < 0.001). Then, we chose chemotherapeutic drugs commonly used in CRC treatment to observe how sensitive individuals in low- and high-risk categories were to them. Interestingly, CRC patients with a high CRG-score had lower IC50 values for shikonin, whereas chemotherapeutics including sorafenib, ABT.888, and gefitinib had considerably lower IC50 values in the low CRG-score group (Figure 6(h), S9).

## 4. Discussion

Despite the introduction of the term cuproptosis, the cellular processes, overall impact, and TME infiltration features mediated by the combined actions of numerous CRGs have yet to be thoroughly understood in CRC. The 27 CRGs were studied in terms of prognostic value, functions in the immune microenvironment, and putative regulatory mechanisms in CRC in this work. Despite the low mutational intensity of CRGs, the majority of CRGs (22/27) were found to be differently expressed between normal and malignant tissues, with a total of 22 genes having substantial prognostic significance. The finding of differences in expression levels of CRGs indicated the latent function of CRGs in CRC tumorigenesis. We then divided CRC patients into three cuproptosis subgroups (Clusters A, B, and C) using the unsupervised clustering approach. Cuproptosis clusters were connected to distinct cellular metabolism-related signaling pathways and immunological infiltrations, and were shown to be independent of clinicopathological characteristics. As a result, we found two gene clusters with diverse immunological activities and roles based on DEGs connected to the subgroups' signature. By LASSO and multivariate Cox regression models, CRG-score was also constructed to quantify the cuproptosis gene subgroups. In the TME, MSI, CSC index, and drug susceptibility, CRC patients with low- and high-risk scores had significantly different prognoses. Specifically, when compared to standard clinical markers like the TNM stage, the CRG-score outperformed them in predicting patient prognosis.

Cuproptosis is a kind of cell death that is dependent on copper and protein lipoylation [14]. It differs from other types of cell death. Copper levels are very high in CRC patients' serum and tissue [20–23]. Furthermore, cancer cells have elevated copper concentrations, which are thought to be critical for angiogenesis and metastasis [24]. Cuproptosis is therefore anticipated to play a key role in the establishment and progression of neoplasms. Our findings consistently showed that differences in mRNA transcriptomes between different cuproptosis subtypes were significantly related to metabolism and immune-related biological pathways, such as patients with cluster A had more metabolism reprogramming features and better DFS, and patients with cluster C had higher TME scores and showed enrichment in pathways associated with immune activation. The cuproptosis-related prognostic signature was found to be a good predictor of CRC patients' prognosis. These results indicate that the cuproptosis could lead to different biological behaviors in CRC patients, confirming the existing conclusions.

According to mounting evidence, the TME of CRC plays a critical role in tumor progression, invasiveness, metastasis, drug resistance, and even the maintenance of the cancer stem-like phenotype [25], which is primarily mediated by immune cells (macrophages, T cells, B cells, and so on), cytokines, chemokines, and exosomes [26]. Throughout cancer development, the TME constantly alters with great complexity. Immune interaction is a fundamental characteristic of CRC and a potential treatment target. The key components of TME are stromal cells and immune cells, and immune and stromal scores are linked to colorectal cancer clinical features and prognosis [27, 28]. The immune-related cuproptosis pattern (cluster C) was linked with a higher CRG-score in the current study, whereas the metabolic reprogramming pattern (cluster A) was associated with a lower CRG-score. The TME features and relative abundance of 22 tumor-infiltrating immune cells changed considerably between the low- and high-risk CRG-score groups, according to our findings. This data points to the importance of CRGs in the evolution of CRC. TILs (tumor-infiltrating lymphocytes) are a kind of immune cell that infiltrates tumors. They are made up of CD4T cells, CD8T cells, B cells, and NK cells, among other T cell subpopulations. TILs can help in tumor immune evasion as well as tumor detection, destruction, and eradication [29]. Plasma cells, T cells CD8, T cells CD4 memory resting and activated, and NK cells resting were found in larger numbers in cluster A and low CRG-score patients with a better prognosis, indicating that they play a favorable role in CRC development. M2 macrophages have been reported to promote tumor growth by a variety of mechanisms, including the production of immunomodulators such as IL-10, IL-6, and TGF-1, as well as the recruitment of Th2 and Treg cells via anti-inflammatory chemokines such as CXCL17, 22, and 24 [30–32]. Meanwhile, mounting data shows that neutrophils promote CRC development and metastasis via the CXCL1/CXCR2 chemokine axis, as well as modify the ECM milieu by generating matrix metalloproteinase MMP9 [33, 34]. This corresponds to our finding that CRC patients with cluster C and high CRG-score had more M2 macrophages and neutrophils compared to those in the low CRG-score group. In addition, among cuproptosis subtypes, cluster C demonstrated the highest expression of three immune checkpoints (*CTLA4*, *PD-1*, and *PD-L1*), with a worse prognosis. Patients with a high CRG-score, as well as increased *PD-1*, *PD-L1*, and *CTLA-4* expression, were shown to be more likely to react to immune checkpoint blocking. The exact mechanism linking cuproptosis with immunity, however, remains unknown. These findings might help us learn more about the association between cuproptosis and CRC TME infiltration cells.

Tumors have metabolic processes that have been altered to support cancer development [35]. The variability and flexibility of metabolism across malignancies and TME have been highlighted in recent research [36]. Metabolic heterogeneity is caused by a variety of mechanisms in the TME, including interactions between cancer cells and other TME components, such as immune cells, and extracellular matrix. In our study, we found that enrichment for multiple metabolism signatures was pronounced in cluster A, such as citrate cycle TCA cycle and pyruvate metabolism. Metabolic heterogeneity is significant because it has an impact on treatment vulnerabilities and may predict clinical outcomes. Copper-based compounds have been proven in several studies to be powerful cytotoxic agents that may specifically target cancer cells in vitro and in vivo [37–39]. Moreover, copper affects the proliferation of cancer cells, which can modulate tumor tissue homeostasis [40, 41]. The growing interest in the design of copper-based compounds as anticancer drugs stems. But, the exploitation of copper toxicity has been less successful in cancer. One of the main factors is that copper ionophore–induced cell death is mediated by the pathways of mitochondrial respiration. Copper ionophores are almost 1000-fold more sensitive in mitochondrial respiration-dependent cells than in glycolysis-dependent cells [14]. The metabolic activity of cancer cells is aberrant; the Warburg Effect is a frequent metabolic trait of cancer cells that entails a preference for aerobic glycolysis over oxidative phosphorylation to create ATP and biosynthetic building blocks [17]. It is not clear whether metabolic changes in CRC patients with cluster A are driven or mediated by cuproptosis-related mechanisms, or whether these metabolic changes are caused by the tumor itself, resulting in different cuproptosis-related changes. However, the molecular subtypes based on CRGs may certainly have some guiding significance for the treatment of copper-based compounds in CRC.

This study has some limitations. First, all analyses are based on several public databases, and the selection bias may affect their robustness, the stability of the type required multiangle and multidatabase validation, and the link between cuproptosis and TME requires additional experimental verification. Furthermore, when assessing the prognosis of cuproptosis status, some treatment modalities such as surgery, radiotherapy, and chemotherapy were not included in the analysis of survival models, which may cause certain accuracy problems. In addition, tumor metabolic heterogeneity is an issue that must be addressed, and with the advancement of cuproptosis-related research, more focused solutions for different types of tumors may be provided.

## 5. Conclusion

In conclusion, this research discovered three subtypes of cuproptosis-related molecules in CRC, as well as their biological function, demonstrating the heterogeneity of the TME in CRC. Finally, we created a CRG-score signature to measure cuproptosis traits statistically. Furthermore, the CRG-score signature might be a useful prediction tool for predicting CRC patients' prognosis and guiding appropriate therapeutic therapy. In summary, this research revealed new information on the involvement of cuproptosis and TME in boosting clinical outcomes and allowing tailored immunotherapy choices for CRC patients.

## Figures and Tables

**Table 1:** Overview of the datasets used in this study.

Dataset	Source	Assay	Sample type	Number of samples
Training set+testing set	TCGA CRC	Infinium	Tissue	CRC = 503
	GSE17536	HG-U133 plus 2.0	Tissue	CRC = 145
	GSE17537	HG-U133 plus 2.0	Tissue	CRC = 42
	GSE39582	HG-U133 plus 2.0	Tissue	CRC = 557
	GSE38832	HG-U133 plus 2.0	Tissue	CRC = 92

Validation set	GSE161158	HG-U133 plus 2.0	Tissue	CRC = 191

**Table 2:** Univariate and multivariate Cox regression analysis of cuproptosis-related genes in datasets.

Variables	Training dataset				Testing dataset				Validation dataset			
	95% CI				95% CI				95% CI			
	HR	Lower	Upper	*P*	HR	Lower	Upper	*P*	HR	Lower	Upper	*P*
Univariate analysis												
Age												
≥ 65 vs. < 65 years	0.761	0.552	1.050	0.096	0.857	0.629	1.169	0.330	0.658	0.461	0.939	0.021
Sex												
Male vs female	0.786	0.567	1.090	0.148	0.868	0.635	1.187	0.376	0.803	0.559	1.153	0.234
TNM stage												
III+IV vs. I+II	2.473	1.764	3.465	< 0.001	2.711	1.967	3.737	< 0.001	3.246	2.228	4.730	< 0.001
CRG-score												
High vs. low risk	2.134	1.668	2.730	< 0.001	1.443	1.163	1.789	0.001	1.127	1.070	1.188	< 0.001
Multivariate analysis												
Age												
≥ 65 vs. < 65 years												
Sex												
Male vs. female												
TNM stage												
III+IV vs. I+II	2.285	1.628	3.208	< 0.001	2.654	1.924	3.659	< 0.001	3.153	2.156	4.610	< 0.001
CRG-score												
High vs. low risk	2.008	1.545	2.609	< 0.001	1.388	1.113	1.732	0.004	1.132	1.067	1.201	< 0.001

## Data Availability

The data used to support the results of this study are available from The Cancer Genome Atlas (TCGA) database (https://portal.gdc.cancer.gov/repository) and the Gene Expression Omnibus (GEO) database (https://www.ncbi.nlm.nih.gov/geo/).

## Supplementary Materials

Supplementary 1 Table S1-S10. Supplementary Table S1: Whole-genome CRISPR screen for the Elesclomol-Cu and Cupric-DDC in cells. Table S2: Summary of 27 cuproptosis-related genes. Table S3: The prognostic value of 27 cuproptosis-related genes in CRC patients. Table S4: The subtype of 27 cuproptosis-related genes in CRC patients. Table S5: The differential states of biological pathways in distinct cuproptosis subtypes by GSVA enrichment analysis. Table S6: Relative fractions of tumor-infiltrating immune cells of CRC patients by CIBERSORT algorithm. Table S7: Functional analysis of the 702 differentially expressed genes between the cuproptosis subtypes. Table S8: The gene subtype of cuproptosis subgroups related genes in CRC patients. Supplementary Table S9 The prognostic values of 428 cuproptosis subtype related genes in CRC patients. Table S10: Multivariate Cox regression analysis of 8 CRGs associated with DFS in CRC patients.

Supplementary 2 Figure S1-S9. Figure S1: Flowchart of the research. Figure S2: Unsupervised clustering of cuproptosis cluster and consensus matrix heatmaps for *k* = 2, 4-9. Figure S3: Unsupervised clustering of cuproptosis-related gene cluster and Consensus matrix heatmaps for *k* = 3 − 9. Figure S4: The identification of candidate prognostic genes by LASSO regression analysis. Figure S5: Construction of CRG_score model in the training dataset. Figure S6: Validation of CRG_score model in the testing dataset. Figure S7: Validation of CRG_score model in the GSE161158 dataset. Figure S8: Development and evaluation of a prognostic nomogram in the training and testing sets. Figure S9: Relationships between CRG_score and chemotherapeutic sensitivity.
