# Supplementary material for: Molecular Subtypes Based on Cuproptosis-Related Genes and Tumor Microenvironment Infiltration Characterization in Colorectal Cancer
Source: J Oncol. 2022 Oct 11;2022:5034092. doi: 10.1155/2022/5034092 (PMC9579866; doi:10.1155/2022/5034092)
Supplement: Supplementary 1 — Table S1-S10. Supplementary Table S1: Whole-genome CRISPR screen for the Elesclomol-Cu and Cupric-DDC in cells. Table S2: Summary of 27 cuproptosis-related genes. Table S3: The prognostic value of 27 cuproptosis-related genes in CRC patients. Table S4: The subtype of 27 cuproptosis-related genes in CRC patients. Table S5: The differential states of biological pathways in distinct cuproptosis subtypes by GSVA enrichment analysis. Table S6: Relative fractions of tumor-infiltrating immune cells of CRC patients by CIBERSORT algorithm. Table S7: Functional analysis of the 702 differentially expressed genes between the cuproptosis subtypes. Table S8: The gene subtype of cuproptosis subgroups related genes in CRC patients. Supplementary Table S9 The prognostic values of 428 cuproptosis subtype related genes in CRC patients. Table S10: Multivariate Cox regression analysis of 8 CRGs associated with DFS in CRC patients. [file 5034092.f1.pdf]

Table S1. Whole genome CRISPR screen for the Elesclomol-Cu and Cupric - DDC in cells .

Elesclomol Cu					Cu-DDC				
Gene	num	logFC	FDR	logFDR	Gene	num	logFC	FDR	logFDR
OXA1L	3	-1.7625	5.73E-04	3.241692	*MTF1*	4	-1.4915	2.15E-04	3.667661
CDKN2A	4	-1.0521	5.73E-04	3.241692	*CDKN2A*	4	-0.81318	2.15E-04	3.667661
TMEM191B	1	-0.99999	5.73E-04	3.241692	*REXO2*	4	0.50318	2.15E-04	3.667661
SOX2	4	-0.94481	5.73E-04	3.241692	*RPUSD3*	4	0.55871	2.15E-04	3.667661
AHR	4	-0.90798	5.73E-04	3.241692	*PDHA1*	3	0.72575	2.15E-04	3.667661
TKT	4	-0.84353	5.73E-04	3.241692	*MRPL17*	4	0.7419	2.15E-04	3.667661
LIPT1	4	0.71486	5.73E-04	3.241692	*SGF29*	4	0.75103	2.15E-04	3.667661
DLAT	4	0.76068	5.73E-04	3.241692	*COX11*	4	0.77223	2.15E-04	3.667661
PDHB	4	0.76656	5.73E-04	3.241692	*NDUFS1*	4	0.77806	2.15E-04	3.667661
LIAS	4	1.1756	5.73E-04	3.241692	*LIPT1*	4	0.79029	2.15E-04	3.667661
MBTPS1	4	1.331	5.73E-04	3.241692	*MRPL21*	4	0.808	2.15E-04	3.667661
MBTPS2	4	1.4814	5.73E-04	3.241692	*LARS2*	4	0.87731	2.15E-04	3.667661
SCAP	4	1.9493	5.73E-04	3.241692	*MRPL41*	4	0.88082	2.15E-04	3.667661
FDX1	2	2.116	5.73E-04	3.241692	*POLG*	4	0.89673	2.15E-04	3.667661
RPAP1	1	3.9344	5.73E-04	3.241692	*MECR*	4	0.89958	2.15E-04	3.667661
RPL3	3	2.6016	0.001612	2.7926	*DLAT*	4	0.90174	2.15E-04	3.667661
UBAP2L	4	0.74202	0.002149	2.667677	*QRSL1*	4	0.9336	2.15E-04	3.667661
PDHA1	3	0.77404	0.002149	2.667677	*MTG1*	4	0.95131	2.15E-04	3.667661
LDLR	4	0.84781	0.002149	2.667677	*STARD7*	4	0.95364	2.15E-04	3.667661
CAPRIN1	3	1.0719	0.002149	2.667677	*FASTKD5*	4	0.98534	2.15E-04	3.667661
ZFAT	4	0.59704	0.002736	2.562919	*PDE12*	4	1.0092	2.15E-04	3.667661
IDH2	4	1.0214	0.002736	2.562919	*TWNK*	4	1.01	2.15E-04	3.667661
SAMM50	3	1.6615	0.004112	2.385938	*MRPL15*	4	1.0212	2.15E-04	3.667661
PITRM1	4	-1.0269	0.004299	2.366635	*CLPP*	3	1.0417	2.15E-04	3.667661
PGP	4	-0.88063	0.004299	2.366635	*MRPS25*	4	1.0455	2.15E-04	3.667661
DLD	3	1.2678	0.004299	2.366635	*CS*	4	1.047	2.15E-04	3.667661
PRKDC	4	-0.68579	0.00504	2.297547	*COX17*	3	1.1324	2.15E-04	3.667661
CERT1	4	0.54329	0.00504	2.297547	*PDHX*	3	1.1332	2.15E-04	3.667661
TSC22D2	4	1.0975	0.00504	2.297547	*CYCS*	3	1.1339	2.15E-04	3.667661
INTS6	4	0.951	0.006592	2.180997	*PDHB*	4	1.1523	2.15E-04	3.667661
TADA2A	4	0.83577	0.006934	2.159026	*AASDHPPT*	4	1.1957	2.15E-04	3.667661
MTF1	4	-1.1664	0.007035	2.152749	*FAU*	4	1.2084	2.15E-04	3.667661
API5	4	0.50207	0.007035	2.152749	*HARS2*	4	1.2207	2.15E-04	3.667661
C6orf136	3	-1.3452	0.007334	2.134681	*SLC25A39*	4	1.3161	2.15E-04	3.667661
GLS	3	-0.33613	0.008107	2.091157	*CDK1*	4	1.3194	2.15E-04	3.667661
CNN1	4	0.64717	0.012658	1.897632	*LIPT2*	2	1.3493	2.15E-04	3.667661
SLC16A1	4	-1.0153	0.015612	1.806547	*GCLM*	4	1.4688	2.15E-04	3.667661
ABCE1	4	1.2552	0.015612	1.806547	*DNM1L*	4	1.4846	2.15E-04	3.667661
MPC1	4	0.69477	0.015653	1.805405	*LIAS*	4	1.6754	2.15E-04	3.667661
CLUH	3	-1.0277	0.015691	1.804346	*RPS27A*	1	2.1434	2.15E-04	3.667661
MCUR1	4	-1.7683	0.018629	1.729811	*WARS2*	4	0.71969	6.14E-04	3.211729
AFG3L2	4	1.2026	0.018629	1.729811	*MRPL36*	4	0.90871	6.14E-04	3.211729
FZD8	4	-0.69895	0.02169	1.663735	*GLS*	3	-0.70812	9.35E-04	3.029405
MAU2	4	0.88801	0.02169	1.663735	*C7orf26*	3	0.79273	9.35E-04	3.029405
HAUS5	4	1.1014	0.022355	1.650622	*MRPS33*	4	0.83518	9.35E-04	3.029405
CEPT1	4	0.54589	0.024485	1.611094	*RPL19*	2	1.9892	9.35E-04	3.029405
PCBP2	4	-0.95693	0.02669	1.573659	*GFM1*	4	0.67987	0.00118	2.928066
CIAO2A	4	-0.69744	0.02669	1.573659	*NDUFA6*	4	0.82878	0.00118	2.928066
REXO2	4	-0.58313	0.039646	1.401797	*GCLC*	4	0.84207	0.00118	2.928066
BRPF1	4	0.33443	0.039646	1.401797	*SDHAF2*	4	0.85921	0.00118	2.928066
CCDC137	4	0.62153	0.039646	1.401797	*COX18*	2	0.89344	0.00118	2.928066
TPK1	4	0.66268	0.039646	1.401797	*COX14*	4	0.70285	0.00129	2.889508
EGLN1	3	0.85858	0.039646	1.401797	*COA5*	3	0.71333	0.00129	2.889508
RAD9A	4	1.5185	0.039646	1.401797	*PTCD3*	4	0.8265	0.00129	2.889508
COQ7	4	-0.78976	0.039766	1.400492	*MRPS11*	4	0.83274	0.00129	2.889508

CHTOP	4	0.69502	0.039766	1.400492	*GATB*	4	0.83531	0.00129	2.889508
C1QBP	4	-0.7928	0.040367	1.393974	*COX15*	3	1.0415	0.00129	2.889508
TRIM32	4	-0.52005	0.040367	1.393974	*MRPL51*	4	1.2871	0.00129	2.889508
PRDM10	4	0.7656	0.040367	1.393974	*DHX15*	4	1.6331	0.00129	2.889508
SLFN11	4	0.10482	0.040841	1.388907	*ATL2*	2	2.1402	0.00129	2.889508
CENPJ	4	0.86569	0.044118	1.355385	*MFF*	4	0.74402	0.001525	2.816602
YEATS2	4	0.77954	0.044793	1.348789	*YARS2*	4	0.86319	0.001525	2.816602
PFKP	3	-0.86557	0.047692	1.321555	*UQCC2*	4	0.63719	0.001644	2.78417
TANK	4	0.53313	0.047692	1.321555	*NDUFA9*	3	0.77652	0.001644	2.78417
MCRIP2	4	-0.97756	0.057277	1.242019	*PPA2*	4	0.78638	0.001644	2.78417
E4F1	3	1.1688	0.059274	1.227136	*NDUFB6*	4	0.80279	0.001644	2.78417
ADRM1	4	0.58563	0.061682	1.209842	*BRPF1*	4	0.8095	0.001644	2.78417
PI4KB	4	0.70133	0.061682	1.209842	*ELOB*	4	1.7232	0.001644	2.78417
POP4	3	1.0475	0.061682	1.209842	*MRPS7*	4	0.63712	0.00172	2.764576
AASDHPPT	4	1.059	0.061782	1.20914	*SLC25A19*	4	0.67323	0.00172	2.764576
ECH1	4	0.46563	0.065999	1.180461	*MRPS16*	4	0.78566	0.00172	2.764576
CS	4	0.59671	0.07076	1.150213	*COX6B1*	4	0.92828	0.00172	2.764576
UBR4	4	0.62408	0.07076	1.150213	*TARS2*	4	0.93974	0.00172	2.764576
USF2	3	0.83335	0.07076	1.150213	*DLD*	3	1.0121	0.00172	2.764576
URI1	4	0.82333	0.071192	1.147569	*COA6*	3	1.0399	0.00172	2.764576
GDI2	3	0.95483	0.072066	1.142267	*EARS2*	4	1.0471	0.001874	2.727244
GGPS1	3	1.3047	0.07247	1.13984	*COPS5*	3	1.1841	0.001874	2.727244
STT3A	4	0.53177	0.075067	1.124553	*CIAO1*	4	1.3909	0.001874	2.727244
SF3B1	4	0.88476	0.083721	1.077163	*RB1*	3	-0.54281	0.002017	2.695332
RPS11	3	1.0618	0.083721	1.077163	*GATC*	3	1.1641	0.002017	2.695332
DCAF13	4	1.1315	0.083961	1.07592	*GTPBP4*	4	1.8149	0.002017	2.695332
SPTLC1	4	0.89484	0.088601	1.052563	*SARS2*	3	0.93092	0.002175	2.662461
MPI	3	0.61274	0.089607	1.047657	*NARS2*	3	0.9656	0.002175	2.662461
RPL27	3	0.43016	0.093658	1.028454	*PARS2*	4	0.82134	0.002354	2.628155
RPS16	4	0.7894	0.095279	1.021002	*UQCRC1*	4	0.60947	0.002471	2.607184
NAA20	4	1.0499	0.095279	1.021002	*PRC1*	4	0.88051	0.002471	2.607184
SGF29	4	0.48015	0.096123	1.017174	*NAPA*	2	1.1216	0.002471	2.607184
ERRFI1	4	0.61167	0.096123	1.017174	*METTL17*	3	0.79888	0.002608	2.583626
CDK1	4	1.084	0.096123	1.017174	*TUBGCP4*	3	1.0937	0.002608	2.583626
DDIT4	4	0.56049	0.100023	0.999901	*MRPL53*	4	0.65855	0.002681	2.571685
ANKRD28	2	-1.1637	0.10458	0.980553	*YEATS2*	4	0.93874	0.002681	2.571685
BHLHE41	4	0.48832	0.10458	0.980553	*CMPK1*	4	1.098	0.002681	2.571685
TIMM8A	4	-0.75789	0.105856	0.975286	*FPGS*	3	1.6118	0.002681	2.571685
IDH3A	4	0.94369	0.106561	0.972403	*MARS2*	4	0.9913	0.002836	2.547368
RPE	4	-1.1076	0.114232	0.942214	*NDUFAF6*	4	0.55167	0.00304	2.517171
SLC47A1	2	-1.0182	0.114232	0.942214	*MRM2*	3	0.69544	0.00304	2.517171
FECH	4	-0.80399	0.114232	0.942214	*MRPS9*	4	0.70588	0.00304	2.517171
BTRC	4	0.57115	0.114232	0.942214	*CENPW*	3	0.96064	0.00304	2.517171
ATXN1L	4	0.61773	0.114232	0.942214	*AFG3L2*	4	0.98544	0.00304	2.517171
SLC7A5	4	0.65854	0.114232	0.942214	*ATPAF2*	4	0.75379	0.00332	2.478866
TNRC18	4	0.66013	0.114232	0.942214	*UBA2*	4	0.7689	0.00332	2.478866
NSMCE3	3	0.71919	0.114232	0.942214	*MTO1*	4	0.7543	0.003423	2.465639
MARCHF5	4	0.74952	0.114232	0.942214	*GPX4*	3	1.0293	0.003423	2.465639
SEC62	3	0.77066	0.114232	0.942214	*PDZD4*	4	-0.54297	0.003886	2.410532
SPTSSA	4	1.0232	0.114232	0.942214	*CHCHD1*	4	0.72328	0.004012	2.396604
H3C11	4	0.63432	0.114666	0.940564	*HSD17B10*	3	0.83177	0.004098	2.387406
VPS51	4	0.78343	0.114666	0.940564	*RPL27*	3	1.1074	0.004098	2.387406
ATAD3A	3	-0.93739	0.11556	0.937193	*RRN3*	3	1.2978	0.004219	2.374753
TIMELESS	3	1.2011	0.11556	0.937193	*MPC1*	4	0.6606	0.004812	2.317693
SSTR2	3	-0.59225	0.116855	0.932351	*TFAM*	4	0.84608	0.00488	2.311589
COG6	4	0.50497	0.11828	0.927089	*RPS25*	2	2.1334	0.00488	2.311589
ARID1B	4	0.24305	0.12437	0.905283	*MRPS14*	4	1.0006	0.005297	2.275981
TGFBRAP1	4	0.70993	0.12437	0.905283	*IDH3A*	4	1.0921	0.005506	2.25919

CIAPIN1	3	1.1154	0.12437	0.905283	*UBE2I*	4	1.2692	0.005506	2.25919
KAT8	3	0.066395	0.131084	0.882451	*RMND1*	4	0.53877	0.005756	2.239848
ZNF418	4	0.45109	0.131084	0.882451	*SHMT2*	4	0.6443	0.005756	2.239848
SELENOV	4	0.64597	0.131084	0.882451	*MRPL34*	4	0.80084	0.005756	2.239848
YPEL5	4	0.81155	0.131084	0.882451	*SLC25A51*	2	0.9277	0.005756	2.239848
DCAF11	3	-0.56097	0.133015	0.876099	*HCCS*	4	0.48377	0.005804	2.236298
GPT	2	-1.1444	0.137352	0.862165	*H2AC6*	3	0.8936	0.005804	2.236298
ATP8B3	4	0.84585	0.139345	0.85591	*TRRAP*	3	0.79369	0.006182	2.208872
HLA-DMA	4	-0.7812	0.140598	0.852021	*SLFN11*	4	0.21358	0.006361	2.196467
SYNE4	4	0.36064	0.144156	0.841166	*MRPS23*	4	0.70035	0.006361	2.196467
CCDC59	3	1.1632	0.144156	0.841166	*UQCR10*	4	0.78063	0.006587	2.181298
KNTC1	4	0.45264	0.146566	0.833968	*CSTF3*	4	0.87727	0.006672	2.175746
PMVK	3	0.60928	0.146566	0.833968	*MRPL13*	4	0.66618	0.006702	2.173766
ZNF564	4	0.64608	0.146566	0.833968	*NDUFB10*	4	0.74919	0.006702	2.173766
ACSL3	4	0.69742	0.146566	0.833968	*TIMM9*	4	0.85384	0.006784	2.168493
WASHC5	2	0.94733	0.146566	0.833968	*MRRF*	4	0.67148	0.007132	2.146814
SLC35E1	3	0.96917	0.151919	0.818387	*RPAIN*	3	0.53487	0.007417	2.12979
PITPNM1	4	0.61036	0.152337	0.817195	*NDUFA8*	3	0.76337	0.007417	2.12979
ANKRD52	3	-0.71242	0.152615	0.816402	*CLPX*	4	0.85083	0.007881	2.103395
WDR18	4	0.99121	0.155476	0.808338	*CYC1*	4	0.82091	0.00802	2.095799
ABHD17B	4	-0.58363	0.156367	0.805854	*RCC1L*	2	1.5474	0.00802	2.095799
PHF6	4	0.50737	0.160305	0.795053	*COPZ1*	3	1.426	0.008343	2.07867
ARHGEF10L	4	-0.32054	0.160851	0.793576	*MTERF1*	3	0.64706	0.00866	2.062468
RPL37	3	0.23856	0.160851	0.793576	*IMP4*	4	0.99808	0.00866	2.062468
SPATA2	3	0.61734	0.163858	0.785532	*TUT1*	4	1.0578	0.00866	2.062468
VPS52	4	0.78073	0.163858	0.785532	*COX7B*	2	0.80274	0.008722	2.059392
C18orf65	4	-0.52176	0.165002	0.782511	*SSBP1*	3	1.1473	0.009028	2.04441
SLC6A13	4	0.5542	0.165002	0.782511	*FARS2*	3	0.81649	0.009086	2.041637
DBR1	3	0.56861	0.165002	0.782511	*NDUFS6*	4	0.69984	0.009385	2.027563
UPK2	4	0.57566	0.165002	0.782511	*DBR1*	3	-0.16	0.00944	2.025037
MAGEB2	3	0.62316	0.165002	0.782511	*MRPL2*	4	0.69538	0.009732	2.011778
UTP20	4	0.65824	0.165002	0.782511	*MRPS30*	4	0.7267	0.009818	2.007981
GGN	4	0.69236	0.165002	0.782511	*RPUSD4*	4	0.73309	0.009818	2.007981
PRC1	4	0.71262	0.165002	0.782511	*POT1*	4	0.76108	0.009818	2.007981
MRPL52	3	0.72016	0.165002	0.782511	*FDX1*	2	1.0253	0.009818	2.007981
JTB	4	0.75294	0.165002	0.782511	*COQ7*	4	0.60593	0.010098	1.995747
HMGCS1	4	0.88167	0.165002	0.782511	*NDUFB9*	4	0.81347	0.010238	1.989766
GADD45GIP1	4	1.0077	0.165002	0.782511	*POLR2K*	4	0.87638	0.010238	1.989766
KDSR	4	1.0395	0.165002	0.782511	*SUPT16H*	4	1.1165	0.010238	1.989766
PHAX	4	0.14515	0.167023	0.777223	*MRPS12*	4	0.78429	0.010552	1.976657
FOXA2	4	0.3891	0.167023	0.777223	*HAUS5*	4	1.0013	0.010552	1.976657
CUL3	4	0.81511	0.167023	0.777223	*MRPS18A*	4	0.73706	0.010595	1.9749
GPR1	4	-0.45053	0.167937	0.774855	*GGPS1*	3	1.3593	0.010637	1.973173
BRIP1	4	0.50836	0.170044	0.769438	*SPATA5*	4	0.85746	0.011117	1.954002
TLCD4	1	1.329	0.173104	0.761693	*NDUFB7*	4	0.6011	0.011194	1.951032
POU2AF1	4	0.51665	0.173204	0.761441	*ATIC*	3	0.71206	0.011194	1.951032
AMFR	4	0.52284	0.173402	0.760947	*N6AMT1*	4	0.73567	0.011375	1.944045
EIF3I	4	0.55212	0.173402	0.760947	*DDX20*	4	1.1626	0.011375	1.944045
EI24	4	0.91411	0.178382	0.74865	*DYNLRB1*	4	0.80288	0.011377	1.943989
NBN	4	0.6244	0.179982	0.74477	*RPLP0*	4	1.0576	0.011377	1.943989
C19orf57	2	0.84656	0.179982	0.74477	*MRPS2*	3	1.12	0.011377	1.943989
STK11	4	0.86131	0.181183	0.741882	*WDR12*	3	1.3984	0.01162	1.934779
COQ10B	3	-0.93606	0.181231	0.741768	*RUVBL2*	4	0.88395	0.011687	1.932289
ERI3	4	-0.43008	0.181897	0.740173	*CHMP4B*	4	0.89844	0.011687	1.932289
GMPPB	4	0.80671	0.184807	0.733282	*MRPL52*	3	0.76237	0.012641	1.898213
QARS1	4	1.3841	0.186308	0.729768	*RSL1D1*	4	0.50846	0.01338	1.873533
DYNC1LI2	4	0.62181	0.186391	0.729575	*OXA1L*	3	-0.87231	0.013647	1.864969
RAB5B	4	0.67134	0.186391	0.729575	*SLC25A26*	4	0.61283	0.013647	1.864969

POLA1	2	1.1085	0.188601	0.724455	*MRPL57*	4	1.1787	0.013647	1.864969
RAP2A	4	0.41005	0.190502	0.720101	*GNL3*	4	1.2821	0.013667	1.864313
ANKRD9	4	0.48638	0.196636	0.706336	*COQ2*	4	0.72062	0.013885	1.857444
USP14	4	0.49393	0.196636	0.706336	*NDUFS2*	3	0.80048	0.013904	1.856855
NEK8	4	0.59434	0.196636	0.706336	*TAF6L*	4	0.4932	0.01396	1.855122
SRP72	4	0.50997	0.198918	0.701326	*ARMH3*	4	0.65949	0.01396	1.855122
PAK4	4	0.74742	0.203883	0.690619	*PMVK*	3	1.0171	0.01396	1.855122
P2RY4	3	-0.67899	0.207169	0.683676	*ALAS1*	4	0.54253	0.014074	1.851589
ADCYAP1R1	3	-0.62906	0.207511	0.682959	*EXOC3*	4	0.64241	0.014378	1.842308
SAMD8	4	0.33759	0.207511	0.682959	*MCUR1*	4	-1.332	0.014488	1.83898
NRIP1	4	0.46424	0.207511	0.682959	*SLC25A32*	4	0.79846	0.015082	1.821549
CAND1	4	0.69459	0.207511	0.682959	*MRPL12*	3	1.0227	0.015082	1.821549
NDUFA7	4	0.74789	0.207511	0.682959	*MAN2C1*	4	0.442	0.01528	1.81587
YKT6	4	0.76655	0.207511	0.682959	*MRPS18B*	4	0.51875	0.015476	1.810328
SRP54	4	0.8072	0.207511	0.682959	*DTYMK*	4	0.66769	0.015486	1.810071
GALNS	4	0.50911	0.209424	0.678973	*NDUFV1*	3	0.71394	0.015695	1.804246
PCDH12	4	0.62159	0.210145	0.67748	*CDC16*	4	0.90508	0.015695	1.804246
GART	4	0.69035	0.212041	0.67358	*KLF5*	4	0.93562	0.015695	1.804246
ABCB7	4	-1.3695	0.21329	0.671029	*MRPL44*	4	0.75959	0.016065	1.794129
TOX3	4	-0.55572	0.21329	0.671029	*SRP54*	4	0.75883	0.016431	1.784344
G6PD	3	-0.4558	0.21329	0.671029	*ACD*	4	0.57494	0.016614	1.779528
MIP	4	-0.44175	0.21329	0.671029	*PTPMT1*	4	0.78005	0.016795	1.774826
GABPB1	4	-0.38035	0.21329	0.671029	*DARS1*	4	0.97944	0.017418	1.759008
VPS39	4	0.38998	0.21329	0.671029	*NDUFB4*	3	0.48649	0.018112	1.742028
SAMD11	4	0.49337	0.21329	0.671029	*YAE1*	3	0.7761	0.018112	1.742028
RUSC1-AS1	4	0.50183	0.21329	0.671029	*DPH1*	3	0.82096	0.018112	1.742028
C19orf67	3	0.55034	0.21329	0.671029	*RPS19*	4	0.98051	0.018608	1.730294
NOB1	4	0.56901	0.21329	0.671029	*GCSH*	2	1.0253	0.018608	1.730294
LIG1	4	0.61896	0.21329	0.671029	*ATP5PD*	2	1.1987	0.018608	1.730294
RFWD3	3	0.67446	0.21329	0.671029	*CDC27*	2	1.3792	0.018608	1.730294
NAPA	2	0.86881	0.21329	0.671029	*COX7C*	4	0.87087	0.019963	1.699778
SMC5	3	0.88242	0.21329	0.671029	*ANKRD49*	3	0.88343	0.020104	1.696724
CDC40	3	0.91469	0.21329	0.671029	*RPL3*	3	1.0725	0.020104	1.696724
RBM33	3	0.99721	0.21329	0.671029	*DYNC1LI2*	4	0.62293	0.02016	1.695516
CTU1	3	1.0675	0.21329	0.671029	*TRAPPC11*	4	0.7469	0.02016	1.695516
PSMA4	3	1.2778	0.21329	0.671029	*CAPRIN1*	3	0.84787	0.020976	1.678283
GCLM	4	0.53227	0.216319	0.664906	*MAD2L2*	3	0.77123	0.021041	1.67694
MAP3K6	2	-0.82962	0.217988	0.661567	*PET117*	2	0.98887	0.022914	1.639892
KDM4A	3	-0.77495	0.219825	0.657924	*CCDC28B*	4	0.55141	0.023664	1.625912
UFL1	4	-1.0352	0.220811	0.655979	*MRPL16*	3	0.61819	0.023664	1.625912
CTCFL	4	-0.51428	0.221879	0.653884	*MFN2*	4	0.69175	0.023664	1.625912
ATL2	2	1.4541	0.224796	0.648212	*MRPL40*	3	0.796	0.023664	1.625912
NUDT10	3	-0.44141	0.226163	0.645579	*EGLN1*	3	1.1423	0.023664	1.625912
DDX39A	4	0.45486	0.227428	0.643156	*MCAT*	4	0.63771	0.024879	1.604165
HMGCLL1	3	-0.56462	0.227864	0.642325	*COX4I1*	3	1.2962	0.024879	1.604165
MAGEA8	4	-0.56355	0.227864	0.642325	*MARCHF5*	4	0.74952	0.025323	1.596487
WNT6	4	0.32905	0.23243	0.633709	*STK11*	4	0.92239	0.025323	1.596487
EIF3L	4	-0.89612	0.233322	0.632044	*GADD45GIP1*	4	1.0622	0.025323	1.596487
AIP	4	-0.46071	0.23351	0.631694	*NDUFB5*	3	0.9319	0.02552	1.593121
CIZ1	3	-0.61496	0.233624	0.631482	*COPB2*	3	1.0845	0.02665	1.5743
LRRTM4	3	-0.63975	0.237061	0.625141	*NFYC*	3	0.63994	0.027028	1.568179
SUV39H1	3	-0.67225	0.240237	0.619361	*VPS45*	3	1.3761	0.027028	1.568179
DLK2	3	-0.34482	0.260657	0.583931	*MRPL30*	4	0.61975	0.028135	1.550747
TMEM234	1	1.1762	0.260657	0.583931	*MCMBP*	4	0.80002	0.028927	1.538692
PABPC1L	3	-0.93681	0.263439	0.57932	*GBF1*	4	0.59939	0.029904	1.52427
RPS25	2	1.5034	0.263439	0.57932	*MBTPS1*	4	0.80267	0.029904	1.52427
FGF12	2	-0.57198	0.263783	0.578754	*PSMD6*	4	0.98533	0.029904	1.52427
EOGT	3	0.63934	0.264207	0.578056	*ZKSCAN5*	3	-0.60125	0.0312	1.505842

CARS1	3	0.51851	0.267947	0.57195	*NDUFA11*	3	0.92605	0.031215	1.505643
PAK2	3	1.5222	0.267947	0.57195	*BUD31*	4	0.6673	0.031675	1.499279
BUB1	2	1.2077	0.268059	0.57177	*KAT8*	3	0.37973	0.031772	1.497954
DCAF12L1	4	-0.4832	0.274804	0.560977	*GTPBP6*	4	0.67633	0.031772	1.497954
HSP90B1	3	0.65096	0.275472	0.559922	*SOX2*	4	-0.54825	0.031788	1.497733
WDR63	3	0.51915	0.276637	0.558089	*NDUFA1*	4	0.4876	0.031788	1.497733
SFMBT1	3	-0.43602	0.28176	0.550121	*UQCRC2*	4	0.66303	0.031788	1.497733
CATSPERB	4	0.55874	0.28176	0.550121	*HNRNPA2B1*	4	0.67506	0.031788	1.497733
PDHX	3	0.58984	0.28176	0.550121	*NUBPL*	3	0.71861	0.031788	1.497733
RFC2	4	0.63805	0.28176	0.550121	*EIF4G1*	4	0.88411	0.031788	1.497733
DAZAP2	3	0.70197	0.28176	0.550121	*RPL28*	4	1.1733	0.031788	1.497733
DOP1B	4	-0.45708	0.282022	0.549717	*PSMA6*	2	1.6755	0.031788	1.497733
TELO2	4	0.59416	0.284366	0.546122	*DNTTIP2*	4	0.48838	0.032224	1.491815
PANK4	2	0.78587	0.284366	0.546122	*TRAIP*	4	0.63076	0.032305	1.490734
YTHDC1	4	0.82494	0.285256	0.544765	*NUP133*	3	0.7753	0.032454	1.488736
NOL4L	3	-0.58281	0.289162	0.538859	*TOP1MT*	3	0.85725	0.032462	1.488624
OPTN	4	-0.16545	0.289162	0.538859	*IFT81*	3	-0.93483	0.032539	1.487594
HNRNPU	3	1.352	0.289162	0.538859	*MCM5*	4	0.90922	0.033312	1.477393
NHP2	4	0.5614	0.292993	0.533143	*PSMB5*	4	0.79336	0.033387	1.476425
MMUT	3	-0.57754	0.295061	0.530088	*KMT2A*	2	0.76052	0.03422	1.465722
ABHD2	3	-0.47484	0.296704	0.527677	*PURG*	3	-0.94884	0.03477	1.458792
PTPMT1	4	0.4156	0.297137	0.527043	*HMBS*	4	0.69199	0.035446	1.450431
SACM1L	4	0.59499	0.297137	0.527043	*MRPS21*	3	0.77315	0.035446	1.450431
ZNF638	3	-0.86494	0.29716	0.52701	*OSBPL9*	2	1.2102	0.035446	1.450431
MTA2	4	0.45167	0.29716	0.52701	*NOP2*	2	1.9007	0.036253	1.440651
UBE2G2	4	0.46681	0.29716	0.52701	*NRF1*	3	0.86234	0.037326	1.427987
TET2	4	0.49555	0.29716	0.52701	*CIT*	4	0.71404	0.037558	1.425296
CCDC84	4	0.58699	0.29716	0.52701	*VARS2*	4	0.92706	0.037558	1.425296
GSTO1	4	0.63568	0.29716	0.52701	*UQCC1*	3	1.0638	0.037558	1.425296
CRKL	4	0.46983	0.297724	0.526187	*SNAPIN*	4	0.44043	0.037666	1.424051
KIN	4	0.76901	0.297724	0.526187	*GSS*	4	0.53883	0.037666	1.424051
TSPAN19	4	-0.51305	0.298427	0.525162	*MRPL14*	3	0.88943	0.038444	1.415168
ICA1L	4	0.37674	0.298427	0.525162	*CERS2*	3	0.93391	0.038625	1.413126
ORC3	4	0.81129	0.298427	0.525162	*ZBTB11*	4	0.44787	0.038658	1.412757
GINS2	4	1.0368	0.298427	0.525162	*USE1*	3	0.85273	0.038658	1.412757
ARL15	4	-0.72114	0.303184	0.518294	*SPDYE1*	3	0.55272	0.039226	1.406427
CRTAP	4	0.31858	0.303184	0.518294	*TAF4*	4	0.39412	0.040049	1.397413
SNF8	4	0.56792	0.303184	0.518294	*HNRNPU*	3	-0.13426	0.040671	1.390712
ADD2	4	-0.46974	0.303339	0.518071	*SLC7A5*	4	0.69803	0.041225	1.384839
PSMC2	4	0.88237	0.307127	0.512682	*SCAP*	4	1.0076	0.042003	1.376718
FAM181B	2	0.93414	0.309113	0.509883	*RPL10A*	3	1.2459	0.042003	1.376718
S100A3	2	0.33965	0.310954	0.507304	*NDUFS3*	4	0.47052	0.04201	1.376645
WDR25	3	0.85026	0.313486	0.503782	*EPC2*	4	0.70117	0.04201	1.376645
ADO	4	-0.96409	0.316128	0.500137	*MRPS22*	4	0.46649	0.042486	1.371757
CCNB1	3	0.060877	0.316128	0.500137	*POLR2B*	4	0.84607	0.043365	1.362862
ZNF571	3	0.85467	0.316128	0.500137	*GTF2B*	3	0.90447	0.043365	1.362862
PGD	4	-0.92848	0.316221	0.500009	*AHR*	4	-0.43886	0.04474	1.349303
ACACA	4	-1.0493	0.321143	0.493302	*ZNF365*	4	0.33825	0.04474	1.349303
KCNQ5	3	-0.4582	0.321143	0.493302	*PPIE*	4	0.47296	0.04474	1.349303
COMMD2	3	0.85466	0.321143	0.493302	*XRN1*	4	0.51783	0.04474	1.349303
ACOT13	4	-0.85758	0.331357	0.479704	*MRPS18C*	4	0.56506	0.04474	1.349303
MAPK6	3	-0.57942	0.331357	0.479704	*BRCA1*	4	0.56981	0.04474	1.349303
ZNF396	4	-0.41465	0.331357	0.479704	*MTIF3*	4	0.65252	0.04474	1.349303
CTCF	3	0.84825	0.331357	0.479704	*EIF3I*	4	0.68142	0.04474	1.349303
COP1	3	1.0444	0.331357	0.479704	*PHF5A*	4	0.90019	0.04474	1.349303
CELF6	3	-0.74519	0.333634	0.476729	*ACTR10*	2	1.3572	0.04474	1.349303
DDX51	4	0.45186	0.336156	0.473459	*DOT1L*	4	0.62254	0.044908	1.347677
LRRC56	3	0.49616	0.336156	0.473459	*SURF6*	4	0.8954	0.044908	1.347677

BANP	3	0.77797	0.336156	0.473459	*U2SURP*	4	0.78957	0.045169	1.34516
RFC3	4	1.1914	0.336156	0.473459	*LONP1*	4	0.49625	0.045747	1.339634
SLC26A6	3	-1.0096	0.336344	0.473216	*FOXM1*	4	0.80893	0.045747	1.339634
CYB5B	3	-0.85537	0.336344	0.473216	*TEFM*	3	0.58294	0.045749	1.339621
WDR13	4	-0.59741	0.336344	0.473216	*ARHGEF39*	4	0.63681	0.045749	1.339621
HIRA	4	-0.5844	0.336344	0.473216	*UTP25*	3	1.1103	0.045749	1.339621
TRPC4	4	-0.50491	0.336344	0.473216	*MDH2*	3	0.67929	0.045963	1.337596
NHSL2	4	-0.49621	0.336344	0.473216	*MTBP*	3	1.4005	0.045963	1.337596
NDUFB7	4	0.42223	0.336344	0.473216	*RPL7A*	3	0.36951	0.046153	1.335799
TMEM182	3	0.42339	0.336344	0.473216	*MRPL55*	2	0.64488	0.046153	1.335799
ADPRHL1	3	0.47625	0.336344	0.473216	*MRPS5*	4	0.69794	0.046153	1.335799
TSEN34	3	0.48238	0.336344	0.473216	*SYMPK*	2	1.0154	0.046153	1.335799
GTF3C4	4	0.48332	0.336344	0.473216	*ENTPD3*	3	-0.70449	0.047161	1.326416
TADA3	4	0.50457	0.336344	0.473216	*NSF*	4	0.82317	0.047161	1.326416
CRLF1	4	0.53752	0.336344	0.473216	*ATP7B*	4	-0.71411	0.047232	1.325761
GJC1	4	0.55307	0.336344	0.473216	*PCGF6*	4	0.41443	0.048155	1.317361
BLM	3	0.57788	0.336344	0.473216	*FAM111A*	3	-1.2305	0.049071	1.309175
KMT2A	2	0.6101	0.336344	0.473216	*TEDC1*	3	0.54821	0.049439	1.305934
FUBP1	4	0.61568	0.336344	0.473216	*ZNF341*	3	0.63233	0.049439	1.305934
SLCO4A1	4	0.62544	0.336344	0.473216	*PET100*	3	0.66761	0.049439	1.305934
TSPAN18	4	0.64448	0.336344	0.473216	*NDUFB11*	3	0.7826	0.049439	1.305934
ACTR6	4	0.65527	0.336344	0.473216	*KIDINS220*	4	0.8144	0.049452	1.305813
POLR1E	4	0.72104	0.336344	0.473216	*DNAJC11*	3	1.0258	0.049452	1.305813
RBSN	3	0.86052	0.336344	0.473216	*MED8*	4	0.77741	0.050123	1.299962
STEAP3	2	0.86586	0.336344	0.473216	*NUP88*	4	0.33072	0.051616	1.287218
ANAPC2	3	0.87415	0.336344	0.473216	*RABEP2*	4	-0.41452	0.05178	1.285836
SYMPK	2	0.8888	0.336344	0.473216	*INTS6*	4	0.57822	0.05178	1.285836
KLF5	4	0.88895	0.336344	0.473216	*MRPL37*	3	0.61416	0.053253	1.273653
PSMD14	4	0.91298	0.336344	0.473216	*NDUFA2*	4	0.65146	0.053357	1.272811
RPS2	3	0.91941	0.336344	0.473216	*TMEM120A*	3	0.91825	0.053677	1.270212
ACTR10	2	1.0142	0.336344	0.473216	*CCDC162P*	4	-0.59348	0.053757	1.269562
C1orf109	3	1.0441	0.336344	0.473216	*RBFA*	4	0.46362	0.053757	1.269562
AGAP11	1	1.2157	0.336344	0.473216	*PPRC1*	3	0.68873	0.053757	1.269562
CENPA	2	1.348	0.336344	0.473216	*SMC6*	4	0.75119	0.053757	1.269562
PCBP4	3	0.50349	0.339739	0.468854	*DHFR2*	3	0.64276	0.054018	1.267463
PEX14	4	0.52241	0.339739	0.468854	*MRPL35*	2	1.0911	0.054703	1.261989
LAMTOR2	4	0.60235	0.340337	0.468091	*LRRC24*	4	0.5626	0.055064	1.259129
CAMK2D	3	-0.68099	0.344321	0.463036	*MRPL32*	3	0.6262	0.055583	1.255059
HPCAL4	4	-0.60507	0.344891	0.462318	*EIF2B3*	4	0.65767	0.056098	1.251051
NXPE2	3	0.32296	0.344891	0.462318	*MTHFD1*	4	0.36093	0.057259	1.242154
GINS1	3	0.46441	0.344891	0.462318	*KCND1*	3	0.59499	0.057259	1.242154
SHKBP1	4	0.27042	0.345509	0.46154	*INTS10*	4	1.0278	0.057259	1.242154
CMPK1	4	0.30511	0.345509	0.46154	*SF3B1*	4	0.75973	0.057451	1.240705
CYB561D2	4	0.63443	0.345509	0.46154	*SSB*	2	0.93773	0.058472	1.233055
RB1	3	-0.36335	0.346739	0.459997	*ELOC*	1	1.1999	0.058606	1.232056
EXOC7	4	0.59088	0.349889	0.45607	*ZFAT*	4	0.51956	0.059567	1.224996
RLIM	4	-0.39424	0.351032	0.454653	*MRPL48*	3	0.56287	0.061447	1.211498
BIRC2	4	0.50212	0.351032	0.454653	*NDUFC1*	4	0.53238	0.062234	1.205972
REEP1	3	-0.3151	0.351109	0.454558	*TRMT12*	3	0.64642	0.062234	1.205972
COMMD6	3	-0.66553	0.351437	0.454153	*DCTN4*	4	0.73402	0.062234	1.205972
FBXO36	4	-0.62425	0.351437	0.454153	*TERF2*	4	0.74602	0.062234	1.205972
TMPRSS2	2	-0.50146	0.351437	0.454153	*MRPL4*	3	0.84851	0.062234	1.205972
PCIF1	4	-0.48537	0.351437	0.454153	*KIF11*	4	0.92362	0.062234	1.205972
ATP6V1FNB	4	-0.42661	0.351437	0.454153	*NME6*	4	0.74881	0.062607	1.203378
OR13D1	4	-0.3639	0.351437	0.454153	*GMPPB*	3	0.73567	0.062625	1.203255
CLDN5	3	0.17454	0.351437	0.454153	*MRPL54*	4	0.5361	0.062661	1.203005
ADAT3	3	0.38685	0.351437	0.454153	*POLD2*	3	1.2855	0.062661	1.203005
CENPK	4	0.42212	0.351437	0.454153	*NMT1*	4	0.77882	0.063276	1.19876

IP6K2	4	0.52755	0.351437	0.454153	*MTG2*	3	0.71077	0.06543	1.184226
XRCC2	3	0.54881	0.351437	0.454153	*RRM1*	2	1.119	0.067074	1.173443
SMIM1	2	0.71913	0.351437	0.454153	*RNF126*	4	0.60226	0.067327	1.171812
YARS1	4	0.75755	0.351437	0.454153	*MTHFD2*	4	0.63506	0.067626	1.169885
MCAT	4	-0.37344	0.352048	0.453398	*MRPS6*	3	0.762	0.067777	1.168918
TECTA	3	0.57722	0.354793	0.450025	*LYRM2*	4	0.25422	0.068663	1.163277
CCDC18	3	0.73253	0.354793	0.450025	*MRPL9*	4	0.45802	0.068663	1.163277
URB1	3	0.64479	0.359217	0.444644	*ZNF148*	4	0.5957	0.068663	1.163277
OR2A42	1	-0.55279	0.36163	0.441735	*POLR1E*	4	0.75714	0.068663	1.163277
CRK	3	-0.9253	0.365681	0.436897	*DPH3*	4	0.81042	0.068663	1.163277
SUMF2	3	-0.58717	0.365681	0.436897	*MRPS34*	3	0.9445	0.068712	1.16297
PON3	3	-0.12085	0.366106	0.436393	*COG6*	4	0.26101	0.070293	1.153088
TP53	4	-0.73312	0.368274	0.433828	*RPS16*	4	0.49041	0.070293	1.153088
WTAP	3	-0.034936	0.368274	0.433828	*KDSR*	4	0.59648	0.070293	1.153088
LIG3	2	-0.35593	0.373515	0.427692	*NSMCE3*	3	0.32274	0.070892	1.149404
FBXO42	3	0.80279	0.37486	0.426131	*ACAD8*	4	0.37985	0.070892	1.149404
RRAGD	2	-0.70719	0.376013	0.424798	*DHX30*	4	0.61752	0.070892	1.149404
IGF2BP1	4	-0.6569	0.377203	0.423425	*CDH1*	3	0.69192	0.070892	1.149404
ATRNL1	4	-0.45731	0.379185	0.421149	*NOP10*	4	0.6363	0.072162	1.141689
PNN	4	0.69604	0.379467	0.420826	*DIMT1*	4	0.91021	0.0722	1.141463
SNRPD1	2	1.0201	0.380446	0.419707	*SNRPG*	1	2.0107	0.072661	1.1387
LKAAEAR1	4	-0.45285	0.381846	0.418112	*MED6*	4	0.91862	0.074055	1.130444
COX5A	4	-0.63878	0.382119	0.417801	*NDUFAF8*	4	0.56647	0.075082	1.124465
POR	4	-0.51829	0.385945	0.413475	*MRPL49*	3	0.8196	0.075082	1.124465
SCYL2	4	-0.31813	0.385945	0.413475	*ALG2*	3	1.1939	0.075082	1.124465
TBCC	4	0.44812	0.385945	0.413475	*PSMB11*	4	0.60828	0.075158	1.124027
CSTF3	4	0.53797	0.385945	0.413475	*CCDC84*	4	0.86885	0.075417	1.122528
BRCA1	4	0.79227	0.385945	0.413475	*TMPRSS4*	4	0.4597	0.077176	1.11252
MPC2	1	0.93357	0.387534	0.41169	*SDHA*	4	0.62271	0.077176	1.11252
GIPC1	2	-1.0167	0.389737	0.409229	*NKAPD1*	4	0.23178	0.077566	1.110329
NDC1	4	-0.075257	0.389737	0.409229	*METTL14*	4	0.57845	0.078777	1.103599
ASPG	4	0.45976	0.389737	0.409229	*PRIM1*	4	0.74768	0.079343	1.100489
TFE3	3	-0.97043	0.390992	0.407832	*PSMC2*	4	0.62967	0.080771	1.092744
DAB2IP	4	-0.61972	0.390992	0.407832	*MTHFD1L*	4	0.74948	0.081648	1.088057
MAGOHB	2	-0.60132	0.390992	0.407832	*SEC24C*	4	0.50811	0.081973	1.086332
PRSS8	4	-0.578	0.390992	0.407832	*F11*	3	0.52045	0.081973	1.086332
ABCC1	4	-0.57091	0.390992	0.407832	*MRPS24*	3	0.78585	0.081973	1.086332
GZMM	4	-0.53741	0.390992	0.407832	*MRPL33*	3	0.96386	0.081973	1.086332
GAPVD1	4	-0.46866	0.390992	0.407832	*ATP6V1B2*	3	1.3556	0.081973	1.086332
ATP7B	4	-0.44413	0.390992	0.407832	*MPI*	3	0.59384	0.082652	1.082745
ACRBP	3	-0.43538	0.390992	0.407832	*UBR4*	4	0.44945	0.082973	1.081065
ELP3	4	-0.40538	0.390992	0.407832	*BIRC2*	4	0.34779	0.083366	1.079013
HDAC5	4	-0.35356	0.390992	0.407832	*JTB*	4	0.54631	0.083366	1.079013
MYO9B	4	0.32533	0.390992	0.407832	*RIOK1*	4	0.69406	0.083366	1.079013
RAB3GAP2	4	0.48822	0.390992	0.407832	*DDX6*	4	0.71468	0.083366	1.079013
MAML2	4	0.52709	0.390992	0.407832	*PSMG3*	4	0.80281	0.083366	1.079013
PLCD3	3	0.68956	0.390992	0.407832	*HSPA9*	4	0.62745	0.083674	1.077408
SAMD9	3	0.6978	0.390992	0.407832	*PRR36*	4	0.46898	0.08399	1.075774
PRKAG3	4	0.76426	0.390992	0.407832	*SIPA1*	4	-0.66402	0.084748	1.07187
IMP4	4	0.79234	0.390992	0.407832	*FZD8*	4	-0.31753	0.084748	1.07187
ATP6V1A	4	0.84161	0.390992	0.407832	*NUF2*	3	0.63231	0.084748	1.07187
FIP1L1	4	0.86404	0.390992	0.407832	*SINHCAF*	3	0.63894	0.084748	1.07187
FANCM	3	0.95572	0.390992	0.407832	*SEPTIN7*	3	0.86185	0.084748	1.07187
INO80C	4	0.31499	0.391272	0.407521	*SUPT6H*	2	1.3484	0.085272	1.069193
TSC2	4	0.45098	0.391272	0.407521	*DIDO1*	4	0.69505	0.085354	1.068775
SYF2	3	-0.12578	0.391319	0.407469	*ARL16*	4	0.38525	0.085619	1.067428
MARK2	4	0.060907	0.391319	0.407469	*AIFM1*	4	0.71785	0.0857	1.067018
NDUFB9	4	0.34815	0.391319	0.407469	*API5*	4	0.3171	0.086217	1.064408

MIR205HG	2	0.5639	0.391319	0.407469	*NDUFS5*	4	0.6438	0.086217	1.064408
OCRL	3	0.65471	0.391319	0.407469	*TXNL4B*	4	0.8657	0.086509	1.062941
GFPT1	4	0.97204	0.391319	0.407469	*NCOA4*	4	-0.65843	0.087271	1.05913
RPL10	2	1.0998	0.391319	0.407469	*NDUFB2*	3	0.59249	0.088791	1.051632
ELP4	3	1.1029	0.391319	0.407469	*CCDC62*	3	-0.19967	0.088856	1.051311
COPZ1	3	0.3779	0.391429	0.407347	*CENPL*	4	0.63912	0.088856	1.051311
P4HA3	3	0.47498	0.391429	0.407347	*POTEF*	2	0.71541	0.090657	1.042598
CERS1	4	0.49939	0.391429	0.407347	*ATP2B1*	4	0.39338	0.090685	1.042463
VKORC1L1	4	0.58024	0.391429	0.407347	*RPL37*	3	1.439	0.090738	1.042211
MRTO4	4	0.6924	0.391429	0.407347	*NUDC*	3	1.5693	0.090738	1.042211
CIAO1	4	0.89068	0.391429	0.407347	*RPAP1*	1	2.3136	0.090738	1.042211
SUPT6H	2	1.006	0.391429	0.407347	*COASY*	4	0.61819	0.090973	1.041086
TMEM185B	3	-0.35782	0.393182	0.405406	*TNFRSF25*	4	0.32638	0.09223	1.03513
FERMT2	4	0.63908	0.393182	0.405406	*ARF6*	2	1.3152	0.09223	1.03513
RANGAP1	4	1.0869	0.394748	0.403681	*MTREX*	4	0.54114	0.092337	1.034622
NOL6	4	0.70114	0.395406	0.402957	*NCAPD2*	4	0.88146	0.092445	1.034117
POLR1C	3	0.78809	0.398069	0.400041	*IMMT*	3	0.57688	0.092468	1.034008
ZNF574	4	0.52302	0.398876	0.399162	*TLE3*	3	0.3455	0.092476	1.033969
PWWP3B	4	0.42435	0.399929	0.398017	*MTRES1*	4	0.46783	0.092476	1.033969
NR2C2AP	3	0.78318	0.400531	0.397364	*NGRN*	4	0.36436	0.092582	1.033471
ATP5F1B	3	-0.77892	0.401816	0.395973	*MVD*	4	0.50036	0.093282	1.030204
GABARAP	2	-0.73316	0.402329	0.395419	*CCT6A*	3	0.99789	0.093282	1.030204
COL12A1	3	-0.37219	0.402839	0.394869	*MRPL42*	4	0.61146	0.093331	1.029975
MTMR9	4	0.38376	0.403206	0.394473	*TAMM41*	4	0.8013	0.093331	1.029975
RINL	4	0.51241	0.403206	0.394473	*NDUFB3*	4	0.32413	0.093856	1.027538
SCAF11	4	0.71266	0.403206	0.394473	*FNTA*	4	0.63212	0.093856	1.027538
ZNRD1	4	0.87885	0.403206	0.394473	*FNDC10*	2	-0.1984	0.094777	1.023299
SRSF7	4	0.84453	0.405227	0.392301	*MYBL2*	3	0.59022	0.094777	1.023299
CRYBG3	4	0.41136	0.409501	0.387745	*ALAD*	3	0.84897	0.094794	1.02322
ALDH1A3	1	-0.89482	0.420677	0.376051	*RPS17*	2	1.3428	0.094855	1.022939
MAPK4	4	-0.71783	0.420677	0.376051	*NDUFS8*	4	0.52538	0.094916	1.02266
YWHAZ	1	-0.69303	0.420677	0.376051	*DDX3X*	3	0.046858	0.09499	1.022322
NME3	4	-0.66186	0.420677	0.376051	*GOLGA6D*	1	0.8267	0.09499	1.022322
DEK	4	-0.58804	0.420677	0.376051	*AGAP11*	1	1.2085	0.09499	1.022322
CPXM2	4	-0.41912	0.420677	0.376051	*TXN*	3	1.3788	0.09499	1.022322
SAR1A	4	0.33014	0.420677	0.376051	*MRPS17*	3	0.55001	0.095363	1.02062
ECSIT	3	0.39373	0.420677	0.376051	*VKORC1L1*	4	0.51018	0.096891	1.013718
CTPS1	4	0.40426	0.420677	0.376051	*DHX38*	4	0.71691	0.096891	1.013718
OAZ1	3	0.40743	0.420677	0.376051	*TSC22D2*	4	0.78307	0.096891	1.013718
WDR26	4	0.42996	0.420677	0.376051	*PAK2*	3	1.4247	0.096982	1.013309
GSK3B	4	0.45453	0.420677	0.376051	*GPR1*	4	-0.42973	0.097798	1.009672
SLC41A3	4	0.49876	0.420677	0.376051	*PCLO*	4	0.64158	0.097798	1.009672
CEP295	4	0.508	0.420677	0.376051	*VAPA*	3	0.7706	0.097798	1.009672
ADGRA2	3	0.53449	0.420677	0.376051	*GLRX5*	4	0.40695	0.098937	1.004643
DDX20	4	0.57093	0.420677	0.376051	*RAD1*	4	0.58398	0.098937	1.004643
DDX41	4	0.6261	0.420677	0.376051	*NIPBL*	3	0.88712	0.099406	1.002587
PGGT1B	4	0.65448	0.420677	0.376051	*TMEM229B*	4	-0.63645	0.099914	1.000373
DERL1	3	0.7421	0.420677	0.376051	*SFPQ*	4	1.522	0.099914	1.000373
ZNF526	4	0.80637	0.420677	0.376051	*CHIC2*	4	0.56994	0.099936	1.000276
SNRNP200	4	-0.94878	0.421003	0.375715	*DDX28*	4	0.62767	0.099936	1.000276
GIT1	3	-0.83859	0.421003	0.375715	*HSD17B12*	3	0.80152	0.099936	1.000276
ABCB6	4	-0.69221	0.421003	0.375715	*MIGA2*	4	0.42936	0.100091	0.999605
ZC3H12B	4	-0.62243	0.421003	0.375715	*GINS3*	4	0.56308	0.100091	0.999605
SMIM22	4	-0.5867	0.421003	0.375715	*SCO1*	4	0.69131	0.100091	0.999605
MED23	4	-0.53158	0.421003	0.375715	*MRPL1*	2	0.87692	0.100091	0.999605
PHKG1	3	-0.53035	0.421003	0.375715	*WNT10A*	1	0.98169	0.100091	0.999605
FUT9	3	-0.48302	0.421003	0.375715	*SNRPA1*	1	1.855	0.100091	0.999605
ARHGEF28	4	-0.46689	0.421003	0.375715	*YY1*	3	0.70864	0.100591	0.997441

IL12RB2	3	-0.44468	0.421003	0.375715	*ZNF217*	3	0.97114	0.100591	0.997441
CPPED1	4	-0.43879	0.421003	0.375715	*PI4KB*	4	0.41291	0.100646	0.997204
PLOD2	4	-0.42265	0.421003	0.375715	*CARS1*	3	0.42641	0.100646	0.997204
NR5A2	4	-0.40106	0.421003	0.375715	*CHCHD5*	4	0.54212	0.100646	0.997204
PKD2L1	4	-0.39956	0.421003	0.375715	*CDC6*	3	1.1279	0.100646	0.997204
MZF1	3	-0.388	0.421003	0.375715	*ACTL6A*	4	0.32053	0.103268	0.986032
ZNF292	4	-0.37101	0.421003	0.375715	*ABT1*	4	0.74388	0.104297	0.98173
KIAA0556	4	-0.35755	0.421003	0.375715	*B4GALT3*	3	0.66524	0.104388	0.981351
FMR1NB	4	-0.33037	0.421003	0.375715	*SYNE4*	4	0.25702	0.104546	0.980693
GHR	4	-0.31581	0.421003	0.375715	*CAP1*	4	0.44122	0.106189	0.973921
FLT4	4	-0.29299	0.421003	0.375715	*ITSN2*	3	0.56461	0.106255	0.973652
SMARCD1	4	-0.27609	0.421003	0.375715	*COPS8*	4	0.54833	0.106406	0.973033
PLAA	3	-0.12144	0.421003	0.375715	*TFB2M*	4	0.76222	0.106406	0.973033
LY86	4	-0.092907	0.421003	0.375715	*DDX46*	4	0.71569	0.106409	0.973023
RET	4	0.17766	0.421003	0.375715	*CNTNAP1*	4	0.77022	0.106409	0.973023
NUDT8	4	0.24241	0.421003	0.375715	*YPEL5*	4	0.45031	0.10644	0.972895
CCND1	4	0.27048	0.421003	0.375715	*PNPT1*	4	0.64429	0.109271	0.961496
ZNF628	4	0.31604	0.421003	0.375715	*SDHB*	4	0.84155	0.109976	0.958702
VPS11	4	0.32208	0.421003	0.375715	*TMEM242*	4	0.34039	0.110683	0.955919
ACD	4	0.33046	0.421003	0.375715	*DPH5*	4	0.75365	0.110683	0.955919
HSBP1	4	0.33332	0.421003	0.375715	*DMAC1*	3	0.81698	0.110683	0.955919
GRAMD1A	3	0.3503	0.421003	0.375715	*FAM98C*	4	0.62953	0.111095	0.954304
TLE6	4	0.36235	0.421003	0.375715	*NUDT3*	2	-0.56255	0.111264	0.953646
ZNF793	4	0.37751	0.421003	0.375715	*PHF6*	4	0.49265	0.111264	0.953646
CMTM4	4	0.3913	0.421003	0.375715	*TIMMDC1*	4	0.49653	0.111264	0.953646
KEAP1	4	0.41779	0.421003	0.375715	*EXOSC7*	3	0.50545	0.112424	0.94914
NIBAN3	4	0.46554	0.421003	0.375715	*PPP2R1A*	4	0.56785	0.112424	0.94914
RXFP3	3	0.47669	0.421003	0.375715	*LAMC3*	4	0.3307	0.112648	0.948276
KANSL3	4	0.49577	0.421003	0.375715	*PFDN2*	4	0.69315	0.112648	0.948276
FADD	4	0.52137	0.421003	0.375715	*SNRNP27*	4	0.79522	0.112648	0.948276
RFXAP	4	0.52262	0.421003	0.375715	*HAGH*	4	0.61384	0.1156	0.937042
NUFIP1	4	0.53637	0.421003	0.375715	*MRPL43*	2	1.0218	0.11589	0.935953
NRBP1	4	0.56228	0.421003	0.375715	*ACTR2*	4	0.56397	0.116467	0.933796
THOC2	4	0.56675	0.421003	0.375715	*THOC2*	4	0.63324	0.116467	0.933796
CTDSPL2	4	0.60213	0.421003	0.375715	*GTF3C2*	2	0.82647	0.117063	0.93158
IGFLR1	3	0.62191	0.421003	0.375715	*GAR1*	4	0.42909	0.118036	0.927987
CHMP1B	2	0.64523	0.421003	0.375715	*RMI1*	4	0.38481	0.118626	0.92582
TBP	3	0.64581	0.421003	0.375715	*RPL14*	2	1.9506	0.119284	0.923417
METTL14	4	0.65958	0.421003	0.375715	*VPS37A*	4	0.6405	0.121697	0.914722
ANXA8	2	0.70482	0.421003	0.375715	*MS4A1*	3	0.44357	0.121758	0.914502
ILK	3	0.71648	0.421003	0.375715	*TPT1*	3	0.55171	0.121758	0.914502
ACO2	3	0.72324	0.421003	0.375715	*TCP1*	3	1.0063	0.121758	0.914502
FLCN	2	0.72512	0.421003	0.375715	*NT5C*	3	0.6174	0.123392	0.908712
UTP25	3	0.76675	0.421003	0.375715	*SLC9A9*	2	-0.93906	0.123793	0.907304
SPATA5	4	0.80608	0.421003	0.375715	*PITRM1*	4	-0.55308	0.124552	0.904649
NDUFB1	1	0.81971	0.421003	0.375715	*AARS1*	4	0.49863	0.124552	0.904649
PLEKHG6	3	0.84151	0.421003	0.375715	*CAND1*	4	0.55011	0.124552	0.904649
EIF4G1	4	0.85048	0.421003	0.375715	*NOA1*	4	0.66992	0.124552	0.904649
SRSF3	3	0.87914	0.421003	0.375715	*NOP14*	4	1.0765	0.124552	0.904649
SLC6A11	1	0.88977	0.421003	0.375715	*SAMM50*	3	1.0942	0.124552	0.904649
MAK16	4	0.92666	0.421003	0.375715	*TRAPPC1*	3	1.1266	0.124552	0.904649
KATNB1	2	1.1222	0.421003	0.375715	*HK1*	4	0.29068	0.126797	0.896891
CCAR2	3	-0.6228	0.421268	0.375442	*PGK1*	4	0.54077	0.126797	0.896891
LIN7C	4	-0.51609	0.421268	0.375442	*TUBGCP3*	4	0.60303	0.126797	0.896891
COQ6	4	-0.49811	0.421268	0.375442	*PREB*	4	0.58059	0.12714	0.895716
FAM234A	4	-0.38959	0.421268	0.375442	*ZPR1*	4	0.66097	0.12714	0.895716
GUCD1	4	0.28397	0.421268	0.375442	*GCNA*	3	-0.82361	0.128157	0.892256
CNFN	4	0.30766	0.421268	0.375442	*MTERF4*	2	0.76503	0.128157	0.892256

MAG	4	0.42286	0.421268	0.375442	*DPY30*	2	1.2425	0.128157	0.892256
ARMH3	4	0.60751	0.421268	0.375442	*PFDN4*	3	-0.53641	0.128381	0.891499
NADSYN1	3	0.67929	0.421268	0.375442	*FOXRED1*	3	0.48856	0.128381	0.891499
SUGP1	2	0.69337	0.421268	0.375442	*CDC37*	2	1.0342	0.128381	0.891499
TERF2	4	0.69435	0.421268	0.375442	*TNFRSF10C*	4	-0.67634	0.128394	0.891454
ZNF596	3	0.71224	0.421268	0.375442	*CIAO2A*	4	-0.5291	0.128394	0.891454
RFXANK	3	0.75418	0.421268	0.375442	*NUCB1*	4	0.35508	0.128394	0.891454
CEMIP2	3	0.78656	0.421268	0.375442	*PEX14*	4	0.3601	0.128394	0.891454
VPS29	3	0.87563	0.421268	0.375442	*MRPL11*	3	0.40384	0.128394	0.891454
NSMCE4A	4	1.0379	0.421268	0.375442	*DHX37*	4	0.42449	0.128394	0.891454
DLX5	3	0.35032	0.423891	0.372746	*COX10*	4	0.45648	0.128394	0.891454
SMARCD3	2	0.57221	0.423891	0.372746	*POLR3K*	4	0.48853	0.128394	0.891454
FAM111A	3	-1.0514	0.427975	0.368582	*CWC25*	4	0.49024	0.128394	0.891454
TBL1XR1	3	-0.76738	0.427975	0.368582	*BECN1*	4	0.64623	0.128394	0.891454
RBM27	3	-0.75181	0.427975	0.368582	*GPKOW*	4	0.79586	0.128394	0.891454
PFKFB3	3	-0.70005	0.427975	0.368582	*PHB*	4	0.89831	0.128394	0.891454
TSPAN14	3	-0.67456	0.427975	0.368582	*TLCD4*	1	1.1332	0.128394	0.891454
ACTN1	3	-0.63677	0.427975	0.368582	*EXOC4*	2	1.2718	0.128394	0.891454
SCARB2	4	-0.54331	0.427975	0.368582	*RPE*	4	-0.58727	0.128542	0.890956
CHUK	4	-0.53035	0.427975	0.368582	*TMEM139*	3	-0.36166	0.128542	0.890956
PCBD1	4	-0.51457	0.427975	0.368582	*EIF2S1*	4	0.63048	0.128542	0.890956
ENPP2	3	0.122	0.427975	0.368582	*TRIR*	4	0.69047	0.128542	0.890956
SETD1B	4	0.24272	0.427975	0.368582	*HARS1*	3	1.0061	0.128542	0.890956
CTSK	4	0.27921	0.427975	0.368582	*MRPL45*	2	0.84638	0.128623	0.890683
C11orf71	4	0.35226	0.427975	0.368582	*KPNA2*	3	0.44801	0.128748	0.890259
DNM2	4	0.35239	0.427975	0.368582	*MSRB1*	3	0.50537	0.128748	0.890259
MON2	4	0.35685	0.427975	0.368582	*COX5A*	4	0.36584	0.130876	0.883141
UBA2	4	0.41204	0.427975	0.368582	*SERBP1*	2	0.85567	0.132178	0.878842
RAD23A	4	0.44749	0.427975	0.368582	*IER5*	4	-0.71596	0.132235	0.878654
SLMAP	4	0.49052	0.427975	0.368582	*ELAC2*	4	0.43894	0.132235	0.878654
MAP3K10	3	0.52809	0.427975	0.368582	*TTI1*	4	0.66972	0.132235	0.878654
YLPM1	4	0.54789	0.427975	0.368582	*FBXL14*	4	-0.39013	0.132279	0.878509
DENR	4	0.55276	0.427975	0.368582	*MALSU1*	4	0.57429	0.134387	0.871641
BAG5	3	0.58861	0.427975	0.368582	*NMD3*	4	0.57447	0.134387	0.871641
CTDNEP1	4	0.61418	0.427975	0.368582	*NUP93*	3	0.72651	0.134387	0.871641
STC2	3	0.61977	0.427975	0.368582	*ZFAND5*	3	0.63285	0.134669	0.870732
TIMM22	4	0.70764	0.427975	0.368582	*ANAPC11*	4	0.63027	0.134931	0.869889
S100A5	3	0.7591	0.427975	0.368582	*SEPHS1*	3	0.65737	0.134931	0.869889
TBCE	4	0.84522	0.427975	0.368582	*EXOSC10*	4	0.67602	0.135891	0.866809
COQ2	4	-0.4663	0.428255	0.368298	*CINP*	4	0.52668	0.135951	0.866618
C10orf95	3	-0.69714	0.428356	0.368196	*TKT*	4	-0.42041	0.136204	0.86581
OTOGL	4	-0.35558	0.428356	0.368196	*ATP5F1D*	4	0.4692	0.13639	0.865217
RRM1	2	0.26443	0.428356	0.368196	*EEF1A1*	1	1.5764	0.13639	0.865217
AUP1	3	0.58077	0.429521	0.367016	*SPTLC1*	4	0.51061	0.138772	0.857699
ZNF563	3	0.58382	0.429521	0.367016	*FASTKD2*	3	0.57232	0.138772	0.857699
TIMM10	4	0.62255	0.429521	0.367016	*NOC4L*	4	0.64799	0.13886	0.857424
PSMB6	3	1.1862	0.429521	0.367016	*LEAP2*	4	0.54129	0.139314	0.856005
KPTN	2	-0.92151	0.430854	0.36567	*ZNF586*	4	0.30779	0.139701	0.854802
GUCY1B1	4	-0.20129	0.430854	0.36567	*FBXO11*	4	0.62694	0.139701	0.854802
PMAIP1	3	-0.52834	0.430883	0.365641	*OR2D2*	4	-0.76859	0.13982	0.854431
TRIM52	3	0.50153	0.430883	0.365641	*ALG1*	4	0.68081	0.140773	0.85148
PHOSPHO2	2	-0.67854	0.43109	0.365432	*ACAD10*	3	0.85476	0.140773	0.85148
TRIR	4	0.46963	0.432646	0.363868	*NAA10*	4	0.54857	0.141213	0.850126
NDUFA11	3	0.71618	0.432646	0.363868	*RBM12*	4	0.67571	0.141213	0.850126
VPS33A	4	0.31554	0.434501	0.362009	*NKPD1*	2	0.53299	0.141267	0.849959
KCNJ13	4	-0.37038	0.434695	0.361815	*AP2M1*	4	0.62644	0.141553	0.849081
DDX21	3	0.78629	0.434695	0.361815	*NFS1*	4	0.87358	0.141553	0.849081
SLC20A1	4	-0.44769	0.434826	0.361684	*SLC35E2B*	3	0.48068	0.141634	0.848832

OR4C15	4	0.24229	0.434826	0.361684	*VRK1*	4	0.51326	0.141925	0.847941
DEFB1	2	-0.73804	0.436645	0.359872	*CLEC17A*	4	0.4246	0.142188	0.847138
NUDT3	2	-0.47327	0.436645	0.359872	*ZNF718*	3	0.40956	0.14319	0.844088
STPG2	4	-0.16803	0.436645	0.359872	*ARID1B*	4	0.54353	0.14319	0.844088
PSMG2	3	0.21777	0.436645	0.359872	*COA8*	3	0.59329	0.14319	0.844088
RABIF	4	0.28869	0.436645	0.359872	*CTNNBL1*	4	0.83341	0.14319	0.844088
RAB43	3	0.30847	0.436645	0.359872	*KRTAP17-1*	3	-0.51413	0.143825	0.842166
PNMA1	4	0.31015	0.436645	0.359872	*ZRSR2*	4	0.37785	0.144026	0.84156
ZNF791	4	0.35963	0.436645	0.359872	*BTBD10*	2	0.58839	0.144026	0.84156
YIPF2	4	0.42595	0.436645	0.359872	*PFDN6*	4	1.1153	0.144026	0.84156
AKIP1	3	0.43255	0.436645	0.359872	*TGFBRAP1*	4	0.52961	0.14434	0.840613
SLC1A5	4	0.43634	0.436645	0.359872	*CTPS1*	4	0.30382	0.144446	0.840294
LCTL	3	0.46387	0.436645	0.359872	*GSTO1*	4	0.32825	0.144446	0.840294
FAF2	4	0.47879	0.436645	0.359872	*KMT2B*	4	0.34563	0.144446	0.840294
CIC	4	0.48437	0.436645	0.359872	*TACC3*	4	0.43709	0.144446	0.840294
TPBGL	4	0.50518	0.436645	0.359872	*NFIA*	4	0.52276	0.144446	0.840294
MED18	4	0.53122	0.436645	0.359872	*C1QBP*	4	0.53634	0.144446	0.840294
COG5	4	0.68614	0.436645	0.359872	*CDAN1*	4	0.54555	0.144446	0.840294
TMEM30A	4	0.70016	0.436645	0.359872	*TSFM*	4	0.64165	0.144446	0.840294
CYP2S1	3	0.70271	0.436645	0.359872	*NDUFV2*	3	0.71642	0.144446	0.840294
ELOF1	4	0.72959	0.436645	0.359872	*SOX17*	3	0.73919	0.144446	0.840294
TRAPPC2L	4	0.74113	0.436645	0.359872	*RPL18*	3	1.3749	0.144446	0.840294
RBM28	3	0.75376	0.436645	0.359872	*ATP6V1E1*	2	0.94759	0.144605	0.839818
EIF4G2	2	0.75713	0.436645	0.359872	*RBM22*	4	0.73625	0.14475	0.839381
LAMTOR3	3	0.91344	0.436645	0.359872	*RPS5*	3	0.95216	0.14475	0.839381
RAD50	3	1.0919	0.436645	0.359872	*ZNF574*	4	0.49775	0.146465	0.834265
FBXW10	4	0.32168	0.438527	0.358003	*GTPBP8*	4	0.56866	0.146465	0.834265
NOC3L	3	0.74789	0.438527	0.358003	*MRPS28*	4	0.43363	0.148583	0.828031
CTAGE6	1	0.94041	0.439026	0.357509	*TBRG4*	4	0.36856	0.149924	0.824128
PFDN6	4	0.18564	0.439687	0.356857	*PALB2*	3	0.57579	0.153056	0.815151
MIS18A	3	0.2725	0.440964	0.355597	*SF3B3*	4	0.94135	0.153056	0.815151
BACH1	4	0.53746	0.440964	0.355597	*HAUS6*	4	0.59507	0.153706	0.813309
NR1I3	3	0.53904	0.440964	0.355597	*PSMD3*	3	1.0066	0.15447	0.811156
TRIM4	4	0.36883	0.442015	0.354563	*BAG5*	3	0.31759	0.154526	0.811
BCAP31	3	0.53232	0.442336	0.354248	*SLC25A3*	4	0.53197	0.155703	0.807703
SWSAP1	4	0.39387	0.442735	0.353856	*WFDC3*	3	-0.47213	0.155704	0.807699
MAST1	4	0.53445	0.444182	0.352439	*SUPV3L1*	2	0.83978	0.155758	0.807551
KDM1A	4	0.59557	0.444182	0.352439	*STAMBP*	3	-0.029323	0.155781	0.807486
FAU	4	0.60979	0.444182	0.352439	*VWCE*	3	0.47073	0.155781	0.807486
CEACAM21	4	0.45016	0.444274	0.352349	*RPS11*	3	0.91708	0.156424	0.805697
MB	3	0.61974	0.444274	0.352349	*DEDD2*	4	0.36958	0.157979	0.8014
MAN1A1	1	-1.5887	0.444946	0.351692	*P2RX7*	4	-0.51609	0.158495	0.799985
SLC25A28	3	-0.90226	0.444946	0.351692	*CPSF3*	3	1.0706	0.158495	0.799985
STOM	4	-0.61379	0.444946	0.351692	*SRSF7*	4	0.97666	0.16004	0.795772
DYNLL2	4	-0.58442	0.444946	0.351692	*GINS2*	4	0.66219	0.160842	0.793602
RELA	4	-0.55751	0.444946	0.351692	*POLA1*	2	0.76769	0.160842	0.793602
INSC	3	-0.5302	0.444946	0.351692	*COX20*	2	0.86246	0.160842	0.793602
COA4	2	-0.49172	0.444946	0.351692	*CA2*	4	-0.32792	0.162043	0.790369
COA1	4	-0.4787	0.444946	0.351692	*SCYL2*	4	-0.2765	0.162043	0.790369
ATPAF2	4	-0.46996	0.444946	0.351692	*NODAL*	4	0.50068	0.162043	0.790369
SUMO3	4	-0.46957	0.444946	0.351692	*DDX47*	3	1.0157	0.16294	0.787973
ANTXR1	3	-0.45285	0.444946	0.351692	*CTCF*	3	0.68852	0.163772	0.785759
SIN3B	4	-0.42821	0.444946	0.351692	*RTF1*	4	0.37609	0.164205	0.784614
AGT	4	-0.39245	0.444946	0.351692	*SRRD*	4	0.47063	0.164205	0.784614
FAT2	4	-0.36012	0.444946	0.351692	*CEP128*	4	-0.40958	0.164764	0.783138
ZCCHC8	4	-0.31284	0.444946	0.351692	*GPD1L*	4	-0.56582	0.165588	0.780971
SAP30L	4	-0.2613	0.444946	0.351692	*THOC1*	3	-0.49066	0.165771	0.780492
PLS3	4	-0.25901	0.444946	0.351692	*G6PD*	3	-0.4247	0.165771	0.780492

KIAA1324	3	-0.24945	0.444946	0.351692	*TOMM22*	3	0.67704	0.165942	0.780044
C6orf99	4	-0.21251	0.444946	0.351692	*TNRC18*	4	0.34869	0.165952	0.780017
THOC6	4	0.038429	0.444946	0.351692	*ST6GAL1*	4	-0.59378	0.167149	0.776897
PHLPP2	4	0.28504	0.444946	0.351692	*GLOD5*	4	-0.38212	0.167439	0.776143
SLC25A39	4	0.37365	0.444946	0.351692	*RING1*	3	0.32923	0.167439	0.776143
ATP6V1G3	4	0.38282	0.444946	0.351692	*SBNO2*	4	0.37011	0.167439	0.776143
RPP14	4	0.41192	0.444946	0.351692	*ATP6V1G1*	3	0.37234	0.167439	0.776143
SNRPB	4	0.4467	0.444946	0.351692	*ITPK1*	4	0.48199	0.167439	0.776143
DOT1L	4	0.47007	0.444946	0.351692	*RAD51D*	3	0.70341	0.167439	0.776143
XRCC4	4	0.5236	0.444946	0.351692	*RAE1*	3	0.77652	0.167439	0.776143
NUP85	4	0.53442	0.444946	0.351692	*C2CD4A*	2	-0.7107	0.167869	0.77503
HTATIP2	3	0.53595	0.444946	0.351692	*ARVCF*	4	-0.65025	0.167869	0.77503
EXOC8	3	0.55002	0.444946	0.351692	*HCN4*	4	0.37818	0.167869	0.77503
PLRG1	4	0.5617	0.444946	0.351692	*TRAPPC5*	3	1.2526	0.167869	0.77503
NOP10	4	0.58787	0.444946	0.351692	*GART*	4	0.63536	0.168348	0.773793
QRICH1	4	0.59161	0.444946	0.351692	*DDX41*	4	0.58196	0.168643	0.773031
FPGS	3	0.61796	0.444946	0.351692	*CCDC90B*	4	-0.47913	0.169311	0.771316
NDUFB6	4	0.62536	0.444946	0.351692	*LSM14B*	4	0.33719	0.170473	0.768345
SPDL1	4	0.66145	0.444946	0.351692	*PANK1*	3	0.57297	0.170473	0.768345
FAM86C1	1	0.67066	0.444946	0.351692	*PTBP1*	4	0.35739	0.171501	0.765733
S100A1	3	0.73083	0.444946	0.351692	*KAT2A*	4	0.49424	0.171501	0.765733
CDH1	3	0.8556	0.444946	0.351692	*UBAP2L*	4	0.55468	0.171658	0.765335
ADSL	4	0.95124	0.444946	0.351692	*YARS1*	4	0.77449	0.172155	0.764081
SRFBP1	3	0.95265	0.444946	0.351692	*CCDC115*	4	0.66547	0.172262	0.763811
PAFAH1B3	3	0.46843	0.445914	0.350749	*YWHAZ*	1	-0.72607	0.172547	0.763091
RAB18	4	0.64387	0.445914	0.350749	*MGMT*	3	-0.56722	0.172803	0.762449
CARD8	3	0.29124	0.447499	0.349208	*NAA20*	4	0.68169	0.172803	0.762449
RBM47	4	-0.53752	0.449511	0.34726	*PLEKHG3*	4	-0.57797	0.173139	0.761606
THBS2	4	-0.36506	0.449511	0.34726	*MTPAP*	3	0.96725	0.173139	0.761606
PSMG4	3	0.49968	0.449511	0.34726	*TRIM16L*	3	-0.073374	0.173887	0.759733
ATAD3C	3	0.85303	0.449511	0.34726	*MRPL27*	4	0.38389	0.174837	0.757367
GPBP1	2	-0.97981	0.449678	0.347099	*UROS*	4	0.39463	0.174837	0.757367
HSDL2	2	0.62411	0.449678	0.347099	*MEIOC*	4	0.39494	0.174837	0.757367
KRTAP1-3	1	0.99342	0.449678	0.347099	*BMP4*	4	0.43804	0.174837	0.757367
SNRPA1	1	1.6747	0.449678	0.347099	*MRM3*	2	0.54127	0.174837	0.757367
IMMT	3	0.24747	0.452182	0.344687	*HIC2*	4	0.60642	0.174837	0.757367
SMIM12	4	-0.62728	0.453222	0.343689	*UTP4*	4	0.7809	0.174837	0.757367
SAMD5	3	0.42705	0.453222	0.343689	*MRPL18*	3	0.39281	0.174885	0.757246
MCM3	3	0.49756	0.453222	0.343689	*URI1*	4	0.48931	0.175313	0.756186
PAIP2	2	0.6563	0.453222	0.343689	*PCIF1*	4	-0.27026	0.176506	0.753241
GRM4	3	-0.45288	0.454639	0.342333	*TTF1*	4	0.62418	0.177317	0.751249
PDLIM2	4	-0.31551	0.454884	0.3421	*GPR82*	4	-0.57916	0.177324	0.751233
PTK7	4	0.30282	0.454884	0.3421	*LKAAEAR1*	4	-0.53062	0.177324	0.751233
MAATS1	4	0.5089	0.454884	0.3421	*SPATC1*	4	0.28673	0.177324	0.751233
HEATR3	4	0.4646	0.455023	0.341967	*POLRMT*	3	0.81818	0.177324	0.751233
PRKCB	3	-0.47709	0.455292	0.34171	*ADSL*	4	0.82137	0.177324	0.751233
NYAP1	4	0.42612	0.459743	0.337485	*BICRA*	4	0.22384	0.177368	0.751124
KIF6	2	-0.58692	0.459814	0.337418	*NDUFA4*	3	0.42524	0.178872	0.747459
GSDMD	4	0.4086	0.459814	0.337418	*TBCC*	4	0.60062	0.179161	0.746757
ASB7	4	0.55806	0.459814	0.337418	*ART3*	3	-0.9341	0.181	0.742321
ELP5	4	0.52134	0.461323	0.335995	*BMF*	4	-0.54458	0.181	0.742321
CD101	3	-0.78124	0.462872	0.33454	*TDRP*	3	-0.51893	0.181	0.742321
FOXF1	1	-0.68606	0.462872	0.33454	*TMEM214*	4	0.34565	0.181	0.742321
LYPD8	3	-0.49878	0.462872	0.33454	*STK40*	4	0.35914	0.181	0.742321
PRSS42P	3	-0.43813	0.462872	0.33454	*ZNF793*	4	0.3764	0.181	0.742321
CELF1	3	0.043548	0.462872	0.33454	*AARS2*	4	0.42348	0.181	0.742321
DAZAP1	3	0.046277	0.462872	0.33454	*SLC7A6OS*	4	0.47141	0.181	0.742321
H2AC1	4	0.2515	0.462872	0.33454	*ARFGEF1*	4	0.48932	0.181	0.742321

NCAM1	4	0.40875	0.462872	0.33454	*EMG1*	4	0.50172	0.181	0.742321
ZNF837	4	0.6339	0.462872	0.33454	*CLP1*	4	0.51378	0.181	0.742321
SAC3D1	3	0.69843	0.462872	0.33454	*CSNK2B*	4	0.62251	0.181	0.742321
OTUB2	4	-0.57184	0.463524	0.333928	*HEATR1*	4	0.63718	0.181	0.742321
ASNSD1	4	-0.29263	0.463524	0.333928	*HNRNPM*	3	0.77063	0.181	0.742321
CDC37L1	3	-0.066246	0.463524	0.333928	*RPL4*	4	0.78371	0.181	0.742321
RACK1	4	0.07408	0.463524	0.333928	*TBL3*	4	0.45393	0.183174	0.737137
COLGALT1	3	0.13678	0.463524	0.333928	*ERRFI1*	4	0.35258	0.184668	0.733608
EMD	4	0.16204	0.463524	0.333928	*MS4A15*	4	0.41212	0.184668	0.733608
ZNF558	4	0.32365	0.463524	0.333928	*DMAP1*	4	0.53126	0.184668	0.733608
LYG2	4	0.33037	0.463524	0.333928	*PDX1*	3	-0.49462	0.186043	0.730386
PDE7A	3	0.34325	0.463524	0.333928	*NSMCE4A*	4	0.77257	0.186559	0.729185
ZCCHC10	3	0.34838	0.463524	0.333928	*TPX2*	4	0.40491	0.186914	0.728359
DLST	4	0.41201	0.463524	0.333928	*UBE2B*	4	0.50536	0.186914	0.728359
USF1	4	0.44344	0.463524	0.333928	*KIF9*	4	0.52475	0.186914	0.728359
TPPP3	4	0.44853	0.463524	0.333928	*SPC24*	3	0.67767	0.186914	0.728359
CIT	4	0.45428	0.463524	0.333928	*NELFA*	3	0.97827	0.186914	0.728359
G3BP1	4	0.53202	0.463524	0.333928	*MUC6*	1	1.3371	0.187936	0.725991
FAM71A	4	0.59558	0.463524	0.333928	*NDUFS7*	4	0.60727	0.187936	0.725989
TRMT10A	4	0.61273	0.463524	0.333928	*RNF20*	3	0.55614	0.188058	0.725708
NHLRC2	4	0.62486	0.463524	0.333928	*SERINC1*	3	0.030524	0.1883	0.72515
NAA40	3	0.62647	0.463524	0.333928	*LIG1*	4	0.35738	0.18893	0.723699
MFF	4	0.69589	0.463524	0.333928	*TELO2*	4	0.60335	0.18905	0.723424
ACHE	3	0.89408	0.463524	0.333928	*CARHSP1*	3	-0.46298	0.189913	0.721445
DDX54	4	0.52937	0.465922	0.331687	*VPS52*	4	0.75778	0.190151	0.720901
INTS1	3	1.0065	0.465922	0.331687	*SNRPB*	4	0.24485	0.190575	0.719934
BARX2	4	0.19943	0.467049	0.330638	*ATXN7L3*	4	0.48852	0.190575	0.719934
CHRFAM7A	2	0.43107	0.468645	0.329156	*COA3*	3	0.52123	0.190575	0.719934
IKZF4	4	0.52736	0.468645	0.329156	*CNOT4*	4	0.55677	0.190575	0.719934
CLNS1A	4	0.63157	0.468645	0.329156	*RFC5*	3	-0.4409	0.190946	0.719089
CAMK2B	3	-0.40568	0.471602	0.326425	*VWA3B*	3	-0.43307	0.190946	0.719089
KMT2B	4	0.34196	0.471602	0.326425	*CCDC59*	3	0.69961	0.190946	0.719089
INSIG2	4	0.38565	0.471602	0.326425	*EIF3A*	4	0.83887	0.190946	0.719089
TYW3	4	0.55454	0.471602	0.326425	*SSU72*	3	0.49801	0.192243	0.716149
PCDHA2	4	0.27344	0.472095	0.325971	*POLR1D*	4	0.8067	0.193374	0.713602
TEX30	2	-0.70685	0.47413	0.324102	*XRCC2*	3	0.34159	0.193459	0.71341
NUTM2G	2	-0.49404	0.47413	0.324102	*DNM2*	4	0.89184	0.194139	0.711887
PUS7L	3	-0.48741	0.47413	0.324102	*ANAPC2*	3	0.49743	0.194791	0.710431
SPATA31C1	3	-0.45718	0.47413	0.324102	*ACAD9*	4	0.37256	0.194961	0.710052
PALD1	4	-0.41773	0.47413	0.324102	*SNF8*	4	0.59343	0.194961	0.710052
RAB40C	4	-0.40188	0.47413	0.324102	*YEATS4*	4	0.49011	0.195213	0.70949
SDSL	3	-0.37346	0.47413	0.324102	*GOT2*	3	-0.66219	0.195474	0.708912
SLC22A2	4	-0.32459	0.47413	0.324102	*SEH1L*	3	0.77918	0.195477	0.708904
KDM4C	4	-0.23761	0.47413	0.324102	*C1orf210*	3	-0.44328	0.195916	0.707929
CD163	4	0.010051	0.47413	0.324102	*LY96*	4	-0.42483	0.195916	0.707929
KLF6	4	0.15936	0.47413	0.324102	*IL18*	3	0.19692	0.195916	0.707929
ABR	4	0.20071	0.47413	0.324102	*HNF1B*	4	0.31338	0.195916	0.707929
SLC25A41	3	0.33905	0.47413	0.324102	*RPRML*	4	0.47604	0.195916	0.707929
CWC25	4	0.35346	0.47413	0.324102	*MRPL28*	3	0.59829	0.195916	0.707929
ASPRV1	4	0.40742	0.47413	0.324102	*RAD23B*	4	0.61273	0.195916	0.707929
ATP10D	4	0.42375	0.47413	0.324102	*MST1L*	4	0.66596	0.195916	0.707929
HSD17B13	4	0.49201	0.47413	0.324102	*GPR137C*	4	-0.39757	0.196195	0.707312
GTF3A	3	0.58363	0.47413	0.324102	*ERCC3*	4	0.23699	0.196195	0.707312
RTBDN	3	0.60456	0.47413	0.324102	*CEL*	4	0.29329	0.196195	0.707312
XRCC5	4	0.67431	0.47413	0.324102	*DDX23*	4	0.53221	0.196195	0.707312
H3-3B	3	0.76152	0.47413	0.324102	*HACD2*	4	0.63044	0.196195	0.707312
HDAC3	4	0.80212	0.47413	0.324102	*PRMT7*	4	0.29803	0.196713	0.706168
UGT1A10	4	0.53426	0.476476	0.321959	*TERT*	2	-0.71494	0.19685	0.705865

CENPQ	4	-0.39991	0.477209	0.321292	*APC*	3	-0.48198	0.19685	0.705865
LYPD1	4	-0.14116	0.477209	0.321292	*NUDT10*	3	-0.3144	0.19685	0.705865
SLC34A1	3	0.63679	0.477209	0.321292	*SCN4A*	4	0.31577	0.19685	0.705865
ABCG5	2	0.67559	0.477209	0.321292	*PRELID1*	4	0.48019	0.19685	0.705865
GTSF1	3	0.38872	0.477253	0.321251	*IP6K2*	4	0.5464	0.19685	0.705865
NPAP1L	4	0.50546	0.477382	0.321134	*GTPBP10*	4	0.55512	0.19685	0.705865
SERPINC1	2	0.60516	0.47789	0.320672	*THOC5*	4	0.62878	0.19685	0.705865
TTI1	4	0.61978	0.478902	0.319754	*NBPF14*	2	0.67951	0.19685	0.705865
TUBG1	4	0.65716	0.479112	0.319563	*GPN2*	3	1.3971	0.197742	0.7039
CHFR	4	-0.52198	0.479238	0.319449	*DOLK*	4	1.0193	0.197776	0.703826
SERAC1	4	-0.47572	0.479238	0.319449	*DDX19A*	4	0.16637	0.198815	0.701551
WDR70	3	-0.096669	0.479779	0.318959	*ZNF623*	4	0.58579	0.198815	0.701551
KRT5	3	0.36455	0.479779	0.318959	*ACRBP*	3	-0.57215	0.198963	0.701229
C19orf38	4	0.49872	0.479779	0.318959	*CCNB1*	3	0.28994	0.198963	0.701229
CLASP1	4	0.49996	0.479779	0.318959	*EMC3*	8	0.29478	0.198963	0.701229
DNAJA2	3	0.2868	0.480028	0.318734	*FAM218A*	3	0.34116	0.198963	0.701229
PITHD1	4	-0.31147	0.480064	0.318701	*ABCF1*	4	0.71714	0.198963	0.701229
RBIS	4	-0.30438	0.480064	0.318701	*SNRNP200*	4	0.85511	0.198963	0.701229
LGALS12	4	-0.17541	0.480064	0.318701	*IL9R*	4	0.54435	0.200268	0.698389
VRK1	4	0.40059	0.480064	0.318701	*SMNDC1*	4	0.52993	0.201346	0.696057
PDCD2L	4	0.25491	0.480306	0.318482	*C11orf80*	4	0.48229	0.201957	0.694741
RBP2	3	0.47076	0.480306	0.318482	*PEBP1*	2	0.56884	0.201957	0.694741
LRTOMT	3	0.61554	0.480306	0.318482	*BRIX1*	4	0.97102	0.202163	0.694299
MZT1	2	0.84453	0.480306	0.318482	*DIS3*	4	1.0444	0.202635	0.693285
RPF1	4	0.46752	0.480344	0.318447	*MIS12*	4	0.60117	0.205012	0.68822
CWC15	4	0.47699	0.480344	0.318447	*PIH1D2*	4	0.53466	0.21015	0.677471
TAF5	4	-0.95927	0.480968	0.317884	*ATP7A*	4	-0.3378	0.210918	0.675886
ME2	4	-0.47471	0.480968	0.317884	*UBA1*	3	0.85752	0.213316	0.670976
DNALI1	4	-0.33834	0.480968	0.317884	*SMIM22*	4	-0.47184	0.213563	0.670473
SLC28A1	4	0.015145	0.480968	0.317884	*ACO2*	3	0.57122	0.213563	0.670473
KIAA1257	4	0.24489	0.480968	0.317884	*KCNC3*	4	0.29136	0.213707	0.670181
SLC22A20P	4	0.24983	0.480968	0.317884	*CENPA*	2	1.211	0.215367	0.66682
MAP7D1	4	0.2551	0.480968	0.317884	*RASA2*	4	0.42824	0.215598	0.666356
CAMSAP1	4	0.31779	0.480968	0.317884	*REL*	4	-0.5968	0.216217	0.665111
NXF1	4	0.32224	0.480968	0.317884	*ALG12*	3	-0.42099	0.216217	0.665111
SGO2	4	0.39721	0.480968	0.317884	*POP4*	3	0.99718	0.216217	0.665111
VWF	4	0.39865	0.480968	0.317884	*ATG9A*	4	-0.58633	0.216355	0.664833
RPL13	3	0.55956	0.480968	0.317884	*ALDH1A1*	4	-0.37871	0.216355	0.664833
GPR63	3	0.56628	0.480968	0.317884	*CST7*	4	0.043153	0.216355	0.664833
SF3A1	3	0.65028	0.480968	0.317884	*AAMP*	4	0.58763	0.216355	0.664833
FAM166A	4	0.33303	0.481933	0.317013	*SMU1*	4	0.62219	0.216355	0.664833
RAB2A	3	0.61807	0.48205	0.316908	*NOL6*	4	0.67706	0.216355	0.664833
OSTF1	4	-0.38156	0.482163	0.316806	*CENPK*	4	0.74893	0.216355	0.664833
TRPC4AP	4	0.14152	0.482163	0.316806	*GOLGA6L9*	1	1.2305	0.216355	0.664833
MYO7B	4	0.32718	0.482797	0.316236	*FOPNL*	4	-0.35689	0.216459	0.664625
MRPL2	4	0.55052	0.482797	0.316236	*CCDC74B*	3	0.2978	0.216459	0.664625
HSD17B14	4	0.3583	0.483152	0.315916	*FHAD1*	3	0.53305	0.216459	0.664625
CCL3	4	-0.43139	0.484253	0.314927	*TSR1*	4	0.70337	0.216459	0.664625
DNAJB5	4	0.096161	0.484253	0.314927	*PLAA*	3	0.18762	0.216668	0.664205
PRKAB1	4	0.45034	0.484253	0.314927	*NDUFS4*	4	0.57342	0.216668	0.664205
SRSF5	4	-0.39425	0.485942	0.313415	*SUZ12*	3	0.4815	0.216975	0.66359
FAM160B2	3	0.48504	0.485942	0.313415	*SCD*	4	0.637	0.217508	0.662525
NOL10	4	0.85521	0.485942	0.313415	*PRR16*	3	0.87531	0.217508	0.662525
B3GNT2	3	-0.77038	0.487568	0.311964	*TSEN15*	3	0.185	0.219007	0.659541
ACSL6	4	-0.36789	0.487568	0.311964	*UBP1*	4	-0.30343	0.220194	0.657194
UNC5CL	4	-0.33012	0.487568	0.311964	*SETD4*	4	0.56061	0.220194	0.657194
GEMIN4	4	-0.046287	0.487568	0.311964	*TOR3A*	4	-0.081432	0.221183	0.655247
RPL28	4	0.8951	0.489334	0.310394	*EXOC3L4*	3	0.64809	0.221183	0.655247

NECTIN2	4	0.14137	0.489953	0.309846	*SMC3*	4	0.92365	0.221183	0.655247
MUSK	2	-0.41591	0.493129	0.307039	*SNRPD3*	4	0.99886	0.221183	0.655247
FIBP	2	-0.26559	0.493308	0.306882	*PAXIP1*	4	0.57396	0.22123	0.655157
HNRNPL	4	-0.21477	0.493369	0.306828	*GGA1*	4	-0.57576	0.221358	0.654904
PDLIM4	1	-0.54159	0.493391	0.306809	*TSSK3*	4	-0.52123	0.221562	0.654505
FAAH	2	-0.81082	0.495215	0.305206	*FOXP1*	4	-0.46125	0.221562	0.654505
CNTLN	3	-0.52465	0.495682	0.304796	*SIN3B*	4	-0.39249	0.221562	0.654505
STX1B	3	-0.43292	0.495682	0.304796	*TMEM265*	4	-0.35387	0.221562	0.654505
ABCB10	4	-0.36864	0.495682	0.304796	*SPINK5*	3	-0.16429	0.221562	0.654505
KRR1	4	0.57167	0.495682	0.304796	*ITLN1*	4	0.16601	0.221562	0.654505
TMPO	4	0.09655	0.495914	0.304593	*OXSM*	4	0.40346	0.221562	0.654505
WDR82	3	0.57592	0.495914	0.304593	*DVL3*	3	0.40406	0.221562	0.654505
TMEM14C	3	-0.66089	0.496279	0.304274	*PEA15*	3	0.42536	0.221562	0.654505
CNOT4	4	0.16505	0.496279	0.304274	*RABGGTB*	3	0.42924	0.221562	0.654505
CHD9	4	0.31242	0.496279	0.304274	*SPRED3*	4	0.44714	0.221562	0.654505
BTK	4	0.48014	0.496603	0.303991	*DRICH1*	2	0.52033	0.221562	0.654505
ITPK1	4	0.50416	0.496603	0.303991	*KIF26B*	3	0.53673	0.221562	0.654505
ZBTB10	4	0.6436	0.496603	0.303991	*CDK2AP1*	3	0.63056	0.221562	0.654505
LUC7L2	1	-0.9004	0.500248	0.300814	*ATRNL1*	4	-0.34106	0.221938	0.653768
REXO1	4	-0.27497	0.500248	0.300814	*CAPS2*	4	-0.34495	0.221973	0.6537
SNX30	4	-0.20852	0.500248	0.300814	*ZKSCAN8*	4	-0.34247	0.221973	0.6537
ACP7	4	0.15723	0.500248	0.300814	*G3BP1*	4	0.47128	0.221973	0.6537
PAFAH1B1	4	0.27861	0.500248	0.300814	*FAM92B*	4	0.4862	0.221973	0.6537
PAGR1	4	0.29165	0.500248	0.300814	*PDPR*	4	0.57202	0.221973	0.6537
EPG5	4	0.30204	0.500248	0.300814	*CENPM*	4	0.79969	0.221973	0.6537
NUP155	4	0.30526	0.500248	0.300814	*TSPAN13*	4	-0.59254	0.222877	0.651936
PLEK2	4	0.33688	0.500248	0.300814	*ZNF180*	4	-0.44917	0.222877	0.651936
NXT1	4	0.42061	0.500248	0.300814	*RELCH*	4	-0.42675	0.222877	0.651936
TBCCD1	4	0.42565	0.500248	0.300814	*JPH1*	3	-0.16653	0.222877	0.651936
NAA10	4	0.66873	0.500248	0.300814	*GLTP*	4	0.15499	0.222877	0.651936
ACTR1A	4	0.79403	0.500248	0.300814	*MAP3K14*	4	0.3685	0.222877	0.651936
FBL	4	0.81419	0.500248	0.300814	*PPOX*	4	0.38494	0.222877	0.651936
PSMB4	4	0.98139	0.500248	0.300814	*CHTOP*	4	0.38899	0.222877	0.651936
UBE2I	4	1.0122	0.500248	0.300814	*ALMS1*	3	0.39825	0.222877	0.651936
COG3	4	-0.004507	0.501193	0.299995	*COPG1*	4	0.41433	0.222877	0.651936
DRC7	4	0.089356	0.501193	0.299995	*AP2S1*	4	0.42305	0.222877	0.651936
RBCK1	4	0.21451	0.501193	0.299995	*RPS6KC1*	3	0.43402	0.222877	0.651936
DCAF15	3	0.56173	0.501506	0.299724	*DNTTIP1*	4	0.45469	0.222877	0.651936
DYNLRB1	4	-0.14496	0.502376	0.298971	*PSMD11*	4	0.46561	0.222877	0.651936
OC10192757.	4	0.34118	0.502376	0.298971	*UQCRB*	3	0.49139	0.222877	0.651936
DCAF12	4	0.40091	0.502376	0.298971	*FERMT2*	4	0.5205	0.222877	0.651936
ARHGEF4	3	0.54868	0.502376	0.298971	*CD3EAP*	4	0.58527	0.222877	0.651936
GPI	2	-1.7587	0.503931	0.297629	*EIF2B1*	3	0.59071	0.222877	0.651936
POSTN	3	0.79352	0.503931	0.297629	*CENPC*	3	0.63215	0.222877	0.651936
EFNA2	3	0.45145	0.504725	0.296945	*ALYREF*	2	0.63911	0.222877	0.651936
FADS2	3	-0.62759	0.506055	0.295803	*DDX42*	4	0.65473	0.222877	0.651936
CCR7	4	-0.58516	0.506055	0.295803	*HIPK3*	3	0.68582	0.222877	0.651936
XPNPEP1	3	-0.56052	0.506055	0.295803	*DCTN5*	4	0.70698	0.222877	0.651936
CDKN1C	4	-0.51417	0.506055	0.295803	*RRP12*	3	1.5399	0.223279	0.651151
ZNF460	4	-0.36953	0.506055	0.295803	*KRT18*	1	-1.2743	0.223458	0.650804
SLC1A7	4	-0.024543	0.506055	0.295803	*UTP20*	4	0.57695	0.22406	0.649636
LRRC4B	4	0.38545	0.506055	0.295803	*CHRFAM7A*	2	0.37885	0.224504	0.648777
AASDH	4	0.49909	0.506055	0.295803	*MMADHC*	4	0.43796	0.224504	0.648777
PAPOLA	4	0.50779	0.506055	0.295803	*PDK3*	3	0.45381	0.224504	0.648777
BNIP2	2	0.81535	0.506055	0.295803	*RPP40*	4	0.46667	0.224504	0.648777
SPEM1	4	-0.39063	0.507353	0.29469	*CRNKL1*	4	0.49536	0.224504	0.648777
ATP5F1C	4	-0.6682	0.508325	0.293858	*PDE6G*	3	0.49739	0.224504	0.648777
ZRANB1	4	-0.48533	0.508325	0.293858	*MRTO4*	4	0.52692	0.224504	0.648777

TNS3	4	-0.37919	0.508325	0.293858	*MTIF2*	3	0.527	0.224504	0.648777
CLEC16A	4	0.23434	0.508325	0.293858	*CDC5L*	4	0.70731	0.224504	0.648777
RNF214	4	0.24847	0.50965	0.292728	*COX5B*	3	0.71303	0.224504	0.648777
H3C1	3	-0.61082	0.510752	0.29179	*POLR3B*	3	0.76704	0.224504	0.648777
CDC37	2	0.61038	0.510752	0.29179	*DNAJC9*	2	1.2206	0.224504	0.648777
WIF1	4	-0.18895	0.510788	0.291759	*PABPC4*	3	0.47245	0.224993	0.647831
KRT74	4	-0.41976	0.510789	0.291758	*MRPS26*	3	0.4833	0.224993	0.647831
LGR6	4	-0.17994	0.510789	0.291758	*AHNAK2*	3	0.68272	0.224993	0.647831
SECISBP2L	3	-0.13715	0.510789	0.291758	*ATG2A*	3	0.55816	0.22505	0.647721
DHX15	4	0.55943	0.510789	0.291758	*XRCC4*	4	0.30111	0.225687	0.646494
MAVS	3	-0.45608	0.513261	0.289662	*SLC29A4*	4	0.44467	0.225687	0.646494
CYP4B1	3	-0.31343	0.513261	0.289662	*TRMT61A*	2	0.5181	0.225687	0.646494
ZC3H4	3	-0.90756	0.513387	0.289555	*MBOAT2*	2	0.62338	0.225687	0.646494
MRPS36	3	-0.79554	0.513387	0.289555	*ORC3*	4	0.82874	0.225687	0.646494
FAM71D	2	0.16435	0.513387	0.289555	*DMXL2*	3	-0.9415	0.226734	0.644482
SOX17	3	0.3679	0.513387	0.289555	*DNPH1*	4	-0.4827	0.227233	0.643528
LRP5	4	0.42321	0.513387	0.289555	*H1-6*	3	0.54705	0.227233	0.643528
PRR35	2	0.45365	0.513387	0.289555	*METTL5*	4	-0.60018	0.227707	0.642624
ZNF446	3	0.73018	0.513387	0.289555	*RNASEH2C*	4	-0.41372	0.227707	0.642624
LIMD2	3	0.094143	0.514789	0.288371	*SLFN13*	4	0.44853	0.227707	0.642624
DHRS3	4	0.67438	0.515612	0.287677	*FKBPL*	4	0.045136	0.228453	0.641202
ALG13	2	0.4426	0.518795	0.285004	*OSR1*	4	0.4889	0.228453	0.641202
NAIF1	3	0.57296	0.518795	0.285004	*MRPL38*	4	0.6178	0.228453	0.641202
FOXP1	4	-0.56991	0.520149	0.283873	*PPA1*	4	0.805	0.228453	0.641202
ATG16L2	4	0.056296	0.520668	0.283439	*RANGAP1*	4	0.91238	0.228453	0.641202
METTL7A	4	0.41259	0.520668	0.283439	*FAM193A*	4	-0.43204	0.229163	0.639855
FXYD4	4	0.45944	0.520668	0.283439	*KCTD20*	4	-0.43789	0.229907	0.638448
BORCS6	3	0.50479	0.520668	0.283439	*DDX52*	4	0.40065	0.23182	0.634849
DDX6	4	0.56749	0.520668	0.283439	*TFE3*	3	-0.46258	0.232292	0.633966
TEAD4	3	0.019503	0.524188	0.280513	*ABCA5*	3	-0.89635	0.233592	0.631542
TMEM175	4	0.40689	0.524188	0.280513	*POLR2E*	3	-0.43665	0.233592	0.631542
PCF11	4	0.53407	0.524188	0.280513	*TREML1*	4	-0.51677	0.234405	0.630033
IFT122	4	0.16922	0.524873	0.279946	*IP6K1*	4	0.4865	0.235334	0.628314
MYH15	4	0.35783	0.524873	0.279946	*FBL*	4	0.79251	0.235334	0.628314
NMI	4	0.50896	0.524873	0.279946	*OR2AE1*	4	0.35123	0.235956	0.62717
PRDX2	3	0.64498	0.524873	0.279946	*HIGD2A*	4	0.46203	0.235956	0.62717
GATA6	4	0.40021	0.525089	0.279767	*SEPHS2*	4	0.25923	0.236016	0.627058
ATP13A1	3	0.41198	0.525089	0.279767	*SEMA6B*	3	0.19408	0.236027	0.627037
LAT2	3	0.10567	0.528509	0.276948	*PROZ*	2	0.67135	0.236027	0.627037
SRL	3	0.54957	0.528509	0.276948	*LPL*	4	-0.68666	0.236412	0.626331
PRIM1	4	0.04405	0.528751	0.276749	*SLC30A1*	1	-1.0645	0.236756	0.625699
ACP5	4	0.52473	0.528751	0.276749	*FAR2*	3	-0.43225	0.236763	0.625686
BNC1	4	0.38815	0.529258	0.276332	*C6orf89*	4	-0.57729	0.236971	0.625305
SMC6	4	0.62041	0.530746	0.275113	*PDE8B*	4	-0.31977	0.236971	0.625305
NAP1L3	4	0.075183	0.531109	0.274816	*INPP4B*	4	-0.2551	0.236971	0.625305
ST6GAL1	4	-0.50596	0.533253	0.273067	*NIPSNAP3B*	4	0.04555	0.236971	0.625305
ZMYND8	4	-0.50568	0.533253	0.273067	*EIF4EBP2*	4	0.213	0.236971	0.625305
LURAP1L	4	-0.42885	0.533253	0.273067	*LAMTOR1*	4	0.33566	0.236971	0.625305
ZNF385A	4	0.36967	0.533253	0.273067	*LSM5*	4	0.48089	0.236971	0.625305
ADAM17	4	-0.3694	0.536955	0.270062	*DR1*	4	0.48683	0.236971	0.625305
C18orf63	2	-0.69933	0.538408	0.268888	*COPA*	3	0.5244	0.236971	0.625305
UPF3B	4	-0.32653	0.538408	0.268888	*UGT1A10*	4	0.52749	0.236971	0.625305
CCDC117	3	0.53628	0.538408	0.268888	*RARS2*	4	0.53567	0.236971	0.625305
PINLYP	3	0.67269	0.538408	0.268888	*ALDOA*	4	0.58576	0.236971	0.625305
NT5C	3	0.9034	0.538408	0.268888	*RBBP8*	4	0.60479	0.236971	0.625305
OR2A2	4	-0.61447	0.540561	0.267155	*ATP5ME*	2	0.60691	0.236971	0.625305
CHP1	4	-0.53889	0.540561	0.267155	*TMEM31*	2	-0.87713	0.237347	0.624616
CNTFR	4	-0.30543	0.544188	0.264251	*YBEY*	3	0.23803	0.237688	0.623993

MS4A15	4	0.33467	0.544188	0.264251	*DAD1*	2	1.3468	0.240773	0.618392
TIPARP	4	0.49307	0.54808	0.261156	*ATL3*	4	-0.35031	0.241644	0.616825
SLC17A9	4	-0.59856	0.54828	0.260997	*CYB5R4*	4	-0.54287	0.242327	0.615599
MAN2C1	4	0.3984	0.54828	0.260997	*OBSL1*	3	-0.43796	0.242327	0.615599
STRA6	3	0.40354	0.54828	0.260997	*MRPS36*	3	-0.71418	0.243732	0.613088
SYTL1	4	0.42005	0.54828	0.260997	*SEC24D*	4	-0.45432	0.245107	0.610644
DDX19B	4	0.52591	0.54828	0.260997	*UGGT1*	4	0.15898	0.245107	0.610644
SPC24	3	0.73809	0.54828	0.260997	*EIF4E*	2	0.65484	0.246883	0.607508
UBA52	3	0.82705	0.54828	0.260997	*UQCRQ*	3	0.72674	0.246883	0.607508
PSMC4	2	-1.2791	0.548625	0.260725	*BTN3A3*	4	-0.34632	0.246934	0.60742
HHATL	3	-0.80656	0.548625	0.260725	*RPS7*	2	0.86652	0.248342	0.60495
FBRS	3	-0.67791	0.548625	0.260725	*METTL16*	3	0.75008	0.248738	0.604258
MAP3K7CL	3	-0.56312	0.548625	0.260725	*LSM11*	4	0.54038	0.249389	0.603123
FBXO25	3	-0.53378	0.548625	0.260725	*PRPF38A*	4	0.44635	0.249599	0.602756
C1QL1	3	-0.50633	0.548625	0.260725	*H2BC21*	1	-1.4818	0.250285	0.601565
SLC16A8	3	-0.46726	0.548625	0.260725	*MFSD8*	4	0.35629	0.250328	0.60149
BVES	4	-0.46157	0.548625	0.260725	*FSTL4*	2	0.78314	0.254646	0.594063
NEURL3	2	-0.44955	0.548625	0.260725	*TCEAL4*	4	0.26158	0.254871	0.593679
VTCN1	3	-0.42862	0.548625	0.260725	*ZNF74*	4	0.42275	0.254871	0.593679
SLC9A3R2	2	-0.42011	0.548625	0.260725	*BTNL9*	4	0.29638	0.255555	0.592516
XDH	4	-0.36925	0.548625	0.260725	*PTPN23*	4	0.5422	0.255555	0.592516
SKAP1	4	-0.33861	0.548625	0.260725	*SRP72*	4	0.57058	0.255555	0.592516
TMEM143	4	-0.28057	0.548625	0.260725	*PTPN11*	1	1.0417	0.255555	0.592516
DCLRE1C	3	-0.2628	0.548625	0.260725	*TMEM208*	4	-0.73139	0.255797	0.592104
MRPL9	4	-0.18852	0.548625	0.260725	*DDAH2*	4	-0.53028	0.255797	0.592104
PPM1A	3	-0.081955	0.548625	0.260725	*KCNJ11*	4	-0.51569	0.255797	0.592104
SLC10A2	3	0.29	0.548625	0.260725	*MYOM3*	4	-0.43303	0.255797	0.592104
NFIB	4	0.32907	0.548625	0.260725	*FANCF*	3	0.37624	0.255797	0.592104
CD22	3	0.36465	0.548625	0.260725	*CENPJ*	4	0.46315	0.255797	0.592104
CD86	3	0.3668	0.548625	0.260725	*NXT1*	4	0.48554	0.255797	0.592104
KLHL35	4	0.37912	0.548625	0.260725	*ARL10*	4	0.51772	0.255797	0.592104
PCDH8	3	0.38342	0.548625	0.260725	*TEDC2*	2	0.56327	0.255797	0.592104
XRRA1	2	0.45603	0.548625	0.260725	*GOSR1*	2	0.70763	0.255797	0.592104
POTEF	2	0.45906	0.548625	0.260725	*SAP30BP*	3	1.0402	0.255797	0.592104
PEX5	4	0.45925	0.548625	0.260725	*TMEM131L*	3	-0.49229	0.257753	0.588796
STRIP1	3	0.47537	0.548625	0.260725	*UQCR11*	3	0.42333	0.258449	0.587625
ANXA8L1	2	0.50801	0.548625	0.260725	*RBBP4*	3	0.51023	0.258449	0.587625
NDUFB11	3	0.5236	0.548625	0.260725	*NUMA1*	3	0.81979	0.258449	0.587625
CEP192	4	0.53271	0.548625	0.260725	*SH3PXD2A*	4	-0.34946	0.259751	0.585442
YWHAG	2	0.55131	0.548625	0.260725	*MAP6*	4	-0.40178	0.261719	0.582164
RAE1	3	0.56184	0.548625	0.260725	*BCKDK*	4	-0.6111	0.262528	0.580825
COL4A4	4	0.5875	0.548625	0.260725	*CCL26*	4	0.31783	0.262528	0.580825
CCDC92	3	0.64454	0.548625	0.260725	*SCAF8*	3	0.37424	0.262528	0.580825
ADAMTS10	2	0.74813	0.548625	0.260725	*SRSF1*	4	0.59998	0.262528	0.580825
KLHL17	1	0.76225	0.548625	0.260725	*MIEF1*	3	0.44014	0.262629	0.580657
STT3B	2	0.78951	0.548625	0.260725	*MED18*	4	0.49235	0.26315	0.579797
WNT10A	1	0.79915	0.548625	0.260725	*NOP16*	4	0.57256	0.26315	0.579797
PEX1	3	0.85268	0.548625	0.260725	*SAMD8*	4	0.46654	0.264278	0.577939
VDAC1	3	-0.045667	0.549242	0.260236	*TNFRSF13B*	3	-0.46813	0.265928	0.575236
UBASH3B	3	-0.74476	0.550808	0.259	*SCIN*	4	-0.47711	0.265973	0.575162
BICRA	4	-0.11556	0.550832	0.258981	*EXOSC2*	3	0.45714	0.266794	0.573825
OVCH2	4	-0.58168	0.551633	0.25835	*MRPL10*	3	0.35177	0.267672	0.572397
PHETA2	3	-0.46769	0.551633	0.25835	*GPR62*	3	0.52298	0.267672	0.572397
TXNRD2	4	-0.44101	0.551633	0.25835	*BCORL1*	4	-0.33366	0.268752	0.570648
MOB3A	4	-0.39026	0.551633	0.25835	*SLK*	3	-0.46535	0.269058	0.570154
MFN1	4	-0.35198	0.551633	0.25835	*PRUNE1*	4	0.16762	0.270763	0.56741
B3GLCT	4	-0.25576	0.551633	0.25835	*MTERF3*	4	0.40786	0.271047	0.566955
ZNF521	4	-0.12678	0.551633	0.25835	*TMEM126A*	4	0.34459	0.271174	0.566752

PSPN	4	0.20022	0.551633	0.25835	*CTRC*	2	0.62808	0.272577	0.564512
TIMM17A	3	0.28848	0.551633	0.25835	*TRIT1*	3	0.4381	0.272592	0.564486
IFI35	3	0.30448	0.551633	0.25835	*CDC73*	3	0.56488	0.272592	0.564486
WDR11	4	0.45106	0.551633	0.25835	*ERLIN1*	2	0.65194	0.272592	0.564486
TUSC1	4	0.54207	0.551633	0.25835	*SF3B5*	3	0.67826	0.272592	0.564486
SERPINB7	3	0.70146	0.551633	0.25835	*B4GALT5*	2	0.86057	0.272592	0.564486
ZNF853	4	-0.45841	0.552614	0.257578	*ALG11*	3	0.90583	0.272592	0.564486
LONRF2	4	-0.005381	0.552614	0.257578	*TUBB*	3	0.57875	0.274016	0.562224
POLDIP2	4	-0.49355	0.553219	0.257103	*ARMC7*	4	0.64666	0.275179	0.560385
GPR84	4	-0.29666	0.553219	0.257103	*MEOX1*	3	0.32631	0.276252	0.558694
RELCH	4	-0.18414	0.553219	0.257103	*ELP5*	4	0.080564	0.276511	0.558288
ZGPAT	4	-0.1046	0.553219	0.257103	*CDS2*	3	0.090201	0.276511	0.558288
ADAMTSL4	4	0.23935	0.553219	0.257103	*OSBPL3*	3	0.60517	0.276511	0.558288
HECTD3	4	0.29461	0.553219	0.257103	*USF3*	2	0.57971	0.276836	0.557778
ATP2B1	4	0.37665	0.553219	0.257103	*TPK1*	4	0.32507	0.277489	0.556754
PTPN4	4	0.27932	0.553634	0.256777	*METTL7A*	4	0.36402	0.278198	0.555645
RCC2	4	0.31331	0.553634	0.256777	*ZDHHC19*	4	0.37775	0.278198	0.555645
NWD2	4	0.31663	0.553634	0.256777	*DIS3L2*	3	0.44391	0.278198	0.555645
DNAJC17	4	0.54634	0.553634	0.256777	*PGS1*	3	0.4612	0.278198	0.555645
EXOC3L1	2	0.5731	0.553634	0.256777	*HNRNPL*	4	0.32731	0.278687	0.554884
SF3B3	4	0.87019	0.553634	0.256777	*RPL35*	4	0.39925	0.278687	0.554884
NCBP3	4	0.064936	0.553942	0.256536	*CACNB1*	4	0.49489	0.278687	0.554884
AHNAK	2	0.59023	0.554629	0.255997	*MAPK13*	4	-0.43554	0.279283	0.553956
FUT1	3	-0.37436	0.555734	0.255133	*SIRPG*	4	-0.43815	0.27938	0.553805
LYRM1	4	-0.10866	0.555734	0.255133	*TAF9*	3	-0.43471	0.27938	0.553805
CCNF	4	0.27814	0.555734	0.255133	*C12orf4*	4	-0.40258	0.27938	0.553805
XPO4	4	0.34439	0.555734	0.255133	*UBR1*	4	-0.080226	0.27938	0.553805
PTPRB	3	0.37988	0.555734	0.255133	*TUBA1C*	3	0.66151	0.27938	0.553805
TCTN3	3	0.41606	0.555734	0.255133	*UBE2M*	2	0.91348	0.280922	0.551414
AP1M1	4	0.42273	0.555734	0.255133	*CRCP*	3	0.64706	0.282189	0.549459
NKX6-2	3	0.42441	0.555734	0.255133	*RPS3*	3	0.068851	0.282217	0.549417
ACOX1	4	0.55034	0.555734	0.255133	*KCNV1*	4	-0.25754	0.283971	0.546726
DIMT1	4	0.57544	0.555734	0.255133	*UFSP2*	4	-0.48072	0.284512	0.545899
SSBP3	2	0.60547	0.555734	0.255133	*GRHL1*	4	-0.27557	0.284512	0.545899
MST1	3	0.61296	0.555734	0.255133	*DCT*	4	-0.29189	0.284521	0.545885
EXOSC8	4	0.79788	0.555734	0.255133	*SLC23A2*	4	-0.62878	0.284854	0.545377
MRM2	3	-0.58052	0.557195	0.253993	*POLR3GL*	4	-0.45996	0.284854	0.545377
SMOC2	4	-0.53523	0.557195	0.253993	*RBM18*	3	0.63673	0.284854	0.545377
CSNK1A1L	4	-0.3485	0.557195	0.253993	*SUPT5H*	3	0.66119	0.284854	0.545377
SHOC2	4	-0.14968	0.557195	0.253993	*MICALL1*	4	-0.56613	0.286071	0.543525
TMEM130	4	-0.068187	0.557195	0.253993	*RNF146*	4	-0.46694	0.286386	0.543049
ADAMTS18	4	-0.04945	0.557195	0.253993	*HNRNPC*	3	0.69675	0.286386	0.543049
LRP11	4	0.009023	0.557195	0.253993	*EEPD1*	4	-0.3492	0.287078	0.542
NXT2	4	0.056526	0.557195	0.253993	*PRPF19*	3	0.79966	0.287619	0.541182
ELAVL1	4	0.078894	0.557195	0.253993	*MAT2B*	3	-0.47355	0.28784	0.540849
UBE2E3	4	0.17863	0.557195	0.253993	*RPN2*	3	0.73985	0.288699	0.539555
NCAPD3	4	0.23818	0.557195	0.253993	*TMEM67*	3	0.35678	0.289737	0.537997
SLC13A4	4	0.35208	0.557195	0.253993	*EIF4B*	1	-0.8245	0.292875	0.533317
RASA2	4	0.36461	0.557195	0.253993	*IL17RE*	4	0.046114	0.292953	0.533203
NIP7	4	0.40155	0.557195	0.253993	*INSIG2*	4	0.25637	0.292953	0.533203
NTN4	4	0.41752	0.557195	0.253993	*RIC8A*	3	0.36877	0.292953	0.533203
MUS81	4	0.41872	0.557195	0.253993	*MRPL19*	4	0.38369	0.292953	0.533203
ZNF687	4	0.48717	0.557195	0.253993	*TGIF1*	3	0.61836	0.292953	0.533203
TAF1D	4	0.49741	0.557195	0.253993	*CCNH*	3	0.8799	0.292953	0.533203
UACA	4	0.52376	0.557195	0.253993	*NDOR1*	2	1.1864	0.292953	0.533203
TAF10	1	0.74288	0.557195	0.253993	*PSMC6*	4	0.62444	0.293599	0.532245
SMU1	4	0.80973	0.557195	0.253993	*CTNS*	4	0.41199	0.293661	0.532153
NCOR1	3	-0.82995	0.55893	0.252642	*NDUFAF7*	4	0.42963	0.293661	0.532153

CRB1	4	-0.50983	0.55893	0.252642	*PPWD1*	4	0.46396	0.293661	0.532153
TNFSF10	4	-0.19774	0.55893	0.252642	*TBCE*	4	0.69667	0.293661	0.532153
SPRED1	4	0.1366	0.55893	0.252642	*LSM10*	4	0.31812	0.293723	0.532063
SRRM5	4	0.15662	0.55893	0.252642	*MAD2L1BP*	4	0.55184	0.293723	0.532063
CLDN19	4	0.1948	0.55893	0.252642	*TERF1*	4	0.23791	0.293863	0.531855
ZPR1	4	0.27803	0.55893	0.252642	*PLK4*	4	0.24281	0.293863	0.531855
TFCP2L1	4	0.34227	0.55893	0.252642	*COPZ2*	4	0.29917	0.293863	0.531855
ZNF730	4	0.36258	0.55893	0.252642	*GOSR2*	4	0.54608	0.293863	0.531855
IL37	4	0.38479	0.55893	0.252642	*CWC15*	4	0.59085	0.293863	0.531855
TEX14	4	0.39081	0.55893	0.252642	*PSMB4*	4	0.84723	0.293863	0.531855
CCDC110	4	0.42405	0.55893	0.252642	*SLC26A7*	4	-0.43117	0.295806	0.528993
PGAM5	4	0.44106	0.55893	0.252642	*CAVIN4*	4	-0.43143	0.296938	0.527334
EDNRB	3	0.50809	0.55893	0.252642	*GPIHBP1*	4	-0.49642	0.297351	0.526731
RPS6	4	0.82791	0.55893	0.252642	*TMEM79*	3	0.33127	0.297932	0.525883
HOXC13	1	1.0079	0.559369	0.252302	*VAMP8*	4	-0.40446	0.298128	0.525597
ASCL3	2	0.60722	0.570019	0.24411	*MAGIX*	4	-0.39998	0.298128	0.525597
OSBP2	2	-0.48624	0.571717	0.242819	*TP53I3*	3	0.71355	0.298128	0.525597
ZMYM2	4	-0.30635	0.571717	0.242819	*LYRM1*	4	-0.37303	0.298836	0.524567
GLUD1	4	-0.58165	0.572115	0.242517	*TLCD1*	4	0.63512	0.298861	0.52453
N4BP2L2	4	0.62538	0.572115	0.242517	*SGPL1*	3	0.29898	0.299847	0.5231
UBE2J1	4	-0.68142	0.572393	0.242306	*GALNT9*	3	0.33225	0.299847	0.5231
RPS6KA5	4	0.050175	0.572393	0.242306	*WDR33*	4	0.36674	0.299847	0.5231
ZNF768	4	0.54976	0.572393	0.242306	*FAM219B*	3	0.3769	0.299847	0.5231
TMEM242	4	-0.43984	0.572425	0.242281	*STC2*	3	0.46806	0.299847	0.5231
ATP5F1A	4	-0.61297	0.573266	0.241644	*BUB1B*	3	0.4833	0.299847	0.5231
ANKIB1	4	-0.47616	0.574645	0.240601	*AQR*	4	0.49096	0.299847	0.5231
CCDC130	1	1.2525	0.574974	0.240352	*GBA*	3	0.49224	0.299847	0.5231
FRG2	1	0.84599	0.578081	0.238011	*EIF3E*	4	0.5166	0.299847	0.5231
MSANTD1	2	0.57534	0.582286	0.234863	*MPP2*	3	0.55279	0.299847	0.5231
ADAMTS8	4	-0.13339	0.582735	0.234529	*INTS8*	3	0.59699	0.299847	0.5231
RBMXL1	4	0.33802	0.582735	0.234529	*CYP4B1*	3	-0.3892	0.300044	0.522814
CENPH	4	0.42956	0.582735	0.234529	*UFL1*	4	-0.59738	0.300187	0.522607
ECPAS	4	0.57312	0.582735	0.234529	*TMEM30B*	4	-0.31267	0.300187	0.522607
ARMC7	4	0.79901	0.583084	0.234269	*CCKAR*	4	-0.26789	0.300187	0.522607
PKD1	1	-0.75215	0.584347	0.233329	*IRX5*	3	-0.096112	0.300324	0.52241
IARS1	4	0.26477	0.584347	0.233329	*DNASE1*	4	0.13543	0.301231	0.5211
SMCO2	4	0.29614	0.584347	0.233329	*CENPQ*	4	-0.43774	0.302315	0.519541
CELSR3	3	0.43316	0.584347	0.233329	*MTUS1*	4	-0.34461	0.302419	0.51939
STIP1	3	0.55457	0.584347	0.233329	*HES6*	4	0.25497	0.302717	0.518964
POMK	4	0.21461	0.584468	0.233239	*RAD18*	4	0.29098	0.302717	0.518964
BRIX1	4	0.45083	0.584468	0.233239	*TAF1D*	4	0.53043	0.30288	0.51873
JAKMIP1	4	0.53851	0.584468	0.233239	*NDUFAF5*	4	0.37929	0.303986	0.517146
PET117	2	-0.79735	0.586452	0.231767	*CPED1*	4	0.4631	0.306416	0.513688
P2RY13	4	-0.45989	0.586452	0.231767	*FKBP3*	4	-0.40318	0.307403	0.512292
RNF185	3	0.33108	0.587871	0.230718	*P2RY1*	4	0.29203	0.307403	0.512292
WWP2	4	0.4221	0.587871	0.230718	*CFL1*	4	0.33398	0.307403	0.512292
SNX1	4	-0.76398	0.589492	0.229522	*HAUS4*	4	0.61725	0.307403	0.512292
TCHH	2	-0.61599	0.589492	0.229522	*IL36G*	3	0.33752	0.307572	0.512054
GCNA	3	-0.60026	0.589492	0.229522	*CLSTN2*	4	0.35369	0.307572	0.512054
GNA12	4	-0.52519	0.589492	0.229522	*FECH*	4	0.4417	0.308091	0.511322
DYNAP	4	-0.49671	0.589492	0.229522	*RTTN*	4	0.49417	0.308091	0.511322
GPR19	4	-0.37298	0.589492	0.229522	*NHP2*	4	0.26648	0.30818	0.511196
MIR1-1HG	4	-0.30826	0.589492	0.229522	*MTCH2*	4	0.42513	0.30818	0.511196
KIT	4	-0.18193	0.589492	0.229522	*EXOSC8*	4	0.56157	0.30818	0.511196
FAM110A	4	-0.14135	0.589492	0.229522	*DHX9*	3	0.84193	0.308715	0.510443
CCDC68	4	-0.12032	0.589492	0.229522	*NEK9*	4	0.24614	0.310002	0.508636
SERINC1	3	-0.059323	0.589492	0.229522	*CNOT2*	3	0.35307	0.310002	0.508636
NABP1	4	0.073566	0.589492	0.229522	*CCT8*	4	0.43782	0.310002	0.508636

RPL11	3	0.12291	0.589492	0.229522	*ACTR5*	3	0.44083	0.310002	0.508636
CUX1	4	0.26495	0.589492	0.229522	*HS2ST1*	4	-0.4978	0.312157	0.505627
PCNX1	4	0.36786	0.589492	0.229522	*INTS1*	3	0.56016	0.313091	0.50433
HM13	3	0.37985	0.589492	0.229522	*MTX1*	3	0.38921	0.313473	0.5038
LUC7L3	4	0.42067	0.589492	0.229522	*ULBP2*	2	0.41825	0.314144	0.502872
PLS1	3	0.4265	0.589492	0.229522	*ARFGEF3*	4	0.17086	0.315507	0.500991
ELAVL3	4	0.43416	0.589492	0.229522	*PITHD1*	4	-0.20862	0.31555	0.500932
KCTD14	2	0.43583	0.589492	0.229522	*USPL1*	4	0.16252	0.31555	0.500932
SEPHS1	3	0.43975	0.589492	0.229522	*PVRIG*	4	0.24007	0.31555	0.500932
POLR2B	4	0.44629	0.589492	0.229522	*NAT10*	4	0.41202	0.31555	0.500932
KIF11	4	0.51239	0.589492	0.229522	*CDK13*	3	-0.51473	0.316642	0.499431
NCAPH	4	0.54009	0.589492	0.229522	*C6orf201*	4	-0.48267	0.316642	0.499431
KIDINS220	4	0.56441	0.589492	0.229522	*PORCN*	4	-0.47359	0.316642	0.499431
NRF1	3	0.61702	0.589492	0.229522	*LRRIQ4*	4	-0.46361	0.316642	0.499431
CDC16	4	0.63186	0.589492	0.229522	*SNAP47*	4	-0.34676	0.316642	0.499431
PRPF6	2	0.89279	0.589492	0.229522	*ABRAXAS1*	4	0.22619	0.316642	0.499431
PTPN11	1	-1.8326	0.589752	0.22933	*ALG10*	4	0.34556	0.316642	0.499431
CRIP1	4	-0.42824	0.589752	0.22933	*CCNYL1*	4	0.37812	0.316642	0.499431
WNT11	2	-0.45444	0.59091	0.228479	*SMARCA2*	4	0.38954	0.316642	0.499431
C8orf87	4	-0.3912	0.592092	0.227611	*PEX5*	4	0.48013	0.316642	0.499431
KCNK3	2	-0.19316	0.592092	0.227611	*PRIM2*	4	0.68749	0.316642	0.499431
POLR3K	4	0.65877	0.592092	0.227611	*ZC3H15*	2	0.42213	0.317331	0.498488
VDAC2	2	-0.41512	0.593016	0.226933	*POLR2L*	4	0.64619	0.317331	0.498488
EIF1AD	4	0.74671	0.593041	0.226915	*LMNA*	2	0.77383	0.317448	0.498328
GUK1	2	1.0669	0.593041	0.226915	*USP24*	1	0.92882	0.317448	0.498328
HSF4	2	-0.63144	0.593237	0.226772	*TGFB1*	3	-0.58775	0.317819	0.497821
SCN2B	3	-0.57838	0.593237	0.226772	*DLEC1*	4	0.4503	0.318643	0.496696
ELOVL5	4	-0.50725	0.593237	0.226772	*MASP2*	4	-0.50157	0.318929	0.496305
THEM4	2	-0.49463	0.593237	0.226772	*NEK4*	4	-0.27791	0.318929	0.496305
TMEM192	4	-0.41969	0.593237	0.226772	*OSBPL6*	4	0.093643	0.318929	0.496305
RLN1	4	-0.38324	0.593237	0.226772	*NFU1*	4	0.31536	0.318929	0.496305
LRRC17	4	-0.2454	0.593237	0.226772	*ARPC1B*	3	0.34539	0.318929	0.496305
SNUPN	4	-0.10531	0.593237	0.226772	*HIGD1A*	3	0.37251	0.318929	0.496305
DGKZ	3	-0.005282	0.593237	0.226772	*E4F1*	3	0.43986	0.318929	0.496305
CDYL	4	0.048782	0.593237	0.226772	*HMGA1*	2	0.71969	0.318929	0.496305
SLC39A3	4	0.052901	0.593237	0.226772	*FH*	4	0.57745	0.319211	0.495922
WDR24	4	0.075726	0.593237	0.226772	*CNBD2*	4	0.47715	0.31938	0.495692
CYP2F1	4	0.078447	0.593237	0.226772	*CRLS1*	3	0.57179	0.319464	0.495578
WDR88	3	0.18951	0.593237	0.226772	*MARCHF6*	1	-0.73488	0.319547	0.495466
PDZD3	4	0.20247	0.593237	0.226772	*MMUT*	3	-0.47054	0.319547	0.495466
ZZZ3	4	0.22536	0.593237	0.226772	*MX2*	3	-0.40513	0.319547	0.495466
TSPO2	4	0.26109	0.593237	0.226772	*ENPP3*	4	-0.31422	0.319547	0.495466
MVD	4	0.28811	0.593237	0.226772	*FGFR3*	4	-0.30011	0.319547	0.495466
ZCCHC4	4	0.29389	0.593237	0.226772	*NCOA7*	4	-0.079917	0.319547	0.495466
DDI1	3	0.35592	0.593237	0.226772	*POLR3C*	3	0.14518	0.319547	0.495466
TAS2R4	4	0.36134	0.593237	0.226772	*GGN*	4	0.38417	0.319547	0.495466
PARP1	4	0.36818	0.593237	0.226772	*CDK2AP2*	3	0.42247	0.319547	0.495466
KCNC3	4	0.37893	0.593237	0.226772	*SPICE1*	4	0.45712	0.319547	0.495466
TYK2	4	0.42179	0.593237	0.226772	*DKC1*	4	0.59927	0.319547	0.495466
TMEM176B	3	0.42594	0.593237	0.226772	*DST*	3	0.64091	0.319547	0.495466
RAB11FIP2	4	0.42803	0.593237	0.226772	*SURF1*	3	0.65538	0.319547	0.495466
AP2S1	4	0.45776	0.593237	0.226772	*UBD*	4	-0.43881	0.320101	0.494712
FOLH1	2	0.49198	0.593237	0.226772	*AHRR*	4	-0.43041	0.321759	0.492469
CCDC88A	4	0.49286	0.593237	0.226772	*FAM107B*	4	-0.39926	0.321759	0.492469
SALL1	4	0.52817	0.593237	0.226772	*RAD21*	4	-0.35856	0.321759	0.492469
ARMC9	3	0.53102	0.593237	0.226772	*PSEN2*	4	-0.31901	0.321759	0.492469
CDCA3	4	0.57332	0.593237	0.226772	*SRXN1*	4	-0.28577	0.321759	0.492469
CD3EAP	4	0.64988	0.593237	0.226772	*CPPED1*	4	-0.21087	0.321759	0.492469

PAX8	3	0.65736	0.593237	0.226772	*EEF1G*	3	0.23868	0.321759	0.492469
CCDC115	4	0.67603	0.593237	0.226772	*VIPR2*	4	0.3338	0.321759	0.492469
MFSD14B	3	-0.86013	0.593405	0.226649	*KLHL35*	4	0.34032	0.321759	0.492469
SOD1	2	-0.021315	0.593405	0.226649	*ZNF783*	3	0.37269	0.321759	0.492469
BRSK1	3	0.31396	0.593405	0.226649	*ECRG4*	3	0.39173	0.321759	0.492469
STK36	4	0.37765	0.593405	0.226649	*SFRP4*	4	0.41802	0.321759	0.492469
CYLD	4	0.43169	0.593405	0.226649	*NR3C1*	2	0.46967	0.321759	0.492469
TP53I13	4	-0.29248	0.59391	0.22628	*RPP25L*	4	0.61167	0.321759	0.492469
SHOX2	3	-0.63456	0.595146	0.225376	*GSTCD*	3	0.61539	0.321759	0.492469
GNRH2	4	-0.5996	0.595146	0.225376	*GFM2*	3	0.69056	0.321759	0.492469
TMEM171	4	-0.58805	0.595146	0.225376	*RPL31*	2	0.73905	0.321759	0.492469
C12orf65	3	-0.59945	0.595593	0.22505	*SMOC2*	4	-0.36738	0.322547	0.491407
DMXL2	3	-0.86387	0.595767	0.224924	*LOC389602*	4	-0.047887	0.322547	0.491407
TATDN1	4	-0.50056	0.596103	0.224678	*EIF3L*	4	-0.00265	0.322547	0.491407
BICD2	4	-0.47653	0.596875	0.224116	*MDN1*	3	0.025986	0.322547	0.491407
FBXL17	3	-0.89202	0.597796	0.223447	*C19orf53*	3	0.16714	0.322547	0.491407
MSN	2	-0.8376	0.597796	0.223447	*HLTF*	3	0.29295	0.322547	0.491407
YPEL3	3	-0.61675	0.597796	0.223447	*KLK11*	4	0.427	0.322547	0.491407
TNFRSF10C	4	-0.57614	0.597796	0.223447	*PSMC5*	4	0.44445	0.322547	0.491407
PIPOX	3	-0.57183	0.597796	0.223447	*NDUFA10*	3	0.60159	0.322547	0.491407
KCNN3	2	-0.55649	0.597796	0.223447	*PRMT5*	4	0.6494	0.322547	0.491407
EMP1	3	-0.50665	0.597796	0.223447	*AKIRIN2*	2	1.1322	0.322547	0.491407
NCOA5	4	-0.38177	0.597796	0.223447	*CCDC130*	1	1.2293	0.322547	0.491407
WDR27	3	-0.37162	0.597796	0.223447	*PTGR2*	3	-0.46215	0.323541	0.490071
ACTG2	4	-0.35918	0.597796	0.223447	*FCRLB*	3	-0.097902	0.323541	0.490071
GBP4	4	-0.33427	0.597796	0.223447	*KCNQ5*	3	-0.58005	0.324204	0.489181
ATP6V0A4	4	-0.32256	0.597796	0.223447	*H4C5*	3	-0.52085	0.324204	0.489181
CA14	4	-0.22641	0.597796	0.223447	*WNT7A*	4	0.12889	0.324204	0.489181
CD99L2	4	-0.1868	0.597796	0.223447	*CBARP*	4	0.28705	0.324273	0.489089
BCL9	3	0.13974	0.597796	0.223447	*ATP6V0D1*	3	0.4455	0.324273	0.489089
ACADSB	4	0.28001	0.597796	0.223447	*NOC2L*	4	0.59034	0.324273	0.489089
RNF216	4	0.30964	0.597796	0.223447	*NYAP1*	4	0.50234	0.324349	0.488987
RBM12	4	0.32485	0.597796	0.223447	*RBX1*	3	0.53968	0.324989	0.488131
DCUN1D5	4	0.40836	0.597796	0.223447	*VARS*	3	1.1604	0.325038	0.488066
PPAT	4	0.41083	0.597796	0.223447	*ITPRIP*	4	-0.26892	0.325789	0.487064
SYT3	4	0.41883	0.597796	0.223447	*PLK1*	4	0.63523	0.325789	0.487064
SATB1	3	0.43162	0.597796	0.223447	*EIF6*	4	0.77944	0.325789	0.487064
ACTN4	4	0.5975	0.597796	0.223447	*ELP4*	3	0.8297	0.325789	0.487064
PDE6G	3	0.63367	0.597796	0.223447	*NSA2*	2	0.84541	0.325789	0.487064
PNKD	2	-0.70915	0.598679	0.222806	*IGSF23*	2	-0.54398	0.326605	0.485978
TRIM35	2	-0.36231	0.598679	0.222806	*SMO*	2	0.46585	0.326605	0.485978
USP39	4	0.18405	0.598679	0.222806	*OR2A42*	1	-0.43685	0.328353	0.483658
EXOSC9	4	0.19911	0.598679	0.222806	*DGAT1*	4	0.35036	0.328353	0.483658
AP1M2	4	0.2193	0.598679	0.222806	*NSUN4*	4	0.43912	0.328353	0.483658
ZNF585B	4	0.26125	0.598679	0.222806	*RFC2*	4	0.50773	0.328353	0.483658
CDK11A	4	0.26364	0.598679	0.222806	*H2BU1*	3	0.67277	0.328353	0.483658
ALKBH8	4	0.30108	0.598679	0.222806	*PLOD2*	4	-0.46671	0.328699	0.483202
PLA2G4C	4	0.40599	0.598679	0.222806	*C12orf80*	3	-0.45211	0.328699	0.483202
ZNF546	4	0.50519	0.598679	0.222806	*MAPK4*	4	-0.4031	0.328699	0.483202
PDE8A	4	0.5273	0.598679	0.222806	*KLHL6*	4	-0.37438	0.328699	0.483202
NBPF10	2	0.66573	0.598679	0.222806	*TTC9*	4	-0.35608	0.328699	0.483202
ATP2A1	4	-0.38019	0.60013	0.221755	*ZNF776*	3	-0.18715	0.328699	0.483202
MYNN	4	0.14397	0.60013	0.221755	*TEX9*	4	-0.015441	0.328699	0.483202
MAGEA10	4	0.22029	0.60013	0.221755	*TEX14*	4	0.18498	0.328699	0.483202
MECOM	4	0.2889	0.60013	0.221755	*MED13L*	4	0.29886	0.328699	0.483202
GPX2	4	0.34422	0.60013	0.221755	*ZNF837*	4	0.31945	0.328699	0.483202
PTPRA	4	0.52148	0.60013	0.221755	*EVI5*	4	0.36363	0.328699	0.483202
C19orf12	3	0.55231	0.60013	0.221755	*ECHS1*	4	0.36618	0.328699	0.483202

LSM11	4	0.56157	0.60013	0.221755	*GEMIN8*	4	0.40225	0.328699	0.483202
P3H4	3	0.69198	0.60013	0.221755	*ITPRIPL1*	4	0.41742	0.328699	0.483202
GLI4	4	0.32619	0.600607	0.221409	*C14orf132*	4	0.44326	0.328699	0.483202
ZNF189	3	-0.96401	0.601688	0.220629	*ARCN1*	4	0.45486	0.328699	0.483202
FCER1G	2	-0.69327	0.601688	0.220629	*EP400*	4	0.46733	0.328699	0.483202
DHRSX	2	-0.43615	0.601688	0.220629	*PSMD7*	3	0.50616	0.328699	0.483202
PATL2	3	-0.42259	0.601688	0.220629	*PRPF4B*	4	0.53154	0.328699	0.483202
PDZD11	3	-0.22581	0.601688	0.220629	*USP7*	4	0.55016	0.328699	0.483202
EXOSC4	4	0.050205	0.601688	0.220629	*PSMA2*	4	0.55715	0.328699	0.483202
CBFB	4	0.15439	0.601688	0.220629	*EIF4G2*	2	0.59742	0.328699	0.483202
HOOK2	4	0.2515	0.601688	0.220629	*BOLA3*	1	0.88022	0.328699	0.483202
ATF5	3	0.34074	0.601688	0.220629	*RPAP2*	1	1.204	0.328699	0.483202
GARS1	4	0.40658	0.601688	0.220629	*ZNRF2*	2	0.64536	0.329237	0.482491
SPATA13	3	0.41575	0.601688	0.220629	*RNF217*	4	-0.30352	0.331688	0.47927
ZNF44	4	0.42999	0.601688	0.220629	*DCAKD*	4	0.34845	0.331688	0.47927
ABCB9	3	0.43335	0.601688	0.220629	*PRSS8*	4	-0.21873	0.33256	0.47813
RB1CC1	4	0.47908	0.601688	0.220629	*OTUB2*	4	-0.31402	0.332627	0.478042
NUP93	3	0.53963	0.601688	0.220629	*RTL8C*	4	-0.4314	0.334378	0.475763
RBM22	4	0.54433	0.601688	0.220629	*UHMK1*	2	-0.63051	0.33465	0.475409
TAMM41	4	0.66659	0.601688	0.220629	*GRK5*	4	-0.25898	0.335853	0.47385
SLC25A17	2	0.67462	0.601688	0.220629	*SIRPD*	4	-0.40818	0.340414	0.467993
MRE11	3	0.71309	0.601688	0.220629	*SPATA9*	3	-0.60521	0.34081	0.467488
HSD17B3	3	0.77066	0.601688	0.220629	*TCTE1*	4	0.2943	0.34117	0.467029
UTP4	4	0.69262	0.602388	0.220123	*CHRNG*	3	0.44458	0.34117	0.467029
APOA2	4	-0.40048	0.602454	0.220076	*IMPDH2*	2	0.58584	0.34117	0.467029
AVPR2	4	-0.30614	0.602454	0.220076	*XRCC6*	3	0.74478	0.34123	0.466953
OGDH	4	0.37538	0.602454	0.220076	*KIF3C*	4	0.29559	0.341752	0.466289
C2orf78	3	0.45048	0.602454	0.220076	*NUTF2*	3	0.038285	0.341965	0.466018
CDH17	4	-0.36789	0.602535	0.220018	*MAFK*	3	0.33085	0.342624	0.465182
DPY30	2	0.98429	0.602538	0.220016	*ADAT3*	3	0.46505	0.342624	0.465182
BMP8A	3	0.67246	0.602721	0.219884	*MICOS10*	4	0.49558	0.342624	0.465182
SLITRK5	4	-0.41524	0.603851	0.21907	*DAZAP2*	3	0.57001	0.342624	0.465182
ATR	3	-0.38406	0.604084	0.218903	*LRRFIP2*	3	0.57508	0.342624	0.465182
POLR3B	3	0.068298	0.60511	0.218166	*FRRS1*	3	0.66486	0.342624	0.465182
PPP1R15A	3	0.69655	0.60511	0.218166	*TMEM176B*	3	0.35648	0.342677	0.465115
KCNJ1	3	0.63902	0.605776	0.217688	*TADA3*	4	0.42911	0.342677	0.465115
ZNF517	3	0.81014	0.605776	0.217688	*STOML2*	2	0.51465	0.342677	0.465115
PLAC9	2	-0.8126	0.61332	0.212313	*GOLGA4*	3	0.61827	0.342677	0.465115
CDC42	1	-0.77498	0.61332	0.212313	*CLSPN*	3	0.39174	0.342971	0.464743
RSAD2	4	-0.59156	0.61332	0.212313	*EIF2S3*	4	0.66791	0.342971	0.464743
DNAH2	4	-0.50677	0.61332	0.212313	*IGLL5*	2	-0.29106	0.346139	0.46075
STMND1	4	-0.37084	0.61332	0.212313	*NMI*	4	0.34631	0.346139	0.46075
GBP5	3	-0.35943	0.61332	0.212313	*KRT16*	4	0.37717	0.346139	0.46075
DEFB124	4	-0.30507	0.61332	0.212313	*NDRG2*	3	-0.45927	0.347386	0.459188
IDO1	4	-0.27279	0.61332	0.212313	*CLASP2*	2	0.26605	0.349951	0.455992
MBD4	4	0.048479	0.61332	0.212313	*ZDHHC8*	3	0.40094	0.349951	0.455992
PBLD	4	0.2203	0.61332	0.212313	*CPNE3*	3	0.44362	0.349951	0.455992
ZNF623	4	0.25865	0.61332	0.212313	*LAMTOR4*	3	0.71693	0.350635	0.455144
HYLS1	4	0.30693	0.61332	0.212313	*FAM234A*	4	-0.37847	0.353472	0.451645
OLFM2	2	0.33052	0.61332	0.212313	*ELOVL5*	4	-0.35124	0.353472	0.451645
LETMD1	4	0.38628	0.61332	0.212313	*PPID*	4	-0.32901	0.353472	0.451645
LCAT	4	0.39861	0.61332	0.212313	*AP1G2*	4	-0.32295	0.353472	0.451645
CEACAM19	2	0.44398	0.61332	0.212313	*KCNQ2*	4	-0.23921	0.353472	0.451645
CEP57	4	0.52682	0.61332	0.212313	*FANCA*	3	0.22947	0.353472	0.451645
NR2F6	2	0.55451	0.61332	0.212313	*SLC35D2*	2	0.35509	0.353472	0.451645
ABHD15	2	0.55493	0.61332	0.212313	*MYBBP1A*	4	0.43994	0.353472	0.451645
DPH3	4	0.58724	0.61332	0.212313	*MVK*	4	0.45467	0.353472	0.451645
FDPS	2	0.63967	0.61332	0.212313	*SF1*	4	0.4985	0.353472	0.451645

FEM1B	3	0.33352	0.614337	0.211593	*RHPN1*	2	0.53102	0.353472	0.451645
MDC1	3	0.53565	0.614337	0.211593	*ACTR1A*	4	0.64172	0.353472	0.451645
SLBP	4	-0.57806	0.614625	0.21139	*PFKFB1*	4	-0.24803	0.353917	0.451099
MRPL19	4	-0.43124	0.614625	0.21139	*EME1*	4	0.23506	0.353917	0.451099
CSPG5	3	-0.4011	0.615412	0.210834	*CKS1B*	2	0.44388	0.353917	0.451099
MYBL2	3	0.46685	0.615412	0.210834	*LIN9*	4	0.46767	0.354914	0.449877
CYP26B1	4	-0.33997	0.615537	0.210746	*FARSA*	4	0.42545	0.355485	0.449179
TNFSF4	4	-0.31991	0.615537	0.210746	*PSMC3*	4	0.32655	0.35608	0.448452
CCDC50	4	-0.30305	0.615537	0.210746	*STARD10*	3	-1.1183	0.356599	0.447819
TEKT1	4	-0.29028	0.615537	0.210746	*SPATA24*	3	-0.25028	0.357641	0.446553
HMGXB3	4	-0.27312	0.615537	0.210746	*FUT4*	3	-0.54342	0.359222	0.444637
RRM2	8	-0.11212	0.615537	0.210746	*FLOT1*	3	-0.14299	0.359222	0.444637
ADAMTS1	4	-0.021974	0.615537	0.210746	*ITGA7*	4	0.15372	0.360159	0.443505
PSMD13	4	0.01031	0.615537	0.210746	*SLC25A28*	3	-0.56285	0.361344	0.44208
SLC5A9	3	0.050821	0.615537	0.210746	*PER2*	4	0.32455	0.361593	0.44178
RNF31	4	0.27499	0.615537	0.210746	*CLVS1*	4	0.40957	0.361593	0.44178
PTPRH	3	0.27963	0.615537	0.210746	*C4A*	1	1.0602	0.361593	0.44178
ITGA6	4	0.29059	0.615537	0.210746	*MAPK6*	3	-0.41115	0.362642	0.440522
IL18BP	4	0.31674	0.615537	0.210746	*FAM210B*	4	-0.36149	0.362642	0.440522
PES1	4	0.36543	0.615537	0.210746	*RUNX2*	4	-0.34516	0.362642	0.440522
MINDY1	4	0.38628	0.615537	0.210746	*BTN3A1*	4	-0.32083	0.362642	0.440522
PTPRR	4	0.3987	0.615537	0.210746	*MYO3A*	4	-0.26126	0.362642	0.440522
S100A8	3	0.4778	0.615537	0.210746	*TMEM253*	3	-0.041649	0.362642	0.440522
DDX3X	3	0.49222	0.615537	0.210746	*KNTC1*	4	0.060583	0.362642	0.440522
FNTA	4	0.50889	0.615537	0.210746	*SWAP70*	4	0.19674	0.362642	0.440522
E2F3	3	0.53764	0.615537	0.210746	*SCAMP3*	3	0.4122	0.362642	0.440522
B3GNT6	3	0.59959	0.615537	0.210746	*YIF1A*	4	0.46483	0.362642	0.440522
OSGEPL1	2	0.6932	0.615537	0.210746	*FOXD4L1*	2	0.52103	0.362642	0.440522
FOXD4L5	3	0.42182	0.616872	0.209805	*ZNF566*	3	0.62831	0.362642	0.440522
TMEM79	3	0.60163	0.616872	0.209805	*IMP3*	4	0.76402	0.362642	0.440522
USP3	2	-0.69056	0.618216	0.20886	*MED13*	4	-0.76533	0.36327	0.43977
CHPF	2	-0.46216	0.618216	0.20886	*ANXA10*	4	-0.47577	0.36327	0.43977
TAFA2	3	-0.38223	0.618216	0.20886	*ZSCAN5B*	4	-0.4388	0.36327	0.43977
YIPF1	4	-0.15486	0.618216	0.20886	*ZNF396*	4	-0.41248	0.36327	0.43977
RPS14	1	1.0302	0.618216	0.20886	*RWDD1*	4	-0.19335	0.36327	0.43977
RPL10A	2	1.0455	0.618216	0.20886	*VANGL1*	3	0.21177	0.36327	0.43977
CRACD	4	-0.58308	0.619231	0.208147	*FBXL5*	3	0.311	0.36327	0.43977
OTOG	4	-0.37057	0.623875	0.204902	*REEP3*	4	0.35957	0.36327	0.43977
LAMC1	4	-0.18156	0.626762	0.202897	*ANAPC5*	4	0.57522	0.36327	0.43977
MAN2B2	4	-0.12671	0.626762	0.202897	*HINT1*	4	-0.65498	0.363277	0.439763
IBTK	4	0.26303	0.626762	0.202897	*PYROXD1*	4	0.42347	0.363277	0.439763
CCDC61	3	0.30761	0.626762	0.202897	*GJB2*	4	-0.37808	0.363719	0.439233
OXSM	4	-0.46515	0.626892	0.202807	*FIGN*	3	0.5514	0.363719	0.439233
RICTOR	3	0.33976	0.628672	0.201576	*UBE2J2*	4	0.029788	0.364535	0.438261
ZDHHC23	2	0.14304	0.62897	0.20137	*TMEM270*	3	0.12282	0.366463	0.43597
IFNLR1	3	0.34523	0.629421	0.201059	*H2BC4*	2	0.59069	0.367662	0.434551
C14orf119	4	0.37937	0.629421	0.201059	*TXN2*	3	0.48812	0.367771	0.434423
TMEM159	3	0.067369	0.629742	0.200837	*ERO1A*	3	-0.55728	0.36795	0.434211
SDCCAG8	4	-0.78683	0.631689	0.199497	*GTF2F1*	4	0.050192	0.36795	0.434211
GABRE	3	-0.7241	0.631689	0.199497	*ZRANB3*	4	0.20563	0.36795	0.434211
C1orf50	4	-0.56125	0.631689	0.199497	*OLFM1*	4	0.36602	0.36795	0.434211
BAHD1	4	-0.53398	0.631689	0.199497	*UPF1*	3	0.84956	0.36795	0.434211
ARHGAP31	4	-0.45318	0.631689	0.199497	*NR2C2AP*	3	0.6382	0.368226	0.433885
TRIM15	4	-0.35754	0.631689	0.199497	*ZNF202*	3	-1.093	0.370837	0.430817
KCNV1	4	-0.32754	0.631689	0.199497	*CTTNBP2NL*	4	0.2614	0.372698	0.428643
SEZ6	4	-0.25302	0.631689	0.199497	*SEC16A*	3	0.38745	0.373163	0.428102
PCED1A	4	-0.24777	0.631689	0.199497	*TMEM182*	3	0.55097	0.375259	0.425668
DIXDC1	4	-0.17942	0.631689	0.199497	*WDR36*	3	0.52504	0.375337	0.425578

PHACTR4	4	-0.10971	0.631689	0.199497	*AKT1*	4	-0.26697	0.37659	0.424131
MYO3A	4	-0.099094	0.631689	0.199497	*RAB6A*	4	0.041211	0.376663	0.424047
PSMG1	4	0.0038	0.631689	0.199497	*TMEM94*	4	0.065626	0.376663	0.424047
DND1	4	0.081128	0.631689	0.199497	*RAN*	4	0.41253	0.376663	0.424047
PHF2	4	0.23472	0.631689	0.199497	*TSPAN31*	4	0.45957	0.376663	0.424047
KCNN1	2	0.24757	0.631689	0.199497	*RPA3*	4	0.79229	0.376663	0.424047
KHNYN	4	0.31629	0.631689	0.199497	*RND2*	4	0.1668	0.377973	0.42254
PTPRJ	3	0.31788	0.631689	0.199497	*MTX2*	4	0.34329	0.377973	0.42254
SPATA32	4	0.3485	0.631689	0.199497	*AVPR1B*	4	0.37674	0.377973	0.42254
HCN4	4	0.39925	0.631689	0.199497	*VPS25*	3	0.53893	0.377973	0.42254
PEX10	4	0.42765	0.631689	0.199497	*EXOC2*	4	0.54905	0.377973	0.42254
APOE	4	0.42932	0.631689	0.199497	*INTS3*	3	0.7381	0.379118	0.421225
PLCB3	4	0.44059	0.631689	0.199497	*RRM2B*	2	0.36143	0.38145	0.418562
OCIAD1	3	0.48244	0.631689	0.199497	*UQCC3*	2	0.64391	0.381831	0.418129
FTSJ3	3	0.52165	0.631689	0.199497	*CCDC144A*	2	-0.48532	0.381911	0.418037
RALB	4	0.53232	0.631689	0.199497	*ECSIT*	3	0.33073	0.381911	0.418037
NR2E3	2	0.54707	0.631689	0.199497	*DNAH3*	3	0.54983	0.381911	0.418037
AAAS	2	0.55101	0.631689	0.199497	*CXCL1*	3	0.61637	0.381911	0.418037
CCT4	4	0.60451	0.631689	0.199497	*LSM12*	3	0.63318	0.381911	0.418037
RAB5C	2	0.63586	0.631689	0.199497	*ZBTB17*	3	0.71252	0.381911	0.418037
COPS5	3	0.82061	0.631689	0.199497	*PEX16*	3	0.75544	0.381911	0.418037
PSMD3	3	0.93228	0.631689	0.199497	*TMSB4X*	1	0.84615	0.381911	0.418037
NOP2	2	0.95985	0.631689	0.199497	*PRR5-ARHGAI*	1	0.9293	0.381911	0.418037
RPL18	3	1.3228	0.631689	0.199497	*TMEM62*	4	-0.37135	0.382785	0.417046
LIPC	3	-0.42404	0.631851	0.199386	*H3-5*	2	0.42664	0.384551	0.415046
CLCN4	4	-0.32978	0.631851	0.199386	*SLC2A11*	3	-0.6283	0.384598	0.414994
GBE1	4	0.37529	0.631851	0.199386	*WDR63*	3	0.052389	0.384947	0.414599
MRPL51	4	0.50317	0.631851	0.199386	*RHPN2*	4	0.42981	0.384947	0.414599
EID2	2	0.58541	0.632959	0.198624	*SKA2*	2	0.57875	0.384947	0.414599
N4BP2L1	4	-0.66536	0.633417	0.19831	*EDA2R*	4	-0.37618	0.385227	0.414283
UBE2J2	4	-0.2564	0.633417	0.19831	*OTUD4*	3	-0.48991	0.385407	0.41408
CD48	4	0.12061	0.633417	0.19831	*KCNMB3*	4	0.33943	0.385407	0.41408
DERL2	3	-1.1977	0.634067	0.197865	*SIKE1*	4	0.44141	0.385407	0.41408
PPP6R1	3	-0.80946	0.634067	0.197865	*SNRNP70*	4	0.49663	0.385407	0.41408
ZNF511	2	-0.77204	0.634067	0.197865	*PFDN5*	4	0.63435	0.385407	0.41408
CCDC81	2	-0.72379	0.634067	0.197865	*SLC19A1*	3	0.47244	0.385686	0.413766
ACBD7	1	-0.70426	0.634067	0.197865	*LYPLAL1*	4	-0.26195	0.386172	0.413219
MAP3K7	4	-0.53877	0.634067	0.197865	*POLE*	4	0.31347	0.386172	0.413219
C1orf210	3	-0.41808	0.634067	0.197865	*PLAAT3*	1	0.74504	0.38666	0.412671
CLTRN	4	-0.41331	0.634067	0.197865	*TAF5*	4	0.69939	0.388241	0.410898
ANKRD37	4	-0.40689	0.634067	0.197865	*ABCB6*	4	-0.48685	0.388332	0.410797
SLC25A46	3	-0.38928	0.634067	0.197865	*SOBP*	4	-0.30191	0.388332	0.410797
PCGF1	4	-0.35525	0.634067	0.197865	*SSPO*	4	-0.29388	0.388332	0.410797
FAM104B	4	-0.32687	0.634067	0.197865	*THUMPD3*	3	0.29549	0.388332	0.410797
PHYHIPL	4	-0.30726	0.634067	0.197865	*RBM33*	3	0.33581	0.388332	0.410797
OASL	4	-0.30471	0.634067	0.197865	*ISCA1*	2	0.4021	0.388332	0.410797
SRP14	3	-0.29369	0.634067	0.197865	*MRPL22*	3	0.4668	0.388332	0.410797
RSPH9	3	-0.27798	0.634067	0.197865	*PSMA5*	4	0.62259	0.388884	0.41018
CCN2	4	-0.1989	0.634067	0.197865	*ZNHIT2*	1	1.3645	0.38962	0.409358
COBL	4	-0.053128	0.634067	0.197865	*MED30*	3	0.48815	0.390657	0.408205
ELF4	4	0.027291	0.634067	0.197865	*DMAC2*	1	0.54421	0.392223	0.406467
TCF25	3	0.070036	0.634067	0.197865	*UCN2*	4	0.36488	0.392648	0.405996
RPP40	4	0.070845	0.634067	0.197865	*CANX*	3	0.46659	0.392648	0.405996
JCHAIN	4	0.094384	0.634067	0.197865	*TMEM255B*	3	0.033532	0.393218	0.405367
ZNF106	3	0.097517	0.634067	0.197865	*CENPI*	3	0.54452	0.393925	0.404586
SSC5D	4	0.11931	0.634067	0.197865	*MIS18A*	3	0.21114	0.394255	0.404223
CCR10	4	0.39838	0.634067	0.197865	*PRXL2C*	3	0.55441	0.394255	0.404223
ARHGAP45	4	0.40453	0.634067	0.197865	*CACFD1*	3	0.43253	0.394385	0.40408

ZNF777	4	0.42393	0.634067	0.197865	*SRPX2*	4	-0.023654	0.394722	0.403709
PAG1	3	0.44354	0.634067	0.197865	*OCIAD1*	3	0.23695	0.395035	0.403365
ZNF154	4	0.54108	0.634067	0.197865	*RNF40*	3	0.27383	0.395115	0.403276
EIF2S1	4	0.55271	0.634067	0.197865	*APOA2*	4	0.33172	0.395115	0.403276
THBS4	3	0.60816	0.634067	0.197865	*UBL5*	4	0.54094	0.395115	0.403276
RPS3A	1	1.2269	0.634067	0.197865	*YKT6*	4	0.61714	0.395115	0.403276
GFM2	3	-1.0492	0.636724	0.196049	*GABRA3*	3	-0.055213	0.395336	0.403034
CRPPA	3	-0.80746	0.636801	0.195996	*MKX*	3	-0.91205	0.395661	0.402677
ART3	3	-0.4822	0.636801	0.195996	*GPATCH4*	4	0.37232	0.395661	0.402677
GNAQ	4	-0.28581	0.636801	0.195996	*ZNF22*	2	0.41151	0.396223	0.40206
USP15	3	0.33741	0.636801	0.195996	*GPSM2*	3	0.32541	0.396706	0.401531
ZNF625	4	0.4097	0.636801	0.195996	*TAF1A*	3	0.86803	0.396706	0.401531
BMS1	2	0.70782	0.636801	0.195996	*SDAD1*	3	0.75467	0.397087	0.401115
EFHC1	3	-0.53138	0.637923	0.195232	*FGF22*	1	0.81028	0.397087	0.401115
BPIFB2	4	0.065303	0.637923	0.195232	*NLE1*	3	1.0633	0.397087	0.401115
PYGO2	4	0.18455	0.637923	0.195232	*B3GNT7*	4	-0.27723	0.397236	0.400951
CCL26	4	0.31655	0.637923	0.195232	*PRAME*	3	-0.17719	0.397544	0.400615
ORC4	4	0.35526	0.637923	0.195232	*COQ8B*	3	-0.3824	0.398442	0.399635
ODC1	4	0.36366	0.637923	0.195232	*ZBTB14*	3	0.42355	0.400268	0.397649
SH3GL1	3	0.45741	0.637923	0.195232	*SLC25A37*	4	-0.48867	0.40038	0.397527
AARS1	4	0.4735	0.637923	0.195232	*STAB2*	4	-0.36369	0.40038	0.397527
RCC1L	2	0.83117	0.637923	0.195232	*HLA-DOB*	2	-0.62569	0.4004	0.397506
RPS24	3	1.2676	0.637923	0.195232	*FBXL4*	2	-0.60098	0.4004	0.397506
CTF1	2	-0.48199	0.63894	0.19454	*PCBP2*	4	-0.52925	0.4004	0.397506
DGKA	3	-0.4445	0.63894	0.19454	*FEM1C*	4	0.066811	0.4004	0.397506
ZNF70	4	0.17415	0.63894	0.19454	*SMCO4*	3	0.19321	0.4004	0.397506
RASGEF1A	4	0.26058	0.63894	0.19454	*RNF214*	4	0.28289	0.4004	0.397506
CLDN1	4	0.34661	0.63894	0.19454	*TRIM28*	4	0.30054	0.4004	0.397506
CASP1	3	0.49856	0.63894	0.19454	*NAA25*	3	0.30353	0.4004	0.397506
ZFHX4	4	-0.23138	0.638963	0.194525	*ATP5F1A*	4	0.37465	0.4004	0.397506
BICRAL	4	-0.47792	0.639345	0.194265	*POLDIP2*	4	0.37689	0.4004	0.397506
GTF2H4	4	0.50199	0.639996	0.193823	*YRDC*	4	0.45362	0.4004	0.397506
TM4SF1	4	0.18084	0.64004	0.193793	*RIOK3*	4	0.46062	0.4004	0.397506
TRMT12	3	0.44687	0.643627	0.191366	*COX6C*	4	0.48076	0.4004	0.397506
PIBF1	3	0.52892	0.643627	0.191366	*PDSS2*	3	0.49395	0.4004	0.397506
TCF4	2	-0.86227	0.644449	0.190812	*BOP1*	4	0.55633	0.4004	0.397506
PSMB10	3	0.31413	0.644449	0.190812	*KAT5*	3	0.79879	0.4004	0.397506
PDPN	2	-0.27228	0.6459	0.189835	*ATP5MPL*	1	0.90922	0.4004	0.397506
H3C7	3	-0.65168	0.646103	0.189698	*ATMIN*	4	-0.45819	0.401688	0.396111
MORC4	3	-0.361	0.646103	0.189698	*SLC33A1*	4	-0.24274	0.401688	0.396111
SNED1	3	0.029165	0.646103	0.189698	*MMAB*	4	-0.22779	0.401688	0.396111
TRIAP1	3	0.87527	0.646103	0.189698	*GNAQ*	4	-0.15251	0.401688	0.396111
STXBP5	3	-0.70845	0.646349	0.189533	*XKR4*	3	0.16176	0.401688	0.396111
ZNF350	3	0.01429	0.646349	0.189533	*KCNJ8*	4	0.29563	0.401688	0.396111
TNNC2	4	0.15479	0.646349	0.189533	*CFAP47*	4	-0.46457	0.401751	0.396043
TOP1MT	3	0.46135	0.646349	0.189533	*LRIG3*	4	-0.35102	0.401751	0.396043
LARP1	4	0.30844	0.646989	0.189103	*NT5E*	3	-0.63017	0.403234	0.394443
GCNT3	4	-0.61161	0.647261	0.188921	*EEF1AKMT3*	4	-0.5036	0.403234	0.394443
METTL9	3	-0.29041	0.647261	0.188921	*NMBR*	4	-0.41852	0.403234	0.394443
PRAM1	2	0.55595	0.647261	0.188921	*ZNF717*	3	-0.3954	0.403234	0.394443
NUP35	3	-0.81927	0.647269	0.188915	*GUCY1B1*	4	0.038313	0.403234	0.394443
SLC38A11	3	-0.50484	0.647269	0.188915	*C1QTNF12*	4	0.19556	0.403234	0.394443
RPS6KA2	4	-0.32382	0.647269	0.188915	*COQ3*	3	0.21939	0.403234	0.394443
ZNF30	4	0.15686	0.647269	0.188915	*KLF1*	4	0.26327	0.403234	0.394443
XPA	4	0.37729	0.647269	0.188915	*FNBP4*	4	0.27971	0.403234	0.394443
AVPR1B	4	0.43317	0.647269	0.188915	*MXD1*	4	0.28111	0.403234	0.394443
NUB1	1	-0.85445	0.647904	0.188489	*KCNJ1*	3	0.3527	0.403234	0.394443
NUDC	3	0.87549	0.64984	0.187194	*SON*	4	0.35587	0.403234	0.394443

BAG4	3	-0.37195	0.649987	0.187095	*TUFM*	4	0.38603	0.403234	0.394443
ZNF157	3	0.26607	0.650045	0.187056	*SMAD9*	4	0.39135	0.403234	0.394443
PTMA	3	-0.86466	0.651386	0.186162	*TRAPPC8*	3	0.40657	0.403234	0.394443
HDAC7	3	-0.8504	0.651386	0.186162	*SH3BP1*	2	0.42928	0.403234	0.394443
SLC25A51	2	0.42691	0.651386	0.186162	*DVL1*	4	0.4434	0.403234	0.394443
SLC41A1	3	-0.32896	0.651408	0.186147	*PATJ*	4	0.44379	0.403234	0.394443
MAP6D1	4	0.2404	0.651408	0.186147	*DDHD1*	4	0.49051	0.403234	0.394443
LEMD2	4	0.33947	0.651408	0.186147	*HMGCS1*	4	0.54134	0.403234	0.394443
CCDC122	4	0.36402	0.651408	0.186147	*ATP1A1*	4	0.58091	0.403234	0.394443
FNDC7	4	0.38111	0.651408	0.186147	*MPC2*	1	0.67159	0.403234	0.394443
CUBN	4	-0.37148	0.652782	0.185232	*RPP38*	3	0.82571	0.403234	0.394443
ZSWIM7	4	0.33434	0.652782	0.185232	*PTPRA*	4	0.4597	0.405081	0.392458
CTNNBL1	4	0.67049	0.652782	0.185232	*ACSL3*	4	0.22603	0.405241	0.392287
SLC30A9	2	-0.9362	0.65321	0.184947	*HGH1*	3	-0.011331	0.405881	0.391601
TTC9B	2	-0.76969	0.65321	0.184947	*ECT2*	3	0.61959	0.406478	0.390963
C6orf226	3	-0.48838	0.65321	0.184947	*MARCO*	3	-0.37959	0.407646	0.389717
ACOT4	3	-0.42639	0.65321	0.184947	*ASPH*	4	0.12413	0.407646	0.389717
H2AW	4	-0.3977	0.65321	0.184947	*UGT1A5*	4	0.065786	0.408201	0.389126
MROH2A	3	-0.38119	0.65321	0.184947	*LSM14A*	4	0.34674	0.408201	0.389126
FBP2	2	-0.35074	0.65321	0.184947	*SNX4*	4	0.29372	0.408445	0.388866
WFDC3	3	-0.34724	0.65321	0.184947	*SBDS*	4	0.28479	0.409958	0.387261
MID1IP1	4	-0.33629	0.65321	0.184947	*PLEKHG6*	3	0.41945	0.409958	0.387261
SPARCL1	4	-0.32108	0.65321	0.184947	*SRCAP*	3	0.67675	0.409958	0.387261
LRRC59	4	-0.29524	0.65321	0.184947	*FAM155B*	3	-0.64018	0.411148	0.386002
BTN2A2	4	-0.27313	0.65321	0.184947	*F5*	3	-0.638	0.411148	0.386002
TSNAXIP1	4	-0.26256	0.65321	0.184947	*OTUD7B*	3	-0.59785	0.411148	0.386002
LZTFL1	4	-0.24338	0.65321	0.184947	*SIM1*	4	-0.58496	0.411148	0.386002
IL12B	2	-0.23934	0.65321	0.184947	*ZFP37*	4	-0.55032	0.411148	0.386002
PI4KA	3	-0.22095	0.65321	0.184947	*DSEL*	3	-0.50135	0.411148	0.386002
AKR1E2	4	-0.18779	0.65321	0.184947	*COIL*	4	-0.44099	0.411148	0.386002
NOVA2	4	-0.14889	0.65321	0.184947	*SMIM24*	3	-0.35659	0.411148	0.386002
ETV6	4	-0.038444	0.65321	0.184947	*CPQ*	4	-0.043412	0.411148	0.386002
PACC1	4	-0.004089	0.65321	0.184947	*RBP2*	3	0.39008	0.411148	0.386002
ZNF211	4	0.20463	0.65321	0.184947	*ARL4D*	3	0.42363	0.411148	0.386002
MMD	3	0.22871	0.65321	0.184947	*SUN1*	2	0.67883	0.411148	0.386002
IQSEC2	4	0.23472	0.65321	0.184947	*RFC3*	4	0.7273	0.411148	0.386002
CCT3	4	0.24185	0.65321	0.184947	*COPE*	3	0.86526	0.411148	0.386002
TRIM3	3	0.27263	0.65321	0.184947	*KIF12*	3	-0.57662	0.41117	0.385979
CCAR1	4	0.27494	0.65321	0.184947	*FAM104B*	4	-0.24892	0.41117	0.385979
ZNF699	4	0.2877	0.65321	0.184947	*PLEC*	3	0.18688	0.41117	0.385979
ZNF732	4	0.29196	0.65321	0.184947	*RPUSD2*	3	0.38959	0.41117	0.385979
GSTP1	4	0.29437	0.65321	0.184947	*PON1*	3	0.46487	0.41117	0.385979
RBM11	4	0.30107	0.65321	0.184947	*RBM8A*	3	0.74671	0.41117	0.385979
A4GNT	3	0.30557	0.65321	0.184947	*STRIP1*	3	0.77728	0.41117	0.385979
DHX37	4	0.35009	0.65321	0.184947	*ZNF492*	1	1.1836	0.41117	0.385979
SGCE	4	0.35338	0.65321	0.184947	*TCN1*	4	-0.54264	0.411878	0.385231
CPT1A	3	0.38229	0.65321	0.184947	*CAD*	3	0.63652	0.412329	0.384756
UBXN10	3	0.41413	0.65321	0.184947	*TLDC2*	4	-0.33917	0.412397	0.384684
SAMD9L	4	0.41833	0.65321	0.184947	*TBPL2*	4	-0.24922	0.412397	0.384684
TTLL3	2	0.42346	0.65321	0.184947	*ESM1*	4	-0.3515	0.413679	0.383336
CBX3	3	0.43074	0.65321	0.184947	*TBL1XR1*	3	-0.67291	0.414576	0.382396
GMEB2	4	0.45291	0.65321	0.184947	*DHPS*	4	0.72998	0.414576	0.382396
UBL3	4	0.46129	0.65321	0.184947	*MAP3K20*	2	-0.48971	0.414684	0.382282
FSD1	4	0.47921	0.65321	0.184947	*ARGLU1*	4	-0.021773	0.414684	0.382282
TINF2	3	0.49054	0.65321	0.184947	*THAP11*	4	0.33138	0.414684	0.382282
HINT3	4	0.49962	0.65321	0.184947	*LIMS1*	2	0.37373	0.414684	0.382282
DAND5	4	0.51998	0.65321	0.184947	*KIAA0100*	4	0.64878	0.414684	0.382282
GET3	4	0.54329	0.65321	0.184947	*KRR1*	4	0.30408	0.415343	0.381594

CNTD2	2	0.56269	0.65321	0.184947	*LIPC*	3	-0.57752	0.4167	0.380177
TFDP1	4	0.56946	0.65321	0.184947	*SLC7A11*	4	0.073024	0.417059	0.379803
KLK1	3	0.59687	0.65321	0.184947	*COL16A1*	3	-0.35772	0.417333	0.379517
HNF1B	4	0.60842	0.65321	0.184947	*KIFAP3*	4	0.18828	0.419279	0.377497
ART1	2	0.62752	0.65321	0.184947	*MSANTD4*	2	-0.56139	0.419334	0.37744
ARHGEF16	2	0.64019	0.65321	0.184947	*HPX*	4	-0.35529	0.419334	0.37744
BNIP3	2	0.66287	0.65321	0.184947	*ARHGEF4*	3	-0.23911	0.419334	0.37744
POLR3E	4	0.69234	0.65321	0.184947	*HSBP1L1*	3	0.038938	0.419352	0.377421
TTK	3	1.0486	0.65321	0.184947	*NEUROD1*	4	-0.025975	0.421037	0.375679
MAP7D2	1	-0.78146	0.653887	0.184497	*CCDC153*	4	0.23943	0.421052	0.375664
MECP2	4	-0.64949	0.653887	0.184497	*MIER2*	4	0.28957	0.421052	0.375664
GMFG	3	0.38431	0.653887	0.184497	*ZCCHC14*	3	0.40474	0.421052	0.375664
KNSTRN	3	0.63537	0.653887	0.184497	*TNPO1*	3	0.51931	0.421052	0.375664
VTN	3	0.48063	0.654508	0.184085	*FARSB*	4	0.57014	0.421052	0.375664
LNPK	3	-0.25604	0.655654	0.183326	*SLC3A2*	3	0.73731	0.421052	0.375664
FOXI3	1	0.78551	0.656041	0.183069	*MRPL20*	3	0.83065	0.421052	0.375664
STS	2	-0.2849	0.656827	0.182549	*NCOR1*	3	-0.57899	0.421453	0.375251
EZHIP	3	-0.33046	0.65701	0.182428	*MAGI3*	3	-0.37112	0.421453	0.375251
XRCC6	3	0.12868	0.65701	0.182428	*SAT2*	4	0.14461	0.421453	0.375251
FAAP24	3	0.397	0.65701	0.182428	*RNMT*	3	0.1755	0.421453	0.375251
TEDDM1	3	0.41506	0.65701	0.182428	*METTL22*	4	0.18219	0.421453	0.375251
BTG4	3	0.45943	0.657751	0.181938	*MICALL2*	4	0.20286	0.421453	0.375251
COX18	2	-0.68035	0.658575	0.181395	*UAP1*	4	0.34285	0.421453	0.375251
RRP12	3	-1.2792	0.659095	0.181052	*CTXN1*	3	0.34341	0.421453	0.375251
KDM5C	1	-0.99558	0.659095	0.181052	*OAZ3*	3	0.40853	0.421453	0.375251
RBFA	4	-0.65304	0.659095	0.181052	*APP*	4	0.40883	0.421453	0.375251
DYRK1A	2	-0.60256	0.659095	0.181052	*PTPN7*	3	0.42055	0.421453	0.375251
FASN	4	-0.53457	0.659095	0.181052	*TAF5L*	3	0.44094	0.421453	0.375251
DHX30	4	-0.50904	0.659095	0.181052	*PYURF*	3	0.45959	0.421453	0.375251
PTGR2	3	-0.50321	0.659095	0.181052	*C12orf45*	4	0.48593	0.421453	0.375251
CXXC4	4	-0.48954	0.659095	0.181052	*STIL*	4	0.53123	0.421453	0.375251
SLC10A3	2	-0.48115	0.659095	0.181052	*C1QTNF9B*	1	0.56379	0.421453	0.375251
LUM	4	-0.43083	0.659095	0.181052	*CABP1*	3	0.57583	0.421453	0.375251
PRDM1	2	-0.39781	0.659095	0.181052	*DNAJC25*	3	0.59377	0.421453	0.375251
H4C1	4	-0.39251	0.659095	0.181052	*WDR18*	4	0.63117	0.421453	0.375251
B9D1	4	-0.3837	0.659095	0.181052	*IL6*	1	0.67092	0.421453	0.375251
PIM1	4	-0.37697	0.659095	0.181052	*CNOT6L*	4	0.027543	0.422289	0.37439
HMGCL	4	-0.32161	0.659095	0.181052	*MYH9*	4	0.2989	0.422289	0.37439
GRHL1	4	-0.3173	0.659095	0.181052	*NUP85*	4	0.58375	0.422289	0.37439
GLOD5	4	-0.31666	0.659095	0.181052	*C16orf90*	4	0.28054	0.422843	0.373821
NR1I2	4	-0.30467	0.659095	0.181052	*ATP5PO*	4	-0.21969	0.422982	0.373678
MKX	3	-0.19945	0.659095	0.181052	*HTR6*	4	0.38958	0.423142	0.373513
TXNDC11	4	-0.17383	0.659095	0.181052	*GPT*	2	-0.74126	0.423966	0.372669
TIMM8B	4	-0.019203	0.659095	0.181052	*MMP28*	2	-0.65854	0.423966	0.372669
TXNRD3	4	0.16763	0.659095	0.181052	*C17orf102*	4	-0.1884	0.423966	0.372669
GPR62	3	0.18099	0.659095	0.181052	*DNAJC2*	4	0.63923	0.423966	0.372669
PLXNB2	4	0.26188	0.659095	0.181052	*KCNG3*	3	-0.48776	0.42713	0.36944
APEH	4	0.29761	0.659095	0.181052	*TICRR*	4	-0.20747	0.427155	0.369414
CHMP5	4	0.31478	0.659095	0.181052	*KIF18A*	4	0.52857	0.427155	0.369414
MMS22L	4	0.3174	0.659095	0.181052	*DOC2A*	4	0.29552	0.42716	0.369409
OC10050642.	4	0.33361	0.659095	0.181052	*IVNS1ABP*	4	0.33809	0.42716	0.369409
DCAF8	4	0.34787	0.659095	0.181052	*RNF39*	1	-0.76667	0.427214	0.369354
WFDC1	4	0.35472	0.659095	0.181052	*AMT*	3	0.086904	0.427289	0.369279
KRTAP5-9	3	0.40163	0.659095	0.181052	*AP2B1*	3	-0.74994	0.428026	0.368529
MUC16	4	0.419	0.659095	0.181052	*AP2A2*	3	-0.45626	0.428026	0.368529
IPO8	3	0.51757	0.659095	0.181052	*MBTPS2*	4	0.16484	0.428026	0.368529
TC2N	4	0.52033	0.659095	0.181052	*NOD2*	4	0.2515	0.428026	0.368529
TTF1	4	0.54485	0.659095	0.181052	*MRPL47*	4	0.30379	0.428026	0.368529

SNRNP25	4	0.59633	0.659095	0.181052	*BRIP1*	4	0.49544	0.428092	0.368463
H2BC4	2	0.60687	0.659095	0.181052	*NHLRC2*	4	0.56289	0.428092	0.368463
H1-2	4	0.62495	0.659095	0.181052	*TSC22D1*	4	0.23612	0.428128	0.368426
MYRF	2	0.64282	0.659095	0.181052	*COPS3*	4	0.27113	0.428128	0.368426
PCDHGA12	2	0.74294	0.659095	0.181052	*LSM2*	4	0.60812	0.428128	0.368426
POLR2C	3	0.97988	0.659095	0.181052	*WDR26*	4	0.50101	0.428364	0.368187
SLC35E2A	1	-1.1526	0.659482	0.180797	*ANXA9*	3	-0.064836	0.429007	0.367536
HMX1	3	-0.9749	0.659482	0.180797	*POTEA*	4	-0.43575	0.431792	0.364725
C6orf47	2	-0.90613	0.659482	0.180797	*TMEM14EP*	4	0.29146	0.431792	0.364725
FBXL4	2	-0.58914	0.659482	0.180797	*RCC2*	4	0.29442	0.431792	0.364725
PPL	2	-0.53679	0.659482	0.180797	*NELFCD*	4	0.57722	0.431792	0.364725
TWIST1	3	-0.4967	0.659482	0.180797	*USP20*	4	-0.54892	0.431972	0.364545
MEF2C	3	-0.49225	0.659482	0.180797	*TSSK6*	2	-0.40461	0.431972	0.364545
LRRCC1	4	-0.4768	0.659482	0.180797	*MKI67*	4	0.061087	0.431972	0.364545
DNAJC14	4	-0.39861	0.659482	0.180797	*ITGA6*	4	0.36631	0.431972	0.364545
CYP27A1	3	-0.37192	0.659482	0.180797	*GTPBP3*	4	0.3759	0.431972	0.364545
HNRNPLL	4	-0.35509	0.659482	0.180797	*TMEM240*	4	0.39313	0.431972	0.364545
TCP11L1	3	-0.33505	0.659482	0.180797	*SPP1*	4	-0.34492	0.43202	0.364496
CLEC19A	3	-0.064015	0.659482	0.180797	*COL12A1*	3	-0.12276	0.43202	0.364496
EDC3	3	-0.060631	0.659482	0.180797	*EMC7*	3	0.71749	0.43202	0.364496
GSTA4	4	-0.039362	0.659482	0.180797	*AKR1B1*	3	0.51916	0.43212	0.364395
RPUSD3	4	0.067461	0.659482	0.180797	*NPTX2*	3	0.5901	0.43212	0.364395
IL17D	3	0.17819	0.659482	0.180797	*RNF113A*	4	0.54734	0.432461	0.364053
PALM	4	0.19024	0.659482	0.180797	*GEMIN6*	3	0.73303	0.432461	0.364053
SP3	4	0.20218	0.659482	0.180797	*GUK1*	2	0.99381	0.433375	0.363136
RAB27B	3	0.20728	0.659482	0.180797	*PSMD14*	4	0.45922	0.433826	0.362684
DUSP5	3	0.26829	0.659482	0.180797	*ITGAV*	4	0.17037	0.434325	0.362185
PI3	4	0.33903	0.659482	0.180797	*HEATR6*	4	0.20786	0.434325	0.362185
SOWAHC	3	0.34309	0.659482	0.180797	*TBCCD1*	4	0.28188	0.434472	0.362038
ZNF710	3	0.35073	0.659482	0.180797	*DRG2*	4	0.29234	0.434472	0.362038
WASHC4	3	0.36985	0.659482	0.180797	*TUT7*	4	0.35319	0.434472	0.362038
SLC8B1	4	0.40397	0.659482	0.180797	*STK33*	2	0.42232	0.434472	0.362038
NINJ1	4	0.40789	0.659482	0.180797	*NDUFAB1*	2	0.46463	0.434472	0.362038
OGFOD3	4	0.44676	0.659482	0.180797	*SMIM6*	2	0.47337	0.434472	0.362038
HEATR5B	4	0.46083	0.659482	0.180797	*HMGXB4*	3	0.4942	0.434472	0.362038
IGFL2	3	0.4693	0.659482	0.180797	*EIF3K*	3	0.52962	0.434472	0.362038
TAS2R5	3	0.48329	0.659482	0.180797	*UBA3*	4	0.58939	0.434472	0.362038
ERCC3	4	0.51611	0.659482	0.180797	*GET4*	3	0.77903	0.434472	0.362038
BUD31	4	0.53239	0.659482	0.180797	*BUB1*	2	1.111	0.434472	0.362038
DTYMK	4	0.56393	0.659482	0.180797	*CITED1*	3	-0.72814	0.434865	0.361646
HARS2	4	0.60039	0.659482	0.180797	*AP3S1*	3	-0.54783	0.434865	0.361646
ECT2	3	0.62158	0.659482	0.180797	*FBXL21P*	2	-0.089934	0.434865	0.361646
RPL27A	1	1.3118	0.659482	0.180797	*ABCC6*	4	-0.002628	0.434865	0.361646
ZBTB5	3	-0.40087	0.659483	0.180796	*ANO9*	4	0.29647	0.434865	0.361646
ANKRD1	3	0.38841	0.659483	0.180796	*MRPL46*	4	0.30824	0.434865	0.361646
CCDC93	4	0.45141	0.659483	0.180796	*SP140*	4	0.47029	0.434865	0.361646
ETFA	3	0.24325	0.65953	0.180766	*AMER1*	2	-0.54709	0.438369	0.35816
TEX43	2	-0.92517	0.660936	0.17984	*TIMM10*	4	0.14829	0.438369	0.35816
ZNF280C	3	-0.45691	0.660936	0.17984	*WASL*	4	-0.29627	0.438994	0.357541
RYR2	2	0.62371	0.660936	0.17984	*FBXW9*	4	0.30941	0.438994	0.357541
HAND2	3	-0.34461	0.66116	0.179693	*RFK*	4	0.34473	0.438994	0.357541
TPGS2	3	-0.71715	0.661979	0.179156	*RAB33B*	4	0.37388	0.438994	0.357541
SLC24A2	3	-0.57926	0.661979	0.179156	*YME1L1*	4	0.37923	0.438994	0.357541
SAP25	3	-0.53203	0.661979	0.179156	*FAT3*	2	0.43811	0.438994	0.357541
DR1	4	0.10418	0.661979	0.179156	*B4GALNT2*	4	0.35161	0.439177	0.35736
CD79A	4	0.36794	0.661979	0.179156	*LRP5L*	3	-0.047545	0.439497	0.357044
KCNG3	3	-0.7265	0.663367	0.178246	*TMEM126B*	3	0.42084	0.439497	0.357044
OSBP	3	-0.6552	0.663367	0.178246	*NOL8*	3	0.44647	0.439497	0.357044

ANKRD31	2	-0.63516	0.663367	0.178246	*PAFAH1B3*	3	0.49371	0.439497	0.357044
NXPH3	4	-0.61423	0.663367	0.178246	*PNISR*	3	0.72655	0.439497	0.357044
BCKDK	4	-0.5568	0.663367	0.178246	*LRTOMT*	3	0.66767	0.439914	0.356632
DARS2	4	-0.50912	0.663367	0.178246	*ZNF529*	4	-0.40228	0.440532	0.356022
CD1D	4	-0.49107	0.663367	0.178246	*TIMELESS*	3	0.22385	0.440532	0.356022
EPHA1	4	-0.44404	0.663367	0.178246	*DCLRE1C*	3	-0.51736	0.440842	0.355717
ZFPL1	4	-0.38182	0.663367	0.178246	*IFI27L1*	4	-0.32555	0.440842	0.355717
TEX38	4	-0.30073	0.663367	0.178246	*PROX1*	4	-0.1957	0.440842	0.355717
C9orf139	4	-0.27878	0.663367	0.178246	*EXOC8*	3	0.64772	0.440842	0.355717
ITPRIP	4	-0.2727	0.663367	0.178246	*ZNF317*	4	-0.56598	0.441551	0.355019
COL27A1	4	-0.23175	0.663367	0.178246	*WNT10B*	2	-0.63749	0.441552	0.355018
WDR54	4	-0.20536	0.663367	0.178246	*TMEM61*	3	-0.3282	0.441552	0.355018
CDNF	3	-0.16944	0.663367	0.178246	*ZWINT*	3	0.24865	0.441552	0.355018
LSMEM2	4	-0.14426	0.663367	0.178246	*IRF8*	3	0.38862	0.441552	0.355018
CNTRL	4	-0.099997	0.663367	0.178246	*OGFOD3*	4	0.5063	0.441552	0.355018
PTGS2	4	-0.026999	0.663367	0.178246	*DNMBP*	3	0.52533	0.441552	0.355018
CHRM4	4	0.04301	0.663367	0.178246	*PTBP3*	4	-0.02016	0.441641	0.354931
BCAS4	3	0.11656	0.663367	0.178246	*MRPS10*	3	0.16193	0.441641	0.354931
CETN3	4	0.17742	0.663367	0.178246	*RER1*	4	0.2321	0.441641	0.354931
CCDC71	4	0.23285	0.663367	0.178246	*TRIAP1*	3	0.2682	0.441641	0.354931
GASK1A	4	0.2469	0.663367	0.178246	*DENND3*	4	0.28975	0.441641	0.354931
CTRL	4	0.25618	0.663367	0.178246	*IRF3*	4	0.29632	0.441641	0.354931
ZNRF1	4	0.27367	0.663367	0.178246	*FIGNL1*	4	0.36374	0.441641	0.354931
JAGN1	4	0.30891	0.663367	0.178246	*CLTCL1*	2	0.42254	0.441641	0.354931
TMEM135	3	0.33565	0.663367	0.178246	*GLE1*	3	0.74834	0.441641	0.354931
YJU2	4	0.34812	0.663367	0.178246	*TBC1D26*	3	-0.54892	0.441644	0.354927
CAMLG	4	0.36509	0.663367	0.178246	*CRIP2*	3	-0.38623	0.441648	0.354924
MAP4K1	4	0.36876	0.663367	0.178246	*UBE2J1*	4	-0.54143	0.442211	0.35437
ZNF506	4	0.39676	0.663367	0.178246	*THAP9*	4	-0.46314	0.442211	0.35437
USF3	2	0.39846	0.663367	0.178246	*MESP2*	4	-0.1056	0.442211	0.35437
ZBTB20	4	0.40512	0.663367	0.178246	*PATE4*	4	-0.052688	0.442211	0.35437
COX20	2	0.41167	0.663367	0.178246	*ABHD8*	3	0.40012	0.442211	0.35437
ACSF2	4	0.41616	0.663367	0.178246	*IFI35*	3	0.49896	0.442211	0.35437
OSBPL9	2	0.45164	0.663367	0.178246	*SAE1*	4	0.54131	0.442211	0.35437
LARP4B	4	0.45319	0.663367	0.178246	*PLIN4*	4	0.27528	0.442217	0.354365
RAB6A	4	0.45329	0.663367	0.178246	*PAFAH1B1*	4	0.70625	0.442217	0.354365
COL6A6	3	0.46134	0.663367	0.178246	*SLBP*	4	-0.66198	0.442627	0.353962
SCAF4	4	0.46847	0.663367	0.178246	*CHMP5*	4	0.30101	0.442627	0.353962
COPS8	4	0.4704	0.663367	0.178246	*DCTN2*	4	0.45273	0.442707	0.353884
LZTS3	3	0.47741	0.663367	0.178246	*RAB32*	3	-0.72896	0.442847	0.353747
DCTN4	4	0.48344	0.663367	0.178246	*KLK5*	4	0.0664	0.442847	0.353747
ST13	4	0.50299	0.663367	0.178246	*WDR91*	4	-0.49762	0.443379	0.353225
BLOC1S2	3	0.52113	0.663367	0.178246	*SH2D3C*	3	-0.34987	0.443379	0.353225
GALNT10	2	0.55306	0.663367	0.178246	*CD52*	4	-0.32324	0.443379	0.353225
FOXM1	4	0.56523	0.663367	0.178246	*ZNF514*	4	0.23091	0.443379	0.353225
AOC1	1	0.66909	0.663367	0.178246	*CEMP1*	3	0.35092	0.443379	0.353225
USE1	3	0.67269	0.663367	0.178246	*COQ5*	3	0.55942	0.443379	0.353225
CSNK2B	4	0.70923	0.663367	0.178246	*MICAL2*	1	-0.85762	0.443594	0.353014
NOP14	4	0.72101	0.663367	0.178246	*LBH*	4	-0.43286	0.443594	0.353014
KXD1	1	0.74121	0.663367	0.178246	*ZNF800*	4	-0.41305	0.443594	0.353014
ABT1	4	0.84735	0.663367	0.178246	*TEX48*	4	-0.39269	0.443594	0.353014
RPL37A	1	1.0583	0.663367	0.178246	*ATG101*	4	-0.31584	0.443594	0.353014
MEX3C	4	0.25803	0.664139	0.177741	*TTC25*	4	0.31052	0.443594	0.353014
COG4	3	0.56929	0.664139	0.177741	*ZNF669*	4	0.31626	0.443594	0.353014
GABRA2	4	-0.45604	0.664722	0.17736	*BUD23*	4	0.39433	0.443594	0.353014
HGD	3	0.29792	0.664722	0.17736	*EIF4A3*	2	0.52326	0.443594	0.353014
SMC4	4	0.45003	0.664722	0.17736	*C15orf62*	3	0.68298	0.443594	0.353014
PAM	3	0.30215	0.665432	0.176896	*GTF3A*	3	0.82035	0.443594	0.353014

CABP4	3	0.62379	0.665432	0.176896	*SYT2*	4	-0.47953	0.443705	0.352905
CEP41	3	0.33758	0.665929	0.176572	*PSEN1*	4	-0.13725	0.443705	0.352905
PELI2	3	0.30472	0.666824	0.175989	*NMNAT1*	3	0.032111	0.443705	0.352905
DUSP22	3	-0.82192	0.667974	0.175241	*RFT1*	3	0.043864	0.443705	0.352905
MTMR6	3	-0.52999	0.667974	0.175241	*ERAL1*	3	0.19227	0.443705	0.352905
ASCC1	3	-0.45128	0.667974	0.175241	*ATP5MC3*	3	0.31951	0.443705	0.352905
AIFM3	4	-0.35707	0.667974	0.175241	*TMEM225B*	3	0.32975	0.443705	0.352905
TAB3	3	-0.096678	0.667974	0.175241	*FAM3D*	4	0.39279	0.443705	0.352905
SIRT4	4	-0.060589	0.667974	0.175241	*MDH1*	4	-0.6072	0.443876	0.352738
CNOT6L	4	0.1251	0.667974	0.175241	*UBE2F*	2	-0.52902	0.443876	0.352738
MVB12A	4	0.2804	0.667974	0.175241	*SYTL4*	4	-0.42561	0.443876	0.352738
TBCEL	4	0.2892	0.667974	0.175241	*DMKN*	3	-0.14493	0.443876	0.352738
TCEA2	4	0.3362	0.667974	0.175241	*POLR2D*	3	0.42488	0.443876	0.352738
ZNF653	4	0.33937	0.667974	0.175241	*ATP5PB*	3	0.48448	0.443876	0.352738
C3orf22	4	0.35329	0.667974	0.175241	*STOX2*	3	0.50991	0.443876	0.352738
RALA	4	0.37292	0.667974	0.175241	*SOWAHC*	3	0.68401	0.443876	0.352738
DDIAS	4	0.39911	0.667974	0.175241	*WDR25*	3	0.71777	0.443876	0.352738
MYOZ1	3	0.41396	0.667974	0.175241	*HINFP*	3	0.77254	0.443876	0.352738
CERS2	3	0.5588	0.667974	0.175241	*DCUN1D3*	4	-0.21248	0.444273	0.35235
EIF3K	3	0.56381	0.667974	0.175241	*GFRA1*	2	0.2118	0.444821	0.351815
CDC6	3	0.78016	0.667974	0.175241	*LETM1*	3	0.6347	0.444821	0.351815
SLC25A20	3	-0.59012	0.667999	0.175224	*MTRNR2L9*	2	0.43765	0.445706	0.350952
RNF41	4	-0.54354	0.667999	0.175224	*ABHD5*	2	0.82147	0.445706	0.350952
PPP1R42	3	-0.36022	0.667999	0.175224	*LGALS7*	1	0.67538	0.445745	0.350913
MINDY3	4	-0.35453	0.667999	0.175224	*RPS3A*	1	1.164	0.445745	0.350913
PLPBP	4	-0.32301	0.667999	0.175224	*ZNF823*	4	-0.45975	0.446047	0.35062
ALKAL2	3	-0.29082	0.667999	0.175224	*ZMYND12*	4	-0.43644	0.446047	0.35062
OTP	3	-0.28691	0.667999	0.175224	*SLC1A7*	4	-0.10894	0.446047	0.35062
ZNF577	3	-0.23902	0.667999	0.175224	*PNPLA4*	4	-0.42492	0.446272	0.350401
DNAH11	4	0.007172	0.667999	0.175224	*XYLT1*	3	-0.74922	0.446964	0.349728
ELMO2	4	0.29978	0.667999	0.175224	*ANGPTL4*	2	-0.79764	0.447316	0.349385
DPY19L3	4	0.3742	0.667999	0.175224	*GALNT12*	3	-0.35714	0.447316	0.349385
PHB	4	0.41284	0.667999	0.175224	*CIZ1*	3	-0.35504	0.447316	0.349385
GBF1	4	0.47593	0.667999	0.175224	*CRELD2*	3	-0.29534	0.447316	0.349385
GOSR2	4	0.53249	0.667999	0.175224	*RALGAPA2*	3	-0.14451	0.447316	0.349385
TOMM34	2	0.63724	0.667999	0.175224	*KLHL8*	4	-0.10381	0.447316	0.349385
SPCS2	1	0.93074	0.667999	0.175224	*BTNL10*	3	0.060233	0.447316	0.349385
OBSL1	3	-0.39055	0.668579	0.174847	*IL1RL2*	4	0.18377	0.447316	0.349385
FAM126B	3	-0.34061	0.668579	0.174847	*RNF123*	4	0.28199	0.447316	0.349385
HMGN2	4	0.41819	0.669083	0.17452	*GABARAPL2*	4	0.35902	0.447316	0.349385
MAP2K5	3	0.47728	0.669083	0.17452	*CFAP157*	3	0.36968	0.447316	0.349385
AURKB	4	0.52222	0.669083	0.17452	*POLG2*	3	0.3768	0.447316	0.349385
TBXT	3	-0.36036	0.670958	0.173305	*TMED9*	3	0.37931	0.447316	0.349385
TANGO6	3	-1.1979	0.671625	0.172873	*STRADA*	4	0.40208	0.447316	0.349385
FAHD1	3	0.3536	0.672106	0.172562	*CTSS*	2	0.4221	0.447316	0.349385
MRFAP1	2	-0.54773	0.672531	0.172288	*FCHSD2*	3	0.42547	0.447316	0.349385
SPIB	2	0.49427	0.673899	0.171405	*KY*	3	0.46997	0.447316	0.349385
ADAM33	3	-0.56318	0.676194	0.169928	*PPP2R5B*	3	0.47689	0.447316	0.349385
TMUB1	3	-0.53317	0.676194	0.169928	*NME5*	3	0.55576	0.447316	0.349385
ANGPTL2	2	-0.49451	0.676194	0.169928	*TMPRSS11A*	3	0.60849	0.447316	0.349385
H1-7	4	-0.49387	0.676194	0.169928	*ARL5B*	3	0.62055	0.447316	0.349385
CCN3	4	-0.34348	0.676194	0.169928	*TSC2*	4	0.62289	0.447316	0.349385
PSMF1	2	-0.31553	0.676194	0.169928	*ECT2L*	3	0.64699	0.447316	0.349385
USP27X	4	-0.18303	0.676194	0.169928	*PSMG4*	3	0.65321	0.447316	0.349385
ZNF133	4	-0.017196	0.676194	0.169928	*EXOC5*	3	0.72473	0.447316	0.349385
EXOSC7	3	0.090165	0.676194	0.169928	*POLR2I*	3	0.89755	0.447316	0.349385
RNF8	4	0.20346	0.676194	0.169928	*TBL2*	4	0.23656	0.448529	0.348209
ALKBH4	4	0.22487	0.676194	0.169928	*CHTF18*	2	0.72399	0.448529	0.348209

ENKD1	4	0.22511	0.676194	0.169928	*PFN1*	4	0.34575	0.450789	0.346027
C11orf94	4	0.25475	0.676194	0.169928	*RINL*	4	0.278	0.451372	0.345465
PSMC6	4	0.28119	0.676194	0.169928	*PRDM7*	4	0.3965	0.451372	0.345465
GRTP1	4	0.30178	0.676194	0.169928	*TMEM164*	2	-0.33254	0.451454	0.345387
PCDHA10	3	0.30835	0.676194	0.169928	*CD59*	4	0.24434	0.451454	0.345387
ZNF432	3	0.3905	0.676194	0.169928	*RAD51C*	4	0.37751	0.451454	0.345387
PIWIL2	3	0.4371	0.676194	0.169928	*AATF*	3	0.4829	0.451454	0.345387
ZSCAN23	4	0.43725	0.676194	0.169928	*FLRT2*	3	0.40326	0.452254	0.344618
B4GALT1	2	0.46997	0.676194	0.169928	*C3orf36*	3	0.42421	0.452254	0.344618
ANKRD49	3	0.87706	0.676194	0.169928	*BEND6*	4	0.31592	0.452336	0.344539
LGALS7B	1	0.92998	0.676194	0.169928	*MFAP1*	4	0.47917	0.452336	0.344539
NOLC1	3	1.1243	0.676194	0.169928	*AURKAIP1*	4	0.4804	0.452336	0.344539
HIC2	4	0.068892	0.677597	0.169029	*PNN*	4	0.22641	0.452546	0.344337
PSG4	3	0.40931	0.68005	0.167459	*YTHDC1*	4	0.30797	0.452546	0.344337
RAB11FIP4	2	0.56603	0.68005	0.167459	*SYNGAP1*	4	0.42232	0.452546	0.344337
KRT18	1	-0.83421	0.680518	0.16716	*EEF2K*	3	-0.65409	0.45297	0.34393
C1orf167	4	0.24493	0.680622	0.167094	*FCGR2B*	1	-0.61017	0.45297	0.34393
SH2D1B	4	0.11251	0.681035	0.166831	*GDAP2*	4	-0.57469	0.45297	0.34393
AJAP1	3	0.11054	0.682532	0.165877	*RPS21*	4	0.50581	0.45297	0.34393
KIF5A	2	0.42107	0.682532	0.165877	*PUS3*	4	0.33202	0.453512	0.343411
TMEM255B	3	-0.24987	0.68306	0.165541	*C1S*	4	0.1797	0.453618	0.34331
TSN	4	0.30283	0.68306	0.165541	*C2CD2*	4	0.2678	0.453814	0.343122
RNF169	4	0.32666	0.68306	0.165541	*LTK*	4	-0.17984	0.454372	0.342588
SNX32	3	0.42974	0.68306	0.165541	*C6orf58*	4	-0.42716	0.4564	0.340655
ATAD1	4	0.64791	0.68306	0.165541	*A4GNT*	3	0.47631	0.457065	0.340022
CLRN3	4	-0.4346	0.683571	0.165216	*TEX261*	3	-0.76786	0.457603	0.339511
BAIAP3	4	0.29085	0.683571	0.165216	*ESPL1*	4	0.35188	0.457603	0.339511
PNPLA2	3	0.28399	0.683665	0.165157	*CTDNEP1*	4	0.37411	0.457603	0.339511
PPRC1	3	0.44562	0.683665	0.165157	*POLR1C*	3	0.91402	0.457603	0.339511
NAA16	3	0.043062	0.684308	0.164748	*TOR1AIP2*	4	-0.38304	0.457633	0.339482
DUSP6	3	-0.71839	0.684499	0.164627	*NOVA2*	4	-0.20945	0.457916	0.339214
YBX1	1	0.51676	0.685566	0.163951	*CHMP7*	3	-0.009817	0.458127	0.339014
SPIN2B	1	0.54091	0.685566	0.163951	*BRCA2*	3	-0.42535	0.45942	0.33779
IRF4	2	0.14563	0.686027	0.163659	*PDCD7*	2	-1.1661	0.4599	0.337337
FAM153B	2	-1.0233	0.686735	0.163211	*VEZT*	2	0.73542	0.461947	0.335407
ERCC6L	3	-0.82864	0.686735	0.163211	*ADAM21*	4	0.23664	0.464262	0.333237
ZNF408	2	-0.79339	0.686735	0.163211	*CCND1*	4	0.25301	0.464262	0.333237
DSC2	2	-0.61511	0.686735	0.163211	*PRKCA*	4	0.38919	0.464262	0.333237
TBCA	2	-0.60684	0.686735	0.163211	*NPC1*	2	0.47584	0.464342	0.333162
INSIG1	3	-0.59643	0.686735	0.163211	*TEKT1*	4	-0.35247	0.464692	0.332835
RBMX	3	-0.55508	0.686735	0.163211	*DYNC1LI1*	4	0.093573	0.46517	0.332388
NBPF3	2	-0.54548	0.686735	0.163211	*C1orf61*	4	0.22087	0.46517	0.332388
ARFIP2	4	-0.5454	0.686735	0.163211	*ASPG*	4	0.35348	0.46517	0.332388
CCNDBP1	3	-0.52538	0.686735	0.163211	*JPT2*	4	0.35653	0.46517	0.332388
AZGP1	4	-0.51943	0.686735	0.163211	*HIBCH*	4	0.4247	0.46517	0.332388
HIBADH	4	-0.50358	0.686735	0.163211	*LRIT3*	2	0.48887	0.46517	0.332388
CLEC6A	3	-0.48698	0.686735	0.163211	*CSF1*	2	-0.47452	0.465446	0.33213
ABAT	3	-0.46795	0.686735	0.163211	*GAPDH*	3	0.4996	0.466874	0.3308
BSG	4	-0.44176	0.686735	0.163211	*SLC35G2*	3	0.50077	0.466874	0.3308
TIMP1	4	-0.43162	0.686735	0.163211	*CCDC33*	4	-0.21736	0.466888	0.330787
BHMT2	4	-0.42537	0.686735	0.163211	*ADARB1*	4	-0.072595	0.466888	0.330787
PTGR1	4	-0.41304	0.686735	0.163211	*PRSS42P*	3	-0.46176	0.467002	0.330681
GTPBP2	4	-0.40611	0.686735	0.163211	*OVCH2*	4	-0.3408	0.467035	0.33065
RPL22L1	4	-0.40363	0.686735	0.163211	*BCAS1*	4	-0.074357	0.467035	0.33065
PLPP1	3	-0.40357	0.686735	0.163211	*AURKB*	4	0.40224	0.467035	0.33065
USP6NL	4	-0.39771	0.686735	0.163211	*IGFN1*	4	0.41981	0.467035	0.33065
SETD7	3	-0.39654	0.686735	0.163211	*CCT5*	3	0.63312	0.467035	0.33065
SLC52A1	2	-0.37469	0.686735	0.163211	*ADD3*	4	-0.4911	0.467235	0.330464

LYSMD4	3	-0.35412	0.686735	0.163211	*AMIGO1*	3	-0.48947	0.467235	0.330464
STMP1	3	-0.34676	0.686735	0.163211	*ZNF200*	3	-0.48915	0.467235	0.330464
GABRQ	4	-0.33659	0.686735	0.163211	*BTN2A2*	4	-0.13268	0.467235	0.330464
ESM1	4	-0.33539	0.686735	0.163211	*HSDL1*	3	0.40387	0.467235	0.330464
DCAF7	4	-0.33364	0.686735	0.163211	*NUP160*	3	0.47108	0.467235	0.330464
GXYLT1	4	-0.31837	0.686735	0.163211	*ZNF589*	2	0.75664	0.470115	0.327796
DIO2	4	-0.31654	0.686735	0.163211	*NOMO1*	1	-0.88078	0.470899	0.327072
KDELR2	3	-0.31025	0.686735	0.163211	*PSMD2*	3	0.097958	0.471473	0.326543
STEAP1B	3	-0.29954	0.686735	0.163211	*IMPA2*	3	0.15456	0.471473	0.326543
ACKR2	4	-0.28844	0.686735	0.163211	*ADM5*	3	0.16882	0.471473	0.326543
KRBOX1	3	-0.21281	0.686735	0.163211	*HDC*	4	0.25927	0.471473	0.326543
EBP	4	-0.17502	0.686735	0.163211	*SPAG7*	4	0.31758	0.471473	0.326543
PSKH1	4	-0.12509	0.686735	0.163211	*NFKBID*	3	0.46611	0.471473	0.326543
NLGN3	4	-0.1104	0.686735	0.163211	*PSMD12*	4	0.5214	0.471473	0.326543
FOXO6	4	-0.080449	0.686735	0.163211	*ARMH1*	3	0.641	0.471473	0.326543
ENPP3	4	-0.031791	0.686735	0.163211	*ALG1L*	2	0.78892	0.471473	0.326543
KATNA1	3	-0.012879	0.686735	0.163211	*ASAH2B*	1	0.8075	0.471473	0.326543
RTL8C	4	0.036424	0.686735	0.163211	*H2AC17*	4	0.19817	0.471706	0.326328
SOAT1	4	0.038081	0.686735	0.163211	*DZIP1*	3	-0.12285	0.472025	0.326035
TNK2	4	0.043454	0.686735	0.163211	*SEC24A*	3	0.1719	0.472025	0.326035
SLC1A4	4	0.098125	0.686735	0.163211	*TOMM20*	3	0.34586	0.472025	0.326035
GABARAPL2	4	0.1125	0.686735	0.163211	*CNEP1R1*	3	0.46119	0.472025	0.326035
TPD52	4	0.13987	0.686735	0.163211	*TTK*	3	1.0542	0.472025	0.326035
LOXL1	3	0.15598	0.686735	0.163211	*FOXD3*	3	-0.73357	0.472275	0.325805
PKMYT1	4	0.16165	0.686735	0.163211	*COMMD6*	3	-0.55532	0.472742	0.325376
EIF1AX	3	0.16673	0.686735	0.163211	*SPATA31C1*	3	-0.52556	0.472742	0.325376
USP1	3	0.17799	0.686735	0.163211	*TAFA1*	4	0.053845	0.472742	0.325376
LYPD6B	4	0.19261	0.686735	0.163211	*STMN3*	4	-0.61347	0.473152	0.325
ACTL10	3	0.1934	0.686735	0.163211	*SLC22A31*	3	0.36483	0.473152	0.325
CDKN2AIP	4	0.20519	0.686735	0.163211	*INTS4*	4	0.57837	0.474222	0.324018
SARS2	3	0.20539	0.686735	0.163211	*SLC19A3*	4	-0.66391	0.474925	0.323375
UBXN6	4	0.21998	0.686735	0.163211	*MXI1*	4	0.27955	0.474979	0.323325
SERPINA12	3	0.2277	0.686735	0.163211	*SERPINE1*	3	-0.27426	0.474999	0.323307
CUTC	4	0.23454	0.686735	0.163211	*NPDC1*	4	0.35964	0.475335	0.323
COPG1	4	0.24912	0.686735	0.163211	*DONSON*	3	1.0702	0.475335	0.323
TOMM20	3	0.25639	0.686735	0.163211	*TTC1*	4	0.46371	0.475895	0.322489
TMEM223	4	0.26097	0.686735	0.163211	*MUC3A*	3	0.27742	0.476873	0.321597
MCMBP	4	0.26214	0.686735	0.163211	*FAM200A*	3	-0.46638	0.477223	0.321279
ACOT8	3	0.27134	0.686735	0.163211	*LRP11*	4	-0.21839	0.477433	0.321088
KCNB2	4	0.27382	0.686735	0.163211	*ZC3H4*	3	-0.45673	0.478288	0.320311
RABAC1	3	0.28477	0.686735	0.163211	*LGR6*	4	-0.014921	0.478288	0.320311
ZNF554	4	0.29764	0.686735	0.163211	*ARID1A*	4	0.45592	0.478288	0.320311
RASGRP2	4	0.29766	0.686735	0.163211	*PLS3*	4	-0.50923	0.479157	0.319523
SLFN13	4	0.31467	0.686735	0.163211	*AHI1*	3	-0.41768	0.479157	0.319523
TRA2A	2	0.33111	0.686735	0.163211	*PHYH*	3	-0.40824	0.479157	0.319523
TRMT6	3	0.33127	0.686735	0.163211	*MYO1C*	3	-0.34018	0.479157	0.319523
DPPA4	4	0.34406	0.686735	0.163211	*MPEG1*	4	-0.31262	0.479157	0.319523
PAK1	3	0.34534	0.686735	0.163211	*PRDM5*	4	-0.29162	0.479157	0.319523
SURF6	4	0.35168	0.686735	0.163211	*SPDYE3*	2	0.37515	0.479157	0.319523
TRIM56	3	0.35439	0.686735	0.163211	*C9orf106*	2	-0.67586	0.48023	0.318551
UBFD1	3	0.35966	0.686735	0.163211	*GHRL*	3	0.51777	0.480368	0.318426
DNAJC10	4	0.36033	0.686735	0.163211	*TRIM7*	2	-0.86536	0.480412	0.318386
NWD1	2	0.362	0.686735	0.163211	*HYAL2*	2	-0.84061	0.480412	0.318386
TAZ	4	0.36437	0.686735	0.163211	*SCARF2*	4	-0.51529	0.480412	0.318386
STK24	2	0.38951	0.686735	0.163211	*RP1L1*	2	-0.48182	0.480412	0.318386
CRYAB	2	0.39332	0.686735	0.163211	*SPDEF*	2	-0.45931	0.480412	0.318386
JADE3	4	0.4026	0.686735	0.163211	*PSME1*	4	-0.41302	0.480412	0.318386
ARMCX6	4	0.4033	0.686735	0.163211	*TTC12*	4	-0.37325	0.480412	0.318386

SEC24C	4	0.41629	0.686735	0.163211	*PLPP2*	4	-0.34639	0.480412	0.318386
GRPEL1	4	0.42655	0.686735	0.163211	*TMEM201*	4	-0.34565	0.480412	0.318386
FAAP20	3	0.42976	0.686735	0.163211	*ZNF814*	4	-0.33792	0.480412	0.318386
HOXD13	3	0.45242	0.686735	0.163211	*NKX3-2*	4	-0.32089	0.480412	0.318386
SPINT2	4	0.52349	0.686735	0.163211	*PCDHB1*	4	-0.32069	0.480412	0.318386
SIRT1	2	0.55504	0.686735	0.163211	*RIPK1*	4	-0.28393	0.480412	0.318386
CIRBP	2	0.58238	0.686735	0.163211	*TMEM266*	3	-0.26339	0.480412	0.318386
VN1R4	1	0.61778	0.686735	0.163211	*FTHL18*	3	-0.25795	0.480412	0.318386
SUPT5H	3	0.62108	0.686735	0.163211	*HOXC4*	4	-0.21487	0.480412	0.318386
GCN1	3	0.62423	0.686735	0.163211	*FASN*	4	-0.15157	0.480412	0.318386
UBA3	4	0.66057	0.686735	0.163211	*GEMIN4*	4	-0.12095	0.480412	0.318386
ATP6V1E1	2	0.67476	0.686735	0.163211	*DPF3*	4	-0.091167	0.480412	0.318386
RPL7A	3	0.78345	0.686735	0.163211	*C1orf159*	3	0.20201	0.480412	0.318386
SNRNP35	4	0.41859	0.686812	0.163162	*TMCO3*	4	0.22157	0.480412	0.318386
SLC5A12	4	-0.36358	0.687222	0.162903	*EIF2AK2*	4	0.2421	0.480412	0.318386
DLK1	4	-0.006045	0.687222	0.162903	*PDXK*	4	0.39747	0.480412	0.318386
EGFR	4	-0.48051	0.688143	0.162321	*CEP104*	3	0.40381	0.480412	0.318386
LPXN	4	-0.34868	0.688143	0.162321	*MGAT5B*	3	0.40962	0.480412	0.318386
C17orf99	3	-0.333	0.688143	0.162321	*CIP2A*	4	0.4258	0.480412	0.318386
ZKSCAN5	3	-0.32802	0.688143	0.162321	*ARL8A*	4	0.42607	0.480412	0.318386
ZNF616	4	-0.32615	0.688143	0.162321	*CENPE*	4	0.44712	0.480412	0.318386
TERB1	4	-0.12359	0.688143	0.162321	*TOR1AIP1*	3	0.48124	0.480412	0.318386
CHRNG	3	0.27377	0.688143	0.162321	*EXOC3L1*	2	0.49007	0.480412	0.318386
TRAPPC10	2	0.27656	0.688143	0.162321	*CCDC136*	3	0.52513	0.480412	0.318386
MAP2K7	3	0.36316	0.688143	0.162321	*HSP90B1*	3	0.58599	0.480412	0.318386
SAP18	3	0.39487	0.688143	0.162321	*PWP1*	3	0.60562	0.480412	0.318386
CLPB	2	0.48039	0.688143	0.162321	*TRAPPC3*	2	0.85835	0.480412	0.318386
SKA2	2	0.53441	0.688143	0.162321	*ECM1*	4	0.29175	0.480802	0.318034
RPN2	3	1.2399	0.688143	0.162321	*PHF20*	4	-0.19674	0.481733	0.317194
MRPL39	4	-0.6915	0.688162	0.162309	*IFNK*	4	0.026285	0.481733	0.317194
KCNJ4	3	-0.68094	0.688162	0.162309	*PCDHB2*	4	-0.52241	0.483451	0.315647
CNTD1	2	-0.56721	0.688162	0.162309	*VAV3*	4	-0.3164	0.483451	0.315647
DENND2B	2	-0.56233	0.688162	0.162309	*CYP4V2*	4	-0.20513	0.483451	0.315647
FOXP2	3	-0.43396	0.688162	0.162309	*ABLIM2*	2	0.44354	0.483451	0.315647
ABCB5	3	-0.41894	0.688162	0.162309	*SETD1A*	2	0.58831	0.483451	0.315647
SYT2	4	-0.37125	0.688162	0.162309	*SPEG*	3	0.49007	0.484376	0.314817
LIN7B	4	-0.37106	0.688162	0.162309	*NDUFB1*	1	0.56631	0.484376	0.314817
C9orf47	4	-0.36033	0.688162	0.162309	*BRD3*	2	-0.66647	0.48441	0.314787
RHOU	4	-0.33808	0.688162	0.162309	*ENOPH1*	3	-0.63151	0.48441	0.314787
ZSCAN9	3	-0.33663	0.688162	0.162309	*MFSD11*	3	-0.54909	0.48441	0.314787
EDA2R	4	-0.32463	0.688162	0.162309	*ZSCAN1*	3	-0.54445	0.48441	0.314787
BLMH	4	-0.31368	0.688162	0.162309	*TJAP1*	4	-0.44035	0.48441	0.314787
RTL8A	3	-0.30523	0.688162	0.162309	*ITLN2*	4	-0.40944	0.48441	0.314787
C4orf19	4	-0.30042	0.688162	0.162309	*FMR1NB*	4	-0.3804	0.48441	0.314787
KCNIP4	4	-0.29963	0.688162	0.162309	*GHITM*	3	-0.34006	0.48441	0.314787
ATP5PO	4	-0.28298	0.688162	0.162309	*C11orf45*	4	-0.25462	0.48441	0.314787
RAB6B	4	-0.25417	0.688162	0.162309	*CCDC18*	3	-0.073153	0.48441	0.314787
SYNJ2	4	-0.22369	0.688162	0.162309	*PAK1IP1*	4	0.001625	0.48441	0.314787
METTL18	4	-0.1432	0.688162	0.162309	*TMEM52*	3	0.013737	0.48441	0.314787
GUCA2B	4	-0.13714	0.688162	0.162309	*ESYT3*	4	0.063166	0.48441	0.314787
CTBP1	4	-0.11503	0.688162	0.162309	*CYP1A2*	4	0.10086	0.48441	0.314787
ATP23	4	-0.1025	0.688162	0.162309	*SELENOI*	4	0.20456	0.48441	0.314787
GPX1	3	-0.036928	0.688162	0.162309	*RSRP1*	4	0.2777	0.48441	0.314787
SLC4A1	4	0.029921	0.688162	0.162309	*ECSCR*	4	0.31691	0.48441	0.314787
E2F1	4	0.036863	0.688162	0.162309	*GLCCI1*	4	0.33302	0.48441	0.314787
IMP3	4	0.1813	0.688162	0.162309	*CCDC137*	4	0.342	0.48441	0.314787
CNTN6	4	0.21927	0.688162	0.162309	*EVPL*	2	0.38843	0.48441	0.314787
NEUROD2	4	0.25021	0.688162	0.162309	*RNF208*	4	0.39213	0.48441	0.314787

INHBB	4	0.25949	0.688162	0.162309	*CACNA2D2*	4	0.4313	0.48441	0.314787
CHST9	4	0.31361	0.688162	0.162309	*DNAJA3*	3	0.46383	0.48441	0.314787
RRN3	3	0.33041	0.688162	0.162309	*DDA1*	3	0.55357	0.48441	0.314787
RCOR2	4	0.33296	0.688162	0.162309	*C1orf50*	4	-0.57343	0.484598	0.314618
LMO7DN	4	0.34659	0.688162	0.162309	*PTGR1*	4	-0.51244	0.484598	0.314618
BTN2A1	4	0.34727	0.688162	0.162309	*DIP2C*	4	-0.34709	0.484598	0.314618
TMEM37	4	0.35907	0.688162	0.162309	*RBM19*	4	0.40055	0.484598	0.314618
ATF4	2	0.40123	0.688162	0.162309	*ZSCAN23*	4	0.40825	0.484895	0.314352
C11orf54	3	0.4085	0.688162	0.162309	*C15orf48*	4	-0.59486	0.4859	0.313453
TPRKB	4	0.45204	0.688162	0.162309	*PSENEN*	4	0.015638	0.4859	0.313453
PHF5A	4	0.4637	0.688162	0.162309	*PIK3C3*	4	0.36478	0.4859	0.313453
PEX6	3	0.49314	0.688162	0.162309	*MB21D2*	4	0.22354	0.486403	0.313003
PRKAB2	3	0.49331	0.688162	0.162309	*COX8A*	4	0.3892	0.486403	0.313003
TSPAN31	4	0.51357	0.688162	0.162309	*WDR7*	4	0.40467	0.486403	0.313003
LHX5	3	0.5312	0.688162	0.162309	*MMP9*	4	-0.43727	0.486887	0.312572
NDE1	3	0.53803	0.688162	0.162309	*FOXP4*	4	-0.3679	0.486887	0.312572
PIK3C3	4	0.53863	0.688162	0.162309	*INSL4*	4	-0.34669	0.486887	0.312572
UBL5	4	0.59724	0.688162	0.162309	*FAM184A*	4	-0.31193	0.486887	0.312572
IL36G	3	0.60821	0.688162	0.162309	*HINT3*	4	-0.30158	0.486887	0.312572
GPX4	3	0.66622	0.688162	0.162309	*SLC35F5*	4	-0.28115	0.486887	0.312572
LSM7	2	0.68546	0.688162	0.162309	*GABPB1*	4	-0.27036	0.486887	0.312572
CXCL1	3	0.69685	0.688162	0.162309	*LAP3*	4	-0.17812	0.486887	0.312572
TBATA	4	-0.55985	0.688621	0.16202	*PTPRJ*	3	-0.14196	0.486887	0.312572
CREB3L2	4	-0.38989	0.688621	0.16202	*IL1A*	4	-0.12516	0.486887	0.312572
FHL3	4	-0.32065	0.688621	0.16202	*PLS1*	3	-0.039414	0.486887	0.312572
BCAR1	2	0.32178	0.688621	0.16202	*PUDP*	4	-0.038846	0.486887	0.312572
CLPX	4	-0.47613	0.689475	0.161481	*PCDHGC4*	2	-0.014931	0.486887	0.312572
NT5C3B	3	0.49961	0.689475	0.161481	*RTN4RL2*	4	0.12986	0.486887	0.312572
ABLIM1	4	-0.25367	0.690022	0.161137	*ZGPAT*	4	0.1302	0.486887	0.312572
GAS7	4	-0.11569	0.690022	0.161137	*GBP4*	4	0.28073	0.486887	0.312572
PAX9	3	0.43912	0.690022	0.161137	*DCTN3*	4	0.31429	0.486887	0.312572
SAPCD2	2	0.52674	0.690801	0.160647	*PRR13*	2	0.31722	0.486887	0.312572
TF	4	0.24001	0.691608	0.16014	*MFAP4*	4	0.3289	0.486887	0.312572
TMEM240	4	0.29126	0.691608	0.16014	*GBE1*	4	0.33818	0.486887	0.312572
ABHD11	4	0.29783	0.691608	0.16014	*KRAS*	4	0.36263	0.486887	0.312572
USP48	4	0.38682	0.691608	0.16014	*SAMD13*	3	0.37649	0.486887	0.312572
SCAPER	4	0.42986	0.691608	0.16014	*OPA1*	3	0.43477	0.486887	0.312572
ASCL2	3	0.54998	0.691608	0.16014	*C20orf78*	3	0.4708	0.486887	0.312572
PCDHB14	3	-0.53548	0.692285	0.159715	*TNPO3*	3	0.52346	0.486887	0.312572
KRT4	3	-0.63283	0.693064	0.159226	*DNA2*	4	0.53631	0.486887	0.312572
CCNI2	4	-0.087681	0.693064	0.159226	*TANGO6*	3	0.56052	0.486887	0.312572
NAPG	2	0.80373	0.693642	0.158865	*FTO*	1	0.58406	0.486887	0.312572
GAL	2	-0.68038	0.694909	0.158072	*CCDC107*	2	0.41315	0.486916	0.312546
TMEM169	3	0.023454	0.694909	0.158072	*MYC*	4	0.35607	0.487495	0.31203
TAC4	3	-0.70328	0.695213	0.157882	*NOP53*	3	0.63893	0.487495	0.31203
TYMP	3	-0.43285	0.695213	0.157882	*RHBDD1*	4	-0.27744	0.487752	0.311801
PRAC1	3	-0.40849	0.695213	0.157882	*ALPK1*	4	0.1307	0.487881	0.311686
RANBP3	3	0.19081	0.695213	0.157882	*MED1*	4	0.23056	0.487881	0.311686
PLSCR4	3	0.53538	0.695213	0.157882	*ZNF143*	4	0.38999	0.487881	0.311686
DRAXIN	3	0.59242	0.695213	0.157882	*PDZK1*	2	0.59332	0.487881	0.311686
RPL7L1	3	0.62947	0.695213	0.157882	*RNASEL*	4	-0.58564	0.487944	0.31163
FAM9A	4	-0.50624	0.695513	0.157694	*RPA1*	4	0.25671	0.487944	0.31163
FOXH1	4	-0.44457	0.695513	0.157694	*INTS11*	3	0.74222	0.487944	0.31163
ALDH8A1	4	-0.25162	0.695513	0.157694	*UTF1*	1	0.76937	0.487944	0.31163
NTF4	3	-0.23094	0.695513	0.157694	*CACNA1D*	3	0.43911	0.488325	0.311291
BTN3A1	4	-0.19734	0.695513	0.157694	*PSMG2*	3	0.5003	0.488325	0.311291
RASSF1	4	0.12434	0.695513	0.157694	*ZNF695*	2	-0.6574	0.488401	0.311224
SLC25A19	4	0.32152	0.695513	0.157694	*FGF12*	2	-0.37785	0.488401	0.311224

KIAA0100	4	0.34525	0.695513	0.157694	*FN3K*	4	0.22692	0.488401	0.311224
HIC1	4	0.35812	0.695513	0.157694	*MCM6*	4	0.23757	0.488401	0.311224
SCTR	3	0.50193	0.695513	0.157694	*ZC3H8*	4	0.29397	0.488401	0.311224
PAF1	3	0.7969	0.695513	0.157694	*CPNE2*	4	0.30557	0.488401	0.311224
PTPN6	3	0.58403	0.695679	0.157591	*RCN1*	2	0.57085	0.488401	0.311224
ZNF280B	3	-0.4377	0.695933	0.157433	*SNRPF*	3	0.94734	0.488401	0.311224
ERGIC1	2	-0.35804	0.695933	0.157433	*PRKACA*	4	-0.52296	0.488656	0.310997
OSBPL11	4	0.10577	0.695933	0.157433	*APOD*	3	-0.056933	0.488656	0.310997
GPR152	4	0.37835	0.695933	0.157433	*HDAC10*	4	0.31709	0.488656	0.310997
IP6K1	4	0.45142	0.695933	0.157433	*BGLAP*	2	0.35775	0.488656	0.310997
PISD	4	0.52248	0.695933	0.157433	*RPS19BP1*	4	0.36947	0.490652	0.309227
ARCN1	4	0.54213	0.695933	0.157433	*RAD54L2*	4	0.25335	0.490994	0.308923
DHPS	4	0.67408	0.695933	0.157433	*GALNT10*	2	0.42641	0.490994	0.308923
JADE2	2	-0.82316	0.699272	0.155354	*LEMD1*	1	0.79774	0.491037	0.308886
SH3D21	2	-0.71451	0.699272	0.155354	*CREBZF*	4	0.373	0.491481	0.308493
ZNF229	3	0.50837	0.699595	0.155153	*HSPA5*	4	0.43141	0.491652	0.308342
TBC1D30	4	-0.45256	0.700274	0.154732	*VDAC2*	2	-0.33084	0.492298	0.307772
CACHD1	4	-0.39861	0.700274	0.154732	*ZNF513*	4	0.24886	0.492298	0.307772
CYP39A1	4	-0.27554	0.700274	0.154732	*TMED3*	4	-0.4578	0.492999	0.307154
PPP1R12C	3	-0.5169	0.700725	0.154452	*ASB4*	4	-0.38886	0.493892	0.306368
MIXL1	4	-0.033817	0.704482	0.15213	*FAM160A1*	4	0.16382	0.493892	0.306368
MCOLN2	4	0.33062	0.706404	0.150947	*KCNAB2*	4	0.18467	0.493892	0.306368
CD52	4	0.35462	0.706404	0.150947	*SIRT4*	4	0.36606	0.493893	0.306367
SLC25A38	4	0.013947	0.706434	0.150928	*MRPS15*	2	0.65432	0.493893	0.306367
FAM217B	1	-0.6604	0.706684	0.150775	*LOC10192843*	4	-0.50056	0.495503	0.304954
C2CD2	4	0.33755	0.706684	0.150775	*PLEKHA5*	4	0.052135	0.495503	0.304954
PPFIBP2	4	0.36335	0.706684	0.150775	*TRAF4*	4	0.15995	0.495503	0.304954
GLIPR2	2	-0.65382	0.707256	0.150424	*KRCC1*	4	-0.069965	0.496222	0.304324
RAB11B	2	0.49005	0.707256	0.150424	*SLC35B1*	3	0.49317	0.496222	0.304324
PON1	3	0.15531	0.707285	0.150405	*HSPBP1*	4	0.032046	0.496389	0.304178
IL6R	3	-0.60396	0.707621	0.150199	*LIMS2*	3	-0.38787	0.497738	0.302999
PDSS2	3	-0.55885	0.707621	0.150199	*MGST3*	4	0.1337	0.498223	0.302576
MANBAL	4	-0.55642	0.707621	0.150199	*DOK7*	3	-0.78835	0.498239	0.302562
CYC1	4	-0.5479	0.707621	0.150199	*MKRN3*	2	-0.50178	0.498239	0.302562
ZC3H7A	3	-0.52919	0.707621	0.150199	*HUNK*	3	-0.39515	0.498239	0.302562
KRT6A	3	-0.49137	0.707621	0.150199	*ACOX3*	4	-0.36367	0.498239	0.302562
WDR6	3	-0.45543	0.707621	0.150199	*CCL20*	4	-0.33458	0.498239	0.302562
RPS6KA4	2	-0.43032	0.707621	0.150199	*ARPP19*	2	-0.33166	0.498239	0.302562
RXFP4	4	-0.42427	0.707621	0.150199	*ZNF277*	4	-0.31342	0.498239	0.302562
ITPR3	3	-0.38514	0.707621	0.150199	*MAGOH*	4	-0.032489	0.498239	0.302562
GABRR2	4	-0.35387	0.707621	0.150199	*DUSP15*	4	0.038458	0.498239	0.302562
KLHL31	4	-0.31693	0.707621	0.150199	*DDIT4*	4	0.1623	0.498239	0.302562
LRRC70	4	-0.31213	0.707621	0.150199	*RLF*	4	0.3163	0.498239	0.302562
ILKAP	4	-0.3032	0.707621	0.150199	*CCDC77*	4	0.32716	0.498239	0.302562
ZBP1	4	-0.27483	0.707621	0.150199	*SLC39A1*	2	0.42061	0.498239	0.302562
TDRP	3	-0.2699	0.707621	0.150199	*SLX4*	4	0.43504	0.498239	0.302562
FASTKD5	4	-0.26699	0.707621	0.150199	*VCP*	4	0.77112	0.498239	0.302562
ZC3H12A	3	-0.24891	0.707621	0.150199	*ANXA8L1*	2	-0.64658	0.4984	0.302422
KLK6	3	-0.2262	0.707621	0.150199	*NOSIP*	2	-0.41071	0.4984	0.302422
TREX2	3	-0.21634	0.707621	0.150199	*SLC22A4*	1	0.58081	0.4984	0.302422
TAS1R1	3	-0.091718	0.707621	0.150199	*TMEM160*	2	-0.85817	0.499529	0.301439
AQP9	3	-0.057985	0.707621	0.150199	*ABL2*	4	-0.048717	0.499898	0.301118
HBE1	4	-0.044013	0.707621	0.150199	*GPRC5B*	4	0.29844	0.500694	0.300428
OLIG3	4	-0.008692	0.707621	0.150199	*MAP4K1*	4	0.30151	0.501393	0.299821
THEG5	3	-0.006676	0.707621	0.150199	*ZCCHC9*	4	0.30063	0.501545	0.29969
ORAI1	4	0.001416	0.707621	0.150199	*CCT2*	3	0.82994	0.501545	0.29969
RNMT	3	0.036617	0.707621	0.150199	*RCHY1*	4	-0.29853	0.502412	0.29894
LPO	4	0.047704	0.707621	0.150199	*HSF1*	3	-0.017271	0.502412	0.29894

ERV3-1	4	0.10462	0.707621	0.150199	*INO80C*	4	0.15632	0.502412	0.29894
MTBP	3	0.11998	0.707621	0.150199	*ITGB5*	4	0.22765	0.502412	0.29894
MRPL50	4	0.19562	0.707621	0.150199	*CCDC38*	4	0.33263	0.502412	0.29894
LRRIQ1	4	0.21671	0.707621	0.150199	*YJU2*	4	0.39517	0.502412	0.29894
PEX26	4	0.30001	0.707621	0.150199	*OSGIN2*	3	0.39911	0.502412	0.29894
DACT2	4	0.32308	0.707621	0.150199	*ABCA4*	3	0.42884	0.502412	0.29894
TADA1	3	0.33346	0.707621	0.150199	*C4orf47*	3	0.46778	0.502412	0.29894
SMIM7	4	0.3473	0.707621	0.150199	*ZNF517*	3	0.54774	0.502412	0.29894
LAS1L	4	0.37852	0.707621	0.150199	*ANKLE2*	3	0.55138	0.502412	0.29894
IL17C	4	0.38212	0.707621	0.150199	*FAM177A1*	4	-0.4785	0.502412	0.29894
NHLH2	4	0.40533	0.707621	0.150199	*SH3TC2*	4	-0.46433	0.502412	0.29894
GYS1	3	0.4495	0.707621	0.150199	*CA8*	4	-0.43327	0.502412	0.29894
CLP1	4	0.45346	0.707621	0.150199	*FDX2*	4	-0.42272	0.502412	0.29894
ASRGL1	2	0.45561	0.707621	0.150199	*HHLA2*	3	-0.35702	0.502412	0.29894
NUCB1	4	0.49116	0.707621	0.150199	*AHSA1*	4	-0.28707	0.502412	0.29894
TXLNG	3	0.51522	0.707621	0.150199	*TRPC5OS*	4	-0.25549	0.502412	0.29894
ITSN2	3	0.63077	0.707621	0.150199	*UEVLD*	4	-0.19877	0.502412	0.29894
POLR3C	3	0.63168	0.707621	0.150199	*TBCEL*	4	0.201	0.502412	0.29894
CDK5RAP3	3	0.64339	0.707621	0.150199	*LTV1*	4	0.22482	0.502412	0.29894
SEC61B	3	0.66867	0.707621	0.150199	*CYP2W1*	4	0.28995	0.502412	0.29894
BYSL	3	0.66978	0.707621	0.150199	*TTBK1*	4	0.32827	0.502412	0.29894
HINFP	3	0.82883	0.707621	0.150199	*RAB6D*	2	0.40115	0.502412	0.29894
SDR42E1	1	0.94977	0.707621	0.150199	*GET3*	4	0.44376	0.502412	0.29894
HEXD	4	-0.28287	0.707656	0.150178	*RNF13*	3	0.58668	0.502412	0.29894
MCCC2	4	-0.26521	0.707656	0.150178	*EMC4*	4	0.25899	0.502467	0.298892
LRGUK	4	0.31967	0.707656	0.150178	*ZNF555*	4	0.34627	0.502467	0.298892
DVL3	3	0.56558	0.707656	0.150178	*AQP6*	2	-0.44497	0.502627	0.298754
SDHB	4	0.62011	0.707656	0.150178	*ANKRD7*	4	0.36674	0.502759	0.29864
KRT15	4	-0.45588	0.707716	0.150141	*COL4A3*	4	0.45819	0.502759	0.29864
B4GALNT3	3	-0.78233	0.708117	0.149895	*WDFY2*	3	-0.015937	0.50302	0.298415
MRPL15	4	0.2583	0.708127	0.149889	*RAD9A*	4	0.40571	0.50302	0.298415
ZNF84	4	0.29831	0.708127	0.149889	*CORT*	2	0.44593	0.50302	0.298415
MGAT1	4	-0.36618	0.708429	0.149704	*ARF5*	3	0.49927	0.50302	0.298415
CLDN3	4	-0.36037	0.708429	0.149704	*THG1L*	4	0.52451	0.50302	0.298415
LRRC3	4	0.031358	0.708429	0.149704	*TARS1*	4	0.58438	0.50302	0.298415
EIF3G	3	0.25152	0.709766	0.148885	*EIF5*	3	0.63555	0.50302	0.298415
LGALS7	1	0.63015	0.709766	0.148885	*IRAK1*	4	-0.00119	0.503213	0.298248
NRG2	1	0.82933	0.709766	0.148885	*NUP107*	4	0.49314	0.503213	0.298248
CIART	4	-0.37845	0.710675	0.148329	*DLX1*	3	0.13222	0.503267	0.298202
LINC01621	1	-0.8862	0.711465	0.147846	*ZNF138*	2	0.36804	0.503267	0.298202
RHBDL1	4	-0.81518	0.711465	0.147846	*ISG20L2*	4	0.017088	0.503358	0.298123
SUMO2	1	-0.62329	0.711465	0.147846	*WFDC1*	4	0.17116	0.503358	0.298123
TOX2	3	-0.61152	0.711465	0.147846	*IFRD1*	4	0.19734	0.503358	0.298123
TBL1X	4	-0.46903	0.711465	0.147846	*GALNS*	4	0.21013	0.503358	0.298123
SLC25A18	4	-0.46873	0.711465	0.147846	*ADD2*	4	0.25715	0.503358	0.298123
UGT1A6	4	-0.44487	0.711465	0.147846	*BICDL2*	4	0.26822	0.503358	0.298123
COX16	2	-0.43994	0.711465	0.147846	*SORD*	2	0.34044	0.503358	0.298123
COX7B	2	-0.42306	0.711465	0.147846	*TSPAN5*	4	0.39203	0.503358	0.298123
SEL1L	3	-0.40255	0.711465	0.147846	*MUC1*	4	-0.12532	0.503885	0.297669
USP17L7	4	-0.38058	0.711465	0.147846	*SIVA1*	3	-0.19296	0.504134	0.297454
SULT2A1	4	-0.36645	0.711465	0.147846	*GK5*	4	-0.33261	0.504544	0.297101
RIMS1	4	-0.35602	0.711465	0.147846	*MRPL39*	4	0.37149	0.504544	0.297101
ASB12	4	-0.31953	0.711465	0.147846	*SAMD4B*	4	0.48875	0.504544	0.297101
VSTM2L	4	-0.31762	0.711465	0.147846	*CDK4*	4	-0.51682	0.504758	0.296917
WDR55	3	-0.31598	0.711465	0.147846	*TYMSOS*	4	-0.069597	0.504758	0.296917
S100Z	4	-0.3098	0.711465	0.147846	*LDLR*	4	0.088431	0.504758	0.296917
SIPA1L2	3	-0.29017	0.711465	0.147846	*RAMP2*	4	0.10488	0.504758	0.296917
HILPDA	4	-0.27822	0.711465	0.147846	*C16orf72*	3	0.16573	0.504758	0.296917

CDK20	3	-0.26551	0.711465	0.147846	*SMC1A*	4	0.21967	0.504758	0.296917
GAS8	4	-0.22972	0.711465	0.147846	*CBLL1*	4	0.31337	0.504758	0.296917
SDHAF1	4	-0.19768	0.711465	0.147846	*PHLDB1*	3	0.34075	0.504758	0.296917
LPAR5	4	-0.16342	0.711465	0.147846	*FAM120AOS*	4	0.38003	0.504758	0.296917
PRX	3	-0.14163	0.711465	0.147846	*IL17RB*	3	0.38148	0.504758	0.296917
ATPAF1	4	-0.11637	0.711465	0.147846	*IQCA1L*	3	0.4083	0.504758	0.296917
USP37	4	-0.068681	0.711465	0.147846	*DYNC1I2*	4	0.41238	0.504758	0.296917
ADCK5	4	-0.048669	0.711465	0.147846	*RASA1*	2	0.43066	0.504758	0.296917
PRSS53	4	-0.04059	0.711465	0.147846	*TOP1*	4	0.44762	0.504758	0.296917
GSTO2	4	-0.01418	0.711465	0.147846	*RAPGEF4*	2	0.56808	0.504758	0.296917
CEL	4	-0.013429	0.711465	0.147846	*ZSWIM9*	4	-0.25559	0.504898	0.296797
PPIL1	4	0.067081	0.711465	0.147846	*RNF5*	3	-0.11793	0.50551	0.29627
ADAT2	4	0.10608	0.711465	0.147846	*YPEL1*	3	-0.5044	0.51025	0.292217
REEP5	4	0.10651	0.711465	0.147846	*SCLT1*	4	-0.10728	0.51025	0.292217
ANO9	4	0.18885	0.711465	0.147846	*CEP44*	3	0.12212	0.51025	0.292217
SPIN1	4	0.20207	0.711465	0.147846	*DDX54*	4	0.20967	0.51025	0.292217
GADD45G	4	0.25942	0.711465	0.147846	*LAIR1*	3	0.24739	0.51025	0.292217
SPATC1L	4	0.28539	0.711465	0.147846	*AK3*	4	0.25504	0.51025	0.292217
HES2	4	0.32548	0.711465	0.147846	*VPS39*	4	0.31034	0.51025	0.292217
MAGEA4	4	0.33392	0.711465	0.147846	*PLIN1*	3	0.35604	0.51025	0.292217
SEC31A	4	0.33527	0.711465	0.147846	*OVOL3*	4	0.38521	0.51025	0.292217
RNF123	4	0.33742	0.711465	0.147846	*VPS72*	3	0.64534	0.51025	0.292217
PRMT7	4	0.34641	0.711465	0.147846	*BCS1L*	3	0.64626	0.51025	0.292217
HAL	3	0.35965	0.711465	0.147846	*FSIP1*	3	-0.36802	0.510253	0.292214
CBX2	4	0.36111	0.711465	0.147846	*PROCA1*	3	-0.31961	0.510253	0.292214
ZNF576	3	0.36375	0.711465	0.147846	*ARL13A*	3	-0.16219	0.510253	0.292214
STRAP	4	0.4087	0.711465	0.147846	*RAC2*	4	0.3841	0.510253	0.292214
CLTCL1	2	0.42777	0.711465	0.147846	*ELAC1*	3	0.41034	0.510253	0.292214
N4BP1	4	0.43424	0.711465	0.147846	*C16orf91*	2	0.50984	0.510253	0.292214
ZNF273	3	0.47103	0.711465	0.147846	*RBP7*	3	0.53992	0.510253	0.292214
BEND3	3	0.47817	0.711465	0.147846	*RTN3*	3	0.55991	0.510253	0.292214
ASH2L	4	0.48016	0.711465	0.147846	*CAMK2D*	3	-0.41514	0.511988	0.29074
DFFA	2	0.48232	0.711465	0.147846	*SLC38A3*	4	0.30288	0.511988	0.29074
EPC1	3	0.51634	0.711465	0.147846	*NBN*	4	0.31334	0.511988	0.29074
CCT7	4	0.54891	0.711465	0.147846	*C3orf70*	3	-0.79962	0.512326	0.290453
POU5F1B	2	0.58488	0.711465	0.147846	*WRAP73*	4	-0.56613	0.512326	0.290453
TAF2	4	0.62381	0.711465	0.147846	*ZC3H12C*	4	-0.55699	0.512326	0.290453
ELOC	1	0.6947	0.711465	0.147846	*CD69*	3	-0.41059	0.512326	0.290453
C2orf81	2	0.69548	0.711465	0.147846	*CYB5R2*	3	-0.39695	0.512326	0.290453
PDS5A	3	0.76473	0.711465	0.147846	*GOLGA7*	4	-0.34896	0.512326	0.290453
NUTF2	3	0.95041	0.711465	0.147846	*RAB23*	3	-0.25424	0.512326	0.290453
RPL14	1	1.5824	0.711465	0.147846	*SEPTIN8*	4	-0.23491	0.512326	0.290453
CELF3	3	-0.52456	0.711693	0.147708	*ATP6V0B*	4	0.089185	0.512326	0.290453
CDKN2AIPNL	4	-0.24792	0.711693	0.147708	*EPM2A*	3	0.14881	0.512326	0.290453
RCE1	4	-0.23072	0.711693	0.147708	*MRE11*	3	0.40036	0.512326	0.290453
MIB1	4	-0.52138	0.712014	0.147512	*UCN*	3	0.53646	0.512326	0.290453
PPDPF	4	-0.26405	0.712014	0.147512	*MOCS3*	2	0.57735	0.512326	0.290453
TUBB6	4	-0.6078	0.712195	0.147401	*TRIM6-TRIM3*	1	-0.53076	0.512561	0.290255
DQX1	4	-0.29463	0.712195	0.147401	*OCLN*	3	-0.40902	0.512561	0.290255
NETO2	3	0.47564	0.712195	0.147401	*SLC30A4*	3	-0.33246	0.512561	0.290255
PRDX1	3	-0.74825	0.712323	0.147323	*DLK2*	3	-0.31548	0.512561	0.290255
EIF2D	4	-0.40604	0.712323	0.147323	*TGM3*	4	-0.19318	0.512561	0.290255
GPATCH3	4	-0.34974	0.712323	0.147323	*MAP3K21*	4	0.2837	0.512561	0.290255
GRM2	4	0.042091	0.712323	0.147323	*CENPH*	4	0.31172	0.512561	0.290255
NFXL1	4	0.072931	0.712323	0.147323	*IGF2R*	3	-0.56969	0.513035	0.289853
MRPL54	4	0.23818	0.712323	0.147323	*CLCN2*	3	-0.45864	0.513035	0.289853
DHFR2	3	0.47092	0.712323	0.147323	*MEP1A*	4	-0.34315	0.513035	0.289853
RAD51	3	0.81118	0.712323	0.147323	*RAD52*	4	-0.11285	0.513035	0.289853

CA2	4	-0.38467	0.712707	0.147089	*RPP38-DT*	4	-0.3549	0.513987	0.289048
XKR9	4	-0.006264	0.712707	0.147089	*TPGS1*	4	0.074896	0.514284	0.288797
PIP4P1	4	0.39101	0.712707	0.147089	*GRM4*	3	-0.3523	0.514489	0.288624
JMJD1C	3	0.49717	0.712707	0.147089	*EFCAB6*	4	0.16092	0.514489	0.288624
TRAM1L1	3	-0.5578	0.714059	0.146266	*NCKAP1L*	2	-0.50058	0.514867	0.288305
JPH1	3	-0.32133	0.714059	0.146266	*NKAIN1*	4	0.14651	0.514867	0.288305
MLX	4	-0.001679	0.714059	0.146266	*AASDH*	4	0.29805	0.514867	0.288305
CLDN9	3	-0.44279	0.714633	0.145917	*AGTPBP1*	4	0.3066	0.514867	0.288305
DMRT1	4	-0.39467	0.714652	0.145906	*GFER*	3	0.63563	0.514867	0.288305
UFSP1	4	-0.12269	0.714652	0.145906	*NFATC2IP*	2	-0.54443	0.516373	0.287036
ZBTB12	4	-0.46731	0.714883	0.145765	*PNCK*	4	0.2354	0.516373	0.287036
SMARCA5	4	-0.14702	0.71608	0.145038	*LAMC2*	4	0.26004	0.516373	0.287036
NPAT	2	0.47269	0.71608	0.145038	*NRIP2*	1	-0.91618	0.517967	0.285698
FTO	1	0.5739	0.71608	0.145038	*HIKESHI*	3	-0.51778	0.517967	0.285698
STK10	3	-0.36357	0.716364	0.144866	*PIK3CD*	4	-0.36292	0.518123	0.285567
SIPA1L3	4	0.41026	0.716364	0.144866	*SLC6A4*	4	0.22282	0.518123	0.285567
AFF1	4	-0.27652	0.716638	0.1447	*CTNND2*	3	-0.72607	0.518227	0.28548
GLRB	4	-0.3248	0.71709	0.144426	*PPL*	2	-0.72155	0.518227	0.28548
AMD1	3	-0.73002	0.717472	0.144195	*FAM90A1*	2	-0.59309	0.518227	0.28548
NGLY1	3	-0.67011	0.717472	0.144195	*TMEM171*	4	-0.5533	0.518227	0.28548
GLI3	4	-0.56649	0.717472	0.144195	*CAAP1*	2	-0.53383	0.518227	0.28548
KCTD13	4	-0.51136	0.717472	0.144195	*XXYLT1*	4	-0.4655	0.518227	0.28548
SHISA9	4	-0.43197	0.717472	0.144195	*ATF6*	4	-0.45553	0.518227	0.28548
ZXDA	3	-0.41337	0.717472	0.144195	*TOX3*	4	-0.39096	0.518227	0.28548
METTL17	3	-0.41137	0.717472	0.144195	*VPS13B*	4	-0.37869	0.518227	0.28548
STARD8	4	-0.38144	0.717472	0.144195	*TRIM15*	4	-0.37478	0.518227	0.28548
ARHGEF40	4	-0.38017	0.717472	0.144195	*FAT2*	4	-0.35118	0.518227	0.28548
AGO4	3	-0.37118	0.717472	0.144195	*ITIH4*	4	-0.34107	0.518227	0.28548
TAF9	3	-0.34517	0.717472	0.144195	*PBLD*	4	-0.22998	0.518227	0.28548
MAPRE2	2	-0.11526	0.717472	0.144195	*CLIC6*	4	-0.089739	0.518227	0.28548
UBP1	4	-0.10199	0.717472	0.144195	*BTK*	4	0.148	0.518227	0.28548
B2M	4	-0.08116	0.717472	0.144195	*B4GALT7*	2	0.16499	0.518227	0.28548
PLEKHN1	3	0.045011	0.717472	0.144195	*PSMA7*	2	0.17774	0.518227	0.28548
COL8A1	4	0.10825	0.717472	0.144195	*ABHD11*	4	0.24223	0.518227	0.28548
NCMAP	4	0.10978	0.717472	0.144195	*CD79A*	4	0.24553	0.518227	0.28548
CHKA	4	0.11917	0.717472	0.144195	*KIN*	4	0.28325	0.518227	0.28548
MAOA	4	0.27306	0.717472	0.144195	*IL17C*	4	0.29186	0.518227	0.28548
ZNF512	4	0.30502	0.717472	0.144195	*SLC52A2*	4	0.30293	0.518227	0.28548
WAC	4	0.3425	0.717472	0.144195	*RPL30*	4	0.35836	0.518227	0.28548
SEC14L5	4	0.34607	0.717472	0.144195	*TOMM40*	4	0.37411	0.518227	0.28548
DEPP1	3	0.35583	0.717472	0.144195	*CNTN5*	4	0.3763	0.518227	0.28548
GBA	3	0.41994	0.717472	0.144195	*ADAM23*	4	0.38283	0.518227	0.28548
KIAA0232	3	0.42177	0.717472	0.144195	*DHRS1*	2	0.38514	0.518227	0.28548
VARS2	4	0.42476	0.717472	0.144195	*LSM6*	4	0.38519	0.518227	0.28548
SBSN	3	0.44592	0.717472	0.144195	*IFNGR2*	3	0.44877	0.518227	0.28548
ZFC3H1	3	0.45156	0.717472	0.144195	*QARS1*	4	0.46807	0.518227	0.28548
RBM42	2	0.45366	0.717472	0.144195	*TOMM34*	2	0.51332	0.518227	0.28548
CCNE1	4	0.45388	0.717472	0.144195	*TTC39C*	4	-0.35127	0.518651	0.285125
DMKN	3	0.45858	0.717472	0.144195	*TMEM183A*	4	0.26127	0.518651	0.285125
EAPP	3	0.48973	0.717472	0.144195	*RAX*	4	0.26631	0.518651	0.285125
SCD	4	0.50111	0.717472	0.144195	*C9orf16*	4	0.40453	0.518651	0.285125
HUS1B	3	0.53845	0.717472	0.144195	*NKIRAS2*	4	0.45732	0.518651	0.285125
TMEM47	3	0.54214	0.717472	0.144195	*DCAF5*	2	0.45897	0.518651	0.285125
LOC388282	3	0.66387	0.717472	0.144195	*NAA50*	4	0.54892	0.518651	0.285125
GOLGA6L9	1	0.91589	0.717472	0.144195	*LTO1*	4	0.70967	0.518651	0.285125
CPSF7	3	-0.70734	0.717542	0.144152	*AHSA2P*	4	0.33515	0.519083	0.284763
GATA1	3	-0.16437	0.717542	0.144152	*CNNM3*	3	-0.41528	0.519602	0.284329
HSD17B7	3	0.093308	0.717542	0.144152	*VSTM5*	4	-0.20243	0.519787	0.284175

ATP1B1	4	0.13673	0.717542	0.144152	*VPS53*	4	0.44584	0.519808	0.284157
PLEKHG4	4	0.28506	0.717542	0.144152	*N4BP2L2*	4	0.46505	0.519808	0.284157
PYGB	3	0.3518	0.717542	0.144152	*FBXO28*	4	0.032785	0.520553	0.283535
WDR5	4	0.37186	0.717542	0.144152	*ACLY*	4	0.53567	0.520553	0.283535
TIPIN	4	0.55185	0.717542	0.144152	*POTEI*	2	0.43467	0.521258	0.282947
NDUFA8	3	0.56208	0.717542	0.144152	*PLEKHG4*	4	0.25042	0.521485	0.282758
TBCB	2	0.57857	0.717542	0.144152	*LOXL2*	4	-0.31738	0.522329	0.282056
LMNA	2	0.67858	0.717542	0.144152	*TNIP3*	4	-0.28961	0.522329	0.282056
TVP23B	2	-0.47294	0.717653	0.144086	*SYNE1*	4	-0.28398	0.522781	0.28168
ERAL1	3	0.091549	0.717653	0.144086	*PAQR9*	3	0.4586	0.522781	0.28168
TRMT11	4	0.10205	0.717653	0.144086	*RALGPS1*	3	-0.45642	0.522841	0.28163
ZFHX3	8	0.261	0.717653	0.144086	*RNASE3*	3	-0.33109	0.522841	0.28163
EMC1	4	0.38027	0.717653	0.144086	*KCTD19*	4	-0.31798	0.522841	0.28163
RGS17	3	-0.45357	0.718303	0.143693	*1-Mar*	3	-0.23023	0.522841	0.28163
H4C5	3	-0.29573	0.718303	0.143693	*C1orf43*	4	0.094114	0.522841	0.28163
TRIM16L	3	-0.19345	0.718303	0.143693	*TTC7B*	4	0.21443	0.522841	0.28163
PPME1	3	0.56827	0.718303	0.143693	*WDR70*	3	0.21645	0.522841	0.28163
GPR37L1	3	-0.45442	0.718958	0.143297	*ACP7*	4	0.24961	0.522841	0.28163
SEC61G	3	-0.84606	0.719665	0.14287	*MTMR14*	4	0.2778	0.522841	0.28163
ZNHIT3	3	-0.70928	0.719665	0.14287	*PCDHB5*	4	0.36914	0.522841	0.28163
C14orf28	3	-0.54705	0.719665	0.14287	*SAMD10*	3	0.48657	0.522841	0.28163
LTC4S	2	-0.38499	0.719665	0.14287	*QPCTL*	2	0.52273	0.522841	0.28163
LOX	4	-0.37332	0.719665	0.14287	*ZNF527*	2	0.63267	0.522841	0.28163
CD160	4	-0.012196	0.719665	0.14287	*URGCP*	4	0.23893	0.523152	0.281372
IGSF9B	4	0.28767	0.719665	0.14287	*PPM1F*	2	-0.53641	0.52384	0.280801
GATAD2A	4	0.29976	0.719665	0.14287	*POLR1A*	4	-0.01025	0.52384	0.280801
ICA1	2	0.39514	0.719665	0.14287	*ATP8A2*	4	0.25912	0.52384	0.280801
APBB2	4	0.45013	0.719665	0.14287	*TDRKH*	4	0.35878	0.52384	0.280801
DNAJC8	4	0.5192	0.719665	0.14287	*ASF1B*	2	0.50514	0.52384	0.280801
CORO1C	4	-0.28225	0.719714	0.14284	*IFRD2*	4	0.057248	0.523964	0.280699
FKBP14	3	-0.4231	0.719812	0.142781	*NRL*	4	-0.40584	0.525282	0.279607
CRHR2	4	-0.20557	0.719812	0.142781	*SLC35E3*	3	0.29404	0.525282	0.279607
PSMB5	4	0.31112	0.719812	0.142781	*EEF2KMT*	3	-0.41355	0.525431	0.279485
C12orf29	4	0.37192	0.719812	0.142781	*RAB11FIP5*	3	-0.39563	0.525431	0.279485
ANKEF1	4	-0.43479	0.720018	0.142657	*ACSF2*	4	0.26838	0.525431	0.279485
MOCOS	4	-0.37232	0.720018	0.142657	*CPNE7*	4	0.31691	0.525431	0.279485
IGFBPL1	4	-0.23354	0.720018	0.142657	*UBE2W*	3	0.33988	0.525431	0.279485
ZCCHC24	4	0.068426	0.720224	0.142532	*TBCK*	2	0.53875	0.525431	0.279485
POLH	4	-0.58419	0.721357	0.14185	*OGT*	4	0.65003	0.525431	0.279485
HNRNPM	3	0.6362	0.721639	0.14168	*PRPF6*	2	0.73496	0.525431	0.279485
CENPW	3	0.64951	0.721639	0.14168	*TBX3*	2	-0.62697	0.525596	0.279348
BIRC3	3	0.35574	0.72175	0.141613	*PIK3R2*	4	-0.51821	0.525596	0.279348
RAN	4	0.57488	0.72175	0.141613	*TBC1D10A*	4	-0.15935	0.525596	0.279348
CCDC112	4	-0.54723	0.722057	0.141429	*SHARPIN*	2	-0.30659	0.527098	0.278109
PHF20	4	-0.32896	0.722057	0.141429	*GGT7*	3	-0.37146	0.529665	0.275999
MED31	4	-0.26052	0.722057	0.141429	*SLC25A30*	3	-0.52453	0.529983	0.275738
EVI2A	4	-0.25514	0.722057	0.141429	*ACOT4*	3	-0.53128	0.530114	0.275631
ANP32A	3	-0.24852	0.722057	0.141429	*ADAM33*	3	-0.44962	0.530114	0.275631
PHF8	4	0.14956	0.722057	0.141429	*TUFT1*	2	0.51613	0.530114	0.275631
DEPDC7	4	0.29797	0.722057	0.141429	*DCST2*	4	0.33767	0.531288	0.27467
TMCC1	4	0.31181	0.722057	0.141429	*CAB39*	3	0.43588	0.531288	0.27467
CDON	4	0.44282	0.722057	0.141429	*GGA3*	3	0.59751	0.53169	0.274341
PMEL	1	-1.0117	0.722634	0.141081	*RABEPK*	4	0.25636	0.531795	0.274256
KLF10	4	0.26624	0.722634	0.141081	*TUSC2*	4	0.086795	0.531918	0.274156
DISP2	4	0.15033	0.722852	0.140951	*COX19*	4	0.11191	0.531918	0.274156
B3GAT2	2	-0.90159	0.723245	0.140715	*POMK*	4	0.33866	0.532701	0.273517
TM4SF20	2	-0.50931	0.723245	0.140715	*DAP*	4	0.4808	0.533186	0.273121
UQCRC2	4	-0.14578	0.723245	0.140715	*NME3*	4	-0.59566	0.533732	0.272677

DAPP1	4	-0.094504	0.723245	0.140715	*ZNF688*	4	-0.19893	0.533732	0.272677
RASGRF1	4	-0.086775	0.723245	0.140715	*ENDOU*	1	0.45719	0.533934	0.272513
TMEM179B	4	0.027579	0.723245	0.140715	*PREPL*	4	-0.095383	0.53422	0.27228
PCDHGA3	4	0.15724	0.723245	0.140715	*VAMP1*	3	0.53287	0.535681	0.271094
NEK3	4	0.22129	0.723245	0.140715	*RRP36*	2	0.58135	0.535681	0.271094
SIGIRR	4	0.29747	0.723245	0.140715	*MCM2*	3	0.42549	0.536178	0.270691
RHEB	2	0.31141	0.723245	0.140715	*CHCHD4*	3	0.093079	0.53666	0.270301
ORC2	4	0.31925	0.723245	0.140715	*ADA*	4	0.46379	0.537254	0.26982
EHD1	4	0.33314	0.723245	0.140715	*CPSF7*	3	-0.52498	0.537284	0.269796
GLMN	4	0.34432	0.723245	0.140715	*KLHL20*	4	0.049191	0.537284	0.269796
PSORS1C1	3	0.34865	0.723245	0.140715	*NT5C3B*	3	0.10001	0.537284	0.269796
TFRC	4	0.36151	0.723245	0.140715	*IWS1*	3	0.21799	0.537284	0.269796
SETX	3	0.54007	0.723245	0.140715	*PCNX3*	4	0.24425	0.537284	0.269796
TMPRSS9	3	0.62425	0.723245	0.140715	*AFTPH*	4	0.26313	0.537284	0.269796
VAPA	3	0.63767	0.723245	0.140715	*TLE2*	4	0.27471	0.537284	0.269796
DCTN6	2	0.77203	0.723245	0.140715	*BCAP31*	3	0.44909	0.537284	0.269796
NEK9	4	0.26364	0.725832	0.139164	*CLNS1A*	4	0.56873	0.537284	0.269796
NT5C2	3	-0.54815	0.726779	0.138598	*LRPPRC*	3	0.64454	0.537284	0.269796
NDUFV2	3	0.57626	0.726779	0.138598	*RDM1*	1	0.67072	0.537284	0.269796
MTMR7	4	-0.34534	0.726838	0.138562	*UGT2A2*	1	0.68639	0.537284	0.269796
MMP19	3	-0.085517	0.726838	0.138562	*ANKRD34A*	2	0.1464	0.537345	0.269747
MRGPRF	3	0.0232	0.726838	0.138562	*PROM2*	4	0.39255	0.537345	0.269747
KRT34	2	0.45727	0.726838	0.138562	*MIR1-1HG*	4	-0.29847	0.538048	0.269179
PCDHA1	3	0.654	0.726838	0.138562	*C3orf20*	4	-0.24174	0.538248	0.269018
AK9	3	-0.35425	0.726869	0.138544	*CAPNS1*	4	0.095527	0.538248	0.269018
ZNF821	2	0.38062	0.72725	0.138316	*PNPLA7*	2	0.68031	0.538264	0.269005
SYTL3	4	-0.55436	0.72735	0.138257	*ADGRL2*	4	0.33431	0.538322	0.268958
ARG1	4	-0.46263	0.72735	0.138257	*SLC24A2*	3	-0.55464	0.538756	0.268607
SLC25A26	4	-0.38452	0.72735	0.138257	*COL18A1*	4	-0.48027	0.538912	0.268482
PHOX2A	4	-0.10131	0.72735	0.138257	*PAQR3*	2	-0.35792	0.538912	0.268482
PCDHB12	2	0.39181	0.72735	0.138257	*LINC01620*	3	-0.14213	0.538912	0.268482
ARRB2	3	-0.72149	0.727372	0.138243	*SUGP2*	4	-0.14144	0.538912	0.268482
EPB41L2	4	-0.5737	0.727372	0.138243	*SPAST*	4	-0.13514	0.538912	0.268482
VAMP8	4	-0.39003	0.727372	0.138243	*TMEM151B*	4	-0.056283	0.538912	0.268482
ZKSCAN8	4	-0.37678	0.727372	0.138243	*DIPK2A*	4	0.042238	0.538912	0.268482
RAP2C	4	-0.35426	0.727372	0.138243	*FLT3LG*	4	0.19273	0.538912	0.268482
ACYP2	3	-0.3387	0.727372	0.138243	*RTN4IP1*	3	0.23103	0.538912	0.268482
C17orf75	4	-0.16657	0.727372	0.138243	*NIBAN3*	4	0.23889	0.538912	0.268482
SEMA3E	3	-0.048152	0.727372	0.138243	*MDC1*	3	0.24409	0.538912	0.268482
INTS5	4	0.31229	0.727372	0.138243	*WIPF2*	4	0.29629	0.538912	0.268482
ZNF235	3	0.35643	0.727372	0.138243	*PLCG2*	4	0.30097	0.538912	0.268482
COLCA2	4	0.36513	0.727372	0.138243	*SEC23B*	3	0.37936	0.538912	0.268482
MCM5	4	0.40673	0.727372	0.138243	*SEC23A*	4	0.43003	0.538912	0.268482
SNRNP70	4	0.43582	0.727372	0.138243	*SLC41A3*	4	0.44931	0.538912	0.268482
ATM	4	0.43664	0.727372	0.138243	*CRB3*	3	0.45124	0.538912	0.268482
STOX2	3	0.49079	0.727372	0.138243	*MRPS27*	3	0.45475	0.538912	0.268482
PREB	4	0.55209	0.727372	0.138243	*MMS22L*	4	0.49094	0.538912	0.268482
LAMTOR4	3	0.15732	0.728247	0.137721	*OSGIN1*	4	0.50507	0.538912	0.268482
RRP15	4	0.069861	0.728278	0.137703	*TLX1*	3	0.54665	0.538912	0.268482
LTA	4	-0.63268	0.728474	0.137586	*MBD5*	3	0.56812	0.538912	0.268482
CBR3	2	-0.77277	0.728591	0.137516	*BUD13*	4	0.57156	0.538912	0.268482
MED7	4	-0.70657	0.728591	0.137516	*STK17A*	3	0.60115	0.538912	0.268482
TMEM106C	4	-0.49693	0.728591	0.137516	*TM7SF2*	1	0.69602	0.538912	0.268482
APC	3	-0.38624	0.728591	0.137516	*SMARCA1*	4	-0.47306	0.539261	0.268201
KCNK5	3	-0.38396	0.728591	0.137516	*ATXN7L1*	4	0.20862	0.539261	0.268201
ALG12	3	-0.33901	0.728591	0.137516	*FKBP9*	3	0.50564	0.54336	0.264912
SESN1	4	-0.31285	0.728591	0.137516	*ARMCX2*	4	-0.50204	0.544697	0.263845
SUPT20H	3	-0.29402	0.728591	0.137516	*DNAJC6*	2	-0.52309	0.545668	0.263071

ABCD3	4	-0.15125	0.728591	0.137516	*SNRPB2*	3	-0.21792	0.545668	0.263071
NUMBL	4	-0.054468	0.728591	0.137516	*EDC4*	4	0.13025	0.545668	0.263071
LLGL2	4	0.092095	0.728591	0.137516	*EIF3M*	4	0.13785	0.545668	0.263071
KCTD3	4	0.098868	0.728591	0.137516	*SULT1A1*	4	0.14717	0.545668	0.263071
TPGS1	4	0.14621	0.728591	0.137516	*NETO1*	4	0.31268	0.545668	0.263071
CEACAM3	4	0.25046	0.728591	0.137516	*TEX29*	3	0.46047	0.545668	0.263071
GNG3	4	0.28095	0.728591	0.137516	*TRMT10B*	3	-0.33495	0.545846	0.26293
VPS35L	4	0.28249	0.728591	0.137516	*BCL9L*	3	0.4626	0.545927	0.262866
CNBP	4	0.28633	0.728591	0.137516	*PCDH20*	3	-0.61283	0.546061	0.262759
GALNT8	4	0.29044	0.728591	0.137516	*WDR72*	3	-0.57473	0.546061	0.262759
CPLANE1	2	0.31757	0.728591	0.137516	*TMLHE*	3	-0.44869	0.546061	0.262759
MRPL34	4	0.33515	0.728591	0.137516	*ENDOV*	4	-0.25557	0.546061	0.262759
ITGAV	4	0.35592	0.728591	0.137516	*TARDBP*	3	-0.23523	0.546061	0.262759
RASSF6	4	0.36513	0.728591	0.137516	*TPM3*	3	0.001224	0.546061	0.262759
SPDYE3	2	0.36922	0.728591	0.137516	*CD86*	3	0.11951	0.546061	0.262759
GSN	4	0.36952	0.728591	0.137516	*TCAIM*	4	0.21332	0.546061	0.262759
DKC1	4	0.38976	0.728591	0.137516	*NOL10*	4	0.21558	0.546061	0.262759
UBE2H	4	0.46312	0.728591	0.137516	*UBB*	3	0.21589	0.546061	0.262759
NDUFB8	2	0.51012	0.728591	0.137516	*NAIF1*	3	0.26482	0.546061	0.262759
ARL5B	3	0.61164	0.728591	0.137516	*ACY3*	3	0.27808	0.546061	0.262759
ZNF24	1	0.68471	0.728591	0.137516	*MAIP1*	4	0.29529	0.546061	0.262759
PCID2	4	0.71414	0.728662	0.137474	*TIMM17B*	2	0.35178	0.546061	0.262759
C17orf102	4	-0.32594	0.728814	0.137383	*SRSF9*	4	0.39026	0.546061	0.262759
TBX10	1	-0.84242	0.728875	0.137347	*PPP6C*	4	0.4186	0.546061	0.262759
GTSCR1	3	-0.4713	0.728875	0.137347	*ZNF207*	4	0.44045	0.546061	0.262759
KATNBL1	4	-0.36724	0.728875	0.137347	*CDPF1*	3	0.44093	0.546061	0.262759
ENTPD3	3	-0.16688	0.728875	0.137347	*CNOT9*	4	0.49161	0.546061	0.262759
RPP38-DT	4	-0.053882	0.728875	0.137347	*TMEM30A*	4	0.58623	0.546061	0.262759
UGT1A7	4	0.17251	0.728875	0.137347	*CDK5RAP3*	3	0.65084	0.546061	0.262759
MEGF10	4	0.26571	0.728875	0.137347	*JMJD6*	2	0.71216	0.546061	0.262759
RNASEH2A	4	0.31605	0.728875	0.137347	*SEC22B*	2	0.75943	0.546061	0.262759
SASH1	4	-0.43918	0.728952	0.137301	*ZFP36*	2	-0.50087	0.547134	0.261907
SCGB1A1	4	0.069621	0.728952	0.137301	*ELK1*	3	0.50724	0.549073	0.26037
LRRC32	4	0.23116	0.728952	0.137301	*POLR3H*	3	0.91895	0.549073	0.26037
NDUFS7	4	0.34833	0.728952	0.137301	*KCTD21*	4	0.19756	0.549274	0.260211
ZNRD2	4	-0.17133	0.72905	0.137243	*APBA3*	3	-0.041365	0.549608	0.259947
KLK3	3	0.35057	0.729825	0.136781	*GEMIN2*	2	0.72096	0.549608	0.259947
GDF7	3	0.39912	0.73079	0.136207	*ZMYND8*	4	-0.38458	0.549876	0.259735
ZBTB14	3	0.14307	0.731117	0.136013	*NUDT16L1*	4	-0.057116	0.550009	0.25963
NUDT21	4	0.7228	0.732731	0.135055	*SLC25A38*	4	-0.41175	0.550856	0.258962
PLIN4	4	-0.00974	0.7328	0.135015	*FLT4*	4	-0.35915	0.550856	0.258962
GNG10	1	0.54363	0.733207	0.134774	*RRAS*	4	-0.21708	0.551272	0.258634
UPP1	4	-0.12903	0.733329	0.134701	*NDUFAF3*	4	0.33283	0.551594	0.258381
GALE	3	0.42955	0.734371	0.134084	*TAX1BP1*	4	-0.60322	0.55265	0.25755
TRPV6	3	0.092688	0.734709	0.133885	*UTP23*	4	0.16394	0.55265	0.25755
CCL28	4	-0.44272	0.735081	0.133665	*GNGT1*	4	0.32334	0.55265	0.25755
MAB21L3	4	-0.42581	0.735081	0.133665	*FAM180B*	3	0.33438	0.552754	0.257468
CC2D1A	4	-0.42011	0.735081	0.133665	*SLCO2B1*	4	0.33492	0.552831	0.257407
JARID2	3	-0.40827	0.735081	0.133665	*PCBP3*	3	-0.51173	0.553009	0.257268
KANK1	4	-0.36626	0.735081	0.133665	*C11orf42*	4	-0.46844	0.553009	0.257268
HEXB	4	-0.19293	0.735081	0.133665	*SSR4*	4	0.029062	0.553009	0.257268
MEPCE	4	-0.066728	0.735081	0.133665	*CDV3*	3	0.038755	0.553009	0.257268
KREMEN2	3	0.009272	0.735081	0.133665	*ATP2B2*	3	0.047468	0.553009	0.257268
ZBTB39	4	0.021166	0.735081	0.133665	*TTC19*	4	0.35912	0.553009	0.257268
KLHL11	4	0.099054	0.735081	0.133665	*PAIP1*	3	0.47847	0.553009	0.257268
GNG7	3	0.11144	0.735081	0.133665	*HCN3*	3	0.48017	0.553009	0.257268
ABCC5	4	0.11175	0.735081	0.133665	*PROCR*	4	0.50334	0.553009	0.257268
FGFBP1	4	0.11783	0.735081	0.133665	*PLPP1*	3	-0.61579	0.553392	0.256967

ARNT2	4	0.12536	0.735081	0.133665	*DAND5*	4	-0.59802	0.553392	0.256967
OPLAH	4	0.22195	0.735081	0.133665	*GAL3ST3*	3	-0.46156	0.553392	0.256967
COCH	2	0.22602	0.735081	0.133665	*POLI*	4	-0.41948	0.553392	0.256967
ITGAE	4	0.29967	0.735081	0.133665	*PANX1*	4	-0.28457	0.553392	0.256967
SEMA3A	4	0.30397	0.735081	0.133665	*SELENOF*	3	-0.23806	0.553392	0.256967
KPNA1	3	0.32386	0.735081	0.133665	*BRWD1*	4	-0.15591	0.553392	0.256967
NMD3	4	0.35354	0.735081	0.133665	*ERBB3*	4	-0.023594	0.553392	0.256967
PARD6A	4	0.38925	0.735081	0.133665	*FNBP1*	4	0.06005	0.553392	0.256967
LINGO1	4	0.39201	0.735081	0.133665	*GTSCR1*	3	0.10745	0.553392	0.256967
EIF4E	2	0.45543	0.735081	0.133665	*VPREB3*	3	0.15399	0.553392	0.256967
GRM1	4	0.46314	0.735081	0.133665	*ARNT2*	4	0.18023	0.553392	0.256967
ATP10B	3	0.4799	0.735081	0.133665	*MTMR11*	4	0.23804	0.553392	0.256967
RPLP0	4	0.48047	0.735081	0.133665	*C6orf120*	4	0.2719	0.553392	0.256967
ZCRB1	4	0.50631	0.735081	0.133665	*TBX20*	4	0.27448	0.553392	0.256967
EIF2B3	4	0.54152	0.735081	0.133665	*ERBB4*	3	0.31728	0.553392	0.256967
ANKLE2	3	0.57627	0.735081	0.133665	*RALBP1*	4	0.32917	0.553392	0.256967
ALYREF	2	0.5967	0.735081	0.133665	*MUCL3*	3	0.40617	0.553392	0.256967
XRN2	4	0.62392	0.735081	0.133665	*ZFX*	4	0.43606	0.553392	0.256967
MDN1	3	0.81841	0.735081	0.133665	*LBP*	3	0.46656	0.553392	0.256967
VPS28	4	0.35114	0.735614	0.13335	*WDR1*	3	0.47254	0.553392	0.256967
SPATA20	2	0.43876	0.735614	0.13335	*MSANTD1*	2	0.53075	0.553392	0.256967
DEPDC4	4	0.18818	0.73612	0.133052	*E2F3*	3	0.54138	0.553392	0.256967
CBY2	2	0.069114	0.736598	0.132769	*IL32*	3	0.54444	0.553392	0.256967
COQ3	3	-0.67469	0.7368	0.13265	*RPS13*	2	0.63078	0.553392	0.256967
CFAP206	1	-0.66784	0.7368	0.13265	*LIN52*	2	0.90313	0.553392	0.256967
NT5C1A	4	-0.64401	0.7368	0.13265	*RNGTT*	4	0.18467	0.553681	0.256741
KCNAB1	3	-0.50309	0.7368	0.13265	*UVRAG*	4	0.28689	0.554009	0.256483
LBHD1	2	-0.46907	0.7368	0.13265	*VPS37B*	3	-0.45451	0.555753	0.255118
RIMKLA	3	-0.31997	0.7368	0.13265	*PLEKHO1*	2	0.25279	0.555753	0.255118
STOX1	4	-0.086654	0.7368	0.13265	*OR7D2*	2	-0.46803	0.556401	0.254612
SHLD1	4	0.021741	0.7368	0.13265	*LINS1*	3	0.17798	0.556401	0.254612
MSLN	4	0.15048	0.7368	0.13265	*TMEM183B*	3	0.26821	0.556401	0.254612
PRELID1	4	0.27239	0.7368	0.13265	*RBM24*	2	-0.5321	0.556842	0.254268
TERF2IP	4	0.27638	0.7368	0.13265	*MIA3*	3	0.46791	0.556842	0.254268
PIGS	4	0.50474	0.7368	0.13265	*EIF1AX*	3	0.6737	0.556842	0.254268
FIGNL1	4	-0.43707	0.736852	0.13262	*CNOT3*	3	0.88158	0.556842	0.254268
RAPGEF3	3	-0.40967	0.736852	0.13262	*GARS1*	4	0.41036	0.557432	0.253808
MRPS31	4	-0.40765	0.736852	0.13262	*CASC3*	3	0.30441	0.557566	0.253704
MAGEH1	4	-0.37824	0.736852	0.13262	*BMP8A*	3	0.38599	0.557566	0.253704
RNASEL	4	-0.35767	0.736852	0.13262	*LCE5A*	4	-0.17845	0.557758	0.253554
SMPD4	4	-0.28261	0.736852	0.13262	*CHD4*	4	0.22676	0.558426	0.253034
CBX7	4	-0.26083	0.736852	0.13262	*CCDC17*	2	-0.60736	0.558591	0.252906
XPNPEP3	4	-0.045433	0.736852	0.13262	*CCDC27*	3	0.005159	0.559383	0.25229
DKK1	4	0.05857	0.736852	0.13262	*F12*	4	0.32359	0.559383	0.25229
SLC22A31	3	0.22601	0.736852	0.13262	*FBXO42*	3	0.34273	0.559383	0.25229
C4orf33	2	0.24842	0.736852	0.13262	*NAA16*	3	0.53987	0.559383	0.25229
CASP4	3	0.28788	0.736852	0.13262	*IBA57*	2	0.3576	0.560352	0.251539
SMARCA2	4	0.29358	0.736852	0.13262	*CHERP*	4	-0.037308	0.562507	0.249872
MTCH2	4	0.33586	0.736852	0.13262	*C1orf229*	3	0.4571	0.562565	0.249828
LIN52	2	0.92565	0.736852	0.13262	*ZNF311*	4	-0.52463	0.564427	0.248392
RPL15	1	1.0532	0.736852	0.13262	*GSTM4*	2	-0.38371	0.565659	0.247445
TMEM218	2	0.51551	0.737034	0.132513	*CNTD2*	2	0.58105	0.565659	0.247445
NOP58	2	0.66672	0.737034	0.132513	*POU5F1B*	2	0.48775	0.566084	0.247119
CLEC18B	3	0.41485	0.737901	0.132002	*PPP3R1*	4	-0.37232	0.566088	0.247116
RHBDL3	3	-0.69253	0.738353	0.131736	*UNC50*	4	0.087466	0.566183	0.247043
REPS2	3	-0.45343	0.738353	0.131736	*GTF2H5*	4	-0.52296	0.566211	0.247022
NR3C2	3	-0.41227	0.738353	0.131736	*TAOK3*	3	-0.28484	0.566211	0.247022
TMEM225B	3	-0.011671	0.738353	0.131736	*C9orf78*	4	0.31015	0.566211	0.247022

DCAF4L1	3	0.18205	0.738353	0.131736	*NAE1*	4	0.3545	0.566211	0.247022
C1orf216	4	-0.55097	0.738414	0.1317	*RAB40C*	4	-0.30662	0.566372	0.246899
PSEN1	4	-0.31626	0.738414	0.1317	*ASCC1*	3	-0.856	0.566607	0.246718
ALKAL1	4	-0.29875	0.738414	0.1317	*LINC01619*	3	-0.72702	0.566607	0.246718
DHRS9	4	-0.28105	0.738414	0.1317	*AKAP1*	3	-0.62304	0.566607	0.246718
SFTA3	3	-0.25578	0.738414	0.1317	*HSPH1*	4	-0.57868	0.566607	0.246718
GBX1	4	0.001848	0.738414	0.1317	*RGMA*	2	-0.46483	0.566607	0.246718
LRRC27	4	0.18742	0.738414	0.1317	*KLHL28*	3	-0.45812	0.566607	0.246718
HMBOX1	4	0.30587	0.738414	0.1317	*EGR2*	3	-0.4466	0.566607	0.246718
CALR	4	0.31527	0.738414	0.1317	*SLC10A7*	4	-0.4314	0.566607	0.246718
PLBD2	4	0.35841	0.738414	0.1317	*TMEM63A*	4	-0.40147	0.566607	0.246718
TMEM120A	3	0.67423	0.738414	0.1317	*GPR84*	4	-0.38008	0.566607	0.246718
MAN2A2	4	-0.47116	0.73855	0.13162	*TIMM8A*	4	-0.37462	0.566607	0.246718
L3MBTL1	4	-0.29451	0.73855	0.13162	*MNT*	2	-0.37355	0.566607	0.246718
TBKBP1	4	-0.049308	0.73855	0.13162	*PLAAT2*	3	-0.36943	0.566607	0.246718
PRR16	3	0.03136	0.73855	0.13162	*EPHB2*	4	-0.356	0.566607	0.246718
NOL11	3	0.042402	0.73855	0.13162	*C8orf88*	4	-0.35465	0.566607	0.246718
PTCH1	3	0.094208	0.73855	0.13162	*GLO1*	4	-0.34373	0.566607	0.246718
THOC5	4	0.14214	0.73855	0.13162	*STPG2*	4	-0.31895	0.566607	0.246718
RASSF2	4	0.3137	0.73855	0.13162	*COLQ*	4	-0.30562	0.566607	0.246718
TBC1D22A	3	0.41857	0.73855	0.13162	*KCNAB1*	3	-0.2984	0.566607	0.246718
MRAS	2	0.46494	0.73855	0.13162	*CLDN23*	3	-0.29817	0.566607	0.246718
KLF4	2	0.51132	0.73855	0.13162	*GMIP*	3	-0.25787	0.566607	0.246718
WNK1	3	0.60484	0.73855	0.13162	*CCS*	4	-0.25778	0.566607	0.246718
INO80	4	0.61738	0.73855	0.13162	*ATP4A*	4	-0.24989	0.566607	0.246718
C15orf62	3	0.27972	0.738808	0.131468	*STXBP5*	3	-0.23555	0.566607	0.246718
IL15RA	3	-0.26906	0.738884	0.131424	*EPHA2*	3	-0.22668	0.566607	0.246718
AFAP1L2	3	-0.48613	0.739148	0.131269	*HFM1*	4	-0.2203	0.566607	0.246718
ODF2	4	-0.47132	0.739148	0.131269	*BCL2L13*	3	-0.19839	0.566607	0.246718
LRRC8A	4	-0.45604	0.739148	0.131269	*KAZN*	4	-0.17645	0.566607	0.246718
CCNE2	4	-0.44439	0.739148	0.131269	*PLA2G2F*	3	-0.14692	0.566607	0.246718
ARHGAP30	4	-0.35444	0.739148	0.131269	*TCTN1*	3	-0.10218	0.566607	0.246718
RABGAP1	4	-0.44445	0.740522	0.130462	*ZNF891*	4	0.023743	0.566607	0.246718
PDSS1	3	-0.42875	0.740522	0.130462	*BBS12*	4	0.09034	0.566607	0.246718
MOCS2	4	-0.40532	0.740522	0.130462	*FXYD5*	4	0.11898	0.566607	0.246718
ZBTB40	3	-0.38491	0.740522	0.130462	*FUBP1*	4	0.15978	0.566607	0.246718
FAM117A	4	-0.38305	0.740522	0.130462	*KHNYN*	4	0.19473	0.566607	0.246718
WDR89	3	-0.3706	0.740522	0.130462	*IGFBP1*	4	0.20888	0.566607	0.246718
UFSP2	4	-0.36776	0.740522	0.130462	*DCTN1*	4	0.2236	0.566607	0.246718
BAG6	4	-0.21845	0.740522	0.130462	*PUS1*	4	0.22539	0.566607	0.246718
HFM1	4	-0.15335	0.740522	0.130462	*WDYHV1*	4	0.23442	0.566607	0.246718
SARAF	4	-0.15065	0.740522	0.130462	*FNIP2*	4	0.24262	0.566607	0.246718
C12orf75	4	-0.13531	0.740522	0.130462	*USP10*	4	0.24494	0.566607	0.246718
CRISP3	4	-0.12514	0.740522	0.130462	*C11orf54*	3	0.25364	0.566607	0.246718
AGMAT	3	0.053529	0.740522	0.130462	*TDRD12*	3	0.255	0.566607	0.246718
RDX	4	0.15692	0.740522	0.130462	*ZNF329*	4	0.25686	0.566607	0.246718
EFHB	4	0.17094	0.740522	0.130462	*NHEJ1*	4	0.25892	0.566607	0.246718
SMYD5	4	0.20032	0.740522	0.130462	*TRMT10C*	3	0.2634	0.566607	0.246718
RXRB	4	0.20877	0.740522	0.130462	*PRDM10*	4	0.26557	0.566607	0.246718
HNRNPAB	4	0.21168	0.740522	0.130462	*SDF2*	4	0.28442	0.566607	0.246718
RAD1	4	0.25359	0.740522	0.130462	*LACTB*	2	0.29003	0.566607	0.246718
HNRNPDL	4	0.25909	0.740522	0.130462	*SLC35A2*	2	0.2933	0.566607	0.246718
WNT7A	4	0.27228	0.740522	0.130462	*PTTG1*	4	0.29767	0.566607	0.246718
TMEM134	4	0.27521	0.740522	0.130462	*EFNA2*	3	0.29847	0.566607	0.246718
CLDN16	4	0.27761	0.740522	0.130462	*TSBP1*	4	0.31519	0.566607	0.246718
AXDND1	4	0.29146	0.740522	0.130462	*FPR3*	3	0.34463	0.566607	0.246718
FAM83C	3	0.30136	0.740522	0.130462	*ALKBH8*	4	0.34836	0.566607	0.246718
LRBA	4	0.32335	0.740522	0.130462	*TRPC4AP*	4	0.36296	0.566607	0.246718

DVL1	4	0.34556	0.740522	0.130462	*C19orf57*	2	0.37629	0.566607	0.246718
CENPU	3	0.37644	0.740522	0.130462	*HSD17B4*	4	0.37873	0.566607	0.246718
ZBTB9	4	0.39517	0.740522	0.130462	*KIAA1143*	2	0.3789	0.566607	0.246718
DHX16	3	0.41874	0.740522	0.130462	*KLC3*	2	0.38362	0.566607	0.246718
TEDC1	3	0.42981	0.740522	0.130462	*AP3M2*	3	0.39819	0.566607	0.246718
PLSCR2	4	0.43545	0.740522	0.130462	*DUT*	4	0.41884	0.566607	0.246718
IFNL2	3	0.3791	0.743124	0.128939	*RPP30*	4	0.42345	0.566607	0.246718
BUB1B	3	0.79749	0.743124	0.128939	*RAD50*	3	0.44626	0.566607	0.246718
PEX11G	3	-0.63351	0.743219	0.128883	*CDC123*	3	0.54344	0.566607	0.246718
PLAUR	3	0.44832	0.743219	0.128883	*C9orf116*	2	0.55327	0.566607	0.246718
RANBP2	1	-0.73038	0.744373	0.12821	*TMEM169*	3	0.57314	0.566607	0.246718
SETD2	3	-0.6705	0.744373	0.12821	*NDUFB8*	2	0.58159	0.566607	0.246718
TRIP6	3	-0.64478	0.744373	0.12821	*CDC40*	3	0.6193	0.566607	0.246718
PJVK	3	-0.55973	0.744373	0.12821	*PARM1*	3	0.62034	0.566607	0.246718
BACH2	4	-0.55551	0.744373	0.12821	*BNIP1*	3	0.66928	0.566607	0.246718
INIP	3	-0.51391	0.744373	0.12821	*CEBPZ*	3	0.74503	0.566607	0.246718
PA2G4	3	-0.50517	0.744373	0.12821	*RBSN*	3	0.81297	0.566607	0.246718
LIN28A	4	-0.50085	0.744373	0.12821	*DRAP1*	4	0.13106	0.566607	0.246718
KCNC1	2	-0.49438	0.744373	0.12821	*MYO7B*	4	0.22421	0.566607	0.246718
MFAP3L	3	-0.47356	0.744373	0.12821	*SLC4A2*	4	0.25357	0.566607	0.246718
TMEM267	4	-0.42711	0.744373	0.12821	*ZNF584*	4	0.31931	0.566607	0.246718
ADAMTS13	3	-0.39077	0.744373	0.12821	*MLXIPL*	4	0.20354	0.567135	0.246314
OR13H1	4	-0.38871	0.744373	0.12821	*LHX9*	4	0.25268	0.567135	0.246314
ALOX15B	3	-0.38098	0.744373	0.12821	*PRRX2*	2	-0.3469	0.567353	0.246146
FBXO15	4	-0.36755	0.744373	0.12821	*IGFBP7*	3	-0.5511	0.567422	0.246094
RND2	4	-0.34246	0.744373	0.12821	*MCCC1*	3	-0.39173	0.567422	0.246094
SRPK3	4	-0.34154	0.744373	0.12821	*ZNF334*	4	0.29022	0.567422	0.246094
YIPF3	4	-0.31241	0.744373	0.12821	*TRMT112*	4	0.46561	0.567422	0.246094
ADAM8	3	-0.31221	0.744373	0.12821	*GPN1*	4	0.48416	0.567422	0.246094
FAM193A	4	-0.29818	0.744373	0.12821	*ICE1*	2	0.4845	0.567422	0.246094
LY6G6C	4	-0.27484	0.744373	0.12821	*MYRIP*	4	-0.1762	0.568198	0.245501
AOPEP	4	-0.27313	0.744373	0.12821	*MAPK12*	2	0.36315	0.568279	0.245439
EFNB1	4	-0.21469	0.744373	0.12821	*POPDC3*	2	0.48058	0.568279	0.245439
LAMB1	3	-0.15874	0.744373	0.12821	*PURB*	3	0.32255	0.569574	0.24445
RERGL	4	-0.11745	0.744373	0.12821	*RAD51*	3	0.80653	0.569574	0.24445
POU4F3	4	-0.11531	0.744373	0.12821	*TMEM140*	3	0.29848	0.569942	0.244169
NAB2	4	-0.07588	0.744373	0.12821	*APBA2*	1	-0.63373	0.57028	0.243912
MOSPD2	3	-0.066508	0.744373	0.12821	*STRAP*	4	0.063458	0.57028	0.243912
MARCHF7	3	-0.050264	0.744373	0.12821	*OCEL1*	3	0.43726	0.57028	0.243912
GPR155	4	-0.013626	0.744373	0.12821	*WDR76*	4	0.45856	0.57028	0.243912
SS18L1	4	0.069931	0.744373	0.12821	*EFCAB12*	3	0.58263	0.57028	0.243912
ADAMTSL1	4	0.076755	0.744373	0.12821	*CLEC4A*	3	-0.59591	0.571166	0.243238
KLHL32	4	0.093553	0.744373	0.12821	*GOLGA7B*	4	0.25502	0.571166	0.243238
MKLN1	4	0.096733	0.744373	0.12821	*ANKRD33B*	4	-0.40498	0.571281	0.24315
PNCK	4	0.13554	0.744373	0.12821	*RET*	4	0.21342	0.57137	0.243082
SPECC1	4	0.13761	0.744373	0.12821	*SUGCT*	4	0.18427	0.571374	0.243079
ZSWIM4	4	0.17407	0.744373	0.12821	*ANKRD36*	2	0.32051	0.571374	0.243079
INSL6	4	0.17604	0.744373	0.12821	*CPSF4*	4	0.38888	0.571374	0.243079
SFR1	3	0.20594	0.744373	0.12821	*RYR3*	2	-0.80561	0.571464	0.243011
EME1	4	0.21596	0.744373	0.12821	*CYB561*	4	-0.46917	0.571578	0.242924
OC10012908	4	0.22983	0.744373	0.12821	*MTHFD2L*	2	-0.47854	0.571659	0.242863
SCP2	4	0.23268	0.744373	0.12821	*KIF3B*	4	0.15129	0.571659	0.242863
CDH11	4	0.24277	0.744373	0.12821	*TLN1*	4	0.371	0.571659	0.242863
HAUS6	4	0.24399	0.744373	0.12821	*DENR*	4	0.52095	0.571659	0.242863
GFY	4	0.25099	0.744373	0.12821	*JAG2*	3	-0.13655	0.572162	0.242481
LIN9	4	0.25344	0.744373	0.12821	*MRPS35*	3	0.25733	0.572602	0.242147
PRMT3	4	0.25549	0.744373	0.12821	*RFPL2*	4	0.16634	0.573242	0.241662
DDX42	4	0.25575	0.744373	0.12821	*KCNIP1*	3	-0.021748	0.573443	0.24151

HEPHL1	4	0.2597	0.744373	0.12821	*SQSTM1*	3	0.01562	0.57372	0.2413
CARNS1	4	0.26204	0.744373	0.12821	*CCNA2*	3	0.18204	0.57372	0.2413
OR10A6	4	0.27443	0.744373	0.12821	*NKD1*	3	-0.49597	0.574485	0.240721
ITIH3	4	0.29368	0.744373	0.12821	*PTER*	4	-0.4434	0.574485	0.240721
LBX1	4	0.29661	0.744373	0.12821	*BATF*	4	-0.27424	0.574485	0.240721
RETREG2	4	0.30387	0.744373	0.12821	*LRRK1*	4	-0.24393	0.574485	0.240721
CHIC2	4	0.30416	0.744373	0.12821	*PRKD2*	4	0.22427	0.574485	0.240721
TMEM126A	4	0.30461	0.744373	0.12821	*MAGEA4*	4	0.24215	0.574485	0.240721
TICRR	4	0.30773	0.744373	0.12821	*COPB1*	4	0.24588	0.574485	0.240721
CRABP1	4	0.31891	0.744373	0.12821	*REG1A*	4	0.27826	0.574485	0.240721
PLK5	4	0.32101	0.744373	0.12821	*HEATR5B*	4	0.28926	0.574485	0.240721
MAIP1	4	0.33401	0.744373	0.12821	*RPL7L1*	3	0.50459	0.574485	0.240721
ZFR	4	0.35885	0.744373	0.12821	*ERLIN2*	3	-0.59293	0.574548	0.240674
LSM14A	4	0.35891	0.744373	0.12821	*TNFRSF10D*	3	-0.57176	0.574548	0.240674
NFYC	3	0.36483	0.744373	0.12821	*NPTX1*	3	0.14593	0.574548	0.240674
SELENOI	4	0.36898	0.744373	0.12821	*TBC1D10B*	4	0.27127	0.574548	0.240674
POC5	4	0.36972	0.744373	0.12821	*TIAL1*	4	0.28433	0.574548	0.240674
ESD	4	0.37264	0.744373	0.12821	*NACA2*	3	0.35219	0.574548	0.240674
SMPD1	4	0.37571	0.744373	0.12821	*SHOC2*	4	0.36983	0.574548	0.240674
FCHSD1	4	0.3822	0.744373	0.12821	*TRIM56*	3	0.59855	0.575046	0.240298
CLECL1	4	0.3839	0.744373	0.12821	*NECTIN4*	4	-0.15224	0.57645	0.239238
ZNF329	4	0.3891	0.744373	0.12821	*DHH*	3	0.37195	0.576685	0.239061
RNF223	4	0.39237	0.744373	0.12821	*SRSF12*	4	-0.29692	0.57689	0.238907
PSMA5	4	0.39977	0.744373	0.12821	*CAPN8*	4	-0.11058	0.57689	0.238907
FAM168A	4	0.4038	0.744373	0.12821	*ATR*	3	-0.062006	0.57689	0.238907
H2AJ	4	0.40909	0.744373	0.12821	*EXOC3L2*	4	-0.035951	0.57689	0.238907
HTR6	4	0.40919	0.744373	0.12821	*SSBP4*	4	0.086521	0.57689	0.238907
CLEC17A	4	0.42385	0.744373	0.12821	*CD46*	2	0.29573	0.57689	0.238907
FCHSD2	3	0.42723	0.744373	0.12821	*TIGIT*	4	0.33395	0.57689	0.238907
OS9	4	0.43002	0.744373	0.12821	*ILVBL*	3	0.38503	0.57689	0.238907
POLR3F	4	0.49251	0.744373	0.12821	*PPM1E*	4	-0.54287	0.577062	0.238778
PRKAR2A	2	0.50269	0.744373	0.12821	*MIPEP*	4	0.36935	0.577062	0.238778
POPDC3	2	0.53434	0.744373	0.12821	*RB1CC1*	4	-0.46941	0.577237	0.238645
FAM219B	3	0.55332	0.744373	0.12821	*C3orf79*	4	-0.33389	0.577237	0.238645
RBBP8	4	0.5664	0.744373	0.12821	*ULBP3*	4	-0.32473	0.577237	0.238645
MTRNR2L2	1	0.609	0.744373	0.12821	*NCR3LG1*	4	-0.28074	0.577237	0.238645
GHRL	3	0.64992	0.744373	0.12821	*ZNF747*	4	-0.16918	0.577237	0.238645
DPF1	2	0.65792	0.744373	0.12821	*SGPP1*	3	-0.16035	0.577237	0.238645
CHTF18	2	0.80056	0.744373	0.12821	*JSRP1*	3	-0.099578	0.577237	0.238645
ZCCHC12	3	0.22622	0.745269	0.127687	*COL22A1*	3	0.31049	0.577237	0.238645
LACC1	4	-0.306	0.745372	0.127627	*LEFTY2*	3	0.37927	0.577237	0.238645
SLC24A5	4	-0.29342	0.745372	0.127627	*TNIP1*	4	0.40068	0.577237	0.238645
PRSS37	4	-0.3011	0.745516	0.127543	*LGALS7B*	1	0.79031	0.577237	0.238645
UFC1	4	-0.019044	0.745754	0.127405	*MAP1LC3C*	3	-0.90113	0.577333	0.238573
THY1	4	0.15109	0.745754	0.127405	*CELSR2*	3	-0.6879	0.577333	0.238573
MSRB2	4	-0.22244	0.746069	0.127221	*PIP4K2C*	3	-0.58346	0.577333	0.238573
VWA2	4	-0.42476	0.746206	0.127141	*RPGR*	3	-0.56742	0.577333	0.238573
MAPK1IP1L	3	-0.30855	0.746206	0.127141	*STMND1*	4	-0.31093	0.577333	0.238573
CDIP1	2	-0.23693	0.746206	0.127141	*APCDD1*	4	-0.16691	0.577333	0.238573
FNDC3B	4	-0.037747	0.746206	0.127141	*MAP3K11*	4	0.095395	0.577333	0.238573
PCNX2	4	-0.021161	0.746206	0.127141	*INTS12*	4	0.15116	0.577333	0.238573
PSMA2	4	0.28344	0.746206	0.127141	*SNRPA*	3	0.20748	0.577333	0.238573
MYL5	2	0.3551	0.746206	0.127141	*MIIP*	4	0.21645	0.577333	0.238573
DPH1	3	0.50081	0.746206	0.127141	*COQ6*	4	0.25202	0.577333	0.238573
ARHGEF19	3	-0.58944	0.746984	0.126689	*NCK2*	3	0.27314	0.577333	0.238573
P2RX7	4	-0.33392	0.746984	0.126689	*CYFIP2*	4	0.29544	0.577333	0.238573
SEMA5A	4	-0.32978	0.746984	0.126689	*TIPARP*	4	0.34443	0.577333	0.238573
ADNP	4	-0.13999	0.746984	0.126689	*ATP2C1*	3	0.38161	0.577333	0.238573

SLC7A4	3	-0.65223	0.747021	0.126667	*CTAGE6*	1	0.59529	0.577333	0.238573
LGALS8	3	-0.49117	0.747021	0.126667	*TMBIM6*	3	-0.52198	0.577428	0.238502
HELZ	3	-0.42241	0.747021	0.126667	*CHST12*	4	0.21548	0.577428	0.238502
SELENOF	3	-0.3787	0.747021	0.126667	*LGR4*	4	0.21967	0.577428	0.238502
AMIGO1	3	-0.32222	0.747021	0.126667	*MECOM*	4	0.43616	0.577475	0.238467
TOB1	4	-0.31442	0.747021	0.126667	*NUP58*	4	0.47288	0.577475	0.238467
MANEA	4	-0.12334	0.747021	0.126667	*ZFYVE16*	2	-0.49991	0.580195	0.236426
CCDC198	4	-0.088126	0.747021	0.126667	*TCTA*	4	-0.40354	0.580195	0.236426
CSF2RB	4	0.011621	0.747021	0.126667	*SH3GLB1*	4	-0.36277	0.580195	0.236426
TP53INP1	4	0.12085	0.747021	0.126667	*PCDH17*	4	-0.35919	0.580195	0.236426
PSG6	4	0.19365	0.747021	0.126667	*SH3D19*	2	-0.28358	0.580195	0.236426
RAB8A	4	0.1972	0.747021	0.126667	*RTL6*	4	-0.21027	0.580195	0.236426
KIAA1143	2	0.30258	0.747021	0.126667	*TMEM50A*	4	-0.042301	0.580195	0.236426
F2	2	0.43188	0.747021	0.126667	*ZCCHC3*	4	0.14659	0.580195	0.236426
DCAF10	3	0.52219	0.747021	0.126667	*DNAH11*	4	0.18739	0.580195	0.236426
LAMTOR1	4	0.52872	0.747021	0.126667	*PES1*	4	0.30696	0.580195	0.236426
PTPN20	3	0.57057	0.747021	0.126667	*POR*	4	0.31734	0.580195	0.236426
PRKRIP1	1	0.64933	0.747021	0.126667	*CEP57*	4	0.32371	0.580195	0.236426
MYLK2	4	0.058255	0.747575	0.126345	*HOXA3*	2	0.32881	0.580195	0.236426
DDX59	3	-0.76049	0.747699	0.126273	*DDX51*	4	0.34611	0.580195	0.236426
LILRA6	3	0.26117	0.747699	0.126273	*ISCU*	4	0.3718	0.580195	0.236426
CAB39	3	0.080965	0.747726	0.126258	*HHATL*	3	-0.32927	0.581954	0.235111
ACAP2	4	0.28223	0.747726	0.126258	*ZDBF2*	3	0.48997	0.582182	0.234941
EFCAB14	2	-0.48562	0.747873	0.126172	*COPS4*	3	0.38679	0.582592	0.234635
EMX2	2	-0.4759	0.747873	0.126172	*FGD1*	4	-0.28511	0.582784	0.234493
CEACAM20	4	0.21484	0.748082	0.126051	*ZNHIT1*	3	0.65773	0.582853	0.234441
GAS1	4	0.32178	0.748082	0.126051	*FBXL17*	3	-0.56203	0.582856	0.234438
DYNLL1	2	0.30312	0.748355	0.125892	*DNAJA2*	3	-0.074771	0.582856	0.234438
NPY	3	-0.60734	0.748919	0.125565	*BMT2*	3	0.3628	0.582856	0.234438
C7orf26	3	0.34741	0.749386	0.125294	*DCUN1D5*	4	0.24986	0.58295	0.234369
SYNRG	3	0.097537	0.749574	0.125185	*NUDT13*	2	0.42816	0.583117	0.234245
TMEM131L	3	-0.41879	0.750561	0.124614	*PAXBP1*	4	0.42089	0.583416	0.234022
CTNS	4	-0.10201	0.750737	0.124512	*ARFGAP1*	3	-0.1733	0.58363	0.233863
SOCS7	4	-0.34383	0.751371	0.124146	*CD151*	3	0.30292	0.58363	0.233863
PCDH17	4	-0.14165	0.751371	0.124146	*TUBAL3*	3	0.41698	0.58363	0.233863
CHTF8	4	0.28669	0.751371	0.124146	*CNP*	3	0.43673	0.58363	0.233863
CRAT	4	0.32329	0.752124	0.12371	*SP2*	3	0.5788	0.58363	0.233863
KLHL30	2	0.41613	0.752124	0.12371	*SRGAP1*	4	-0.35228	0.58493	0.232896
TIMM17B	2	0.46157	0.752124	0.12371	*SENP6*	2	0.5603	0.585252	0.232657
UNG	3	-0.53306	0.752216	0.123657	*RAB11FIP4*	2	0.49189	0.58701	0.231354
NEDD9	3	-0.53252	0.752216	0.123657	*KCNB2*	4	0.21994	0.587729	0.230823
TRAP1	4	-0.3376	0.752216	0.123657	*SETD2*	3	-0.58133	0.587798	0.230772
SERPINB2	3	-0.33736	0.752216	0.123657	*KLLN*	2	-0.21265	0.587798	0.230772
BTBD6	4	-0.30343	0.752216	0.123657	*C5orf24*	3	0.14295	0.587798	0.230772
NEFH	4	-0.2127	0.752216	0.123657	*CSK*	4	0.18415	0.587798	0.230772
MAGEF1	3	-0.13441	0.752216	0.123657	*VNN2*	4	0.20245	0.587798	0.230772
ALDH7A1	3	-0.10364	0.752216	0.123657	*CELSR3*	3	0.23824	0.587798	0.230772
FLRT2	3	-0.043916	0.752216	0.123657	*B2M*	4	0.31202	0.587798	0.230772
FETUB	4	0.084032	0.752216	0.123657	*TNFRSF1A*	4	0.31223	0.587798	0.230772
ZNF268	4	0.14964	0.752216	0.123657	*EIF2B2*	4	0.37243	0.587798	0.230772
ERG28	4	0.32536	0.752216	0.123657	*CMTM6*	3	0.44186	0.587798	0.230772
MAJIN	4	0.39029	0.752216	0.123657	*SPOPL*	3	0.45462	0.587798	0.230772
UNC45B	3	0.44792	0.752216	0.123657	*CDK5*	3	0.54585	0.587798	0.230772
TPT1	3	0.47323	0.752216	0.123657	*VHL*	4	0.54635	0.587798	0.230772
RPS17	2	0.50249	0.752216	0.123657	*ASB16*	3	0.50938	0.588005	0.230619
CD14	3	0.53359	0.752216	0.123657	*HSPD1*	1	0.55044	0.588005	0.230619
CASP7	1	-0.58546	0.753181	0.123101	*CCNC*	3	-0.67182	0.588115	0.230538
DET1	2	0.41838	0.753377	0.122987	*S100A8*	3	0.36313	0.588115	0.230538

NCK2	3	0.32961	0.753698	0.122802	*TTF2*	3	0.3244	0.588433	0.230303
ACY3	3	0.45375	0.753698	0.122802	*DLX4*	3	-0.67871	0.588715	0.230095
TMC7	4	-0.62477	0.754237	0.122492	*INSL3*	1	-0.64043	0.588715	0.230095
PCDHB2	4	-0.41254	0.754237	0.122492	*LARP4*	4	-0.40678	0.588715	0.230095
TUBA4A	4	-0.37281	0.754237	0.122492	*C5orf66*	4	-0.39956	0.588715	0.230095
FAM167A	4	-0.30748	0.754237	0.122492	*HEXD*	4	-0.23688	0.588715	0.230095
LEMD3	4	-0.28786	0.754237	0.122492	*OSGEP*	3	0.26705	0.588715	0.230095
BMF	4	-0.21202	0.754237	0.122492	*TANK*	4	0.27763	0.588715	0.230095
HAT1	4	-0.053343	0.754237	0.122492	*RIF1*	3	0.44664	0.588715	0.230095
DHRS13	4	0.05327	0.754237	0.122492	*MILR1*	4	0.34997	0.588884	0.22997
FMO4	4	0.20969	0.754237	0.122492	*DROSHA*	3	0.65782	0.588884	0.22997
POM121L2	4	0.34583	0.754237	0.122492	*LGALS1*	4	0.22352	0.589296	0.229666
ERCC6L2	4	0.36379	0.754237	0.122492	*PLAAT1*	2	0.28127	0.589338	0.229635
DIS3L	3	0.47004	0.754237	0.122492	*POLR3A*	2	-0.97302	0.591117	0.228327
EFCAB6	4	0.48557	0.754237	0.122492	*IKZF2*	2	-0.6148	0.591117	0.228327
UHRF1BP1L	3	-0.23489	0.754604	0.122281	*TWIST1*	3	-0.60494	0.591117	0.228327
KIF18B	4	0.47478	0.754604	0.122281	*TPSAB1*	2	-0.5494	0.591117	0.228327
CENPE	4	0.36789	0.755072	0.122011	*MAPK3*	3	-0.36816	0.591117	0.228327
PHF1	4	-0.15433	0.755164	0.121959	*RAB10*	4	-0.36794	0.591117	0.228327
UBXN8	3	0.40881	0.755164	0.121959	*WBP1*	1	-0.36551	0.591117	0.228327
SPA17	4	-0.04685	0.756048	0.12145	*ZNF629*	4	-0.34335	0.591117	0.228327
OC10798661	2	-0.39211	0.756672	0.121092	*TRIM65*	4	-0.34193	0.591117	0.228327
FLOT1	3	-0.16507	0.756672	0.121092	*ELFN2*	4	-0.31696	0.591117	0.228327
ENPP5	2	0.038753	0.756672	0.121092	*HDAC5*	4	-0.28598	0.591117	0.228327
RAB11FIP3	4	0.07172	0.756672	0.121092	*GSTM2*	3	-0.23985	0.591117	0.228327
CRISPLD1	4	0.12638	0.756672	0.121092	*SUGT1*	2	-0.094426	0.591117	0.228327
CYP4F8	4	0.22739	0.756672	0.121092	*MEST*	4	-0.006828	0.591117	0.228327
CHCHD5	4	0.30974	0.756672	0.121092	*CLDN19*	4	0.002535	0.591117	0.228327
CXCR4	4	0.33985	0.756672	0.121092	*TIA1*	2	0.077663	0.591117	0.228327
SCN4A	4	0.29796	0.757094	0.12085	*ERGIC2*	3	0.13189	0.591117	0.228327
PBRM1	4	0.30195	0.757094	0.12085	*FAP*	4	0.14671	0.591117	0.228327
SURF1	3	-0.63985	0.758865	0.119836	*VMAC*	2	0.15135	0.591117	0.228327
MBD1	4	-0.24858	0.758865	0.119836	*ATOX1*	3	0.1948	0.591117	0.228327
PAH	1	-0.70712	0.759471	0.119489	*TEX2*	4	0.21133	0.591117	0.228327
GMFB	4	-0.375	0.759471	0.119489	*MYLK4*	4	0.2132	0.591117	0.228327
STK32C	4	-0.2986	0.759471	0.119489	*RAB24*	4	0.21607	0.591117	0.228327
NR2F2	3	-0.21753	0.759471	0.119489	*ARHGDIG*	4	0.21748	0.591117	0.228327
KLHL25	4	-0.21225	0.759471	0.119489	*WHRN*	2	0.22264	0.591117	0.228327
REEP6	3	-0.11741	0.759471	0.119489	*MRPS31*	4	0.23669	0.591117	0.228327
ACER2	4	-0.063604	0.759471	0.119489	*WDR24*	4	0.2422	0.591117	0.228327
BCAT2	4	0.20679	0.759471	0.119489	*TESK1*	4	0.24643	0.591117	0.228327
RAG2	4	0.2223	0.759471	0.119489	*FAM167B*	3	0.2486	0.591117	0.228327
RPUSD2	3	0.24142	0.759471	0.119489	*ZNF101*	4	0.25676	0.591117	0.228327
PRKCH	4	0.27692	0.759471	0.119489	*STX18*	4	0.27078	0.591117	0.228327
MMP23B	2	0.28332	0.759471	0.119489	*ILK*	3	0.27874	0.591117	0.228327
R3HDM4	4	0.28547	0.759471	0.119489	*TEK*	2	0.29736	0.591117	0.228327
PDGFD	4	0.31093	0.759471	0.119489	*TOLLIP*	2	0.29974	0.591117	0.228327
ZBTB11	4	0.32094	0.759471	0.119489	*PPP4R2*	3	0.31292	0.591117	0.228327
PSMD12	4	0.38797	0.759471	0.119489	*RARA*	4	0.32014	0.591117	0.228327
EMC4	4	0.40075	0.759471	0.119489	*RGS12*	4	0.32732	0.591117	0.228327
MTX1	3	0.4061	0.759471	0.119489	*WASHC5*	2	0.38281	0.591117	0.228327
HP	3	0.51279	0.759471	0.119489	*TIMM22*	4	0.39772	0.591117	0.228327
CNTN4	3	-0.50646	0.760371	0.118975	*MLXIP*	4	0.398	0.591117	0.228327
C16orf91	2	-0.47518	0.760371	0.118975	*COG7*	4	0.42448	0.591117	0.228327
WDR60	3	-0.44883	0.760371	0.118975	*TNK1*	3	0.42591	0.591117	0.228327
PROM2	4	-0.026391	0.760371	0.118975	*NOB1*	4	0.44892	0.591117	0.228327
TAF1B	4	0.17768	0.760371	0.118975	*CHST8*	3	0.46714	0.591117	0.228327
CENPX	4	0.29288	0.760371	0.118975	*FOXE1*	3	0.47385	0.591117	0.228327

TMEM161A	3	0.40934	0.760371	0.118975	*CNDP2*	3	0.48048	0.591117	0.228327
DDX1	3	0.51611	0.760371	0.118975	*KDM4B*	3	0.49031	0.591117	0.228327
C17orf50	3	-0.65825	0.76109	0.118564	*AHCY*	3	0.64051	0.591117	0.228327
RPRD1B	4	-0.47671	0.76109	0.118564	*TMEFF1*	1	0.71851	0.591117	0.228327
ZNF630	4	-0.42137	0.76109	0.118564	*PTCD2*	4	0.191	0.591368	0.228142
CAMSAP2	3	-0.40431	0.76109	0.118564	*BPTF*	4	0.056161	0.591929	0.22773
SERPINA3	3	-0.11943	0.76109	0.118564	*CTBP2*	4	0.39462	0.591929	0.22773
ZNF555	4	0.1659	0.76109	0.118564	*DHRS11*	4	0.38699	0.592157	0.227563
ZNF35	4	0.25124	0.76109	0.118564	*FCHO1*	3	-0.53059	0.592214	0.227521
TFPT	4	0.28016	0.76109	0.118564	*MXD4*	3	-0.46743	0.592214	0.227521
MED13	4	-0.47749	0.761325	0.11843	*CHDH*	4	0.18971	0.592896	0.227022
NKD1	3	-0.1934	0.7615	0.11833	*PPP1R13B*	4	0.33933	0.592896	0.227022
RUNX1	4	0.15127	0.7615	0.11833	*PXMP2*	3	0.45688	0.592896	0.227022
CDK14	4	0.18194	0.7615	0.11833	*ZC3H11A*	3	-0.45854	0.593049	0.226909
ZNF684	4	0.25891	0.7615	0.11833	*SLC22A2*	4	-0.3923	0.593049	0.226909
FRMD4B	4	0.28661	0.7615	0.11833	*TBC1D3E*	2	-0.29216	0.593049	0.226909
CDKL4	3	0.34941	0.7615	0.11833	*KLB*	3	-0.18468	0.593049	0.226909
REPIN1	3	0.44087	0.761948	0.118075	*MYO9A*	4	0.27385	0.593049	0.226909
SCGB1C1	1	0.50703	0.761948	0.118075	*CHMP4C*	2	-0.50133	0.593171	0.22682
TBC1D13	3	0.54485	0.761948	0.118075	*LRRIQ3*	3	-0.57018	0.594351	0.225957
OVCA2	2	-0.62389	0.762846	0.117563	*TEP1*	3	-0.45046	0.594351	0.225957
TRAF2	4	0.39136	0.763173	0.117377	*NAP1L2*	3	-0.34145	0.594351	0.225957
IARS2	3	-0.6356	0.763334	0.117285	*KRT83*	3	-0.31842	0.594351	0.225957
CNIH1	4	-0.023415	0.763334	0.117285	*RBM28*	3	-0.16559	0.594351	0.225957
ZNF646	4	0.058695	0.763334	0.117285	*MFSD1*	3	0.31722	0.594351	0.225957
UBE2B	4	0.16271	0.763334	0.117285	*ATP5PF*	3	0.47007	0.594674	0.225721
PTTG1	4	0.27509	0.763334	0.117285	*CABP4*	3	0.49359	0.594674	0.225721
CLEC2D	4	0.29723	0.763334	0.117285	*PRDX1*	3	-0.48329	0.594896	0.225559
NCKAP5	3	0.33484	0.763334	0.117285	*SLC2A4*	3	-0.2345	0.594896	0.225559
PABPC4	3	0.35067	0.763334	0.117285	*CHGA*	2	0.52719	0.594896	0.225559
CPSF4	4	0.40133	0.763334	0.117285	*FBXO43*	4	0.10653	0.5949	0.225556
RFNG	4	-0.030219	0.76402	0.116896	*NPEPPS*	4	-0.28499	0.595565	0.225071
ANKRD27	4	0.23469	0.764843	0.116428	*DNAJC5*	4	-0.19367	0.596952	0.22406
KCNQ2	4	-0.35799	0.765829	0.115868	*STX2*	4	0.081963	0.596952	0.22406
LAP3	4	-0.31018	0.765829	0.115868	*PPAT*	4	0.34211	0.596966	0.22405
ZNF131	4	-0.082924	0.765829	0.115868	*RASGRP1*	3	0.36258	0.596966	0.22405
CHCHD2	1	0.57216	0.765829	0.115868	*NEMP1*	3	0.4966	0.596966	0.22405
MET	3	-0.45698	0.76709	0.115154	*CLTC*	4	0.71223	0.596966	0.22405
WFS1	3	-0.21715	0.76709	0.115154	*NBPF11*	2	-0.5914	0.597664	0.223543
FBLN1	4	0.003316	0.76709	0.115154	*LGALS12*	4	-0.14698	0.597864	0.223398
PPP6R3	2	0.48786	0.767621	0.114853	*ZNF221*	3	-0.3261	0.597925	0.223354
TAF1	3	1.1682	0.767621	0.114853	*RBM4B*	4	0.30515	0.598378	0.223024
ZMYM6	3	0.32201	0.767805	0.114749	*WDR38*	4	-0.86425	0.599155	0.222461
PCDHGA2	3	-0.44635	0.768024	0.114625	*PFAS*	3	0.049574	0.599558	0.222169
CLIP1	4	-0.32722	0.768024	0.114625	*ATP5MG*	2	0.21063	0.599558	0.222169
UQCRFS1	2	0.71277	0.770549	0.1132	*TADA2B*	3	0.34347	0.599558	0.222169
SLC35G1	4	-0.53544	0.770884	0.113011	*RPS23*	3	0.40798	0.599558	0.222169
GFRA3	4	-0.44065	0.770884	0.113011	*ADAM18*	2	0.30097	0.59969	0.222073
EIF5B	4	-0.42305	0.770884	0.113011	*FFAR4*	3	0.37609	0.59969	0.222073
PIGN	4	-0.4073	0.770884	0.113011	*SUV39H1*	3	-0.35505	0.600172	0.221724
ZMIZ2	3	-0.38903	0.770884	0.113011	*RAB5A*	3	-0.08452	0.600172	0.221724
FAM78B	4	-0.30618	0.770884	0.113011	*RPLP2*	3	0.42428	0.600172	0.221724
C1GALT1	3	-0.070582	0.770884	0.113011	*SLU7*	3	0.70558	0.600172	0.221724
KLHDC9	3	-0.00205	0.770884	0.113011	*TIMM23*	3	-0.77961	0.60094	0.221169
ARPC3	4	0.22232	0.770884	0.113011	*ELOVL4*	3	-0.37292	0.60094	0.221169
NME4	4	0.2743	0.770884	0.113011	*MAP2K7*	3	0.3349	0.60094	0.221169
RWDD2B	4	0.32695	0.770884	0.113011	*NOVA1*	2	0.40063	0.60094	0.221169
SMAP2	3	0.43276	0.770884	0.113011	*KDM2A*	4	-0.40012	0.601008	0.22112

RUVBL2	4	0.56326	0.770884	0.113011	*SH2D1B*	4	-0.20694	0.601008	0.22112
POLR2G	3	0.9448	0.770884	0.113011	*RRAD*	3	0.31878	0.601008	0.22112
LSM14B	4	0.11688	0.771009	0.112941	*GNB1L*	3	0.56628	0.601008	0.22112
LONP1	4	-0.23224	0.771991	0.112388	*DGKG*	3	0.67726	0.601008	0.22112
CYP27C1	4	-0.1659	0.771991	0.112388	*CYREN*	2	-0.31281	0.601102	0.221052
RASEF	4	-0.036536	0.771991	0.112388	*RBM41*	3	-0.76295	0.601276	0.220926
CEMIP	3	0.054884	0.771991	0.112388	*AXIN1*	3	-0.54052	0.601392	0.220842
RPA3	4	0.13951	0.771991	0.112388	*UBE2E2*	3	-0.6239	0.601457	0.220795
PACSIN2	4	0.25209	0.771991	0.112388	*UPK2*	4	0.26935	0.601457	0.220795
FOXB1	4	0.25444	0.771991	0.112388	*AVPI1*	2	0.36346	0.601457	0.220795
NFYB	3	0.44771	0.771991	0.112388	*LARS1*	2	0.38652	0.601457	0.220795
ALDH3A1	4	-0.27748	0.77217	0.112287	*RGS6*	3	-0.38723	0.601985	0.220414
PLEKHG7	4	0.32623	0.77217	0.112287	*FOXB2*	4	0.23166	0.602192	0.220265
MLH1	3	-0.067726	0.77234	0.112192	*SLC28A1*	4	0.23399	0.602411	0.220107
GNG13	4	-0.24022	0.773256	0.111677	*ENO3*	3	-0.56193	0.602711	0.219891
RBM41	3	-0.22759	0.773256	0.111677	*DDX58*	3	0.17542	0.602711	0.219891
EEF2	4	-0.14151	0.773256	0.111677	*SERAC1*	4	-0.42949	0.602776	0.219844
ART5	4	0.25082	0.773256	0.111677	*KLKB1*	4	0.24916	0.602776	0.219844
ECT2L	3	0.33883	0.773527	0.111525	*SVBP*	4	0.25113	0.602776	0.219844
TBC1D1	4	0.35202	0.77381	0.111366	*MRPL58*	4	0.38142	0.602776	0.219844
HMGB3	4	0.12409	0.77428	0.111102	*FADS6*	4	0.40455	0.602776	0.219844
ATP5MD	2	0.30369	0.77428	0.111102	*PLAGL1*	1	-0.86621	0.602857	0.219786
ABLIM3	4	0.30896	0.77428	0.111102	*SPARC*	3	0.054826	0.603344	0.219435
TFDP2	3	0.3689	0.77428	0.111102	*ACHE*	3	0.10278	0.603344	0.219435
LCA5	2	0.37887	0.77428	0.111102	*TTC14*	4	0.1945	0.603344	0.219435
GOLGA7B	4	0.38888	0.77428	0.111102	*MRPL50*	4	0.27087	0.603344	0.219435
PTDSS1	2	0.52328	0.77428	0.111102	*PEX6*	3	0.38006	0.603344	0.219435
ERAP2	2	-0.35725	0.775067	0.110661	*CMTM3*	3	0.41368	0.603344	0.219435
BTN3A3	4	-0.32907	0.775067	0.110661	*BORCS6*	3	0.45194	0.603344	0.219435
NBR1	4	0.34853	0.775067	0.110661	*LRRC19*	3	-0.002494	0.603528	0.219302
SREBF1	4	0.3742	0.775067	0.110661	*NECTIN3*	3	0.31584	0.603528	0.219302
RPP25L	4	0.48813	0.775067	0.110661	*PHLDB2*	3	0.40139	0.60386	0.219064
SPRYD3	3	0.48954	0.775067	0.110661	*NOL9*	3	0.6055	0.60386	0.219064
U2AF2	4	0.53388	0.775067	0.110661	*MPHOSPH9*	1	-0.84104	0.604156	0.218851
TEX22	3	-0.35946	0.775775	0.110264	*F2*	2	-0.67556	0.604783	0.218401
IGSF1	4	-0.26112	0.775775	0.110264	*RAP1GAP*	4	-0.41238	0.604783	0.218401
PPP1CB	3	-0.49522	0.775882	0.110205	*TOX4*	4	-0.37551	0.604783	0.218401
ZNF726	4	-0.23737	0.775882	0.110205	*ELOF1*	4	-0.082721	0.604783	0.218401
IMPA2	3	0.18531	0.776003	0.110137	*AMACR*	2	0.38218	0.605531	0.217864
NFKBIE	1	0.62316	0.776418	0.109904	*SPRED1*	4	0.12546	0.605792	0.217676
SDR39U1	3	0.021703	0.776698	0.109748	*USP33*	4	0.17767	0.605792	0.217676
APLF	4	-0.52552	0.776864	0.109655	*SPI1*	4	0.26768	0.605792	0.217676
OC10013439	4	-0.13949	0.776864	0.109655	*TADA2A*	4	0.40882	0.605985	0.217538
LRRC29	4	0.048186	0.776864	0.109655	*ANKMY2*	1	-0.41034	0.606019	0.217514
VPS16	3	0.31388	0.776864	0.109655	*RAP1GAP2*	4	-0.28188	0.60628	0.217327
NFATC1	4	-0.05988	0.777614	0.109236	*GAS7*	4	-0.24734	0.60628	0.217327
PDPR	4	0.17887	0.777614	0.109236	*HAL*	3	0.30514	0.60628	0.217327
C5orf34	2	-0.47495	0.77777	0.109149	*TRAF3*	3	0.64086	0.60628	0.217327
TNRC6A	3	-0.3841	0.77777	0.109149	*ASNSD1*	4	-0.18122	0.606359	0.21727
LIFR	3	-0.27013	0.77777	0.109149	*EMILIN1*	4	-0.39626	0.606371	0.217262
NIF3L1	4	0.27462	0.77777	0.109149	*NMNAT2*	4	-0.30135	0.606371	0.217262
HIPK3	3	0.28398	0.77777	0.109149	*ALB*	4	0.11311	0.606371	0.217262
CHST8	3	0.48347	0.77777	0.109149	*NAGK*	4	-0.49235	0.606833	0.216931
STAT5A	3	-0.64826	0.778343	0.108829	*EVA1A*	3	-0.24895	0.606833	0.216931
IPCEF1	3	-0.61601	0.778343	0.108829	*GJB1*	4	-0.080533	0.606833	0.216931
IFT81	3	-0.51516	0.778343	0.108829	*ZDHHC14*	4	-0.041757	0.606833	0.216931
RCN2	3	-0.16646	0.778343	0.108829	*HNRNPH1*	4	0.029766	0.606833	0.216931
ZNF830	3	-0.097846	0.778343	0.108829	*NEU3*	4	0.21023	0.606833	0.216931

POLD4	4	0.24294	0.778343	0.108829	*CYP2B6*	4	0.28477	0.606833	0.216931
SLC15A3	4	-0.47244	0.778728	0.108614	*FAM117B*	4	0.31953	0.606833	0.216931
ZNF324B	3	0.30994	0.778728	0.108614	*RFX7*	4	0.37662	0.606833	0.216931
REXO5	4	-0.32502	0.77906	0.108429	*TAF1B*	4	0.44053	0.606833	0.216931
BCOR	3	-0.63075	0.779229	0.108335	*LRRC8B*	3	-0.46988	0.607787	0.216248
MT2A	2	-0.60743	0.779229	0.108335	*CARD11*	4	-0.17367	0.607787	0.216248
STAB2	4	-0.48419	0.779229	0.108335	*ARNTL2*	2	-0.003263	0.608056	0.216057
LDLRAD1	4	-0.44209	0.779229	0.108335	*IQSEC3*	2	0.23258	0.608056	0.216057
DNAH17	3	-0.39681	0.779229	0.108335	*MZT2A*	2	0.41583	0.608056	0.216057
ZNF142	4	-0.38396	0.779229	0.108335	*ALDH1B1*	3	-0.35608	0.608344	0.21585
MESP1	3	-0.30981	0.779229	0.108335	*RAET1E*	3	-0.31825	0.608344	0.21585
ZNF717	3	-0.27189	0.779229	0.108335	*FAHD1*	3	-0.30035	0.608344	0.21585
IL13RA1	4	-0.071179	0.779229	0.108335	*FEN1*	3	0.32292	0.608344	0.21585
EARS2	4	0.010786	0.779229	0.108335	*RCC1*	2	0.48047	0.608344	0.21585
SCAF8	3	0.10953	0.779229	0.108335	*LYAR*	2	-0.40548	0.608467	0.215763
RCAN1	4	0.22718	0.779229	0.108335	*ABCA12*	4	-0.44833	0.609045	0.215351
LRP3	2	0.27401	0.779229	0.108335	*RPA2*	3	0.2389	0.609045	0.215351
ABHD14A	2	0.29493	0.779229	0.108335	*NEFH*	4	0.26729	0.609045	0.215351
FAT3	2	0.33622	0.779229	0.108335	*ALDH1A2*	3	0.30258	0.609045	0.215351
SLC11A1	4	0.38336	0.779229	0.108335	*ANKLE1*	3	0.48856	0.609045	0.215351
RAB24	4	0.41037	0.779229	0.108335	*SLC7A7*	2	-0.30823	0.609256	0.2152
ZNF790	3	0.46468	0.779229	0.108335	*REM2*	3	0.24791	0.610774	0.21412
IFNA1	1	0.53085	0.779229	0.108335	*INTS14*	3	-0.051473	0.611185	0.213827
MAD2L1BP	4	0.54951	0.779229	0.108335	*GSTA2*	4	-0.25933	0.613337	0.212301
KISS1	3	-0.61394	0.779846	0.107991	*SOX6*	3	-0.58386	0.613441	0.212227
CKMT2	2	-0.5658	0.779846	0.107991	*KRT34*	2	-0.46737	0.613441	0.212227
XAGE3	3	-0.52925	0.779846	0.107991	*PLXNC1*	4	0.027187	0.613441	0.212227
SLC38A10	4	-0.44646	0.779846	0.107991	*SLC39A9*	3	-0.11885	0.613829	0.211953
GLB1L2	2	-0.39845	0.779846	0.107991	*HSPA6*	1	-0.71764	0.614194	0.211694
FNDC10	2	-0.38276	0.779846	0.107991	*KLHDC3*	3	-0.62318	0.614194	0.211694
RBBP8NL	2	-0.35984	0.779846	0.107991	*ARG1*	4	-0.42073	0.614194	0.211694
CLCN6	4	-0.35303	0.779846	0.107991	*CDC42BPG*	4	-0.20246	0.614194	0.211694
TFAP4	4	-0.34882	0.779846	0.107991	*SHPRH*	3	-0.16176	0.614194	0.211694
FGGY	4	-0.33762	0.779846	0.107991	*IAH1*	4	0.20365	0.614194	0.211694
IGSF9	4	-0.33026	0.779846	0.107991	*SPDL1*	4	0.2997	0.614194	0.211694
GJC3	4	-0.30579	0.779846	0.107991	*CENPT*	4	0.33194	0.614194	0.211694
RAD54B	4	-0.29692	0.779846	0.107991	*WDR3*	4	0.33463	0.614194	0.211694
NEXMIF	4	-0.29133	0.779846	0.107991	*CRYAB*	2	0.34906	0.614194	0.211694
TRNAU1AP	4	-0.2684	0.779846	0.107991	*TOE1*	3	0.35972	0.614194	0.211694
CCDC66	4	-0.11278	0.779846	0.107991	*ZNF432*	3	0.37573	0.614194	0.211694
MSL1	4	0.071271	0.779846	0.107991	*BUB3*	4	0.4794	0.614194	0.211694
GNL1	3	0.12502	0.779846	0.107991	*NACA*	3	0.64435	0.614194	0.211694
CYP17A1	4	0.13744	0.779846	0.107991	*EPHA10*	4	-0.32074	0.614322	0.211604
MCRIP1	4	0.18996	0.779846	0.107991	*PNMT*	4	-0.30821	0.614322	0.211604
KMT2C	4	0.19716	0.779846	0.107991	*SERPINC1*	2	0.33417	0.61433	0.211598
SPAG16-DT	4	0.20772	0.779846	0.107991	*AOPEP*	4	-0.32406	0.614848	0.211232
TUBGCP4	3	0.20933	0.779846	0.107991	*ABCB5*	3	-0.61217	0.615816	0.210549
GIMAP4	4	0.22211	0.779846	0.107991	*ATP2A3*	3	-0.51893	0.615816	0.210549
SEMA7A	4	0.26193	0.779846	0.107991	*GATA5*	4	-0.32161	0.615816	0.210549
KLK2	4	0.28483	0.779846	0.107991	*GSG1L*	3	-0.21776	0.615816	0.210549
EIF3H	4	0.30865	0.779846	0.107991	*CCL3L3*	3	0.047749	0.615816	0.210549
CBFA2T3	4	0.3219	0.779846	0.107991	*DPM1*	4	0.21418	0.615816	0.210549
TLE3	3	0.35434	0.779846	0.107991	*ECM2*	3	0.26977	0.615816	0.210549
NDUFAF2	3	0.35851	0.779846	0.107991	*DDX11*	2	0.48813	0.615816	0.210549
MYDGF	4	0.35921	0.779846	0.107991	*RUVBL1*	3	0.49476	0.615816	0.210549
PPARA	2	0.39067	0.779846	0.107991	*CCDC70*	4	-0.24951	0.615964	0.210444
CCDC14	2	0.39594	0.779846	0.107991	*NTN4*	4	-0.036458	0.615964	0.210444
TMOD4	4	0.40267	0.779846	0.107991	*HSPA4*	4	0.20984	0.615969	0.210441

USP32	4	0.4214	0.779846	0.107991	*EXOSC9*	4	0.54385	0.615969	0.210441
ITM2A	3	0.42253	0.779846	0.107991	*CARD19*	3	-0.3217	0.616021	0.210404
TRIM44	3	0.4996	0.779846	0.107991	*NRG1*	4	-0.19742	0.616855	0.209817
SRRT	4	0.52913	0.779846	0.107991	*BRI3BP*	3	0.059328	0.61694	0.209757
AK6	4	0.59726	0.779846	0.107991	*NFIX*	3	0.32738	0.618554	0.208622
VPS45	3	0.86269	0.779846	0.107991	*RGL1*	3	0.2311	0.618832	0.208427
TAGAP	1	-0.55559	0.785046	0.105105	*DSPP*	3	0.34293	0.618832	0.208427
TGFBR3L	4	0.12932	0.785046	0.105105	*RBL2*	3	-0.50709	0.619078	0.208255
HNRNPUL1	4	0.26703	0.785046	0.105105	*CAPZA1*	4	-0.39037	0.619078	0.208255
FLRT1	4	0.37088	0.785046	0.105105	*BRWD3*	3	-0.16955	0.619078	0.208255
IL20RA	4	-0.4764	0.785238	0.104999	*TNFRSF14*	4	-0.10455	0.619078	0.208255
SETSIP	3	0.13798	0.785642	0.104775	*DLX5*	3	0.097919	0.619078	0.208255
NPM1	1	0.53644	0.785642	0.104775	*PLEKHM1*	4	0.10113	0.619078	0.208255
ZNF706	1	-0.90582	0.785657	0.104767	*PRPF39*	4	0.28837	0.619078	0.208255
ARL6IP6	3	-0.5946	0.785657	0.104767	*LGMN*	2	0.30396	0.619078	0.208255
TMEM230	2	-0.55088	0.785657	0.104767	*RNF32*	3	0.33557	0.619078	0.208255
PRDM12	3	-0.37423	0.785657	0.104767	*ULK1*	4	0.3765	0.619078	0.208255
TMEM106A	4	-0.33936	0.785657	0.104767	*EIF3J*	3	0.44795	0.619078	0.208255
FAM229A	4	-0.32904	0.785657	0.104767	*FOXA1*	3	0.54308	0.619078	0.208255
CCDC17	2	-0.30269	0.785657	0.104767	*PDP1*	4	-0.45836	0.619621	0.207874
CTNNBIP1	4	-0.27435	0.785657	0.104767	*STING1*	4	0.27211	0.620044	0.207578
RER1	4	-0.26917	0.785657	0.104767	*ZNF862*	4	0.14813	0.620168	0.207491
ERI2	3	-0.255	0.785657	0.104767	*MMACHC*	4	0.27585	0.620168	0.207491
LANCL2	3	-0.2259	0.785657	0.104767	*SELENOV*	4	0.30572	0.620168	0.207491
PARP9	4	-0.2179	0.785657	0.104767	*ERP44*	4	0.3351	0.620168	0.207491
RAP1A	4	-0.13796	0.785657	0.104767	*SLC9A3R2*	2	-0.28488	0.621179	0.206783
GK5	4	0.027608	0.785657	0.104767	*TIMM50*	3	-0.19023	0.621644	0.206458
MED29	4	0.13412	0.785657	0.104767	*PDXP*	3	0.26486	0.621852	0.206313
HPD	4	0.25049	0.785657	0.104767	*IL20RB*	3	0.31958	0.621852	0.206313
RANBP6	4	0.2505	0.785657	0.104767	*PPP2CB*	4	-0.17705	0.623124	0.205426
CLIC2	4	0.30515	0.785657	0.104767	*SH3BP5L*	4	-0.17319	0.623216	0.205361
H1-10	3	0.30528	0.785657	0.104767	*CHRNA3*	3	-0.29801	0.623309	0.205297
ALDH1A2	3	0.30853	0.785657	0.104767	*CYYR1*	1	0.55835	0.624175	0.204693
CPSF3	3	0.50348	0.785657	0.104767	*MCRIP2*	4	-0.49955	0.624512	0.204459
PPP4C	3	-0.006464	0.78662	0.104235	*DPH2*	3	0.25828	0.624512	0.204459
ERICH1	2	-0.30911	0.787137	0.10395	*DGCR6L*	4	0.22046	0.624788	0.204267
SPATA5L1	4	0.1826	0.787137	0.10395	*UCK1*	4	0.26226	0.624788	0.204267
HEATR1	4	0.24355	0.787137	0.10395	*PCDH12*	4	0.30218	0.624788	0.204267
DOCK6	4	0.28821	0.787137	0.10395	*ZBED1*	3	-0.17976	0.625249	0.203947
UGP2	2	0.45073	0.787695	0.103642	*LAMA3*	3	0.29372	0.625482	0.203785
ZNF800	4	-0.30119	0.78779	0.10359	*PIK3R4*	4	-0.59739	0.626274	0.203236
SYT16	4	0.13502	0.78779	0.10359	*RAB40B*	3	-0.56145	0.626274	0.203236
DUSP4	4	-0.13612	0.787793	0.103588	*DRC3*	3	-0.48365	0.626274	0.203236
HROB	3	-0.76037	0.788108	0.103415	*PIH1D3*	4	-0.4301	0.626274	0.203236
TTC31	4	-0.50656	0.788108	0.103415	*GPR22*	4	-0.39932	0.626274	0.203236
CAMK2N1	4	0.0752	0.788108	0.103415	*PLEKHH3*	4	-0.39602	0.626274	0.203236
HOXA3	2	0.26917	0.788108	0.103415	*PMAIP1*	3	-0.37459	0.626274	0.203236
RECQL4	4	0.28005	0.788108	0.103415	*GBP7*	2	-0.32607	0.626274	0.203236
CHD5	1	0.54063	0.788108	0.103415	*LOC389895*	2	-0.31787	0.626274	0.203236
ROCK1	3	0.28333	0.788133	0.103401	*PCDHGB6*	4	-0.28832	0.626274	0.203236
UBE2K	3	-0.11464	0.788375	0.103267	*PCDH1*	4	-0.060937	0.626274	0.203236
SLC27A1	3	-0.89272	0.788703	0.103087	*ASAH1*	4	-0.039946	0.626274	0.203236
EYA4	4	-0.35886	0.78995	0.102401	*PCNP*	3	-0.00867	0.626274	0.203236
SIRPD	4	-0.32172	0.78995	0.102401	*C8G*	3	0.077438	0.626274	0.203236
PER3	4	-0.21605	0.78995	0.102401	*NTN1*	4	0.081988	0.626274	0.203236
ZCCHC18	3	-0.025985	0.78995	0.102401	*TMEM87A*	4	0.155	0.626274	0.203236
MRPL11	3	0.061505	0.78995	0.102401	*SPIRE1*	4	0.19381	0.626274	0.203236
EFNA4	2	0.13696	0.78995	0.102401	*APOOL*	4	0.27306	0.626274	0.203236

SERP1	4	0.21336	0.78995	0.102401	*PLBD2*	4	0.32352	0.626274	0.203236
TCEANC2	4	0.34189	0.78995	0.102401	*PRKAG1*	4	0.32774	0.626274	0.203236
GTF2H1	4	0.31508	0.790017	0.102363	*PCGF2*	3	0.33516	0.626274	0.203236
RCHY1	4	-0.30581	0.790949	0.101852	*UNC45B*	3	0.38136	0.626274	0.203236
AKAP9	3	-0.44377	0.791074	0.101783	*PDIA5*	3	0.43608	0.626274	0.203236
PCDHGA9	3	-0.41882	0.791074	0.101783	*CCT3*	4	0.60149	0.626274	0.203236
WDR91	4	-0.32492	0.791074	0.101783	*MOB4*	1	0.73319	0.626274	0.203236
NUDT15	4	-0.27055	0.791074	0.101783	*LGALS8*	3	-0.49635	0.626504	0.203076
PRR13	2	0.27709	0.791074	0.101783	*FOSL2*	3	-0.77179	0.62769	0.202254
BRMS1	2	0.28288	0.791074	0.101783	*THNSL2*	4	-0.40004	0.62769	0.202254
NPPB	2	0.4593	0.791074	0.101783	*DCAF7*	4	-0.16304	0.62769	0.202254
BAG2	4	-0.34958	0.7918	0.101385	*MTNR1A*	4	-0.61565	0.627935	0.202085
MTSS2	4	-0.10481	0.7918	0.101385	*NBPF7*	3	-0.55011	0.627935	0.202085
KIAA0895	4	-0.20864	0.792006	0.101271	*TCF7L2*	4	-0.015936	0.627935	0.202085
ADGRF1	4	-0.42312	0.79228	0.101122	*RHBDD3*	4	0.27733	0.627935	0.202085
TYMS	4	-0.14649	0.79228	0.101122	*KLF9*	4	0.2865	0.627935	0.202085
TMEM255A	4	-0.36247	0.792336	0.101091	*KIAA1324L*	4	0.09224	0.628153	0.201934
PIM3	4	-0.31966	0.792336	0.101091	*PDE1A*	3	0.12789	0.628168	0.201924
PRG4	3	-0.20791	0.792336	0.101091	*NPY4R2*	1	-0.67649	0.628225	0.201885
ANK1	4	-0.17603	0.792336	0.101091	*RUFY3*	3	-0.57585	0.628758	0.201516
CATSPER3	4	-0.15571	0.792336	0.101091	*LGI4*	4	-0.34479	0.628758	0.201516
PSMD1	4	-0.085034	0.792336	0.101091	*MEAK7*	4	-0.33687	0.628758	0.201516
IPO11	4	0.00137	0.792336	0.101091	*TRIM68*	3	-0.32534	0.628758	0.201516
CNN2	4	0.006044	0.792336	0.101091	*ZBED6CL*	4	-0.28772	0.628758	0.201516
TARDBP	3	0.071303	0.792336	0.101091	*E2F4*	4	-0.25341	0.628758	0.201516
PILRA	4	0.19155	0.792336	0.101091	*TMEM268*	4	-0.24973	0.628758	0.201516
BLVRB	2	0.21484	0.792336	0.101091	*WNT9A*	4	-0.24548	0.628758	0.201516
CYP27B1	4	0.29974	0.792336	0.101091	*MPP7*	4	-0.20889	0.628758	0.201516
ERMN	4	0.30819	0.792336	0.101091	*SCYL3*	2	-0.11278	0.628758	0.201516
EN2	3	0.38789	0.792336	0.101091	*GPER1*	3	0.16021	0.628758	0.201516
USP34	3	0.43931	0.792336	0.101091	*C19orf25*	3	0.17007	0.628758	0.201516
FAM76A	3	0.47001	0.792336	0.101091	*WASHC4*	3	0.21037	0.628758	0.201516
SAP30	2	0.51734	0.792336	0.101091	*ZNF404*	3	0.22264	0.628758	0.201516
ADGRE5	3	-1.021	0.793818	0.100279	*GRSF1*	4	0.24143	0.628758	0.201516
METTL2B	1	-0.85775	0.793818	0.100279	*NRAS*	3	0.27453	0.628758	0.201516
DHX40	3	-0.80237	0.793818	0.100279	*C19orf38*	4	0.28708	0.628758	0.201516
MAP2K2	2	-0.71718	0.793818	0.100279	*BYSL*	3	0.5659	0.628758	0.201516
SELENOM	2	-0.70619	0.793818	0.100279	*VPS11*	4	0.098137	0.628953	0.201382
FXR2	3	-0.59003	0.793818	0.100279	*BSN*	4	-0.022054	0.629287	0.201151
ACP2	4	-0.57545	0.793818	0.100279	*RPP21*	1	-1.4026	0.630026	0.200641
NNT	2	-0.55309	0.793818	0.100279	*DUSP18*	4	-0.37809	0.630242	0.200492
NECTIN1	3	-0.51609	0.793818	0.100279	*ZFP92*	4	-0.23557	0.630565	0.20027
IFNA8	3	-0.5031	0.793818	0.100279	*MEI1*	4	-0.29536	0.63072	0.200164
MRS2	3	-0.49012	0.793818	0.100279	*OR56A4*	2	0.17053	0.63072	0.200164
TERT	2	-0.4889	0.793818	0.100279	*LYPD8*	3	-0.65094	0.632716	0.198791
FAM90A1	2	-0.45001	0.793818	0.100279	*PRDX3*	2	-0.4453	0.632716	0.198791
IER5	4	-0.43744	0.793818	0.100279	*FBXO2*	3	-0.44049	0.632716	0.198791
PRSS36	4	-0.39953	0.793818	0.100279	*RTRAF*	4	-0.44011	0.632716	0.198791
MAPK13	4	-0.39819	0.793818	0.100279	*TBC1D24*	4	-0.33427	0.632716	0.198791
UBQLN4	4	-0.38188	0.793818	0.100279	*GEM*	4	-0.3315	0.632716	0.198791
DGKG	3	-0.37318	0.793818	0.100279	*ALKAL1*	4	-0.31969	0.632716	0.198791
ID3	4	-0.37157	0.793818	0.100279	*USH1G*	4	-0.26801	0.632716	0.198791
ZNF883	4	-0.35295	0.793818	0.100279	*TSPAN19*	4	-0.20011	0.632716	0.198791
LRRC10B	3	-0.35288	0.793818	0.100279	*DEFB126*	4	-0.10024	0.632716	0.198791
HCAR2	4	-0.34512	0.793818	0.100279	*GUF1*	4	-0.0488	0.632716	0.198791
ZSCAN29	4	-0.33376	0.793818	0.100279	*LAS1L*	4	0.10007	0.632716	0.198791
LCA5L	2	-0.31679	0.793818	0.100279	*MED9*	4	0.21088	0.632716	0.198791
KCNH2	4	-0.31586	0.793818	0.100279	*GPR108*	4	0.2353	0.632716	0.198791

GPAT3	3	-0.30701	0.793818	0.100279	*ABCA13*	4	0.23545	0.632716	0.198791
ROCK2	4	-0.30242	0.793818	0.100279	*MYADML2*	4	0.23567	0.632716	0.198791
FCGR3B	2	-0.30164	0.793818	0.100279	*CCR10*	4	0.23898	0.632716	0.198791
TEX45	4	-0.29781	0.793818	0.100279	*ARPC2*	4	0.2567	0.632716	0.198791
ELFN2	4	-0.28889	0.793818	0.100279	*ZNF605*	4	0.26126	0.632716	0.198791
IQGAP3	4	-0.28171	0.793818	0.100279	*GRID2IP*	4	0.26985	0.632716	0.198791
ING2	3	-0.28164	0.793818	0.100279	*RWDD3*	4	0.27684	0.632716	0.198791
KDF1	2	-0.27923	0.793818	0.100279	*SYVN1*	4	0.28098	0.632716	0.198791
PIAS2	4	-0.2716	0.793818	0.100279	*WDR61*	4	0.29523	0.632716	0.198791
SH3GLB1	4	-0.25956	0.793818	0.100279	*ADPRM*	4	0.30112	0.632716	0.198791
PSMC1	3	-0.24326	0.793818	0.100279	*MSI1*	4	0.30722	0.632716	0.198791
RINT1	3	-0.21095	0.793818	0.100279	*P3H3*	4	0.31145	0.632716	0.198791
IRAK2	3	-0.20498	0.793818	0.100279	*USP37*	4	0.35755	0.632716	0.198791
SETD5	4	-0.18599	0.793818	0.100279	*PARD6A*	4	0.36708	0.632716	0.198791
GPR135	4	-0.18147	0.793818	0.100279	*FAM86C1*	1	0.38095	0.632716	0.198791
TIAM1	4	-0.1681	0.793818	0.100279	*PITPNB*	3	0.42461	0.632716	0.198791
IGDCC3	4	-0.15275	0.793818	0.100279	*SLC37A2*	3	0.43268	0.632716	0.198791
E2F4	4	-0.13015	0.793818	0.100279	*MAK16*	4	0.47551	0.632716	0.198791
PXDC1	4	-0.095703	0.793818	0.100279	*CALCA*	3	0.54174	0.632716	0.198791
NKD2	4	-0.093345	0.793818	0.100279	*GMNN*	3	0.59687	0.632716	0.198791
RCAN2	3	-0.092288	0.793818	0.100279	*RAPGEF6*	3	-0.7921	0.633269	0.198412
OAF	4	-0.072946	0.793818	0.100279	*VIP*	3	-0.59788	0.633269	0.198412
OGG1	4	-0.027964	0.793818	0.100279	*ARL5A*	3	0.27282	0.633269	0.198412
ANGPTL3	4	-0.015521	0.793818	0.100279	*ZNF7*	2	0.29315	0.633596	0.198187
RFX4	4	0.007284	0.793818	0.100279	*ERC2*	3	-0.10976	0.633656	0.198147
SPATS2	4	0.013855	0.793818	0.100279	*CYHR1*	3	0.024442	0.633656	0.198147
DDX50	3	0.049845	0.793818	0.100279	*RPL21*	2	0.38339	0.633656	0.198147
KCNK7	4	0.057446	0.793818	0.100279	*INTS2*	3	0.58238	0.633656	0.198147
ME3	4	0.093372	0.793818	0.100279	*MATN1*	3	0.11368	0.633857	0.198009
EFR3A	4	0.097505	0.793818	0.100279	*PGP*	4	-0.27547	0.634362	0.197663
CIB2	2	0.12538	0.793818	0.100279	*NEK6*	4	-0.45978	0.634995	0.19723
GID8	3	0.13203	0.793818	0.100279	*IGSF9*	4	-0.1273	0.634995	0.19723
DCHS2	4	0.13244	0.793818	0.100279	*PKD1*	1	-0.5416	0.63508	0.197172
NRDC	4	0.16728	0.793818	0.100279	*C2orf50*	4	-0.31059	0.635081	0.197171
SPATA12	3	0.16859	0.793818	0.100279	*TBC1D25*	4	-0.29419	0.635081	0.197171
ESPL1	4	0.17991	0.793818	0.100279	*FGF14*	3	-0.27691	0.635081	0.197171
AKR1A1	4	0.19307	0.793818	0.100279	*RAP2B*	4	-0.26751	0.635081	0.197171
LRRC8D	4	0.2167	0.793818	0.100279	*PIAS2*	4	0.031103	0.635081	0.197171
KPNA7	4	0.22206	0.793818	0.100279	*MYO9B*	4	0.22392	0.635081	0.197171
TPTE2	3	0.22324	0.793818	0.100279	*ADAMTS9*	4	0.22905	0.635081	0.197171
COA8	3	0.22464	0.793818	0.100279	*THUMPD1*	4	0.28123	0.635081	0.197171
G6PC	3	0.22758	0.793818	0.100279	*FGF9*	1	0.41831	0.635081	0.197171
IRF3	4	0.23134	0.793818	0.100279	*UBTF*	3	0.54973	0.635081	0.197171
COX7A1	4	0.24077	0.793818	0.100279	*CCDC50*	4	-0.30981	0.635148	0.197125
OCIAD2	4	0.24332	0.793818	0.100279	*RIN3*	4	-0.29864	0.635148	0.197125
ZNF17	4	0.24466	0.793818	0.100279	*CMAS*	3	-0.15617	0.635148	0.197125
NEIL2	4	0.25311	0.793818	0.100279	*SYT11*	4	0.030434	0.635608	0.196811
NUDT17	4	0.25775	0.793818	0.100279	*OASL*	4	-0.36954	0.636517	0.19619
CLN8	4	0.26068	0.793818	0.100279	*RGS14*	4	0.16605	0.636517	0.19619
CCDC74B	3	0.26549	0.793818	0.100279	*JCHAIN*	4	-0.19396	0.636675	0.196082
GPA33	4	0.26756	0.793818	0.100279	*PCLAF*	4	-0.36527	0.637249	0.195691
ZSCAN10	4	0.26941	0.793818	0.100279	*ZNF547*	4	-0.20557	0.637323	0.19564
WDR12	3	0.27145	0.793818	0.100279	*MYOT*	4	0.088236	0.637323	0.19564
SON	4	0.27146	0.793818	0.100279	*TMTC2*	4	0.21444	0.637323	0.19564
ATP1B2	4	0.27923	0.793818	0.100279	*SOAT1*	4	0.25636	0.637323	0.19564
ALDH9A1	4	0.28027	0.793818	0.100279	*FOLH1B*	2	-0.29497	0.637564	0.195476
DUT	4	0.28059	0.793818	0.100279	*GLRX3*	3	-0.24122	0.637564	0.195476
KHDC4	4	0.28308	0.793818	0.100279	*PIP5K1A*	3	-0.99265	0.637921	0.195233

ZNF135	4	0.29836	0.793818	0.100279	*ARRB2*	3	-0.52847	0.637921	0.195233
KRT80	4	0.29889	0.793818	0.100279	*CD40*	4	-0.30572	0.637921	0.195233
JAK2	4	0.3009	0.793818	0.100279	*FER1L5*	4	-0.20049	0.637921	0.195233
TTLL10	4	0.30114	0.793818	0.100279	*GLUL*	4	-0.17514	0.637921	0.195233
SLC52A2	4	0.30516	0.793818	0.100279	*C1orf127*	4	-0.15421	0.637921	0.195233
PCDHB5	4	0.31149	0.793818	0.100279	*OAF*	4	-0.052758	0.637921	0.195233
CYB561A3	4	0.32222	0.793818	0.100279	*MYLK2*	4	0.054657	0.637921	0.195233
CATSPER1	2	0.3238	0.793818	0.100279	*ATRX*	3	0.060446	0.637921	0.195233
PSME4	4	0.33241	0.793818	0.100279	*SMARCC2*	4	0.065848	0.637921	0.195233
DUSP27	3	0.33304	0.793818	0.100279	*GTF3C1*	4	0.19196	0.637921	0.195233
GRIN2B	4	0.33404	0.793818	0.100279	*FRK*	4	0.20243	0.637921	0.195233
ITLN1	4	0.34404	0.793818	0.100279	*ATPSCKMT*	4	0.2421	0.637921	0.195233
EID2B	4	0.34605	0.793818	0.100279	*WDHD1*	4	0.26223	0.637921	0.195233
PLXNA2	3	0.34773	0.793818	0.100279	*EPN3*	4	0.27935	0.637921	0.195233
SMC1A	4	0.35171	0.793818	0.100279	*ST8SIA5*	4	-0.39249	0.638398	0.194909
ANAPC10	4	0.35833	0.793818	0.100279	*FBF1*	3	0.079785	0.638398	0.194909
ZCWPW2	4	0.36802	0.793818	0.100279	*PLEKHH1*	3	-0.27474	0.63907	0.194451
B3GNT5	3	0.37219	0.793818	0.100279	*GAB2*	2	-0.62006	0.639261	0.194322
ZNF275	3	0.38467	0.793818	0.100279	*GAL3ST1*	2	-0.59453	0.639261	0.194322
GCSH	2	0.45217	0.793818	0.100279	*TPGS2*	3	-0.58872	0.639261	0.194322
STIL	4	0.47211	0.793818	0.100279	*S100A7L2*	3	-0.56319	0.639261	0.194322
RNF34	4	0.50893	0.793818	0.100279	*ZNF641*	2	-0.54984	0.639261	0.194322
RPL35	4	0.51571	0.793818	0.100279	*PRKN*	3	-0.53042	0.639261	0.194322
IMPDH2	2	0.52795	0.793818	0.100279	*SLC35F6*	2	-0.4961	0.639261	0.194322
HABP2	3	0.53339	0.793818	0.100279	*NUDT14*	4	-0.49241	0.639261	0.194322
ATOH8	3	0.53589	0.793818	0.100279	*CITED2*	2	-0.48022	0.639261	0.194322
SART1	3	0.53819	0.793818	0.100279	*PHKG1*	3	-0.47061	0.639261	0.194322
SMG1	3	0.55189	0.793818	0.100279	*COL4A2*	3	-0.44747	0.639261	0.194322
PMS1	3	0.62171	0.793818	0.100279	*PANK3*	4	-0.42665	0.639261	0.194322
OSTC	3	0.65462	0.793818	0.100279	*KRT74*	4	-0.42498	0.639261	0.194322
PGAP6	2	0.73378	0.793818	0.100279	*WDFY3*	4	-0.40779	0.639261	0.194322
PSMD6	4	0.79887	0.793818	0.100279	*TMF1*	4	-0.35578	0.639261	0.194322
FRY	4	-0.34556	0.794424	0.099947	*SLFN12*	4	-0.3546	0.639261	0.194322
IAPP	3	-0.31275	0.794424	0.099947	*FLNA*	4	-0.31941	0.639261	0.194322
ANKRD18A	3	-0.10341	0.794424	0.099947	*C14orf28*	3	-0.31893	0.639261	0.194322
CHRNA3	3	-0.085445	0.794424	0.099947	*FAM78B*	4	-0.31768	0.639261	0.194322
MT1E	3	0.00877	0.794424	0.099947	*TMEM221*	4	-0.31279	0.639261	0.194322
KIF16B	4	0.29583	0.794424	0.099947	*FGF21*	4	-0.28457	0.639261	0.194322
SKP2	3	0.85969	0.794424	0.099947	*BACH2*	4	-0.26755	0.639261	0.194322
CD46	2	0.29966	0.794426	0.099947	*PHF7*	4	-0.26603	0.639261	0.194322
CRYBA4	3	-0.60132	0.795818	0.099187	*MST1R*	4	-0.24606	0.639261	0.194322
HLCS	4	-0.30482	0.795818	0.099187	*C2orf42*	3	-0.20611	0.639261	0.194322
SCN9A	2	-0.28787	0.795818	0.099187	*ATP1B1*	4	-0.2051	0.639261	0.194322
HIVEP3	2	-0.12896	0.795818	0.099187	*KLF8*	3	-0.20127	0.639261	0.194322
SEMA5B	3	-0.10868	0.795818	0.099187	*DYNAP*	4	-0.19944	0.639261	0.194322
ZBED1	3	-0.076426	0.795818	0.099187	*TMC5*	3	-0.15476	0.639261	0.194322
SEMA3C	3	0.27119	0.795818	0.099187	*IRGC*	4	-0.13886	0.639261	0.194322
TMEM44	4	-0.2767	0.796021	0.099075	*TOGARAM1*	4	-0.087336	0.639261	0.194322
RFPL2	4	-0.26377	0.796021	0.099075	*TCEAL3*	3	-0.037815	0.639261	0.194322
RBM20	3	-0.10181	0.796021	0.099075	*ETV4*	3	-0.001833	0.639261	0.194322
TOGARAM1	4	-0.0681	0.796021	0.099075	*NSUN2*	3	0.001364	0.639261	0.194322
CEP76	4	0.061361	0.796021	0.099075	*RACGAP1*	3	0.01332	0.639261	0.194322
NRG4	4	0.16359	0.796021	0.099075	*C7orf50*	3	0.028312	0.639261	0.194322
MED26	4	0.16635	0.796021	0.099075	*CCHCR1*	3	0.041969	0.639261	0.194322
RSF1	4	0.2719	0.796021	0.099075	*MDM2*	4	0.051547	0.639261	0.194322
ZNF254	4	0.2944	0.796021	0.099075	*DENND2A*	4	0.055248	0.639261	0.194322
PDGFRB	4	0.39322	0.796021	0.099075	*ZHX3*	4	0.064438	0.639261	0.194322
NQO1	4	0.42017	0.796021	0.099075	*SNX32*	3	0.074827	0.639261	0.194322

RPAIN	3	0.57554	0.796021	0.099075	*CHST11*	3	0.093327	0.639261	0.194322
DNPH1	4	-0.4407	0.796186	0.098985	*KIAA1109*	4	0.098156	0.639261	0.194322
SLC23A2	4	0.19983	0.796186	0.098985	*PRKCH*	4	0.13312	0.639261	0.194322
ACADVL	3	-0.38506	0.79688	0.098607	*RXFP1*	4	0.15217	0.639261	0.194322
ACVR1C	4	-0.35914	0.797625	0.098201	*SLC44A4*	4	0.19742	0.639261	0.194322
PAWR	4	-0.062152	0.797625	0.098201	*ZBTB7B*	4	0.2047	0.639261	0.194322
FBLN5	4	0.029239	0.797625	0.098201	*MUS81*	4	0.22074	0.639261	0.194322
RASGRF2	4	-0.41426	0.797769	0.098123	*ZNF467*	4	0.22187	0.639261	0.194322
POLG	4	-0.38919	0.797769	0.098123	*TENT5B*	4	0.22374	0.639261	0.194322
SLC25A12	3	-0.24564	0.797769	0.098123	*CGRRF1*	4	0.22599	0.639261	0.194322
ASB1	4	-0.18998	0.797769	0.098123	*CHIA*	4	0.22755	0.639261	0.194322
CPXM1	4	0.028154	0.797769	0.098123	*ATM*	4	0.23127	0.639261	0.194322
FAM83E	4	0.13295	0.797769	0.098123	*DNAJB6*	4	0.23867	0.639261	0.194322
CYS1	4	0.21168	0.797769	0.098123	*CCAR1*	4	0.25861	0.639261	0.194322
PATJ	4	0.32562	0.797769	0.098123	*ZNF774*	3	0.25969	0.639261	0.194322
ACR	3	-0.64137	0.798551	0.097698	*SMPD3*	4	0.26146	0.639261	0.194322
HOXB9	2	-0.6011	0.798551	0.097698	*SRRM1*	4	0.26625	0.639261	0.194322
FGL2	3	-0.55179	0.798551	0.097698	*TOR1B*	4	0.26627	0.639261	0.194322
MEX3B	2	-0.50071	0.798551	0.097698	*TMEM159*	3	0.26798	0.639261	0.194322
IRF2BP2	4	-0.46386	0.798551	0.097698	*RBM39*	4	0.2701	0.639261	0.194322
APEX1	3	-0.39341	0.798551	0.097698	*WNK3*	4	0.27557	0.639261	0.194322
MANBA	4	-0.35152	0.798551	0.097698	*PSPN*	4	0.29247	0.639261	0.194322
TMEM70	4	-0.27831	0.798551	0.097698	*RPTOR*	4	0.30366	0.639261	0.194322
CCDC90B	4	-0.25242	0.798551	0.097698	*ACOX1*	4	0.31486	0.639261	0.194322
CCDC85B	2	-0.20403	0.798551	0.097698	*NUFIP1*	4	0.32261	0.639261	0.194322
EMB	3	-0.19069	0.798551	0.097698	*ZYG11B*	4	0.32728	0.639261	0.194322
ESR1	4	-0.18428	0.798551	0.097698	*CCL22*	3	0.3291	0.639261	0.194322
MB21D2	4	-0.13276	0.798551	0.097698	*NME7*	3	0.3313	0.639261	0.194322
CLDN8	4	0.009796	0.798551	0.097698	*PDGFRB*	4	0.33414	0.639261	0.194322
ZCCHC3	4	0.04374	0.798551	0.097698	*CEP57L1*	4	0.33515	0.639261	0.194322
ZDHHC11	4	0.083001	0.798551	0.097698	*MPHOSPH1(*	4	0.33657	0.639261	0.194322
RIF1	3	0.20454	0.798551	0.097698	*ADPRHL2*	4	0.3479	0.639261	0.194322
SIAH1	4	0.21094	0.798551	0.097698	*FAM131C*	4	0.34986	0.639261	0.194322
PTP4A2	2	0.28486	0.798551	0.097698	*ADGRE2*	3	0.36313	0.639261	0.194322
NDUFA2	4	0.30772	0.798551	0.097698	*FAM220A*	3	0.36471	0.639261	0.194322
GHSR	4	0.30846	0.798551	0.097698	*ETFB*	4	0.37338	0.639261	0.194322
HSD11B1L	2	0.31495	0.798551	0.097698	*SNRNP25*	4	0.37434	0.639261	0.194322
THUMPD3	3	0.36643	0.798551	0.097698	*CGGBP1*	3	0.37572	0.639261	0.194322
FAM20C	3	0.36929	0.798551	0.097698	*TOMM70*	4	0.37809	0.639261	0.194322
VSIG10L	3	0.41221	0.798551	0.097698	*CCDC174*	3	0.40622	0.639261	0.194322
IST1	4	0.4145	0.798551	0.097698	*ZNF703*	3	0.41149	0.639261	0.194322
LIPT2	2	0.44452	0.798551	0.097698	*COA7*	3	0.41486	0.639261	0.194322
E2F2	4	0.44674	0.798551	0.097698	*TFRC*	4	0.41604	0.639261	0.194322
ATOX1	3	0.49279	0.798551	0.097698	*NUP155*	4	0.42041	0.639261	0.194322
SUMO1	1	0.50733	0.798551	0.097698	*MRGBP*	3	0.42283	0.639261	0.194322
CDS2	3	0.55927	0.798551	0.097698	*DNAJC15*	1	0.43134	0.639261	0.194322
RBX1	3	0.61717	0.798551	0.097698	*COLCA1*	3	0.45878	0.639261	0.194322
RPS6KL1	4	0.15269	0.798812	0.097555	*SRP9*	1	0.46403	0.639261	0.194322
HCN3	3	0.028345	0.798932	0.09749	*SMC5*	3	0.47477	0.639261	0.194322
PSG5	2	0.29186	0.799033	0.097435	*SNRPE*	3	0.48225	0.639261	0.194322
POLR2A	2	-0.83457	0.801601	0.096042	*VCX*	1	0.49229	0.639261	0.194322
CARD9	2	-0.65627	0.801601	0.096042	*KDM5C*	1	0.51496	0.639261	0.194322
SPIN4	3	-0.56057	0.801601	0.096042	*GNL2*	3	0.52771	0.639261	0.194322
GINS4	3	-0.50693	0.801601	0.096042	*UBE2N*	2	0.56129	0.639261	0.194322
EPHA5	4	-0.29872	0.80178	0.095945	*CDC7*	4	0.71543	0.639261	0.194322
NAP1L4	2	-0.51592	0.802095	0.095774	*DDB1*	1	0.7575	0.639261	0.194322
ATAT1	3	-0.50144	0.802095	0.095774	*POLK*	3	-0.56015	0.639471	0.194179
PURG	3	-0.39218	0.802095	0.095774	*C5AR1*	4	0.20827	0.639471	0.194179

MAPK14	4	-0.31368	0.802095	0.095774	*MAML2*	4	0.26166	0.639471	0.194179
TGFBI	4	-0.30849	0.802095	0.095774	*CALM3*	4	-0.34956	0.639541	0.194132
EZH1	4	-0.2724	0.802095	0.095774	*HAVCR1*	4	-0.30277	0.639541	0.194132
MRPL1	2	-0.10759	0.802095	0.095774	*HSD17B3*	3	0.50584	0.639541	0.194132
HOXD11	4	-0.024028	0.802095	0.095774	*IPPK*	4	0.50585	0.639801	0.193955
RIMKLB	3	-0.002757	0.802095	0.095774	*VIPR1*	2	0.25487	0.63983	0.193935
TACSTD2	3	0.002628	0.802095	0.095774	*KLK4*	4	-0.023179	0.63988	0.193902
TNNT1	4	0.31123	0.802095	0.095774	*DCLRE1B*	4	0.27917	0.640149	0.193719
LOC728392	4	0.32208	0.802095	0.095774	*MGAT3*	3	-0.79073	0.641174	0.193024
PHF21B	4	0.3953	0.802095	0.095774	*HPN*	3	-0.76169	0.641174	0.193024
HIGD1B	3	0.49617	0.802095	0.095774	*STK10*	3	-0.57517	0.641174	0.193024
XIRP1	4	0.19629	0.802182	0.095727	*TLR4*	3	-0.56807	0.641174	0.193024
TMEM151A	4	0.21249	0.802182	0.095727	*HLA-DRA*	3	-0.55926	0.641174	0.193024
ARMC6	4	0.26453	0.802182	0.095727	*HK3*	4	-0.39499	0.641174	0.193024
RBPJ	4	-0.53599	0.803186	0.095184	*PODNL1*	4	-0.38223	0.641174	0.193024
ATPSCKMT	4	-0.43841	0.803186	0.095184	*OTP*	3	-0.37163	0.641174	0.193024
PBX4	3	-0.42888	0.803186	0.095184	*MAP1A*	3	-0.34024	0.641174	0.193024
FBXO39	4	-0.2802	0.803186	0.095184	*XRCC1*	4	-0.32223	0.641174	0.193024
KRTAP5-10	3	-0.27009	0.803186	0.095184	*DQX1*	4	-0.31771	0.641174	0.193024
MAP4K4	3	-0.071695	0.803186	0.095184	*RASA3*	4	-0.31619	0.641174	0.193024
NRP2	3	-0.071221	0.803186	0.095184	*UGT1A6*	4	-0.3077	0.641174	0.193024
MLANA	3	-0.049597	0.803186	0.095184	*MAZ*	4	-0.30496	0.641174	0.193024
MGP	3	0.37052	0.803186	0.095184	*TEX38*	4	-0.2918	0.641174	0.193024
TAS2R30	4	0.28924	0.803901	0.094798	*PCDHB9*	4	-0.17155	0.641174	0.193024
TSEN15	3	0.28865	0.803905	0.094795	*TMEM35A*	4	-0.15762	0.641174	0.193024
TPRG1L	4	0.49679	0.803905	0.094795	*POFUT2*	4	-0.12532	0.641174	0.193024
ATP5MC1	2	0.64128	0.803905	0.094795	*NEUROD2*	4	-0.09699	0.641174	0.193024
SLC9A7	4	-0.30916	0.804122	0.094678	*SLC46A2*	4	-0.036861	0.641174	0.193024
COL28A1	4	-0.2575	0.804122	0.094678	*LTF*	4	0.006369	0.641174	0.193024
NAA50	4	0.16287	0.804122	0.094678	*KRT7*	3	0.009196	0.641174	0.193024
RNF170	4	0.27989	0.804122	0.094678	*EHMT2*	4	0.078595	0.641174	0.193024
CARNMT1	4	0.3204	0.804122	0.094678	*ZNF503*	4	0.087758	0.641174	0.193024
KRI1	3	0.32051	0.804122	0.094678	*LAPTM4A*	3	0.091117	0.641174	0.193024
PCMT1	3	0.39131	0.804122	0.094678	*PDP2*	4	0.097587	0.641174	0.193024
PIP4K2B	4	0.21432	0.80662	0.093331	*GLI4*	4	0.11029	0.641174	0.193024
PITX2	4	0.21562	0.80662	0.093331	*KPNA4*	4	0.11056	0.641174	0.193024
C4orf47	3	0.25667	0.80662	0.093331	*CSTB*	3	0.13237	0.641174	0.193024
TOE1	3	0.26524	0.80662	0.093331	*MON1B*	4	0.14076	0.641174	0.193024
SEMA3F	4	-0.27724	0.806739	0.093267	*TCERG1*	4	0.14536	0.641174	0.193024
KIF3A	4	-0.17981	0.806739	0.093267	*TLR3*	4	0.15396	0.641174	0.193024
DEDD2	4	0.3029	0.806739	0.093267	*PEG10*	4	0.16995	0.641174	0.193024
SLC7A6	4	0.34249	0.806739	0.093267	*AP5S1*	4	0.18391	0.641174	0.193024
FABP6	4	0.35357	0.806954	0.093151	*CP*	4	0.18561	0.641174	0.193024
ERMAP	2	-0.49965	0.80701	0.093121	*SCN7A*	4	0.18581	0.641174	0.193024
H3-5	2	0.24752	0.807857	0.092665	*CCDC73*	3	0.20839	0.641174	0.193024
CYSTM1	3	0.16347	0.808823	0.092147	*SULT4A1*	4	0.21978	0.641174	0.193024
PPIC	3	-0.58513	0.809728	0.091661	*TP53BP2*	4	0.22033	0.641174	0.193024
SLC9A2	3	-0.59673	0.810211	0.091402	*CYSTM1*	3	0.23026	0.641174	0.193024
PRRX2	2	-0.40795	0.810211	0.091402	*ILF2*	4	0.23722	0.641174	0.193024
ADCY10	4	-0.25236	0.810211	0.091402	*CHMP1A*	4	0.24176	0.641174	0.193024
PPP2R2D	3	-0.36938	0.810949	0.091006	*TGFBR3L*	4	0.24879	0.641174	0.193024
C1orf198	4	-0.33085	0.810949	0.091006	*FAM104A*	4	0.27603	0.641174	0.193024
PLEKHO2	4	-0.001054	0.810949	0.091006	*TTC21A*	4	0.27806	0.641174	0.193024
RNF135	4	-0.27976	0.811806	0.090548	*GPN3*	3	0.28967	0.641174	0.193024
RIBC2	4	0.13081	0.811806	0.090548	*DARS2*	4	0.34239	0.641174	0.193024
FURIN	4	0.28785	0.811806	0.090548	*FNDC3A*	4	0.35582	0.641174	0.193024
PLCXD2	4	0.34119	0.811806	0.090548	*ZBTB2*	4	0.36277	0.641174	0.193024
DHX33	4	0.52956	0.811806	0.090548	*USP32*	4	0.39312	0.641174	0.193024

ATXN10	2	-0.67768	0.811833	0.090533	*CDC45*	4	0.41169	0.641174	0.193024
XYLT2	1	-0.61107	0.811833	0.090533	*SKIL*	4	0.41688	0.641174	0.193024
SLC66A1	2	-0.54756	0.811833	0.090533	*BET1L*	3	0.44916	0.641174	0.193024
COL6A1	4	-0.48115	0.811833	0.090533	*WDR43*	4	0.48834	0.641174	0.193024
RSPH10B	1	-0.47217	0.811833	0.090533	*UTP11*	4	0.52896	0.641174	0.193024
FBXO30	4	-0.46102	0.811833	0.090533	*CKAP5*	4	0.54081	0.641174	0.193024
UBE2D4	3	-0.4482	0.811833	0.090533	*MGME1*	1	0.54307	0.641174	0.193024
FAM118A	4	-0.43216	0.811833	0.090533	*UTP3*	3	0.54999	0.641174	0.193024
SLC34A2	4	-0.4224	0.811833	0.090533	*COP1*	3	0.56016	0.641174	0.193024
PLXNA3	3	-0.42213	0.811833	0.090533	*BTBD7*	3	-0.69719	0.641303	0.192937
CCDC162P	4	-0.41239	0.811833	0.090533	*VRK2*	3	-0.66164	0.641303	0.192937
RPL13A	3	-0.4045	0.811833	0.090533	*B3GALT4*	3	-0.44203	0.641303	0.192937
TMEM123	3	-0.40287	0.811833	0.090533	*HLA-DRB5*	2	-0.334	0.641303	0.192937
GABPA	2	-0.38779	0.811833	0.090533	*ING2*	3	-0.31355	0.641303	0.192937
PTPN22	4	-0.38106	0.811833	0.090533	*FGD3*	4	0.019343	0.641303	0.192937
AGA	4	-0.3712	0.811833	0.090533	*IFIT5*	4	0.086616	0.641303	0.192937
PAPPA	4	-0.36714	0.811833	0.090533	*GRIK5*	3	0.16583	0.641303	0.192937
A2M	3	-0.36201	0.811833	0.090533	*LYPD6*	4	0.27782	0.641303	0.192937
TFB1M	3	-0.3581	0.811833	0.090533	*GTF3C4*	4	0.39438	0.641303	0.192937
MCHR1	4	-0.34864	0.811833	0.090533	*MTFP1*	2	0.49165	0.641303	0.192937
TNKS2	4	-0.31947	0.811833	0.090533	*LOC10065275*	3	0.23779	0.641557	0.192765
GNMT	2	-0.31395	0.811833	0.090533	*ZNF225*	4	-0.1613	0.642005	0.192462
PYM1	4	-0.30078	0.811833	0.090533	*XRN2*	4	0.60818	0.642005	0.192462
SLC2A14	4	-0.2918	0.811833	0.090533	*PABPN1L*	3	0.41844	0.642336	0.192238
AATK	4	-0.27031	0.811833	0.090533	*WNT9B*	4	-0.29799	0.642342	0.192233
ZNF547	4	-0.25442	0.811833	0.090533	*TRAFD1*	3	-0.19026	0.642342	0.192233
RNF115	4	-0.25319	0.811833	0.090533	*FBXO27*	4	0.30039	0.642342	0.192233
ARHGAP20	4	-0.23396	0.811833	0.090533	*SYNE2*	4	0.071275	0.642641	0.192031
ALG5	4	-0.22687	0.811833	0.090533	*SENP8*	4	0.25719	0.642641	0.192031
RASA3	4	-0.22143	0.811833	0.090533	*SLC6A13*	4	0.30382	0.642641	0.192031
SLC30A6	4	-0.21449	0.811833	0.090533	*SHC4*	3	0.23646	0.642747	0.19196
HTRA1	4	-0.20329	0.811833	0.090533	*OR2A2*	4	-0.43189	0.642912	0.191849
RSAD1	4	-0.2024	0.811833	0.090533	*HLCS*	4	-0.25512	0.643601	0.191383
DUSP9	4	-0.19818	0.811833	0.090533	*AGPAT3*	4	0.23973	0.643601	0.191383
NIPSNAP2	3	-0.19109	0.811833	0.090533	*HBE1*	4	0.20082	0.644641	0.190682
IMPAD1	4	-0.17988	0.811833	0.090533	*CMSS1*	3	0.31513	0.645165	0.190329
C5orf67	4	-0.16444	0.811833	0.090533	*IRF7*	4	-0.36322	0.645832	0.18988
C10orf25	4	-0.11064	0.811833	0.090533	*ANKAR*	4	0.05518	0.645832	0.18988
CTDSP2	4	-0.089184	0.811833	0.090533	*TTC39A*	3	-0.48051	0.645887	0.189844
ZNF391	4	-0.086286	0.811833	0.090533	*ZNF326*	3	-0.020682	0.645887	0.189844
THUMPD2	4	-0.012923	0.811833	0.090533	*KANSL3*	4	0.44118	0.646108	0.189695
MRPL20	3	-0.008725	0.811833	0.090533	*RABL3*	4	-0.30905	0.647169	0.188982
FAM120AOS	4	-5.15E-04	0.811833	0.090533	*PPCDC*	3	0.25674	0.647169	0.188982
CALHM1	4	0.032319	0.811833	0.090533	*CNIH2*	3	0.29198	0.647169	0.188982
GHRHR	4	0.032522	0.811833	0.090533	*CLDN12*	4	-0.072797	0.64774	0.1886
TGFBR3	4	0.068302	0.811833	0.090533	*JPH3*	2	0.27313	0.64852	0.188077
PTPRF	4	0.085113	0.811833	0.090533	*SEC24B*	4	0.29375	0.64852	0.188077
MAD2L2	3	0.091404	0.811833	0.090533	*CEBPB*	2	0.24618	0.648565	0.188046
SGIP1	4	0.09779	0.811833	0.090533	*ZNF664*	4	-0.001449	0.649403	0.187486
AKAP14	4	0.11042	0.811833	0.090533	*GTF2H4*	4	0.38177	0.649403	0.187486
SLC45A3	4	0.14291	0.811833	0.090533	*TEAD3*	3	-0.26807	0.649464	0.187445
C5orf24	3	0.15189	0.811833	0.090533	*COL1A1*	4	0.16057	0.649464	0.187445
SORT1	4	0.15904	0.811833	0.090533	*NCMAP*	4	0.16515	0.649464	0.187445
FGA	3	0.17458	0.811833	0.090533	*RPL24*	2	0.26873	0.649464	0.187445
CAP1	4	0.1846	0.811833	0.090533	*GTF3C3*	3	0.44432	0.649464	0.187445
STAM2	4	0.19091	0.811833	0.090533	*ST6GALNAC2*	4	0.30697	0.649809	0.187214
ADAM21	4	0.20919	0.811833	0.090533	*SVOPL*	2	-0.42521	0.649825	0.187203
OVGP1	4	0.21338	0.811833	0.090533	*SVOP*	4	-0.24046	0.650273	0.186904

HSD11B1	4	0.23816	0.811833	0.090533	*ZNF385A*	4	0.28984	0.650273	0.186904
DOCK7	4	0.24699	0.811833	0.090533	*GPR18*	4	-0.4761	0.650449	0.186787
LGI3	4	0.2539	0.811833	0.090533	*CSGALNACT*	4	-0.22307	0.650449	0.186787
SCGB2B2	4	0.27102	0.811833	0.090533	*DPYD*	2	-0.6576	0.650742	0.186591
TAF11	4	0.2804	0.811833	0.090533	*COL24A1*	3	-0.51567	0.650742	0.186591
KPNA3	4	0.28312	0.811833	0.090533	*PJA1*	2	-0.40652	0.650742	0.186591
HIF1AN	4	0.28875	0.811833	0.090533	*KRT39*	4	-0.36705	0.650742	0.186591
ABHD3	4	0.29593	0.811833	0.090533	*PHF1*	4	-0.36132	0.650742	0.186591
SRD5A1	4	0.30304	0.811833	0.090533	*RPS6KB1*	4	-0.32888	0.650742	0.186591
EHMT1	4	0.31158	0.811833	0.090533	*FZD1*	4	-0.30182	0.650742	0.186591
INTS3	3	0.31415	0.811833	0.090533	*PTPDC1*	4	-0.12963	0.650742	0.186591
MAB21L4	4	0.31816	0.811833	0.090533	*ZC3H6*	4	-0.061974	0.650742	0.186591
NBPF14	2	0.33778	0.811833	0.090533	*RCE1*	4	-0.034615	0.650742	0.186591
VWA3A	4	0.34351	0.811833	0.090533	*HGFAC*	4	0.033195	0.650742	0.186591
POU6F1	4	0.34923	0.811833	0.090533	*SMYD3*	4	0.041496	0.650742	0.186591
HDGFL2	3	0.34996	0.811833	0.090533	*FGD4*	3	0.12887	0.650742	0.186591
NKAP	4	0.37055	0.811833	0.090533	*SH3BP4*	4	0.13342	0.650742	0.186591
ZNF14	3	0.43875	0.811833	0.090533	*KRT13*	4	0.1577	0.650742	0.186591
RCC1	2	0.50069	0.811833	0.090533	*STON2*	4	0.17981	0.650742	0.186591
ZFX	4	0.50953	0.811833	0.090533	*PCOLCE*	4	0.18829	0.650742	0.186591
SLC35D1	1	0.51502	0.811833	0.090533	*MT1M*	2	0.19364	0.650742	0.186591
RCN1	2	0.51658	0.811833	0.090533	*GLYCTK*	4	0.2329	0.650742	0.186591
COPB1	4	0.54406	0.811833	0.090533	*TTC9C*	3	0.24212	0.650742	0.186591
C9orf116	2	0.57761	0.811833	0.090533	*SEZ6*	4	0.25095	0.650742	0.186591
TM7SF2	1	0.6183	0.811833	0.090533	*DBNL*	4	0.25751	0.650742	0.186591
RNF20	3	0.6694	0.811833	0.090533	*ZNF570*	4	0.26058	0.650742	0.186591
UHMK1	2	-0.37572	0.812664	0.090089	*P3H4*	3	0.29136	0.650742	0.186591
ZSWIM6	2	-0.85699	0.814163	0.089288	*SWSAP1*	4	0.30893	0.650742	0.186591
SIGLEC6	3	-0.75765	0.814163	0.089288	*DPP7*	3	0.32321	0.650742	0.186591
PARP12	1	-0.74975	0.814163	0.089288	*B4GALNT4*	4	0.34368	0.650742	0.186591
DNAJC16	3	-0.66747	0.814163	0.089288	*ZNF581*	4	0.34472	0.650742	0.186591
PHF12	4	-0.63906	0.814163	0.089288	*GAN*	4	0.38532	0.650742	0.186591
SERPINB9	3	-0.57474	0.814163	0.089288	*FMNL2*	2	0.4029	0.650742	0.186591
SCAF1	1	-0.53229	0.814163	0.089288	*TPRKB*	4	0.41053	0.650742	0.186591
SULT1C2	2	-0.53214	0.814163	0.089288	*ZNF676*	1	0.5051	0.650742	0.186591
GRSF1	4	-0.52445	0.814163	0.089288	*IRF1*	1	0.51053	0.650742	0.186591
AKAP11	4	-0.47236	0.814163	0.089288	*DLGAP5*	3	0.57147	0.650742	0.186591
C16orf87	3	-0.43485	0.814163	0.089288	*ELF4*	4	0.067633	0.651312	0.186211
PKP2	4	-0.43362	0.814163	0.089288	*CORIN*	4	-0.15505	0.65148	0.186099
IKZF5	4	-0.41817	0.814163	0.089288	*LRRC17*	4	-0.31802	0.651609	0.186013
SLIRP	4	-0.41114	0.814163	0.089288	*NOL3*	4	-0.25924	0.651609	0.186013
ELL2	4	-0.38978	0.814163	0.089288	*UNK*	4	-0.22632	0.651609	0.186013
LGSN	4	-0.36459	0.814163	0.089288	*ARHGAP22*	3	-0.08111	0.651609	0.186013
ANK2	4	-0.36198	0.814163	0.089288	*PLK3*	3	0.13502	0.651609	0.186013
SPTBN1	2	-0.35239	0.814163	0.089288	*XDH*	4	0.17772	0.651609	0.186013
UBD	4	-0.34878	0.814163	0.089288	*FRAT2*	4	0.22831	0.651609	0.186013
FN3K	4	-0.33129	0.814163	0.089288	*CBY1*	4	0.25739	0.651609	0.186013
KIF7	4	-0.31937	0.814163	0.089288	*RPP14*	4	0.26976	0.651609	0.186013
KBTBD2	4	-0.31308	0.814163	0.089288	*CNPY4*	3	0.34895	0.651609	0.186013
LRIF1	4	-0.30746	0.814163	0.089288	*GEMIN5*	3	0.52886	0.651609	0.186013
NPPA	4	-0.30416	0.814163	0.089288	*MAB21L2*	4	0.32083	0.651663	0.185977
LYPLAL1	4	-0.30052	0.814163	0.089288	*OSTC*	3	0.56404	0.651887	0.185827
SYTL4	4	-0.29803	0.814163	0.089288	*AXIN2*	3	0.059727	0.652198	0.185621
CSGALNACT1	4	-0.29753	0.814163	0.089288	*LONP2*	3	-0.5931	0.652639	0.185327
JUP	4	-0.29585	0.814163	0.089288	*DDX21*	3	-0.58663	0.652639	0.185327
PFDN4	3	-0.28812	0.814163	0.089288	*PON2*	3	-0.52264	0.652639	0.185327
EML4	4	-0.28258	0.814163	0.089288	*ARMC3*	3	-0.43483	0.652639	0.185327
SFTPD	4	-0.27606	0.814163	0.089288	*SPTBN5*	2	-0.40726	0.652639	0.185327

ZNF765	4	-0.2754	0.814163	0.089288	*AIFM3*	4	-0.36725	0.652639	0.185327
SLC11A2	4	-0.27052	0.814163	0.089288	*CYP2A6*	3	-0.36466	0.652639	0.185327
ZNF610	4	-0.26216	0.814163	0.089288	*SMIM15*	4	-0.27795	0.652639	0.185327
THPO	4	-0.25893	0.814163	0.089288	*ZNF691*	4	-0.25963	0.652639	0.185327
KMT2E	4	-0.25575	0.814163	0.089288	*DAG1*	4	-0.25944	0.652639	0.185327
HSD11B2	4	-0.25398	0.814163	0.089288	*SOX10*	3	-0.23172	0.652639	0.185327
PPP2CB	4	-0.24938	0.814163	0.089288	*ZBTB40*	3	-0.2054	0.652639	0.185327
IRX5	3	-0.24401	0.814163	0.089288	*ZNF287*	2	-0.18556	0.652639	0.185327
SIRPG	4	-0.24205	0.814163	0.089288	*ZNF136*	4	-0.1748	0.652639	0.185327
RAD21L1	4	-0.22564	0.814163	0.089288	*MS4A3*	4	-0.1642	0.652639	0.185327
TBC1D3E	2	-0.20329	0.814163	0.089288	*ASCC3*	4	-0.14388	0.652639	0.185327
ADH6	4	-0.19769	0.814163	0.089288	*PDCL2*	4	0.13397	0.652639	0.185327
EHD4	4	-0.18441	0.814163	0.089288	*DNASE2B*	4	0.14582	0.652639	0.185327
RMC1	4	-0.17696	0.814163	0.089288	*ZC3H18*	4	0.1906	0.652639	0.185327
B4GALT2	4	-0.1597	0.814163	0.089288	*SNX13*	4	0.19294	0.652639	0.185327
ADM5	3	-0.13601	0.814163	0.089288	*KLHL11*	4	0.2409	0.652639	0.185327
SIPA1	4	-0.11983	0.814163	0.089288	*EGFL7*	4	0.2489	0.652639	0.185327
PLEKHH1	3	-0.11011	0.814163	0.089288	*ZZZ3*	4	0.31445	0.652639	0.185327
SPATA45	4	-0.0889	0.814163	0.089288	*ZNF195*	3	0.31702	0.652639	0.185327
TMEM35B	4	-0.084647	0.814163	0.089288	*RFXANK*	3	0.35279	0.652639	0.185327
VAX1	3	-0.078253	0.814163	0.089288	*TUBG1*	4	0.44252	0.652639	0.185327
TRIP10	4	0.038091	0.814163	0.089288	*WDR87*	2	0.44775	0.652639	0.185327
WDR7	4	0.041359	0.814163	0.089288	*STUB1*	3	0.52353	0.652639	0.185327
CDC20B	3	0.041923	0.814163	0.089288	*RPL10*	2	0.81538	0.652639	0.185327
BRWD1	4	0.048214	0.814163	0.089288	*KRIT1*	4	-0.41334	0.653668	0.184643
C5orf66	4	0.074565	0.814163	0.089288	*BACE1*	3	-0.1932	0.653668	0.184643
CAMKK2	3	0.089347	0.814163	0.089288	*HSF4*	2	-0.56127	0.65381	0.184548
ZNF225	4	0.093546	0.814163	0.089288	*EML4*	4	-0.34499	0.65381	0.184548
SPINK7	2	0.097334	0.814163	0.089288	*SSC5D*	4	0.17712	0.654014	0.184413
MOB2	4	0.11941	0.814163	0.089288	*CDHR2*	3	0.26913	0.654014	0.184413
CALCOCO1	3	0.12335	0.814163	0.089288	*SOX13*	4	0.30705	0.654014	0.184413
CHIA	4	0.13895	0.814163	0.089288	*TTC24*	4	0.31278	0.654014	0.184413
UNC50	4	0.13974	0.814163	0.089288	*CSE1L*	4	0.32139	0.654014	0.184413
OXNAD1	4	0.15006	0.814163	0.089288	*TSHZ3*	4	0.12723	0.654329	0.184204
FCAMR	4	0.15185	0.814163	0.089288	*RNLS*	4	0.17548	0.654738	0.183932
RELT	4	0.16313	0.814163	0.089288	*ZNF41*	4	-0.55496	0.654781	0.183904
GPC4	4	0.18245	0.814163	0.089288	*PCCA*	4	-0.3619	0.654781	0.183904
TMEM91	4	0.18919	0.814163	0.089288	*TRPC4*	4	-0.26365	0.654781	0.183904
KIAA0040	4	0.19941	0.814163	0.089288	*C4BPA*	4	-0.21331	0.654926	0.183808
GTF2IRD1	4	0.20189	0.814163	0.089288	*COL27A1*	4	-0.33727	0.655316	0.183549
CXCL6	3	0.22505	0.814163	0.089288	*GALR3*	3	0.14665	0.655597	0.183363
CFL1	4	0.25391	0.814163	0.089288	*TBC1D13*	3	0.45517	0.655597	0.183363
TBRG1	4	0.25563	0.814163	0.089288	*TRERF1*	3	-0.094015	0.655615	0.183351
TPM3	3	0.25574	0.814163	0.089288	*GUCY1A2*	4	0.13087	0.655615	0.183351
IL17RA	2	0.25575	0.814163	0.089288	*FBXL18*	3	0.2879	0.655615	0.183351
TMEM115	4	0.25974	0.814163	0.089288	*PSMB6*	3	0.69521	0.655615	0.183351
SLC27A3	4	0.26398	0.814163	0.089288	*LEFTY1*	3	-0.62876	0.655732	0.183274
FANK1	4	0.27082	0.814163	0.089288	*PSPC1*	4	0.22894	0.655984	0.183107
FOXD4L1	2	0.28517	0.814163	0.089288	*CCDC88C*	3	0.4648	0.655984	0.183107
PPP4R2	3	0.28605	0.814163	0.089288	*NOLC1*	3	0.52754	0.656428	0.182813
RBM6	4	0.29172	0.814163	0.089288	*HERC3*	4	-0.2853	0.656461	0.182791
PCGF6	4	0.29219	0.814163	0.089288	*IL12RB2*	3	-0.43955	0.656708	0.182628
RFK	4	0.30557	0.814163	0.089288	*CHEK1*	3	0.49706	0.657199	0.182303
TTC24	4	0.3074	0.814163	0.089288	*SLC38A2*	4	-0.59319	0.657379	0.182184
HESX1	4	0.31082	0.814163	0.089288	*IL12A*	4	-0.32281	0.657379	0.182184
THEGL	4	0.3255	0.814163	0.089288	*IL20RA*	4	-0.18305	0.657379	0.182184
PEA15	3	0.34285	0.814163	0.089288	*KLHL22*	4	-0.40587	0.657714	0.181963
CHRNA10	3	0.34935	0.814163	0.089288	*CHRM4*	4	-0.038474	0.657714	0.181963

GSK3A	3	0.35534	0.814163	0.089288	*ELL2*	4	0.092268	0.657714	0.181963
AAGAB	2	0.35582	0.814163	0.089288	*TMEM108*	4	0.24313	0.657714	0.181963
RAC2	4	0.3583	0.814163	0.089288	*VAC14*	4	0.24847	0.657714	0.181963
TMEM154	3	0.36263	0.814163	0.089288	*TOP2A*	4	0.27394	0.657714	0.181963
CYP26A1	4	0.37025	0.814163	0.089288	*IFNA1*	1	0.46683	0.657714	0.181963
GPKOW	4	0.39542	0.814163	0.089288	*GPX8*	4	-0.40706	0.658498	0.181446
PPP1R16B	3	0.40066	0.814163	0.089288	*GALR2*	2	-0.37904	0.658498	0.181446
ZNF23	2	0.40173	0.814163	0.089288	*RIPOR2*	3	-0.37731	0.658498	0.181446
C2CD2L	3	0.41325	0.814163	0.089288	*ANXA5*	4	-0.34186	0.658498	0.181446
TSGA10	3	0.41507	0.814163	0.089288	*ATP6V1FNB*	4	-0.25111	0.658498	0.181446
TDP1	3	0.43262	0.814163	0.089288	*HS1BP3*	4	-0.21279	0.658498	0.181446
DBF4	2	0.43314	0.814163	0.089288	*ADNP*	4	0.20841	0.658498	0.181446
GRIK5	3	0.47992	0.814163	0.089288	*PKMYT1*	4	0.37769	0.658498	0.181446
IREB2	4	0.49719	0.814163	0.089288	*DHRS4*	2	-0.46084	0.658622	0.181364
MVP	3	0.50567	0.814163	0.089288	*DHX40*	3	-0.73227	0.659797	0.180589
ZC3H15	2	0.60309	0.814163	0.089288	*ZNF443*	4	-0.51605	0.659797	0.180589
KAT5	3	0.62979	0.814163	0.089288	*SBSN*	3	-0.5096	0.659797	0.180589
CIAO2B	3	0.89296	0.814163	0.089288	*STRBP*	3	-0.49236	0.659797	0.180589
LSM6	4	-0.24743	0.815385	0.088637	*GPRASP1*	3	-0.43319	0.659797	0.180589
RPL19	2	-0.7893	0.815488	0.088582	*BAHD1*	4	-0.3861	0.659797	0.180589
TJP2	3	-0.6681	0.815488	0.088582	*CBX5*	4	-0.3759	0.659797	0.180589
TTC32	1	-0.62941	0.815488	0.088582	*EPB41L4B*	4	-0.24588	0.659797	0.180589
CTBS	4	-0.5453	0.815488	0.088582	*HIBADH*	4	-0.22889	0.659797	0.180589
H2BC5	4	-0.52501	0.815488	0.088582	*TAS2R42*	4	-0.19279	0.659797	0.180589
SOCS4	4	-0.50377	0.815488	0.088582	*AZIN2*	4	-0.092533	0.659797	0.180589
RSPH3	3	-0.50254	0.815488	0.088582	*ABLIM3*	4	-0.068247	0.659797	0.180589
WDR72	3	-0.49726	0.815488	0.088582	*COMMD2*	3	-0.068142	0.659797	0.180589
OC10192843	4	-0.49018	0.815488	0.088582	*HESX1*	4	-0.05657	0.659797	0.180589
BPIFB1	3	-0.48562	0.815488	0.088582	*GRIFIN*	3	-0.032604	0.659797	0.180589
CPTP	3	-0.47076	0.815488	0.088582	*ARRDC3*	4	9.03E-06	0.659797	0.180589
WNT2B	3	-0.47049	0.815488	0.088582	*GTF3C6*	4	0.032059	0.659797	0.180589
LRP8	4	-0.39246	0.815488	0.088582	*MOB3C*	4	0.046076	0.659797	0.180589
STXBP1	4	-0.38075	0.815488	0.088582	*NABP2*	3	0.082417	0.659797	0.180589
TBC1D26	3	-0.35685	0.815488	0.088582	*AREL1*	4	0.084017	0.659797	0.180589
GPX8	4	-0.35226	0.815488	0.088582	*NDUFA5*	3	0.11932	0.659797	0.180589
TGDS	4	-0.34903	0.815488	0.088582	*SMC4*	4	0.14714	0.659797	0.180589
MINK1	3	-0.34795	0.815488	0.088582	*SLC13A2*	4	0.15948	0.659797	0.180589
TRPT1	4	-0.34485	0.815488	0.088582	*ZNF418*	4	0.1746	0.659797	0.180589
MAP3K14	4	-0.3375	0.815488	0.088582	*CYP4F8*	4	0.19617	0.659797	0.180589
CABLES2	4	-0.31867	0.815488	0.088582	*RAB12*	4	0.21661	0.659797	0.180589
POLM	4	-0.3148	0.815488	0.088582	*PGM2L1*	3	0.2246	0.659797	0.180589
FBH1	4	-0.29564	0.815488	0.088582	*KRT19*	3	0.28146	0.659797	0.180589
KIAA0513	4	-0.29381	0.815488	0.088582	*IFITM10*	4	0.3007	0.659797	0.180589
MEP1A	4	-0.27924	0.815488	0.088582	*TFB1M*	3	0.30756	0.659797	0.180589
GPRC6A	2	-0.27629	0.815488	0.088582	*TIMP2*	3	0.30972	0.659797	0.180589
CTTN	4	-0.27488	0.815488	0.088582	*TFCP2*	4	0.33709	0.659797	0.180589
ZNF280A	4	-0.27486	0.815488	0.088582	*ZNRD1*	4	0.46942	0.659797	0.180589
SHROOM2	3	-0.25391	0.815488	0.088582	*SNU13*	3	0.49077	0.659797	0.180589
ELMOD2	4	-0.24176	0.815488	0.088582	*URB1*	3	0.54627	0.659797	0.180589
FUT3	4	-0.22781	0.815488	0.088582	*AURKA*	1	1.0217	0.659797	0.180589
INAVA	4	-0.21872	0.815488	0.088582	*ZCCHC18*	3	-0.35237	0.660027	0.180438
ISLR2	4	-0.20504	0.815488	0.088582	*GAS2L1*	3	0.17891	0.660027	0.180438
TMEM253	3	-0.19988	0.815488	0.088582	*CNST*	4	0.27467	0.660027	0.180438
NEK6	4	-0.1895	0.815488	0.088582	*HMX2*	4	0.25229	0.660242	0.180297
KIFC1	4	-0.17489	0.815488	0.088582	*BCAS2*	4	0.1906	0.660604	0.180059
PRSS57	3	-0.12325	0.815488	0.088582	*MAATS1*	4	0.16347	0.660766	0.179953
SLC26A1	4	-0.12238	0.815488	0.088582	*SPATA2*	3	0.37332	0.660766	0.179953
SUZ12	3	-0.11701	0.815488	0.088582	*FAHD2A*	3	0.40363	0.660961	0.179824

PLA2R1	4	-0.087687	0.815488	0.088582	*NQO2*	4	0.23696	0.661117	0.179722
MAP2K3	3	-0.081729	0.815488	0.088582	*ATP5F1C*	4	0.27166	0.661117	0.179722
ZBTB44	4	-0.061452	0.815488	0.088582	*COLGALT1*	3	0.4222	0.661117	0.179722
LDHAL6A	3	-0.03789	0.815488	0.088582	*GFOD1*	3	-0.066417	0.661473	0.179488
PNPLA4	4	-0.014078	0.815488	0.088582	*RCOR2*	4	0.17225	0.661473	0.179488
NRM	3	0.016184	0.815488	0.088582	*ARL5C*	2	0.30805	0.661473	0.179488
HPS1	3	0.0214	0.815488	0.088582	*H2BC3*	3	-0.5958	0.661829	0.179254
GLMP	3	0.06444	0.815488	0.088582	*CD226*	3	-0.43667	0.661829	0.179254
PMPCB	4	0.07761	0.815488	0.088582	*GAK*	4	0.26071	0.661829	0.179254
ATF6B	4	0.083004	0.815488	0.088582	*SLC12A2*	2	-0.56796	0.661997	0.179144
PXT1	4	0.092265	0.815488	0.088582	*DLG4*	3	-0.63989	0.662362	0.178905
CDK16	4	0.11932	0.815488	0.088582	*ZNF573*	4	-0.55022	0.662362	0.178905
GCNT2	4	0.12719	0.815488	0.088582	*PJA2*	4	-0.46363	0.662362	0.178905
CREB3	4	0.1472	0.815488	0.088582	*CARD14*	4	-0.38536	0.662362	0.178905
LAMB3	4	0.16457	0.815488	0.088582	*EPO*	4	-0.36729	0.662362	0.178905
RTN4RL2	4	0.17243	0.815488	0.088582	*GALNT14*	4	-0.3266	0.662362	0.178905
ZNF669	4	0.18672	0.815488	0.088582	*SIT1*	4	-0.26	0.662362	0.178905
LIMK2	4	0.226	0.815488	0.088582	*IDS*	4	-0.25733	0.662362	0.178905
YPEL4	4	0.23137	0.815488	0.088582	*BLZF1*	4	-0.2509	0.662362	0.178905
TMEM259	4	0.24141	0.815488	0.088582	*ACOT11*	4	-0.20589	0.662362	0.178905
DOHH	4	0.2545	0.815488	0.088582	*IL13*	2	-0.18157	0.662362	0.178905
PLAU	4	0.25689	0.815488	0.088582	*BACE2*	4	-0.13686	0.662362	0.178905
CDPF1	3	0.25842	0.815488	0.088582	*UBE2K*	3	-0.040937	0.662362	0.178905
NCDN	4	0.26974	0.815488	0.088582	*RSPO4*	4	-0.029204	0.662362	0.178905
CTNNB1	3	0.27373	0.815488	0.088582	*HROB*	3	-0.025043	0.662362	0.178905
ZNF140	4	0.29107	0.815488	0.088582	*H3-4*	3	0.019618	0.662362	0.178905
CCDC153	4	0.29224	0.815488	0.088582	*LAMC1*	4	0.027618	0.662362	0.178905
HMX3	3	0.31618	0.815488	0.088582	*RIMS1*	4	0.037074	0.662362	0.178905
ATG4D	2	0.34548	0.815488	0.088582	*COL13A1*	4	0.042548	0.662362	0.178905
PSMD2	3	0.36517	0.815488	0.088582	*CCN6*	4	0.11527	0.662362	0.178905
TUBAL3	3	0.37436	0.815488	0.088582	*ADAM11*	4	0.1635	0.662362	0.178905
TAF1C	4	0.37837	0.815488	0.088582	*ANKS6*	4	0.20523	0.662362	0.178905
OBSCN	3	0.39255	0.815488	0.088582	*ELP3*	4	0.20602	0.662362	0.178905
TMPPE	3	0.39437	0.815488	0.088582	*FGF8*	4	0.23055	0.662362	0.178905
MYCBP2	4	0.40915	0.815488	0.088582	*HOXA13*	4	0.23785	0.662362	0.178905
MUC3A	3	0.4216	0.815488	0.088582	*ASB6*	4	0.24874	0.662362	0.178905
TOR1AIP1	3	0.42163	0.815488	0.088582	*ARHGAP40*	4	0.25832	0.662362	0.178905
CNNM2	4	0.43091	0.815488	0.088582	*DDOST*	4	0.29389	0.662362	0.178905
CNPY2	3	0.45526	0.815488	0.088582	*CTC1*	4	0.3022	0.662362	0.178905
CAB39L	2	0.45578	0.815488	0.088582	*IPO9*	4	0.30359	0.662362	0.178905
H2BC13	2	0.48294	0.815488	0.088582	*MNAT1*	3	0.3186	0.662362	0.178905
KIF18A	4	0.52532	0.815488	0.088582	*FAM193B*	4	0.40278	0.662362	0.178905
C19orf44	1	0.53295	0.815488	0.088582	*MTFMT*	4	0.44654	0.662362	0.178905
PFDN2	4	0.55996	0.815488	0.088582	*RABGGTA*	3	0.83565	0.662362	0.178905
JAZF1	1	0.631	0.815488	0.088582	*VIRMA*	4	-0.10496	0.663008	0.178481
PCBP1	1	0.6573	0.815488	0.088582	*OTUB1*	4	0.33784	0.663427	0.178207
COPE	3	0.79009	0.815488	0.088582	*MAP2K2*	2	0.33181	0.663765	0.177986
GJA5	3	0.003926	0.81691	0.087826	*SLC35D3*	4	-0.4611	0.663952	0.177863
GPR182	3	0.30939	0.81691	0.087826	*KMT2E*	4	-0.28364	0.663952	0.177863
KBTBD11	3	0.31475	0.81693	0.087815	*ANXA13*	4	-0.19154	0.663952	0.177863
NOP16	4	0.37799	0.81693	0.087815	*ZRANB1*	4	-0.28269	0.664624	0.177424
LRRIQ3	3	0.39562	0.81693	0.087815	*MBLAC2*	4	-0.34937	0.66496	0.177204
IRGC	4	-0.026895	0.816956	0.087801	*SLC16A6*	4	-0.36182	0.665677	0.176737
AGAP2	4	0.27852	0.816956	0.087801	*CMTM2*	2	-0.34129	0.665678	0.176736
DISP3	2	-0.55089	0.817214	0.087664	*NINL*	2	-0.45109	0.665784	0.176667
CNOT8	3	-0.45135	0.817214	0.087664	*C20orf202*	3	-0.42203	0.665784	0.176667
AKR7A2	2	-0.38456	0.817214	0.087664	*DHRS7*	4	-0.39735	0.665784	0.176667
LRP4	3	-0.36583	0.817214	0.087664	*ME2*	4	-0.30725	0.665784	0.176667

AOAH	4	-0.34229	0.817214	0.087664	*ADAM8*	3	-0.28634	0.665784	0.176667
ZNF41	4	-0.32859	0.817214	0.087664	*COL5A3*	4	-0.26764	0.665784	0.176667
FBXO43	4	-0.32621	0.817214	0.087664	*TBC1D8*	4	-0.25716	0.665784	0.176667
LCMT2	4	-0.32566	0.817214	0.087664	*GYPC*	4	-0.2467	0.665784	0.176667
HOXB13	4	-0.2883	0.817214	0.087664	*CPLX1*	3	-0.16684	0.665784	0.176667
MTUS1	4	-0.2527	0.817214	0.087664	*KBTBD3*	4	-0.1304	0.665784	0.176667
TRPC5OS	4	-0.23189	0.817214	0.087664	*CEP97*	3	0.0185	0.665784	0.176667
AHI1	3	-0.21803	0.817214	0.087664	*ZDHHC24*	4	0.075921	0.665784	0.176667
TMEM155	3	-0.21529	0.817214	0.087664	*TIMD4*	4	0.10915	0.665784	0.176667
LYSMD3	3	-0.19926	0.817214	0.087664	*UAP1L1*	4	0.1817	0.665784	0.176667
LETM1	3	-0.19662	0.817214	0.087664	*SEPTIN9*	3	0.2368	0.665784	0.176667
SLC25A30	3	-0.15136	0.817214	0.087664	*F11R*	4	0.24093	0.665784	0.176667
ZNF227	3	-0.14064	0.817214	0.087664	*GJC3*	4	0.25519	0.665784	0.176667
DDX49	3	-0.13822	0.817214	0.087664	*RASGRP3*	3	0.33642	0.665784	0.176667
ACOT11	4	-0.10217	0.817214	0.087664	*ZNF600*	2	0.35364	0.665784	0.176667
TSACC	3	-0.10198	0.817214	0.087664	*VPS28*	4	0.36427	0.665784	0.176667
NREP	3	-0.071192	0.817214	0.087664	*KATNB1*	2	0.37748	0.665784	0.176667
RNF187	4	0.027614	0.817214	0.087664	*WDR82*	3	0.38834	0.665784	0.176667
PSMB7	4	0.034609	0.817214	0.087664	*PSMD4*	4	0.51591	0.665784	0.176667
AP5B1	3	0.079356	0.817214	0.087664	*XRCC5*	4	0.59778	0.665784	0.176667
PTPN23	4	0.090892	0.817214	0.087664	*POLR3F*	4	-0.079792	0.665853	0.176622
ANGPT4	4	0.21194	0.817214	0.087664	*SAMD12*	4	0.12856	0.665853	0.176622
C5AR1	4	0.22131	0.817214	0.087664	*MICU2*	3	0.19166	0.665853	0.176622
STAG2	4	0.26264	0.817214	0.087664	*SCRN2*	4	0.23307	0.665853	0.176622
FKBP15	4	0.27909	0.817214	0.087664	*SH2B1*	3	0.30418	0.665853	0.176622
CCDC28B	4	0.31173	0.817214	0.087664	*CAPRIN2*	3	0.33533	0.665853	0.176622
TMEM14B	3	0.31853	0.817214	0.087664	*NPAS2*	3	0.35148	0.665853	0.176622
DNAJC11	3	0.33671	0.817214	0.087664	*EOGT*	3	0.42194	0.665853	0.176622
DNAJB2	4	0.3378	0.817214	0.087664	*CCZ1*	1	0.46389	0.665853	0.176622
TEAD2	4	0.34205	0.817214	0.087664	*EXO1*	3	0.4967	0.665853	0.176622
ENTPD8	2	0.42688	0.817214	0.087664	*MRPL3*	2	0.49836	0.665853	0.176622
ATP6V1B2	3	0.42783	0.817214	0.087664	*POLR3D*	3	-0.63537	0.665902	0.176589
GTPBP4	4	0.44302	0.817214	0.087664	*TTC13*	4	-0.57063	0.665902	0.176589
INTS8	3	0.61174	0.817214	0.087664	*STAP2*	3	-0.35394	0.665902	0.176589
FRMD4A	2	0.62482	0.817214	0.087664	*PRKCG*	4	-0.31591	0.665902	0.176589
ARF6	2	0.63671	0.817214	0.087664	*WASHC3*	3	-0.10971	0.665902	0.176589
SLC40A1	4	-0.3427	0.817713	0.087399	*SYCE1*	4	0.058241	0.665902	0.176589
SLCO1B1	4	0.010331	0.817713	0.087399	*PCYOX1*	4	0.069064	0.665902	0.176589
IER2	4	0.23721	0.817988	0.087253	*PDS5A*	3	0.09299	0.665902	0.176589
MTHFD1L	4	0.26949	0.817988	0.087253	*PPP1CA*	4	0.12039	0.665902	0.176589
TRPC7	4	-0.5599	0.818551	0.086955	*FAM83E*	4	0.17243	0.665902	0.176589
SMG9	4	-0.45005	0.818551	0.086955	*SCAF11*	4	0.17303	0.665902	0.176589
EMID1	2	-0.36788	0.818551	0.086955	*RASGRP2*	4	0.21023	0.665902	0.176589
FZD5	4	-0.20513	0.818551	0.086955	*TENT5C*	4	0.25813	0.665902	0.176589
STK3	3	0.18527	0.818551	0.086955	*GNL3L*	4	0.31141	0.665902	0.176589
HERC1	4	0.20939	0.818551	0.086955	*KSR1*	4	0.32659	0.665902	0.176589
CAPSL	4	0.40526	0.818551	0.086955	*EIF4E2*	3	0.44958	0.665902	0.176589
AHNAK2	3	0.45076	0.818551	0.086955	*MZT1*	2	0.55967	0.665902	0.176589
HHLA3	4	-0.17484	0.818566	0.086946	*ZNF516*	2	-0.47398	0.666378	0.176279
UBE2R2	3	0.23186	0.818566	0.086946	*MKNK2*	4	0.30817	0.666378	0.176279
IGFBP7	3	-0.32386	0.818934	0.086751	*POLR2G*	3	0.48299	0.666378	0.176279
NUMB	4	0.21117	0.818968	0.086733	*VPS36*	3	0.46122	0.666478	0.176214
NDUFS1	4	0.31586	0.818968	0.086733	*TMEM215*	2	-0.35319	0.666648	0.176103
ZNF75D	3	-0.44184	0.819695	0.086348	*CDKN2B*	2	-0.35137	0.666876	0.175955
MOB3B	3	-0.4098	0.819695	0.086348	*EZH1*	4	-0.030115	0.666876	0.175955
ZNF527	2	0.56438	0.820596	0.085871	*SPTBN4*	2	0.14324	0.666876	0.175955
GTF2H5	4	-0.53764	0.821658	0.085309	*LIN54*	4	0.19549	0.666876	0.175955
ATP13A4	4	-0.46517	0.821658	0.085309	*SAMD7*	2	0.37184	0.666876	0.175955

YWHAB	4	-0.39422	0.821658	0.085309	*SCAF1*	1	-0.54978	0.667241	0.175717
SLC2A1	4	-0.24574	0.821658	0.085309	*CCNQ*	4	-0.029628	0.6676	0.175484
KLK4	4	-0.060019	0.821658	0.085309	*ZFP64*	4	0.1045	0.6676	0.175484
RGS20	3	0.001236	0.821658	0.085309	*SPAG8*	4	0.10506	0.6676	0.175484
ZNF776	3	0.16351	0.821658	0.085309	*DEPDC7*	4	0.10753	0.6676	0.175484
TXNDC17	3	0.37001	0.821658	0.085309	*PHF14*	4	0.098659	0.667824	0.175338
ATP6V0D1	3	0.89034	0.821658	0.085309	*KRBA2*	4	0.14169	0.667824	0.175338
CHD1	4	-0.27835	0.821794	0.085237	*RBM6*	4	-0.03167	0.669811	0.174048
MOB4	1	0.69936	0.821796	0.085236	*ARHGAP24*	4	0.14537	0.670485	0.173611
CYP4Z1	2	0.31504	0.822428	0.084902	*CLTRN*	4	-0.28831	0.670804	0.173404
RHBDD2	4	0.1283	0.823795	0.084181	*TASOR*	4	-0.36292	0.671079	0.173226
SERBP1	2	0.43627	0.824975	0.083559	*SNAPC2*	2	0.29969	0.671079	0.173226
ENO1	3	-0.79282	0.825149	0.083468	*PRXL2B*	3	-0.29065	0.671345	0.173055
RAB40B	3	-0.49425	0.825149	0.083468	*CASKIN1*	4	-0.37131	0.672159	0.172528
TRMT5	4	-0.39388	0.825149	0.083468	*TEF*	1	-0.55178	0.672238	0.172477
MFSD5	4	-0.26264	0.825149	0.083468	*ATXN3*	3	-0.5281	0.672238	0.172477
GSTK1	4	-0.25298	0.825149	0.083468	*SLC16A1*	4	-0.30395	0.67237	0.172392
LRRC8E	4	-0.18211	0.825149	0.083468	*RORA*	3	0.11749	0.67237	0.172392
ABCC6	4	-0.032352	0.825149	0.083468	*PPP2R2D*	3	-0.37176	0.672678	0.172193
LINC02694	4	0.097869	0.825149	0.083468	*DENND1C*	3	-0.002594	0.672678	0.172193
DLX6	4	0.23016	0.825149	0.083468	*H2AX*	4	0.088664	0.672766	0.172136
SERPIND1	4	0.2939	0.825149	0.083468	*RPL12*	3	-0.64823	0.672873	0.172067
CLIC5	2	-0.43047	0.825627	0.083216	*C1orf194*	3	-0.40341	0.672873	0.172067
CFAP47	4	-0.12607	0.826079	0.082978	*DOK3*	4	-0.16333	0.672873	0.172067
RASD2	4	-0.39851	0.826249	0.082889	*TRMT10A*	4	-0.066908	0.672873	0.172067
C2CD4A	2	-0.29286	0.826249	0.082889	*TMBIM1*	4	-0.021678	0.672873	0.172067
WBP4	3	-0.061937	0.826383	0.082819	*ADAMTS12*	4	-0.01795	0.672873	0.172067
FIGNL2	3	0.35965	0.826669	0.082668	*RTN4*	4	0.037401	0.672873	0.172067
BICDL1	3	0.44144	0.826669	0.082668	*LCORL*	4	0.09295	0.672873	0.172067
RBBP4	3	0.60365	0.826669	0.082668	*WDR88*	3	0.10162	0.672873	0.172067
VPS36	3	0.67542	0.826669	0.082668	*DNM1*	4	0.18567	0.672873	0.172067
ITPRID2	3	-0.009441	0.826996	0.082497	*VWA5A*	4	0.32148	0.672873	0.172067
PYCR1	3	-0.26024	0.827781	0.082085	*WSB1*	4	0.34399	0.672873	0.172067
TIGD5	2	-0.47229	0.827802	0.082073	*TMEM154*	3	0.36623	0.672873	0.172067
TRIM36	4	-0.47041	0.827802	0.082073	*YIPF2*	4	0.43241	0.673154	0.171886
SAMD12	4	-0.46257	0.827802	0.082073	*HOXD11*	4	-0.34261	0.674138	0.171251
SSPN	3	-0.45672	0.827802	0.082073	*ST3GAL6*	4	-0.16412	0.674138	0.171251
GLRA3	3	-0.45296	0.827802	0.082073	*HSPA14*	4	-0.024537	0.674138	0.171251
EXOG	4	-0.43663	0.827802	0.082073	*CISH*	4	0.051939	0.674138	0.171251
CITED4	3	-0.43579	0.827802	0.082073	*ACP2*	4	0.1103	0.674138	0.171251
CMC4	3	-0.43305	0.827802	0.082073	*BMPR2*	4	0.16747	0.674138	0.171251
SQSTM1	3	-0.43161	0.827802	0.082073	*PEX2*	4	0.24497	0.674138	0.171251
ATAD3B	2	-0.41901	0.827802	0.082073	*WWOX*	3	0.26586	0.674138	0.171251
ST6GALNAC5	4	-0.4176	0.827802	0.082073	*ARSD*	3	0.30519	0.674138	0.171251
PDE2A	4	-0.39385	0.827802	0.082073	*PDZD7*	4	0.3342	0.674138	0.171251
CEP120	4	-0.38862	0.827802	0.082073	*TFIP11*	4	0.36419	0.674138	0.171251
DSTN	1	-0.38467	0.827802	0.082073	*WAC*	4	-0.59969	0.676445	0.169768
USP25	4	-0.38115	0.827802	0.082073	*LOC339862*	3	-0.54475	0.676445	0.169768
NAT14	4	-0.37	0.827802	0.082073	*FHOD3*	4	-0.4248	0.676445	0.169768
CCBE1	4	-0.34032	0.827802	0.082073	*ABHD6*	4	-0.41612	0.676445	0.169768
CPA4	3	-0.33962	0.827802	0.082073	*SECISBP2L*	3	-0.40154	0.676445	0.169768
CNOT11	4	-0.33711	0.827802	0.082073	*PI4K2B*	4	-0.34224	0.676445	0.169768
UROC1	4	-0.32853	0.827802	0.082073	*ZNF805*	4	-0.25098	0.676445	0.169768
DPPA2	3	-0.32663	0.827802	0.082073	*CFHR4*	4	-0.24594	0.676445	0.169768
MISP	3	-0.31517	0.827802	0.082073	*PISD*	4	-0.16408	0.676445	0.169768
TSSC4	4	-0.31483	0.827802	0.082073	*JAKMIP1*	4	-0.13146	0.676445	0.169768
SLC25A44	4	-0.30663	0.827802	0.082073	*SNX3*	4	-0.038397	0.676445	0.169768
TBL2	4	-0.27737	0.827802	0.082073	*AMN1*	4	0.005999	0.676445	0.169768

SNX12	4	-0.27602	0.827802	0.082073	*RAMP1*	4	0.007395	0.676445	0.169768
SCIN	4	-0.26212	0.827802	0.082073	*SUPT20H*	3	0.15275	0.676445	0.169768
MDFIC	4	-0.2607	0.827802	0.082073	*NFRKB*	3	0.26939	0.676445	0.169768
TSC22D3	3	-0.25912	0.827802	0.082073	*CERS5*	4	0.31396	0.676445	0.169768
C20orf78	3	-0.25032	0.827802	0.082073	*FABP4*	3	0.38993	0.676445	0.169768
SERHL2	2	-0.24805	0.827802	0.082073	*UTP14A*	4	0.39386	0.676445	0.169768
SLAIN2	4	-0.24003	0.827802	0.082073	*MYO5B*	4	0.08611	0.677019	0.169399
ASIC1	4	-0.23825	0.827802	0.082073	*NUDT21*	4	0.14832	0.677192	0.169288
SH3RF3	4	-0.23002	0.827802	0.082073	*MIDN*	4	0.30316	0.677192	0.169288
CFHR4	4	-0.22965	0.827802	0.082073	*SNX1*	4	0.317	0.677192	0.169288
KLHL29	4	-0.22533	0.827802	0.082073	*TMTC4*	3	0.37704	0.677192	0.169288
ZNF816	4	-0.22129	0.827802	0.082073	*GID8*	3	0.39364	0.677192	0.169288
SLC29A2	3	-0.21454	0.827802	0.082073	*WRNIP1*	2	0.39396	0.677192	0.169288
PDK1	4	-0.19904	0.827802	0.082073	*SIGLEC11*	4	-0.31592	0.677251	0.16925
TIRAP	4	-0.19527	0.827802	0.082073	*ACTBL2*	3	0.003074	0.67745	0.169123
CXCL8	4	-0.19361	0.827802	0.082073	*KCP*	3	-0.58883	0.67769	0.168969
PRR22	4	-0.18875	0.827802	0.082073	*BAG4*	3	-0.37783	0.67769	0.168969
RPGR	3	-0.17961	0.827802	0.082073	*MARCHF2*	4	-0.53322	0.677796	0.168901
ACAD9	4	-0.15419	0.827802	0.082073	*NAPRT*	1	-0.53248	0.677796	0.168901
GK	4	-0.13464	0.827802	0.082073	*SLC22A13*	4	-0.51236	0.677796	0.168901
OSBPL2	4	-0.13255	0.827802	0.082073	*SPIB*	2	-0.49767	0.677796	0.168901
NET1	4	-0.12073	0.827802	0.082073	*ROCK2*	4	-0.38993	0.677796	0.168901
IMPG1	3	-0.096611	0.827802	0.082073	*PPP1R12C*	3	-0.34515	0.677796	0.168901
GPRIN3	4	-0.07695	0.827802	0.082073	*NUP153*	4	-0.32351	0.677796	0.168901
AAR2	4	-0.058989	0.827802	0.082073	*DECR1*	4	-0.30588	0.677796	0.168901
HERPUD1	4	-0.049633	0.827802	0.082073	*ALDH3A1*	4	-0.29872	0.677796	0.168901
PIGH	4	-0.031069	0.827802	0.082073	*REPS1*	4	-0.29408	0.677796	0.168901
EMP3	4	-0.023559	0.827802	0.082073	*AQP3*	4	-0.27896	0.677796	0.168901
PLP1	4	-0.011174	0.827802	0.082073	*IMPA1*	4	-0.27878	0.677796	0.168901
GAS2L1	3	0.008015	0.827802	0.082073	*OR7C1*	4	-0.27388	0.677796	0.168901
QTRT2	4	0.025344	0.827802	0.082073	*SYCE2*	3	-0.19722	0.677796	0.168901
NAXD	3	0.031848	0.827802	0.082073	*OLR1*	4	-0.14479	0.677796	0.168901
PSTPIP1	4	0.05415	0.827802	0.082073	*SERHL2*	2	-0.13189	0.677796	0.168901
HINT2	3	0.057208	0.827802	0.082073	*LRRC32*	4	-0.12773	0.677796	0.168901
ZFP14	4	0.057708	0.827802	0.082073	*NKAP*	4	-0.11122	0.677796	0.168901
MPHOSPH10	4	0.05852	0.827802	0.082073	*MFHAS1*	3	-0.10951	0.677796	0.168901
HDGFL3	3	0.064068	0.827802	0.082073	*PSMB8*	4	-0.072729	0.677796	0.168901
SFSWAP	3	0.065939	0.827802	0.082073	*PPARGC1B*	4	-0.07259	0.677796	0.168901
TEX9	4	0.072623	0.827802	0.082073	*TBC1D12*	4	-0.059177	0.677796	0.168901
MRPS12	4	0.079445	0.827802	0.082073	*LTB4R*	4	-0.048633	0.677796	0.168901
BCAR3	4	0.088117	0.827802	0.082073	*ALOX12*	4	-0.04551	0.677796	0.168901
LMAN2	3	0.12029	0.827802	0.082073	*MEIG1*	4	-0.038203	0.677796	0.168901
TXNDC2	4	0.12609	0.827802	0.082073	*PAX2*	3	0.003661	0.677796	0.168901
POLE2	3	0.15279	0.827802	0.082073	*AGAP3*	4	0.016987	0.677796	0.168901
BHLHE40	4	0.15454	0.827802	0.082073	*PRPF31*	4	0.028633	0.677796	0.168901
NQO2	4	0.18248	0.827802	0.082073	*ZNF486*	4	0.033524	0.677796	0.168901
ZNF836	4	0.1901	0.827802	0.082073	*MTRF1L*	2	0.050608	0.677796	0.168901
TAF1A	3	0.20397	0.827802	0.082073	*BCAR3*	4	0.088298	0.677796	0.168901
ECHS1	4	0.20642	0.827802	0.082073	*JADE3*	4	0.094686	0.677796	0.168901
SERINC3	4	0.21196	0.827802	0.082073	*ZBTB6*	4	0.13528	0.677796	0.168901
RBM39	4	0.21261	0.827802	0.082073	*PLCH2*	3	0.13567	0.677796	0.168901
CHGA	2	0.21682	0.827802	0.082073	*ZNF25*	4	0.14982	0.677796	0.168901
RRBP1	4	0.21971	0.827802	0.082073	*OARD1*	4	0.18901	0.677796	0.168901
GAN	4	0.22409	0.827802	0.082073	*XPA*	4	0.20934	0.677796	0.168901
PBX3	4	0.22622	0.827802	0.082073	*PGAM5*	4	0.21058	0.677796	0.168901
ARID5B	4	0.22885	0.827802	0.082073	*STK36*	4	0.21216	0.677796	0.168901
SETMAR	3	0.23507	0.827802	0.082073	*IFIT1*	4	0.21862	0.677796	0.168901
TBC1D10C	4	0.23573	0.827802	0.082073	*ZNF667*	4	0.23598	0.677796	0.168901

SPDYE5	2	0.23597	0.827802	0.082073	*SYT16*	4	0.23792	0.677796	0.168901
UBE2T	4	0.23844	0.827802	0.082073	*ZNF594*	2	0.25602	0.677796	0.168901
RAX	4	0.24802	0.827802	0.082073	*TTLL10*	4	0.25922	0.677796	0.168901
IRX4	4	0.24812	0.827802	0.082073	*KPNA6*	4	0.27844	0.677796	0.168901
PEX11A	4	0.25275	0.827802	0.082073	*PODXL2*	3	0.28413	0.677796	0.168901
PCNX4	2	0.2529	0.827802	0.082073	*MKLN1*	4	0.29186	0.677796	0.168901
CLPTM1	4	0.254	0.827802	0.082073	*ERCC4*	4	0.30098	0.677796	0.168901
SPNS1	4	0.25627	0.827802	0.082073	*PSMB3*	4	0.31086	0.677796	0.168901
TBC1D10A	4	0.25889	0.827802	0.082073	*ZNF613*	3	0.3492	0.677796	0.168901
WT1	4	0.26183	0.827802	0.082073	*AK8*	3	0.37795	0.677796	0.168901
NDUFB10	4	0.26324	0.827802	0.082073	*PIGB*	3	0.44328	0.677796	0.168901
KCNT1	4	0.27244	0.827802	0.082073	*SDHC*	2	0.57193	0.677796	0.168901
COL11A1	4	0.27822	0.827802	0.082073	*MTFR1L*	4	-0.24479	0.678404	0.168512
CLK2	4	0.28396	0.827802	0.082073	*RXFP3*	3	0.4733	0.679114	0.168058
GOLGA5	4	0.28568	0.827802	0.082073	*KDM4A*	3	-0.46382	0.679185	0.168012
TOLLIP	2	0.28649	0.827802	0.082073	*ABCB7*	4	-0.46312	0.679185	0.168012
GNAS	4	0.29338	0.827802	0.082073	*CHRDL1*	4	-0.45036	0.679185	0.168012
ZNF503	4	0.29744	0.827802	0.082073	*PTPN3*	3	-0.042057	0.679185	0.168012
DPF3	4	0.29842	0.827802	0.082073	*PTGIS*	3	0.065467	0.679185	0.168012
PPP1R14A	4	0.3052	0.827802	0.082073	*UBE2T*	4	0.16247	0.679185	0.168012
IQCA1	4	0.31469	0.827802	0.082073	*CFTR*	3	0.21751	0.679185	0.168012
TRAPPC11	4	0.31522	0.827802	0.082073	*ZNF275*	3	0.30854	0.679185	0.168012
C14orf132	4	0.31648	0.827802	0.082073	*FBLN2*	3	0.33452	0.679185	0.168012
PYROXD1	4	0.31735	0.827802	0.082073	*TANC1*	4	0.43189	0.679185	0.168012
CHCHD4	3	0.31799	0.827802	0.082073	*DIO1*	4	-0.36945	0.679478	0.167825
SLC25A5	3	0.32389	0.827802	0.082073	*CDC25A*	3	0.085807	0.679478	0.167825
TMEM177	3	0.32405	0.827802	0.082073	*RFWD3*	3	0.32254	0.679478	0.167825
RNF126	4	0.32912	0.827802	0.082073	*TAS2R3*	4	-0.20486	0.679655	0.167712
TUSC2	4	0.33249	0.827802	0.082073	*MCF2L2*	2	-0.47442	0.680105	0.167424
THAP8	3	0.34057	0.827802	0.082073	*GCKR*	2	-0.45212	0.680316	0.167289
AGPAT2	4	0.35444	0.827802	0.082073	*STAMBPL1*	1	-0.90282	0.681	0.166853
DMBX1	3	0.36033	0.827802	0.082073	*RGS13*	4	-0.25537	0.681	0.166853
AP5Z1	4	0.36263	0.827802	0.082073	*TMEM218*	2	0.48562	0.681	0.166853
UCHL3	3	0.37092	0.827802	0.082073	*C7orf61*	4	-0.40556	0.681753	0.166373
TCF3	3	0.39025	0.827802	0.082073	*B3GLCT*	4	-0.28597	0.681753	0.166373
FBXO11	4	0.39058	0.827802	0.082073	*KIZ*	2	-0.751	0.681972	0.166233
FKBP3	4	0.39583	0.827802	0.082073	*CENPX*	4	0.22669	0.682199	0.166089
DGAT2	4	0.39974	0.827802	0.082073	*VSNL1*	2	-0.26453	0.683041	0.165553
LAPTM4A	3	0.40629	0.827802	0.082073	*PDSS1*	3	0.27593	0.683041	0.165553
BRSK2	2	0.44939	0.827802	0.082073	*FAM25A*	2	0.35823	0.683041	0.165553
RFTN2	3	0.46957	0.827802	0.082073	*MYH3*	2	0.43493	0.683041	0.165553
NOC4L	4	0.48027	0.827802	0.082073	*DHX8*	3	0.49519	0.683041	0.165553
TRAFD1	3	0.49644	0.827802	0.082073	*NSL1*	3	0.65723	0.683041	0.165553
DOLK	4	0.51056	0.827802	0.082073	*SDSL*	3	-0.29035	0.683599	0.165198
LAMA1	2	0.54684	0.827802	0.082073	*FGFR1OP2*	4	-0.26237	0.68397	0.164963
TEX10	3	0.56338	0.827802	0.082073	*DLX6*	4	0.13301	0.684606	0.16456
CYB5R1	3	-0.42032	0.827815	0.082067	*CWF19L2*	4	0.29909	0.684606	0.16456
HEYL	2	0.17402	0.827815	0.082067	*OCRL*	3	0.61316	0.684606	0.16456
TOMM40	4	0.20103	0.827815	0.082067	*AVEN*	4	-0.17913	0.685069	0.164266
CORT	2	0.27065	0.827815	0.082067	*KLHL2*	4	-0.23457	0.685211	0.164176
ADNP2	3	0.34445	0.827815	0.082067	*RIIAD1*	4	0.018762	0.685211	0.164176
SYTL2	3	0.36946	0.827815	0.082067	*RC3H2*	4	0.26829	0.685211	0.164176
HNMT	4	-0.089289	0.827943	0.082	*LPCAT3*	3	0.14245	0.685551	0.16396
TMEM168	4	0.2457	0.828682	0.081612	*PPP1R13L*	3	0.29047	0.685551	0.16396
MUTYH	3	-0.43049	0.829091	0.081398	*LRG1*	3	-0.37611	0.685793	0.163807
HCRTR1	3	-0.35894	0.829091	0.081398	*ZBTB4*	3	-0.60047	0.685904	0.163737
TALDO1	3	0.23073	0.829091	0.081398	*ZNF75D*	3	-0.53552	0.685904	0.163737
INPP1	2	0.39151	0.829091	0.081398	*HIVEP2*	2	-0.42209	0.685904	0.163737

CMTM6	3	0.451	0.829091	0.081398	*PPP1CB*	3	-0.39722	0.685904	0.163737
MAGEA6	1	0.8204	0.829091	0.081398	*ZNF705E*	2	-0.38683	0.685904	0.163737
SECISBP2	2	-0.5494	0.829595	0.081134	*CBR4*	3	-0.36807	0.685904	0.163737
ATP5PB	3	-0.36111	0.829595	0.081134	*ADRA1B*	3	-0.3119	0.685904	0.163737
LYZ	4	-0.34429	0.829595	0.081134	*ADHFE1*	3	-0.29878	0.685904	0.163737
SCN2A	4	-0.26557	0.829595	0.081134	*ETFRF1*	4	-0.14749	0.685904	0.163737
OR2H2	3	-0.19991	0.829595	0.081134	*HLA-DOA*	4	-0.14232	0.685904	0.163737
ZKSCAN3	4	-0.15194	0.829595	0.081134	*ZNF319*	3	-0.042227	0.685904	0.163737
PRAF2	4	-0.12925	0.829595	0.081134	*NUDT2*	4	0.037686	0.685904	0.163737
S100P	4	0.044256	0.829595	0.081134	*OTX1*	4	0.079427	0.685904	0.163737
PDP1	4	0.24837	0.829595	0.081134	*EPB41L4A*	3	0.17577	0.685904	0.163737
LRRC42	4	0.24864	0.829595	0.081134	*KMT2C*	4	0.17683	0.685904	0.163737
HES5	2	0.24901	0.829595	0.081134	*PELO*	4	0.20331	0.685904	0.163737
PLEKHG4B	4	0.25632	0.829595	0.081134	*CD2BP2*	4	0.22185	0.685904	0.163737
FPR3	3	0.25882	0.829595	0.081134	*SLC25A29*	2	0.26242	0.685904	0.163737
NFYA	4	0.34928	0.829595	0.081134	*ZNF445*	2	0.26732	0.685904	0.163737
RAPGEFL1	4	0.47393	0.829595	0.081134	*DICER1*	2	0.27468	0.685904	0.163737
PRPF19	3	0.77011	0.829595	0.081134	*CTDSPL*	4	0.28808	0.685904	0.163737
ARHGEF9	4	-0.076094	0.829992	0.080926	*VMO1*	4	0.30829	0.685904	0.163737
TRPV3	3	-0.69012	0.830363	0.080732	*TBC1D23*	4	0.31763	0.685904	0.163737
AK7	3	-0.48795	0.830363	0.080732	*GOPC*	3	0.44411	0.685904	0.163737
CCDC60	3	-0.47355	0.830363	0.080732	*PSMB1*	4	0.44927	0.685904	0.163737
CRYBA2	2	-0.44182	0.830363	0.080732	*ERGIC1*	2	-0.5402	0.686077	0.163627
GYG2	4	-0.40061	0.830363	0.080732	*MAPKAP1*	4	0.13331	0.686077	0.163627
CPLX2	4	-0.34908	0.830363	0.080732	*LRMP*	3	0.14868	0.686077	0.163627
PCSK7	3	-0.30792	0.830363	0.080732	*EBF1*	3	0.10386	0.686208	0.163544
MAP1A	3	-0.30135	0.830363	0.080732	*RNASEH1*	2	-0.38514	0.686427	0.163406
FRMD3	3	-0.23733	0.830363	0.080732	*CIAPIN1*	3	0.30259	0.686427	0.163406
ZNF529	4	-0.22247	0.830363	0.080732	*SEL1L*	3	-0.38288	0.686877	0.163121
CHML	4	-0.18511	0.830363	0.080732	*PPP1R26*	3	-0.25072	0.686877	0.163121
EPN1	4	-0.17781	0.830363	0.080732	*HELQ*	3	0.28094	0.686877	0.163121
ARHGAP18	4	-0.16845	0.830363	0.080732	*IGSF6*	2	-0.53325	0.68713	0.162961
VPS13B	4	-0.066589	0.830363	0.080732	*PLEKHF1*	3	-0.46534	0.68713	0.162961
ORM1	3	-0.051682	0.830363	0.080732	*TRAF1*	3	-0.44696	0.68713	0.162961
NT5M	4	-0.034994	0.830363	0.080732	*RPS6KA2*	4	-0.39883	0.68713	0.162961
UBE3C	4	0.072255	0.830363	0.080732	*NUDCD1*	3	-0.3913	0.68713	0.162961
MAEA	4	0.22478	0.830363	0.080732	*SNAP91*	3	-0.32415	0.68713	0.162961
RAPGEF4	2	0.22868	0.830363	0.080732	*NCAN*	3	-0.27882	0.68713	0.162961
KCNJ8	4	0.26212	0.830363	0.080732	*BVES*	4	-0.23954	0.68713	0.162961
DRICH1	2	0.26462	0.830363	0.080732	*SLC45A4*	4	-0.22087	0.68713	0.162961
ASL	3	0.30922	0.830363	0.080732	*CMPK2*	3	-0.13374	0.68713	0.162961
AP3B2	4	0.36402	0.830363	0.080732	*HSD17B7*	3	-0.078294	0.68713	0.162961
HAP1	3	0.38986	0.830363	0.080732	*CYFIP1*	4	-0.075557	0.68713	0.162961
TEK	2	0.40631	0.830363	0.080732	*CNIH1*	4	-0.067153	0.68713	0.162961
NDOR1	2	0.85793	0.830363	0.080732	*RNF14*	4	-0.061675	0.68713	0.162961
COA6	3	-0.54996	0.830514	0.080653	*B4GALT2*	4	-0.058383	0.68713	0.162961
SCML2	4	-0.026361	0.831326	0.080229	*TNFSF13B*	4	-0.058143	0.68713	0.162961
SERPINF2	4	0.20863	0.831744	0.08001	*CFAP300*	4	-0.050087	0.68713	0.162961
IFITM10	4	-0.3784	0.831769	0.079997	*TMEM120B*	3	0.014504	0.68713	0.162961
MAK	3	0.34378	0.832371	0.079683	*ZNF212*	4	0.05342	0.68713	0.162961
FBXW2	3	-0.28992	0.832693	0.079515	*TP73*	4	0.15451	0.68713	0.162961
COX11	4	-0.25114	0.832693	0.079515	*UGT2B7*	4	0.19651	0.68713	0.162961
CD300A	4	-0.23885	0.832693	0.079515	*FIZ1*	4	0.19652	0.68713	0.162961
FOXQ1	4	-0.088655	0.832693	0.079515	*IFI6*	4	0.22963	0.68713	0.162961
EIF4EBP2	4	0.12983	0.832693	0.079515	*TGFB1I1*	2	0.24014	0.68713	0.162961
TPSD1	4	0.25271	0.832693	0.079515	*TRAPPC10*	2	0.26164	0.68713	0.162961
DRD2	3	0.25961	0.832693	0.079515	*BTBD11*	3	0.28739	0.68713	0.162961
RNF166	2	0.3873	0.832693	0.079515	*RDH12*	3	0.31689	0.68713	0.162961

OGFRL1	2	-0.50252	0.83421	0.078724	*NARS1*	3	0.3499	0.68713	0.162961
PARVB	4	-0.17092	0.83421	0.078724	*TCEAL9*	3	0.4455	0.68713	0.162961
CDK2AP1	3	0.40713	0.834576	0.078534	*USP4*	4	-0.43113	0.687418	0.162779
MAPK3	3	-0.29002	0.834891	0.07837	*ATAD1*	4	-0.16348	0.688876	0.161859
DCUN1D2	3	0.3578	0.834891	0.07837	*ARL4C*	4	-0.066692	0.688876	0.161859
ZFYVE27	2	0.30762	0.83509	0.078267	*ZFP69B*	4	-0.021654	0.688876	0.161859
LRPPRC	3	-0.85922	0.836036	0.077775	*MAMSTR*	4	0.15668	0.688876	0.161859
XPO1	2	-0.76036	0.836036	0.077775	*PIP4P2*	3	-0.42043	0.689451	0.161496
CREB1	3	-0.66698	0.836036	0.077775	*ALS2CL*	3	-0.40361	0.689451	0.161496
NOL7	2	-0.63467	0.836036	0.077775	*ZNF462*	3	-0.3318	0.689451	0.161496
GAL3ST1	2	-0.62482	0.836036	0.077775	*EMP3*	4	-0.31661	0.689451	0.161496
RBM4	2	-0.61704	0.836036	0.077775	*PIGZ*	3	-0.13283	0.689451	0.161496
RPL26L1	2	-0.58322	0.836036	0.077775	*CAPN7*	4	-0.23887	0.689505	0.161463
FBLN7	4	-0.54684	0.836036	0.077775	*COLGALT2*	4	-0.23265	0.689505	0.161463
HYOU1	4	-0.52155	0.836036	0.077775	*ODF2*	4	-0.23166	0.689505	0.161463
TCTN1	3	-0.51809	0.836036	0.077775	*RBCK1*	4	-0.18417	0.689505	0.161463
UBTD1	3	-0.46103	0.836036	0.077775	*PRPF3*	3	0.19369	0.689505	0.161463
PIGW	4	-0.45095	0.836036	0.077775	*RANBP3*	3	0.34512	0.689505	0.161463
SOS1	4	-0.43156	0.836036	0.077775	*CBX7*	4	-0.057093	0.689716	0.16133
RUFY4	3	-0.42068	0.836036	0.077775	*P2RY6*	3	-0.51763	0.689765	0.161299
SLC5A10	1	-0.4201	0.836036	0.077775	*SPG7*	4	-0.09019	0.689765	0.161299
SALL4	4	-0.41623	0.836036	0.077775	*MEGF9*	4	0.052797	0.689765	0.161299
SERTAD1	2	-0.40975	0.836036	0.077775	*TRANK1*	2	0.057035	0.689765	0.161299
LMTK3	3	-0.40948	0.836036	0.077775	*ZCWPW2*	4	0.081906	0.689765	0.161299
PGM1	3	-0.40327	0.836036	0.077775	*HECTD3*	4	0.14299	0.689765	0.161299
ESRP2	3	-0.39904	0.836036	0.077775	*NQO1*	4	0.40531	0.689765	0.161299
RBM14	2	-0.39333	0.836036	0.077775	*ZBTB10*	4	0.26795	0.690053	0.161117
NEUROG2	3	-0.38975	0.836036	0.077775	*SPOUT1*	4	0.4489	0.690053	0.161117
NCR3LG1	4	-0.37753	0.836036	0.077775	*WDR77*	4	-0.87771	0.690237	0.161002
FGF14	3	-0.37344	0.836036	0.077775	*LOXL3*	3	-0.68929	0.690237	0.161002
CCDC150	3	-0.35285	0.836036	0.077775	*TMEM125*	2	-0.67308	0.690237	0.161002
EXTL2	4	-0.33191	0.836036	0.077775	*NOTUM*	3	-0.6079	0.690237	0.161002
IMMP1L	4	-0.3312	0.836036	0.077775	*GPBP1*	2	-0.59177	0.690237	0.161002
BEX1	3	-0.32542	0.836036	0.077775	*USP38*	3	-0.55082	0.690237	0.161002
RP2	4	-0.31845	0.836036	0.077775	*ANGPTL8*	4	-0.52355	0.690237	0.161002
PCDHB16	4	-0.29654	0.836036	0.077775	*PKP3*	4	-0.49124	0.690237	0.161002
CHRD	4	-0.28886	0.836036	0.077775	*H1-1*	4	-0.46956	0.690237	0.161002
ECE1	4	-0.28173	0.836036	0.077775	*EXD1*	4	-0.46301	0.690237	0.161002
TSPAN13	4	-0.27936	0.836036	0.077775	*ATP5MC2*	4	-0.43429	0.690237	0.161002
IL10RA	4	-0.2753	0.836036	0.077775	*ZNF711*	4	-0.4316	0.690237	0.161002
IFT52	4	-0.27506	0.836036	0.077775	*ARL2BP*	3	-0.42662	0.690237	0.161002
HECW2	4	-0.27495	0.836036	0.077775	*ENTPD5*	3	-0.42367	0.690237	0.161002
C1orf54	3	-0.26882	0.836036	0.077775	*COQ8A*	3	-0.42329	0.690237	0.161002
FZR1	4	-0.25653	0.836036	0.077775	*GLB1L*	4	-0.42164	0.690237	0.161002
MFSD10	3	-0.25241	0.836036	0.077775	*SLC2A6*	4	-0.41285	0.690237	0.161002
SFT2D1	4	-0.23698	0.836036	0.077775	*SNRK*	4	-0.4124	0.690237	0.161002
LRRC37B	2	-0.23601	0.836036	0.077775	*TMEM219*	4	-0.40295	0.690237	0.161002
PPP1R1B	4	-0.22962	0.836036	0.077775	*SRGAP2B*	1	-0.39801	0.690237	0.161002
OR56A4	2	-0.22711	0.836036	0.077775	*LHX5*	3	-0.39142	0.690237	0.161002
NRXN2	3	-0.20689	0.836036	0.077775	*IKZF1*	3	-0.35961	0.690237	0.161002
NTRK1	4	-0.20335	0.836036	0.077775	*OIT3*	3	-0.35825	0.690237	0.161002
MILR1	4	-0.19541	0.836036	0.077775	*NAXE*	4	-0.33786	0.690237	0.161002
SEC61A2	4	-0.19338	0.836036	0.077775	*BLOC1S3*	2	-0.33743	0.690237	0.161002
CYTH4	4	-0.19112	0.836036	0.077775	*ZFP28*	4	-0.33001	0.690237	0.161002
CMAS	3	-0.13807	0.836036	0.077775	*EFNA5*	4	-0.32269	0.690237	0.161002
TFEB	3	-0.1353	0.836036	0.077775	*IMMP1L*	4	-0.31946	0.690237	0.161002
TAAR9	4	-0.13503	0.836036	0.077775	*SPATA46*	3	-0.31809	0.690237	0.161002
KCMF1	4	-0.1335	0.836036	0.077775	*SNX6*	4	-0.31536	0.690237	0.161002

CHMP4C	2	-0.13207	0.836036	0.077775	*SKAP1*	4	-0.30596	0.690237	0.161002
WDR92	4	-0.11282	0.836036	0.077775	*KCNH3*	4	-0.30285	0.690237	0.161002
MYLK3	4	-0.10928	0.836036	0.077775	*MACROD1*	4	-0.30217	0.690237	0.161002
HOXD10	3	-0.10124	0.836036	0.077775	*BIRC6*	4	-0.29386	0.690237	0.161002
NCOA2	3	-0.083476	0.836036	0.077775	*DHCR24*	4	-0.28837	0.690237	0.161002
ENTPD5	3	-0.054068	0.836036	0.077775	*LSP1*	4	-0.28614	0.690237	0.161002
NCBP2	3	-0.047044	0.836036	0.077775	*NRXN2*	3	-0.28478	0.690237	0.161002
AGPAT4	4	-0.025108	0.836036	0.077775	*GNB2*	4	-0.28142	0.690237	0.161002
OTUD6B	3	-0.022506	0.836036	0.077775	*KATNAL1*	4	-0.27407	0.690237	0.161002
KIAA0586	4	-0.020872	0.836036	0.077775	*SKOR1*	2	-0.27113	0.690237	0.161002
BTLA	4	-0.012951	0.836036	0.077775	*PROSER1*	4	-0.26744	0.690237	0.161002
CEND1	3	0.019478	0.836036	0.077775	*TXNIP*	4	-0.26049	0.690237	0.161002
CNBD2	4	0.035538	0.836036	0.077775	*INPP4A*	4	-0.2574	0.690237	0.161002
BOD1L1	4	0.066955	0.836036	0.077775	*A3GALT2*	4	-0.25264	0.690237	0.161002
GLYCTK	4	0.07522	0.836036	0.077775	*ERAP2*	2	-0.24601	0.690237	0.161002
RNF24	4	0.10025	0.836036	0.077775	*C15orf32*	4	-0.24453	0.690237	0.161002
RNF125	3	0.11626	0.836036	0.077775	*ANXA11*	4	-0.22433	0.690237	0.161002
ZNF90	3	0.11907	0.836036	0.077775	*KDM6A*	2	-0.21903	0.690237	0.161002
PDCD5	4	0.13152	0.836036	0.077775	*NUP214*	2	-0.21271	0.690237	0.161002
CORO1B	4	0.14277	0.836036	0.077775	*SNTB1*	4	-0.20294	0.690237	0.161002
OVOL3	4	0.14712	0.836036	0.077775	*GPAT3*	3	-0.20258	0.690237	0.161002
MGARP	3	0.15972	0.836036	0.077775	*ANTKMT*	3	-0.2012	0.690237	0.161002
SAMD4B	4	0.1609	0.836036	0.077775	*OTUD6B*	3	-0.19067	0.690237	0.161002
SP8	4	0.16388	0.836036	0.077775	*RGS10*	4	-0.17819	0.690237	0.161002
ETFB	4	0.17763	0.836036	0.077775	*GABRE*	3	-0.17215	0.690237	0.161002
PLA2G4A	4	0.17959	0.836036	0.077775	*KLF12*	4	-0.1364	0.690237	0.161002
FBXO27	4	0.18177	0.836036	0.077775	*STEAP1B*	3	-0.12619	0.690237	0.161002
OIP5	3	0.21243	0.836036	0.077775	*TSPAN3*	4	-0.12227	0.690237	0.161002
XAB2	4	0.21341	0.836036	0.077775	*ATP23*	4	-0.11576	0.690237	0.161002
ARL1	4	0.21571	0.836036	0.077775	*OR2L2*	3	-0.088147	0.690237	0.161002
NEU3	4	0.22234	0.836036	0.077775	*ZBTB41*	4	-0.085051	0.690237	0.161002
CPAMD8	4	0.22249	0.836036	0.077775	*SYCP3*	4	-0.083505	0.690237	0.161002
MSRA	4	0.22283	0.836036	0.077775	*MR1*	3	-0.070067	0.690237	0.161002
ESRRA	3	0.22296	0.836036	0.077775	*G6PC*	3	-0.065632	0.690237	0.161002
ADGRG3	4	0.22532	0.836036	0.077775	*ESRRG*	4	-0.064951	0.690237	0.161002
PHF23	4	0.23095	0.836036	0.077775	*PIF1*	3	-0.059643	0.690237	0.161002
HDDC3	4	0.23227	0.836036	0.077775	*SIPA1L1*	4	-0.059057	0.690237	0.161002
CHMP1A	4	0.24006	0.836036	0.077775	*XKR8*	4	-0.053108	0.690237	0.161002
C6orf120	4	0.24984	0.836036	0.077775	*CD320*	4	-0.038634	0.690237	0.161002
ERLIN1	2	0.25926	0.836036	0.077775	*BCL9*	3	-0.01327	0.690237	0.161002
CHST10	2	0.26821	0.836036	0.077775	*PCCB*	3	-0.004478	0.690237	0.161002
SLC16A7	4	0.27711	0.836036	0.077775	*COL11A2*	4	0.001465	0.690237	0.161002
MCL1	4	0.28316	0.836036	0.077775	*TRAPPC6A*	4	0.010709	0.690237	0.161002
FBP1	4	0.28399	0.836036	0.077775	*CCL5*	4	0.022797	0.690237	0.161002
PAXBP1	4	0.28577	0.836036	0.077775	*YWHAG*	2	0.02961	0.690237	0.161002
CMTM8	4	0.28985	0.836036	0.077775	*MITF*	4	0.034804	0.690237	0.161002
A1BG	3	0.30451	0.836036	0.077775	*FITM2*	3	0.049925	0.690237	0.161002
ATXN2L	2	0.31325	0.836036	0.077775	*EPM2AIP1*	4	0.052162	0.690237	0.161002
SLC25A3	4	0.32795	0.836036	0.077775	*ADRA2C*	3	0.052549	0.690237	0.161002
STOML2	2	0.33326	0.836036	0.077775	*TPPP*	4	0.053912	0.690237	0.161002
UGDH	3	0.33687	0.836036	0.077775	*RPL8*	4	0.057701	0.690237	0.161002
DNAJC27	3	0.33776	0.836036	0.077775	*MED16*	4	0.067592	0.690237	0.161002
ACTR3	4	0.33784	0.836036	0.077775	*SLC7A10*	4	0.077689	0.690237	0.161002
IRF6	3	0.36221	0.836036	0.077775	*PNLIPRP2*	4	0.096891	0.690237	0.161002
MRGPRD	4	0.36223	0.836036	0.077775	*MTHFS*	3	0.10114	0.690237	0.161002
DDOST	4	0.365	0.836036	0.077775	*OCA2*	4	0.10815	0.690237	0.161002
ECSCR	4	0.36782	0.836036	0.077775	*CACNA2D4*	4	0.11296	0.690237	0.161002
HMMR	4	0.37801	0.836036	0.077775	*FSTL5*	4	0.11326	0.690237	0.161002

HNRNPA1	3	0.38485	0.836036	0.077775	*KCNK1*	4	0.11589	0.690237	0.161002
EMG1	4	0.39101	0.836036	0.077775	*SCRIB*	4	0.11801	0.690237	0.161002
VXN	1	0.40411	0.836036	0.077775	*C1orf216*	4	0.11901	0.690237	0.161002
RRP1	2	0.50996	0.836036	0.077775	*STAT5B*	3	0.1319	0.690237	0.161002
APLP2	2	0.51972	0.836036	0.077775	*HTR1E*	4	0.15259	0.690237	0.161002
PDRG1	3	0.60322	0.836036	0.077775	*CPNE4*	4	0.1585	0.690237	0.161002
MAL2	3	-0.47295	0.83623	0.077674	*CRYBG3*	4	0.16052	0.690237	0.161002
C6orf223	2	-0.42376	0.83623	0.077674	*ORC4*	4	0.1652	0.690237	0.161002
B3GAT3	1	-0.38932	0.83623	0.077674	*CASP1*	3	0.1806	0.690237	0.161002
GAB1	4	-0.37858	0.83623	0.077674	*DAP3*	4	0.19242	0.690237	0.161002
EPHX2	4	-0.34949	0.83623	0.077674	*DACT1*	4	0.19517	0.690237	0.161002
ZNF692	3	-0.31588	0.83623	0.077674	*TRAPPC13*	4	0.2079	0.690237	0.161002
FOXO4	4	-0.30245	0.83623	0.077674	*PHACTR4*	4	0.21135	0.690237	0.161002
SOX10	3	-0.29637	0.83623	0.077674	*CSNK2A1*	4	0.21995	0.690237	0.161002
HSPA13	3	-0.2091	0.83623	0.077674	*NFIB*	4	0.22084	0.690237	0.161002
CACNA1B	4	-0.18126	0.83623	0.077674	*NUDT17*	4	0.22216	0.690237	0.161002
EYA3	4	-0.095562	0.83623	0.077674	*TRPV4*	4	0.22888	0.690237	0.161002
HOXD9	4	-0.032132	0.83623	0.077674	*IL17D*	3	0.23268	0.690237	0.161002
CCDC73	3	0.028793	0.83623	0.077674	*NAA30*	4	0.23408	0.690237	0.161002
PROM1	3	0.096029	0.83623	0.077674	*RIPOR3*	4	0.23943	0.690237	0.161002
RAB37	3	0.12988	0.83623	0.077674	*ERLEC1*	4	0.24018	0.690237	0.161002
GRID2	4	0.21593	0.83623	0.077674	*LYPD6B*	4	0.24661	0.690237	0.161002
TAF7	4	0.22628	0.83623	0.077674	*PRRT4*	3	0.24914	0.690237	0.161002
UGT2B7	4	0.23893	0.83623	0.077674	*ZNF112*	4	0.24975	0.690237	0.161002
DMP1	3	0.23936	0.83623	0.077674	*TMEM165*	4	0.26152	0.690237	0.161002
NUP37	3	0.32275	0.83623	0.077674	*KBTBD2*	4	0.27015	0.690237	0.161002
GINM1	3	0.36908	0.83623	0.077674	*SESTD1*	4	0.2773	0.690237	0.161002
RPL26	1	0.83797	0.83623	0.077674	*TC2N*	4	0.27926	0.690237	0.161002
AGBL2	4	0.1961	0.836341	0.077617	*STEAP1*	3	0.28176	0.690237	0.161002
RASGEF1B	3	-0.3082	0.836452	0.077559	*WDR92*	4	0.28774	0.690237	0.161002
KRBOX4	2	0.31231	0.836946	0.077303	*FAM221A*	3	0.29337	0.690237	0.161002
TOR1A	4	0.37329	0.836946	0.077303	*GCM2*	3	0.2956	0.690237	0.161002
NDUFA13	3	0.48152	0.836946	0.077303	*FAAP24*	3	0.2968	0.690237	0.161002
PDZD8	4	-0.019927	0.836976	0.077287	*ZC3H3*	3	0.29715	0.690237	0.161002
MORN3	2	-0.49006	0.837029	0.077259	*TDRD5*	4	0.29747	0.690237	0.161002
DIP2A	2	-0.41667	0.837029	0.077259	*GMEB1*	4	0.30535	0.690237	0.161002
CEP128	4	-0.28936	0.837029	0.077259	*AKR1C3*	4	0.30676	0.690237	0.161002
KLC1	4	-0.26464	0.837029	0.077259	*TMEM203*	3	0.30698	0.690237	0.161002
SLC38A9	4	-0.048089	0.837029	0.077259	*C16orf58*	4	0.31016	0.690237	0.161002
ATRX	3	0.19189	0.837029	0.077259	*MIOS*	4	0.31172	0.690237	0.161002
SNAPC4	3	0.40917	0.837029	0.077259	*XPC*	3	0.31284	0.690237	0.161002
PPP1R3D	4	-0.45353	0.83709	0.077228	*DPP8*	3	0.31827	0.690237	0.161002
ERBB2	3	-0.39335	0.83709	0.077228	*ICK*	3	0.32652	0.690237	0.161002
RPS15	1	-1.3649	0.8372	0.077171	*FAAP20*	3	0.3279	0.690237	0.161002
TTC12	4	-0.27153	0.837231	0.077155	*BTBD16*	3	0.33813	0.690237	0.161002
ZBTB33	4	-0.27287	0.837501	0.077014	*ALS2*	4	0.33922	0.690237	0.161002
CABIN1	4	-0.52244	0.837514	0.077008	*ELP1*	4	0.34046	0.690237	0.161002
LMAN1L	4	-0.4865	0.837514	0.077008	*PPP5D1*	3	0.34586	0.690237	0.161002
NCOA1	4	-0.43333	0.837514	0.077008	*CUL2*	4	0.34598	0.690237	0.161002
CEACAM1	4	-0.33743	0.837514	0.077008	*OS9*	4	0.35439	0.690237	0.161002
L1TD1	4	-0.28594	0.837514	0.077008	*SLC7A9*	3	0.36268	0.690237	0.161002
ZNF827	4	-0.22119	0.837514	0.077008	*TAF12*	3	0.39304	0.690237	0.161002
PCDHGB6	4	-0.20875	0.837514	0.077008	*ESRRA*	3	0.41139	0.690237	0.161002
SIDT2	4	-0.18756	0.837514	0.077008	*GIPR*	3	0.44723	0.690237	0.161002
UBE2D1	4	-0.17203	0.837514	0.077008	*RAB6C*	1	0.50945	0.690237	0.161002
C2orf16	4	-0.043269	0.837514	0.077008	*KIFBP*	1	0.53792	0.690237	0.161002
SLC7A11	4	0.053819	0.837514	0.077008	*SKP1*	4	0.54213	0.690237	0.161002
PDCD11	3	-0.58752	0.839141	0.076165	*MYRF*	2	0.55262	0.690237	0.161002

SEC22B	2	-0.55848	0.839141	0.076165	*DPH7*	2	0.5778	0.690237	0.161002
TRIM27	2	-0.55516	0.839141	0.076165	*DHX33*	4	0.66293	0.690237	0.161002
INPP5E	4	-0.43769	0.839141	0.076165	*FOS*	1	0.68045	0.690237	0.161002
ZNF200	3	-0.42858	0.839141	0.076165	*SNRPD1*	2	0.80099	0.690237	0.161002
RSKR	2	-0.40855	0.839141	0.076165	*ZNF506*	4	-0.10228	0.690529	0.160818
SPATA24	3	-0.40763	0.839141	0.076165	*ACKR4*	3	0.21682	0.690529	0.160818
YME1L1	4	-0.39587	0.839141	0.076165	*SLC25A6*	4	0.21885	0.690529	0.160818
PCDHB6	4	-0.3812	0.839141	0.076165	*ZNF775*	4	0.27365	0.690529	0.160818
PPP2CA	4	-0.36366	0.839141	0.076165	*FAM169A*	4	-0.43913	0.690745	0.160682
C7orf25	4	-0.34995	0.839141	0.076165	*ARSK*	4	-0.20756	0.690745	0.160682
MXD3	3	-0.32756	0.839141	0.076165	*HOXB7*	4	0.25184	0.690745	0.160682
CEP290	4	-0.30022	0.839141	0.076165	*LIN7B*	4	-0.42185	0.691027	0.160505
APPL1	4	-0.26941	0.839141	0.076165	*PRPF8*	3	-0.31687	0.691027	0.160505
MYOCD	4	-0.25995	0.839141	0.076165	*CADM4*	4	-0.28829	0.691027	0.160505
TATDN3	4	-0.25337	0.839141	0.076165	*STAT6*	4	-0.28061	0.691027	0.160505
HHIPL2	4	-0.24707	0.839141	0.076165	*SLC5A9*	3	-0.26925	0.691027	0.160505
SHC1	4	-0.24125	0.839141	0.076165	*RASL11B*	4	-0.14832	0.691027	0.160505
EIF2S3	4	-0.23777	0.839141	0.076165	*PLSCR2*	4	0.15408	0.691027	0.160505
ALKBH2	4	-0.23348	0.839141	0.076165	*TBC1D22A*	3	0.17705	0.691027	0.160505
ZNF124	4	-0.22543	0.839141	0.076165	*CDK5RAP1*	4	0.20961	0.691027	0.160505
GLTP	4	-0.17296	0.839141	0.076165	*IPO7*	4	0.27769	0.691027	0.160505
LYSMD2	4	-0.15607	0.839141	0.076165	*ZNF512B*	2	0.35359	0.691027	0.160505
SHD	3	-0.15474	0.839141	0.076165	*WIPF3*	2	0.4958	0.691027	0.160505
COLGALT2	4	-0.10172	0.839141	0.076165	*SLC25A48*	4	0.25823	0.691059	0.160485
TRAPPC9	3	-9.52E-04	0.839141	0.076165	*CMTM4*	4	0.30013	0.691059	0.160485
ERC1	3	0.033073	0.839141	0.076165	*LPGAT1*	3	0.37318	0.691059	0.160485
CBY1	4	0.084342	0.839141	0.076165	*PRKAB2*	3	0.14142	0.691808	0.160015
USP38	3	0.13433	0.839141	0.076165	*ST6GALNAC1*	4	-0.48256	0.691929	0.159938
HHEX	4	0.1434	0.839141	0.076165	*CXCL14*	3	-0.29846	0.694226	0.158499
SPDYE1	3	0.18921	0.839141	0.076165	*METAP1D*	4	-0.27277	0.694226	0.158499
NAT10	4	0.19901	0.839141	0.076165	*SLC8B1*	4	-0.25297	0.694226	0.158499
RUSC1	4	0.21673	0.839141	0.076165	*MEIOB*	4	-0.19215	0.694226	0.158499
FAM43B	4	0.22922	0.839141	0.076165	*STX7*	3	-0.092308	0.694226	0.158499
ADGRG2	4	0.23504	0.839141	0.076165	*NCR1*	4	0.13679	0.694226	0.158499
H3C3	4	0.27217	0.839141	0.076165	*RASSF7*	4	0.21063	0.694226	0.158499
FCN3	4	0.2753	0.839141	0.076165	*ALKBH6*	4	0.28841	0.694226	0.158499
HOXC8	3	0.28735	0.839141	0.076165	*CCDC117*	3	0.34738	0.694226	0.158499
MED16	4	0.30207	0.839141	0.076165	*GTF2A2*	3	0.40225	0.694226	0.158499
WSB1	4	0.30548	0.839141	0.076165	*PYGM*	4	-0.2009	0.695059	0.157978
TRAPPC6B	4	0.3236	0.839141	0.076165	*TMEM167B*	4	0.035434	0.695159	0.157916
RUBCNL	3	0.32378	0.839141	0.076165	*OR2B6*	2	0.36989	0.695159	0.157916
BAG1	3	0.33187	0.839141	0.076165	*HDHD3*	2	0.45281	0.695159	0.157916
SLC7A10	4	0.3416	0.839141	0.076165	*TVP23B*	2	-0.5635	0.695272	0.157845
ANKRD17	3	0.34585	0.839141	0.076165	*SELENON*	4	-0.2999	0.695339	0.157804
MYL3	4	0.3482	0.839141	0.076165	*PRAF2*	4	-0.2412	0.695339	0.157804
POLR2K	4	0.35895	0.839141	0.076165	*SELENOS*	2	-0.51275	0.695658	0.157604
SERPINI1	2	0.37909	0.839141	0.076165	*LGSN*	4	-0.24812	0.695658	0.157604
DMAP1	4	0.39154	0.839141	0.076165	*ATP5MGL*	2	0.20528	0.697112	0.156698
YAE1	3	0.43875	0.839141	0.076165	*VAV2*	4	-0.32283	0.697145	0.156677
TRIM13	2	0.51524	0.839141	0.076165	*THEMIS2*	4	-0.28776	0.697266	0.156602
PLPPR3	3	0.52261	0.839141	0.076165	*CENPO*	4	-0.060377	0.697266	0.156602
RERE	1	0.41142	0.839565	0.075946	*DNAJB3*	3	-0.45021	0.698245	0.155992
IQSEC3	2	0.29964	0.840146	0.075645	*EGLN3*	2	-0.49038	0.699374	0.155291
CDKN2B	2	-0.45445	0.840262	0.075585	*MON1A*	3	0.37509	0.699374	0.155291
PANK2	4	-0.3449	0.840262	0.075585	*DPF1*	2	0.591	0.700643	0.154503
MGAT4A	4	-0.32569	0.840262	0.075585	*CCDC152*	4	-0.3339	0.700975	0.154298
GSTM4	2	-0.30323	0.840262	0.075585	*ALX3*	4	-0.26016	0.700975	0.154298
MALT1	3	-0.62878	0.842611	0.074373	*C6orf99*	4	-0.046725	0.700975	0.154298

FAM53C	3	-0.428	0.842611	0.074373	*TRHDE*	3	0.37897	0.701062	0.154244
SEC14L2	3	-0.42702	0.842611	0.074373	*MLLT10*	2	-0.40953	0.701104	0.154218
ZNF585A	3	-0.42111	0.842611	0.074373	*GPR83*	4	-0.28863	0.701104	0.154218
NDEL1	2	-0.40621	0.842611	0.074373	*SLC24A4*	4	-0.22222	0.701104	0.154218
RHOXF2	1	-0.33249	0.842611	0.074373	*ART5*	4	-0.20563	0.701104	0.154218
FAM222A	3	-0.30457	0.842611	0.074373	*LY86*	4	-0.034405	0.701104	0.154218
SRXN1	4	-0.23424	0.842611	0.074373	*ZGRF1*	3	0.34302	0.701104	0.154218
CRELD1	3	-0.23374	0.842611	0.074373	*AMBP*	3	0.41097	0.701104	0.154218
PIK3CA	4	-0.20751	0.842611	0.074373	*ANXA8*	2	-0.4182	0.701269	0.154115
SMCR8	4	-0.19572	0.842611	0.074373	*DEAF1*	2	-0.56654	0.701466	0.153993
AKR1C2	2	-0.162	0.842611	0.074373	*EPN2*	4	-0.22193	0.702102	0.1536
ESCO1	3	-0.14941	0.842611	0.074373	*MYL5*	2	0.34996	0.702479	0.153367
PLEKHG1	3	-0.14625	0.842611	0.074373	*RRAGD*	2	-0.4823	0.702479	0.153366
PPFIBP1	3	-0.097156	0.842611	0.074373	*SNTB2*	4	0.042562	0.702479	0.153366
PRDM15	2	-0.052899	0.842611	0.074373	*MYLK3*	4	0.092903	0.702479	0.153366
CUL9	4	-1.33E-04	0.842611	0.074373	*RNPEPL1*	4	0.1351	0.702479	0.153366
LOC403312	4	0.001041	0.842611	0.074373	*L3MBTL2*	3	0.14502	0.702479	0.153366
TADA2B	3	0.019987	0.842611	0.074373	*LRRC40*	4	0.19625	0.702479	0.153366
TARS2	4	0.060828	0.842611	0.074373	*PLAAT5*	4	0.25552	0.702479	0.153366
SNX3	4	0.18215	0.842611	0.074373	*HADHA*	4	0.26801	0.702479	0.153366
FNBP4	4	0.19464	0.842611	0.074373	*SLC35A5*	4	0.2883	0.702479	0.153366
P2RY8	4	0.20921	0.842611	0.074373	*NPM1*	1	0.43066	0.702479	0.153366
SHARPIN	2	0.21563	0.842611	0.074373	*FFAR3*	1	0.48499	0.702479	0.153366
PDCD4	4	0.22153	0.842611	0.074373	*PPFIA3*	4	0.004025	0.702495	0.153357
ITPRIPL1	4	0.22375	0.842611	0.074373	*SCUBE3*	4	0.083941	0.702495	0.153357
RELB	2	0.24122	0.842611	0.074373	*TKTL1*	4	0.18951	0.702495	0.153357
MORF4L2	4	0.25507	0.842611	0.074373	*SLC8A2*	1	0.44943	0.702495	0.153357
SLC10A4	4	0.25601	0.842611	0.074373	*C9orf153*	4	-0.45212	0.702738	0.153207
PIAS3	1	0.288	0.842611	0.074373	*SNX10*	4	-0.30725	0.702738	0.153207
NHSL1	2	0.28938	0.842611	0.074373	*CDC42BPA*	4	-0.29851	0.702738	0.153207
MCM3AP	4	0.28989	0.842611	0.074373	*GPM6A*	4	-0.15926	0.702738	0.153207
PRRG2	4	0.29042	0.842611	0.074373	*DMRTA1*	4	-0.37989	0.702862	0.15313
SBNO2	4	0.29655	0.842611	0.074373	*PXDN*	4	-0.25555	0.702862	0.15313
ITPR2	4	0.29816	0.842611	0.074373	*BMP7*	4	0.14005	0.704572	0.152075
IL20RB	3	0.33452	0.842611	0.074373	*HVCN1*	4	0.07177	0.704673	0.152012
HSPA5	4	0.36645	0.842611	0.074373	*SDC1*	4	0.14142	0.704743	0.151969
DAGLA	4	0.3965	0.842611	0.074373	*VTN*	3	0.42324	0.705811	0.151312
PCNA	2	0.40803	0.842611	0.074373	*MAP3K4*	3	-0.41708	0.705919	0.151245
ARHGAP12	3	0.48139	0.842611	0.074373	*TANGO2*	3	-0.33372	0.705919	0.151245
CNOT3	3	0.49539	0.842611	0.074373	*DKK4*	3	0.16639	0.705996	0.151198
SIX2	2	0.2098	0.843185	0.074077	*DKK1*	4	-0.34975	0.708777	0.14949
HMGCS2	4	-0.59678	0.843387	0.073973	*PIM1*	4	-0.29711	0.708955	0.149381
GTPBP8	4	-0.46962	0.843387	0.073973	*KIF13B*	4	0.040993	0.709491	0.149053
GCKR	2	-0.42592	0.843387	0.073973	*HRH1*	4	0.17345	0.709893	0.148807
PRKN	3	-0.41217	0.843387	0.073973	*PLRG1*	4	0.36688	0.709893	0.148807
IFNAR2	4	-0.39727	0.843387	0.073973	*MIOX*	3	-0.11276	0.710214	0.148611
PPP1R18	2	-0.37943	0.843387	0.073973	*GNPDA1*	4	-0.10408	0.710214	0.148611
SPAG16	4	-0.35435	0.843387	0.073973	*C9orf50*	3	0.44236	0.710214	0.148611
ARHGAP33	2	-0.34254	0.843387	0.073973	*CYP27A1*	3	-0.05838	0.710467	0.148456
CAV3	4	-0.33786	0.843387	0.073973	*QDPR*	3	0.32781	0.710467	0.148456
CELF5	4	-0.33638	0.843387	0.073973	*BBOF1*	3	0.35048	0.710947	0.148163
OSER1	3	-0.29928	0.843387	0.073973	*KCNIP4*	4	-0.37713	0.711019	0.148119
RGS2	4	-0.27467	0.843387	0.073973	*LGI1*	4	-0.36825	0.711019	0.148119
IP6K3	4	-0.26956	0.843387	0.073973	*SLC5A12*	4	-0.23666	0.711019	0.148119
FGFR3	4	-0.26106	0.843387	0.073973	*PRRT1*	2	-0.3026	0.711153	0.148037
SRGAP1	4	-0.2494	0.843387	0.073973	*GDPGP1*	4	-0.24568	0.711153	0.148037
PLPP6	4	-0.23087	0.843387	0.073973	*GRAMD1B*	4	-0.20621	0.711153	0.148037
CASP3	4	-0.20889	0.843387	0.073973	*ITGB1BP1*	4	-0.13993	0.711153	0.148037

YBX3	4	-0.20714	0.843387	0.073973	*PPP2R5A*	4	-0.12149	0.711153	0.148037
ATP6V1C1	3	-0.20411	0.843387	0.073973	*ZNF732*	4	0.19104	0.711153	0.148037
YBEY	3	-0.20004	0.843387	0.073973	*GSTM5*	4	0.27044	0.711153	0.148037
TMEM92	4	-0.1876	0.843387	0.073973	*EN2*	3	0.29272	0.711153	0.148037
SGSM2	3	-0.15513	0.843387	0.073973	*KRT80*	4	0.33167	0.711153	0.148037
CSNK1G2	3	-0.14196	0.843387	0.073973	*RUNDC3A*	2	0.36585	0.711153	0.148037
CAPN6	4	-0.13855	0.843387	0.073973	*UTP6*	2	-0.84411	0.711349	0.147917
TBX20	4	-0.13296	0.843387	0.073973	*TTC8*	2	-0.55502	0.711349	0.147917
NOG	4	-0.11827	0.843387	0.073973	*ATG5*	3	-0.50229	0.711349	0.147917
ZNF473	4	-0.11368	0.843387	0.073973	*LEPR*	3	-0.39455	0.711349	0.147917
ANXA7	4	-0.080883	0.843387	0.073973	*ANTXR1*	3	-0.3872	0.711349	0.147917
NLRP6	4	-0.068068	0.843387	0.073973	*EHD3*	4	-0.34668	0.711349	0.147917
USP33	4	-0.064491	0.843387	0.073973	*RETREG1*	4	-0.33114	0.711349	0.147917
APOM	4	-0.045318	0.843387	0.073973	*KRT15*	4	-0.31108	0.711349	0.147917
ABHD18	3	-0.032079	0.843387	0.073973	*CA14*	4	-0.30245	0.711349	0.147917
SAR1B	2	0.006266	0.843387	0.073973	*OST4*	4	-0.29769	0.711349	0.147917
ZC2HC1C	3	0.027155	0.843387	0.073973	*RRP8*	4	-0.2804	0.711349	0.147917
DZIP1L	4	0.063568	0.843387	0.073973	*CLRN3*	4	-0.25167	0.711349	0.147917
RRP7A	4	0.078856	0.843387	0.073973	*SS18*	4	-0.25046	0.711349	0.147917
ZNF565	3	0.10489	0.843387	0.073973	*MBTD1*	4	-0.24795	0.711349	0.147917
KAT2B	4	0.10864	0.843387	0.073973	*ZNF521*	4	-0.22293	0.711349	0.147917
FCGRT	3	0.16167	0.843387	0.073973	*FYCO1*	4	-0.19806	0.711349	0.147917
NDUFB3	4	0.16467	0.843387	0.073973	*GPR160*	4	-0.13005	0.711349	0.147917
NOL8	3	0.17448	0.843387	0.073973	*TAT*	4	-0.096064	0.711349	0.147917
ZNF862	4	0.18659	0.843387	0.073973	*CDK19*	4	-0.026624	0.711349	0.147917
DRAP1	4	0.18965	0.843387	0.073973	*PPP1R37*	4	0.098458	0.711349	0.147917
MLLT3	4	0.20974	0.843387	0.073973	*FBXL13*	4	0.13718	0.711349	0.147917
RBP4	4	0.21528	0.843387	0.073973	*TSSK4*	4	0.20344	0.711349	0.147917
PLCB4	4	0.22071	0.843387	0.073973	*CEACAM3*	4	0.20502	0.711349	0.147917
PCDHB10	4	0.24459	0.843387	0.073973	*VAT1*	4	0.22879	0.711349	0.147917
RNLS	4	0.25253	0.843387	0.073973	*OR13H1*	4	0.23541	0.711349	0.147917
NPR3	4	0.27178	0.843387	0.073973	*MTMR10*	4	0.23641	0.711349	0.147917
TBC1D23	4	0.27774	0.843387	0.073973	*OVGP1*	4	0.24664	0.711349	0.147917
SLC6A6	3	0.28533	0.843387	0.073973	*MCF2*	4	0.26422	0.711349	0.147917
ULK4	4	0.28667	0.843387	0.073973	*PHKA1*	3	0.29611	0.711349	0.147917
NRARP	4	0.29092	0.843387	0.073973	*KNSTRN*	3	0.32241	0.711349	0.147917
GRAMD4	3	0.29828	0.843387	0.073973	*RSPO1*	4	0.32632	0.711349	0.147917
COA3	3	0.29942	0.843387	0.073973	*CCT4*	4	0.43811	0.711349	0.147917
SEC14L3	3	0.30094	0.843387	0.073973	*ATP5F1E*	2	0.35295	0.711506	0.147821
PRPS2	4	0.30285	0.843387	0.073973	*LSM3*	2	0.42691	0.711506	0.147821
TMBIM4	2	0.31507	0.843387	0.073973	*COL26A1*	4	-0.19235	0.712073	0.147476
PDC	2	0.31612	0.843387	0.073973	*S100A1*	3	0.33301	0.712074	0.147475
TUBGCP3	4	0.32412	0.843387	0.073973	*METTL23*	3	0.35012	0.712074	0.147475
CAND2	3	0.32437	0.843387	0.073973	*CNPY2*	3	0.36835	0.712074	0.147475
MAOB	3	0.33054	0.843387	0.073973	*CPM*	4	-0.30871	0.712346	0.147309
VAMP2	4	0.33175	0.843387	0.073973	*GXYLT1*	4	-0.2741	0.712346	0.147309
PIH1D2	4	0.33209	0.843387	0.073973	*UPK1A*	4	-0.056069	0.712346	0.147309
SLC50A1	2	0.35799	0.843387	0.073973	*RNF115*	4	0.024112	0.712346	0.147309
CD4	3	0.36022	0.843387	0.073973	*MAP2K5*	3	0.18217	0.712346	0.147309
ZFAND5	3	0.37581	0.843387	0.073973	*DLK1*	4	-0.070813	0.712398	0.147278
DDX27	4	0.42685	0.843387	0.073973	*FBXO30*	4	-0.27069	0.712483	0.147225
MALL	3	0.44104	0.843387	0.073973	*PRPSAP2*	4	-0.23603	0.712483	0.147225
PFDN5	4	0.46121	0.843387	0.073973	*DHRS4L1*	4	-0.22287	0.712483	0.147225
SRR	3	0.46809	0.843387	0.073973	*IDO1*	4	-0.20729	0.712483	0.147225
CHMP4A	3	0.51304	0.843387	0.073973	*LOC10192757*	4	0.093329	0.712483	0.147225
LYRM4	3	0.66927	0.843387	0.073973	*ASH2L*	4	0.29805	0.712483	0.147225
TRAPPC5	3	1.0233	0.843387	0.073973	*RPE65*	3	-0.50751	0.712489	0.147222
EPHB1	4	-0.028419	0.84342	0.073956	*ZSWIM5*	4	-0.2101	0.712489	0.147222

BCL3	3	0.23502	0.84342	0.073956	*RSAD2*	4	-0.15913	0.712489	0.147222
TUFT1	2	0.31793	0.84342	0.073956	*AAK1*	2	0.19911	0.712489	0.147222
CHAF1A	2	0.51964	0.84342	0.073956	*FNDC7*	4	-0.26314	0.712497	0.147217
ARL4A	1	-0.76235	0.843536	0.073896	*TMEM86B*	3	0.097276	0.712497	0.147217
ELF5	4	-0.11472	0.843536	0.073896	*SMIM20*	4	0.28096	0.712497	0.147217
ZFYVE9	3	-0.03905	0.843536	0.073896	*GALNT15*	3	-0.45344	0.712862	0.146995
CHST11	3	0.056633	0.843536	0.073896	*ZNF282*	2	0.096268	0.712862	0.146995
CBARP	4	0.25073	0.843536	0.073896	*ELMOD3*	4	0.12291	0.712862	0.146995
NDUFV1	3	0.27974	0.843536	0.073896	*SENP5*	4	0.12701	0.712862	0.146995
NUP160	3	0.46573	0.843536	0.073896	*KDELR2*	3	-0.36237	0.713236	0.146767
DAPK1	4	-0.36566	0.843716	0.073804	*LIN28A*	4	-0.31602	0.713236	0.146767
NUMA1	3	0.58024	0.843743	0.07379	*TAF1*	3	-0.15241	0.713236	0.146767
ZNF304	1	0.64846	0.843923	0.073697	*MAPK15*	2	0.052007	0.713236	0.146767
RYR3	2	-0.60647	0.844256	0.073526	*HSH2D*	4	0.15182	0.713236	0.146767
SCUBE3	4	-0.50306	0.844829	0.073231	*MGAT1*	4	0.15702	0.713236	0.146767
LHFPL3	1	-0.4859	0.844829	0.073231	*AXL*	3	0.22533	0.713236	0.146767
NLK	4	-0.34453	0.844829	0.073231	*AZIN1*	2	0.31409	0.713236	0.146767
C20orf144	4	-0.31074	0.844829	0.073231	*ARHGAP17*	3	0.32195	0.713236	0.146767
TGM2	4	-0.2956	0.844829	0.073231	*STEAP4*	4	-0.45387	0.713247	0.14676
NSUN6	4	-0.27877	0.844829	0.073231	*UBL4B*	4	-0.39523	0.713247	0.14676
MCMDC2	4	-0.26694	0.844829	0.073231	*PPEF1*	4	-0.35802	0.713247	0.14676
PDE8B	4	-0.24917	0.844829	0.073231	*PSMF1*	2	-0.3342	0.713247	0.14676
TYMSOS	4	-0.18843	0.844829	0.073231	*TMEM106A*	4	-0.31981	0.713247	0.14676
APAF1	4	-0.12782	0.844829	0.073231	*CCR7*	4	-0.28802	0.713247	0.14676
VAMP3	3	-0.10375	0.844829	0.073231	*CREBRF*	4	-0.26256	0.713247	0.14676
TTN	3	-0.013918	0.844829	0.073231	*NUDT15*	4	-0.24821	0.713247	0.14676
ZSCAN25	4	-0.003865	0.844829	0.073231	*MPIG6B*	4	-0.19736	0.713247	0.14676
KDM2A	4	0.01784	0.844829	0.073231	*SPATA45*	4	-0.16878	0.713247	0.14676
DNMBP	3	0.035387	0.844829	0.073231	*KIFC1*	4	-0.14918	0.713247	0.14676
NEIL3	4	0.15304	0.844829	0.073231	*KIF1A*	3	-0.23927	0.713614	0.146537
TFPI2	4	0.15599	0.844829	0.073231	*ATP5MD*	2	0.35783	0.713614	0.146537
NECAP1	4	0.25742	0.844829	0.073231	*SFTPD*	4	-0.09783	0.713722	0.146471
ZNF486	4	0.30463	0.844829	0.073231	*PPP2R5E*	3	0.24151	0.713722	0.146471
NXPH4	3	0.31763	0.844829	0.073231	*SYAP1*	4	-0.062369	0.713781	0.146435
PHKA2	3	0.33933	0.844829	0.073231	*A4GALT*	3	-0.43924	0.714264	0.146141
TRMT1	3	0.37025	0.844829	0.073231	*NAT14*	4	-0.34987	0.714314	0.146111
MRPS27	3	0.38898	0.844829	0.073231	*KIAA0895L*	2	-0.033701	0.714831	0.145797
PCNP	3	0.56184	0.844829	0.073231	*EIF2AK1*	2	0.15785	0.714831	0.145797
STAMBP	2	-0.47749	0.845012	0.073137	*RYR2*	2	0.27171	0.714831	0.145797
NAXE	4	-0.36223	0.845012	0.073137	*SART1*	3	0.33065	0.714831	0.145797
CCN6	4	-0.22416	0.845012	0.073137	*PPP1R7*	2	0.59422	0.714831	0.145797
EEF1AKMT3	4	-0.17315	0.845012	0.073137	*RBP1*	3	-0.41827	0.715176	0.145587
ARIH2	3	-0.30758	0.845102	0.073091	*ETS1*	2	-0.67266	0.715275	0.145527
TM9SF2	3	-0.22973	0.845102	0.073091	*SERTAD4*	3	-0.30155	0.715275	0.145527
ZNF805	4	-0.21543	0.845102	0.073091	*SLCO4C1*	3	-0.15296	0.715275	0.145527
CTSO	4	-0.090271	0.845102	0.073091	*COLEC12*	4	0.11509	0.715275	0.145527
KCNJ16	4	0.008718	0.845102	0.073091	*MROH6*	4	0.20912	0.715275	0.145527
S1PR4	3	0.0996	0.845102	0.073091	*TNRC6B*	4	-0.43205	0.715381	0.145463
GNPTG	4	0.23973	0.845102	0.073091	*ERI1*	4	-0.30041	0.715381	0.145463
SMAD1	3	0.31225	0.845102	0.073091	*H2BC11*	3	-0.18766	0.715381	0.145463
NMT1	4	0.40734	0.845102	0.073091	*AJM1*	4	0.093742	0.715381	0.145463
ELF2	4	-0.25565	0.845293	0.072993	*TAS2R30*	4	0.19848	0.715381	0.145463
C7orf61	4	0.086781	0.845293	0.072993	*SVIL*	4	-0.36969	0.716308	0.1449
CCDC183	4	0.098622	0.845293	0.072993	*PURA*	2	-0.42711	0.71656	0.144748
NPFFR2	4	0.14032	0.845293	0.072993	*ZFAND2B*	3	-0.36062	0.71656	0.144748
PICALM	4	0.15626	0.845293	0.072993	*KCTD13*	4	-0.28864	0.71656	0.144748
PUS3	4	0.22398	0.845293	0.072993	*EEF1A2*	2	-0.26474	0.71656	0.144748
USP20	4	0.28277	0.845293	0.072993	*SLCO2A1*	2	0.04051	0.71656	0.144748

FRMD5	2	0.33505	0.845293	0.072993	*SIGLEC15*	2	0.0472	0.71656	0.144748
ARGLU1	4	0.46923	0.845293	0.072993	*EZR*	3	0.27817	0.71667	0.144681
DICER1	2	0.61319	0.845293	0.072993	*TASOR2*	4	-0.21824	0.717044	0.144454
CXXC1	4	0.23408	0.845422	0.072926	*POF1B*	4	-0.24973	0.718413	0.143626
POC1A	3	0.44152	0.845422	0.072926	*DNLZ*	4	0.27998	0.718801	0.143391
ADCY7	3	0.3265	0.845599	0.072835	*HAMP*	2	-0.48857	0.71883	0.143374
BATF	4	-0.41059	0.845777	0.072744	*CMC4*	3	-0.42759	0.71883	0.143374
HCN2	4	-0.36353	0.846109	0.072574	*TBC1D4*	4	-0.32876	0.71883	0.143374
SBK3	3	-0.2009	0.846109	0.072574	*TSPO*	3	-0.31241	0.71883	0.143374
SPEF1	4	0.032182	0.846109	0.072574	*EPHA3*	4	-0.31195	0.71883	0.143374
DALRD3	2	0.17521	0.846109	0.072574	*ANTXR2*	4	-0.16591	0.71883	0.143374
MMP2	2	0.12927	0.846188	0.072533	*PLEKHH2*	3	-0.066805	0.71883	0.143374
PLCD1	1	0.27886	0.846188	0.072533	*SERPINA4*	4	-0.022342	0.71883	0.143374
CACNG8	2	0.4843	0.846188	0.072533	*ZC2HC1A*	4	0.018699	0.71883	0.143374
DPP4	3	-0.51666	0.846492	0.072377	*FAM124A*	3	0.02141	0.71883	0.143374
TMEM17	4	0.099963	0.846492	0.072377	*SLC7A8*	2	0.21894	0.71883	0.143374
BCDIN3D	1	0.57282	0.846492	0.072377	*COL8A1*	4	0.24275	0.71883	0.143374
PJA1	2	-0.33639	0.846845	0.072196	*XPR1*	4	0.34638	0.71883	0.143374
PLBD1	2	0.31868	0.846845	0.072196	*CLIP2*	3	0.38421	0.71883	0.143374
CCDC151	4	-0.03938	0.846872	0.072182	*IQCH*	3	0.45855	0.71883	0.143374
POLG2	3	-0.54784	0.84713	0.07205	*RIMS4*	2	0.47875	0.71883	0.143374
FOXRED1	3	-0.47973	0.84713	0.07205	*C18orf65*	4	-0.2112	0.719608	0.142904
NEURL1B	3	-0.3764	0.84713	0.07205	*C1QL4*	4	-0.33624	0.719799	0.142789
TMLHE	3	-0.36184	0.84713	0.07205	*CPA6*	4	-0.43132	0.720175	0.142562
NPC1L1	3	-0.009627	0.84713	0.07205	*LIX1*	4	-0.32442	0.720175	0.142562
LINC01620	3	0.39205	0.84713	0.07205	*CLIP1*	4	-0.26842	0.720175	0.142562
EIF3D	3	0.62843	0.84713	0.07205	*KCTD17*	4	-0.24606	0.720175	0.142562
CD300C	4	-0.49105	0.847142	0.072044	*KLHL15*	3	-0.23569	0.720175	0.142562
C1QTNF9	1	-0.47734	0.847142	0.072044	*ALDH1L1*	3	-0.11826	0.720175	0.142562
XK	4	-0.46327	0.847142	0.072044	*SLC35A3*	4	0.080368	0.720175	0.142562
DNAJC22	4	-0.42883	0.847142	0.072044	*ETAA1*	3	-0.2454	0.720495	0.142369
UBE2D2	3	-0.40073	0.847142	0.072044	*TMEM158*	4	-0.091004	0.720495	0.142369
MMP28	2	-0.30549	0.847142	0.072044	*PDPN*	2	0.12523	0.720495	0.142369
C10orf99	4	-0.29278	0.847142	0.072044	*TMC2*	2	0.24554	0.720495	0.142369
AHSA1	4	-0.19392	0.847142	0.072044	*TAC4*	3	0.50673	0.720495	0.142369
PLEKHH2	3	-0.19312	0.847142	0.072044	*DDX18*	3	0.54048	0.720495	0.142369
FSTL5	4	0.3456	0.847142	0.072044	*PCED1B*	4	0.16398	0.720813	0.142178
RUNDC3A	2	0.37992	0.847142	0.072044	*RNF11*	4	-0.28227	0.720943	0.142099
ELOVL6	3	0.48698	0.847142	0.072044	*FBXL6*	3	0.27891	0.721244	0.141918
ZNF79	3	-0.74623	0.847373	0.071925	*C1orf116*	3	0.33592	0.721244	0.141918
DCP2	4	-0.36438	0.847373	0.071925	*SSBP3*	2	0.42658	0.721244	0.141918
TSEN54	4	-0.33058	0.847373	0.071925	*EGF*	3	0.082024	0.721621	0.141691
BSPRY	4	-0.22413	0.847373	0.071925	*INPP1*	2	0.28169	0.722125	0.141388
JMY	4	-0.21599	0.847373	0.071925	*ZMAT5*	4	0.28079	0.722519	0.141151
CAPN1	3	0.39671	0.847373	0.071925	*YTHDF1*	1	0.48353	0.722519	0.141151
CBY3	4	-0.29991	0.847623	0.071797	*ADORA1*	4	-0.00437	0.722759	0.141007
HOXC12	4	-0.18252	0.848246	0.071478	*CERK*	4	0.14012	0.722759	0.141007
TTC26	3	-0.47502	0.848376	0.071411	*TAS1R1*	3	0.19446	0.722759	0.141007
TOMM7	4	-0.20346	0.848376	0.071411	*OPTN*	4	-0.21174	0.723523	0.140548
CCNB1IP1	4	0.074981	0.848376	0.071411	*IPMK*	4	-0.46335	0.72439	0.140028
SIGMAR1	4	0.17351	0.848376	0.071411	*CXXC4*	4	-0.36148	0.72439	0.140028
GPR61	2	0.19639	0.848376	0.071411	*CES4A*	4	-0.30284	0.72439	0.140028
IL10RB	4	0.30252	0.848376	0.071411	*CRISPLD2*	4	-0.25242	0.72439	0.140028
ZNF658	2	0.38355	0.848376	0.071411	*SYTL1*	4	0.24852	0.72439	0.140028
ZDBF2	3	0.42108	0.848376	0.071411	*PCSK2*	4	0.2807	0.72439	0.140028
PGGHG	4	-0.48567	0.848978	0.071103	*RBM42*	2	0.36203	0.72439	0.140028
NMB	2	-0.36486	0.848978	0.071103	*HEBP2*	3	-0.77339	0.724943	0.139696
FAM43A	4	-0.27194	0.848978	0.071103	*ERICH5*	3	-0.31954	0.725444	0.139396

HRH3	3	-0.11853	0.848978	0.071103	*TMEM39A*	4	-0.27395	0.725444	0.139396
RIC8A	3	0.32235	0.848978	0.071103	*ZYX*	4	-0.25832	0.725444	0.139396
HOOK3	3	0.35012	0.848978	0.071103	*CCDC149*	4	-0.12833	0.725444	0.139396
RPP38	3	0.38624	0.848978	0.071103	*CNNM2*	4	0.16133	0.725827	0.139167
NT5DC3	3	0.41504	0.848978	0.071103	*H3C4*	3	0.38353	0.725827	0.139167
CHADL	2	0.57426	0.848978	0.071103	*PXN*	4	0.10796	0.726246	0.138916
MAGT1	3	-0.26961	0.849551	0.070811	*NPNT*	3	-0.38239	0.726857	0.138551
SRGAP3	4	-0.2061	0.849551	0.070811	*C20orf194*	3	-0.31485	0.726857	0.138551
SF1	4	-0.12853	0.849551	0.070811	*CCDC181*	4	0.11236	0.726857	0.138551
ORC1	4	0.42306	0.849551	0.070811	*ARMCX4*	3	0.17871	0.726857	0.138551
CLSTN3	3	0.42802	0.849551	0.070811	*EHD4*	4	0.21678	0.726857	0.138551
ASTE1	1	-0.45395	0.849678	0.070745	*XG*	3	-0.36171	0.726992	0.13847
ZNF287	2	-0.38826	0.849678	0.070745	*PPP1R14B*	2	0.1939	0.727055	0.138433
STUB1	3	0.036329	0.849678	0.070745	*PAIP2*	2	0.51811	0.727055	0.138433
ANKRD46	3	0.17075	0.849678	0.070745	*SDR9C7*	3	-0.15886	0.727313	0.138279
MUC5AC	4	0.2473	0.849678	0.070745	*PTPN18*	3	0.29384	0.727313	0.138279
NSF	4	0.40685	0.849678	0.070745	*UBE4B*	3	0.40928	0.727313	0.138279
TCP1	3	0.54031	0.849678	0.070745	*ROMO1*	2	-0.80211	0.727567	0.138127
NOL9	3	0.56756	0.849678	0.070745	*TMEM64*	2	-0.37993	0.727567	0.138127
FBXW9	4	0.27304	0.850667	0.07024	*ZNF461*	4	-0.23484	0.727567	0.138127
TMC8	3	0.38159	0.851211	0.069963	*MGP*	3	0.014515	0.727567	0.138127
PSRC1	3	-0.47035	0.851583	0.069773	*SECTM1*	2	0.089527	0.727567	0.138127
UNC13A	4	-0.45524	0.851583	0.069773	*CTSB*	2	0.31218	0.727567	0.138127
DYM	4	-0.4549	0.851583	0.069773	*CCN5*	4	0.17357	0.727579	0.13812
ACADL	4	-0.43358	0.851583	0.069773	*USP22*	4	0.20915	0.727579	0.13812
NAT8	4	-0.43271	0.851583	0.069773	*RPS15*	1	0.83503	0.727579	0.13812
DAPK2	3	-0.36719	0.851583	0.069773	*LRRC46*	4	-0.24094	0.727745	0.13802
HOXB6	3	-0.34845	0.851583	0.069773	*SLC25A27*	4	-0.16235	0.727745	0.13802
ABCA12	4	-0.31801	0.851583	0.069773	*UXT*	3	0.47356	0.727769	0.138006
PNMT	4	-0.31293	0.851583	0.069773	*SGTB*	4	0.039618	0.727817	0.137978
BEND5	3	-0.3081	0.851583	0.069773	*STYXL1*	4	0.13833	0.727817	0.137978
DACH2	4	-0.2949	0.851583	0.069773	*TRMT2B*	2	0.055291	0.728314	0.137682
DCUN1D4	2	-0.28528	0.851583	0.069773	*PCDHA4*	4	-0.22974	0.728989	0.137279
FAS	4	-0.27014	0.851583	0.069773	*ADAT1*	4	-0.029719	0.728989	0.137279
FRRS1L	4	-0.25791	0.851583	0.069773	*GNA14*	4	-0.351	0.729192	0.137158
MUC1	4	-0.25092	0.851583	0.069773	*ZC3H12B*	4	-0.20796	0.729192	0.137158
MFAP3	4	-0.21228	0.851583	0.069773	*CSNK1G2*	3	0.2436	0.729192	0.137158
ONECUT1	4	-0.21057	0.851583	0.069773	*SP3*	4	-0.19026	0.729651	0.136885
S1PR5	3	-0.13736	0.851583	0.069773	*STX1A*	3	0.094581	0.729681	0.136867
STAG1	3	-0.094375	0.851583	0.069773	*PAN3*	4	-0.32488	0.72969	0.136862
SLC2A7	3	-0.052832	0.851583	0.069773	*PPIL2*	4	0.33144	0.730377	0.136453
DNAH6	4	-0.037617	0.851583	0.069773	*ECE1*	4	-0.17733	0.730585	0.136329
CDA	4	-0.037394	0.851583	0.069773	*PSMD8*	4	-0.041325	0.730585	0.136329
SETD4	4	-0.021264	0.851583	0.069773	*MCIDAS*	4	-0.31314	0.730655	0.136288
SLC25A53	4	0.014942	0.851583	0.069773	*TRIM3*	3	0.089498	0.730655	0.136288
SUMO4	4	0.10708	0.851583	0.069773	*PA2G4*	3	0.11036	0.730655	0.136288
MRPS21	3	0.1331	0.851583	0.069773	*E2F1*	4	0.037864	0.730741	0.136237
FUT5	2	0.13412	0.851583	0.069773	*INTS6L*	3	0.29175	0.730741	0.136237
HAPLN1	3	0.13635	0.851583	0.069773	*MLH1*	3	0.29707	0.730741	0.136237
YAF2	4	0.13876	0.851583	0.069773	*NBPF8*	3	0.38517	0.730741	0.136237
CREB3L1	4	0.15846	0.851583	0.069773	*TFPI*	3	0.39656	0.730741	0.136237
MTF2	4	0.17353	0.851583	0.069773	*USP8*	3	0.53176	0.730741	0.136237
BUD23	4	0.18065	0.851583	0.069773	*SLC38A4*	2	0.28173	0.731727	0.135651
CRTC3	4	0.20395	0.851583	0.069773	*ZNF583*	4	-0.20855	0.732265	0.135331
IGFBP1	4	0.20404	0.851583	0.069773	*PDE6A*	4	0.092724	0.732265	0.135331
PRPF3	3	0.20425	0.851583	0.069773	*ARHGEF28*	4	-0.25882	0.732406	0.135248
ZNF518A	4	0.20754	0.851583	0.069773	*NBPF3*	2	-0.6304	0.732591	0.135138
MEIS3	4	0.222	0.851583	0.069773	*SEMA3F*	4	-0.33187	0.732591	0.135138

SIN3A	4	0.23223	0.851583	0.069773	*TBCA*	2	-0.25209	0.732591	0.135138
PRIMA1	4	0.24066	0.851583	0.069773	*SKP2*	3	0.81788	0.732591	0.135138
CCDC154	4	0.24252	0.851583	0.069773	*CST3*	4	-0.23831	0.732761	0.135037
SRRD	4	0.24501	0.851583	0.069773	*LOC730183*	3	0.46825	0.732761	0.135037
KLF1	4	0.2554	0.851583	0.069773	*PUM2*	3	0.47153	0.732761	0.135037
RAD21	4	0.26398	0.851583	0.069773	*CSPG5*	3	-0.58029	0.733019	0.134885
TMEM104	4	0.2665	0.851583	0.069773	*MYLK*	3	0.26985	0.733019	0.134885
HOXD12	4	0.27177	0.851583	0.069773	*LITAF*	4	0.090794	0.733152	0.134806
THAP11	4	0.27211	0.851583	0.069773	*TCEA3*	4	0.12921	0.733152	0.134806
AP3D1	4	0.28213	0.851583	0.069773	*NLRP12*	4	0.13646	0.733152	0.134806
DCP1A	4	0.29559	0.851583	0.069773	*N4BP1*	4	0.2609	0.733152	0.134806
NFRKB	3	0.29672	0.851583	0.069773	*PDE1B*	3	-0.30772	0.733887	0.134371
FIS1	4	0.29768	0.851583	0.069773	*KLHL42*	3	-0.21048	0.733887	0.134371
CCDC124	4	0.30634	0.851583	0.069773	*ISLR2*	4	-0.037266	0.733887	0.134371
HEG1	3	0.30832	0.851583	0.069773	*SLCO1B1*	4	0.12716	0.733887	0.134371
C17orf107	4	0.3115	0.851583	0.069773	*TMCC2*	4	0.14093	0.733887	0.134371
FREM2	3	0.33546	0.851583	0.069773	*RHOF*	4	0.151	0.733887	0.134371
KIAA1614	4	0.33669	0.851583	0.069773	*SLC28A2*	4	0.16343	0.733887	0.134371
NMU	3	0.33781	0.851583	0.069773	*PPIL3*	4	0.26766	0.733887	0.134371
HSD17B12	3	0.34913	0.851583	0.069773	*LAMTOR2*	4	0.30453	0.733887	0.134371
PRPF4B	4	0.38219	0.851583	0.069773	*SUFU*	3	0.47768	0.733887	0.134371
DNAJC2	4	0.40414	0.851583	0.069773	*ZNF17*	4	-0.17124	0.734062	0.134267
SEPTIN7	3	0.42872	0.851583	0.069773	*HJURP*	3	0.35669	0.734062	0.134267
CAPRIN2	3	0.44655	0.851583	0.069773	*FNDC5*	3	-0.61828	0.734618	0.133939
PDGFB	2	0.44821	0.851583	0.069773	*MED19*	3	-0.47576	0.734618	0.133939
CENPM	4	0.47714	0.851583	0.069773	*MYL6*	3	-0.46049	0.734618	0.133939
DNA2	4	0.48061	0.851583	0.069773	*EEF1D*	3	-0.39125	0.734618	0.133939
CHMP2A	2	0.70098	0.851583	0.069773	*ANO8*	2	-0.35242	0.734618	0.133939
DAD1	2	0.80788	0.851583	0.069773	*CLPTM1L*	4	-0.34291	0.734618	0.133939
CD58	4	-0.57131	0.851583	0.069773	*RUBCNL*	3	-0.33487	0.734618	0.133939
HIGD2A	4	-0.47336	0.851583	0.069773	*VTCN1*	3	-0.31956	0.734618	0.133939
PPP4R3A	4	-0.39745	0.851583	0.069773	*MAGEL2*	4	-0.3014	0.734618	0.133939
ARL5C	2	-0.35969	0.851583	0.069773	*METTL27*	3	-0.23222	0.734618	0.133939
ARVCF	4	-0.3468	0.851583	0.069773	*RAB26*	3	-0.2025	0.734618	0.133939
DIPK2A	4	-0.34343	0.851583	0.069773	*DDN*	4	-0.18256	0.734618	0.133939
CEP170	4	-0.34277	0.851583	0.069773	*HDAC7*	3	-0.16358	0.734618	0.133939
ABTB1	2	-0.31545	0.851583	0.069773	*NUMBL*	4	-0.16207	0.734618	0.133939
IL12A	4	-0.30304	0.851583	0.069773	*C17orf67*	3	-0.078237	0.734618	0.133939
TIGD3	4	-0.30004	0.851583	0.069773	*SMIM12*	4	-0.015157	0.734618	0.133939
ZSCAN21	4	-0.22054	0.851583	0.069773	*EPGN*	3	0.10252	0.734618	0.133939
DSE	4	-0.19811	0.851583	0.069773	*BCAS4*	3	0.12957	0.734618	0.133939
RIMBP2	4	-0.1771	0.851583	0.069773	*HCFC1*	2	0.14521	0.734618	0.133939
TRAM1	4	-0.15717	0.851583	0.069773	*SHH*	4	0.20087	0.734618	0.133939
H3C8	4	-0.15621	0.851583	0.069773	*SGCE*	4	0.2202	0.734618	0.133939
PIKFYVE	3	-0.1204	0.851583	0.069773	*STT3B*	2	0.27733	0.734618	0.133939
BST2	3	-0.095936	0.851583	0.069773	*DPP9-AS1*	2	0.3412	0.734618	0.133939
TFAM	4	-0.083553	0.851583	0.069773	*S100A13*	3	0.3522	0.734618	0.133939
PKIB	3	0.017477	0.851583	0.069773	*UBR5*	2	0.36908	0.734618	0.133939
ARMCX1	2	0.098152	0.851583	0.069773	*SPATA20*	2	0.44281	0.734618	0.133939
ISL2	4	0.12385	0.851583	0.069773	*KXD1*	1	0.46396	0.734618	0.133939
RIOK3	4	0.1626	0.851583	0.069773	*CD37*	2	0.51031	0.734618	0.133939
LGR4	4	0.17485	0.851583	0.069773	*PTPN13*	4	-0.26542	0.734814	0.133823
DPEP2NB	2	0.19235	0.851583	0.069773	*SLC29A1*	3	-0.22122	0.734864	0.133793
ABCG2	4	0.20298	0.851583	0.069773	*KLF11*	4	0.13166	0.734864	0.133793
ALG10	4	0.22428	0.851583	0.069773	*ABCC11*	3	0.14106	0.734864	0.133793
CIITA	4	0.23511	0.851583	0.069773	*ABR*	4	0.15149	0.734864	0.133793
KRT40	4	0.2423	0.851583	0.069773	*DUSP27*	3	0.20435	0.734864	0.133793
LUC7L	3	0.25021	0.851583	0.069773	*ZNF98*	3	0.23182	0.734864	0.133793

CHST12	4	0.25169	0.851583	0.069773	*MAP3K10*	3	0.24898	0.734864	0.133793
FRAT1	4	0.28156	0.851583	0.069773	*EDNRB*	3	0.26578	0.734864	0.133793
BTBD3	4	0.30216	0.851583	0.069773	*USP34*	3	0.27757	0.734864	0.133793
TWSG1	3	0.33257	0.851583	0.069773	*ZW10*	4	0.29088	0.734864	0.133793
VHL	4	0.59058	0.851583	0.069773	*GDI2*	3	0.31125	0.734864	0.133793
C4A	1	0.67632	0.851583	0.069773	*BRI3*	4	0.16841	0.73511	0.133648
NF2	2	0.70003	0.851583	0.069773	*CRYGS*	4	0.28109	0.73511	0.133648
FAM157B	1	-0.79791	0.851656	0.069736	*GSPT2*	4	-0.36205	0.736013	0.133115
NECAB3	4	-0.40644	0.851656	0.069736	*ZNF865*	3	-0.31827	0.736013	0.133115
SLC28A2	4	0.14211	0.851656	0.069736	*FZR1*	4	-0.2418	0.736013	0.133115
CSPG4	3	-0.15788	0.852693	0.069208	*DTNB*	4	-0.19014	0.736013	0.133115
MYH3	2	-0.25531	0.852861	0.069122	*LDLRAD4*	3	0.32747	0.736013	0.133115
THRAP3	4	-0.32526	0.853244	0.068927	*SIK2*	4	-0.13203	0.736475	0.132842
TBC1D24	4	-0.27955	0.853244	0.068927	*C22orf23*	4	0.19268	0.736475	0.132842
ITSN1	4	-0.16282	0.853244	0.068927	*SLC31A1*	4	0.28973	0.737753	0.132089
RC3H1	2	0.26418	0.853244	0.068927	*H2BC15*	2	0.12827	0.737803	0.132059
SELENOK	2	0.30003	0.853244	0.068927	*ARHGEF25*	4	0.068188	0.73807	0.131903
ARF5	3	0.37184	0.853244	0.068927	*F2R*	3	-0.3054	0.738408	0.131703
RIN2	3	0.38055	0.853244	0.068927	*NAP1L4*	2	-0.55952	0.738458	0.131674
C9orf57	2	-0.034317	0.854085	0.068499	*HEXIM1*	3	0.28101	0.739013	0.131348
MRPL48	3	0.33216	0.854085	0.068499	*ZFP36L1*	4	-0.46609	0.739332	0.13116
CLEC18A	2	-0.56941	0.85453	0.068273	*TRIM32*	4	-0.24409	0.739332	0.13116
NDUFA4	3	-0.46271	0.85453	0.068273	*LRRC20*	4	-0.022282	0.739332	0.13116
TMEM119	2	-0.43779	0.85453	0.068273	*USP14*	4	0.20973	0.739332	0.13116
SPAG6	4	-0.42275	0.85453	0.068273	*VAMP7*	3	0.28877	0.739332	0.13116
PIK3IP1	3	-0.40302	0.85453	0.068273	*PLEKHA7*	4	-0.28861	0.73967	0.130962
SYT4	4	-0.38106	0.85453	0.068273	*ZKSCAN7*	3	-0.073486	0.740306	0.130589
TAF7L	3	-0.36884	0.85453	0.068273	*SH3RF3*	4	0.087785	0.740306	0.130589
DMGDH	2	-0.30722	0.85453	0.068273	*KCNK7*	4	0.1041	0.740306	0.130589
PTPN5	4	-0.30589	0.85453	0.068273	*HES1*	4	0.11442	0.740306	0.130589
PLEKHM1	4	-0.30496	0.85453	0.068273	*SCOC*	4	0.13147	0.740306	0.130589
SLC26A11	4	-0.2997	0.85453	0.068273	*VEZF1*	3	0.13746	0.740306	0.130589
ARMCX3	4	-0.29225	0.85453	0.068273	*TFPT*	4	0.16291	0.740306	0.130589
CAPZA1	4	-0.28063	0.85453	0.068273	*SGTA*	3	0.21534	0.740306	0.130589
PTPRM	4	-0.27975	0.85453	0.068273	*TMEM47*	3	0.25068	0.740306	0.130589
ARSK	4	-0.27638	0.85453	0.068273	*NES*	3	0.25879	0.740306	0.130589
ADD3	4	-0.24407	0.85453	0.068273	*RBBP7*	3	-0.30922	0.740619	0.130405
C15orf54	2	-0.23373	0.85453	0.068273	*BICDL1*	3	0.12036	0.740619	0.130405
ULK2	4	-0.21153	0.85453	0.068273	*CPLANE2*	4	-0.39528	0.741171	0.130081
H1-0	4	-0.15648	0.85453	0.068273	*EPB41L3*	3	0.2306	0.741436	0.129926
LIPH	4	-0.15637	0.85453	0.068273	*STAT2*	4	0.022775	0.741916	0.129646
ESRP1	4	-0.14308	0.85453	0.068273	*MYCBP2*	4	-0.41088	0.742055	0.129564
ZNF747	4	-0.13575	0.85453	0.068273	*ANAPC7*	4	-0.30238	0.742055	0.129564
LINC01750	4	-0.11395	0.85453	0.068273	*TXNRD1*	3	-0.28406	0.742055	0.129564
LAMA5	3	-0.097401	0.85453	0.068273	*ZNF43*	4	-0.26186	0.742055	0.129564
PPM1H	3	-0.062994	0.85453	0.068273	*SLC39A13*	4	-0.23888	0.742055	0.129564
CHRNA6	4	-0.054687	0.85453	0.068273	*SV2A*	4	-0.23363	0.742055	0.129564
PROCA1	3	-0.053789	0.85453	0.068273	*MASP1*	4	-0.22994	0.742055	0.129564
HOMER2	3	-0.034695	0.85453	0.068273	*ZNF37A*	3	-0.024176	0.742055	0.129564
TOM1L1	4	-0.018712	0.85453	0.068273	*FAM110A*	4	0.002741	0.742055	0.129564
RELN	4	-0.017515	0.85453	0.068273	*NRBP1*	4	0.032667	0.742055	0.129564
OVOL2	4	0.03987	0.85453	0.068273	*SCNN1A*	4	0.067572	0.742055	0.129564
VPS13A	3	0.048754	0.85453	0.068273	*OTOA*	4	0.072413	0.742055	0.129564
MPP5	2	0.051316	0.85453	0.068273	*CFAP44*	4	0.092952	0.742055	0.129564
GDF9	4	0.13758	0.85453	0.068273	*PLEKHG4B*	4	0.10962	0.742055	0.129564
UBE2E1	4	0.15426	0.85453	0.068273	*UBE2D3*	4	0.15193	0.742055	0.129564
TPX2	4	0.15719	0.85453	0.068273	*ZBTB34*	4	0.15711	0.742055	0.129564
PCDHA8	2	0.18334	0.85453	0.068273	*CCN4*	4	0.17342	0.742055	0.129564

COLEC11	4	0.2067	0.85453	0.068273	*HELT*	4	0.18332	0.742055	0.129564
CILP2	4	0.23906	0.85453	0.068273	*TK1*	2	0.21221	0.742055	0.129564
CSNK2A1	4	0.2656	0.85453	0.068273	*SEC61G*	3	0.25806	0.742055	0.129564
TUBB2B	2	0.27183	0.85453	0.068273	*LRR1*	4	0.32237	0.742055	0.129564
H2BC9	3	0.27294	0.85453	0.068273	*LUC7L3*	4	0.34995	0.742055	0.129564
LRRC23	4	0.2863	0.85453	0.068273	*MAU2*	4	0.35685	0.742055	0.129564
ANGPTL6	4	0.28656	0.85453	0.068273	*SAPCD2*	2	0.38796	0.742055	0.129564
MAN2B1	4	0.29761	0.85453	0.068273	*MADCAM1*	3	0.39685	0.742055	0.129564
COX7B2	4	0.30078	0.85453	0.068273	*SP1*	3	0.40885	0.742055	0.129564
PUS10	2	0.31262	0.85453	0.068273	*SEC61A1*	4	0.43031	0.742055	0.129564
SHPRH	3	0.34791	0.85453	0.068273	*RPS24*	3	0.90532	0.742055	0.129564
NDRG3	2	0.35031	0.85453	0.068273	*PPP4R3B*	4	0.063606	0.742223	0.129466
LRR1	4	0.3731	0.85453	0.068273	*TMPRSS3*	4	0.16104	0.742223	0.129466
BORA	4	0.38201	0.85453	0.068273	*ZNF875*	4	-0.38937	0.742393	0.129366
C1QTNF4	3	0.42489	0.85453	0.068273	*CD1D*	4	-0.31814	0.742393	0.129366
SARS1	3	0.45048	0.85453	0.068273	*HCAR2*	4	-0.26721	0.742393	0.129366
RBMS2	1	0.47355	0.85453	0.068273	*OGG1*	4	-0.18419	0.742393	0.129366
BUB3	4	0.60966	0.85453	0.068273	*ELL*	4	0.18974	0.742393	0.129366
RPAP2	1	0.75643	0.85453	0.068273	*ARHGAP36*	4	0.0648	0.742916	0.12906
MTREX	4	-0.37804	0.854834	0.068118	*DCAF13*	4	0.21763	0.742916	0.12906
RAET1E	3	-0.10609	0.854834	0.068118	*CDC42*	1	-0.46715	0.743299	0.128837
STN1	4	0.22233	0.854834	0.068118	*MYO6*	4	0.1337	0.743299	0.128837
HHLA1	4	0.2963	0.854834	0.068118	*ADAMTS14*	4	0.20377	0.743299	0.128837
WASHC2C	1	0.30323	0.854834	0.068118	*ELMO2*	4	0.21819	0.743299	0.128837
LENG9	3	0.35741	0.854834	0.068118	*NR1D2*	4	0.22105	0.743299	0.128837
CDK9	1	0.89468	0.854834	0.068118	*KLF6*	4	-0.16615	0.743537	0.128698
SPNS2	3	-0.53366	0.854922	0.068074	*AUH*	4	0.032392	0.743537	0.128698
CREG2	2	-0.30149	0.854922	0.068074	*SRSF3*	3	0.68204	0.743584	0.12867
ETFBKMT	4	-0.084818	0.854922	0.068074	*ZXDC*	3	-0.5244	0.743799	0.128545
MRPS18A	4	0.10459	0.854922	0.068074	*FAM8A1*	3	-0.47184	0.743799	0.128545
SLC20A2	4	0.17448	0.854922	0.068074	*PTGER1*	3	-0.22103	0.743799	0.128545
FAM71F1	4	0.19283	0.854922	0.068074	*CCP110*	4	0.10574	0.743988	0.128434
PPP1R11	4	0.2167	0.854922	0.068074	*FSD1L*	3	-0.063282	0.744605	0.128074
ST3GAL2	3	0.32016	0.854922	0.068074	*POFUT1*	4	-0.060832	0.744605	0.128074
EEF1G	3	0.40013	0.854922	0.068074	*RNF157*	4	0.11139	0.744605	0.128074
GPR27	3	0.58404	0.854922	0.068074	*DDX31*	3	0.20569	0.744605	0.128074
TMEM38A	4	0.23516	0.85511	0.067978	*ZBTB22*	3	-0.30114	0.746119	0.127192
FEM1A	3	0.48086	0.85511	0.067978	*POLR2H*	2	0.26187	0.746119	0.127192
H3C4	3	-0.59391	0.855178	0.067943	*ZNF821*	2	0.3397	0.747135	0.126601
LDHA	4	-0.32136	0.855178	0.067943	*SLC50A1*	2	0.35997	0.747135	0.126601
MSMB	3	0.32649	0.855178	0.067943	*ANKRD28*	2	-0.28549	0.747324	0.126491
THNSL1	4	-0.31535	0.856757	0.067142	*SCFD1*	3	-0.41009	0.747722	0.12626
DSG3	4	0.070424	0.856757	0.067142	*PDZD11*	3	-0.33287	0.747722	0.12626
APTX	4	0.1077	0.856757	0.067142	*MAFB*	4	-0.24807	0.747722	0.12626
COG7	4	0.29473	0.856757	0.067142	*PLAC8*	4	-0.21682	0.747722	0.12626
ASAH1	4	-0.27835	0.856799	0.067121	*NR1I2*	4	-0.067677	0.747722	0.12626
ANKRD6	4	0.029053	0.856799	0.067121	*TMX3*	2	0.32403	0.747722	0.12626
ORAI2	3	0.13581	0.856799	0.067121	*RPL23A*	1	0.47871	0.747722	0.12626
FANCI	4	0.2547	0.856799	0.067121	*CAPZB*	4	-0.55462	0.74804	0.126075
DDX47	3	0.85064	0.856799	0.067121	*VAMP4*	3	-0.53179	0.74804	0.126075
H2BC6	2	-0.36737	0.857004	0.067017	*ZSCAN5A*	4	-0.27864	0.74804	0.126075
NTHL1	2	-0.3493	0.857004	0.067017	*SLC10A5*	4	-0.25907	0.74804	0.126075
NLRP11	3	-0.34656	0.857004	0.067017	*CPEB1*	4	-0.25687	0.74804	0.126075
UTRN	3	-0.28281	0.857004	0.067017	*ANXA7*	4	-0.23132	0.74804	0.126075
GABRA3	3	-0.22964	0.857004	0.067017	*LRRC61*	4	-0.098205	0.74804	0.126075
CFAP45	2	0.1214	0.857004	0.067017	*PANO1*	4	-0.085551	0.74804	0.126075
CDK7	3	0.51616	0.857027	0.067006	*ZC3H7B*	4	-0.003198	0.74804	0.126075
SLC38A6	3	-0.14886	0.85719	0.066923	*S100A9*	3	0.036219	0.74804	0.126075

RPLP1	2	0.4718	0.857566	0.066733	*PARP1*	4	0.14241	0.74804	0.126075
GTF2A2	3	-0.81448	0.857941	0.066543	*LACC1*	4	0.17052	0.74804	0.126075
HMBS	4	-0.38276	0.858032	0.066497	*LASP1*	4	0.18725	0.74804	0.126075
FOSL2	3	-0.36923	0.858032	0.066497	*HMMR*	4	0.1924	0.74804	0.126075
PCYOX1	4	-0.35368	0.858032	0.066497	*C2orf78*	3	0.19897	0.74804	0.126075
TNFRSF1A	4	-0.33426	0.858032	0.066497	*DIABLO*	4	0.21298	0.74804	0.126075
IL1B	4	-0.33186	0.858032	0.066497	*LILRB5*	4	0.25925	0.74804	0.126075
ADAR	4	-0.3315	0.858032	0.066497	*ZFC3H1*	3	0.28443	0.74804	0.126075
KLRD1	4	-0.29966	0.858032	0.066497	*FAM20B*	2	0.31356	0.74804	0.126075
PPP2R1A	4	-0.25725	0.858032	0.066497	*ZXDA*	3	-0.3925	0.748932	0.125557
CCSAP	4	-0.2225	0.858032	0.066497	*DAPK2*	3	-0.3798	0.749	0.125518
CCNB3	4	-0.19326	0.858032	0.066497	*CLIC1*	4	0.16697	0.749	0.125518
TNFRSF13B	3	-0.19306	0.858032	0.066497	*PGGT1B*	4	0.53492	0.749	0.125518
PRMT9	3	-0.14651	0.858032	0.066497	*NDEL1*	2	-0.4512	0.749323	0.125331
MYRIP	4	-0.024924	0.858032	0.066497	*RARG*	4	-0.40737	0.749323	0.125331
WIPF1	3	0.019266	0.858032	0.066497	*FRS3*	4	-0.37466	0.749323	0.125331
ZNF540	4	0.04173	0.858032	0.066497	*B3GALT5*	2	-0.121	0.749323	0.125331
ARMH1	3	0.067289	0.858032	0.066497	*SOSTDC1*	4	-0.10893	0.749323	0.125331
VSTM5	4	0.090066	0.858032	0.066497	*FCN3*	4	-0.049985	0.749323	0.125331
NDUFS5	4	0.1123	0.858032	0.066497	*ODF2L*	4	0.20025	0.749323	0.125331
FLT3	4	0.11286	0.858032	0.066497	*ACTR1B*	2	-0.3546	0.749415	0.125278
ARFGAP2	4	0.16707	0.858032	0.066497	*PRRX1*	4	0.16491	0.749415	0.125278
TSPYL6	4	0.17955	0.858032	0.066497	*TRAP1*	4	-0.42989	0.749632	0.125152
CD3D	4	0.18087	0.858032	0.066497	*ZFAND1*	4	-0.36162	0.749632	0.125152
GPAA1	4	0.1896	0.858032	0.066497	*KCTD9*	3	-0.29942	0.749632	0.125152
TFG	4	0.19686	0.858032	0.066497	*C12orf57*	4	-0.19619	0.749632	0.125152
CYP1A1	4	0.22252	0.858032	0.066497	*MPP3*	4	-0.19308	0.749632	0.125152
ZNF101	4	0.22343	0.858032	0.066497	*CACNA1E*	4	-0.17251	0.749632	0.125152
ZNF112	4	0.25428	0.858032	0.066497	*ATG16L2*	4	-0.11859	0.749632	0.125152
ETV2	4	0.27485	0.858032	0.066497	*CBLB*	4	-0.023579	0.749632	0.125152
TGM7	4	0.2891	0.858032	0.066497	*GRK4*	4	0.014192	0.749632	0.125152
GPR150	4	0.30746	0.858032	0.066497	*UGT8*	4	0.021015	0.749632	0.125152
TRAPPC6A	4	0.31674	0.858032	0.066497	*CDK5RAP2*	4	0.038451	0.749632	0.125152
PMPCA	4	0.32981	0.858032	0.066497	*MORN1*	4	0.041979	0.749632	0.125152
RND1	3	0.33358	0.858032	0.066497	*UBAP1*	4	0.10634	0.749632	0.125152
C11orf58	3	0.40425	0.858032	0.066497	*IZUMO4*	4	0.17374	0.749632	0.125152
SYNPO2L	3	0.5187	0.858032	0.066497	*ICAM3*	3	0.19746	0.749632	0.125152
POLR3H	3	0.54742	0.858032	0.066497	*IRX4*	4	0.21309	0.749632	0.125152
STK19	4	-0.29494	0.858146	0.066439	*CHMP3*	1	0.39114	0.749632	0.125152
RPRML	4	0.007932	0.858146	0.066439	*BLK*	4	0.22827	0.749794	0.125058
ADAMTSL3	3	-0.52809	0.858167	0.066428	*RASGEF1A*	4	0.25398	0.749794	0.125058
DNTT	2	-0.37535	0.858185	0.066419	*SZT2*	3	-0.48556	0.750162	0.124845
EXTL1	4	-0.092538	0.858185	0.066419	*PPP1R15A*	3	-0.17003	0.750162	0.124845
ZBTB6	4	0.034523	0.858185	0.066419	*TMEM41B*	4	0.020537	0.750162	0.124845
PSMB2	4	0.62063	0.858185	0.066419	*OIP5*	3	0.038853	0.750162	0.124845
NTNG2	4	0.017587	0.858487	0.066266	*DNAH8*	3	0.36498	0.750162	0.124845
SLC39A1	2	0.26146	0.859068	0.065973	*ARHGAP11A*	1	-0.64092	0.750298	0.124766
TEPSIN	2	-0.59684	0.859203	0.065904	*DYRK3*	2	-0.55039	0.750298	0.124766
FZD2	4	-0.23739	0.859203	0.065904	*LZTR1*	3	0.17767	0.750298	0.124766
CDC14B	3	0.26558	0.859203	0.065904	*SOGA3*	1	-0.60807	0.750739	0.124511
ZKSCAN4	4	-0.27731	0.859644	0.065682	*TUBA3D*	3	-0.49066	0.750739	0.124511
PPP1R13B	4	-0.25151	0.860294	0.065353	*SLCO1C1*	3	0.062808	0.750924	0.124404
TTLL12	4	-0.47876	0.861013	0.06499	*ATP6V0E2*	2	-0.5547	0.751016	0.124351
CPLANE2	4	-0.31275	0.861244	0.064874	*NSD1*	4	-0.53767	0.751016	0.124351
RRAS2	4	-0.23936	0.861365	0.064813	*TCTEX1D2*	3	-0.43438	0.751016	0.124351
OC10050554.	4	-0.2064	0.861365	0.064813	*SLC35G1*	4	-0.42366	0.751016	0.124351
FAR1	4	-0.15542	0.861365	0.064813	*CORO1B*	4	-0.41593	0.751016	0.124351
ZNF3	4	0.16586	0.861365	0.064813	*BPGM*	4	-0.38343	0.751016	0.124351

SDC3	4	0.19311	0.861365	0.064813	*ACOT13*	4	-0.35548	0.751016	0.124351
QKI	4	0.21141	0.861365	0.064813	*PACSIN1*	4	-0.31531	0.751016	0.124351
ATCAY	4	0.27126	0.861365	0.064813	*UCKL1*	4	-0.264	0.751016	0.124351
ZRSR2	4	0.31021	0.861365	0.064813	*CPZ*	4	-0.23918	0.751016	0.124351
DENND5A	3	0.37197	0.861365	0.064813	*PALLD*	4	-0.23841	0.751016	0.124351
PDE5A	2	-0.47009	0.861388	0.064801	*ADGRF2*	4	-0.23006	0.751016	0.124351
PLCD4	3	-0.43481	0.861388	0.064801	*TRIM62*	4	-0.20932	0.751016	0.124351
ATP5MC3	3	-0.38585	0.861388	0.064801	*PRICKLE4*	4	-0.14894	0.751016	0.124351
TCF12	4	-0.27937	0.861388	0.064801	*PTPN5*	4	-0.086183	0.751016	0.124351
TRPV2	4	-0.14174	0.861388	0.064801	*KANK1*	4	-0.044538	0.751016	0.124351
MTMR4	3	-0.06357	0.861388	0.064801	*ABCA2*	1	0.39585	0.751016	0.124351
GSS	4	0.044425	0.861388	0.064801	*RNF149*	3	0.39615	0.751016	0.124351
GCNT1	4	0.1815	0.861388	0.064801	*TRIM9*	3	0.43117	0.751016	0.124351
FLNA	4	0.21465	0.861388	0.064801	*CANT1*	2	-0.64104	0.75156	0.124037
GSTM2	3	0.30601	0.861388	0.064801	*NDUFA4L2*	4	-0.41224	0.75156	0.124037
ADGRL3	3	0.37818	0.861388	0.064801	*CHST7*	4	-0.39424	0.75156	0.124037
OTUD7B	3	-0.27305	0.861888	0.064549	*SPAG16*	4	-0.36823	0.75156	0.124037
CNGB1	4	-0.047061	0.861888	0.064549	*PHETA2*	3	-0.35305	0.75156	0.124037
PCDHAC1	4	0.17414	0.861888	0.064549	*POU2F3*	4	-0.33993	0.75156	0.124037
MATR3	4	0.19453	0.861888	0.064549	*CFAP221*	2	-0.31687	0.75156	0.124037
SERPINB6	2	0.20653	0.861909	0.064538	*GDNF-AS1*	2	-0.31428	0.75156	0.124037
SPAG1	3	-0.51486	0.862381	0.064301	*PLP2*	4	-0.21318	0.75156	0.124037
BPGM	4	-0.31977	0.862381	0.064301	*APOL1*	4	-0.19861	0.75156	0.124037
ALDH4A1	3	-0.31828	0.862381	0.064301	*FGF17*	3	-0.14423	0.75156	0.124037
TAS2R14	3	-0.31476	0.862381	0.064301	*KLRG2*	4	-0.097659	0.75156	0.124037
ECEL1	4	-0.10821	0.862381	0.064301	*SESN1*	4	-0.09571	0.75156	0.124037
ASTN1	4	0.17783	0.862381	0.064301	*PLLP*	4	-0.055216	0.75156	0.124037
ZNF48	4	-0.25201	0.86261	0.064185	*KIAA0930*	4	0.051764	0.75156	0.124037
ACRV1	4	0.29313	0.862631	0.064175	*FABP6*	4	0.12617	0.75156	0.124037
KCNIP2	2	0.47416	0.862721	0.06413	*PTAR1*	4	0.13027	0.75156	0.124037
AMACR	2	-0.45444	0.862852	0.064064	*IGFL4*	4	0.16141	0.75156	0.124037
RAB26	3	-0.225	0.862852	0.064064	*PRR35*	2	0.176	0.75156	0.124037
PPP1CA	4	0.2626	0.862852	0.064064	*MAP3K15*	4	0.20236	0.75156	0.124037
ZNF346	4	0.056295	0.862914	0.064032	*GNG3*	4	0.22018	0.75156	0.124037
LRRC28	4	0.24331	0.862914	0.064032	*PCDHB12*	2	0.23209	0.75156	0.124037
ZNF492	1	0.78202	0.862914	0.064032	*SERPINB7*	3	0.28726	0.75156	0.124037
SOX6	3	-0.37189	0.863073	0.063952	*WRAP53*	2	0.30296	0.75156	0.124037
SIL1	1	-0.56089	0.863888	0.063543	*PRPH2*	3	0.36338	0.75156	0.124037
SLC13A2	4	-0.44733	0.863888	0.063543	*DDX5*	3	0.54047	0.75156	0.124037
CHRDL1	4	-0.26493	0.863888	0.063543	*HDDC2*	3	-0.48292	0.7516	0.124013
RP9	4	-0.25965	0.863888	0.063543	*VPS54*	3	-0.37449	0.7516	0.124013
CEP250	4	-0.16042	0.863888	0.063543	*LIMD1*	4	-0.3432	0.7516	0.124013
CTDSPL	4	0.20778	0.863888	0.063543	*TRPM8*	3	-0.18257	0.7516	0.124013
DPAGT1	3	0.37833	0.864115	0.063428	*SEC14L4*	4	-0.12213	0.7516	0.124013
HCFC2	2	0.31005	0.864895	0.063036	*PCDHGB3*	4	-0.020825	0.7516	0.124013
CNTROB	3	-0.39201	0.86547	0.062748	*DDX39A*	4	0.032749	0.7516	0.124013
EVI2B	4	-0.37077	0.86547	0.062748	*NT5DC2*	4	0.05934	0.7516	0.124013
PACS2	3	-0.36099	0.86547	0.062748	*ACOT9*	4	0.08153	0.7516	0.124013
EIF4E2	3	-0.32873	0.86547	0.062748	*MPV17L2*	4	0.10574	0.7516	0.124013
FAM169A	4	-0.30566	0.86547	0.062748	*COLEC10*	4	0.16736	0.7516	0.124013
MGRN1	4	-0.27328	0.86547	0.062748	*FAM83D*	4	0.21461	0.7516	0.124013
PIK3C2B	4	-0.086864	0.86547	0.062748	*FBLIM1*	4	0.2458	0.7516	0.124013
DIS3	4	0.31347	0.86547	0.062748	*TSPAN1*	4	0.27208	0.7516	0.124013
TMEM86A	2	-0.27403	0.866357	0.062303	*SYT14*	4	0.28066	0.7516	0.124013
LAD1	4	-0.21403	0.866357	0.062303	*SETX*	3	0.33012	0.7516	0.124013
PLAAT3	1	0.45062	0.866357	0.062303	*PITPNM1*	4	0.35194	0.7516	0.124013
MUC21	3	-0.30699	0.866485	0.062239	*DCAF10*	3	0.47919	0.7516	0.124013
SHISA5	4	-0.1917	0.866485	0.062239	*PCID2*	4	0.50247	0.7516	0.124013

NUDT19	3	0.36015	0.866485	0.062239	*LSM7*	2	0.50734	0.7516	0.124013
NOX1	3	-0.51158	0.866731	0.062115	*NF2*	2	0.56345	0.7516	0.124013
TMEM98	4	-0.31789	0.866731	0.062115	*ENPEP*	3	0.24003	0.751758	0.123922
KCTD20	4	-0.30543	0.866731	0.062115	*C1QTNF4*	3	0.36212	0.751758	0.123922
TTC7B	4	-0.2426	0.866731	0.062115	*ZNF490*	4	-0.003434	0.752122	0.123712
TDRD3	4	-0.21804	0.866731	0.062115	*CTRL*	4	0.21055	0.752122	0.123712
PRKCE	4	-0.18501	0.866731	0.062115	*SF3A1*	3	0.42729	0.752304	0.123607
RBM34	4	0.28608	0.866731	0.062115	*OLFML2B*	4	-0.14676	0.75276	0.123343
UGT2A2	1	0.51211	0.866731	0.062115	*METTL24*	2	0.23046	0.75333	0.123015
RPS8	1	1.0346	0.866731	0.062115	*STUM*	2	0.23345	0.75333	0.123015
TXLNA	4	-0.29506	0.867183	0.061889	*TM9SF2*	3	-0.30404	0.753443	0.12295
KCNRG	3	0.30048	0.867183	0.061889	*AGXT*	3	0.31071	0.753899	0.122687
ARNTL2	2	-0.12157	0.867683	0.061639	*TRIP6*	3	-0.50542	0.754973	0.122069
CYP2A6	3	-0.51293	0.868595	0.061182	*ZNF382*	3	-0.41821	0.754973	0.122069
JADE1	3	0.034653	0.869439	0.060761	*GPD1*	3	-0.39639	0.754973	0.122069
TMEM65	3	-0.27744	0.869801	0.06058	*AGO3*	3	-0.34631	0.754973	0.122069
GNAL	3	-0.40579	0.869896	0.060533	*NHS*	3	-0.32446	0.754973	0.122069
PCDH9	3	-0.35261	0.869896	0.060533	*UBXN7*	3	-0.28968	0.754973	0.122069
JAG2	3	0.072696	0.869896	0.060533	*DIAPH2*	3	-0.22697	0.754973	0.122069
PROZ	2	0.37585	0.869896	0.060533	*CCER2*	4	-0.002562	0.754973	0.122069
EEF1AKMT2	2	0.45121	0.869896	0.060533	*HTRA4*	4	0.008209	0.754973	0.122069
C15orf65	2	0.23163	0.87004	0.060461	*TMOD3*	3	0.011367	0.754973	0.122069
ZNF641	2	0.25954	0.87004	0.060461	*POLR3G*	3	0.036324	0.754973	0.122069
NPW	3	0.36228	0.87004	0.060461	*SPRY3*	4	0.15353	0.754973	0.122069
NCKAP1	2	0.58512	0.87004	0.060461	*ZNF806*	4	-0.25858	0.755085	0.122004
AMZ1	3	-0.76969	0.870744	0.060109	*SLC9A4*	3	0.04896	0.755129	0.121979
SORCS2	3	-0.50554	0.870744	0.060109	*FBXO39*	4	0.018037	0.75567	0.121668
TMOD1	3	-0.4235	0.870744	0.060109	*PRDM8*	3	0.28529	0.75567	0.121668
HLA-A	3	-0.36875	0.870744	0.060109	*TSR2*	3	0.37353	0.75567	0.121668
DCTD	3	-0.36519	0.870744	0.060109	*ZDHHC11*	4	-0.34359	0.755782	0.121604
KRT33A	3	-0.36072	0.870744	0.060109	*PPHLN1*	4	-0.28601	0.755818	0.121583
CACTIN	3	-0.34859	0.870744	0.060109	*ANKRD26*	4	-0.23345	0.755818	0.121583
CDHR4	4	-0.30793	0.870744	0.060109	*HACD1*	4	-0.18469	0.755818	0.121583
TNFSF14	4	-0.2222	0.870744	0.060109	*C2orf68*	3	0.37879	0.755818	0.121583
OR11H4	3	0.005472	0.870744	0.060109	*ARHGEF11*	4	-0.26995	0.755998	0.121479
DRAM1	3	0.055036	0.870744	0.060109	*GLA*	1	-0.56118	0.75671	0.12107
PDCD10	3	0.1804	0.870744	0.060109	*SFTA2*	4	-0.35073	0.75671	0.12107
C17orf80	4	-0.5368	0.870937	0.060013	*MAN1C1*	4	-0.25851	0.75671	0.12107
HERC3	4	-0.22198	0.870937	0.060013	*ZNF134*	4	-0.2006	0.75671	0.12107
MCRS1	3	0.37762	0.870937	0.060013	*TIMM21*	3	-0.14785	0.75671	0.12107
GPCPD1	4	-0.28979	0.871384	0.05979	*NSUN6*	4	-0.075932	0.75671	0.12107
PIK3R1	4	-0.25553	0.871384	0.05979	*HBP1*	4	0.032475	0.75671	0.12107
MEDAG	3	-0.445	0.872227	0.059371	*KLRD1*	4	-0.29374	0.756933	0.120943
ESYT2	3	-0.39575	0.872227	0.059371	*RUSC1*	4	-0.029435	0.756933	0.120943
C3orf35	3	-0.20885	0.872227	0.059371	*TFPI2*	4	0.18932	0.757223	0.120776
SERTAD4	3	-0.086564	0.872227	0.059371	*TNFRSF19*	4	0.20176	0.757223	0.120776
EXOSC3	2	0.35083	0.872227	0.059371	*COG2*	3	0.34804	0.757266	0.120752
ST20	2	-0.37888	0.873444	0.058765	*RAB11FIP2*	4	0.063588	0.757513	0.12061
ADGRE1	4	-0.08944	0.873444	0.058765	*BNIP3*	2	0.44227	0.757624	0.120546
CENPT	4	0.19319	0.873444	0.058765	*ROR1*	1	-0.47262	0.757803	0.120443
HMX2	4	0.28754	0.873444	0.058765	*FGFRL1*	3	-0.083724	0.758187	0.120224
TMX3	2	0.29718	0.873444	0.058765	*METTL26*	3	0.39954	0.758706	0.119926
NKPD1	2	0.32635	0.873444	0.058765	*DENND5B*	4	0.1145	0.758817	0.119863
ODF2L	4	0.32968	0.873444	0.058765	*COLEC11*	4	0.14411	0.759174	0.119659
CTTNBP2	4	-0.19039	0.87353	0.058722	*LRIG2*	4	0.15735	0.759174	0.119659
SP110	3	-0.57661	0.873532	0.058721	*MUC15*	4	-0.13831	0.759437	0.119508
NONO	3	-0.48834	0.873532	0.058721	*NDUFAF2*	3	0.14213	0.759437	0.119508
GUCY1A1	3	-0.40015	0.873532	0.058721	*HNRNPAB*	4	0.26535	0.759437	0.119508

CASC3	3	-0.37307	0.873532	0.058721	*RAB4A*	6	-0.26939	0.76004	0.119163
RUNX3	4	-0.34509	0.873532	0.058721	*EVL*	4	0.055838	0.76004	0.119163
BTN3A2	3	-0.2904	0.873532	0.058721	*GIMAP2*	4	0.22979	0.76004	0.119163
TMEM243	4	-0.25368	0.873532	0.058721	*NIPSNAP1*	4	-0.049975	0.76049	0.118906
CCDC62	3	-0.21782	0.873532	0.058721	*FCGRT*	3	-0.49092	0.760532	0.118882
BMP7	4	-0.20632	0.873532	0.058721	*ACADSB*	4	-0.045941	0.760904	0.11867
LGALS9	3	-0.17824	0.873532	0.058721	*GATA6*	4	0.1288	0.760904	0.11867
ASAP3	2	-0.14549	0.873532	0.058721	*FGFR1OP*	4	0.13575	0.760904	0.11867
OR1M1	4	-0.065245	0.873532	0.058721	*MFSD2B*	3	0.19627	0.760904	0.11867
DEPTOR	4	-0.050668	0.873532	0.058721	*RSPH14*	4	-0.39865	0.762047	0.118018
EIF4H	4	-0.027465	0.873532	0.058721	*GTPBP2*	4	-0.21462	0.762047	0.118018
TUT7	4	0.20671	0.873532	0.058721	*RIDA*	2	0.26846	0.762047	0.118018
SPEGNB	4	0.20836	0.873532	0.058721	*TSNAX*	1	-0.63181	0.762146	0.117962
GATA2	3	0.24	0.873532	0.058721	*DNAJB14*	3	-0.50785	0.762146	0.117962
CFAP221	2	0.24446	0.873532	0.058721	*PTK2B*	4	-0.26195	0.762146	0.117962
CCND3	4	0.27677	0.873532	0.058721	*FOXL2NB*	3	0.38785	0.762146	0.117962
LRRC74B	4	-0.41342	0.873754	0.058611	*IFNAR2*	4	-0.46018	0.762151	0.117959
RCSD1	4	-0.29896	0.874264	0.058358	*VGF*	4	-0.32035	0.762151	0.117959
BLK	4	0.29443	0.874264	0.058358	*ZNF330*	4	-0.29842	0.762151	0.117959
PUF60	4	-0.016246	0.874503	0.058239	*SPTY2D1*	4	-0.25303	0.762151	0.117959
SPRY4	3	0.30478	0.874503	0.058239	*PARP9*	4	0.017159	0.762151	0.117959
GPR82	4	-0.32801	0.874554	0.058213	*ANKRD6*	4	-0.23827	0.762166	0.11795
TIA1	2	-0.28606	0.874554	0.058213	*MTUS2*	4	-0.21772	0.762166	0.11795
FAM9C	4	-0.23255	0.874554	0.058213	*RAPGEFL1*	4	0.31915	0.762343	0.11785
PTPA	4	-0.21807	0.874554	0.058213	*INA*	4	0.074401	0.762587	0.11771
SARNP	4	-0.15881	0.874554	0.058213	*PAM*	3	0.252	0.763008	0.117471
ALPP	4	-0.1355	0.874554	0.058213	*MMP3*	2	0.28288	0.763008	0.117471
LOXL2	4	0.034072	0.874554	0.058213	*ZNF638*	3	-0.53551	0.763281	0.117315
PCDHB7	4	0.067695	0.874554	0.058213	*MINAR1*	2	-0.41163	0.763281	0.117315
SPG7	4	0.070083	0.874554	0.058213	*SAMD5*	3	-0.39346	0.763281	0.117315
NFAT5	4	0.075446	0.874554	0.058213	*PHF12*	4	-0.21282	0.763281	0.117315
MGAM	4	0.089866	0.874554	0.058213	*ANG*	4	-0.20717	0.763281	0.117315
TNC	4	0.10689	0.874554	0.058213	*MVP*	3	-0.19593	0.763281	0.117315
TMPRSS11D	4	0.17203	0.874554	0.058213	*GSTK1*	4	-0.17129	0.763281	0.117315
KIF21B	4	0.19454	0.874554	0.058213	*PHF24*	3	-0.092193	0.763281	0.117315
TMPRSS3	4	0.20372	0.874554	0.058213	*MLLT3*	4	0.096645	0.763281	0.117315
HRH2	4	0.20888	0.874554	0.058213	*GREB1*	4	0.11998	0.763281	0.117315
ZNF544	3	0.23355	0.874554	0.058213	*RAP2A*	4	0.1736	0.763281	0.117315
RABL2B	2	0.24344	0.874554	0.058213	*ABCG2*	4	0.18559	0.763281	0.117315
TENT4B	4	0.35255	0.874554	0.058213	*NAALADL1*	4	0.195	0.763281	0.117315
ADRB1	3	0.35847	0.874554	0.058213	*ZNF133*	4	0.20771	0.763281	0.117315
ATG5	3	0.51593	0.874554	0.058213	*JRK*	4	0.21078	0.763281	0.117315
SHQ1	4	0.53256	0.874554	0.058213	*GABRB3*	3	0.23319	0.763281	0.117315
TCN1	4	-0.3129	0.874774	0.058104	*SSRP1*	4	0.26094	0.763281	0.117315
SLC9A8	4	0.036761	0.875078	0.057953	*TAF1C*	4	0.26144	0.763281	0.117315
VAMP7	3	0.16447	0.875078	0.057953	*JPT1*	3	0.27876	0.763281	0.117315
PSMA7	2	-1.1745	0.875179	0.057903	*CENPN*	4	0.28262	0.763281	0.117315
HK2	3	-0.66726	0.875179	0.057903	*RPL35A*	3	0.2902	0.763281	0.117315
EIF4A3	2	-0.61767	0.875179	0.057903	*GOLGA8N*	1	0.32761	0.763281	0.117315
NAB1	3	-0.60511	0.875179	0.057903	*SLC25A2*	2	0.35073	0.763281	0.117315
FRAS1	2	-0.46155	0.875179	0.057903	*CSGALNACT2*	2	-0.68709	0.763331	0.117287
SLC52A3	3	-0.44644	0.875179	0.057903	*JUNB*	1	-0.50545	0.763331	0.117287
TMEM87B	4	-0.32392	0.875179	0.057903	*STX10*	3	-0.43137	0.763331	0.117287
SLCO3A1	4	-0.31618	0.875179	0.057903	*SAP25*	3	-0.42053	0.763331	0.117287
PTPN13	4	-0.24472	0.875179	0.057903	*ITM2A*	3	-0.39958	0.763331	0.117287
SPIDR	4	-0.24295	0.875179	0.057903	*LRRFIP1*	3	-0.3164	0.763331	0.117287
C3orf20	4	-0.23663	0.875179	0.057903	*USP49*	3	-0.26301	0.763331	0.117287
DHRS7	4	-0.20508	0.875179	0.057903	*KDM6B*	4	-0.25873	0.763331	0.117287

EQTN	4	-0.17224	0.875179	0.057903	*CGNL1*	4	-0.25595	0.763331	0.117287
CARD19	3	-0.12048	0.875179	0.057903	*PAK1*	3	-0.253	0.763331	0.117287
GPRASP1	3	-0.071506	0.875179	0.057903	*CACNA1A*	3	-0.19497	0.763331	0.117287
RHAG	4	-0.037979	0.875179	0.057903	*LRRTM2*	4	-0.18518	0.763331	0.117287
UBAP1	4	0.056025	0.875179	0.057903	*QPCT*	4	-0.13778	0.763331	0.117287
EDEM3	4	0.06692	0.875179	0.057903	*PSME3*	4	-0.12251	0.763331	0.117287
PTS	3	0.1179	0.875179	0.057903	*LMOD1*	4	-0.045989	0.763331	0.117287
MCM9	4	0.12003	0.875179	0.057903	*GLT1D1*	3	-4.48E-04	0.763331	0.117287
ADAMTS9	4	0.23634	0.875179	0.057903	*CDR2*	2	0.011949	0.763331	0.117287
KLHL3	3	0.30989	0.875179	0.057903	*FAM76A*	3	0.031838	0.763331	0.117287
NDUFS2	3	0.44498	0.875179	0.057903	*ENY2*	4	0.046105	0.763331	0.117287
C2orf15	1	0.48512	0.875179	0.057903	*OR4D1*	4	0.05016	0.763331	0.117287
DDX5	3	0.66814	0.875179	0.057903	*TBC1D19*	4	0.11379	0.763331	0.117287
MRPS16	4	-0.27451	0.875558	0.057715	*PELP1*	4	0.13464	0.763331	0.117287
A3GALT2	4	-0.24129	0.875558	0.057715	*ZFAND4*	4	0.17722	0.763331	0.117287
SH2B1	3	-0.22538	0.875558	0.057715	*IL18BP*	4	0.18464	0.763331	0.117287
TSC22D1	4	-0.16562	0.875558	0.057715	*LDB3*	4	0.19776	0.763331	0.117287
PDE9A	3	-0.05271	0.875558	0.057715	*MARCHF8*	4	0.21619	0.763331	0.117287
CEACAM7	4	-5.90E-04	0.875558	0.057715	*FIS1*	4	0.22136	0.763331	0.117287
MADD	4	0.14754	0.875558	0.057715	*EIF4A2*	3	0.26374	0.763331	0.117287
LRRC34	4	0.14935	0.875558	0.057715	*SCIMP*	1	0.26577	0.763331	0.117287
TNFRSF14	4	0.16058	0.875558	0.057715	*MUC13*	3	0.29252	0.763331	0.117287
ZNF586	4	0.16156	0.875558	0.057715	*CCDC6*	3	0.2967	0.763331	0.117287
OR52B6	4	0.16191	0.875558	0.057715	*ATF2*	3	0.3041	0.763331	0.117287
SCRN2	4	0.17291	0.875558	0.057715	*PIFO*	3	0.32879	0.763331	0.117287
RAD51AP1	4	0.17806	0.875558	0.057715	*COG4*	3	0.39952	0.763331	0.117287
SPAG9	4	0.18174	0.875558	0.057715	*ELF1*	4	-0.21452	0.763505	0.117188
IVD	4	0.20002	0.875558	0.057715	*MIB1*	4	-0.38835	0.763587	0.117142
CKAP4	4	0.20685	0.875558	0.057715	*DHX57*	4	-0.2134	0.763587	0.117142
CCNB2	4	0.20865	0.875558	0.057715	*ARPC3*	4	0.11241	0.763828	0.117005
SRGN	3	0.36321	0.875558	0.057715	*FANCD2*	4	0.27281	0.764269	0.116754
DYRK1B	4	0.11494	0.87561	0.057689	*PPIL6*	4	-0.15476	0.764443	0.116655
FFAR1	4	0.20434	0.87561	0.057689	*FAM92A*	2	-0.48572	0.764738	0.116487
TTC21B	4	0.24154	0.87561	0.057689	*SHISAL1*	3	-0.42131	0.764738	0.116487
TRAF3IP1	3	-0.36283	0.875713	0.057638	*ZNF154*	4	-0.34427	0.764738	0.116487
RGS11	4	-0.26675	0.875713	0.057638	*XPO6*	3	0.31758	0.764738	0.116487
POLR3G	3	-0.078537	0.875713	0.057638	*NLRP11*	3	-0.34635	0.765952	0.115798
GPHN	3	0.27697	0.875713	0.057638	*SMG9*	4	-0.24358	0.765952	0.115798
PTF1A	3	0.33282	0.875713	0.057638	*KCNC1*	2	-0.28673	0.766165	0.115677
VPS33B	3	0.39087	0.875713	0.057638	*VMP1*	2	-0.21163	0.766165	0.115677
MCM4	3	-0.36728	0.876501	0.057247	*LRRC3*	4	-0.25604	0.766206	0.115655
FCGR2B	1	-0.36332	0.876501	0.057247	*MYCBPAP*	1	0.60986	0.766579	0.115443
CNOT6	3	-0.34017	0.876501	0.057247	*TAS2R46*	3	-0.39501	0.766791	0.115323
CSRP2	4	-0.32131	0.876501	0.057247	*UNC93A*	4	-0.27048	0.766791	0.115323
CCDC47	4	-0.22331	0.876501	0.057247	*PGAM2*	3	-0.24461	0.766791	0.115323
FOXL2NB	3	-0.21234	0.876501	0.057247	*MOB2*	4	0.084984	0.766791	0.115323
YIPF4	4	-0.19413	0.876501	0.057247	*ETV2*	4	0.089323	0.766791	0.115323
OR7D2	2	-0.17158	0.876501	0.057247	*NRSN1*	4	0.1092	0.766791	0.115323
VWA7	4	0.025118	0.876501	0.057247	*U2AF2*	4	0.31632	0.766791	0.115323
HJURP	3	0.10898	0.876501	0.057247	*ZNF425*	3	-0.5246	0.766937	0.11524
PRH2	3	0.21551	0.876501	0.057247	*PRKCQ*	4	-0.12902	0.766937	0.11524
RPL5	3	0.32138	0.876501	0.057247	*TTC30A*	3	-0.096229	0.767044	0.11518
PRSS35	3	0.39842	0.876501	0.057247	*C2orf16*	4	-0.23909	0.76707	0.115165
NPTX2	3	0.40735	0.876501	0.057247	*COG5*	4	-0.029616	0.76707	0.115165
ATP6V1G1	3	0.52309	0.876501	0.057247	*MLYCD*	3	0.26685	0.76707	0.115165
NDUFA9	3	0.19316	0.876784	0.057107	*GLMP*	3	0.30198	0.76707	0.115165
CMTM1	3	0.19209	0.877133	0.056935	*IL16*	3	-0.39203	0.767481	0.114932
C7orf31	4	-0.18375	0.877283	0.05686	*MFN1*	4	-0.27873	0.767481	0.114932

ITM2C	4	-0.21723	0.877333	0.056836	*AP1AR*	3	-0.2549	0.767627	0.11485
MTFR1	4	0.11363	0.877333	0.056836	*ALPP*	4	0.1693	0.767627	0.11485
PPIL2	4	0.42783	0.877333	0.056836	*ARHGAP42*	4	-0.37627	0.767648	0.114838
GNB1L	3	0.5029	0.87735	0.056827	*ANGPTL2*	2	-0.34173	0.767648	0.114838
AP2A2	3	-0.60145	0.87743	0.056787	*RNF148*	2	-0.30617	0.767648	0.114838
ACLY	4	-0.44764	0.87743	0.056787	*C9orf135*	4	-0.29298	0.767648	0.114838
MIGA1	3	-0.43078	0.87743	0.056787	*PCBD1*	4	-0.20411	0.767648	0.114838
KLF8	3	-0.42129	0.87743	0.056787	*ZC2HC1C*	3	-0.14697	0.767648	0.114838
CELSR1	3	-0.39909	0.87743	0.056787	*ADORA2B*	2	-0.090419	0.767648	0.114838
METTL24	2	-0.38013	0.87743	0.056787	*PCDHGA5*	4	-0.026838	0.767648	0.114838
CTNNA1	2	-0.37194	0.87743	0.056787	*HOXB5*	4	4.54E-04	0.767648	0.114838
METTL25	3	-0.37121	0.87743	0.056787	*HS6ST2*	4	0.003267	0.767648	0.114838
MPND	4	-0.36675	0.87743	0.056787	*DNAL1*	4	0.022649	0.767648	0.114838
GOLGA1	3	-0.36432	0.87743	0.056787	*SLC26A9*	4	0.065676	0.767648	0.114838
ADAT1	4	-0.3485	0.87743	0.056787	*TAF13*	4	0.069473	0.767648	0.114838
SLC44A5	4	-0.3442	0.87743	0.056787	*TXNL1*	4	0.095686	0.767648	0.114838
ZNF493	3	-0.33146	0.87743	0.056787	*RUBCN*	4	0.13962	0.767648	0.114838
OSTM1	4	-0.32186	0.87743	0.056787	*LY6K*	4	0.171	0.767648	0.114838
MRPS10	3	-0.31444	0.87743	0.056787	*SLC20A2*	4	0.1994	0.767648	0.114838
GPR143	4	-0.31439	0.87743	0.056787	*MED27*	3	0.19964	0.767648	0.114838
SLC19A3	4	-0.30371	0.87743	0.056787	*DUSP11*	4	0.22058	0.767648	0.114838
ZNF667	4	-0.27933	0.87743	0.056787	*SYT3*	4	0.22752	0.767648	0.114838
CDO1	4	-0.27471	0.87743	0.056787	*AICDA*	3	0.25044	0.767648	0.114838
FA2H	4	-0.27352	0.87743	0.056787	*SLC9A6*	3	0.32795	0.767648	0.114838
CTAG2	4	-0.2702	0.87743	0.056787	*LMNTD2*	3	0.33826	0.767648	0.114838
FMR1	4	-0.26038	0.87743	0.056787	*DDX49*	3	0.57883	0.767648	0.114838
OR13A1	4	-0.25516	0.87743	0.056787	*SCLY*	1	-0.90869	0.767948	0.114668
SPATA6	4	-0.25288	0.87743	0.056787	*SLC4A7*	2	-0.54809	0.767948	0.114668
UBAP1L	4	-0.22247	0.87743	0.056787	*AKAP8*	4	-0.20582	0.767948	0.114668
SBF2	3	-0.21908	0.87743	0.056787	*RNF4*	4	-0.13814	0.767948	0.114668
ADCY1	4	-0.2177	0.87743	0.056787	*DUOXA1*	4	-0.01158	0.767948	0.114668
RTKN2	4	-0.19448	0.87743	0.056787	*RAD51AP1*	4	0.041114	0.767948	0.114668
GPR75	4	-0.19167	0.87743	0.056787	*CEP72*	4	0.0417	0.767948	0.114668
HLF	4	-0.15513	0.87743	0.056787	*DLG1*	4	0.11322	0.767948	0.114668
TRIM2	4	-0.1385	0.87743	0.056787	*TMEM151A*	4	0.14275	0.767948	0.114668
CCDC69	4	-0.13075	0.87743	0.056787	*ZFAND2A*	4	0.23808	0.767948	0.114668
RRAS	4	-0.12259	0.87743	0.056787	*WFDC2*	4	0.25075	0.767948	0.114668
ZFP1	4	-0.10047	0.87743	0.056787	*CCDC127*	4	-0.45263	0.76821	0.11452
HEATR4	4	-0.089251	0.87743	0.056787	*PPFIA4*	4	-0.22576	0.76821	0.11452
STEAP1	3	-0.075667	0.87743	0.056787	*OCIAD2*	4	0.13353	0.76821	0.11452
NUDT6	3	-0.058259	0.87743	0.056787	*CHD1*	4	0.16903	0.76821	0.11452
ROM1	4	-0.047552	0.87743	0.056787	*ZNF564*	4	0.18893	0.76821	0.11452
ZNF780A	4	-0.020373	0.87743	0.056787	*NUP35*	3	-0.39703	0.768301	0.114469
MLKL	4	0.022972	0.87743	0.056787	*FAM171A2*	3	-0.12635	0.768301	0.114469
CP	4	0.023569	0.87743	0.056787	*DPEP1*	4	0.036545	0.768301	0.114469
IGBP1	4	0.044082	0.87743	0.056787	*FTH1*	3	0.38899	0.768301	0.114469
XKR6	4	0.04954	0.87743	0.056787	*DNAJB9*	2	-0.51585	0.768955	0.114099
STX1A	3	0.064133	0.87743	0.056787	*MMAA*	3	-0.477	0.768955	0.114099
PCK2	3	0.073387	0.87743	0.056787	*AP1S3*	4	-0.38855	0.768955	0.114099
CD53	4	0.083326	0.87743	0.056787	*NKX6-1*	4	-0.34973	0.768955	0.114099
ANKRD45	4	0.084312	0.87743	0.056787	*NCKAP5L*	4	-0.32411	0.768955	0.114099
FOXN3	4	0.090241	0.87743	0.056787	*RASGRF2*	4	-0.31154	0.768955	0.114099
SIRT6	3	0.095054	0.87743	0.056787	*APOLD1*	2	-0.30291	0.768955	0.114099
MEAF6	3	0.096876	0.87743	0.056787	*MICB*	4	-0.29387	0.768955	0.114099
PCDH7	3	0.10085	0.87743	0.056787	*VN1R5*	4	-0.23866	0.768955	0.114099
ZBED3	4	0.11062	0.87743	0.056787	*RNF19B*	4	-0.18852	0.768955	0.114099
GIMAP8	4	0.11916	0.87743	0.056787	*EGFL6*	2	-0.18598	0.768955	0.114099
NEUROD1	4	0.1232	0.87743	0.056787	*NCOR2*	3	-0.15183	0.768955	0.114099

VAT1	4	0.13739	0.87743	0.056787	*PCYOX1L*	4	-0.12401	0.768955	0.114099
C5orf15	4	0.16468	0.87743	0.056787	*C22orf34*	4	-0.091874	0.768955	0.114099
TEKT2	3	0.16998	0.87743	0.056787	*DDX10*	3	-0.051584	0.768955	0.114099
NARS2	3	0.20439	0.87743	0.056787	*TENT4A*	4	-0.021581	0.768955	0.114099
FCGR2A	4	0.20462	0.87743	0.056787	*SERPINB8*	4	-0.008522	0.768955	0.114099
METTL5	4	0.2133	0.87743	0.056787	*C5orf30*	3	-0.002189	0.768955	0.114099
VAC14	4	0.22036	0.87743	0.056787	*ATXN7*	4	0.003074	0.768955	0.114099
SPIN2A	1	0.22804	0.87743	0.056787	*DCK*	4	0.008592	0.768955	0.114099
ZNF43	4	0.23756	0.87743	0.056787	*ARHGAP25*	4	0.037509	0.768955	0.114099
AMIGO3	4	0.24446	0.87743	0.056787	*PON3*	3	0.038039	0.768955	0.114099
HYI	3	0.25479	0.87743	0.056787	*CCDC188*	2	0.070397	0.768955	0.114099
SPINDOC	2	0.25569	0.87743	0.056787	*LPCAT4*	4	0.10122	0.768955	0.114099
LDHB	3	0.26339	0.87743	0.056787	*GNE*	4	0.19456	0.768955	0.114099
BRPF3	3	0.26466	0.87743	0.056787	*SHC2*	4	0.19545	0.768955	0.114099
PPP2R5B	3	0.28283	0.87743	0.056787	*RNF187*	4	0.21052	0.768955	0.114099
GALNT12	3	0.31628	0.87743	0.056787	*FAM83A*	4	0.24281	0.768955	0.114099
PCYT1B	3	0.3195	0.87743	0.056787	*VPS33B*	3	0.25927	0.768955	0.114099
PSD2	4	0.31988	0.87743	0.056787	*SMYD5*	4	0.27534	0.768955	0.114099
ANKUB1	3	0.3372	0.87743	0.056787	*ZNF236*	4	0.30558	0.768955	0.114099
P2RY2	3	0.35035	0.87743	0.056787	*PTGS1*	3	0.34866	0.768955	0.114099
DHX38	4	0.35114	0.87743	0.056787	*PCNA*	2	0.53171	0.769974	0.113524
CNGA4	2	0.35169	0.87743	0.056787	*FGF2*	3	-0.54687	0.770032	0.113491
PHLDB2	3	0.36212	0.87743	0.056787	*RAD54B*	4	-0.29788	0.770032	0.113491
APCDD1L	3	0.49109	0.87743	0.056787	*ZNF473*	4	-0.2814	0.770032	0.113491
PFAS	3	0.492	0.87743	0.056787	*RGS11*	4	-0.12528	0.770032	0.113491
USP24	1	0.5273	0.87743	0.056787	*PID1*	4	-0.07274	0.770032	0.113491
LRRC37A2	2	-0.54914	0.877562	0.056722	*C11orf65*	4	0.051727	0.770032	0.113491
CFAP46	2	-0.47402	0.877562	0.056722	*CEP164*	4	0.095128	0.770032	0.113491
FUT8	3	-0.27561	0.877562	0.056722	*CALHM6*	3	0.22374	0.770032	0.113491
ZNF708	2	-0.26466	0.877562	0.056722	*FOXA3*	3	0.22707	0.770032	0.113491
PTH1R	2	-0.23236	0.877562	0.056722	*TMEM51*	4	0.26691	0.770032	0.113491
TMEM97	4	0.056357	0.877562	0.056722	*SIAH2*	4	-0.21997	0.770108	0.113448
KCP	3	0.14371	0.877562	0.056722	*ENTR1*	3	0.19204	0.770108	0.113448
MUCL3	3	0.32733	0.877562	0.056722	*HSPA2*	4	-0.2826	0.770158	0.11342
DNHD1	2	-0.30988	0.877791	0.056609	*KIAA1586*	2	0.23339	0.770158	0.11342
ZBTB25	4	-0.2935	0.877791	0.056609	*FLCN*	2	0.33795	0.770158	0.11342
BAZ2A	2	0.27368	0.877791	0.056609	*NOX4*	3	-0.38891	0.770442	0.11326
ANKRD65	1	0.34792	0.877791	0.056609	*RUFY4*	3	-0.30725	0.770442	0.11326
DNAJC4	2	0.35066	0.877791	0.056609	*MFAP3*	4	0.066672	0.770442	0.11326
TUBB4B	2	0.577	0.877791	0.056609	*CA11*	4	0.090749	0.770442	0.11326
KLF15	4	-0.16788	0.877791	0.056609	*MEGF10*	4	-0.083669	0.770579	0.113183
WARS2	4	-0.004791	0.877791	0.056609	*CNN2*	4	0.053216	0.770579	0.113183
UBE4B	3	0.16766	0.877791	0.056609	*INTS13*	4	0.093073	0.770579	0.113183
MDH2	3	0.28024	0.877791	0.056609	*LLGL1*	4	0.20505	0.770579	0.113183
ARPC5L	3	-0.48591	0.878749	0.056135	*UBXN10*	3	0.24344	0.770579	0.113183
SPACA9	3	-0.48522	0.878749	0.056135	*RIN2*	3	0.31294	0.770579	0.113183
RMND5B	3	-0.32709	0.878749	0.056135	*ANKS3*	3	0.32039	0.770579	0.113183
HOOK1	3	-0.29719	0.878749	0.056135	*MTMR3*	4	-0.34667	0.771667	0.11257
PRG2	4	-0.28288	0.878749	0.056135	*FPR1*	4	-0.23231	0.771667	0.11257
KDM2B	3	-0.2486	0.878749	0.056135	*ZNF23*	2	0.20068	0.771667	0.11257
PCLO	4	0.097748	0.878749	0.056135	*OAS1*	3	-0.46587	0.771689	0.112557
RAB29	4	0.1745	0.878749	0.056135	*MED24*	3	-0.33961	0.771689	0.112557
STX3	3	0.18789	0.878749	0.056135	*LYSMD3*	3	-0.29577	0.771689	0.112557
COL4A3	4	0.21141	0.878749	0.056135	*AGPAT4*	4	-0.2865	0.771689	0.112557
SLC2A3	4	0.21851	0.878749	0.056135	*ZNF700*	4	-0.26238	0.771689	0.112557
MFAP2	4	0.25008	0.878749	0.056135	*FUT10*	3	-0.20403	0.771689	0.112557
ZNF414	4	0.26343	0.878749	0.056135	*FBXW2*	3	-0.13982	0.771689	0.112557
ZCCHC9	4	0.29863	0.878749	0.056135	*CPXM2*	4	-0.12399	0.771689	0.112557

WDR43	4	0.37877	0.878749	0.056135	*ZSCAN22*	3	-0.10748	0.771689	0.112557
TINAGL1	2	-0.44781	0.880255	0.055391	*SCFD2*	4	-0.083389	0.771689	0.112557
RUBCN	4	0.066226	0.880805	0.05512	*ADAM20*	4	0.066604	0.771689	0.112557
TAF4	4	0.22929	0.880805	0.05512	*ASPRV1*	4	0.069911	0.771689	0.112557
PDE3A	2	-0.4648	0.881921	0.05457	*SND1*	4	0.080347	0.771689	0.112557
ICK	3	-0.41606	0.881952	0.054555	*BCL11A*	4	0.18575	0.771689	0.112557
ACTN3	3	0.14226	0.881952	0.054555	*SGSH*	4	0.2509	0.771689	0.112557
GHITM	3	-0.2048	0.882063	0.0545	*ABCE1*	4	0.46836	0.771689	0.112557
ARHGEF17	2	0.23647	0.882063	0.0545	*BMP1*	3	-0.43949	0.771733	0.112533
MID2	3	0.35662	0.882063	0.0545	*PIP5KL1*	3	-0.35135	0.771733	0.112533
CCR1	4	-0.37588	0.882479	0.054296	*CCNY*	3	-0.24751	0.771733	0.112533
TDRD7	4	-0.34164	0.882479	0.054296	*WDR89*	3	-0.072393	0.771733	0.112533
C7orf66	4	-0.18396	0.882479	0.054296	*RASL11A*	4	-0.06475	0.771733	0.112533
KCTD1	3	0.20689	0.882479	0.054296	*MAX*	4	-0.006421	0.771733	0.112533
CFAP65	4	0.28093	0.882479	0.054296	*PLTP*	3	0.16957	0.771733	0.112533
LGALSL	2	0.32263	0.882479	0.054296	*MYL12B*	1	0.31359	0.771733	0.112533
LAGE3	2	0.40635	0.88314	0.05397	*C7orf66*	4	0.1935	0.771835	0.112476
AP2B1	3	-0.47876	0.883519	0.053784	*DGKQ*	3	-0.42562	0.771894	0.112442
MYL9	3	-0.36993	0.883519	0.053784	*LYL1*	3	-7.49E-04	0.771894	0.112442
PRXL2B	3	-0.35437	0.883519	0.053784	*GNG5*	2	0.087947	0.771894	0.112442
PPCDC	3	0.19826	0.883519	0.053784	*CALCOCO1*	3	0.11455	0.771894	0.112442
TROAP	4	0.2015	0.883519	0.053784	*SLIT1*	2	0.38352	0.771894	0.112442
SLC36A4	3	0.29405	0.883519	0.053784	*PPP4C*	3	0.20646	0.771975	0.112397
FLOT2	3	0.30221	0.883519	0.053784	*MEGF6*	3	0.21761	0.771975	0.112397
CCT2	3	0.63929	0.883519	0.053784	*ACTR3C*	2	0.2354	0.771975	0.112397
LRRK2	3	-0.091754	0.884258	0.053421	*FTCD*	2	0.29674	0.771975	0.112397
MPV17L	4	-0.016796	0.884258	0.053421	*IMPG1*	3	0.32285	0.771975	0.112397
NCOA7	4	0.1981	0.884402	0.05335	*FMNL3*	3	0.33148	0.771975	0.112397
C9orf64	2	0.37864	0.884482	0.053311	*HES3*	3	0.34135	0.771975	0.112397
POMT1	4	-0.28971	0.885012	0.053051	*NDE1*	3	0.42626	0.771975	0.112397
SEPHS2	4	-0.37971	0.885106	0.053005	*PTDSS1*	2	0.4953	0.771975	0.112397
SH2D3A	4	-0.17862	0.885106	0.053005	*CAPN14*	3	-0.36417	0.77198	0.112394
EEF1A2	2	-0.31298	0.88512	0.052998	*GSTM3*	3	-0.1763	0.77198	0.112394
GANC	4	-0.059419	0.885402	0.05286	*ZBTB3*	3	-0.046348	0.77198	0.112394
SH3KBP1	4	0.17251	0.885402	0.05286	*ADCY5*	2	-0.004641	0.77198	0.112394
SLC15A1	3	0.28365	0.885402	0.05286	*S100A3*	2	0.25252	0.77198	0.112394
EXOSC6	4	0.32788	0.885402	0.05286	*COPS6*	3	0.38478	0.77198	0.112394
MDM2	4	0.40708	0.885402	0.05286	*ARL6IP1*	3	0.59693	0.77198	0.112394
AGK	2	0.45967	0.885402	0.05286	*TEPSIN*	2	-0.51059	0.772156	0.112295
AGGF1	3	-0.40399	0.885635	0.052745	*FAAP100*	3	0.28763	0.772156	0.112295
C1D	2	-0.35712	0.885635	0.052745	*TMEM141*	3	0.30143	0.772156	0.112295
HOXD1	4	-0.31567	0.885635	0.052745	*G0S2*	4	-0.29809	0.772509	0.112096
SLC16A14	4	-0.28627	0.885635	0.052745	*RUSC2*	3	-0.072271	0.772509	0.112096
CNDP1	4	-0.28601	0.885635	0.052745	*CBX8*	4	0.14297	0.772509	0.112096
C2CD5	4	-0.2572	0.885635	0.052745	*ACOXL*	3	0.15895	0.772509	0.112096
TCF24	3	-0.22289	0.885635	0.052745	*LDB1*	4	0.17135	0.772509	0.112096
IFT140	4	-0.21512	0.885635	0.052745	*CPOX*	3	0.29566	0.772509	0.112096
MAMSTR	4	-0.19319	0.885635	0.052745	*ITPRIPL2*	4	-0.35659	0.772844	0.111908
LY6G5C	4	-0.15631	0.885635	0.052745	*MRFAP1*	2	-0.171	0.772844	0.111908
ANKRD7	4	-0.011174	0.885635	0.052745	*CAMK1G*	4	-0.069444	0.772844	0.111908
KRCC1	4	0.062078	0.885635	0.052745	*RPF1*	4	0.047679	0.772844	0.111908
TARS3	4	0.19689	0.885635	0.052745	*NHLH1*	4	0.049886	0.772844	0.111908
FAM83D	4	0.25058	0.885635	0.052745	*EPHB4*	4	0.14677	0.772844	0.111908
TEX29	3	0.30112	0.885635	0.052745	*UBL3*	4	0.15991	0.772844	0.111908
GLE1	3	0.52349	0.885635	0.052745	*CHTF8*	4	0.22687	0.772844	0.111908
ZNF575	3	-0.50379	0.886723	0.052212	*TAPBPL*	4	0.38553	0.772844	0.111908
PNPLA6	4	-0.41031	0.886723	0.052212	*MAFG*	4	-0.29053	0.774099	0.111203
HDAC2	3	-0.40959	0.886723	0.052212	*TRMO*	4	-0.31829	0.774264	0.111111

SYT7	2	-0.36241	0.886723	0.052212	*SBF2*	3	-0.53581	0.77432	0.111079
MZT2B	2	-0.33776	0.886723	0.052212	*CREB3L2*	4	-0.36466	0.77432	0.111079
GAPT	4	-0.32051	0.886723	0.052212	*UPF3B*	4	-0.21118	0.77432	0.111079
SIRT3	3	-0.32047	0.886723	0.052212	*ZNF234*	4	-0.095267	0.77432	0.111079
CD82	4	-0.31992	0.886723	0.052212	*DOK4*	4	-0.050924	0.77432	0.111079
HOXA7	4	-0.31675	0.886723	0.052212	*CTRB1*	1	-0.30758	0.774369	0.111052
ETS2	4	-0.30508	0.886723	0.052212	*XK*	4	-0.29282	0.774369	0.111052
C17orf77	3	-0.30222	0.886723	0.052212	*CTCFL*	4	-0.20626	0.774369	0.111052
ORAI3	4	-0.28596	0.886723	0.052212	*CFAP99*	4	-0.012196	0.774369	0.111052
ALDH1A1	4	-0.24512	0.886723	0.052212	*LMBRD1*	4	0.065043	0.774369	0.111052
VAV1	4	-0.23998	0.886723	0.052212	*KLK7*	4	0.1893	0.774369	0.111052
PDLIM1	3	-0.22908	0.886723	0.052212	*IFIT2*	4	0.21304	0.774369	0.111052
C3orf67	4	-0.22653	0.886723	0.052212	*MAPKAPK3*	4	0.21844	0.774369	0.111052
ADIPOR2	4	-0.22129	0.886723	0.052212	*KLHL17*	1	0.36519	0.774369	0.111052
TET1	4	-0.20684	0.886723	0.052212	*PGA3*	1	0.40314	0.774369	0.111052
STARD5	3	-0.18377	0.886723	0.052212	*OVOL2*	4	-0.33594	0.774761	0.110832
TXNL1	4	-0.16761	0.886723	0.052212	*YAF2*	4	-0.11435	0.774761	0.110832
NUP62CL	4	-0.14156	0.886723	0.052212	*IKBKG*	1	0.4641	0.775181	0.110597
RPS19	4	-0.10518	0.886723	0.052212	*SLC25A40*	4	0.29138	0.775601	0.110362
TCAIM	4	-0.076794	0.886723	0.052212	*EIF4EBP1*	4	-0.50982	0.776731	0.10973
MNX1	2	-0.060526	0.886723	0.052212	*UTS2B*	4	-0.30733	0.776731	0.10973
CLTB	4	0.009866	0.886723	0.052212	*PNRC1*	2	0.16365	0.776731	0.10973
UNK	4	0.020197	0.886723	0.052212	*INSC*	3	-0.39925	0.776856	0.109659
SLC35F1	4	0.020662	0.886723	0.052212	*PSKH1*	4	-0.36219	0.776856	0.109659
ZNF792	2	0.024946	0.886723	0.052212	*GAB1*	4	-0.35913	0.776856	0.109659
DIP2B	4	0.044367	0.886723	0.052212	*ZNF770*	2	-0.24477	0.776856	0.109659
SEC24A	3	0.046963	0.886723	0.052212	*ZNF559*	4	-0.10075	0.776856	0.109659
RNF183	4	0.049703	0.886723	0.052212	*DEF8*	4	0.14884	0.776856	0.109659
TMEM222	4	0.050019	0.886723	0.052212	*MGAT2*	4	0.15189	0.776856	0.109659
ZC3H6	4	0.052761	0.886723	0.052212	*GTF2IRD2*	1	-0.5074	0.778829	0.108558
KIAA0930	4	0.056432	0.886723	0.052212	*GPLD1*	3	-0.41314	0.778829	0.108558
HSH2D	4	0.059867	0.886723	0.052212	*CPNE9*	3	-0.40169	0.778829	0.108558
LYPD6	4	0.085514	0.886723	0.052212	*IL21R*	4	-0.37374	0.778829	0.108558
DDRGK1	4	0.10534	0.886723	0.052212	*CHPF*	2	-0.35978	0.778829	0.108558
SLC4A2	4	0.10702	0.886723	0.052212	*TRAPPC12*	4	-0.31404	0.778829	0.108558
MACROD1	4	0.15632	0.886723	0.052212	*C2orf74*	4	-0.27808	0.778829	0.108558
RFX7	4	0.15772	0.886723	0.052212	*DYDC2*	4	-0.21149	0.778829	0.108558
MAGIX	4	0.15883	0.886723	0.052212	*SLC39A6*	4	-0.20702	0.778829	0.108558
C3orf18	4	0.1616	0.886723	0.052212	*RAI2*	4	-0.20401	0.778829	0.108558
VPS13C	4	0.17812	0.886723	0.052212	*DSC1*	4	-0.14466	0.778829	0.108558
MYO10	4	0.19358	0.886723	0.052212	*RGS2*	4	-0.12369	0.778829	0.108558
ATP6V0E1	2	0.20577	0.886723	0.052212	*SLC5A1*	3	-0.11793	0.778829	0.108558
FAHD2B	4	0.20734	0.886723	0.052212	*CRTC1*	3	-0.088134	0.778829	0.108558
CES3	4	0.22582	0.886723	0.052212	*UGT1A3*	4	-0.024359	0.778829	0.108558
TAPBP	3	0.24605	0.886723	0.052212	*SCCPDH*	2	0.06173	0.778829	0.108558
ZFYVE21	3	0.25502	0.886723	0.052212	*SHROOM1*	3	0.068792	0.778829	0.108558
ELMSAN1	4	0.25793	0.886723	0.052212	*ACADL*	4	0.084436	0.778829	0.108558
RPS18	2	0.26418	0.886723	0.052212	*CHRD*	4	0.090642	0.778829	0.108558
RASGRP3	3	0.26495	0.886723	0.052212	*TCL1B*	3	0.09232	0.778829	0.108558
TCAF1	2	0.26812	0.886723	0.052212	*PRR22*	4	0.13397	0.778829	0.108558
CACNA2D2	4	0.27002	0.886723	0.052212	*SNORC*	4	0.1955	0.778829	0.108558
PPA2	4	0.27802	0.886723	0.052212	*SEC62*	3	0.21691	0.778829	0.108558
GOLGA6D	1	0.32892	0.886723	0.052212	*CMC1*	2	0.26771	0.778829	0.108558
FAHD2A	3	0.39249	0.886723	0.052212	*NNT*	2	0.29246	0.778829	0.108558
POLR1A	4	0.47526	0.886723	0.052212	*YWHAE*	3	0.29342	0.778829	0.108558
PECR	4	0.20162	0.886991	0.052081	*KCNH6*	3	0.29664	0.778829	0.108558
CPOX	3	-0.19888	0.887188	0.051984	*PROB1*	4	0.33697	0.778829	0.108558
CYTH2	3	0.24778	0.887188	0.051984	*MND1*	2	0.34092	0.778829	0.108558

SSC4D	2	0.29338	0.887188	0.051984	*PGM3*	3	0.35549	0.778829	0.108558
ENTPD7	3	0.36633	0.887188	0.051984	*ALKBH5*	1	0.40243	0.778829	0.108558
RCL1	3	0.51248	0.887188	0.051984	*C19orf54*	3	0.42444	0.778829	0.108558
MFSD3	3	-0.60954	0.887306	0.051927	*LBHD1*	2	-0.57389	0.779244	0.108326
C8orf33	2	-0.60615	0.887306	0.051927	*SNN*	2	-0.42835	0.779386	0.108247
LARP7	1	-0.54334	0.887306	0.051927	*ACTN3*	3	-0.31141	0.779386	0.108247
AKT3	3	-0.41866	0.887306	0.051927	*SFMBT1*	3	-0.24072	0.779386	0.108247
DMXL1	3	-0.40378	0.887306	0.051927	*SORBS3*	4	-0.2398	0.779386	0.108247
ACTRT3	4	-0.3689	0.887306	0.051927	*PRAG1*	4	-0.26852	0.779542	0.10816
GLUD2	3	-0.3673	0.887306	0.051927	*SYT4*	4	-0.25058	0.779542	0.10816
CDS1	4	-0.34314	0.887306	0.051927	*PARP11*	3	-0.033356	0.779542	0.10816
CARHSP1	3	-0.32207	0.887306	0.051927	*TM4SF1*	4	-0.025615	0.779542	0.10816
SIGLEC11	4	-0.31302	0.887306	0.051927	*YTHDF2*	4	0.028687	0.779542	0.10816
ANKRD12	3	-0.30538	0.887306	0.051927	*EIF3H*	4	0.040345	0.779542	0.10816
MGMT	3	-0.27986	0.887306	0.051927	*MRPL24*	4	0.17253	0.779542	0.10816
DCBLD2	4	-0.2629	0.887306	0.051927	*RSPH4A*	4	0.20406	0.779542	0.10816
UHRF1BP1	4	-0.24607	0.887306	0.051927	*C20orf96*	4	-0.42384	0.779817	0.108007
TRHDE	3	-0.24539	0.887306	0.051927	*SAMD11*	4	-0.28193	0.779817	0.108007
LRMP	3	-0.23227	0.887306	0.051927	*KDM7A*	4	-0.25934	0.779817	0.108007
ABHD17C	4	-0.22951	0.887306	0.051927	*CRYZL1*	4	-0.25786	0.779817	0.108007
ITGB2	2	-0.22028	0.887306	0.051927	*ITGB8*	4	-0.11593	0.779817	0.108007
EPM2AIP1	4	-0.19352	0.887306	0.051927	*BEX1*	3	-0.10885	0.779817	0.108007
CPT1C	4	-0.17422	0.887306	0.051927	*IRAK2*	3	-0.23137	0.779852	0.107988
KLF14	4	-0.14846	0.887306	0.051927	*TSPAN17*	4	0.16331	0.779958	0.107929
C15orf48	4	-0.14167	0.887306	0.051927	*TMOD4*	4	0.22043	0.779958	0.107929
SMLR1	4	-0.099327	0.887306	0.051927	*ATXN10*	2	0.33758	0.779958	0.107929
PRDM7	4	-0.088201	0.887306	0.051927	*ARHGEF40*	4	-0.21978	0.780441	0.10766
KCNK12	4	-0.081407	0.887306	0.051927	*TRIO*	4	-0.066882	0.780441	0.10766
VPS25	3	-0.076414	0.887306	0.051927	*LINC00452*	4	0.018877	0.780441	0.10766
SLCO1B7	3	-0.071316	0.887306	0.051927	*ZNF213*	4	-0.041941	0.781525	0.107057
PRPF18	3	-0.055627	0.887306	0.051927	*PAFAH2*	4	0.046188	0.781525	0.107057
TACO1	4	-0.050704	0.887306	0.051927	*CDKN2AIP*	4	0.052499	0.781525	0.107057
TANC1	4	-0.038814	0.887306	0.051927	*HIPK2*	4	0.087538	0.781525	0.107057
PKP1	3	-0.01663	0.887306	0.051927	*RTCB*	4	-0.079193	0.781629	0.106999
TCP11L2	4	-0.004379	0.887306	0.051927	*RNF38*	3	0.23817	0.781629	0.106999
RBL1	4	0.015011	0.887306	0.051927	*TYW3*	4	0.28977	0.781629	0.106999
FAM133A	3	0.045873	0.887306	0.051927	*U2AF1L4*	3	-0.53625	0.781774	0.106919
GLRX3	3	0.05001	0.887306	0.051927	*RTL8A*	3	-0.43321	0.781774	0.106919
CWC22	3	0.05553	0.887306	0.051927	*METTL15*	2	-0.31103	0.781774	0.106919
ATG3	4	0.071729	0.887306	0.051927	*STXBP5L*	3	-0.11757	0.781774	0.106919
SPON2	4	0.10593	0.887306	0.051927	*NIBAN2*	1	0.2233	0.781774	0.106919
NDUFAF8	4	0.13572	0.887306	0.051927	*SH3BGRL*	3	0.25569	0.781774	0.106919
SLC25A11	4	0.16034	0.887306	0.051927	*FMO5*	1	-0.63346	0.782067	0.106756
SWAP70	4	0.16287	0.887306	0.051927	*METRNL*	4	-0.30723	0.782067	0.106756
TP73	4	0.16673	0.887306	0.051927	*PRSS36*	4	-0.22139	0.782067	0.106756
YWHAE	3	0.16699	0.887306	0.051927	*PRR7*	2	-0.47025	0.782105	0.106735
MBD3L1	4	0.19358	0.887306	0.051927	*COCH*	2	-0.45983	0.782105	0.106735
VEGFA	4	0.2185	0.887306	0.051927	*CTAG2*	4	-0.29936	0.782105	0.106735
RAB30	4	0.2475	0.887306	0.051927	*NOG*	4	-0.19878	0.782105	0.106735
ITPR1	2	0.25363	0.887306	0.051927	*CCR8*	4	-0.09408	0.782105	0.106735
CFAP36	4	0.26957	0.887306	0.051927	*C1orf167*	4	-0.060738	0.782105	0.106735
LPCAT3	3	0.28752	0.887306	0.051927	*PKP4*	4	0.022296	0.782105	0.106735
NOP53	3	0.28888	0.887306	0.051927	*SSH2*	4	0.13222	0.782105	0.106735
SLC18B1	2	0.2947	0.887306	0.051927	*PROX2*	4	0.14911	0.782105	0.106735
VGLL2	3	0.29888	0.887306	0.051927	*TECR*	3	0.26749	0.782105	0.106735
UNC45A	4	0.30587	0.887306	0.051927	*NELFB*	3	0.41427	0.782105	0.106735
NDUFB2	3	0.31685	0.887306	0.051927	*PRMT2*	3	0.45395	0.782105	0.106735
TXNDC12	3	0.33407	0.887306	0.051927	*GLYR1*	4	-0.37901	0.782932	0.106276

EEF1AKMT1	4	0.3514	0.887306	0.051927	*ANKRD52*	3	-0.33745	0.782932	0.106276
RSRC2	3	0.36118	0.887306	0.051927	*XKR5*	4	-0.33495	0.782932	0.106276
DHH	3	0.45217	0.887306	0.051927	*ASB12*	4	0.007365	0.782932	0.106276
COL5A2	2	-0.34881	0.88782	0.051675	*NT5C2*	3	0.032885	0.783	0.106238
EBNA1BP2	2	-0.56329	0.888431	0.051376	*DDI1*	3	0.25729	0.783	0.106238
C7orf69	3	-0.40827	0.888431	0.051376	*ZNF286A*	2	-0.325	0.783285	0.10608
VIP	3	-0.37644	0.888431	0.051376	*ANKRD24*	4	0.17702	0.783865	0.105759
CAAP1	2	-0.37196	0.888431	0.051376	*SNRPD2*	3	0.17749	0.783865	0.105759
RGPD8	1	-0.35646	0.888431	0.051376	*FAM162A*	4	0.19605	0.783865	0.105759
FRS3	4	-0.33453	0.888431	0.051376	*CD81*	4	0.20753	0.783865	0.105759
TREML1	4	-0.31549	0.888431	0.051376	*TAL2*	4	0.22583	0.783865	0.105759
RAB33B	4	-0.27886	0.888431	0.051376	*SEC14L5*	4	0.2296	0.783865	0.105759
ACOX3	4	-0.22753	0.888431	0.051376	*ZNF883*	4	-0.26805	0.784211	0.105567
INHA	3	-0.21983	0.888431	0.051376	*RPL9*	2	-0.49574	0.784794	0.105244
ST8SIA5	4	-0.21354	0.888431	0.051376	*ATP11A*	4	-0.46948	0.784794	0.105244
ZKSCAN1	4	-0.19623	0.888431	0.051376	*CD209*	3	-0.45701	0.784794	0.105244
GNPTAB	4	-0.1834	0.888431	0.051376	*FLNC*	3	-0.28612	0.784794	0.105244
UEVLD	4	-0.17386	0.888431	0.051376	*EMP1*	3	-0.18124	0.784794	0.105244
ITGB8	4	-0.16198	0.888431	0.051376	*LRRC29*	4	-0.13498	0.784794	0.105244
ENTPD6	2	-0.13591	0.888431	0.051376	*TSPAN18*	4	0.041476	0.784794	0.105244
LTBR	3	-0.08153	0.888431	0.051376	*GALE*	3	0.25791	0.784794	0.105244
ZNF484	3	-0.027813	0.888431	0.051376	*NCF4*	4	-0.21628	0.784987	0.105138
NKX3-2	4	0.031046	0.888431	0.051376	*PROP1*	4	0.16395	0.784987	0.105138
DCN	3	0.06782	0.888431	0.051376	*PHF10*	4	-0.3193	0.785148	0.105049
MTMR11	4	0.099983	0.888431	0.051376	*ABI3*	4	-0.25007	0.785148	0.105049
E2F8	3	0.15598	0.888431	0.051376	*MMEL1*	4	-0.24106	0.785148	0.105049
ATP5MGL	2	0.19484	0.888431	0.051376	*ZNF280C*	3	-0.20977	0.785148	0.105049
EIF4E1B	3	0.21686	0.888431	0.051376	*BTBD6*	4	-0.19459	0.785148	0.105049
HTR3D	4	0.23101	0.888431	0.051376	*BAIAP2L2*	4	-0.19402	0.785148	0.105049
GMEB1	4	0.2688	0.888431	0.051376	*FEZ2*	3	-0.008752	0.785148	0.105049
EFEMP1	2	0.28306	0.888431	0.051376	*HNF4G*	4	0.008457	0.785148	0.105049
CFTR	3	0.3206	0.888431	0.051376	*TRIM47*	4	0.016565	0.785148	0.105049
SLC26A10	2	0.38137	0.888431	0.051376	*NCALD*	4	0.067314	0.785148	0.105049
COPS2	3	0.47337	0.888431	0.051376	*ADD1*	4	0.078195	0.785148	0.105049
HNRNPC	3	0.49044	0.888431	0.051376	*KCMF1*	4	0.17363	0.785148	0.105049
NUP205	1	-1.1259	0.888458	0.051363	*RP1*	3	0.24603	0.785148	0.105049
OC10272495	4	-0.32325	0.888458	0.051363	*MMS19*	2	0.61465	0.785148	0.105049
LMO7	4	-0.38849	0.888658	0.051265	*TGFB3*	2	-0.41925	0.785588	0.104805
BMPR2	4	-0.31212	0.888753	0.051219	*TNS1*	4	-0.3488	0.785588	0.104805
SCNN1A	4	-0.004811	0.888753	0.051219	*TIGD3*	4	-0.45434	0.785655	0.104768
DHCR7	4	0.10679	0.888753	0.051219	*TYSND1*	4	-0.11017	0.785655	0.104768
ZNF354C	4	0.13882	0.888753	0.051219	*DNER*	4	-0.1674	0.786219	0.104457
PNLDC1	4	0.19868	0.888753	0.051219	*SLC35F2*	4	-0.12915	0.786219	0.104457
TTC25	4	0.35133	0.888753	0.051219	*TATDN1*	4	-0.24142	0.786223	0.104454
H1-6	3	0.42162	0.888753	0.051219	*VPS29*	3	0.056575	0.786223	0.104454
PPIL6	4	-0.22904	0.889214	0.050994	*EHBP1L1*	3	-0.15604	0.786725	0.104177
CLASP2	2	-0.024796	0.889214	0.050994	*CDH2*	4	0.10181	0.786725	0.104177
RGL4	2	-0.60574	0.890227	0.050499	*C8orf31*	1	-0.48768	0.786776	0.104149
CHRNB1	2	-0.5362	0.890227	0.050499	*MT1F*	3	-0.32112	0.786776	0.104149
SPRYD4	3	-0.43502	0.890227	0.050499	*CPXM1*	4	0.015638	0.786776	0.104149
DNAJC5	4	-0.40907	0.890227	0.050499	*UGT1A9*	4	0.075114	0.786776	0.104149
ACSS2	3	-0.37697	0.890227	0.050499	*REEP5*	4	0.14676	0.786776	0.104149
NCLN	3	-0.37674	0.890227	0.050499	*ATP13A2*	4	0.15288	0.786776	0.104149
DNAJB3	3	-0.29798	0.890227	0.050499	*ZNF439*	4	0.18841	0.786776	0.104149
DCAF17	4	-0.26677	0.890227	0.050499	*CWC22*	3	0.40339	0.786776	0.104149
DCDC2	3	-0.26424	0.890227	0.050499	*ATP6V1D*	2	0.43784	0.786776	0.104149
ACOX2	4	-0.26385	0.890227	0.050499	*RASD2*	4	-0.4576	0.786865	0.1041
CDCA7L	3	-0.25797	0.890227	0.050499	*NRGN*	4	-0.23923	0.786865	0.1041

STX2	4	-0.23903	0.890227	0.050499	*PCDHB13*	4	-0.22073	0.786865	0.1041
C9orf135	4	-0.22803	0.890227	0.050499	*KBTBD12*	4	-0.22049	0.786865	0.1041
DAB1	4	-0.20578	0.890227	0.050499	*ZNF347*	4	-0.20867	0.786865	0.1041
LRP12	4	-0.17571	0.890227	0.050499	*GATA3*	4	-0.19137	0.786865	0.1041
ZNF688	4	-0.15983	0.890227	0.050499	*POM121L2*	4	8.34E-04	0.786865	0.1041
BBS4	4	-0.1532	0.890227	0.050499	*FBP1*	4	0.039191	0.786865	0.1041
USP9Y	4	-0.12527	0.890227	0.050499	*CHST10*	2	0.27792	0.786865	0.1041
POLL	4	-0.10225	0.890227	0.050499	*UHRF1BP1L*	3	0.30871	0.786865	0.1041
CPD	3	-0.1001	0.890227	0.050499	*UBN2*	3	0.31939	0.786865	0.1041
SYN3	4	-0.082157	0.890227	0.050499	*UBE2S*	2	0.44958	0.786865	0.1041
MAST2	4	-0.063245	0.890227	0.050499	*TMEM187*	4	-0.25461	0.787302	0.103859
ACTL7B	4	-0.027293	0.890227	0.050499	*SENP1*	4	-0.15172	0.787302	0.103859
HAMP	2	0.012331	0.890227	0.050499	*CASP2*	2	-0.29708	0.787738	0.103618
SLC13A5	4	0.018644	0.890227	0.050499	*CLUL1*	4	0.038997	0.787738	0.103618
NR2C1	4	0.026473	0.890227	0.050499	*MAGEB17*	4	0.17142	0.788018	0.103464
MTO1	4	0.037259	0.890227	0.050499	*PKD2L1*	4	-0.42899	0.788207	0.10336
PPM1K	4	0.039151	0.890227	0.050499	*ADAMTS13*	3	-0.13798	0.788207	0.10336
ENDOV	4	0.042148	0.890227	0.050499	*MAB21L1*	4	-0.25066	0.788317	0.103299
ABL1	4	0.076427	0.890227	0.050499	*TRIM71*	4	-0.21537	0.788317	0.103299
SLC9B1	4	0.079398	0.890227	0.050499	*PRKAR1B*	3	-0.20415	0.788317	0.103299
SPINK13	4	0.096235	0.890227	0.050499	*LPAR3*	4	-0.098653	0.788317	0.103299
TBC1D8B	4	0.15533	0.890227	0.050499	*ABHD15*	2	0.19829	0.788317	0.103299
WDR45	4	0.16206	0.890227	0.050499	*ADIPOR1*	3	0.21704	0.788317	0.103299
CLDN11	4	0.16471	0.890227	0.050499	*DISP1*	3	0.25115	0.788317	0.103299
LIPE	4	0.20687	0.890227	0.050499	*TMEM132A*	3	0.27145	0.788317	0.103299
PRRC2B	4	0.21118	0.890227	0.050499	*EIF5A*	2	0.48032	0.788317	0.103299
NAP1L2	3	0.21166	0.890227	0.050499	*CES2*	4	-0.26789	0.788846	0.103008
RSBN1	3	0.21951	0.890227	0.050499	*ZDHHC9*	4	0.1608	0.788846	0.103008
ANKRD24	4	0.21955	0.890227	0.050499	*SLC2A13*	2	0.3047	0.788846	0.103008
KLF7	4	0.22342	0.890227	0.050499	*PPP1R32*	3	0.29366	0.78965	0.102565
RAB1A	4	0.22399	0.890227	0.050499	*TPM1*	2	0.31518	0.78965	0.102565
S100A9	3	0.24634	0.890227	0.050499	*ITGA3*	4	-0.31305	0.789683	0.102547
CST6	3	0.261	0.890227	0.050499	*PRKACB*	3	-0.36707	0.790116	0.102309
HMGA1	2	0.27508	0.890227	0.050499	*GPM6B*	3	-0.28858	0.790116	0.102309
OTULIN	4	0.28705	0.890227	0.050499	*VLDLR*	3	-0.19013	0.790336	0.102188
WDHD1	4	0.31902	0.890227	0.050499	*OR10A6*	4	0.11118	0.790336	0.102188
ANKLE1	3	0.32772	0.890227	0.050499	*TRNAU1AP*	4	0.12797	0.790336	0.102188
FAM218A	3	0.34956	0.890227	0.050499	*HERC5*	3	-0.44434	0.7904	0.102153
EXO1	3	0.35481	0.890227	0.050499	*NCOA1*	4	-0.44143	0.7904	0.102153
SLC22A4	1	0.37223	0.890227	0.050499	*LDHAL6B*	3	-0.4175	0.7904	0.102153
MAMDC4	2	0.38217	0.890227	0.050499	*DZIP1L*	4	-0.41359	0.7904	0.102153
PFN1	4	0.38405	0.890227	0.050499	*POU6F2*	2	-0.33598	0.7904	0.102153
AHSG	3	0.43904	0.890227	0.050499	*VASP*	3	-0.31933	0.7904	0.102153
ZNF589	2	0.43956	0.890227	0.050499	*SYK*	3	-0.31487	0.7904	0.102153
NBAS	2	0.46252	0.890227	0.050499	*PARVB*	4	-0.13218	0.7904	0.102153
C2CD4B	1	0.4729	0.890227	0.050499	*OVCA2*	2	0.014623	0.7904	0.102153
GAPDH	3	0.75417	0.890227	0.050499	*RHBDL1*	4	0.061521	0.7904	0.102153
KRTAP5-7	2	0.021656	0.891038	0.050104	*FDPS*	2	0.16541	0.7904	0.102153
TAS2R43	4	-0.41179	0.891483	0.049887	*L3MBTL1*	4	0.16704	0.7904	0.102153
SAFB	4	-0.2932	0.891483	0.049887	*NOS1AP*	3	0.19052	0.7904	0.102153
IRX3	4	-0.20789	0.891483	0.049887	*INPP5D*	2	0.20099	0.7904	0.102153
ARSB	4	-0.12422	0.891483	0.049887	*MYOF*	4	0.22308	0.7904	0.102153
C16orf89	4	-0.021544	0.891483	0.049887	*RAB18*	4	0.22458	0.7904	0.102153
ACSM1	1	0.41429	0.891483	0.049887	*MANF*	4	0.22513	0.7904	0.102153
CHRNA1	4	0.04431	0.891495	0.049881	*ZNF624*	3	0.25002	0.7904	0.102153
MST1L	4	0.17683	0.891595	0.049832	*MBNL3*	2	0.26485	0.7904	0.102153
IL1F10	3	0.2665	0.891595	0.049832	*SLC25A36*	3	0.29732	0.7904	0.102153
TSSK4	4	0.28825	0.891595	0.049832	*RAB11B*	2	0.29932	0.7904	0.102153

INSR	4	-0.072134	0.891682	0.04979	*TEX22*	3	-0.099355	0.79086	0.1019
PTPN12	4	0.013689	0.891682	0.04979	*ARRDC4*	4	-0.33612	0.791321	0.101647
OSBPL8	2	-0.61055	0.892496	0.049394	*TTL*	4	0.15381	0.791752	0.101411
PCDHB3	4	-0.47824	0.892496	0.049394	*POLD4*	4	0.3075	0.791752	0.101411
FAM110C	2	-0.45955	0.892496	0.049394	*BRSK1*	3	0.18479	0.791784	0.101394
SYNGR2	3	-0.44049	0.892496	0.049394	*APBB3*	4	-0.066444	0.791999	0.101275
KATNAL2	3	-0.44015	0.892496	0.049394	*LPCAT2*	2	0.13353	0.792216	0.101156
BRD9	4	-0.43152	0.892496	0.049394	*GLDN*	4	0.24246	0.792216	0.101156
BROX	3	-0.38972	0.892496	0.049394	*RBM48*	4	0.27093	0.792216	0.101156
HEPH	3	-0.38851	0.892496	0.049394	*ZNF24*	1	0.42422	0.793592	0.100403
SENP2	4	-0.37946	0.892496	0.049394	*MEFV*	3	-0.46879	0.794694	0.0998
HEY1	4	-0.35987	0.892496	0.049394	*IGF2BP2*	4	-0.099958	0.794694	0.0998
ZNF736	3	-0.35982	0.892496	0.049394	*MMRN2*	3	0.43616	0.795275	0.099483
DLC1	3	-0.3554	0.892496	0.049394	*SNX29*	3	-0.35038	0.795552	0.099331
PRICKLE1	4	-0.30895	0.892496	0.049394	*IL24*	3	-0.052075	0.795552	0.099331
PCDHA13	2	-0.30345	0.892496	0.049394	*PMFBP1*	4	0.028791	0.795552	0.099331
CD74	3	-0.29399	0.892496	0.049394	*KRBA1*	4	0.084544	0.795552	0.099331
HSPH1	4	-0.27546	0.892496	0.049394	*NAA15*	4	0.35311	0.795552	0.099331
ZMYM1	3	-0.26732	0.892496	0.049394	*UFD1*	3	-0.83115	0.795661	0.099272
SH3D19	2	-0.26425	0.892496	0.049394	*S100A14*	3	-0.54172	0.795661	0.099272
NFU1	4	-0.22918	0.892496	0.049394	*PLXDC2*	3	-0.51526	0.795661	0.099272
OC10012969	4	-0.20381	0.892496	0.049394	*DENND2B*	2	-0.46135	0.795661	0.099272
IGFBP3	3	-0.14494	0.892496	0.049394	*LY6D*	3	-0.38955	0.795661	0.099272
PHLDA3	4	-0.14247	0.892496	0.049394	*LOC10798661*	2	-0.37007	0.795661	0.099272
PSAT1	4	-0.13022	0.892496	0.049394	*DCUN1D4*	2	-0.33718	0.795661	0.099272
FOXN1	4	-0.084233	0.892496	0.049394	*ARSL*	4	-0.33382	0.795661	0.099272
TMEM107	4	-0.052175	0.892496	0.049394	*SHOX2*	3	-0.33046	0.795661	0.099272
PCGF5	4	-0.022891	0.892496	0.049394	*DEUP1*	3	-0.3249	0.795661	0.099272
AGFG2	4	-0.022174	0.892496	0.049394	*TSPAN7*	3	-0.30197	0.795661	0.099272
ZBTB1	3	-0.012729	0.892496	0.049394	*LCN12*	2	-0.29939	0.795661	0.099272
TIAF1	4	-0.004995	0.892496	0.049394	*CHRNA7*	3	-0.2969	0.795661	0.099272
FAM149B1	4	0.004031	0.892496	0.049394	*DCTD*	3	-0.29169	0.795661	0.099272
CNST	4	0.027993	0.892496	0.049394	*BAG2*	4	-0.26098	0.795661	0.099272
TMEM63C	4	0.077267	0.892496	0.049394	*RCBTB1*	4	-0.2577	0.795661	0.099272
B4GALT3	3	0.091362	0.892496	0.049394	*TES*	4	-0.25246	0.795661	0.099272
SLC13A3	4	0.095878	0.892496	0.049394	*RERGL*	4	-0.23035	0.795661	0.099272
DAB2	4	0.097278	0.892496	0.049394	*PPM1A*	3	-0.22276	0.795661	0.099272
CADPS	4	0.11842	0.892496	0.049394	*CRISPLD1*	4	-0.22253	0.795661	0.099272
TNIP1	4	0.16789	0.892496	0.049394	*NLK*	4	-0.16917	0.795661	0.099272
BTNL9	4	0.18815	0.892496	0.049394	*TBATA*	4	-0.15482	0.795661	0.099272
PPP1R1C	4	0.20418	0.892496	0.049394	*KLHL25*	4	-0.13605	0.795661	0.099272
EGFL6	2	0.21172	0.892496	0.049394	*UBAC2*	4	-0.074849	0.795661	0.099272
PADI1	3	0.23108	0.892496	0.049394	*FER*	4	-0.045905	0.795661	0.099272
FLT3LG	4	0.2493	0.892496	0.049394	*CELF4*	3	-0.024869	0.795661	0.099272
MED27	3	0.26036	0.892496	0.049394	*ACSF3*	4	2.78E-04	0.795661	0.099272
KIF15	3	0.27443	0.892496	0.049394	*SULT1A2*	4	0.18751	0.795661	0.099272
CYHR1	3	0.28047	0.892496	0.049394	*GFI1B*	2	0.21058	0.795661	0.099272
FXYD7	4	0.28272	0.892496	0.049394	*TLL2*	4	0.21106	0.795661	0.099272
DLEU7	2	0.2882	0.892496	0.049394	*PATL2*	3	0.27315	0.795661	0.099272
ATE1	4	0.2937	0.892496	0.049394	*LSMEM2*	4	0.2847	0.795661	0.099272
AMOT	4	0.31469	0.892496	0.049394	*WDTC1*	4	0.33281	0.795661	0.099272
SGMS2	4	0.32084	0.892496	0.049394	*EHMT1*	4	0.14712	0.796178	0.09899
CHERP	4	0.33012	0.892496	0.049394	*JOSD1*	3	0.23646	0.796178	0.09899
FAM217A	3	0.33347	0.892496	0.049394	*ECHDC1*	3	0.31384	0.796178	0.09899
PRMT5	4	0.36328	0.892496	0.049394	*IER3*	4	-0.3493	0.796209	0.098973
SAT1	3	0.43113	0.892496	0.049394	*TNFRSF13C*	3	0.25799	0.7963	0.098923
KNG1	2	0.4482	0.892496	0.049394	*ZFHX4*	4	-0.29414	0.796331	0.098906
TBX2	2	0.29293	0.89275	0.04927	*TKFC*	4	-0.2142	0.796331	0.098906

LIPA	1	-0.71386	0.892992	0.049152	*ADPRHL1*	3	0.17032	0.796331	0.098906
USP31	2	-0.39721	0.892992	0.049152	*LIPG*	3	-0.57775	0.796364	0.098888
PAN2	4	-0.35143	0.892992	0.049152	*RAB11FIP1*	3	-0.29544	0.796364	0.098888
RNASEH1	2	-0.34923	0.892992	0.049152	*LMX1B*	4	-0.24058	0.796364	0.098888
CYP4A22	2	-0.34605	0.892992	0.049152	*NFX1*	3	-0.22607	0.796364	0.098888
MARCHF3	4	-0.31332	0.892992	0.049152	*OR13D1*	4	-0.20229	0.796364	0.098888
TBC1D25	4	-0.254	0.892992	0.049152	*COL3A1*	4	0.13816	0.796364	0.098888
CYP2R1	4	-0.2244	0.892992	0.049152	*MPRIP*	3	0.15997	0.796364	0.098888
TNFSF13B	4	-0.22309	0.892992	0.049152	*ARID5A*	3	0.02323	0.796367	0.098887
LDAH	4	-0.21393	0.892992	0.049152	*CH25H*	3	0.23722	0.796367	0.098887
ECHDC2	4	-0.19762	0.892992	0.049152	*CBX3*	3	0.27748	0.796367	0.098887
DSP	4	-0.15486	0.892992	0.049152	*MNS1*	3	0.28882	0.796367	0.098887
VMP1	2	-0.13907	0.892992	0.049152	*RGMB*	3	0.37182	0.796367	0.098887
TPRG1	4	-0.1365	0.892992	0.049152	*RPL5*	3	0.49478	0.796367	0.098887
ZFP92	4	-0.12752	0.892992	0.049152	*USP40*	2	0.39703	0.796397	0.09887
PLXNC1	4	-0.12309	0.892992	0.049152	*ENPP2*	3	0.072564	0.796488	0.098821
EPHA10	4	-0.11875	0.892992	0.049152	*KIF3A*	4	-0.31351	0.796761	0.098672
SKA1	4	-0.089628	0.892992	0.049152	*ZNF627*	4	-0.2848	0.796761	0.098672
TSPAN3	4	-0.070244	0.892992	0.049152	*MGAT5*	3	-0.27213	0.796761	0.098672
RHBDD1	4	-0.051111	0.892992	0.049152	*ERP29*	4	-0.25742	0.796761	0.098672
KANK3	4	-0.045957	0.892992	0.049152	*TRIM54*	4	-0.11164	0.796761	0.098672
SELENOP	4	-0.034862	0.892992	0.049152	*ARF4*	3	0.2404	0.797033	0.098524
PPHLN1	4	-0.020767	0.892992	0.049152	*TLCD3A*	4	0.051408	0.797124	0.098474
CLN3	4	0.025082	0.892992	0.049152	*PLD3*	4	0.098284	0.797124	0.098474
PXMP4	4	0.13892	0.892992	0.049152	*ISOC1*	4	0.17399	0.797124	0.098474
LOC388813	4	0.15151	0.892992	0.049152	*SGK2*	4	0.10506	0.797546	0.098244
TPMT	3	0.15357	0.892992	0.049152	*PDE6C*	4	0.28928	0.797546	0.098244
LMCD1	4	0.162	0.892992	0.049152	*LRRN3*	3	-0.26033	0.797577	0.098228
HSD17B4	4	0.18892	0.892992	0.049152	*CTSA*	4	-0.31983	0.797667	0.098178
FRAT2	4	0.20745	0.892992	0.049152	*CCDC121*	4	0.19872	0.798119	0.097932
FBRSL1	4	0.21721	0.892992	0.049152	*ADRB2*	4	-0.27362	0.79851	0.09772
SLC6A17	4	0.2217	0.892992	0.049152	*RTN4R*	3	0.22963	0.79854	0.097703
ARHGEF1	3	0.23779	0.892992	0.049152	*C1orf109*	3	0.48757	0.798871	0.097523
NEDD4L	3	0.24474	0.892992	0.049152	*HSPB8*	4	-0.38602	0.799262	0.097311
CAPG	4	0.24953	0.892992	0.049152	*TRO*	4	-0.06506	0.799502	0.09718
MARCO	3	0.25721	0.892992	0.049152	*MMP13*	4	0.069124	0.799502	0.09718
MEI4	4	0.26448	0.892992	0.049152	*CTHRC1*	4	-0.20947	0.799592	0.097131
PRDM14	3	0.28215	0.892992	0.049152	*TMEM237*	3	-0.44416	0.799647	0.097102
XG	3	0.30564	0.892992	0.049152	*S1PR5*	3	-0.38771	0.799647	0.097102
KRT83	3	0.31804	0.892992	0.049152	*ZNF639*	3	-0.38068	0.799647	0.097102
RP1	3	0.31975	0.892992	0.049152	*HDX*	4	-0.35579	0.799647	0.097102
F11	3	0.32692	0.892992	0.049152	*FAM49A*	4	-0.27789	0.799647	0.097102
HS3ST3A1	3	0.35466	0.892992	0.049152	*ESRP1*	4	-0.27175	0.799647	0.097102
MTHFD2	4	0.36101	0.892992	0.049152	*OGN*	4	-0.24197	0.799647	0.097102
CDCA5	4	0.37888	0.892992	0.049152	*RAB2A*	3	-0.19637	0.799647	0.097102
MADCAM1	3	0.38257	0.892992	0.049152	*ARHGAP20*	4	-0.17426	0.799647	0.097102
VPS4B	2	0.38909	0.892992	0.049152	*ZNF551*	4	-0.13975	0.799647	0.097102
IMPG2	3	0.39016	0.892992	0.049152	*PRPS1*	3	-0.017699	0.799647	0.097102
CCNA2	3	0.41501	0.892992	0.049152	*NLRP3*	3	-0.001846	0.799647	0.097102
TIMD4	4	-0.28711	0.893244	0.04903	*PGD*	4	0.081389	0.799647	0.097102
RALGDS	2	-0.46638	0.893893	0.048714	*NOTCH1*	4	0.089703	0.799647	0.097102
CALML6	3	-0.46593	0.893893	0.048714	*DEPDC4*	4	0.11272	0.799647	0.097102
TUBB8	2	-0.40593	0.893893	0.048714	*POC1A*	3	0.11806	0.799647	0.097102
SERPINB1	4	-0.39688	0.893893	0.048714	*PNLDC1*	4	0.13069	0.799647	0.097102
LTB4R	4	-0.26994	0.893893	0.048714	*FADD*	4	0.13884	0.799647	0.097102
SMPDL3B	4	-0.21013	0.893893	0.048714	*CCNL2*	4	0.14664	0.799647	0.097102
PCYOX1L	4	-0.15717	0.893893	0.048714	*GPR3*	4	0.19108	0.799647	0.097102
PIGT	3	-0.13535	0.893893	0.048714	*FKBP15*	4	0.19996	0.799647	0.097102

A4GALT	3	-0.10532	0.893893	0.048714	*MYH15*	4	0.22344	0.799647	0.097102
CYBRD1	4	0.1343	0.893893	0.048714	*ACTL10*	3	0.26825	0.799647	0.097102
SOWAHA	4	0.2313	0.893893	0.048714	*TRA2A*	2	0.27029	0.799647	0.097102
EML2	4	0.25195	0.893893	0.048714	*TCP11L1*	3	-0.36663	0.800275	0.096761
GSTCD	3	0.2779	0.893893	0.048714	*H2BC5*	4	-0.37936	0.801046	0.096342
NCCRP1	2	0.36471	0.893893	0.048714	*CCNT1*	3	-0.35724	0.801046	0.096342
STAT1	4	-0.26566	0.894204	0.048563	*SLC5A10*	1	-0.35684	0.801046	0.096342
DEFB126	4	-0.37623	0.894396	0.04847	*RABL2B*	2	-0.24241	0.801046	0.096342
ACKR4	3	0.18581	0.894551	0.048395	*ARHGDIA*	3	-0.069826	0.801046	0.096342
SIX1	3	0.24269	0.894551	0.048395	*KCNJ10*	3	-0.033068	0.801046	0.096342
MYBBP1A	4	0.24812	0.894551	0.048395	*MIXL1*	4	-0.025644	0.801046	0.096342
AKT1	4	-0.22936	0.894563	0.048389	*BRF1*	4	0.026081	0.801046	0.096342
YAP1	3	0.33466	0.895232	0.048064	*FIBP*	2	0.12775	0.801046	0.096342
NABP2	3	-0.33815	0.895423	0.047972	*CLDN15*	4	0.17889	0.801046	0.096342
UNC119B	3	-0.46562	0.896163	0.047613	*KLHL24*	3	0.25868	0.801046	0.096342
ISM2	3	-0.20108	0.896163	0.047613	*ZNF230*	3	0.26651	0.801046	0.096342
ZNF490	4	0.21467	0.896234	0.047579	*MAD2L1*	3	0.29364	0.801046	0.096342
CDC25B	2	0.28509	0.896484	0.047457	*SYBU*	3	0.35759	0.801046	0.096342
PTGIS	3	0.2377	0.897283	0.047071	*CRPPA*	3	-0.38514	0.801193	0.096263
SH3RF2	3	0.31836	0.897283	0.047071	*MTMR4*	3	-0.33065	0.801193	0.096263
PPP2R5C	3	-0.45101	0.897388	0.04702	*TPH1*	3	-0.065459	0.801193	0.096263
LRP10	4	-0.24376	0.897388	0.04702	*PLA2G7*	4	0.079281	0.801193	0.096263
NAALADL2	4	-0.071266	0.897388	0.04702	*MAP4K5*	4	0.1878	0.801193	0.096263
GLDN	4	0.16257	0.897388	0.04702	*SRF*	3	0.4626	0.801222	0.096247
CDC26	2	-0.60621	0.897907	0.046768	*TBX15*	4	0.052325	0.8014	0.096151
URB2	2	-0.6019	0.897907	0.046768	*BBS5*	4	0.17874	0.8014	0.096151
CAVIN3	1	-0.46363	0.897907	0.046768	*PRKD3*	4	-0.33125	0.801578	0.096054
ATP5F1D	4	-0.34665	0.897907	0.046768	*B4GALT1*	2	0.52852	0.801578	0.096054
NHLRC1	4	-0.34453	0.897907	0.046768	*FZD2*	4	-0.28489	0.801845	0.09591
COX7A2L	4	-0.33889	0.897907	0.046768	*TCTEX1D4*	2	-0.36526	0.801873	0.095894
KCNK1	4	-0.31331	0.897907	0.046768	*RASGEF1C*	2	-0.24334	0.801873	0.095894
RGMA	2	-0.30695	0.897907	0.046768	*WDR55*	3	0.33196	0.801873	0.095894
RNF144A	4	-0.27929	0.897907	0.046768	*PLEKHD1*	3	-0.4067	0.802407	0.095605
ANKS1A	4	-0.26169	0.897907	0.046768	*TMEM14C*	3	-0.042087	0.802407	0.095605
ZDHHC20	4	-0.25324	0.897907	0.046768	*SUMO2*	1	-0.38854	0.802517	0.095546
CSPP1	4	-0.24924	0.897907	0.046768	*CLCN4*	4	-0.23514	0.802517	0.095546
UQCC2	4	-0.23391	0.897907	0.046768	*SLC25A46*	3	-0.036661	0.802517	0.095546
SYT13	4	-0.23355	0.897907	0.046768	*CPNE1*	4	0.081798	0.802517	0.095546
GLRX2	4	-0.2333	0.897907	0.046768	*TRPV2*	4	0.08884	0.802517	0.095546
OLR1	4	-0.22876	0.897907	0.046768	*HOXD9*	4	0.11724	0.802517	0.095546
ESR2	4	-0.22789	0.897907	0.046768	*GIPC2*	4	0.12464	0.802517	0.095546
RUSC2	3	-0.22598	0.897907	0.046768	*RAB38*	3	0.20116	0.802517	0.095546
PROSER2	4	-0.22375	0.897907	0.046768	*ULBP1*	4	0.20719	0.802517	0.095546
TRANK1	2	-0.2075	0.897907	0.046768	*MRTFA*	4	0.24582	0.802517	0.095546
MRPL57	4	-0.1995	0.897907	0.046768	*ZNF568*	4	0.35289	0.802517	0.095546
GALNT5	4	-0.18623	0.897907	0.046768	*RPS6*	4	0.39462	0.802517	0.095546
MYH9	4	-0.16555	0.897907	0.046768	*TACR1*	4	0.22042	0.802902	0.095337
PCDHGA11	4	-0.16114	0.897907	0.046768	*EFEMP2*	4	0.066797	0.803048	0.095258
FKRP	2	-0.15164	0.897907	0.046768	*ZNF791*	4	0.20643	0.803048	0.095258
TMEM181	4	-0.14325	0.897907	0.046768	*MUC22*	4	0.2893	0.803048	0.095258
LSAMP	3	-0.13633	0.897907	0.046768	*ZSCAN21*	4	-0.27605	0.803165	0.095195
TMCO1	3	-0.12457	0.897907	0.046768	*MORN4*	4	-0.26098	0.803165	0.095195
PUDP	4	-0.10711	0.897907	0.046768	*DIS3L*	3	-0.20411	0.803223	0.095164
BMX	4	-0.10229	0.897907	0.046768	*SPATA7*	4	0.10991	0.803223	0.095164
DSCR4	2	-0.097183	0.897907	0.046768	*MCM4*	3	-0.38356	0.803447	0.095043
BCAS1	4	-0.090677	0.897907	0.046768	*SNCA*	2	-0.19417	0.803447	0.095043
GCSAM	3	-0.067466	0.897907	0.046768	*ENOSF1*	4	-0.18307	0.803447	0.095043
FBXO8	3	-0.050406	0.897907	0.046768	*MYO3B*	4	-0.099966	0.803447	0.095043

ORMDL1	4	-0.046314	0.897907	0.046768	*FAM9A*	4	-0.005286	0.803447	0.095043
OPA3	4	-0.028327	0.897907	0.046768	*EPHA6*	3	0.005201	0.803447	0.095043
P3H3	4	-0.020809	0.897907	0.046768	*ZNF407*	3	0.041107	0.803447	0.095043
NPR1	4	-0.020686	0.897907	0.046768	*SGCB*	4	0.10983	0.803447	0.095043
PTAFR	4	-0.019966	0.897907	0.046768	*ZWILCH*	3	0.13536	0.803447	0.095043
ATXN7L3B	3	-0.012057	0.897907	0.046768	*SH3GL2*	4	0.15588	0.803447	0.095043
PHB2	4	-0.00159	0.897907	0.046768	*RASGRF1*	4	0.1599	0.803447	0.095043
ST3GAL4	4	0.002085	0.897907	0.046768	*LPIN3*	4	0.17224	0.803447	0.095043
COL1A1	4	0.013262	0.897907	0.046768	*RIC8B*	4	0.18787	0.803447	0.095043
RADX	3	0.02085	0.897907	0.046768	*BRS3*	4	0.24564	0.803447	0.095043
NFE2	4	0.086394	0.897907	0.046768	*DNAH6*	4	0.066831	0.803681	0.094916
LHX9	4	0.092793	0.897907	0.046768	*CACUL1*	4	0.16842	0.803681	0.094916
EPDR1	4	0.10359	0.897907	0.046768	*CBL*	2	0.25416	0.804124	0.094677
KCNAB2	4	0.10447	0.897907	0.046768	*PABPC1L*	3	-0.63206	0.804304	0.09458
MIGA2	4	0.10456	0.897907	0.046768	*CLNK*	2	-0.57316	0.804304	0.09458
BEX2	4	0.11763	0.897907	0.046768	*ZNF764*	2	-0.49349	0.804304	0.09458
ITGA7	4	0.12895	0.897907	0.046768	*ZNF155*	3	-0.46768	0.804304	0.09458
ATP1A1	4	0.13859	0.897907	0.046768	*H6PD*	4	-0.45974	0.804304	0.09458
ALG1	4	0.16048	0.897907	0.046768	*FCER1G*	2	-0.40888	0.804304	0.09458
VSIR	4	0.17187	0.897907	0.046768	*TSPAN14*	3	-0.36539	0.804304	0.09458
RPL9	2	0.18289	0.897907	0.046768	*PPIP5K2*	3	-0.33612	0.804304	0.09458
SEMA4D	4	0.18765	0.897907	0.046768	*SPG11*	3	-0.33285	0.804304	0.09458
LPCAT4	4	0.18949	0.897907	0.046768	*RIBC2*	4	-0.33071	0.804304	0.09458
DNMT1	4	0.19686	0.897907	0.046768	*HLA-DMA*	4	-0.31591	0.804304	0.09458
FANCA	3	0.21596	0.897907	0.046768	*USP6NL*	4	-0.31057	0.804304	0.09458
FBXW11	2	0.22891	0.897907	0.046768	*ZNF85*	3	-0.30993	0.804304	0.09458
GJD4	4	0.24198	0.897907	0.046768	*CFAP61*	3	-0.29592	0.804304	0.09458
FBXL19	2	0.24434	0.897907	0.046768	*LYRM7*	4	-0.23677	0.804304	0.09458
ARL8A	4	0.26315	0.897907	0.046768	*ESR2*	4	-0.23328	0.804304	0.09458
GPAT2	1	0.26489	0.897907	0.046768	*PMP22*	2	-0.21742	0.804304	0.09458
SCN1B	3	0.27308	0.897907	0.046768	*SMC1B*	4	-0.21129	0.804304	0.09458
ANKRD13A	1	0.27336	0.897907	0.046768	*TRAM1*	4	-0.19086	0.804304	0.09458
DDX23	4	0.29126	0.897907	0.046768	*SEC14L6*	4	-0.17516	0.804304	0.09458
TMEM145	2	0.30912	0.897907	0.046768	*PLXNB2*	4	-0.074189	0.804304	0.09458
SUN1	2	0.33026	0.897907	0.046768	*APPL2*	3	-0.074016	0.804304	0.09458
SRF	3	0.34005	0.897907	0.046768	*DHX58*	4	-0.067654	0.804304	0.09458
TMTC4	3	0.35286	0.897907	0.046768	*IQGAP3*	4	-0.066123	0.804304	0.09458
TMEM42	2	0.36992	0.897907	0.046768	*ZNF324B*	3	-0.047596	0.804304	0.09458
ICMT	4	0.3725	0.897907	0.046768	*HEATR9*	4	-0.04666	0.804304	0.09458
UBA7	2	0.38834	0.897907	0.046768	*CNPPD1*	4	-0.037973	0.804304	0.09458
C10orf126	3	0.40368	0.897907	0.046768	*ZNF281*	2	0.030732	0.804304	0.09458
CENPBD1	2	0.44181	0.897907	0.046768	*EPHX3*	4	0.03482	0.804304	0.09458
DDX46	4	0.5533	0.897907	0.046768	*PYCR3*	4	0.062903	0.804304	0.09458
FCF1	1	0.55598	0.897907	0.046768	*SIN3A*	4	0.11967	0.804304	0.09458
H2BC21	1	-0.53777	0.898223	0.046616	*TRIM61*	3	0.1346	0.804304	0.09458
TCTEX1D2	3	-0.032365	0.898223	0.046616	*PADI2*	4	0.13886	0.804304	0.09458
DTWD1	4	0.019736	0.898223	0.046616	*NBPF12*	4	0.16642	0.804304	0.09458
IFNA21	3	0.022746	0.898223	0.046616	*TROAP*	4	0.17074	0.804304	0.09458
UPF3A	3	0.040456	0.898223	0.046616	*SPATS2*	4	0.1861	0.804304	0.09458
DZIP1	3	0.081559	0.898223	0.046616	*PIGW*	4	0.20157	0.804304	0.09458
TSC22D4	4	0.083185	0.898223	0.046616	*LCAT*	4	0.2304	0.804304	0.09458
C11orf65	4	0.15049	0.898223	0.046616	*TM4SF18*	1	0.24894	0.804304	0.09458
ADAM19	4	0.16007	0.898223	0.046616	*FCRL5*	3	0.26149	0.804304	0.09458
PRPF39	4	0.19319	0.898223	0.046616	*CSNK2A3*	2	0.28116	0.804304	0.09458
ACER3	4	0.21278	0.898223	0.046616	*RPL38*	4	0.30623	0.804304	0.09458
SMIM24	3	0.22959	0.898223	0.046616	*TAF7*	4	0.31032	0.804304	0.09458
CDX2	4	0.25127	0.898223	0.046616	*RIC1*	4	0.31268	0.804304	0.09458
PRKD2	4	0.2775	0.898223	0.046616	*INIP*	3	0.33633	0.804304	0.09458

PRKAA1	3	0.30212	0.898223	0.046616	*KLHL3*	3	0.35513	0.804304	0.09458
HDC	4	0.37449	0.898223	0.046616	*SEC13*	2	0.36164	0.804304	0.09458
FAM124A	3	0.3926	0.898223	0.046616	*PPEF2*	3	0.38556	0.804304	0.09458
NARS1	3	0.63394	0.898223	0.046616	*CTDP1*	3	0.39784	0.804304	0.09458
WBP2NL	4	-0.10189	0.898245	0.046605	*PLCH1*	2	0.40795	0.804304	0.09458
UTP23	4	0.24324	0.898245	0.046605	*TMEM234*	1	0.40854	0.804304	0.09458
TSNAX	1	-0.53322	0.899594	0.045954	*UQCRFS1*	2	0.51426	0.804304	0.09458
KIZ	2	-0.46725	0.899594	0.045954	*MPZL1*	4	0.262	0.804449	0.094501
ID1	3	-0.45087	0.899594	0.045954	*TIMM10B*	4	-0.20011	0.804882	0.094268
ARNT	3	-0.43956	0.899594	0.045954	*GPX2*	4	0.18663	0.804882	0.094268
PEX3	4	-0.4226	0.899594	0.045954	*LOC10012908*	4	0.22615	0.804882	0.094268
AMPH	3	-0.42097	0.899594	0.045954	*PECAM1*	4	0.22985	0.804882	0.094268
MLLT11	2	-0.39453	0.899594	0.045954	*TYK2*	4	0.28504	0.804882	0.094268
EPB41L1	4	-0.38316	0.899594	0.045954	*ROBO2*	3	-0.45006	0.804989	0.09421
RPUSD1	4	-0.3725	0.899594	0.045954	*SALL4*	4	-0.37205	0.804989	0.09421
GOLGA7	4	-0.36928	0.899594	0.045954	*SLC4A8*	4	-0.22278	0.804989	0.09421
ELAC1	3	-0.36663	0.899594	0.045954	*LRIF1*	4	-0.10699	0.804989	0.09421
ADHFE1	3	-0.36418	0.899594	0.045954	*LRRIQ1*	4	-0.057746	0.804989	0.09421
SLCO5A1	2	-0.35999	0.899594	0.045954	*C17orf75*	4	0.080899	0.804989	0.09421
CRHBP	3	-0.34397	0.899594	0.045954	*SLC15A2*	4	0.11552	0.804989	0.09421
TPSAB1	2	-0.32935	0.899594	0.045954	*CPLX2*	4	0.16904	0.804989	0.09421
PCDHGC4	2	-0.32924	0.899594	0.045954	*COL23A1*	4	-0.27357	0.805101	0.094149
ZDHHC9	4	-0.32201	0.899594	0.045954	*ZCCHC10*	3	0.16901	0.805101	0.094149
DAO	3	-0.31366	0.899594	0.045954	*KCTD5*	3	0.21617	0.805101	0.094149
OAZ2	4	-0.29355	0.899594	0.045954	*GCN1*	3	0.30469	0.805101	0.094149
BMERB1	4	-0.27999	0.899594	0.045954	*AMOTL2*	3	-0.40569	0.805827	0.093758
TRIM25	4	-0.27916	0.899594	0.045954	*ZNF697*	4	0.10096	0.805827	0.093758
ELOVL1	4	-0.27839	0.899594	0.045954	*SGCA*	4	0.23873	0.805827	0.093758
STMN2	4	-0.27649	0.899594	0.045954	*TMEM104*	4	0.15553	0.806146	0.093586
CHCHD1	4	-0.26967	0.899594	0.045954	*CLDN11*	4	-0.25346	0.806282	0.093513
DYNC2H1	3	-0.24901	0.899594	0.045954	*MT1A*	3	-0.145	0.806282	0.093513
SERPINE3	4	-0.221	0.899594	0.045954	*PDIA4*	4	-0.014878	0.806282	0.093513
CATSPERE	4	-0.20135	0.899594	0.045954	*TNC*	4	0.072417	0.806282	0.093513
UST	4	-0.18688	0.899594	0.045954	*RFTN2*	3	0.19209	0.806282	0.093513
PPIA	4	-0.18679	0.899594	0.045954	*CEP295*	4	0.21096	0.806282	0.093513
PCDHA4	4	-0.16602	0.899594	0.045954	*MACROH2A2*	3	0.23487	0.806282	0.093513
CAVIN4	4	-0.15045	0.899594	0.045954	*SYNCRIP*	3	-0.3148	0.806436	0.09343
PCSK6	4	-0.14942	0.899594	0.045954	*NFE2*	4	-0.16407	0.806436	0.09343
ESAM	4	-0.13872	0.899594	0.045954	*SIRPA*	3	-0.095393	0.806436	0.09343
BDKRB2	4	-0.11979	0.899594	0.045954	*MTF2*	4	-0.020816	0.806436	0.09343
CCDC3	4	-0.11863	0.899594	0.045954	*GDAP1L1*	4	0.036399	0.806436	0.09343
TMOD3	3	-0.11471	0.899594	0.045954	*NIFK*	4	0.057853	0.806436	0.09343
FASTKD1	4	-0.10985	0.899594	0.045954	*FLVCR2*	3	0.075215	0.806436	0.09343
RITA1	2	-0.041689	0.899594	0.045954	*APTX*	4	0.10939	0.806436	0.09343
ARID5A	3	-0.028374	0.899594	0.045954	*NDUFA3*	4	0.15896	0.806436	0.09343
CD163L1	3	-0.004504	0.899594	0.045954	*TGFB2*	3	0.19405	0.806436	0.09343
KRT39	4	0.012072	0.899594	0.045954	*CYLD*	4	0.20706	0.806436	0.09343
SEPTIN4	8	0.012773	0.899594	0.045954	*GSN*	4	0.23409	0.806436	0.09343
RAB36	4	0.020289	0.899594	0.045954	*MBD6*	4	0.28119	0.806436	0.09343
RS1	4	0.031739	0.899594	0.045954	*TXLNG*	3	0.28901	0.806436	0.09343
LY6K	4	0.050252	0.899594	0.045954	*PHTF2*	4	-0.3344	0.807123	0.09306
HACD2	4	0.090832	0.899594	0.045954	*RNF111*	4	-0.32072	0.807123	0.09306
TEKT5	4	0.11733	0.899594	0.045954	*CALHM2*	3	-0.26684	0.807123	0.09306
SLCO2A1	2	0.13055	0.899594	0.045954	*CYP2R1*	4	-0.22897	0.807123	0.09306
XKR7	4	0.13204	0.899594	0.045954	*BHMT*	4	-0.19297	0.807123	0.09306
ZFP2	4	0.13392	0.899594	0.045954	*PARD3B*	4	-0.020903	0.807123	0.09306
ZNF879	3	0.15471	0.899594	0.045954	*SKA1*	4	0.20244	0.807151	0.093045
ASTL	4	0.16532	0.899594	0.045954	*SAMD15*	4	-0.40534	0.807178	0.093031

JAM2	3	0.16932	0.899594	0.045954	*CCDC116*	3	-0.46455	0.808013	0.092581
RBM18	3	0.18776	0.899594	0.045954	*CRYBA2*	2	-0.41449	0.808013	0.092581
EPHA8	4	0.19067	0.899594	0.045954	*UPRT*	4	-0.31687	0.808013	0.092581
FUT4	3	0.19161	0.899594	0.045954	*TMEM63B*	3	-0.28462	0.808013	0.092581
MAFB	4	0.1985	0.899594	0.045954	*IPP*	3	-0.26362	0.808013	0.092581
BICD1	4	0.20097	0.899594	0.045954	*SLC25A18*	4	-0.24873	0.808013	0.092581
PDE1A	3	0.21079	0.899594	0.045954	*GASK1B*	2	-0.21005	0.808013	0.092581
RTL8B	4	0.21723	0.899594	0.045954	*CSNK1A1L*	4	-0.18123	0.808013	0.092581
HGSNAT	4	0.23457	0.899594	0.045954	*NT5C1A*	4	-0.17839	0.808013	0.092581
ZNF302	3	0.23832	0.899594	0.045954	*COL28A1*	4	-0.001744	0.808013	0.092581
PLTP	3	0.24911	0.899594	0.045954	*CCN1*	4	0.062594	0.808013	0.092581
RAB1B	1	0.25255	0.899594	0.045954	*ZNF141*	4	0.10975	0.808013	0.092581
PBDC1	4	0.25697	0.899594	0.045954	*CAPN1*	3	0.11113	0.808013	0.092581
GALC	3	0.28949	0.899594	0.045954	*NIPAL3*	3	0.20138	0.808013	0.092581
IQCA1L	3	0.29268	0.899594	0.045954	*SAMD14*	3	0.21215	0.808013	0.092581
WWOX	3	0.29372	0.899594	0.045954	*TMEM38B*	3	0.2134	0.808013	0.092581
GPSM1	4	0.31691	0.899594	0.045954	*QRICH1*	4	0.21591	0.808013	0.092581
FKBP2	3	0.32942	0.899594	0.045954	*MYL12A*	2	0.2467	0.808013	0.092581
DUSP7	3	0.35165	0.899594	0.045954	*ZNF70*	4	0.25913	0.808013	0.092581
BTC	3	0.36831	0.899594	0.045954	*MAPKAPK5*	3	0.31655	0.808013	0.092581
TMA16	4	0.36961	0.899594	0.045954	*KIAA1107*	4	-0.27319	0.808098	0.092536
RACGAP1	3	0.37299	0.899594	0.045954	*STARD3NL*	4	-0.17224	0.808119	0.092525
MRPL10	3	0.38435	0.899594	0.045954	*LCOR*	8	0.077545	0.808119	0.092525
TNK1	3	0.39522	0.899594	0.045954	*NUBP2*	4	0.13028	0.808119	0.092525
C19orf54	3	0.39987	0.899594	0.045954	*LOC10050642*	4	0.17327	0.808119	0.092525
POLR2F	2	0.42783	0.899594	0.045954	*ZSCAN31*	1	0.38353	0.808119	0.092525
ACSL1	2	0.43872	0.899594	0.045954	*MYCL*	3	-0.5349	0.808454	0.092345
WDR75	3	0.44598	0.899594	0.045954	*KLHL14*	3	-0.27223	0.808454	0.092345
GEMIN6	3	0.47629	0.899594	0.045954	*SH2D4A*	3	-0.12842	0.808454	0.092345
RBM8A	3	0.48485	0.899594	0.045954	*MPHOSPH8*	3	0.06292	0.808454	0.092345
CDK5RAP1	4	-0.45893	0.899626	0.045938	*ARPC1A*	2	0.14308	0.808454	0.092345
OPN3	2	-0.37934	0.899626	0.045938	*ELOVL3*	4	0.37294	0.808454	0.092345
CD59	4	-0.2882	0.899626	0.045938	*STAP1*	3	-0.35436	0.808504	0.092318
IQGAP1	3	-0.20761	0.899626	0.045938	*ATP8A1*	2	-0.059145	0.808504	0.092318
CDK18	4	-0.19922	0.899626	0.045938	*RPL7*	2	0.046009	0.808504	0.092318
CPLX1	3	-0.19137	0.899626	0.045938	*SLC38A7*	1	0.36296	0.808504	0.092318
STAR	4	-0.13651	0.899626	0.045938	*PHF21A*	3	-0.21202	0.808589	0.092272
SLC27A4	4	-0.10002	0.899626	0.045938	*SLCO4A1*	4	0.2124	0.808616	0.092258
CPA6	4	-2.76E-04	0.899626	0.045938	*PUS7L*	3	-0.19346	0.809015	0.092043
GTF2F1	4	0.069697	0.899626	0.045938	*ZNF18*	2	0.30199	0.809015	0.092043
C22orf23	4	0.11067	0.899626	0.045938	*SLC38A11*	3	-0.28536	0.80915	0.091971
STING1	4	0.17605	0.899626	0.045938	*PTS*	3	-0.17497	0.80915	0.091971
CLEC2L	3	0.3198	0.899626	0.045938	*CXCR6*	3	0.15398	0.80915	0.091971
SLA	3	0.35131	0.899626	0.045938	*PLD1*	3	0.30542	0.80915	0.091971
FGF17	3	-0.51118	0.899677	0.045913	*KANSL1*	3	0.52027	0.80915	0.091971
FOXL1	2	-0.49135	0.899677	0.045913	*CCNG1*	3	0.32163	0.809407	0.091833
FGD4	3	-0.45776	0.899677	0.045913	*MIF4GD*	4	-0.27319	0.80951	0.091778
GGT6	4	-0.40066	0.899677	0.045913	*SEMA5A*	4	-0.24797	0.80951	0.091778
GSTM5	4	-0.37744	0.899677	0.045913	*SREBF2*	3	0.12093	0.80951	0.091778
CLNK	2	-0.37588	0.899677	0.045913	*DHX29*	2	0.16523	0.80951	0.091778
CCP110	4	-0.33877	0.899677	0.045913	*KRT5*	3	0.20333	0.80951	0.091778
GLT1D1	3	-0.334	0.899677	0.045913	*TAF8*	3	0.26701	0.80951	0.091778
ASIP	3	-0.3102	0.899677	0.045913	*COPG2*	4	-0.40189	0.810121	0.09145
B4GAT1	4	-0.27606	0.899677	0.045913	*ARHGAP31*	4	-0.21223	0.810121	0.09145
PHLDB3	4	-0.2558	0.899677	0.045913	*ESCO1*	3	0.16842	0.810121	0.09145
THEMIS2	4	-0.24091	0.899677	0.045913	*ZNF543*	3	0.19429	0.810121	0.09145
ADCY9	4	-0.22913	0.899677	0.045913	*CASTOR2*	3	0.22533	0.810121	0.09145
SLAIN1	2	-0.20472	0.899677	0.045913	*ZNF383*	3	0.22915	0.810121	0.09145

EPSTI1	3	-0.14679	0.899677	0.045913	*PCDHA1*	3	0.28264	0.810121	0.09145
ATP5ME	2	-0.12745	0.899677	0.045913	*PPM1H*	3	0.29016	0.810121	0.09145
DIP2C	4	-0.10431	0.899677	0.045913	*CHORDC1*	3	0.31277	0.810121	0.09145
BTBD7	3	-0.091531	0.899677	0.045913	*SPCS2*	1	0.50544	0.810121	0.09145
SERPINF1	4	-0.0497	0.899677	0.045913	*ANKRD42*	3	-0.28192	0.810258	0.091377
HENMT1	4	-0.04481	0.899677	0.045913	*THAP12*	1	0.36165	0.810258	0.091377
TMEM221	4	-0.035484	0.899677	0.045913	*RPL15*	1	0.65796	0.810258	0.091377
DIABLO	4	-0.03396	0.899677	0.045913	*HSBP1*	4	0.096989	0.810513	0.09124
CA11	4	2.94E-04	0.899677	0.045913	*PABPN1*	2	-0.61086	0.810593	0.091197
SLC29A4	4	0.008881	0.899677	0.045913	*CT55*	4	0.13765	0.810593	0.091197
PSME2	4	0.043325	0.899677	0.045913	*MAJIN*	4	0.16341	0.810593	0.091197
GPRC5B	4	0.052114	0.899677	0.045913	*IL6R*	3	0.012927	0.811536	0.090692
QRFP	3	0.066375	0.899677	0.045913	*PBXIP1*	4	-0.20214	0.811583	0.090667
HDHD2	3	0.070501	0.899677	0.045913	*FBXO44*	4	0.07364	0.811583	0.090667
MGST3	4	0.076019	0.899677	0.045913	*SHQ1*	4	0.20227	0.811583	0.090667
MRGPRX3	4	0.084165	0.899677	0.045913	*SRSF2*	1	0.30262	0.811583	0.090667
ZNF678	4	0.096383	0.899677	0.045913	*FAM214B*	4	-0.36439	0.811803	0.090549
PCYT1A	2	0.098515	0.899677	0.045913	*EHD2*	4	-0.1753	0.811803	0.090549
CDC27	2	0.10159	0.899677	0.045913	*OLFM4*	4	-0.16932	0.811803	0.090549
DOLPP1	4	0.10796	0.899677	0.045913	*C22orf24*	3	0.11684	0.811803	0.090549
MCM6	4	0.13333	0.899677	0.045913	*JAKMIP3*	3	0.23169	0.812027	0.09043
KIF2A	4	0.15032	0.899677	0.045913	*PHF20L1*	3	0.26229	0.812027	0.09043
BCKDHA	4	0.166	0.899677	0.045913	*ANXA1*	4	-0.20742	0.812241	0.090315
SLC24A4	4	0.18925	0.899677	0.045913	*LAMB1*	3	-0.14819	0.812241	0.090315
BRD7	4	0.19326	0.899677	0.045913	*RNF223*	4	0.1546	0.812241	0.090315
LAMP3	4	0.2185	0.899677	0.045913	*SNW1*	4	0.20162	0.812241	0.090315
STARD6	4	0.23497	0.899677	0.045913	*KIAA0319L*	3	0.23397	0.812241	0.090315
FNIP2	4	0.23694	0.899677	0.045913	*PPIG*	4	0.24143	0.812241	0.090315
WAS	4	0.24975	0.899677	0.045913	*AGFG1*	3	0.073899	0.813523	0.08963
TRIM11	2	0.25253	0.899677	0.045913	*TET3*	3	-0.37981	0.813746	0.089511
RRAGA	4	0.27103	0.899677	0.045913	*MEF2C*	3	-0.31252	0.813746	0.089511
MFN2	4	0.27801	0.899677	0.045913	*ARFGAP3*	4	-0.26428	0.813746	0.089511
ENOPH1	3	0.29129	0.899677	0.045913	*TBL1X*	4	-0.25951	0.813746	0.089511
ROS1	2	0.29212	0.899677	0.045913	*LRRCC1*	4	-0.2479	0.813746	0.089511
ANKRD39	3	0.2961	0.899677	0.045913	*NFATC4*	4	-0.24254	0.813746	0.089511
CHRNB4	3	0.3183	0.899677	0.045913	*KANSL2*	3	-0.223	0.813746	0.089511
PPWD1	4	0.32661	0.899677	0.045913	*RAG1*	4	-0.21958	0.813746	0.089511
GTF2IRD2	1	0.34846	0.899677	0.045913	*PNMA1*	4	-0.19099	0.813746	0.089511
TPH1	3	0.41481	0.899677	0.045913	*C3orf35*	3	-0.079665	0.813746	0.089511
SNU13	3	0.46959	0.899677	0.045913	*EPHA1*	4	-0.072725	0.813746	0.089511
SHROOM4	4	-0.30758	0.899698	0.045903	*POLM*	4	0.003759	0.813746	0.089511
CASKIN1	4	-0.23712	0.899698	0.045903	*ERMAP*	2	0.11074	0.813746	0.089511
CD151	3	0.23795	0.899708	0.045898	*VTI1B*	4	0.11198	0.813746	0.089511
APOOL	4	-0.20712	0.900081	0.045718	*PLPBP*	4	0.16379	0.813746	0.089511
SPOCK1	2	-0.18096	0.900081	0.045718	*FLYWCH2*	3	0.16515	0.813746	0.089511
PAX6	4	-0.11714	0.900081	0.045718	*STX3*	3	0.19084	0.813746	0.089511
FDXACB1	3	-0.028591	0.900081	0.045718	*HDAC9*	4	0.1932	0.813746	0.089511
UVRAG	4	0.01208	0.900081	0.045718	*ANKRD53*	4	0.19949	0.813746	0.089511
CSRNP1	4	0.018608	0.900081	0.045718	*SFXN4*	3	0.21421	0.813746	0.089511
TAF4B	4	0.15587	0.900081	0.045718	*DEPDC1*	3	0.2188	0.813746	0.089511
STK40	4	0.16681	0.900081	0.045718	*LRRC4*	3	0.21899	0.813746	0.089511
NR1D2	4	0.17887	0.900081	0.045718	*SCAMP2*	3	0.23148	0.813746	0.089511
ZIC5	4	0.21538	0.900081	0.045718	*SNUPN*	4	0.25258	0.813746	0.089511
JMJD6	2	0.21883	0.900081	0.045718	*GABBR2*	4	0.26073	0.813746	0.089511
ITFG2	4	0.23712	0.900081	0.045718	*CARNMT1*	4	0.26161	0.813746	0.089511
RBM48	4	0.27548	0.900081	0.045718	*KBTBD11*	3	0.28313	0.813746	0.089511
ADAMTS12	4	0.3044	0.900081	0.045718	*DSCC1*	3	0.29755	0.813746	0.089511
MUC5B	2	-0.39195	0.900122	0.045698	*NDUFAF1*	3	0.30719	0.813746	0.089511

CKM	4	-0.33838	0.900122	0.045698	*EPOR*	3	0.33623	0.813746	0.089511
FAM98A	3	-0.28513	0.900122	0.045698	*NRP2*	3	0.34873	0.813746	0.089511
H2BC12	1	-0.26238	0.900122	0.045698	*CNTD1*	2	0.37326	0.813746	0.089511
PRMT1	4	-0.25069	0.900122	0.045698	*SMG5*	3	0.40197	0.813746	0.089511
FOXB2	4	-0.14218	0.900122	0.045698	*SORBS1*	3	-0.02236	0.813942	0.089406
ZKSCAN7	3	-0.049484	0.900122	0.045698	*PCDHB7*	4	0.24749	0.814365	0.089181
SLC4A4	4	0.038318	0.900122	0.045698	*FAM110D*	4	-0.18021	0.814643	0.089033
FADS6	4	0.18355	0.900122	0.045698	*XPNPEP3*	4	0.069421	0.814643	0.089033
SLAMF7	3	0.20475	0.900122	0.045698	*SERINC4*	4	-0.16967	0.814688	0.089009
RGS8	4	0.23768	0.900122	0.045698	*NKX1-2*	4	-0.036669	0.814688	0.089009
SLC5A2	3	0.29039	0.900122	0.045698	*CAMK2B*	3	0.11041	0.814688	0.089009
AP3M2	3	0.31599	0.900122	0.045698	*FAM168B*	2	0.1309	0.814688	0.089009
KDM4B	3	0.35107	0.900122	0.045698	*CCDC150*	3	-0.43901	0.814966	0.088861
IL32	3	0.40971	0.900122	0.045698	*BTBD3*	4	-0.007525	0.814966	0.088861
GDPD1	4	-0.36554	0.90022	0.045651	*DUSP1*	4	-0.22533	0.815728	0.088455
EEF2K	3	-0.32259	0.90022	0.045651	*ANKRD22*	4	0.051289	0.815809	0.088411
NT5E	3	-0.13274	0.90022	0.045651	*RFC1*	4	0.1243	0.815885	0.088371
COQ10A	3	-0.063127	0.90022	0.045651	*ARX*	3	0.23589	0.815885	0.088371
HID1	4	-0.39509	0.900688	0.045426	*EMC2*	4	0.29949	0.815885	0.088371
SEC63	4	-0.34093	0.900688	0.045426	*GABRR2*	4	-0.05018	0.816162	0.088224
TSTA3	4	-0.30167	0.900688	0.045426	*KCNK9*	4	0.1722	0.816162	0.088224
KPNA4	4	-0.1057	0.900688	0.045426	*KIF23*	4	0.38461	0.816583	0.088
C16orf70	4	0.01827	0.900688	0.045426	*CYP7A1*	4	-0.2643	0.817136	0.087706
SUGP2	4	0.085157	0.900688	0.045426	*ANKRD1*	3	-0.099962	0.817136	0.087706
ZNF812P	4	0.17516	0.900688	0.045426	*GRIP2*	4	0.004663	0.817136	0.087706
HELT	4	-0.097015	0.900981	0.045285	*FDXR*	3	0.061484	0.817136	0.087706
H2BC15	2	-0.65315	0.901281	0.04514	*S100A4*	4	-0.20788	0.817217	0.087663
DAP	4	-0.38986	0.901281	0.04514	*METRN*	3	-0.57551	0.818246	0.087116
SLC2A4RG	2	-0.30357	0.901281	0.04514	*PCDHGA2*	3	-0.47722	0.818246	0.087116
SOX13	4	-0.30327	0.901281	0.04514	*WBP11*	4	-0.44959	0.818246	0.087116
COX14	4	-0.1711	0.901281	0.04514	*PRSS27*	3	-0.41208	0.818246	0.087116
CDC42EP1	3	-0.16345	0.901281	0.04514	*BCAT1*	3	-0.41111	0.818246	0.087116
MKRN1	4	-0.14707	0.901281	0.04514	*GNAL*	3	-0.40706	0.818246	0.087116
PYY	3	0.056469	0.901281	0.04514	*MINK1*	3	-0.36191	0.818246	0.087116
OR7C1	4	0.085372	0.901281	0.04514	*GPR180*	4	-0.32633	0.818246	0.087116
IRF8	3	0.17589	0.901281	0.04514	*TNFRSF21*	4	-0.31825	0.818246	0.087116
OXCT2	1	0.30403	0.901281	0.04514	*TNFAIP8L1*	4	-0.2387	0.818246	0.087116
EFCAB2	3	0.37404	0.901281	0.04514	*RAB3IL1*	4	-0.23861	0.818246	0.087116
RASGRP1	3	0.48936	0.901281	0.04514	*CNKSR3*	3	-0.23725	0.818246	0.087116
CCKAR	4	-0.22637	0.902137	0.044727	*GSTZ1*	4	-0.21715	0.818246	0.087116
CDKL3	2	-0.60101	0.902247	0.044675	*RIPPLY3*	4	-0.20852	0.818246	0.087116
EEF2KMT	3	-0.36265	0.902247	0.044675	*CDC42EP3*	3	-0.2051	0.818246	0.087116
SLC18A2	4	-0.33663	0.902247	0.044675	*ERFE*	4	0.036439	0.818246	0.087116
ZBED6	4	-0.28305	0.902247	0.044675	*ATP10D*	4	0.18667	0.818246	0.087116
CA8	4	-0.27512	0.902247	0.044675	*KMT2D*	4	0.20183	0.818246	0.087116
DAAM2	4	-0.27258	0.902247	0.044675	*ACVR1B*	4	0.23166	0.818246	0.087116
OXCT1	4	-0.26886	0.902247	0.044675	*NPW*	3	0.26011	0.818246	0.087116
OXT	4	-0.22506	0.902247	0.044675	*EIF3G*	3	0.27999	0.818246	0.087116
RMND1	4	-0.18599	0.902247	0.044675	*CDC37L1*	3	0.24354	0.818721	0.086864
SERPINB12	4	-0.15237	0.902247	0.044675	*GABPA*	2	-0.43133	0.818881	0.086779
HPX	4	-0.1511	0.902247	0.044675	*ZNF284*	3	-0.37959	0.818881	0.086779
SH3GL2	4	-0.071538	0.902247	0.044675	*OTUD1*	4	-0.32171	0.818881	0.086779
UGT1A9	4	0.002894	0.902247	0.044675	*MFSD4B*	4	-0.25983	0.818881	0.086779
GCLC	4	0.025457	0.902247	0.044675	*PLAC1*	4	-0.20676	0.818881	0.086779
ZBTB21	4	0.077546	0.902247	0.044675	*ATXN2*	4	-0.14323	0.818881	0.086779
GEN1	3	0.078069	0.902247	0.044675	*GLRA3*	3	-0.1431	0.818881	0.086779
ANXA11	4	0.098303	0.902247	0.044675	*C8orf58*	4	0.048279	0.818881	0.086779
ZNF785	3	0.10664	0.902247	0.044675	*BEGAIN*	4	0.065069	0.818881	0.086779

NUDT16L1	4	0.10703	0.902247	0.044675	*MDFIC*	4	0.092993	0.818881	0.086779
SPAG7	4	0.11138	0.902247	0.044675	*C10orf55*	3	0.19582	0.818881	0.086779
OR4D11	4	0.13168	0.902247	0.044675	*HTATSF1*	3	0.21173	0.818881	0.086779
PLA2G15	4	0.14195	0.902247	0.044675	*ARRDC1*	3	0.21902	0.818881	0.086779
TOP3A	4	0.19999	0.902247	0.044675	*SCX*	4	0.22469	0.818881	0.086779
FBXL13	4	0.21448	0.902247	0.044675	*FUBP3*	3	0.23301	0.818881	0.086779
ECI2	1	0.23312	0.902247	0.044675	*FAM47C*	3	0.30958	0.818881	0.086779
ANKS6	4	0.2411	0.902247	0.044675	*PHYHD1*	3	0.33463	0.818881	0.086779
EGR1	4	0.26597	0.902247	0.044675	*ACAT2*	1	0.35267	0.818881	0.086779
CLDN18	3	0.28071	0.902247	0.044675	*SARNP*	4	0.11	0.819099	0.086664
CCHCR1	3	0.28656	0.902247	0.044675	*DHCR7*	4	0.23729	0.819099	0.086664
NR3C1	2	0.32956	0.902247	0.044675	*ADGRA3*	3	0.15786	0.819347	0.086532
RRAGC	4	0.33404	0.902247	0.044675	*RNASE6*	4	0.011254	0.819821	0.086281
NRSN2	2	0.36099	0.902247	0.044675	*P2RX1*	2	-0.48923	0.820134	0.086115
SLC35B1	3	0.39669	0.902247	0.044675	*RAB36*	4	-0.39106	0.820134	0.086115
RAD51D	3	0.59547	0.902247	0.044675	*UNC79*	4	0.008592	0.820134	0.086115
SCAMP1	3	-0.067506	0.902369	0.044616	*TGFBI*	4	0.1331	0.820134	0.086115
DCLK2	3	-0.35456	0.902715	0.044449	*CNKSR1*	4	0.14128	0.820134	0.086115
NKX2-4	1	-0.47129	0.904322	0.043677	*VAMP2*	4	0.30199	0.820134	0.086115
POFUT1	4	-0.26575	0.904322	0.043677	*RBAK*	4	-0.27042	0.82027	0.086043
POPDC2	4	-0.25128	0.904322	0.043677	*NCKAP1*	2	0.30684	0.821159	0.085573
MYOM3	4	-0.14405	0.904322	0.043677	*ARHGEF16*	2	0.33723	0.821159	0.085573
PHF21A	3	-0.38166	0.90437	0.043654	*SOD1*	2	-0.59684	0.821542	0.08537
ZNF695	2	-0.36431	0.90437	0.043654	*LDLRAD1*	4	-0.30546	0.821542	0.08537
EVA1A	3	-0.2672	0.90437	0.043654	*ABCB1*	3	-0.24667	0.821542	0.08537
RNASEH2C	4	0.037575	0.90437	0.043654	*CDH20*	4	-0.21465	0.821542	0.08537
AGTPBP1	4	0.13133	0.90437	0.043654	*RS1*	4	-0.16269	0.821542	0.08537
ALS2	4	0.18475	0.90437	0.043654	*C16orf86*	4	-0.1078	0.821542	0.08537
PRR15L	4	0.21673	0.90437	0.043654	*CSTF1*	4	0.002425	0.821542	0.08537
INTS7	2	0.21685	0.90437	0.043654	*ADAMTS8*	4	0.020979	0.821542	0.08537
ZNF829	3	0.23975	0.90437	0.043654	*CD48*	4	0.070751	0.821542	0.08537
RNF149	3	0.33224	0.90437	0.043654	*FNDC3B*	4	0.095533	0.821542	0.08537
CFAP57	2	0.37294	0.90437	0.043654	*CRHR1*	2	0.15162	0.821542	0.08537
FAM189A2	4	-0.16531	0.904547	0.043569	*ADGRG2*	4	0.18506	0.821542	0.08537
PTPRC	3	0.22455	0.904612	0.043538	*ECI2*	1	0.21232	0.821542	0.08537
RSL24D1	2	0.50633	0.904845	0.043426	*RPS26*	1	0.43945	0.821542	0.08537
PRDM2	4	0.093965	0.905524	0.0431	*RWDD4*	1	0.4706	0.821542	0.08537
NRXN3	3	0.037036	0.905645	0.043042	*DDX27*	4	0.57488	0.821542	0.08537
ZNF676	1	0.37462	0.905654	0.043037	*BEST3*	4	-0.36752	0.821685	0.085295
RASL10A	4	-0.35452	0.905784	0.042976	*GRIP1*	3	-0.35113	0.821685	0.085295
ACSL5	4	-0.15929	0.905784	0.042976	*RAPGEF3*	3	-0.27248	0.821685	0.085295
POLR2I	3	-0.10415	0.905784	0.042976	*TMEM63C*	4	-0.23401	0.821685	0.085295
DCLK1	4	-0.034942	0.905784	0.042976	*DNAJC3*	4	-0.13634	0.821685	0.085295
GJD3	4	0.024245	0.905784	0.042976	*CLEC2B*	3	-0.13009	0.821685	0.085295
FAM168B	2	0.15115	0.905784	0.042976	*THYN1*	4	0.036734	0.821988	0.085135
FCHO1	3	0.26572	0.905784	0.042976	*WFS1*	3	-0.43516	0.82233	0.084954
MRPS18C	4	0.29369	0.905784	0.042976	*APBB2*	4	-0.24529	0.82233	0.084954
PABPN1	2	-0.2733	0.905969	0.042887	*PDCD4*	4	-0.23176	0.82233	0.084954
COPS6	3	0.44835	0.905969	0.042887	*CR2*	3	0.063726	0.82233	0.084954
DYRK3	2	-0.52272	0.90626	0.042747	*PDLIM5*	4	0.071116	0.82233	0.084954
FOXA1	3	-0.37648	0.90626	0.042747	*RADX*	3	-0.4031	0.822484	0.084873
HSPG2	4	-0.20926	0.90626	0.042747	*SMARCA5*	4	-0.31666	0.822484	0.084873
PRR14	4	-0.17018	0.90626	0.042747	*CDC42SE2*	3	-0.30703	0.822484	0.084873
TNNI3	4	-0.16924	0.90626	0.042747	*DYNLRB2*	4	-0.30544	0.822484	0.084873
SERPINE2	4	-0.16527	0.90626	0.042747	*LARP6*	4	-0.23748	0.822484	0.084873
PIN1	4	-0.1432	0.90626	0.042747	*LLGL2*	4	-0.22472	0.822484	0.084873
HACD1	4	-0.12739	0.90626	0.042747	*TRIML2*	4	-0.17534	0.822484	0.084873
ENOX1	3	-0.1227	0.90626	0.042747	*HID1*	4	-0.12531	0.822484	0.084873

PHLPP1	4	-0.12095	0.90626	0.042747	*PTPRM*	4	-0.091535	0.822484	0.084873
MAGOH	4	-0.11189	0.90626	0.042747	*NCCRP1*	2	-0.071617	0.822484	0.084873
PLCE1	4	-0.082489	0.90626	0.042747	*NADK*	3	-0.054682	0.822484	0.084873
ARMC8	4	0.015478	0.90626	0.042747	*ZFHX2*	3	-0.013637	0.822484	0.084873
SORBS2	4	0.027871	0.90626	0.042747	*CLCA2*	4	-0.010022	0.822484	0.084873
NFKB1	4	0.05742	0.90626	0.042747	*IL3RA*	3	-0.00975	0.822484	0.084873
C1S	4	0.076273	0.90626	0.042747	*SGSM2*	3	0.066881	0.822484	0.084873
FBXO32	4	0.083437	0.90626	0.042747	*GRWD1*	3	0.11777	0.822484	0.084873
ZNF652	4	0.095982	0.90626	0.042747	*DACH1*	4	0.14192	0.822484	0.084873
ILDR1	4	0.11072	0.90626	0.042747	*GPR20*	2	0.18368	0.822484	0.084873
PBX2	4	0.13677	0.90626	0.042747	*CTSV*	3	0.18621	0.822484	0.084873
MBNL3	2	0.14157	0.90626	0.042747	*NUPR2*	2	0.24969	0.822484	0.084873
RIC1	4	0.1691	0.90626	0.042747	*MICU1*	4	0.26535	0.822484	0.084873
EEF1AKNMT	4	0.17559	0.90626	0.042747	*TRAPPC2L*	4	0.2931	0.822484	0.084873
LSR	4	0.17746	0.90626	0.042747	*ACSM1*	1	0.34991	0.822484	0.084873
KCNQ1	3	0.22925	0.90626	0.042747	*AGT*	4	-0.35518	0.82292	0.084643
ENDOD1	1	0.35319	0.90626	0.042747	*SREBF1*	4	-0.17229	0.82292	0.084643
PEAR1	4	0.073362	0.907823	0.041999	*AIG1*	4	-0.23355	0.823131	0.084531
TRPM7	1	-0.54962	0.908395	0.041725	*IFT74*	3	-0.13228	0.823131	0.084531
TMPRSS11F	3	-0.45025	0.908395	0.041725	*TTC37*	4	-0.036815	0.823131	0.084531
BCL2L11	3	0.29212	0.908459	0.041695	*SEMA3C*	3	-0.029451	0.823131	0.084531
VRK3	3	-0.47206	0.909158	0.041361	*GRN*	4	0.11443	0.823131	0.084531
RENBP	4	-0.18687	0.909158	0.041361	*SLC6A17*	4	0.1147	0.823131	0.084531
SOSTDC1	4	-0.11233	0.909158	0.041361	*SELENBP1*	4	0.15917	0.823131	0.084531
ERCC1	3	0.33321	0.909158	0.041361	*NINJ2*	3	0.25399	0.823131	0.084531
CNPY4	3	-0.37752	0.909379	0.041255	*CYB5D2*	3	0.27749	0.823131	0.084531
CBX4	4	-0.31203	0.909379	0.041255	*KLHDC2*	4	-0.01199	0.823201	0.084494
MRPL44	4	-0.28324	0.909379	0.041255	*TUBB2B*	2	0.10328	0.823201	0.084494
SYCP3	4	-0.22526	0.909379	0.041255	*HTR1D*	2	0.28477	0.823201	0.084494
LRRC58	4	-0.17726	0.909379	0.041255	*SLC25A25*	3	-0.35397	0.823414	0.084382
MECR	4	-0.17238	0.909379	0.041255	*DDR1*	4	-0.23857	0.823414	0.084382
HSP90AA1	2	-0.096769	0.909379	0.041255	*GLI1*	4	-0.17107	0.823516	0.084328
GJB4	4	-0.067704	0.909379	0.041255	*NAA60*	3	0.239	0.823516	0.084328
MIIP	4	-0.05176	0.909379	0.041255	*SEMA3E*	3	-0.38062	0.823862	0.084145
TTLL9	4	0.17456	0.909379	0.041255	*TMC6*	4	-0.14657	0.823862	0.084145
NAIP	2	0.23981	0.909379	0.041255	*C2orf72*	1	0.31047	0.823862	0.084145
RNF6	4	0.25575	0.909379	0.041255	*MFSD9*	3	0.14356	0.823996	0.084075
ZNF18	2	0.31024	0.909379	0.041255	*CPT1C*	4	-0.28036	0.824256	0.083938
ARHGAP27	4	-0.51072	0.909791	0.041059	*SHISA9*	4	-0.26366	0.824256	0.083938
ELN	3	-0.43256	0.909791	0.041059	*ANOS1*	3	-0.10311	0.824256	0.083938
GNB5	2	-0.39732	0.909791	0.041059	*CTDSP2*	4	-0.097258	0.824256	0.083938
FAM24B	3	-0.34705	0.909791	0.041059	*PLOD3*	4	-0.09205	0.824256	0.083938
CCDC144A	2	-0.32022	0.909791	0.041059	*CCDC170*	4	-0.054564	0.824256	0.083938
PLEKHF1	3	-0.2902	0.909791	0.041059	*SHB*	4	-0.012635	0.824256	0.083938
POU4F1	4	-0.27334	0.909791	0.041059	*KLC2*	2	0.18266	0.824256	0.083938
UBN1	2	-0.26096	0.909791	0.041059	*RAB5B*	4	0.21896	0.824256	0.083938
TMED6	4	-0.21528	0.909791	0.041059	*AGAP1*	3	0.23273	0.824256	0.083938
ARC	4	-0.20289	0.909791	0.041059	*C8orf82*	3	0.24068	0.824256	0.083938
CD55	4	-0.13539	0.909791	0.041059	*IGIP*	4	0.24317	0.824256	0.083938
CERS5	4	-0.13422	0.909791	0.041059	*GRM6*	3	0.26702	0.824256	0.083938
GCC2	4	-0.1095	0.909791	0.041059	*NFYB*	3	0.26904	0.824256	0.083938
GPRC5C	4	0.003142	0.909791	0.041059	*PRLHR*	4	0.28122	0.824256	0.083938
OLFM4	4	0.01341	0.909791	0.041059	*CDCA3*	4	0.33982	0.824256	0.083938
FCAR	4	0.057629	0.909791	0.041059	*MFSD14B*	3	-0.4326	0.824656	0.083727
CYP11A1	4	0.062097	0.909791	0.041059	*DPYSL4*	3	-0.36853	0.824656	0.083727
CD47	4	0.06512	0.909791	0.041059	*TTC23L*	4	-0.018698	0.824656	0.083727
SUCO	4	0.07572	0.909791	0.041059	*GAREM1*	3	-0.22538	0.82518	0.083451
RHBDD3	4	0.08395	0.909791	0.041059	*KIF4B*	4	0.20091	0.82518	0.083451

FBXO45	4	0.087579	0.909791	0.041059	*FOXD4L5*	3	0.24679	0.82518	0.083451
FJX1	4	0.09146	0.909791	0.041059	*STRA6*	3	0.3304	0.82518	0.083451
DRG2	4	0.091659	0.909791	0.041059	*BANP*	3	0.37741	0.82518	0.083451
CTIF	4	0.10429	0.909791	0.041059	*GRB2*	4	0.43069	0.82518	0.083451
SEMA3B	4	0.10539	0.909791	0.041059	*DCHS1*	3	-0.59384	0.825184	0.083449
ZMAT2	4	0.1355	0.909791	0.041059	*RIMS2*	4	-0.33014	0.825184	0.083449
CEACAM6	2	0.16099	0.909791	0.041059	*SLC41A1*	3	-0.27962	0.825184	0.083449
AVEN	4	0.16864	0.909791	0.041059	*APEH*	4	-0.21856	0.825184	0.083449
PCSK2	4	0.18171	0.909791	0.041059	*C10orf99*	4	-0.15997	0.825184	0.083449
SLC49A4	3	0.22644	0.909791	0.041059	*PHTF1*	3	-0.069897	0.825184	0.083449
SOAT2	4	0.24037	0.909791	0.041059	*CPLX3*	4	-0.067217	0.825184	0.083449
FANCE	4	0.25366	0.909791	0.041059	*IRF6*	3	-0.061195	0.825184	0.083449
PTPRG	4	0.26548	0.909791	0.041059	*CHRNA5*	4	0.076523	0.825184	0.083449
SMIM5	4	0.27322	0.909791	0.041059	*EIF4E3*	4	0.10807	0.825184	0.083449
SRPRA	4	0.28558	0.909791	0.041059	*MEI4*	4	0.1505	0.825184	0.083449
SMNDC1	4	0.29757	0.909791	0.041059	*SF3B6*	3	0.19479	0.825184	0.083449
NIFK	4	0.26602	0.910238	0.040845	*FXYD4*	4	0.22905	0.825184	0.083449
CBWD1	1	-0.54107	0.910446	0.040746	*SACM1L*	4	0.2403	0.825184	0.083449
ZNF436	4	-0.41611	0.910446	0.040746	*AGPAT1*	3	0.24928	0.825184	0.083449
SNX22	3	-0.41098	0.910446	0.040746	*PLA2G3*	3	0.32311	0.825184	0.083449
TMEM31	2	-0.3554	0.910446	0.040746	*PGLYRP1*	4	0.33653	0.825184	0.083449
DNAH12	4	-0.32152	0.910446	0.040746	*ATG12*	4	-0.25321	0.825714	0.08317
RPARP-AS1	1	-0.3012	0.910446	0.040746	*DTX3*	3	0.28101	0.825714	0.08317
ZFYVE19	4	-0.28656	0.910446	0.040746	*EIF1AD*	4	0.35393	0.825714	0.08317
SHISA7	3	-0.28104	0.910446	0.040746	*TAF2*	4	0.55294	0.825714	0.08317
NIM1K	4	-0.25818	0.910446	0.040746	*APOBEC3G*	4	-0.12629	0.825732	0.083161
ZBTB46	4	-0.25002	0.910446	0.040746	*E2F2*	4	-0.095324	0.825732	0.083161
NT5DC2	4	-0.24143	0.910446	0.040746	*SPEF1*	4	-0.012865	0.825732	0.083161
GNA14	4	-0.19636	0.910446	0.040746	*C3orf52*	4	0.056722	0.825732	0.083161
TXNIP	4	-0.18906	0.910446	0.040746	*NOTCH3*	4	0.081973	0.825732	0.083161
SULF2	4	-0.13843	0.910446	0.040746	*SLC25A23*	4	0.20396	0.825732	0.083161
RALGAPA2	3	-0.12236	0.910446	0.040746	*PWWP3B*	4	0.22072	0.825732	0.083161
GPR158	3	-0.11203	0.910446	0.040746	*MAP4*	2	0.54802	0.825732	0.083161
ZNF277	4	-0.045141	0.910446	0.040746	*DRAXIN*	3	0.13767	0.826138	0.082948
CAPS2	4	-0.036891	0.910446	0.040746	*FBXO41*	4	0.036921	0.826415	0.082802
TMED10	4	-0.001906	0.910446	0.040746	*ADAM28*	4	0.056673	0.826415	0.082802
STOML1	3	0.002274	0.910446	0.040746	*LARP1B*	4	0.13988	0.826415	0.082802
EPHA7	3	0.06129	0.910446	0.040746	*AKR1E2*	4	0.17818	0.826415	0.082802
CXCL5	4	0.11785	0.910446	0.040746	*ZFP30*	3	0.32526	0.826415	0.082802
MAFF	3	0.15004	0.910446	0.040746	*ATP6V0A1*	3	0.27105	0.82693	0.082531
CEP135	4	0.15991	0.910446	0.040746	*MSL1*	4	-0.24878	0.827065	0.082461
ARHGEF39	4	0.17477	0.910446	0.040746	*TYMP*	3	-0.15406	0.827065	0.082461
ATMIN	4	0.18596	0.910446	0.040746	*CFH*	4	0.10484	0.827065	0.082461
PCMTD2	4	0.19867	0.910446	0.040746	*JMJD8*	4	0.14714	0.827065	0.082461
SNAPC2	2	0.26412	0.910446	0.040746	*FAM3A*	4	0.26376	0.827065	0.082461
ATG2B	4	0.32282	0.910446	0.040746	*EIF2A*	2	0.31394	0.827065	0.082461
FSBP	3	0.34052	0.910446	0.040746	*PTPRG*	4	-0.26142	0.827087	0.082449
TSPAN6	1	0.44141	0.910446	0.040746	*LGALS3BP*	4	-0.16943	0.827241	0.082368
MRPL35	2	0.54871	0.910446	0.040746	*ITGA10*	4	0.10922	0.827241	0.082368
SUMF1	3	0.24516	0.911274	0.040351	*ACIN1*	3	-0.19723	0.827264	0.082356
RPIA	4	0.21724	0.911609	0.040191	*MIS18BP1*	3	0.27128	0.827636	0.082161
ATG4A	4	-0.1457	0.911914	0.040046	*NT5C3A*	3	0.31435	0.827636	0.082161
STARD3NL	4	-0.017465	0.911914	0.040046	*TMEM131*	1	-0.50104	0.827849	0.082049
HPF1	4	-0.016536	0.911914	0.040046	*COMMD10*	3	-0.24412	0.827849	0.082049
FAM166C	3	0.2812	0.911914	0.040046	*WNT3A*	4	-0.20941	0.827849	0.082049
PFKFB1	4	-0.1683	0.912195	0.039912	*SCN8A*	3	-0.19107	0.827849	0.082049
NAGK	4	-0.36817	0.912319	0.039853	*C18orf63*	2	-0.18443	0.827849	0.082049
S100A7L2	3	0.028945	0.912319	0.039853	*CASK*	4	-0.055034	0.827849	0.082049

EP300	3	0.11155	0.912491	0.039771	*UTS2R*	4	0.019845	0.827849	0.082049
CKAP2	4	0.029273	0.912577	0.039731	*APBA1*	4	0.25367	0.827849	0.082049
CD36	4	0.1322	0.912577	0.039731	*RPL29*	1	0.28326	0.827849	0.082049
CST3	4	0.21283	0.912577	0.039731	*ASB7*	4	0.29413	0.827849	0.082049
ELOVL3	4	0.43081	0.912577	0.039731	*GOLT1B*	4	0.33042	0.827849	0.082049
SLC27A5	1	0.42359	0.91302	0.03952	*RPF2*	4	0.35965	0.827849	0.082049
ADH5	2	-0.37003	0.913198	0.039435	*SOS1*	4	0.159	0.828165	0.081883
AARS2	4	-0.2502	0.913198	0.039435	*AMOT*	4	0.2305	0.828165	0.081883
LOC389602	4	-0.21253	0.913198	0.039435	*ENDOD1*	1	-0.46738	0.828309	0.081808
ZNF750	4	0.005286	0.913198	0.039435	*MGST1*	4	0.026557	0.828309	0.081808
ILRUN	3	0.11948	0.913198	0.039435	*NTF4*	3	0.14518	0.828309	0.081808
TRPV1	3	0.22127	0.913198	0.039435	*NR2F2*	3	0.16245	0.828309	0.081808
PWWP2B	2	0.25708	0.913198	0.039435	*C11orf68*	4	0.18038	0.829496	0.081186
SLK	3	0.27014	0.913198	0.039435	*MCM3*	3	0.53453	0.829496	0.081186
KCNH6	3	0.31399	0.913198	0.039435	*USF2*	3	0.26058	0.829844	0.081004
CRIM1	3	-0.12471	0.913377	0.03935	*TSTA3*	4	-0.27389	0.829975	0.080935
ZNF155	3	0.11251	0.913377	0.03935	*KANK2*	2	-0.45275	0.829997	0.080924
VAMP4	3	-0.31588	0.913445	0.039317	*RAB6B*	4	-0.084989	0.829997	0.080924
CCDC74A	2	-0.27796	0.913445	0.039317	*CEP290*	4	-0.082692	0.829997	0.080924
JAK1	4	0.05267	0.913445	0.039317	*COL25A1*	4	-0.00117	0.829997	0.080924
NLGN2	4	0.10699	0.913445	0.039317	*ARAF*	4	0.064962	0.829997	0.080924
FAM210A	4	0.11925	0.913445	0.039317	*TNIP2*	2	0.15149	0.829997	0.080924
DCAKD	4	0.13347	0.913445	0.039317	*MCRS1*	3	-0.2222	0.830258	0.080787
ZNF423	4	0.17701	0.913445	0.039317	*H4C3*	2	0.14845	0.830258	0.080787
PAK1IP1	4	0.19544	0.913445	0.039317	*ZMYND11*	3	0.21723	0.830258	0.080787
METAP1	4	0.4892	0.913445	0.039317	*CD109*	3	0.24458	0.830258	0.080787
FRZB	3	0.18638	0.913561	0.039262	*C10orf126*	3	0.29996	0.830258	0.080787
CRLF2	2	-0.45037	0.913708	0.039192	*PELI2*	3	0.32385	0.830258	0.080787
GTF2H2C	1	-0.42136	0.913708	0.039192	*NPPC*	1	0.38764	0.830258	0.080787
AAMDC	3	-0.4098	0.913708	0.039192	*NLRP2*	4	-0.16913	0.830811	0.080498
SVOPL	2	-0.3727	0.913708	0.039192	*ABITRAM*	3	-0.12695	0.830811	0.080498
ARL3	4	-0.34262	0.913708	0.039192	*PIGO*	4	0.073598	0.830811	0.080498
ZMYND10	2	-0.30156	0.913708	0.039192	*MRAP2*	4	-0.18555	0.830909	0.080447
GCNT7	4	-0.18395	0.913708	0.039192	*NLRP7*	4	-0.15264	0.830909	0.080447
CHL1	3	-0.18121	0.913708	0.039192	*ANKRD46*	3	-0.36939	0.830994	0.080402
SNX27	4	-0.17569	0.913708	0.039192	*PLIN2*	4	-0.25584	0.830994	0.080402
MAZ	4	-0.16797	0.913708	0.039192	*H1-8*	4	-0.21753	0.830994	0.080402
TOX	4	-0.15587	0.913708	0.039192	*FRMPD2*	4	-0.20732	0.830994	0.080402
TAF5L	3	-0.11752	0.913708	0.039192	*NWD2*	4	-0.1612	0.830994	0.080402
MAPKAPK2	4	-0.11344	0.913708	0.039192	*HSPB9*	4	0.009802	0.830994	0.080402
PITPNM3	4	-0.1058	0.913708	0.039192	*PSG1*	3	0.12659	0.830994	0.080402
SCARF2	4	-0.094324	0.913708	0.039192	*KRTAP1-1*	3	0.24232	0.830994	0.080402
PTGER2	4	-0.08005	0.913708	0.039192	*KBTBD7*	1	0.34221	0.830994	0.080402
C12orf76	4	-0.075099	0.913708	0.039192	*ILRUN*	3	-0.46896	0.831206	0.080291
ANP32E	3	-0.0647	0.913708	0.039192	*NONO*	3	-0.44258	0.831206	0.080291
TRAPPC3L	4	-0.061652	0.913708	0.039192	*CARD9*	2	-0.41568	0.831206	0.080291
CHID1	4	-0.053167	0.913708	0.039192	*FGL2*	3	-0.40089	0.831206	0.080291
EXD1	4	0.075702	0.913708	0.039192	*HIRA*	4	-0.39506	0.831206	0.080291
CDKN3	3	0.07866	0.913708	0.039192	*PPP6R1*	3	-0.38395	0.831206	0.080291
SNX24	4	0.097199	0.913708	0.039192	*SH3BGR*	3	-0.36688	0.831206	0.080291
TMEM262	4	0.10355	0.913708	0.039192	*SIRPB1*	4	-0.36087	0.831206	0.080291
BCLAF3	4	0.10505	0.913708	0.039192	*ATAD3A*	3	-0.31752	0.831206	0.080291
PRKCSH	2	0.1275	0.913708	0.039192	*PYGO1*	4	-0.31033	0.831206	0.080291
PHF19	4	0.13561	0.913708	0.039192	*LINC02801*	3	-0.2621	0.831206	0.080291
COASY	4	0.14652	0.913708	0.039192	*MFSD5*	4	-0.25674	0.831206	0.080291
NRDE2	4	0.15906	0.913708	0.039192	*MTURN*	4	-0.22094	0.831206	0.080291
CCDC15	4	0.16511	0.913708	0.039192	*TRIM38*	4	-0.17321	0.831206	0.080291
STX8	3	0.22119	0.913708	0.039192	*CDH6*	4	-0.10815	0.831206	0.080291

PATZ1	3	0.28315	0.913708	0.039192	*JOSD2*	4	-0.099743	0.831206	0.080291
KIFC2	4	0.29983	0.913708	0.039192	*KLK10*	4	-0.082092	0.831206	0.080291
PNISR	3	0.33093	0.913708	0.039192	*TAL1*	4	-0.074246	0.831206	0.080291
TIMM50	3	0.33463	0.913708	0.039192	*PRADC1*	4	-0.057234	0.831206	0.080291
ZNF207	4	0.3411	0.913708	0.039192	*CEP152*	4	0.1047	0.831206	0.080291
CHMP6	4	0.35121	0.913708	0.039192	*TMEM223*	4	0.10801	0.831206	0.080291
NUDT1	3	0.36142	0.913708	0.039192	*ZBED5*	4	0.1646	0.831206	0.080291
CRCP	3	0.38006	0.913708	0.039192	*SNAPC5*	3	0.18089	0.831206	0.080291
CAD	3	0.38309	0.913708	0.039192	*SLIT3*	4	0.18204	0.831206	0.080291
SLC3A2	3	0.45195	0.913708	0.039192	*PROK1*	4	0.18748	0.831206	0.080291
OSBPL6	4	0.20563	0.914039	0.039035	*CYP2C9*	4	0.18957	0.831206	0.080291
SCN11A	4	-0.037006	0.91427	0.038926	*TAFA5*	4	0.20225	0.831206	0.080291
RASL11A	4	0.14254	0.91427	0.038926	*WASHC2C*	1	0.22061	0.831206	0.080291
ASAP2	1	-0.46167	0.914355	0.038885	*C1GALT1*	3	0.22317	0.831206	0.080291
PWWP3A	3	-0.45207	0.914355	0.038885	*UPK1B*	4	0.2289	0.831206	0.080291
MEOX2	2	-0.39158	0.914355	0.038885	*CDYL*	4	0.23715	0.831206	0.080291
NTS	4	-0.36322	0.914355	0.038885	*TSEN2*	4	0.28099	0.831206	0.080291
IRAK4	4	-0.35205	0.914355	0.038885	*POLD3*	4	0.29305	0.831206	0.080291
MTMR1	2	-0.26708	0.914355	0.038885	*PDE6D*	3	0.29918	0.831206	0.080291
DNAJB8	3	-0.24961	0.914355	0.038885	*CEP350*	4	0.32134	0.831206	0.080291
DGKE	4	-0.24783	0.914355	0.038885	*CFAP57*	2	-0.51621	0.831924	0.079916
A2ML1	4	-0.242	0.914355	0.038885	*MT2A*	2	-0.39171	0.831924	0.079916
ENTPD4	4	-0.23376	0.914355	0.038885	*INTS7*	2	-0.38454	0.831924	0.079916
DUSP15	4	-0.23109	0.914355	0.038885	*ZMYM5*	2	-0.34047	0.831924	0.079916
SLC39A6	4	-0.18549	0.914355	0.038885	*IL4I1*	4	-0.33381	0.831924	0.079916
RNF2	4	-0.1553	0.914355	0.038885	*TRIM5*	3	-0.32587	0.831924	0.079916
IRF5	4	-0.11853	0.914355	0.038885	*TUBGCP2*	3	-0.30417	0.831924	0.079916
BFSP1	3	-0.11415	0.914355	0.038885	*DUSP13*	4	-0.27546	0.831924	0.079916
TP53AIP1	3	-0.10977	0.914355	0.038885	*TMEM145*	2	-0.24778	0.831924	0.079916
COL9A2	4	-0.10659	0.914355	0.038885	*TRMU*	4	-0.24645	0.831924	0.079916
CDC42BPA	4	-0.088404	0.914355	0.038885	*STIM1*	4	-0.22778	0.831924	0.079916
GUCY1A2	4	-0.084263	0.914355	0.038885	*SLC9A5*	4	-0.22575	0.831924	0.079916
CADM4	4	-0.04733	0.914355	0.038885	*NFAM1*	4	-0.22163	0.831924	0.079916
ELF1	4	-0.047307	0.914355	0.038885	*SLC52A3*	3	-0.21532	0.831924	0.079916
HGFAC	4	-0.024944	0.914355	0.038885	*RBM15B*	4	-0.20762	0.831924	0.079916
STAC2	4	-0.020298	0.914355	0.038885	*CLDN20*	4	-0.20581	0.831924	0.079916
SSRP1	4	-0.010216	0.914355	0.038885	*DNAJC10*	4	-0.20157	0.831924	0.079916
KCNMB2	4	-0.005256	0.914355	0.038885	*GTPBP1*	4	-0.20145	0.831924	0.079916
C20orf203	4	0.008219	0.914355	0.038885	*ERO1B*	4	-0.19852	0.831924	0.079916
TBC1D5	4	0.017157	0.914355	0.038885	*KCNN1*	2	-0.1897	0.831924	0.079916
TRPM3	4	0.037424	0.914355	0.038885	*RINT1*	3	-0.15762	0.831924	0.079916
ONECUT3	4	0.048382	0.914355	0.038885	*CYP2C19*	4	-0.15274	0.831924	0.079916
MCF2	4	0.098034	0.914355	0.038885	*PRKRA*	4	-0.15076	0.831924	0.079916
PSD	4	0.10145	0.914355	0.038885	*HMGA2-AS1*	4	-0.15042	0.831924	0.079916
PLEKHG5	4	0.12966	0.914355	0.038885	*AP1G1*	4	-0.14617	0.831924	0.079916
HCAR3	3	0.15317	0.914355	0.038885	*KCNH4*	3	-0.14194	0.831924	0.079916
BGLAP	2	0.16966	0.914355	0.038885	*TMED2*	4	-0.12071	0.831924	0.079916
APBB3	4	0.1805	0.914355	0.038885	*C1orf54*	3	-0.11828	0.831924	0.079916
SLCO2B1	4	0.23289	0.914355	0.038885	*FNIP1*	3	-0.11561	0.831924	0.079916
AK5	3	0.24931	0.914355	0.038885	*GJD4*	4	-0.10902	0.831924	0.079916
DSPP	3	0.26582	0.914355	0.038885	*CCDC102A*	3	-0.10442	0.831924	0.079916
FITM1	3	0.31445	0.914355	0.038885	*ITFG1*	4	-0.10007	0.831924	0.079916
PANK1	3	0.31573	0.914355	0.038885	*ACBD3*	3	-0.098897	0.831924	0.079916
GJB5	3	0.31721	0.914355	0.038885	*WDR31*	4	-0.095213	0.831924	0.079916
IL1R2	3	0.32931	0.914355	0.038885	*ST3GAL4*	4	-0.09005	0.831924	0.079916
PTAR1	4	0.34485	0.914355	0.038885	*SPHK2*	4	-0.080474	0.831924	0.079916
PHTF1	3	0.34845	0.914355	0.038885	*C1orf100*	4	-0.080301	0.831924	0.079916
THSD7B	2	0.34908	0.914355	0.038885	*CHFR*	4	-0.077712	0.831924	0.079916

CDKN2D	4	0.35824	0.914355	0.038885	*NIPSNAP3A*	4	-0.071071	0.831924	0.079916
CLSPN	3	0.45096	0.914355	0.038885	*FKBP1B*	4	-0.059753	0.831924	0.079916
REST	3	0.49483	0.914355	0.038885	*DNASE1L2*	2	-0.04158	0.831924	0.079916
RGS6	3	-0.016019	0.914537	0.038799	*MAP6D1*	4	-0.037519	0.831924	0.079916
TCF19	3	0.011009	0.914537	0.038799	*NEDD1*	4	-0.037423	0.831924	0.079916
U2AF1L4	3	0.38117	0.914537	0.038799	*C3orf14*	4	-0.034217	0.831924	0.079916
SYNPO2	3	-0.35105	0.91468	0.038731	*KIAA1614*	4	-0.02041	0.831924	0.079916
S100A14	3	-0.11856	0.91468	0.038731	*R3HCC1L*	4	-0.004679	0.831924	0.079916
ACBD3	3	0.21246	0.91468	0.038731	*ASB9*	3	0.001209	0.831924	0.079916
TRNT1	2	0.48119	0.91468	0.038731	*TGM1*	4	0.002244	0.831924	0.079916
GPN2	3	0.75602	0.91468	0.038731	*RERG*	3	0.013868	0.831924	0.079916
GRIFIN	3	-0.26608	0.914724	0.03871	*GALT*	3	0.018122	0.831924	0.079916
SLC51B	4	-0.22808	0.914724	0.03871	*WDR44*	3	0.049139	0.831924	0.079916
HPS5	4	-0.10296	0.914724	0.03871	*IFT52*	4	0.067837	0.831924	0.079916
PEG10	4	0.097515	0.914724	0.03871	*DESI1*	4	0.069086	0.831924	0.079916
SLC37A3	3	0.2493	0.914724	0.03871	*SCNN1G*	4	0.075838	0.831924	0.079916
PNKP	3	0.51225	0.914724	0.03871	*A1CF*	4	0.084275	0.831924	0.079916
PPP1R36	3	-0.14668	0.915104	0.038529	*FGD5*	3	0.0967	0.831924	0.079916
EFEMP2	4	0.12868	0.915254	0.038458	*MAGED2*	4	0.098694	0.831924	0.079916
IFIT1B	4	0.15029	0.915254	0.038458	*C5orf34*	2	0.098919	0.831924	0.079916
SEPTIN6	4	0.17662	0.915254	0.038458	*CDX2*	4	0.11772	0.831924	0.079916
CD200	4	0.23063	0.915254	0.038458	*PLK5*	4	0.12306	0.831924	0.079916
CHD2	4	0.23658	0.915254	0.038458	*LPIN1*	4	0.13007	0.831924	0.079916
PSMB9	4	0.31297	0.915254	0.038458	*HYI*	3	0.15461	0.831924	0.079916
SACS	2	-0.33545	0.915329	0.038423	*WASF3*	3	0.15577	0.831924	0.079916
CALB1	4	-0.27783	0.915329	0.038423	*HHEX*	4	0.15943	0.831924	0.079916
EEA1	4	-0.049828	0.915329	0.038423	*GABBR1*	2	0.19048	0.831924	0.079916
ACSS3	3	-0.19611	0.915719	0.038238	*RAB31*	4	0.19468	0.831924	0.079916
LTB	4	-0.007322	0.915719	0.038238	*RAB27A*	4	0.19597	0.831924	0.079916
FER1L5	4	0.019876	0.915719	0.038238	*ETFA*	3	0.1967	0.831924	0.079916
MYO5B	4	0.053397	0.915719	0.038238	*APRT*	4	0.19808	0.831924	0.079916
ICAM4	4	0.086558	0.915719	0.038238	*METTL2A*	1	0.20316	0.831924	0.079916
CA3	4	0.13978	0.915719	0.038238	*ZMAT2*	4	0.20322	0.831924	0.079916
RNF139	4	0.21082	0.915719	0.038238	*CCDC91*	2	0.20467	0.831924	0.079916
MSANTD4	2	0.23451	0.915719	0.038238	*SNAPC1*	4	0.21623	0.831924	0.079916
ANKDD1A	4	0.26695	0.915719	0.038238	*OAZ1*	3	0.2185	0.831924	0.079916
TDGF1	3	0.29716	0.915719	0.038238	*MYPN*	4	0.22198	0.831924	0.079916
ZBTB47	4	0.21564	0.915939	0.038134	*CNBP*	4	0.22482	0.831924	0.079916
UCKL1	4	-0.072988	0.916059	0.038077	*NXN*	4	0.22687	0.831924	0.079916
LEO1	3	0.44356	0.916059	0.038077	*LCA5*	2	0.23038	0.831924	0.079916
NSMF	4	-0.41453	0.916161	0.038028	*YLPM1*	4	0.23049	0.831924	0.079916
KLK13	4	-0.20833	0.916161	0.038028	*CISD2*	2	0.23174	0.831924	0.079916
ATXN7	4	-0.11197	0.916161	0.038028	*SLC6A15*	3	0.23613	0.831924	0.079916
C1orf195	4	-0.073191	0.916161	0.038028	*PPIA*	4	0.2436	0.831924	0.079916
MASP1	4	0.10301	0.916161	0.038028	*FLG2*	4	0.24706	0.831924	0.079916
TRIO	4	0.15071	0.916161	0.038028	*FAM13A*	3	0.25296	0.831924	0.079916
STPG4	3	0.24902	0.916161	0.038028	*CCDC47*	4	0.25367	0.831924	0.079916
DHX34	3	-0.28419	0.916546	0.037846	*PRKAA1*	3	0.26693	0.831924	0.079916
SRGAP2B	1	-0.28278	0.916546	0.037846	*SDR39U1*	3	0.27337	0.831924	0.079916
CCDC142	3	-0.45137	0.916549	0.037845	*ANKUB1*	3	0.275	0.831924	0.079916
PLXND1	4	-0.39722	0.916549	0.037845	*CEACAM21*	4	0.28034	0.831924	0.079916
ZNF594	2	-0.24951	0.916549	0.037845	*SPAG1*	3	0.28833	0.831924	0.079916
GASK1B	2	-0.218	0.916549	0.037845	*GNAI1*	3	0.30859	0.831924	0.079916
CABCOCO1	4	-0.092772	0.916549	0.037845	*ADAM9*	3	0.30886	0.831924	0.079916
MTMR3	4	0.027678	0.916549	0.037845	*B3GNT6*	3	0.33573	0.831924	0.079916
MEF2A	3	0.21651	0.916549	0.037845	*IKZF3*	3	0.34501	0.831924	0.079916
CLYBL	1	0.38587	0.916549	0.037845	*WDR75*	3	0.34724	0.831924	0.079916
NSUN2	3	-0.26077	0.916815	0.037718	*PSMA3*	4	0.42393	0.831924	0.079916

ELOVL4	3	-0.023451	0.916815	0.037718	*RPLP1*	2	0.44208	0.831924	0.079916
NGF	4	0.055198	0.916815	0.037718	*KDM2B*	3	-0.40568	0.832739	0.079491
MBIP	4	0.17052	0.916815	0.037718	*SHISA2*	4	-0.37441	0.833945	0.078862
HSPA12B	4	0.20646	0.916815	0.037718	*RASGEF1B*	3	-0.28184	0.833945	0.078862
KDM4D	4	0.22804	0.916815	0.037718	*TMEM190*	2	-0.51065	0.83396	0.078855
ATG13	4	0.24146	0.916815	0.037718	*MOCS2*	4	-0.4102	0.83396	0.078855
PDE10A	4	0.28421	0.916815	0.037718	*NR1H3*	4	-0.26662	0.83396	0.078855
IL2RG	4	-0.28229	0.917253	0.037511	*ZNF616*	4	-0.2648	0.83396	0.078855
PABPC4L	4	-0.18734	0.917253	0.037511	*UBXN2A*	3	-0.22997	0.83396	0.078855
CCDC120	4	0.002589	0.917253	0.037511	*LDHC*	4	-0.17374	0.83396	0.078855
TMEM249	3	0.085665	0.917253	0.037511	*FCRLA*	4	-0.12267	0.83396	0.078855
C2orf42	3	0.22542	0.917253	0.037511	*TLE6*	4	0.23097	0.83396	0.078855
AADAT	2	0.25952	0.917253	0.037511	*RHOG*	4	0.26179	0.83396	0.078855
CPSF2	2	0.27836	0.917253	0.037511	*FLAD1*	2	0.30819	0.83396	0.078855
FBXW7	3	0.30509	0.917253	0.037511	*SAMD9*	3	0.35292	0.83396	0.078855
SPC25	3	0.36614	0.917253	0.037511	*PAQR5*	2	-0.44607	0.834034	0.078816
MUC6	1	0.56941	0.917253	0.037511	*EDARADD*	3	-0.4351	0.83455	0.078548
ABCC4	3	-0.26554	0.91726	0.037507	*CAMSAP3*	1	0.3864	0.83455	0.078548
VPS26C	4	0.18792	0.918541	0.036902	*HYAL3*	4	-0.50057	0.834632	0.078505
EIF3E	4	0.24652	0.918541	0.036902	*PYCARD*	1	-0.46875	0.834632	0.078505
C19orf53	3	0.31786	0.918811	0.036774	*CHUK*	4	-0.40127	0.834632	0.078505
METTL4	4	-0.029094	0.919517	0.03644	*GNGT2*	2	-0.38798	0.834632	0.078505
ZDHHC1	4	0.11764	0.919517	0.03644	*TTC7A*	4	-0.35555	0.834632	0.078505
DNAJB13	4	0.27323	0.919517	0.03644	*TJP1*	3	-0.34867	0.834632	0.078505
MRM3	2	-0.35868	0.919787	0.036313	*TMEM155*	3	-0.31638	0.834632	0.078505
RPS27A	1	0.71327	0.920057	0.036185	*POU2F2*	2	-0.29244	0.834632	0.078505
YIPF6	4	-0.3139	0.920135	0.036149	*MYO10*	4	-0.2513	0.834632	0.078505
PSME1	4	-0.20902	0.920135	0.036149	*RHEBL1*	4	-0.24027	0.834632	0.078505
SLC25A45	4	0.15222	0.920135	0.036149	*CEP170*	4	-0.21356	0.834632	0.078505
CSF1R	3	0.33406	0.920135	0.036149	*NECAB1*	4	-0.1856	0.834632	0.078505
GON4L	3	-0.49436	0.92014	0.036146	*ITFG2*	4	-0.18449	0.834632	0.078505
C1orf116	3	-0.4097	0.92014	0.036146	*SLC40A1*	4	-0.17485	0.834632	0.078505
CAMKMT	4	-0.38252	0.92014	0.036146	*GPRIN2*	4	-0.15181	0.834632	0.078505
ASPHD2	3	-0.35605	0.92014	0.036146	*STOM*	4	-0.13865	0.834632	0.078505
H4C4	3	-0.32825	0.92014	0.036146	*MKRN1*	4	-0.12996	0.834632	0.078505
OVOL1	3	-0.28901	0.92014	0.036146	*ODAPH*	4	-0.082591	0.834632	0.078505
WDR37	4	-0.27927	0.92014	0.036146	*SHROOM2*	3	-0.067119	0.834632	0.078505
NDUFAF5	4	-0.25155	0.92014	0.036146	*ATF7IP2*	4	-0.022401	0.834632	0.078505
MAP3K15	4	-0.2304	0.92014	0.036146	*CCNF*	4	-0.010173	0.834632	0.078505
MTHFS	3	-0.16619	0.92014	0.036146	*ABHD4*	3	0.012549	0.834632	0.078505
UMPS	3	-0.1458	0.92014	0.036146	*ST7*	4	0.027605	0.834632	0.078505
CMTR2	3	-0.049206	0.92014	0.036146	*GP6*	4	0.042324	0.834632	0.078505
TBC1D15	3	-0.02292	0.92014	0.036146	*LENG1*	4	0.045699	0.834632	0.078505
ADARB1	4	0.125	0.92014	0.036146	*SMIM14*	4	0.060806	0.834632	0.078505
CRYZL1	4	0.15224	0.92014	0.036146	*C20orf141*	4	0.082587	0.834632	0.078505
SEMA4C	4	0.18241	0.92014	0.036146	*FGG*	4	0.10706	0.834632	0.078505
FSCN1	4	0.18922	0.92014	0.036146	*C1GALT1C1L*	4	0.11725	0.834632	0.078505
MGLL	4	0.18935	0.92014	0.036146	*NIM1K*	4	0.12064	0.834632	0.078505
HAGH	4	0.20443	0.92014	0.036146	*KCTD3*	4	0.12239	0.834632	0.078505
ARPC2	4	0.24215	0.92014	0.036146	*ATP6V0C*	4	0.14883	0.834632	0.078505
IL6	1	0.33823	0.92014	0.036146	*ZNF576*	3	0.18207	0.834632	0.078505
NUF2	3	0.37344	0.92014	0.036146	*DCHS2*	4	0.19068	0.834632	0.078505
DGCR8	4	0.40307	0.92014	0.036146	*STXBP2*	4	0.19314	0.834632	0.078505
FAM227A	3	0.40435	0.92014	0.036146	*ACTR8*	4	0.21271	0.834632	0.078505
FTH1	3	0.44078	0.92014	0.036146	*DOCK11*	3	0.2132	0.834632	0.078505
RPL31	2	0.5431	0.92014	0.036146	*RAB9B*	3	0.24726	0.834632	0.078505
DENND1C	3	-0.26869	0.920307	0.036067	*SMARCD2*	3	0.25264	0.834632	0.078505
SRI	2	-0.21638	0.920307	0.036067	*CLUH*	3	0.25663	0.834632	0.078505

HOMEZ	3	-0.086925	0.920307	0.036067	*PTF1A*	3	0.26326	0.834632	0.078505
ERAP1	4	0.13977	0.920307	0.036067	*ZFR*	4	0.2674	0.834632	0.078505
SLC35E3	3	0.19714	0.920307	0.036067	*SRFBP1*	3	0.27768	0.834632	0.078505
FXYD1	3	0.29099	0.920307	0.036067	*BAHCC1*	4	0.31098	0.834632	0.078505
GABRB3	3	0.29242	0.920307	0.036067	*TRAPPC4*	4	0.3448	0.834632	0.078505
TEC	3	0.33808	0.920307	0.036067	*ATP1A3*	4	0.35147	0.834632	0.078505
SCYL1	3	0.34565	0.920307	0.036067	*PSMG1*	4	0.36329	0.834632	0.078505
C1QTNF12	4	0.38518	0.920307	0.036067	*POLR3E*	4	0.44815	0.834632	0.078505
TCF15	3	-0.34317	0.920529	0.035963	*LSR*	4	0.13525	0.83483	0.078402
CATIP	3	0.22981	0.920529	0.035963	*NME4*	4	0.25169	0.83483	0.078402
HMGXB4	3	0.20449	0.920849	0.035811	*UCP3*	2	-0.15854	0.834903	0.078364
C2orf27B	1	-0.42563	0.920947	0.035765	*CFAP77*	1	-0.58524	0.835008	0.078309
SLCO4C1	3	-0.4233	0.920947	0.035765	*EHD1*	4	-0.32894	0.835008	0.078309
ATP2A3	3	-0.39577	0.920947	0.035765	*NSD3*	3	-0.31802	0.835008	0.078309
TTC13	4	-0.37301	0.920947	0.035765	*NRM*	3	-0.31649	0.835008	0.078309
ATP11AUN	3	-0.36573	0.920947	0.035765	*ARTN*	3	-0.26157	0.835008	0.078309
PDS5B	3	-0.35394	0.920947	0.035765	*PDE10A*	4	-0.22154	0.835008	0.078309
LHX6	4	-0.21849	0.920947	0.035765	*ADGRF5*	4	-0.10965	0.835008	0.078309
DSC1	4	-0.13879	0.920947	0.035765	*YWHAH*	3	-0.058085	0.835008	0.078309
SLC3A1	4	-0.048394	0.920947	0.035765	*CCDC134*	4	0.024341	0.835008	0.078309
NFASC	4	0.039046	0.920947	0.035765	*ZSWIM7*	4	0.057678	0.835008	0.078309
GMPPA	3	0.042469	0.920947	0.035765	*TUBB4B*	2	0.16462	0.835008	0.078309
PRSS21	4	0.056999	0.920947	0.035765	*TAZ*	4	0.208	0.835008	0.078309
DHRS1	2	0.11451	0.920947	0.035765	*DYSF*	4	0.20985	0.835008	0.078309
MPP2	3	0.11909	0.920947	0.035765	*ZMAT3*	4	-0.21236	0.83543	0.07809
ATP6V1D	2	0.31533	0.920947	0.035765	*SHROOM4*	4	-0.16733	0.83543	0.07809
DDA1	3	0.35026	0.920947	0.035765	*PTPRF*	4	0.066186	0.83543	0.07809
CNGA1	3	-0.39059	0.920965	0.035757	*FYB2*	4	0.13378	0.83543	0.07809
NPC2	3	-0.30987	0.920965	0.035757	*MYO16*	4	0.15855	0.83543	0.07809
L3MBTL2	3	-0.17476	0.920965	0.035757	*DEPDC1B*	4	0.18351	0.83543	0.07809
CPNE8	3	-0.32323	0.921256	0.03562	*SNX24*	4	0.19492	0.83543	0.07809
LRIG3	4	-0.26241	0.921256	0.03562	*AMPD3*	4	0.25832	0.83543	0.07809
KDM8	4	-0.21753	0.921256	0.03562	*C1QTNF9*	1	-0.38582	0.835908	0.077842
SFMBT2	4	-0.098893	0.921256	0.03562	*CARMIL2*	2	0.076971	0.835908	0.077842
TSPAN4	4	0.084756	0.921256	0.03562	*SLC35E4*	2	0.14565	0.835908	0.077842
UMAD1	3	-0.45586	0.9214	0.035552	*CDKL2*	3	0.074023	0.83598	0.077804
CCDC42	4	-0.42949	0.9214	0.035552	*ZNF714*	3	-0.3955	0.836007	0.07779
NTF3	3	-0.4222	0.9214	0.035552	*MYO1E*	3	-0.39248	0.836007	0.07779
NUPR1	4	-0.37485	0.9214	0.035552	*TMEM177*	3	-0.18534	0.836007	0.07779
PRSS27	3	-0.32274	0.9214	0.035552	*ADA2*	3	0.21535	0.836007	0.07779
SLC2A6	4	-0.27835	0.9214	0.035552	*EIF1B*	3	0.20128	0.836015	0.077786
DMRTA1	4	-0.27134	0.9214	0.035552	*PIBF1*	3	0.32031	0.836015	0.077786
MYO5A	4	-0.21285	0.9214	0.035552	*LMAN2*	3	0.42853	0.836015	0.077786
AUH	4	-0.16533	0.9214	0.035552	*CCDC13*	4	-0.13181	0.836035	0.077776
CARD14	4	-0.15196	0.9214	0.035552	*ASF1A*	1	-0.51431	0.836172	0.077704
EHBP1	4	-0.10442	0.9214	0.035552	*ID1*	3	-0.39623	0.836172	0.077704
SPSB3	4	-0.050597	0.9214	0.035552	*AP3B2*	4	-0.33477	0.836172	0.077704
GOLPH3L	4	-0.025692	0.9214	0.035552	*LMTK3*	3	-0.30262	0.836172	0.077704
CHODL	4	-0.015577	0.9214	0.035552	*LMO7*	4	-0.20711	0.836172	0.077704
TPH2	3	-0.014081	0.9214	0.035552	*PAGE1*	4	-0.1989	0.836172	0.077704
DNAJB7	4	-0.003517	0.9214	0.035552	*BTLA*	4	-0.1614	0.836172	0.077704
SPATA46	3	0.097215	0.9214	0.035552	*EGFR*	4	0.013295	0.836172	0.077704
ASAP1	3	0.1893	0.9214	0.035552	*RAB8B*	4	0.030765	0.836172	0.077704
ZNF441	4	0.1961	0.9214	0.035552	*TMX1*	2	0.059704	0.836172	0.077704
KIF20A	4	0.23933	0.9214	0.035552	*C6orf118*	4	0.075583	0.836172	0.077704
ACAP1	2	0.26064	0.9214	0.035552	*ZNF689*	4	0.11508	0.836172	0.077704
INTS14	3	0.3439	0.9214	0.035552	*TEKT2*	3	0.1632	0.836172	0.077704
KANK2	2	0.35729	0.9214	0.035552	*PMM2*	4	0.24575	0.836172	0.077704

RPS3	3	0.57769	0.9214	0.035552	*AKNA*	4	0.2755	0.836172	0.077704
PSMA6	2	0.70814	0.921562	0.035475	*MRAS*	2	0.28532	0.836172	0.077704
LMAN2L	4	0.2116	0.922867	0.034861	*DRG1*	2	0.32293	0.836172	0.077704
HGF	4	-0.14139	0.923034	0.034782	*ERICH3*	2	0.38513	0.836172	0.077704
SLIT2	4	0.22424	0.923034	0.034782	*BRSK2*	2	0.39584	0.836172	0.077704
MFSD2B	3	-0.32349	0.923051	0.034774	*MTA1*	2	0.41708	0.836172	0.077704
RAB35	4	0.17887	0.923051	0.034774	*CARNS1*	4	-0.06912	0.836504	0.077532
NCAPD2	4	0.28765	0.923051	0.034774	*GIPC1*	2	-0.51751	0.8369	0.077327
SAFB2	1	-0.57873	0.923161	0.034723	*NUTM2A*	1	-0.46958	0.8369	0.077327
TIAL1	4	0.017209	0.923893	0.034378	*ZNF408*	2	-0.41043	0.8369	0.077327
ARMH4	4	-0.24482	0.923897	0.034377	*PIKFYVE*	3	-0.29211	0.8369	0.077327
VBP1	4	-0.12552	0.923897	0.034377	*ZKSCAN4*	4	-0.28799	0.8369	0.077327
GIPC3	4	-0.071062	0.923897	0.034377	*H3C7*	3	-0.26713	0.8369	0.077327
ZNF32	4	0.063296	0.923897	0.034377	*ARMT1*	4	-0.25355	0.8369	0.077327
NAT8L	4	0.077541	0.923897	0.034377	*CASQ2*	3	-0.22423	0.8369	0.077327
TMEM150C	4	0.14432	0.923897	0.034377	*NLGN4Y*	4	-0.22288	0.8369	0.077327
PLA2G2D	4	0.23705	0.923897	0.034377	*GSKIP*	4	-0.19096	0.8369	0.077327
ZBTB3	3	0.24905	0.923897	0.034377	*ITPKB*	4	-0.17967	0.8369	0.077327
VASP	3	0.2625	0.923897	0.034377	*TRAPPC9*	3	-0.14453	0.8369	0.077327
SUN2	1	0.47996	0.923897	0.034377	*C2orf69*	4	-0.12195	0.8369	0.077327
LRRN4	4	-0.26143	0.924706	0.033996	*PRPS2*	4	-0.036714	0.8369	0.077327
ZNF263	4	-0.24627	0.924706	0.033996	*KIAA1841*	4	-0.011657	0.8369	0.077327
MC5R	4	-0.16407	0.924706	0.033996	*USP43*	4	-0.000865	0.8369	0.077327
NFKBIZ	4	-0.093443	0.924706	0.033996	*BNIP2*	2	0.079716	0.8369	0.077327
CABP1	3	-0.008424	0.924706	0.033996	*SPIDR*	4	0.088127	0.8369	0.077327
RNASEH2B	4	0.29234	0.924706	0.033996	*USP9Y*	4	0.10682	0.8369	0.077327
ZNF2	3	-0.18175	0.924723	0.033989	*CACNG7*	4	0.12647	0.8369	0.077327
KCNH1	3	0.1352	0.924723	0.033989	*KCNG4*	4	0.13795	0.8369	0.077327
TMEM266	3	0.28111	0.924723	0.033989	*TPTE2*	3	0.15913	0.8369	0.077327
CITED2	2	-0.27512	0.924744	0.033978	*CTR9*	4	0.17194	0.8369	0.077327
APOH	2	-0.22329	0.924744	0.033978	*LRP3*	2	0.26687	0.8369	0.077327
AP5M1	2	-0.18779	0.924744	0.033978	*TRIM52*	3	0.27267	0.8369	0.077327
OXTR	4	-0.03527	0.924744	0.033978	*SLC25A5*	3	0.31675	0.8369	0.077327
DACT1	4	0.23131	0.924905	0.033903	*SAT1*	3	0.34458	0.8369	0.077327
CWF19L2	4	-0.037079	0.925557	0.033597	*METAP1*	4	0.34683	0.8369	0.077327
RASL12	4	-0.004207	0.925557	0.033597	*COMTD1*	4	-0.044407	0.837062	0.077242
TRIP4	4	0.042639	0.925557	0.033597	*BCL6*	4	-0.029182	0.837062	0.077242
IL7R	4	0.17163	0.925557	0.033597	*ZFP69*	4	0.28338	0.837062	0.077242
CDC5L	4	0.17977	0.925557	0.033597	*DHRS7B*	4	-0.2723	0.838231	0.076636
POLD3	4	0.31223	0.925557	0.033597	*ZNF266*	4	-0.26897	0.838231	0.076636
ANKRD44	3	-0.34166	0.925856	0.033456	*NFE2L1*	4	-0.045275	0.838231	0.076636
DCAF5	2	-0.33639	0.925856	0.033456	*CALR*	4	0.04786	0.838231	0.076636
FEZ2	3	-0.21175	0.925856	0.033456	*CNTN6*	4	0.11698	0.838231	0.076636
GTF2I	4	-0.11859	0.925856	0.033456	*CLPSL2*	3	0.26075	0.838231	0.076636
E2F7	4	0.085934	0.925856	0.033456	*FAM157B*	1	0.39584	0.838231	0.076636
MIR1915HG	1	0.26102	0.925856	0.033456	*C7orf57*	3	0.28476	0.839024	0.076226
NLRP13	4	0.2712	0.925856	0.033456	*TOX2*	3	-0.36349	0.839101	0.076186
HAND1	4	0.29747	0.925856	0.033456	*IFNAR1*	3	-0.18202	0.839101	0.076186
CDRT15	2	0.11029	0.926187	0.033302	*LRRC14B*	4	0.10957	0.839101	0.076186
FGB	2	0.14082	0.926187	0.033302	*DMTN*	4	0.18016	0.839101	0.076186
FOXJ1	4	0.18427	0.926187	0.033302	*NAGPA*	4	-0.21482	0.839261	0.076103
CD96	3	0.25138	0.926187	0.033302	*TRIQK*	4	-0.18132	0.839261	0.076103
MINAR2	4	-0.22807	0.926202	0.033294	*MLLT1*	3	0.17101	0.839261	0.076103
LEPR	3	-0.15764	0.926202	0.033294	*RBM4*	2	-0.2499	0.839384	0.07604
NBPF8	3	0.22084	0.926202	0.033294	*PPCS*	3	-0.038488	0.839666	0.075893
PDZD7	4	0.01824	0.926315	0.033241	*SF3B4*	3	-0.030764	0.839666	0.075893
SSH3	4	0.23644	0.926315	0.033241	*SLC41A2*	4	0.16106	0.839666	0.075893
FAM209A	1	-0.36145	0.926475	0.033166	*KLF16*	1	0.39607	0.839666	0.075893

KIFC3	4	-0.066787	0.926995	0.032923	*ITGB4*	3	0.11453	0.840046	0.075697
THSD7A	2	-0.40103	0.927189	0.032832	*ANKDD1B*	4	-0.34784	0.840444	0.075491
FPR1	4	-0.19514	0.927189	0.032832	*B3GNTL1*	4	-0.14294	0.840444	0.075491
KIAA1109	4	-0.16461	0.927189	0.032832	*CREB1*	3	-0.52205	0.840484	0.07547
FCER2	3	-0.007935	0.927189	0.032832	*ENO4*	3	-0.44525	0.840484	0.07547
ACSL4	4	0.037249	0.927189	0.032832	*PPIC*	3	-0.4144	0.840484	0.07547
AVIL	4	0.21113	0.927189	0.032832	*CELA3B*	3	-0.30374	0.840484	0.07547
RALBP1	4	0.21319	0.927189	0.032832	*IGHMBP2*	3	-0.27431	0.840484	0.07547
MAT2A	4	0.236	0.927189	0.032832	*PRDM11*	3	-0.24452	0.840484	0.07547
GSDMC	2	-0.47486	0.927209	0.032822	*TMEM191B*	1	-0.24122	0.840484	0.07547
LPCAT2	2	-0.17692	0.927209	0.032822	*DCAF11*	3	-0.20396	0.840484	0.07547
HPSE	3	0.10246	0.927209	0.032822	*AGMO*	3	-0.18086	0.840484	0.07547
CAPNS1	4	0.22175	0.927209	0.032822	*GPR61*	2	-0.15969	0.840484	0.07547
MAP7	3	0.087854	0.927317	0.032772	*FUT3*	4	-0.15498	0.840484	0.07547
CPNE9	3	-0.39316	0.927401	0.032733	*TACR2*	4	-0.10728	0.840484	0.07547
BCL2	2	-0.32058	0.927401	0.032733	*VAX1*	3	-0.097158	0.840484	0.07547
ZNF282	2	-0.30879	0.927401	0.032733	*AAR2*	4	-0.071797	0.840484	0.07547
SELENOS	2	-0.22605	0.927401	0.032733	*TNKS*	3	-0.06659	0.840484	0.07547
ZNF212	4	-0.21793	0.927401	0.032733	*GPRC5A*	3	-0.026232	0.840484	0.07547
ZBED4	4	-0.14559	0.927401	0.032733	*OLIG3*	4	0.00998	0.840484	0.07547
PPP2R2B	4	-0.045936	0.927401	0.032733	*MPZ*	4	0.023857	0.840484	0.07547
PLA2G12A	4	-0.035278	0.927401	0.032733	*MAOA*	4	0.054525	0.840484	0.07547
CTPS2	4	0.067843	0.927401	0.032733	*EIF1AY*	2	0.075407	0.840484	0.07547
KLHDC7B	3	0.09803	0.927401	0.032733	*TBC1D28*	3	0.10188	0.840484	0.07547
FAM135A	4	0.16747	0.927401	0.032733	*GRIN3A*	4	0.1207	0.840484	0.07547
IFT22	4	0.19252	0.927401	0.032733	*PLCB3*	4	0.13142	0.840484	0.07547
NPRL3	4	0.1931	0.927401	0.032733	*H2AJ*	4	0.13433	0.840484	0.07547
KLK14	3	0.24027	0.927401	0.032733	*SULT1C4*	4	0.15918	0.840484	0.07547
RGMB	3	0.25554	0.927401	0.032733	*CMTR2*	3	0.178	0.840484	0.07547
RRAD	3	0.30577	0.927401	0.032733	*TRAF2*	4	0.2022	0.840484	0.07547
CLCC1	2	0.67687	0.927401	0.032733	*HOXA5*	2	0.21101	0.840484	0.07547
PPP2R3A	3	-0.14022	0.92764	0.032621	*TRIP12*	4	0.23842	0.840484	0.07547
ERBB3	4	-0.094811	0.92764	0.032621	*CKMT2*	2	0.26263	0.840484	0.07547
CETN2	3	0.014238	0.92764	0.032621	*VPS9D1*	3	0.28505	0.840484	0.07547
SNX4	4	0.12833	0.92764	0.032621	*C6orf15*	3	0.31074	0.840484	0.07547
SCNN1G	4	0.12977	0.92764	0.032621	*FBXO48*	2	-0.024829	0.840657	0.075381
IRGQ	4	0.13763	0.92764	0.032621	*C2CD2L*	3	-0.3809	0.840761	0.075327
ACADS	4	0.257	0.92764	0.032621	*LACTB2*	4	-0.28607	0.840761	0.075327
IL1RL2	4	-0.2682	0.927798	0.032546	*ZNF485*	4	-0.19615	0.840761	0.075327
ZNF180	4	0.064492	0.927859	0.032518	*IFITM1*	2	-0.1062	0.840761	0.075327
SNW1	4	0.23885	0.927859	0.032518	*PWWP2B*	2	-0.097364	0.840761	0.075327
RBMS1	4	-0.35169	0.927922	0.032488	*MYO1A*	3	-0.069932	0.840761	0.075327
SWI5	4	-0.27006	0.927922	0.032488	*CBFB*	4	-0.014654	0.840761	0.075327
REM1	4	-0.26822	0.927922	0.032488	*NANS*	4	0.12491	0.840761	0.075327
BORCS5	4	-0.2449	0.927922	0.032488	*ADAM12*	3	0.13885	0.840761	0.075327
ADORA1	4	-0.15629	0.927922	0.032488	*SPRED2*	3	0.22093	0.840761	0.075327
GPR3	4	-0.14727	0.927922	0.032488	*PTPRB*	3	0.25494	0.840761	0.075327
DRAM2	4	-0.067386	0.927922	0.032488	*FIGNL2*	3	0.22477	0.840901	0.075255
DNASE1L2	2	-0.024405	0.927922	0.032488	*SKA3*	3	0.36558	0.840901	0.075255
KIAA0754	4	-0.003714	0.927922	0.032488	*STRN3*	3	-0.3435	0.841003	0.075202
KCNJ9	4	0.10049	0.927922	0.032488	*PACSIN2*	4	-0.30501	0.841003	0.075202
LDB3	4	0.13589	0.927922	0.032488	*TUBB8*	2	-0.28701	0.841003	0.075202
ZNF428	4	0.16781	0.927922	0.032488	*TLR2*	2	-0.28319	0.841003	0.075202
TESPA1	4	0.23805	0.927922	0.032488	*GALNT16*	4	-0.24227	0.841003	0.075202
SIGLEC10	3	-0.25225	0.928488	0.032224	*TMEM86A*	2	-0.18368	0.841003	0.075202
TAF6	2	-0.78358	0.928609	0.032167	*CT45A10*	4	-0.17856	0.841003	0.075202
CCDC39	4	-0.13224	0.928609	0.032167	*C3orf18*	4	-0.17146	0.841003	0.075202
RASSF7	4	0.17649	0.928609	0.032167	*SYNPO2*	3	-0.14859	0.841003	0.075202

R3HCC1L	4	0.22486	0.928609	0.032167	*ZDHHC7*	4	-0.13025	0.841003	0.075202
CALHM6	3	0.23714	0.929481	0.03176	*TLK1*	4	-0.12598	0.841003	0.075202
WDR49	3	-0.3605	0.929546	0.031729	*POGLUT2*	4	-0.10676	0.841003	0.075202
JMJD4	3	-0.1034	0.929546	0.031729	*BOK*	4	-0.10278	0.841003	0.075202
LRRC15	4	0.20291	0.929546	0.031729	*ELOVL2*	2	-0.083733	0.841003	0.075202
CYP4V2	4	0.022907	0.929652	0.03168	*USP27X*	4	-0.081131	0.841003	0.075202
RBMX2	4	-0.21706	0.92967	0.031671	*NR2E1*	4	-0.051815	0.841003	0.075202
CCDC175	4	-0.20066	0.92967	0.031671	*GREM1*	4	-0.047611	0.841003	0.075202
PEF1	4	-0.19618	0.92967	0.031671	*ANGPT1*	4	-0.046932	0.841003	0.075202
GNPDA1	4	-0.01029	0.92967	0.031671	*H2BC9*	3	-0.024452	0.841003	0.075202
SEH1L	3	0.33775	0.930286	0.031384	*JAML*	4	-0.020304	0.841003	0.075202
DDX60	3	0.31658	0.930596	0.031239	*LNX1*	4	-0.01547	0.841003	0.075202
CCDC88B	2	-0.17089	0.931262	0.030928	*KIF20A*	4	-0.005934	0.841003	0.075202
CHMP7	3	0.037439	0.932288	0.03045	*ARL14EPL*	4	0.043269	0.841003	0.075202
KIAA1324L	4	0.18381	0.932288	0.03045	*PLXNB3*	4	0.074181	0.841003	0.075202
CDV3	3	-0.19225	0.932809	0.030207	*UNCX*	4	0.17936	0.841003	0.075202
GRB7	4	-0.1745	0.932809	0.030207	*CAMLG*	4	0.27044	0.841003	0.075202
B4GALT6	4	-0.13466	0.932809	0.030207	*CRELD1*	3	0.27194	0.841003	0.075202
DDHD2	4	-0.28305	0.93297	0.030132	*TEX12*	4	0.2888	0.841003	0.075202
PAQR4	4	0.1814	0.93297	0.030132	*PAICS*	3	0.3475	0.841003	0.075202
ULK3	3	-0.38804	0.933029	0.030105	*TMEM69*	2	0.35927	0.841003	0.075202
VWA3B	3	-0.066324	0.933029	0.030105	*TMIGD3*	4	0.096659	0.841701	0.074842
ABHD5	2	0.46004	0.933134	0.030056	*CACNA1H*	4	0.25864	0.841701	0.074842
KHSRP	3	-0.29551	0.933416	0.029925	*OR2A14*	4	-0.31297	0.84233	0.074518
NDRG4	4	-0.2382	0.933416	0.029925	*CYP3A5*	3	-0.48964	0.842351	0.074507
SLC18A3	2	-0.2095	0.933416	0.029925	*TNFAIP8*	3	-0.27664	0.842351	0.074507
MCAM	3	-0.18862	0.933416	0.029925	*HMGA2*	4	-0.27337	0.842351	0.074507
TLR6	4	-0.17411	0.933416	0.029925	*SMUG1*	4	-0.26058	0.842351	0.074507
MRLN	3	0.12629	0.933416	0.029925	*CSAG1*	4	-0.24498	0.842351	0.074507
GPR78	3	0.37628	0.933416	0.029925	*ZNF843*	4	-0.23655	0.842351	0.074507
GOLGA6L2	3	-0.05477	0.933982	0.029661	*EPS15L1*	4	-0.23547	0.842351	0.074507
ARHGAP17	3	0.25484	0.933982	0.029661	*CCDC112*	4	-0.20501	0.842351	0.074507
S100A2	4	0.006326	0.934088	0.029612	*MGST2*	3	-0.15757	0.842351	0.074507
LIMS2	3	-0.50871	0.934657	0.029348	*GDPD5*	4	-0.15192	0.842351	0.074507
MNAT1	3	-0.39341	0.934657	0.029348	*PCDHGA10*	3	-0.1426	0.842351	0.074507
USP49	3	-0.13895	0.934657	0.029348	*TAF9B*	4	-0.095196	0.842351	0.074507
FAM49B	2	0.1418	0.934715	0.029321	*KHDRBS3*	4	-0.068795	0.842351	0.074507
VLDLR	3	0.26228	0.934715	0.029321	*KCNS3*	4	0.003996	0.842351	0.074507
TASOR2	4	-0.24111	0.934773	0.029294	*HDAC11*	2	0.050219	0.842351	0.074507
IRAK1	4	-0.13222	0.934773	0.029294	*ATP12A*	4	0.050944	0.842351	0.074507
S100A4	4	0.026501	0.934991	0.029193	*KPNA7*	4	0.085272	0.842351	0.074507
PUM3	4	0.31453	0.934991	0.029193	*DMBT1*	4	0.11185	0.842351	0.074507
EXOSC2	3	0.40211	0.934991	0.029193	*EHF*	4	0.14631	0.842351	0.074507
TUT1	4	0.47036	0.934991	0.029193	*DNAH14*	3	0.17622	0.842351	0.074507
RBBP5	1	-0.51423	0.935352	0.029025	*TM4SF4*	4	0.19328	0.842351	0.074507
PLEKHO1	2	-0.093959	0.935352	0.029025	*ZNF48*	4	0.19662	0.842351	0.074507
DENND4C	3	-0.38839	0.935908	0.028767	*NFE4*	4	0.29941	0.842351	0.074507
HOXD4	1	-0.3039	0.935908	0.028767	*H2BC13*	2	0.37348	0.842351	0.074507
ATAD5	2	-0.2384	0.935908	0.028767	*C4orf3*	2	0.42128	0.842351	0.074507
AGO3	3	-0.20217	0.935908	0.028767	*RPS4X*	3	0.45236	0.842351	0.074507
CYP51A1	4	-0.1875	0.935908	0.028767	*RNF19A*	4	-0.26878	0.84248	0.074441
CSF2RA	3	-0.17542	0.935908	0.028767	*MYBPC1*	4	-0.24918	0.84248	0.074441
CBL	2	-0.15712	0.935908	0.028767	*ALOX15*	4	-0.19557	0.84248	0.074441
IGLL5	2	-0.070766	0.935908	0.028767	*SMURF2*	4	-0.18274	0.84248	0.074441
ZNF701	4	0.094035	0.935908	0.028767	*GPR32*	4	4.80E-04	0.84248	0.074441
AGAP1	3	0.2313	0.935908	0.028767	*MAP3K1*	4	0.001637	0.84248	0.074441
BMP2	3	0.28197	0.935908	0.028767	*ARL1*	4	0.092102	0.84248	0.074441
FBXW8	3	0.29325	0.935908	0.028767	*AKTIP*	2	0.040712	0.84265	0.074353

ULBP2	2	0.3376	0.935908	0.028767	*OPN3*	2	-0.49975	0.842686	0.074334
CNNM3	3	0.35288	0.935908	0.028767	*HLA-DRB1*	2	-0.14403	0.842686	0.074334
SNAPC3	3	-0.54557	0.936326	0.028573	*UBASH3B*	3	-0.35686	0.842899	0.074224
CYTIP	3	-0.34993	0.936326	0.028573	*FGF18*	3	-0.27796	0.842899	0.074224
SERPINB8	4	-0.2179	0.936326	0.028573	*TCAF1*	2	-0.11383	0.842899	0.074224
GYPC	4	0.058925	0.936326	0.028573	*TMEM102*	4	-0.085364	0.842899	0.074224
TOM1	3	-0.50477	0.936804	0.028351	*KCNMB1*	4	-0.06315	0.842899	0.074224
CNKSR3	3	-0.33824	0.936804	0.028351	*ZNF677*	4	-0.046269	0.842899	0.074224
PANK3	4	-0.20611	0.936804	0.028351	*NSRP1*	4	-0.031547	0.842899	0.074224
EPS8L1	4	-0.16056	0.936804	0.028351	*SDK2*	4	0.066371	0.842899	0.074224
TRIP13	3	0.003848	0.936804	0.028351	*M1AP*	3	0.068602	0.842899	0.074224
MED30	2	0.21916	0.936804	0.028351	*NPFFR2*	4	0.072173	0.842899	0.074224
GRWD1	3	0.37163	0.936804	0.028351	*ILDR2*	4	0.085859	0.842899	0.074224
STK39	3	-0.42135	0.937124	0.028203	*SMIM3*	4	0.091721	0.842899	0.074224
FHOD3	4	-0.21987	0.937124	0.028203	*ZNF385C*	4	0.12424	0.842899	0.074224
SPPL2A	4	-0.079038	0.937124	0.028203	*HEXA*	3	0.14057	0.842899	0.074224
DBNL	4	0.065284	0.937124	0.028203	*TMEM91*	4	0.20335	0.842899	0.074224
NUP98	4	0.25583	0.937124	0.028203	*MAEA*	4	0.21509	0.842899	0.074224
SLC47A2	3	-0.2972	0.93748	0.028038	*SNCG*	3	0.28983	0.842899	0.074224
MKNK1	1	0.38894	0.937886	0.02785	*POLR2F*	2	0.30376	0.842899	0.074224
ZNF677	4	-0.29482	0.937993	0.0278	*RBBP5*	1	0.35015	0.842899	0.074224
SBDS	4	0.29268	0.937993	0.0278	*C17orf50*	3	0.35858	0.842899	0.074224
ZFP57	4	-0.40041	0.938201	0.027704	*GRAP*	1	-0.60074	0.842972	0.074187
CDHR5	3	-0.3059	0.938201	0.027704	*GSTA1*	1	-0.47523	0.842972	0.074187
NUP43	4	-0.23739	0.938201	0.027704	*RNF152*	4	-0.13342	0.842972	0.074187
OXR1	4	-0.23479	0.938201	0.027704	*CFAP410*	2	0.10823	0.842972	0.074187
ANKRD40	4	-0.19301	0.938201	0.027704	*GOLIM4*	3	-0.29842	0.843008	0.074168
SCPEP1	4	-0.15762	0.938201	0.027704	*SH3PXD2B*	3	-0.1271	0.843008	0.074168
TAS2R42	4	-0.13583	0.938201	0.027704	*ELOVL1*	4	-0.4296	0.843304	0.074016
SLC39A14	2	-0.10239	0.938201	0.027704	*SPOP*	2	-0.30818	0.843304	0.074016
TMEM132B	4	-0.073066	0.938201	0.027704	*MTCP1*	4	-0.10097	0.843304	0.074016
LEFTY2	3	-0.059177	0.938201	0.027704	*ZDHHC20*	4	-0.093871	0.843304	0.074016
LRRC75A	4	-0.043846	0.938201	0.027704	*FIP1L1*	4	0.26109	0.843304	0.074016
TRAPPC8	3	-0.017463	0.938201	0.027704	*THOC7*	4	0.28223	0.843304	0.074016
NAV1	4	0.045856	0.938201	0.027704	*ACP6*	4	0.29656	0.843304	0.074016
ASB9	3	0.06717	0.938201	0.027704	*LOC10192732*	1	0.32445	0.843304	0.074016
GSPT2	4	0.075372	0.938201	0.027704	*LEPROT*	3	-0.44719	0.84334	0.073997
LOC149373	2	0.11574	0.938201	0.027704	*HIP1R*	3	-0.056537	0.84334	0.073997
NLRC5	4	0.23517	0.938201	0.027704	*WNT4*	4	-0.23701	0.843444	0.073944
TRIB1	2	0.24121	0.938201	0.027704	*CFAP97D1*	4	0.004812	0.843444	0.073944
BCORL1	4	-0.16638	0.93821	0.0277	*SNX22*	3	0.17361	0.843444	0.073944
AHCYL1	4	-0.074084	0.93821	0.0277	*SLC44A1*	2	-0.085511	0.843448	0.073942
CIDEB	3	-0.005721	0.93821	0.0277	*THTPA*	4	0.18374	0.843448	0.073942
OPHN1	2	-0.30751	0.938263	0.027675	*PHLDA2*	1	0.49543	0.843448	0.073942
EEF1B2	1	-0.34509	0.938621	0.02751	*SH3D21*	2	-0.11659	0.843642	0.073842
MRPL3	2	0.30437	0.938621	0.02751	*SGIP1*	4	-0.070932	0.843642	0.073842
H2BC11	3	0.14185	0.93863	0.027506	*RNF225*	4	-0.043317	0.843642	0.073842
CLEC11A	4	0.22639	0.93863	0.027506	*SLC23A3*	4	0.10316	0.843642	0.073842
TWNK	4	0.27581	0.93863	0.027506	*HEMK1*	4	0.12547	0.843642	0.073842
MARF1	4	-0.24553	0.93874	0.027455	*SMARCE1*	4	0.15661	0.843642	0.073842
ANKFN1	4	-0.23324	0.93874	0.027455	*YBX1*	1	0.23493	0.843642	0.073842
FBXO10	4	-0.18549	0.93874	0.027455	*KCNIP2*	2	0.32223	0.843642	0.073842
DES	2	-0.3647	0.938743	0.027453	*EIF2AK3*	4	-0.10005	0.844749	0.073273
PRSS45P	4	0.16758	0.938846	0.027406	*PAGE2B*	2	0.12298	0.844749	0.073273
ABCA4	3	-0.46442	0.938999	0.027335	*ELP2*	4	0.24759	0.844749	0.073273
MCUB	3	-0.3018	0.939105	0.027286	*NUP98*	4	-0.016951	0.844985	0.073151
AFF2	3	-0.05799	0.939105	0.027286	*NRN1L*	4	0.053534	0.844985	0.073151
PLXDC2	3	-0.43638	0.939474	0.027115	*USP48*	4	-0.023697	0.84522	0.07303

HLA-E	4	0.15445	0.939474	0.027115	*TLE4*	4	0.1041	0.84522	0.07303
KCNIP1	3	0.16836	0.939474	0.027115	*SFRP1*	4	-0.095456	0.845639	0.072815
NAALADL1	4	0.17064	0.939474	0.027115	*UBE3A*	2	0.19943	0.845806	0.072729
H2BU1	3	0.26185	0.939474	0.027115	*RBP5*	4	-0.24732	0.845892	0.072685
MIS18BP1	3	0.37341	0.939474	0.027115	*DHX35*	4	-0.2437	0.845892	0.072685
MANF	4	0.34124	0.939727	0.026998	*FAM189B*	3	-0.42283	0.846077	0.07259
GDNF-AS1	2	-0.38137	0.940081	0.026835	*TRIM25*	4	-0.3592	0.846077	0.07259
MKRN3	2	-0.25597	0.940081	0.026835	*CCNB2*	4	-0.22213	0.846167	0.072544
TMEM69	2	-0.22439	0.940081	0.026835	*GSDME*	4	-0.19353	0.846167	0.072544
CASZ1	4	-0.15879	0.940081	0.026835	*FAM149A*	4	-0.12444	0.846167	0.072544
CCDC191	4	-0.15114	0.940081	0.026835	*SYTL2*	3	0.1091	0.846167	0.072544
MMAB	4	-0.099908	0.940081	0.026835	*D2HGDH*	4	0.25287	0.846167	0.072544
RAVER1	4	-0.004869	0.940081	0.026835	*ZBTB20*	4	0.25443	0.846167	0.072544
TTC5	4	0.047485	0.940081	0.026835	*GRK2*	4	0.26893	0.846167	0.072544
TXNDC15	4	0.061077	0.940081	0.026835	*PRDX2*	3	0.33972	0.846167	0.072544
PTPN18	3	0.19431	0.940081	0.026835	*MOB1B*	4	-0.16366	0.846434	0.072407
NARF	4	0.19676	0.940081	0.026835	*NT5DC3*	3	0.21226	0.847051	0.07209
ABHD4	3	0.21685	0.940081	0.026835	*C10orf95*	3	-0.24342	0.847169	0.07203
POLK	3	0.2804	0.940081	0.026835	*ATAD2*	3	-0.3586	0.847691	0.071762
UQCRH	1	0.28667	0.940081	0.026835	*ALOX15B*	3	-0.16458	0.847691	0.071762
ZNF488	3	0.29026	0.940081	0.026835	*PSD3*	4	-0.084596	0.847691	0.071762
MERTK	3	0.31384	0.940081	0.026835	*BCL2*	2	-0.058506	0.847691	0.071762
SEMA6B	3	0.33072	0.940081	0.026835	*OR13J1*	3	-0.018902	0.847691	0.071762
KLHL5	3	0.3953	0.940081	0.026835	*RAB43*	3	-0.018595	0.847691	0.071762
OTOP2	1	0.52417	0.940081	0.026835	*TRPC7*	4	0.020267	0.847691	0.071762
PCGF3	4	0.013339	0.940682	0.026557	*LAX1*	4	0.094819	0.847691	0.071762
GRIN1	2	-0.43912	0.940698	0.02655	*CDK17*	2	0.1091	0.847691	0.071762
ZNF846	4	0.077924	0.940698	0.02655	*SOD2*	3	0.51125	0.847691	0.071762
NUP210	4	0.20974	0.940698	0.02655	*CHD5*	1	-0.45032	0.847708	0.071754
ARL10	4	0.26651	0.940698	0.02655	*GPATCH2*	4	0.17226	0.848772	0.071209
BUD13	4	0.2987	0.940698	0.02655	*USP9X*	2	0.56669	0.848889	0.071149
NIPAL4	4	0.34094	0.940698	0.02655	*TEX44*	4	-0.35249	0.849838	0.070664
CAPZA2	3	0.24299	0.940851	0.026479	*HSPBAP1*	4	-0.16578	0.849838	0.070664
RIC8B	4	-0.012662	0.941262	0.026289	*SYP*	4	-0.077289	0.849838	0.070664
CRMP1	4	0.10394	0.941262	0.026289	*PALM*	4	-0.052547	0.849838	0.070664
PRKD3	4	0.20373	0.941262	0.026289	*FAM83B*	4	0.036373	0.849838	0.070664
RNF208	4	0.21372	0.941262	0.026289	*RHEX*	4	0.2282	0.849838	0.070664
TMEM229B	4	0.23776	0.941262	0.026289	*EPHA4*	4	0.029973	0.850153	0.070503
UQCR10	4	-0.1964	0.941763	0.026058	*STK38L*	4	-0.094444	0.851846	0.069639
SPART	3	0.32071	0.941964	0.025965	*ACTR6*	4	0.24774	0.851846	0.069639
FGF2	3	-0.32114	0.942066	0.025918	*SRR*	3	0.39206	0.851846	0.069639
OAS2	4	-0.21797	0.942121	0.025893	*TMEM121*	4	-0.29193	0.852159	0.069479
RBM15	4	-0.18039	0.942121	0.025893	*SPON1*	3	-0.28333	0.852159	0.069479
FAM131A	4	-0.23987	0.942422	0.025754	*KLC1*	4	-0.21003	0.852159	0.069479
SLC38A5	3	-0.2813	0.942574	0.025685	*ZNF419*	4	-0.19689	0.852159	0.069479
PPP6R2	4	-0.24644	0.942633	0.025657	*ACADVL*	3	-0.13364	0.852159	0.069479
MELK	4	-0.22351	0.942633	0.025657	*RAB3GAP2*	4	0.001183	0.852159	0.069479
ARHGEF26	3	0.053856	0.942633	0.025657	*ABL1*	4	0.052215	0.852159	0.069479
AMDHD2	4	0.11126	0.942633	0.025657	*CRLF1*	4	0.075424	0.852159	0.069479
TSTD3	4	0.10133	0.943831	0.025106	*HTR3D*	4	0.086258	0.852159	0.069479
ACTR8	4	0.16585	0.943831	0.025106	*IL34*	4	0.1014	0.852159	0.069479
SLC6A1	4	-0.22692	0.944137	0.024965	*HUS1*	4	0.12106	0.852159	0.069479
OR10A3	4	-0.16678	0.944137	0.024965	*ARHGAP44*	4	0.12204	0.852159	0.069479
TMEM127	4	-0.040759	0.944137	0.024965	*ORC1*	4	0.17416	0.852159	0.069479
SCUBE2	2	0.053427	0.944137	0.024965	*MAST1*	4	0.17423	0.852159	0.069479
CNTNAP3B	1	-0.62909	0.944432	0.024829	*RNF10*	2	0.1985	0.852159	0.069479
MYO1H	3	-0.43525	0.944432	0.024829	*RECQL4*	4	0.21197	0.852159	0.069479
NF1	4	-0.40543	0.944432	0.024829	*NFYA*	4	0.22471	0.852159	0.069479

STK31	2	-0.39744	0.944432	0.024829	*ATP8B3*	4	0.22482	0.852159	0.069479
EIF3J	3	-0.39213	0.944432	0.024829	*NUDCD3*	4	0.24244	0.852159	0.069479
MEA1	3	-0.37866	0.944432	0.024829	*SREK1IP1*	1	0.25173	0.852159	0.069479
PSG8	1	-0.3699	0.944432	0.024829	*DZIP3*	4	0.2636	0.852159	0.069479
EMILIN2	3	-0.35808	0.944432	0.024829	*TSR3*	3	0.38886	0.852159	0.069479
C8orf44	3	-0.34667	0.944432	0.024829	*CHL1*	3	-0.34081	0.852308	0.069403
EPPK1	2	-0.32259	0.944432	0.024829	*P2RX4*	3	-0.27951	0.852308	0.069403
FOXD3	3	-0.31935	0.944432	0.024829	*RHBDF1*	3	0.24292	0.852308	0.069403
KLF9	4	-0.31457	0.944432	0.024829	*TTLL1*	3	-0.45352	0.852764	0.069171
TMEM64	2	-0.31164	0.944432	0.024829	*FAM83H*	3	-0.41464	0.852764	0.069171
THAP7	4	-0.30194	0.944432	0.024829	*DEK*	4	-0.34973	0.852764	0.069171
TDP2	4	-0.29001	0.944432	0.024829	*MFSD13A*	4	-0.25497	0.852764	0.069171
TUSC3	3	-0.25534	0.944432	0.024829	*JAK1*	4	-0.18414	0.852764	0.069171
ARHGAP10	3	-0.24952	0.944432	0.024829	*LAMP5*	4	-0.16585	0.852764	0.069171
SMPD2	3	-0.24523	0.944432	0.024829	*KLHL4*	4	-0.10184	0.852764	0.069171
CFAP44	4	-0.2361	0.944432	0.024829	*IER2*	4	-0.082888	0.852764	0.069171
COX7A2	4	-0.22752	0.944432	0.024829	*ABRAXAS2*	4	-0.079464	0.852764	0.069171
PABPC3	4	-0.22449	0.944432	0.024829	*B3GNT9*	4	-0.077068	0.852764	0.069171
CTDSP1	4	-0.22326	0.944432	0.024829	*CEMIP2*	3	-0.054298	0.852764	0.069171
ZNF649	4	-0.22037	0.944432	0.024829	*TBC1D9*	4	-0.034364	0.852764	0.069171
SORBS3	4	-0.20007	0.944432	0.024829	*SULT2A1*	4	0.039027	0.852764	0.069171
KCNN4	3	-0.18664	0.944432	0.024829	*TSPAN2*	4	0.051692	0.852764	0.069171
ADRB2	4	-0.18259	0.944432	0.024829	*ABHD10*	4	0.05189	0.852764	0.069171
RPS6KA6	3	-0.18186	0.944432	0.024829	*EMC1*	4	0.081512	0.852764	0.069171
ZADH2	4	-0.17561	0.944432	0.024829	*THEG5*	3	0.082707	0.852764	0.069171
OTOA	4	-0.16847	0.944432	0.024829	*TMPRSS11D*	4	0.095843	0.852764	0.069171
CCIN	4	-0.16601	0.944432	0.024829	*OSCAR*	3	0.10032	0.852764	0.069171
CCT6A	3	-0.16514	0.944432	0.024829	*ST13*	4	0.11672	0.852764	0.069171
FGFR1OP	4	-0.15946	0.944432	0.024829	*SCAMP5*	3	0.11728	0.852764	0.069171
NFAM1	4	-0.15873	0.944432	0.024829	*UCK2*	4	0.12114	0.852764	0.069171
HOMER1	4	-0.15802	0.944432	0.024829	*EBPL*	2	0.15303	0.852764	0.069171
GP1BA	2	-0.14189	0.944432	0.024829	*USP31*	2	0.16202	0.852764	0.069171
ABCA7	4	-0.12359	0.944432	0.024829	*PPP1R42*	3	0.18061	0.852764	0.069171
RNFT2	4	-0.12133	0.944432	0.024829	*ZNF502*	2	0.18205	0.852764	0.069171
GLIS2	2	-0.10707	0.944432	0.024829	*ZFYVE21*	3	0.18318	0.852764	0.069171
PI4K2B	4	-0.1033	0.944432	0.024829	*SMAD4*	4	0.18597	0.852764	0.069171
UBQLNL	3	-0.080549	0.944432	0.024829	*CCDC96*	3	0.19767	0.852764	0.069171
ZNF559	4	-0.068587	0.944432	0.024829	*INPP5A*	4	0.20522	0.852764	0.069171
SLC2A12	4	-0.058317	0.944432	0.024829	*GBGT1*	3	0.20893	0.852764	0.069171
STX6	3	-0.05445	0.944432	0.024829	*PPP1R12B*	4	0.24596	0.852764	0.069171
MEST	4	-0.048499	0.944432	0.024829	*VXN*	1	0.25347	0.852764	0.069171
CHN1	4	-0.046762	0.944432	0.024829	*PNKP*	3	0.25817	0.852764	0.069171
RCOR3	4	-0.026432	0.944432	0.024829	*B3GALT1*	4	0.30152	0.852764	0.069171
SYNJ1	4	-0.022923	0.944432	0.024829	*MARS1*	4	0.30899	0.852764	0.069171
IKZF3	3	-0.01934	0.944432	0.024829	*FANCM*	3	0.31669	0.852764	0.069171
RAB31	4	-0.002032	0.944432	0.024829	*CCL2*	3	0.31696	0.852764	0.069171
ZNF132	4	0.018034	0.944432	0.024829	*UGT1A8*	1	0.33908	0.852764	0.069171
NCF4	4	0.022042	0.944432	0.024829	*SAP18*	3	0.42487	0.852764	0.069171
DCAF8L2	4	0.033361	0.944432	0.024829	*ISX*	3	-0.26072	0.854028	0.068528
GEMIN8	4	0.035535	0.944432	0.024829	*CRIP1*	4	-0.23988	0.854028	0.068528
DTX1	2	0.036319	0.944432	0.024829	*PRR34-AS1*	1	-0.32458	0.854141	0.06847
FBXL12	4	0.042514	0.944432	0.024829	*SYT7*	2	0.038298	0.854141	0.06847
MAP9	2	0.047852	0.944432	0.024829	*ERC1*	3	0.12928	0.854141	0.06847
PHTF2	4	0.048666	0.944432	0.024829	*ZNF441*	4	0.21284	0.854141	0.06847
CCSER2	4	0.055449	0.944432	0.024829	*SMS*	2	-0.32195	0.854501	0.068287
RDH13	4	0.066178	0.944432	0.024829	*CXCR2*	3	-0.12722	0.854599	0.068238
ZNF358	4	0.069545	0.944432	0.024829	*OR5P2*	3	0.20351	0.854599	0.068238
TBC1D12	4	0.07732	0.944432	0.024829	*EPB41L5*	3	0.32792	0.854599	0.068238

GFPT2	3	0.078962	0.944432	0.024829	*ASPDH*	4	-0.20624	0.854664	0.068205
ZNF326	3	0.10103	0.944432	0.024829	*SLC25A1*	3	-0.34909	0.854827	0.068122
TP63	4	0.1017	0.944432	0.024829	*MFGE8*	1	-0.47596	0.855143	0.067962
C8orf34	4	0.10312	0.944432	0.024829	*ATP6V1E2*	3	-0.45953	0.855143	0.067962
NR4A2	4	0.11528	0.944432	0.024829	*ADGRF4*	2	-0.45025	0.855143	0.067962
TTC23	4	0.1198	0.944432	0.024829	*RPL22L1*	4	-0.35156	0.855143	0.067962
GALNT1	3	0.12026	0.944432	0.024829	*TMEM54*	3	-0.33084	0.855143	0.067962
DNAAF3	4	0.12238	0.944432	0.024829	*GPANK1*	4	-0.31224	0.855143	0.067962
SLC6A20	4	0.12856	0.944432	0.024829	*CYP4F2*	3	-0.29929	0.855143	0.067962
EPOP	3	0.13077	0.944432	0.024829	*TMPO*	4	-0.29663	0.855143	0.067962
NIPSNAP3A	4	0.13272	0.944432	0.024829	*CD3D*	4	-0.28653	0.855143	0.067962
COQ9	4	0.16964	0.944432	0.024829	*CIDEB*	3	-0.27623	0.855143	0.067962
ZC3H8	4	0.17146	0.944432	0.024829	*PAEP*	2	-0.25976	0.855143	0.067962
BLOC1S3	2	0.17394	0.944432	0.024829	*RHOXF2*	1	-0.25125	0.855143	0.067962
C11orf49	4	0.1742	0.944432	0.024829	*C19orf71*	4	-0.24942	0.855143	0.067962
RBFOX2	3	0.1816	0.944432	0.024829	*PLGRKT*	3	-0.23294	0.855143	0.067962
ARFGAP3	4	0.18231	0.944432	0.024829	*P2RY4*	3	-0.23086	0.855143	0.067962
RALGPS2	4	0.18326	0.944432	0.024829	*ATP8B4*	2	-0.22883	0.855143	0.067962
ARHGAP24	4	0.18606	0.944432	0.024829	*ZNF518B*	4	-0.19329	0.855143	0.067962
FAM83B	4	0.19361	0.944432	0.024829	*SPATA25*	4	-0.1828	0.855143	0.067962
SIX4	1	0.19653	0.944432	0.024829	*TNFAIP2*	3	-0.18154	0.855143	0.067962
DARS1	4	0.19661	0.944432	0.024829	*DIRAS3*	4	-0.17964	0.855143	0.067962
ASXL1	4	0.20206	0.944432	0.024829	*CLCN1*	4	-0.13809	0.855143	0.067962
TRIM65	4	0.20446	0.944432	0.024829	*MMD*	3	-0.12369	0.855143	0.067962
CIB1	4	0.20563	0.944432	0.024829	*PPIL1*	4	-0.11937	0.855143	0.067962
TMED1	4	0.20651	0.944432	0.024829	*PYCR1*	3	-0.11182	0.855143	0.067962
IKBIP	4	0.2214	0.944432	0.024829	*FOSL1*	4	-0.10894	0.855143	0.067962
SLC24A1	3	0.22296	0.944432	0.024829	*FBXO32*	4	-0.075448	0.855143	0.067962
NFIA	4	0.22962	0.944432	0.024829	*SMURF1*	4	-0.055927	0.855143	0.067962
CARD6	4	0.23053	0.944432	0.024829	*LRCH3*	4	-0.012038	0.855143	0.067962
MTCL1	4	0.23561	0.944432	0.024829	*ACTL7B*	4	-0.011658	0.855143	0.067962
DMAC2	1	0.24067	0.944432	0.024829	*KIAA1958*	4	-0.011467	0.855143	0.067962
WSCD1	4	0.24091	0.944432	0.024829	*CCDC148*	4	-0.006585	0.855143	0.067962
TNPO3	3	0.24384	0.944432	0.024829	*TMEM74B*	4	0.020609	0.855143	0.067962
SZRD1	2	0.25031	0.944432	0.024829	*SNX5*	4	0.026431	0.855143	0.067962
CMTM2	2	0.2504	0.944432	0.024829	*FOXH1*	4	0.072167	0.855143	0.067962
CCM2	3	0.25997	0.944432	0.024829	*OTOG*	4	0.095167	0.855143	0.067962
ADCY4	4	0.27061	0.944432	0.024829	*TCIRG1*	3	0.09969	0.855143	0.067962
CNOT2	3	0.27342	0.944432	0.024829	*NAGS*	4	0.11186	0.855143	0.067962
CSDE1	3	0.27455	0.944432	0.024829	*TECPR1*	4	0.12019	0.855143	0.067962
TMEM117	4	0.27851	0.944432	0.024829	*DALRD3*	2	0.13725	0.855143	0.067962
TSEN2	4	0.28045	0.944432	0.024829	*AGMAT*	3	0.1418	0.855143	0.067962
RAPGEF2	3	0.28054	0.944432	0.024829	*NECAP1*	4	0.1796	0.855143	0.067962
PSMB11	4	0.29718	0.944432	0.024829	*RAB41*	4	0.19164	0.855143	0.067962
PROSER3	3	0.31783	0.944432	0.024829	*PXYLP1*	3	0.20591	0.855143	0.067962
DCAF1	3	0.32078	0.944432	0.024829	*RRNAD1*	3	0.21178	0.855143	0.067962
MYO7A	3	0.32126	0.944432	0.024829	*SERPINA12*	3	0.21827	0.855143	0.067962
CEACAM5	4	0.32193	0.944432	0.024829	*CBFA2T3*	4	0.21987	0.855143	0.067962
APOBEC3D	2	0.33201	0.944432	0.024829	*KLHDC7A*	3	0.25848	0.855143	0.067962
NMUR1	3	0.34094	0.944432	0.024829	*NUMB*	4	0.27654	0.855143	0.067962
SPATA2L	4	0.36336	0.944432	0.024829	*CELA3A*	2	0.28178	0.855143	0.067962
RPL23A	1	0.3826	0.944432	0.024829	*DPH6*	4	0.287	0.855143	0.067962
DEXI	1	0.42073	0.944432	0.024829	*LAMA5*	3	0.3079	0.855143	0.067962
NGDN	3	0.52882	0.944432	0.024829	*TIAM2*	3	0.32582	0.855143	0.067962
NPTX1	3	-0.44066	0.944629	0.024739	*TPI1*	4	0.3311	0.855143	0.067962
AIFM1	4	0.29524	0.944631	0.024738	*RPS18*	2	0.38455	0.855143	0.067962
MBNL2	1	-0.3889	0.945755	0.024221	*C1orf137*	3	-0.24952	0.855792	0.067632
GPR107	3	-0.38391	0.945974	0.024121	*ARC*	4	-0.018262	0.855968	0.067543

PTCD2	4	-0.26107	0.945974	0.024121	*PDGFD*	4	0.046432	0.855968	0.067543
YEATS4	4	-0.22471	0.945974	0.024121	*PLPPR3*	3	0.063874	0.855968	0.067543
FAM222B	4	-0.12563	0.945974	0.024121	*C2orf27B*	1	0.2846	0.855968	0.067543
ITGB4	3	-0.12349	0.945974	0.024121	*IGFLR1*	3	0.35454	0.855968	0.067543
DNM1	4	-0.007837	0.945974	0.024121	*VTI1A*	4	-0.21175	0.856291	0.067379
ANGPT1	4	0.076102	0.945974	0.024121	*ARMC8*	4	-0.1948	0.856291	0.067379
ZNF333	3	0.13511	0.945974	0.024121	*THEM4*	2	-0.29907	0.856479	0.067283
IGFALS	4	0.15058	0.945974	0.024121	*CDC14A*	4	-0.28706	0.856479	0.067283
CNIH2	3	0.17106	0.945974	0.024121	*CHP1*	4	-0.18243	0.856479	0.067283
DCTN1	4	0.32323	0.945974	0.024121	*ALKBH2*	4	-0.16847	0.856479	0.067283
ASAH2B	1	0.41255	0.945974	0.024121	*PDE4C*	4	0.15762	0.856479	0.067283
SHTN1	3	0.43265	0.945974	0.024121	*MMGT1*	4	0.18227	0.856479	0.067283
LCOR	8	0.066584	0.94656	0.023852	*POGZ*	3	0.25912	0.856479	0.067283
GCNT4	3	-0.13062	0.946615	0.023826	*SOX3*	4	0.28134	0.856479	0.067283
SPCS3	4	0.11398	0.946615	0.023826	*SUMO1*	1	0.28221	0.856479	0.067283
LDB1	4	0.16091	0.946615	0.023826	*GPR158*	3	-0.32038	0.856508	0.067269
ARL13A	3	0.18376	0.946615	0.023826	*HSPA13*	3	-0.14652	0.856508	0.067269
GDPGP1	4	-0.23383	0.946763	0.023759	*ITGA4*	4	-0.10429	0.856508	0.067269
ZC3HAV1L	3	-0.16765	0.947251	0.023535	*PSMB10*	3	0.21376	0.856508	0.067269
CTSW	3	0.12012	0.947497	0.023422	*DNAJC5B*	3	0.29733	0.856508	0.067269
TAF12	3	0.33128	0.947497	0.023422	*FAM217B*	1	-0.34805	0.856548	0.067248
ERCC8	3	-0.13525	0.947552	0.023397	*OXCT2*	1	-0.34549	0.856548	0.067248
CLCA2	4	-0.11779	0.947552	0.023397	*TPSB2*	1	-0.32488	0.856548	0.067248
ZNF624	3	0.15879	0.947552	0.023397	*DUOX2*	4	-0.21837	0.856548	0.067248
TUBB	3	0.43657	0.947552	0.023397	*FGF5*	4	-0.19909	0.856548	0.067248
WSCD2	3	-0.075987	0.947651	0.023352	*STAC*	4	-0.19342	0.856548	0.067248
BAP1	2	-0.2411	0.94809	0.023151	*SOCS7*	4	-0.17138	0.856548	0.067248
ZNF264	4	-0.036091	0.948285	0.023061	*PHLPP1*	4	-0.17103	0.856548	0.067248
SF3B4	3	-0.12274	0.948387	0.023014	*USP50*	4	-0.099652	0.856548	0.067248
SYK	3	0.21376	0.948387	0.023014	*SUGP1*	2	-0.064903	0.856548	0.067248
UNC13D	4	0.21717	0.948387	0.023014	*OSBPL2*	4	-0.058344	0.856548	0.067248
TTC30B	4	-0.22835	0.949026	0.022722	*TAC1*	4	-0.043317	0.856548	0.067248
FGF5	4	-0.14449	0.949026	0.022722	*RITA1*	2	-0.019848	0.856548	0.067248
ORC5	4	-0.019322	0.949026	0.022722	*IL10RB*	4	-0.017338	0.856548	0.067248
FAM155A	3	-0.002865	0.949026	0.022722	*ENOX2*	4	-0.009455	0.856548	0.067248
TIMMDC1	4	0.15312	0.949026	0.022722	*PLEKHA4*	2	0.030963	0.856548	0.067248
OR2B6	2	0.21876	0.949026	0.022722	*SLC4A1*	4	0.05699	0.856548	0.067248
ZNF285	4	-0.056833	0.94903	0.02272	*TULP3*	3	0.062304	0.856548	0.067248
FAM120A	4	0.15626	0.94903	0.02272	*PACRGL*	4	0.068449	0.856548	0.067248
LZTS2	4	0.15939	0.94903	0.02272	*TNNC2*	4	0.075707	0.856548	0.067248
TAAR6	3	-0.28915	0.949084	0.022695	*KIF24*	2	0.1237	0.856548	0.067248
SHANK1	3	-0.080359	0.949084	0.022695	*PAPLN*	4	0.18013	0.856548	0.067248
PPM1G	3	0.13236	0.949084	0.022695	*FAM210A*	4	0.19365	0.856548	0.067248
CCDC32	3	0.32229	0.949084	0.022695	*ZDHHC16*	4	0.20837	0.856548	0.067248
RRP9	3	-0.024246	0.949134	0.022673	*CDO1*	4	0.21624	0.856548	0.067248
FAT1	4	-0.25709	0.949139	0.02267	*MTCH1*	3	0.23638	0.856548	0.067248
RAPSN	4	-0.24089	0.949139	0.02267	*EFTUD2*	4	0.23797	0.856548	0.067248
OTULINL	4	-0.13952	0.949139	0.02267	*USP39*	4	0.25803	0.856548	0.067248
LACTB	2	-0.025363	0.949139	0.02267	*GATD1*	4	0.2663	0.856548	0.067248
PTP4A1	3	-0.52892	0.949786	0.022374	*SP4*	4	0.29829	0.856548	0.067248
NAPRT	1	-0.32864	0.949786	0.022374	*BAP1*	2	0.30355	0.856548	0.067248
PRPH2	3	-0.30172	0.949786	0.022374	*AAGAB*	2	0.30522	0.856548	0.067248
CNGB3	4	-0.19523	0.949786	0.022374	*C11orf98*	1	0.32541	0.856548	0.067248
CHRNB2	3	-0.17198	0.949786	0.022374	*CCDC12*	3	0.52476	0.856548	0.067248
MRPS34	3	-0.018892	0.949786	0.022374	*AAMDC*	3	-0.19862	0.856611	0.067216
TUBG2	3	0.05802	0.949786	0.022374	*MTMR2*	4	-0.35996	0.856675	0.067184
IFT43	3	0.077641	0.949786	0.022374	*MOB3A*	4	-0.22695	0.856932	0.067054
PDE6C	4	0.077902	0.949786	0.022374	*NCF2*	4	-0.15103	0.857059	0.066989

C16orf95	4	0.08434	0.949786	0.022374	*MPG*	4	-0.12192	0.857059	0.066989
TM7SF3	3	0.24483	0.949786	0.022374	*CLDN3*	4	-0.25448	0.857235	0.0669
CFAP20	3	0.26826	0.949786	0.022374	*EYA4*	4	-0.14517	0.857235	0.0669
NFKBIB	2	0.31769	0.949786	0.022374	*GAREM2*	3	-0.557	0.857245	0.066895
CDC42SE1	3	0.38025	0.949786	0.022374	*MAML3*	4	-0.22879	0.857245	0.066895
TMEM71	3	0.24758	0.950077	0.022241	*ZNF816*	4	-0.14954	0.857245	0.066895
SOGA3	1	-0.42528	0.950609	0.021998	*PARK7*	4	-0.046254	0.857245	0.066895
UBXN7	3	0.012751	0.9509	0.021865	*QSOX1*	3	-0.024771	0.857245	0.066895
KCND3	3	-0.50528	0.951053	0.021795	*CLDN8*	4	-0.006233	0.857245	0.066895
EDIL3	3	-0.48256	0.951053	0.021795	*KCNK13*	4	0.15578	0.857245	0.066895
H2AC16	2	-0.45173	0.951053	0.021795	*CETP*	4	-0.16611	0.85755	0.066741
SENP5	4	-0.21713	0.951053	0.021795	*MARCHF11*	4	0.12062	0.85774	0.066644
DEFB4A	2	-0.17632	0.951053	0.021795	*HDGFL2*	3	0.20094	0.85774	0.066644
SLAMF9	4	-0.14909	0.951053	0.021795	*FAM228B*	3	0.22185	0.85774	0.066644
EBF1	3	0.091004	0.951053	0.021795	*SLC25A12*	3	-0.19655	0.858044	0.06649
SMG6	3	0.17046	0.951053	0.021795	*EME2*	3	-0.55986	0.858304	0.066359
KLHL28	3	-0.41945	0.952236	0.021255	*CD101*	3	-0.44026	0.858304	0.066359
VRK2	3	-0.40391	0.952236	0.021255	*CD163*	4	-0.3212	0.858304	0.066359
HOXC9	3	-0.39747	0.952236	0.021255	*KLHL33*	3	-0.30617	0.858304	0.066359
TPM2	4	-0.35613	0.952236	0.021255	*H2BC6*	2	-0.30125	0.858304	0.066359
SNX8	3	-0.34791	0.952236	0.021255	*SSH1*	3	-0.28058	0.858304	0.066359
TAF13	4	-0.22461	0.952236	0.021255	*TMEM50B*	4	-0.25221	0.858304	0.066359
CYP46A1	4	-0.20009	0.952236	0.021255	*EDN2*	3	-0.244	0.858304	0.066359
SNAP29	3	-0.090339	0.952236	0.021255	*FABP5*	2	-0.2356	0.858304	0.066359
COMT	4	-0.068947	0.952236	0.021255	*ZNFX1*	4	-0.23247	0.858304	0.066359
DACT3	4	-0.068839	0.952236	0.021255	*MAPKAPK2*	4	-0.22226	0.858304	0.066359
GPR32	4	-0.065908	0.952236	0.021255	*APOH*	2	-0.19773	0.858304	0.066359
PKM	3	-0.040261	0.952236	0.021255	*THADA*	4	-0.18702	0.858304	0.066359
FOXK1	3	0.005906	0.952236	0.021255	*ZNF250*	4	-0.18532	0.858304	0.066359
TRMT61A	2	0.007893	0.952236	0.021255	*SLC13A3*	4	-0.18071	0.858304	0.066359
BTG3	2	0.037114	0.952236	0.021255	*ZNF619*	3	-0.17935	0.858304	0.066359
GATA3	4	0.078397	0.952236	0.021255	*CXXC5*	4	-0.17782	0.858304	0.066359
POGZ	3	0.083118	0.952236	0.021255	*MARK1*	4	-0.17164	0.858304	0.066359
ZNF331	4	0.12625	0.952236	0.021255	*STIM2*	4	-0.16179	0.858304	0.066359
ADAM15	4	0.14299	0.952236	0.021255	*SULF2*	4	-0.15926	0.858304	0.066359
MALRD1	4	0.15186	0.952236	0.021255	*ARRDC2*	4	-0.14682	0.858304	0.066359
KRBA1	4	0.16704	0.952236	0.021255	*LHB*	4	-0.1397	0.858304	0.066359
ITIH6	4	0.18915	0.952236	0.021255	*CNOT8*	3	-0.11876	0.858304	0.066359
SYNGR4	4	0.19734	0.952236	0.021255	*ARHGAP27*	4	-0.11769	0.858304	0.066359
MFAP1	4	0.22806	0.952236	0.021255	*SNPH*	3	-0.10987	0.858304	0.066359
CARF	3	0.27147	0.952236	0.021255	*ZNF280D*	4	-0.098137	0.858304	0.066359
MGAM2	3	0.30046	0.952236	0.021255	*GPR182*	3	-0.073908	0.858304	0.066359
UBE3D	3	0.36141	0.952236	0.021255	*TSPAN11*	4	-0.015009	0.858304	0.066359
SLC16A10	2	0.46725	0.952236	0.021255	*SASH3*	4	-6.40E-04	0.858304	0.066359
NAA15	4	0.47823	0.952236	0.021255	*RAB11FIP3*	4	0.00147	0.858304	0.066359
RBFOX1	4	-0.35537	0.952437	0.021164	*ASCC2*	4	0.034767	0.858304	0.066359
GNG12	4	-0.34573	0.952437	0.021164	*INPP5E*	4	0.047314	0.858304	0.066359
LPL	4	-0.30408	0.952437	0.021164	*PMPCB*	4	0.052088	0.858304	0.066359
IRAK3	4	-0.28169	0.952437	0.021164	*KRTAP3-1*	4	0.082778	0.858304	0.066359
CCNY	3	-0.18422	0.952437	0.021164	*C17orf98*	2	0.088352	0.858304	0.066359
SEC24B	4	-0.089988	0.952437	0.021164	*CCDC25*	3	0.10761	0.858304	0.066359
FLII	4	-0.072712	0.952437	0.021164	*TDRD1*	4	0.13538	0.858304	0.066359
RTN4R	3	0.041087	0.952437	0.021164	*PCDHGC5*	4	0.14288	0.858304	0.066359
CRIPT	3	0.26869	0.952437	0.021164	*HIGD1B*	3	0.14389	0.858304	0.066359
GTF3C2	2	0.46771	0.952437	0.021164	*GRM1*	4	0.15637	0.858304	0.066359
RILPL2	4	-0.32302	0.952501	0.021135	*HTR2B*	2	0.15839	0.858304	0.066359
TMEM99	4	-0.27397	0.952501	0.021135	*ASL*	3	0.16267	0.858304	0.066359
ARFGEF2	3	-0.17539	0.952501	0.021135	*CYP8B1*	4	0.16829	0.858304	0.066359

FAM78A	4	-0.15669	0.952501	0.021135	*TMEM130*	4	0.1683	0.858304	0.066359
OSM	2	-0.11235	0.952501	0.021135	*KRTCAP3*	3	0.1754	0.858304	0.066359
DCUN1D1	4	-0.032796	0.952501	0.021135	*MEF2D*	4	0.17827	0.858304	0.066359
NEMP1	3	-0.020095	0.952501	0.021135	*POMP*	4	0.18291	0.858304	0.066359
PGAP3	3	0.019399	0.952501	0.021135	*ZNF175*	4	0.1873	0.858304	0.066359
TPCN1	4	0.047707	0.952501	0.021135	*AHCYL2*	4	0.18831	0.858304	0.066359
TMEM204	3	0.19341	0.952501	0.021135	*WFDC5*	3	0.19511	0.858304	0.066359
ALDH16A1	3	0.20787	0.952501	0.021135	*CBX6*	3	0.1992	0.858304	0.066359
ENAH	3	0.2408	0.952501	0.021135	*RBMXL1*	4	0.20469	0.858304	0.066359
ZDHHC24	4	0.25723	0.952501	0.021135	*MFSD6*	4	0.2095	0.858304	0.066359
AQP11	3	0.27321	0.952501	0.021135	*AMN*	3	0.21105	0.858304	0.066359
STYK1	3	0.28027	0.952501	0.021135	*DEPTOR*	4	0.21675	0.858304	0.066359
MRPS24	3	0.29645	0.952501	0.021135	*THAP8*	3	0.24696	0.858304	0.066359
ZNF19	3	0.30794	0.952501	0.021135	*ZNF273*	3	0.2669	0.858304	0.066359
TMEM217	3	0.32221	0.952501	0.021135	*OSM*	2	0.28641	0.858304	0.066359
ANKS3	3	0.32774	0.952501	0.021135	*SPDYA*	3	0.29189	0.858304	0.066359
ARMC4	3	-0.28506	0.952647	0.021068	*CDK2*	4	0.42238	0.858304	0.066359
AP4S1	2	0.064064	0.952647	0.021068	*FCF1*	1	0.42971	0.858304	0.066359
MOCS3	2	0.52042	0.952647	0.021068	*XRCC3*	3	0.43862	0.858304	0.066359
GGCX	3	-0.53915	0.95292	0.020943	*IMMP2L*	4	0.022656	0.858653	0.066182
ASB16	3	-0.51946	0.95292	0.020943	*CSF1R*	3	-0.39395	0.858713	0.066152
UXT	3	-0.46773	0.95292	0.020943	*LEKR1*	3	-0.26418	0.858713	0.066152
APOBEC3A	2	-0.42867	0.95292	0.020943	*ANKRD35*	4	-0.086175	0.858713	0.066152
SLC39A8	2	-0.4015	0.95292	0.020943	*PIEZO2*	4	0.079145	0.858713	0.066152
TNXB	3	-0.38405	0.95292	0.020943	*REXO5*	4	-0.056573	0.8589	0.066057
ADAP1	4	-0.34997	0.95292	0.020943	*NR4A1*	3	-0.039593	0.8589	0.066057
CHN2	4	-0.33076	0.95292	0.020943	*BTG2*	4	0.17672	0.8589	0.066057
TRIM26	3	-0.31601	0.95292	0.020943	*ADAM2*	4	-0.12319	0.858915	0.06605
PROS1	3	-0.31538	0.95292	0.020943	*MSN*	2	-0.047982	0.859088	0.065962
TOMM40L	3	-0.31513	0.95292	0.020943	*ST20*	2	0.070388	0.859088	0.065962
KRTAP17-1	3	-0.31065	0.95292	0.020943	*SLC48A1*	4	-0.20977	0.859341	0.065834
CMTM3	3	-0.27884	0.95292	0.020943	*VEGFB*	3	-0.16289	0.859737	0.065634
P2RX6	4	-0.26501	0.95292	0.020943	*FAM229B*	3	-0.03922	0.859943	0.065531
ROPN1	3	-0.24704	0.95292	0.020943	*ATG3*	4	-0.11267	0.86021	0.065395
IL17RC	2	-0.24578	0.95292	0.020943	*GLCE*	4	0.10618	0.86021	0.065395
ZP2	4	-0.24146	0.95292	0.020943	*NOMO3*	2	-0.30479	0.860305	0.065348
AXIN2	3	-0.23723	0.95292	0.020943	*TMEM60*	3	-0.20413	0.860305	0.065348
MLLT1	3	-0.23488	0.95292	0.020943	*SLC26A11*	4	-0.17755	0.860305	0.065348
FGF1	4	-0.23468	0.95292	0.020943	*CASP9*	4	-0.11684	0.860305	0.065348
ZNF121	3	-0.21055	0.95292	0.020943	*TAS2R19*	3	-0.090954	0.860305	0.065348
VPS37D	2	-0.20895	0.95292	0.020943	*B4GALT4*	4	0.024524	0.860305	0.065348
CLN6	3	-0.19317	0.95292	0.020943	*ZHX1*	4	0.051776	0.860305	0.065348
GFI1	4	-0.19253	0.95292	0.020943	*LYG1*	4	0.076471	0.860305	0.065348
SLC5A11	4	-0.19121	0.95292	0.020943	*PTK6*	2	0.077251	0.860305	0.065348
COMMD1	4	-0.19087	0.95292	0.020943	*IL1B*	4	0.15959	0.860305	0.065348
TMEM183B	3	-0.17946	0.95292	0.020943	*ZNF181*	3	0.2068	0.860305	0.065348
RBM12B	4	-0.17704	0.95292	0.020943	*ORM1*	3	0.21419	0.860305	0.065348
SLC22A7	3	-0.16535	0.95292	0.020943	*NCAPG*	4	0.29667	0.860305	0.065348
EDDM3B	4	-0.16196	0.95292	0.020943	*SCGN*	2	-0.4332	0.860869	0.065063
ID4	4	-0.15595	0.95292	0.020943	*PRSS53*	4	-0.39841	0.860869	0.065063
CCT6B	4	-0.15436	0.95292	0.020943	*LIMD2*	3	-0.37344	0.860869	0.065063
MARVELD1	4	-0.15262	0.95292	0.020943	*ROPN1L*	4	-0.20291	0.860869	0.065063
ZNF143	4	-0.14556	0.95292	0.020943	*PRAC1*	3	-0.11756	0.860869	0.065063
VEGFB	3	-0.14353	0.95292	0.020943	*CMKLR1*	3	-0.077313	0.860869	0.065063
TMEM167B	4	-0.13985	0.95292	0.020943	*ATP5F1B*	3	0.079927	0.860869	0.065063
SSTR1	3	-0.13936	0.95292	0.020943	*SPART*	3	0.17119	0.860869	0.065063
RPS6KA3	4	-0.12682	0.95292	0.020943	*TTLL3*	2	0.19294	0.860869	0.065063
LMF1	3	-0.1186	0.95292	0.020943	*ZNF229*	3	0.21026	0.860869	0.065063

ING4	2	-0.11067	0.95292	0.020943	*KRT10*	4	0.25542	0.860869	0.065063
SCFD2	4	-0.085028	0.95292	0.020943	*VPS13A*	3	0.25589	0.860869	0.065063
COL20A1	2	-0.072474	0.95292	0.020943	*ODF3B*	4	0.26072	0.860869	0.065063
TMC1	4	-0.072101	0.95292	0.020943	*ABCB9*	3	0.27038	0.860869	0.065063
OGN	4	-0.066245	0.95292	0.020943	*BEX5*	2	0.30103	0.860869	0.065063
ABCC12	4	-0.048265	0.95292	0.020943	*C9orf43*	3	0.33443	0.860869	0.065063
TNRC6C	4	-0.044303	0.95292	0.020943	*CERS4*	2	-0.34726	0.861212	0.06489
MSRB3	4	-0.040964	0.95292	0.020943	*CD36*	4	-0.29385	0.861212	0.06489
C9orf72	4	-0.038042	0.95292	0.020943	*CLGN*	2	-0.27207	0.861212	0.06489
TCEA1	2	0.008558	0.95292	0.020943	*PPP1CC*	3	-0.25854	0.861212	0.06489
MFAP5	3	0.009842	0.95292	0.020943	*GNA11*	3	-0.20624	0.861212	0.06489
GET4	3	0.020283	0.95292	0.020943	*PPP2R3A*	3	-0.18705	0.861212	0.06489
RAB13	4	0.033409	0.95292	0.020943	*PCBD2*	4	-0.15694	0.861212	0.06489
ACPP	4	0.037012	0.95292	0.020943	*CHD9*	4	-0.15153	0.861212	0.06489
PRMT6	2	0.065142	0.95292	0.020943	*TMEM273*	4	-0.11979	0.861212	0.06489
S100B	4	0.066221	0.95292	0.020943	*FOXO1*	4	-0.11704	0.861212	0.06489
MOAP1	4	0.068382	0.95292	0.020943	*ADPGK*	3	-0.091015	0.861212	0.06489
CLDN12	4	0.085523	0.95292	0.020943	*BCL2L14*	4	-0.088276	0.861212	0.06489
PARP2	4	0.085885	0.95292	0.020943	*CHST6*	4	-0.081632	0.861212	0.06489
BCL2L14	4	0.086848	0.95292	0.020943	*RASSF1*	4	-0.072665	0.861212	0.06489
OR52I1	2	0.11465	0.95292	0.020943	*SORL1*	3	-0.065078	0.861212	0.06489
SERPINH1	4	0.12034	0.95292	0.020943	*ADGRG7*	4	-0.053239	0.861212	0.06489
GTF3C5	4	0.12384	0.95292	0.020943	*NFKB2*	4	-0.040258	0.861212	0.06489
CLHC1	4	0.12878	0.95292	0.020943	*CPSF6*	4	-0.013253	0.861212	0.06489
CPNE6	2	0.12958	0.95292	0.020943	*PDIA2*	4	-0.00262	0.861212	0.06489
TRAF3IP3	4	0.13037	0.95292	0.020943	*PDZD8*	4	0.009045	0.861212	0.06489
NKX2-2	4	0.13143	0.95292	0.020943	*TBC1D8B*	4	0.038641	0.861212	0.06489
CCNT2	4	0.13316	0.95292	0.020943	*CPAMD8*	4	0.076528	0.861212	0.06489
VWCE	3	0.13539	0.95292	0.020943	*PM20D2*	4	0.076796	0.861212	0.06489
GALNT13	4	0.13909	0.95292	0.020943	*CPO*	4	0.085589	0.861212	0.06489
RGS3	4	0.1395	0.95292	0.020943	*TEAD1*	4	0.11318	0.861212	0.06489
USP2	4	0.16888	0.95292	0.020943	*SCML4*	4	0.15675	0.861212	0.06489
CLIP3	4	0.17247	0.95292	0.020943	*UST*	4	0.17285	0.861212	0.06489
DGKD	4	0.17605	0.95292	0.020943	*ZNF33A*	4	0.17524	0.861212	0.06489
NLRP1	2	0.17666	0.95292	0.020943	*AP3M1*	4	0.18732	0.861212	0.06489
DDX31	3	0.1871	0.95292	0.020943	*RBMX2*	4	0.18738	0.861212	0.06489
NME5	3	0.19074	0.95292	0.020943	*SGO2*	4	0.20633	0.861212	0.06489
NOL3	4	0.20789	0.95292	0.020943	*KRTAP5-1*	3	0.21268	0.861212	0.06489
MPZL2	2	0.20838	0.95292	0.020943	*KPNA1*	3	0.2243	0.861212	0.06489
SFTPA2	3	0.20905	0.95292	0.020943	*CEP95*	4	0.23149	0.861212	0.06489
KCNG4	4	0.21649	0.95292	0.020943	*HABP2*	3	0.26374	0.861212	0.06489
FBLIM1	4	0.22172	0.95292	0.020943	*COPS2*	3	0.34051	0.861212	0.06489
ZNF91	4	0.22975	0.95292	0.020943	*UBA52*	3	0.45777	0.861212	0.06489
EMC2	4	0.23853	0.95292	0.020943	*MPND*	4	-0.37512	0.861468	0.064761
PDXK	4	0.24179	0.95292	0.020943	*BASP1*	2	-0.29484	0.861468	0.064761
VPS53	4	0.25933	0.95292	0.020943	*PRSS45P*	4	-0.1876	0.861468	0.064761
PXN	4	0.26454	0.95292	0.020943	*CTDSP1*	4	-0.18341	0.861468	0.064761
MACROH2A2	3	0.26468	0.95292	0.020943	*FAM71D*	2	-0.15922	0.861468	0.064761
HOXD8	3	0.26557	0.95292	0.020943	*NSDHL*	4	-0.15819	0.861468	0.064761
COMMD9	3	0.26725	0.95292	0.020943	*MX1*	3	0.041443	0.861468	0.064761
FBXO4	2	0.26877	0.95292	0.020943	*RBM14*	2	0.21718	0.861468	0.064761
CCDC107	2	0.27365	0.95292	0.020943	*MED14OS*	4	-0.30509	0.861638	0.064675
LDLRAD4	3	0.28091	0.95292	0.020943	*S100PBP*	4	-0.049919	0.861638	0.064675
RPS23	3	0.28702	0.95292	0.020943	*BEND3*	3	0.23074	0.861794	0.064597
BCL9L	3	0.29671	0.95292	0.020943	*HLA-DQB1*	3	-0.44089	0.861796	0.064596
PSG9	3	0.30409	0.95292	0.020943	*TAB1*	2	-0.39034	0.861796	0.064596
FUNDC2	3	0.30595	0.95292	0.020943	*SOX18*	3	-0.36755	0.861796	0.064596
DEFB125	2	0.31094	0.95292	0.020943	*GIPC3*	4	-0.29348	0.861796	0.064596

UBE2Z	3	0.31212	0.95292	0.020943	*G2E3*	3	-0.27601	0.861796	0.064596
GOPC	3	0.32188	0.95292	0.020943	*USB1*	4	-0.22924	0.861796	0.064596
SPTLC2	1	0.33112	0.95292	0.020943	*TRIM22*	3	-0.13414	0.861796	0.064596
ZSCAN31	1	0.34407	0.95292	0.020943	*TTLL5*	3	-0.10785	0.861796	0.064596
TXN	3	0.34572	0.95292	0.020943	*PIANP*	3	-0.071326	0.861796	0.064596
PRXL2C	3	0.36598	0.95292	0.020943	*ALG13*	2	-0.070722	0.861796	0.064596
CCNL1	3	0.3711	0.95292	0.020943	*LDLRAD3*	3	-0.033771	0.861796	0.064596
PRKAA2	3	0.38443	0.95292	0.020943	*CLEC2D*	4	-0.002831	0.861796	0.064596
EIF2B1	3	0.47659	0.95292	0.020943	*ADAM10*	3	8.16E-04	0.861796	0.064596
NOMO1	1	-0.38369	0.953067	0.020876	*MCRIP1*	4	0.064996	0.861796	0.064596
ARRB1	2	-0.04545	0.953067	0.020876	*TREM1*	2	0.0705	0.861796	0.064596
TMBIM1	4	-0.016504	0.953067	0.020876	*ARNTL*	4	0.085403	0.861796	0.064596
ARFGEF3	4	0.15807	0.953067	0.020876	*ACADS*	4	0.15262	0.861796	0.064596
MAST3	4	0.20131	0.953067	0.020876	*RPSA*	2	0.18427	0.861796	0.064596
BAX	4	0.22892	0.953067	0.020876	*PCMTD1*	4	0.20103	0.861796	0.064596
DCAF12L2	4	0.30959	0.953067	0.020876	*ITGA1*	3	0.23334	0.861796	0.064596
PPEF2	3	0.32768	0.953067	0.020876	*ARHGEF26*	3	0.24276	0.861796	0.064596
ASB2	3	0.096388	0.953068	0.020876	*FOXJ2*	1	0.26094	0.861796	0.064596
CCDC138	4	-0.13331	0.953351	0.020747	*GSG1*	3	0.28419	0.861796	0.064596
SLC46A1	3	-0.49621	0.953715	0.020582	*TMEM147*	3	0.29096	0.861796	0.064596
MFHAS1	3	-0.41515	0.953715	0.020582	*TFDP2*	3	0.29961	0.861796	0.064596
SUB1	3	-0.38896	0.953715	0.020582	*KLHL5*	3	0.3224	0.861796	0.064596
KLHL15	3	-0.36795	0.953715	0.020582	*IDI1*	3	0.34352	0.861796	0.064596
GPAT4	4	-0.32607	0.953715	0.020582	*TMEM167A*	1	0.36876	0.861796	0.064596
NSD3	3	-0.31369	0.953715	0.020582	*BRF2*	3	0.37738	0.861796	0.064596
VPS54	3	-0.29706	0.953715	0.020582	*RPL11*	3	0.72044	0.861796	0.064596
NUDCD1	3	-0.26627	0.953715	0.020582	*SELENOT*	3	-0.49813	0.861848	0.064569
CNIH3	4	-0.25518	0.953715	0.020582	*GNMT*	2	-0.35371	0.861848	0.064569
MPHOSPH8	3	-0.25029	0.953715	0.020582	*HP*	3	-0.010149	0.861848	0.064569
NBPF11	2	-0.24908	0.953715	0.020582	*REV1*	3	0.018076	0.861848	0.064569
CHST1	3	-0.24764	0.953715	0.020582	*RGPD3*	2	0.11523	0.861848	0.064569
CEP97	3	-0.23715	0.953715	0.020582	*FBP2*	2	0.13914	0.861848	0.064569
SENP1	4	-0.22596	0.953715	0.020582	*ARMC9*	3	0.21023	0.861848	0.064569
GPR183	4	-0.22386	0.953715	0.020582	*SPINDOC*	2	-0.38001	0.862788	0.064096
UGT1A3	4	-0.20864	0.953715	0.020582	*H2BC1*	3	-0.37861	0.862788	0.064096
DNAJB4	4	-0.19515	0.953715	0.020582	*SBSPON*	1	-0.32136	0.862788	0.064096
ENPP1	4	-0.19491	0.953715	0.020582	*RAB37*	3	-0.26151	0.862788	0.064096
ADORA2B	2	-0.19488	0.953715	0.020582	*STOX1*	4	-0.25413	0.862788	0.064096
SPP1	4	-0.19075	0.953715	0.020582	*PGLS*	3	-0.21666	0.862788	0.064096
BOP1	4	-0.14081	0.953715	0.020582	*MORF4L1*	3	-0.1881	0.862788	0.064096
DYDC2	4	-0.11171	0.953715	0.020582	*TMSB15B*	4	-0.1303	0.862788	0.064096
ANTKMT	3	-0.098867	0.953715	0.020582	*CLIC3*	4	-0.11378	0.862788	0.064096
SH3BGRL	3	-0.097481	0.953715	0.020582	*CLEC11A*	4	-0.099489	0.862788	0.064096
TRIM66	4	-0.096525	0.953715	0.020582	*KCTD2*	4	-0.085341	0.862788	0.064096
TTF2	3	-0.091075	0.953715	0.020582	*TOGARAM2*	4	-0.061058	0.862788	0.064096
SFXN2	3	-0.076941	0.953715	0.020582	*KLF10*	4	-0.020556	0.862788	0.064096
GMCL1	3	-0.073107	0.953715	0.020582	*SAR1B*	2	-0.005898	0.862788	0.064096
TBC1D17	4	-0.054082	0.953715	0.020582	*SUMF1*	3	-0.003643	0.862788	0.064096
PAIP2B	3	-0.035259	0.953715	0.020582	*SERGEF*	4	0.074926	0.862788	0.064096
FNDC8	4	-0.034642	0.953715	0.020582	*MYOZ2*	2	0.13572	0.862788	0.064096
GLTPD2	4	0.020822	0.953715	0.020582	*GCSAM*	3	0.15091	0.862788	0.064096
H1-3	3	0.031036	0.953715	0.020582	*VASN*	2	0.1693	0.862788	0.064096
SLC4A8	4	0.041216	0.953715	0.020582	*DOHH*	4	0.17766	0.862788	0.064096
SLC25A10	4	0.14499	0.953715	0.020582	*CEP170B*	4	0.17827	0.862788	0.064096
OR1L8	4	0.16021	0.953715	0.020582	*PHLPP2*	4	0.18135	0.862788	0.064096
OTX1	4	0.16595	0.953715	0.020582	*ZNF2*	3	0.18203	0.862788	0.064096
CD320	4	0.1662	0.953715	0.020582	*LARP1*	4	0.20505	0.862788	0.064096
GCDH	4	0.16969	0.953715	0.020582	*CEP63*	3	0.25996	0.862788	0.064096

DGCR6L	4	0.17448	0.953715	0.020582	*MAPRE1*	2	0.29518	0.862788	0.064096
KCTD15	4	0.19986	0.953715	0.020582	*IREB2*	4	0.34866	0.862788	0.064096
ANKRD36	2	0.20296	0.953715	0.020582	*RANBP17*	4	-0.26622	0.86311	0.063934
FBXL2	3	0.21814	0.953715	0.020582	*TUBB6*	4	-0.26522	0.86311	0.063934
ITGA2	4	0.21853	0.953715	0.020582	*ZBTB21*	4	0.070483	0.86311	0.063934
PER1	3	0.22575	0.953715	0.020582	*TMEM178A*	3	-0.50452	0.863843	0.063565
IL17RB	3	0.23098	0.953715	0.020582	*PAN2*	4	-0.34968	0.863843	0.063565
PRSS1	3	0.24061	0.953715	0.020582	*TMC4*	2	-0.34578	0.863843	0.063565
CAPN15	4	0.25125	0.953715	0.020582	*HTT*	4	-0.33976	0.863843	0.063565
PRDX3	2	0.26832	0.953715	0.020582	*HIRIP3*	3	-0.30806	0.863843	0.063565
NIN	4	0.27404	0.953715	0.020582	*ASB14*	3	-0.28597	0.863843	0.063565
NLRP12	4	0.28294	0.953715	0.020582	*COL4A1*	4	-0.2684	0.863843	0.063565
PEX13	4	0.29812	0.953715	0.020582	*TGM2*	4	-0.25759	0.863843	0.063565
GBP1	3	0.31953	0.953715	0.020582	*MICA*	2	-0.24771	0.863843	0.063565
HERC2	4	0.36086	0.953715	0.020582	*ADO*	4	-0.23735	0.863843	0.063565
FIGN	3	-0.38895	0.953817	0.020535	*DLG3*	4	-0.23663	0.863843	0.063565
GPR160	4	-0.36986	0.953817	0.020535	*LHFPL6*	4	-0.23533	0.863843	0.063565
EPGN	3	-0.31121	0.953817	0.020535	*ITGB3BP*	4	-0.19873	0.863843	0.063565
MED12L	4	-0.13294	0.953817	0.020535	*BHLHE40*	4	-0.1912	0.863843	0.063565
DLX2	3	-0.008611	0.953817	0.020535	*NAF1*	3	-0.18508	0.863843	0.063565
PSPH	4	-0.001775	0.953817	0.020535	*USP54*	4	-0.14093	0.863843	0.063565
ARRDC3	4	0.091458	0.953817	0.020535	*FLYWCH1*	4	-0.13903	0.863843	0.063565
SEC61A1	4	0.14707	0.953817	0.020535	*DYNLT3*	4	-0.1381	0.863843	0.063565
DNTTIP1	4	0.17444	0.953817	0.020535	*NBEA*	4	-0.10944	0.863843	0.063565
MEIS2	4	0.18211	0.953817	0.020535	*CIPC*	3	-0.091472	0.863843	0.063565
HEPACAM	4	0.19191	0.953817	0.020535	*TNS4*	4	-0.090086	0.863843	0.063565
GDPD5	4	0.21857	0.953817	0.020535	*RCOR1*	4	-0.085076	0.863843	0.063565
MYL4	4	0.21932	0.953817	0.020535	*NXPH3*	4	-0.074945	0.863843	0.063565
MAPK8IP1	3	0.25315	0.953817	0.020535	*MMP10*	4	-0.058584	0.863843	0.063565
TECR	3	0.34682	0.953817	0.020535	*RNFT2*	4	-0.057135	0.863843	0.063565
MMP25	1	-0.46404	0.954296	0.020317	*KRT86*	2	-0.04184	0.863843	0.063565
RPL7	2	-0.26562	0.954296	0.020317	*PARVG*	4	-0.002318	0.863843	0.063565
FHL1	4	-0.21475	0.954296	0.020317	*TUBE1*	4	0.006923	0.863843	0.063565
SPOCD1	4	-0.19333	0.954296	0.020317	*BATF2*	4	0.023206	0.863843	0.063565
IRS1	4	-0.16376	0.954296	0.020317	*CAMP*	4	0.027132	0.863843	0.063565
NDUFAF7	4	-0.15876	0.954296	0.020317	*CZIB*	3	0.030174	0.863843	0.063565
PSAP	4	-0.13036	0.954296	0.020317	*MYOCD*	4	0.035576	0.863843	0.063565
ARHGAP42	4	-0.086685	0.954296	0.020317	*BNIP5*	4	0.037397	0.863843	0.063565
MAPK7	3	-0.077957	0.954296	0.020317	*ADIPOR2*	4	0.056559	0.863843	0.063565
SSR3	4	-0.045397	0.954296	0.020317	*SLC4A3*	4	0.067478	0.863843	0.063565
ZNF256	4	0.007168	0.954296	0.020317	*KCNK2*	4	0.071508	0.863843	0.063565
FOXO1	4	0.053693	0.954296	0.020317	*OXCT1*	4	0.095472	0.863843	0.063565
VNN3	4	0.11039	0.954296	0.020317	*ITPKA*	2	0.10223	0.863843	0.063565
MRM1	4	0.12391	0.954296	0.020317	*TPP1*	4	0.11325	0.863843	0.063565
ISCU	4	0.13754	0.954296	0.020317	*SCPEP1*	4	0.11674	0.863843	0.063565
ANAPC5	4	0.18511	0.954296	0.020317	*SRC*	2	0.12179	0.863843	0.063565
VPS37A	4	0.18641	0.954296	0.020317	*LMCD1*	4	0.13097	0.863843	0.063565
ADGRA3	3	0.19048	0.954296	0.020317	*CXXC1*	4	0.1429	0.863843	0.063565
NEBL	4	0.19526	0.954296	0.020317	*INAFM2*	4	0.15937	0.863843	0.063565
FAM3A	4	0.19752	0.954296	0.020317	*PAQR4*	4	0.16257	0.863843	0.063565
IKZF2	2	0.213	0.954296	0.020317	*CDCA8*	4	0.18865	0.863843	0.063565
MAB21L1	4	0.23456	0.954296	0.020317	*MTSS2*	4	0.18912	0.863843	0.063565
PRDX6	4	0.27427	0.954296	0.020317	*ELAVL1*	4	0.19211	0.863843	0.063565
MOBP	4	0.28336	0.954296	0.020317	*LYRM4*	3	0.20361	0.863843	0.063565
ATP6AP1	4	0.45685	0.954296	0.020317	*POU2AF1*	4	0.22828	0.863843	0.063565
LINC00452	4	0.11523	0.954389	0.020275	*SHF*	3	0.2313	0.863843	0.063565
PRKAR1A	3	-0.31664	0.954762	0.020105	*CD27*	3	0.2729	0.863843	0.063565
STX7	3	-0.21435	0.954762	0.020105	*KATNA1*	3	0.27358	0.863843	0.063565

SCGB3A2	4	-0.028634	0.954762	0.020105	*TSPAN12*	4	0.27833	0.863843	0.063565
S100G	4	-0.020856	0.954762	0.020105	*NAB1*	3	0.29701	0.863843	0.063565
GTF3C6	4	-0.36879	0.954949	0.02002	*SUPT4H1*	2	0.29928	0.863843	0.063565
DLG3	4	-0.34098	0.954949	0.02002	*LTBP1*	4	0.30183	0.863843	0.063565
KLHL41	2	-0.21207	0.954949	0.02002	*DDX59*	3	0.31489	0.863843	0.063565
COL5A3	4	-0.16019	0.954949	0.02002	*BAALC*	2	0.31762	0.863843	0.063565
CHCHD6	4	-0.13348	0.954949	0.02002	*PIK3CA*	4	0.33849	0.863843	0.063565
ALDOB	2	0.3068	0.954949	0.02002	*SBNO1*	4	-0.45352	0.86386	0.063556
ISG20L2	4	-0.47384	0.954966	0.020012	*IL2RB*	2	-0.35267	0.86386	0.063556
IQCH	3	-0.46298	0.954966	0.020012	*LRRC71*	3	-0.33066	0.86386	0.063556
RPRM	1	-0.45153	0.954966	0.020012	*TLN2*	4	-0.33021	0.86386	0.063556
MNS1	3	-0.3551	0.954966	0.020012	*B3GNT8*	3	-0.28741	0.86386	0.063556
GGNBP2	4	-0.34069	0.954966	0.020012	*HEATR5A*	3	-0.27113	0.86386	0.063556
HUWE1	3	-0.32576	0.954966	0.020012	*SYNPO*	4	-0.24583	0.86386	0.063556
THUMPD1	4	-0.27856	0.954966	0.020012	*CD55*	4	-0.23992	0.86386	0.063556
PDZK1IP1	4	-0.25284	0.954966	0.020012	*SLC39A7*	4	-0.22995	0.86386	0.063556
F5	3	-0.24057	0.954966	0.020012	*DENND4A*	4	-0.22336	0.86386	0.063556
CTRB1	1	-0.22556	0.954966	0.020012	*THAP6*	4	-0.21392	0.86386	0.063556
CCDC160	4	-0.21151	0.954966	0.020012	*CCDC168*	4	-0.1862	0.86386	0.063556
SH3BP5	4	-0.20346	0.954966	0.020012	*IL10RA*	4	-0.14089	0.86386	0.063556
PDAP1	2	-0.19491	0.954966	0.020012	*ABRACL*	4	-0.13413	0.86386	0.063556
GRIN2C	2	-0.19173	0.954966	0.020012	*C20orf144*	4	-0.080949	0.86386	0.063556
STX12	4	-0.16952	0.954966	0.020012	*DCAF4L1*	3	-0.079692	0.86386	0.063556
CHRNA5	4	-0.14186	0.954966	0.020012	*AVIL*	4	-0.043837	0.86386	0.063556
CYB5A	4	-0.04649	0.954966	0.020012	*GSTO2*	4	-0.018664	0.86386	0.063556
GJC2	4	-0.04533	0.954966	0.020012	*MAPT*	4	-0.01657	0.86386	0.063556
TOR3A	4	0.016634	0.954966	0.020012	*PTPN4*	4	-0.002232	0.86386	0.063556
HSDL1	3	0.023645	0.954966	0.020012	*SLC35B4*	4	0.059616	0.86386	0.063556
LRRC3C	4	0.02699	0.954966	0.020012	*COX7B2*	4	0.099441	0.86386	0.063556
FBXL21P	2	0.0426	0.954966	0.020012	*EPS8L2*	4	0.10101	0.86386	0.063556
CCDC78	3	0.048904	0.954966	0.020012	*ALPG*	4	0.17268	0.86386	0.063556
IHH	4	0.096659	0.954966	0.020012	*ADAT2*	4	0.30341	0.86386	0.063556
CYP4X1	4	0.10308	0.954966	0.020012	*NR2F6*	2	0.040381	0.865348	0.062809
PLEKHM3	4	0.13552	0.954966	0.020012	*TTC27*	4	-0.59782	0.865751	0.062607
GPR52	4	0.15056	0.954966	0.020012	*OTOGL*	4	-0.21624	0.865751	0.062607
TP53BP2	4	0.1533	0.954966	0.020012	*PRSS37*	4	-0.19421	0.865751	0.062607
TTC34	2	0.19042	0.954966	0.020012	*JAK2*	4	0.034863	0.865751	0.062607
PPP4R1	4	0.1975	0.954966	0.020012	*RETREG3*	4	0.10888	0.865751	0.062607
CEP131	4	0.2153	0.954966	0.020012	*C9orf57*	2	0.13918	0.865751	0.062607
CDC73	3	0.22799	0.954966	0.020012	*GABARAP*	2	-0.3457	0.866034	0.062465
ZMAT5	4	0.23375	0.954966	0.020012	*ADGRL3*	3	-0.10872	0.866034	0.062465
PAPOLG	4	0.23645	0.954966	0.020012	*LMAN1L*	4	-0.10768	0.866034	0.062465
POT1	4	0.25497	0.954966	0.020012	*ABCB4*	3	0.17696	0.866034	0.062465
PARM1	3	0.36601	0.954966	0.020012	*INO80D*	4	0.27413	0.866093	0.062435
EIF2B2	4	0.37197	0.954966	0.020012	*FAM160B1*	4	-0.074581	0.866199	0.062382
EEF1D	3	-0.33408	0.955064	0.019968	*KMT5A*	2	0.45297	0.866396	0.062284
MTMR8	4	-0.28831	0.955064	0.019968	*CTNNAL1*	3	-0.46222	0.866528	0.062218
MTAP	4	-0.25359	0.955064	0.019968	*NKX2-4*	1	-0.41701	0.866528	0.062218
C17orf82	3	-0.22947	0.955064	0.019968	*TMPRSS11F*	3	-0.062189	0.866528	0.062218
TMEM200C	4	-0.22889	0.955064	0.019968	*GLRA1*	3	-0.007395	0.866528	0.062218
ADGRF2	4	-0.16057	0.955064	0.019968	*AGA*	4	0.013385	0.866528	0.062218
PROK1	4	-0.15529	0.955064	0.019968	*ATAD2B*	4	0.086406	0.866528	0.062218
TLR5	4	0.004081	0.955064	0.019968	*PHEX*	4	0.090242	0.866528	0.062218
COPRS	4	0.12418	0.955064	0.019968	*ANKRD23*	4	0.13353	0.866528	0.062218
TEF	1	0.25835	0.955064	0.019968	*NCAPH2*	2	0.15671	0.866528	0.062218
ISG15	3	0.25851	0.955064	0.019968	*STRN4*	3	0.2413	0.866528	0.062218
PLK4	4	0.33972	0.955064	0.019968	*ZNF721*	4	-0.1746	0.866738	0.062112
SCLY	1	-0.61094	0.955619	0.019715	*ABTB1*	2	-0.00845	0.866738	0.062112

OTOF	1	-0.48356	0.955619	0.019715	*LNX2*	2	-0.43457	0.866856	0.062053
ZBTB18	4	-0.26946	0.955619	0.019715	*APOBEC3A*	2	-0.37023	0.866856	0.062053
SLC2A9	3	-0.25986	0.955619	0.019715	*IFNA8*	3	-0.35641	0.866856	0.062053
YPEL2	4	-0.23495	0.955619	0.019715	*DGCR2*	3	-0.31136	0.866856	0.062053
ZNF443	4	-0.009813	0.955619	0.019715	*KDM3B*	1	-0.30861	0.866856	0.062053
SNX33	3	0.07974	0.955619	0.019715	*TRAF6*	3	-0.28682	0.866856	0.062053
EFNA3	4	0.15618	0.955619	0.019715	*AFAP1L2*	3	-0.28642	0.866856	0.062053
KATNAL1	4	0.17158	0.955619	0.019715	*SLC17A9*	4	-0.28088	0.866856	0.062053
GSAP	4	0.17472	0.955619	0.019715	*TARBP1*	2	-0.24566	0.866856	0.062053
VWDE	3	0.23554	0.955619	0.019715	*ANGPTL1*	2	-0.22271	0.866856	0.062053
OGT	4	-0.060498	0.955894	0.01959	*GJB6*	4	-0.21688	0.866856	0.062053
TWF1	1	-0.54005	0.955897	0.019589	*EPB41L1*	4	-0.20752	0.866856	0.062053
SLC12A2	2	-0.38741	0.955897	0.019589	*CCDC97*	3	-0.18165	0.866856	0.062053
CPB2	4	-0.21775	0.955897	0.019589	*S100Z*	4	-0.14207	0.866856	0.062053
ZFP62	4	-0.12373	0.955897	0.019589	*RHOBTB1*	4	-0.14119	0.866856	0.062053
TFR2	4	-0.10202	0.955897	0.019589	*KDELR1*	4	-0.13771	0.866856	0.062053
ZMYM4	4	-0.097373	0.955897	0.019589	*NRBF2*	2	-0.13369	0.866856	0.062053
DDX43	4	-0.031458	0.955897	0.019589	*CRHR2*	4	-0.12688	0.866856	0.062053
PRLHR	4	0.19925	0.955897	0.019589	*LRRC70*	4	-0.124	0.866856	0.062053
MIER1	4	0.22349	0.955897	0.019589	*STK17B*	3	-0.076531	0.866856	0.062053
VEGFC	4	-0.19432	0.955898	0.019588	*FGGY*	4	-0.049911	0.866856	0.062053
C6orf89	4	-0.072045	0.955898	0.019588	*C2orf80*	2	-0.046476	0.866856	0.062053
CHRNA2	2	0.21074	0.955898	0.019588	*SEMA5B*	3	-0.04401	0.866856	0.062053
DIRAS2	3	-0.34506	0.956311	0.019401	*TPO*	4	-0.003321	0.866856	0.062053
METTL27	3	-0.16058	0.956311	0.019401	*TMEM97*	4	0.003187	0.866856	0.062053
COL22A1	3	0.18701	0.956311	0.019401	*ORC2*	4	0.010181	0.866856	0.062053
ACO1	2	-0.27343	0.956402	0.019359	*CUTC*	4	0.052742	0.866856	0.062053
EPHB4	4	-0.083128	0.956402	0.019359	*ZNF135*	4	0.057872	0.866856	0.062053
ATP2A2	4	-0.4981	0.957	0.019088	*FA2H*	4	0.068512	0.866856	0.062053
PIGM	4	-0.36955	0.957	0.019088	*CHD3*	4	0.079388	0.866856	0.062053
METRN	3	-0.33927	0.957	0.019088	*SFT2D2*	4	0.083418	0.866856	0.062053
OR2AT4	3	-0.24824	0.957	0.019088	*STX6*	3	0.13951	0.866856	0.062053
VIPR1	2	-0.19072	0.957	0.019088	*INTS5*	4	0.16081	0.866856	0.062053
VAV2	4	-0.17434	0.957	0.019088	*SKIV2L*	4	0.22223	0.866856	0.062053
TKTL2	3	-0.098427	0.957	0.019088	*SMOX*	4	0.38919	0.866856	0.062053
SPOPL	3	-0.086787	0.957	0.019088	*ZNF526*	4	0.41098	0.866856	0.062053
ZFP41	4	0.034334	0.957	0.019088	*SDE2*	2	0.4325	0.866856	0.062053
ZNF257	4	0.09373	0.957	0.019088	*ANXA4*	4	-0.19571	0.867554	0.061704
ACBD5	4	0.14434	0.957	0.019088	*PCDHA8*	2	-0.005883	0.867638	0.061661
ANKRD11	4	0.14977	0.957	0.019088	*CGN*	4	0.050583	0.867638	0.061661
CCER2	4	0.17093	0.957	0.019088	*TUBB2A*	2	0.16146	0.867638	0.061661
FOSL1	4	0.26678	0.957	0.019088	*DDAH1*	2	-0.31611	0.867964	0.061498
TMPRSS11A	3	0.27645	0.957	0.019088	*DDX17*	4	-0.062398	0.867964	0.061498
OSGIN2	3	0.27875	0.957	0.019088	*PCDH7*	3	0.055221	0.867964	0.061498
MICA	2	-0.3755	0.957046	0.019067	*OTOP3*	3	0.21479	0.867964	0.061498
PAQR5	2	-0.26406	0.957046	0.019067	*ADRA2A*	3	-0.23816	0.868054	0.061453
ZNF705E	2	-0.23661	0.957046	0.019067	*GNAZ*	3	-0.1186	0.868054	0.061453
HSD17B6	3	0.20146	0.95732	0.018943	*AKR7A2*	2	0.030712	0.868054	0.061453
ABHD8	3	0.21409	0.95732	0.018943	*STAG1*	3	0.099859	0.868054	0.061453
TMEM88B	3	0.27938	0.95732	0.018943	*HOXD8*	3	0.13758	0.868054	0.061453
ZNF114	3	0.37419	0.95732	0.018943	*NEPRO*	3	0.24238	0.868054	0.061453
TCAP	3	-0.023318	0.95732	0.018943	*GRHL2*	2	0.3297	0.868054	0.061453
GRIN3B	3	-0.2939	0.957776	0.018736	*EEA1*	4	-0.20093	0.868358	0.061301
SEC14L6	4	-0.29147	0.957776	0.018736	*BLM*	3	-0.12201	0.868358	0.061301
SLC17A8	2	-0.26334	0.957776	0.018736	*LIMA1*	3	-0.087337	0.868358	0.061301
MN1	4	-0.24022	0.957776	0.018736	*PRPSAP1*	4	0.023854	0.868358	0.061301
CTR9	4	-0.18463	0.957776	0.018736	*TUBB4A*	4	0.087722	0.868358	0.061301
CD164	4	-0.16301	0.957776	0.018736	*DDX55*	3	0.13541	0.868358	0.061301

ETS1	2	-0.14035	0.957776	0.018736	*P2RX2*	4	-0.32919	0.868657	0.061152
ZNF507	4	0.057341	0.957776	0.018736	*CEBPA*	4	-0.096271	0.868657	0.061152
SEC22C	4	0.21265	0.957776	0.018736	*ICAM5*	3	0.038627	0.868865	0.061048
GBP2	3	0.23745	0.957776	0.018736	*PARP8*	4	0.051384	0.868865	0.061048
CXorf58	4	0.25125	0.958049	0.018612	*SEM1*	8	0.20763	0.869449	0.060756
FAM81B	3	-0.26143	0.958049	0.018612	*NCBP3*	4	0.21476	0.869449	0.060756
KLHL7	3	-0.26004	0.958049	0.018612	*CIAO3*	4	0.28324	0.869449	0.060756
PHF24	3	-0.25195	0.958049	0.018612	*DDX1*	3	0.12186	0.870371	0.060296
UVSSA	3	-0.24903	0.958049	0.018612	*GNLY*	4	-0.034698	0.870429	0.060267
MAP1-GIMA	2	-0.22442	0.958049	0.018612	*AAAS*	2	-0.47528	0.870433	0.060265
ROPN1B	3	-0.14946	0.958049	0.018612	*HS3ST3A1*	3	-0.26047	0.870433	0.060265
JPH3	2	-0.094074	0.958049	0.018612	*PUM1*	4	-0.13093	0.870433	0.060265
OIT3	3	-0.007718	0.958049	0.018612	*PLCE1*	4	0.14267	0.870433	0.060265
COX5B	3	0.11539	0.958049	0.018612	*DGKD*	4	-0.16618	0.871082	0.059941
ZEB1	4	0.11676	0.958049	0.018612	*RHOT1*	3	0.02617	0.871086	0.059939
LRCH3	4	0.16046	0.958049	0.018612	*CNN3*	3	0.070625	0.871086	0.059939
SPX	4	0.22453	0.958049	0.018612	*CBWD6*	2	0.13317	0.871086	0.059939
SIRT2	4	0.23847	0.958049	0.018612	*MALL*	3	0.26196	0.871086	0.059939
SYNDIG1L	3	0.2687	0.958049	0.018612	*NR1H2*	2	-0.46396	0.871416	0.059774
PGRMC2	2	0.31663	0.958049	0.018612	*RNF216*	4	0.1127	0.871565	0.0597
RFC1	3	0.37865	0.958049	0.018612	*ZNF646*	4	-0.22799	0.871738	0.059614
GATC	3	0.38333	0.958049	0.018612	*RABEP1*	4	-0.19275	0.871738	0.059614
NCOR2	3	-0.48996	0.958366	0.018468	*DDX19B*	4	0.07516	0.871738	0.059614
MATN4	2	-0.44553	0.958366	0.018468	*HOXC5*	4	-0.06267	0.872113	0.059427
TSC1	3	-0.31036	0.958366	0.018468	*PKD2*	2	0.13031	0.872896	0.059037
ARTN	3	-0.27406	0.958366	0.018468	*STAU1*	4	0.1051	0.873283	0.058845
ZC3H12D	3	-0.19399	0.958366	0.018468	*SEC31A*	4	0.16653	0.873283	0.058845
TEX264	3	-0.13649	0.958366	0.018468	*IFI44*	4	-0.2013	0.873625	0.058675
AGRN	4	0.001006	0.958366	0.018468	*ZNF429*	4	-0.072881	0.873625	0.058675
CCDC6	3	0.052471	0.958366	0.018468	*CACNG8*	2	0.2257	0.874	0.058489
WNT8B	4	0.11418	0.958366	0.018468	*ARL4A*	1	-0.52436	0.874353	0.058313
PRPS1	3	0.12862	0.958366	0.018468	*PRR4*	1	-0.43546	0.874353	0.058313
DNAJB9	2	0.16091	0.958366	0.018468	*FAM98A*	3	-0.020696	0.874353	0.058313
FAM53B	4	0.19808	0.958366	0.018468	*GUCY1A1*	3	-0.32617	0.874526	0.058227
SENP7	3	0.27143	0.958366	0.018468	*A2M*	3	-0.093536	0.874526	0.058227
ZNF10	3	0.28637	0.958366	0.018468	*GCHFR*	2	0.08227	0.874526	0.058227
CDK10	3	0.30696	0.958366	0.018468	*C3orf22*	4	0.084503	0.874526	0.058227
RNF13	3	0.35475	0.958366	0.018468	*KIF4A*	3	0.13695	0.874526	0.058227
STK35	3	-0.27689	0.958955	0.018202	*ESRRB*	4	0.14511	0.874526	0.058227
FGF11	4	-0.096322	0.958955	0.018202	*PTGES2*	4	0.42594	0.874526	0.058227
BRD8	3	-0.38956	0.95972	0.017856	*PTP4A2*	2	0.21498	0.875172	0.057907
OC10192809	2	-0.35626	0.95972	0.017856	*VDAC1*	3	0.033141	0.875229	0.057878
GRM6	3	-0.31204	0.95972	0.017856	*STXBP6*	4	-0.39934	0.875552	0.057718
CTC1	4	-0.27619	0.95972	0.017856	*SHBG*	4	-0.26808	0.875552	0.057718
TMC2	2	-0.24903	0.95972	0.017856	*TCP11L2*	4	-0.23617	0.875552	0.057718
LGALS3	4	-0.22641	0.95972	0.017856	*PLPP6*	4	-0.20763	0.875552	0.057718
FTHL18	3	-0.20987	0.95972	0.017856	*NUDT19*	3	-0.12388	0.875552	0.057718
POLR3D	3	-0.17742	0.95972	0.017856	*TMEM241*	4	-0.08874	0.875552	0.057718
PARD3B	4	-0.11276	0.95972	0.017856	*ABHD12*	4	-0.018739	0.875552	0.057718
SUSD1	4	-0.052729	0.95972	0.017856	*STX12*	4	0.019772	0.875552	0.057718
NPL	2	-0.037705	0.95972	0.017856	*NDUFA13*	3	0.041007	0.875552	0.057718
NTAN1	4	-0.033348	0.95972	0.017856	*CAND2*	3	0.094361	0.875552	0.057718
ARHGAP25	4	-0.031563	0.95972	0.017856	*KCNK18*	4	0.1572	0.875552	0.057718
MICU2	3	-0.019391	0.95972	0.017856	*FANCE*	4	0.19362	0.875552	0.057718
HARBI1	4	0.00139	0.95972	0.017856	*TMEM248*	4	0.19868	0.875552	0.057718
CKS2	3	0.011003	0.95972	0.017856	*C6orf52*	3	0.20191	0.875552	0.057718
BAIAP2L1	4	0.012271	0.95972	0.017856	*HUS1B*	3	0.29625	0.875552	0.057718
KLHL42	3	0.087593	0.95972	0.017856	*XCL1*	1	0.40272	0.875552	0.057718

ADGRG7	4	0.12222	0.95972	0.017856	*HIF1AN*	4	0.11488	0.87588	0.057556
SLC16A6	4	0.20257	0.95972	0.017856	*TRMT6*	3	0.16814	0.875903	0.057544
GPR153	4	0.22927	0.95972	0.017856	*KLF2*	3	0.18289	0.875903	0.057544
AICDA	3	0.25715	0.95972	0.017856	*VSIG1*	4	0.046972	0.876005	0.057494
TMSB4X	1	-0.49462	0.959807	0.017816	*ARMH4*	4	-0.27608	0.876334	0.05733
SQLE	2	-0.39896	0.959807	0.017816	*RIMBP2*	4	-0.2513	0.876334	0.05733
CASC1	4	-0.003298	0.959807	0.017816	*TBC1D29P*	3	-0.076015	0.876334	0.05733
NOD2	4	0.014061	0.959807	0.017816	*NDC1*	4	-0.009761	0.876334	0.05733
ITGB1	4	0.053537	0.959807	0.017816	*CDA*	4	0.028805	0.876334	0.05733
NLRX1	2	0.10779	0.959807	0.017816	*NXF1*	4	0.12858	0.876334	0.05733
PRRC2A	4	0.161	0.959807	0.017816	*EXOSC6*	4	0.19351	0.876334	0.05733
CDC123	3	0.17159	0.959807	0.017816	*ARHGAP11B*	1	0.34578	0.876334	0.05733
PROCR	4	0.23074	0.959807	0.017816	*ADGRG6*	4	0.346	0.876334	0.05733
OAS1	3	0.27239	0.959807	0.017816	*NUP37*	3	-0.011406	0.876571	0.057213
CHMP4B	4	0.32942	0.959807	0.017816	*ITGB7*	4	-0.09413	0.876988	0.057006
FEM1C	4	-0.18531	0.960122	0.017673	*SHANK3*	4	-0.30831	0.877158	0.056922
ERN1	3	-0.40393	0.960162	0.017656	*CEP112*	3	-0.28469	0.877158	0.056922
IL24	3	-0.34249	0.960162	0.017656	*ZNF880*	3	0.16561	0.877158	0.056922
LOXL4	4	-0.21986	0.960162	0.017656	*TRIM36*	4	-0.096584	0.877316	0.056844
PBK	4	-0.21195	0.960162	0.017656	*CFAP73*	4	-0.034508	0.877316	0.056844
ZNFX1	4	-0.18358	0.960162	0.017656	*PCK1*	4	0.068324	0.877316	0.056844
PAQR6	4	-0.1593	0.960162	0.017656	*TLR5*	4	0.14679	0.877316	0.056844
KCNC4	4	-0.15351	0.960162	0.017656	*SET*	1	0.29457	0.877316	0.056844
TRPM4	4	-0.13539	0.960162	0.017656	*IGFBP4*	2	0.31973	0.877316	0.056844
SOBP	4	-0.080786	0.960162	0.017656	*CORO1C*	4	-0.165	0.877699	0.056654
TMEM63A	4	-0.063019	0.960162	0.017656	*FAM47B*	4	-0.005844	0.877699	0.056654
GRK4	4	-0.051533	0.960162	0.017656	*HPS3*	4	-0.26938	0.877958	0.056526
MGAT5B	3	-0.001218	0.960162	0.017656	*KCNQ3*	4	-0.18638	0.877958	0.056526
EPHX3	4	0.089093	0.960162	0.017656	*SPHK1*	3	-0.1797	0.877958	0.056526
SNORC	4	0.091498	0.960162	0.017656	*H4C6*	4	-0.15006	0.877958	0.056526
SLC16A9	4	0.18779	0.960162	0.017656	*HHLA1*	4	0.060814	0.877958	0.056526
CENPI	3	0.27054	0.960162	0.017656	*UBALD2*	4	0.10581	0.877958	0.056526
EFCAB9	4	0.27272	0.960162	0.017656	*MICOS13*	4	0.17381	0.877958	0.056526
FAM83A	4	0.28308	0.960162	0.017656	*SLC25A20*	3	-0.19495	0.878037	0.056487
THAP10	2	0.29988	0.960162	0.017656	*CETN3*	4	-0.065972	0.878037	0.056487
PNO1	2	-0.65848	0.96043	0.017534	*PTGER2*	4	0.11897	0.878037	0.056487
NT5C3A	3	-0.34773	0.96043	0.017534	*AQP9*	3	-0.093127	0.878475	0.056271
SLC16A2	3	-0.21566	0.96043	0.017534	*AUP1*	3	0.032092	0.878475	0.056271
SPAG5	3	0.30865	0.96043	0.017534	*PAF1*	3	0.26281	0.878475	0.056271
CYB561	4	-0.3133	0.960519	0.017494	*RNASET2*	4	-0.30576	0.878542	0.056238
SMIM29	4	0.043894	0.960519	0.017494	*RTKN2*	4	-0.15616	0.878542	0.056238
MCM10	4	0.13654	0.960519	0.017494	*CDCA7L*	3	-0.21641	0.879518	0.055755
MYCN	1	0.21143	0.960564	0.017474	*FZD5*	4	0.21555	0.879518	0.055755
H4C2	3	-0.40241	0.96065	0.017435	*CALHM3*	1	0.34668	0.879518	0.055755
BCHE	3	-0.31431	0.96065	0.017435	*CYB5R1*	3	-0.13694	0.879551	0.055739
SYT1	3	-0.28798	0.96065	0.017435	*RFX4*	4	0.096174	0.879551	0.055739
ITCH	3	-0.24553	0.96065	0.017435	*TIE1*	2	0.27375	0.879551	0.055739
COMMD10	3	-0.22613	0.96065	0.017435	*MED23*	4	-0.26379	0.879584	0.055723
INPP4A	4	-0.16853	0.96065	0.017435	*OGA*	4	-0.1631	0.879584	0.055723
RAB33A	4	-0.14385	0.96065	0.017435	*C1orf52*	4	-0.027742	0.879584	0.055723
ANKRD16	3	-0.014425	0.96065	0.017435	*PLCD1*	1	0.15954	0.879651	0.05569
EMSY	2	0.16261	0.96065	0.017435	*MTTP*	3	0.244	0.879651	0.05569
ELOVL7	2	-0.36487	0.960781	0.017375	*CRBN*	4	-0.14681	0.880222	0.055408
CD81	4	-0.32234	0.960781	0.017375	*C3orf33*	4	-0.12943	0.880222	0.055408
AKAP12	2	-0.31386	0.960781	0.017375	*BDKRB2*	4	-0.11292	0.880222	0.055408
OC10013035	1	-0.30819	0.960781	0.017375	*FOXC2*	2	-0.42362	0.880423	0.055309
PLPP2	4	-0.27944	0.960781	0.017375	*BAZ2B*	4	-0.090412	0.880423	0.055309
BTD	3	-0.08554	0.960781	0.017375	*PEMT*	3	-0.51703	0.880743	0.055151

PDK4	2	-0.043622	0.960781	0.017375	*SERPINI1*	2	-0.30717	0.880743	0.055151
C19orf73	4	-0.013313	0.960781	0.017375	*RAPGEF5*	4	-0.27177	0.880743	0.055151
MYT1	4	0.13809	0.960781	0.017375	*NGEF*	4	-0.20211	0.880743	0.055151
NPHP4	4	-0.28565	0.961094	0.017234	*TBC1D17*	4	-0.18848	0.880743	0.055151
PPOX	4	-0.037386	0.961138	0.017214	*PIGQ*	2	-0.15588	0.880743	0.055151
GOLGA6B	1	0.29713	0.961138	0.017214	*MYPOP*	4	-0.14934	0.880743	0.055151
VPS72	3	0.33693	0.961138	0.017214	*PGRMC1*	4	-0.12456	0.880743	0.055151
RAB27A	4	-0.25518	0.961405	0.017093	*MOSPD1*	4	-0.004056	0.880743	0.055151
POM121C	2	-0.23462	0.961405	0.017093	*TRIM46*	4	0.17994	0.880743	0.055151
SYT12	2	0.30256	0.962568	0.016569	*PRR11*	4	0.20573	0.880743	0.055151
DMBT1	4	-0.23244	0.963147	0.016307	*ESYT2*	3	0.22119	0.880743	0.055151
ZNF672	4	-0.15219	0.963147	0.016307	*SNAPC4*	3	0.24742	0.880743	0.055151
SYNGR1	4	0.044247	0.963147	0.016307	*MCF2L*	2	-0.33234	0.880977	0.055035
TAOK3	3	-0.26328	0.963278	0.016249	*ARHGAP30*	4	-0.18202	0.881151	0.054949
JPT1	3	0.021003	0.963278	0.016249	*AVPR2*	4	-0.12837	0.881151	0.054949
EIF3F	3	0.22371	0.963278	0.016249	*PDE4D*	3	-0.12067	0.881151	0.054949
SREK1IP1	1	0.24793	0.963278	0.016249	*PLCD3*	3	-0.07513	0.881151	0.054949
YBX2	3	0.25897	0.963278	0.016249	*CRB1*	4	-0.053055	0.881151	0.054949
CCDC157	1	-0.48798	0.963543	0.016129	*EVI2A*	4	-0.033232	0.881151	0.054949
PHKG2	4	-0.2613	0.963543	0.016129	*CHAD*	3	0.037461	0.881151	0.054949
TCF7L2	4	-0.065052	0.963543	0.016129	*ATF4*	2	0.17856	0.881151	0.054949
ZNF570	4	0.1021	0.963543	0.016129	*LHX6*	4	0.18377	0.881151	0.054949
ZNF12	4	-0.22025	0.96381	0.016009	*TPM4*	4	0.18443	0.881151	0.054949
VCX	1	-0.36893	0.964267	0.015803	*DGCR8*	4	0.23047	0.881151	0.054949
TIGD1	3	-0.32075	0.964267	0.015803	*TRIM27*	2	0.33648	0.881151	0.054949
SLC31A2	3	-0.29914	0.964267	0.015803	*CFAP206*	1	-0.3466	0.881184	0.054934
TAGLN2	4	-0.29567	0.964267	0.015803	*RALA*	4	0.091547	0.881184	0.054934
GNB2	4	-0.29178	0.964267	0.015803	*DXO*	4	0.42663	0.881184	0.054934
MUL1	4	-0.27972	0.964267	0.015803	*PTDSS2*	4	-0.3372	0.881193	0.054929
PIP4P2	3	-0.27723	0.964267	0.015803	*GRAMD2B*	3	-0.24639	0.881193	0.054929
PET100	3	-0.253	0.964267	0.015803	*POLH*	4	-0.18026	0.881193	0.054929
LRP6	4	-0.25177	0.964267	0.015803	*VPS26C*	4	-0.019707	0.881193	0.054929
CACNA1E	4	-0.24689	0.964267	0.015803	*PASK*	4	0.09246	0.881193	0.054929
ZC3H7B	4	-0.23574	0.964267	0.015803	*RYK*	4	0.058524	0.881303	0.054875
SNRNP27	4	-0.20052	0.964267	0.015803	*DGKE*	4	0.077029	0.881303	0.054875
ANKRD33	3	-0.19632	0.964267	0.015803	*RASA4B*	1	0.21095	0.881537	0.054759
MRPS23	4	-0.18528	0.964267	0.015803	*HDAC3*	4	-0.56237	0.881684	0.054687
COQ8A	3	-0.17344	0.964267	0.015803	*APMAP*	4	-0.22759	0.881684	0.054687
KCNJ11	4	-0.15106	0.964267	0.015803	*SURF2*	4	-0.1631	0.881684	0.054687
ZNF92	3	-0.11438	0.964267	0.015803	*RGN*	4	-0.14501	0.881684	0.054687
AGL	4	-0.089527	0.964267	0.015803	*CHIC1*	4	-0.14127	0.881684	0.054687
RABL3	4	-0.068736	0.964267	0.015803	*RASSF4*	4	-0.11451	0.881684	0.054687
ENTPD1	4	-0.062136	0.964267	0.015803	*ACVR2A*	4	-0.031812	0.881684	0.054687
UBR1	4	-0.047737	0.964267	0.015803	*NLRP6*	4	0.072128	0.881684	0.054687
SCEL	2	-0.022011	0.964267	0.015803	*HAX1*	4	0.089857	0.881684	0.054687
CAPN5	3	-0.007026	0.964267	0.015803	*HPCAL4*	4	0.096816	0.881684	0.054687
SMTNL1	4	-0.006645	0.964267	0.015803	*LRCH2*	4	0.11606	0.881684	0.054687
MXD1	4	-0.002147	0.964267	0.015803	*PIP4P1*	4	0.13275	0.881684	0.054687
STAP1	3	0.052311	0.964267	0.015803	*PEX1*	3	0.15112	0.881684	0.054687
BBS10	4	0.17447	0.964267	0.015803	*BCAT2*	4	0.16686	0.881684	0.054687
LIN37	4	0.17617	0.964267	0.015803	*HM13*	3	0.18694	0.881684	0.054687
CLDND1	4	0.18052	0.964267	0.015803	*BOD1L1*	4	0.19765	0.881684	0.054687
UGCG	4	0.18374	0.964267	0.015803	*COMMD7*	4	0.26244	0.881684	0.054687
CYBA	4	0.18461	0.964267	0.015803	*C8orf37*	2	0.29833	0.881684	0.054687
C19orf25	3	0.19441	0.964267	0.015803	*RPS29*	2	0.43152	0.881784	0.054638
TMED8	4	0.20533	0.964267	0.015803	*USP44*	4	-0.17963	0.881883	0.054589
C2orf49	4	0.27757	0.964267	0.015803	*GNG11*	3	-0.061672	0.882038	0.054513
RSPH14	4	0.30431	0.964267	0.015803	*SSR1*	3	0.13937	0.882038	0.054513

C2orf68	3	0.33015	0.964267	0.015803	*KEAP1*	4	-0.18605	0.882469	0.0543
TRUB2	1	0.35172	0.964267	0.015803	*ZNF444*	4	-0.13337	0.882469	0.0543
PEX16	3	0.36157	0.964267	0.015803	*IQSEC2*	4	-0.077137	0.882469	0.0543
HELQ	3	0.48476	0.964267	0.015803	*TXK*	3	-0.33661	0.882476	0.054297
KIAA0895L	2	-0.38126	0.964428	0.01573	*RAD17*	3	-0.28543	0.882476	0.054297
TPBG	2	-0.33558	0.964428	0.01573	*IFNGR1*	3	-0.27436	0.882476	0.054297
ARL4D	3	-0.25371	0.964428	0.01573	*UCHL5*	3	-0.26274	0.882476	0.054297
DDAH1	2	-0.24257	0.964428	0.01573	*TTLL4*	3	-0.24745	0.882476	0.054297
NRBF2	2	-0.17344	0.964428	0.01573	*HLA-A*	3	-0.22575	0.882476	0.054297
RNF10	2	-0.14416	0.964428	0.01573	*POU4F1*	4	-0.20249	0.882476	0.054297
PTPRU	2	-0.1163	0.964428	0.01573	*CENPP*	2	-0.19529	0.882476	0.054297
DNAJC19	2	-0.076758	0.964428	0.01573	*EFNB3*	4	-0.13169	0.882476	0.054297
ZHX3	4	-0.019651	0.964428	0.01573	*SERP1*	4	-0.093968	0.882476	0.054297
RBM26	2	-0.016236	0.964428	0.01573	*STKLD1*	4	-0.091234	0.882476	0.054297
NISCH	4	0.042775	0.964428	0.01573	*PRRC2A*	4	-0.086072	0.882476	0.054297
NLRP10	4	0.1208	0.964428	0.01573	*FABP3*	4	-0.071316	0.882476	0.054297
MFSD8	4	0.12746	0.964428	0.01573	*ATG4B*	4	-0.065979	0.882476	0.054297
PCYT2	4	0.13568	0.964428	0.01573	*CHODL*	4	9.94E-04	0.882476	0.054297
ATXN7L2	1	0.1784	0.964428	0.01573	*ZNF280A*	4	0.014684	0.882476	0.054297
MATN1	3	0.20066	0.964428	0.01573	*NLGN2*	4	0.034657	0.882476	0.054297
CNN3	3	0.2475	0.964428	0.01573	*LOC10192937*	3	0.075612	0.882476	0.054297
MT1M	2	0.29454	0.964428	0.01573	*LUM*	4	0.10749	0.882476	0.054297
KIF26B	3	0.31388	0.964428	0.01573	*ATP6V1C1*	3	0.2062	0.882476	0.054297
EIF3B	3	0.39227	0.964428	0.01573	*WDR73*	4	0.20678	0.882476	0.054297
SEC16A	3	-0.20185	0.964647	0.015632	*DDR2*	4	0.21794	0.882476	0.054297
AIG1	4	0.25252	0.964647	0.015632	*ACKR3*	4	0.22908	0.882476	0.054297
FAM184A	4	-0.18656	0.964867	0.015533	*CHRNE*	4	0.24357	0.882476	0.054297
GABPB2	4	0.23221	0.964867	0.015533	*ANAPC4*	4	0.33376	0.882476	0.054297
NOMO3	2	-0.17987	0.965172	0.015395	*TRNT1*	2	0.39598	0.882476	0.054297
UBL7	4	-0.1543	0.965172	0.015395	*RSL24D1*	2	0.42178	0.882929	0.054074
POLE3	4	-0.093998	0.965172	0.015395	*ZDHHC5*	4	-0.14635	0.883028	0.054025
RCAN3	4	0.26196	0.965172	0.015395	*BST2*	3	-0.21034	0.883039	0.05402
MEFV	3	-0.39566	0.965299	0.015338	*ZNF19*	3	-0.3112	0.883166	0.053958
INSL5	2	-0.28511	0.965299	0.015338	*UTRN*	3	-0.10181	0.883166	0.053958
RING1	3	0.057766	0.965299	0.015338	*TLCD2*	2	-0.033517	0.883166	0.053958
SLC9A9	2	0.073566	0.965299	0.015338	*PITPNM2*	3	-0.026588	0.883166	0.053958
SLC35F3	3	0.21618	0.965299	0.015338	*POLR2C*	3	0.016595	0.883166	0.053958
SART3	3	0.36626	0.965299	0.015338	*SLC12A5*	4	0.052002	0.883166	0.053958
BFAR	4	-0.14338	0.965329	0.015324	*CCM2L*	3	0.12254	0.883166	0.053958
FLYWCH1	4	-0.13477	0.965329	0.015324	*DNM3*	3	0.16104	0.883166	0.053958
SLC6A8	2	-0.046642	0.965329	0.015324	*ALDOC*	4	0.17108	0.883574	0.053757
RAD23B	4	-0.024591	0.965329	0.015324	*SEC23IP*	3	-0.37378	0.883599	0.053745
ZNF385C	4	0.037076	0.965329	0.015324	*TEX264*	3	-0.32319	0.883599	0.053745
SMAD3	4	0.092995	0.965329	0.015324	*CCNL1*	3	-0.27871	0.883599	0.053745
ACSS1	3	0.13414	0.965329	0.015324	*GALNT1*	3	-0.24553	0.883599	0.053745
CCDC169	4	0.14254	0.965329	0.015324	*C6orf132*	3	-0.23156	0.883599	0.053745
FRMD6	3	0.16638	0.965329	0.015324	*PCDHB6*	4	-0.092447	0.883599	0.053745
DIPK2B	4	0.16752	0.965329	0.015324	*RSRC1*	2	-0.09096	0.883599	0.053745
SEC23B	3	0.20919	0.965329	0.015324	*KCNE3*	3	-0.081575	0.883599	0.053745
TRIM61	3	0.23102	0.965329	0.015324	*NR3C2*	3	0.033495	0.883599	0.053745
CALCOCO2	2	0.26194	0.965329	0.015324	*HSPA12B*	4	0.21344	0.883599	0.053745
ID2	3	0.30546	0.965329	0.015324	*LTBP3*	2	0.32442	0.883599	0.053745
CRYBB2	2	-0.10773	0.966078	0.014988	*NCK1*	2	-0.26955	0.883786	0.053653
GTF2F2	2	-0.50305	0.966132	0.014963	*HSPB6*	2	-0.29021	0.884173	0.053463
ZNF764	2	-0.41445	0.966132	0.014963	*CKM*	4	-0.28082	0.884173	0.053463
BCLAF1	2	-0.37437	0.966132	0.014963	*CLDND1*	4	-0.21174	0.884173	0.053463
TRMT9B	1	-0.29931	0.966132	0.014963	*SPRR3*	4	-0.14509	0.884173	0.053463
SLC25A22	2	-0.27209	0.966132	0.014963	*RIN1*	4	-0.081381	0.884173	0.053463

EIF2AK3	4	-0.22403	0.966132	0.014963	*ZSWIM6*	2	-0.030189	0.884173	0.053463
MYPN	4	-0.1695	0.966132	0.014963	*ADAMTS3*	3	-0.029522	0.884173	0.053463
SNN	2	-0.16928	0.966132	0.014963	*STAM2*	4	0.040556	0.884173	0.053463
DNAAF2	2	-0.13499	0.966132	0.014963	*SPEGNB*	4	0.057657	0.884173	0.053463
SNRPD2	3	-0.068157	0.966132	0.014963	*IER3IP1*	4	0.12902	0.884173	0.053463
GMPR	4	-0.053152	0.966132	0.014963	*SLC16A7*	4	0.14041	0.884173	0.053463
ANKRD33B	4	-0.008343	0.966132	0.014963	*H3-3B*	3	0.25052	0.884173	0.053463
P2RX2	4	0.026426	0.966132	0.014963	*NRG2*	1	-0.60946	0.884247	0.053426
USP30	4	0.049098	0.966132	0.014963	*DNHD1*	2	-0.15048	0.884247	0.053426
EMILIN3	4	0.078818	0.966132	0.014963	*KIF14*	4	0.10621	0.884247	0.053426
ANO6	4	0.12587	0.966132	0.014963	*PHETA1*	3	-0.01458	0.884478	0.053313
SCRT1	4	0.14579	0.966132	0.014963	*SH3RF2*	3	-0.26941	0.88506	0.053027
RAPH1	3	0.15102	0.966132	0.014963	*FNDC1*	4	-0.11236	0.885466	0.052828
PRR5	4	0.1549	0.966132	0.014963	*SMC2*	2	0.47211	0.885476	0.052823
SYT11	4	0.16161	0.966132	0.014963	*TBC1D32*	4	-0.20443	0.886287	0.052426
EIF1AY	2	0.16398	0.966132	0.014963	*PMS2*	4	-0.10474	0.886287	0.052426
PLEKHH3	4	0.17962	0.966132	0.014963	*PRR12*	4	-0.16177	0.886439	0.052351
GTF2H3	4	0.20035	0.966132	0.014963	*HAT1*	4	-0.054154	0.886439	0.052351
HTR3A	4	0.22054	0.966132	0.014963	*KLRF1*	3	-0.13529	0.886459	0.052342
HLA-DQA1	3	0.23813	0.966132	0.014963	*PAX8*	3	0.28441	0.886459	0.052342
TTC29	3	0.2391	0.966132	0.014963	*SMAD3*	4	-0.20503	0.886464	0.052339
SFRP1	4	0.27423	0.966132	0.014963	*PRDM15*	2	-0.12683	0.886464	0.052339
CTRC	2	0.28547	0.966132	0.014963	*FAM117A*	4	-0.046383	0.886464	0.052339
CDCP1	3	0.30345	0.966132	0.014963	*ATG9B*	4	0.12142	0.886464	0.052339
MORC2	2	0.31964	0.966132	0.014963	*LRGUK*	4	0.19468	0.886464	0.052339
VCP	4	0.46074	0.966132	0.014963	*SMPD4*	4	-0.24969	0.886718	0.052214
DNASE1L1	4	-0.30899	0.966161	0.014951	*ZMYM2*	4	-0.24244	0.886718	0.052214
RNF39	1	-0.29898	0.966161	0.014951	*PIN4*	2	-0.17211	0.886718	0.052214
FAM174C	3	-0.24035	0.966161	0.014951	*GJC1*	4	-0.048537	0.886718	0.052214
CLOCK	4	-0.21381	0.966161	0.014951	*PAPOLA*	4	0.002097	0.886718	0.052214
APOL3	4	-0.20955	0.966161	0.014951	*KBTBD4*	4	0.081729	0.886718	0.052214
C19orf71	4	-0.11995	0.966161	0.014951	*ZNF839*	4	0.14585	0.886718	0.052214
USP44	4	-0.11029	0.966161	0.014951	*PPP1R12A*	4	0.19473	0.886718	0.052214
MATN3	4	-0.084955	0.966161	0.014951	*ACOT2*	4	0.1419	0.886869	0.05214
MARK4	4	-0.013803	0.966161	0.014951	*ACTN4*	4	0.18012	0.886869	0.05214
CD99	4	0.064506	0.966161	0.014951	*SYF2*	3	-0.39755	0.887142	0.052007
ST3GAL5	4	0.12924	0.966161	0.014951	*SF3B2*	4	0.2511	0.887152	0.052002
ZFYVE28	4	0.15465	0.966161	0.014951	*ZNF90*	3	-0.3992	0.887298	0.05193
IPP	3	0.17771	0.966161	0.014951	*CLDN2*	3	-0.24903	0.887298	0.05193
AK8	3	0.3663	0.966161	0.014951	*MOB3B*	3	-0.22228	0.887298	0.05193
OR2L2	3	0.34501	0.966466	0.014813	*VSIR*	4	-0.16289	0.887298	0.05193
LEPROT	3	-0.38244	0.966597	0.014755	*ALKBH1*	3	-0.13691	0.887298	0.05193
ARID3A	3	-0.6039	0.966845	0.014643	*AUNIP*	3	0.1087	0.887298	0.05193
FCHO2	3	-0.27448	0.966845	0.014643	*PDE4A*	4	-0.2957	0.887526	0.051819
ELOA	4	-0.23675	0.966845	0.014643	*PRSS46P*	4	-0.29349	0.887526	0.051819
C10orf53	4	-0.16029	0.966845	0.014643	*DUSP12*	4	-0.20269	0.887526	0.051819
HIBCH	4	-0.14543	0.966845	0.014643	*SCML1*	4	-0.20206	0.887526	0.051819
CLMN	4	-0.14171	0.966845	0.014643	*TYMS*	4	-0.1847	0.887526	0.051819
INPP5J	4	-0.12621	0.966845	0.014643	*SPARCL1*	4	-0.095278	0.887526	0.051819
CFAP69	3	-0.11259	0.966845	0.014643	*HERC4*	4	-0.06982	0.887526	0.051819
SLX4IP	4	-0.021478	0.966845	0.014643	*EXOSC5*	4	0.06691	0.887526	0.051819
OCA2	4	-0.006161	0.966845	0.014643	*DCAF8L2*	4	0.0685	0.887526	0.051819
HEPN1	4	0.11074	0.966845	0.014643	*AIPL1*	3	0.14058	0.887526	0.051819
RRM2B	2	0.21265	0.966845	0.014643	*RAB42*	2	-0.29305	0.887861	0.051655
PGAP1	4	-0.081218	0.96715	0.014506	*PFKP*	3	-0.28285	0.887861	0.051655
MAP1LC3A	3	-0.43726	0.967435	0.014378	*PRPF4*	2	-0.19335	0.887861	0.051655
CCDC174	3	-0.33551	0.967435	0.014378	*KCTD6*	3	-0.16698	0.887861	0.051655
TCN2	1	-0.33287	0.967435	0.014378	*CDC14B*	3	-0.074269	0.887861	0.051655

GOLM1	3	-0.29181	0.967435	0.014378	*FZD7*	2	0.02552	0.887861	0.051655
GRK6	4	-0.19947	0.967435	0.014378	*ISM2*	3	0.067935	0.887861	0.051655
SRRM3	3	-0.17589	0.967435	0.014378	*WDR81*	3	0.085385	0.887861	0.051655
RNASE6	4	-0.14354	0.967435	0.014378	*GSDMD*	4	0.10051	0.887861	0.051655
ZNF787	4	0.033871	0.967435	0.014378	*SOAT2*	4	0.1781	0.887861	0.051655
SYTL5	3	0.080769	0.967435	0.014378	*FOSB*	3	0.21038	0.887861	0.051655
NUDT18	4	0.14032	0.967435	0.014378	*INHBC*	2	0.24306	0.887861	0.051655
NAF1	3	0.2438	0.967435	0.014378	*ICAM1*	4	0.17289	0.889006	0.051095
TCIRG1	3	0.24802	0.967435	0.014378	*GIT2*	3	-0.23679	0.889169	0.051016
NPAS2	3	0.25807	0.967435	0.014378	*HOXC12*	4	-0.20822	0.889169	0.051016
SLC23A1	3	0.27454	0.967435	0.014378	*RNF169*	4	0.009901	0.889169	0.051016
CLIP2	3	0.30915	0.967435	0.014378	*ZYG11A*	4	0.059764	0.889169	0.051016
FKBP9	3	0.35945	0.967435	0.014378	*SIRT1*	2	0.11527	0.889169	0.051016
MRPS2	3	0.36423	0.967435	0.014378	*TTLL9*	4	0.13442	0.889169	0.051016
TMEM214	4	-0.064964	0.967514	0.014343	*RHEB*	2	0.17517	0.889169	0.051016
ZBED2	3	-0.034237	0.967514	0.014343	*HGS*	4	0.23486	0.889169	0.051016
C9orf50	3	0.14051	0.967514	0.014343	*YES1*	4	-0.33313	0.889717	0.050748
STX19	3	0.18156	0.967514	0.014343	*MSH3*	4	-0.31256	0.889717	0.050748
SCNN1B	3	0.22336	0.967514	0.014343	*TBPL1*	4	-0.29684	0.889717	0.050748
SYT15	1	0.31564	0.967514	0.014343	*PPP3CA*	4	-0.27879	0.889717	0.050748
PLAAT1	2	0.042791	0.967557	0.014324	*UBA6*	3	-0.26713	0.889717	0.050748
SLC14A2	4	0.18243	0.967685	0.014266	*IL2RG*	4	-0.24873	0.889717	0.050748
MTX2	4	0.18714	0.967685	0.014266	*ZNF580*	2	-0.24574	0.889717	0.050748
PLAG1	4	-0.16706	0.968461	0.013918	*TTC33*	3	-0.24553	0.889717	0.050748
LMO2	4	-0.13864	0.968461	0.013918	*L1CAM*	2	-0.23106	0.889717	0.050748
PYCR3	4	-0.13646	0.968461	0.013918	*CRNN*	4	-0.22855	0.889717	0.050748
ATP6V0E2	2	-0.075857	0.968461	0.013918	*PIK3C2A*	3	-0.22653	0.889717	0.050748
PNMA2	4	-0.070495	0.968461	0.013918	*H2BC7*	3	-0.22176	0.889717	0.050748
USP9X	2	0.47608	0.968461	0.013918	*HMGCS2*	4	-0.2166	0.889717	0.050748
HOXA2	4	-0.29589	0.968535	0.013885	*MAT1A*	4	-0.21442	0.889717	0.050748
DIO1	4	-0.18662	0.968535	0.013885	*ZSCAN18*	4	-0.21109	0.889717	0.050748
RHPN2	4	-0.060667	0.968535	0.013885	*TVP23A*	4	-0.2038	0.889717	0.050748
STAC3	4	0.063071	0.968535	0.013885	*FEZ1*	4	-0.20207	0.889717	0.050748
AHSA2P	4	0.19232	0.968535	0.013885	*ZIC1*	3	-0.18392	0.889717	0.050748
DNASE1	4	0.20224	0.968535	0.013885	*DMRT1*	4	-0.17386	0.889717	0.050748
AMOTL1	3	0.23138	0.968535	0.013885	*FBRSL1*	4	-0.15965	0.889717	0.050748
PLEKHS1	3	0.25454	0.968535	0.013885	*RASAL1*	4	-0.15791	0.889717	0.050748
RPL32	1	0.35265	0.968535	0.013885	*TAS2R4*	4	-0.15168	0.889717	0.050748
BIN1	4	0.032829	0.968651	0.013833	*LMO2*	4	-0.15132	0.889717	0.050748
FKBP1B	4	0.051644	0.968651	0.013833	*P4HA2*	4	-0.12183	0.889717	0.050748
SETD1A	2	0.13718	0.968651	0.013833	*TMCC1*	4	-0.11963	0.889717	0.050748
ASB4	4	0.15318	0.968651	0.013833	*PSG5*	2	-0.11829	0.889717	0.050748
CFAP410	2	0.1894	0.968651	0.013833	*RETSAT*	4	-0.1071	0.889717	0.050748
NBEAL2	3	0.22272	0.968651	0.013833	*SORT1*	4	-0.10608	0.889717	0.050748
BRK1	3	0.24646	0.968651	0.013833	*AEN*	4	-0.10383	0.889717	0.050748
CBR1	2	0.25081	0.968651	0.013833	*TMEM42*	2	-0.10236	0.889717	0.050748
ERICH4	3	0.28216	0.968651	0.013833	*PHOX2A*	4	-0.078065	0.889717	0.050748
LRRC19	3	0.29637	0.968651	0.013833	*MPV17*	3	-0.06628	0.889717	0.050748
CLPP	3	-0.36848	0.968767	0.013781	*VSTM1*	3	-0.057409	0.889717	0.050748
TNFRSF12A	4	-0.34203	0.968767	0.013781	*FNDC4*	4	-0.05576	0.889717	0.050748
TENT5A	3	-0.34061	0.968767	0.013781	*UBL4A*	4	-0.052163	0.889717	0.050748
TMCC3	4	-0.23681	0.968767	0.013781	*ZXDB*	4	-0.044004	0.889717	0.050748
DNAJC18	4	-0.14047	0.968767	0.013781	*TM9SF4*	4	-0.041612	0.889717	0.050748
DECR2	4	-0.11056	0.968767	0.013781	*SRBD1*	4	-0.02668	0.889717	0.050748
WBP2	4	-0.10306	0.968767	0.013781	*CDKAL1*	4	-0.023982	0.889717	0.050748
CDH6	4	0.025245	0.968767	0.013781	*PCSK6*	4	-0.017725	0.889717	0.050748
TNFRSF9	4	0.056223	0.968767	0.013781	*ZNF777*	4	-0.016559	0.889717	0.050748
TRMT2B	2	0.10593	0.968767	0.013781	*LRRC74B*	4	-0.004134	0.889717	0.050748

HERC4	4	-0.00484	0.968851	0.013743	*HNMT*	4	-0.003288	0.889717	0.050748
TSPAN17	4	0.066782	0.968851	0.013743	*RASSF9*	4	0.002751	0.889717	0.050748
PRR7	2	-0.38482	0.969192	0.01359	*ERGIC3*	4	0.004396	0.889717	0.050748
GAA	3	-0.078799	0.969192	0.01359	*SLC29A3*	4	0.010096	0.889717	0.050748
PNMA8A	4	-0.05508	0.969192	0.01359	*DPP3*	4	0.016984	0.889717	0.050748
DNAH10	4	-0.034995	0.969192	0.01359	*NR2C1*	4	0.024508	0.889717	0.050748
CCNJL	3	-0.34633	0.969313	0.013536	*RAB28*	4	0.047043	0.889717	0.050748
KRAS	4	-0.097342	0.969313	0.013536	*PPP4R4*	4	0.058848	0.889717	0.050748
ANGPTL8	4	-0.08774	0.969313	0.013536	*RALGDS*	2	0.060557	0.889717	0.050748
LPAR6	4	0.004348	0.969313	0.013536	*FBXO3*	3	0.078321	0.889717	0.050748
TCL1A	4	0.13801	0.969313	0.013536	*PRDM13*	4	0.093756	0.889717	0.050748
EPS8L2	4	0.33012	0.969313	0.013536	*CD244*	4	0.10341	0.889717	0.050748
ATP5MPL	1	-0.53444	0.969377	0.013507	*NLN*	4	0.10986	0.889717	0.050748
TTI2	3	-0.50809	0.969377	0.013507	*SYNGR4*	4	0.12068	0.889717	0.050748
HOXA10	4	-0.45774	0.969377	0.013507	*PCDHA13*	2	0.14322	0.889717	0.050748
RESF1	4	-0.29138	0.969377	0.013507	*C15orf65*	2	0.14837	0.889717	0.050748
FOXN2	3	-0.27704	0.969377	0.013507	*MAGEA3*	2	0.15506	0.889717	0.050748
DIAPH2	3	-0.25963	0.969377	0.013507	*DDX50*	3	0.15965	0.889717	0.050748
C22orf15	4	-0.24739	0.969377	0.013507	*GDF1*	2	0.16292	0.889717	0.050748
NSFL1C	4	-0.23586	0.969377	0.013507	*PTPRH*	3	0.20824	0.889717	0.050748
GLT8D2	3	-0.14236	0.969377	0.013507	*SALL1*	4	0.22016	0.889717	0.050748
SUCLA2	4	-0.13734	0.969377	0.013507	*TMSB10*	2	0.28749	0.889717	0.050748
PKD1L1	3	-0.12611	0.969377	0.013507	*CYB561D2*	4	0.32322	0.889717	0.050748
RAB7B	4	-0.078938	0.969377	0.013507	*SPATA5L1*	4	0.38527	0.889717	0.050748
ZNF707	4	-0.055761	0.969377	0.013507	*CENPBD1*	2	0.40157	0.889717	0.050748
SLC22A18	4	0.1309	0.969377	0.013507	*CIRBP*	2	0.45034	0.889717	0.050748
CDK2	4	0.14881	0.969377	0.013507	*TRAPPC2*	1	-0.23716	0.889874	0.050671
USP17L2	1	-0.46306	0.970311	0.013089	*NOL4L*	3	0.038996	0.889874	0.050671
FNIP1	3	-0.36455	0.970311	0.013089	*WSCD1*	4	0.13162	0.889874	0.050671
HLA-DOA	4	-0.32478	0.970311	0.013089	*LRP6*	4	-0.23203	0.890391	0.050419
UMODL1	4	-0.23342	0.970311	0.013089	*PPM1M*	4	-0.17115	0.890391	0.050419
TRA2B	3	-0.19443	0.970311	0.013089	*YIPF3*	4	-0.14116	0.890391	0.050419
SRA1	3	-0.1915	0.970311	0.013089	*FABP1*	4	-0.065103	0.890391	0.050419
SPIRE1	4	-0.18429	0.970311	0.013089	*TMEM170A*	4	-0.061459	0.890391	0.050419
TRMT13	4	-0.1629	0.970311	0.013089	*GMEB2*	4	0.006209	0.890391	0.050419
CCDC9	4	-0.15586	0.970311	0.013089	*PXDC1*	4	0.007138	0.890391	0.050419
USP28	4	-0.14308	0.970311	0.013089	*TAPT1*	4	0.023004	0.890391	0.050419
PCDHGA1	4	-0.13324	0.970311	0.013089	*CACNA1S*	4	0.024705	0.890391	0.050419
NOD1	4	-0.10483	0.970311	0.013089	*SPATA2L*	4	0.075089	0.890391	0.050419
MYO1E	3	-0.072245	0.970311	0.013089	*PDS5B*	3	0.13526	0.890391	0.050419
LRRC24	4	-0.063892	0.970311	0.013089	*MAB21L4*	4	0.14937	0.890391	0.050419
SIMC1	4	-0.059549	0.970311	0.013089	*ACMSD*	2	0.17308	0.890391	0.050419
GREM1	4	-0.058846	0.970311	0.013089	*CATSPERD*	3	0.22904	0.890391	0.050419
ROBO2	3	-0.053196	0.970311	0.013089	*COL17A1*	1	-0.36765	0.89041	0.05041
UTP11	4	-0.039735	0.970311	0.013089	*UCP2*	4	-0.069618	0.89041	0.05041
TNIP3	4	-0.030269	0.970311	0.013089	*C4orf48*	2	-0.44223	0.890662	0.050287
ZNF696	4	-0.027302	0.970311	0.013089	*LILRA6*	3	-0.3263	0.890662	0.050287
MXRA5	4	0.00447	0.970311	0.013089	*ABHD2*	3	-0.32167	0.890662	0.050287
NFKBIL1	4	0.01717	0.970311	0.013089	*SSR3*	4	-0.025965	0.890662	0.050287
SVIL	4	0.024518	0.970311	0.013089	*ZNF468*	4	-0.23559	0.890701	0.050268
MPHOSPH6	4	0.056411	0.970311	0.013089	*STAT1*	4	-0.018302	0.890701	0.050268
ZNF845	4	0.060259	0.970311	0.013089	*EIF2B5*	4	0.007979	0.890701	0.050268
PGAP4	3	0.080092	0.970311	0.013089	*PLAUR*	3	0.022938	0.890701	0.050268
PRKCQ	4	0.085407	0.970311	0.013089	*PLP1*	4	0.024252	0.890701	0.050268
C17orf112	4	0.090704	0.970311	0.013089	*KIAA1328*	4	0.027864	0.890701	0.050268
IL16	3	0.11874	0.970311	0.013089	*CSPG4*	3	0.035741	0.890701	0.050268
SEC11C	3	0.13178	0.970311	0.013089	*ROCK1*	3	0.059582	0.890701	0.050268
AKT2	3	0.17758	0.970311	0.013089	*GLMN*	4	0.21742	0.890701	0.050268

SCGB2A1	4	0.19278	0.970311	0.013089	*FDFT1*	4	-0.042731	0.890753	0.050243
CHRM5	4	0.19338	0.970311	0.013089	*L3HYPDH*	3	-0.48346	0.890771	0.050234
PTPN2	3	0.23448	0.970311	0.013089	*DCLK2*	3	-0.27632	0.890771	0.050234
IPO9	4	0.28416	0.970311	0.013089	*CCDC85C*	3	-0.30264	0.890929	0.050157
ZSCAN32	3	0.30852	0.970311	0.013089	*BFAR*	4	-0.19524	0.890929	0.050157
SMC2	2	-0.36625	0.970392	0.013053	*NCLN*	3	-0.16785	0.890929	0.050157
UNC13C	3	-0.25895	0.970392	0.013053	*MYH11*	2	-0.13165	0.890929	0.050157
TAAR5	3	0.31192	0.970392	0.013053	*TET1*	4	-0.11483	0.890929	0.050157
MAP3K13	2	-0.21859	0.97046	0.013022	*RASSF10*	4	0.027541	0.890929	0.050157
FBXO31	4	-0.10792	0.97046	0.013022	*PATL1*	3	0.10693	0.890929	0.050157
SLC43A2	4	-0.005558	0.97046	0.013022	*NATD1*	2	0.24335	0.890929	0.050157
FHIT	4	0.009474	0.97046	0.013022	*KATNBL1*	4	-0.19681	0.891497	0.04988
STIM1	4	0.02064	0.97046	0.013022	*CT62*	3	-0.30746	0.891601	0.04983
DNASE2	4	0.1214	0.97046	0.013022	*DSN1*	3	0.007291	0.891601	0.04983
TAF15	4	0.15219	0.97046	0.013022	*GPI*	2	-0.074809	0.891781	0.049742
CSF3R	4	0.15319	0.97046	0.013022	*CAPZA2*	3	0.008643	0.892392	0.049445
SLC22A11	4	0.20202	0.97046	0.013022	*SLC27A1*	3	-0.16985	0.892798	0.049247
C12orf10	4	0.21987	0.97046	0.013022	*FAM169B*	3	-0.13108	0.892798	0.049247
TP53BP1	4	0.23773	0.97046	0.013022	*FBXO15*	4	-0.08201	0.892798	0.049247
YTHDF1	1	-0.43359	0.970533	0.01299	*IMPDH1*	2	-0.040613	0.892798	0.049247
ERO1A	3	-0.28997	0.970533	0.01299	*VAV1*	4	-0.030805	0.892798	0.049247
NPTN	3	-0.25489	0.970533	0.01299	*BLOC1S2*	3	-0.020545	0.892798	0.049247
MCPH1	3	-0.22452	0.970533	0.01299	*TMEM251*	4	-0.003615	0.892798	0.049247
INAFM1	4	-0.11433	0.970533	0.01299	*LURAP1*	4	0.054938	0.892798	0.049247
ASB8	3	-0.10001	0.970533	0.01299	*MANBAL*	4	0.14198	0.892798	0.049247
QRFPR	4	0.006397	0.970533	0.01299	*ASTL*	4	0.17525	0.892798	0.049247
LENG1	4	0.28623	0.970533	0.01299	*MRM1*	4	0.18445	0.892798	0.049247
GNB1	3	-0.33897	0.970569	0.012974	*FKBP5*	4	0.19145	0.892798	0.049247
TMEM39B	3	-0.23633	0.970569	0.012974	*ZNF100*	3	-0.29314	0.892987	0.049155
IVNS1ABP	4	0.21686	0.970569	0.012974	*BBS4*	4	-0.23243	0.892987	0.049155
ANAPC13	2	0.2316	0.970569	0.012974	*C5orf51*	3	-0.2451	0.893159	0.049071
IFRD1	4	-0.31782	0.970727	0.012903	*SRPK3*	4	-0.19849	0.893159	0.049071
ZG16B	3	-0.24667	0.970727	0.012903	*TMEM269*	2	-0.1095	0.893159	0.049071
PRKAR1B	3	-0.21781	0.970727	0.012903	*HILPDA*	4	-5.68E-04	0.893159	0.049071
DNAH5	4	-0.16652	0.970727	0.012903	*DNAJC24*	4	0.095233	0.893159	0.049071
BAGE2	2	-0.14983	0.970727	0.012903	*INPP5F*	4	0.098763	0.893168	0.049067
PPP1R2	4	0.059552	0.970727	0.012903	*PSRC1*	3	-0.34866	0.893305	0.049
NOMO2	2	0.10332	0.970727	0.012903	*HMCES*	4	0.04662	0.893356	0.048975
TTLL5	3	0.22193	0.970727	0.012903	*BEST2*	1	-0.37773	0.893536	0.048888
GSC	4	-0.045813	0.971622	0.012503	*WDR46*	3	0.043876	0.894187	0.048572
CD2BP2	4	0.17321	0.971622	0.012503	*HMX1*	3	-0.52153	0.89423	0.048551
ANKRD34A	2	-0.25782	0.971877	0.012389	*RBM7*	3	-0.36496	0.89423	0.048551
KRT19	3	-0.47263	0.972127	0.012277	*FAM126A*	3	-0.33632	0.89423	0.048551
CT45A1	1	-0.21897	0.972127	0.012277	*VWA2*	4	-0.33095	0.89423	0.048551
CDR2	2	-0.091315	0.972127	0.012277	*RBKS*	3	-0.31074	0.89423	0.048551
FAM122B	4	0.064528	0.972127	0.012277	*TUSC3*	3	-0.24045	0.89423	0.048551
SRC	2	0.23181	0.972167	0.012259	*NPL*	2	-0.14709	0.89423	0.048551
C11orf98	1	-0.40567	0.972354	0.012176	*ALG5*	4	-0.12736	0.89423	0.048551
RBM38	1	-0.34497	0.972354	0.012176	*ACSBG1*	4	-0.10476	0.89423	0.048551
TEX19	4	-0.3117	0.972354	0.012176	*ANGEL2*	3	0.022811	0.89423	0.048551
H2BC10	3	-0.28308	0.972354	0.012176	*CLSTN1*	2	0.0577	0.89423	0.048551
ZNF317	4	-0.27764	0.972354	0.012176	*UROD*	4	0.066731	0.89423	0.048551
MYO1C	3	-0.26895	0.972354	0.012176	*BOLL*	4	0.073022	0.89423	0.048551
REEP2	4	-0.26257	0.972354	0.012176	*TMEM200B*	3	0.073714	0.89423	0.048551
CDYL2	4	-0.25939	0.972354	0.012176	*AOC2*	4	0.15309	0.89423	0.048551
ZNF806	4	-0.25758	0.972354	0.012176	*EBF4*	4	0.15439	0.89423	0.048551
ABHD6	4	-0.25217	0.972354	0.012176	*SELPLG*	4	0.16552	0.89423	0.048551
KIAA1841	4	-0.24426	0.972354	0.012176	*SLC25A10*	4	0.17536	0.89423	0.048551

SNTA1	4	-0.20143	0.972354	0.012176	*RC3H1*	2	0.30661	0.89423	0.048551
IDS	4	-0.14561	0.972354	0.012176	*VSIG8*	2	0.36834	0.89423	0.048551
LITAF	4	-0.14149	0.972354	0.012176	*VPS35*	3	-0.33474	0.894537	0.048402
FZD1	4	-0.12384	0.972354	0.012176	*DBI*	1	-0.45257	0.894631	0.048356
ARR3	4	-0.056612	0.972354	0.012176	*RHOD*	2	-0.34065	0.894631	0.048356
CAPN10	4	-0.045943	0.972354	0.012176	*ABTB2*	4	0.036268	0.894631	0.048356
PABPC1	4	-0.043465	0.972354	0.012176	*LRRN4*	4	0.097607	0.894631	0.048356
PPP1R3C	3	0.007628	0.972354	0.012176	*RNF145*	4	0.1306	0.894631	0.048356
NANOS2	4	0.019731	0.972354	0.012176	*IFIT1B*	4	0.31459	0.894631	0.048356
RARRES2	4	0.033888	0.972354	0.012176	*SHTN1*	3	0.030419	0.894682	0.048331
COX8C	4	0.073226	0.972354	0.012176	*CARMIL3*	3	-0.29041	0.894775	0.048286
PYCR2	4	0.089491	0.972354	0.012176	*GINS1*	3	-0.28508	0.894775	0.048286
KRTCAP2	4	0.12005	0.972354	0.012176	*DNAJC14*	4	-0.21408	0.894775	0.048286
PSMD4	4	0.14698	0.972354	0.012176	*CLDN7*	4	-0.18577	0.894775	0.048286
EFHC2	4	0.15225	0.972354	0.012176	*CAMKK2*	3	-0.16275	0.894775	0.048286
AKR1C1	1	0.20431	0.972354	0.012176	*LOC10028703*	4	0.031556	0.894775	0.048286
KIF13B	4	0.21096	0.972354	0.012176	*CISD3*	4	0.096031	0.894775	0.048286
PPIG	4	0.22039	0.972354	0.012176	*ABI1*	4	0.15565	0.894775	0.048286
VWA8	3	0.22489	0.972354	0.012176	*CCNK*	4	0.18168	0.894775	0.048286
NKIRAS1	4	0.23864	0.972354	0.012176	*DPF2*	4	0.22011	0.894775	0.048286
ALOX12B	2	0.24196	0.972354	0.012176	*ATP5MC1*	2	0.27628	0.894775	0.048286
ATP2B4	4	0.24976	0.972354	0.012176	*TMPRSS2*	2	-0.27331	0.895209	0.048075
RTN4	4	0.27516	0.972354	0.012176	*AQP5*	4	0.021794	0.895601	0.047886
ZNF219	4	0.29078	0.972354	0.012176	*STEAP2*	3	-0.35108	0.895633	0.04787
DUSP13	4	0.31533	0.972354	0.012176	*RND1*	3	-0.31696	0.895633	0.04787
ERCC2	4	0.44358	0.972354	0.012176	*PPARD*	4	-0.1839	0.895633	0.04787
FTCD	2	0.18465	0.972735	0.012006	*RBM5*	4	-0.15888	0.895633	0.04787
UBA6	3	0.15442	0.972945	0.011912	*INKA1*	4	-0.11813	0.895633	0.04787
HSPB3	3	-0.26011	0.973539	0.011647	*PANX2*	3	-0.11197	0.895633	0.04787
SLU7	3	-0.42186	0.973619	0.011611	*IQCN*	4	0.10373	0.895633	0.04787
RPL30	4	-0.36639	0.973619	0.011611	*CDH3*	3	0.23939	0.895633	0.04787
ZC3H13	4	-0.3545	0.973619	0.011611	*UNKL*	1	0.421	0.895633	0.04787
HSPA6	1	-0.33506	0.973619	0.011611	*RELT*	4	-0.32388	0.895708	0.047834
MICAL2	1	-0.31688	0.973619	0.011611	*ADRA1D*	2	-0.28659	0.895708	0.047834
SWT1	4	-0.31488	0.973619	0.011611	*ING1*	3	-0.21526	0.895708	0.047834
FOXRED2	3	-0.30653	0.973619	0.011611	*TFAP2A*	3	-0.19829	0.895708	0.047834
ZNF85	3	-0.28766	0.973619	0.011611	*FRG1*	3	-0.14239	0.895708	0.047834
TSPAN9	3	-0.28005	0.973619	0.011611	*OLA1*	4	0.13518	0.895708	0.047834
XYLT1	3	-0.26874	0.973619	0.011611	*TSC22D4*	4	0.17778	0.895708	0.047834
OSGEP	3	-0.26814	0.973619	0.011611	*SYNM*	1	0.27091	0.895708	0.047834
BAZ1B	4	-0.26219	0.973619	0.011611	*CUL3*	4	0.27814	0.895708	0.047834
SLC4A7	2	-0.26101	0.973619	0.011611	*SLC39A4*	4	-0.12693	0.89586	0.04776
TMEM89	4	-0.24007	0.973619	0.011611	*SLC22A1*	4	-0.007074	0.89586	0.04776
RBPJL	3	-0.23659	0.973619	0.011611	*NPAS1*	4	0.14152	0.89586	0.04776
SENP6	2	-0.22239	0.973619	0.011611	*PALM3*	3	-0.051136	0.895885	0.047748
NUDT2	4	-0.21808	0.973619	0.011611	*ARMC12*	3	0.13169	0.895885	0.047748
FILIP1	3	-0.21687	0.973619	0.011611	*EIF3F*	3	0.17041	0.895885	0.047748
GCAT	4	-0.20976	0.973619	0.011611	*PEX11G*	3	-0.38855	0.89619	0.0476
ARF3	3	-0.19959	0.973619	0.011611	*GP1BA*	2	-0.073614	0.896334	0.04753
KIAA1549L	4	-0.1987	0.973619	0.011611	*RRP1*	2	0.007142	0.896334	0.04753
ANKDD1B	4	-0.19845	0.973619	0.011611	*C11orf94*	4	-0.067732	0.896444	0.047477
HDAC6	3	-0.16783	0.973619	0.011611	*GTF2H1*	4	0.057988	0.896444	0.047477
NPM3	4	-0.1579	0.973619	0.011611	*CRKL*	4	0.16667	0.896444	0.047477
ADCYAP1	3	-0.15234	0.973619	0.011611	*UBN1*	2	-0.14509	0.896587	0.047408
NMNAT3	4	-0.14688	0.973619	0.011611	*S100A7A*	2	-0.014974	0.896587	0.047408
PLIN1	3	-0.14466	0.973619	0.011611	*FASTKD3*	4	0.11663	0.896638	0.047383
TSPYL5	4	-0.12358	0.973619	0.011611	*CAT*	4	-0.22626	0.896739	0.047334
PGBD4	4	-0.12177	0.973619	0.011611	*CDON*	4	8.46E-04	0.896739	0.047334

PPP3CA	4	-0.11833	0.973619	0.011611	*FYN*	2	-0.15353	0.896916	0.047248
AHCYL2	4	-0.11813	0.973619	0.011611	*WRN*	3	-0.015952	0.896924	0.047244
NUP188	4	-0.10156	0.973619	0.011611	*MEDAG*	3	-0.34082	0.897447	0.046991
PCBD2	4	-0.098793	0.973619	0.011611	*RSPH3*	3	-0.25252	0.897447	0.046991
ZNF551	4	-0.097605	0.973619	0.011611	*DGKH*	3	-0.25087	0.897447	0.046991
CRB2	4	-0.066389	0.973619	0.011611	*GCNT3*	4	-0.07983	0.897447	0.046991
SOD2	3	-0.055981	0.973619	0.011611	*RNF183*	4	0.2017	0.897447	0.046991
HNRNPA1L2	3	-0.054913	0.973619	0.011611	*NCAPD3*	4	0.31262	0.897447	0.046991
SPRED2	3	-0.033227	0.973619	0.011611	*TWF1*	1	0.32114	0.897447	0.046991
RPL38	4	0.003023	0.973619	0.011611	*EVX1*	3	-0.40022	0.897633	0.046901
COQ8B	3	0.02085	0.973619	0.011611	*MZT2B*	2	-0.36251	0.897633	0.046901
CD72	4	0.032562	0.973619	0.011611	*MUC21*	3	-0.31965	0.897633	0.046901
ITGA10	4	0.039857	0.973619	0.011611	*CD163L1*	3	-0.24634	0.897633	0.046901
ANPEP	3	0.043247	0.973619	0.011611	*NTNG1*	3	-0.18519	0.897633	0.046901
NKAIN4	3	0.049293	0.973619	0.011611	*OSTF1*	4	-0.15602	0.897633	0.046901
KYAT1	2	0.070855	0.973619	0.011611	*IKZF4*	4	0.031214	0.897633	0.046901
CYB5R2	3	0.080574	0.973619	0.011611	*KRI1*	3	0.036154	0.897633	0.046901
LIX1L	4	0.090658	0.973619	0.011611	*DNAH5*	4	0.046034	0.897633	0.046901
H4C9	4	0.10433	0.973619	0.011611	*DNAH10*	4	0.075669	0.897633	0.046901
UBE2W	3	0.12105	0.973619	0.011611	*ADCK2*	4	0.08117	0.897633	0.046901
IL36RN	4	0.13069	0.973619	0.011611	*ZNF860*	4	0.11077	0.897633	0.046901
STK4	3	0.13745	0.973619	0.011611	*TTLL7*	4	0.12088	0.897633	0.046901
C5orf64	2	0.15145	0.973619	0.011611	*ACBD4*	4	0.1501	0.897633	0.046901
NIBAN2	1	0.16234	0.973619	0.011611	*EIF1*	2	0.1796	0.897633	0.046901
FAM71E1	3	0.17372	0.973619	0.011611	*AP3D1*	4	0.18072	0.897633	0.046901
SCIMP	1	0.18431	0.973619	0.011611	*MMP24*	4	0.19587	0.897633	0.046901
MFSD9	3	0.18579	0.973619	0.011611	*UACA*	4	0.22792	0.897633	0.046901
C15orf39	4	0.18703	0.973619	0.011611	*ZBED9*	4	0.005797	0.897708	0.046865
PTCHD4	3	0.18845	0.973619	0.011611	*NLRC5*	4	0.20502	0.897708	0.046865
EPHX1	3	0.18972	0.973619	0.011611	*LEF1*	2	0.24304	0.897708	0.046865
MYOZ2	2	0.19911	0.973619	0.011611	*ABCG5*	2	0.25624	0.897708	0.046865
CAPN8	4	0.19921	0.973619	0.011611	*ALKBH4*	4	-0.03554	0.897977	0.046735
RIMS4	2	0.20371	0.973619	0.011611	*TMEM259*	4	0.02634	0.897977	0.046735
CBLN3	3	0.21258	0.973619	0.011611	*PRSS56*	2	-0.17296	0.898196	0.046629
KCNJ14	4	0.21371	0.973619	0.011611	*DLEU7*	2	-0.50953	0.898225	0.046615
PDGFC	3	0.21405	0.973619	0.011611	*ANKFY1*	1	-0.38301	0.898225	0.046615
ATL1	4	0.21657	0.973619	0.011611	*KAZALD1*	4	-0.30336	0.898225	0.046615
BAIAP2	4	0.21797	0.973619	0.011611	*DYNC2H1*	3	-0.2621	0.898225	0.046615
LMNB2	3	0.22054	0.973619	0.011611	*PGBD5*	4	-0.25309	0.898225	0.046615
RNF32	3	0.22175	0.973619	0.011611	*KIAA0754*	4	-0.24618	0.898225	0.046615
OR52N4	3	0.22626	0.973619	0.011611	*SOD3*	3	-0.24534	0.898225	0.046615
MBOAT4	4	0.22789	0.973619	0.011611	*CX3CR1*	2	-0.23281	0.898225	0.046615
KLHL12	3	0.24011	0.973619	0.011611	*ZNF853*	4	-0.22325	0.898225	0.046615
ZNF442	3	0.24479	0.973619	0.011611	*MAPRE3*	4	-0.19884	0.898225	0.046615
MAP3K9	3	0.25964	0.973619	0.011611	*RAB7B*	4	-0.19862	0.898225	0.046615
SPTB	3	0.26006	0.973619	0.011611	*MSRB2*	4	-0.19718	0.898225	0.046615
CSTB	3	0.26097	0.973619	0.011611	*ZNF184*	4	-0.193	0.898225	0.046615
RPL22	1	0.2657	0.973619	0.011611	*CCL28*	4	-0.18972	0.898225	0.046615
MTRR	3	0.27921	0.973619	0.011611	*EPG5*	4	-0.14231	0.898225	0.046615
BAZ1A	3	0.27927	0.973619	0.011611	*LAT2*	3	-0.13333	0.898225	0.046615
RPS21	4	0.28135	0.973619	0.011611	*OSBPL11*	4	-0.012957	0.898225	0.046615
QPCTL	2	0.29	0.973619	0.011611	*SLC6A1*	4	0.011175	0.898225	0.046615
DBP	1	0.33851	0.973619	0.011611	*NEK10*	4	0.012128	0.898225	0.046615
C1orf159	3	0.39832	0.973619	0.011611	*BIRC7*	4	0.16715	0.898225	0.046615
UPF1	3	0.42548	0.973619	0.011611	*B4GALT6*	4	0.21298	0.898225	0.046615
NOP56	3	0.48175	0.973619	0.011611	*LENG8*	3	0.24258	0.898225	0.046615
FCGR1A	1	0.16489	0.973743	0.011556	*KRTAP1-3*	1	0.29446	0.898225	0.046615
ZNF681	3	-0.16875	0.973783	0.011538	*MCM3AP*	4	0.32314	0.898225	0.046615

ARMCX4	3	-0.5434	0.973813	0.011525	*RPL26*	1	0.46692	0.898225	0.046615
DUSP19	3	-0.4451	0.973813	0.011525	*NR4A3*	4	0.12866	0.898653	0.046408
DNAJB11	3	-0.2746	0.973813	0.011525	*SPRY2*	3	-0.22174	0.898745	0.046364
TANGO2	3	-0.21901	0.973813	0.011525	*CYB561D1*	4	-0.18922	0.898879	0.046299
PALB2	3	0.14924	0.973813	0.011525	*ANKZF1*	4	-0.32356	0.899062	0.04621
FGF13	3	0.20078	0.973813	0.011525	*GLB1L2*	2	-0.25612	0.899062	0.04621
C5orf51	3	-0.30156	0.974302	0.011306	*ALDH4A1*	3	-0.15677	0.89912	0.046182
OSR2	4	-0.003502	0.974302	0.011306	*CCDC78*	3	0.12285	0.89912	0.046182
SEPTIN10	3	0.03974	0.974302	0.011306	*PIAS3*	1	-0.20877	0.899148	0.046169
CD34	4	0.06142	0.974302	0.011306	*PDE3A*	2	-0.16721	0.899148	0.046169
SLC66A1L	4	0.079318	0.974302	0.011306	*ZNF620*	4	0.006996	0.899148	0.046169
DPP9-AS1	2	0.22167	0.974302	0.011306	*TBX21*	3	0.04811	0.899148	0.046169
ESPN	4	0.28157	0.974302	0.011306	*GSE1*	4	0.065946	0.899148	0.046169
NPPC	1	-0.4345	0.974725	0.011118	*SNIP1*	4	0.10199	0.899148	0.046169
ZMYM3	3	-0.42424	0.974725	0.011118	*NCAPH*	4	0.13556	0.899148	0.046169
ATP8A1	2	-0.26263	0.974725	0.011118	*NKX2-2*	4	0.19312	0.899148	0.046169
RIOX1	3	-0.26261	0.974725	0.011118	*MED14*	4	0.19347	0.899148	0.046169
SYDE2	4	-0.22542	0.974725	0.011118	*TRMT2A*	4	-0.088765	0.899499	0.045999
BZW1	3	-0.22372	0.974725	0.011118	*RASSF6*	4	0.034075	0.899499	0.045999
RIPOR2	3	-0.22278	0.974725	0.011118	*ARAP1*	3	-0.13051	0.899507	0.045996
KCNE4	4	-0.21466	0.974725	0.011118	*CCDC3*	4	-0.19678	0.899766	0.04587
CFAP73	4	-0.20361	0.974725	0.011118	*HAS3*	4	-0.024219	0.899838	0.045835
H2AP	4	-0.15645	0.974725	0.011118	*NEFL*	2	0.11453	0.899838	0.045835
GLRA1	3	-0.028908	0.974725	0.011118	*EXOSC4*	4	0.15166	0.899838	0.045835
PIGR	3	0.05354	0.974725	0.011118	*PDK2*	4	0.19491	0.899838	0.045835
BNC2	4	0.054268	0.974725	0.011118	*MMP25*	1	-0.43574	0.899912	0.0458
SLC29A3	4	0.16755	0.974725	0.011118	*ACR*	3	-0.43076	0.899912	0.0458
CD8A	4	0.17364	0.974725	0.011118	*ZNF585A*	3	-0.33333	0.899912	0.0458
ZNF266	4	0.20424	0.974725	0.011118	*PSD*	4	-0.2776	0.899912	0.0458
KRTAP5-1	3	0.28007	0.974725	0.011118	*OVOL1*	3	-0.26651	0.899912	0.0458
PSMG3	4	0.3689	0.974725	0.011118	*CADM2*	3	-0.23219	0.899912	0.0458
HELLS	2	-0.36892	0.974795	0.011087	*REV3L*	2	-0.22461	0.899912	0.0458
ADGRF4	2	-0.30517	0.974795	0.011087	*PARP10*	2	-0.20852	0.899912	0.0458
GRB2	4	-0.28732	0.974795	0.011087	*UBIAD1*	3	-0.19597	0.899912	0.0458
RAP2B	4	-0.079722	0.974795	0.011087	*LGR5*	4	-0.19159	0.899912	0.0458
SPINT1	4	0.10009	0.974795	0.011087	*C10orf62*	4	-0.17134	0.899912	0.0458
TSTD2	4	0.1389	0.974795	0.011087	*NSFL1C*	4	-0.14738	0.899912	0.0458
CD200R1	4	0.14575	0.975086	0.010957	*SLC7A4*	3	-0.11221	0.899912	0.0458
CDH16	3	-0.16791	0.975407	0.010814	*GABARAPL1*	3	-0.093613	0.899912	0.0458
CTSB	2	-0.091837	0.975407	0.010814	*WDCP*	4	-0.081979	0.899912	0.0458
ZC4H2	3	0.18134	0.975407	0.010814	*TPP2*	4	-0.081302	0.899912	0.0458
TBX3	2	0.21297	0.975407	0.010814	*TNFRSF1B*	4	-0.070317	0.899912	0.0458
WDR36	3	0.22223	0.975407	0.010814	*PDE4B*	4	-0.067411	0.899912	0.0458
SUFU	3	0.3792	0.975407	0.010814	*CCDC186*	4	-0.038249	0.899912	0.0458
TMEM151B	4	-0.14743	0.975444	0.010797	*TMEM178B*	4	-0.029533	0.899912	0.0458
ARL13B	4	0.038397	0.975444	0.010797	*APBB1*	3	-0.021386	0.899912	0.0458
SLC39A10	1	-0.36355	0.975479	0.010782	*CSNK1G3*	4	-0.006543	0.899912	0.0458
KLF12	4	-0.24698	0.975479	0.010782	*HDLBP*	4	0.010934	0.899912	0.0458
RNF217	4	0.033313	0.975479	0.010782	*GCNT7*	4	0.015197	0.899912	0.0458
ODR4	3	-0.14479	0.975519	0.010764	*STAT4*	3	0.045875	0.899912	0.0458
RPA2	3	-0.56221	0.975623	0.010718	*SNRNP35*	4	0.061927	0.899912	0.0458
CHRAC1	2	-0.54837	0.975623	0.010718	*KCNQ4*	4	0.066842	0.899912	0.0458
F2R	3	-0.43873	0.975623	0.010718	*EML2*	4	0.069062	0.899912	0.0458
FAM8A1	3	-0.38996	0.975623	0.010718	*RAB25*	3	0.073259	0.899912	0.0458
PPP1R26	3	-0.36402	0.975623	0.010718	*GHSR*	4	0.081814	0.899912	0.0458
NFATC2IP	2	-0.3439	0.975623	0.010718	*WDR66*	4	0.10015	0.899912	0.0458
TMEM86B	3	-0.34202	0.975623	0.010718	*TSG101*	4	0.11271	0.899912	0.0458
SHANK3	4	-0.31063	0.975623	0.010718	*PTPRK*	3	0.11924	0.899912	0.0458

DZANK1	3	-0.25728	0.975623	0.010718	*PNPLA1*	3	0.12435	0.899912	0.0458
AASS	4	-0.22044	0.975623	0.010718	*SRL*	3	0.12451	0.899912	0.0458
NUDCD2	3	-0.21954	0.975623	0.010718	*PFDN1*	4	0.14652	0.899912	0.0458
ANKMY2	1	-0.19606	0.975623	0.010718	*RASA4*	1	0.15408	0.899912	0.0458
ZMAT1	4	-0.18212	0.975623	0.010718	*ZNF3*	4	0.15782	0.899912	0.0458
OR13J1	3	-0.17346	0.975623	0.010718	*ST14*	4	0.17401	0.899912	0.0458
AMOTL2	3	-0.15535	0.975623	0.010718	*TMC7*	4	0.1907	0.899912	0.0458
SLAMF1	3	-0.15441	0.975623	0.010718	*SEMA3G*	4	0.20302	0.899912	0.0458
FYB2	4	-0.14634	0.975623	0.010718	*FBXO31*	4	0.21635	0.899912	0.0458
LNP1	4	-0.1362	0.975623	0.010718	*TMEM150C*	4	0.23508	0.899912	0.0458
CECR2	4	-0.13232	0.975623	0.010718	*ENTPD7*	3	0.2499	0.899912	0.0458
ERICH6	4	-0.12296	0.975623	0.010718	*GTF3C5*	4	0.25169	0.899912	0.0458
CDRT1	3	-0.11429	0.975623	0.010718	*ZNF546*	4	0.25548	0.899912	0.0458
THYN1	4	-0.091853	0.975623	0.010718	*TUT4*	3	0.26281	0.899912	0.0458
RTRAF	4	-0.01663	0.975623	0.010718	*SLC12A7*	4	0.28124	0.899912	0.0458
CDHR2	3	0.00154	0.975623	0.010718	*AK5*	3	-0.2621	0.900211	0.045656
BET1	3	0.014562	0.975623	0.010718	*ILDR1*	4	-0.20684	0.900241	0.045641
EPHA2	3	0.075902	0.975623	0.010718	*TMEM25*	4	-0.18318	0.900241	0.045641
ADAMTS15	4	0.094215	0.975623	0.010718	*SLC16A2*	3	0.030824	0.900241	0.045641
LHFPL2	4	0.10349	0.975623	0.010718	*C5orf22*	4	0.055697	0.900241	0.045641
PDLIM5	4	0.14299	0.975623	0.010718	*XAB2*	4	-0.10736	0.900343	0.045592
SYDE1	4	0.18099	0.975623	0.010718	*PAX6*	4	7.44E-04	0.900343	0.045592
TRAF3	3	0.19085	0.975623	0.010718	*HOXC10*	2	0.024674	0.900343	0.045592
G2E3	3	0.19412	0.975623	0.010718	*IQCA1*	4	0.04126	0.900343	0.045592
HAVCR1	4	0.19548	0.975623	0.010718	*TACO1*	4	0.088997	0.900343	0.045592
NDUFA10	3	0.19695	0.975623	0.010718	*ESD*	4	0.15632	0.900343	0.045592
FOSB	3	0.19705	0.975623	0.010718	*NIPA1*	4	0.16093	0.900343	0.045592
TRMT112	4	0.20766	0.975623	0.010718	*ANAPC10*	4	0.24986	0.900343	0.045592
DISP1	3	0.21128	0.975623	0.010718	*ATP13A4*	4	-0.30147	0.901083	0.045235
BMP1	3	0.21173	0.975623	0.010718	*ATP6V1C2*	3	-0.25569	0.901083	0.045235
ADAM23	4	0.21901	0.975623	0.010718	*B3GNT5*	3	-0.16485	0.901083	0.045235
SRCAP	3	0.23134	0.975623	0.010718	*TPMT*	3	-0.095866	0.901083	0.045235
PPP1R14D	4	0.24083	0.975623	0.010718	*CAMSAP1*	4	0.007231	0.901083	0.045235
SERTAD3	3	0.25028	0.975623	0.010718	*FRMD6*	3	0.05189	0.901083	0.045235
FARSA	4	0.30721	0.975623	0.010718	*TONSL*	4	0.053939	0.901083	0.045235
LRFN3	3	0.33793	0.975623	0.010718	*SBK3*	3	0.087305	0.901083	0.045235
FXR1	2	0.36241	0.975623	0.010718	*GPR27*	3	0.18581	0.901083	0.045235
PHLDA2	1	0.43895	0.975623	0.010718	*TEAD4*	3	0.20972	0.901083	0.045235
LRRC71	3	-0.41543	0.975955	0.01057	*PPP1R16B*	3	-0.29836	0.901123	0.045216
HRH4	4	-0.3085	0.975955	0.01057	*BTBD17*	3	-0.22155	0.901123	0.045216
TLE1	4	-0.23527	0.975955	0.01057	*IL1RN*	3	-0.081801	0.901123	0.045216
ULBP3	4	-0.18816	0.975955	0.01057	*BPNT1*	4	0.033978	0.901123	0.045216
NIPAL2	4	-0.15736	0.975955	0.01057	*FAM161A*	4	0.04617	0.901123	0.045216
CD40LG	4	-0.1056	0.975955	0.01057	*ZNF552*	4	0.074311	0.901123	0.045216
ARHGEF7	3	-0.10428	0.975955	0.01057	*ZNF844*	4	0.096507	0.901123	0.045216
TNS1	4	-0.067359	0.975955	0.01057	*HES2*	4	0.10777	0.901123	0.045216
BDH2	2	-0.001992	0.975955	0.01057	*JDP2*	4	0.13467	0.901123	0.045216
SLC32A1	4	0.069399	0.975955	0.01057	*ZNF211*	4	0.23183	0.901123	0.045216
ZZEF1	4	0.088708	0.975955	0.01057	*DERL1*	3	0.36918	0.901123	0.045216
C1GALT1C1L	4	0.088981	0.975955	0.01057	*CYB5RL*	3	-0.013266	0.901172	0.045193
FAM91A1	4	0.17323	0.975955	0.01057	*FAM20A*	3	-0.3856	0.90164	0.044967
TAF1L	2	0.20202	0.975955	0.01057	*FAM126B*	3	-0.33523	0.90164	0.044967
SELENON	4	0.20318	0.975955	0.01057	*SPNS2*	3	-0.33504	0.90164	0.044967
EFTUD2	4	0.26292	0.975955	0.01057	*TSPYL1*	3	-0.24631	0.90164	0.044967
EML1	4	0.28481	0.975955	0.01057	*EPB41L2*	4	-0.24011	0.90164	0.044967
RFC5	3	0.28594	0.975955	0.01057	*CD74*	3	-0.2365	0.90164	0.044967
FBXO2	3	-0.033016	0.976116	0.010499	*GMPR*	4	-0.18363	0.90164	0.044967
RPS26	1	0.35305	0.976116	0.010499	*SPOCK2*	4	-0.15507	0.90164	0.044967

ARHGEF37	3	-0.41125	0.976479	0.010337	*CNOT6*	3	0.002721	0.90164	0.044967
POC1B	3	-0.2822	0.976479	0.010337	*TBC1D20*	3	0.027781	0.90164	0.044967
PPP3R1	4	-0.10504	0.976479	0.010337	*TTN*	3	0.084121	0.90164	0.044967
UBQLN2	3	0.19678	0.976479	0.010337	*UPP1*	4	0.2057	0.90164	0.044967
CLMP	3	-0.1471	0.976725	0.010228	*PDCL*	4	0.23114	0.90164	0.044967
AFMID	4	-0.24153	0.97709	0.010065	*ADAMTSL3*	3	-0.385	0.901672	0.044951
RAMP2	4	-0.018359	0.97709	0.010065	*ENOX1*	3	-0.29289	0.901672	0.044951
NAT16	4	0.1337	0.97709	0.010065	*WDR27*	3	-0.26811	0.901672	0.044951
P2RX4	3	-0.2098	0.977212	0.010011	*ZNF182*	3	-0.18808	0.901672	0.044951
WDR81	3	-0.076916	0.977287	0.009978	*CFI*	4	-0.14073	0.901672	0.044951
RPS4Y1	4	0.094166	0.977287	0.009978	*DYM*	4	-0.06318	0.901672	0.044951
RAB3D	4	0.17827	0.977287	0.009978	*ABCC5*	4	0.007969	0.901672	0.044951
C16orf54	4	-0.091217	0.977688	0.0098	*GOLGB1*	3	0.11621	0.901672	0.044951
CFAP299	4	0.11914	0.977688	0.0098	*ARHGAP10*	3	0.12777	0.901672	0.044951
ATP5IF1	4	0.17534	0.977688	0.0098	*PRORP*	4	0.20923	0.901672	0.044951
HAUS7	4	0.2117	0.977688	0.0098	*TM2D2*	3	-0.32018	0.90184	0.044871
TAC1	4	0.22877	0.977688	0.0098	*SCTR*	3	-0.30839	0.90184	0.044871
FLVCR1	3	-0.31204	0.978137	0.0096	*RHBDL3*	3	-0.23008	0.90184	0.044871
AMMECR1	3	-0.3112	0.978137	0.0096	*TYW5*	3	0.13591	0.90184	0.044871
SHF	3	-0.31031	0.978137	0.0096	*ONECUT2*	2	0.21705	0.90184	0.044871
SOX9	3	-0.30538	0.978137	0.0096	*ZNF140*	4	0.21887	0.90184	0.044871
KDM6A	2	-0.28923	0.978137	0.0096	*ZNF69*	2	-0.46349	0.901847	0.044867
ZMYM5	2	-0.27846	0.978137	0.0096	*CNGA3*	2	0.19913	0.902308	0.044645
WWTR1	4	-0.27125	0.978137	0.0096	*KCNA7*	4	0.10996	0.902612	0.044499
SOCS1	4	-0.26867	0.978137	0.0096	*VPS33A*	4	0.25219	0.902612	0.044499
TIMM29	3	-0.25065	0.978137	0.0096	*ZNF10*	3	0.081584	0.903197	0.044218
ALDH1B1	3	-0.24989	0.978137	0.0096	*DUSP7*	3	-0.33528	0.903638	0.044006
MED24	3	-0.24035	0.978137	0.0096	*ZBP1*	4	-0.24296	0.903638	0.044006
AREG	3	-0.21444	0.978137	0.0096	*EOMES*	4	-0.22307	0.903638	0.044006
CYP7A1	4	-0.20698	0.978137	0.0096	*ETS2*	4	-0.0482	0.903638	0.044006
TTLL4	3	-0.20164	0.978137	0.0096	*SCAI*	2	0.24137	0.903769	0.043943
SPRY1	3	-0.1862	0.978137	0.0096	*RIMKLB*	3	-0.346	0.903941	0.04386
SLC39A2	4	-0.1648	0.978137	0.0096	*TMPRSS11E*	4	-0.1064	0.903948	0.043857
C16orf96	4	-0.1609	0.978137	0.0096	*ELMOD2*	4	-0.004535	0.904285	0.043695
TTC23L	4	-0.090197	0.978137	0.0096	*SPATA32*	4	-0.006284	0.904601	0.043543
LCT	3	-0.05206	0.978137	0.0096	*CDH16*	3	1.94E-04	0.904601	0.043543
MPV17	3	0.015083	0.978137	0.0096	*BTAF1*	4	0.14027	0.904601	0.043543
SCN3B	3	0.03093	0.978137	0.0096	*MEIS2*	4	0.1472	0.904601	0.043543
AHSP	3	0.050082	0.978137	0.0096	*BNC2*	4	0.025802	0.905514	0.043105
ZNF749	4	0.095639	0.978137	0.0096	*ZNF781*	2	-0.27862	0.90581	0.042963
UBB	3	0.10669	0.978137	0.0096	*C7orf31*	4	-0.20744	0.906153	0.042799
MSRB1	3	0.14148	0.978137	0.0096	*C4orf45*	4	0.021102	0.906153	0.042799
PHF13	3	0.15574	0.978137	0.0096	*ANKRD55*	3	-0.3089	0.906316	0.04272
UPB1	4	0.15745	0.978137	0.0096	*PTP4A1*	3	-0.2954	0.906316	0.04272
TEFM	3	0.16315	0.978137	0.0096	*ISL1*	3	-0.21084	0.906316	0.04272
PCDHGB5	3	0.18581	0.978137	0.0096	*ARMC4*	3	-0.2071	0.906316	0.04272
LSMEM1	3	0.2057	0.978137	0.0096	*TMEM43*	3	-0.072089	0.906316	0.04272
YWHAQ	3	0.2512	0.978137	0.0096	*TGFBR3*	4	0.017401	0.906316	0.04272
TP53TG5	3	0.27565	0.978137	0.0096	*ZASP*	2	0.21065	0.906405	0.042678
MRPL14	3	0.27593	0.978137	0.0096	*IQUB*	4	-0.35632	0.906445	0.042658
SSBP1	3	0.30001	0.978137	0.0096	*MLANA*	3	-0.33153	0.906445	0.042658
LDOC1	3	-0.15826	0.978382	0.009492	*MTM1*	2	-0.26972	0.906445	0.042658
IL18R1	4	-0.044518	0.978749	0.009329	*PRG4*	3	-0.10773	0.906445	0.042658
RTL10	3	-0.2504	0.978993	0.00922	*KREMEN1*	3	-0.092647	0.906445	0.042658
H2AC15	4	-0.24704	0.979427	0.009028	*TNFSF9*	2	0.28873	0.906445	0.042658
NTSR1	4	-0.1629	0.979427	0.009028	*RNASEH2B*	4	0.10625	0.907075	0.042357
GORASP2	4	-0.043367	0.979427	0.009028	*HOXB9*	2	0.27862	0.907075	0.042357
FIZ1	4	0.095974	0.979427	0.009028	*PCDHGA6*	2	0.34969	0.907582	0.042114

TPRA1	4	0.13882	0.979427	0.009028	*C2orf81*	2	0.38757	0.907582	0.042114
DOCK4	4	0.14125	0.979427	0.009028	*RASSF8*	3	-0.016065	0.907629	0.042091
BSND	3	0.25045	0.979427	0.009028	*TEAD2*	4	0.23469	0.907677	0.042069
GTSE1	2	0.13099	0.979544	0.008976	*CDH23*	2	-0.29611	0.907683	0.042066
TRAPPC3	2	0.43851	0.979544	0.008976	*FAM124B*	4	-0.29586	0.90855	0.041651
TBRG4	4	-0.19633	0.979615	0.008944	*LMAN1*	3	-0.28049	0.90855	0.041651
B3GNT9	4	-0.14756	0.979615	0.008944	*DNASE1L1*	4	-0.27049	0.90855	0.041651
NKAIN1	4	0.10575	0.979615	0.008944	*EFCAB5*	4	-0.20837	0.90855	0.041651
ISYNA1	4	0.37958	0.979615	0.008944	*SYT13*	4	-0.12042	0.90855	0.041651
ZNF774	3	0.025449	0.979692	0.00891	*ORMDL3*	4	-0.007393	0.90855	0.041651
GRPR	4	0.10188	0.979692	0.00891	*KIF21B*	4	0.022513	0.90855	0.041651
CACNA1H	4	-0.15232	0.979763	0.008879	*EDA*	2	-0.40268	0.908768	0.041547
PHPT1	4	-0.040476	0.979763	0.008879	*ACYP1*	2	-0.28128	0.908768	0.041547
ING1	3	-0.03967	0.979763	0.008879	*HSPA4L*	1	0.2685	0.909061	0.041407
PCDHA3	4	0.14227	0.979763	0.008879	*PRPF18*	3	-0.43018	0.90908	0.041398
RCBTB1	4	0.041045	0.980373	0.008609	*TRPM7*	1	-0.42736	0.90908	0.041398
PHF14	4	0.095305	0.980373	0.008609	*ZNF681*	3	-0.33917	0.90908	0.041398
ACIN1	3	0.20403	0.980616	0.008501	*SHANK2*	1	-0.31471	0.90908	0.041398
HS6ST2	4	0.087582	0.980818	0.008412	*HBEGF*	3	-0.26442	0.90908	0.041398
TCTE3	4	0.22424	0.980897	0.008377	*JMJD4*	3	-0.15919	0.90908	0.041398
EFCAB5	4	-0.25818	0.980935	0.00836	*BMPR1A*	3	-0.15047	0.90908	0.041398
MT1G	4	-0.013702	0.981462	0.008126	*S100A16*	4	-0.14018	0.90908	0.041398
EXOC6B	4	0.15603	0.981462	0.008126	*FZD3*	4	-0.13258	0.90908	0.041398
RSPO1	4	-0.29428	0.981496	0.008111	*TRAK2*	4	-0.10145	0.90908	0.041398
C22orf34	4	-0.28252	0.981496	0.008111	*OR4C15*	4	-0.096791	0.90908	0.041398
CDKL5	4	-0.21263	0.981496	0.008111	*TRIM24*	4	-0.042892	0.90908	0.041398
SGSM1	4	-0.18599	0.981496	0.008111	*CDCA4*	3	-0.012537	0.90908	0.041398
ATP13A2	4	-0.1247	0.981496	0.008111	*ZNF222*	3	-0.00357	0.90908	0.041398
HOXB2	4	-0.091165	0.981496	0.008111	*RAB27B*	3	0.022576	0.90908	0.041398
GOLIM4	3	-0.084679	0.981496	0.008111	*PINX1*	3	0.023835	0.90908	0.041398
BTN1A1	4	-0.066197	0.981496	0.008111	*ACER2*	4	0.024097	0.90908	0.041398
MRPL17	4	-0.015026	0.981496	0.008111	*ZNF397*	3	0.038808	0.90908	0.041398
PLPP7	4	0.001643	0.981496	0.008111	*SERPINA10*	4	0.043538	0.90908	0.041398
ZNF33A	4	0.002512	0.981496	0.008111	*NFKB1*	4	0.045399	0.90908	0.041398
OTUD1	4	0.073029	0.981496	0.008111	*LRRC15*	4	0.084239	0.90908	0.041398
TMEM41B	4	0.096409	0.981496	0.008111	*GPR55*	4	0.10094	0.90908	0.041398
WDR83OS	4	0.14136	0.981496	0.008111	*ROM1*	4	0.11735	0.90908	0.041398
ARHGAP15	4	0.21165	0.981496	0.008111	*TRPM4*	4	0.1209	0.90908	0.041398
SUPT16H	4	0.41353	0.981496	0.008111	*RBIS*	4	0.1422	0.90908	0.041398
CRYL1	3	-0.36635	0.981618	0.008058	*SNCAIP*	4	0.15105	0.90908	0.041398
HMGA2	4	-0.33683	0.981618	0.008058	*AVL9*	3	0.15564	0.90908	0.041398
H2BC3	3	-0.08964	0.981618	0.008058	*MESP1*	3	0.20522	0.90908	0.041398
DHRS2	4	-0.084557	0.981618	0.008058	*GTF2IRD2B*	1	0.2308	0.90908	0.041398
ROPN1L	4	-0.024573	0.981618	0.008058	*DNAJB7*	4	0.028005	0.909137	0.041371
ANKRD50	4	-0.016291	0.981618	0.008058	*ZNF585B*	4	0.053263	0.909137	0.041371
KLK11	4	0.086853	0.981618	0.008058	*PPP1R27*	4	0.12389	0.909137	0.041371
FAM172A	3	0.090075	0.981618	0.008058	*GLRX2*	4	0.13191	0.909137	0.041371
SPATC1	4	0.12175	0.981618	0.008058	*HBG2*	1	0.17179	0.909137	0.041371
TLN1	4	0.12879	0.981618	0.008058	*SPA17*	4	0.19106	0.909137	0.041371
PSD4	4	0.1609	0.981618	0.008058	*PIP4K2B*	4	0.19437	0.909137	0.041371
GUCY2C	4	0.24267	0.981618	0.008058	*AMZ1*	3	0.23285	0.909137	0.041371
NES	3	0.28542	0.981618	0.008058	*TMEM143*	4	0.2553	0.909137	0.041371
TTLL11	3	0.31555	0.981618	0.008058	*ANKRD27*	4	-0.052332	0.909551	0.041173
WIZ	2	-0.33429	0.981719	0.008013	*DSC2*	2	-0.30534	0.909617	0.041142
HDAC4	4	-0.25021	0.981719	0.008013	*HYAL1*	4	0.025627	0.909617	0.041142
TMEM139	3	-0.19722	0.981719	0.008013	*TNFAIP8L2*	4	0.051424	0.909617	0.041142
MAPK9	4	-0.17678	0.981719	0.008013	*LAMP2*	4	0.17928	0.909617	0.041142
ETFRF1	4	0.072912	0.981719	0.008013	*PHPT1*	4	-0.14485	0.910119	0.040902

GALNTL5	4	0.082843	0.981719	0.008013	*TIRAP*	4	-0.058769	0.910119	0.040902
ABHD13	4	0.12909	0.981719	0.008013	*PPP2R5D*	1	0.22586	0.910288	0.040821
ETV4	3	-0.33393	0.981876	0.007943	*GLRA2*	3	0.019574	0.911163	0.040404
ZNF16	3	0.11719	0.981876	0.007943	*MED29*	4	0.17945	0.911163	0.040404
TBC1D14	3	-0.092558	0.981996	0.00789	*ABCF3*	4	0.21085	0.911163	0.040404
PARN	3	0.21225	0.98236	0.00773	*SLC35D1*	1	0.23117	0.912392	0.039819
TRAM2	3	-0.27959	0.982463	0.007684	*SLFN5*	4	-0.35657	0.912439	0.039796
TMEM191C	1	-0.21006	0.982463	0.007684	*CCDC8*	1	-0.36703	0.912584	0.039727
SNX2	3	-0.20502	0.982463	0.007684	*ATG4D*	2	-0.36346	0.912584	0.039727
DMWD	4	-0.13336	0.982463	0.007684	*RNF122*	3	-0.35852	0.912584	0.039727
LHFPL6	4	-0.06377	0.982463	0.007684	*TRIB1*	2	-0.2358	0.912584	0.039727
SMARCE1	4	0.02983	0.982463	0.007684	*TMEM220*	3	-0.09176	0.912584	0.039727
ZNF324	4	0.16991	0.982664	0.007595	*FBXO22*	3	-0.079987	0.912584	0.039727
AZIN1	2	0.03115	0.982824	0.007524	*CPT1A*	3	-0.064274	0.912584	0.039727
PLCG1	4	-0.16813	0.982958	0.007465	*TCF7*	4	-0.023481	0.912584	0.039727
LYPLA1	3	-0.11939	0.982958	0.007465	*LAMB3*	4	0.092339	0.912584	0.039727
LRRC40	4	-0.11435	0.982958	0.007465	*SHLD1*	4	0.1107	0.912584	0.039727
OXER1	4	-0.018884	0.982958	0.007465	*NUFIP2*	2	0.14273	0.912584	0.039727
TIMM10B	4	0.14621	0.982958	0.007465	*OFD1*	4	0.15653	0.912584	0.039727
USP36	4	0.14819	0.982958	0.007465	*GAB3*	4	0.16128	0.912584	0.039727
CHRNB3	3	0.21313	0.982958	0.007465	*SIX2*	2	0.19387	0.912584	0.039727
BRAT1	3	0.35732	0.982958	0.007465	*CAVIN3*	1	0.21952	0.912584	0.039727
SMC3	4	0.42642	0.982958	0.007465	*RPL6*	1	0.25087	0.912584	0.039727
BTBD17	3	-0.24483	0.983155	0.007378	*NOL7*	2	0.26242	0.912584	0.039727
CTBP2	4	0.26494	0.983155	0.007378	*DEXI*	1	0.30431	0.912584	0.039727
ASF1A	1	-0.36655	0.983548	0.007205	*LRRC75B*	4	-0.26305	0.912642	0.0397
PAK3	4	-0.26365	0.983548	0.007205	*CIART*	4	-0.25917	0.912642	0.0397
FAM214B	4	-0.16818	0.983548	0.007205	*SLC39A2*	4	-0.25165	0.912642	0.0397
PCDHGB2	4	-0.093003	0.983548	0.007205	*ZNF654*	2	-0.2495	0.912642	0.0397
SASS6	4	-0.08983	0.983548	0.007205	*ADCY9*	4	-0.24138	0.912642	0.0397
PNPLA3	3	-0.064026	0.983548	0.007205	*INSL5*	2	-0.2274	0.912642	0.0397
NECTIN4	4	-0.042507	0.983548	0.007205	*ATXN7L2*	1	-0.20048	0.912642	0.0397
SLC43A1	4	0.040833	0.983548	0.007205	*CC2D1B*	4	-0.18204	0.912642	0.0397
LARGE2	4	0.070197	0.983548	0.007205	*MSI2*	4	-0.15334	0.912642	0.0397
BRAF	4	0.10142	0.983548	0.007205	*HPS5*	4	-0.14995	0.912642	0.0397
GNL3	4	0.13677	0.983548	0.007205	*TLR6*	4	-0.13275	0.912642	0.0397
PI16	4	0.14516	0.983548	0.007205	*IRAK3*	4	-0.127	0.912642	0.0397
WDR38	4	0.15269	0.983548	0.007205	*CBLN1*	4	-0.11797	0.912642	0.0397
AANAT	4	0.1844	0.983548	0.007205	*SRGN*	3	-0.095262	0.912642	0.0397
MPC1L	1	0.22933	0.983548	0.007205	*HERC2*	4	-0.081993	0.912642	0.0397
CCDC51	3	0.35829	0.983548	0.007205	*HMGN3*	4	-0.080003	0.912642	0.0397
PAICS	3	0.41134	0.983548	0.007205	*FAM163B*	4	-0.049235	0.912642	0.0397
GPR132	3	-0.31528	0.983814	0.007087	*WHAMM*	4	0.015155	0.912642	0.0397
ZNF431	3	-0.24588	0.983814	0.007087	*STPG1*	4	0.071726	0.912642	0.0397
MME	3	-0.22829	0.983814	0.007087	*AHCYL1*	4	0.082695	0.912642	0.0397
TCTN2	4	-0.20577	0.983814	0.007087	*FCGR2A*	4	0.085003	0.912642	0.0397
NRL	4	-0.12552	0.983814	0.007087	*TTLL6*	4	0.10279	0.912642	0.0397
WLS	4	-0.12405	0.983814	0.007087	*SCRT1*	4	0.12236	0.912642	0.0397
COX15	3	-0.12272	0.983814	0.007087	*FBXO24*	2	0.12501	0.912642	0.0397
RPL4	4	-0.045364	0.983814	0.007087	*EEF1AKNMT*	4	0.12538	0.912642	0.0397
GPR176	4	-0.017643	0.983814	0.007087	*LFNG*	4	0.13911	0.912642	0.0397
RBBP7	3	-0.002124	0.983814	0.007087	*IL6ST*	4	0.20782	0.912642	0.0397
RABEP2	4	0.10841	0.983814	0.007087	*PCDHA2*	4	0.21415	0.912642	0.0397
DTNB	4	0.18133	0.983814	0.007087	*ZNF395*	3	0.21803	0.912642	0.0397
LAYN	4	0.18365	0.983814	0.007087	*PLEKHF2*	2	0.26009	0.912642	0.0397
NDNF	3	0.1841	0.983814	0.007087	*RHO*	4	0.28199	0.912642	0.0397
BCAT1	3	0.1876	0.983814	0.007087	*INHBE*	3	-0.34449	0.91267	0.039686
PSENEN	4	0.1904	0.983814	0.007087	*SNX8*	3	-0.29709	0.91267	0.039686

SMTNL2	4	0.21367	0.983814	0.007087	*POLA2*	4	-0.29033	0.91267	0.039686
S1PR2	4	0.25787	0.983814	0.007087	*SIM2*	4	-0.28609	0.91267	0.039686
ZNF711	4	-0.29752	0.98409	0.006965	*ATRN*	3	-0.24221	0.91267	0.039686
TSSK3	4	-0.24844	0.98409	0.006965	*ADGRB2*	4	-0.12373	0.91267	0.039686
KIF22	2	-0.26594	0.984246	0.006896	*ARSG*	4	-0.11424	0.91267	0.039686
LRRC1	3	-0.1188	0.984246	0.006896	*ASCL3*	2	-0.083807	0.91267	0.039686
C1QTNF3	1	0.1972	0.984283	0.00688	*KHDRBS2*	3	-0.03854	0.91267	0.039686
DLG5	4	0.12202	0.984519	0.006776	*CEACAM16*	4	-0.017398	0.91267	0.039686
ALDOC	4	0.20788	0.984519	0.006776	*ZNF787*	4	-0.002243	0.91267	0.039686
FAM107A	4	-0.10403	0.98488	0.006617	*WWP2*	4	0.051477	0.91267	0.039686
SMAD7	1	-0.30852	0.98491	0.006603	*ITSN1*	4	0.05737	0.91267	0.039686
MAPK12	2	-0.13762	0.98491	0.006603	*PI3*	4	0.07599	0.91267	0.039686
MAPKAPK5	3	0.31525	0.98491	0.006603	*SIGIRR*	4	0.07751	0.91267	0.039686
FAM241A	3	0.0014	0.984947	0.006587	*KDM8*	4	0.10887	0.91267	0.039686
CCND2	4	-0.10556	0.985267	0.006446	*NXT2*	4	0.12682	0.91267	0.039686
H4C3	2	0.056383	0.985385	0.006394	*ADAMTSL1*	4	0.14448	0.91267	0.039686
PLEKHA8	3	-0.32969	0.985445	0.006368	*DUSP8*	4	0.14996	0.91267	0.039686
CXCL16	4	-0.08451	0.985445	0.006368	*PYGO2*	4	0.15658	0.91267	0.039686
GLRX5	4	-0.053257	0.985445	0.006368	*ASMTL*	2	0.1673	0.91267	0.039686
ZNF532	4	-0.008259	0.985445	0.006368	*SETD1B*	4	0.18294	0.91267	0.039686
SPG21	3	0.12501	0.985445	0.006368	*MRC2*	4	0.19872	0.91267	0.039686
TIMM9	4	0.29544	0.985445	0.006368	*CEP85L*	4	0.22789	0.91267	0.039686
ZNF83	3	-0.27473	0.985791	0.006215	*C12orf65*	3	0.31073	0.91267	0.039686
LSG1	4	-0.2052	0.985791	0.006215	*NBPF20*	2	0.33726	0.91267	0.039686
KBTBD6	4	-0.16109	0.985791	0.006215	*FUT11*	3	-0.25648	0.912958	0.039549
USP22	4	-0.14815	0.985791	0.006215	*GNG10*	1	0.20762	0.913529	0.039277
SGK2	4	-0.023134	0.985791	0.006215	*OSBPL10*	4	-0.20131	0.913534	0.039275
MED25	3	0.39242	0.985868	0.006181	*TPST2*	4	-0.2003	0.913534	0.039275
C1orf158	3	-0.30998	0.98608	0.006088	*MACC1*	4	-0.088805	0.913534	0.039275
ZNF841	3	-0.29834	0.98608	0.006088	*RHCE*	4	0.065612	0.913534	0.039275
JUNB	1	-0.29559	0.98608	0.006088	*RNF2*	4	0.1207	0.913534	0.039275
ADIPOR1	3	-0.17976	0.98608	0.006088	*BACH1*	4	0.13249	0.913534	0.039275
FLJ44635	3	-0.15381	0.98608	0.006088	*NXPE3*	4	0.14691	0.913534	0.039275
SPNS3	3	-0.15287	0.98608	0.006088	*MARCKSL1*	4	0.14727	0.913534	0.039275
ABCB1	3	-0.075625	0.98608	0.006088	*PRTG*	4	-0.26606	0.9137	0.039196
ETFDH	4	0.055217	0.98608	0.006088	*INSYN2A*	4	-0.093504	0.9137	0.039196
OPN1SW	4	0.095518	0.98608	0.006088	*TLNRD1*	4	-0.068583	0.9137	0.039196
PANX1	4	0.10144	0.98608	0.006088	*CKAP2*	4	-0.034288	0.9137	0.039196
EPS15L1	4	0.10263	0.98608	0.006088	*MAP1LC3A*	3	-0.033315	0.9137	0.039196
PALLD	4	0.1062	0.98608	0.006088	*PRTN3*	4	-0.01494	0.9137	0.039196
DYNC1I1	4	0.14574	0.98608	0.006088	*STC1*	4	0.018614	0.9137	0.039196
CYP2A7	4	0.19529	0.98608	0.006088	*UBE2H*	4	0.23964	0.9137	0.039196
CFAP97D1	4	0.19594	0.98608	0.006088	*SARM1*	4	0.19479	0.914108	0.039002
ARSD	3	0.25324	0.98608	0.006088	*THNSL1*	4	-0.15416	0.914235	0.038942
TENT2	2	0.27376	0.98608	0.006088	*TXNDC5*	1	-0.28644	0.914327	0.038898
UPP2	2	0.28138	0.98608	0.006088	*MTFR1*	4	-0.2736	0.914327	0.038898
ABLIM2	2	0.28292	0.98608	0.006088	*PPP1R9A*	4	-0.22925	0.914327	0.038898
TDG	3	0.37355	0.98608	0.006088	*CDKN1C*	4	-0.18481	0.914327	0.038898
CTXN1	3	0.024401	0.986315	0.005985	*CAGE1*	4	-0.16389	0.914327	0.038898
MND1	2	0.16291	0.986315	0.005985	*HGSNAT*	4	-0.126	0.914327	0.038898
STAT5B	3	-0.3538	0.986597	0.00586	*TNRC6C*	4	-0.058061	0.914327	0.038898
TMEM50A	4	-0.23764	0.986597	0.00586	*HYLS1*	4	-0.050142	0.914327	0.038898
CHD3	4	-0.22405	0.986597	0.00586	*WDR78*	3	-0.02839	0.914327	0.038898
MTDH	4	-0.19426	0.986597	0.00586	*ID2*	3	0.010638	0.914327	0.038898
EBPL	2	-0.062364	0.986597	0.00586	*NIF3L1*	4	0.011048	0.914327	0.038898
POU3F1	3	0.13573	0.986597	0.00586	*GUCA1C*	4	0.016432	0.914327	0.038898
LRRFIP1	3	0.18122	0.986597	0.00586	*BCAS3*	4	0.049423	0.914327	0.038898
SIX3	2	0.22447	0.986597	0.00586	*CD70*	4	0.059642	0.914327	0.038898

TRAF7	3	0.23708	0.986597	0.00586	*TGM7*	4	0.068251	0.914327	0.038898
PRPF8	2	0.36058	0.986597	0.00586	*HMBOX1*	4	0.077395	0.914327	0.038898
ILF2	4	0.38028	0.986597	0.00586	*NDST1*	3	0.085232	0.914327	0.038898
SOX7	4	0.19891	0.987357	0.005526	*CERT1*	4	0.16468	0.914327	0.038898
WDR45B	4	-0.001613	0.987796	0.005333	*ALDH3B2*	4	0.16591	0.914327	0.038898
EPHX4	3	-0.10369	0.987901	0.005287	*USP1*	3	0.18165	0.914327	0.038898
PDX1	3	0.13262	0.987901	0.005287	*ZDHHC17*	4	0.18445	0.914327	0.038898
SLC16A4	4	0.15557	0.987901	0.005287	*DENND10*	2	0.20199	0.914327	0.038898
DTX3L	3	0.15699	0.987901	0.005287	*CTU1*	3	0.2749	0.914327	0.038898
LHPP	4	-0.29594	0.988094	0.005202	*UBE2G2*	4	0.29624	0.914327	0.038898
TM2D1	3	0.12593	0.988094	0.005202	*IQCG*	3	-0.28779	0.914481	0.038826
KIAA1191	4	-0.24241	0.988192	0.005159	*TTC38*	3	-0.27527	0.914481	0.038826
HINT1	4	-0.23507	0.988192	0.005159	*MYO19*	3	-0.12112	0.914481	0.038826
LIN28B	4	-0.097026	0.988192	0.005159	*PPP2R2A*	3	-0.10032	0.914481	0.038826
MRPL42	4	-0.053954	0.988192	0.005159	*TRAF7*	3	-0.048803	0.914481	0.038826
SMURF2	4	0.003207	0.988192	0.005159	*SLC25A16*	4	0.26312	0.914481	0.038826
SLC35D2	2	0.20106	0.988192	0.005159	*SAXO2*	4	0.07051	0.915088	0.038537
SDF2	4	0.25037	0.988269	0.005125	*PLPP4*	4	-0.10735	0.915339	0.038418
TMC5	3	-0.22085	0.988578	0.004989	*FAM183A*	4	0.068648	0.915339	0.038418
ACSM5	3	-0.004154	0.988578	0.004989	*SLC25A44*	4	-0.047764	0.915345	0.038415
SLC44A3	3	0.25469	0.988578	0.004989	*TFR2*	4	-0.028783	0.915516	0.038334
S100A16	4	0.1603	0.988815	0.004885	*CYP2F1*	4	0.019009	0.915516	0.038334
CCDC25	3	0.1867	0.989008	0.0048	*CPEB3*	4	-0.15029	0.915537	0.038324
LMNTD2	3	0.25328	0.989008	0.0048	*STBD1*	4	0.046753	0.915537	0.038324
STYXL1	4	0.20592	0.989324	0.004661	*HPCAL1*	4	0.19247	0.915537	0.038324
ROMO1	2	-0.57434	0.98961	0.004536	*ZNF354B*	4	0.25488	0.915537	0.038324
CLDN14	2	-0.36275	0.98961	0.004536	*RNF222*	4	0.13343	0.916504	0.037865
DLX4	3	-0.3419	0.98961	0.004536	*CTTNBP2*	4	-0.19644	0.916551	0.037843
CMC1	2	-0.30094	0.98961	0.004536	*CCR1*	4	-0.1087	0.916551	0.037843
ADAM9	3	-0.30011	0.98961	0.004536	*TLE5*	4	-0.030907	0.916551	0.037843
ACTR3C	2	-0.19518	0.98961	0.004536	*SLIRP*	4	-0.013679	0.916551	0.037843
LRP2BP	3	-0.17643	0.98961	0.004536	*SEPTIN14*	4	-0.010329	0.916551	0.037843
SMIM14	4	-0.15347	0.98961	0.004536	*RAB11A*	3	0.067353	0.916551	0.037843
C12orf50	4	-0.13439	0.98961	0.004536	*TPBGL*	4	0.1159	0.916551	0.037843
TMIGD3	4	-0.091899	0.98961	0.004536	*MORC2*	2	0.17055	0.916551	0.037843
SYNE2	4	-0.087939	0.98961	0.004536	*RHOBTB3*	4	0.19937	0.916551	0.037843
DNAH14	3	-8.34E-05	0.98961	0.004536	*OR11H4*	3	0.010769	0.916637	0.037803
PDE12	4	0.016889	0.98961	0.004536	*VCL*	4	0.028088	0.916807	0.037722
ZNF689	4	0.097181	0.98961	0.004536	*N4BP2*	4	0.098816	0.916807	0.037722
IL1RL1	4	0.17516	0.98961	0.004536	*MTRNR2L4*	4	-0.1508	0.916852	0.037701
MYL6B	4	0.21261	0.98961	0.004536	*FRMD4B*	4	-0.33157	0.916988	0.037636
ZC3H18	4	0.26018	0.98961	0.004536	*KLHL30*	2	-0.32495	0.916988	0.037636
PLEC	3	0.26642	0.98961	0.004536	*ARG2*	4	-5.70E-04	0.916988	0.037636
BRF2	3	0.30057	0.98961	0.004536	*FLRT3*	3	-0.28627	0.917073	0.037596
COG8	3	-0.63015	0.99005	0.004343	*GALC*	3	-0.33063	0.917335	0.037472
DGLUCY	3	-0.45411	0.99005	0.004343	*TEPP*	3	-0.30853	0.917335	0.037472
H3C6	1	-0.44284	0.99005	0.004343	*LYSMD4*	3	-0.30604	0.917335	0.037472
OSBPL1A	3	-0.34446	0.99005	0.004343	*SLC26A8*	4	-0.26801	0.917335	0.037472
CCDC58	2	-0.34238	0.99005	0.004343	*GNL1*	3	-0.24814	0.917335	0.037472
RAB2B	3	-0.3416	0.99005	0.004343	*ABHD16B*	3	-0.21821	0.917335	0.037472
GTPBP1	4	-0.33779	0.99005	0.004343	*PERP*	3	-0.2119	0.917335	0.037472
SMYD4	3	-0.33113	0.99005	0.004343	*LIFR*	3	-0.20644	0.917335	0.037472
WDR53	3	-0.31565	0.99005	0.004343	*SRGAP2*	3	-0.17308	0.917335	0.037472
ADAM12	3	-0.31189	0.99005	0.004343	*ADCY1*	4	-0.16453	0.917335	0.037472
GOLPH3	3	-0.29709	0.99005	0.004343	*ADGRE1*	4	-0.080992	0.917335	0.037472
ARL6IP4	4	-0.29541	0.99005	0.004343	*TXNDC11*	4	-0.073456	0.917335	0.037472
C5AR2	3	-0.29458	0.99005	0.004343	*CDKL1*	3	0.025632	0.917335	0.037472
POGK	4	-0.28394	0.99005	0.004343	*CNGB3*	4	0.068672	0.917335	0.037472

SDC4	3	-0.28284	0.99005	0.004343	*RTP4*	4	0.083181	0.917335	0.037472
AMN	3	-0.27882	0.99005	0.004343	*ORMDL1*	4	0.086056	0.917335	0.037472
C1orf21	4	-0.26136	0.99005	0.004343	*CCDC71*	4	0.10193	0.917335	0.037472
TTYH1	4	-0.2606	0.99005	0.004343	*PGBD2*	2	0.14329	0.917335	0.037472
MCCC1	3	-0.25745	0.99005	0.004343	*CSF3R*	4	0.16481	0.917335	0.037472
GRAMD1C	2	-0.24208	0.99005	0.004343	*ZNF595*	4	0.24041	0.917335	0.037472
FAAH2	2	-0.22869	0.99005	0.004343	*NEDD9*	3	-0.33455	0.917505	0.037392
PGAM4	2	-0.21791	0.99005	0.004343	*ZNF670*	3	0.14335	0.917505	0.037392
MEGF11	3	-0.21575	0.99005	0.004343	*GCH1*	4	-0.012158	0.917869	0.037219
PPP2R5E	3	-0.21271	0.99005	0.004343	*ERV3-1*	4	-0.074453	0.917929	0.037191
UIMC1	3	-0.2079	0.99005	0.004343	*SCARA3*	4	0.007758	0.917929	0.037191
CAPZA3	3	-0.20208	0.99005	0.004343	*RNF213*	4	0.099841	0.917929	0.037191
MSL2	4	-0.20124	0.99005	0.004343	*LOC10050638*	2	0.20127	0.917929	0.037191
ZNF548	2	-0.19651	0.99005	0.004343	*IST1*	4	-0.025241	0.918337	0.036998
FAM107B	4	-0.19591	0.99005	0.004343	*CLEC16A*	4	0.034692	0.918337	0.036998
TCEAL8	3	-0.1936	0.99005	0.004343	*ANKRD2*	3	0.051876	0.91874	0.036807
DKK4	3	-0.19083	0.99005	0.004343	*CUEDC1*	4	0.03593	0.919317	0.036534
STARD7	4	-0.18365	0.99005	0.004343	*FAM53B*	4	0.072797	0.919317	0.036534
KCTD19	4	-0.18037	0.99005	0.004343	*ZBTB45*	2	0.18154	0.919317	0.036534
PROC	2	-0.16492	0.99005	0.004343	*PSMB7*	4	0.31051	0.919317	0.036534
KCTD6	3	-0.1633	0.99005	0.004343	*FADS1*	3	-0.29497	0.919322	0.036532
PKDCC	2	-0.15599	0.99005	0.004343	*MTCL1*	4	0.050702	0.919451	0.036471
FFAR4	3	-0.15363	0.99005	0.004343	*ZNF554*	4	0.11084	0.919451	0.036471
PKD2	2	-0.14652	0.99005	0.004343	*ANP32B*	2	-0.29409	0.919456	0.036469
LRRC14B	4	-0.14388	0.99005	0.004343	*ODR4*	3	-0.3839	0.919502	0.036447
TULP4	4	-0.13975	0.99005	0.004343	*METTL9*	3	-0.35906	0.919502	0.036447
HLA-B	4	-0.12923	0.99005	0.004343	*CBWD2*	3	-0.3473	0.919502	0.036447
ZMYND11	3	-0.11505	0.99005	0.004343	*CD79B*	4	-0.28067	0.919502	0.036447
LARP4	4	-0.11486	0.99005	0.004343	*EBP*	4	-0.20925	0.919502	0.036447
ARPP19	2	-0.11124	0.99005	0.004343	*CCDC171*	4	-0.18619	0.919502	0.036447
PTK2B	4	-0.099059	0.99005	0.004343	*PARVA*	2	-0.17604	0.919502	0.036447
FSD1L	3	-0.098136	0.99005	0.004343	*SLC13A5*	4	-0.15803	0.919502	0.036447
IFIT3	3	-0.051728	0.99005	0.004343	*NCKAP5*	3	-0.15674	0.919502	0.036447
TMEM39A	4	-0.038959	0.99005	0.004343	*CPT2*	4	-0.14925	0.919502	0.036447
FOXD4	2	-0.032001	0.99005	0.004343	*PCDHA10*	3	-0.13435	0.919502	0.036447
PHACTR1	2	-0.029238	0.99005	0.004343	*TSC22D3*	3	-0.13427	0.919502	0.036447
SLC35F2	4	-0.021685	0.99005	0.004343	*SRSF4*	4	-0.132	0.919502	0.036447
ZNF100	3	-0.017424	0.99005	0.004343	*FNDC8*	4	-0.058179	0.919502	0.036447
PPM1N	3	0.039367	0.99005	0.004343	*IGFBP6*	3	-0.057414	0.919502	0.036447
GSE1	4	0.055321	0.99005	0.004343	*ZNF829*	3	-0.012523	0.919502	0.036447
TRIM38	4	0.057657	0.99005	0.004343	*APOM*	4	-0.009115	0.919502	0.036447
GLUL	4	0.067742	0.99005	0.004343	*FAM160B2*	3	0.017013	0.919502	0.036447
BCKDHB	4	0.071586	0.99005	0.004343	*TLE1*	4	0.038075	0.919502	0.036447
COMMD5	3	0.085597	0.99005	0.004343	*PPP1R3G*	3	0.057086	0.919502	0.036447
GALK1	3	0.085739	0.99005	0.004343	*PREX1*	4	0.061591	0.919502	0.036447
ZFAND4	4	0.095915	0.99005	0.004343	*PFKFB2*	4	0.071429	0.919502	0.036447
ANKRD22	4	0.10996	0.99005	0.004343	*CYP2C8*	3	0.14292	0.919502	0.036447
DENND4B	4	0.11655	0.99005	0.004343	*ZNF768*	4	0.15146	0.919502	0.036447
RGPD4	2	0.11827	0.99005	0.004343	*SS18L2*	4	0.19406	0.919502	0.036447
PAQR7	4	0.12041	0.99005	0.004343	*VWA8*	3	0.19614	0.919502	0.036447
ZBTB48	3	0.12718	0.99005	0.004343	*EFNB1*	4	-0.16544	0.919745	0.036333
MPLKIP	3	0.13164	0.99005	0.004343	*CAMTA1*	2	0.34946	0.919987	0.036218
HAPLN2	4	0.13917	0.99005	0.004343	*USP17L2*	1	-0.43883	0.920169	0.036132
ZNF781	2	0.14233	0.99005	0.004343	*SCN5A*	2	-0.4259	0.920169	0.036132
ZNF860	4	0.1444	0.99005	0.004343	*VIM*	3	-0.35071	0.920169	0.036132
DDX11	2	0.14444	0.99005	0.004343	*SLC25A11*	4	-0.32518	0.920169	0.036132
TSR2	3	0.15227	0.99005	0.004343	*OTULINL*	4	-0.31941	0.920169	0.036132
RBM5	4	0.15972	0.99005	0.004343	*STEAP3*	2	-0.31186	0.920169	0.036132

ME1	3	0.16596	0.99005	0.004343	*EXTL3*	3	-0.2868	0.920169	0.036132
PRRT4	3	0.16817	0.99005	0.004343	*TAS2R31*	4	-0.26673	0.920169	0.036132
GMPR2	3	0.17304	0.99005	0.004343	*RP9*	4	-0.26613	0.920169	0.036132
ST7L	4	0.17479	0.99005	0.004343	*TTPA*	4	-0.26477	0.920169	0.036132
RYR1	4	0.20402	0.99005	0.004343	*RPGRIP1*	3	-0.26422	0.920169	0.036132
PGM2	3	0.22208	0.99005	0.004343	*TMEM138*	2	-0.23126	0.920169	0.036132
GNA11	3	0.22829	0.99005	0.004343	*DNAJC1*	3	-0.22838	0.920169	0.036132
SLC17A5	4	0.23211	0.99005	0.004343	*SETD9*	4	-0.22339	0.920169	0.036132
WDR83	4	0.23221	0.99005	0.004343	*PXK*	4	-0.22293	0.920169	0.036132
GRIPAP1	4	0.2333	0.99005	0.004343	*CCDC60*	3	-0.19814	0.920169	0.036132
LOC339862	3	0.23347	0.99005	0.004343	*ARHGEF10L*	4	-0.16604	0.920169	0.036132
UBE3B	4	0.23795	0.99005	0.004343	*RLN1*	4	-0.16068	0.920169	0.036132
ELAC2	4	0.23946	0.99005	0.004343	*MPP6*	4	-0.14935	0.920169	0.036132
PAIP1	3	0.24103	0.99005	0.004343	*RAB20*	4	-0.13467	0.920169	0.036132
SCAMP5	3	0.24115	0.99005	0.004343	*MACO1*	2	-0.12374	0.920169	0.036132
BEST2	1	0.2432	0.99005	0.004343	*CCDC93*	4	-0.1013	0.920169	0.036132
PSG1	3	0.24716	0.99005	0.004343	*ANK3*	4	-0.086802	0.920169	0.036132
C2CD3	3	0.24736	0.99005	0.004343	*CLK1*	4	-0.084263	0.920169	0.036132
GALNT9	3	0.24894	0.99005	0.004343	*CSTF2T*	4	-0.081164	0.920169	0.036132
ZER1	4	0.24901	0.99005	0.004343	*FAM50B*	4	-0.056099	0.920169	0.036132
LYAR	2	0.25002	0.99005	0.004343	*SEC11A*	4	-0.045687	0.920169	0.036132
MRPL12	3	0.25116	0.99005	0.004343	*C15orf40*	4	-0.003515	0.920169	0.036132
MLYCD	3	0.25702	0.99005	0.004343	*NTAN1*	4	0.034096	0.920169	0.036132
OAZ3	3	0.25925	0.99005	0.004343	*HIVEP1*	4	0.044575	0.920169	0.036132
PINX1	3	0.26009	0.99005	0.004343	*RBM12B*	4	0.054276	0.920169	0.036132
INTS2	3	0.28077	0.99005	0.004343	*CAST*	4	0.058096	0.920169	0.036132
CHD8	3	0.3252	0.99005	0.004343	*FAM53A*	4	0.064631	0.920169	0.036132
ERAS	2	0.34722	0.99005	0.004343	*THEGL*	4	0.064767	0.920169	0.036132
CLEC4F	3	-0.29555	0.990189	0.004282	*SDHAF3*	3	0.069452	0.920169	0.036132
MAGEE1	4	-0.25088	0.990189	0.004282	*TCTE3*	4	0.077595	0.920169	0.036132
RPS5	3	-0.051836	0.990189	0.004282	*ANKRD12*	3	0.082957	0.920169	0.036132
CCDC34	3	0.1745	0.990189	0.004282	*CEP295NL*	4	0.11415	0.920169	0.036132
SUCLG2	3	0.19173	0.990189	0.004282	*NR4A2*	4	0.14209	0.920169	0.036132
GATM	4	-0.26148	0.99052	0.004137	*PKIG*	4	0.14734	0.920169	0.036132
LPCAT1	3	-0.21883	0.99052	0.004137	*EMB*	3	0.15864	0.920169	0.036132
MAPRE3	4	-0.17326	0.99052	0.004137	*USP51*	4	0.17865	0.920169	0.036132
GALNT18	3	-0.15938	0.99052	0.004137	*EFNA4*	2	0.20756	0.920169	0.036132
ERCC6	7	0.054503	0.99052	0.004137	*DHTKD1*	4	0.22908	0.920169	0.036132
KIF13A	3	0.16779	0.99052	0.004137	*NEK8*	4	0.23092	0.920169	0.036132
CDC42BPB	3	-0.4392	0.991052	0.003904	*CTNNA1*	2	0.23716	0.920169	0.036132
RBPMS2	3	-0.093782	0.991052	0.003904	*SUMO4*	4	0.25724	0.920169	0.036132
ZNF786	4	-0.005208	0.991052	0.003904	*TMEM11*	4	0.19077	0.920568	0.035944
GADD45B	4	0.085119	0.991052	0.003904	*PRDM16*	3	-0.37138	0.921066	0.035709
LGR5	4	0.15344	0.991052	0.003904	*AGGF1*	3	-0.3028	0.921066	0.035709
MAN1A2	3	-0.32736	0.991178	0.003848	*FXYD3*	3	-0.29778	0.921066	0.035709
LAT	1	-0.31305	0.991178	0.003848	*SPCS1*	3	-0.29764	0.921066	0.035709
NOTUM	3	-0.3065	0.991178	0.003848	*PLXNA1*	4	-0.28079	0.921066	0.035709
POU5F1	3	-0.29358	0.991178	0.003848	*NPBWR1*	3	-0.26691	0.921066	0.035709
CYP4F3	4	-0.29328	0.991178	0.003848	*GSTM1*	3	-0.22295	0.921066	0.035709
ANO8	2	-0.25487	0.991178	0.003848	*NSUN5*	4	-0.2172	0.921066	0.035709
RNF144B	4	-0.24879	0.991178	0.003848	*IL17F*	4	-0.18366	0.921066	0.035709
SSH1	3	-0.23946	0.991178	0.003848	*SIRT7*	3	-0.17775	0.921066	0.035709
KLHDC8A	3	-0.23745	0.991178	0.003848	*LSM8*	4	0.044096	0.921066	0.035709
ARL14EP	4	-0.23679	0.991178	0.003848	*CASP10*	3	0.098936	0.921066	0.035709
IRS2	4	-0.23677	0.991178	0.003848	*GTF2E2*	4	0.11597	0.921066	0.035709
WBP11	4	-0.23546	0.991178	0.003848	*PIK3IP1*	3	0.14134	0.921066	0.035709
C20orf194	3	-0.23145	0.991178	0.003848	*IFITM2*	4	-0.21816	0.921093	0.035696
OPA1	3	-0.23107	0.991178	0.003848	*LPP*	4	-0.17751	0.921093	0.035696

OTUD4	3	-0.21398	0.991178	0.003848	*CCDC82*	3	-0.16167	0.921093	0.035696
IL33	2	-0.20109	0.991178	0.003848	*ZNF528*	4	0.061313	0.921093	0.035696
CERCAM	4	-0.20006	0.991178	0.003848	*CD7*	3	0.15097	0.921093	0.035696
PCDH1	4	-0.18371	0.991178	0.003848	*ZNF696*	4	0.15856	0.921093	0.035696
CCNJ	3	-0.1745	0.991178	0.003848	*FBXW8*	3	-0.28445	0.921098	0.035694
MPST	3	-0.17394	0.991178	0.003848	*NR2F1*	4	-0.12227	0.921225	0.035634
LRFN4	3	-0.17234	0.991178	0.003848	*TMCO1*	3	-0.001331	0.921225	0.035634
KLHL6	4	-0.16519	0.991178	0.003848	*H4C2*	3	-0.24785	0.921396	0.035554
KLHL8	4	-0.16231	0.991178	0.003848	*ADSS1*	3	-0.23632	0.921396	0.035554
C12orf45	4	-0.13934	0.991178	0.003848	*NHSL2*	4	0.039707	0.921396	0.035554
FICD	4	-0.13578	0.991178	0.003848	*CIB4*	4	-0.24761	0.921409	0.035547
SMAD6	2	-0.13531	0.991178	0.003848	*LINC01750*	4	-0.17579	0.921409	0.035547
ZNHIT1	3	-0.10611	0.991178	0.003848	*TEKT3*	3	0.16078	0.921409	0.035547
MKRN2	4	-0.097581	0.991178	0.003848	*POLN*	4	0.017037	0.921532	0.03549
ZFP64	4	-0.067658	0.991178	0.003848	*OGFR*	3	-0.23083	0.921536	0.035488
MAP3K19	4	-0.058099	0.991178	0.003848	*BTBD9*	3	-0.30683	0.921651	0.035434
PRRX1	4	-0.043241	0.991178	0.003848	*CTTN*	4	-0.27455	0.921651	0.035434
OTUD5	4	-0.04263	0.991178	0.003848	*NOD1*	4	-0.0962	0.921651	0.035434
CLEC2B	3	-0.019098	0.991178	0.003848	*BCLAF1*	2	-0.008463	0.921651	0.035434
GSTA2	4	-0.015891	0.991178	0.003848	*PARD3*	4	-0.003137	0.921651	0.035434
SCARA3	4	-0.014424	0.991178	0.003848	*DSE*	4	0.095435	0.921651	0.035434
TMEM35A	4	0.01266	0.991178	0.003848	*SP140L*	4	0.10998	0.921651	0.035434
CCT8	4	0.019305	0.991178	0.003848	*AHNAK*	2	0.24086	0.921651	0.035434
KPNA6	4	0.082607	0.991178	0.003848	*GK*	4	-0.24581	0.922056	0.035243
USP43	4	0.09912	0.991178	0.003848	*OPLAH*	4	-0.22435	0.922056	0.035243
FAM183A	4	0.10447	0.991178	0.003848	*ERCC6*	7	0.15364	0.922056	0.035243
PUM1	4	0.16223	0.991178	0.003848	*TMEM263*	3	0.13642	0.922257	0.035148
GANAB	4	0.1636	0.991178	0.003848	*CLASRP*	3	-0.11444	0.923054	0.034773
MRVI1	2	0.17689	0.991178	0.003848	*FAM81A*	3	-0.008664	0.923054	0.034773
KRTAP5-6	3	0.17813	0.991178	0.003848	*FLOT2*	3	0.14611	0.923054	0.034773
FAM193B	4	0.19408	0.991178	0.003848	*HOXA9*	4	-0.2179	0.923219	0.034695
MROH8	3	0.19773	0.991178	0.003848	*MTDH*	4	-0.10344	0.923219	0.034695
LRRFIP2	3	0.20503	0.991178	0.003848	*NEK1*	4	-0.078954	0.923315	0.03465
SAV1	3	0.21969	0.991178	0.003848	*PIGV*	4	0.094179	0.923315	0.03465
FOXJ2	1	0.22668	0.991178	0.003848	*AMDHD2*	4	0.12134	0.923315	0.03465
TCEA3	4	0.2318	0.991178	0.003848	*TMEM132B*	4	0.16413	0.923315	0.03465
ATG14	4	0.24632	0.991178	0.003848	*AGRN*	4	-0.25353	0.923649	0.034493
DDX17	4	0.25741	0.991178	0.003848	*FCAR*	4	-0.2113	0.923649	0.034493
SUPT3H	3	0.2646	0.991178	0.003848	*CD83*	4	-0.18333	0.923649	0.034493
METTL26	3	0.31152	0.991178	0.003848	*TMEM179*	4	-0.12322	0.923649	0.034493
PWP2	1	0.32173	0.991178	0.003848	*CLEC18A*	2	0.28695	0.923649	0.034493
USP47	2	0.33933	0.991178	0.003848	*ZNF708*	2	-0.41734	0.92381	0.034417
EDA	2	-0.47543	0.991213	0.003833	*FOLR3*	2	-0.01507	0.923932	0.03436
UNKL	1	0.40739	0.991483	0.003715	*USHBP1*	3	-0.19912	0.924058	0.034301
EIF4ENIF1	4	-0.39421	0.991588	0.003669	*PAXX*	3	0.23225	0.924058	0.034301
SMS	2	0.12026	0.991588	0.003669	*MBP*	4	-0.052377	0.924183	0.034242
CX3CR1	2	0.18863	0.991588	0.003669	*SARAF*	4	-0.036365	0.924183	0.034242
RIMS2	4	-0.27994	0.991646	0.003643	*GFPT2*	3	-0.25539	0.924313	0.034181
RAP1GAP2	4	-0.096694	0.991646	0.003643	*ZDHHC6*	4	-0.23704	0.924313	0.034181
CCL25	4	-0.005755	0.991646	0.003643	*NR1D1*	4	0.053523	0.924313	0.034181
GTF2A1	4	0.12595	0.991646	0.003643	*EXD2*	2	-0.20962	0.924865	0.033922
ALDH1L2	4	0.28966	0.991646	0.003643	*HAUS8*	4	-0.3983	0.925585	0.033584
ANO4	4	-0.067808	0.991751	0.003597	*DMAC2L*	4	-0.19481	0.925585	0.033584
JKAMP	4	0.19491	0.991751	0.003597	*ADGRB3*	3	-0.039862	0.925585	0.033584
ZNF202	3	0.2178	0.991751	0.003597	*CYBC1*	3	0.14155	0.925585	0.033584
ATP6V1C2	3	-0.30441	0.991981	0.003497	*ARMCX5*	2	-0.27466	0.925745	0.033509
BLOC1S4	4	-0.27951	0.992244	0.003382	*SETMAR*	3	-0.17674	0.926261	0.033267
SHOC1	3	-0.2755	0.992244	0.003382	*ARHGEF9*	4	-0.15778	0.926261	0.033267

FAM207A	3	-0.2634	0.992244	0.003382	*RECQL5*	1	0.25264	0.926343	0.033228
PRR34-AS1	1	-0.23163	0.992244	0.003382	*CSRP2*	4	-0.37746	0.926712	0.033055
SDF4	4	-0.12224	0.992244	0.003382	*ADRB1*	3	-0.32968	0.926712	0.033055
HMGA2-AS1	4	-0.070832	0.992244	0.003382	*PRRG4*	4	-0.27178	0.926712	0.033055
RNF4	4	-0.025857	0.992244	0.003382	*CDC25B*	2	-0.22642	0.926712	0.033055
KALRN	4	0.00771	0.992244	0.003382	*MPST*	3	-0.22621	0.926712	0.033055
TRIM67	4	0.018595	0.992244	0.003382	*ACSS1*	3	-0.20449	0.926712	0.033055
GPR68	4	0.070995	0.992244	0.003382	*DIPK1A*	4	-0.19994	0.926712	0.033055
SLC35A5	4	0.12908	0.992244	0.003382	*FBLN1*	4	-0.19485	0.926712	0.033055
VEZT	2	0.33685	0.992244	0.003382	*ITGB1BP2*	2	-0.18911	0.926712	0.033055
NUDT11	3	-0.1893	0.992825	0.003127	*C17orf99*	3	-0.18641	0.926712	0.033055
WRAP53	2	-0.50553	0.993223	0.002953	*XAGE3*	3	-0.17815	0.926712	0.033055
PIGA	2	-0.39766	0.993223	0.002953	*ZNF276*	2	-0.15946	0.926712	0.033055
EVX1	3	-0.35172	0.993223	0.002953	*INKA2*	4	-0.1445	0.926712	0.033055
PRR3	3	-0.32129	0.993223	0.002953	*JMY*	4	-0.10825	0.926712	0.033055
CYP2E1	4	-0.31349	0.993223	0.002953	*GSTA4*	4	0.002287	0.926712	0.033055
COX6C	4	-0.28195	0.993223	0.002953	*TAOK1*	4	0.014252	0.926712	0.033055
RDH16	4	-0.23742	0.993223	0.002953	*ARMC1*	4	0.044137	0.926712	0.033055
ZNF704	4	-0.23209	0.993223	0.002953	*SMARCD1*	4	0.07551	0.926712	0.033055
SLC39A13	4	-0.22971	0.993223	0.002953	*ZBTB47*	4	0.10016	0.926712	0.033055
DCTPP1	4	-0.22479	0.993223	0.002953	*TSTD2*	4	0.11871	0.926712	0.033055
TMEM273	4	-0.22306	0.993223	0.002953	*ARL14*	4	0.12571	0.926712	0.033055
RASAL2	4	-0.22104	0.993223	0.002953	*OR10A3*	4	0.16394	0.926712	0.033055
CXCL13	4	-0.21658	0.993223	0.002953	*SLC17A7*	4	0.18094	0.926712	0.033055
NACA2	3	-0.21212	0.993223	0.002953	*CHCHD2*	1	0.2223	0.926712	0.033055
RGS22	4	-0.20924	0.993223	0.002953	*PSMA1*	4	0.27888	0.926712	0.033055
TTPAL	4	-0.20627	0.993223	0.002953	*AQP1*	3	-0.34931	0.927008	0.032917
COL14A1	4	-0.19819	0.993223	0.002953	*BAAT*	3	-0.29151	0.927008	0.032917
ZNF587B	2	-0.19447	0.993223	0.002953	*CCDC146*	3	-0.25702	0.927008	0.032917
IRF7	4	-0.19191	0.993223	0.002953	*CHN2*	4	-0.15124	0.927008	0.032917
DNAH3	3	-0.19043	0.993223	0.002953	*PAOX*	3	-0.10524	0.927008	0.032917
MESP2	4	-0.17822	0.993223	0.002953	*PTGFRN*	3	0.13472	0.927008	0.032917
NOTO	3	-0.17749	0.993223	0.002953	*SMOC1*	4	-0.20969	0.927143	0.032853
PMS2	4	-0.17188	0.993223	0.002953	*SLC2A5*	4	-0.19323	0.927143	0.032853
C9orf78	4	-0.15985	0.993223	0.002953	*CHRNB2*	3	-0.1575	0.927143	0.032853
MYO6	4	-0.14987	0.993223	0.002953	*KIF20B*	3	0.12158	0.927143	0.032853
OR1F1	4	-0.14581	0.993223	0.002953	*SART3*	3	0.16614	0.927143	0.032853
ZDHHC2	4	-0.1264	0.993223	0.002953	*GTF2F2*	2	-0.073425	0.927769	0.03256
ST3GAL6	4	-0.11144	0.993223	0.002953	*IL13RA2*	4	-0.212	0.927854	0.03252
RIPK1	4	-0.091156	0.993223	0.002953	*TLX3*	4	-0.082022	0.927854	0.03252
MAT1A	4	-0.083091	0.993223	0.002953	*LRRC10B*	3	-0.28578	0.927858	0.032519
SLITRK6	4	-0.08066	0.993223	0.002953	*SELENOM*	2	-0.25402	0.927858	0.032519
TRMT2A	4	-0.077782	0.993223	0.002953	*ACTN1*	3	-0.19861	0.927858	0.032519
RPL24	2	-0.075903	0.993223	0.002953	*RREB1*	2	-0.18811	0.927858	0.032519
PCCA	4	-0.073434	0.993223	0.002953	*TRIM55*	2	-0.1218	0.927858	0.032519
ANKRD13C	4	-0.072594	0.993223	0.002953	*CXCL3*	3	-0.096657	0.927858	0.032519
SST	4	-0.071778	0.993223	0.002953	*PSTK*	4	-0.019595	0.927858	0.032519
CHCHD10	4	-0.062368	0.993223	0.002953	*FOXB1*	4	0.039977	0.927858	0.032519
FKBPL	4	-0.060828	0.993223	0.002953	*TRMT1L*	4	0.04248	0.927858	0.032519
LMO4	4	-0.059117	0.993223	0.002953	*BTBD1*	3	0.044255	0.927858	0.032519
IFFO1	2	-0.039957	0.993223	0.002953	*INPP5B*	3	0.068578	0.927858	0.032519
ZMYND19	4	-0.033769	0.993223	0.002953	*THAP2*	3	0.079501	0.927858	0.032519
HAPLN3	3	-0.028576	0.993223	0.002953	*PRIMA1*	4	0.085699	0.927858	0.032519
DNAH1	4	-0.024288	0.993223	0.002953	*VWF*	4	0.086747	0.927858	0.032519
VAV3	4	-0.024185	0.993223	0.002953	*CCDC63*	4	0.094507	0.927858	0.032519
ZBTB41	4	-0.016777	0.993223	0.002953	*RDX*	4	0.099875	0.927858	0.032519
H2AC20	3	0.003393	0.993223	0.002953	*IMPACT*	4	0.12577	0.927858	0.032519
GPR45	4	0.015554	0.993223	0.002953	*MYH14*	3	0.16688	0.927858	0.032519

DLG2	4	0.059038	0.993223	0.002953	*BARX2*	4	0.17625	0.927858	0.032519
VPS37C	4	0.085464	0.993223	0.002953	*APOC1*	2	0.19496	0.927858	0.032519
MAN2A1	4	0.090232	0.993223	0.002953	*SATB1*	3	0.20194	0.927858	0.032519
TRIM50	4	0.111	0.993223	0.002953	*RNF8*	4	0.29181	0.927858	0.032519
CH25H	3	0.11337	0.993223	0.002953	*TYW1*	4	0.12008	0.928171	0.032372
HOXC5	4	0.13198	0.993223	0.002953	*ATF5*	3	-0.2758	0.928369	0.032279
UBE2QL1	4	0.15554	0.993223	0.002953	*KLF7*	4	0.14883	0.928566	0.032187
OC10028703	4	0.15869	0.993223	0.002953	*SERPINB5*	3	-0.089165	0.928957	0.032004
GOLGA6A	1	0.16875	0.993223	0.002953	*LRRC49*	2	-0.4964	0.929046	0.031963
KLHL24	3	0.17115	0.993223	0.002953	*DNAJC16*	3	-0.43925	0.929046	0.031963
PHLDB1	3	0.17373	0.993223	0.002953	*C21orf62*	3	-0.37742	0.929046	0.031963
SETBP1	4	0.1783	0.993223	0.002953	*POGLUT1*	3	-0.31505	0.929046	0.031963
PCDHGA7	3	0.18581	0.993223	0.002953	*SPIN4*	3	-0.31123	0.929046	0.031963
SLC29A1	3	0.20242	0.993223	0.002953	*HOXC11*	4	-0.29532	0.929046	0.031963
ZYG11A	4	0.21292	0.993223	0.002953	*FUT6*	3	-0.29345	0.929046	0.031963
GRAMD2A	3	0.2145	0.993223	0.002953	*DMPK*	3	-0.28575	0.929046	0.031963
SRP9	1	0.21863	0.993223	0.002953	*COQ10A*	3	-0.28034	0.929046	0.031963
ZSWIM8	3	0.22299	0.993223	0.002953	*ACVR1C*	4	-0.25074	0.929046	0.031963
TMTC3	3	0.25789	0.993223	0.002953	*PFN2*	4	-0.23265	0.929046	0.031963
PHACTR3	4	0.26739	0.993223	0.002953	*PYROXD2*	3	-0.23148	0.929046	0.031963
PUM2	3	0.31581	0.993223	0.002953	*TMEM9*	4	-0.1785	0.929046	0.031963
SRSF1	4	0.38353	0.993223	0.002953	*FOXN4*	4	-0.16443	0.929046	0.031963
HEXIM1	3	0.3882	0.993223	0.002953	*CYS1*	4	-0.1615	0.929046	0.031963
PSMD7	3	0.45247	0.993223	0.002953	*PYM1*	4	-0.13981	0.929046	0.031963
SSTR5	4	-0.19066	0.993258	0.002938	*MYT1*	4	-0.10875	0.929046	0.031963
ETHE1	3	-0.16559	0.993258	0.002938	*SHE*	4	-0.10713	0.929046	0.031963
RNF222	4	-0.074459	0.993258	0.002938	*CHPT1*	4	-0.090296	0.929046	0.031963
TIPRL	3	-0.023134	0.993258	0.002938	*ARMC2*	4	-0.067889	0.929046	0.031963
GLP2R	4	-0.003785	0.993258	0.002938	*SLC16A4*	4	-0.042444	0.929046	0.031963
ANKRD35	4	0.10302	0.993258	0.002938	*KCTD10*	4	-0.036964	0.929046	0.031963
BAHCC1	4	0.12168	0.993258	0.002938	*PLA2G2D*	4	-0.034421	0.929046	0.031963
ABCF3	4	0.12537	0.993258	0.002938	*ARMCX3*	4	0.005674	0.929046	0.031963
PIM2	4	0.14564	0.993258	0.002938	*DTNA*	3	0.011521	0.929046	0.031963
KREMEN1	3	0.16741	0.993258	0.002938	*MAMDC4*	2	0.027326	0.929046	0.031963
GAR1	4	0.16865	0.993258	0.002938	*KLHL32*	4	0.035212	0.929046	0.031963
RGS16	1	0.18244	0.993258	0.002938	*PPIH*	3	0.053295	0.929046	0.031963
KRT16	4	0.18973	0.993258	0.002938	*IL27RA*	4	0.12258	0.929046	0.031963
INTS12	4	0.21763	0.993258	0.002938	*FAM118B*	4	0.18438	0.929046	0.031963
AP4M1	3	0.21844	0.993258	0.002938	*KNOP1*	4	0.18782	0.929046	0.031963
TNFSF15	2	0.22313	0.993258	0.002938	*UHRF1*	3	0.19914	0.929046	0.031963
LIMK1	3	0.2612	0.993258	0.002938	*SERPINB9*	3	0.22112	0.929046	0.031963
EMC3	8	0.26161	0.993258	0.002938	*TUBD1*	3	0.23879	0.929046	0.031963
MRPL37	3	0.28331	0.993258	0.002938	*MPZL2*	2	0.28623	0.929046	0.031963
CLDN7	4	-0.2611	0.993952	0.002635	*CNN1*	4	0.29172	0.929046	0.031963
H4C13	4	-0.24695	0.993952	0.002635	*C1QTNF2*	3	-0.26123	0.929063	0.031955
CA4	4	-0.16158	0.993952	0.002635	*FASTKD1*	4	0.042248	0.929063	0.031955
ABI3	4	-0.094791	0.993952	0.002635	*PDCD5*	4	0.12565	0.929063	0.031955
SLC4A9	2	-0.084899	0.993952	0.002635	*HACD4*	4	0.15663	0.929063	0.031955
PLEKHA5	4	-0.036692	0.993952	0.002635	*GPX7*	4	0.17823	0.929063	0.031955
GOT1	3	-0.019473	0.993952	0.002635	*EIF4G3*	3	-0.10002	0.929186	0.031897
LURAP1	4	0.077284	0.993952	0.002635	*CEP41*	3	-0.006868	0.929186	0.031897
UBR7	4	0.12041	0.993952	0.002635	*RAP1B*	2	-0.018885	0.929344	0.031824
PPCS	3	-0.16448	0.994046	0.002593	*FAM89B*	3	-0.15238	0.929698	0.031658
FOXE3	4	0.12617	0.994046	0.002593	*GOLM1*	3	0.091071	0.929698	0.031658
ZNF398	3	0.13402	0.994046	0.002593	*SDC4*	3	-0.26254	0.929924	0.031552
ZP3	3	0.20199	0.994046	0.002593	*TERB1*	4	-0.15106	0.929924	0.031552
DNAJA1	4	0.28348	0.994046	0.002593	*FKBP8*	4	-0.11902	0.929924	0.031552
EID1	4	0.15615	0.994234	0.002511	*CFAP65*	4	-0.014017	0.929924	0.031552

PADI3	3	-0.24575	0.994242	0.002508	*MLF1*	4	-0.004062	0.929924	0.031552
KIAA2013	4	-0.23811	0.994242	0.002508	*WBP2NL*	4	0.008376	0.929924	0.031552
CHST3	4	-0.19247	0.994242	0.002508	*ADGRB1*	4	0.02675	0.929924	0.031552
MMP15	4	-0.18445	0.994242	0.002508	*RGP1*	3	0.17506	0.929924	0.031552
IL1RAP	3	-0.13228	0.994242	0.002508	*XPO5*	4	0.21108	0.929924	0.031552
ACADM	4	-0.1291	0.994242	0.002508	*FSD1*	4	0.21726	0.929924	0.031552
TMEM186	4	-0.11124	0.994242	0.002508	*WDR4*	3	-0.27644	0.930171	0.031437
LY96	4	-0.091589	0.994242	0.002508	*ZNF283*	3	-0.18326	0.930171	0.031437
ABCA3	3	0.058388	0.994242	0.002508	*HSPB2*	3	-0.17691	0.930171	0.031437
ERFE	4	0.078064	0.994242	0.002508	*DNAJB8*	3	-0.1625	0.930171	0.031437
PANO1	4	0.13948	0.994242	0.002508	*KL*	4	-0.16043	0.930171	0.031437
ULK1	4	0.14305	0.994242	0.002508	*ELMSAN1*	4	-0.13658	0.930171	0.031437
RPS6KB1	4	0.15183	0.994242	0.002508	*CATSPER1*	2	-0.058186	0.930171	0.031437
ABHD14B	4	0.17851	0.994242	0.002508	*CXCL5*	4	-0.006907	0.930171	0.031437
MYH7	4	0.24573	0.994242	0.002508	*PDCL3*	3	0.030857	0.930171	0.031437
PLEKHA3	1	0.26926	0.994242	0.002508	*ZFPL1*	4	0.034169	0.930171	0.031437
DPP9	4	-0.071563	0.994348	0.002461	*KNG1*	2	0.083337	0.930171	0.031437
CAPS	3	0.07917	0.994348	0.002461	*RNF170*	4	0.14335	0.930171	0.031437
NDUFB5	3	-0.12673	0.994489	0.0024	*MEAF6*	3	0.16294	0.930171	0.031437
UPK1A	4	0.10229	0.994489	0.0024	*SERINC3*	4	0.31503	0.930171	0.031437
GLS2	2	0.26294	0.994489	0.0024	*C2orf49*	4	0.32561	0.930171	0.031437
GTPBP10	4	-0.3494	0.994596	0.002353	*SDR42E1*	1	0.34631	0.930171	0.031437
PML	4	-0.15417	0.994596	0.002353	*CELSR1*	3	-0.35432	0.930219	0.031415
SNX31	3	-0.10054	0.995111	0.002128	*ZNF343*	3	-0.20408	0.930219	0.031415
SLC4A1AP	4	-0.029447	0.995111	0.002128	*ARHGEF19*	3	0.084042	0.930219	0.031415
SDHA	4	0.060725	0.995111	0.002128	*ZNF625*	4	0.22332	0.930799	0.031144
SLC4A3	4	0.13701	0.995111	0.002128	*CDK16*	4	0.10552	0.930994	0.031053
RBBP6	4	0.24716	0.995111	0.002128	*POP1*	4	0.23038	0.931112	0.030998
TTC33	3	-0.11526	0.995145	0.002114	*DGKA*	3	-0.32382	0.931125	0.030992
NOTCH3	4	0.079534	0.995333	0.002032	*AMDHD1*	4	0.009688	0.931125	0.030992
MFSD1	3	-0.39309	0.995673	0.001883	*TRAF3IP3*	4	0.17049	0.931125	0.030992
PPTC7	3	-0.12727	0.996005	0.001738	*FAM168A*	4	0.23777	0.931125	0.030992
EIF4EBP3	3	-0.114	0.996005	0.001738	*PCSK9*	3	-0.44783	0.931244	0.030937
ARX	3	0.11038	0.996005	0.001738	*TCHH*	2	-0.32562	0.931288	0.030916
SPATA18	4	-0.31275	0.996035	0.001725	*PAX5*	2	-0.035648	0.931288	0.030916
OR52B2	4	-0.30106	0.996035	0.001725	*MIB2*	3	-0.31875	0.93202	0.030575
TNFRSF10D	3	-0.2765	0.996035	0.001725	*PPP1R1B*	4	-0.10946	0.932714	0.030252
ERVMER34-1	4	-0.26192	0.996035	0.001725	*RAB34*	8	0.075706	0.933139	0.030054
ABL2	4	-0.25608	0.996035	0.001725	*ZSWIM8*	3	-0.19638	0.933148	0.03005
POLR2D	3	-0.24682	0.996035	0.001725	*FAM24B*	3	0.11197	0.933148	0.03005
PCDHGA5	4	-0.245	0.996035	0.001725	*METAP2*	3	0.24828	0.933148	0.03005
NCOA6	4	-0.21219	0.996035	0.001725	*ZNF692*	3	-0.15683	0.934035	0.029637
TRIM71	4	-0.20863	0.996035	0.001725	*TRIB2*	4	0.16517	0.934035	0.029637
DIRAS1	2	-0.20652	0.996035	0.001725	*ZG16B*	3	-0.13325	0.934162	0.029578
PRKX	2	-0.19187	0.996035	0.001725	*LRRC56*	3	8.82E-05	0.934162	0.029578
AXL	3	-0.19041	0.996035	0.001725	*RASAL2*	4	0.050854	0.934162	0.029578
STK25	3	-0.17953	0.996035	0.001725	*CMTR1*	4	0.20188	0.934162	0.029578
PDCL2	4	-0.1783	0.996035	0.001725	*LY6G5C*	4	-0.33655	0.934827	0.029269
ZNF880	3	-0.1569	0.996035	0.001725	*PRR19*	4	-0.1757	0.934827	0.029269
IL21R	4	-0.14735	0.996035	0.001725	*SYN3*	4	-0.12846	0.934827	0.029269
CFI	4	-0.13766	0.996035	0.001725	*KLHL31*	4	-0.10125	0.934827	0.029269
RNF19B	4	-0.10593	0.996035	0.001725	*LBX2*	4	0.19273	0.934827	0.029269
TMEM179	4	-0.099496	0.996035	0.001725	*ITGAM*	3	-0.29049	0.935062	0.02916
PARP8	4	-0.098627	0.996035	0.001725	*ZFP41*	4	0.086312	0.935062	0.02916
CRTAC1	4	-0.096479	0.996035	0.001725	*SERPINE2*	4	-0.23351	0.935128	0.029129
PLA2G7	4	-0.073922	0.996035	0.001725	*CAMK2N2*	4	-0.22764	0.935128	0.029129
TNNI2	3	-0.073242	0.996035	0.001725	*YIPF1*	4	-0.22445	0.935128	0.029129
APOBEC3G	4	-0.072438	0.996035	0.001725	*THY1*	4	-0.20797	0.935128	0.029129

INTS9	4	-0.059106	0.996035	0.001725	*PTPRCAP*	3	-0.16552	0.935128	0.029129
KIF1A	3	-0.05055	0.996035	0.001725	*QKI*	4	0.02174	0.935128	0.029129
TEX15	4	0.044345	0.996035	0.001725	*FAM166A*	4	0.033139	0.935128	0.029129
SMAD9	4	0.052196	0.996035	0.001725	*OGDH*	4	0.039971	0.935128	0.029129
ZGRF1	3	0.084409	0.996035	0.001725	*FGF1*	4	0.055133	0.935128	0.029129
NOSTRIN	4	0.087334	0.996035	0.001725	*POU6F1*	4	0.13681	0.935128	0.029129
SLC7A9	3	0.093749	0.996035	0.001725	*TUBGCP5*	4	0.20121	0.935322	0.029039
WEE2	3	0.11892	0.996035	0.001725	*TMEM211*	3	-0.031595	0.935477	0.028967
MKNK2	4	0.14295	0.996035	0.001725	*OLFML2A*	3	-0.28889	0.935684	0.028871
SYCE2	3	0.14811	0.996035	0.001725	*RYR1*	4	-0.28548	0.935684	0.028871
DHRS11	4	0.16306	0.996035	0.001725	*CLEC7A*	4	-0.13597	0.935684	0.028871
WRNIP1	2	0.16795	0.996035	0.001725	*GJC2*	4	-0.097293	0.935684	0.028871
PGBD2	2	0.19081	0.996035	0.001725	*SIGLEC9*	4	-0.09233	0.935684	0.028871
TAPBPL	4	0.19389	0.996035	0.001725	*KIAA0586*	4	0.02704	0.935684	0.028871
AGTRAP	2	0.21898	0.996035	0.001725	*ASAP1*	3	-0.10712	0.935878	0.028781
SLC6A16	2	0.23312	0.996035	0.001725	*MED25*	3	0.12876	0.936303	0.028583
CEBPZ	3	0.24914	0.996035	0.001725	*PCDHB11*	3	0.19485	0.936303	0.028583
ZNF283	3	0.25219	0.996035	0.001725	*MAL*	4	-0.11777	0.937031	0.028246
SYNM	1	0.25371	0.996035	0.001725	*ENTPD6*	2	-0.35916	0.937797	0.027891
TRPV4	4	0.25648	0.996035	0.001725	*ZNF28*	4	-0.17061	0.938036	0.027781
CFAP58	4	-0.31397	0.996504	0.001521	*CDIP1*	2	-0.15623	0.938036	0.027781
C2orf50	4	-0.16662	0.996504	0.001521	*PHF2*	4	-0.019279	0.938036	0.027781
TRAF3IP2	4	-0.080652	0.996504	0.001521	*BCL2A1*	4	-0.013125	0.938036	0.027781
GPR15	4	-0.063439	0.996504	0.001521	*CCND3*	4	-0.30687	0.938079	0.027761
BCL2L13	3	0.085879	0.996504	0.001521	*MPL*	4	-0.13407	0.938079	0.027761
RNASE4	4	0.21292	0.996504	0.001521	*MATN4*	2	0.19662	0.938234	0.027689
PIGP	4	-0.16804	0.996723	0.001425	*FAM207A*	3	0.09663	0.938541	0.027547
ARHGEF2	3	0.031164	0.996723	0.001425	*FAM72B*	4	-0.51898	0.938676	0.027484
ANKRD54	3	-0.011054	0.997138	0.001245	*APOL6*	4	-0.27981	0.938676	0.027484
TAX1BP1	4	-0.30997	0.997243	0.001199	*MACF1*	2	-0.169	0.938676	0.027484
PLEKHG3	4	0.1644	0.997243	0.001199	*ZNF416*	3	-0.16735	0.938676	0.027484
DPP3	4	0.022263	0.998572	6.21E-04	*LY6G6C*	4	-0.14462	0.938676	0.027484
IBA57	2	-0.36433	0.999156	3.67E-04	*GRM2*	4	-0.10754	0.938676	0.027484
NRIP2	1	-0.31349	0.999156	3.67E-04	*BSG*	4	-0.074764	0.938676	0.027484
CCL22	3	-0.29128	0.999156	3.67E-04	*BTG1*	4	-0.020012	0.938676	0.027484
WDR86	2	-0.24696	0.999156	3.67E-04	*LPAR5*	4	-0.007849	0.938676	0.027484
MAP10	3	-0.24653	0.999156	3.67E-04	*ADAM17*	4	-0.32447	0.938759	0.027446
HLA-DRB5	2	-0.22793	0.999156	3.67E-04	*FSTL3*	4	0.16862	0.938759	0.027446
DKKL1	3	-0.22695	0.999156	3.67E-04	*MC1R*	4	0.16941	0.938759	0.027446
HBG2	1	-0.21147	0.999156	3.67E-04	*ZMYND15*	4	-0.2421	0.939264	0.027213
H2AC11	4	-0.2078	0.999156	3.67E-04	*ELF5*	4	-0.24097	0.939264	0.027213
UNCX	4	-0.20668	0.999156	3.67E-04	*ZCCHC8*	4	-0.20175	0.939264	0.027213
KRTDAP	4	-0.17439	0.999156	3.67E-04	*PIGL*	4	-0.18746	0.939264	0.027213
ERI1	4	-0.17269	0.999156	3.67E-04	*SNX27*	4	-0.18608	0.939264	0.027213
PTPN9	3	-0.1709	0.999156	3.67E-04	*MT1E*	3	-0.17741	0.939264	0.027213
MST1R	4	-0.14809	0.999156	3.67E-04	*ETNK1*	4	-0.15877	0.939264	0.027213
CTSE	3	-0.14512	0.999156	3.67E-04	*CCR6*	4	-0.13328	0.939264	0.027213
MTRES1	4	-0.1423	0.999156	3.67E-04	*TMEM181*	4	-0.0882	0.939264	0.027213
IGFN1	4	-0.11655	0.999156	3.67E-04	*SPATS2L*	3	-0.087706	0.939264	0.027213
PPM1D	3	-0.11345	0.999156	3.67E-04	*APOF*	4	-0.082944	0.939264	0.027213
HPS6	4	-0.1076	0.999156	3.67E-04	*PREX2*	3	-0.065917	0.939264	0.027213
STAM	4	-0.07128	0.999156	3.67E-04	*HYDIN*	4	-0.065563	0.939264	0.027213
MRGBP	3	-0.068032	0.999156	3.67E-04	*ACKR2*	4	-0.044776	0.939264	0.027213
FDX2	4	-0.066445	0.999156	3.67E-04	*DUSP23*	4	-0.010598	0.939264	0.027213
PCDHB9	4	-0.065438	0.999156	3.67E-04	*FOLR2*	4	0.11276	0.939264	0.027213
VGF	4	-0.063447	0.999156	3.67E-04	*IRAK4*	4	0.12207	0.939264	0.027213
KCNK13	4	-0.062545	0.999156	3.67E-04	*EFCC1*	3	0.16344	0.939264	0.027213
2-Mar	4	-0.056242	0.999156	3.67E-04	*IQCE*	3	0.16675	0.939264	0.027213

FLG2	4	-0.04518	0.999156	3.67E-04	*MTMR9*	4	0.16858	0.939264	0.027213
SCAND1	3	-0.040183	0.999156	3.67E-04	*CLCC1*	2	0.35365	0.939264	0.027213
CACNA2D3	4	-0.018498	0.999156	3.67E-04	*CNOT10*	3	-0.42581	0.939342	0.027176
FGF8	4	-2.17E-04	0.999156	3.67E-04	*SMG6*	3	-0.36217	0.939342	0.027176
KBTBD8	3	0.004228	0.999156	3.67E-04	*ZBTB5*	3	-0.34374	0.939342	0.027176
C1orf53	4	0.006791	0.999156	3.67E-04	*FRMPD3*	4	-0.34329	0.939342	0.027176
IL18	3	0.062372	0.999156	3.67E-04	*PMS1*	3	-0.28492	0.939342	0.027176
FNDC4	4	0.075996	0.999156	3.67E-04	*NLRP1*	2	-0.24827	0.939342	0.027176
SATB2	4	0.093675	0.999156	3.67E-04	*FBN1*	3	-0.20424	0.939342	0.027176
TMEM170A	4	0.095798	0.999156	3.67E-04	*TMEM163*	3	-0.19465	0.939342	0.027176
SLC35B4	4	0.10638	0.999156	3.67E-04	*ESRP2*	3	-0.19236	0.939342	0.027176
PRKACB	3	0.11225	0.999156	3.67E-04	*LRRC63*	4	-0.18215	0.939342	0.027176
COPG2	4	0.11553	0.999156	3.67E-04	*EBI3*	4	-0.17935	0.939342	0.027176
MYPOP	4	0.13638	0.999156	3.67E-04	*UBE2D4*	3	-0.17892	0.939342	0.027176
INKA2	4	0.1399	0.999156	3.67E-04	*ZCWPW1*	4	-0.16263	0.939342	0.027176
SHMT2	4	0.14186	0.999156	3.67E-04	*CSPP1*	4	-0.16006	0.939342	0.027176
TOPBP1	3	0.15096	0.999156	3.67E-04	*TBC1D5*	4	-0.13861	0.939342	0.027176
LIMS1	2	0.15172	0.999156	3.67E-04	*PPP6R2*	4	-0.1152	0.939342	0.027176
FAM160A2	4	0.16489	0.999156	3.67E-04	*ZNF524*	4	-0.11406	0.939342	0.027176
UBE2A	2	0.17276	0.999156	3.67E-04	*ANKRD50*	4	-0.098974	0.939342	0.027176
SF3B6	3	0.17291	0.999156	3.67E-04	*OR2AG2*	4	-0.08301	0.939342	0.027176
LARS2	4	0.21671	0.999156	3.67E-04	*AURKC*	4	-0.0515	0.939342	0.027176
EVI5L	3	0.22818	0.999156	3.67E-04	*FBXO45*	4	-0.033387	0.939342	0.027176
EDN2	3	0.27187	0.999156	3.67E-04	*AGPAT2*	4	-0.030662	0.939342	0.027176
CALHM3	1	0.30304	0.999156	3.67E-04	*CASS4*	4	-0.007025	0.939342	0.027176
UBE2C	4	0.32847	0.999156	3.67E-04	*CCDC87*	4	-0.001954	0.939342	0.027176
TBC1D2B	3	-0.32507	0.999174	3.59E-04	*LYPD1*	4	0.006179	0.939342	0.027176
LMX1B	4	-0.12895	0.999174	3.59E-04	*TDO2*	4	0.010225	0.939342	0.027176
ABCG8	4	0.15378	0.999174	3.59E-04	*SSR2*	4	0.033694	0.939342	0.027176
D2HGDH	4	0.21828	0.999174	3.59E-04	*ZER1*	4	0.051101	0.939342	0.027176
NDUFAB1	2	-0.61999	1	0	*CRTC3*	4	0.057381	0.939342	0.027176
RPS29	2	-0.61241	1	0	*FAM20C*	3	0.060083	0.939342	0.027176
BIRC5	2	-0.60124	1	0	*ZNF701*	4	0.0717	0.939342	0.027176
AURKA	1	-0.58004	1	0	*SNAI2*	4	0.078817	0.939342	0.027176
UFD1	3	-0.56065	1	0	*MYO18A*	4	0.084013	0.939342	0.027176
RPS28	2	-0.55116	1	0	*CCDC124*	4	0.084241	0.939342	0.027176
EIF2B5	4	-0.54105	1	0	*RFXAP*	4	0.10483	0.939342	0.027176
SDE2	2	-0.5116	1	0	*GATA1*	3	0.11405	0.939342	0.027176
CDC23	3	-0.50143	1	0	*XIAP*	4	0.13005	0.939342	0.027176
PDCD7	2	-0.48553	1	0	*CBX2*	4	0.13048	0.939342	0.027176
CSNK1A1	2	-0.48523	1	0	*PRRC2B*	4	0.13093	0.939342	0.027176
HAUS3	2	-0.47138	1	0	*RHOV*	4	0.13541	0.939342	0.027176
SFXN3	2	-0.46342	1	0	*C12orf77*	1	0.1455	0.939342	0.027176
NDUFAF1	3	-0.46036	1	0	*NPRL3*	4	0.15283	0.939342	0.027176
IFITM3	2	-0.45317	1	0	*ZNF142*	4	0.15866	0.939342	0.027176
CCNC	3	-0.44905	1	0	*ORC5*	4	0.166	0.939342	0.027176
ANXA2R	2	-0.44716	1	0	*IGSF22*	3	0.18089	0.939342	0.027176
PPP1R15B	3	-0.44584	1	0	*TMEM59L*	4	0.18838	0.939342	0.027176
ANKRA2	2	-0.44046	1	0	*PRPF40A*	4	0.19822	0.939342	0.027176
CCDC86	3	-0.43696	1	0	*SPTAN1*	4	0.20401	0.939342	0.027176
FAM83H	3	-0.42847	1	0	*STX17*	4	0.21555	0.939342	0.027176
DLGAP1	4	-0.42331	1	0	*ASIC3*	4	0.24426	0.939342	0.027176
HAGHL	2	-0.42266	1	0	*SYNE3*	4	0.26462	0.939342	0.027176
HNRNPCL1	2	-0.42063	1	0	*PHB2*	4	0.44114	0.939342	0.027176
FSD2	2	-0.42014	1	0	*SLC9A2*	3	0.22847	0.939382	0.027158
TUBGCP2	3	-0.41976	1	0	*TAS2R13*	3	-0.22066	0.939686	0.027017
L1CAM	2	-0.41627	1	0	*COG8*	3	-0.5181	0.939692	0.027014
TMEM138	2	-0.41014	1	0	*AFF2*	3	-0.21719	0.939692	0.027014

CA1	3	-0.40763	1	0	*PACS2*	3	0.14069	0.939692	0.027014
MFSD11	3	-0.405	1	0	*GLG1*	2	0.22439	0.939732	0.026996
FRMD8	4	-0.39908	1	0	*CITED4*	3	-0.35156	0.939839	0.026946
SLC44A2	3	-0.38859	1	0	*MPP1*	3	-0.30968	0.939839	0.026946
TCL1B	3	-0.38733	1	0	*RNF41*	4	-0.20046	0.939839	0.026946
MMAA	3	-0.38594	1	0	*VHLL*	4	-0.16145	0.939839	0.026946
LTV1	4	-0.38482	1	0	*TMEM260*	4	-0.15575	0.939839	0.026946
MYCBPAP	1	-0.38152	1	0	*CYP21A2*	4	-0.14188	0.939839	0.026946
UAP1L1	4	-0.38103	1	0	*S100A11*	3	-0.13479	0.939839	0.026946
TSSK6	2	-0.38042	1	0	*EYA1*	4	-0.11378	0.939839	0.026946
USP8	3	-0.37742	1	0	*H2AC11*	4	-0.073426	0.939839	0.026946
VPS41	3	-0.37652	1	0	*GIGYF1*	4	-0.025359	0.939839	0.026946
DNAJA3	3	-0.37368	1	0	*TNNI3*	4	-0.022036	0.939839	0.026946
USO1	3	-0.37351	1	0	*IQCD*	4	-0.003657	0.939839	0.026946
SNRPC	2	-0.37212	1	0	*CD300C*	4	-3.60E-04	0.939839	0.026946
ZNF217	3	-0.37076	1	0	*UBAC1*	4	0.008344	0.939839	0.026946
WNT10B	2	-0.37029	1	0	*ZNF749*	4	0.013236	0.939839	0.026946
CLDN15	4	-0.37007	1	0	*TMEM89*	4	0.037051	0.939839	0.026946
TDRKH	4	-0.36974	1	0	*OSBP2*	2	0.076787	0.939839	0.026946
SDF2L1	2	-0.36929	1	0	*RCBTB2*	4	0.078628	0.939839	0.026946
LSM4	3	-0.36913	1	0	*HOXB2*	4	0.091333	0.939839	0.026946
CHST7	4	-0.36514	1	0	*SLC15A3*	4	0.098396	0.939839	0.026946
TMEM125	2	-0.36472	1	0	*GDI1*	4	0.099468	0.939839	0.026946
NAMPT	4	-0.36348	1	0	*YDJC*	4	0.099956	0.939839	0.026946
HNRNPK	4	-0.36337	1	0	*ATXN1L*	4	0.1189	0.939839	0.026946
B3GNT3	1	-0.36285	1	0	*SLC35F1*	4	0.13267	0.939839	0.026946
RASA1	2	-0.36107	1	0	*UPF2*	4	0.13874	0.939839	0.026946
TMEM211	3	-0.36086	1	0	*TET2*	4	0.14615	0.939839	0.026946
MAPKAP1	4	-0.36043	1	0	*LTB4R2*	4	0.15454	0.939839	0.026946
C8orf76	1	-0.35991	1	0	*ZNF268*	4	0.15903	0.939839	0.026946
FGFBP3	3	-0.35855	1	0	*IGSF5*	4	0.18438	0.939839	0.026946
COMMD3	1	-0.35814	1	0	*SMTNL2*	4	0.19835	0.939839	0.026946
NACAD	2	-0.35639	1	0	*NR0B2*	4	0.22224	0.939839	0.026946
IFI27L2	3	-0.35599	1	0	*CUL1*	4	0.28034	0.939839	0.026946
C17orf67	3	-0.35483	1	0	*ZSCAN26*	3	-0.3969	0.939888	0.026924
ZNF318	3	-0.35385	1	0	*OAS2*	4	-0.18053	0.939888	0.026924
DPYD	2	-0.35126	1	0	*WDR93*	3	-0.083903	0.939888	0.026924
HOXB8	3	-0.35075	1	0	*CTSW*	3	0.1414	0.939888	0.026924
GCM2	3	-0.34984	1	0	*NCAM1*	4	0.19613	0.939888	0.026924
EVA1B	2	-0.3492	1	0	*E2F8*	3	-0.2271	0.94042	0.026678
ZFYVE16	2	-0.34743	1	0	*CLDN5*	3	0.035834	0.94042	0.026678
C11orf42	4	-0.34686	1	0	*PIP4K2A*	3	0.18578	0.94042	0.026678
VCPIP1	4	-0.34651	1	0	*MAP3K9*	3	-0.15312	0.940684	0.026556
CCDC71L	4	-0.34479	1	0	*ACSL6*	4	-0.027843	0.940918	0.026448
SH3BGRL3	4	-0.342	1	0	*TUB*	3	-7.70E-04	0.940918	0.026448
HIVEP2	2	-0.3409	1	0	*TREX2*	3	0.047462	0.940918	0.026448
ZFP36	2	-0.34007	1	0	*STN1*	4	0.23412	0.940918	0.026448
PTTG2	2	-0.33838	1	0	*SLCO5A1*	2	-0.46664	0.941546	0.026158
GMPS	3	-0.33746	1	0	*H2AC16*	2	-0.41923	0.941546	0.026158
COL6A3	2	-0.33708	1	0	*PIGR*	3	-0.30284	0.941546	0.026158
TMEM250	3	-0.33629	1	0	*IGFBPL1*	4	-0.25916	0.941546	0.026158
RHBDL2	4	-0.3362	1	0	*TMA7*	3	-0.2336	0.941546	0.026158
CUL1	4	-0.33609	1	0	*PPP1R11*	4	-0.22449	0.941546	0.026158
SIRT5	2	-0.33556	1	0	*FAM241A*	3	-0.22429	0.941546	0.026158
ZCCHC2	2	-0.33509	1	0	*LYSMD2*	4	-0.22392	0.941546	0.026158
RTF2	2	-0.33453	1	0	*TATDN3*	4	-0.22369	0.941546	0.026158
RASSF5	3	-0.33339	1	0	*LAMA2*	3	-0.21529	0.941546	0.026158
IL22RA1	3	-0.33258	1	0	*JARID2*	3	-0.20469	0.941546	0.026158

TMEM140	3	-0.33254	1	0	*CYB5B*	3	-0.19422	0.941546	0.026158
OLFML2A	3	-0.33246	1	0	*KLF3*	3	-0.19107	0.941546	0.026158
MMEL1	4	-0.33171	1	0	*CCNJ*	3	-0.18456	0.941546	0.026158
RNF145	4	-0.33109	1	0	*ANKRD13D*	4	-0.18126	0.941546	0.026158
TOMM70	4	-0.33042	1	0	*DCBLD1*	4	-0.17064	0.941546	0.026158
SRGAP2	3	-0.3298	1	0	*PCNX2*	4	-0.16169	0.941546	0.026158
DLEC1	4	-0.32943	1	0	*FLG*	4	-0.16114	0.941546	0.026158
ERC2	3	-0.32933	1	0	*ING4*	2	-0.15813	0.941546	0.026158
NIPBL	3	-0.32927	1	0	*SERPINB12*	4	-0.14972	0.941546	0.026158
WASF2	3	-0.32908	1	0	*RBFOX1*	4	-0.14597	0.941546	0.026158
PRPF4	2	-0.32861	1	0	*TMED1*	4	-0.1409	0.941546	0.026158
UQCRQ	3	-0.3285	1	0	*TMEM127*	4	-0.13514	0.941546	0.026158
ABCC2	3	-0.32845	1	0	*PDCD2L*	4	-0.13507	0.941546	0.026158
GABRP	3	-0.32836	1	0	*WIPF1*	3	-0.10201	0.941546	0.026158
METTL8	4	-0.32733	1	0	*PRF1*	3	-0.099352	0.941546	0.026158
HASPIN	3	-0.32636	1	0	*RRH*	4	-0.098762	0.941546	0.026158
TMEM200A	4	-0.32384	1	0	*MLH3*	4	-0.096477	0.941546	0.026158
LINC02843	4	-0.32275	1	0	*APLP1*	4	-0.067131	0.941546	0.026158
OC10192732.	1	-0.32187	1	0	*TRPT1*	4	-0.05752	0.941546	0.026158
RPEL1	4	-0.32173	1	0	*TMEM18*	4	-0.04518	0.941546	0.026158
C22orf24	3	-0.3216	1	0	*ABHD17B*	4	-0.039392	0.941546	0.026158
L3MBTL3	1	-0.32144	1	0	*ZNF239*	4	-0.029229	0.941546	0.026158
SCAI	2	-0.32064	1	0	*ABCD3*	4	-0.005494	0.941546	0.026158
DDX10	3	-0.32019	1	0	*DSG3*	4	0.004065	0.941546	0.026158
GRAP	1	-0.31992	1	0	*RGS9*	4	0.012006	0.941546	0.026158
NRROS	4	-0.31852	1	0	*UNC93B1*	4	0.04836	0.941546	0.026158
IQCN	4	-0.31716	1	0	*BLOC1S4*	4	0.062928	0.941546	0.026158
MGAT3	3	-0.31636	1	0	*GNA12*	4	0.086328	0.941546	0.026158
FSIP1	3	-0.31559	1	0	*LRRN1*	4	0.088997	0.941546	0.026158
HRCT1	4	-0.31377	1	0	*N4BP2L1*	4	0.1003	0.941546	0.026158
SPATS2L	3	-0.3133	1	0	*TEX10*	3	0.11018	0.941546	0.026158
TUBA3D	3	-0.31265	1	0	*KCNV2*	4	0.12948	0.941546	0.026158
FLACC1	3	-0.31238	1	0	*SMARCC1*	4	0.13021	0.941546	0.026158
MGST2	3	-0.31156	1	0	*IQGAP1*	3	0.13732	0.941546	0.026158
SNCA	2	-0.31118	1	0	*AGTRAP*	2	0.14974	0.941546	0.026158
IKBKG	1	-0.31001	1	0	*ZNF367*	4	0.15313	0.941546	0.026158
PARVA	2	-0.30959	1	0	*INO80*	4	0.16029	0.941546	0.026158
ARPC5	3	-0.30955	1	0	*TEX43*	2	0.16635	0.941546	0.026158
UROS	4	-0.30949	1	0	*STK35*	3	0.17596	0.941546	0.026158
LRSAM1	4	-0.30913	1	0	*BARD1*	3	0.21113	0.941546	0.026158
EFNA5	4	-0.30873	1	0	*NF1*	4	0.2776	0.941546	0.026158
ACP1	3	-0.30865	1	0	*NAA35*	2	0.3659	0.941546	0.026158
CFDP1	3	-0.30788	1	0	*CD276*	2	-0.3396	0.941811	0.026036
TUBA8	3	-0.30732	1	0	*COMMD5*	3	-0.33791	0.941811	0.026036
NAGLU	4	-0.3062	1	0	*GJB3*	2	0.23194	0.941888	0.026001
IL13RA2	4	-0.30594	1	0	*DENND5A*	3	-0.49021	0.94191	0.02599
ZNF639	3	-0.30475	1	0	*SMYD4*	3	-0.22854	0.94191	0.02599
PIFO	3	-0.30418	1	0	*PRDM4*	4	-0.22612	0.94191	0.02599
ETV1	3	-0.30404	1	0	*CRIM1*	3	-0.21479	0.94191	0.02599
SGPP1	3	-0.30381	1	0	*SEPTIN11*	4	-0.18474	0.94191	0.02599
EPM2A	3	-0.30373	1	0	*TRIM4*	4	-0.10272	0.94191	0.02599
FAM89A	3	-0.30369	1	0	*FAM120C*	3	-0.085278	0.94191	0.02599
GIGYF2	4	-0.30358	1	0	*USP25*	4	-0.030942	0.94191	0.02599
BRAP	3	-0.30291	1	0	*MIEF2*	4	0.037326	0.94191	0.02599
SERPINA1	3	-0.30257	1	0	*GRPEL1*	4	0.14762	0.94191	0.02599
RANBP1	3	-0.30171	1	0	*LMAN2L*	4	0.16745	0.94191	0.02599
RSPH4A	4	-0.30167	1	0	*NDUFA12*	4	0.30372	0.94191	0.02599
MICOS13	4	-0.30162	1	0	*CLMP*	3	-0.20556	0.942063	0.02592

AEBP2	3	-0.30087	1	0	*TXNDC17*	3	0.057299	0.942063	0.02592
EWSR1	4	-0.30058	1	0	*TNFSF11*	4	-0.2539	0.942151	0.02588
UTP15	3	-0.29826	1	0	*GPR143*	4	-0.22158	0.942151	0.02588
CABP7	2	-0.29704	1	0	*HSP90AB1*	4	-0.16154	0.942151	0.02588
PPIP5K2	3	-0.29695	1	0	*STAC2*	4	-0.095178	0.942151	0.02588
ACOT7	4	-0.2969	1	0	*TP53BP1*	4	-0.083179	0.942151	0.02588
GAL3ST3	3	-0.29687	1	0	*HENMT1*	4	-0.05824	0.942151	0.02588
PXMP2	3	-0.29686	1	0	*CD99L2*	4	0.046944	0.942151	0.02588
GAB2	2	-0.29684	1	0	*OR2AT4*	3	-0.14677	0.942301	0.02581
LMLN	4	-0.29654	1	0	*KPTN*	2	-0.31397	0.942489	0.025724
L3MBTL4	3	-0.29629	1	0	*RBBP9*	3	0.01512	0.942491	0.025723
BEX5	2	-0.29611	1	0	*RNF135*	4	-0.24799	0.942569	0.025687
ZNF773	3	-0.29564	1	0	*SMPDL3A*	4	0.006208	0.942569	0.025687
ASPM	3	-0.29486	1	0	*DENND2C*	4	-0.30768	0.943241	0.025377
MPHOSPH9	1	-0.29466	1	0	*PPT1*	4	-0.084702	0.943241	0.025377
SAXO2	4	-0.29466	1	0	*USO1*	3	-0.45312	0.943277	0.025361
NIPA2	2	-0.29441	1	0	*DDX60*	3	-0.42777	0.943277	0.025361
PFKFB4	4	-0.29423	1	0	*C17orf82*	3	-0.38612	0.943277	0.025361
LCN12	2	-0.29407	1	0	*GADD45A*	3	-0.3197	0.943277	0.025361
TCF7L1	3	-0.29377	1	0	*CLEC19A*	3	-0.19614	0.943277	0.025361
CNR1	3	-0.29375	1	0	*CLIC5*	2	-0.19377	0.943277	0.025361
PTPN3	3	-0.29281	1	0	*CELF6*	3	-0.16072	0.943277	0.025361
ERICH2	4	-0.29265	1	0	*NAA80*	4	-0.043338	0.943277	0.025361
CYP2W1	4	-0.29262	1	0	*ZNF878*	4	-0.027629	0.943277	0.025361
KIAA0319	4	-0.29191	1	0	*CGAS*	4	9.97E-04	0.943277	0.025361
DTL	3	-0.29162	1	0	*ASPHD2*	3	0.010321	0.943277	0.025361
RADIL	4	-0.29156	1	0	*SLC17A5*	4	0.046203	0.943277	0.025361
PRRC1	4	-0.29139	1	0	*ANKRD13B*	3	0.05501	0.943277	0.025361
SETDB1	4	-0.29102	1	0	*BTRC*	4	0.081738	0.943277	0.025361
UQCRB	3	-0.2907	1	0	*KLK14*	3	0.11063	0.943277	0.025361
SF3A2	3	-0.29055	1	0	*CCNE1*	4	0.16541	0.943277	0.025361
SPTSSB	2	-0.29036	1	0	*SRRM5*	4	0.16839	0.943277	0.025361
OSBPL7	4	-0.29029	1	0	*RNF44*	4	0.16872	0.943277	0.025361
CHSY3	4	-0.28961	1	0	*INF2*	3	0.22182	0.943277	0.025361
GPBAR1	3	-0.28961	1	0	*MET*	3	0.26027	0.943277	0.025361
GPS2	3	-0.28927	1	0	*CDCA5*	4	0.37767	0.943277	0.025361
ARFRP1	3	-0.28908	1	0	*ALG1L2*	2	-0.043853	0.943613	0.025206
ATP5F1E	2	-0.28889	1	0	*ARHGAP5*	2	-0.19367	0.944135	0.024966
CCDC168	4	-0.28881	1	0	*PDK4*	2	-0.13388	0.944135	0.024966
CCZ1B	1	-0.28873	1	0	*WNK1*	3	-0.21708	0.944141	0.024963
ZBTB8OS	3	-0.28816	1	0	*DNAJA4*	2	0.15688	0.944141	0.024963
ZNF185	1	-0.2875	1	0	*PRSS1*	3	0.19288	0.944141	0.024963
GNGT2	2	-0.28654	1	0	*RBMX*	3	0.20739	0.944141	0.024963
EXOC1	3	-0.2865	1	0	*CD4*	3	-0.28695	0.944365	0.02486
ARRDC4	4	-0.28609	1	0	*APOBR*	4	-0.010948	0.944516	0.024791
SMCO3	3	-0.28598	1	0	*NR5A1*	4	2.58E-04	0.944516	0.024791
MINAR1	2	-0.28569	1	0	*SLC5A11*	4	-0.24244	0.944777	0.024671
MSI1	4	-0.28508	1	0	*TARBP2*	4	-0.031359	0.944853	0.024636
H2AC8	4	-0.28438	1	0	*OSBPL1A*	3	-0.013695	0.945002	0.024567
PARD6G	4	-0.28376	1	0	*C22orf39*	1	0.12471	0.945374	0.024396
KLHL33	3	-0.2833	1	0	*NTS*	4	-0.30713	0.945616	0.024285
CRYBG1	3	-0.2831	1	0	*LUC7L2*	1	-0.25614	0.945616	0.024285
IGFBP4	2	-0.28303	1	0	*GGT6*	4	-0.23981	0.945616	0.024285
NAGPA	4	-0.28078	1	0	*ZP2*	4	-0.18826	0.945616	0.024285
TRIM68	3	-0.28016	1	0	*FBXW12*	4	-0.17814	0.945616	0.024285
ABI2	4	-0.27987	1	0	*ZFP1*	4	-0.16152	0.945616	0.024285
EFCC1	3	-0.27986	1	0	*ASIP*	3	-0.1607	0.945616	0.024285
HYAL2	2	-0.27982	1	0	*ARL8B*	3	-0.13618	0.945616	0.024285

RNF103	3	-0.2793	1	0	*NCOA3*	4	-0.1271	0.945616	0.024285
EIF2AK1	2	-0.2783	1	0	*TMUB2*	4	-0.052229	0.945616	0.024285
TULP2	2	-0.2783	1	0	*STAG3*	4	0.030536	0.945616	0.024285
CENPP	2	-0.27827	1	0	*POLE2*	3	0.086943	0.945616	0.024285
EXTL3	3	-0.27782	1	0	*ST8SIA4*	4	0.13245	0.945616	0.024285
AHRR	4	-0.27744	1	0	*TINF2*	3	0.2038	0.945616	0.024285
PSMC3IP	4	-0.27724	1	0	*TCF15*	3	-0.40594	0.945804	0.024199
CCDC170	4	-0.27647	1	0	*NUP62CL*	4	-0.21481	0.945804	0.024199
OMA1	4	-0.27634	1	0	*DMWD*	4	-0.14628	0.945954	0.02413
GLYR1	4	-0.27619	1	0	*RBFOX3*	4	-0.03459	0.945954	0.02413
BEST3	4	-0.27597	1	0	*AKAP12*	2	-0.27325	0.946113	0.024057
STUM	2	-0.27556	1	0	*ERI2*	3	-0.19738	0.946113	0.024057
NUSAP1	4	-0.27553	1	0	*ADM*	4	-0.098759	0.946113	0.024057
LCLAT1	3	-0.27528	1	0	*SERPINH1*	4	-0.059448	0.946113	0.024057
DNM3	3	-0.27473	1	0	*NFKBIZ*	4	-0.036163	0.946113	0.024057
LMNB1	3	-0.27469	1	0	*FSTL1*	4	0.018014	0.946113	0.024057
ZNF430	4	-0.27411	1	0	*CCDC61*	3	0.14533	0.946113	0.024057
UBE2G1	4	-0.27406	1	0	*APOBEC3D*	2	0.31411	0.946113	0.024057
POU3F2	4	-0.27399	1	0	*ASTN1*	4	0.2052	0.946262	0.023989
RREB1	2	-0.27394	1	0	*FBXO34*	4	-0.29801	0.946531	0.023865
CAV2	3	-0.27372	1	0	*GPR85*	3	-0.29111	0.946531	0.023865
TMEM254	3	-0.27354	1	0	*ZNF107*	3	-0.21924	0.946531	0.023865
ICE2	3	-0.27239	1	0	*NFATC3*	4	-0.12775	0.946531	0.023865
SUDS3	4	-0.27219	1	0	*PNMA8A*	4	-0.10832	0.946531	0.023865
BDH1	3	-0.27198	1	0	*PHF3*	4	0.001828	0.946531	0.023865
PMM1	4	-0.27155	1	0	*TRIM2*	4	0.02694	0.946531	0.023865
MED4	4	-0.27127	1	0	*GRK6*	4	0.13132	0.946531	0.023865
AMY2B	3	-0.27123	1	0	*SPAG4*	3	-0.26241	0.94668	0.023797
FOXD1	3	-0.27098	1	0	*FMN1*	3	-0.028476	0.946902	0.023695
AURKAIP1	4	-0.27084	1	0	*PEBP4*	2	-0.30367	0.947126	0.023592
CSTF1	4	-0.27068	1	0	*NUDCD2*	3	-0.27689	0.947126	0.023592
MRPL46	4	-0.27059	1	0	*IRX3*	4	-0.2398	0.947274	0.023524
TNRC6B	4	-0.27045	1	0	*C12orf50*	4	0.071194	0.947275	0.023524
CCL3L3	3	-0.27037	1	0	*DUS1L*	4	0.16758	0.947719	0.023321
AKTIP	2	-0.27035	1	0	*FAM153B*	2	-0.42759	0.948019	0.023183
PAGE2B	2	-0.27035	1	0	*TIGD5*	2	-0.28366	0.948019	0.023183
SETD9	4	-0.27026	1	0	*EFCAB14*	2	-0.23914	0.948019	0.023183
PLXDC1	1	-0.27005	1	0	*SPOCK1*	2	-0.23199	0.948019	0.023183
DMAC2L	4	-0.26984	1	0	*ADI1*	2	-0.019316	0.948019	0.023183
ZNF648	2	-0.26962	1	0	*HMGN2*	4	0.036606	0.948057	0.023166
C20orf96	4	-0.26945	1	0	*WDR83OS*	4	-0.13201	0.948132	0.023131
PDZK1	2	-0.26929	1	0	*SPINK7*	2	0.11627	0.94839	0.023013
GTF3C3	3	-0.26919	1	0	*LYST*	3	-0.31973	0.948429	0.022995
GLT8D1	3	-0.26903	1	0	*C11orf44*	3	-0.17982	0.948429	0.022995
PTPN14	4	-0.269	1	0	*ANXA3*	4	-0.357	0.948617	0.022909
KAZALD1	4	-0.26899	1	0	*GZF1*	4	-0.27511	0.948617	0.022909
ORMDL3	4	-0.26888	1	0	*HMG20B*	4	-0.20425	0.948617	0.022909
ZNF280D	4	-0.26873	1	0	*TMED8*	4	0.096078	0.948765	0.022841
TOB2	3	-0.26859	1	0	*SNAI1*	3	-0.031302	0.948766	0.022841
ENO3	3	-0.26837	1	0	*VOPP1*	2	-0.37372	0.948804	0.022824
PDIA3	4	-0.26835	1	0	*ANAPC1*	4	-0.32861	0.948952	0.022756
FAM124B	4	-0.26812	1	0	*ATOH7*	4	-0.054479	0.949284	0.022604
HAUS2	4	-0.26809	1	0	*NPFFR1*	3	-0.17462	0.950277	0.02215
TTC16	3	-0.26804	1	0	*IQCB1*	1	-0.26241	0.950902	0.021864
PYROXD2	3	-0.26792	1	0	*SDS*	3	-0.42717	0.951017	0.021812
ABHD10	4	-0.26756	1	0	*CIB2*	2	-0.19627	0.951017	0.021812
KCNJ2	4	-0.26736	1	0	*PNMA3*	4	-0.1799	0.951017	0.021812
CLEC4E	3	-0.26734	1	0	*ATP6V0A2*	2	0.050454	0.951017	0.021812

SAGE1	4	-0.26706	1	0	*ELMOD1*	3	0.15815	0.951017	0.021812
POLR2H	2	-0.26698	1	0	*SPATC1L*	4	0.20758	0.951017	0.021812
ZNF618	3	-0.2668	1	0	*UBXN4*	3	-0.28619	0.951057	0.021794
PPP1R27	4	-0.26674	1	0	*HERPUD2*	3	-0.065676	0.951057	0.021794
PCSK9	3	-0.26669	1	0	*GRAMD1C*	2	0.14232	0.951057	0.021794
ABCC10	1	-0.26646	1	0	*CCDC92*	3	0.040158	0.951975	0.021374
ZNF865	3	-0.26644	1	0	*PCBP1*	1	0.25695	0.951975	0.021374
ADAP2	4	-0.26638	1	0	*SBK2*	4	0.14143	0.95216	0.02129
SERPINE1	3	-0.26637	1	0	*KCNG2*	4	0.18813	0.95216	0.02129
PSORS1C2	2	-0.26626	1	0	*ACTG1*	3	-0.098223	0.952273	0.021238
PIK3R4	4	-0.26613	1	0	*UGT1A7*	4	0.05521	0.952273	0.021238
PPIH	3	-0.26535	1	0	*EEF1AKMT2*	2	0.10896	0.952273	0.021238
CRY1	3	-0.26534	1	0	*RPS8*	1	0.52158	0.952273	0.021238
HUNK	3	-0.26519	1	0	*SQOR*	3	-0.24442	0.952677	0.021054
TARS1	4	-0.26519	1	0	*AUTS2*	2	-0.21352	0.952678	0.021054
AFF4	4	-0.26513	1	0	*AMFR*	4	-0.025194	0.952753	0.02102
CCDC9B	2	-0.2651	1	0	*HRH2*	4	0.00303	0.952753	0.02102
ARG2	4	-0.26507	1	0	*CCL24*	4	0.10328	0.952753	0.02102
MED12	3	-0.26492	1	0	*FAH*	2	-0.23566	0.952937	0.020936
HNRNPF	4	-0.26473	1	0	*TNK2*	4	0.11669	0.953084	0.020869
MARCHF11	4	-0.26463	1	0	*CCNO*	3	-0.30842	0.953203	0.020815
ANP32C	3	-0.26382	1	0	*LHX4*	2	-0.27556	0.953203	0.020815
FOXD4L4	1	-0.2637	1	0	*MTX3*	4	-0.27316	0.953203	0.020815
C1orf56	3	-0.26315	1	0	*HERC6*	4	-0.22931	0.953203	0.020815
LRPAP1	3	-0.26313	1	0	*BPIFB1*	3	-0.18387	0.953203	0.020815
FAXC	3	-0.26305	1	0	*SLC16A12*	3	-0.13588	0.953203	0.020815
CAVIN1	3	-0.26304	1	0	*ZNF614*	4	-0.10386	0.953203	0.020815
CAMK4	4	-0.26301	1	0	*RPRD1B*	4	0.007628	0.953203	0.020815
AKAP1	3	-0.263	1	0	*TM2D3*	4	0.04025	0.953203	0.020815
GGT7	3	-0.26279	1	0	*GPR78*	3	0.056235	0.953203	0.020815
RPTOR	4	-0.2622	1	0	*GSAP*	4	0.073003	0.953203	0.020815
PRKG2	4	-0.262	1	0	*CCNJL*	3	0.074901	0.953203	0.020815
GGA3	3	-0.26172	1	0	*CKAP4*	4	0.10236	0.953203	0.020815
EPHA3	4	-0.2617	1	0	*SMAGP*	4	0.20619	0.953203	0.020815
UBLCP1	2	-0.26118	1	0	*LYN*	4	0.24484	0.953203	0.020815
SAMD15	4	-0.26063	1	0	*GFPT1*	4	0.32944	0.953203	0.020815
OARD1	4	-0.26036	1	0	*NEURL1B*	3	0.074003	0.953278	0.020781
IFT20	4	-0.26012	1	0	*BET1*	3	0.1362	0.953278	0.020781
COL9A1	4	-0.25983	1	0	*MAP4K4*	3	0.15658	0.953278	0.020781
PSEN2	4	-0.25978	1	0	*AMIGO3*	4	-0.26988	0.953501	0.020679
NAGS	4	-0.25966	1	0	*HBQ1*	2	0.028913	0.953501	0.020679
RALGPS1	3	-0.25932	1	0	*MAP7*	3	0.029579	0.953501	0.020679
FAM72B	4	-0.25925	1	0	*TNS2*	4	0.07329	0.953501	0.020679
GPIHBP1	4	-0.25882	1	0	*AOAH*	4	0.099234	0.953501	0.020679
TMC4	2	-0.25882	1	0	*UBE2C*	4	0.21726	0.953501	0.020679
SLC8A2	1	-0.25871	1	0	*CHAF1B*	4	0.37536	0.953501	0.020679
SMO	2	-0.25851	1	0	*MOSPD3*	4	-0.18735	0.953575	0.020645
IFNAR1	3	-0.25831	1	0	*H2BC17*	4	-0.1804	0.953575	0.020645
TIGD4	2	-0.25801	1	0	*SLC27A2*	4	0.027292	0.953575	0.020645
SLIT3	4	-0.25754	1	0	*GAS6*	3	0.10592	0.953758	0.020562
BCL10	3	-0.25752	1	0	*KCNC4*	4	-0.037736	0.953905	0.020495
IPMK	4	-0.25724	1	0	*H3C6*	1	0.24589	0.954088	0.020412
FRRS1	3	-0.25666	1	0	*ZMYM4*	4	-0.24894	0.954125	0.020395
CAGE1	4	-0.25646	1	0	*LEMD2*	4	0.13869	0.954125	0.020395
PIP5K1C	4	-0.25646	1	0	*CCDC173*	2	-0.36406	0.954346	0.020294
SHISAL1	3	-0.25642	1	0	*OR2AG1*	2	-0.20585	0.954346	0.020294
MASP2	4	-0.25609	1	0	*SNX30*	4	-0.19074	0.954346	0.020294
PP2D1	3	-0.25604	1	0	*PREP*	4	0.068406	0.954346	0.020294

P3H1	2	-0.25592	1	0	*MRVI1*	2	0.14873	0.954785	0.020095
C2orf74	4	-0.25572	1	0	*SURF4*	2	0.20927	0.954785	0.020095
SEC31B	2	-0.25552	1	0	*SLC45A2*	4	-0.18824	0.954787	0.020093
LST1	3	-0.25545	1	0	*CCDC68*	4	-0.16683	0.954787	0.020093
GLA	1	-0.2553	1	0	*PDE1C*	4	-0.16179	0.954787	0.020093
GALNT2	3	-0.25498	1	0	*SPAG17*	4	-0.009659	0.954787	0.020093
SYCP2L	4	-0.25495	1	0	*NNMT*	4	0.013793	0.954787	0.020093
HDGF	2	-0.25457	1	0	*NFKBIE*	1	0.21624	0.954787	0.020093
RHOXF2B	1	-0.25417	1	0	*TRIM13*	2	0.31934	0.954787	0.020093
TNFRSF8	4	-0.25389	1	0	*ARHGEF7*	3	-0.25258	0.955189	0.019911
IFNB1	3	-0.25362	1	0	*GNPDA2*	4	0.13183	0.955189	0.019911
SBK1	4	-0.25357	1	0	*TSC1*	3	-0.32246	0.955663	0.019695
ARID3B	3	-0.25356	1	0	*CLEC3B*	3	-0.27674	0.955663	0.019695
ACAT1	2	-0.25335	1	0	*CHD1L*	3	-0.29052	0.955774	0.019645
PLD1	3	-0.25294	1	0	*TAFA4*	4	-0.065857	0.955774	0.019645
MED10	4	-0.25286	1	0	*TDRD9*	3	-0.001692	0.955774	0.019645
FAM120B	3	-0.25285	1	0	*VILL*	3	0.033679	0.955774	0.019645
TUBD1	3	-0.25257	1	0	*MAP2*	4	0.13134	0.955774	0.019645
NUFIP2	2	-0.25217	1	0	*SLC25A53*	4	0.13325	0.955774	0.019645
CDH10	2	-0.25201	1	0	*CSRNP2*	1	-0.19148	0.955993	0.019545
MT1A	3	-0.25191	1	0	*SAMSN1*	2	0.12662	0.956211	0.019446
NAA35	2	-0.25184	1	0	*LRIG1*	3	-0.046538	0.956248	0.01943
DPM2	4	-0.25139	1	0	*DNAI2*	3	-0.43103	0.956257	0.019426
GPR20	2	-0.25125	1	0	*GPR37L1*	3	-0.32159	0.956257	0.019426
IL4R	4	-0.25123	1	0	*LRRC47*	4	-0.3104	0.956257	0.019426
SUN3	3	-0.25116	1	0	*RXFP4*	4	-0.28791	0.956257	0.019426
ARIH1	3	-0.25109	1	0	*KRT33A*	3	-0.25547	0.956257	0.019426
TLR1	4	-0.25088	1	0	*FUCA1*	4	-0.17906	0.956257	0.019426
GSTA1	1	-0.25078	1	0	*NGLY1*	3	-0.17566	0.956257	0.019426
RBPMS	4	-0.25078	1	0	*KIF5A*	2	-0.16201	0.956257	0.019426
CCDC152	4	-0.25073	1	0	*SOX30*	4	-0.16086	0.956257	0.019426
MFSD13A	4	-0.2506	1	0	*LIN28B*	4	-0.13769	0.956257	0.019426
GRHL3	4	-0.25057	1	0	*ZBTB7A*	4	-0.12743	0.956257	0.019426
COL16A1	3	-0.25056	1	0	*SH2D6*	3	-0.12423	0.956257	0.019426
OC10065275	3	-0.2502	1	0	*UBAP1L*	4	-0.11246	0.956257	0.019426
SEC22A	4	-0.2502	1	0	*C8orf86*	4	-0.076634	0.956257	0.019426
ZNF808	4	-0.24986	1	0	*LINGO2*	3	-0.060136	0.956257	0.019426
HAUS8	4	-0.24968	1	0	*C8orf87*	4	0.004239	0.956257	0.019426
OR2A14	4	-0.24962	1	0	*BOC*	2	0.005681	0.956257	0.019426
GFM1	4	-0.24951	1	0	*GANAB*	4	0.008993	0.956257	0.019426
SLC39A5	4	-0.24903	1	0	*SYNC*	4	0.025987	0.956257	0.019426
FUBP3	3	-0.24892	1	0	*DNAJC13*	4	0.026352	0.956257	0.019426
PSMA3	4	-0.24876	1	0	*KRT79*	4	0.10311	0.956257	0.019426
B3GNT4	4	-0.24865	1	0	*HS3ST3B1*	4	0.11233	0.956257	0.019426
NINJ2	3	-0.24841	1	0	*ACVRL1*	4	0.11626	0.956257	0.019426
ARIH2OS	3	-0.24828	1	0	*KCTD14*	2	0.11694	0.956257	0.019426
ACTBL2	3	-0.24819	1	0	*CDADC1*	4	0.11752	0.956257	0.019426
KIAA1671	3	-0.24812	1	0	*DNAJC17*	4	0.1186	0.956257	0.019426
PPM1E	4	-0.24778	1	0	*FIG4*	4	0.12173	0.956257	0.019426
CMYA5	3	-0.24755	1	0	*RAD51B*	4	0.12274	0.956257	0.019426
HTRA2	3	-0.24744	1	0	*ATG10*	4	0.12839	0.956257	0.019426
ZSCAN22	3	-0.24728	1	0	*HSPG2*	4	0.13676	0.956257	0.019426
CLVS1	4	-0.2465	1	0	*SDHD*	1	0.16764	0.956257	0.019426
EDEM1	3	-0.24636	1	0	*ZNF699*	4	0.17177	0.956257	0.019426
CIDEC	4	-0.24622	1	0	*DOCK4*	4	0.17342	0.956257	0.019426
FIBCD1	4	-0.24601	1	0	*NUP210*	4	0.175	0.956257	0.019426
SP4	4	-0.24572	1	0	*ZNF778*	4	0.17654	0.956257	0.019426
FGL1	4	-0.24567	1	0	*NMNAT3*	4	0.17901	0.956257	0.019426

FAXDC2	1	-0.24523	1	0	*ACTR3*	4	0.21141	0.956257	0.019426
HARS1	3	-0.24509	1	0	*ZFPM1*	4	0.21302	0.956257	0.019426
DDIT3	4	-0.24464	1	0	*NCL*	4	0.22103	0.956257	0.019426
CZIB	3	-0.24458	1	0	*EXOC7*	4	0.2793	0.956257	0.019426
CDH23	2	-0.24452	1	0	*OTOP2*	1	0.30773	0.956257	0.019426
CDH15	4	-0.24433	1	0	*R3HCC1*	3	0.16308	0.95651	0.01931
FGFR1	3	-0.24424	1	0	*ARMCX6*	4	0.21751	0.956547	0.019294
NOX4	3	-0.24406	1	0	*CHCHD3*	4	-0.199	0.956583	0.019277
MTTP	3	-0.244	1	0	*SOX4*	3	-0.38194	0.956584	0.019277
ASMTL	2	-0.24385	1	0	*ZNF706*	1	-0.37424	0.956584	0.019277
PCOLCE2	3	-0.2438	1	0	*SHC3*	4	-0.1837	0.956584	0.019277
MAFG	4	-0.24375	1	0	*CCDC102B*	3	-0.051829	0.956584	0.019277
SNAI3	4	-0.2437	1	0	*S1PR1*	3	0.054552	0.956584	0.019277
MAGEA12	2	-0.24364	1	0	*ANKRD54*	3	-0.26438	0.956802	0.019178
UQCC3	2	-0.24338	1	0	*AP5M1*	2	-0.19065	0.956802	0.019178
COX19	4	-0.2432	1	0	*LTA4H*	4	-0.12622	0.956802	0.019178
LRRC73	2	-0.24294	1	0	*PIK3AP1*	2	-0.030235	0.956802	0.019178
TGFA	3	-0.24293	1	0	*DTNBP1*	2	0.12788	0.956802	0.019178
ATG2A	3	-0.24272	1	0	*PLAT*	2	0.12451	0.9572	0.018997
HES4	4	-0.24259	1	0	*SRGAP2C*	1	0.14232	0.957272	0.018965
MMP21	3	-0.24245	1	0	*CDH4*	3	-0.32745	0.957272	0.018964
TNPO1	3	-0.24245	1	0	*TMPRSS9*	3	0.22128	0.957272	0.018964
SAAL1	4	-0.24221	1	0	*MCOLN3*	3	-0.29812	0.95731	0.018947
BIN3	3	-0.242	1	0	*PCDHGC3*	3	-0.23835	0.95731	0.018947
MAX	4	-0.24195	1	0	*RCN3*	4	-0.22277	0.95731	0.018947
TRMU	4	-0.24189	1	0	*FKBP14*	3	-0.22144	0.95731	0.018947
HLA-DQB1	3	-0.24176	1	0	*ZNF93*	1	-0.21007	0.95731	0.018947
GLIPR1L1	4	-0.24157	1	0	*SPOCK3*	4	-0.17757	0.95731	0.018947
HHIPL1	3	-0.24108	1	0	*SCN2B*	3	-0.13006	0.95731	0.018947
MYEOV	3	-0.24099	1	0	*EFCAB7*	4	-0.050003	0.95731	0.018947
COX6A1	2	-0.24096	1	0	*ZBTB12*	4	-0.018198	0.95731	0.018947
STK16	4	-0.24071	1	0	*PRSS3*	4	-0.007863	0.95731	0.018947
LRRC47	4	-0.2404	1	0	*GASK1A*	4	0.024996	0.95731	0.018947
TJP1	3	-0.24039	1	0	*MDK*	4	0.085172	0.95731	0.018947
SPEG	3	-0.24031	1	0	*MTRR*	3	0.11614	0.95731	0.018947
CEP95	4	-0.24001	1	0	*MAPK11*	4	0.13838	0.95731	0.018947
SNRPE	3	-0.24001	1	0	*CDK12*	4	0.29089	0.95731	0.018947
CLTA	2	-0.23981	1	0	*AGAP2*	4	-0.3032	0.957346	0.018931
ASS1	3	-0.2397	1	0	*HNRNPR*	4	-0.22184	0.957346	0.018931
SIM1	4	-0.23913	1	0	*ZSCAN2*	4	-0.18652	0.957346	0.018931
GFI1B	2	-0.23899	1	0	*RSPO3*	4	0.10446	0.957382	0.018915
PRSS55	4	-0.23835	1	0	*IFNL2*	3	-0.44588	0.958248	0.018522
MICB	4	-0.23823	1	0	*C17orf97*	2	-0.40401	0.958248	0.018522
ZNF181	3	-0.23821	1	0	*FBXO4*	2	-0.16138	0.958248	0.018522
CAPZB	4	-0.23794	1	0	*BSDC1*	4	-0.066851	0.958248	0.018522
HS3ST6	4	-0.2379	1	0	*DBT*	3	-0.28991	0.958356	0.018473
NELFE	4	-0.23748	1	0	*MLEC*	4	-0.24836	0.958356	0.018473
KDM6B	4	-0.23743	1	0	*LGALS9*	3	-0.22278	0.958356	0.018473
HK3	4	-0.23721	1	0	*ETHE1*	3	-0.19324	0.958356	0.018473
PIGB	3	-0.2366	1	0	*C1QL1*	3	-0.16416	0.958356	0.018473
TMEM219	4	-0.23654	1	0	*SASH1*	4	-0.15462	0.958356	0.018473
NUP153	4	-0.23644	1	0	*PRICKLE3*	4	-0.15156	0.958356	0.018473
ZMAT3	4	-0.23631	1	0	*B3GAT1*	4	-0.11559	0.958356	0.018473
TESK2	4	-0.23625	1	0	*UBXN2B*	4	-0.10187	0.958356	0.018473
CYP2U1	3	-0.23617	1	0	*CCDC51*	3	0.004068	0.958356	0.018473
LPIN2	4	-0.23573	1	0	*PKNOX1*	4	0.071753	0.958356	0.018473
FAM92A	2	-0.23565	1	0	*SH3BGRL3*	4	0.095885	0.958356	0.018473
MIPEP	4	-0.2355	1	0	*TGM5*	2	0.15041	0.958356	0.018473

IL11	3	-0.23543	1	0	*BID*	4	0.16304	0.958356	0.018473
PGBD5	4	-0.23541	1	0	*NETO2*	3	-0.072091	0.958464	0.018424
IRF2BP1	3	-0.23536	1	0	*TMEM39B*	3	-0.057404	0.958464	0.018424
OAS3	4	-0.23532	1	0	*UMPS*	3	-0.055837	0.958464	0.018424
WDR59	3	-0.23475	1	0	*KLK6*	3	0.14132	0.958464	0.018424
STAT3	3	-0.23467	1	0	*HPF1*	4	-0.24026	0.958572	0.018375
H1-5	3	-0.23408	1	0	*CABIN1*	4	-0.15311	0.958572	0.018375
EED	3	-0.23406	1	0	*KIF27*	3	-0.29082	0.958679	0.018327
TRMO	4	-0.23401	1	0	*PEX13*	4	0.027254	0.958679	0.018327
CNR2	3	-0.234	1	0	*GIN1*	3	0.10513	0.958679	0.018327
POLI	4	-0.23395	1	0	*TEX30*	2	0.11754	0.958679	0.018327
ZNF274	3	-0.23379	1	0	*ILKAP*	4	-0.17792	0.958787	0.018278
RDH11	4	-0.23342	1	0	*ANKRD36B*	4	0.043886	0.958859	0.018245
SH2D2A	3	-0.23307	1	0	*POLL*	4	0.051235	0.958859	0.018245
NKTR	4	-0.23298	1	0	*ZNF26*	3	-0.24482	0.959074	0.018148
BLVRA	3	-0.23271	1	0	*GRAMD2A*	3	0.057467	0.959074	0.018148
ULBP1	4	-0.23245	1	0	*PRKRIP1*	1	0.21138	0.959074	0.018148
AP4B1	4	-0.23234	1	0	*CTBP1*	4	-0.15512	0.959182	0.018099
KBTBD7	1	-0.23188	1	0	*DAZAP1*	3	0.17498	0.959182	0.018099
LBX2	4	-0.23186	1	0	*MST1*	3	0.22772	0.959182	0.018099
HS6ST1	2	-0.23184	1	0	*FGFBP3*	3	0.26771	0.959182	0.018099
RARG	4	-0.23174	1	0	*DNAAF3*	4	-0.090348	0.959361	0.018018
NPLOC4	3	-0.23149	1	0	*TBC1D30*	4	0.056489	0.959361	0.018018
LAMA2	3	-0.23121	1	0	*ARHGEF18*	2	-0.34641	0.959394	0.018003
C9orf16	4	-0.23117	1	0	*TDGF1*	3	-0.25371	0.959394	0.018003
NHEJ1	4	-0.23109	1	0	*DCP1B*	2	-0.1774	0.959394	0.018003
MED14	4	-0.23103	1	0	*ZFP3*	3	-0.16722	0.959394	0.018003
CRYGS	4	-0.23092	1	0	*EID2B*	4	-0.15438	0.959394	0.018003
NAA25	3	-0.23065	1	0	*C2orf76*	4	-0.084686	0.959394	0.018003
TMEM265	4	-0.23064	1	0	*ESPNL*	4	-0.06485	0.959394	0.018003
ZNF28	4	-0.23063	1	0	*SHKBP1*	4	-0.013341	0.959394	0.018003
PRR25	4	-0.2305	1	0	*ZNF358*	4	0.010451	0.959394	0.018003
MIEF1	3	-0.23044	1	0	*CLN3*	4	0.012916	0.959394	0.018003
ACAT2	1	-0.23042	1	0	*ETFDH*	4	0.03923	0.959394	0.018003
PCDHB15	3	-0.23041	1	0	*WTAP*	3	0.047065	0.959394	0.018003
SHISA2	4	-0.23027	1	0	*DHDH*	4	0.047333	0.959394	0.018003
RBM25	4	-0.23025	1	0	*PAGR1*	4	0.095624	0.959394	0.018003
CRACR2A	2	-0.23017	1	0	*SLC25A17*	2	0.1083	0.959394	0.018003
ARMC3	3	-0.23	1	0	*TSPOAP1*	2	0.11677	0.959394	0.018003
LRP5L	3	-0.22936	1	0	*ANKRD9*	4	0.15857	0.959394	0.018003
GGA2	4	-0.22888	1	0	*H3C3*	4	0.16167	0.959394	0.018003
ANKS1B	4	-0.22864	1	0	*ZC3H12A*	3	0.21038	0.959394	0.018003
CLASRP	3	-0.22841	1	0	*GDPD2*	3	-0.28125	0.959788	0.017825
LILRB3	3	-0.22834	1	0	*ARHGAP23*	3	-0.090761	0.959788	0.017825
PPP2R5D	1	-0.22825	1	0	*BCHE*	3	-0.035195	0.959788	0.017825
ABHD12B	4	-0.22818	1	0	*MEA1*	3	0.023957	0.959788	0.017825
SYNCRIP	3	-0.22817	1	0	*CISD1*	2	-0.34657	0.960145	0.017663
SMYD2	4	-0.22804	1	0	*C10orf90*	2	0.004354	0.960145	0.017663
RNF5	3	-0.228	1	0	*PER1*	3	-0.31305	0.960678	0.017422
PCDHGC3	3	-0.22783	1	0	*MYH10*	4	-0.22228	0.960678	0.017422
HSPA4L	1	-0.22777	1	0	*NEDD4L*	3	-0.21231	0.960678	0.017422
GIPC2	4	-0.22762	1	0	*METTL3*	4	-0.14651	0.960678	0.017422
SNAI1	3	-0.22736	1	0	*ICE2*	3	-0.13633	0.960678	0.017422
FRMPD1	3	-0.22735	1	0	*NDUFV3*	4	-0.12496	0.960678	0.017422
SCARF1	3	-0.22733	1	0	*FAM53C*	3	-0.093742	0.960678	0.017422
FERMT3	2	-0.22706	1	0	*SMAD6*	2	0.18749	0.960678	0.017422
FIBIN	3	-0.22705	1	0	*SLC25A33*	2	0.20143	0.960678	0.017422
GPC3	3	-0.22697	1	0	*MAPK8*	2	0.22043	0.960678	0.017422

TRIT1	3	-0.22679	1	0	*NPAT*	2	0.24546	0.960678	0.017422
PKNOX2	4	-0.22675	1	0	*ITPR1*	2	0.25999	0.960678	0.017422
RBP1	3	-0.2264	1	0	*ADAMTS10*	2	0.28932	0.960678	0.017422
RPGRIP1	3	-0.22631	1	0	*DCTN6*	2	0.37609	0.960678	0.017422
MTERF2	4	-0.22628	1	0	*TRMT11*	4	-0.027505	0.960893	0.017325
TXNDC5	1	-0.22602	1	0	*ZDHHC12*	4	-0.067546	0.960999	0.017277
LTBP1	4	-0.226	1	0	*MGAT4A*	4	0.082305	0.960999	0.017277
NCKAP1L	2	-0.22598	1	0	*NDUFAF4*	4	0.14186	0.960999	0.017277
BECN2	4	-0.22594	1	0	*INSR*	4	0.14363	0.960999	0.017277
ZNF597	4	-0.22592	1	0	*METTL18*	4	0.17848	0.961855	0.01689
CDHR1	3	-0.2255	1	0	*IFT122*	4	0.26718	0.961855	0.01689
FBXL14	4	-0.2255	1	0	*ERCC1*	3	-0.15351	0.96189	0.016875
PRICKLE2	4	-0.2255	1	0	*SERPINB2*	3	0.007412	0.96189	0.016875
NSL1	3	-0.22533	1	0	*WBP4*	3	0.21102	0.96189	0.016875
KCNB1	4	-0.2253	1	0	*TNFRSF18*	4	0.20936	0.961925	0.016859
MICALL1	4	-0.22511	1	0	*LZIC*	3	-0.21674	0.962139	0.016762
SIAE	3	-0.22505	1	0	*SRPRB*	4	0.036607	0.962174	0.016746
BCAM	3	-0.22494	1	0	*DUOXA2*	4	-0.21883	0.962314	0.016683
METTL6	3	-0.22489	1	0	*CFAP45*	2	-0.20807	0.962314	0.016683
GPRC5A	3	-0.22487	1	0	*CAPN2*	4	-0.10359	0.962314	0.016683
TMEM216	3	-0.22459	1	0	*FAM228A*	4	-0.10305	0.962314	0.016683
GPD1	3	-0.22449	1	0	*EDEM3*	4	-0.044707	0.962314	0.016683
ACE	2	-0.22442	1	0	*RNF150*	4	-0.016797	0.962314	0.016683
KHDC1	3	-0.22432	1	0	*OR2K2*	3	0.026784	0.962314	0.016683
TMEM215	2	-0.22423	1	0	*DPY19L1*	2	-0.37553	0.963787	0.016019
MFSD4A	3	-0.22389	1	0	*FZD4*	1	-0.26704	0.963787	0.016019
HOGA1	4	-0.22366	1	0	*ENKUR*	3	-0.23791	0.963787	0.016019
PGAM2	3	-0.22363	1	0	*NGDN*	3	-0.22807	0.963787	0.016019
TMEM160	2	-0.2234	1	0	*GREB1L*	4	-0.19255	0.963787	0.016019
GPATCH11	3	-0.22326	1	0	*DUSP16*	3	-0.18624	0.963787	0.016019
CCDC181	4	-0.22325	1	0	*DNAH2*	4	-0.18304	0.963787	0.016019
SPON1	3	-0.22274	1	0	*ADGRF1*	4	-0.18047	0.963787	0.016019
GPR25	4	-0.22271	1	0	*DOCK9*	4	-0.17754	0.963787	0.016019
ABITRAM	3	-0.22258	1	0	*CDC20*	3	-0.17162	0.963787	0.016019
LIMD1	4	-0.22234	1	0	*AIP*	4	-0.16707	0.963787	0.016019
DGKQ	3	-0.22209	1	0	*FRA10AC1*	4	-0.1332	0.963787	0.016019
CAMSAP3	1	-0.22208	1	0	*PHF8*	4	-0.11699	0.963787	0.016019
SLIT1	2	-0.22204	1	0	*MVB12A*	4	-0.11103	0.963787	0.016019
OLFML2B	4	-0.22198	1	0	*KMO*	4	-0.099094	0.963787	0.016019
ZRANB2	4	-0.22196	1	0	*ZNF784*	4	-0.085812	0.963787	0.016019
MTERF4	2	-0.22157	1	0	*INSYN1*	4	-0.077467	0.963787	0.016019
SEMA6A	3	-0.22154	1	0	*FANK1*	4	-0.061169	0.963787	0.016019
HR	2	-0.22138	1	0	*ZNF302*	3	-0.053827	0.963787	0.016019
ACVR1	3	-0.22133	1	0	*RPS6KA1*	4	-0.049577	0.963787	0.016019
ARHGEF18	2	-0.22129	1	0	*SH2D2A*	3	-0.038819	0.963787	0.016019
TLK2	2	-0.22107	1	0	*ENDOG*	3	0.005127	0.963787	0.016019
CHST15	4	-0.22059	1	0	*RNF31*	4	0.012025	0.963787	0.016019
ADAM18	2	-0.22043	1	0	*SCN11A*	4	0.058733	0.963787	0.016019
CXorf40B	1	-0.22032	1	0	*BTN1A1*	4	0.10633	0.963787	0.016019
PCMTD1	4	-0.2202	1	0	*TOR2A*	4	0.11379	0.963787	0.016019
FMNL1	2	-0.2201	1	0	*ERVMER34-1*	4	0.1473	0.963787	0.016019
CNPY3	2	-0.22004	1	0	*SALL2*	4	0.15216	0.963787	0.016019
NPTXR	4	-0.22003	1	0	*SLC9A3R1*	4	0.15387	0.963787	0.016019
CRB3	3	-0.22001	1	0	*ZNF737*	2	0.16079	0.963787	0.016019
SLC35G2	3	-0.21986	1	0	*RRAGA*	4	0.17616	0.963787	0.016019
HHLA2	3	-0.21971	1	0	*UBXN8*	3	0.17802	0.963787	0.016019
AGO2	4	-0.21965	1	0	*CHRNA1*	4	0.18019	0.963787	0.016019
CAMK2G	2	-0.21952	1	0	*PRR29*	3	0.20632	0.963787	0.016019

CCL20	4	-0.21944	1	0	*MINDY2*	4	0.21484	0.963787	0.016019
ZNF416	3	-0.21939	1	0	*EGR4*	2	0.22994	0.963787	0.016019
GRPEL2	3	-0.21933	1	0	*EID3*	2	0.24698	0.963787	0.016019
PKIA	4	-0.21931	1	0	*ITGAE*	4	0.26259	0.963787	0.016019
TSPAN2	4	-0.21921	1	0	*EPRS1*	4	0.33527	0.963787	0.016019
PRCP	3	-0.21913	1	0	*ARPC5L*	3	0.17199	0.964284	0.015795
FRS2	3	-0.21912	1	0	*FERMT3*	2	-0.16025	0.964743	0.015588
GLIPR1	4	-0.21896	1	0	*MIP*	4	-0.14098	0.964743	0.015588
SLC26A2	4	-0.21885	1	0	*CC2D1A*	4	0.067615	0.964743	0.015588
DEUP1	3	-0.21872	1	0	*GPRC5D*	4	0.15781	0.964743	0.015588
PKN2	4	-0.21861	1	0	*CSAD*	4	-0.20346	0.964775	0.015574
BMPER	3	-0.21838	1	0	*UHRF2*	4	0.084003	0.964775	0.015574
PKDREJ	4	-0.21838	1	0	*PSMC3IP*	4	0.21087	0.964775	0.015574
LOXL3	3	-0.21816	1	0	*CDK9*	1	0.40105	0.964775	0.015574
HSPA1L	4	-0.21813	1	0	*SP9*	4	-0.21022	0.96527	0.015351
ZNF205	4	-0.21803	1	0	*PAQR8*	2	0.022373	0.96527	0.015351
PLA2G6	4	-0.21795	1	0	*KYAT1*	2	0.061875	0.96527	0.015351
CRHR1	2	-0.21791	1	0	*RPL26L1*	2	-0.46746	0.965295	0.01534
MID1	4	-0.21775	1	0	*SDCBP*	3	-0.37791	0.965295	0.01534
ALG1L2	2	-0.21757	1	0	*FCER2*	3	-0.34697	0.965295	0.01534
ALDH5A1	3	-0.21746	1	0	*RSPH1*	2	-0.33056	0.965295	0.01534
C1orf122	2	-0.21745	1	0	*SLC25A41*	3	-0.22853	0.965295	0.01534
SH3GL3	3	-0.21742	1	0	*CEP55*	4	-0.20291	0.965295	0.01534
ANKRD13B	3	-0.21737	1	0	*DYRK4*	4	-0.09464	0.965295	0.01534
DNAH8	3	-0.21727	1	0	*MFSD12*	3	-0.024191	0.965295	0.01534
TTLL6	4	-0.21709	1	0	*NDFIP1*	4	0.008265	0.965295	0.01534
CALM3	4	-0.21686	1	0	*ERBB2*	3	0.028783	0.965295	0.01534
HLTF	3	-0.21686	1	0	*SEC14L1*	4	0.048666	0.965295	0.01534
TGFB3	2	-0.2168	1	0	*TOPBP1*	3	0.059014	0.965295	0.01534
COX17	3	-0.21665	1	0	*HMCN2*	4	0.15179	0.965295	0.01534
GREB1L	4	-0.21665	1	0	*HOGA1*	4	-0.15062	0.965362	0.01531
AP2A1	3	-0.21658	1	0	*RBM47*	4	-0.13174	0.965362	0.01531
ARHGAP23	3	-0.21603	1	0	*KIF26A*	4	-0.054111	0.965362	0.01531
SELENOW	4	-0.21595	1	0	*ZNF678*	4	-0.003426	0.965362	0.01531
HERC5	3	-0.21591	1	0	*CDH13*	3	0.18756	0.965362	0.01531
NNAT	4	-0.21591	1	0	*IRF5*	4	-0.14267	0.965502	0.015247
USP53	3	-0.21588	1	0	*IGSF3*	4	0.034783	0.965502	0.015247
FMO5	1	-0.21582	1	0	*ARF3*	3	-0.2762	0.965925	0.015057
BTNL10	3	-0.21562	1	0	*CAPNS2*	3	-0.25278	0.965925	0.015057
TTBK2	3	-0.21557	1	0	*TMEM199*	4	-0.4343	0.965967	0.015038
MYEF2	4	-0.21539	1	0	*SPRYD4*	3	-0.34716	0.965967	0.015038
CD6	4	-0.21532	1	0	*KIAA1217*	3	-0.3399	0.965967	0.015038
TCFL5	3	-0.2152	1	0	*MAP3K6*	2	-0.3262	0.965967	0.015038
GALNT14	4	-0.21515	1	0	*CBX4*	4	-0.26473	0.965967	0.015038
ENKUR	3	-0.21476	1	0	*EML5*	4	-0.26163	0.965967	0.015038
ZNHIT2	1	-0.21476	1	0	*RBMXL2*	4	-0.25491	0.965967	0.015038
MIF4GD	4	-0.21471	1	0	*RNF182*	4	-0.23909	0.965967	0.015038
HIRIP3	3	-0.21461	1	0	*DKK3*	4	-0.21999	0.965967	0.015038
PPP1CC	3	-0.21445	1	0	*STK32B*	3	-0.20257	0.965967	0.015038
CHIC1	4	-0.21439	1	0	*APOL3*	4	-0.19487	0.965967	0.015038
SRPX2	4	-0.21434	1	0	*SLC9A7*	4	-0.18538	0.965967	0.015038
WSB2	4	-0.21427	1	0	*ESR1*	4	-0.17698	0.965967	0.015038
PIGC	4	-0.21416	1	0	*FADS2*	3	-0.17156	0.965967	0.015038
PNRC2	2	-0.2138	1	0	*GPAT4*	4	-0.16742	0.965967	0.015038
TMEM43	3	-0.21375	1	0	*TBC1D15*	3	-0.15564	0.965967	0.015038
CCR2	4	-0.21306	1	0	*TMIE*	3	-0.15552	0.965967	0.015038
WNK4	3	-0.21293	1	0	*AOC3*	4	-0.091027	0.965967	0.015038
METTL7B	4	-0.21279	1	0	*VPS4A*	4	-0.069526	0.965967	0.015038

DYSF	4	-0.21278	1	0	*HELZ*	3	-0.033354	0.965967	0.015038
TLR4	3	-0.21264	1	0	*C19orf33*	4	-0.028087	0.965967	0.015038
CEP83	4	-0.21231	1	0	*LAG3*	4	0.025318	0.965967	0.015038
ARAP2	4	-0.2122	1	0	*DCAF12L2*	4	0.039309	0.965967	0.015038
HECTD1	3	-0.21201	1	0	*CES1*	3	0.048964	0.965967	0.015038
RHPN1	2	-0.21198	1	0	*KCNN3*	2	0.054881	0.965967	0.015038
MPIG6B	4	-0.21191	1	0	*IDH2*	4	0.059807	0.965967	0.015038
C2orf69	4	-0.21189	1	0	*PCSK1N*	4	0.074753	0.965967	0.015038
TAS1R3	4	-0.21184	1	0	*CNRIP1*	4	0.094791	0.965967	0.015038
ARHGDIA	3	-0.21139	1	0	*RHOB*	3	0.11513	0.965967	0.015038
ALG6	3	-0.21107	1	0	*TMBIM4*	2	0.16347	0.965967	0.015038
NPNT	3	-0.21092	1	0	*MSTN*	4	0.19068	0.965967	0.015038
OC10013037	2	-0.21066	1	0	*RSBN1L*	4	0.198	0.965967	0.015038
MOSPD3	4	-0.2106	1	0	*COPS7A*	4	0.2116	0.965967	0.015038
WDR93	3	-0.21045	1	0	*MAPK10*	4	-0.12954	0.96653	0.014785
CCDC82	3	-0.2104	1	0	*DLL3*	2	-0.35892	0.966871	0.014632
ADAM22	4	-0.21006	1	0	*TST*	4	-0.26055	0.966871	0.014632
RPAP3	2	-0.21005	1	0	*BCOR*	3	-0.23546	0.966871	0.014632
GEM	4	-0.20999	1	0	*SUSD6*	4	-0.096635	0.966871	0.014632
TTC3	1	-0.20983	1	0	*YOD1*	3	-0.094026	0.966871	0.014632
ST6GALNAC1	4	-0.20974	1	0	*MROH1*	3	-0.018485	0.966871	0.014632
LANCL3	2	-0.20967	1	0	*PDE7B*	3	0.030018	0.966871	0.014632
RALGAPA1	4	-0.20923	1	0	*MSANTD2*	3	0.094256	0.966871	0.014632
GYG1	4	-0.20904	1	0	*SHISA7*	3	0.12737	0.966871	0.014632
ECD	4	-0.209	1	0	*CDC34*	4	0.15632	0.966871	0.014632
DENND10	2	-0.20895	1	0	*C3orf62*	1	0.16662	0.966871	0.014632
PTCD3	4	-0.20887	1	0	*FBXO17*	3	0.18332	0.966871	0.014632
PTPN21	3	-0.20868	1	0	*CALHM1*	4	-0.094016	0.967186	0.01449
SAMHD1	4	-0.20858	1	0	*GPAA1*	4	0.11679	0.967186	0.01449
PRR5L	3	-0.2084	1	0	*ZNF480*	4	-0.19971	0.967247	0.014463
SMAD2	4	-0.20835	1	0	*ROBO3*	4	-0.19222	0.967247	0.014463
CR1L	3	-0.20833	1	0	*ANKIB1*	4	-0.15858	0.967247	0.014463
DTNBP1	2	-0.20829	1	0	*PPP4R3A*	4	-0.13983	0.967247	0.014463
MXI1	4	-0.20815	1	0	*CCDC85A*	4	-0.12338	0.967247	0.014463
ITPKC	4	-0.20795	1	0	*CCDC39*	4	-0.10841	0.967247	0.014463
ANP32B	2	-0.20788	1	0	*MT1G*	4	-0.090947	0.967247	0.014463
HECW1	3	-0.20778	1	0	*ANKRD17*	3	0.10094	0.967247	0.014463
RPE65	3	-0.20772	1	0	*NHLRC3*	3	0.16315	0.967247	0.014463
B4GALNT2	4	-0.20768	1	0	*PCGF3*	4	-0.049338	0.967278	0.014449
TAS2R10	4	-0.20766	1	0	*CNOT1*	4	0.061733	0.967278	0.014449
PDZD9	2	-0.20764	1	0	*MTA2*	4	0.24546	0.967278	0.014449
TDRD6	4	-0.20741	1	0	*COL6A3*	2	0.26764	0.967278	0.014449
G0S2	4	-0.2074	1	0	*SETDB1*	4	-0.2122	0.967696	0.014261
RPL41	3	-0.2074	1	0	*NRIP1*	4	0.047633	0.967696	0.014261
CA12	4	-0.20739	1	0	*SLC22A11*	4	0.081275	0.967696	0.014261
ACE2	4	-0.20733	1	0	*PRKAG3*	4	0.34642	0.967696	0.014261
FAM20A	3	-0.20725	1	0	*NECTIN1*	3	-0.33808	0.967832	0.0142
REC8	4	-0.20722	1	0	*CCDC58*	2	-0.10571	0.967832	0.0142
ZNF843	4	-0.2072	1	0	*TEX55*	4	0.18888	0.967832	0.0142
BCAS2	4	-0.20707	1	0	*ADRM1*	4	0.20391	0.967832	0.0142
CDK5	3	-0.20703	1	0	*DDIT4L*	4	-0.22487	0.96796	0.014143
AQP1	3	-0.2069	1	0	*PNPO*	3	-0.17082	0.96796	0.014143
ZNF727	4	-0.20688	1	0	*LRRC8E*	4	-0.07714	0.96796	0.014143
MAP2K4	3	-0.20685	1	0	*ZNF79*	3	-0.013266	0.96796	0.014143
TES	4	-0.2068	1	0	*FUNDC2*	3	0.001001	0.96796	0.014143
NUTM2F	3	-0.20664	1	0	*WDR45B*	4	0.001291	0.96796	0.014143
TRAF4	4	-0.20623	1	0	*HOXB3*	3	0.019709	0.96796	0.014143
BABAM2	3	-0.20619	1	0	*DCAF8*	4	0.02946	0.96796	0.014143

DUSP2	3	-0.20599	1	0	*MEF2A*	3	0.044578	0.96796	0.014143
ZNF770	2	-0.20592	1	0	*KRT17*	4	0.1046	0.96796	0.014143
MRPS14	4	-0.2058	1	0	*ITGA11*	2	0.20259	0.96796	0.014143
NKX3-1	2	-0.20574	1	0	*PLSCR1*	2	0.24299	0.96796	0.014143
WRAP73	4	-0.20573	1	0	*NOP58*	2	0.097983	0.968025	0.014113
ZNF148	4	-0.20567	1	0	*OLFM2*	2	0.11751	0.968025	0.014113
MARCKS	4	-0.20561	1	0	*TMEM99*	4	0.13974	0.968025	0.014113
RO60	4	-0.20558	1	0	*ADCK5*	4	0.14856	0.968025	0.014113
NGRN	4	-0.20557	1	0	*SOCS4*	4	-0.24066	0.968332	0.013976
ATXN7L3	4	-0.20549	1	0	*MYOM1*	3	-0.15274	0.968332	0.013976
RGS19	2	-0.20535	1	0	*ZNF852*	4	0.083595	0.968332	0.013976
ERLIN2	3	-0.20534	1	0	*KIAA1191*	4	0.1019	0.968332	0.013976
SMIM8	4	-0.2049	1	0	*SCGB1C1*	1	0.15375	0.968332	0.013976
KRT24	4	-0.20459	1	0	*WIPI1*	4	0.17257	0.968332	0.013976
C17orf100	3	-0.20453	1	0	*ACSM3*	4	0.20014	0.968332	0.013976
SFT2D2	4	-0.20429	1	0	*TSPAN6*	1	0.21474	0.968332	0.013976
DDN	4	-0.2042	1	0	*TGFA*	3	0.094556	0.968819	0.013757
ZNF510	4	-0.20414	1	0	*DET1*	2	0.19204	0.968819	0.013757
YTHDF2	4	-0.2041	1	0	*SNAP29*	3	0.37144	0.968819	0.013757
HLA-DPA1	3	-0.20356	1	0	*BTBD2*	4	0.10043	0.969026	0.013664
CFAP74	3	-0.20353	1	0	*SRPRA*	4	0.27539	0.969026	0.013664
MFSD12	3	-0.20335	1	0	*ZDHHC3*	1	-0.30633	0.970145	0.013163
PLVAP	4	-0.20319	1	0	*APOL2*	2	-0.32891	0.970179	0.013148
KLK8	4	-0.20293	1	0	*URB2*	2	0.07606	0.970283	0.013102
RBM17	4	-0.20263	1	0	*PDCD10*	3	-0.29733	0.970487	0.01301
MLST8	2	-0.20261	1	0	*NANP*	3	-0.24111	0.970487	0.01301
TMCO6	3	-0.20233	1	0	*EDN3*	4	0.026557	0.970487	0.01301
SAYSD1	3	-0.20222	1	0	*EBNA1BP2*	2	0.21861	0.970487	0.01301
RPS20	2	-0.2021	1	0	*SEC14L2*	3	-0.29806	0.970729	0.012902
ZNF248	4	-0.20203	1	0	*ARHGAP4*	4	-0.085756	0.970729	0.012902
FAM234B	4	-0.20191	1	0	*FHL3*	4	-0.21389	0.970829	0.012857
ZNF622	4	-0.20188	1	0	*RENBP*	4	0.033079	0.970829	0.012857
UGT1A5	4	-0.20185	1	0	*PSAT1*	4	0.090311	0.970829	0.012857
NEMP2	4	-0.20181	1	0	*GABRG2*	4	0.19482	0.970829	0.012857
DNAJC12	3	-0.20174	1	0	*PAPSS1*	3	0.009751	0.970932	0.012811
COL4A2	3	-0.20161	1	0	*PHGR1*	3	-0.2822	0.971063	0.012752
PLA2G4E	4	-0.20153	1	0	*BHMT2*	4	-0.23627	0.971063	0.012752
KCNH4	3	-0.20136	1	0	*STT3A*	4	-0.019729	0.971063	0.012752
TMEM100	4	-0.2013	1	0	*KLHL13*	4	-0.018623	0.971063	0.012752
NXPE3	4	-0.20094	1	0	*SLC31A2*	4	0.015493	0.971063	0.012752
ITGB1BP2	2	-0.20091	1	0	*OR9A4*	4	0.111	0.971063	0.012752
MUC4	4	-0.20087	1	0	*PCK2*	3	-0.15581	0.971551	0.012534
RSPO4	4	-0.20072	1	0	*C11orf49*	4	-0.30342	0.971633	0.012498
PSMC3	4	-0.20071	1	0	*MIEN1*	4	-0.30182	0.971633	0.012498
CSNK1D	3	-0.20065	1	0	*EPS8L1*	4	-0.28034	0.971633	0.012498
ITGAM	3	-0.20064	1	0	*ATP6V1G2*	1	-0.22626	0.971633	0.012498
CC2D1B	4	-0.20049	1	0	*ITGA5*	4	-0.19605	0.971633	0.012498
DCHS1	3	-0.20031	1	0	*S100A5*	3	-0.18089	0.971633	0.012498
PON2	3	-0.20029	1	0	*NAV1*	4	-0.15472	0.971633	0.012498
ZNF462	3	-0.20024	1	0	*PHYHIPL*	4	-0.12567	0.971633	0.012498
PPP1R3F	3	-0.20023	1	0	*AGFG2*	4	-0.092631	0.971633	0.012498
ASCC2	4	-0.20019	1	0	*AMIGO2*	4	-0.090491	0.971633	0.012498
AQP4	4	-0.20017	1	0	*PPP1R1C*	4	-0.054649	0.971633	0.012498
LEPROTL1	4	-0.20017	1	0	*SCML2*	4	0.061859	0.971633	0.012498
TMEM51	4	-0.20015	1	0	*CUL9*	4	0.089518	0.971633	0.012498
TTC28	3	-0.20011	1	0	*UBTD1*	3	0.098578	0.971633	0.012498
MCTS1	4	-0.20007	1	0	*TGM4*	3	0.24966	0.971633	0.012498
MRFAP1L1	3	-0.19996	1	0	*FAM189A1*	4	0.059386	0.971872	0.012391

CLCN7	4	-0.19995	1	0	*OTULIN*	4	0.13267	0.971872	0.012391
TSKU	3	-0.19978	1	0	*EEF1B2*	1	0.1419	0.971872	0.012391
HPCAL1	4	-0.19961	1	0	*PSMB2*	4	0.1602	0.971872	0.012391
NCL	4	-0.19953	1	0	*CCDC69*	4	-0.062653	0.972246	0.012224
FHAD1	3	-0.19949	1	0	*HOXC9*	3	-0.055386	0.972246	0.012224
PDIA6	4	-0.19945	1	0	*PTPN2*	3	0.015214	0.972246	0.012224
TMEM63B	3	-0.1994	1	0	*DYNLL1*	2	0.061502	0.972246	0.012224
COLEC10	4	-0.19929	1	0	*EZHIP*	3	0.094866	0.972246	0.012224
MELTF	4	-0.19924	1	0	*PLXNA3*	3	0.1358	0.972246	0.012224
HPRT1	4	-0.1992	1	0	*UCHL3*	3	-0.26505	0.972592	0.012069
CYP8B1	4	-0.19915	1	0	*NEURL4*	4	-0.049527	0.97273	0.012008
SMPDL3A	4	-0.19894	1	0	*FLJ45513*	4	-0.33692	0.972936	0.011916
CALD1	4	-0.19871	1	0	*THRAP3*	4	-0.061403	0.972936	0.011916
UBAC1	4	-0.19867	1	0	*FMO4*	4	0.13858	0.973004	0.011885
ABRA	4	-0.19864	1	0	*HNRNPA3*	2	0.25681	0.974534	0.011203
RANBP10	4	-0.19863	1	0	*GPR19*	4	-0.18448	0.9746	0.011174
KYAT3	3	-0.19845	1	0	*MAT2A*	4	-0.12375	0.9746	0.011174
DDX18	3	-0.19828	1	0	*EFEMP1*	2	-0.26345	0.974981	0.011004
VWC2L	4	-0.19796	1	0	*H2AZ2*	2	-0.18616	0.975466	0.010788
UBN2	3	-0.19791	1	0	*ZNF346*	4	-0.083492	0.975951	0.010572
PAN3	4	-0.19772	1	0	*PARP4*	3	-0.18714	0.976051	0.010527
GAREM2	3	-0.19763	1	0	*SIPA1L2*	3	-0.022165	0.976051	0.010527
CDH24	4	-0.1976	1	0	*RIMKLA*	3	-0.089119	0.97615	0.010483
MAP3K20	2	-0.19751	1	0	*TIMM17A*	3	-0.013405	0.97615	0.010483
SMIM15	4	-0.19737	1	0	*MUC5B*	2	0.1258	0.97615	0.010483
COX6B1	4	-0.19725	1	0	*RANBP2*	1	-0.26983	0.976423	0.010362
TMEM30B	4	-0.19719	1	0	*KRT23*	4	-0.21812	0.976423	0.010362
SEMA4A	2	-0.19718	1	0	*NLRX1*	2	-0.16569	0.976423	0.010362
PEX11B	3	-0.19715	1	0	*LOXL4*	4	-0.1466	0.976423	0.010362
PRSS46P	4	-0.19715	1	0	*TMEM217*	3	-0.088207	0.976423	0.010362
MICALCL	3	-0.19711	1	0	*GPA33*	4	-0.075191	0.976423	0.010362
USP46	3	-0.19702	1	0	*TIAM1*	4	-0.03419	0.976423	0.010362
LOC158434	3	-0.19698	1	0	*CTSF*	4	0.020639	0.976423	0.010362
ZNF286A	2	-0.19676	1	0	*DPY19L4*	3	0.06259	0.976423	0.010362
TRIM69	4	-0.19667	1	0	*EIF4EBP3*	3	0.10008	0.976423	0.010362
WWC3	3	-0.19658	1	0	*COX7A1*	4	0.11055	0.976423	0.010362
FASTK	4	-0.19656	1	0	*NRDC*	4	0.11081	0.976423	0.010362
CXXC5	4	-0.1964	1	0	*ZNF274*	3	0.11104	0.976423	0.010362
FNDC5	3	-0.19639	1	0	*RAP1GDS1*	4	0.11794	0.976423	0.010362
CSKMT	3	-0.19638	1	0	*SFXN1*	4	0.12876	0.976423	0.010362
ISCA2	4	-0.19637	1	0	*NEK3*	4	0.1317	0.976423	0.010362
FRA10AC1	4	-0.19622	1	0	*INPP5K*	4	0.14473	0.976423	0.010362
GRID2IP	4	-0.19622	1	0	*MCTP1*	1	0.19657	0.976423	0.010362
SHPK	4	-0.1962	1	0	*BAD*	4	0.30021	0.976423	0.010362
DIS3L2	3	-0.196	1	0	*FCHSD1*	4	0.33085	0.976423	0.010362
ST6GAL2	3	-0.19589	1	0	*CYGB*	3	-0.32612	0.976721	0.010229
EHD2	4	-0.19588	1	0	*C6orf136*	3	-0.31437	0.976721	0.010229
CLDN23	3	-0.19577	1	0	*RGS17*	3	-0.28036	0.976721	0.010229
FCRLA	4	-0.19575	1	0	*SELL*	3	-0.24557	0.976721	0.010229
RSRC1	2	-0.19574	1	0	*EPHB3*	4	-0.1177	0.976721	0.010229
DEF6	4	-0.19567	1	0	*GAPT*	4	-0.077729	0.976721	0.010229
DOCK8-AS1	4	-0.19566	1	0	*ARHGEF5*	2	0.10093	0.976721	0.010229
MPP6	4	-0.1956	1	0	*PLXDC1*	1	-0.4303	0.976778	0.010204
YDJC	4	-0.19559	1	0	*CLU*	4	-0.27713	0.976778	0.010204
PCSK5	3	-0.1954	1	0	*TRPV6*	3	-0.21449	0.976778	0.010204
FKBP10	4	-0.19538	1	0	*ZKSCAN2*	4	-9.82E-05	0.976778	0.010204
TEKT3	3	-0.19537	1	0	*SASS6*	4	0.22852	0.976778	0.010204
BOLA3	1	-0.19535	1	0	*MASTL*	3	0.27327	0.976778	0.010204

OSR1	4	-0.19516	1	0	*ITPR3*	3	-0.27721	0.976809	0.01019
STARD13	3	-0.1951	1	0	*ASB2*	3	-0.15321	0.976809	0.01019
HDAC1	4	-0.19502	1	0	*PLEKHA2*	4	0.098406	0.976841	0.010176
IQCD	4	-0.19499	1	0	*RSPH10B*	1	-0.20652	0.977247	0.009996
SLC25A13	3	-0.19496	1	0	*PLA1A*	3	-0.20432	0.977247	0.009996
EXOC6	4	-0.19472	1	0	*ACOT8*	3	-0.16204	0.977247	0.009996
TMEM199	4	-0.19438	1	0	*IGSF10*	3	-0.094387	0.977247	0.009996
TMPRSS11E	4	-0.19434	1	0	*CTSZ*	4	0.062448	0.977247	0.009996
SMG8	4	-0.19433	1	0	*TAFA3*	3	-0.10283	0.977798	0.009751
SPDEF	2	-0.19418	1	0	*IGSF11*	4	0.046931	0.977935	0.00969
BEGAIN	4	-0.19415	1	0	*TFAP2E*	4	-0.16484	0.977956	0.00968
CBLN1	4	-0.19404	1	0	*HCAR1*	4	-0.13525	0.977956	0.00968
RAB21	2	-0.19384	1	0	*SLC26A4*	3	-0.12485	0.977956	0.00968
KCNS3	4	-0.19379	1	0	*MAGEA8*	4	-0.015615	0.977956	0.00968
DENND6A	4	-0.19377	1	0	*THBS2*	4	0.003908	0.977956	0.00968
TMEM237	3	-0.19363	1	0	*SLF2*	3	0.2193	0.977956	0.00968
GALNT7	4	-0.19345	1	0	*ACSL1*	2	-0.1492	0.978162	0.009589
SLC9C1	4	-0.19321	1	0	*PIGC*	4	-0.093314	0.978436	0.009467
ADAM2	4	-0.19313	1	0	*LILRB3*	3	-0.28901	0.978944	0.009242
ITGB1BP1	4	-0.19299	1	0	*MPPE1*	4	-0.22818	0.978944	0.009242
IER3	4	-0.19298	1	0	*GXYLT2*	4	-0.071875	0.978944	0.009242
H3C12	3	-0.19265	1	0	*ERG28*	4	0.18053	0.978944	0.009242
PDE6A	4	-0.19263	1	0	*KARS1*	4	0.20266	0.978944	0.009242
GSDMB	4	-0.19253	1	0	*HTR2C*	4	-0.27552	0.97902	0.009208
TET3	3	-0.19233	1	0	*GANC*	4	-0.23841	0.97902	0.009208
ZNF562	3	-0.19232	1	0	*ZNF684*	4	-0.2357	0.97902	0.009208
CDH13	3	-0.19219	1	0	*TAPBP*	3	-0.19287	0.97902	0.009208
FBXW12	4	-0.19219	1	0	*GJD3*	4	-0.12134	0.97902	0.009208
XXYLT1	4	-0.19213	1	0	*THAP3*	4	-0.10458	0.97902	0.009208
MUC22	4	-0.19211	1	0	*TBP*	3	-0.016945	0.97902	0.009208
CD244	4	-0.19206	1	0	*UBE2QL1*	4	-7.83E-04	0.97902	0.009208
APBA1	4	-0.19204	1	0	*NOCT*	4	0.043284	0.97902	0.009208
TMEM19	3	-0.19199	1	0	*PLPP7*	4	0.070037	0.97902	0.009208
GPM6B	3	-0.19197	1	0	*CD82*	4	0.15601	0.97902	0.009208
DHRS4	2	-0.19196	1	0	*GTF2H3*	4	0.23873	0.97902	0.009208
SGTB	4	-0.1919	1	0	*PDGFB*	2	-0.28967	0.979121	0.009163
PTPRN2	3	-0.19171	1	0	*TRMT9B*	1	-0.22647	0.979147	0.009152
PKP3	4	-0.19163	1	0	*NPHP4*	4	-0.14356	0.979147	0.009152
KCNQ4	4	-0.19156	1	0	*P3H2*	3	-0.051599	0.979147	0.009152
NKAIN2	4	-0.19148	1	0	*TCHP*	3	-0.044426	0.979147	0.009152
SH2B2	3	-0.19123	1	0	*FAM13B*	3	-0.2699	0.980296	0.008643
VPS9D1	3	-0.19107	1	0	*SLC10A4*	4	-0.2277	0.980296	0.008643
NDUFA4L2	4	-0.19099	1	0	*FGR*	3	-0.19168	0.980296	0.008643
GCHFR	2	-0.19094	1	0	*SDC2*	3	-0.12725	0.980296	0.008643
WIPI2	2	-0.19094	1	0	*C10orf91*	4	0.008728	0.980296	0.008643
PCLAF	4	-0.19093	1	0	*RAB8A*	4	0.077542	0.980296	0.008643
SDHAF3	3	-0.19071	1	0	*CIB1*	4	0.11554	0.980296	0.008643
TAS2R31	4	-0.19058	1	0	*PPM1J*	4	0.14483	0.980296	0.008643
TMEM150A	3	-0.19044	1	0	*APLP2*	2	0.19888	0.980296	0.008643
LY6G5B	4	-0.1904	1	0	*TIMM44*	2	0.37692	0.980296	0.008643
CPVL	4	-0.19013	1	0	*CRYBA4*	3	-0.28356	0.98052	0.008544
RAB6D	2	-0.19006	1	0	*H1-0*	4	-0.19634	0.98052	0.008544
ZDHHC3	1	-0.19005	1	0	*PNPLA3*	3	-0.11774	0.98052	0.008544
PIGG	3	-0.19	1	0	*KIAA1755*	3	0.057644	0.98052	0.008544
TFAP2E	4	-0.18988	1	0	*C3AR1*	3	0.18261	0.98052	0.008544
HNRNPH1	4	-0.18987	1	0	*NAA40*	3	0.19192	0.98052	0.008544
ZMPSTE24	4	-0.18986	1	0	*GLIPR2*	2	0.21718	0.98052	0.008544
H3C10	4	-0.18979	1	0	*FSD2*	2	-0.29718	0.980995	0.008333

USP11	4	-0.18979	1	0	*BROX*	3	0.061217	0.980995	0.008333
UPF2	4	-0.18961	1	0	*ZNF563*	3	0.13847	0.980995	0.008333
ZSCAN30	4	-0.18944	1	0	*USP21*	3	-0.31956	0.981022	0.008321
EHF	4	-0.18939	1	0	*PEPD*	3	-0.27721	0.981022	0.008321
LSS	4	-0.18928	1	0	*KLHL12*	3	-0.27166	0.981022	0.008321
PFN4	4	-0.18928	1	0	*TRIM17*	4	-0.22936	0.981565	0.008081
ATP6V1E2	3	-0.1892	1	0	*MGAT4B*	2	-0.17352	0.981565	0.008081
SRGAP2C	1	-0.18911	1	0	*CYP11A1*	4	0.071398	0.981565	0.008081
LDLRAD2	1	-0.18885	1	0	*SPTBN1*	2	-0.24609	0.981835	0.007962
CRYZ	3	-0.18884	1	0	*LOC10014459*	2	-0.2203	0.981835	0.007962
TRIM33	2	-0.18882	1	0	*CREG2*	2	-0.39797	0.982058	0.007863
FAM169B	3	-0.18876	1	0	*GCNT2*	4	-0.34401	0.982058	0.007863
IFI27	4	-0.18871	1	0	*MOXD1*	4	-0.33657	0.982058	0.007863
NAPEPLD	4	-0.18871	1	0	*ADCYAP1*	3	-0.30693	0.982058	0.007863
HLA-C	3	-0.18863	1	0	*EMX2*	2	-0.29599	0.982058	0.007863
LMAN1	3	-0.18856	1	0	*C12orf75*	4	-0.28685	0.982058	0.007863
CFAP43	3	-0.18839	1	0	*SUSD5*	4	-0.27524	0.982058	0.007863
UBE2F	2	-0.18832	1	0	*TTC32*	1	-0.2663	0.982058	0.007863
ANGPT2	4	-0.18825	1	0	*HRAS*	4	-0.22305	0.982058	0.007863
VPS26A	4	-0.18824	1	0	*CCDC66*	4	-0.21975	0.982058	0.007863
CD27	3	-0.18805	1	0	*TCOF1*	4	-0.2166	0.982058	0.007863
NEURL1	3	-0.18801	1	0	*GZMB*	2	-0.21592	0.982058	0.007863
VN1R5	4	-0.18801	1	0	*LPAR6*	4	-0.21511	0.982058	0.007863
CANT1	2	-0.18795	1	0	*SLC9A3*	2	-0.2117	0.982058	0.007863
CAPN14	3	-0.18786	1	0	*SMIM19*	4	-0.20684	0.982058	0.007863
UBL4B	4	-0.18781	1	0	*ZNF362*	4	-0.18332	0.982058	0.007863
MEX3A	2	-0.18767	1	0	*LMF2*	4	-0.17243	0.982058	0.007863
PTGFRN	3	-0.18765	1	0	*CHM*	4	-0.17185	0.982058	0.007863
CSRNP3	3	-0.1875	1	0	*HES4*	4	-0.14187	0.982058	0.007863
EPX	4	-0.18741	1	0	*PLCB1*	4	-0.12154	0.982058	0.007863
TESK1	4	-0.18731	1	0	*NPRL2*	2	-0.12108	0.982058	0.007863
NUP133	3	-0.18719	1	0	*ARHGEF6*	4	-0.11941	0.982058	0.007863
COL4A5	4	-0.18702	1	0	*ADAMTS6*	4	-0.091668	0.982058	0.007863
ASGR1	4	-0.18697	1	0	*CFB*	4	-0.080282	0.982058	0.007863
CCDC171	4	-0.18695	1	0	*NET1*	4	-0.077018	0.982058	0.007863
UNC93A	4	-0.18694	1	0	*PLAC8L1*	4	-0.068206	0.982058	0.007863
WNT9A	4	-0.18688	1	0	*KLF15*	4	-0.061809	0.982058	0.007863
MTIF2	3	-0.18665	1	0	*NRK*	4	-0.0582	0.982058	0.007863
C1orf43	4	-0.18659	1	0	*RAB3IP*	3	-0.033892	0.982058	0.007863
UBE2L3	1	-0.18657	1	0	*MIER1*	4	-0.015492	0.982058	0.007863
RAG1	4	-0.18655	1	0	*PDK1*	4	-0.010835	0.982058	0.007863
BAZ2B	4	-0.18646	1	0	*ATCAY*	4	-0.009156	0.982058	0.007863
WDR17	4	-0.18639	1	0	*PGAP1*	4	0.002333	0.982058	0.007863
RFESD	2	-0.18629	1	0	*GTF2I*	4	0.044276	0.982058	0.007863
ECI1	3	-0.18587	1	0	*LOC10012969*	4	0.051519	0.982058	0.007863
FLRT3	3	-0.18582	1	0	*DDX25*	3	0.058618	0.982058	0.007863
FANCB	4	-0.18577	1	0	*KITLG*	3	0.068225	0.982058	0.007863
APOBR	4	-0.18567	1	0	*LRRC3C*	4	0.12183	0.982058	0.007863
NFX1	3	-0.18552	1	0	*TADA1*	3	0.14093	0.982058	0.007863
GALR3	3	-0.18544	1	0	*RABIF*	4	0.17205	0.982058	0.007863
CCR3	4	-0.18538	1	0	*PAPSS2*	4	0.1725	0.982058	0.007863
DBT	3	-0.1853	1	0	*LRBA*	4	0.19129	0.982058	0.007863
FAM171A2	3	-0.18528	1	0	*SLC13A4*	4	0.19335	0.982058	0.007863
APH1B	4	-0.1852	1	0	*HGF*	4	0.19775	0.982058	0.007863
ZIK1	4	-0.18516	1	0	*SLC16A9*	4	0.21447	0.982058	0.007863
UBR5	2	-0.18515	1	0	*PWP2*	1	0.21539	0.982058	0.007863
IL7	4	-0.18512	1	0	*PANK4*	2	0.22602	0.982058	0.007863
PYGO1	4	-0.18485	1	0	*ALX4*	4	-0.23286	0.982153	0.007821

WDR44	3	-0.18478	1	0	*CRY2*	4	-0.12257	0.982153	0.007821
ZNF654	2	-0.18475	1	0	*NT5DC1*	4	0.15583	0.982153	0.007821
BTBD10	2	-0.18467	1	0	*RPL13A*	3	-0.27323	0.982337	0.007739
SCFD1	3	-0.18466	1	0	*LRCH4*	3	-0.25317	0.982337	0.007739
CYB5D1	4	-0.18436	1	0	*NPAS3*	4	-0.18863	0.982337	0.007739
DNAAF5	4	-0.1843	1	0	*RAB35*	4	-0.1856	0.982337	0.007739
RIPOR1	3	-0.18425	1	0	*ELFN1*	4	-0.12722	0.982337	0.007739
HNF4A	4	-0.18422	1	0	*SRD5A1*	4	0.036654	0.982337	0.007739
IMMP2L	4	-0.1842	1	0	*AKT2*	3	0.066278	0.982337	0.007739
ELK4	4	-0.18418	1	0	*FSBP*	3	0.071483	0.982337	0.007739
MED6	4	-0.18416	1	0	*NEK7*	2	-0.12263	0.983033	0.007432
MED22	4	-0.18407	1	0	*SLAMF7*	3	-0.10456	0.983033	0.007432
TNFAIP8L1	4	-0.18403	1	0	*LMNB1*	3	-0.069694	0.983033	0.007432
ZNF572	3	-0.18399	1	0	*PTGDR*	3	-0.034759	0.983033	0.007432
THEM6	3	-0.18394	1	0	*CEACAM7*	4	0.013396	0.983033	0.007432
ENPP6	3	-0.18386	1	0	*CASP3*	4	0.044831	0.983033	0.007432
OR7G2	4	-0.18341	1	0	*IFFO2*	3	0.077089	0.983033	0.007432
EDF1	4	-0.18322	1	0	*MYBPC2*	3	0.19776	0.983033	0.007432
PRRG1	3	-0.18294	1	0	*C12orf43*	2	-0.046747	0.983335	0.007298
LNX2	2	-0.18282	1	0	*COX16*	2	0.11857	0.983335	0.007298
S1PR1	3	-0.18258	1	0	*FAM89A*	3	0.026187	0.983435	0.007254
ZNF221	3	-0.18199	1	0	*MAD1L1*	3	-0.42143	0.983494	0.007228
BABAM1	3	-0.18197	1	0	*GDPD3*	3	-0.26171	0.983494	0.007228
KCTD5	3	-0.1819	1	0	*UBLCP1*	2	-0.18298	0.983494	0.007228
MYO1G	4	-0.18175	1	0	*ARL6IP6*	3	-0.14913	0.983494	0.007228
UBAC2	4	-0.18174	1	0	*AMD1*	3	-0.13216	0.983494	0.007228
WIPF2	4	-0.18158	1	0	*AKR1C2*	2	-0.015738	0.983494	0.007228
CABYR	4	-0.18157	1	0	*FIBIN*	3	0.053968	0.983494	0.007228
CXCL2	3	-0.18119	1	0	*KRTAP5-8*	3	0.054028	0.983494	0.007228
CNOT9	4	-0.18107	1	0	*SLAIN1*	2	0.058483	0.983494	0.007228
IER5L	4	-0.1809	1	0	*CREBBP*	4	0.10359	0.983494	0.007228
FAM133B	3	-0.18078	1	0	*SLC35G6*	3	0.10997	0.983494	0.007228
PDE4D	3	-0.18069	1	0	*PELI1*	3	0.11998	0.983494	0.007228
ZNF789	4	-0.18069	1	0	*NUDT8*	4	0.1271	0.983494	0.007228
C1orf112	4	-0.18037	1	0	*ZNF394*	4	0.15345	0.983494	0.007228
DNMT3B	3	-0.18024	1	0	*AMPD2*	3	0.24899	0.983494	0.007228
MAEL	4	-0.18024	1	0	*RANBP9*	2	0.33221	0.983494	0.007228
PSTK	4	-0.18022	1	0	*KIAA1671*	3	-0.30421	0.983727	0.007125
OR5K2	3	-0.18014	1	0	*SPSB1*	3	-0.16885	0.983727	0.007125
PPFIA3	4	-0.17991	1	0	*PIGN*	4	-0.25072	0.984156	0.006936
PELI3	4	-0.17984	1	0	*TALDO1*	3	-0.023508	0.984156	0.006936
CD276	2	-0.17979	1	0	*TNNT2*	2	0.010476	0.984156	0.006936
RECQL	2	-0.17973	1	0	*RAC1*	2	0.022268	0.984156	0.006936
BMT2	3	-0.17967	1	0	*CYP26A1*	4	0.21266	0.984156	0.006936
PNP	3	-0.17964	1	0	*PTX3*	3	-0.22954	0.984174	0.006928
NDUFA1	4	-0.1796	1	0	*MBD3*	3	-0.19851	0.984174	0.006928
TNFAIP1	4	-0.17955	1	0	*DEGS1*	3	-0.082502	0.984174	0.006928
NKAPD1	4	-0.17953	1	0	*NAGA*	3	-0.062314	0.984174	0.006928
INTS11	3	-0.17941	1	0	*SPATA6*	4	-0.051065	0.984174	0.006928
ZNF766	4	-0.17937	1	0	*SLC30A9*	2	0.35474	0.984174	0.006928
DDAH2	4	-0.17933	1	0	*SETD5*	4	-0.33711	0.984197	0.006918
C7orf65	4	-0.17932	1	0	*PRR3*	3	-0.29786	0.984197	0.006918
GSG1L	3	-0.1792	1	0	*SOCS2*	4	-0.10217	0.984197	0.006918
MREG	4	-0.17907	1	0	*ASH1L*	4	0.028921	0.984197	0.006918
DNAJC7	3	-0.17898	1	0	*PPIF*	4	-0.14151	0.984636	0.006724
ABCA5	3	-0.17897	1	0	*RELL2*	4	-0.4411	0.984747	0.006676
ZNF592	3	-0.17886	1	0	*CACTIN*	3	-0.27223	0.984747	0.006676
SH3GLB2	4	-0.17882	1	0	*CTSL*	3	-0.26474	0.984747	0.006676

ACTL8	4	-0.1787	1	0	*ZRANB2*	4	-0.24793	0.984747	0.006676
DNAJC28	4	-0.17867	1	0	*HDHD2*	3	-0.21915	0.984747	0.006676
ARMCX2	4	-0.17863	1	0	*HCRTR1*	3	-0.21494	0.984747	0.006676
GGH	3	-0.17857	1	0	*COL8A2*	3	-0.20505	0.984747	0.006676
IGF2BP3	3	-0.17843	1	0	*FRS2*	3	-0.20214	0.984747	0.006676
PLA2G2F	3	-0.17837	1	0	*DOCK8-AS1*	4	-0.19604	0.984747	0.006676
SALL2	4	-0.17833	1	0	*RAD21L1*	4	-0.18451	0.984747	0.006676
C2CD6	4	-0.1783	1	0	*HIF1A*	4	-0.17895	0.984747	0.006676
FUCA1	4	-0.17829	1	0	*CYP3A43*	3	-0.023011	0.984747	0.006676
RSU1	3	-0.17809	1	0	*LOC10013439*	4	0.022141	0.984747	0.006676
LRRC4	3	-0.17777	1	0	*RHOC*	4	0.061938	0.984747	0.006676
PHACTR2	2	-0.17775	1	0	*SNHG32*	4	0.097711	0.984747	0.006676
SLC6A14	4	-0.17772	1	0	*ATP6V1G3*	4	0.11366	0.984747	0.006676
AHCTF1	4	-0.1777	1	0	*TRIM40*	2	0.13176	0.984747	0.006676
EML6	3	-0.17754	1	0	*TIPIN*	4	0.1962	0.984747	0.006676
C6orf163	4	-0.17745	1	0	*NCAPG2*	4	0.1989	0.984747	0.006676
BAMBI	4	-0.17736	1	0	*C17orf107*	4	0.20145	0.984747	0.006676
BACE1	3	-0.17729	1	0	*PLAC4*	3	0.20522	0.984747	0.006676
ATP8B2	2	-0.17722	1	0	*PPP1R16A*	2	0.013908	0.985117	0.006512
AIMP1	2	-0.17696	1	0	*SCUBE2*	2	-0.099772	0.985216	0.006469
CLEC3B	3	-0.17693	1	0	*BECN2*	4	-0.19852	0.985538	0.006327
CRTC1	3	-0.17685	1	0	*PDE9A*	3	-0.19702	0.985538	0.006327
TMEM132A	3	-0.17684	1	0	*MYO1G*	4	-0.14348	0.985538	0.006327
VPS26B	3	-0.17683	1	0	*ERN2*	4	-0.066906	0.985538	0.006327
MCTP1	1	-0.17682	1	0	*HOXD13*	3	-0.046494	0.985538	0.006327
ANKRD2	3	-0.17677	1	0	*FOXRED2*	3	0.12606	0.985538	0.006327
SH3YL1	3	-0.17668	1	0	*CLDN9*	3	-0.35541	0.985868	0.006181
CLSTN2	4	-0.17661	1	0	*SCG2*	2	-0.29956	0.985868	0.006181
NXN	4	-0.17654	1	0	*AKIP1*	3	-0.28247	0.985868	0.006181
KLHDC8B	4	-0.17652	1	0	*ARHGEF1*	3	-0.2804	0.985868	0.006181
MICAL1	3	-0.17652	1	0	*PACRG*	1	-0.26887	0.985868	0.006181
MINPP1	4	-0.17649	1	0	*MEX3A*	2	-0.26819	0.985868	0.006181
RAB32	3	-0.17643	1	0	*HNRNPH2*	4	-0.23148	0.985868	0.006181
KIF20B	3	-0.17638	1	0	*SLAMF9*	4	-0.22428	0.985868	0.006181
CASQ2	3	-0.17632	1	0	*PTPN20*	3	-0.21131	0.985868	0.006181
CXCR5	3	-0.17624	1	0	*VEPH1*	2	-0.20266	0.985868	0.006181
PRKCI	4	-0.17624	1	0	*ALDH1A3*	1	-0.19302	0.985868	0.006181
SRM	3	-0.17619	1	0	*LOC388780*	4	-0.14828	0.985868	0.006181
SKAP2	3	-0.17614	1	0	*TMEM168*	4	-0.14463	0.985868	0.006181
ZSCAN5B	4	-0.17608	1	0	*SLC35E1*	3	-0.14116	0.985868	0.006181
SLC16A3	4	-0.17602	1	0	*LOC403312*	4	-0.12012	0.985868	0.006181
ZFP69	4	-0.17594	1	0	*DEFB1*	2	-0.10087	0.985868	0.006181
SPEM2	3	-0.17592	1	0	*C17orf58*	4	-0.055433	0.985868	0.006181
C12orf73	4	-0.17584	1	0	*DDB2*	3	-0.029087	0.985868	0.006181
RGS13	4	-0.17575	1	0	*SNCB*	4	-0.026573	0.985868	0.006181
COA7	3	-0.1757	1	0	*ZADH2*	4	-0.00839	0.985868	0.006181
ENC1	4	-0.17565	1	0	*CREB3L3*	4	-0.008215	0.985868	0.006181
SPARC	3	-0.17565	1	0	*CACNA2D3*	4	-0.001287	0.985868	0.006181
MED19	3	-0.17556	1	0	*LCTL*	3	0.028872	0.985868	0.006181
GSKIP	4	-0.1755	1	0	*HEY2*	4	0.049223	0.985868	0.006181
BASP1	2	-0.1753	1	0	*SLC9B2*	4	0.051203	0.985868	0.006181
NOSIP	2	-0.17525	1	0	*C4orf33*	2	0.066986	0.985868	0.006181
BCL7A	4	-0.17523	1	0	*ZIC5*	4	0.082649	0.985868	0.006181
C10orf90	2	-0.17517	1	0	*TTYH3*	4	0.0927	0.985868	0.006181
POLR3A	2	-0.17516	1	0	*CMTM8*	4	0.097579	0.985868	0.006181
PPIB	4	-0.17512	1	0	*MOCOS*	4	0.12292	0.985868	0.006181
C4BPA	4	-0.17502	1	0	*ARSA*	4	0.12603	0.985868	0.006181
C8orf31	1	-0.17501	1	0	*AZGP1*	4	0.12809	0.985868	0.006181

HMOX1	4	-0.175	1	0	*STAM*	4	0.1314	0.985868	0.006181
HKDC1	4	-0.17475	1	0	*NADSYN1*	3	0.13385	0.985868	0.006181
CSTA	3	-0.17462	1	0	*FNDC11*	4	0.13972	0.985868	0.006181
FAM219A	3	-0.17459	1	0	*UXS1*	4	0.14906	0.985868	0.006181
EEFSEC	4	-0.17458	1	0	*SGMS1*	4	0.15221	0.985868	0.006181
ENO4	3	-0.17454	1	0	*RANBP10*	4	0.16468	0.985868	0.006181
C3orf70	3	-0.17448	1	0	*TAF11*	4	0.16501	0.985868	0.006181
EPB41L4A	3	-0.1741	1	0	*FAF2*	4	0.17622	0.985868	0.006181
CCDC155	3	-0.17408	1	0	*NRSN2*	2	0.18904	0.985868	0.006181
FAM220A	3	-0.17407	1	0	*GAS1*	4	0.19512	0.985868	0.006181
PNRC1	2	-0.17392	1	0	*ARHGAP15*	4	0.21325	0.985868	0.006181
HOXC4	4	-0.17384	1	0	*CXorf58*	4	0.21609	0.985868	0.006181
CDH20	4	-0.17375	1	0	*HAUS3*	2	0.24329	0.985868	0.006181
C6orf118	4	-0.17374	1	0	*UBAP2*	2	0.2903	0.985868	0.006181
TAS2R13	3	-0.1734	1	0	*EIF2S2*	2	0.32412	0.985868	0.006181
STK17B	3	-0.17319	1	0	*NOL12*	4	0.41169	0.985868	0.006181
NALCN	3	-0.17318	1	0	*RPL36*	2	0.43626	0.985868	0.006181
PLCH2	3	-0.17316	1	0	*FKBP2*	3	-0.40202	0.985958	0.006142
UBIAD1	3	-0.17309	1	0	*IPO11*	4	-0.028947	0.985958	0.006142
TMEM45B	3	-0.17282	1	0	*ROBO1*	4	0.038807	0.985958	0.006142
H2AC12	4	-0.17274	1	0	*RPL13*	3	0.44406	0.985958	0.006142
GAS2	4	-0.17265	1	0	*GPX3*	4	-0.35559	0.986427	0.005935
USHBP1	3	-0.1726	1	0	*ELK4*	4	-0.24476	0.987061	0.005656
MX2	3	-0.17259	1	0	*EID2*	2	0.10005	0.987061	0.005656
NUDT7	4	-0.17245	1	0	*GCAT*	4	-0.039724	0.987257	0.00557
SLC51A	4	-0.17244	1	0	*GPR155*	4	0.14076	0.987257	0.00557
KIAA1107	4	-0.17237	1	0	*PARG*	4	-0.39243	0.987385	0.005513
ZNF552	4	-0.17235	1	0	*BRD8*	3	-0.31332	0.987385	0.005513
MED28	3	-0.17234	1	0	*HSDL2*	2	-0.26329	0.987385	0.005513
VPS35	3	-0.1723	1	0	*ME1*	3	-0.25527	0.987385	0.005513
GJA1	4	-0.17226	1	0	*TMEM179B*	4	-0.25258	0.987385	0.005513
TAB1	2	-0.17206	1	0	*HCFC2*	2	-0.23941	0.987385	0.005513
CD2AP	3	-0.1719	1	0	*UBQLN4*	4	-0.20094	0.987385	0.005513
SEPTIN12	4	-0.17188	1	0	*SLC3A1*	4	-0.17945	0.987385	0.005513
GPR179	4	-0.17173	1	0	*YPEL3*	3	-0.15414	0.987385	0.005513
REXO4	2	-0.17171	1	0	*IGF2BP3*	3	-0.15114	0.987385	0.005513
CPNE5	3	-0.17165	1	0	*LMBR1L*	3	-0.13782	0.987385	0.005513
BLCAP	4	-0.17157	1	0	*MMP15*	4	-0.077086	0.987385	0.005513
GRIK2	4	-0.17147	1	0	*FRZB*	3	-0.063794	0.987385	0.005513
SUSD3	3	-0.17146	1	0	*TRIP10*	4	0.008027	0.987385	0.005513
NFIL3	4	-0.17135	1	0	*NLRC4*	4	0.053341	0.987385	0.005513
CAMTA1	2	-0.17133	1	0	*CACNA2D1*	3	0.088948	0.987385	0.005513
TRIM29	2	-0.17129	1	0	*SMARCAD1*	4	0.11446	0.987385	0.005513
MXRA8	3	-0.17128	1	0	*IL1RL1*	4	0.14307	0.987385	0.005513
GPHA2	2	-0.17108	1	0	*UBE2Q2L*	1	0.15252	0.987385	0.005513
CLEC5A	3	-0.17103	1	0	*PRUNE2*	3	0.1528	0.987385	0.005513
IL4I1	4	-0.17088	1	0	*PDPK1*	1	0.16014	0.987385	0.005513
ACOT1	4	-0.17084	1	0	*ZNF185*	1	0.16585	0.987385	0.005513
FBXO7	3	-0.17084	1	0	*ARMCX1*	2	0.23301	0.987385	0.005513
IMPDH1	2	-0.17083	1	0	*C1orf131*	4	0.35053	0.987385	0.005513
CLCN2	3	-0.17081	1	0	*DCXR*	4	-0.21964	0.987535	0.005448
MTERF1	3	-0.17079	1	0	*CFL2*	4	-0.10592	0.987535	0.005448
SLC38A4	2	-0.1707	1	0	*ARAP2*	4	-0.047513	0.987535	0.005448
RNH1	1	-0.17061	1	0	*LPA*	4	0.034584	0.987535	0.005448
SNAPC1	4	-0.17052	1	0	*ANKRD18B*	4	0.048565	0.987535	0.005448
RDH5	1	-0.1705	1	0	*CHP2*	4	0.14139	0.987535	0.005448
IGIP	4	-0.17049	1	0	*DSC3*	4	-0.19411	0.987965	0.005258
STON2	4	-0.17047	1	0	*AFG1L*	4	-0.15537	0.987965	0.005258

VIPAS39	4	-0.1703	1	0	*CBR3*	2	-0.1467	0.987974	0.005255
KIAA1522	4	-0.17028	1	0	*NSMCE1*	4	0.011185	0.987974	0.005255
TLR9	4	-0.17025	1	0	*SOWAHB*	4	0.036005	0.987974	0.005255
ECRG4	3	-0.17018	1	0	*FAM102B*	3	0.061998	0.987974	0.005255
GLCCI1	4	-0.17012	1	0	*IL1R1*	4	0.096944	0.987974	0.005255
NSD1	4	-0.17011	1	0	*VPS37C*	4	0.17564	0.987974	0.005255
C12orf56	3	-0.17009	1	0	*ARMC5*	4	0.1769	0.987974	0.005255
CD207	4	-0.17003	1	0	*ARID4B*	4	0.17755	0.987974	0.005255
MRPL58	4	-0.16969	1	0	*GLUD1*	4	-0.15831	0.988452	0.005045
GABRG2	4	-0.16943	1	0	*LOC283710*	4	-0.003499	0.988452	0.005045
TNFAIP6	3	-0.16938	1	0	*HMGCL*	4	0.036487	0.988452	0.005045
ZNF382	3	-0.16929	1	0	*GPATCH8*	4	0.056132	0.988452	0.005045
ODF3L1	2	-0.16917	1	0	*ONECUT3*	4	0.058577	0.988452	0.005045
NR1H3	4	-0.16906	1	0	*PLB1*	4	0.08382	0.988452	0.005045
PLAGL1	1	-0.16899	1	0	*HGC6.3*	1	0.13245	0.988452	0.005045
NRCAM	4	-0.16893	1	0	*PEX26*	4	0.15357	0.988452	0.005045
CHST6	4	-0.1687	1	0	*SZRD1*	2	-0.27644	0.9885	0.005023
TIMM21	3	-0.16866	1	0	*PCP4*	4	-0.14056	0.9885	0.005023
TTC8	2	-0.16865	1	0	*TSPYL2*	2	-0.062136	0.9885	0.005023
TMEM184C	4	-0.16857	1	0	*ZMIZ2*	3	0.020468	0.9885	0.005023
EMX1	4	-0.16845	1	0	*ANXA2R*	2	0.22622	0.9885	0.005023
TMSB15B	4	-0.16843	1	0	*SLPI*	2	0.26246	0.9885	0.005023
AVL9	3	-0.16841	1	0	*TIFA*	4	-0.17741	0.988645	0.00496
SMIM6	2	-0.1684	1	0	*MISP3*	3	-0.11086	0.988645	0.00496
MAP3K4	3	-0.16835	1	0	*SULT2B1*	4	-0.036372	0.988645	0.00496
IL9R	4	-0.16828	1	0	*CD248*	4	0.11189	0.988645	0.00496
PIK3R2	4	-0.16819	1	0	*EFHD2*	4	0.12511	0.988645	0.00496
EZH2	3	-0.16817	1	0	*CHST2*	4	0.12764	0.988645	0.00496
ZNF501	4	-0.16817	1	0	*NLRP13*	4	0.15209	0.988645	0.00496
NEK5	4	-0.16807	1	0	*ANO7*	3	0.068782	0.988809	0.004888
FAM180B	3	-0.16804	1	0	*NRP1*	4	-0.1846	0.989141	0.004742
MDM4	2	-0.16792	1	0	*FCMR*	2	-0.076384	0.989171	0.004729
TUBGCP5	4	-0.16789	1	0	*IFT172*	3	-0.065673	0.989556	0.00456
LIMCH1	3	-0.16787	1	0	*SIGLEC10*	3	-0.014071	0.989556	0.00456
C9orf62	4	-0.16784	1	0	*DIPK1C*	4	0.017525	0.989556	0.00456
MYD88	4	-0.16771	1	0	*CCSER2*	4	0.12164	0.989556	0.00456
METTL23	3	-0.16766	1	0	*PPP1R9B*	4	0.12763	0.989556	0.00456
GLO1	4	-0.16763	1	0	*AGO1*	3	-0.23646	0.989954	0.004385
IL17F	4	-0.16751	1	0	*LIN37*	4	0.083406	0.990118	0.004313
IMPACT	4	-0.16746	1	0	*SEMA3D*	4	-0.14991	0.990249	0.004256
NEO1	4	-0.16731	1	0	*SEC22C*	4	0.18808	0.99068	0.004067
CCDC91	2	-0.16726	1	0	*ADAMTS20*	2	-0.26781	0.991126	0.003871
PRR36	4	-0.16722	1	0	*ZMPSTE24*	4	-0.24904	0.991126	0.003871
B3GALT6	4	-0.16721	1	0	*REEP1*	3	-0.15363	0.991126	0.003871
RPS27L	4	-0.16715	1	0	*MYO7A*	3	-0.15026	0.991126	0.003871
XKR5	4	-0.16705	1	0	*SLC23A1*	3	-0.12471	0.991126	0.003871
DPY19L4	3	-0.16657	1	0	*RAI1*	4	-0.0829	0.991126	0.003871
PTK6	2	-0.16646	1	0	*MARCHF3*	4	-0.0793	0.991126	0.003871
KIF2C	4	-0.16643	1	0	*ZNF350*	3	-0.046297	0.991126	0.003871
WDFY3	4	-0.16618	1	0	*PPFIBP1*	3	0.035558	0.991126	0.003871
BRD2	2	-0.16615	1	0	*FBXL20*	3	0.036086	0.991126	0.003871
CREB3L2-AS1	4	-0.16604	1	0	*DSP*	4	0.055337	0.991126	0.003871
SEMA3D	4	-0.16591	1	0	*CSNK1G1*	3	0.13717	0.991126	0.003871
AARD	3	-0.16584	1	0	*ERCC2*	4	0.14925	0.991126	0.003871
FNDC1	4	-0.16573	1	0	*FUOM*	4	0.18025	0.991126	0.003871
LINC02693	4	-0.16564	1	0	*RHBDF2*	2	0.27567	0.991126	0.003871
GSG1	3	-0.16551	1	0	*GPS1*	3	0.60771	0.991126	0.003871
ZNF775	4	-0.16538	1	0	*FYTTD1*	4	0.14096	0.991223	0.003829

KTN1	3	-0.16537	1	0	*AEBP2*	3	-0.17517	0.991517	0.0037
SLC19A1	3	-0.16531	1	0	*RNF103*	3	0.074126	0.991517	0.0037
MAP3K8	3	-0.16529	1	0	*ERAS*	2	-0.28998	0.991847	0.003555
MLF1	4	-0.16525	1	0	*ASS1*	3	0.053487	0.991977	0.003499
MYBPC1	4	-0.16523	1	0	*PSME3IP1*	4	-0.18052	0.991996	0.00349
LPAR2	3	-0.16522	1	0	*RASSF3*	4	-0.13937	0.991996	0.00349
HRH1	4	-0.16516	1	0	*PRXL2A*	4	-0.13879	0.991996	0.00349
NKX6-1	4	-0.16508	1	0	*ANO6*	4	-0.097044	0.991996	0.00349
RFFL	4	-0.16482	1	0	*MFNG*	3	-0.060381	0.992026	0.003477
SERPINA5	4	-0.1648	1	0	*KLHL29*	4	-0.19371	0.992085	0.003451
MARCHF4	4	-0.16478	1	0	*DMC1*	4	-0.06932	0.992085	0.003451
C16orf72	3	-0.16473	1	0	*SELENOW*	4	0.03934	0.992141	0.003426
KIF24	2	-0.16471	1	0	*AGL*	4	0.13292	0.992141	0.003426
SLC15A4	3	-0.16466	1	0	*CRYBB3*	4	0.20079	0.992141	0.003426
TRAF1	3	-0.16459	1	0	*DCST1*	4	-0.21581	0.992553	0.003246
TMEM54	3	-0.16445	1	0	*USP17L15*	1	-0.20439	0.992553	0.003246
SORL1	3	-0.16439	1	0	*SPATA18*	4	-0.14645	0.992553	0.003246
PDCD1LG2	4	-0.16431	1	0	*PKDCC*	2	-0.13639	0.992553	0.003246
PPP5D1	3	-0.16427	1	0	*INTS9*	4	-0.12201	0.992553	0.003246
IRF2	3	-0.16408	1	0	*SAV1*	3	-0.11814	0.992553	0.003246
LRRC43	2	-0.16401	1	0	*GMPR2*	3	0.10446	0.99265	0.003204
NAT1	4	-0.1639	1	0	*ZFAND3*	4	-0.15939	0.992739	0.003165
PAGE1	4	-0.16387	1	0	*ALOX5AP*	4	-0.1437	0.992739	0.003165
F8	4	-0.1638	1	0	*ARL14EP*	4	-0.093423	0.992739	0.003165
MKKS	4	-0.16376	1	0	*ASTE1*	1	-0.21079	0.992768	0.003152
S100A7A	2	-0.16374	1	0	*HEYL*	2	-0.19547	0.993061	0.003024
LAIR1	3	-0.16354	1	0	*C16orf87*	3	-0.15714	0.993061	0.003024
PRSS56	2	-0.16351	1	0	*EDC3*	3	-0.15469	0.993061	0.003024
H2AB1	1	-0.16345	1	0	*CCDC151*	4	-0.13449	0.993061	0.003024
DENND2A	4	-0.16337	1	0	*SKAP2*	3	-0.057919	0.993061	0.003024
ZNF670	3	-0.16332	1	0	*STOML1*	3	-0.036501	0.993061	0.003024
YY1AP1	3	-0.16321	1	0	*METTL7B*	4	0.06555	0.993061	0.003024
TRAK2	4	-0.16315	1	0	*KIF1C*	3	0.075791	0.993061	0.003024
CDC7	4	-0.16311	1	0	*KRBOX1*	3	0.1511	0.993061	0.003024
IKBKE	4	-0.16308	1	0	*ADCY7*	3	0.15527	0.993061	0.003024
PSMD5	4	-0.16303	1	0	*SLC38A1*	3	0.16645	0.993061	0.003024
CUEDC1	4	-0.16295	1	0	*BIN2*	2	-0.25909	0.993911	0.002653
KSR2	4	-0.1629	1	0	*KCNMB4*	3	-0.14857	0.993911	0.002653
RHOT2	3	-0.16289	1	0	*ELMO3*	3	-0.1073	0.993911	0.002653
ZNF75A	3	-0.1628	1	0	*DGKZ*	3	-0.10673	0.993911	0.002653
OR52W1	3	-0.16273	1	0	*ITCH*	3	-0.089416	0.993911	0.002653
INPPL1	3	-0.16272	1	0	*C17orf77*	3	-0.012025	0.993911	0.002653
DIAPH1	4	-0.16262	1	0	*INCENP*	4	0.083167	0.993911	0.002653
DDB2	3	-0.1626	1	0	*FBXL2*	3	0.14171	0.993911	0.002653
AP3S1	3	-0.16255	1	0	*DGLUCY*	3	0.15551	0.993911	0.002653
PTMS	1	-0.16252	1	0	*KCNMB2*	4	0.15607	0.993911	0.002653
DEAF1	2	-0.1623	1	0	*PRMT1*	4	0.2311	0.993911	0.002653
IL6ST	4	-0.16221	1	0	*ATP13A3*	4	0.25775	0.993911	0.002653
SNX5	4	-0.16218	1	0	*CCT7*	4	0.29202	0.993911	0.002653
ZDHHC7	4	-0.16211	1	0	*TMEM81*	2	-0.2978	0.993974	0.002625
IKBKB	3	-0.1621	1	0	*ARHGEF2*	3	-0.2223	0.994029	0.002601
POU2F1	4	-0.162	1	0	*UBALD1*	4	-0.11816	0.994029	0.002601
NSG1	3	-0.16194	1	0	*OXR1*	4	-0.11707	0.994029	0.002601
1-Mar	3	-0.16187	1	0	*TMEM19*	3	-0.16678	0.994084	0.002577
POTEI	2	-0.16164	1	0	*GHR*	4	-0.14217	0.994084	0.002577
RAB14	4	-0.16158	1	0	*PTP4A3*	4	-0.13815	0.994084	0.002577
GPR180	4	-0.16157	1	0	*TAF1L*	2	0.12388	0.994147	0.00255
ARL16	4	-0.16151	1	0	*COX7A2*	4	-0.33772	0.994244	0.002507

ABHD16B	3	-0.16148	1	0	*DLGAP1*	4	-0.31202	0.994244	0.002507
ARHGAP5	2	-0.16142	1	0	*COPS9*	2	-0.30838	0.994244	0.002507
ZNF184	4	-0.16138	1	0	*ABO*	4	-0.25915	0.994244	0.002507
HSF5	3	-0.1612	1	0	*KLK8*	4	-0.21398	0.994244	0.002507
LRRTM2	4	-0.16096	1	0	*ECHDC2*	4	-0.21232	0.994244	0.002507
RAB3GAP1	2	-0.16094	1	0	*PLPP5*	4	-0.20322	0.994244	0.002507
GAL3ST2	4	-0.16084	1	0	*PSD4*	4	-0.20008	0.994244	0.002507
TMEM74	4	-0.16084	1	0	*HABP4*	3	-0.16883	0.994244	0.002507
LYRM2	4	-0.1608	1	0	*SHMT1*	3	-0.16461	0.994244	0.002507
CDH4	3	-0.16075	1	0	*GABRA2*	4	-0.13452	0.994244	0.002507
PRAME	3	-0.16075	1	0	*CELF1*	3	-0.12818	0.994244	0.002507
CDC20	3	-0.16053	1	0	*MAST4*	4	-0.12432	0.994244	0.002507
CTNND2	3	-0.16051	1	0	*PARP16*	4	-0.11742	0.994244	0.002507
COG2	3	-0.16018	1	0	*WDR6*	3	-0.08754	0.994244	0.002507
ZNF709	4	-0.16016	1	0	*RPS4Y1*	4	-0.060604	0.994244	0.002507
ALDH18A1	3	-0.16001	1	0	*XBP1*	4	-0.048187	0.994244	0.002507
KRT81	2	-0.15993	1	0	*CXCL16*	4	-0.037419	0.994244	0.002507
MIER2	4	-0.15989	1	0	*H1-10*	3	-0.015469	0.994244	0.002507
ISX	3	-0.15988	1	0	*ENPP6*	3	-0.007885	0.994244	0.002507
CLDN6	2	-0.15981	1	0	*LRP2*	4	-0.0017	0.994244	0.002507
NSMCE1	4	-0.15957	1	0	*SLC18B1*	2	-0.001547	0.994244	0.002507
NECAB1	4	-0.15955	1	0	*RGL2*	4	0.004398	0.994244	0.002507
MOCS1	4	-0.15929	1	0	*PGAP2*	4	0.017904	0.994244	0.002507
GORASP1	4	-0.15917	1	0	*TBRG1*	4	0.025954	0.994244	0.002507
PSIP1	4	-0.15893	1	0	*EPS8*	3	0.037805	0.994244	0.002507
ITGA1	3	-0.15887	1	0	*ONECUT1*	4	0.093619	0.994244	0.002507
ZASP	2	-0.15886	1	0	*CEACAM20*	4	0.095372	0.994244	0.002507
SNX25	4	-0.15873	1	0	*SEMA3A*	4	0.10435	0.994244	0.002507
M6PR	4	-0.1587	1	0	*GPBAR1*	3	0.11506	0.994244	0.002507
SSU72	3	-0.15865	1	0	*APOBEC3C*	4	0.12996	0.994244	0.002507
PCNT	3	-0.15858	1	0	*TMEM37*	4	0.13073	0.994244	0.002507
ADCK2	4	-0.15852	1	0	*GADD45G*	4	0.17622	0.994244	0.002507
RSPH1	2	-0.15851	1	0	*USP18*	4	0.1781	0.994244	0.002507
TBC1D28	3	-0.15838	1	0	*CATSPERB*	4	0.18636	0.994244	0.002507
URM1	3	-0.15832	1	0	*TRMT5*	4	0.19684	0.994244	0.002507
APOL2	2	-0.1583	1	0	*EEFSEC*	4	-0.20896	0.994365	0.002454
MBOAT1	3	-0.15814	1	0	*MALRD1*	4	-0.032117	0.994365	0.002454
RUFY3	3	-0.15803	1	0	*PWWP2A*	4	0.019048	0.994365	0.002454
SH3PXD2B	3	-0.15802	1	0	*SLC30A7*	4	-0.092662	0.994456	0.002414
TSNARE1	2	-0.15794	1	0	*DNAJC18*	4	0.016559	0.994456	0.002414
SASH3	4	-0.15782	1	0	*CDKN1A*	3	-0.00223	0.994746	0.002288
RAP1GAP	4	-0.15781	1	0	*ALPK2*	3	0.22795	0.994746	0.002288
ATG7	4	-0.15772	1	0	*GZMM*	4	-0.20088	0.995024	0.002166
FAM200A	3	-0.15766	1	0	*C19orf67*	3	-0.18185	0.995024	0.002166
ZNF512B	2	-0.15765	1	0	*EBLN2*	4	-0.18078	0.995024	0.002166
KMT5C	3	-0.15764	1	0	*RMDN1*	2	-0.17408	0.995024	0.002166
SYNE1	4	-0.15757	1	0	*GATM*	4	-0.1478	0.995024	0.002166
PORCN	4	-0.15756	1	0	*OR1N1*	4	-0.12846	0.995024	0.002166
TOR1AIP2	4	-0.15754	1	0	*COL7A1*	4	-0.11294	0.995024	0.002166
TM2D2	3	-0.15752	1	0	*C1orf226*	4	-0.095096	0.995024	0.002166
OTOP3	3	-0.15748	1	0	*PPP1R3D*	4	-0.066806	0.995024	0.002166
ZWILCH	3	-0.1574	1	0	*DSTYK*	3	-0.045233	0.995024	0.002166
ZNF425	3	-0.15738	1	0	*MPDZ*	3	-0.040369	0.995024	0.002166
WDR35	3	-0.15736	1	0	*SLC47A1*	2	-0.040347	0.995024	0.002166
MRPS11	4	-0.15725	1	0	*MME*	3	-0.032444	0.995024	0.002166
PDCL	4	-0.15723	1	0	*PRR25*	4	-0.032212	0.995024	0.002166
HLA-G	3	-0.1572	1	0	*HIVEP3*	2	-0.029075	0.995024	0.002166
SP6	3	-0.15717	1	0	*SNX2*	3	-0.019306	0.995024	0.002166

CABLES1	3	-0.15692	1	0	*PPP1R36*	3	-0.008501	0.995024	0.002166
TBX15	4	-0.15689	1	0	*IL17RA*	2	0.00551	0.995024	0.002166
SNAP91	3	-0.15687	1	0	*IGF2BP1*	4	0.005744	0.995024	0.002166
SPTY2D1	4	-0.15687	1	0	*KBTBD6*	4	0.030804	0.995024	0.002166
PAEP	2	-0.15677	1	0	*EAF2*	3	0.045112	0.995024	0.002166
SYT5	3	-0.15661	1	0	*DIP2A*	2	0.064259	0.995024	0.002166
GABBR1	2	-0.1566	1	0	*UBE2Z*	3	0.067219	0.995024	0.002166
HDAC9	4	-0.15656	1	0	*PEX19*	4	0.081474	0.995024	0.002166
UBALD2	4	-0.15656	1	0	*RFNG*	4	0.086411	0.995024	0.002166
PRKACA	4	-0.15622	1	0	*HEPACAM*	4	0.13977	0.995024	0.002166
ZNF461	4	-0.15621	1	0	*NHLRC4*	3	0.16022	0.995024	0.002166
BPHL	3	-0.15598	1	0	*CIR1*	3	0.17101	0.995024	0.002166
FCER1A	4	-0.15596	1	0	*ANLN*	4	0.23775	0.995024	0.002166
STEAP4	4	-0.15582	1	0	*VPS51*	4	0.315	0.995024	0.002166
ESRRB	4	-0.15576	1	0	*RPS20*	2	0.51917	0.995024	0.002166
LCMT1	3	-0.15568	1	0	*DENND1A*	4	-0.24011	0.995038	0.00216
UQCR11	3	-0.15564	1	0	*SMG1*	3	-0.058649	0.995038	0.00216
IMPA1	4	-0.15559	1	0	*REG4*	4	-0.021047	0.995038	0.00216
TPST2	4	-0.15553	1	0	*C6orf141*	3	0.11816	0.995038	0.00216
SERINC5	4	-0.15546	1	0	*CDX1*	3	0.15016	0.995038	0.00216
C10orf55	3	-0.15538	1	0	*IGDCC3*	4	-0.013917	0.995199	0.00209
ACCS	4	-0.15535	1	0	*H3C2*	3	-0.25734	0.995323	0.002036
GDPD3	3	-0.15523	1	0	*CFAP74*	3	0.162	0.995323	0.002036
ACVRL1	4	-0.15518	1	0	*HSD17B13*	4	0.13842	0.995348	0.002025
DOK7	3	-0.15517	1	0	*RNF24*	4	0.19919	0.995348	0.002025
FHOD1	3	-0.15493	1	0	*RIBC1*	3	-0.055603	0.995641	0.001897
C12orf74	4	-0.15484	1	0	*VAX2*	4	0.21502	0.996163	0.00167
LRCH2	4	-0.1548	1	0	*HAP1*	3	-0.2441	0.996308	0.001606
SOX15	2	-0.15477	1	0	*POP7*	2	-0.21168	0.996308	0.001606
MSH3	4	-0.15459	1	0	*RASL12*	4	-0.14034	0.996308	0.001606
NME9	3	-0.15454	1	0	*REX1BD*	2	0.17282	0.996308	0.001606
PWWP2A	4	-0.15445	1	0	*MXD3*	3	0.26475	0.996308	0.001606
HEATR6	4	-0.15438	1	0	*SCN2A*	4	-0.063861	0.99635	0.001588
ZNF415	4	-0.1543	1	0	*ARSB*	4	-0.013281	0.99635	0.001588
STX5	4	-0.15425	1	0	*SPINT1*	4	-0.002391	0.99635	0.001588
SFRP4	4	-0.15421	1	0	*NEK5*	4	0.003201	0.99635	0.001588
NR2F1	4	-0.15398	1	0	*WDR19*	4	0.11139	0.99635	0.001588
ELP6	4	-0.15384	1	0	*GNA13*	4	0.11143	0.99635	0.001588
SPAG4	3	-0.15383	1	0	*CCDC88B*	2	-0.32965	0.99651	0.001518
CNPPD1	4	-0.15376	1	0	*METTL8*	4	-0.049438	0.996605	0.001477
ITGA2B	4	-0.15375	1	0	*GOLGA8K*	1	-0.46884	0.996618	0.001471
FAM160A1	4	-0.1537	1	0	*LOC10192809*	2	-0.21478	0.996618	0.001471
STARD4	4	-0.15356	1	0	*DBF4*	2	-0.17216	0.996618	0.001471
EPHB2	4	-0.15354	1	0	*BBX*	3	-0.13504	0.996618	0.001471
CD69	3	-0.1535	1	0	*SPPL2B*	2	0.24845	0.996618	0.001471
ESRRG	4	-0.1535	1	0	*HECW1*	3	-0.16201	0.996643	0.00146
DUOXA1	4	-0.15348	1	0	*WDR5*	4	0.27642	0.996643	0.00146
KIF5C	4	-0.15344	1	0	*PPP1R14A*	4	-0.18717	0.99693	0.001335
TMTC1	4	-0.15341	1	0	*SUMF2*	3	-0.12054	0.99693	0.001335
GPNMB	3	-0.15328	1	0	*SLTM*	4	0.0371	0.997287	0.00118
PDE1B	3	-0.15324	1	0	*ZBTB39*	4	-0.26938	0.997985	8.76E-04
SLFN14	3	-0.15316	1	0	*KCNB1*	4	-0.12313	0.997985	8.76E-04
PRAG1	4	-0.15305	1	0	*TRAPPC6B*	4	-0.081056	0.997985	8.76E-04
WNT3	2	-0.15272	1	0	*OSCP1*	4	0.015026	0.997985	8.76E-04
COX8A	4	-0.15264	1	0	*C7orf25*	4	0.12987	0.997985	8.76E-04
DRD4	4	-0.15262	1	0	*AOX1*	3	0.21972	0.997985	8.76E-04
NUP62	2	-0.15242	1	0	*A2ML1*	4	-0.27528	0.998226	7.71E-04
RBMXL2	4	-0.15237	1	0	*ZNF440*	4	-0.25656	0.998226	7.71E-04

PARP14	4	-0.15235	1	0	*SLC17A1*	4	0.009233	0.998226	7.71E-04
ASPHD1	4	-0.15204	1	0	*SLC24A3*	3	0.043021	0.998226	7.71E-04
THAP4	4	-0.15193	1	0	*TMEM44*	4	0.046765	0.998226	7.71E-04
CDKN1A	3	-0.15171	1	0	*PAG1*	3	0.010315	0.998583	6.16E-04
RAB10	4	-0.1517	1	0	*SOCS6*	4	-0.17158	0.999033	4.20E-04
SDK2	4	-0.15161	1	0	*PHGDH*	4	-0.17105	0.999033	4.20E-04
MAD1L1	3	-0.15158	1	0	*NTM*	3	-0.27397	0.999273	3.16E-04
ACYP1	2	-0.15148	1	0	*CYP20A1*	4	-0.27045	0.999273	3.16E-04
DYNLRB2	4	-0.15145	1	0	*BDH2*	2	-0.24953	0.999273	3.16E-04
HSPD1	1	-0.15145	1	0	*YJEFN3*	4	-0.2312	0.999273	3.16E-04
ZNF253	4	-0.15142	1	0	*SOX12*	3	-0.14049	0.999273	3.16E-04
GALNT11	3	-0.15138	1	0	*RBPMS2*	3	-0.1205	0.999273	3.16E-04
CRIP2	3	-0.15125	1	0	*MORF4L2*	4	-0.1023	0.999273	3.16E-04
TNIK	3	-0.1512	1	0	*CHST3*	4	-0.045602	0.999273	3.16E-04
ZFP69B	4	-0.1512	1	0	*ASPSCR1*	3	-0.020731	0.999273	3.16E-04
PHF10	4	-0.15115	1	0	*WDR35*	3	0.03855	0.999273	3.16E-04
UGT8	4	-0.15106	1	0	*DUSP5*	3	0.049892	0.999273	3.16E-04
BARD1	3	-0.15097	1	0	*CAMKMT*	4	0.064889	0.999273	3.16E-04
CEP78	4	-0.15093	1	0	*PITPNA*	4	0.13714	0.999273	3.16E-04
ZBTB34	4	-0.15076	1	0	*DAAM2*	4	-0.13567	0.999534	2.02E-04
FNDC3A	4	-0.15075	1	0	*SNX31*	3	-0.1053	0.999534	2.02E-04
JOSD1	3	-0.15072	1	0	*EPHX4*	3	-0.10264	0.999534	2.02E-04
KIAA1755	3	-0.15066	1	0	*ZNF214*	4	-0.04724	0.999534	2.02E-04
PGC	4	-0.15062	1	0	*ZNF219*	4	-0.045048	0.999534	2.02E-04
PHGR1	3	-0.15061	1	0	*PTCH2*	3	0.05647	0.999534	2.02E-04
C2orf72	1	-0.15049	1	0	*TBC1D3I*	1	0.22755	0.999534	2.02E-04
JAK3	4	-0.15026	1	0	*PMPCA*	4	0.29132	0.999534	2.02E-04
TMEM269	2	-0.15021	1	0	*BIRC5*	2	-0.58871	1	0
BBS7	3	-0.15019	1	0	*CACNB3*	2	-0.49715	1	0
GFOD2	4	-0.15014	1	0	*TAF6*	2	-0.4717	1	0
RASD1	4	-0.15006	1	0	*ARIH1*	3	-0.43561	1	0
SUSD6	4	-0.14999	1	0	*UTP15*	3	-0.42657	1	0
GLOD4	3	-0.14992	1	0	*IL17REL*	4	-0.41037	1	0
GPD1L	4	-0.14992	1	0	*EWSR1*	4	-0.40522	1	0
AKT1S1	4	-0.14983	1	0	*CA4*	4	-0.40363	1	0
CUL5	4	-0.14983	1	0	*RCCD1*	3	-0.38928	1	0
MRPL41	4	-0.1498	1	0	*RBBP8NL*	2	-0.38739	1	0
CSNK1G3	4	-0.14969	1	0	*ASAP3*	2	-0.3866	1	0
S100A11	3	-0.14968	1	0	*MSTO1*	2	-0.38392	1	0
INS-IGF2	4	-0.14963	1	0	*MAP2K4*	3	-0.38368	1	0
CALB2	4	-0.14941	1	0	*TMEM116*	3	-0.37525	1	0
STRIP2	2	-0.14936	1	0	*ZNF318*	3	-0.374	1	0
LBP	3	-0.14925	1	0	*GLT8D2*	3	-0.37195	1	0
ACTG1	3	-0.14923	1	0	*ST6GALNAC5*	4	-0.36896	1	0
CPNE3	3	-0.14911	1	0	*EEF2*	4	-0.36647	1	0
DPF2	4	-0.149	1	0	*GPR63*	3	-0.36467	1	0
KIF14	4	-0.14896	1	0	*GSPT1*	1	-0.3628	1	0
C11orf1	4	-0.14882	1	0	*AREG*	3	-0.35893	1	0
TNS4	4	-0.14858	1	0	*PCP4L1*	4	-0.35826	1	0
SUPT4H1	2	-0.14847	1	0	*LRP1*	4	-0.35789	1	0
DPP7	3	-0.14835	1	0	*TFEB*	3	-0.35457	1	0
ESPNL	4	-0.14835	1	0	*CCDC183*	4	-0.35343	1	0
CTH	3	-0.14834	1	0	*CCDC9B*	2	-0.35296	1	0
MFSD2A	4	-0.14798	1	0	*GPS2*	3	-0.34956	1	0
LRRK1	4	-0.14772	1	0	*TRIM11*	2	-0.34812	1	0
RECK	4	-0.14761	1	0	*ZNF493*	3	-0.34324	1	0
PLGRKT	3	-0.1476	1	0	*SIRT2*	4	-0.34189	1	0
ATP6V0A2	2	-0.14759	1	0	*RAB7A*	3	-0.34028	1	0

EBI3	4	-0.14738	1	0	*LRRC45*	2	-0.338	1	0
MCTP2	4	-0.14726	1	0	*GSR*	4	-0.33763	1	0
LRG1	3	-0.14723	1	0	*TCF4*	2	-0.33623	1	0
MAGED1	4	-0.14722	1	0	*FBXO46*	2	-0.3348	1	0
MDH1	4	-0.1472	1	0	*FPGT*	2	-0.3338	1	0
ZNF367	4	-0.14701	1	0	*SLC44A2*	3	-0.33332	1	0
TLE5	4	-0.14698	1	0	*LIPA*	1	-0.3328	1	0
MICOS10	4	-0.14694	1	0	*PTPN1*	2	-0.33271	1	0
PERM1	2	-0.14691	1	0	*CCM2*	3	-0.32945	1	0
C15orf32	4	-0.14686	1	0	*GGCX*	3	-0.32874	1	0
MTPN	4	-0.14679	1	0	*RBM10*	4	-0.32811	1	0
FAM102B	3	-0.14654	1	0	*SRP68*	3	-0.32292	1	0
CFHR3	4	-0.14642	1	0	*CLEC18B*	3	-0.32281	1	0
ALPK2	3	-0.14609	1	0	*KHK*	2	-0.3228	1	0
SP100	4	-0.14606	1	0	*DMRTA2*	2	-0.32267	1	0
LRRC49	2	-0.14591	1	0	*PRKAR1A*	3	-0.32167	1	0
ABCB8	4	-0.1458	1	0	*GPR162*	4	-0.32037	1	0
SLC38A2	4	-0.14559	1	0	*RBPJ*	4	-0.3191	1	0
TTYH3	4	-0.14552	1	0	*LRRC69*	2	-0.31876	1	0
PHIP	3	-0.14541	1	0	*MED11*	3	-0.31828	1	0
SNIP1	4	-0.14531	1	0	*DEFB125*	2	-0.31711	1	0
NPEPPS	4	-0.14529	1	0	*ARHGEF12*	4	-0.31669	1	0
C1orf162	4	-0.14526	1	0	*HP1BP3*	4	-0.31645	1	0
CASP10	3	-0.14523	1	0	*TRIM8*	2	-0.31642	1	0
GCA	4	-0.14521	1	0	*CDCA7*	2	-0.3146	1	0
SLC44A1	2	-0.14518	1	0	*CFAP36*	4	-0.31348	1	0
ANKMY1	4	-0.14514	1	0	*CRAMP1*	3	-0.31317	1	0
OXLD1	2	-0.1451	1	0	*VRK3*	3	-0.31299	1	0
IFNGR1	3	-0.14494	1	0	*ZNF511*	2	-0.31284	1	0
COMMD4	4	-0.14482	1	0	*SMPD1*	4	-0.31234	1	0
ITPKB	4	-0.14475	1	0	*SMDT1*	4	-0.31201	1	0
SPINK2	4	-0.14472	1	0	*WDR5B*	2	-0.31015	1	0
HLA-F	4	-0.14467	1	0	*RGS19*	2	-0.31	1	0
PPIF	4	-0.14461	1	0	*SLC11A2*	4	-0.30997	1	0
TMEM201	4	-0.14444	1	0	*GINS4*	3	-0.30956	1	0
GORAB	4	-0.14443	1	0	*TLR1*	4	-0.30848	1	0
KHDRBS1	2	-0.14423	1	0	*FLACC1*	3	-0.30846	1	0
PLEKHG2	4	-0.14422	1	0	*PNKD*	2	-0.3078	1	0
DPYSL3	2	-0.14417	1	0	*ZNF449*	3	-0.3071	1	0
DYNLT1	4	-0.14395	1	0	*FBRS*	3	-0.30623	1	0
BCR	4	-0.14394	1	0	*STK32C*	4	-0.30581	1	0
SMARCB1	4	-0.14394	1	0	*ATOH8*	3	-0.30554	1	0
ZNF737	2	-0.14384	1	0	*CRACD*	4	-0.30489	1	0
RUFY1	3	-0.14376	1	0	*TMA16*	4	-0.30475	1	0
SIGLEC1	3	-0.14372	1	0	*ALG3*	4	-0.30456	1	0
ADCY5	2	-0.14371	1	0	*TAC3*	3	-0.30324	1	0
FHL2	4	-0.1437	1	0	*SULT1C2*	2	-0.30301	1	0
TCEAL9	3	-0.1437	1	0	*EXOSC1*	4	-0.30263	1	0
AP3M1	4	-0.14355	1	0	*RAB1A*	4	-0.30257	1	0
TNIP2	2	-0.14338	1	0	*NOM1*	2	-0.30217	1	0
WWC1	4	-0.14337	1	0	*RTF2*	2	-0.30146	1	0
KLHL13	4	-0.14331	1	0	*ALDH3B1*	4	-0.3014	1	0
TRAPPC13	4	-0.14326	1	0	*TTC9B*	2	-0.30076	1	0
AP1S2	2	-0.14321	1	0	*PIGA*	2	-0.30067	1	0
SLFN12	4	-0.14318	1	0	*ACTRT3*	4	-0.29883	1	0
TMEM232	4	-0.14313	1	0	*L2HGDH*	3	-0.29807	1	0
AKAP13	2	-0.14309	1	0	*NUP43*	4	-0.29746	1	0
LRRC6	4	-0.14302	1	0	*CHD8*	3	-0.29697	1	0

PPM1L	4	-0.14291	1	0	*PARD6B*	2	-0.29645	1	0
MAN1C1	4	-0.14284	1	0	*CYP2E1*	4	-0.29608	1	0
H2BC7	3	-0.14256	1	0	*USP3*	2	-0.29537	1	0
SNX29	3	-0.14243	1	0	*HEY1*	4	-0.29434	1	0
TEAD1	4	-0.14239	1	0	*SUB1*	3	-0.2943	1	0
HS3ST1	3	-0.14235	1	0	*TOB2*	3	-0.29318	1	0
TSPAN7	3	-0.1423	1	0	*SIRT5*	2	-0.29291	1	0
DOK4	4	-0.14225	1	0	*DLX3*	2	-0.29268	1	0
CCDC57	3	-0.14221	1	0	*MFSD3*	3	-0.29208	1	0
RETREG1	4	-0.1422	1	0	*AKAP11*	4	-0.28915	1	0
DLGAP5	3	-0.14219	1	0	*VMA21*	2	-0.28849	1	0
LPA	4	-0.14219	1	0	*TEX19*	4	-0.2884	1	0
CCDC40	3	-0.14215	1	0	*EXOG*	4	-0.2883	1	0
PTTG1IP	4	-0.14188	1	0	*PSMC4*	2	-0.28794	1	0
CDC42EP5	4	-0.14173	1	0	*ZNF76*	2	-0.28783	1	0
TLK1	4	-0.14173	1	0	*CCDC142*	3	-0.28693	1	0
DNAJC15	1	-0.14163	1	0	*CNTROB*	3	-0.28599	1	0
NECAB2	4	-0.14161	1	0	*KCNJ2*	4	-0.28586	1	0
TOP3B	3	-0.14158	1	0	*CENPF*	3	-0.28578	1	0
C18orf21	3	-0.1415	1	0	*ZNF565*	3	-0.28517	1	0
GADL1	4	-0.14147	1	0	*TMCO6*	3	-0.28488	1	0
EFNB3	4	-0.14139	1	0	*ARHGAP33*	2	-0.28473	1	0
PCDH19	3	-0.1413	1	0	*GDPD1*	4	-0.28356	1	0
ASCL5	3	-0.14109	1	0	*NBPF9*	2	-0.28343	1	0
ATP6AP1L	4	-0.14104	1	0	*RAB29*	4	-0.28235	1	0
GPR22	4	-0.14103	1	0	*OR2H2*	3	-0.28224	1	0
CDKL1	3	-0.14097	1	0	*POSTN*	3	-0.28199	1	0
ZNF587	1	-0.14077	1	0	*SPTB*	3	-0.28191	1	0
TMEM25	4	-0.14066	1	0	*COMMD1*	4	-0.2813	1	0
DOCK9	4	-0.14029	1	0	*HPSE*	3	-0.28055	1	0
SLC38A7	1	-0.14018	1	0	*ADAM22*	4	-0.28054	1	0
TBC1D29P	3	-0.14008	1	0	*WDR53*	3	-0.27997	1	0
ALKBH1	3	-0.14003	1	0	*IL22RA1*	3	-0.27996	1	0
GTF2E2	4	-0.14002	1	0	*ZBBX*	4	-0.27952	1	0
ZNF500	4	-0.13996	1	0	*LMF1*	3	-0.27936	1	0
PROX2	4	-0.1399	1	0	*PCDHAC2*	4	-0.27848	1	0
FAM47B	4	-0.13987	1	0	*TSHZ2*	3	-0.27755	1	0
HOXC10	2	-0.13981	1	0	*CACNG1*	4	-0.27698	1	0
TLN2	4	-0.13967	1	0	*CENPB*	2	-0.27666	1	0
FOXO3	3	-0.13965	1	0	*PIGM*	4	-0.27654	1	0
NFE2L1	4	-0.13965	1	0	*RNF7*	4	-0.27637	1	0
PCDHGB3	4	-0.13965	1	0	*PKD1L2*	3	-0.27504	1	0
SINHCAF	3	-0.13965	1	0	*CDKN3*	3	-0.2748	1	0
SOX5	4	-0.1396	1	0	*CRLF3*	4	-0.27466	1	0
CDK6	4	-0.13958	1	0	*TAAR6*	3	-0.27434	1	0
ZC3HAV1	4	-0.13958	1	0	*ACOT1*	4	-0.27384	1	0
RASIP1	3	-0.1395	1	0	*GLRB*	4	-0.27331	1	0
TMX1	2	-0.13947	1	0	*PROM1*	3	-0.27298	1	0
DDB1	1	-0.13944	1	0	*CHADL*	2	-0.27259	1	0
DMC1	4	-0.13944	1	0	*ZNF391*	4	-0.27235	1	0
APEX2	4	-0.13931	1	0	*E2F6*	2	-0.27228	1	0
NPRL2	2	-0.1392	1	0	*P2RY13*	4	-0.27211	1	0
CHST13	4	-0.13918	1	0	*TMEM87B*	4	-0.27203	1	0
ABCC3	2	-0.13917	1	0	*PPP1R15B*	3	-0.27202	1	0
APOF	4	-0.13915	1	0	*AKT1S1*	4	-0.27119	1	0
TP53RK	3	-0.13905	1	0	*RPL37A*	1	-0.27082	1	0
DSTYK	3	-0.13893	1	0	*SPINK2*	4	-0.26974	1	0
ERP29	4	-0.13878	1	0	*LGI2*	2	-0.2689	1	0

SH3BGR	3	-0.13876	1	0	*H3C11*	4	-0.26811	1	0
UTP3	3	-0.13875	1	0	*BLVRB*	2	-0.26782	1	0
BMP2K	4	-0.13874	1	0	*TMEM184B*	3	-0.26743	1	0
LRRC8B	3	-0.13869	1	0	*CBWD1*	1	-0.267	1	0
FOXA3	3	-0.13866	1	0	*FAM187B*	3	-0.26666	1	0
CPEB1	4	-0.13865	1	0	*MFSD10*	3	-0.26632	1	0
JSRP1	3	-0.13844	1	0	*EXOC1*	3	-0.26535	1	0
OLFML3	4	-0.13844	1	0	*DCAF15*	3	-0.2653	1	0
LBH	4	-0.13842	1	0	*GGCT*	3	-0.2646	1	0
TAT	4	-0.13834	1	0	*TIGD1*	3	-0.26443	1	0
COL4A1	4	-0.13833	1	0	*ASB8*	3	-0.26437	1	0
ZNF620	4	-0.13832	1	0	*ZNF597*	4	-0.26412	1	0
IFNK	4	-0.13825	1	0	*SP110*	3	-0.26399	1	0
SYN2	3	-0.13824	1	0	*FRAS1*	2	-0.26311	1	0
S100PBP	4	-0.13817	1	0	*ARHGAP29*	4	-0.26271	1	0
STYX	4	-0.13813	1	0	*ABAT*	3	-0.2623	1	0
COPZ2	4	-0.13798	1	0	*POLD1*	2	-0.26208	1	0
AFAP1L1	3	-0.13772	1	0	*NHLRC1*	4	-0.26193	1	0
ERVW-1	3	-0.13766	1	0	*UNC13C*	3	-0.26171	1	0
RFX3	4	-0.13765	1	0	*ZNF442*	3	-0.26103	1	0
RAD52	4	-0.13742	1	0	*TTC3*	1	-0.26051	1	0
EMC9	4	-0.13739	1	0	*ATP6AP2*	3	-0.2601	1	0
HTR2B	2	-0.13732	1	0	*SRD5A3*	4	-0.25965	1	0
PIAS4	4	-0.13724	1	0	*C16orf89*	4	-0.25964	1	0
CSTF2	4	-0.1372	1	0	*S1PR4*	3	-0.25955	1	0
DSEL	3	-0.13715	1	0	*ATPAF1*	4	-0.25921	1	0
HHIP	4	-0.13708	1	0	*PHOSPHO2*	2	-0.25918	1	0
SEPTIN2	4	-0.13705	1	0	*VPS26A*	4	-0.25876	1	0
UGT1A1	4	-0.13694	1	0	*CSDE1*	3	-0.25872	1	0
RAB28	4	-0.13687	1	0	*PCDHB16*	4	-0.25861	1	0
SLC35B2	2	-0.13686	1	0	*ZNF607*	4	-0.25839	1	0
RCOR1	4	-0.1368	1	0	*SCRN3*	4	-0.25805	1	0
SLC4A5	4	-0.13668	1	0	*PHF13*	3	-0.25733	1	0
OGFOD1	4	-0.13665	1	0	*FKBP4*	4	-0.25691	1	0
SPINK4	2	-0.13663	1	0	*SBK1*	4	-0.25627	1	0
TFIP11	4	-0.13661	1	0	*ATAD3B*	2	-0.256	1	0
BCL11B	3	-0.1366	1	0	*SETSIP*	3	-0.25568	1	0
PPP1R13L	3	-0.13643	1	0	*CHSY3*	4	-0.25526	1	0
AMZ2	4	-0.13636	1	0	*GPD2*	4	-0.25516	1	0
ANKRD34B	4	-0.13636	1	0	*TSSC4*	4	-0.25497	1	0
TMEM120B	3	-0.13634	1	0	*ZNF45*	3	-0.25454	1	0
STK32B	3	-0.13625	1	0	*MNX1*	2	-0.25391	1	0
SARDH	4	-0.13623	1	0	*C8orf33*	2	-0.25371	1	0
ANXA9	3	-0.13618	1	0	*SEMA6A*	3	-0.25361	1	0
KPNA5	4	-0.13599	1	0	*MAP3K19*	4	-0.25326	1	0
SREK1	4	-0.13589	1	0	*WDR59*	3	-0.25276	1	0
UCHL5	3	-0.13581	1	0	*ZNF519*	3	-0.2525	1	0
CNDP2	3	-0.13567	1	0	*SCAMP1*	3	-0.25227	1	0
CCDC33	4	-0.13565	1	0	*CEP19*	3	-0.25175	1	0
PLPP5	4	-0.13533	1	0	*WDR47*	3	-0.25107	1	0
GAD1	4	-0.1353	1	0	*MAVS*	3	-0.25064	1	0
SERPING1	4	-0.13516	1	0	*ISYNA1*	4	-0.2506	1	0
ZEB2	4	-0.13512	1	0	*NBR1*	4	-0.25048	1	0
EFCAB11	4	-0.13504	1	0	*PLEKHA8*	3	-0.25014	1	0
EREG	4	-0.13457	1	0	*FAM149B1*	4	-0.24954	1	0
MEI1	4	-0.13445	1	0	*MCEMP1*	4	-0.24908	1	0
TM9SF1	3	-0.13445	1	0	*TBC1D22B*	4	-0.24887	1	0
CCNA1	3	-0.13442	1	0	*YWHAB*	4	-0.24839	1	0

TAP1	4	-0.13432	1	0	*EEF1AKMT1*	4	-0.24815	1	0
CSAG1	4	-0.13397	1	0	*MYDGF*	4	-0.24812	1	0
B3GNTL1	4	-0.13394	1	0	*HHIPL2*	4	-0.24797	1	0
ARID2	2	-0.13391	1	0	*TMTC3*	3	-0.24773	1	0
MYL12B	1	-0.13391	1	0	*RUFY1*	3	-0.24769	1	0
CASP2	2	-0.13389	1	0	*NAXD*	3	-0.2472	1	0
ABI3BP	4	-0.13382	1	0	*POLR2A*	2	-0.24712	1	0
PER2	4	-0.13382	1	0	*GMFB*	4	-0.24708	1	0
IFT74	3	-0.13371	1	0	*KANK3*	4	-0.24695	1	0
LUZP4	3	-0.13367	1	0	*RAB15*	3	-0.2467	1	0
KCTD17	4	-0.13361	1	0	*BBS1*	2	-0.24652	1	0
GATB	4	-0.13357	1	0	*REEP2*	4	-0.24648	1	0
HMGN4	3	-0.13344	1	0	*HRCT1*	4	-0.24612	1	0
F2RL1	4	-0.13336	1	0	*FGL1*	4	-0.24609	1	0
FGFRL1	3	-0.13336	1	0	*RSU1*	3	-0.24572	1	0
ALAS2	2	-0.13335	1	0	*WIPI2*	2	-0.24572	1	0
TCF20	3	-0.13321	1	0	*EPPK1*	2	-0.24563	1	0
ZNF71	4	-0.13319	1	0	*C12orf56*	3	-0.24551	1	0
PHF7	4	-0.13317	1	0	*FAM76B*	4	-0.2455	1	0
KCNH3	4	-0.13299	1	0	*PAK3*	4	-0.24549	1	0
GALNT3	4	-0.13289	1	0	*EPS8L3*	4	-0.24525	1	0
ZNF579	4	-0.13262	1	0	*STX8*	3	-0.24461	1	0
RIPK3	4	-0.13261	1	0	*DNAJC7*	3	-0.24415	1	0
HELZ2	4	-0.13259	1	0	*CNOT7*	3	-0.24388	1	0
SNX10	4	-0.13258	1	0	*XPO1*	2	-0.24379	1	0
ZNF419	4	-0.13251	1	0	*BZW1*	3	-0.2437	1	0
TMF1	4	-0.13243	1	0	*ABI2*	4	-0.24361	1	0
LINC02860	3	-0.13238	1	0	*GRHPR*	3	-0.24289	1	0
SHMT1	3	-0.13197	1	0	*PDZD9*	2	-0.24277	1	0
EHBP1L1	3	-0.13187	1	0	*USP45*	4	-0.24188	1	0
NUCKS1	4	-0.13186	1	0	*CACNG4*	3	-0.24174	1	0
RABEP1	4	-0.1316	1	0	*LCMT1*	3	-0.24138	1	0
TEX261	3	-0.13157	1	0	*SYPL2*	2	-0.24043	1	0
ACAA2	4	-0.13142	1	0	*WIZ*	2	-0.24036	1	0
RNF168	3	-0.13125	1	0	*MAG*	4	-0.24026	1	0
SRSF12	4	-0.13121	1	0	*TUBB3*	4	-0.2399	1	0
ARSJ	3	-0.13119	1	0	*NLRC3*	2	-0.2397	1	0
PSCA	4	-0.13118	1	0	*RASL10A*	4	-0.23935	1	0
PHKB	4	-0.13114	1	0	*MCTP2*	4	-0.239	1	0
C9orf163	4	-0.13113	1	0	*IFITM3*	2	-0.23892	1	0
HSPA14	4	-0.13108	1	0	*FMNL1*	2	-0.23886	1	0
FOXJ3	4	-0.13091	1	0	*DPYSL3*	2	-0.23863	1	0
SNX19	3	-0.13088	1	0	*PLD4*	2	-0.23854	1	0
CCKBR	3	-0.13085	1	0	*CDK18*	4	-0.23853	1	0
MORN4	4	-0.13082	1	0	*THBD*	4	-0.23836	1	0
GNA13	4	-0.13068	1	0	*AHCTF1*	4	-0.23795	1	0
SNAP25	4	-0.13067	1	0	*MORC3*	3	-0.23743	1	0
BMP8B	3	-0.13055	1	0	*KIF2A*	4	-0.23739	1	0
KCTD18	4	-0.13054	1	0	*FBXW11*	2	-0.23735	1	0
PUSL1	4	-0.1305	1	0	*ABCD4*	4	-0.237	1	0
MTERF3	4	-0.13046	1	0	*COL11A1*	4	-0.23699	1	0
UHRF2	4	-0.13042	1	0	*BCDIN3D*	1	-0.23666	1	0
PYCARD	1	-0.13033	1	0	*CEP162*	3	-0.23658	1	0
QPRT	3	-0.13028	1	0	*LANCL3*	2	-0.23636	1	0
TAF3	4	-0.13024	1	0	*ACYP2*	3	-0.23636	1	0
MIEN1	4	-0.13018	1	0	*ANKS1B*	4	-0.23616	1	0
NHLH1	4	-0.13016	1	0	*ARHGAP35*	3	-0.23603	1	0
CSK	4	-0.13011	1	0	*PPM1D*	3	-0.23602	1	0

AP1S1	4	-0.13001	1	0	*DPP4*	3	-0.236	1	0
SP5	1	-0.13001	1	0	*CNIH3*	4	-0.23594	1	0
TAP2	4	-0.12989	1	0	*KIT*	4	-0.2358	1	0
CREG1	2	-0.12983	1	0	*MIGA1*	3	-0.23557	1	0
AOX1	3	-0.12974	1	0	*SMYD2*	4	-0.23504	1	0
SRSF2	1	-0.12965	1	0	*CLN6*	3	-0.23496	1	0
ZFAND2A	4	-0.12958	1	0	*C2CD5*	4	-0.23471	1	0
CAMKK1	4	-0.1295	1	0	*PPP2R2B*	4	-0.23469	1	0
BEST4	4	-0.1294	1	0	*GNB1*	3	-0.23461	1	0
COL3A1	4	-0.12939	1	0	*FOXP2*	3	-0.23408	1	0
MYOZ3	4	-0.12929	1	0	*ACTN2*	3	-0.23371	1	0
SH3RF1	4	-0.12923	1	0	*UNC13B*	4	-0.23367	1	0
MZB1	3	-0.12921	1	0	*KLC4*	3	-0.2334	1	0
C10orf62	4	-0.12919	1	0	*PRRG1*	3	-0.2333	1	0
WBP1L	4	-0.12905	1	0	*LETMD1*	4	-0.23325	1	0
PAPPA2	4	-0.12889	1	0	*CLCN7*	4	-0.23319	1	0
DGKH	3	-0.12884	1	0	*WAPL*	3	-0.23296	1	0
CD44	3	-0.12878	1	0	*SAFB2*	1	-0.23292	1	0
LAMA4	2	-0.12873	1	0	*CRACR2B*	3	-0.23244	1	0
ZNRF3	4	-0.1287	1	0	*TUBA8*	3	-0.23224	1	0
NSUN3	4	-0.12864	1	0	*SSPN*	3	-0.23206	1	0
EPS8	3	-0.12862	1	0	*FBN3*	4	-0.23176	1	0
EML3	2	-0.1286	1	0	*BLVRA*	3	-0.23171	1	0
ACAA1	4	-0.12857	1	0	*ASRGL1*	2	-0.23161	1	0
CD40	4	-0.12857	1	0	*UFC1*	4	-0.23157	1	0
TGIF1	3	-0.12857	1	0	*MSMB*	3	-0.23138	1	0
SVBP	4	-0.12851	1	0	*SUV39H2*	4	-0.23128	1	0
RAB20	4	-0.1284	1	0	*SCAF4*	4	-0.23127	1	0
RABGAP1L	4	-0.1284	1	0	*ABCC3*	2	-0.23104	1	0
OC10050638	2	-0.12825	1	0	*FXR1*	2	-0.2306	1	0
TMED5	1	-0.12817	1	0	*TCF19*	3	-0.23056	1	0
CGB7	1	-0.12814	1	0	*WDR83*	4	-0.23054	1	0
TSPAN15	3	-0.12809	1	0	*SMCO3*	3	-0.23031	1	0
FXN	4	-0.12805	1	0	*WDR49*	3	-0.23006	1	0
VCPKMT	2	-0.12804	1	0	*IL31RA*	4	-0.2298	1	0
GNRHR	3	-0.12796	1	0	*ZNF587B*	2	-0.2297	1	0
PDIA2	4	-0.12788	1	0	*KCND3*	3	-0.22961	1	0
GLRA2	3	-0.12787	1	0	*SLCO1A2*	3	-0.22933	1	0
TSPAN11	4	-0.12777	1	0	*NHSL1*	2	-0.22913	1	0
SLC12A4	4	-0.12761	1	0	*RUNX1*	4	-0.22896	1	0
ADAM11	4	-0.12758	1	0	*TP53RK*	3	-0.22883	1	0
NPAS3	4	-0.12749	1	0	*CLYBL*	1	-0.22875	1	0
GATAD1	3	-0.12733	1	0	*RPP25*	4	-0.22843	1	0
CLEC7A	4	-0.12727	1	0	*C2orf73*	3	-0.22842	1	0
MPEG1	4	-0.12722	1	0	*UNC5B*	3	-0.2283	1	0
SLC38A1	3	-0.12718	1	0	*FBLN7*	4	-0.22808	1	0
KIAA0408	4	-0.12712	1	0	*GRIN1*	2	-0.22777	1	0
AJM1	4	-0.12708	1	0	*HR*	2	-0.22737	1	0
HFE	2	-0.12708	1	0	*ZNF671*	2	-0.2272	1	0
ASPDH	4	-0.1269	1	0	*IFT20*	4	-0.22705	1	0
FGF21	4	-0.1268	1	0	*IFT43*	3	-0.22705	1	0
LRCH4	3	-0.12675	1	0	*SPAG5*	3	-0.22696	1	0
XPO7	3	-0.12673	1	0	*FAM174C*	3	-0.2267	1	0
CLDN4	4	-0.12672	1	0	*COMT*	4	-0.22651	1	0
SLC19A2	4	-0.12671	1	0	*VAMP3*	3	-0.22629	1	0
HOXA4	3	-0.12663	1	0	*MEIS1*	3	-0.22619	1	0
DHX32	4	-0.12661	1	0	*NAT8*	4	-0.22611	1	0
TMEM187	4	-0.12659	1	0	*MISP*	3	-0.22578	1	0

SLC7A8	2	-0.12651	1	0	*VN1R4*	1	-0.22559	1	0
PECAM1	4	-0.12648	1	0	*CCIN*	4	-0.22509	1	0
IFT27	4	-0.12618	1	0	*CRB2*	4	-0.22477	1	0
KLHL20	4	-0.12609	1	0	*TNFAIP1*	4	-0.22473	1	0
FAM186A	4	-0.12607	1	0	*HSPA12A*	3	-0.22469	1	0
THNSL2	4	-0.12605	1	0	*BCAM*	3	-0.22454	1	0
PPP3CC	4	-0.12603	1	0	*FHIT*	4	-0.22437	1	0
ATXN2	4	-0.126	1	0	*CCNE2*	4	-0.22419	1	0
SLC12A5	4	-0.12585	1	0	*CCDC74A*	2	-0.2241	1	0
EXD2	2	-0.12581	1	0	*DOP1B*	4	-0.22408	1	0
GM2A	4	-0.12581	1	0	*NTHL1*	2	-0.22384	1	0
TPSB2	1	-0.12575	1	0	*TEC*	3	-0.22311	1	0
FLI1	3	-0.12574	1	0	*HSPA1L*	4	-0.22308	1	0
SNX11	4	-0.12571	1	0	*ZNF81*	3	-0.22282	1	0
SUPT7L	3	-0.1256	1	0	*STMP1*	3	-0.22271	1	0
FABP5	2	-0.12557	1	0	*UBQLN1*	3	-0.22268	1	0
SPATA21	4	-0.12555	1	0	*AARD*	3	-0.22249	1	0
KLHDC10	4	-0.12552	1	0	*ANKRD44*	3	-0.22242	1	0
CD38	4	-0.1255	1	0	*RHBDD2*	4	-0.22185	1	0
HECTD4	4	-0.12549	1	0	*C12orf29*	4	-0.22183	1	0
CYP24A1	4	-0.12546	1	0	*GALNT18*	3	-0.22169	1	0
HSPBAP1	4	-0.1254	1	0	*DLL1*	3	-0.22169	1	0
DGAT1	4	-0.12533	1	0	*PDIA6*	4	-0.2216	1	0
CD79B	4	-0.12528	1	0	*REC8*	4	-0.22159	1	0
ZIC1	3	-0.12527	1	0	*TCF3*	3	-0.22156	1	0
TACC2	4	-0.12523	1	0	*MAGOHB*	2	-0.22152	1	0
CSRNP2	1	-0.12517	1	0	*ERVFRD-1*	4	-0.22139	1	0
RASSF8	3	-0.12513	1	0	*IL13RA1*	4	-0.22117	1	0
ADRA1D	2	-0.12512	1	0	*IFT57*	4	-0.22112	1	0
FGF18	3	-0.12499	1	0	*DHRSX*	2	-0.22097	1	0
RFX2	4	-0.12493	1	0	*GIT1*	3	-0.22085	1	0
CARMIL1	3	-0.12491	1	0	*ARHGAP45*	4	-0.2208	1	0
VPS8	4	-0.12484	1	0	*MYEF2*	4	-0.22023	1	0
MXD4	3	-0.12478	1	0	*TMOD1*	3	-0.21992	1	0
TXNDC16	4	-0.1247	1	0	*ADAMTS7*	3	-0.21972	1	0
WDR3	4	-0.12469	1	0	*SLC4A9*	2	-0.21955	1	0
CRNN	4	-0.12465	1	0	*GABRG3*	2	-0.21937	1	0
SFI1	4	-0.12459	1	0	*LZTS1*	3	-0.21907	1	0
BRICD5	4	-0.12456	1	0	*PITPNC1*	2	-0.21904	1	0
BRI3BP	3	-0.12453	1	0	*EVI2B*	4	-0.21882	1	0
SCGB1D2	3	-0.1245	1	0	*E2F5*	3	-0.21878	1	0
RNF207	3	-0.12446	1	0	*NARF*	4	-0.21864	1	0
GATA5	4	-0.12444	1	0	*SCNN1B*	3	-0.21827	1	0
TRIB3	4	-0.12437	1	0	*MYBL1*	4	-0.21813	1	0
TBX21	3	-0.12413	1	0	*METTL2B*	1	-0.21798	1	0
ADM2	4	-0.1241	1	0	*CDK11B*	3	-0.21793	1	0
RBM24	2	-0.12407	1	0	*PPP1R3B*	3	-0.2178	1	0
CD302	1	-0.124	1	0	*STARD4*	4	-0.21762	1	0
PPP5C	3	-0.12399	1	0	*PRX*	3	-0.21741	1	0
VIM	3	-0.12394	1	0	*GTSF1*	3	-0.21736	1	0
NAPB	4	-0.12383	1	0	*TMEM88*	3	-0.21652	1	0
MARS2	4	-0.12377	1	0	*ZNF501*	4	-0.21641	1	0
TBX6	3	-0.12376	1	0	*LNPEP*	3	-0.21637	1	0
KCNF1	4	-0.12373	1	0	*PRDX5*	3	-0.21618	1	0
C3orf33	4	-0.12359	1	0	*ETV7*	3	-0.21609	1	0
MRPS6	3	-0.12353	1	0	*ABHD12B*	4	-0.21582	1	0
GPATCH2L	4	-0.12346	1	0	*TRPV1*	3	-0.21575	1	0
AOC2	4	-0.12344	1	0	*FAM83F*	3	-0.2157	1	0

EYA1	4	-0.12338	1	0	*ZNF704*	4	-0.21562	1	0
TMED4	4	-0.12335	1	0	*ABCG1*	3	-0.21537	1	0
TTC38	3	-0.1233	1	0	*ELN*	3	-0.2153	1	0
OR2AE1	4	-0.12325	1	0	*HK2*	3	-0.21528	1	0
TRERF1	3	-0.12319	1	0	*CCL17*	2	-0.21468	1	0
RNF152	4	-0.12308	1	0	*YIF1B*	4	-0.21454	1	0
KIF1B	3	-0.12307	1	0	*PRIMPOL*	3	-0.21433	1	0
RPS9	2	-0.12301	1	0	*MTAP*	4	-0.21407	1	0
POLR3GL	4	-0.12294	1	0	*ST7L*	4	-0.21405	1	0
BTBD16	3	-0.12282	1	0	*TRHR*	4	-0.21382	1	0
HCFC1	2	-0.1228	1	0	*NACC1*	4	-0.21347	1	0
H2AC7	4	-0.12273	1	0	*RAG2*	4	-0.21343	1	0
TSPAN12	4	-0.12271	1	0	*PFKM*	4	-0.21329	1	0
DDX24	4	-0.12255	1	0	*LIMCH1*	3	-0.21325	1	0
EIF5A	2	-0.12249	1	0	*LRRC37A2*	2	-0.21317	1	0
TJAP1	4	-0.12238	1	0	*CHD6*	2	-0.21316	1	0
AGMO	3	-0.12232	1	0	*SIX3*	2	-0.21301	1	0
MMP1	2	-0.1223	1	0	*SLC6A16*	2	-0.21289	1	0
NRGN	4	-0.1221	1	0	*BTN3A2*	3	-0.21284	1	0
SLC25A34	4	-0.12209	1	0	*ETNPPL*	4	-0.212	1	0
CPEB2	3	-0.12206	1	0	*EBF3*	2	-0.21172	1	0
PPP1R14C	4	-0.12204	1	0	*SLC12A8*	3	-0.2117	1	0
IL17RD	3	-0.12193	1	0	*PSMC1*	3	-0.21167	1	0
HNRNPA2B1	4	-0.12192	1	0	*NPPA*	4	-0.21164	1	0
WNT9B	4	-0.12189	1	0	*IL1R2*	3	-0.21152	1	0
CCDC189	3	-0.12184	1	0	*NRXN3*	3	-0.21145	1	0
PHOSPHO1	4	-0.1218	1	0	*ETV1*	3	-0.21142	1	0
SAMD4A	2	-0.12171	1	0	*PRR15*	4	-0.21118	1	0
NEURL4	4	-0.12161	1	0	*TBK1*	4	-0.21113	1	0
CCS	4	-0.12154	1	0	*MAPK9*	4	-0.21071	1	0
ZMIZ1	4	-0.12153	1	0	*MARCHF4*	4	-0.21057	1	0
CCDC184	3	-0.12148	1	0	*ZNF337*	3	-0.21048	1	0
SCOC	4	-0.12148	1	0	*OR8G2P*	2	-0.21015	1	0
FAM171B	4	-0.12144	1	0	*TPH2*	3	-0.20993	1	0
ATP7A	4	-0.12142	1	0	*NCSTN*	2	-0.20987	1	0
FAM118B	4	-0.12138	1	0	*CBY2*	2	-0.20974	1	0
TAF6L	4	-0.12119	1	0	*NVL*	4	-0.20965	1	0
MGAT4B	2	-0.12118	1	0	*PROSER2*	4	-0.20963	1	0
ANXA6	4	-0.12116	1	0	*AP1B1*	2	-0.20952	1	0
VKORC1	4	-0.12115	1	0	*FHOD1*	3	-0.20947	1	0
IDUA	4	-0.12108	1	0	*IRF2BP2*	4	-0.2094	1	0
SEC14L4	4	-0.12104	1	0	*RPS6KA4*	2	-0.20937	1	0
PNMA3	4	-0.12081	1	0	*CNTNAP3B*	1	-0.20917	1	0
CDK5RAP2	4	-0.12075	1	0	*NIPAL4*	4	-0.20908	1	0
SMIM25	3	-0.12074	1	0	*R3HDM2*	3	-0.20907	1	0
YRDC	4	-0.12071	1	0	*ZAP70*	4	-0.20896	1	0
C1orf226	4	-0.1207	1	0	*F10*	3	-0.20895	1	0
TGM4	3	-0.1206	1	0	*MELTF*	4	-0.20876	1	0
RPS4X	3	-0.12055	1	0	*C10orf88*	4	-0.20875	1	0
DVL2	4	-0.12025	1	0	*APLF*	4	-0.20873	1	0
PIH1D3	4	-0.12004	1	0	*WBP1L*	4	-0.20829	1	0
PRUNE1	4	-0.11998	1	0	*H2AZ1*	4	-0.20776	1	0
ACTR5	3	-0.1198	1	0	*RNF130*	2	-0.20772	1	0
DDR1	4	-0.11968	1	0	*ANKRD31*	2	-0.20762	1	0
ZNF740	4	-0.11965	1	0	*KCNF1*	4	-0.20744	1	0
DNAJC3	4	-0.11961	1	0	*SFSWAP*	3	-0.20699	1	0
CDK5R2	4	-0.11958	1	0	*NAMPT*	4	-0.20686	1	0
NAP1L5	4	-0.11958	1	0	*WDSUB1*	3	-0.20685	1	0

LRWD1	4	-0.11952	1	0	*CRH*	4	-0.20675	1	0
CAP2	3	-0.1195	1	0	*ACCS*	4	-0.20661	1	0
RAB11FIP1	3	-0.11938	1	0	*WFDC6*	2	-0.20654	1	0
MYO1F	4	-0.11937	1	0	*SPECC1*	4	-0.20633	1	0
DDIT4L	4	-0.11905	1	0	*PHKB*	4	-0.20593	1	0
TMEM121B	3	-0.11899	1	0	*SH3BGRL2*	4	-0.20591	1	0
SPSB1	3	-0.11882	1	0	*CTF1*	2	-0.20583	1	0
POLR2E	3	-0.11878	1	0	*KRT31*	2	-0.20568	1	0
LRRC39	4	-0.11858	1	0	*SEPTIN2*	4	-0.20565	1	0
PLAAT4	4	-0.11853	1	0	*TIMP4*	4	-0.20551	1	0
SLC9A3R1	4	-0.11845	1	0	*CCNG2*	4	-0.20496	1	0
EGR3	4	-0.11837	1	0	*CEACAM19*	2	-0.2049	1	0
FASTKD2	3	-0.11836	1	0	*S100A12*	4	-0.20478	1	0
QRICH2	4	-0.1183	1	0	*FTL*	2	-0.2046	1	0
LVRN	3	-0.11811	1	0	*DCPS*	3	-0.20453	1	0
PCDHB4	3	-0.11794	1	0	*CHRNB1*	2	-0.20448	1	0
NTNG1	3	-0.11792	1	0	*DENND6B*	4	-0.20445	1	0
ARMCX5	2	-0.11786	1	0	*GABRP*	3	-0.20439	1	0
CLEC4O	4	-0.11782	1	0	*FLJ44635*	3	-0.20436	1	0
ZBTB4	3	-0.11772	1	0	*KIAA0232*	3	-0.20408	1	0
MPPE1	4	-0.11762	1	0	*DDX56*	4	-0.20407	1	0
SFXN4	3	-0.11761	1	0	*KCNH2*	4	-0.20401	1	0
COX6B2	4	-0.1176	1	0	*PI16*	4	-0.204	1	0
CDC42SE2	3	-0.11745	1	0	*ACP1*	3	-0.20389	1	0
GRHPR	3	-0.1174	1	0	*FAM3C*	2	-0.20388	1	0
FSCB	4	-0.11737	1	0	*KSR2*	4	-0.2038	1	0
GATA4	4	-0.11734	1	0	*REEP4*	3	-0.20308	1	0
NDRG2	3	-0.11734	1	0	*MARF1*	4	-0.20305	1	0
UTS2B	4	-0.11728	1	0	*RIPK3*	4	-0.20268	1	0
LRRC20	4	-0.11725	1	0	*SIDT1*	3	-0.20263	1	0
TXK	3	-0.1172	1	0	*ARIH2OS*	3	-0.20259	1	0
CDH3	3	-0.11713	1	0	*DHRS12*	4	-0.20222	1	0
PATL1	3	-0.11711	1	0	*LRP5*	4	-0.20217	1	0
CDH5	4	-0.11701	1	0	*YBX3*	4	-0.20183	1	0
RAB42	2	-0.11683	1	0	*SLC25A42*	4	-0.20175	1	0
LHFPL5	4	-0.11674	1	0	*SUCLG1*	4	-0.20173	1	0
CELA3B	3	-0.11669	1	0	*SHROOM3*	3	-0.2017	1	0
LMBR1L	3	-0.11664	1	0	*CHST15*	4	-0.20164	1	0
SLC48A1	4	-0.11661	1	0	*KCNK12*	4	-0.20157	1	0
TMEM190	2	-0.11656	1	0	*DENND2D*	4	-0.20103	1	0
TRPM1	3	-0.11654	1	0	*TDRD3*	4	-0.20098	1	0
DHRS12	4	-0.11651	1	0	*PRSS22*	4	-0.20082	1	0
PRPSAP2	4	-0.11638	1	0	*ZNF438*	4	-0.20055	1	0
SOX8	4	-0.11628	1	0	*ZNF114*	3	-0.20047	1	0
RPS6KC1	3	-0.11625	1	0	*GOT1*	3	-0.20034	1	0
HYAL1	4	-0.11619	1	0	*ZEB2*	4	-0.20031	1	0
IGFBP6	3	-0.11617	1	0	*PLD2*	4	-0.19994	1	0
BAIAP2L2	4	-0.11616	1	0	*INSIG1*	3	-0.19992	1	0
EPS8L3	4	-0.11605	1	0	*ARID5B*	4	-0.19989	1	0
CNTNAP1	4	-0.116	1	0	*TMCC3*	4	-0.19963	1	0
PPP1R3B	3	-0.11593	1	0	*KIAA1211L*	4	-0.19956	1	0
PIK3C2A	3	-0.11591	1	0	*COA4*	2	-0.19949	1	0
CISD2	2	-0.1157	1	0	*KRBOX4*	2	-0.19936	1	0
UBE2Q1	4	-0.11568	1	0	*SSTR5*	4	-0.19911	1	0
DSC3	4	-0.11553	1	0	*CCDC42*	4	-0.1991	1	0
MS4A3	4	-0.1155	1	0	*VSTM2L*	4	-0.19909	1	0
YARS2	4	-0.1155	1	0	*MCCC2*	4	-0.19908	1	0
PARP15	2	-0.11545	1	0	*CEACAM5*	4	-0.19904	1	0

AP4E1	4	-0.11541	1	0	*S100B*	4	-0.19882	1	0
FBXO48	2	-0.11535	1	0	*ADAP1*	4	-0.19874	1	0
ADAM10	3	-0.11526	1	0	*CYSRT1*	4	-0.19872	1	0
TNFRSF13C	3	-0.1151	1	0	*SHCBP1L*	3	-0.19856	1	0
C11orf68	4	-0.11509	1	0	*DDX4*	3	-0.1984	1	0
PAXIP1	4	-0.11504	1	0	*TYRP1*	4	-0.1984	1	0
GPR55	4	-0.11503	1	0	*SYNRG*	3	-0.19836	1	0
ITGB3BP	4	-0.11502	1	0	*GLOD4*	3	-0.19833	1	0
NAGA	3	-0.11502	1	0	*CAMK2G*	2	-0.19831	1	0
RAB8B	4	-0.11501	1	0	*AP1S2*	2	-0.19815	1	0
PERP	3	-0.11497	1	0	*DTX2*	4	-0.19805	1	0
PPP1R35	1	-0.11497	1	0	*C8orf44*	3	-0.19794	1	0
CENPV	3	-0.11495	1	0	*APOBEC2*	3	-0.19769	1	0
NAP1L1	3	-0.11494	1	0	*SMIM5*	4	-0.19763	1	0
ITGBL1	4	-0.11479	1	0	*DDRGK1*	4	-0.19762	1	0
POGLUT1	3	-0.11459	1	0	*FILIP1*	3	-0.19759	1	0
CYP4F2	3	-0.11453	1	0	*MANEA*	4	-0.19753	1	0
RAI1	4	-0.11453	1	0	*NPR3*	4	-0.19694	1	0
TXN2	3	-0.11451	1	0	*GLS2*	2	-0.19693	1	0
LRRN3	3	-0.11442	1	0	*NUB1*	1	-0.19651	1	0
RNF180	4	-0.11436	1	0	*CCDC22*	3	-0.1964	1	0
HS1BP3	4	-0.11433	1	0	*C17orf80*	4	-0.19625	1	0
ETV5	4	-0.11432	1	0	*MYL9*	3	-0.19605	1	0
C2orf73	3	-0.11431	1	0	*OTOF*	1	-0.19585	1	0
SOX12	3	-0.11417	1	0	*ATXN7L3B*	3	-0.19585	1	0
CST7	4	-0.11412	1	0	*H2AC7*	4	-0.1958	1	0
PCDH20	3	-0.11401	1	0	*KYAT3*	3	-0.19577	1	0
C9	3	-0.11397	1	0	*GPNMB*	3	-0.19544	1	0
ALB	4	-0.11394	1	0	*BLMH*	4	-0.19543	1	0
REG1A	4	-0.11392	1	0	*ENHO*	3	-0.19543	1	0
VPS18	4	-0.11389	1	0	*IFT88*	4	-0.19534	1	0
TLX2	3	-0.11387	1	0	*HAUS2*	4	-0.19527	1	0
POM121	3	-0.11356	1	0	*CUTA*	3	-0.19516	1	0
GBX2	4	-0.11347	1	0	*PNPLA8*	2	-0.19506	1	0
TAGLN	4	-0.11337	1	0	*SLC25A14*	4	-0.1949	1	0
SLC9A5	4	-0.11336	1	0	*PLIN5*	3	-0.19489	1	0
DPH3P1	4	-0.11319	1	0	*SLC7A1*	4	-0.19487	1	0
MACF1	2	-0.11318	1	0	*ASPM*	3	-0.19466	1	0
ZNF80	4	-0.11311	1	0	*KRT20*	4	-0.19465	1	0
HSPB9	4	-0.11307	1	0	*TTC39B*	2	-0.19455	1	0
LTO1	4	-0.113	1	0	*CYTH3*	2	-0.19451	1	0
LANCL1	4	-0.11296	1	0	*C1orf195*	4	-0.19443	1	0
KLHL38	4	-0.11295	1	0	*PRMT6*	2	-0.19432	1	0
SPATA9	3	-0.11293	1	0	*FPR2*	4	-0.19427	1	0
HSD17B11	3	-0.11284	1	0	*HSF2*	4	-0.19426	1	0
TRIP11	4	-0.11278	1	0	*NUTM2G*	2	-0.19424	1	0
RRAGB	3	-0.11277	1	0	*PPP1R1A*	3	-0.19419	1	0
SPAST	4	-0.11268	1	0	*MED12*	3	-0.19407	1	0
CARD10	4	-0.11253	1	0	*ZNF562*	3	-0.19394	1	0
ARPC1A	2	-0.11244	1	0	*LRRC37B*	2	-0.19392	1	0
OFD1	4	-0.11242	1	0	*PIN1*	4	-0.19359	1	0
AIF1	4	-0.11241	1	0	*SLC2A8*	2	-0.19351	1	0
KRT20	4	-0.11239	1	0	*ATP6V0A4*	4	-0.19351	1	0
PPP2R2A	3	-0.11236	1	0	*DHX16*	3	-0.19325	1	0
SNX7	4	-0.11229	1	0	*CARMIL1*	3	-0.19319	1	0
PGRMC1	4	-0.11226	1	0	*MYO5A*	4	-0.19308	1	0
EDN1	4	-0.11225	1	0	*TESMIN*	1	-0.19302	1	0
MAP2K1	3	-0.11224	1	0	*ASIC1*	4	-0.19275	1	0

GPRIN1	1	-0.11204	1	0	*NUDT12*	4	-0.19249	1	0
LEKR1	3	-0.11202	1	0	*SELENOK*	2	-0.19237	1	0
DUS3L	3	-0.11198	1	0	*HLA-DMB*	3	-0.19232	1	0
SRPK1	4	-0.11198	1	0	*CCNB3*	4	-0.19209	1	0
EIF2S2	2	-0.11176	1	0	*PCDHB15*	3	-0.19204	1	0
H2BC8	4	-0.11176	1	0	*MIA2*	7	-0.19171	1	0
ZNF354A	3	-0.11175	1	0	*RAVER1*	4	-0.19159	1	0
SH3BP4	4	-0.11166	1	0	*RNASE4*	4	-0.19141	1	0
C6orf132	3	-0.1116	1	0	*WDR37*	4	-0.1914	1	0
DELE1	4	-0.11157	1	0	*CYP26B1*	4	-0.19133	1	0
RBM10	4	-0.11156	1	0	*NEURL3*	2	-0.1911	1	0
ALDH6A1	4	-0.11153	1	0	*TMEM100*	4	-0.19107	1	0
BTAF1	4	-0.11149	1	0	*EML1*	4	-0.19106	1	0
CPEB4	4	-0.11144	1	0	*PTPA*	4	-0.19078	1	0
YPEL1	3	-0.1114	1	0	*EXPH5*	4	-0.19076	1	0
NUDT12	4	-0.11138	1	0	*MREG*	4	-0.19075	1	0
CARD16	4	-0.11126	1	0	*GON4L*	3	-0.19075	1	0
U2SURP	4	-0.1112	1	0	*LRRC73*	2	-0.19069	1	0
BPNT1	4	-0.11111	1	0	*TFAP4*	4	-0.19068	1	0
SPTBN4	2	-0.11104	1	0	*CHAC2*	3	-0.19065	1	0
QSER1	4	-0.11097	1	0	*RBM27*	3	-0.19047	1	0
PIK3CD	4	-0.11096	1	0	*PSG4*	3	-0.19045	1	0
FPGT	2	-0.11085	1	0	*MFAP3L*	3	-0.1902	1	0
MED1	4	-0.11069	1	0	*SLC1A1*	4	-0.1901	1	0
SH2D4B	4	-0.11069	1	0	*SLC6A14*	4	-0.19005	1	0
ZNF281	2	-0.11046	1	0	*FAM71E1*	3	-0.19001	1	0
FKBP4	4	-0.11044	1	0	*SPAG6*	4	-0.18995	1	0
REP15	4	-0.11037	1	0	*MAGEF1*	3	-0.18991	1	0
AQP7	3	-0.11036	1	0	*CFDP1*	3	-0.18985	1	0
C2	4	-0.11036	1	0	*PLPP3*	4	-0.18968	1	0
MTOR	3	-0.11034	1	0	*SYN2*	3	-0.18959	1	0
CPNE7	4	-0.11033	1	0	*GGH*	3	-0.18954	1	0
ZBTB26	2	-0.11024	1	0	*LCA5L*	2	-0.18953	1	0
FKBP11	4	-0.11023	1	0	*SLC66A2*	2	-0.18952	1	0
PDZD2	4	-0.11014	1	0	*DOCK8*	3	-0.18942	1	0
CLPTM1L	4	-0.11008	1	0	*CHRNB3*	3	-0.18922	1	0
TFF1	4	-0.11006	1	0	*CARD10*	4	-0.18918	1	0
VAMP1	3	-0.11006	1	0	*PDCD6*	2	-0.18905	1	0
YY2	4	-0.11001	1	0	*ACAT1*	2	-0.18888	1	0
CLCN3	4	-0.10971	1	0	*OR1L8*	4	-0.18879	1	0
KYNU	2	-0.10969	1	0	*KDM4D*	4	-0.18871	1	0
MAP2K6	4	-0.10966	1	0	*AR*	4	-0.18867	1	0
APELA	4	-0.10964	1	0	*MAN1A2*	3	-0.18865	1	0
TRIM21	2	-0.10962	1	0	*CABYR*	4	-0.18864	1	0
SOD3	3	-0.10961	1	0	*PHYKPL*	4	-0.18849	1	0
CLBA1	3	-0.10958	1	0	*RMC1*	4	-0.18802	1	0
CNTN1	3	-0.10957	1	0	*PSMB9*	4	-0.18793	1	0
IFI44	4	-0.10954	1	0	*SRCIN1*	3	-0.18793	1	0
ZNF534	3	-0.10954	1	0	*ADCY10*	4	-0.18788	1	0
MMP10	4	-0.1094	1	0	*CA12*	4	-0.18777	1	0
FAM114A1	4	-0.10938	1	0	*AGER*	4	-0.18777	1	0
CLDN10	3	-0.10923	1	0	*SMPD2*	3	-0.18777	1	0
GNB4	4	-0.10919	1	0	*ANKRD34B*	4	-0.18776	1	0
HSD3B2	4	-0.10919	1	0	*NLGN3*	4	-0.18768	1	0
GRB10	4	-0.10917	1	0	*HSPB3*	3	-0.1876	1	0
MKRN2OS	4	-0.10915	1	0	*ZNF226*	2	-0.18731	1	0
MARCHF1	3	-0.10913	1	0	*PMM1*	4	-0.18722	1	0
IL31RA	4	-0.10895	1	0	*LARP7*	1	-0.18694	1	0

TEKT4	4	-0.10891	1	0	*LHPP*	4	-0.18687	1	0
ZNF720	3	-0.10889	1	0	*RHOBTB2*	4	-0.18685	1	0
WDPCP	3	-0.10883	1	0	*SPTBN2*	3	-0.18675	1	0
ZBTB7A	4	-0.10871	1	0	*IL12B*	2	-0.18661	1	0
FCGR1B	1	-0.10867	1	0	*APEX1*	3	-0.18661	1	0
ZDHHC6	4	-0.10858	1	0	*FSCB*	4	-0.18659	1	0
HCCS	4	-0.10852	1	0	*ZNF827*	4	-0.1865	1	0
DHX35	4	-0.10851	1	0	*GCFC2*	3	-0.18648	1	0
CDR2L	4	-0.10828	1	0	*LCLAT1*	3	-0.18647	1	0
C2orf88	3	-0.10818	1	0	*JPH2*	4	-0.18638	1	0
PRDM4	4	-0.10816	1	0	*TIGD4*	2	-0.18631	1	0
TMEM116	3	-0.10811	1	0	*AKAP9*	3	-0.18631	1	0
GJB2	4	-0.1081	1	0	*TOM1L1*	4	-0.18616	1	0
STXBP3	3	-0.10798	1	0	*NDRG4*	4	-0.18616	1	0
OR10H1	3	-0.10793	1	0	*SLC17A8*	2	-0.18598	1	0
C9orf170	4	-0.10781	1	0	*GBP5*	3	-0.18597	1	0
TCEANC	4	-0.10771	1	0	*CHML*	4	-0.18596	1	0
CHM	4	-0.10767	1	0	*UPF3A*	3	-0.18591	1	0
HS3ST5	3	-0.10756	1	0	*HMCN1*	4	-0.18586	1	0
MTHFD2L	2	-0.10755	1	0	*GLDC*	3	-0.18575	1	0
RAB3IL1	4	-0.10743	1	0	*TMEM249*	3	-0.18566	1	0
CAMK1D	1	-0.10729	1	0	*CALR3*	4	-0.18566	1	0
ENAM	4	-0.10723	1	0	*MYL4*	4	-0.18527	1	0
MLXIPL	4	-0.10722	1	0	*FRMD3*	3	-0.18518	1	0
CLIP4	4	-0.10678	1	0	*H2AC1*	4	-0.18513	1	0
CAMKV	4	-0.10675	1	0	*MED31*	4	-0.18509	1	0
TRH	4	-0.10671	1	0	*PCDHB14*	3	-0.18484	1	0
KLK10	4	-0.1067	1	0	*ECEL1*	4	-0.18445	1	0
CHMP3	1	-0.10661	1	0	*MCL1*	4	-0.18442	1	0
DTNA	3	-0.10659	1	0	*SRRM2*	4	-0.1843	1	0
STARD3	4	-0.10656	1	0	*PKIA*	4	-0.18397	1	0
CCL5	4	-0.10652	1	0	*DPH3P1*	4	-0.18383	1	0
ALOX5AP	4	-0.1064	1	0	*CHMP2A*	2	-0.18381	1	0
CCDC36	4	-0.10632	1	0	*HSD11B2*	4	-0.18378	1	0
CGRRF1	4	-0.10631	1	0	*AMY2B*	3	-0.18375	1	0
SRSF11	4	-0.10628	1	0	*SLC29A2*	3	-0.18358	1	0
IPO7	4	-0.10623	1	0	*RPS6KA5*	4	-0.18356	1	0
ERICH6B	4	-0.10622	1	0	*EFL1*	3	-0.18338	1	0
RORC	4	-0.10622	1	0	*REPS2*	3	-0.18334	1	0
ZNF613	3	-0.10617	1	0	*TTC26*	3	-0.18323	1	0
C1orf105	3	-0.10615	1	0	*GGNBP2*	4	-0.18317	1	0
HSBP1L1	3	-0.10612	1	0	*AQP7*	3	-0.1827	1	0
RAPGEF6	3	-0.10605	1	0	*C10orf25*	4	-0.18259	1	0
NUDCD3	4	-0.10597	1	0	*WDR11*	4	-0.18249	1	0
NICN1	2	-0.10588	1	0	*ZFP62*	4	-0.18238	1	0
DHX57	4	-0.10584	1	0	*FCHO2*	3	-0.18238	1	0
TBXAS1	4	-0.10581	1	0	*PHF23*	4	-0.18217	1	0
GARNL3	3	-0.10565	1	0	*MICAL1*	3	-0.18213	1	0
ZNF496	4	-0.10563	1	0	*SYDE2*	4	-0.18202	1	0
ZFYVE1	3	-0.10557	1	0	*ELF2*	4	-0.182	1	0
MYH10	4	-0.10556	1	0	*C4orf36*	4	-0.18188	1	0
TCTA	4	-0.10556	1	0	*ENPP4*	4	-0.18173	1	0
PDCL3	3	-0.10524	1	0	*ST3GAL2*	3	-0.1817	1	0
LDHD	4	-0.1052	1	0	*CLSTN3*	3	-0.18153	1	0
IFT172	3	-0.10511	1	0	*ZP3*	3	-0.18149	1	0
RNF11	4	-0.10508	1	0	*C12orf10*	4	-0.18147	1	0
RMI2	4	-0.10501	1	0	*DCBLD2*	4	-0.18147	1	0
EPB41L3	3	-0.10498	1	0	*C7orf65*	4	-0.18142	1	0

MSX1	4	-0.10497	1	0	*SMIM10*	4	-0.18136	1	0
ZNF675	4	-0.10497	1	0	*LRRN4CL*	4	-0.18136	1	0
C1QTNF6	4	-0.10484	1	0	*CFP*	4	-0.18136	1	0
PPP1R9B	4	-0.10467	1	0	*TNFRSF11A*	4	-0.18127	1	0
PPARD	4	-0.10465	1	0	*RABL2A*	1	-0.18111	1	0
ACVR2B	3	-0.10462	1	0	*ARID3C*	2	-0.18109	1	0
CFB	4	-0.1046	1	0	*HMGXB3*	4	-0.18102	1	0
WASF3	3	-0.10458	1	0	*COA1*	4	-0.181	1	0
ARMC10	2	-0.10454	1	0	*GAPVD1*	4	-0.18087	1	0
PCDHA5	3	-0.1045	1	0	*RALB*	4	-0.1807	1	0
SUOX	3	-0.10446	1	0	*AGBL4*	4	-0.18057	1	0
PDGFA	4	-0.10441	1	0	*TRIM31*	2	-0.18024	1	0
PTPRQ	2	-0.10441	1	0	*MYB*	4	-0.1801	1	0
FKTN	4	-0.10439	1	0	*ANK1*	4	-0.18003	1	0
TNKS	3	-0.10436	1	0	*HSP90AA1*	2	-0.17985	1	0
CPS1	4	-0.10417	1	0	*DNAJB12*	3	-0.1797	1	0
ZNF141	4	-0.10405	1	0	*DNAI1*	3	-0.17969	1	0
KPNB1	4	-0.10398	1	0	*DAXX*	2	-0.17967	1	0
TRIM45	4	-0.10398	1	0	*FZD9*	3	-0.1796	1	0
APOL6	4	-0.10397	1	0	*FBXO9*	4	-0.17946	1	0
NRSN1	4	-0.10394	1	0	*G3BP2*	2	-0.17891	1	0
CTU2	3	-0.10386	1	0	*LANCL2*	3	-0.17882	1	0
HIPK1	3	-0.10376	1	0	*TMEM98*	4	-0.1788	1	0
IL1A	4	-0.10374	1	0	*PP2D1*	3	-0.17867	1	0
BSDC1	4	-0.10367	1	0	*SLC1A3*	4	-0.17856	1	0
DNAJB12	3	-0.10363	1	0	*SPINK13*	4	-0.17853	1	0
ANO7	3	-0.10348	1	0	*LGI3*	4	-0.17852	1	0
KRT78	2	-0.10345	1	0	*AIF1L*	2	-0.17841	1	0
GPC2	2	-0.10334	1	0	*CCL25*	4	-0.1782	1	0
ZNF549	4	-0.10334	1	0	*MSX1*	4	-0.17818	1	0
RAF1	4	-0.10333	1	0	*LCP2*	4	-0.17813	1	0
AZU1	3	-0.10326	1	0	*ITGA9*	4	-0.17813	1	0
LYPD3	4	-0.10324	1	0	*SH2B2*	3	-0.17796	1	0
CTSD	4	-0.10323	1	0	*PRKAR2A*	2	-0.17781	1	0
BOD1	4	-0.10321	1	0	*PCNX1*	4	-0.17758	1	0
UBR2	4	-0.10296	1	0	*POGK*	4	-0.17756	1	0
CMC2	4	-0.10286	1	0	*USP2*	4	-0.17753	1	0
LSP1	4	-0.10285	1	0	*TSNAXIP1*	4	-0.17729	1	0
SMAP1	4	-0.10285	1	0	*EMSY*	2	-0.17722	1	0
GLIPR1L2	2	-0.10283	1	0	*CNGB1*	4	-0.17705	1	0
PLD2	4	-0.10276	1	0	*LIPH*	4	-0.17698	1	0
LPIN1	4	-0.10274	1	0	*ESF1*	3	-0.17667	1	0
RIIAD1	4	-0.10265	1	0	*MCTS1*	4	-0.17665	1	0
PGAP2	4	-0.1026	1	0	*SLC2A14*	4	-0.17638	1	0
ZNF385B	4	-0.1025	1	0	*TCN2*	1	-0.17629	1	0
FBXO33	4	-0.10246	1	0	*RAB21*	2	-0.17628	1	0
CAMK2N2	4	-0.10243	1	0	*AGO2*	4	-0.17617	1	0
TRAF5	3	-0.10235	1	0	*SHD*	3	-0.17616	1	0
C10orf82	4	-0.1023	1	0	*CMYA5*	3	-0.17615	1	0
DCAF6	4	-0.10222	1	0	*CRYBB2*	2	-0.176	1	0
RAPGEF1	4	-0.10216	1	0	*C18orf54*	3	-0.17575	1	0
TM4SF4	4	-0.10215	1	0	*PLEKHG5*	4	-0.17563	1	0
MLLT6	4	-0.10212	1	0	*PKHD1*	4	-0.17557	1	0
DUSP18	4	-0.10211	1	0	*ATAD3C*	3	-0.17553	1	0
ZNF134	4	-0.10202	1	0	*ZNF841*	3	-0.17545	1	0
RGS10	4	-0.102	1	0	*HOXA10*	4	-0.17542	1	0
GNLY	4	-0.10198	1	0	*IFT27*	4	-0.17541	1	0
FAM136A	4	-0.10193	1	0	*SLC39A10*	1	-0.17538	1	0

EPB41L4B	4	-0.10192	1	0	*LXN*	4	-0.17538	1	0
GSPT1	1	-0.10177	1	0	*GLIS1*	4	-0.17528	1	0
ZNF438	4	-0.10176	1	0	*KAT2B*	4	-0.17516	1	0
ARMT1	4	-0.10166	1	0	*TM7SF3*	3	-0.17497	1	0
GDA	4	-0.10161	1	0	*RAB3A*	3	-0.17473	1	0
ZNF772	4	-0.10161	1	0	*GDE1*	2	-0.17465	1	0
DNAI1	3	-0.1016	1	0	*LARP4B*	4	-0.17465	1	0
DSG2	4	-0.1016	1	0	*ZFP42*	4	-0.17461	1	0
HSPA2	4	-0.10158	1	0	*CLUAP1*	3	-0.17453	1	0
FAM49A	4	-0.10151	1	0	*DMBX1*	3	-0.17451	1	0
SNX6	4	-0.10145	1	0	*APOBEC3F*	3	-0.17443	1	0
ASPSCR1	3	-0.1013	1	0	*P3H1*	2	-0.17427	1	0
NLGN4Y	4	-0.10129	1	0	*IRF9*	3	-0.17405	1	0
PEX12	3	-0.10122	1	0	*ZDHHC18*	3	-0.174	1	0
JHY	4	-0.10116	1	0	*ZBED4*	4	-0.17385	1	0
ZNF557	3	-0.10115	1	0	*ID3*	4	-0.17383	1	0
SKIDA1	2	-0.10106	1	0	*FAM221B*	4	-0.17355	1	0
FAM149A	4	-0.101	1	0	*C12orf76*	4	-0.17353	1	0
LRRC26	4	-0.10093	1	0	*VCPKMT*	2	-0.17353	1	0
TRUB1	4	-0.1009	1	0	*TMEM185B*	3	-0.17345	1	0
PIDD1	3	-0.10083	1	0	*POU3F2*	4	-0.17338	1	0
ADTRP	4	-0.10082	1	0	*AP5B1*	3	-0.17336	1	0
ATP6V0A1	3	-0.1007	1	0	*ZNF879*	3	-0.17305	1	0
NADK2	3	-0.1006	1	0	*LIF*	3	-0.17296	1	0
CYB5R4	4	-0.10051	1	0	*PLOD1*	4	-0.17271	1	0
THAP12	1	-0.10043	1	0	*AFF3*	3	-0.1726	1	0
TEAD3	3	-0.10041	1	0	*PCDHA7*	4	-0.17256	1	0
PRDM6	3	-0.10032	1	0	*PCDH9*	3	-0.17254	1	0
EMILIN1	4	-0.10013	1	0	*FOXD2*	3	-0.17252	1	0
LACTB2	4	-0.10013	1	0	*CLCN3*	4	-0.17245	1	0
FCRL5	3	-0.10007	1	0	*ENO1*	3	-0.1724	1	0
MYO15A	4	-0.10004	1	0	*LYPLA1*	3	-0.17237	1	0
SLC35C2	3	-0.09985	1	0	*VCPIP1*	4	-0.17229	1	0
HMGN5	3	-0.099807	1	0	*NCBP1*	4	-0.17226	1	0
NAAA	4	-0.099787	1	0	*ZNF660*	4	-0.17223	1	0
ERICH5	3	-0.099647	1	0	*ATP1A2*	4	-0.17219	1	0
ATAD2	3	-0.099604	1	0	*ANKRD39*	3	-0.17212	1	0
CNTN5	4	-0.0996	1	0	*SLC25A35*	4	-0.17208	1	0
SHC3	4	-0.099579	1	0	*DES*	2	-0.17203	1	0
CACNG7	4	-0.099377	1	0	*IQCC*	3	-0.17203	1	0
AGR2	4	-0.099326	1	0	*OTUD5*	4	-0.17202	1	0
USH1C	4	-0.099299	1	0	*CYP24A1*	4	-0.17189	1	0
GPR162	4	-0.099111	1	0	*HSF5*	3	-0.17175	1	0
SNX17	4	-0.099085	1	0	*RDH11*	4	-0.17165	1	0
RAD17	3	-0.099077	1	0	*GPR68*	4	-0.17161	1	0
C22orf46	3	-0.098893	1	0	*ZNF92*	3	-0.17155	1	0
INVS	4	-0.098763	1	0	*KCNE1*	1	-0.1715	1	0
ZNF813	3	-0.09873	1	0	*MKRN2OS*	4	-0.17143	1	0
CTSV	3	-0.098668	1	0	*RBM15*	4	-0.17136	1	0
SMURF1	4	-0.098638	1	0	*ALOXE3*	4	-0.17122	1	0
PRNP	4	-0.098536	1	0	*PIWIL4*	3	-0.17121	1	0
CRH	4	-0.098531	1	0	*SEPTIN1*	3	-0.1712	1	0
FOLR2	4	-0.098488	1	0	*CASTOR3*	2	-0.17106	1	0
TMEM176A	4	-0.098459	1	0	*LSMEM1*	3	-0.17105	1	0
ASXL2	3	-0.098452	1	0	*BDP1*	4	-0.17104	1	0
MIPOL1	4	-0.098378	1	0	*FAM136A*	4	-0.17102	1	0
KDM5D	3	-0.098042	1	0	*KCNT1*	4	-0.171	1	0
KLHL26	2	-0.097974	1	0	*WDR13*	4	-0.17087	1	0

CPE	3	-0.097927	1	0	*GATA4*	4	-0.17085	1	0
HOXA1	3	-0.097867	1	0	*HFE*	2	-0.17084	1	0
NUAK1	4	-0.097856	1	0	*NPR2*	3	-0.1708	1	0
ADRA2A	3	-0.097844	1	0	*VKORC1*	4	-0.17063	1	0
FBN1	3	-0.097763	1	0	*TRIM35*	2	-0.17053	1	0
TAF9B	4	-0.097725	1	0	*FCSK*	4	-0.17051	1	0
TENM2	4	-0.097709	1	0	*GNAI3*	4	-0.17047	1	0
LPIN3	4	-0.097693	1	0	*PYGL*	3	-0.1704	1	0
CALML4	4	-0.097681	1	0	*COL4A5*	4	-0.17009	1	0
C3orf62	1	-0.097552	1	0	*AMBRA1*	4	-0.16995	1	0
TLCD1	4	-0.097514	1	0	*NECAP2*	2	-0.16994	1	0
ZNF655	4	-0.097381	1	0	*EML6*	3	-0.16987	1	0
TTC30A	3	-0.097362	1	0	*STPG4*	3	-0.16982	1	0
ANGPTL4	2	-0.097354	1	0	*PCSK5*	3	-0.16971	1	0
FUCA2	4	-0.097306	1	0	*TMEM245*	4	-0.16966	1	0
SYN1	3	-0.097237	1	0	*NOX1*	3	-0.16964	1	0
DDX4	3	-0.09722	1	0	*ZNF106*	3	-0.16961	1	0
SNHG32	4	-0.097207	1	0	*CYP27C1*	4	-0.16961	1	0
CSTF2T	4	-0.097125	1	0	*KRT4*	3	-0.16957	1	0
FBXL22	4	-0.097119	1	0	*RNF220*	3	-0.16946	1	0
INO80E	4	-0.09703	1	0	*TRAK1*	2	-0.16944	1	0
MRPL40	3	-0.097006	1	0	*FAM120B*	3	-0.16936	1	0
STK38L	4	-0.096987	1	0	*TRIM66*	4	-0.16931	1	0
GPR85	3	-0.096978	1	0	*MELK*	4	-0.16928	1	0
TUT4	3	-0.096961	1	0	*B3GNT3*	1	-0.16924	1	0
XAGE2	3	-0.096956	1	0	*TECTA*	3	-0.16917	1	0
MBLAC2	4	-0.096953	1	0	*RPS6KA3*	4	-0.16912	1	0
LRATD1	8	-0.096937	1	0	*DPRX*	4	-0.16899	1	0
EME2	3	-0.096875	1	0	*ZNF436*	4	-0.16895	1	0
RAB40AL	1	-0.096823	1	0	*MTMR12*	3	-0.16888	1	0
RTKN	4	-0.096795	1	0	*ABCC2*	3	-0.16872	1	0
EPHA4	4	-0.096588	1	0	*PLEKHA6*	4	-0.1686	1	0
C18orf54	3	-0.096533	1	0	*MEGF11*	3	-0.16858	1	0
WFDC12	4	-0.096482	1	0	*DAW1*	3	-0.16852	1	0
PSMA8	4	-0.096421	1	0	*C1orf53*	4	-0.16844	1	0
FDXR	3	-0.096363	1	0	*DGAT2*	4	-0.16837	1	0
APOB	4	-0.096362	1	0	*SLC66A3*	3	-0.16822	1	0
JPT2	4	-0.096346	1	0	*ZNF286B*	3	-0.16819	1	0
RAB22A	4	-0.096284	1	0	*BBS9*	4	-0.168	1	0
MTURN	4	-0.096168	1	0	*ABHD18*	3	-0.16794	1	0
HIP1	4	-0.096151	1	0	*TMEM101*	3	-0.16793	1	0
GBA2	4	-0.096092	1	0	*ADAL*	4	-0.16781	1	0
MCFD2	4	-0.096074	1	0	*PPP6R3*	2	-0.16762	1	0
TGM5	2	-0.095998	1	0	*WNT5B*	3	-0.1676	1	0
VSIG10	4	-0.095977	1	0	*ATAT1*	3	-0.16741	1	0
VSIG1	4	-0.095954	1	0	*NIPAL1*	2	-0.16737	1	0
C2orf83	4	-0.095952	1	0	*KLHDC8B*	4	-0.16736	1	0
AKAP7	4	-0.095837	1	0	*PTMS*	1	-0.1673	1	0
ATRAID	3	-0.095804	1	0	*TRIM16*	3	-0.1672	1	0
ZCCHC14	3	-0.095786	1	0	*MBD1*	4	-0.16706	1	0
FOXN4	4	-0.095785	1	0	*SPEM1*	4	-0.1669	1	0
XKR4	3	-0.095716	1	0	*TAAR8*	4	-0.16662	1	0
AKR1C3	4	-0.095655	1	0	*SDCCAG8*	4	-0.16655	1	0
FBXO6	3	-0.095647	1	0	*ZNF431*	3	-0.16647	1	0
PRPF40B	3	-0.095568	1	0	*NKX2-3*	3	-0.16644	1	0
TENT4A	4	-0.095482	1	0	*CDKN2D*	4	-0.16627	1	0
ZNF136	4	-0.095477	1	0	*HPDL*	3	-0.16618	1	0
PHRF1	4	-0.095304	1	0	*KLK13*	4	-0.16615	1	0

SSR2	4	-0.095298	1	0	*HMX3*	3	-0.16612	1	0
NUP88	4	-0.095292	1	0	*SP7*	3	-0.16611	1	0
MAPK8IP3	3	-0.095237	1	0	*ELOA*	4	-0.166	1	0
SMARCA1	4	-0.095234	1	0	*H3C8*	4	-0.16589	1	0
NCEH1	4	-0.09523	1	0	*RETREG2*	4	-0.16589	1	0
RFX6	3	-0.095087	1	0	*PTCHD4*	3	-0.16587	1	0
MRPS5	4	-0.09503	1	0	*CNGA4*	2	-0.16566	1	0
TRNP1	4	-0.09502	1	0	*CCDC65*	4	-0.16566	1	0
DCT	4	-0.094887	1	0	*CHMP4A*	3	-0.16548	1	0
PGF	1	-0.094871	1	0	*MZB1*	3	-0.16545	1	0
RHO	4	-0.094854	1	0	*ZNF665*	4	-0.16539	1	0
SLC12A8	3	-0.09474	1	0	*RNF17*	3	-0.16525	1	0
ENDOG	3	-0.094689	1	0	*SNX16*	4	-0.16524	1	0
BHMT	4	-0.094645	1	0	*DNAJC4*	2	-0.16518	1	0
SULT1A1	4	-0.094615	1	0	*TAOK2*	4	-0.16515	1	0
EDRF1	4	-0.094497	1	0	*ZFYVE28*	4	-0.16508	1	0
CES2	4	-0.094486	1	0	*LOC728485*	2	-0.16502	1	0
COL23A1	4	-0.094458	1	0	*MAPRE2*	2	-0.16501	1	0
NLRC4	4	-0.094448	1	0	*ZNF786*	4	-0.16484	1	0
TMEM165	4	-0.094413	1	0	*GM2A*	4	-0.16475	1	0
IKZF1	3	-0.094386	1	0	*DMRT3*	3	-0.16461	1	0
F7	4	-0.094343	1	0	*COMMD4*	4	-0.16449	1	0
RBAK	4	-0.094313	1	0	*NTF3*	3	-0.1644	1	0
IQSEC1	2	-0.094311	1	0	*LSS*	4	-0.16434	1	0
VGLL4	3	-0.094305	1	0	*HTRA1*	4	-0.16425	1	0
MTFR1L	4	-0.094299	1	0	*CAMK4*	4	-0.1641	1	0
ZNF250	4	-0.094186	1	0	*HES5*	2	-0.16404	1	0
FBXO38	4	-0.09411	1	0	*ME3*	4	-0.164	1	0
DPRX	4	-0.094059	1	0	*SCARB1*	4	-0.16393	1	0
SOX30	4	-0.094018	1	0	*DNAJC12*	3	-0.16382	1	0
ZDHHC14	4	-0.093987	1	0	*VASH1*	4	-0.16378	1	0
SPIN3	4	-0.093962	1	0	*SLA*	3	-0.16376	1	0
SGCB	4	-0.093953	1	0	*ARF1*	2	-0.16355	1	0
NPBWR1	3	-0.093883	1	0	*GDF7*	3	-0.16353	1	0
NCOA3	4	-0.093857	1	0	*PATZ1*	3	-0.16346	1	0
DMRT3	3	-0.09383	1	0	*PDZD2*	4	-0.16342	1	0
PRPF31	4	-0.09382	1	0	*CYB5D1*	4	-0.16321	1	0
UQCC1	3	-0.093786	1	0	*MERTK*	3	-0.16315	1	0
AHDC1	3	-0.093784	1	0	*GALNT2*	3	-0.16313	1	0
KANSL1	3	-0.093725	1	0	*CAMK1D*	1	-0.16308	1	0
ATF6	4	-0.09368	1	0	*SLC20A1*	4	-0.16307	1	0
ATXN3	3	-0.093662	1	0	*XCL2*	2	-0.16292	1	0
PHF3	4	-0.09359	1	0	*KCNG1*	4	-0.16272	1	0
KRT7	3	-0.093463	1	0	*ZNF80*	4	-0.16264	1	0
RNF220	3	-0.093406	1	0	*CCND2*	4	-0.16254	1	0
HJV	4	-0.093387	1	0	*MARVELD1*	4	-0.16252	1	0
STARD9	4	-0.093263	1	0	*TCEANC*	4	-0.16244	1	0
ZC3H11A	3	-0.093203	1	0	*POU3F1*	3	-0.1623	1	0
CYB561D1	4	-0.093133	1	0	*WWC2*	4	-0.1622	1	0
H2AC17	4	-0.093102	1	0	*TMEM128*	4	-0.16192	1	0
DYNC1I2	4	-0.093003	1	0	*TPSD1*	4	-0.16182	1	0
CEBPD	2	-0.092962	1	0	*BMP8B*	3	-0.16171	1	0
DHX58	4	-0.092913	1	0	*HECA*	4	-0.16158	1	0
IL3RA	3	-0.092773	1	0	*NACAD*	2	-0.16154	1	0
AKAP8L	3	-0.092769	1	0	*SLC30A2*	4	-0.16147	1	0
MIOX	3	-0.092753	1	0	*SLC24A5*	4	-0.16109	1	0
DCUN1D3	4	-0.092745	1	0	*MLX*	4	-0.16108	1	0
GPR34	4	-0.092731	1	0	*LTA*	4	-0.16081	1	0

SESN2	4	-0.092709	1	0	*CFAP43*	3	-0.16057	1	0
QDPR	3	-0.092683	1	0	*CCDC189*	3	-0.16028	1	0
ZFYVE26	4	-0.092682	1	0	*MOV10*	3	-0.16023	1	0
SERINC4	4	-0.09268	1	0	*C16orf70*	4	-0.16009	1	0
CMSS1	3	-0.092631	1	0	*LRP10*	4	-0.16	1	0
PARP16	4	-0.09252	1	0	*HOXD4*	1	-0.15994	1	0
EEPD1	4	-0.092512	1	0	*STPG3*	1	-0.15979	1	0
CREBRF	4	-0.09248	1	0	*CDHR1*	3	-0.15966	1	0
OR10C1	4	-0.092456	1	0	*CHRAC1*	2	-0.15965	1	0
ITGB7	4	-0.092422	1	0	*SLC25A47*	2	-0.15952	1	0
ABCD4	4	-0.092379	1	0	*MYCN*	1	-0.15942	1	0
GPR18	4	-0.0923	1	0	*TMEM255A*	4	-0.15932	1	0
RIPPLY1	4	-0.092287	1	0	*SLC26A10*	2	-0.1593	1	0
REL	4	-0.092179	1	0	*NUAK1*	4	-0.15929	1	0
ARSI	2	-0.092174	1	0	*MAOB*	3	-0.1591	1	0
ZFAND6	4	-0.092088	1	0	*RSPH6A*	3	-0.1591	1	0
IL36B	4	-0.092061	1	0	*GON7*	4	-0.15904	1	0
SHC2	4	-0.091993	1	0	*WDR41*	3	-0.15863	1	0
HNRNPH2	4	-0.091832	1	0	*MARVELD3*	2	-0.15842	1	0
TRIM46	4	-0.091829	1	0	*TRA2B*	3	-0.15827	1	0
KIF27	3	-0.091762	1	0	*SLC49A4*	3	-0.15824	1	0
C7orf50	3	-0.091718	1	0	*ENG*	2	-0.15813	1	0
SLC17A7	4	-0.091652	1	0	*CNTN4*	3	-0.15811	1	0
LBR	3	-0.091592	1	0	*ZBTB8OS*	3	-0.15801	1	0
KLHDC3	3	-0.091405	1	0	*NTRK1*	4	-0.15795	1	0
ATP4A	4	-0.091232	1	0	*CYTH4*	4	-0.15788	1	0
MYLIP	3	-0.091118	1	0	*BIRC3*	3	-0.15752	1	0
LOC730183	3	-0.091106	1	0	*JUN*	3	-0.15726	1	0
MCEMP1	4	-0.091103	1	0	*BEX3*	4	-0.15722	1	0
ECHDC3	4	-0.091016	1	0	*FAM78A*	4	-0.15711	1	0
XPO6	3	-0.090966	1	0	*PFKFB3*	3	-0.15707	1	0
MRPL33	3	-0.090592	1	0	*CARD8*	3	-0.15705	1	0
COL24A1	3	-0.090559	1	0	*CDC42EP5*	4	-0.15692	1	0
MMP11	4	-0.090392	1	0	*ATP8B1*	4	-0.1569	1	0
ITPKA	2	-0.090383	1	0	*S100A6*	4	-0.1568	1	0
ALOXE3	4	-0.090345	1	0	*BST1*	4	-0.15673	1	0
DDO	4	-0.090315	1	0	*ZNF177*	4	-0.15651	1	0
ZC3HC1	4	-0.090198	1	0	*CYP1A1*	4	-0.15651	1	0
STAT4	3	-0.090044	1	0	*FAHD2B*	4	-0.1565	1	0
FAM102A	4	-0.090035	1	0	*SFT2D1*	4	-0.15646	1	0
SCRN3	4	-0.090034	1	0	*PDE7A*	3	-0.15645	1	0
C3orf14	4	-0.090014	1	0	*TP53I11*	2	-0.15645	1	0
TSPAN1	4	-0.089712	1	0	*MBD4*	4	-0.1564	1	0
MRPL47	4	-0.089494	1	0	*ZNF530*	4	-0.15602	1	0
PTBP2	4	-0.0894	1	0	*MATN3*	4	-0.15601	1	0
STAU1	4	-0.089372	1	0	*AJUBA*	4	-0.15598	1	0
HUS1	4	-0.089342	1	0	*BCL11B*	3	-0.15593	1	0
SZT2	3	-0.089285	1	0	*ZNF789*	4	-0.15591	1	0
ANXA3	4	-0.089258	1	0	*ATG4C*	4	-0.15588	1	0
CAMK1G	4	-0.089258	1	0	*CD302*	1	-0.15576	1	0
ZNF580	2	-0.089226	1	0	*NAV3*	4	-0.15574	1	0
PRDM11	3	-0.089208	1	0	*COL14A1*	4	-0.15555	1	0
ZNF714	3	-0.089186	1	0	*KIF13A*	3	-0.15546	1	0
NRTN	3	-0.089085	1	0	*LCN2*	4	-0.15536	1	0
CYP21A2	4	-0.089077	1	0	*ZBTB46*	4	-0.1553	1	0
MRPL45	2	-0.089026	1	0	*SOX8*	4	-0.15527	1	0
SCRN1	3	-0.089006	1	0	*BNIPL*	4	-0.15522	1	0
TK1	2	-0.088923	1	0	*C16orf97*	4	-0.1552	1	0

NELL2	2	-0.088905	1	0	*ZNF410*	4	-0.15506	1	0
C8orf58	4	-0.088813	1	0	*TGFBR1*	4	-0.15489	1	0
PMP22	2	-0.088797	1	0	*ASTN2*	4	-0.15479	1	0
DFFB	4	-0.088729	1	0	*PCNT*	3	-0.1546	1	0
INPP5K	4	-0.08871	1	0	*AMMECR1*	3	-0.15455	1	0
WNT5A	3	-0.088706	1	0	*MYOZ3*	4	-0.15451	1	0
SLC9A1	4	-0.08864	1	0	*LENG9*	3	-0.15449	1	0
GSR	4	-0.088479	1	0	*LRRC41*	1	-0.15444	1	0
RPH3A	4	-0.088449	1	0	*UVSSA*	3	-0.15443	1	0
TRIM7	2	-0.08842	1	0	*VGLL1*	3	-0.15438	1	0
ACTN2	3	-0.088366	1	0	*BIN3*	3	-0.15428	1	0
SLC33A1	4	-0.088343	1	0	*USP11*	4	-0.15415	1	0
RBM15B	4	-0.088342	1	0	*GBP6*	2	-0.15408	1	0
IQCF1	4	-0.088328	1	0	*CBLC*	4	-0.15404	1	0
TYSND1	4	-0.088304	1	0	*MMP2*	2	-0.15398	1	0
DPYSL4	3	-0.088236	1	0	*ANKRD65*	1	-0.15386	1	0
PCBP3	3	-0.088182	1	0	*SDHAF1*	4	-0.15383	1	0
AQP6	2	-0.088161	1	0	*HERPUD1*	4	-0.15383	1	0
GUCY2D	4	-0.088155	1	0	*CDR2L*	4	-0.15383	1	0
CYP2C18	4	-0.088104	1	0	*DHRS9*	4	-0.15374	1	0
NHLRC3	3	-0.088046	1	0	*GRK3*	4	-0.15363	1	0
PRRT1	2	-0.088016	1	0	*KISS1*	3	-0.15361	1	0
PARD3	4	-0.087963	1	0	*LMO4*	4	-0.1536	1	0
AFAP1	2	-0.087947	1	0	*IGLL1*	4	-0.15358	1	0
TMEM45A	3	-0.08792	1	0	*GALNTL5*	4	-0.15354	1	0
TBC1D8	4	-0.087838	1	0	*ZDHHC13*	4	-0.15342	1	0
SOWAHD	3	-0.08783	1	0	*C19orf12*	3	-0.15342	1	0
DECR1	4	-0.087746	1	0	*RASSF5*	3	-0.15338	1	0
CLDN20	4	-0.087656	1	0	*FAM234B*	4	-0.15314	1	0
HDHD5	4	-0.087623	1	0	*ZNF471*	2	-0.15301	1	0
INSL4	4	-0.08758	1	0	*NIBAN1*	3	-0.15296	1	0
MAGEL2	4	-0.087442	1	0	*RP2*	4	-0.15295	1	0
FAM151B	4	-0.087428	1	0	*KDELR3*	2	-0.15276	1	0
SPATA6L	3	-0.087369	1	0	*KCNE5*	4	-0.15253	1	0
AMN1	4	-0.087283	1	0	*GPX1*	3	-0.15245	1	0
FAM177A1	4	-0.087267	1	0	*PAH*	1	-0.15225	1	0
TWISTNB	1	-0.087166	1	0	*FAM114A2*	4	-0.15195	1	0
THOC1	3	-0.087129	1	0	*HMGN5*	3	-0.15186	1	0
FUT10	3	-0.087105	1	0	*THRB*	4	-0.15175	1	0
ISOC2	3	-0.086997	1	0	*TDG*	3	-0.15162	1	0
TEX2	4	-0.086995	1	0	*TCF20*	3	-0.1516	1	0
ALDH3B1	4	-0.086858	1	0	*GORASP1*	4	-0.15157	1	0
TENM1	4	-0.08679	1	0	*KRT78*	2	-0.15149	1	0
PCK1	4	-0.086765	1	0	*CHID1*	4	-0.15133	1	0
TRIM16	3	-0.086746	1	0	*ARID4A*	3	-0.15131	1	0
HPS4	3	-0.086741	1	0	*GPR176*	4	-0.1513	1	0
ZBTB24	3	-0.086537	1	0	*PLA2G2A*	4	-0.15127	1	0
ASZ1	4	-0.086469	1	0	*LRRK2*	3	-0.15125	1	0
TESC	3	-0.086447	1	0	*FBXO8*	3	-0.15101	1	0
GMIP	3	-0.086353	1	0	*VEGFC*	4	-0.15091	1	0
CSF1	2	-0.086189	1	0	*HDGF*	2	-0.15078	1	0
HSFX2	1	-0.086188	1	0	*TCFL5*	3	-0.15075	1	0
TUBA1A	3	-0.086158	1	0	*AKAP10*	4	-0.15074	1	0
BCO1	4	-0.08611	1	0	*CUBN*	4	-0.15069	1	0
PLOD1	4	-0.086101	1	0	*SPC25*	3	-0.15065	1	0
IFITM5	3	-0.086056	1	0	*PTPRE*	3	-0.15055	1	0
TRIM5	3	-0.086034	1	0	*AP4E1*	4	-0.15046	1	0
SRD5A3	4	-0.086008	1	0	*ROGDI*	4	-0.15045	1	0

SUGT1	2	-0.08598	1	0	*ZNF345*	4	-0.15041	1	0
TMEM144	4	-0.085871	1	0	*PUS7*	3	-0.1504	1	0
SOCS6	4	-0.085813	1	0	*DNAJC28*	4	-0.15037	1	0
ERGIC3	4	-0.085768	1	0	*CKS2*	3	-0.15032	1	0
MAP2	4	-0.085751	1	0	*DPY19L3*	4	-0.15032	1	0
PPM1F	2	-0.085698	1	0	*CHST13*	4	-0.15026	1	0
TBC1D3I	1	-0.085631	1	0	*TP53*	4	-0.14983	1	0
CDC14A	4	-0.085628	1	0	*LPO*	4	-0.1498	1	0
ADAMTS20	2	-0.085565	1	0	*GLIPR1*	4	-0.14979	1	0
IRF1	1	-0.085531	1	0	*NEXMIF*	4	-0.14973	1	0
SPR	4	-0.085493	1	0	*MAGT1*	3	-0.14964	1	0
TBK1	4	-0.085451	1	0	*DDX43*	4	-0.14964	1	0
PRPF38B	4	-0.085422	1	0	*IL17RC*	2	-0.14959	1	0
LXN	4	-0.08539	1	0	*PGPEP1L*	4	-0.1494	1	0
COL25A1	4	-0.085369	1	0	*TMC1*	4	-0.14926	1	0
DCDC1	7	-0.085345	1	0	*CHST9*	4	-0.14916	1	0
OCLN	3	-0.085344	1	0	*IL2RA*	4	-0.14916	1	0
GDAP1L1	4	-0.085299	1	0	*SERPINA1*	3	-0.14915	1	0
DCLRE1B	4	-0.085241	1	0	*RNF168*	3	-0.14911	1	0
HEATR9	4	-0.085216	1	0	*GRIN3B*	3	-0.14899	1	0
SLC37A4	4	-0.085049	1	0	*TNS3*	4	-0.14879	1	0
SCCPDH	2	-0.08497	1	0	*ORAI1*	4	-0.14865	1	0
MMP14	4	-0.084962	1	0	*GPATCH11*	3	-0.14862	1	0
MDGA1	4	-0.084919	1	0	*BCR*	4	-0.14862	1	0
FADS3	4	-0.084879	1	0	*CCDC80*	4	-0.14846	1	0
MRRF	4	-0.084787	1	0	*PIK3R1*	4	-0.14841	1	0
LRRC36	2	-0.084768	1	0	*MGARP*	3	-0.14839	1	0
RNF121	3	-0.084634	1	0	*SYN1*	3	-0.14839	1	0
ADAL	4	-0.084568	1	0	*CPA4*	3	-0.14835	1	0
VANGL2	3	-0.084546	1	0	*NENF*	4	-0.14832	1	0
SLC38A3	4	-0.084502	1	0	*RSPH9*	3	-0.14824	1	0
POGLUT2	4	-0.084441	1	0	*TRAF3IP2*	4	-0.14818	1	0
KDM1B	4	-0.08441	1	0	*RNF185*	3	-0.14815	1	0
HOXC6	4	-0.084328	1	0	*CHMP2B*	3	-0.1481	1	0
KRT25	4	-0.084262	1	0	*TIMP3*	4	-0.14807	1	0
ADM	4	-0.084261	1	0	*KLHL10*	4	-0.14798	1	0
TATDN2	4	-0.084219	1	0	*GTF2H2C*	1	-0.14795	1	0
NEIL1	3	-0.084216	1	0	*NUDT7*	4	-0.14789	1	0
USP4	4	-0.084181	1	0	*YAP1*	3	-0.14789	1	0
FGD1	4	-0.08416	1	0	*SLC35E2A*	1	-0.14787	1	0
AP1G1	4	-0.084126	1	0	*PHKG2*	4	-0.14784	1	0
SLC66A3	3	-0.084118	1	0	*PRRT2*	3	-0.14782	1	0
SBSPON	1	-0.084104	1	0	*ZBTB32*	2	-0.14773	1	0
HS2ST1	4	-0.084071	1	0	*GUCA1B*	4	-0.14772	1	0
ANG	4	-0.084056	1	0	*ANXA6*	4	-0.14769	1	0
SAMD7	2	-0.084037	1	0	*NWD1*	2	-0.14753	1	0
APLN	3	-0.083966	1	0	*CAPZA3*	3	-0.14752	1	0
GJB3	2	-0.083913	1	0	*APAF1*	4	-0.14725	1	0
PIK3C2G	4	-0.083797	1	0	*DFFB*	4	-0.1472	1	0
FAM186B	3	-0.083755	1	0	*METTL6*	3	-0.14717	1	0
PTP4A3	4	-0.083692	1	0	*ZNF845*	4	-0.14715	1	0
PRAP1	3	-0.083684	1	0	*FAM151A*	3	-0.14715	1	0
TRIQK	4	-0.083545	1	0	*STXBP4*	4	-0.14713	1	0
GNS	4	-0.083528	1	0	*MED28*	3	-0.14709	1	0
POLR2J2	4	-0.083506	1	0	*CNTFR*	4	-0.14697	1	0
PYGL	3	-0.083395	1	0	*SCG3*	4	-0.14688	1	0
STXBP4	4	-0.083387	1	0	*TOMM6*	3	-0.14678	1	0
NIPSNAP3B	4	-0.083333	1	0	*ERVW-1*	3	-0.14678	1	0

UHRF1	3	-0.083321	1	0	*VPS8*	4	-0.14672	1	0
TLX1	3	-0.083294	1	0	*SHC1*	4	-0.14658	1	0
PPP1R21	4	-0.083288	1	0	*FUT8*	3	-0.14653	1	0
OSBPL10	4	-0.08328	1	0	*LRRC4B*	4	-0.14649	1	0
CENPB	2	-0.083131	1	0	*ACACA*	4	-0.14645	1	0
PDP2	4	-0.082957	1	0	*TMPPE*	3	-0.14617	1	0
HMG20B	4	-0.082942	1	0	*IL36B*	4	-0.14616	1	0
SRPX	4	-0.082899	1	0	*EDF1*	4	-0.14603	1	0
CHRNE	4	-0.082873	1	0	*JAK3*	4	-0.14596	1	0
B3GALNT1	4	-0.082841	1	0	*GNAO1*	4	-0.14587	1	0
RPL6	1	-0.082786	1	0	*MALT1*	3	-0.14582	1	0
RNF181	2	-0.082744	1	0	*WFIKKN1*	4	-0.1457	1	0
LZTR1	3	-0.082677	1	0	*CST1*	3	-0.14566	1	0
PPP1R37	4	-0.082643	1	0	*FEM1A*	3	-0.14562	1	0
PSD3	4	-0.08263	1	0	*SLC2A3*	4	-0.14554	1	0
FAM161B	4	-0.082512	1	0	*AJAP1*	3	-0.14532	1	0
MYOM1	3	-0.08248	1	0	*CATIP*	3	-0.14525	1	0
HSPA8	4	-0.082452	1	0	*ABCC12*	4	-0.14521	1	0
ITGA5	4	-0.082355	1	0	*HSD11B1L*	2	-0.1452	1	0
L2HGDH	3	-0.082342	1	0	*PTPN22*	4	-0.1451	1	0
CACUL1	4	-0.082333	1	0	*OPHN1*	2	-0.145	1	0
RNF225	4	-0.082326	1	0	*IAPP*	3	-0.14491	1	0
MKI67	4	-0.082311	1	0	*SLC38A9*	4	-0.1449	1	0
C20orf27	3	-0.082239	1	0	*CCAR2*	3	-0.14487	1	0
UBASH3A	4	-0.08216	1	0	*CRLF2*	2	-0.14485	1	0
H4C8	4	-0.082117	1	0	*KLHDC7B*	3	-0.14465	1	0
PPP1R3E	3	-0.082063	1	0	*RBMS1*	4	-0.14449	1	0
BMP6	3	-0.082061	1	0	*ZNF765*	4	-0.14448	1	0
FAM104A	4	-0.081881	1	0	*NECAB2*	4	-0.14447	1	0
KRIT1	4	-0.081846	1	0	*KIAA0355*	4	-0.1444	1	0
C19orf48	4	-0.081723	1	0	*WEE2*	3	-0.14437	1	0
ZNF518B	4	-0.081719	1	0	*CDC23*	3	-0.14436	1	0
THSD4	4	-0.081718	1	0	*DACT2*	4	-0.14435	1	0
TMEM121	4	-0.081654	1	0	*AEBP1*	3	-0.14434	1	0
IFIT1	4	-0.081601	1	0	*CHEK2*	4	-0.14428	1	0
PCARE	3	-0.081512	1	0	*UNC119B*	3	-0.14427	1	0
DHRS4L1	4	-0.081508	1	0	*CEP70*	4	-0.14419	1	0
TNFRSF18	4	-0.081498	1	0	*PSME2*	4	-0.14416	1	0
PARP3	2	-0.081418	1	0	*VWA1*	2	-0.14404	1	0
PSPC1	4	-0.081415	1	0	*MED12L*	4	-0.14399	1	0
C5orf49	3	-0.08141	1	0	*PCDHGB4*	3	-0.14397	1	0
PDXDC1	3	-0.081393	1	0	*TSPYL5*	4	-0.14394	1	0
TOP2B	4	-0.081385	1	0	*MAN2B2*	4	-0.14393	1	0
USP18	4	-0.08132	1	0	*NCBP2*	3	-0.14385	1	0
INPP5A	4	-0.081285	1	0	*NGF*	4	-0.14384	1	0
KIAA1328	4	-0.081168	1	0	*ZNF577*	3	-0.1438	1	0
GPATCH8	4	-0.08105	1	0	*SNX25*	4	-0.14378	1	0
PRKRA	4	-0.080951	1	0	*IMPAD1*	4	-0.1437	1	0
CORO2A	4	-0.080823	1	0	*CLEC5A*	3	-0.1437	1	0
EVA1C	2	-0.0807	1	0	*MCEE*	3	-0.14362	1	0
HHAT	4	-0.080664	1	0	*TMEM106C*	4	-0.14358	1	0
ZNF320	3	-0.080639	1	0	*ZNF572*	3	-0.1435	1	0
GTPBP6	4	-0.080607	1	0	*ZFP57*	4	-0.14348	1	0
CCDC96	3	-0.080574	1	0	*BPHL*	3	-0.14344	1	0
ENOX2	4	-0.080294	1	0	*TCF7L1*	3	-0.14338	1	0
GZMB	2	-0.080274	1	0	*MLLT6*	4	-0.14332	1	0
TNFRSF1B	4	-0.08027	1	0	*LPAR1*	3	-0.14331	1	0
SLC1A3	4	-0.080052	1	0	*IGFBP5*	4	-0.14307	1	0

OR7E154P	1	-0.079986	1	0	*TNFAIP6*	3	-0.14305	1	0
KIAA2026	3	-0.079973	1	0	*USP13*	4	-0.14296	1	0
PPID	4	-0.079936	1	0	*ZIK1*	4	-0.14291	1	0
KCNG1	4	-0.079859	1	0	*PARP14*	4	-0.1429	1	0
TRPC1	3	-0.079842	1	0	*FJX1*	4	-0.14288	1	0
SIK2	4	-0.079702	1	0	*ASB13*	4	-0.14285	1	0
ZSCAN1	3	-0.0797	1	0	*ANKRD37*	4	-0.14284	1	0
NCAPG	4	-0.079662	1	0	*MAP2K1*	3	-0.14273	1	0
CLIC1	4	-0.079618	1	0	*RALGPS2*	4	-0.14257	1	0
UBC	4	-0.079584	1	0	*SLFNL1*	2	-0.14255	1	0
ZSCAN18	4	-0.079534	1	0	*C10orf82*	4	-0.14252	1	0
LHX1	3	-0.079533	1	0	*EAF1*	3	-0.14236	1	0
DUSP3	4	-0.079446	1	0	*KLF14*	4	-0.14236	1	0
CREBL2	4	-0.079412	1	0	*SMAD5*	3	-0.14236	1	0
SCML4	4	-0.079387	1	0	*EVA1B*	2	-0.14233	1	0
SLC2A10	4	-0.079323	1	0	*PXT1*	4	-0.14228	1	0
PI4K2A	3	-0.079317	1	0	*ECI1*	3	-0.14222	1	0
GDF15	4	-0.079265	1	0	*FXR2*	3	-0.1421	1	0
SLC12A6	3	-0.079251	1	0	*AK4*	3	-0.14206	1	0
EN1	1	-0.079184	1	0	*CCDC113*	3	-0.14204	1	0
PLK1	4	-0.079138	1	0	*C19orf47*	3	-0.14203	1	0
PCTP	4	-0.07907	1	0	*RECK*	4	-0.14192	1	0
SCN4B	4	-0.079041	1	0	*NAP1L5*	4	-0.14191	1	0
TAOK2	4	-0.079012	1	0	*ZNHIT3*	3	-0.14176	1	0
GRAMD2B	3	-0.079002	1	0	*C5orf67*	4	-0.14175	1	0
ARL11	3	-0.07887	1	0	*KLHDC10*	4	-0.14173	1	0
TSPYL2	2	-0.078818	1	0	*LDAH*	4	-0.14168	1	0
LRRC25	3	-0.078662	1	0	*LMBR1*	4	-0.14163	1	0
ELK1	3	-0.078578	1	0	*SLC36A1*	4	-0.14155	1	0
SHCBP1L	3	-0.078504	1	0	*FAM43B*	4	-0.14146	1	0
C3orf36	3	-0.078472	1	0	*MYLPF*	4	-0.14138	1	0
TEX55	4	-0.078371	1	0	*LTN1*	4	-0.14138	1	0
C6orf52	3	-0.078305	1	0	*OPN5*	4	-0.14136	1	0
IGSF10	3	-0.078279	1	0	*YTHDF3*	4	-0.14133	1	0
MPL	4	-0.078255	1	0	*NTPCR*	3	-0.14126	1	0
ISG20	3	-0.078244	1	0	*DRP2*	3	-0.14125	1	0
C15orf41	2	-0.078226	1	0	*ADH6*	4	-0.14113	1	0
CDAN1	4	-0.078175	1	0	*TCEAL8*	3	-0.14104	1	0
VWA1	2	-0.078145	1	0	*CTU2*	3	-0.14086	1	0
ZNF671	2	-0.078127	1	0	*H2BC18*	1	-0.14053	1	0
SIAH2	4	-0.078094	1	0	*BAK1*	4	-0.14048	1	0
CYTH1	4	-0.078073	1	0	*SCARB2*	4	-0.14044	1	0
ALKBH5	1	-0.078061	1	0	*MCHR1*	4	-0.14042	1	0
NIPSNAP1	4	-0.077999	1	0	*CYP4Z1*	2	-0.14039	1	0
SLC2A5	4	-0.077908	1	0	*CRABP2*	1	-0.14019	1	0
OTUD3	4	-0.077798	1	0	*CACNG6*	3	-0.14017	1	0
NCF2	4	-0.077729	1	0	*PYGB*	3	-0.14009	1	0
FBXO41	4	-0.077701	1	0	*MAGI1*	4	-0.13998	1	0
SYT14	4	-0.077692	1	0	*HMGCLL1*	3	-0.13996	1	0
TXNRD1	3	-0.077621	1	0	*DZANK1*	3	-0.1399	1	0
SESN3	3	-0.077578	1	0	*ZNF582*	3	-0.1398	1	0
PGPEP1L	4	-0.077505	1	0	*C2orf83*	4	-0.13976	1	0
FGD2	3	-0.077471	1	0	*CPLANE1*	2	-0.13973	1	0
TMEM62	4	-0.077465	1	0	*ITGB2*	2	-0.13964	1	0
LGI2	2	-0.077452	1	0	*AIMP1*	2	-0.13952	1	0
GPER1	3	-0.077446	1	0	*TRIP13*	3	-0.13952	1	0
SEZ6L2	4	-0.077386	1	0	*PRICKLE2*	4	-0.13944	1	0
ZNF609	3	-0.077337	1	0	*PSIP1*	4	-0.13936	1	0

TBPL1	4	-0.07731	1	0	*MACROH2A1*	2	-0.1393	1	0
NRAS	3	-0.077303	1	0	*FOXE3*	4	-0.13925	1	0
TPP1	4	-0.077241	1	0	*NOTO*	3	-0.13921	1	0
PPP1R1A	3	-0.077114	1	0	*GCC1*	2	-0.13916	1	0
IGSF22	3	-0.077064	1	0	*PRDM12*	3	-0.13914	1	0
MTNR1A	4	-0.077064	1	0	*BCO1*	4	-0.13913	1	0
ZNF445	2	-0.077018	1	0	*GSC*	4	-0.13907	1	0
SVIP	4	-0.077011	1	0	*TEX45*	4	-0.13907	1	0
UBXN1	3	-0.076856	1	0	*DDC*	4	-0.13905	1	0
SP140L	4	-0.076681	1	0	*ATP2B4*	4	-0.13902	1	0
MARCHF8	4	-0.076608	1	0	*ALKAL2*	3	-0.13899	1	0
PCDHA7	4	-0.076577	1	0	*IER5L*	4	-0.1389	1	0
PFKFB2	4	-0.076529	1	0	*EPHA5*	4	-0.13887	1	0
MARVELD3	2	-0.076469	1	0	*MORN2*	4	-0.13884	1	0
MAP3K5	4	-0.076437	1	0	*GRPEL2*	3	-0.13881	1	0
ETAA1	3	-0.076405	1	0	*RNF138*	2	-0.1388	1	0
MARK1	4	-0.076388	1	0	*KDM5D*	3	-0.13876	1	0
MACC1	4	-0.076363	1	0	*TTC16*	3	-0.13874	1	0
HSCB	4	-0.076313	1	0	*PPM1L*	4	-0.13869	1	0
GKAP1	4	-0.076302	1	0	*APH1B*	4	-0.13869	1	0
HPS3	4	-0.076218	1	0	*FAT4*	3	-0.13862	1	0
DDR2	4	-0.076161	1	0	*SPATA31C2*	3	-0.13858	1	0
THAP5	3	-0.07613	1	0	*HPS6*	4	-0.13857	1	0
TLDC2	4	-0.076104	1	0	*TPCN1*	4	-0.1384	1	0
F2RL3	3	-0.076053	1	0	*PPP3CC*	4	-0.13818	1	0
CACFD1	3	-0.075922	1	0	*PDXDC1*	3	-0.13808	1	0
SCYL3	2	-0.075874	1	0	*TRMT44*	3	-0.138	1	0
XYLB	4	-0.075869	1	0	*CPVL*	4	-0.13796	1	0
ARHGEF10	4	-0.075748	1	0	*NFE2L2*	3	-0.13791	1	0
VIL1	4	-0.075584	1	0	*CCDC120*	4	-0.13783	1	0
SLC22A14	4	-0.07558	1	0	*OR13A1*	4	-0.13783	1	0
PVR	4	-0.075557	1	0	*CEBPD*	2	-0.13782	1	0
C3	4	-0.075505	1	0	*CYP7B1*	4	-0.13778	1	0
NRBP2	4	-0.075364	1	0	*TOR1A*	4	-0.13773	1	0
CDC42EP3	3	-0.07535	1	0	*VWA7*	4	-0.13762	1	0
PPP1R9A	4	-0.075315	1	0	*WBP2*	4	-0.13756	1	0
NEU1	4	-0.075181	1	0	*PHC1*	1	-0.13755	1	0
CACNB2	3	-0.075084	1	0	*VIL1*	4	-0.13751	1	0
MRNIP	3	-0.075067	1	0	*ATXN1*	4	-0.13745	1	0
ITGA4	4	-0.074927	1	0	*THBS3*	4	-0.13744	1	0
LRRC31	4	-0.074912	1	0	*CCBE1*	4	-0.13735	1	0
FAM221B	4	-0.074864	1	0	*MON2*	4	-0.13728	1	0
CBX5	4	-0.074743	1	0	*PMEL*	1	-0.13728	1	0
NPFFR1	3	-0.074659	1	0	*TULP4*	4	-0.13717	1	0
KIF3B	4	-0.074571	1	0	*CCDC15*	4	-0.13715	1	0
SEMA4G	2	-0.074477	1	0	*FAM170A*	4	-0.13714	1	0
TGFBR1	4	-0.074405	1	0	*C12orf42*	3	-0.1371	1	0
ATP12A	4	-0.074359	1	0	*GPR137*	4	-0.13707	1	0
SLC35F5	4	-0.074343	1	0	*MYO5C*	4	-0.13698	1	0
SCARB1	4	-0.074321	1	0	*XIRP1*	4	-0.1369	1	0
SCN7A	4	-0.074241	1	0	*H3C1*	3	-0.13677	1	0
SLC22A23	3	-0.074119	1	0	*PARD6G*	4	-0.13668	1	0
DCBLD1	4	-0.074083	1	0	*MN1*	4	-0.13662	1	0
CNTN2	3	-0.074074	1	0	*AK7*	3	-0.13662	1	0
FGFR1OP2	4	-0.07406	1	0	*ZNF750*	4	-0.13645	1	0
VAMP5	4	-0.074054	1	0	*UNC5A*	4	-0.13643	1	0
RDH10	4	-0.073995	1	0	*EPHX2*	4	-0.13637	1	0
ZBED8	4	-0.073994	1	0	*ZNF609*	3	-0.13624	1	0

INSYN2B	4	-0.073937	1	0	*ELP6*	4	-0.13624	1	0
ITPA	4	-0.073896	1	0	*PSMA8*	4	-0.13618	1	0
CD68	3	-0.073895	1	0	*PHLDA1*	4	-0.13615	1	0
GOLGA8K	1	-0.073879	1	0	*SYTL3*	4	-0.13608	1	0
ARHGAP36	4	-0.073832	1	0	*OXTR*	4	-0.13603	1	0
CERKL	4	-0.073618	1	0	*PRSS23*	4	-0.13603	1	0
SDK1	4	-0.073582	1	0	*MAPK1*	3	-0.13595	1	0
RIN3	4	-0.073527	1	0	*ZBED6*	4	-0.13587	1	0
MORC3	3	-0.073522	1	0	*MMP17*	2	-0.13585	1	0
ODAPH	4	-0.073514	1	0	*MTMR7*	4	-0.13579	1	0
OR2D2	4	-0.073508	1	0	*VCAN*	3	-0.13576	1	0
BCL7C	4	-0.073466	1	0	*NOL4*	3	-0.1357	1	0
ZNF778	4	-0.073388	1	0	*CRISP3*	4	-0.13569	1	0
NUBP2	4	-0.073358	1	0	*EGR3*	4	-0.13564	1	0
TUBB1	4	-0.073325	1	0	*GTF2A1*	4	-0.13547	1	0
RAB19	4	-0.07329	1	0	*SYNJ2*	4	-0.13535	1	0
NUDT14	4	-0.073172	1	0	*TCP10L*	2	-0.13529	1	0
SPATA25	4	-0.073153	1	0	*SOGA1*	3	-0.13528	1	0
BIRC6	4	-0.073117	1	0	*MAL2*	3	-0.13527	1	0
ZBTB8B	4	-0.073069	1	0	*TMEM262*	4	-0.13525	1	0
FEZ1	4	-0.073022	1	0	*NOTCH4*	4	-0.13511	1	0
ATP5PF	3	-0.072988	1	0	*EIF2D*	4	-0.13503	1	0
PRSS16	4	-0.072973	1	0	*KLHL41*	2	-0.13496	1	0
KHDRBS2	3	-0.072945	1	0	*HORMAD1*	4	-0.13491	1	0
DTWD2	3	-0.072888	1	0	*CD99*	4	-0.13487	1	0
DESI2	3	-0.07287	1	0	*TMEM52B*	4	-0.13486	1	0
BNIPL	4	-0.072807	1	0	*CADPS2*	4	-0.13485	1	0
CKB	4	-0.072781	1	0	*C21orf58*	4	-0.13485	1	0
PARP4	3	-0.072754	1	0	*PPP3CB*	4	-0.13485	1	0
FOXC2	2	-0.072741	1	0	*MOSPD2*	3	-0.13483	1	0
HEBP2	3	-0.072737	1	0	*RAB22A*	4	-0.13481	1	0
TCOF1	4	-0.072697	1	0	*MEMO1*	4	-0.13479	1	0
GPR83	4	-0.072647	1	0	*JUND*	4	-0.13476	1	0
FN1	4	-0.072524	1	0	*KIAA1522*	4	-0.1347	1	0
PRSS22	4	-0.07247	1	0	*EP300*	3	-0.13467	1	0
CASTOR3	2	-0.07242	1	0	*MED15*	4	-0.13449	1	0
GUF1	4	-0.072375	1	0	*TYR*	4	-0.13437	1	0
JRK	4	-0.072311	1	0	*MANSC1*	3	-0.13427	1	0
KIF4B	4	-0.072301	1	0	*PCDHGA9*	3	-0.13419	1	0
CCDC87	4	-0.072268	1	0	*CAV3*	4	-0.13409	1	0
SPRN	4	-0.072248	1	0	*CEP76*	4	-0.13402	1	0
FIG4	4	-0.072215	1	0	*FRRS1L*	4	-0.13369	1	0
ARID4B	4	-0.072134	1	0	*CC2D2A*	4	-0.13367	1	0
PITX3	4	-0.072114	1	0	*HKDC1*	4	-0.13362	1	0
FERMT1	4	-0.072097	1	0	*MAMDC2*	4	-0.13361	1	0
KLF13	3	-0.071982	1	0	*KCNK15*	1	-0.13358	1	0
RPL29	1	-0.071892	1	0	*LINC02693*	4	-0.13354	1	0
TUB	3	-0.071755	1	0	*GLI2*	2	-0.13348	1	0
CRELD2	3	-0.071503	1	0	*HMG20A*	4	-0.13333	1	0
RFTN1	4	-0.071466	1	0	*MAST2*	4	-0.13331	1	0
IL2RA	4	-0.071459	1	0	*CYP2J2*	4	-0.13318	1	0
POMGNT1	3	-0.071378	1	0	*RAPH1*	3	-0.1331	1	0
B3GNT7	4	-0.071346	1	0	*DAB2IP*	4	-0.13304	1	0
CNOT1	4	-0.071199	1	0	*TUBA1A*	3	-0.13301	1	0
MAST4	4	-0.071153	1	0	*BCAR1*	2	-0.13293	1	0
HCLS1	4	-0.071079	1	0	*ADCYAP1R1*	3	-0.1329	1	0
PLAAT2	3	-0.070983	1	0	*TRIM37*	4	-0.13285	1	0
CRISPLD2	4	-0.070966	1	0	*RAC3*	3	-0.13281	1	0

MYBL1	4	-0.070889	1	0	*LMTK2*	4	-0.13281	1	0
AIDA	2	-0.070845	1	0	*C14orf119*	4	-0.13279	1	0
PAQR8	2	-0.07084	1	0	*RSAD1*	4	-0.13259	1	0
ASAH2	3	-0.070786	1	0	*MFAP5*	3	-0.13258	1	0
TMEM260	4	-0.070723	1	0	*GYG2*	4	-0.13253	1	0
RAB17	3	-0.070701	1	0	*NICN1*	2	-0.13217	1	0
MAN1B1	2	-0.07068	1	0	*TIGAR*	3	-0.13214	1	0
DUSP11	4	-0.070566	1	0	*HSD11B1*	4	-0.13208	1	0
SRPK2	3	-0.070379	1	0	*RO60*	4	-0.13203	1	0
GLB1	3	-0.07026	1	0	*PILRA*	4	-0.13202	1	0
DDHD1	4	-0.070179	1	0	*LURAP1L*	4	-0.13193	1	0
EBF3	2	-0.07016	1	0	*ARRB1*	2	-0.13191	1	0
PRR15	4	-0.069992	1	0	*DNAH7*	4	-0.13186	1	0
ARFGAP1	3	-0.069939	1	0	*CCDC30*	4	-0.13185	1	0
CUL2	4	-0.069806	1	0	*C16orf71*	3	-0.13183	1	0
MRPL16	3	-0.069707	1	0	*EPX*	4	-0.13153	1	0
HMG20A	4	-0.069702	1	0	*GSDMB*	4	-0.1315	1	0
CBLL1	4	-0.069638	1	0	*THAP1*	4	-0.13148	1	0
ATP2C1	3	-0.069632	1	0	*CUL4B*	2	-0.1314	1	0
EBAG9	3	-0.069515	1	0	*ABI3BP*	4	-0.13127	1	0
KHDRBS3	4	-0.069468	1	0	*KPNA5*	4	-0.13126	1	0
FADS1	3	-0.069444	1	0	*KMT5B*	3	-0.13123	1	0
TASOR	4	-0.069421	1	0	*HECW2*	4	-0.13122	1	0
SLC39A11	4	-0.069192	1	0	*INVS*	4	-0.13121	1	0
USPL1	4	-0.069081	1	0	*FAM131A*	4	-0.13121	1	0
AP1G2	4	-0.06899	1	0	*LIPJ*	4	-0.13114	1	0
TBC1D31	4	-0.068976	1	0	*PPP1R2*	4	-0.1311	1	0
WDR66	4	-0.068964	1	0	*BRICD5*	4	-0.13107	1	0
HP1BP3	4	-0.06893	1	0	*POMGNT1*	3	-0.13098	1	0
MSI2	4	-0.068854	1	0	*IDNK*	4	-0.13097	1	0
TPCN2	4	-0.068792	1	0	*LOC388282*	3	-0.13085	1	0
C8orf88	4	-0.068745	1	0	*DDX3Y*	4	-0.13073	1	0
OST4	4	-0.068736	1	0	*RPS9*	2	-0.13073	1	0
DYNC1LI1	4	-0.068672	1	0	*DUSP2*	3	-0.13069	1	0
CFH	4	-0.068626	1	0	*CREB3L4*	4	-0.13067	1	0
CBFA2T2	4	-0.068513	1	0	*GRB10*	4	-0.1306	1	0
SMOX	4	-0.068441	1	0	*MAP9*	2	-0.13053	1	0
EPHA6	3	-0.068411	1	0	*NOMO2*	2	-0.13037	1	0
CYP2C19	4	-0.068346	1	0	*THUMPD2*	4	-0.13026	1	0
PNMA5	4	-0.068346	1	0	*OR52N4*	3	-0.13022	1	0
TRIM24	4	-0.068325	1	0	*PRDM6*	3	-0.13002	1	0
SERPINA10	4	-0.068279	1	0	*B4GALNT3*	3	-0.12998	1	0
AIPL1	3	-0.068245	1	0	*C6orf163*	4	-0.1299	1	0
SMARCAL1	4	-0.068152	1	0	*AKR7A3*	4	-0.12985	1	0
H1-8	4	-0.068117	1	0	*ZBTB18*	4	-0.12982	1	0
EDARADD	3	-0.067949	1	0	*NDRG3*	2	-0.12976	1	0
NIPAL1	2	-0.067889	1	0	*MPDU1*	4	-0.12973	1	0
LAMC2	4	-0.067767	1	0	*KIFC2*	4	-0.12967	1	0
RXRA	3	-0.067752	1	0	*LY6G5B*	4	-0.12956	1	0
KRTCAP3	3	-0.06767	1	0	*NCOA5*	4	-0.12948	1	0
MMP7	4	-0.067611	1	0	*TMEM243*	4	-0.12934	1	0
FAM174A	4	-0.067582	1	0	*DENND11*	2	-0.1292	1	0
FNBP1L	3	-0.067517	1	0	*TENM3*	4	-0.12918	1	0
RTL6	4	-0.067452	1	0	*CCR2*	4	-0.12917	1	0
RABL2A	1	-0.067372	1	0	*OGFOD2*	4	-0.12909	1	0
ALOX15	4	-0.067307	1	0	*NUDT5*	4	-0.12904	1	0
ZDHHC5	4	-0.067244	1	0	*ERCC6L2*	4	-0.12902	1	0
GRM3	4	-0.067152	1	0	*TP53I13*	4	-0.12882	1	0

CHRM3	4	-0.067075	1	0	*UROC1*	4	-0.12875	1	0
KLB	3	-0.067033	1	0	*CDK5R2*	4	-0.12874	1	0
PGM2L1	3	-0.067015	1	0	*TNFSF10*	4	-0.12869	1	0
MYBPH	3	-0.066919	1	0	*IRS1*	4	-0.12835	1	0
SLC30A3	4	-0.066893	1	0	*SERPINE3*	4	-0.12822	1	0
FOLR1	4	-0.066892	1	0	*IL4R*	4	-0.12815	1	0
C1RL	3	-0.066712	1	0	*TNKS2*	4	-0.12792	1	0
HSPB11	4	-0.066695	1	0	*TTI2*	3	-0.12787	1	0
DCST2	4	-0.06669	1	0	*CSRNP1*	4	-0.12775	1	0
SNPH	3	-0.066564	1	0	*CXCL10*	4	-0.12763	1	0
RNF128	3	-0.066533	1	0	*NEO1*	4	-0.12752	1	0
ZNF169	3	-0.066496	1	0	*PLAGL2*	3	-0.12732	1	0
C1orf52	4	-0.066414	1	0	*CLN5*	1	-0.12729	1	0
OR6A2	4	-0.066184	1	0	*CCR3*	4	-0.12728	1	0
GCK	3	-0.066179	1	0	*C1D*	2	-0.1272	1	0
FAM184B	4	-0.066171	1	0	*SBF1*	3	-0.12718	1	0
ZNF483	4	-0.066157	1	0	*CCDC9*	4	-0.12714	1	0
RHOBTB1	4	-0.066084	1	0	*FBXO36*	4	-0.1271	1	0
CHPF2	4	-0.065911	1	0	*IARS1*	4	-0.12706	1	0
THRA	4	-0.065901	1	0	*CASC1*	4	-0.12704	1	0
NSRP1	4	-0.065881	1	0	*HHIP*	4	-0.127	1	0
MPDU1	4	-0.065782	1	0	*CREB5*	4	-0.12699	1	0
UFM1	3	-0.065706	1	0	*IL15*	4	-0.12697	1	0
RNF141	4	-0.065705	1	0	*FAM81B*	3	-0.12695	1	0
TRPM8	3	-0.065704	1	0	*COQ9*	4	-0.12693	1	0
SNRPB2	3	-0.065609	1	0	*ZNF850*	4	-0.12686	1	0
XRCC1	4	-0.065584	1	0	*DOCK5*	4	-0.12681	1	0
FAR2	3	-0.065558	1	0	*SHISAL2B*	4	-0.12677	1	0
TMEM158	4	-0.065506	1	0	*PVR*	4	-0.12674	1	0
KRTAP5-8	3	-0.065478	1	0	*ACSM5*	3	-0.12669	1	0
CEP152	4	-0.065374	1	0	*C20orf27*	3	-0.12669	1	0
GATD1	4	-0.065299	1	0	*CCDC81*	2	-0.12664	1	0
TUBA3C	4	-0.065073	1	0	*SYCP2L*	4	-0.12661	1	0
HIVEP1	4	-0.064886	1	0	*STRADB*	4	-0.12659	1	0
HMGN3	4	-0.064845	1	0	*FAM32A*	3	-0.12654	1	0
CAVIN2	4	-0.064747	1	0	*SUMO3*	4	-0.12647	1	0
COX7C	4	-0.064684	1	0	*GJB5*	3	-0.12632	1	0
PCDHGB1	3	-0.064684	1	0	*RWDD2B*	4	-0.12631	1	0
SLC25A4	4	-0.064682	1	0	*GAL*	2	-0.12618	1	0
ARAP3	2	-0.064492	1	0	*C15orf56*	4	-0.12607	1	0
GUCA1C	4	-0.06449	1	0	*STYK1*	3	-0.12603	1	0
PRORP	4	-0.064439	1	0	*B4GALNT1*	4	-0.12595	1	0
SPI1	4	-0.064336	1	0	*NKAIN4*	3	-0.12592	1	0
ERP44	4	-0.064335	1	0	*LRFN1*	4	-0.1259	1	0
NOVA1	2	-0.064239	1	0	*CD58*	4	-0.12588	1	0
DLGAP3	4	-0.064209	1	0	*TTC28*	3	-0.12587	1	0
KRT79	4	-0.064156	1	0	*BEND5*	3	-0.12578	1	0
DMRTA2	2	-0.064071	1	0	*KNDC1*	4	-0.12575	1	0
ALOX12	4	-0.064028	1	0	*RFC4*	3	-0.12574	1	0
BBX	3	-0.063956	1	0	*KNL1*	4	-0.12568	1	0
FAM20B	2	-0.063951	1	0	*TMEM107*	4	-0.12567	1	0
DYRK2	4	-0.063941	1	0	*ACP5*	4	-0.12561	1	0
SLFN5	4	-0.063906	1	0	*SLC39A8*	2	-0.12554	1	0
PHC3	4	-0.063857	1	0	*FAM166C*	3	-0.12547	1	0
SULF1	4	-0.063807	1	0	*PPP2R2C*	4	-0.12546	1	0
ETNPPL	4	-0.063796	1	0	*UBXN1*	3	-0.12545	1	0
MED17	2	-0.063736	1	0	*SEMA4C*	4	-0.1254	1	0
GPR75-ASB3	1	-0.06371	1	0	*UCP1*	3	-0.12535	1	0

POTEA	4	-0.063651	1	0	*TPRA1*	4	-0.12527	1	0
SLC25A24	4	-0.063637	1	0	*GUCY2D*	4	-0.12525	1	0
RHOBTB2	4	-0.063604	1	0	*ACO1*	2	-0.12514	1	0
NVL	4	-0.063461	1	0	*CSKMT*	3	-0.12512	1	0
NASP	4	-0.06338	1	0	*WWC3*	3	-0.12505	1	0
ZPLD1	4	-0.063333	1	0	*ASZ1*	4	-0.12499	1	0
FBXO21	4	-0.063304	1	0	*ENO2*	4	-0.12498	1	0
MYBPC2	3	-0.063201	1	0	*ZNF772*	4	-0.12495	1	0
CCDC125	4	-0.063158	1	0	*GORASP2*	4	-0.12488	1	0
ELOB	4	-0.063087	1	0	*FAM216A*	3	-0.12478	1	0
GALNT15	3	-0.063055	1	0	*FAM9C*	4	-0.12473	1	0
NOS1	4	-0.062934	1	0	*COL5A1*	4	-0.12472	1	0
PDCD6	2	-0.06269	1	0	*BMX*	4	-0.12471	1	0
SLC37A1	3	-0.062616	1	0	*ZNF540*	4	-0.12471	1	0
SH2D4A	3	-0.062561	1	0	*RBM17*	4	-0.12471	1	0
IFIT5	4	-0.062496	1	0	*TSGA10*	3	-0.12462	1	0
H2AC4	4	-0.062452	1	0	*ZNF549*	4	-0.12445	1	0
UNC5C	4	-0.06245	1	0	*SNX11*	4	-0.12444	1	0
ZNF182	3	-0.062319	1	0	*SRSF10*	4	-0.12437	1	0
APMAP	4	-0.062299	1	0	*ZSWIM4*	4	-0.12435	1	0
MGAT2	4	-0.062273	1	0	*PGPEP1*	4	-0.1241	1	0
ZNF330	4	-0.062203	1	0	*PHRF1*	4	-0.12384	1	0
ARSA	4	-0.062193	1	0	*CLECL1*	4	-0.12381	1	0
NDUFA5	3	-0.06216	1	0	*SIRPB2*	4	-0.12374	1	0
TRDMT1	4	-0.062159	1	0	*C15orf54*	2	-0.12343	1	0
LRRC66	4	-0.061974	1	0	*AGO4*	3	-0.12337	1	0
TBC1D19	4	-0.061876	1	0	*ZNF606*	4	-0.12335	1	0
SRSF9	4	-0.061855	1	0	*JADE1*	3	-0.12329	1	0
SRSF10	4	-0.061839	1	0	*TMPRSS6*	4	-0.12324	1	0
RIDA	2	-0.061792	1	0	*DUSP9*	4	-0.12318	1	0
FKBP5	4	-0.06179	1	0	*GPATCH2L*	4	-0.12316	1	0
CORO1A	4	-0.061763	1	0	*PCP2*	3	-0.12311	1	0
CEP57L1	4	-0.06151	1	0	*RRP7A*	4	-0.12308	1	0
SLC5A4	4	-0.061499	1	0	*JRKL*	4	-0.123	1	0
ANGEL1	3	-0.061411	1	0	*ARHGEF37*	3	-0.12298	1	0
FTSJ1	2	-0.061365	1	0	*EIF5B*	4	-0.12281	1	0
RRP8	4	-0.061285	1	0	*CLBA1*	3	-0.12279	1	0
SLC9A3	2	-0.061228	1	0	*TAS2R50*	4	-0.12278	1	0
BCL2A1	4	-0.061203	1	0	*SLC43A1*	4	-0.12269	1	0
SH3PXD2A	4	-0.061183	1	0	*CCDC40*	3	-0.12268	1	0
MCF2L2	2	-0.061121	1	0	*CEPT1*	4	-0.12266	1	0
GRIP2	4	-0.060984	1	0	*PTGES3*	3	-0.1226	1	0
USP6	2	-0.060939	1	0	*SLC47A2*	3	-0.12253	1	0
CELF4	3	-0.060858	1	0	*ADAMTS17*	4	-0.12246	1	0
ZXDB	4	-0.060663	1	0	*TAS2R43*	4	-0.12238	1	0
AVPI1	2	-0.060656	1	0	*C22orf46*	3	-0.12232	1	0
SRPRB	4	-0.0606	1	0	*RADIL*	4	-0.12225	1	0
NID1	4	-0.06049	1	0	*EPDR1*	4	-0.12221	1	0
ANXA1	4	-0.060441	1	0	*PEAR1*	4	-0.12218	1	0
SDHAF2	4	-0.060171	1	0	*ICA1L*	4	-0.12217	1	0
SKP1	4	-0.060159	1	0	*TMEM38A*	4	-0.12216	1	0
ALLC	4	-0.060121	1	0	*FAM222B*	4	-0.12208	1	0
RGS14	4	-0.060089	1	0	*INAFM1*	4	-0.12203	1	0
PLXNB3	4	-0.060085	1	0	*SSBP2*	4	-0.12203	1	0
NUCB2	4	-0.060025	1	0	*ST6GALNAC6*	3	-0.1218	1	0
C1orf137	3	-0.059952	1	0	*AIF1*	4	-0.12178	1	0
MORN5	4	-0.059841	1	0	*PLSCR4*	3	-0.12153	1	0
MYO1B	4	-0.059702	1	0	*UBE2Q1*	4	-0.12151	1	0

MSH4	4	-0.059626	1	0	*BRCC3*	3	-0.12145	1	0
WASL	4	-0.059401	1	0	*HNRNPA0*	4	-0.12132	1	0
OR8G2P	2	-0.059309	1	0	*SPOCD1*	4	-0.12125	1	0
IFRD2	4	-0.059268	1	0	*ISL2*	4	-0.12119	1	0
TM9SF4	4	-0.059164	1	0	*CD164*	4	-0.12119	1	0
STRN3	3	-0.059156	1	0	*SLC25A45*	4	-0.12113	1	0
ZNF718	3	-0.059129	1	0	*SLC5A2*	3	-0.12109	1	0
SLC35A4	3	-0.059126	1	0	*CD34*	4	-0.12097	1	0
P3H2	3	-0.059097	1	0	*STMN1*	2	-0.12097	1	0
POLD1	2	-0.059069	1	0	*MAP11*	3	-0.12094	1	0
TMEM178A	3	-0.059032	1	0	*PGC*	4	-0.12077	1	0
GCM1	3	-0.058987	1	0	*FOXD1*	3	-0.12074	1	0
CC2D2A	4	-0.058952	1	0	*DRD2*	3	-0.12066	1	0
ALX3	4	-0.058858	1	0	*ZNF655*	4	-0.12065	1	0
ZNF93	1	-0.058857	1	0	*POMT2*	3	-0.12044	1	0
STMN3	4	-0.058855	1	0	*LHFPL2*	4	-0.12036	1	0
RAD54L2	4	-0.058836	1	0	*GPR39*	4	-0.12033	1	0
PCDHGA6	2	-0.058808	1	0	*PRKDC*	4	-0.12033	1	0
CISD1	2	-0.058721	1	0	*FLI1*	3	-0.12028	1	0
PDE4A	4	-0.058626	1	0	*KYNU*	2	-0.12023	1	0
ARHGAP32	3	-0.05852	1	0	*ADCY4*	4	-0.12016	1	0
HTT	4	-0.058518	1	0	*CLHC1*	4	-0.12014	1	0
NIT1	4	-0.058447	1	0	*CATSPERG*	2	-0.12014	1	0
PLCXD1	3	-0.058293	1	0	*ZGLP1*	4	-0.12011	1	0
OR2A1	1	-0.058289	1	0	*PIGP*	4	-0.1201	1	0
LENEP	4	-0.058219	1	0	*IZUMO1*	3	-0.12006	1	0
TAS2R19	3	-0.058216	1	0	*TBC1D2B*	3	-0.12001	1	0
STRN	3	-0.058205	1	0	*LMLN*	4	-0.11979	1	0
CHCHD3	4	-0.058153	1	0	*GAS2*	4	-0.11974	1	0
PXDNL	3	-0.05814	1	0	*KIF5C*	4	-0.11968	1	0
CCK	4	-0.05813	1	0	*ZNF608*	2	-0.11958	1	0
GPC1	3	-0.05797	1	0	*PNMA2*	4	-0.11951	1	0
PLAAT5	4	-0.057923	1	0	*CASC4*	4	-0.11949	1	0
WDR48	3	-0.057921	1	0	*C19orf44*	1	-0.11942	1	0
AGER	4	-0.057856	1	0	*SMCHD1*	4	-0.1194	1	0
WDFY1	3	-0.057719	1	0	*DACT3*	4	-0.11928	1	0
SRD5A2	4	-0.057566	1	0	*RAPGEF2*	3	-0.11913	1	0
CCDC188	2	-0.057546	1	0	*SLC2A1*	4	-0.119	1	0
NAA60	3	-0.057389	1	0	*PEF1*	4	-0.11889	1	0
C6orf141	3	-0.057378	1	0	*RAP2C*	4	-0.11889	1	0
CEP164	4	-0.057372	1	0	*MAP1LC3B*	3	-0.11878	1	0
ZNF233	4	-0.057314	1	0	*RIPK4*	4	-0.11875	1	0
KCNMB4	3	-0.057288	1	0	*ARID2*	2	-0.11871	1	0
HCAR1	4	-0.057287	1	0	*NKTR*	4	-0.11869	1	0
MYH11	2	-0.05728	1	0	*WNT2B*	3	-0.11862	1	0
ZNF433	2	-0.057084	1	0	*SAXO1*	4	-0.11859	1	0
ZDHHC16	4	-0.05702	1	0	*LPAR2*	3	-0.11842	1	0
ZNF234	4	-0.057005	1	0	*IL1RAP*	3	-0.11842	1	0
SLF1	3	-0.056965	1	0	*MPP4*	4	-0.11835	1	0
LYN	4	-0.056953	1	0	*CELF3*	3	-0.11811	1	0
MORN2	4	-0.05693	1	0	*STAT5A*	3	-0.11795	1	0
CACNG4	3	-0.056864	1	0	*MATN2*	4	-0.11776	1	0
CEP72	4	-0.056816	1	0	*PDLIM1*	3	-0.11776	1	0
SMCHD1	4	-0.05675	1	0	*CAV2*	3	-0.11773	1	0
DUSP8	4	-0.056721	1	0	*SPATA12*	3	-0.11771	1	0
SLC22A18AS	4	-0.056614	1	0	*LOX*	4	-0.11762	1	0
SRRM2	4	-0.056614	1	0	*SACS*	2	-0.11745	1	0
AEBP1	3	-0.056593	1	0	*TACSTD2*	3	-0.11744	1	0

NPSR1	4	-0.056499	1	0	*BEX2*	4	-0.11742	1	0
ETNK1	4	-0.056451	1	0	*TNF*	4	-0.11734	1	0
NIPA1	4	-0.056434	1	0	*TMEM236*	4	-0.1173	1	0
BAK1	4	-0.056428	1	0	*GLUD2*	3	-0.11728	1	0
MOB3C	4	-0.056423	1	0	*DCTPP1*	4	-0.11722	1	0
DAGLB	4	-0.056403	1	0	*PINLYP*	3	-0.11721	1	0
LMF2	4	-0.056403	1	0	*ITGA2*	4	-0.11721	1	0
RAP1B	2	-0.056403	1	0	*PRSS16*	4	-0.11712	1	0
PIP5KL1	3	-0.056276	1	0	*MYSM1*	4	-0.1169	1	0
ARID1A	4	-0.056148	1	0	*GNB4*	4	-0.11683	1	0
TMEM256	2	-0.056108	1	0	*FERMT1*	4	-0.11682	1	0
FOXP4	4	-0.056085	1	0	*TUBA3C*	4	-0.11682	1	0
ALS2CL	3	-0.056082	1	0	*MSH4*	4	-0.11682	1	0
PLCB2	4	-0.05594	1	0	*BOLA1*	4	-0.11669	1	0
APBA3	3	-0.055937	1	0	*AP1S1*	4	-0.11667	1	0
DENND2C	4	-0.055935	1	0	*PARP6*	4	-0.11657	1	0
FBXO28	4	-0.055904	1	0	*PCDHGA11*	4	-0.11657	1	0
WIPI1	4	-0.055857	1	0	*ZNF780B*	4	-0.11655	1	0
HACE1	4	-0.055827	1	0	*PLEKHG2*	4	-0.11653	1	0
TRIML2	4	-0.055826	1	0	*KIAA0319*	4	-0.11648	1	0
PFKM	4	-0.055814	1	0	*SRI*	2	-0.11633	1	0
LAG3	4	-0.055772	1	0	*THEM6*	3	-0.11618	1	0
LAMP5	4	-0.055652	1	0	*PAQR6*	4	-0.11612	1	0
CAMK1	2	-0.055623	1	0	*SH2D4B*	4	-0.11602	1	0
GUCA1B	4	-0.055576	1	0	*LAMP3*	4	-0.11593	1	0
SLC25A42	4	-0.055555	1	0	*ZNF71*	4	-0.11581	1	0
RHOD	2	-0.055543	1	0	*BICD2*	4	-0.11575	1	0
LSM1	3	-0.055501	1	0	*IL7R*	4	-0.11571	1	0
C9orf85	4	-0.055396	1	0	*ARFGAP2*	4	-0.11569	1	0
RIPPLY3	4	-0.055372	1	0	*CYP46A1*	4	-0.11564	1	0
RTN4IP1	3	-0.055371	1	0	*TRUB1*	4	-0.11561	1	0
TRAF6	3	-0.055211	1	0	*EPHX1*	3	-0.11551	1	0
ELOVL2	2	-0.055184	1	0	*RDH10*	4	-0.1155	1	0
TANC2	4	-0.055153	1	0	*VPS26B*	3	-0.11539	1	0
APCDD1	4	-0.055126	1	0	*CDNF*	3	-0.11537	1	0
ACSBG2	4	-0.055104	1	0	*DYNC1I1*	4	-0.11524	1	0
TIGD2	4	-0.05505	1	0	*C9orf62*	4	-0.11523	1	0
HOXA11	4	-0.055011	1	0	*ZNF460*	4	-0.1152	1	0
PRRT3	4	-0.0549	1	0	*NPTN*	3	-0.11518	1	0
MEIOC	4	-0.054715	1	0	*MAB21L3*	4	-0.11513	1	0
SPDYA	3	-0.054609	1	0	*FAM122A*	4	-0.11508	1	0
LTBP3	2	-0.054576	1	0	*TRAF5*	3	-0.11495	1	0
RFC4	3	-0.054568	1	0	*HOXC6*	4	-0.11493	1	0
PPP1R10	4	-0.054521	1	0	*TWF2*	4	-0.11484	1	0
ARL6IP5	4	-0.054477	1	0	*SLC6A12*	4	-0.11476	1	0
SPTBN2	3	-0.054437	1	0	*ALDH3A2*	3	-0.11471	1	0
CCNH	3	-0.054432	1	0	*NOC3L*	3	-0.1147	1	0
PXDN	4	-0.054404	1	0	*ZNF808*	4	-0.11469	1	0
CASD1	2	-0.054397	1	0	*COL1A2*	4	-0.11464	1	0
GAST	4	-0.054361	1	0	*RASAL3*	3	-0.11451	1	0
RFX1	4	-0.054318	1	0	*RBM34*	4	-0.11447	1	0
UBXN2A	3	-0.054315	1	0	*GNG13*	4	-0.11447	1	0
GPAM	3	-0.054206	1	0	*PIMREG*	2	-0.11443	1	0
RGS9	4	-0.054153	1	0	*LIN7C*	4	-0.11442	1	0
FAM200B	1	-0.054083	1	0	*ZNF593*	4	-0.11409	1	0
ZNF814	4	-0.053954	1	0	*VAMP5*	4	-0.11403	1	0
NBPF7	3	-0.053922	1	0	*HLA-C*	3	-0.11396	1	0
ADGRB2	4	-0.05386	1	0	*TCF12*	4	-0.11367	1	0

PRDM13	4	-0.053837	1	0	*SIX1*	3	-0.11361	1	0
MSMO1	4	-0.053795	1	0	*MTMR6*	3	-0.11355	1	0
CCDC102B	3	-0.053587	1	0	*TBX6*	3	-0.11355	1	0
DUSP12	4	-0.053519	1	0	*UBASH3A*	4	-0.11347	1	0
TCIM	4	-0.053513	1	0	*CUL5*	4	-0.11341	1	0
SORBS1	3	-0.053451	1	0	*IL23A*	2	-0.1134	1	0
UBOX5	4	-0.053399	1	0	*C1orf174*	4	-0.11336	1	0
TOR4A	4	-0.053363	1	0	*FAM214A*	3	-0.11302	1	0
ZNF213	4	-0.053352	1	0	*GFAP*	3	-0.11296	1	0
MTX3	4	-0.053332	1	0	*IDE*	4	-0.11292	1	0
C1GALT1C1	3	-0.053314	1	0	*KCNH1*	3	-0.11286	1	0
FOXE1	3	-0.053314	1	0	*WLS*	4	-0.11278	1	0
ELMOD1	3	-0.053211	1	0	*ABHD1*	4	-0.11278	1	0
KCNAB3	3	-0.053176	1	0	*COL5A2*	2	-0.11268	1	0
TMEM203	3	-0.053169	1	0	*SLC44A3*	3	-0.11266	1	0
WDR34	3	-0.053126	1	0	*MID2*	3	-0.11263	1	0
TNNI1	4	-0.053018	1	0	*BCO2*	3	-0.11262	1	0
ADD1	4	-0.052854	1	0	*CDH15*	4	-0.11261	1	0
NSMAF	2	-0.052822	1	0	*SNTA1*	4	-0.11252	1	0
TEP1	3	-0.052801	1	0	*C15orf61*	4	-0.11249	1	0
PXYLP1	3	-0.052781	1	0	*TFF3*	4	-0.1124	1	0
ITLN2	4	-0.052776	1	0	*MXRA5*	4	-0.11232	1	0
STBD1	4	-0.052706	1	0	*TUBA4A*	4	-0.11227	1	0
GRK7	4	-0.052697	1	0	*THAP10*	2	-0.11213	1	0
ATXN1	4	-0.052492	1	0	*USP19*	4	-0.11213	1	0
HAS1	4	-0.05243	1	0	*GADD45B*	4	-0.11209	1	0
TARBP1	2	-0.052348	1	0	*SFTPB*	4	-0.11203	1	0
RDM1	1	-0.052318	1	0	*DPPA4*	4	-0.112	1	0
PANX2	3	-0.052312	1	0	*RNF181*	2	-0.11196	1	0
HBG1	1	-0.052191	1	0	*TOPORS*	4	-0.11196	1	0
C10orf88	4	-0.052143	1	0	*SLC46A3*	3	-0.11187	1	0
FMN1	3	-0.052125	1	0	*POU4F3*	4	-0.11185	1	0
VWA5B2	4	-0.052115	1	0	*TAF10*	1	-0.11185	1	0
ALDOA	4	-0.051944	1	0	*AFAP1L1*	3	-0.11183	1	0
MMP17	2	-0.051892	1	0	*SRPK2*	3	-0.1118	1	0
MLEC	4	-0.051886	1	0	*GFOD2*	4	-0.11165	1	0
TP53INP2	4	-0.051881	1	0	*SELENOH*	3	-0.11155	1	0
DYNC1H1	4	-0.051877	1	0	*SYCP2*	4	-0.11153	1	0
DTD1	3	-0.051825	1	0	*SLC35A1*	4	-0.11153	1	0
FGFR2	4	-0.051824	1	0	*F3*	4	-0.11146	1	0
FOXP3	4	-0.051777	1	0	*SYNGR2*	3	-0.11144	1	0
N4BP2	4	-0.051746	1	0	*SUSD2*	4	-0.11138	1	0
MAGEA1	4	-0.051678	1	0	*OTUD7A*	3	-0.11137	1	0
RAPGEF5	4	-0.051632	1	0	*EDN1*	4	-0.11134	1	0
EID3	2	-0.051625	1	0	*C9orf139*	4	-0.11131	1	0
CUL7	4	-0.051595	1	0	*ELOVL7*	2	-0.11129	1	0
DIPK1A	4	-0.051594	1	0	*ACRV1*	4	-0.11128	1	0
USP45	4	-0.051554	1	0	*DEFB4A*	2	-0.11125	1	0
LMTK2	4	-0.051509	1	0	*TREH*	3	-0.11121	1	0
NDUFAF4	4	-0.051502	1	0	*KIAA1549L*	4	-0.11119	1	0
SNX18	3	-0.051343	1	0	*PLA2G15*	4	-0.11116	1	0
GABARAPL1	3	-0.051207	1	0	*GDF9*	4	-0.11102	1	0
SLC10A6	4	-0.051207	1	0	*DCAF12L1*	4	-0.11099	1	0
ITGAL	3	-0.051186	1	0	*JAM2*	3	-0.11093	1	0
RPL12	3	-0.051099	1	0	*SPINK4*	2	-0.11089	1	0
SLC36A1	4	-0.05103	1	0	*ZDHHC2*	4	-0.11072	1	0
COMMD7	4	-0.050996	1	0	*MAN1B1*	2	-0.11068	1	0
ACSF3	4	-0.050992	1	0	*CYB561A3*	4	-0.11058	1	0

AFG1L	4	-0.050938	1	0
SRSF4	4	-0.050825	1	0
CCDC85C	3	-0.050786	1	0
SOCS5	3	-0.050629	1	0
HOXB5	4	-0.050619	1	0
JCAD	4	-0.050592	1	0
RXFP1	4	-0.050553	1	0
RNPEP	3	-0.050543	1	0
CCDC70	4	-0.050403	1	0
SPRYD7	4	-0.050402	1	0
ZNF614	4	-0.050362	1	0
MRPL28	3	-0.050358	1	0
RFX5	3	-0.050341	1	0
OR10A2	3	-0.050293	1	0
SNTB2	4	-0.050236	1	0
MTUS2	4	-0.050138	1	0
CASQ1	2	-0.050112	1	0
IFIH1	4	-0.050095	1	0
AXIN1	3	-0.050088	1	0
CPEB3	4	-0.050085	1	0
MAP1LC3B	3	-0.050046	1	0
SMC1B	4	-0.050006	1	0
POU6F2	2	-0.049827	1	0
SEPTIN8	4	-0.049693	1	0
LTN1	4	-0.049646	1	0
CASP5	4	-0.049574	1	0
SLC17A1	4	-0.049483	1	0
CTRB2	1	-0.049389	1	0
POU2F3	4	-0.049366	1	0
LZIC	3	-0.049338	1	0
PPM1M	4	-0.04928	1	0
GCFC2	3	-0.04921	1	0
APOBEC2	3	-0.049195	1	0
PDZD4	4	-0.049161	1	0
TRPM5	4	-0.049119	1	0
UPK1B	4	-0.049073	1	0
CAPN2	4	-0.049071	1	0
KRTAP3-1	4	-0.048978	1	0
PHEX	4	-0.048953	1	0
DENND2D	4	-0.048951	1	0
WFIKKN1	4	-0.04882	1	0
GVQW2	1	-0.048812	1	0
KIRREL2	4	-0.048812	1	0
GNG4	4	-0.048657	1	0
IL34	4	-0.048487	1	0
SLF2	3	-0.048248	1	0
CCDC65	4	-0.048143	1	0
SNAPC5	3	-0.048114	1	0
GLG1	2	-0.0481	1	0
CCDC159	4	-0.04803	1	0
SLC9A6	3	-0.048027	1	0
COX10	4	-0.048022	1	0
C19orf47	3	-0.048008	1	0
IQCC	3	-0.047998	1	0
NCAM2	4	-0.047953	1	0
ZNF107	3	-0.047948	1	0
BMP4	4	-0.047943	1	0
ILVBL	3	-0.047925	1	0

LTB	4	-0.11051	1	0
ADGRL1	4	-0.1105	1	0
ZC3H12D	3	-0.11039	1	0
CSF2	4	-0.11026	1	0
NGB	4	-0.11022	1	0
ZNF354A	3	-0.11017	1	0
FAM167A	4	-0.11016	1	0
SYT8	3	-0.11016	1	0
CCDC160	4	-0.11013	1	0
TSPO2	4	-0.1101	1	0
GAS8	4	-0.11005	1	0
WTIP	2	-0.10996	1	0
RABL6	4	-0.10989	1	0
GMFG	3	-0.10988	1	0
KIRREL2	4	-0.10983	1	0
NUAK2	4	-0.10978	1	0
RPS27L	4	-0.10974	1	0
PDLIM4	1	-0.10971	1	0
C11orf71	4	-0.10945	1	0
SEMA4A	2	-0.10944	1	0
NAGLU	4	-0.10943	1	0
MAF1	3	-0.10939	1	0
HEATR3	4	-0.10934	1	0
ING3	2	-0.1092	1	0
MINDY1	4	-0.10919	1	0
DIO2	4	-0.1091	1	0
SLC12A4	4	-0.10908	1	0
STRIP2	2	-0.10902	1	0
ERI3	4	-0.10894	1	0
NFXL1	4	-0.10894	1	0
RNF144B	4	-0.10892	1	0
PLAG1	4	-0.10888	1	0
CD300A	4	-0.10887	1	0
MKRN2	4	-0.10887	1	0
TMEM115	4	-0.10884	1	0
B3GNT2	3	-0.10873	1	0
LOC158434	3	-0.1087	1	0
LMBRD2	4	-0.10862	1	0
ENTPD4	4	-0.10862	1	0
FCER1A	4	-0.10858	1	0
MED21	2	-0.10857	1	0
SLC25A22	2	-0.10838	1	0
TNFRSF10B	4	-0.10833	1	0
ZNF596	3	-0.10828	1	0
CD177	4	-0.10822	1	0
FCGR1A	1	-0.10809	1	0
RNF215	4	-0.10808	1	0
PIM2	4	-0.10803	1	0
BAZ1B	4	-0.108	1	0
TMEM33	4	-0.108	1	0
QSER1	4	-0.10799	1	0
TMED5	1	-0.10796	1	0
ZNF672	4	-0.10796	1	0
FOXQ1	4	-0.1078	1	0
BLOC1S6	4	-0.10779	1	0
ZNF454	4	-0.10778	1	0
STAR	4	-0.1077	1	0
PSCA	4	-0.10769	1	0

C5	4	-0.047912	1	0	*OSTM1*	4	-0.10766	1	0
DOCK5	4	-0.047799	1	0	*BLCAP*	4	-0.1076	1	0
ZBED5	4	-0.047725	1	0	*G6PC3*	4	-0.10756	1	0
ZNF175	4	-0.047692	1	0	*HES7*	3	-0.10754	1	0
UNC13B	4	-0.047619	1	0	*PRPF38B*	4	-0.10752	1	0
LZTS1	3	-0.047601	1	0	*SUPT20HL2*	3	-0.10752	1	0
TMEM40	4	-0.047572	1	0	*CDK5R1*	4	-0.10747	1	0
PICK1	4	-0.047477	1	0	*SEMA7A*	4	-0.10738	1	0
FAM204A	4	-0.04747	1	0	*FRG2*	1	-0.10734	1	0
STAU2	4	-0.047441	1	0	*GPR179*	4	-0.10732	1	0
DNER	4	-0.047422	1	0	*BAIAP2L1*	4	-0.10731	1	0
FBXO44	4	-0.047406	1	0	*ACAA1*	4	-0.10728	1	0
CPED1	4	-0.047402	1	0	*CLDN16*	4	-0.1072	1	0
SLC12A7	4	-0.047374	1	0	*CCDC103*	3	-0.1072	1	0
KLK9	3	-0.0473	1	0	*TNPO2*	4	-0.10717	1	0
SSB	2	-0.04729	1	0	*NR1I3*	3	-0.10713	1	0
PPP1R12A	4	-0.047234	1	0	*UBOX5*	4	-0.10697	1	0
PTER	4	-0.047225	1	0	*KBTBD8*	3	-0.10681	1	0
RORA	3	-0.047195	1	0	*TP53INP2*	4	-0.10676	1	0
ZNF674	4	-0.04718	1	0	*MYO15A*	4	-0.10675	1	0
ZACN	4	-0.047157	1	0	*DAPP1*	4	-0.10661	1	0
NID2	4	-0.047149	1	0	*B3GNT4*	4	-0.1065	1	0
EIF5A2	4	-0.047131	1	0	*ENPP1*	4	-0.10647	1	0
RNF122	3	-0.047061	1	0	*RPRD1A*	4	-0.10642	1	0
BOLA1	4	-0.047051	1	0	*SIAE*	3	-0.1064	1	0
COL9A3	4	-0.047028	1	0	*TLK2*	2	-0.10629	1	0
ZSCAN26	3	-0.047001	1	0	*DNAJC22*	4	-0.1062	1	0
PACRG	1	-0.046961	1	0	*SPPL3*	4	-0.10619	1	0
SP9	4	-0.046953	1	0	*ZNF662*	2	-0.10617	1	0
CEP68	4	-0.046918	1	0	*FOCAD*	4	-0.1061	1	0
RD3	3	-0.046891	1	0	*PIGS*	4	-0.10604	1	0
EGR4	2	-0.046888	1	0	*STK31*	2	-0.10603	1	0
PARP6	4	-0.046821	1	0	*SOCS5*	3	-0.10599	1	0
RSPO3	4	-0.046762	1	0	*ZNF77*	4	-0.10579	1	0
HSD3B7	4	-0.04675	1	0	*TMEM8B*	2	-0.10572	1	0
UBAP2	2	-0.046648	1	0	*NCKIPSD*	2	-0.1057	1	0
ERG	4	-0.046591	1	0	*PKNOX2*	4	-0.10568	1	0
BECN1	4	-0.046579	1	0	*PUF60*	4	-0.10567	1	0
GNL2	3	-0.046534	1	0	*ARHGAP18*	4	-0.10553	1	0
NSUN7	4	-0.046507	1	0	*RAB17*	3	-0.10551	1	0
NANP	3	-0.046473	1	0	*UGGT2*	3	-0.1055	1	0
RPRD2	4	-0.046459	1	0	*MAGEA10*	4	-0.10543	1	0
USH1G	4	-0.046437	1	0	*PDE2A*	4	-0.10541	1	0
HMGCR	4	-0.046434	1	0	*MAP7D1*	4	-0.10536	1	0
HLX	1	-0.046302	1	0	*CCDC191*	4	-0.10532	1	0
KCND1	3	-0.046267	1	0	*SOCS1*	4	-0.1053	1	0
UCHL1	4	-0.046265	1	0	*UBA5*	3	-0.10519	1	0
PREPL	4	-0.046189	1	0	*DACH2*	4	-0.10512	1	0
DPCD	4	-0.046107	1	0	*PCYT1B*	3	-0.10509	1	0
PAFAH1B2	3	-0.046086	1	0	*PIGF*	3	-0.10497	1	0
ANXA4	4	-0.04606	1	0	*TICAM1*	4	-0.10495	1	0
FAM162A	4	-0.046055	1	0	*ELOA2*	3	-0.10493	1	0
LRRC69	2	-0.046011	1	0	*NR2C2*	4	-0.10488	1	0
ADSS1	3	-0.046008	1	0	*XKR9*	4	-0.10487	1	0
FSCN2	3	-0.046008	1	0	*LGALS3*	4	-0.10477	1	0
BEAN1	4	-0.045933	1	0	*OR1M1*	4	-0.10474	1	0
SLC8A1	4	-0.045888	1	0	*TBX1*	4	-0.10467	1	0
HADHB	3	-0.045819	1	0	*ODC1*	4	-0.10467	1	0

C7orf57	3	-0.045735	1	0	*CTSC*	1	-0.10462	1	0
DUOX2	4	-0.045729	1	0	*CERS1*	4	-0.10461	1	0
S100A13	3	-0.045714	1	0	*SLITRK6*	4	-0.1046	1	0
TEPP	3	-0.04565	1	0	*NRDE2*	4	-0.10458	1	0
WFDC6	2	-0.045605	1	0	*CLEC4D*	4	-0.10458	1	0
FRMPD2	4	-0.045587	1	0	*GRIK2*	4	-0.10443	1	0
RNF213	4	-0.045553	1	0	*SPATA17*	3	-0.1044	1	0
B3GALNT2	4	-0.045502	1	0	*C9orf64*	2	-0.10438	1	0
DOCK10	3	-0.045479	1	0	*FOXO3*	3	-0.10437	1	0
CEP162	3	-0.045465	1	0	*RFX6*	3	-0.10437	1	0
FAM13C	4	-0.045457	1	0	*DOCK6*	4	-0.10437	1	0
SLC35B3	3	-0.045413	1	0	*SHANK1*	3	-0.1043	1	0
HES6	4	-0.04533	1	0	*PIM3*	4	-0.10425	1	0
SGMS1	4	-0.045295	1	0	*SLC22A14*	4	-0.10417	1	0
NLRP2	4	-0.045227	1	0	*CABLES1*	3	-0.10412	1	0
DCTN3	4	-0.04521	1	0	*RCSD1*	4	-0.10405	1	0
FYB1	2	-0.045208	1	0	*HSD3B7*	4	-0.10405	1	0
C4orf36	4	-0.045128	1	0	*CCDC7*	4	-0.10404	1	0
EPCAM	4	-0.04507	1	0	*LOC728392*	4	-0.10392	1	0
IFNE	4	-0.045046	1	0	*RALGAPA1*	4	-0.10384	1	0
VNN1	4	-0.045041	1	0	*FBXO7*	3	-0.10383	1	0
FBXL16	4	-0.044878	1	0	*MTRF1*	4	-0.1037	1	0
FAM161A	4	-0.044837	1	0	*MDM4*	2	-0.10342	1	0
P4HA2	4	-0.044804	1	0	*PLCD4*	3	-0.1034	1	0
RPS6KB2	4	-0.044769	1	0	*AZU1*	3	-0.10337	1	0
H2BC1	3	-0.044693	1	0	*ITPA*	4	-0.10336	1	0
WDR20	4	-0.044637	1	0	*MUSK*	2	-0.10327	1	0
TTC39C	4	-0.044496	1	0	*ATP8B2*	2	-0.10321	1	0
PLAC8L1	4	-0.044489	1	0	*ACTA1*	1	-0.10321	1	0
TSPYL4	4	-0.044406	1	0	*SCEL*	2	-0.10321	1	0
NGEF	4	-0.044384	1	0	*KLHDC4*	3	-0.10307	1	0
NEK2	3	-0.044321	1	0	*KIAA0895*	4	-0.10305	1	0
FAM13A	3	-0.044303	1	0	*SCMH1*	4	-0.10305	1	0
FAM122A	4	-0.044282	1	0	*GPR34*	4	-0.10302	1	0
SLC26A8	4	-0.044255	1	0	*TLCD3B*	3	-0.10293	1	0
GRINA	4	-0.044242	1	0	*MAPK8IP1*	3	-0.10293	1	0
PARPBP	4	-0.044195	1	0	*TTC30B*	4	-0.10293	1	0
NEU4	4	-0.044093	1	0	*RAD23A*	4	-0.10291	1	0
ZSCAN12	4	-0.044049	1	0	*DENND4C*	3	-0.10289	1	0
NDUFA3	4	-0.043911	1	0	*WIF1*	4	-0.1028	1	0
SEMA4B	4	-0.043779	1	0	*CDC26*	2	-0.10277	1	0
C16orf58	4	-0.043451	1	0	*FIGLA*	3	-0.10277	1	0
HNRNPR	4	-0.04344	1	0	*GRK7*	4	-0.10264	1	0
MNT	2	-0.043376	1	0	*ROPN1B*	3	-0.1026	1	0
NKX2-8	4	-0.04334	1	0	*ARSJ*	3	-0.10256	1	0
GPR87	4	-0.043264	1	0	*C6orf223*	2	-0.10251	1	0
SSNA1	3	-0.043259	1	0	*H2BW1*	4	-0.10239	1	0
TPRN	3	-0.04325	1	0	*SIX4*	1	-0.10237	1	0
TTLL1	3	-0.043161	1	0	*EI24*	4	-0.10227	1	0
MOV10	3	-0.043141	1	0	*SGMS2*	4	-0.10227	1	0
DPYSL2	2	-0.043125	1	0	*GNPAT*	3	-0.10226	1	0
CD177	4	-0.04309	1	0	*CD160*	4	-0.10214	1	0
OLFML1	4	-0.042961	1	0	*DYNC2LI1*	4	-0.10213	1	0
MRPL13	4	-0.042958	1	0	*CAP2*	3	-0.10212	1	0
FABP1	4	-0.042936	1	0	*ZNF384*	4	-0.10211	1	0
SLC28A3	4	-0.042804	1	0	*TMEM123*	3	-0.1021	1	0
EPN2	4	-0.042754	1	0	*KIRREL1*	4	-0.10206	1	0
PAPSS1	3	-0.042699	1	0	*GINM1*	3	-0.10204	1	0

PRTFDC1	4	-0.042571	1	0	*BTBD19*	4	-0.10204	1	0
RWDD2A	4	-0.042397	1	0	*NTSR1*	4	-0.10196	1	0
ADGRE2	3	-0.042383	1	0	*ANKRD33*	3	-0.1019	1	0
MAP3K1	4	-0.042282	1	0	*FETUB*	3	-0.1019	1	0
TTL	4	-0.042245	1	0	*TSEN54*	4	-0.10187	1	0
LHX2	2	-0.042241	1	0	*HGD*	3	-0.10185	1	0
RNF148	2	-0.042225	1	0	*CXCL13*	4	-0.10183	1	0
OR9A4	4	-0.042202	1	0	*TMEM80*	2	-0.10176	1	0
HORMAD1	4	-0.042076	1	0	*YIPF6*	4	-0.10175	1	0
LMOD1	4	-0.041918	1	0	*DNAH9*	4	-0.10174	1	0
BOC	2	-0.041852	1	0	*REN*	4	-0.10168	1	0
NCF1	2	-0.041673	1	0	*MAPK8IP3*	3	-0.10161	1	0
TRIM54	4	-0.041536	1	0	*VPS18*	4	-0.10155	1	0
TCEAL4	4	-0.0415	1	0	*SCGB3A2*	4	-0.10153	1	0
ETV3	4	-0.041341	1	0	*FAM186A*	4	-0.10149	1	0
PRSS12	3	-0.041334	1	0	*IFT140*	4	-0.10136	1	0
ZNF852	4	-0.041309	1	0	*BORCS5*	4	-0.1013	1	0
SYAP1	4	-0.041195	1	0	*HLA-DQA1*	3	-0.10128	1	0
LNX1	4	-0.041052	1	0	*PIH1D1*	3	-0.10122	1	0
C4orf48	2	-0.041051	1	0	*GOLGA1*	3	-0.10121	1	0
KLHDC2	4	-0.04104	1	0	*SMPDL3B*	4	-0.10112	1	0
ARFIP1	3	-0.041006	1	0	*ETNK2*	4	-0.10095	1	0
ZNF174	4	-0.040981	1	0	*ZNF491*	4	-0.10088	1	0
ISM1	4	-0.040965	1	0	*LRRC75A*	4	-0.10086	1	0
MYSM1	4	-0.040919	1	0	*ZNF746*	4	-0.1008	1	0
MGST1	4	-0.040914	1	0	*AFF1*	4	-0.1008	1	0
ZNF397	3	-0.040762	1	0	*ATXN2L*	2	-0.10079	1	0
ANXA13	4	-0.040678	1	0	*ANO2*	4	-0.10079	1	0
VEPH1	2	-0.04063	1	0	*CASD1*	2	-0.10072	1	0
DDX25	3	-0.040556	1	0	*NUP54*	4	-0.10063	1	0
SPRR3	4	-0.040421	1	0	*FAM229A*	4	-0.10057	1	0
BRS3	4	-0.040382	1	0	*IKZF5*	4	-0.10047	1	0
HOXA13	4	-0.040357	1	0	*C9orf40*	4	-0.10045	1	0
XPO5	4	-0.040313	1	0	*CHKA*	4	-0.10032	1	0
ARHGDIG	4	-0.040262	1	0	*AFF4*	4	-0.10031	1	0
RNASE10	4	-0.040251	1	0	*HOXC13*	1	-0.1003	1	0
RHOV	4	-0.040184	1	0	*ARHGAP6*	4	-0.10028	1	0
KCTD16	4	-0.040162	1	0	*PHLDA3*	4	-0.10026	1	0
RAB34	8	-0.040138	1	0	*PLA2G4E*	4	-0.1001	1	0
TCTE1	4	-0.040111	1	0	*TMEM256*	2	-0.10008	1	0
CORO6	4	-0.040062	1	0	*NKX3-1*	2	-0.099995	1	0
EVPL	2	-0.040062	1	0	*NOS1*	4	-0.09997	1	0
CACNA1D	3	-0.040059	1	0	*FBXL3*	4	-0.099959	1	0
TMEM60	3	-0.04004	1	0	*RANBP1*	3	-0.099862	1	0
PPP1R16A	2	-0.039981	1	0	*DDO*	4	-0.099854	1	0
FLG	4	-0.039941	1	0	*CNKSR2*	4	-0.099808	1	0
DEPDC5	4	-0.039897	1	0	*CREG1*	2	-0.09979	1	0
SPOCK3	4	-0.039897	1	0	*SH3BP2*	4	-0.099773	1	0
CLIC3	4	-0.039863	1	0	*TRUB2*	1	-0.099773	1	0
MPP3	4	-0.03985	1	0	*ATP2A2*	4	-0.099765	1	0
ACP6	4	-0.039775	1	0	*IFI16*	4	-0.099736	1	0
PODXL	4	-0.03967	1	0	*SERPINA6*	3	-0.099714	1	0
SAMD13	3	-0.039656	1	0	*PTK7*	4	-0.09971	1	0
ALMS1	3	-0.039557	1	0	*HECTD4*	4	-0.099695	1	0
CRBN	4	-0.039493	1	0	*PABPC3*	4	-0.099652	1	0
NANS	4	-0.039416	1	0	*ITM2C*	4	-0.099568	1	0
CD84	4	-0.039387	1	0	*UBR2*	4	-0.099498	1	0
MYH7B	3	-0.039387	1	0	*TBKBP1*	4	-0.099325	1	0

IFI6	4	-0.039382	1	0	*PTHLH*	3	-0.099292	1	0
CPQ	4	-0.039377	1	0	*GLB1*	3	-0.099289	1	0
QPCT	4	-0.039336	1	0	*ID4*	4	-0.099264	1	0
NEK1	4	-0.039324	1	0	*ALG10B*	2	-0.099219	1	0
RHEX	4	-0.039299	1	0	*SCGB2A1*	4	-0.09913	1	0
MIER3	4	-0.039245	1	0	*CDC42EP1*	3	-0.099124	1	0
FOPNL	4	-0.039141	1	0	*EGR1*	4	-0.099012	1	0
SH2D6	3	-0.039044	1	0	*COL20A1*	2	-0.098998	1	0
FOCAD	4	-0.039038	1	0	*LDHB*	3	-0.098978	1	0
EML5	4	-0.038999	1	0	*PRR14*	4	-0.098975	1	0
VAX2	4	-0.038956	1	0	*MAP3K7CL*	3	-0.098946	1	0
APOL1	4	-0.038934	1	0	*PML*	4	-0.09893	1	0
NRP1	4	-0.038929	1	0	*SESN3*	3	-0.098885	1	0
NSG2	4	-0.038853	1	0	*MDGA1*	4	-0.098879	1	0
SLC27A2	4	-0.038837	1	0	*ZC4H2*	3	-0.098867	1	0
CELSR2	3	-0.038834	1	0	*OR10H1*	3	-0.098849	1	0
ATP5MG	2	-0.038788	1	0	*CBX1*	3	-0.098779	1	0
C21orf58	4	-0.03871	1	0	*HNRNPF*	4	-0.098758	1	0
CADM2	3	-0.038665	1	0	*ZUP1*	3	-0.098705	1	0
STX10	3	-0.038663	1	0	*PPP2R1B*	3	-0.098699	1	0
CNGA3	2	-0.038575	1	0	*PDIK1L*	4	-0.098688	1	0
S100A6	4	-0.038545	1	0	*COL9A2*	4	-0.098619	1	0
APPBP2	4	-0.038483	1	0	*ZNF256*	4	-0.098555	1	0
DLG4	3	-0.038342	1	0	*SH3YL1*	3	-0.098487	1	0
RNF114	3	-0.038096	1	0	*TRIOBP*	4	-0.098396	1	0
MTG1	4	-0.037974	1	0	*L1TD1*	4	-0.098233	1	0
CLUAP1	3	-0.037969	1	0	*MITD1*	4	-0.098164	1	0
MED13L	4	-0.037948	1	0	*ATP2A1*	4	-0.09815	1	0
NFKBIA	4	-0.037946	1	0	*RTBDN*	3	-0.098143	1	0
ADGRB3	3	-0.037853	1	0	*UBQLN2*	3	-0.097974	1	0
SDS	3	-0.03783	1	0	*NOXO1*	4	-0.097966	1	0
SIRPB1	4	-0.037829	1	0	*CDS1*	4	-0.097961	1	0
CXorf40A	2	-0.037713	1	0	*H2AC20*	3	-0.097936	1	0
ZFP42	4	-0.03765	1	0	*ZNF648*	2	-0.097901	1	0
LETM2	2	-0.037544	1	0	*INSL6*	4	-0.097697	1	0
TRIM37	4	-0.037511	1	0	*SCAPER*	4	-0.097573	1	0
RPP30	4	-0.0375	1	0	*TCAP*	3	-0.097545	1	0
C1QTNF1	4	-0.037481	1	0	*CLDND2*	4	-0.097502	1	0
SYPL2	2	-0.037408	1	0	*ERF*	4	-0.097386	1	0
ERBIN	3	-0.037249	1	0	*SLC4A4*	4	-0.09734	1	0
C19orf18	3	-0.037164	1	0	*FAM174A*	4	-0.097336	1	0
SUGCT	4	-0.03715	1	0	*GRID1*	3	-0.097316	1	0
PCDHGA10	3	-0.037141	1	0	*CASKIN2*	4	-0.097307	1	0
FAM50B	4	-0.037103	1	0	*OSBP*	3	-0.097286	1	0
ABRACL	4	-0.037087	1	0	*PPM1K*	4	-0.097245	1	0
RAB11FIP5	3	-0.036867	1	0	*PLEKHM2*	3	-0.097231	1	0
KCNK18	4	-0.03674	1	0	*MOCS1*	4	-0.096985	1	0
UNC79	4	-0.03668	1	0	*C10orf53*	4	-0.096973	1	0
GSTM1	3	-0.03664	1	0	*SRPK1*	4	-0.09696	1	0
SLC44A4	4	-0.036511	1	0	*ZNF121*	3	-0.096891	1	0
KNDC1	4	-0.036503	1	0	*DERL2*	3	-0.096851	1	0
FANCD2	4	-0.036498	1	0	*CCDC32*	3	-0.09681	1	0
MYB	4	-0.036463	1	0	*PI4KA*	3	-0.096797	1	0
THADA	4	-0.036453	1	0	*IDUA*	4	-0.096681	1	0
TIMP4	4	-0.036445	1	0	*RAP1A*	4	-0.096671	1	0
METRNL	4	-0.036328	1	0	*SLC26A1*	4	-0.096573	1	0
CALCB	4	-0.03632	1	0	*RWDD2A*	4	-0.096502	1	0
SLC10A7	4	-0.036268	1	0	*TLX2*	3	-0.096381	1	0

RPL35A	3	-0.036019	1	0	*C17orf100*	3	-0.096354	1	0
TIGD6	3	-0.035908	1	0	*TRAM2*	3	-0.096223	1	0
ATP11A	4	-0.0359	1	0	*PLA2G6*	4	-0.096213	1	0
RARS2	4	-0.035863	1	0	*TMEM175*	4	-0.096209	1	0
AGBL3	4	-0.035808	1	0	*AMER2*	4	-0.09615	1	0
SULT1A2	4	-0.035797	1	0	*DTD1*	3	-0.096045	1	0
KDM7A	4	-0.035708	1	0	*ANKRD16*	3	-0.095937	1	0
MEIOB	4	-0.035689	1	0	*APIP*	2	-0.095911	1	0
KCTD9	3	-0.035576	1	0	*GRIA4*	2	-0.095836	1	0
ZNF311	4	-0.035424	1	0	*METTL25*	3	-0.095755	1	0
LASP1	4	-0.035406	1	0	*C14orf93*	4	-0.095749	1	0
MRPS22	4	-0.035346	1	0	*STRN*	3	-0.095697	1	0
SLC46A2	4	-0.035249	1	0	*EQTN*	4	-0.095664	1	0
RASSF4	4	-0.035194	1	0	*DERL3*	4	-0.095624	1	0
ZNF337	3	-0.035016	1	0	*R3HDM1*	4	-0.095499	1	0
GALNT6	4	-0.034985	1	0	*SRD5A2*	4	-0.09547	1	0
MACROD2	4	-0.034963	1	0	*OR52I1*	2	-0.095459	1	0
ZNF581	4	-0.034867	1	0	*ZNF579*	4	-0.095352	1	0
LTA4H	4	-0.03476	1	0	*CKB*	4	-0.095287	1	0
FKBP7	3	-0.034622	1	0	*NCEH1*	4	-0.095286	1	0
LAPTM5	3	-0.034594	1	0	*PFN4*	4	-0.09517	1	0
RTN1	2	-0.034583	1	0	*TSHZ1*	4	-0.095001	1	0
IGSF6	2	-0.034468	1	0	*MGRN1*	4	-0.094927	1	0
GBP3	4	-0.034464	1	0	*LGALS9B*	1	-0.094706	1	0
SMUG1	4	-0.034455	1	0	*FGF13*	3	-0.094569	1	0
ELK3	4	-0.034399	1	0	*CHN1*	4	-0.094561	1	0
TOP1	4	-0.034395	1	0	*LNP1*	4	-0.094453	1	0
SLC25A37	4	-0.034385	1	0	*ZNF610*	4	-0.09442	1	0
IGSF11	4	-0.034359	1	0	*SYDE1*	4	-0.094402	1	0
INPP5F	4	-0.03431	1	0	*RBMS2*	1	-0.094356	1	0
MICU1	4	-0.034306	1	0	*LOC10192884*	4	-0.094343	1	0
HTR1E	4	-0.034289	1	0	*CMTM1*	3	-0.094323	1	0
SPAM1	4	-0.034146	1	0	*PUM3*	4	-0.094227	1	0
PSMB8	4	-0.034139	1	0	*RNF180*	4	-0.094221	1	0
SCML1	4	-0.034059	1	0	*FOXR1*	4	-0.094219	1	0
MLPH	4	-0.034012	1	0	*NXPH4*	3	-0.094211	1	0
PINK1	4	-0.033903	1	0	*ADGRE5*	3	-0.094205	1	0
ZNF404	3	-0.03385	1	0	*TRPC1*	3	-0.094149	1	0
ATG16L1	4	-0.033841	1	0	*TRABD2B*	3	-0.094144	1	0
COA5	3	-0.033806	1	0	*CALY*	3	-0.094112	1	0
ATP11C	4	-0.033804	1	0	*PNPLA2*	3	-0.093981	1	0
MYLK4	4	-0.0338	1	0	*SPAM1*	4	-0.09397	1	0
NSDHL	4	-0.033788	1	0	*PIEZO1*	4	-0.093933	1	0
TTC7A	4	-0.033786	1	0	*CHAMP1*	4	-0.093763	1	0
TNFAIP2	3	-0.033746	1	0	*ZBTB49*	4	-0.093753	1	0
FCSK	4	-0.033694	1	0	*GCC2*	4	-0.093611	1	0
IFT46	3	-0.033664	1	0	*JAG1*	4	-0.093587	1	0
TMOD2	3	-0.03366	1	0	*WASF2*	3	-0.093523	1	0
ABCA2	1	-0.033573	1	0	*TDRD6*	4	-0.093457	1	0
SEPTIN11	4	-0.033563	1	0	*PLEKHG1*	3	-0.093438	1	0
CCDC186	4	-0.033515	1	0	*GLT8D1*	3	-0.093421	1	0
KLC3	2	-0.033507	1	0	*FAM71F1*	4	-0.093404	1	0
WASHC3	3	-0.033503	1	0	*DCDC2B*	1	-0.093375	1	0
IDE	4	-0.033451	1	0	*FOXJ3*	4	-0.093318	1	0
MIEF2	4	-0.033327	1	0	*IRX6*	3	-0.093273	1	0
SBNO1	4	-0.033287	1	0	*EXTL1*	4	-0.093265	1	0
ESF1	3	-0.033233	1	0	*CDC25C*	4	-0.09322	1	0
GPR35	4	-0.03313	1	0	*CATSPERE*	4	-0.093108	1	0

TMEM101	3	-0.033127	1	0	*PBX2*	4	-0.093097	1	0
RHOH	3	-0.033074	1	0	*SGSM1*	4	-0.093069	1	0
MLH3	4	-0.033058	1	0	*RAB3D*	4	-0.093018	1	0
PREX2	3	-0.033057	1	0	*GRM3*	4	-0.092867	1	0
PELI1	3	-0.033006	1	0	*CAPSL*	4	-0.092819	1	0
INA	4	-0.032913	1	0	*PDRG1*	3	-0.092756	1	0
PPARG	4	-0.032909	1	0	*KCNJ16*	4	-0.092706	1	0
TMEM81	2	-0.032714	1	0	*CD8A*	4	-0.092683	1	0
ZBED9	4	-0.032692	1	0	*TXNDC16*	4	-0.092547	1	0
C12orf57	4	-0.032638	1	0	*EFCAB13*	3	-0.092388	1	0
PMEPA1	4	-0.032629	1	0	*ENC1*	4	-0.092351	1	0
ZAR1L	4	-0.032623	1	0	*GPHN*	3	-0.09227	1	0
ERO1B	4	-0.032502	1	0	*NECAB3*	4	-0.092106	1	0
EPO	4	-0.032444	1	0	*NANOS2*	4	-0.092084	1	0
ERMARD	4	-0.032394	1	0	*ELF3*	4	-0.091951	1	0
HERC6	4	-0.032387	1	0	*CSNK1D*	3	-0.091911	1	0
ZNF195	3	-0.03233	1	0	*POMGNT2*	3	-0.091756	1	0
PCDHGB7	4	-0.032296	1	0	*MCMDC2*	4	-0.091754	1	0
TMCO3	4	-0.032291	1	0	*MSL2*	4	-0.091745	1	0
CARMIL2	2	-0.032274	1	0	*NID2*	4	-0.091708	1	0
DDX19A	4	-0.032267	1	0	*KLK9*	3	-0.091669	1	0
AMER1	2	-0.032158	1	0	*HOXD1*	4	-0.091662	1	0
PQBP1	4	-0.03215	1	0	*RXYLT1*	4	-0.091619	1	0
KDM5A	3	-0.032048	1	0	*UGT1A1*	4	-0.091617	1	0
VPS37B	3	-0.032018	1	0	*SUCLA2*	4	-0.091551	1	0
AZIN2	4	-0.032001	1	0	*METTL4*	4	-0.091546	1	0
SRBD1	4	-0.031976	1	0	*CLIP3*	4	-0.0915	1	0
CT62	3	-0.031965	1	0	*KDM4C*	4	-0.091352	1	0
PLEKHA2	4	-0.031884	1	0	*CETN2*	3	-0.091294	1	0
BEX4	4	-0.031875	1	0	*ERICH6*	4	-0.091249	1	0
H4C7	4	-0.031833	1	0	*PTRHD1*	3	-0.091246	1	0
SYNE3	4	-0.031741	1	0	*DNAJB4*	4	-0.091046	1	0
ZMYND12	4	-0.031741	1	0	*CD19*	4	-0.091026	1	0
CASKIN2	4	-0.03168	1	0	*C5orf49*	3	-0.091024	1	0
PRELID3A	4	-0.031479	1	0	*TRIM26*	3	-0.091006	1	0
PRRG4	4	-0.031429	1	0	*SYNPO2L*	3	-0.09098	1	0
TNFSF11	4	-0.031397	1	0	*CDK20*	3	-0.090977	1	0
GMNN	3	-0.031387	1	0	*ZSCAN25*	4	-0.0909	1	0
KLK5	4	-0.031313	1	0	*ZNF726*	4	-0.090897	1	0
GPATCH2	4	-0.031268	1	0	*CDC42EP2*	4	-0.090887	1	0
AMER2	4	-0.031221	1	0	*CDKN1B*	4	-0.090849	1	0
TMED9	3	-0.031128	1	0	*UBE2A*	2	-0.090821	1	0
CCDC88C	3	-0.031028	1	0	*ABCA3*	3	-0.090775	1	0
KIAA1958	4	-0.030967	1	0	*LRRC28*	4	-0.090763	1	0
BTG1	4	-0.030963	1	0	*CARM1*	4	-0.090732	1	0
SNRK	4	-0.030952	1	0	*SCGB1D2*	3	-0.090707	1	0
STAMBPL1	1	-0.030911	1	0	*TJP3*	3	-0.090634	1	0
MMP20	4	-0.030799	1	0	*PBX4*	3	-0.090631	1	0
TRIL	4	-0.030721	1	0	*H4C7*	4	-0.090605	1	0
NANOS1	4	-0.030685	1	0	*ASB1*	4	-0.090601	1	0
TBC1D9	4	-0.030671	1	0	*ANGPTL6*	4	-0.090575	1	0
GRAMD1B	4	-0.030647	1	0	*CEP83*	4	-0.090574	1	0
CCL24	4	-0.030642	1	0	*FBXL22*	4	-0.090531	1	0
GOLGA3	4	-0.030617	1	0	*PDE4DIP*	2	-0.090446	1	0
NEFL	2	-0.030535	1	0	*CPSF4L*	4	-0.09039	1	0
C3orf79	4	-0.030533	1	0	*HNRNPK*	4	-0.09038	1	0
KLLN	2	-0.030517	1	0	*IP6K3*	4	-0.090377	1	0
C15orf40	4	-0.03041	1	0	*GCNT1*	4	-0.090372	1	0

NCBP1	4	-0.030254	1	0	*GALNT7*	4	-0.090346	1	0
TDRD1	4	-0.03024	1	0	*ACOX2*	4	-0.09033	1	0
BTBD2	4	-0.030196	1	0	*MBNL1*	3	-0.090196	1	0
PCDHB13	4	-0.030118	1	0	*PATE2*	4	-0.090129	1	0
TMEM164	2	-0.030103	1	0	*HSD3B2*	4	-0.090111	1	0
MYO1A	3	-0.030096	1	0	*TRIP4*	4	-0.090049	1	0
CFAP61	3	-0.030019	1	0	*C2CD4C*	3	-0.089998	1	0
H4-16	4	-0.029939	1	0	*ISM1*	4	-0.089934	1	0
DTX3	3	-0.029918	1	0	*BTN2A1*	4	-0.089906	1	0
CCNQ	4	-0.029871	1	0	*LAMP1*	4	-0.08985	1	0
GPR39	4	-0.029809	1	0	*GLRX*	4	-0.089826	1	0
MARCKSL1	4	-0.029797	1	0	*ADCY6*	3	-0.089773	1	0
VTA1	3	-0.029796	1	0	*ANKEF1*	4	-0.089725	1	0
JAG1	4	-0.029686	1	0	*BCL2L12*	4	-0.089672	1	0
ZDHHC8	3	-0.029639	1	0	*FAM189A2*	4	-0.089491	1	0
ARHGAP11B	1	-0.029603	1	0	*CUL4A*	2	-0.089308	1	0
CALR3	4	-0.029502	1	0	*FBXO10*	4	-0.089261	1	0
SDAD1	3	-0.029472	1	0	*SCN3B*	3	-0.089189	1	0
C4orf45	4	-0.029388	1	0	*SLAMF1*	3	-0.089171	1	0
ZFP28	4	-0.029342	1	0	*TXNRD2*	4	-0.089163	1	0
GPR137C	4	-0.029314	1	0	*GLIPR1L1*	4	-0.089147	1	0
SOS2	4	-0.029195	1	0	*GPATCH3*	4	-0.08913	1	0
ZCCHC17	3	-0.029055	1	0	*SAR1A*	4	-0.089074	1	0
ALPK1	4	-0.028981	1	0	*HINT2*	3	-0.089053	1	0
ZNF226	2	-0.02897	1	0	*CAMK2N1*	4	-0.089051	1	0
GIT2	3	-0.02893	1	0	*INS-IGF2*	4	-0.08896	1	0
COLQ	4	-0.028898	1	0	*LRRC66*	4	-0.088886	1	0
RBM44	4	-0.028842	1	0	*PLAC9*	2	-0.088863	1	0
CGAS	4	-0.028839	1	0	*CYP4F22*	4	-0.088694	1	0
CCDC30	4	-0.028728	1	0	*GNG12*	4	-0.08869	1	0
OSGIN1	4	-0.028686	1	0	*WNT8B*	4	-0.088671	1	0
AKAP17A	4	-0.028679	1	0	*H2BW2*	4	-0.088501	1	0
TLR7	4	-0.028679	1	0	*RAPSN*	4	-0.088452	1	0
GJB6	4	-0.028566	1	0	*C5*	4	-0.088419	1	0
ASH1L	4	-0.028519	1	0	*DTX3L*	3	-0.088411	1	0
ALKBH7	2	-0.02842	1	0	*RNF34*	4	-0.088393	1	0
PDE4B	4	-0.0284	1	0	*CEP135*	4	-0.088374	1	0
SCO1	4	-0.028241	1	0	*EXO5*	4	-0.088353	1	0
E2F6	2	-0.028116	1	0	*RPL22*	1	-0.088268	1	0
EHMT2	4	-0.028109	1	0	*AACS*	4	-0.088211	1	0
FAM171A1	4	-0.028088	1	0	*CCDC167*	2	-0.087967	1	0
MAGEC2	4	-0.028085	1	0	*NDUFA7*	4	-0.087867	1	0
DNAH7	4	-0.028038	1	0	*EIF4H*	4	-0.087763	1	0
SIT1	4	-0.027984	1	0	*FHL1*	4	-0.087749	1	0
OTUD7A	3	-0.027964	1	0	*CD164L2*	4	-0.087748	1	0
SPATA17	3	-0.02793	1	0	*AATK*	4	-0.087712	1	0
NFKBID	3	-0.027898	1	0	*GUSB*	4	-0.087684	1	0
CASP9	4	-0.027871	1	0	*CHD2*	4	-0.087675	1	0
NAA80	4	-0.027865	1	0	*TIPRL*	3	-0.087609	1	0
CHD1L	3	-0.027813	1	0	*PRKAR2B*	4	-0.087606	1	0
BIN2	2	-0.027755	1	0	*TXNDC12*	3	-0.08749	1	0
ATG4C	4	-0.027733	1	0	*LRRC57*	4	-0.087388	1	0
CALHM2	3	-0.027691	1	0	*CATSPER4*	2	-0.08734	1	0
SFTPB	4	-0.027686	1	0	*GIMAP4*	4	-0.087317	1	0
MCM8	4	-0.027679	1	0	*ZNF44*	4	-0.087295	1	0
FSIP2	4	-0.027666	1	0	*ATP6V1H*	4	-0.087294	1	0
GAREM1	3	-0.027664	1	0	*DPAGT1*	3	-0.087153	1	0
NSUN5	4	-0.027623	1	0	*VPS16*	3	-0.087096	1	0

APP	4	-0.027341	1	0	*NEBL*	4	-0.086949	1	0
DRP2	3	-0.027288	1	0	*PDF*	4	-0.086932	1	0
ANXA2	4	-0.027224	1	0	*BICD1*	4	-0.086745	1	0
NBEAL1	4	-0.027216	1	0	*NEXN*	4	-0.086673	1	0
VPREB3	3	-0.027093	1	0	*ABCG4*	4	-0.086626	1	0
ZNF165	2	-0.027	1	0	*POU2F1*	4	-0.086569	1	0
ADI1	2	-0.026971	1	0	*HHLA3*	4	-0.086459	1	0
OSBPL5	4	-0.02694	1	0	*MMP23B*	2	-0.086389	1	0
TMEM185A	3	-0.026936	1	0	*LRRTM4*	3	-0.086361	1	0
NCR1	4	-0.026887	1	0	*BORA*	4	-0.086354	1	0
NRG1	4	-0.026868	1	0	*MMP14*	4	-0.086316	1	0
TUFM	4	-0.026817	1	0	*PIGX*	4	-0.086307	1	0
ADGRV1	4	-0.026813	1	0	*ATP6V1A*	4	-0.086264	1	0
TENT5B	4	-0.026785	1	0	*AKIRIN1*	3	-0.08626	1	0
NOL4	3	-0.026632	1	0	*ACBD6*	4	-0.086246	1	0
SIRPA	3	-0.026607	1	0	*CCDC198*	4	-0.086197	1	0
ANKRD18B	4	-0.026444	1	0	*CD40LG*	4	-0.086174	1	0
C1orf131	4	-0.026394	1	0	*PYCR2*	4	-0.086151	1	0
LMBRD1	4	-0.026356	1	0	*DNAJC19*	2	-0.086064	1	0
C15orf61	4	-0.026333	1	0	*RUFY2*	4	-0.086036	1	0
CCDC148	4	-0.02632	1	0	*FMR1*	4	-0.085844	1	0
SCX	4	-0.026265	1	0	*PERM1*	2	-0.085817	1	0
EFCAB12	3	-0.026181	1	0	*TRAPPC3L*	4	-0.085691	1	0
TDRD9	3	-0.026169	1	0	*MAEL*	4	-0.085687	1	0
NKX2-3	3	-0.026119	1	0	*IL11*	3	-0.085587	1	0
KCNJ10	3	-0.025969	1	0	*NUP210L*	4	-0.085506	1	0
RAP1GDS1	4	-0.025949	1	0	*NELFE*	4	-0.085452	1	0
IFFO2	3	-0.025908	1	0	*UBE3C*	4	-0.085451	1	0
TM6SF1	3	-0.025895	1	0	*ACOT6*	4	-0.085418	1	0
RBFOX3	4	-0.025893	1	0	*PLCB4*	4	-0.085389	1	0
FOXC1	4	-0.025837	1	0	*SYCE3*	4	-0.085103	1	0
PHETA1	3	-0.025752	1	0	*TMEM267*	4	-0.08504	1	0
MRPL32	3	-0.025686	1	0	*DAGLB*	4	-0.084999	1	0
POFUT2	4	-0.025609	1	0	*ETFBKMT*	4	-0.084971	1	0
CD247	4	-0.025593	1	0	*IDH1*	4	-0.084968	1	0
MOSMO	4	-0.02557	1	0	*A1BG*	3	-0.084894	1	0
PIGZ	3	-0.025498	1	0	*IGFL1*	4	-0.084718	1	0
C11orf45	4	-0.025475	1	0	*FZD6*	4	-0.084569	1	0
METAP1D	4	-0.025393	1	0	*LONRF2*	4	-0.084443	1	0
UTP18	4	-0.025374	1	0	*DNAH17*	3	-0.084441	1	0
TEX48	4	-0.025335	1	0	*ZBTB37*	3	-0.084433	1	0
HSPA4	4	-0.025262	1	0	*SYT5*	3	-0.084402	1	0
PRIMPOL	3	-0.025123	1	0	*TIGD6*	3	-0.084377	1	0
C16orf86	4	-0.024966	1	0	*TBCD*	4	-0.08426	1	0
PC	3	-0.024904	1	0	*SPG21*	3	-0.084206	1	0
MAPK10	4	-0.024881	1	0	*NYX*	4	-0.083995	1	0
MTHFSD	4	-0.024856	1	0	*HLA-DPA1*	3	-0.083993	1	0
CYP2C9	4	-0.024822	1	0	*L3MBTL4*	3	-0.083865	1	0
PLB1	4	-0.024796	1	0	*CHCHD7*	4	-0.083815	1	0
ILDR2	4	-0.024767	1	0	*HMGB3*	4	-0.083813	1	0
LCE5A	4	-0.024741	1	0	*AGPS*	4	-0.083753	1	0
NAPSA	4	-0.024669	1	0	*NFKBIL1*	4	-0.083609	1	0
CHRDL2	4	-0.024652	1	0	*GPC2*	2	-0.083515	1	0
ZNF771	3	-0.024514	1	0	*PTMA*	3	-0.083509	1	0
NEK11	4	-0.024402	1	0	*PCDHB10*	4	-0.083508	1	0
PI15	4	-0.024374	1	0	*SPEN*	4	-0.083495	1	0
SSR4	4	-0.024354	1	0	*PDZK1IP1*	4	-0.083491	1	0
KRTAP1-1	3	-0.024309	1	0	*LGALSL*	2	-0.083442	1	0

APOL4	4	-0.02426	1	0	*RPRM*	1	-0.083387	1	0
B4GALT7	2	-0.02426	1	0	*KHDC1*	3	-0.083383	1	0
SYT17	4	-0.024252	1	0	*IHH*	4	-0.08336	1	0
NEK10	4	-0.024069	1	0	*GNPTG*	4	-0.083301	1	0
FBXL18	3	-0.023946	1	0	*SWT1*	4	-0.083196	1	0
GLIS3	4	-0.023809	1	0	*LRRC1*	3	-0.083147	1	0
CISH	4	-0.023721	1	0	*MAMLD1*	4	-0.083114	1	0
MAPKAPK3	4	-0.023696	1	0	*C16orf95*	4	-0.082965	1	0
SLC7A1	4	-0.023608	1	0	*TRIM23*	4	-0.08289	1	0
PCGF2	3	-0.023534	1	0	*SLC2A10*	4	-0.082823	1	0
KIAA1211L	4	-0.023533	1	0	*PCGF5*	4	-0.082692	1	0
DCAF16	4	-0.02347	1	0	*TYW1B*	3	-0.082661	1	0
CLEC4A	3	-0.023452	1	0	*C8orf34*	4	-0.082478	1	0
PIGQ	2	-0.023451	1	0	*GPC4*	4	-0.082477	1	0
PDLIM7	3	-0.02338	1	0	*PJVK*	3	-0.082449	1	0
SEMA6C	4	-0.023331	1	0	*ANP32A*	3	-0.082372	1	0
B4GALT5	2	-0.023279	1	0	*GCM1*	3	-0.082329	1	0
CORIN	4	-0.0232	1	0	*BRK1*	3	-0.082218	1	0
INPP5B	3	-0.023183	1	0	*GPR75-ASB3*	1	-0.082015	1	0
WNT4	4	-0.023142	1	0	*ZNF652*	4	-0.081953	1	0
TMCO4	4	-0.023126	1	0	*ABCC1*	4	-0.081952	1	0
CD209	3	-0.023119	1	0	*DNAJA1*	4	-0.081862	1	0
SNCAIP	4	-0.022889	1	0	*BSPRY*	4	-0.081705	1	0
TMEM18	4	-0.022857	1	0	*LEMD3*	4	-0.081605	1	0
RUFY2	4	-0.022765	1	0	*APOBEC3B*	4	-0.081541	1	0
ECM1	4	-0.022544	1	0	*TRDMT1*	4	-0.081497	1	0
KRT8	2	-0.022525	1	0	*DCDC2*	3	-0.081487	1	0
ZSWIM9	4	-0.022513	1	0	*TTLL11*	3	-0.081378	1	0
SSPO	4	-0.022493	1	0	*PHOSPHO1*	4	-0.081315	1	0
BEND7	3	-0.02248	1	0	*TMTC1*	4	-0.081311	1	0
LRRN1	4	-0.022366	1	0	*SH3BP5*	4	-0.081285	1	0
MBOAT2	2	-0.022299	1	0	*CORO2A*	4	-0.081234	1	0
PRKCZ	2	-0.022184	1	0	*LRRC31*	4	-0.081114	1	0
SPATA4	3	-0.022181	1	0	*TRIM67*	4	-0.081106	1	0
PADI2	4	-0.022172	1	0	*SLC5A3*	4	-0.081059	1	0
PCSK1N	4	-0.022158	1	0	*VANGL2*	3	-0.081052	1	0
FKBP8	4	-0.022143	1	0	*GBA2*	4	-0.081019	1	0
PRXL2A	4	-0.02212	1	0	*QRFPR*	4	-0.080977	1	0
TYR	4	-0.022046	1	0	*DCP1A*	4	-0.080871	1	0
PID1	4	-0.022011	1	0	*SLC25A52*	3	-0.080715	1	0
CAT	4	-0.021964	1	0	*CYP4F12*	4	-0.080707	1	0
RETREG3	4	-0.021963	1	0	*DNAAF2*	2	-0.080539	1	0
HTR2C	4	-0.021961	1	0	*C4orf19*	4	-0.080523	1	0
PODNL1	4	-0.021923	1	0	*TM4SF20*	2	-0.080493	1	0
ITFG1	4	-0.021838	1	0	*SYNDIG1L*	3	-0.080419	1	0
PTGS1	3	-0.021657	1	0	*ANKRD11*	4	-0.080416	1	0
GLI2	2	-0.021641	1	0	*HAGHL*	2	-0.080355	1	0
GJB1	4	-0.021624	1	0	*KCTD16*	4	-0.08035	1	0
OR1N1	4	-0.021609	1	0	*CRYBG2*	4	-0.080328	1	0
UBALD1	4	-0.021543	1	0	*CD72*	4	-0.080327	1	0
C10orf105	4	-0.02152	1	0	*SKIDA1*	2	-0.080317	1	0
PRDX4	4	-0.021512	1	0	*CCDC175*	4	-0.080271	1	0
PLAGL2	3	-0.0215	1	0	*CREB3*	4	-0.080211	1	0
GBP7	2	-0.021476	1	0	*SLC49A3*	4	-0.080166	1	0
PCP2	3	-0.021395	1	0	*ZNF428*	4	-0.080109	1	0
RBM7	3	-0.021368	1	0	*TPR*	4	-0.080003	1	0
TRADD	4	-0.021351	1	0	*C2orf66*	4	-0.079948	1	0
CHSY1	3	-0.02132	1	0	*CHRM3*	4	-0.079898	1	0

PNPO	3	-0.021315	1	0	*DNAJB5*	4	-0.079841	1	0
SEC13	2	-0.021303	1	0	*DHFR*	1	-0.079594	1	0
EIF4EBP1	4	-0.021251	1	0	*HLA-G*	3	-0.079593	1	0
DOCK11	3	-0.021239	1	0	*CALB1*	4	-0.079549	1	0
MARCHF9	4	-0.021202	1	0	*HAPLN3*	3	-0.079532	1	0
EMC8	4	-0.021186	1	0	*HRH3*	3	-0.079456	1	0
NOCT	4	-0.021179	1	0	*TRPM3*	4	-0.079453	1	0
BTBD9	3	-0.021153	1	0	*TOMM40L*	3	-0.079446	1	0
PCDHGA4	3	-0.021129	1	0	*SLC25A43*	3	-0.079429	1	0
FAM3D	4	-0.021086	1	0	*IRGQ*	4	-0.079369	1	0
WDR87	2	-0.020993	1	0	*IGFALS*	4	-0.079353	1	0
DTD2	4	-0.020964	1	0	*FGFBP1*	4	-0.079335	1	0
HDHD3	2	-0.02095	1	0	*MFSD4A*	3	-0.07931	1	0
TAFA4	4	-0.020941	1	0	*CABP7*	2	-0.079251	1	0
ARID4A	3	-0.020936	1	0	*NAT8L*	4	-0.079238	1	0
ZNF449	3	-0.020903	1	0	*RELN*	4	-0.079226	1	0
RNPS1	3	-0.020768	1	0	*FAM174B*	3	-0.079199	1	0
MRPS30	4	-0.020554	1	0	*SATB2*	4	-0.079078	1	0
SH2D5	4	-0.020525	1	0	*ZBTB48*	3	-0.079068	1	0
NSD2	3	-0.020502	1	0	*ARL6IP5*	4	-0.079058	1	0
FOXF2	4	-0.020483	1	0	*H4C13*	4	-0.078981	1	0
NOTCH1	4	-0.02045	1	0	*GABRQ*	4	-0.078949	1	0
WBP1	1	-0.020417	1	0	*NKRF*	4	-0.078945	1	0
ZSWIM3	2	-0.020352	1	0	*RGS8*	4	-0.078915	1	0
TRIM55	2	-0.020124	1	0	*TPRG1*	4	-0.078832	1	0
IL13	2	-0.020065	1	0	*PRRC1*	4	-0.078831	1	0
EFL1	3	-0.019989	1	0	*HRG*	3	-0.078706	1	0
GPLD1	3	-0.019861	1	0	*LHX2*	2	-0.078626	1	0
TRMT10B	3	-0.019853	1	0	*ZSCAN9*	3	-0.078605	1	0
POMT2	3	-0.019832	1	0	*EFHB*	4	-0.0786	1	0
ZFHX2	3	-0.019802	1	0	*HSCB*	4	-0.078579	1	0
ZSWIM1	3	-0.019794	1	0	*PIGK*	4	-0.078559	1	0
SEPTIN9	3	-0.019763	1	0	*H2AC4*	4	-0.078526	1	0
KLHL10	4	-0.019746	1	0	*SETBP1*	4	-0.078504	1	0
ALPG	4	-0.019661	1	0	*ENTPD1*	4	-0.078501	1	0
CAV1	4	-0.019546	1	0	*FBXW5*	2	-0.078469	1	0
AP3B1	3	-0.019542	1	0	*TNIK*	3	-0.078402	1	0
TNFAIP8L2	4	-0.019531	1	0	*SLC25A24*	4	-0.078375	1	0
UCN2	4	-0.019525	1	0	*CCL4*	3	-0.078356	1	0
UNC5B	3	-0.019494	1	0	*RBPJL*	3	-0.078334	1	0
FAM174B	3	-0.019451	1	0	*STARD13*	3	-0.078249	1	0
NTPCR	3	-0.019433	1	0	*PDCD1LG2*	4	-0.078145	1	0
CASC4	4	-0.019384	1	0	*CLPTM1*	4	-0.078122	1	0
MORF4L1	3	-0.019384	1	0	*SLC10A2*	3	-0.078109	1	0
GPD2	4	-0.019331	1	0	*DNTT*	2	-0.07805	1	0
BBS2	4	-0.01931	1	0	*HMGN1*	1	-0.078036	1	0
GGACT	2	-0.019287	1	0	*RELB*	2	-0.078034	1	0
ADA2	3	-0.019282	1	0	*CABLES2*	4	-0.078022	1	0
PLEKHB2	4	-0.019268	1	0	*SOX15*	2	-0.07798	1	0
GPR157	4	-0.019256	1	0	*C16orf54*	4	-0.077977	1	0
TENM3	4	-0.019199	1	0	*MMP11*	4	-0.077899	1	0
IGF1R	4	-0.019178	1	0	*TMEM17*	4	-0.077868	1	0
VGLL1	3	-0.019165	1	0	*SLC24A1*	3	-0.077819	1	0
HNRNPH3	4	-0.018981	1	0	*TAAR5*	3	-0.077746	1	0
MTHFR	3	-0.018956	1	0	*CAMK1*	2	-0.077727	1	0
TNFSF13	2	-0.018896	1	0	*SEC31B*	2	-0.077693	1	0
GTPBP3	4	-0.018807	1	0	*CCDC166*	4	-0.077624	1	0
MT1F	3	-0.018732	1	0	*NAPEPLD*	4	-0.077465	1	0

LTF	4	-0.018703	1	0	*MPP5*	2	-0.077407	1	0
TNS2	4	-0.018579	1	0	*BIN1*	4	-0.077392	1	0
EHD3	4	-0.018552	1	0	*WWP1*	4	-0.077267	1	0
HSD17B10	3	-0.018523	1	0	*TAGLN2*	4	-0.077257	1	0
LMBRD2	4	-0.018479	1	0	*CASR*	4	-0.07719	1	0
TSPAN32	2	-0.018289	1	0	*GNRHR*	3	-0.077188	1	0
SECTM1	2	-0.01827	1	0	*ZNF578*	4	-0.077171	1	0
E2F5	3	-0.018224	1	0	*FAM83G*	3	-0.077065	1	0
PLK3	3	-0.018126	1	0	*TMEM92*	4	-0.077025	1	0
KCNS1	4	-0.018088	1	0	*GDA*	4	-0.076966	1	0
PTPRO	3	-0.017936	1	0	*ZNF649*	4	-0.076966	1	0
TMEM231	3	-0.017904	1	0	*ALDH9A1*	4	-0.076964	1	0
ZNF599	4	-0.017904	1	0	*MEX3D*	3	-0.076941	1	0
ARAF	4	-0.017848	1	0	*NAP1L1*	3	-0.076929	1	0
IL23A	2	-0.017839	1	0	*DNMT3A*	3	-0.076904	1	0
ZFAND2B	3	-0.017824	1	0	*OGFRL1*	2	-0.076901	1	0
CACNA1G	3	-0.017779	1	0	*GALNT11*	3	-0.076885	1	0
INSYN1	4	-0.01777	1	0	*TAS2R14*	3	-0.076875	1	0
MITD1	4	-0.017764	1	0	*N4BP3*	4	-0.076661	1	0
ZC3H3	3	-0.017726	1	0	*HYAL4*	4	-0.07664	1	0
SNRPD3	4	-0.017671	1	0	*STK26*	4	-0.076589	1	0
WHAMM	4	-0.017592	1	0	*PACSIN3*	4	-0.07646	1	0
TNNC1	4	-0.017577	1	0	*DYRK2*	4	-0.07642	1	0
ANKRD23	4	-0.017367	1	0	*CLIC4*	4	-0.076392	1	0
DCAF4	3	-0.017337	1	0	*HDAC6*	3	-0.076279	1	0
GSTM3	3	-0.017297	1	0	*BIK*	3	-0.076271	1	0
SPINK5	3	-0.017294	1	0	*EN1*	1	-0.076241	1	0
BID	4	-0.017281	1	0	*KRT6A*	3	-0.076221	1	0
IFT57	4	-0.017216	1	0	*MBOAT7*	3	-0.076216	1	0
CYP7B1	4	-0.017192	1	0	*TOMM7*	4	-0.076058	1	0
CELA3A	2	-0.017153	1	0	*PLEKHJ1*	4	-0.076043	1	0
ARL4C	4	-0.017028	1	0	*GCDH*	4	-0.07603	1	0
LSM10	4	-0.016913	1	0	*RCAN3*	4	-0.075834	1	0
CBWD2	3	-0.016909	1	0	*UNC5CL*	4	-0.07581	1	0
TLCD3B	3	-0.01688	1	0	*DYNLL2*	4	-0.075769	1	0
TNFRSF11A	4	-0.016855	1	0	*SEPTIN6*	4	-0.075735	1	0
ZDHHC13	4	-0.016842	1	0	*VNN1*	4	-0.075704	1	0
GPR161	3	-0.016781	1	0	*OGFOD1*	4	-0.075567	1	0
TCP11	4	-0.016776	1	0	*CALM1*	3	-0.075558	1	0
LCN2	4	-0.016667	1	0	*CSNK2A2*	4	-0.075551	1	0
DDX3Y	4	-0.016665	1	0	*MBD2*	4	-0.07553	1	0
RHEBL1	4	-0.016638	1	0	*HPS1*	3	-0.07551	1	0
ZSWIM5	4	-0.016569	1	0	*ZBTB8B*	4	-0.075484	1	0
PRDM16	3	-0.016533	1	0	*PCDH8*	3	-0.075464	1	0
SSBP2	4	-0.016523	1	0	*SFMBT2*	4	-0.07544	1	0
TOX4	4	-0.016513	1	0	*H1-2*	4	-0.075397	1	0
SYBU	3	-0.016477	1	0	*FGB*	2	-0.075313	1	0
MED15	4	-0.016382	1	0	*DPYSL2*	2	-0.075138	1	0
EGFL7	4	-0.016355	1	0	*SLC28A3*	4	-0.074952	1	0
CDK19	4	-0.016346	1	0	*SHLD2*	2	-0.074895	1	0
BICDL2	4	-0.01633	1	0	*FOXO4*	4	-0.074819	1	0
AHCY	3	-0.016287	1	0	*OLFML1*	4	-0.074766	1	0
PNPLA8	2	-0.016154	1	0	*ISOC2*	3	-0.074745	1	0
TDRD10	4	-0.016139	1	0	*IQSEC1*	2	-0.074741	1	0
ZNF823	4	-0.01606	1	0	*CXCL17*	4	-0.074667	1	0
APIP	2	-0.016021	1	0	*CCDC34*	3	-0.074615	1	0
AMT	3	-0.016003	1	0	*RBL1*	4	-0.074592	1	0
RBP5	4	-0.015975	1	0	*RNPEP*	3	-0.074414	1	0

NLRP7	4	-0.01591	1	0	*ERG*	4	-0.074297	1	0
ANKZF1	4	-0.015885	1	0	*PKDREJ*	4	-0.074282	1	0
PPFIA2	3	-0.015859	1	0	*ALG9*	4	-0.074239	1	0
RUNX2	4	-0.01585	1	0	*CLDN14*	2	-0.074217	1	0
RGPD3	2	-0.015802	1	0	*TM2D1*	3	-0.074183	1	0
RILPL1	4	-0.015585	1	0	*MEX3B*	2	-0.074176	1	0
DNASE2B	4	-0.015392	1	0	*EREG*	4	-0.074085	1	0
SIM2	4	-0.015383	1	0	*MINDY3*	4	-0.074069	1	0
SGSH	4	-0.015373	1	0	*BCKDHA*	4	-0.074029	1	0
SH3TC2	4	-0.015281	1	0	*SARDH*	4	-0.073848	1	0
PKD2L2	4	-0.015279	1	0	*HOXA1*	3	-0.073566	1	0
SKOR1	2	-0.015266	1	0	*XYLT2*	1	-0.073564	1	0
SMARCA4	3	-0.015262	1	0	*SYNGR1*	4	-0.073453	1	0
TMEM248	4	-0.015256	1	0	*RNF167*	4	-0.07343	1	0
ADRA2C	3	-0.015231	1	0	*ZNF414*	4	-0.073395	1	0
ZNF347	4	-0.01516	1	0	*MSMO1*	4	-0.073357	1	0
CXCR2	3	-0.015151	1	0	*KIF22*	2	-0.07331	1	0
TMEM238	3	-0.015118	1	0	*SLC16A8*	3	-0.07328	1	0
BNIP5	4	-0.015092	1	0	*IFIH1*	4	-0.073267	1	0
CSRP1	4	-0.015004	1	0	*JHY*	4	-0.073222	1	0
CKAP5	4	-0.01491	1	0	*VSIG10*	4	-0.073219	1	0
APBB1	3	-0.014817	1	0	*TGDS*	4	-0.07321	1	0
ZNF600	2	-0.014792	1	0	*WDR90*	3	-0.073165	1	0
SIRPB2	4	-0.014698	1	0	*FCAMR*	4	-0.073101	1	0
HLA-DMB	3	-0.014631	1	0	*TMEM35B*	4	-0.073099	1	0
PEBP4	2	-0.014627	1	0	*SLC2A7*	3	-0.073036	1	0
B3GALT5	2	-0.014583	1	0	*PRKCB*	3	-0.073005	1	0
FH	4	-0.014583	1	0	*BICRAL*	4	-0.073	1	0
NPHS1	4	-0.014471	1	0	*ZBTB25*	4	-0.072981	1	0
KNOP1	4	-0.014454	1	0	*USP28*	4	-0.072874	1	0
CPNE2	4	-0.014436	1	0	*LOC10013037*	2	-0.07271	1	0
FAM229B	3	-0.014377	1	0	*STARD8*	4	-0.072691	1	0
ZNF644	1	-0.014375	1	0	*DUSP22*	3	-0.07252	1	0
C12orf66	3	-0.014296	1	0	*C17orf112*	4	-0.072505	1	0
C9orf153	4	-0.014291	1	0	*NPY*	3	-0.072504	1	0
SLC16A12	3	-0.014157	1	0	*SLC22A7*	3	-0.07244	1	0
ZNF763	3	-0.014121	1	0	*CDRT15*	2	-0.072424	1	0
RNF111	4	-0.013924	1	0	*LCT*	3	-0.072357	1	0
ABCA10	3	-0.013807	1	0	*TRIM69*	4	-0.072335	1	0
ARHGAP29	4	-0.013774	1	0	*KANSL1L*	4	-0.072303	1	0
ZXDC	3	-0.013761	1	0	*RFTN1*	4	-0.072224	1	0
SC5D	4	-0.013714	1	0	*BNIP3L*	3	-0.072147	1	0
FBXO24	2	-0.013704	1	0	*PLPPR2*	2	-0.072127	1	0
GID4	2	-0.013641	1	0	*SFXN3*	2	-0.072094	1	0
ZNF513	4	-0.013641	1	0	*ZNF709*	4	-0.071947	1	0
PTPN7	3	-0.013569	1	0	*DSTN*	1	-0.071923	1	0
PRELID2	4	-0.013552	1	0	*RAB13*	4	-0.071899	1	0
CTSA	4	-0.013463	1	0	*CD274*	4	-0.071888	1	0
NFIX	3	-0.013424	1	0	*CPEB2*	3	-0.071755	1	0
FARP2	2	-0.013377	1	0	*NID1*	4	-0.071727	1	0
UGGT1	4	-0.013375	1	0	*PGBD1*	3	-0.071662	1	0
CAMP	4	-0.013362	1	0	*ADAMTS2*	4	-0.071609	1	0
STRADB	4	-0.01336	1	0	*RPH3A*	4	-0.071603	1	0
CRYM	2	-0.013297	1	0	*GMPPA*	3	-0.07145	1	0
MANSC1	3	-0.013211	1	0	*IFNE*	4	-0.071442	1	0
ZNF608	2	-0.01317	1	0	*LSM4*	3	-0.071398	1	0
TIMM23	3	-0.013048	1	0	*CALB2*	4	-0.071339	1	0
CDK15	4	-0.012977	1	0	*BCCIP*	3	-0.071243	1	0

RAI14	4	-0.012824	1	0	*HSD17B6*	3	-0.07119	1	0
STAT6	4	-0.01281	1	0	*CROCC*	4	-0.071177	1	0
ARSL	4	-0.012802	1	0	*ZNF385B*	4	-0.071011	1	0
HOXA5	2	-0.012772	1	0	*CDH10*	2	-0.070978	1	0
HRG	3	-0.012577	1	0	*ERICH4*	3	-0.070935	1	0
TMEM52B	4	-0.012575	1	0	*KTN1*	3	-0.070894	1	0
H3C2	3	-0.012505	1	0	*NPM2*	4	-0.070874	1	0
NLN	4	-0.012487	1	0	*GNS*	4	-0.070829	1	0
UBE2Q2	4	-0.012449	1	0	*FGF11*	4	-0.07078	1	0
PIWIL4	3	-0.012441	1	0	*CEP120*	4	-0.070759	1	0
PLEKHF2	2	-0.012414	1	0	*KDM1B*	4	-0.070704	1	0
CPM	4	-0.012405	1	0	*FAM151B*	4	-0.070651	1	0
MPV17L2	4	-0.012364	1	0	*VSIG10L*	3	-0.070647	1	0
NIBAN1	3	-0.012362	1	0	*PKN3*	3	-0.070638	1	0
SLC15A2	4	-0.012321	1	0	*ZNF736*	3	-0.07057	1	0
H1-4	3	-0.012112	1	0	*VIPAS39*	4	-0.070352	1	0
AGBL4	4	-0.012103	1	0	*FSCN3*	4	-0.070343	1	0
GNAO1	4	-0.01178	1	0	*TXNDC15*	4	-0.070252	1	0
AZI2	3	-0.011764	1	0	*GRASP*	4	-0.070226	1	0
ANKK1	4	-0.011688	1	0	*GRHL3*	4	-0.070207	1	0
SMAD4	4	-0.011529	1	0	*CTIF*	4	-0.070172	1	0
ALG10B	2	-0.011516	1	0	*OSGEPL1*	2	-0.070165	1	0
USP7	4	-0.011484	1	0	*SPIN2B*	1	-0.070148	1	0
CDC25C	4	-0.011416	1	0	*TBX19*	4	-0.069913	1	0
DUSP16	3	-0.011382	1	0	*ACSBG2*	4	-0.069904	1	0
DSN1	3	-0.011238	1	0	*NRROS*	4	-0.069852	1	0
HSF2BP	2	-0.011223	1	0	*PRR5L*	3	-0.069794	1	0
METTL22	4	-0.01122	1	0	*PLXNA2*	3	-0.069737	1	0
VASH1	4	-0.011217	1	0	*SLC38A5*	3	-0.06969	1	0
MCU	3	-0.011216	1	0	*GALNT5*	4	-0.069669	1	0
FMNL3	3	-0.011154	1	0	*EXOC6*	4	-0.069642	1	0
CSNK2A2	4	-0.01085	1	0	*PIAS4*	4	-0.069631	1	0
ZNF76	2	-0.010842	1	0	*SNX21*	4	-0.06962	1	0
C6orf58	4	-0.010785	1	0	*RNF112*	4	-0.0696	1	0
CEP350	4	-0.010781	1	0	*TP53INP1*	4	-0.069542	1	0
ZC2HC1A	4	-0.010767	1	0	*EDDM3B*	4	-0.069471	1	0
ZSCAN16	3	-0.010647	1	0	*PRMT3*	4	-0.069463	1	0
C12orf60	4	-0.010624	1	0	*PHACTR2*	2	-0.069388	1	0
ADGRF5	4	-0.010562	1	0	*SLC2A12*	4	-0.069383	1	0
FABP3	4	-0.010555	1	0	*MAFA*	2	-0.069297	1	0
PDIA4	4	-0.01055	1	0	*UBE2D2*	3	-0.069286	1	0
LDLRAP1	4	-0.010469	1	0	*ADH5*	2	-0.069268	1	0
RAB25	3	-0.010457	1	0	*PADI3*	3	-0.069266	1	0
SDC2	3	-0.010386	1	0	*CDYL2*	4	-0.06924	1	0
KCNQ3	4	-0.010379	1	0	*CDK6*	4	-0.069235	1	0
TJP3	3	-0.010357	1	0	*MCAM*	3	-0.069188	1	0
TIMM44	2	-0.010234	1	0	*SENP2*	4	-0.069109	1	0
ADPRHL2	4	-0.01019	1	0	*ZMYM3*	3	-0.069109	1	0
LCP2	4	-0.010174	1	0	*CCN3*	4	-0.069053	1	0
NBEA	4	-0.010145	1	0	*PRKD1*	4	-0.069034	1	0
PTPRCAP	3	-0.010086	1	0	*STXBP3*	3	-0.068984	1	0
LYRM9	4	-0.010069	1	0	*PLA2G12A*	4	-0.068978	1	0
CLCN1	4	-0.009919	1	0	*POLE3*	4	-0.068978	1	0
GPX7	4	-0.009897	1	0	*MAGED1*	4	-0.068929	1	0
SNX16	4	-0.00978	1	0	*PTTG1IP*	4	-0.068922	1	0
ITGA3	4	-0.009733	1	0	*CLDN22*	2	-0.068906	1	0
STMN1	2	-0.009718	1	0	*FBLN5*	4	-0.068906	1	0
C1QL4	4	-0.009664	1	0	*FGFR4*	3	-0.068844	1	0

NPHP1	4	-0.00964	1	0	*KRT1*	4	-0.068781	1	0
FAM160B1	4	-0.009635	1	0	*AANAT*	4	-0.068717	1	0
REEP3	4	-0.009417	1	0	*TESC*	3	-0.068599	1	0
DOCK8	3	-0.009394	1	0	*SCRN1*	3	-0.068538	1	0
PYURF	3	-0.009394	1	0	*TMEM230*	2	-0.068404	1	0
RWDD3	4	-0.009306	1	0	*LUZP4*	3	-0.068402	1	0
ADAMTS16	4	-0.009269	1	0	*DTD2*	4	-0.068369	1	0
HES7	3	-0.009209	1	0	*TSKU*	3	-0.068232	1	0
MAP4K3	3	-0.009195	1	0	*NSMF*	4	-0.068173	1	0
PCED1B	4	-0.009122	1	0	*RPARP-AS1*	1	-0.06809	1	0
TLR3	4	-0.009106	1	0	*ALCAM*	4	-0.068043	1	0
CFAP97	4	-0.009064	1	0	*TRIM63*	4	-0.068037	1	0
MYO18A	4	-0.009052	1	0	*RIC3*	4	-0.068022	1	0
CACNG1	4	-0.009024	1	0	*ZNF682*	4	-0.067991	1	0
MCOLN3	3	-0.008914	1	0	*TCF25*	3	-0.067926	1	0
CYP2C8	3	-0.008803	1	0	*GRAP2*	2	-0.067845	1	0
NUDT16	3	-0.008786	1	0	*AIDA*	2	-0.067825	1	0
TMCC2	4	-0.008475	1	0	*POGLUT3*	3	-0.067686	1	0
MBNL1	3	-0.008458	1	0	*PARPBP*	4	-0.067608	1	0
PCCB	3	-0.008403	1	0	*EVA1C*	2	-0.067478	1	0
MTM1	2	-0.008398	1	0	*MLST8*	2	-0.06746	1	0
ZNF528	4	-0.008379	1	0	*LVRN*	3	-0.067367	1	0
ROGDI	4	-0.008379	1	0	*CXCL6*	3	-0.067366	1	0
LGALS9B	1	-0.008356	1	0	*CCDC36*	4	-0.067328	1	0
GOLT1A	4	-0.008281	1	0	*H3C10*	4	-0.067325	1	0
TMEM208	4	-0.008247	1	0	*SMG7*	4	-0.067241	1	0
TTC21A	4	-0.008238	1	0	*RNF165*	4	-0.067199	1	0
MRPL24	4	-0.008225	1	0	*MARCHF9*	4	-0.067072	1	0
KLK7	4	-0.008224	1	0	*APC2*	3	-0.067045	1	0
FZD9	3	-0.008215	1	0	*RIPOR1*	3	-0.066916	1	0
RASSF10	4	-0.008194	1	0	*RRAS2*	4	-0.066901	1	0
TTBK1	4	-0.008112	1	0	*CLEC4F*	3	-0.066755	1	0
HEXA	3	-0.007937	1	0	*C9orf72*	4	-0.06674	1	0
AQP3	4	-0.007924	1	0	*CACNB2*	3	-0.066725	1	0
MKS1	3	-0.007815	1	0	*KIAA2013*	4	-0.066618	1	0
SLC26A9	4	-0.007763	1	0	*HAS1*	4	-0.06661	1	0
LINC02801	3	-0.007697	1	0	*C16orf96*	4	-0.066583	1	0
COX4I1	3	-0.007651	1	0	*ZNF285*	4	-0.06645	1	0
LIX1	4	-0.007641	1	0	*WASHC2A*	1	-0.066286	1	0
BEX3	4	-0.007574	1	0	*FFAR1*	4	-0.066237	1	0
DEFA5	4	-0.0075	1	0	*ALG6*	3	-0.066232	1	0
THBS3	4	-0.007353	1	0	*CAMKK1*	4	-0.066074	1	0
PPP4R3B	4	-0.007324	1	0	*NAT1*	4	-0.06603	1	0
RIOK1	4	-0.00721	1	0	*ZCCHC12*	3	-0.065966	1	0
DNAAF1	4	-0.007203	1	0	*MFAP2*	4	-0.06587	1	0
PRICKLE3	4	-0.007076	1	0	*PRKCI*	4	-0.065831	1	0
SUV39H2	4	-0.007035	1	0	*SAP30*	2	-0.065817	1	0
KLHL22	4	-0.006996	1	0	*RGS4*	4	-0.065809	1	0
GGT1	3	-0.006948	1	0	*DNAJB11*	3	-0.06578	1	0
BICC1	3	-0.006857	1	0	*PSG6*	4	-0.065771	1	0
GRIP1	3	-0.006831	1	0	*CLEC4O*	4	-0.06577	1	0
CASS4	4	-0.006787	1	0	*NKX2-1*	3	-0.065765	1	0
USP50	4	-0.006785	1	0	*MMP19*	3	-0.06576	1	0
RELL2	4	-0.00675	1	0	*TFG*	4	-0.06562	1	0
CTNNAL1	3	-0.006746	1	0	*TRIM21*	2	-0.065607	1	0
ZNF850	4	-0.006707	1	0	*DOK1*	4	-0.065555	1	0
LSM2	4	-0.006697	1	0	*PLD6*	4	-0.065482	1	0
FLJ45513	4	-0.006697	1	0	*CA13*	4	-0.065443	1	0

RAB3IP	3	-0.006694	1	0	*PTPRR*	4	-0.06541	1	0
ANGPTL1	2	-0.006692	1	0	*ZNF415*	4	-0.065409	1	0
FN3KRP	4	-0.00665	1	0	*CRYBG1*	3	-0.065289	1	0
CMTR1	4	-0.006618	1	0	*GPR37*	2	-0.065161	1	0
USP54	4	-0.006572	1	0	*RHOH*	3	-0.065126	1	0
CCDC126	3	-0.006568	1	0	*ALKBH7*	2	-0.065082	1	0
HDDC2	3	-0.006543	1	0	*NHLH2*	4	-0.065074	1	0
FAP	4	-0.006535	1	0	*PRELID3A*	4	-0.065073	1	0
NUP214	2	-0.006502	1	0	*UNC13A*	4	-0.064987	1	0
GGA1	4	-0.006467	1	0	*SAG*	4	-0.064983	1	0
CHAC2	3	-0.006379	1	0	*FGFR2*	4	-0.064976	1	0
UBL4A	4	-0.006367	1	0	*MOGS*	3	-0.064867	1	0
RNF224	4	-0.00636	1	0	*IFIT3*	3	-0.064766	1	0
ESCO2	3	-0.00633	1	0	*TBXT*	3	-0.064756	1	0
SESTD1	4	-0.006271	1	0	*LSG1*	4	-0.064743	1	0
DGUOK	4	-0.006266	1	0	*HPCA*	4	-0.064727	1	0
ESYT3	4	-0.006203	1	0	*SLC26A6*	3	-0.064577	1	0
LRRC41	1	-0.006157	1	0	*FN1*	4	-0.064531	1	0
HAO2	4	-0.006146	1	0	*IK*	4	-0.064519	1	0
ZNF468	4	-0.006059	1	0	*ZNF813*	3	-0.064518	1	0
EPAS1	3	-0.006022	1	0	*CFAP53*	4	-0.064517	1	0
FAM53A	4	-0.005939	1	0	*DNAAF1*	4	-0.064402	1	0
RIC3	4	-0.00573	1	0	*ZSCAN30*	4	-0.064309	1	0
S1PR3	4	-0.005509	1	0	*VASH2*	4	-0.064254	1	0
ALDH3A2	3	-0.005503	1	0	*NALCN*	3	-0.064144	1	0
RAB39A	4	-0.005373	1	0	*RTL8B*	4	-0.064079	1	0
ABHD16A	4	-0.00536	1	0	*NCAM2*	4	-0.064034	1	0
ANKRD55	3	-0.00532	1	0	*SCARF1*	3	-0.063992	1	0
FBLN2	3	-0.005264	1	0	*CASP4*	3	-0.063968	1	0
SMDT1	4	-0.005219	1	0	*TNFAIP3*	4	-0.063918	1	0
SHROOM3	3	-0.005209	1	0	*ZNF157*	3	-0.063872	1	0
CACNA1A	3	-0.005202	1	0	*HOXD10*	3	-0.063617	1	0
SMIM10	4	-0.005199	1	0	*TNFRSF12A*	4	-0.063563	1	0
MBD2	4	-0.005135	1	0	*PRNP*	4	-0.063465	1	0
LRRN2	2	-0.005081	1	0	*ADAMTS16*	4	-0.063431	1	0
APBA2	1	-0.004964	1	0	*MACROD2*	4	-0.063411	1	0
SGPP2	3	-0.00488	1	0	*ABHD13*	4	-0.063389	1	0
USP10	4	-0.004872	1	0	*RXRB*	4	-0.063366	1	0
GIPR	3	-0.004825	1	0	*PCSK1*	4	-0.06335	1	0
PKHD1	4	-0.004766	1	0	*HNRNPLL*	4	-0.063279	1	0
WDR19	4	-0.00445	1	0	*RAB33A*	4	-0.063277	1	0
CCDC146	3	-0.004445	1	0	*MOSMO*	4	-0.063272	1	0
MDFI	4	-0.004435	1	0	*LRRC59*	4	-0.063227	1	0
ALPL	4	-0.004394	1	0	*TCEAL6*	4	-0.063213	1	0
MYO5C	4	-0.004375	1	0	*LIG4*	4	-0.063143	1	0
EIF4G3	3	-0.004372	1	0	*SLC25A34*	4	-0.063073	1	0
H4C6	4	-0.004367	1	0	*SLCO3A1*	4	-0.062907	1	0
HPDL	3	-0.004344	1	0	*C1QL3*	3	-0.062858	1	0
TCEAL6	4	-0.00433	1	0	*SCAND1*	3	-0.062827	1	0
XPR1	4	-0.004321	1	0	*CASP7*	1	-0.062806	1	0
VASH2	4	-0.004284	1	0	*ADGRG5*	3	-0.062697	1	0
PCDHGC5	4	-0.004255	1	0	*MYNN*	4	-0.062672	1	0
THRB	4	-0.004233	1	0	*ASCL2*	3	-0.062502	1	0
TMSB10	2	-0.004223	1	0	*XKR6*	4	-0.062492	1	0
RRH	4	-0.004164	1	0	*FAM171A1*	4	-0.062377	1	0
ZNF700	4	-0.004152	1	0	*MUC4*	4	-0.062294	1	0
PLCH1	2	-0.004147	1	0	*PPP1R18*	2	-0.062159	1	0
PDE1C	4	-0.004119	1	0	*MKKS*	4	-0.062054	1	0

PAX5	2	-0.004031	1	0	*MCOLN1*	2	-0.06198	1	0
TMEM236	4	-0.004003	1	0	*CCDC71L*	4	-0.061977	1	0
C3orf80	4	-0.003914	1	0	*TGFBR2*	4	-0.06196	1	0
NDST2	3	-0.003866	1	0	*PIP5K1B*	4	-0.061953	1	0
MSH6	4	-0.003857	1	0	*SLC22A18*	4	-0.061944	1	0
ALDH2	4	-0.00368	1	0	*SPATA21*	4	-0.061853	1	0
SLC16A5	4	-0.003621	1	0	*CHGB*	4	-0.0618	1	0
BST1	4	-0.003527	1	0	*HAND2*	3	-0.061775	1	0
ERN2	4	-0.0035	1	0	*XCR1*	4	-0.061676	1	0
RTL5	4	-0.003477	1	0	*CASZ1*	4	-0.061654	1	0
ANKRD42	3	-0.003434	1	0	*TFCP2L1*	4	-0.061614	1	0
NKRF	4	-0.003424	1	0	*HHIPL1*	3	-0.061411	1	0
GTF2E1	3	-0.003344	1	0	*ARL11*	3	-0.061298	1	0
DNAI2	3	-0.003302	1	0	*GNRH1*	4	-0.061262	1	0
CMBL	3	-0.003215	1	0	*HRH4*	4	-0.061136	1	0
TBX1	4	-0.003128	1	0	*XKRX*	4	-0.06112	1	0
CACNA1S	4	-0.003128	1	0	*STARD6*	4	-0.061067	1	0
PKN3	3	-0.003125	1	0	*CCDC157*	1	-0.060983	1	0
TPD52L2	4	-0.003097	1	0	*SOX7*	4	-0.060962	1	0
ATP6V1B1	4	-0.003085	1	0	*MAP10*	3	-0.060914	1	0
CSF2	4	-0.003066	1	0	*YY1AP1*	3	-0.060832	1	0
C11orf44	3	-0.002953	1	0	*NUDT11*	3	-0.060775	1	0
CCDC28A	4	-0.002952	1	0	*DPEP2NB*	2	-0.060724	1	0
UGGT2	3	-0.002921	1	0	*CNOT11*	4	-0.06062	1	0
TMEM61	3	-0.002898	1	0	*NR6A1*	4	-0.060611	1	0
IPO13	4	-0.002891	1	0	*TRABD*	4	-0.060586	1	0
XPOT	4	-0.002769	1	0	*PICALM*	4	-0.060579	1	0
WDR31	4	-0.002746	1	0	*YPEL2*	4	-0.060509	1	0
NPY4R2	1	-0.002743	1	0	*CNIH4*	4	-0.06031	1	0
RGL3	3	-0.002661	1	0	*MRI1*	3	-0.060262	1	0
NFATC2	3	-0.002542	1	0	*ABCA1*	3	-0.060213	1	0
STAC	4	-0.002525	1	0	*RNH1*	1	-0.060206	1	0
STX4	4	-0.002396	1	0	*GPR75*	4	-0.060205	1	0
DCDC2B	1	-0.002352	1	0	*GOLPH3L*	4	-0.060189	1	0
SLC25A33	2	-0.002332	1	0	*OXLD1*	2	-0.060141	1	0
MYADML2	4	-0.00229	1	0	*C9orf47*	4	-0.060133	1	0
TBX4	4	-0.002252	1	0	*PTGS2*	4	-0.060127	1	0
GNG11	3	-0.002248	1	0	*ZNF507*	4	-0.060119	1	0
TPR	4	-0.002248	1	0	*JAZF1*	1	-0.060096	1	0
RNF112	4	-0.002092	1	0	*ACVR1*	3	-0.060086	1	0
RNF150	4	-0.002053	1	0	*BAG1*	3	-0.06006	1	0
RBM23	4	-0.002052	1	0	*ORAI3*	4	-0.060047	1	0
GNAZ	3	-0.001972	1	0	*SLC34A3*	4	-0.059981	1	0
MITF	4	-0.001923	1	0	*C16orf74*	4	-0.059918	1	0
TMEM109	3	-0.001908	1	0	*OR56B4*	4	-0.059903	1	0
CLK1	4	-0.001907	1	0	*LUZP1*	4	-0.059706	1	0
ARHGEF11	4	-0.001905	1	0	*PTGES*	2	-0.059662	1	0
C2CD4C	3	-0.001792	1	0	*LINGO1*	4	-0.059647	1	0
DHRS7B	4	-0.001708	1	0	*LPXN*	4	-0.059352	1	0
TOM1L2	4	-0.001694	1	0	*NPR1*	4	-0.059315	1	0
GDPD4	4	-0.001635	1	0	*CNTRL*	4	-0.059305	1	0
TSPAN8	4	-0.001612	1	0	*SNAP23*	3	-0.059271	1	0
PLEKHB1	2	-0.001607	1	0	*TRIM29*	2	-0.05924	1	0
TMEM245	4	-0.001591	1	0	*LYSMD1*	4	-0.059238	1	0
FGD6	4	-0.001579	1	0	*AKR1B10*	3	-0.059207	1	0
GDAP1	4	-0.001561	1	0	*CEND1*	3	-0.059202	1	0
CACNA2D1	3	-0.001518	1	0	*NEUROG2*	3	-0.059153	1	0
ENHO	3	-0.001397	1	0	*GAS2L2*	4	-0.058944	1	0

ZNF485	4	-0.001387	1	0	*SMARCA4*	3	-0.058886	1	0
RCBTB2	4	-0.001377	1	0	*PUSL1*	4	-0.058843	1	0
AKAP6	4	-0.001377	1	0	*ADGRG1*	3	-0.058789	1	0
FARP1	3	-0.001359	1	0	*PKD2L2*	4	-0.058747	1	0
FNBP1	4	-0.001348	1	0	*YPEL4*	4	-0.058698	1	0
PTGDR2	4	-0.001308	1	0	*NPHS1*	4	-0.058695	1	0
GIGYF1	4	-0.001306	1	0	*PRG2*	4	-0.058693	1	0
MAP1S	2	-0.001261	1	0	*GOLGA6A*	1	-0.058626	1	0
OC10014459.	2	-0.00125	1	0	*ANGEL1*	3	-0.058617	1	0
NPM2	4	-0.001228	1	0	*GPR52*	4	-0.058606	1	0
HSPB8	4	-0.001178	1	0	*TMEM41A*	4	-0.058587	1	0
AR	4	-0.00117	1	0	*ZNHIT6*	4	-0.058542	1	0
TTC19	4	-0.001144	1	0	*CRYL1*	3	-0.058514	1	0
C19orf81	4	-0.001124	1	0	*UBE2L6*	4	-0.058488	1	0
PAPLN	4	-0.001107	1	0	*CACNA1G*	3	-0.058293	1	0
NR4A3	4	-0.001089	1	0	*MTHFSD*	4	-0.058229	1	0
B4GALT4	4	-0.00108	1	0	*CAPN10*	4	-0.058224	1	0
HECTD2	4	-0.001026	1	0	*STAT3*	3	-0.05817	1	0
SERPINA6	3	-9.94E-04	1	0	*SNX14*	4	-0.058164	1	0
TMEM209	4	-8.86E-04	1	0	*HCLS1*	4	-0.058161	1	0
MEGF9	4	-8.51E-04	1	0	*DAAM1*	4	-0.058122	1	0
SLC9B2	4	-7.10E-04	1	0	*SSTR2*	3	-0.05793	1	0
TCP10L	2	-6.71E-04	1	0	*IL12RB1*	4	-0.057871	1	0
EXD3	4	-6.57E-04	1	0	*MADD*	4	-0.057849	1	0
ARL14EPL	4	-6.43E-04	1	0	*PKIB*	3	-0.057825	1	0
SPG11	3	-6.40E-04	1	0	*PIK3R5*	4	-0.057729	1	0
RFT1	3	-6.22E-04	1	0	*TMEM200C*	4	-0.057725	1	0
MYL6	3	-6.21E-04	1	0	*C1QTNF1*	4	-0.057704	1	0
MAP7D3	4	-5.41E-04	1	0	*TLCD5*	4	-0.057702	1	0
PMP2	4	-4.79E-04	1	0	*ARNT*	3	-0.057695	1	0
ZNF440	4	-4.59E-04	1	0	*CSRP1*	4	-0.057687	1	0
PTCH2	3	-3.68E-04	1	0	*ZNF763*	3	-0.057638	1	0
ZNF578	4	-3.31E-04	1	0	*DAB1*	4	-0.057471	1	0
RHBG	4	-3.20E-04	1	0	*MSS51*	4	-0.057428	1	0
ZFR2	3	-2.99E-04	1	0	*SLC12A9*	3	-0.057389	1	0
TNFRSF25	4	-2.79E-04	1	0	*SIDT2*	4	-0.057303	1	0
RABGEF1	2	-2.16E-04	1	0	*DEDD*	4	-0.05723	1	0
FRG1	3	-1.96E-04	1	0	*MBIP*	4	-0.057206	1	0
ACACB	3	-1.82E-04	1	0	*BAIAP2*	4	-0.057072	1	0
PPP4R4	4	-1.70E-04	1	0	*ZNF335*	4	-0.057065	1	0
ZNF215	4	-1.34E-04	1	0	*IFNA21*	3	-0.056987	1	0
MDK	4	-1.03E-04	1	0	*NEU4*	4	-0.056921	1	0
AURKC	4	-7.89E-05	1	0	*TRH*	4	-0.056848	1	0
ATP6V1G2	1	-6.55E-05	1	0	*SRSF11*	4	-0.056834	1	0
PTBP3	4	-6.43E-05	1	0	*C21orf91*	4	-0.056804	1	0
SMIM13	3	-1.08E-05	1	0	*TMEM184A*	4	-0.056694	1	0
MEIS1	3	-5.76E-06	1	0	*RILPL1*	4	-0.056615	1	0
KLC2	2	1.02E-04	1	0	*STS*	2	-0.05661	1	0
ENPEP	3	1.42E-04	1	0	*CYP4F3*	4	-0.05656	1	0
MACO1	2	1.65E-04	1	0	*FBXL8*	4	-0.056553	1	0
ZBTB7C	4	1.73E-04	1	0	*PPP2R3B*	4	-0.056465	1	0
MAPK1	3	2.76E-04	1	0	*FSIP2*	4	-0.056453	1	0
ZNF316	4	3.57E-04	1	0	*ATG2B*	4	-0.056423	1	0
CGNL1	4	3.67E-04	1	0	*DMD*	3	-0.056422	1	0
OPRD1	4	3.71E-04	1	0	*PCDHAC1*	4	-0.05617	1	0
FAM13B	3	3.71E-04	1	0	*UBE2L3*	1	-0.056127	1	0
FAM228A	4	4.54E-04	1	0	*RLIM*	4	-0.056126	1	0
DOK5	3	4.80E-04	1	0	*PXMP4*	4	-0.056126	1	0

HAX1	4	5.40E-04	1	0	*FADS3*	4	-0.056099	1	0
SIPA1L1	4	5.73E-04	1	0	*FAM135A*	4	-0.0559	1	0
ITM2B	4	6.02E-04	1	0	*TEX15*	4	-0.055879	1	0
ZNF595	4	7.10E-04	1	0	*CDK10*	3	-0.055785	1	0
DESI1	4	7.94E-04	1	0	*GNPTAB*	4	-0.055762	1	0
UBE2S	2	8.04E-04	1	0	*OPRD1*	4	-0.055657	1	0
SPRY2	3	8.47E-04	1	0	*MAP3K7*	4	-0.055563	1	0
ZNF782	4	8.70E-04	1	0	*CRY1*	3	-0.055413	1	0
EDAR	4	8.87E-04	1	0	*ZNF707*	4	-0.055371	1	0
ATP9A	4	0.00106	1	0	*SEC61B*	3	-0.055346	1	0
CPZ	4	0.001074	1	0	*STK3*	3	-0.055342	1	0
TMIE	3	0.001183	1	0	*EFR3A*	4	-0.055319	1	0
ELMOD3	4	0.00129	1	0	*UBE2G1*	4	-0.0553	1	0
CASTOR1	4	0.001343	1	0	*SCN4B*	4	-0.055263	1	0
PACSIN1	4	0.001416	1	0	*FOXA2*	4	-0.055011	1	0
C15orf56	4	0.001419	1	0	*CASTOR1*	4	-0.054895	1	0
SLC35G6	3	0.001431	1	0	*ARAP3*	2	-0.054825	1	0
TOPORS	4	0.001486	1	0	*RALGAPB*	4	-0.054822	1	0
ERMP1	4	0.001506	1	0	*AKAP8L*	3	-0.054594	1	0
VDAC3	4	0.001551	1	0	*OBI1*	3	-0.054584	1	0
MRPL36	4	0.001552	1	0	*TRMT1*	3	-0.054465	1	0
PLA1A	3	0.001565	1	0	*MAGEC2*	4	-0.054432	1	0
HOXA6	2	0.001687	1	0	*LANCL1*	4	-0.0544	1	0
CD19	4	0.001816	1	0	*CNTLN*	3	-0.054393	1	0
SEC23IP	3	0.001823	1	0	*MTPN*	4	-0.054357	1	0
EAF1	3	0.001855	1	0	*FUCA2*	4	-0.054354	1	0
SLC23A3	4	0.001881	1	0	*AGPAT5*	4	-0.054241	1	0
PSME3IP1	4	0.001884	1	0	*C19orf81*	4	-0.054239	1	0
ICAM1	4	0.001929	1	0	*MDFI*	4	-0.054223	1	0
C1orf229	3	0.00203	1	0	*SP6*	3	-0.05421	1	0
MGAT5	3	0.002057	1	0	*FKRP*	2	-0.054093	1	0
GCC1	2	0.002247	1	0	*PTPRQ*	2	-0.054064	1	0
TRIM47	4	0.002291	1	0	*CKAP2L*	4	-0.054016	1	0
MAPT	4	0.002297	1	0	*GALK2*	3	-0.054012	1	0
MED9	4	0.00241	1	0	*PRKG2*	4	-0.054011	1	0
RND3	4	0.002505	1	0	*STX11*	4	-0.053973	1	0
PTBP1	4	0.002522	1	0	*LONRF1*	4	-0.053908	1	0
ADAMTS6	4	0.002536	1	0	*PALM2AKAP2*	11	-0.053883	1	0
BBS12	4	0.002629	1	0	*OR6A2*	4	-0.053801	1	0
PRR4	1	0.002701	1	0	*ATF6B*	4	-0.053753	1	0
CHST2	4	0.002803	1	0	*ISG20*	3	-0.053715	1	0
TBC1D4	4	0.002884	1	0	*IFI30*	2	-0.053601	1	0
SYNGAP1	4	0.002891	1	0	*ANKRD40*	4	-0.053528	1	0
CNRIP1	4	0.002893	1	0	*RGS18*	3	-0.053478	1	0
ANTXR2	4	0.002903	1	0	*LMO7DN*	4	-0.053466	1	0
NUBPL	3	0.002991	1	0	*PCDHB4*	3	-0.05345	1	0
SCMH1	4	0.003052	1	0	*NEIL2*	4	-0.053405	1	0
ZBTB32	2	0.003151	1	0	*ZSCAN29*	4	-0.053334	1	0
MSC	3	0.003187	1	0	*FOXN2*	3	-0.053318	1	0
SCGN	2	0.003214	1	0	*WT1*	4	-0.053247	1	0
PIP4K2A	3	0.003232	1	0	*ADAR*	4	-0.053245	1	0
SHISA8	4	0.003313	1	0	*ETV6*	4	-0.053173	1	0
CEBPG	2	0.003313	1	0	*B4GAT1*	4	-0.053166	1	0
SLC2A4	3	0.003317	1	0	*MGA*	2	-0.053075	1	0
CFAP52	4	0.003321	1	0	*C1orf21*	4	-0.053011	1	0
GAS2L2	4	0.003342	1	0	*ACSS2*	3	-0.05297	1	0
OBI1	3	0.003367	1	0	*FOLR1*	4	-0.052943	1	0
RTCA	4	0.00343	1	0	*PLAU*	4	-0.052848	1	0

LOC283710	4	0.003516	1	0	*H2AC12*	4	-0.052843	1	0
SELENBP1	4	0.003575	1	0	*C6orf226*	3	-0.052715	1	0
CFAP157	3	0.003645	1	0	*RABAC1*	3	-0.052649	1	0
FZD4	1	0.003669	1	0	*ALDH2*	4	-0.052575	1	0
CHGB	4	0.003679	1	0	*H3C12*	3	-0.052459	1	0
ZNF497	4	0.0037	1	0	*LRRC36*	2	-0.052456	1	0
C2orf66	4	0.003721	1	0	*TPD52L1*	4	-0.052424	1	0
KLKB1	4	0.003759	1	0	*C12orf49*	4	-0.052333	1	0
MPP1	3	0.003768	1	0	*TMEM161A*	3	-0.052221	1	0
ARHGAP26	4	0.003789	1	0	*TMEM68*	4	-0.05221	1	0
GOT2	3	0.0038	1	0	*SLC2A9*	3	-0.052206	1	0
MDH1B	4	0.003815	1	0	*CTSK*	4	-0.052192	1	0
ANKH	4	0.00385	1	0	*C9orf163*	4	-0.052172	1	0
APOO	4	0.003868	1	0	*FOXD4*	2	-0.052138	1	0
PLK2	4	0.003898	1	0	*BANK1*	4	-0.052077	1	0
PLEKHA7	4	0.003977	1	0	*PRELID2*	4	-0.05206	1	0
PITPNA	4	0.003994	1	0	*MLLT11*	2	-0.051958	1	0
FOXG1	4	0.004111	1	0	*DIPK2B*	4	-0.051954	1	0
CHD7	4	0.004138	1	0	*ZNF296*	4	-0.051869	1	0
CDK2AP2	3	0.004193	1	0	*CAPN9*	2	-0.051856	1	0
HMOX2	4	0.004273	1	0	*TMEM121B*	3	-0.051711	1	0
RNF215	4	0.00429	1	0	*CHI3L1*	4	-0.05171	1	0
CCDC7	4	0.004301	1	0	*GPR150*	4	-0.051532	1	0
KIF3C	4	0.004323	1	0	*IRF2*	3	-0.051527	1	0
OR56B4	4	0.00441	1	0	*ZC3H14*	4	-0.051496	1	0
CHAC1	1	0.004447	1	0	*SLC2A4RG*	2	-0.051476	1	0
CCDC173	2	0.004497	1	0	*ADAMTS1*	4	-0.051297	1	0
ELL3	3	0.004512	1	0	*BEND7*	3	-0.051274	1	0
SLC2A11	3	0.004527	1	0	*GOLPH3*	3	-0.05125	1	0
TFAP2A	3	0.004542	1	0	*FOXN1*	4	-0.051158	1	0
ING5	4	0.004602	1	0	*TXNDC2*	4	-0.05112	1	0
PLIN2	4	0.004645	1	0	*SUSD1*	4	-0.051038	1	0
LAX1	4	0.004671	1	0	*NOTCH2*	3	-0.051035	1	0
SUCLG1	4	0.004864	1	0	*GNRH2*	4	-0.051021	1	0
FAM187B	3	0.004895	1	0	*KDF1*	2	-0.050857	1	0
DHX8	3	0.004912	1	0	*ZNF160*	4	-0.050815	1	0
CTSH	4	0.004924	1	0	*TMPRSS13*	4	-0.050749	1	0
CRAMP1	3	0.005037	1	0	*PRKX*	2	-0.050702	1	0
CHP2	4	0.005042	1	0	*ZBED2*	3	-0.050658	1	0
ZNF384	4	0.005067	1	0	*SLC27A3*	4	-0.050596	1	0
NOS3	4	0.005161	1	0	*BTD*	3	-0.050564	1	0
APOBEC3B	4	0.005212	1	0	*USP17L7*	4	-0.050549	1	0
IGFBP2	3	0.005274	1	0	*HTATIP2*	3	-0.050504	1	0
RAD54L	4	0.005434	1	0	*XPO4*	4	-0.050464	1	0
RPF2	4	0.005522	1	0	*DOCK2*	4	-0.05044	1	0
HEMK1	4	0.005536	1	0	*HOXA4*	3	-0.050391	1	0
MAP3K12	3	0.005542	1	0	*TECPR2*	4	-0.050345	1	0
PCDHB8	4	0.005626	1	0	*SAMD4A*	2	-0.050327	1	0
LIF	3	0.005657	1	0	*SAP30L*	4	-0.050274	1	0
PLCG2	4	0.00569	1	0	*LDHAL6A*	3	-0.050135	1	0
LSM12	3	0.005698	1	0	*BCKDHB*	4	-0.050106	1	0
SMYD3	4	0.005774	1	0	*CDHR5*	3	-0.050096	1	0
RAI2	4	0.005813	1	0	*SRSF5*	4	-0.050042	1	0
MC1R	4	0.005847	1	0	*ZMYM1*	3	-0.049949	1	0
PJA2	4	0.005876	1	0	*GJA4*	4	-0.049937	1	0
CDC42EP4	4	0.006056	1	0	*LCMT2*	4	-0.049927	1	0
NUDT9	4	0.006155	1	0	*ERICH6B*	4	-0.049879	1	0
SLC35G5	4	0.006193	1	0	*SERPINF1*	4	-0.049854	1	0

FAM47C	3	0.006199	1	0	*UBE4A*	3	-0.049784	1	0
ARMC5	4	0.006263	1	0	*SLC19A2*	4	-0.049783	1	0
RNF44	4	0.006334	1	0	*CFAP299*	4	-0.049755	1	0
GPR37	2	0.006355	1	0	*SLC35A4*	3	-0.049739	1	0
ZHX2	4	0.006406	1	0	*LYPD3*	4	-0.049625	1	0
CXCL10	4	0.006442	1	0	*HNRNPH3*	4	-0.049611	1	0
IQUB	4	0.006567	1	0	*XRRA1*	2	-0.049601	1	0
ZNF343	3	0.006608	1	0	*PHC3*	4	-0.049489	1	0
NPY4R	2	0.006648	1	0	*ZFYVE1*	3	-0.049484	1	0
SLC30A2	4	0.006751	1	0	*RRBP1*	4	-0.049454	1	0
CEP104	3	0.006902	1	0	*SPTLC2*	1	-0.049426	1	0
ADAMTS7	3	0.006936	1	0	*SMIM29*	4	-0.04941	1	0
SYP	4	0.006954	1	0	*FRYL*	4	-0.0494	1	0
ZIC2	3	0.006957	1	0	*PGF*	1	-0.049311	1	0
P2RY6	3	0.00711	1	0	*PARP15*	2	-0.049278	1	0
KCNK15	1	0.007142	1	0	*TRPM1*	3	-0.049205	1	0
PLIN3	2	0.007255	1	0	*TPSG1*	2	-0.049205	1	0
MRPS28	4	0.007286	1	0	*FBXW10*	4	-0.049196	1	0
PIANP	3	0.007408	1	0	*TMEM161B*	4	-0.049186	1	0
GFAP	3	0.007443	1	0	*MPHOSPH6*	4	-0.049157	1	0
CT45A10	4	0.007486	1	0	*RIOX2*	3	-0.049087	1	0
ATRN	3	0.007649	1	0	*JCAD*	4	-0.049066	1	0
GNAI2	4	0.00771	1	0	*MLKL*	4	-0.048986	1	0
AMBRA1	4	0.007763	1	0	*FBXO6*	3	-0.04883	1	0
AGO1	3	0.007766	1	0	*LAPTM5*	3	-0.048793	1	0
FAM210B	4	0.007794	1	0	*KCNN4*	3	-0.048763	1	0
TAL2	4	0.007865	1	0	*PI15*	4	-0.048701	1	0
FZD3	4	0.007919	1	0	*PLCB2*	4	-0.048686	1	0
INTU	4	0.00793	1	0	*PEX7*	4	-0.048665	1	0
DCXR	4	0.007933	1	0	*NAV2*	4	-0.048619	1	0
BEND6	4	0.007943	1	0	*ZNF324*	4	-0.048597	1	0
ARHGDIB	4	0.008028	1	0	*OR1F1*	4	-0.048539	1	0
NUP210L	4	0.008038	1	0	*EPHA7*	3	-0.048371	1	0
GSDME	4	0.008103	1	0	*POMT1*	4	-0.048363	1	0
INSL3	1	0.008163	1	0	*PODXL*	4	-0.048349	1	0
DLX1	3	0.008268	1	0	*RIPK2*	4	-0.048288	1	0
PTX3	3	0.008275	1	0	*EFNB2*	1	-0.048162	1	0
GAL3ST4	3	0.008444	1	0	*ZNF544*	3	-0.048074	1	0
HSF2	4	0.008455	1	0	*EIPR1*	3	-0.048042	1	0
ZNF875	4	0.008487	1	0	*TMEM170B*	3	-0.047953	1	0
ITGAD	4	0.00849	1	0	*IGF1R*	4	-0.047907	1	0
DHX36	4	0.008522	1	0	*CLASP1*	4	-0.047881	1	0
KANSL1L	4	0.008831	1	0	*POLQ*	4	-0.047874	1	0
VANGL1	3	0.008876	1	0	*AP2A1*	3	-0.047778	1	0
ATP8B1	4	0.009057	1	0	*CTNNBIP1*	4	-0.047756	1	0
GPS1	3	0.009127	1	0	*ZDHHC23*	2	-0.047698	1	0
FANCL	3	0.009127	1	0	*SLAIN2*	4	-0.047639	1	0
BBC3	3	0.009146	1	0	*SAMD9L*	4	-0.047559	1	0
RIPK4	4	0.009207	1	0	*PCDHB3*	4	-0.047538	1	0
ZYG11B	4	0.009242	1	0	*YIPF5*	3	-0.047455	1	0
CREB3L4	4	0.009399	1	0	*CLDN10*	3	-0.047403	1	0
ZNF214	4	0.00941	1	0	*ADPRH*	4	-0.047362	1	0
EIF3M	4	0.009498	1	0	*OR4D11*	4	-0.047358	1	0
NOTCH2	3	0.009575	1	0	*FAT1*	4	-0.047106	1	0
ZNF664	4	0.009621	1	0	*SPIN2A*	1	-0.047013	1	0
PDPK1	1	0.009639	1	0	*SLC16A10*	2	-0.046961	1	0
ATP6V1H	4	0.009685	1	0	*ZBTB7C*	4	-0.046936	1	0
MYO1D	4	0.00976	1	0	*NAB2*	4	-0.046825	1	0

SLC39A4	4	0.009933	1	0	*CFD*	3	-0.046811	1	0
CCDC166	4	0.009941	1	0	*CYBA*	4	-0.046772	1	0
ENTPD2	3	0.009942	1	0	*SEMA4B*	4	-0.046704	1	0
PIN4	2	0.009981	1	0	*NYNRIN*	4	-0.046616	1	0
SEPTIN14	4	0.010088	1	0	*IFT46*	3	-0.046587	1	0
KY	3	0.010092	1	0	*HOMEZ*	3	-0.04652	1	0
TMEM52	3	0.010102	1	0	*NANOS1*	4	-0.046401	1	0
P4HA1	3	0.010167	1	0	*RACK1*	4	-0.046335	1	0
FES	4	0.01017	1	0	*KCTD1*	3	-0.046234	1	0
GAB3	4	0.010213	1	0	*TNFSF14*	4	-0.046191	1	0
PCDHAC2	4	0.010235	1	0	*ZNF34*	4	-0.046155	1	0
JAM3	4	0.010307	1	0	*EZH2*	3	-0.046092	1	0
STPG1	4	0.010353	1	0	*DCAF6*	4	-0.046089	1	0
COL26A1	4	0.010449	1	0	*TMCO4*	4	-0.046075	1	0
PRR12	4	0.010471	1	0	*DNAAF5*	4	-0.046064	1	0
ATG9A	4	0.010477	1	0	*RHNO1*	3	-0.04604	1	0
TMEM141	3	0.010624	1	0	*TPPP3*	4	-0.045917	1	0
RAB7A	3	0.010649	1	0	*SLC7A2*	2	-0.045844	1	0
ITGB3	4	0.010677	1	0	*HSFX2*	1	-0.045812	1	0
AK2	4	0.0107	1	0	*ZNF622*	4	-0.045594	1	0
MRC2	4	0.010757	1	0	*BABAM1*	3	-0.045515	1	0
VIPR2	4	0.01077	1	0	*DAGLA*	4	-0.045499	1	0
DIAPH3	4	0.010885	1	0	*STK4*	3	-0.04537	1	0
PLOD3	4	0.010928	1	0	*CD247*	4	-0.045145	1	0
HOXA9	4	0.010935	1	0	*MECP2*	4	-0.045081	1	0
CBX1	3	0.010951	1	0	*ALPK3*	4	-0.045027	1	0
SLC22A1	4	0.011023	1	0	*SERPINB1*	4	-0.045009	1	0
ASB14	3	0.011056	1	0	*CIDEC*	4	-0.045	1	0
PMFBP1	4	0.011148	1	0	*NNAT*	4	-0.044979	1	0
ZNF721	4	0.011193	1	0	*OMG*	4	-0.044841	1	0
IPO4	4	0.0112	1	0	*MAP4K2*	4	-0.044775	1	0
KRT17	4	0.011202	1	0	*ANKRD18A*	3	-0.044761	1	0
TEX44	4	0.011306	1	0	*DECR2*	4	-0.044661	1	0
GPATCH1	2	0.01135	1	0	*SGK1*	3	-0.044497	1	0
SNTB1	4	0.011373	1	0	*HNF4A*	4	-0.044474	1	0
MAMLD1	4	0.011433	1	0	*BCL7C*	4	-0.044424	1	0
TIGAR	3	0.011457	1	0	*SLC25A13*	3	-0.044367	1	0
C12orf77	1	0.011483	1	0	*SP100*	4	-0.04434	1	0
SEC14L1	4	0.011512	1	0	*ZNF57*	3	-0.044182	1	0
RHD	3	0.011586	1	0	*ZNF35*	4	-0.044091	1	0
TMEM80	2	0.011642	1	0	*ANPEP*	3	-0.04397	1	0
NUP42	3	0.011728	1	0	*DIO3*	4	-0.043962	1	0
CPSF6	4	0.011766	1	0	*ARL3*	4	-0.043864	1	0
H3-4	3	0.011818	1	0	*MAP7D2*	1	-0.043806	1	0
TRAPPC1	3	0.01183	1	0	*DSG2*	4	-0.043801	1	0
PFN2	4	0.011855	1	0	*KLHL18*	3	-0.043673	1	0
MARCHF6	1	0.011863	1	0	*GARNL3*	3	-0.043499	1	0
RAB44	4	0.011895	1	0	*KLHDC8A*	3	-0.04339	1	0
TMEM268	4	0.011896	1	0	*GVQW2*	1	-0.043359	1	0
VOPP1	2	0.011922	1	0	*RNASE10*	4	-0.043346	1	0
FLYWCH2	3	0.011923	1	0	*CCNB1IP1*	4	-0.043292	1	0
FAM92B	4	0.012022	1	0	*FIBCD1*	4	-0.043242	1	0
RIN1	4	0.012032	1	0	*CWF19L1*	4	-0.043232	1	0
ADAMTS5	3	0.012142	1	0	*SUCO*	4	-0.043199	1	0
CDK13	3	0.012153	1	0	*TMEM209*	4	-0.043155	1	0
TMEM198	3	0.012159	1	0	*LINC02694*	4	-0.043094	1	0
ACVR2A	4	0.012172	1	0	*GPR152*	4	-0.042863	1	0
EOMES	4	0.012259	1	0	*SUDS3*	4	-0.042849	1	0

BAG3	3	0.012314	1	0	*EFNA1*	3	-0.042789	1	0
MTFR2	4	0.012366	1	0	*PRSS55*	4	-0.042684	1	0
CACNB4	3	0.012496	1	0	*RPRD2*	4	-0.042665	1	0
EIF2A	2	0.012546	1	0	*LRRC26*	4	-0.042577	1	0
RRP1B	4	0.01255	1	0	*HDHD5*	4	-0.04251	1	0
PRDM8	3	0.012625	1	0	*PITX1*	4	-0.042454	1	0
KANSL2	3	0.012648	1	0	*BEX4*	4	-0.042366	1	0
AGPS	4	0.012746	1	0	*UIMC1*	3	-0.042348	1	0
PCP4	4	0.012748	1	0	*ZNF124*	4	-0.04234	1	0
MARCHF10	1	0.012828	1	0	*MXRA8*	3	-0.042324	1	0
PRUNE2	3	0.012855	1	0	*NLRP10*	4	-0.042186	1	0
AUTS2	2	0.012877	1	0	*EVC*	4	-0.042138	1	0
ZHX1	4	0.012893	1	0	*DUSP4*	4	-0.042135	1	0
BRD1	3	0.012909	1	0	*CCDC106*	4	-0.042059	1	0
AKAP5	4	0.012973	1	0	*ZCCHC17*	3	-0.042045	1	0
COIL	4	0.012982	1	0	*SHCBP1*	3	-0.042039	1	0
TGFB1	3	0.013072	1	0	*LDLRAD2*	1	-0.042022	1	0
PRKAR2B	4	0.01312	1	0	*ARFGEF2*	3	-0.041955	1	0
ABRAXAS2	4	0.013126	1	0	*FAR1*	4	-0.041937	1	0
MAP1B	4	0.013132	1	0	*TLR7*	4	-0.041879	1	0
MDM1	3	0.013187	1	0	*RDH5*	1	-0.041849	1	0
JPH2	4	0.013381	1	0	*SFI1*	4	-0.041825	1	0
TTPA	4	0.013453	1	0	*RFX3*	4	-0.041617	1	0
ARHGEF38	4	0.013454	1	0	*GRAMD1A*	3	-0.041604	1	0
PDE7B	3	0.013454	1	0	*FAAH*	2	-0.041447	1	0
WDSUB1	3	0.01346	1	0	*AK2*	4	-0.041446	1	0
TMEM33	4	0.013466	1	0	*CCN2*	4	-0.041425	1	0
TRMT1L	4	0.0135	1	0	*GJA5*	3	-0.041413	1	0
ACTR2	4	0.013593	1	0	*DDHD2*	4	-0.041327	1	0
ZNF784	4	0.013605	1	0	*CGREF1*	4	-0.041303	1	0
BBS9	4	0.013667	1	0	*PRKAA2*	3	-0.041277	1	0
LTB4R2	4	0.013699	1	0	*INTU*	4	-0.041204	1	0
WRN	3	0.013712	1	0	*APOL4*	4	-0.041198	1	0
RBMS3	4	0.013781	1	0	*GUCD1*	4	-0.041172	1	0
FYN	2	0.013888	1	0	*SLC10A3*	2	-0.041131	1	0
MTCH1	3	0.014053	1	0	*SPRYD7*	4	-0.041128	1	0
ANAPC1	4	0.014102	1	0	*PIK3C2G*	4	-0.041077	1	0
GPN1	4	0.014112	1	0	*ZNF174*	4	-0.041063	1	0
PTPRD	2	0.01418	1	0	*TNRC6A*	3	-0.04106	1	0
P4HTM	3	0.014328	1	0	*ZNF534*	3	-0.041023	1	0
ATP13A3	4	0.014333	1	0	*TINAGL1*	2	-0.041013	1	0
ZNF45	3	0.014343	1	0	*PTPRU*	2	-0.040985	1	0
HOMER3	3	0.014346	1	0	*TNFSF4*	4	-0.040935	1	0
OGDHL	4	0.014459	1	0	*MLF2*	3	-0.04093	1	0
TDRD5	4	0.014461	1	0	*CLEC4E*	3	-0.040872	1	0
ARHGAP1	4	0.014468	1	0	*CDKL5*	4	-0.040858	1	0
LPAR3	4	0.014608	1	0	*SNX7*	4	-0.040738	1	0
CERK	4	0.014694	1	0	*NR5A2*	4	-0.040701	1	0
CTSL	3	0.014694	1	0	*MYORG*	4	-0.04067	1	0
RETSAT	4	0.014798	1	0	*PGM1*	3	-0.040664	1	0
ZNF891	4	0.014823	1	0	*PYY*	3	-0.040581	1	0
GATAD2B	3	0.014852	1	0	*ZFAND6*	4	-0.040477	1	0
FAM228B	3	0.014969	1	0	*ABLIM1*	4	-0.040462	1	0
SYT8	3	0.014999	1	0	*PDGFRA*	2	-0.040408	1	0
INHBC	2	0.01502	1	0	*LGALS4*	4	-0.040266	1	0
RHOBTB3	4	0.015154	1	0	*PRRC2C*	4	-0.040233	1	0
ZKSCAN2	4	0.015384	1	0	*HDAC8*	4	-0.040173	1	0
RPP25	4	0.015398	1	0	*B3GAT3*	1	-0.040173	1	0

EDN3	4	0.015415	1	0	*ZNF14*	3	-0.040169	1	0
TNFRSF10B	4	0.015486	1	0	*PTPRN2*	3	-0.040167	1	0
SOCS3	3	0.015507	1	0	*EPS15*	4	-0.040108	1	0
TLCD3A	4	0.015655	1	0	*ITGAX*	4	-0.040095	1	0
STAG3	4	0.015683	1	0	*MINPP1*	4	-0.039815	1	0
EP400	4	0.015685	1	0	*ZNF599*	4	-0.039784	1	0
C8orf37	2	0.015699	1	0	*ZNF571*	3	-0.039753	1	0
B4GALNT4	4	0.015725	1	0	*ZNF713*	2	-0.039665	1	0
DAPK3	4	0.015725	1	0	*ZNF569*	4	-0.039636	1	0
AFP	3	0.015753	1	0	*SLC30A5*	3	-0.039633	1	0
KIF5B	4	0.015781	1	0	*PBDC1*	4	-0.039633	1	0
NPC1	2	0.015785	1	0	*PEX10*	4	-0.039532	1	0
KLRG1	2	0.015878	1	0	*ABRA*	4	-0.039367	1	0
PTGES	2	0.015882	1	0	*MMP21*	3	-0.039348	1	0
DNMT3A	3	0.015897	1	0	*SNX33*	3	-0.039337	1	0
MAP11	3	0.015934	1	0	*MTERF2*	4	-0.039285	1	0
NMT2	2	0.015994	1	0	*AKR1A1*	4	-0.039264	1	0
ARHGAP11A	1	0.01607	1	0	*MAGEA6*	1	-0.039191	1	0
TOR1B	4	0.016133	1	0	*KRT24*	4	-0.039179	1	0
TOR2A	4	0.016161	1	0	*ODF3L1*	2	-0.039128	1	0
VGLL3	4	0.016227	1	0	*GPR156*	3	-0.038861	1	0
PLA2G3	3	0.016366	1	0	*ZNF567*	4	-0.038795	1	0
MRPS17	3	0.016377	1	0	*EPCAM*	4	-0.038727	1	0
PRIM2	4	0.016381	1	0	*HOXA6*	2	-0.038667	1	0
KLHL9	4	0.016419	1	0	*KIAA0825*	4	-0.038614	1	0
C1QTNF2	3	0.016471	1	0	*TNNT3*	3	-0.038493	1	0
CILP	4	0.016511	1	0	*TMEM117*	4	-0.038479	1	0
MRPS7	4	0.016525	1	0	*TAAR9*	4	-0.038312	1	0
PRDX5	3	0.016569	1	0	*TARS3*	4	-0.038216	1	0
CACNB1	4	0.016598	1	0	*PTPRO*	3	-0.03821	1	0
H2BC17	4	0.016633	1	0	*CTRB2*	1	-0.038179	1	0
CCNL2	4	0.016708	1	0	*APOO*	4	-0.03813	1	0
FUOM	4	0.016708	1	0	*SLC5A6*	4	-0.037929	1	0
PIK3AP1	2	0.016733	1	0	*ALOX12B*	2	-0.03775	1	0
NKAPL	4	0.01683	1	0	*FRY*	4	-0.037728	1	0
ATXN7L1	4	0.016933	1	0	*EFCAB11*	4	-0.037673	1	0
EIF1B	3	0.016936	1	0	*GNG4*	4	-0.037588	1	0
CIPC	3	0.016941	1	0	*DPEP2*	4	-0.037554	1	0
USP21	3	0.016959	1	0	*SPIN3*	4	-0.037497	1	0
HOXD3	3	0.016984	1	0	*SRPX*	4	-0.037482	1	0
SRCIN1	3	0.016984	1	0	*DIRAS2*	3	-0.037468	1	0
SP7	3	0.017007	1	0	*EIF4ENIF1*	4	-0.037463	1	0
ZNF780B	4	0.017186	1	0	*PLCL1*	4	-0.037284	1	0
MYOT	4	0.017233	1	0	*EYS*	3	-0.037199	1	0
TST	4	0.017233	1	0	*SLC22A17*	4	-0.037117	1	0
RHOC	4	0.017268	1	0	*SPX*	4	-0.037083	1	0
DENND6B	4	0.017324	1	0	*COMMD8*	4	-0.037065	1	0
ZSCAN5A	4	0.017487	1	0	*MRS2*	3	-0.037044	1	0
NCAN	3	0.017541	1	0	*URM1*	3	-0.037036	1	0
HYDIN	4	0.017783	1	0	*CLIP4*	4	-0.036956	1	0
PLAC1	4	0.017785	1	0	*ERMARD*	4	-0.036943	1	0
TMX2	2	0.017854	1	0	*PEX11B*	3	-0.036894	1	0
CYSRT1	4	0.017904	1	0	*CXorf40B*	1	-0.036815	1	0
KCNV2	4	0.017933	1	0	*RBMS3*	4	-0.036795	1	0
KAZN	4	0.017949	1	0	*ZFP36L2*	4	-0.036718	1	0
TSR3	3	0.017984	1	0	*DPP9*	4	-0.036642	1	0
ACMSD	2	0.018009	1	0	*C1orf198*	4	-0.036541	1	0
TINAG	4	0.018116	1	0	*GMCL1*	3	-0.036535	1	0

ZW10	4	0.018128	1	0	*TMSB15A*	1	-0.036495	1	0
ZDHHC4	4	0.018135	1	0	*LHFPL5*	4	-0.036457	1	0
ATOH7	4	0.018596	1	0	*DLGAP4*	2	-0.036362	1	0
OAT	3	0.018707	1	0	*FAM131B*	3	-0.036351	1	0
COL5A1	4	0.018738	1	0	*ZNF280B*	3	-0.036339	1	0
SMARCC1	4	0.018782	1	0	*H4C9*	4	-0.036332	1	0
SPOCK2	4	0.018824	1	0	*LOC10013035*	1	-0.036325	1	0
ACAP3	4	0.018913	1	0	*ELAVL3*	4	-0.036169	1	0
FBXO16	4	0.019014	1	0	*NDST2*	3	-0.036109	1	0
MRTFB	4	0.019026	1	0	*VGLL2*	3	-0.036003	1	0
SPINK1	4	0.019053	1	0	*KDM3A*	4	-0.035982	1	0
MRAP2	4	0.019245	1	0	*FBXL12*	4	-0.035922	1	0
AKR7A3	4	0.019277	1	0	*NPM3*	4	-0.035916	1	0
SH3BGRL2	4	0.019321	1	0	*INPP5J*	4	-0.035882	1	0
NCKAP5L	4	0.019357	1	0	*FST*	4	-0.035827	1	0
CTHRC1	4	0.019364	1	0	*THPO*	4	-0.035721	1	0
GPBP1L1	3	0.019459	1	0	*APOBEC3H*	4	-0.035685	1	0
BRCC3	3	0.019475	1	0	*HNRNPCL2*	1	-0.035503	1	0
TSHZ1	4	0.019522	1	0	*PNO1*	2	-0.035389	1	0
PAFAH2	4	0.019594	1	0	*INSYN2B*	4	-0.035332	1	0
ZNF345	4	0.019599	1	0	*RAB44*	4	-0.035323	1	0
ABI1	4	0.01976	1	0	*SSX2IP*	3	-0.035319	1	0
SLC49A3	4	0.019784	1	0	*SLC9A1*	4	-0.035292	1	0
MAGEA3	2	0.019805	1	0	*RFX5*	3	-0.035212	1	0
CETP	4	0.019916	1	0	*ZCCHC24*	4	-0.035146	1	0
ANAPC7	4	0.019994	1	0	*SLC22A23*	3	-0.03511	1	0
SHANK2	1	0.020025	1	0	*ZNF846*	4	-0.035077	1	0
AMBP	3	0.020126	1	0	*CD96*	3	-0.034943	1	0
ARRDC1	3	0.020142	1	0	*SNRNP40*	4	-0.034876	1	0
GPR137B	3	0.020147	1	0	*LRRC58*	4	-0.034742	1	0
UBXN2B	4	0.020186	1	0	*NDNF*	3	-0.034718	1	0
TNFRSF17	4	0.020268	1	0	*ENAM*	4	-0.034618	1	0
FBXL5	3	0.020273	1	0	*TMEM192*	4	-0.034614	1	0
NKX2-1	3	0.020279	1	0	*ERICH1*	2	-0.034593	1	0
SLC5A3	4	0.020343	1	0	*HAVCR2*	3	-0.034558	1	0
RTN2	4	0.020378	1	0	*LRRC39*	4	-0.034495	1	0
SNAI2	4	0.020469	1	0	*KIF6*	2	-0.034428	1	0
KIF17	2	0.020552	1	0	*OSR2*	4	-0.034358	1	0
PTHLH	3	0.020658	1	0	*IKBKE*	4	-0.034264	1	0
GNGT1	4	0.020664	1	0	*RMND5B*	3	-0.034258	1	0
KRT1	4	0.020667	1	0	*PTPRD*	2	-0.034234	1	0
MMRN2	3	0.020671	1	0	*FKBP10*	4	-0.034214	1	0
PCDHA12	4	0.020806	1	0	*FSCN1*	4	-0.034118	1	0
CDK5R1	4	0.020836	1	0	*CACNA1B*	4	-0.034016	1	0
TAS2R50	4	0.020848	1	0	*GDF15*	4	-0.034009	1	0
ITGB6	4	0.020857	1	0	*ATRAID*	3	-0.033924	1	0
MRPS9	4	0.020875	1	0	*IPO5*	4	-0.033917	1	0
OSCAR	3	0.020879	1	0	*RTN2*	4	-0.033887	1	0
CWC27	3	0.020885	1	0	*SLC9A8*	4	-0.03387	1	0
CAST	4	0.020951	1	0	*LRP12*	4	-0.033869	1	0
SLFNL1	2	0.021266	1	0	*THAP7*	4	-0.033821	1	0
LYPD5	4	0.021289	1	0	*LAMA4*	2	-0.033772	1	0
SPAG17	4	0.021309	1	0	*COX7A2L*	4	-0.033755	1	0
LRRC75B	4	0.021347	1	0	*ATP1B3*	2	-0.033668	1	0
VWA5A	4	0.021446	1	0	*RGPD4*	2	-0.03365	1	0
NME7	3	0.021491	1	0	*KRTAP5-10*	3	-0.033641	1	0
STRBP	3	0.021509	1	0	*ZNF548*	2	-0.03362	1	0
SPHK1	3	0.021519	1	0	*LRRC37A3*	1	-0.03362	1	0

TG	3	0.021521	1	0	*SUPT3H*	3	-0.033457	1	0
TMEM251	4	0.021565	1	0	*PDE5A*	2	-0.033443	1	0
DMTF1	4	0.021652	1	0	*FGFR1*	3	-0.033347	1	0
TCEAL1	4	0.021699	1	0	*CREB3L2-AS1*	4	-0.033261	1	0
CCDC97	3	0.021752	1	0	*RAB9A*	2	-0.033259	1	0
ACAD8	4	0.021813	1	0	*SMLR1*	4	-0.033224	1	0
DPH2	3	0.021855	1	0	*ABHD16A*	4	-0.033142	1	0
TNPO2	4	0.021886	1	0	*KALRN*	4	-0.033118	1	0
SHC4	3	0.021891	1	0	*CALCOCO2*	2	-0.033087	1	0
AGPAT5	4	0.021919	1	0	*KCNK3*	2	-0.033029	1	0
LRIG1	3	0.021949	1	0	*MC5R*	4	-0.032912	1	0
UBXN11	3	0.021976	1	0	*ANKMY1*	4	-0.032906	1	0
PDGFRL	4	0.022047	1	0	*TCEAL1*	4	-0.032873	1	0
TMEFF1	1	0.022051	1	0	*MZF1*	3	-0.032854	1	0
WWC2	4	0.022158	1	0	*HUWE1*	3	-0.032799	1	0
LARP6	4	0.022232	1	0	*CYP51A1*	4	-0.032751	1	0
EXOC3	4	0.022379	1	0	*SERTAD2*	2	-0.032705	1	0
SUSD5	4	0.022402	1	0	*NAPB*	4	-0.032693	1	0
SMKR1	3	0.02241	1	0	*BTG3*	2	-0.032667	1	0
ETV7	3	0.022452	1	0	*DYRK1A*	2	-0.032654	1	0
SERGEF	4	0.022562	1	0	*NAA38*	3	-0.032493	1	0
CACNA2D4	4	0.022695	1	0	*VDR*	3	-0.032484	1	0
PARP11	3	0.022891	1	0	*PEAK1*	4	-0.032476	1	0
GDE1	2	0.022923	1	0	*TEKT5*	4	-0.032467	1	0
CXCL17	4	0.022978	1	0	*CLN8*	4	-0.032442	1	0
PRKAG1	4	0.02298	1	0	*FAM184B*	4	-0.032428	1	0
PCDHB1	4	0.023021	1	0	*HAPLN2*	4	-0.032342	1	0
DENND4A	4	0.023081	1	0	*TRAF3IP1*	3	-0.032329	1	0
SOWAHB	4	0.023101	1	0	*DEGS2*	4	-0.032324	1	0
PGLYRP1	4	0.023111	1	0	*OR52B2*	4	-0.03232	1	0
RNF165	4	0.023172	1	0	*ARHGAP9*	4	-0.032312	1	0
ADAM32	4	0.023182	1	0	*PCARE*	3	-0.032296	1	0
GRHL2	2	0.023248	1	0	*DAPK3*	4	-0.032229	1	0
PLEKHA4	2	0.023254	1	0	*SLC66A1L*	4	-0.032216	1	0
DYNC2LI1	4	0.023357	1	0	*LAT*	1	-0.032184	1	0
CAPN7	4	0.02337	1	0	*ERH*	4	-0.032167	1	0
DUOXA2	4	0.023433	1	0	*ZNF727*	4	-0.032074	1	0
RABEPK	4	0.023467	1	0	*ANKH*	4	-0.03203	1	0
KIF4A	3	0.023512	1	0	*TMED10*	4	-0.032008	1	0
SPEF2	3	0.023581	1	0	*GLIPR1L2*	2	-0.031978	1	0
BCL7B	3	0.023595	1	0	*WDR20*	4	-0.03183	1	0
BBOF1	3	0.023602	1	0	*MMP7*	4	-0.031813	1	0
CYB5RL	3	0.023659	1	0	*PMP2*	4	-0.031653	1	0
TMEM53	4	0.023811	1	0	*RGS22*	4	-0.031628	1	0
SRRM1	4	0.023912	1	0	*NMB*	2	-0.031515	1	0
SSX2IP	3	0.023912	1	0	*MTMR1*	2	-0.031472	1	0
ALG2	3	0.023916	1	0	*DAO*	3	-0.031447	1	0
LRRC8C	4	0.023933	1	0	*PDC*	2	-0.031445	1	0
ADAMTS14	4	0.023937	1	0	*FAM160A2*	4	-0.031366	1	0
GRK3	4	0.023943	1	0	*MDH1B*	4	-0.031345	1	0
RNF25	4	0.023993	1	0	*AKAP17A*	4	-0.031323	1	0
PRR19	4	0.024147	1	0	*PTAFR*	4	-0.031257	1	0
MTRNR2L8	1	0.024238	1	0	*THAP4*	4	-0.031246	1	0
USP42	3	0.024275	1	0	*LRAT*	4	-0.031178	1	0
DIPK1C	4	0.024357	1	0	*SYT1*	3	-0.031165	1	0
LRRIQ4	4	0.024371	1	0	*PKN1*	4	-0.031145	1	0
CD226	3	0.024376	1	0	*FGD2*	3	-0.031102	1	0
ALX4	4	0.024433	1	0	*HBG1*	1	-0.031082	1	0

SPCS1	3	0.024512	1	0	*SLC36A4*	3	-0.030998	1	0
DDC	4	0.024527	1	0	*PAPPA2*	4	-0.030911	1	0
PSME3	4	0.024613	1	0	*CD38*	4	-0.030904	1	0
CLU	4	0.024651	1	0	*TCL1A*	4	-0.03086	1	0
EVC	4	0.02476	1	0	*HAND1*	4	-0.030712	1	0
GBP6	2	0.024832	1	0	*SEC61A2*	4	-0.030684	1	0
GLB1L	4	0.024883	1	0	*CLCN5*	3	-0.030639	1	0
HDAC11	2	0.024993	1	0	*GCA*	4	-0.030635	1	0
MAML3	4	0.025005	1	0	*RSBN1*	3	-0.030632	1	0
PLAT	2	0.025011	1	0	*PLK2*	4	-0.03059	1	0
SSH2	4	0.025028	1	0	*RHOXF2B*	1	-0.030555	1	0
GLRX	4	0.025074	1	0	*CDKN2AIPNl*	4	-0.03055	1	0
CDCA4	3	0.025075	1	0	*SLC26A2*	4	-0.030353	1	0
NUTM2A	1	0.025086	1	0	*OTUD3*	4	-0.030269	1	0
TUBA1C	3	0.025124	1	0	*CCNA1*	3	-0.030193	1	0
VSTM4	4	0.025154	1	0	*DMTF1*	4	-0.030148	1	0
PXK	4	0.025171	1	0	*ZNF740*	4	-0.030116	1	0
SEC24D	4	0.025303	1	0	*ZNF131*	4	-0.030102	1	0
C9orf43	3	0.025336	1	0	*NPPB*	2	-0.030097	1	0
TRAPPC12	4	0.025379	1	0	*PEX3*	4	-0.0298	1	0
SCN3A	4	0.025402	1	0	*ZNF83*	3	-0.029786	1	0
BRMS1L	3	0.02549	1	0	*PLEK*	4	-0.029745	1	0
ARHGAP40	4	0.025528	1	0	*CDC20B*	3	-0.029702	1	0
KIF12	3	0.025626	1	0	*COG1*	4	-0.029581	1	0
DYNLT3	4	0.025647	1	0	*SH3TC1*	3	-0.029574	1	0
NSUN4	4	0.02565	1	0	*CD9*	3	-0.029508	1	0
ST3GAL3	4	0.025703	1	0	*IFI27L2*	3	-0.029447	1	0
DAXX	2	0.025838	1	0	*PHLDB3*	4	-0.029443	1	0
TMEM161B	4	0.025853	1	0	*MTMR8*	4	-0.029391	1	0
SMARCAD1	4	0.025854	1	0	*DRC7*	4	-0.029366	1	0
CLDN22	2	0.025888	1	0	*AGR2*	4	-0.029343	1	0
NUP58	4	0.025904	1	0	*TNNI2*	3	-0.029324	1	0
NUDT5	4	0.025928	1	0	*H4-16*	4	-0.029285	1	0
TMPRSS13	4	0.025934	1	0	*AQP4*	4	-0.029231	1	0
ACTA2	3	0.026001	1	0	*PCDHGB2*	4	-0.029174	1	0
KSR1	4	0.026012	1	0	*GBP3*	4	-0.029156	1	0
RGS1	3	0.026041	1	0	*RPGRIP1L*	3	-0.029142	1	0
CSMD1	3	0.026042	1	0	*GRIN2C*	2	-0.029047	1	0
TPM4	4	0.026059	1	0	*RPL36AP37*	1	-0.028972	1	0
ABRAXAS1	4	0.026103	1	0	*TMEM53*	4	-0.028961	1	0
APH1A	3	0.026212	1	0	*CGB7*	1	-0.028749	1	0
ADAM20	4	0.026309	1	0	*ZNF205*	4	-0.028683	1	0
IL15	4	0.026326	1	0	*DOCK3*	4	-0.028678	1	0
PATE4	4	0.026375	1	0	*MYO1F*	4	-0.028648	1	0
PPM1J	4	0.026399	1	0	*RTL10*	3	-0.028593	1	0
CREBZF	4	0.026495	1	0	*SPRN*	4	-0.028544	1	0
MYLK	3	0.026546	1	0	*KRTCAP2*	4	-0.028543	1	0
SRMS	4	0.026568	1	0	*ZCCHC4*	4	-0.028529	1	0
HSPB2	3	0.026623	1	0	*C1orf112*	4	-0.028506	1	0
TPST1	4	0.026672	1	0	*PQBP1*	4	-0.028366	1	0
SENP8	4	0.026685	1	0	*KIF1B*	3	-0.028345	1	0
PNLIPRP2	4	0.026745	1	0	*AP3B1*	3	-0.028326	1	0
ERF	4	0.027085	1	0	*AKAP3*	3	-0.028249	1	0
DUSP10	4	0.02718	1	0	*TMEM156*	3	-0.028231	1	0
CGGBP1	3	0.02728	1	0	*ACSS3*	3	-0.028122	1	0
ZNF284	3	0.027293	1	0	*UHRF1BP1*	4	-0.028056	1	0
SLC25A35	4	0.027337	1	0	*H4C4*	3	-0.028051	1	0
SPSB2	3	0.027348	1	0	*TMEM250*	3	-0.028019	1	0

CYBC1	3	0.027366	1	0	*COLCA2*	4	-0.028011	1	0
ADCK1	3	0.027415	1	0	*LAMB4*	3	-0.028007	1	0
DNAJC6	2	0.027524	1	0	*SUN2*	1	-0.027999	1	0
NRK	4	0.027536	1	0	*FAM155A*	3	-0.027882	1	0
PHYHD1	3	0.027563	1	0	*CHRM5*	4	-0.027875	1	0
IGSF3	4	0.027565	1	0	*TG*	3	-0.027871	1	0
EIF2AK2	4	0.027576	1	0	*RBBP6*	4	-0.027535	1	0
DEDD	4	0.027624	1	0	*CSF2RA*	3	-0.027483	1	0
CCDC114	3	0.027782	1	0	*MARCKS*	4	-0.027241	1	0
TMEM108	4	0.027878	1	0	*ALDH18A1*	3	-0.027116	1	0
NTMT1	4	0.02799	1	0	*CD2AP*	3	-0.027099	1	0
AEN	4	0.027991	1	0	*PTBP2*	4	-0.027042	1	0
ZNF878	4	0.028123	1	0	*ZCCHC2*	2	-0.027037	1	0
GPALPP1	4	0.028143	1	0	*CORO6*	4	-0.026922	1	0
KRT23	4	0.028165	1	0	*SESN2*	4	-0.026921	1	0
MRPL55	2	0.02821	1	0	*BZW2*	3	-0.02691	1	0
WDR90	3	0.028325	1	0	*PGRMC2*	2	-0.026897	1	0
SDCBP	3	0.028335	1	0	*ICA1*	2	-0.026596	1	0
CMIP	2	0.02834	1	0	*TRIL*	4	-0.02658	1	0
GOLT1B	4	0.028393	1	0	*KRTAP5-6*	3	-0.026552	1	0
LYST	3	0.028454	1	0	*DPY19L2*	4	-0.02655	1	0
MEGF6	3	0.028464	1	0	*ATF7IP*	4	-0.026527	1	0
TAPT1	4	0.028467	1	0	*TMEM238*	3	-0.026414	1	0
PIGL	4	0.028517	1	0	*ICMT*	4	-0.026342	1	0
IRAK1BP1	4	0.028545	1	0	*NANOS3*	4	-0.026288	1	0
TVP23A	4	0.028588	1	0	*BEST4*	4	-0.026273	1	0
DUOX1	4	0.028629	1	0	*PPP1R3F*	3	-0.026272	1	0
EFCAB13	3	0.028713	1	0	*ATP9B*	3	-0.026211	1	0
ENG	2	0.028758	1	0	*GAD1*	4	-0.026047	1	0
PCDHA6	4	0.028774	1	0	*GRTP1*	4	-0.026019	1	0
CPT2	4	0.028867	1	0	*ANGPTL3*	4	-0.025999	1	0
PARVG	4	0.028891	1	0	*RABGAP1*	4	-0.025958	1	0
EFHD2	4	0.028909	1	0	*C6orf62*	4	-0.025907	1	0
PTK2	4	0.028967	1	0	*ARIH2*	3	-0.025776	1	0
CYP2D6	2	0.029003	1	0	*C9orf152*	4	-0.025769	1	0
CSNK2A3	2	0.029053	1	0	*AKAP5*	4	-0.025756	1	0
BCL2L15	4	0.029072	1	0	*ITPR2*	4	-0.025687	1	0
ACTR3B	4	0.029146	1	0	*ARL13B*	4	-0.025655	1	0
LILRB1	3	0.029216	1	0	*MAP3K8*	3	-0.025618	1	0
SPTBN5	2	0.029285	1	0	*SCD5*	4	-0.025568	1	0
PARS2	4	0.029305	1	0	*VPS13C*	4	-0.025544	1	0
APOC1	2	0.029346	1	0	*SMAD2*	4	-0.025484	1	0
TNFAIP8	3	0.029358	1	0	*GCNT4*	3	-0.025483	1	0
ZNF619	3	0.029367	1	0	*MORN3*	2	-0.025437	1	0
HAAO	4	0.029368	1	0	*NAPSA*	4	-0.025354	1	0
STIM2	4	0.029462	1	0	*TTC36*	3	-0.025156	1	0
SF3B2	4	0.029539	1	0	*NMUR1*	3	-0.025078	1	0
RERG	3	0.029545	1	0	*PSMD10*	2	-0.024799	1	0
CAPN13	4	0.029552	1	0	*DSCR4*	2	-0.024745	1	0
TMSB15A	1	0.029585	1	0	*UTP18*	4	-0.024745	1	0
ABCC11	3	0.029665	1	0	*UBE2O*	3	-0.024729	1	0
SH3TC1	3	0.029668	1	0	*ANXA2*	4	-0.024666	1	0
PLD4	2	0.029708	1	0	*ZNF510*	4	-0.024552	1	0
VPS4A	4	0.029794	1	0	*SH2B3*	4	-0.024437	1	0
CDC42BPG	4	0.029886	1	0	*GOLGA6B*	1	-0.024386	1	0
MBTD1	4	0.029919	1	0	*SOX5*	4	-0.024368	1	0
TMEM241	4	0.029938	1	0	*PBRM1*	4	-0.024346	1	0
HNF4G	4	0.030048	1	0	*MYOZ1*	3	-0.024341	1	0

UBXN4	3	0.030065	1	0	*TSTD3*	4	-0.024323	1	0
FAM185A	2	0.03009	1	0	*KLK3*	3	-0.0243	1	0
C5orf22	4	0.030127	1	0	*ZNF550*	4	-0.024248	1	0
TLE4	4	0.030132	1	0	*PCDHGB7*	4	-0.02424	1	0
TMEM14EP	4	0.030175	1	0	*MRFAP1L1*	3	-0.024165	1	0
UBE2L6	4	0.030217	1	0	*MARCHF7*	3	-0.024098	1	0
MPP4	4	0.030237	1	0	*TPD52L2*	4	-0.024095	1	0
TAAR8	4	0.030261	1	0	*TMED6*	4	-0.024087	1	0
SLC26A7	4	0.030268	1	0	*HEBP1*	3	-0.024074	1	0
TBCK	2	0.030343	1	0	*DRD4*	4	-0.024066	1	0
DENND5B	4	0.030386	1	0	*PRR15L*	4	-0.023994	1	0
KL	4	0.030392	1	0	*IGSF9B*	4	-0.023951	1	0
ZDHHC17	4	0.030423	1	0	*HDAC2*	3	-0.02394	1	0
PIEZO1	4	0.03056	1	0	*CAPN15*	4	-0.023845	1	0
BAD	4	0.030616	1	0	*C2CD4B*	1	-0.023722	1	0
MRPS18B	4	0.030627	1	0	*UNC13D*	4	-0.023596	1	0
SMIM4	2	0.030659	1	0	*RPIA*	4	-0.023575	1	0
C11orf95	3	0.030738	1	0	*MT1X*	2	-0.023509	1	0
TPD52L1	4	0.030824	1	0	*KLHL36*	4	-0.023412	1	0
IL27	3	0.03084	1	0	*BFSP2*	4	-0.023255	1	0
ICAM2	4	0.030846	1	0	*H2AP*	4	-0.023243	1	0
RUNDC3B	4	0.030984	1	0	*BRINP1*	3	-0.023219	1	0
ALOX5	4	0.031003	1	0	*MSH2*	4	-0.023215	1	0
ARID3C	2	0.031142	1	0	*HEXB*	4	-0.023166	1	0
STAT2	4	0.031162	1	0	*BBC3*	3	-0.022981	1	0
CASR	4	0.031195	1	0	*SLC9C1*	4	-0.022973	1	0
RNF130	2	0.031203	1	0	*RHOU*	4	-0.02296	1	0
C16orf74	4	0.031269	1	0	*CFAP69*	3	-0.022951	1	0
TUBE1	4	0.031275	1	0	*IVD*	4	-0.02292	1	0
PNPT1	4	0.031332	1	0	*SSUH2*	4	-0.022732	1	0
CCDC113	3	0.031338	1	0	*UBE2E3*	4	-0.022534	1	0
YES1	4	0.031378	1	0	*BSND*	3	-0.022516	1	0
TAFA3	3	0.031382	1	0	*CEP89*	3	-0.022508	1	0
H2BC18	1	0.031394	1	0	*CYB5A*	4	-0.022503	1	0
NMBR	4	0.031469	1	0	*C9orf170*	4	-0.022492	1	0
PMM2	4	0.031535	1	0	*PITX2*	4	-0.022481	1	0
ARSG	4	0.031604	1	0	*TAF7L*	3	-0.022477	1	0
INTS13	4	0.031648	1	0	*NCOA6*	4	-0.022476	1	0
TP53I3	3	0.031715	1	0	*FBXO25*	3	-0.022462	1	0
ZFAND3	4	0.031731	1	0	*TCTN2*	4	-0.022448	1	0
ARHGEF6	4	0.031737	1	0	*ZBTB33*	4	-0.022441	1	0
LRRC14	3	0.031793	1	0	*SMCR8*	4	-0.022413	1	0
PLEK	4	0.031824	1	0	*ZNF497*	4	-0.022303	1	0
PTPRS	4	0.031845	1	0	*ZNF227*	3	-0.022276	1	0
TARBP2	4	0.031856	1	0	*GOLGA2*	4	-0.022273	1	0
HTR1D	2	0.031916	1	0	*DLG2*	4	-0.022063	1	0
SERPINA4	4	0.032081	1	0	*CHCHD10*	4	-0.022021	1	0
MTMR12	3	0.032086	1	0	*C1QTNF6*	4	-0.021968	1	0
INKA1	4	0.032129	1	0	*AFMID*	4	-0.02194	1	0
MYLPF	4	0.032169	1	0	*COX6B2*	4	-0.021924	1	0
AFDN	3	0.03218	1	0	*OPRL1*	3	-0.021916	1	0
VPS13D	3	0.032187	1	0	*MYADM*	4	-0.021905	1	0
LYG1	4	0.032374	1	0	*HNRNPDL*	4	-0.021888	1	0
GAS6	3	0.032376	1	0	*SCGB1A1*	4	-0.021827	1	0
ALDH1L1	3	0.032522	1	0	*RIOX1*	3	-0.021779	1	0
TSHZ3	4	0.032523	1	0	*ICAM2*	4	-0.021617	1	0
PCM1	4	0.032541	1	0	*ABHD14A*	2	-0.021552	1	0
CA13	4	0.032624	1	0	*ABCD1*	4	-0.02154	1	0

TASP1	3	0.032738	1	0	*TPST1*	4	-0.02139	1	0
LNPEP	3	0.032806	1	0	*VPS13D*	3	-0.021377	1	0
ATP2B2	3	0.032841	1	0	*SYCE1L*	4	-0.021376	1	0
DENND1B	4	0.032931	1	0	*RARS1*	4	-0.021325	1	0
CCL4	3	0.032935	1	0	*VPS37D*	2	-0.021314	1	0
ZNF222	3	0.03296	1	0	*ASPHD1*	4	-0.021231	1	0
TRIM28	4	0.033018	1	0	*CAPG*	4	-0.021207	1	0
KRT31	2	0.033061	1	0	*APOB*	4	-0.02115	1	0
UBR3	4	0.033096	1	0	*PUS10*	2	-0.021143	1	0
USP13	4	0.033103	1	0	*TTYH2*	4	-0.021061	1	0
ZNF300	4	0.033177	1	0	*TMEM14A*	3	-0.021006	1	0
SMAD5	3	0.033182	1	0	*BBS7*	3	-0.020923	1	0
NT5DC1	4	0.033199	1	0	*HNRNPUL1*	4	-0.020791	1	0
TMEM129	4	0.033233	1	0	*CLK4*	4	-0.020731	1	0
EIF4B	1	0.033283	1	0	*ULK2*	4	-0.020723	1	0
PPT1	4	0.033287	1	0	*DBF4B*	3	-0.020659	1	0
MICALL2	4	0.033325	1	0	*ANAPC13*	2	-0.020504	1	0
TTC39B	2	0.033465	1	0	*ENKD1*	4	-0.020488	1	0
PRKCG	4	0.033496	1	0	*BAIAP3*	4	-0.020472	1	0
NADK	3	0.033537	1	0	*DUSP3*	4	-0.020361	1	0
C9orf152	4	0.0336	1	0	*CD68*	3	-0.020314	1	0
HK1	4	0.033641	1	0	*HPGD*	4	-0.020281	1	0
EXPH5	4	0.033717	1	0	*CYTH1*	4	-0.020263	1	0
LPGAT1	3	0.033843	1	0	*RAPGEF1*	4	-0.020211	1	0
ACOT6	4	0.033906	1	0	*IPO8*	3	-0.020162	1	0
CYREN	2	0.033907	1	0	*HDDC3*	4	-0.020125	1	0
MRPS25	4	0.034021	1	0	*MAK*	3	-0.020105	1	0
DDI2	3	0.034053	1	0	*HSF2BP*	2	-0.01998	1	0
MMP9	4	0.034231	1	0	*GPR45*	4	-0.019951	1	0
APBB1IP	3	0.034437	1	0	*ZNF773*	3	-0.019938	1	0
DNAL4	3	0.034438	1	0	*UGP2*	2	-0.01973	1	0
FER	4	0.034443	1	0	*ZNF267*	2	-0.019718	1	0
OGFR	3	0.034446	1	0	*PARP12*	1	-0.019657	1	0
EBLN2	4	0.03467	1	0	*RASEF*	4	-0.019578	1	0
THAP1	4	0.034771	1	0	*ZNF621*	4	-0.019576	1	0
FUS	4	0.034801	1	0	*KIF17*	2	-0.019552	1	0
LRRC57	4	0.034813	1	0	*DDIAS*	4	-0.019489	1	0
PURA	2	0.034904	1	0	*PCDHA6*	4	-0.01948	1	0
TMEM74B	4	0.034922	1	0	*H4C1*	4	-0.019436	1	0
DOCK2	4	0.034968	1	0	*CECR2*	4	-0.019435	1	0
PROSER1	4	0.03498	1	0	*SMTNL1*	4	-0.019392	1	0
JAML	4	0.035071	1	0	*FBXO16*	4	-0.019359	1	0
DNAL1	4	0.035088	1	0	*LPIN2*	4	-0.019311	1	0
H2BW1	4	0.035125	1	0	*COL6A2*	3	-0.019242	1	0
CBR4	3	0.03513	1	0	*RPAP3*	2	-0.019202	1	0
CCDC8	1	0.035139	1	0	*GORAB*	4	-0.019104	1	0
CHAD	3	0.035312	1	0	*MAP3K2*	2	-0.019044	1	0
TBC1D16	4	0.035367	1	0	*ZFHX3*	8	-0.019042	1	0
MFSD4B	4	0.035391	1	0	*BRD9*	4	-0.019031	1	0
NDST1	3	0.035438	1	0	*TNNC1*	4	-0.019001	1	0
MAP3K2	2	0.035439	1	0	*TCP11*	4	-0.018978	1	0
RALGAPB	4	0.035451	1	0	*ASIC4*	4	-0.018891	1	0
SHE	4	0.035586	1	0	*PRCP*	3	-0.018884	1	0
CNEP1R1	3	0.035673	1	0	*DPPA2*	3	-0.018863	1	0
TMEM200B	3	0.035763	1	0	*ACVR2B*	3	-0.018802	1	0
C1QL3	3	0.035773	1	0	*CASP5*	4	-0.018738	1	0
NRN1	4	0.0358	1	0	*CUL7*	4	-0.018653	1	0
FMNL2	2	0.035806	1	0	*ZSCAN12*	4	-0.018651	1	0

SOX18	3	0.035854	1	0	*ZNF730*	4	-0.018634	1	0
ARHGAP44	4	0.035886	1	0	*WSB2*	4	-0.018607	1	0
GDPD2	3	0.035966	1	0	*ZBTB44*	4	-0.018576	1	0
ADGRF3	3	0.036009	1	0	*HNRNPCL1*	2	-0.018567	1	0
FAM166B	3	0.036015	1	0	*NUTM2F*	3	-0.018566	1	0
ZDHHC12	4	0.036038	1	0	*PALD1*	4	-0.018564	1	0
YIPF5	3	0.03614	1	0	*TMEM132E*	3	-0.018402	1	0
ST3GAL1	3	0.036153	1	0	*RDH13*	4	-0.018395	1	0
WNT7B	4	0.036161	1	0	*GPR183*	4	-0.018387	1	0
CD24	3	0.036162	1	0	*MCM8*	4	-0.018338	1	0
NR0B2	4	0.036336	1	0	*NKX2-8*	4	-0.018317	1	0
IGDCC4	3	0.036352	1	0	*BBS10*	4	-0.018278	1	0
TBX19	4	0.036369	1	0	*PCOLCE2*	3	-0.018234	1	0
HSF1	3	0.036432	1	0	*PNP*	3	-0.018201	1	0
EFCAB7	4	0.036447	1	0	*OSTN*	3	-0.018199	1	0
MPRIP	3	0.036456	1	0	*SLC39A5*	4	-0.018197	1	0
RRNAD1	3	0.036502	1	0	*MB*	3	-0.018179	1	0
LRRC37A3	1	0.036553	1	0	*ARL15*	4	-0.018123	1	0
HSPB6	2	0.036583	1	0	*PLIN3*	2	-0.018015	1	0
MOB1A	4	0.036593	1	0	*HOMER2*	3	-0.017879	1	0
ZNF593	4	0.03665	1	0	*SPAG16-DT*	4	-0.017659	1	0
SYNC	4	0.036659	1	0	*ERN1*	3	-0.01762	1	0
SMAGP	4	0.036685	1	0	*TAP1*	4	-0.017596	1	0
ACSBG1	4	0.03679	1	0	*KCNMA1*	3	-0.017536	1	0
DPYSL5	4	0.036828	1	0	*PIGG*	3	-0.017503	1	0
EIF4A2	3	0.036901	1	0	*MED17*	2	-0.017501	1	0
TGFBR2	4	0.036935	1	0	*C12orf66*	3	-0.017438	1	0
NDFIP1	4	0.036947	1	0	*ANK2*	4	-0.017428	1	0
WNT3A	4	0.037138	1	0	*SOWAHD*	3	-0.017399	1	0
POLRMT	3	0.037234	1	0	*CCDC125*	4	-0.017308	1	0
CD63	4	0.037239	1	0	*RERE*	1	-0.017263	1	0
ELP2	4	0.037277	1	0	*CASQ1*	2	-0.017106	1	0
CMTM7	3	0.037322	1	0	*ATP9A*	4	-0.017092	1	0
DENND1A	4	0.037351	1	0	*BCL6B*	3	-0.017053	1	0
SHISA4	4	0.037383	1	0	*CC2D2B*	5	-0.017019	1	0
SIGLEC15	2	0.03754	1	0	*PROSER3*	3	-0.016966	1	0
VEZF1	3	0.037559	1	0	*LDOC1*	3	-0.016782	1	0
GNB3	4	0.037633	1	0	*SLC7A6*	4	-0.016632	1	0
CES1	3	0.037706	1	0	*TMEM232*	4	-0.016625	1	0
C1orf61	4	0.037722	1	0	*WDR45*	4	-0.016619	1	0
PCNX3	4	0.037731	1	0	*EMX1*	4	-0.016595	1	0
CDHR3	4	0.037739	1	0	*CHCHD6*	4	-0.016572	1	0
NFE4	4	0.03777	1	0	*ZNF248*	4	-0.016521	1	0
ARRDC2	4	0.037818	1	0	*EMILIN3*	4	-0.016437	1	0
UBE4A	3	0.03785	1	0	*KCNS1*	4	-0.016222	1	0
ETNK2	4	0.037989	1	0	*DOK5*	3	-0.016212	1	0
NDRG1	4	0.038013	1	0	*CALD1*	4	-0.015887	1	0
CEMP1	3	0.038102	1	0	*EED*	3	-0.01587	1	0
PCDHGA8	4	0.038135	1	0	*DIAPH3*	4	-0.01583	1	0
ZNF260	3	0.038139	1	0	*RDH16*	4	-0.015744	1	0
JOSD2	4	0.03817	1	0	*PRPF40B*	3	-0.015553	1	0
HIKESHI	3	0.038241	1	0	*B9D2*	4	-0.015513	1	0
ZNF57	3	0.038291	1	0	*WDR86*	2	-0.015471	1	0
MEIG1	4	0.038328	1	0	*CYP39A1*	4	-0.015467	1	0
FARS2	3	0.038353	1	0	*ZNF668*	4	-0.015423	1	0
ANKRD26	4	0.038412	1	0	*ZDHHC1*	4	-0.015419	1	0
RNF7	4	0.038413	1	0	*HMOX2*	4	-0.015385	1	0
COMTD1	4	0.038555	1	0	*APELA*	4	-0.015314	1	0

SS18	4	0.038585	1	0	*TAS1R3*	4	-0.01529	1	0
PTGDR	3	0.038591	1	0	*VBP1*	4	-0.015287	1	0
AKNA	4	0.03876	1	0	*PKN2*	4	-0.015196	1	0
PREX1	4	0.038837	1	0	*CCDC155*	3	-0.015122	1	0
MTMR10	4	0.038957	1	0	*HJV*	4	-0.015103	1	0
ADAMTS2	4	0.038976	1	0	*MLPH*	4	-0.01509	1	0
POTEE	2	0.039003	1	0	*KIAA0753*	3	-0.015068	1	0
R3HDM2	3	0.039055	1	0	*SLC34A2*	4	-0.015067	1	0
C3orf52	4	0.03907	1	0	*AHSG*	3	-0.015022	1	0
ANK3	4	0.039127	1	0	*PGAP6*	2	-0.014991	1	0
NUGGC	3	0.039209	1	0	*FTSJ1*	2	-0.014942	1	0
ARHGEF33	4	0.039245	1	0	*NPHP1*	4	-0.01493	1	0
FBXW4	4	0.03926	1	0	*NUBP1*	3	-0.014863	1	0
PRTG	4	0.039419	1	0	*H1-5*	3	-0.014778	1	0
HRAS	4	0.039488	1	0	*TMEM72*	2	-0.014765	1	0
MX1	3	0.039495	1	0	*ZKSCAN1*	4	-0.014735	1	0
CEP55	4	0.039509	1	0	*NAIP*	2	-0.014716	1	0
SIRT7	3	0.039529	1	0	*SCUBE1*	3	-0.014689	1	0
CNOT7	3	0.039899	1	0	*TSPAN8*	4	-0.014646	1	0
ZNF844	4	0.0399	1	0	*CARD6*	4	-0.014633	1	0
GON7	4	0.039927	1	0	*KCNE4*	4	-0.014628	1	0
SMIM20	4	0.039953	1	0	*EIF2B4*	4	-0.0146	1	0
NEK4	4	0.040026	1	0	*NRCAM*	4	-0.01459	1	0
SIK3	4	0.040109	1	0	*IL18R1*	4	-0.014584	1	0
SDC1	4	0.040114	1	0	*MATK*	4	-0.014508	1	0
FAM221A	3	0.040195	1	0	*TUBB1*	4	-0.014453	1	0
YWHAH	3	0.040228	1	0	*OR5K2*	3	-0.014383	1	0
DTX4	4	0.04029	1	0	*UMODL1*	4	-0.014311	1	0
PRLR	3	0.040396	1	0	*TMEM204*	3	-0.014303	1	0
ICAM3	3	0.040459	1	0	*AADAT*	2	-0.014258	1	0
TP53I11	2	0.040696	1	0	*KLHL38*	4	-0.01422	1	0
KCTD10	4	0.0407	1	0	*BCL2L15*	4	-0.014217	1	0
DLL4	4	0.040764	1	0	*SPAG9*	4	-0.014215	1	0
AACS	4	0.040765	1	0	*SLC15A1*	3	-0.014198	1	0
PPEF1	4	0.040766	1	0	*FAF1*	4	-0.014165	1	0
GNRH1	4	0.040843	1	0	*ACAP2*	4	-0.014145	1	0
IFI44L	4	0.040858	1	0	*RHAG*	4	-0.014107	1	0
IK	4	0.040943	1	0	*DISP3*	2	-0.014097	1	0
TTC36	3	0.041095	1	0	*ENAH*	3	-0.014081	1	0
ITIH4	4	0.041169	1	0	*CD47*	4	-0.013962	1	0
FNDC9	3	0.041188	1	0	*C3orf80*	4	-0.0139	1	0
REG4	4	0.041252	1	0	*SLC51A*	4	-0.013882	1	0
RECQL5	1	0.041275	1	0	*UBE2D1*	4	-0.01387	1	0
TMEM184A	4	0.041275	1	0	*DRAM2*	4	-0.013745	1	0
LCP1	4	0.041379	1	0	*ZNF618*	3	-0.013694	1	0
CDCA8	4	0.041394	1	0	*ZNF835*	3	-0.013658	1	0
CDKN1B	4	0.041414	1	0	*KLHL9*	4	-0.013556	1	0
DHODH	4	0.041492	1	0	*ERCC6L*	3	-0.013548	1	0
TNFRSF10A	4	0.041492	1	0	*SEC11C*	3	-0.013545	1	0
PACSIN3	4	0.041537	1	0	*GOLGA3*	4	-0.013538	1	0
CD300LG	4	0.041543	1	0	*FRMD5*	2	-0.013414	1	0
STEAP2	3	0.041743	1	0	*MIER3*	4	-0.013361	1	0
EPHB3	4	0.041802	1	0	*ZNF433*	2	-0.01326	1	0
PBX1	3	0.041806	1	0	*SH2D3A*	4	-0.013208	1	0
CHST4	4	0.041819	1	0	*CCDC24*	3	-0.013127	1	0
TRIM23	4	0.041838	1	0	*CSMD1*	3	-0.013077	1	0
CAPN12	4	0.041849	1	0	*GPAT2*	1	-0.012948	1	0
CARM1	4	0.04188	1	0	*RARB*	3	-0.012896	1	0

ZDHHC21	3	0.041903	1	0	*PARP2*	4	-0.012894	1	0
ZNF22	2	0.041927	1	0	*BRMS1L*	3	-0.012893	1	0
IGSF23	2	0.041936	1	0	*PLVAP*	4	-0.012842	1	0
TMA7	3	0.041989	1	0	*RAB30*	4	-0.012826	1	0
LAMP2	4	0.04206	1	0	*ANKDD1A*	4	-0.01278	1	0
LSM5	4	0.042106	1	0	*PIPOX*	3	-0.012715	1	0
CFL2	4	0.042109	1	0	*SEMA3B*	4	-0.01264	1	0
QSOX2	4	0.042159	1	0	*SPNS1*	4	-0.012422	1	0
ZNF37A	3	0.042171	1	0	*FANCB*	4	-0.012403	1	0
CNIH4	4	0.042209	1	0	*PLCXD1*	3	-0.012197	1	0
ATG4B	4	0.04234	1	0	*RND3*	4	-0.012192	1	0
KBTBD12	4	0.042396	1	0	*PDGFC*	3	-0.012141	1	0
LY6D	3	0.042409	1	0	*PIP5K1C*	4	-0.012123	1	0
RAB41	4	0.042441	1	0	*HECTD2*	4	-0.012093	1	0
TNFRSF21	4	0.042501	1	0	*KRT81*	2	-0.01206	1	0
TAS2R20	4	0.042639	1	0	*PRH2*	3	-0.011991	1	0
FGG	4	0.042642	1	0	*C22orf15*	4	-0.011987	1	0
TRABD	4	0.042751	1	0	*KCNJ9*	4	-0.011952	1	0
WHRN	2	0.042786	1	0	*PSMD9*	4	-0.011938	1	0
EFNB2	1	0.042798	1	0	*HCST*	2	-0.011793	1	0
ASCC3	4	0.042887	1	0	*CYP4A22*	2	-0.011692	1	0
KIAA0355	4	0.042993	1	0	*CLTB*	4	-0.011676	1	0
DTX2	4	0.043055	1	0	*ADAMTS15*	4	-0.011592	1	0
TRPS1	1	0.043159	1	0	*KIF2C*	4	-0.011577	1	0
SLC7A7	2	0.043345	1	0	*FOLH1*	2	-0.011565	1	0
MAL	4	0.043349	1	0	*DIXDC1*	4	-0.011548	1	0
ZNF444	4	0.043403	1	0	*FAN1*	4	-0.011453	1	0
MRTFA	4	0.043575	1	0	*COPRS*	4	-0.011402	1	0
WNK2	4	0.043598	1	0	*NXPE2*	3	-0.011363	1	0
CHCHD7	4	0.04361	1	0	*COPS7B*	4	-0.011324	1	0
ACTA1	1	0.043636	1	0	*USP53*	3	-0.011307	1	0
MRPL18	3	0.043669	1	0	*MID1*	4	-0.011279	1	0
CAPN9	2	0.043704	1	0	*YWHAQ*	3	-0.011212	1	0
LGALS3BP	4	0.043864	1	0	*KATNAL2*	3	-0.011182	1	0
TMEM167A	1	0.04387	1	0	*ANGPT2*	4	-0.011175	1	0
OC10050584	3	0.043875	1	0	*FNBP1L*	3	-0.011136	1	0
TMEM11	4	0.043883	1	0	*RAB1B*	1	-0.011094	1	0
ZNF480	4	0.043954	1	0	*IQGAP2*	3	-0.011076	1	0
MBOAT7	3	0.043993	1	0	*CDK11A*	4	-0.011076	1	0
PSTPIP2	4	0.044019	1	0	*PPM1G*	3	-0.011068	1	0
KLC4	3	0.04404	1	0	*CPN1*	4	-0.011046	1	0
ARL6IP1	3	0.044088	1	0	*ITIH3*	4	-0.010953	1	0
TNFRSF19	4	0.044172	1	0	*ASAH2*	3	-0.010903	1	0
ZNF341	3	0.044286	1	0	*TRABD2A*	3	-0.010877	1	0
ACOT9	4	0.044337	1	0	*DHX36*	4	-0.010734	1	0
MRPL4	3	0.044459	1	0	*ALDH8A1*	4	-0.010515	1	0
RTL9	3	0.044517	1	0	*PHACTR3*	4	-0.010425	1	0
TACC1	3	0.044568	1	0	*MAST3*	4	-0.010424	1	0
TMEM147	3	0.044587	1	0	*FAM166B*	3	-0.010423	1	0
ZNF276	2	0.044591	1	0	*CCL3*	4	-0.010391	1	0
ISCA1	2	0.044605	1	0	*HLA-B*	4	-0.010386	1	0
LTK	4	0.044636	1	0	*ACPP*	4	-0.010361	1	0
KLHL36	4	0.044661	1	0	*SHFL*	4	-0.010355	1	0
VILL	3	0.044669	1	0	*ZFYVE27*	2	-0.010344	1	0
MIB2	3	0.044693	1	0	*USP15*	3	-0.010338	1	0
ADAMTS17	4	0.044719	1	0	*PRDX6*	4	-0.010212	1	0
SPATA33	4	0.044813	1	0	*GPR107*	3	-0.01013	1	0
SLC39A7	4	0.044824	1	0	*MAGI2*	4	-0.010057	1	0

ADCY3	3	0.044894	1	0	*RTL5*	4	-0.010039	1	0
PIP5K1B	4	0.044904	1	0	*ANP32E*	3	-0.010025	1	0
SLC22A13	4	0.044956	1	0	*SYPL1*	4	-0.010017	1	0
BLOC1S6	4	0.044965	1	0	*GPR137B*	3	-0.009888	1	0
ATL3	4	0.044973	1	0	*NIT1*	4	-0.009872	1	0
NMNAT2	4	0.044981	1	0	*GPR4*	3	-0.00974	1	0
ZNF584	4	0.044987	1	0	*TENT2*	2	-0.009664	1	0
NIPAL3	3	0.04499	1	0	*CPB2*	4	-0.009656	1	0
C6orf201	4	0.045072	1	0	*C9orf85*	4	-0.009539	1	0
FST	4	0.045136	1	0	*REP15*	4	-0.009522	1	0
CMKLR1	3	0.045154	1	0	*MED10*	4	-0.00949	1	0
IZUMO4	4	0.045163	1	0	*EPHA8*	4	-0.009478	1	0
IFIT2	4	0.045191	1	0	*PRKAG2*	4	-0.009316	1	0
PTPRZ1	3	0.045337	1	0	*LRWD1*	4	-0.009249	1	0
TACC3	4	0.045452	1	0	*CNFN*	4	-0.009219	1	0
AREL1	4	0.045481	1	0	*IRF2BPL*	4	-0.009197	1	0
ZNF138	2	0.045486	1	0	*EMID1*	2	-0.009087	1	0
THAP3	4	0.045542	1	0	*GJA3*	3	-0.009037	1	0
NR2E1	4	0.045567	1	0	*CBFA2T2*	4	-0.00898	1	0
WFDC10A	3	0.045663	1	0	*ERICH2*	4	-0.008934	1	0
RNF40	3	0.04569	1	0	*NMRK1*	4	-0.008876	1	0
JMJD8	4	0.045713	1	0	*SEC63*	4	-0.008867	1	0
TLCD5	4	0.045787	1	0	*LRRC25*	3	-0.008804	1	0
NFATC3	4	0.045792	1	0	*IFI44L*	4	-0.008762	1	0
CCDC167	2	0.045956	1	0	*ST3GAL5*	4	-0.0087	1	0
SCLT1	4	0.045958	1	0	*CCDC110*	4	-0.008636	1	0
PROB1	4	0.04597	1	0	*FUT1*	3	-0.008533	1	0
MED14OS	4	0.045989	1	0	*PIDD1*	3	-0.008533	1	0
FAM71E2	4	0.046054	1	0	*DNAJC21*	3	-0.008497	1	0
PEMT	3	0.046086	1	0	*OAS3*	4	-0.008491	1	0
PPP2R5A	4	0.046094	1	0	*FOXP3*	4	-0.00848	1	0
DNAH9	4	0.046119	1	0	*GPR132*	3	-0.008465	1	0
NNMT	4	0.046223	1	0	*RORC*	4	-0.008339	1	0
MYORG	4	0.04631	1	0	*F7*	4	-0.008317	1	0
CDCA7	2	0.046372	1	0	*TULP2*	2	-0.008297	1	0
CDH2	4	0.046375	1	0	*TOM1*	3	-0.00817	1	0
PPP2R1B	3	0.046439	1	0	*RBFOX2*	3	-0.008151	1	0
RPUSD4	4	0.046459	1	0	*YY2*	4	-0.008145	1	0
MYADM	4	0.046605	1	0	*FUNDC1*	4	-0.008143	1	0
SLC25A52	3	0.046656	1	0	*GID4*	2	-0.008108	1	0
FAM3C	2	0.046725	1	0	*CROT*	2	-0.007919	1	0
ABO	4	0.046859	1	0	*DHX32*	4	-0.007869	1	0
CCN5	4	0.046867	1	0	*CORO1A*	4	-0.00785	1	0
TFPI	3	0.046901	1	0	*MTRNR2L2*	1	-0.007824	1	0
TMEM94	4	0.046962	1	0	*TOMM5*	3	-0.007824	1	0
CSNK1G1	3	0.047043	1	0	*RUNDC3B*	4	-0.007723	1	0
SCG3	4	0.047136	1	0	*QRFP*	3	-0.007704	1	0
ARHGEF25	4	0.047199	1	0	*SLC25A4*	4	-0.007668	1	0
LMO1	4	0.047209	1	0	*C2*	4	-0.007498	1	0
HAS3	4	0.047227	1	0	*ATP11C*	4	-0.00748	1	0
FBXO34	4	0.047281	1	0	*NBEAL2*	3	-0.00742	1	0
USP51	4	0.047318	1	0	*RAI14*	4	-0.007412	1	0
SH3BP2	4	0.047321	1	0	*SLC37A4*	4	-0.007275	1	0
LOC389895	2	0.04736	1	0	*CTSO*	4	-0.007237	1	0
CCDC136	3	0.047368	1	0	*RUNDC1*	4	-0.007214	1	0
RTF1	4	0.04745	1	0	*SRRM3*	3	-0.007155	1	0
ITGA9	4	0.047473	1	0	*CFHR3*	4	-0.007126	1	0
SLC31A1	4	0.047545	1	0	*COBLL1*	4	-0.007095	1	0

KCNK9	4	0.047638	1	0	*PLEKHB1*	2	-0.006936	1	0
RP1L1	2	0.047645	1	0	*LRRC14*	3	-0.006897	1	0
TCEAL3	3	0.047713	1	0	*SST*	4	-0.006863	1	0
CBLB	4	0.047724	1	0	*MIPOL1*	4	-0.006819	1	0
MMGT1	4	0.047763	1	0	*CASP8*	4	-0.006684	1	0
ANO2	4	0.04779	1	0	*PCGF1*	4	-0.006649	1	0
LGI1	4	0.047793	1	0	*ELK3*	4	-0.006484	1	0
TAOK1	4	0.047862	1	0	*CDH5*	4	-0.006466	1	0
SULT1C4	4	0.047908	1	0	*TXNRD3*	4	-0.006368	1	0
CX3CL1	3	0.047918	1	0	*PLCG1*	4	-0.006255	1	0
ITIH2	3	0.047958	1	0	*LRCH1*	4	-0.006255	1	0
MTRNR2L4	4	0.047997	1	0	*STX19*	3	-0.006249	1	0
SLC25A14	4	0.048038	1	0	*PPP1R8*	3	-0.006214	1	0
ADPRM	4	0.048052	1	0	*CRYZ*	3	-0.006205	1	0
PHYKPL	4	0.04807	1	0	*ABHD14B*	4	-0.00615	1	0
ZNF335	4	0.048115	1	0	*2-Mar*	4	-0.006017	1	0
ADCY6	3	0.048134	1	0	*ZNF331*	4	-0.005911	1	0
UBE2O	3	0.048151	1	0	*MSC*	3	-0.00587	1	0
FCRLB	3	0.048207	1	0	*RHCG*	4	-0.005854	1	0
AP1B1	2	0.048215	1	0	*FCGR3B*	2	-0.005848	1	0
DERL3	4	0.048216	1	0	*GPR25*	4	-0.005813	1	0
SFTA2	4	0.04822	1	0	*UNC45A*	4	-0.005773	1	0
OPRL1	3	0.048239	1	0	*SAAL1*	4	-0.005683	1	0
CTSF	4	0.048254	1	0	*SHISA8*	4	-0.005678	1	0
TLE2	4	0.048316	1	0	*GRPR*	4	-0.005666	1	0
MCM7	3	0.048409	1	0	*SLC8A1*	4	-0.005506	1	0
IL17RE	4	0.048417	1	0	*COG3*	4	-0.005501	1	0
ANKFY1	1	0.048488	1	0	*GSDMC*	2	-0.005398	1	0
MAGI3	3	0.04854	1	0	*OR10A2*	3	-0.005283	1	0
VHLL	4	0.048649	1	0	*TENT4B*	4	-0.005263	1	0
SLC35A1	4	0.048677	1	0	*ZNF720*	3	-0.005187	1	0
OPN5	4	0.04876	1	0	*ZNF333*	3	-0.005142	1	0
NFATC4	4	0.048824	1	0	*RRAGC*	4	-0.005139	1	0
ASIC3	4	0.048958	1	0	*PRSS2*	2	-0.00513	1	0
OLA1	4	0.048993	1	0	*NIT2*	2	-0.005083	1	0
RNGTT	4	0.049094	1	0	*WNT8A*	3	-0.005046	1	0
IQCE	3	0.049095	1	0	*FUT5*	2	-0.004969	1	0
CTSC	1	0.049118	1	0	*PHF19*	4	-0.004928	1	0
DDX52	4	0.049122	1	0	*FURIN*	4	-0.004893	1	0
MASTL	3	0.049142	1	0	*CCNI2*	4	-0.004873	1	0
DIRAS3	4	0.049165	1	0	*DEPDC5*	4	-0.004855	1	0
BCS1L	3	0.049172	1	0	*PPP1R14C*	4	-0.004797	1	0
ATG9B	4	0.049475	1	0	*SOS2*	4	-0.004785	1	0
CRY2	4	0.049481	1	0	*ADAMTSL4*	4	-0.004774	1	0
TGFB2	3	0.049511	1	0	*PKM*	3	-0.004747	1	0
INHBA	4	0.049601	1	0	*NAPG*	2	-0.004741	1	0
ST6GALNAC6	3	0.049609	1	0	*TJP2*	3	-0.004733	1	0
NCSTN	2	0.049643	1	0	*PSTPIP2*	4	-0.004709	1	0
MATN2	4	0.049656	1	0	*SPATA4*	3	-0.004678	1	0
UNC5A	4	0.049839	1	0	*H2BC12*	1	-0.004594	1	0
PGK1	4	0.049867	1	0	*RNF25*	4	-0.004581	1	0
KLRF1	3	0.049961	1	0	*NSG2*	4	-0.004578	1	0
TWF2	4	0.049984	1	0	*SCNN1D*	4	-0.004571	1	0
CENPC	3	0.050041	1	0	*ACSL5*	4	-0.004566	1	0
MTSS1	3	0.050056	1	0	*COL10A1*	3	-0.004404	1	0
GPRIN2	4	0.050098	1	0	*MAGEH1*	4	-0.004366	1	0
BRWD3	3	0.050105	1	0	*PEX12*	3	-0.004356	1	0
WDTC1	4	0.050202	1	0	*ARHGAP1*	4	-0.004348	1	0

WTIP	2	0.050261	1	0	*TRIM41*	4	-0.004292	1	0
CD164L2	4	0.0503	1	0	*PCDHGA1*	4	-0.004281	1	0
TBC1D32	4	0.050337	1	0	*MED7*	4	-0.004184	1	0
CYYR1	1	0.050347	1	0	*FUT9*	3	-0.004143	1	0
CEP170B	4	0.050423	1	0	*AMMECR1L*	4	-0.004115	1	0
THSD1	3	0.050455	1	0	*ZNF611*	4	-0.004021	1	0
KBTBD4	4	0.050466	1	0	*DLX2*	3	-0.003986	1	0
SLC45A4	4	0.050583	1	0	*C9*	3	-0.003933	1	0
TERF1	4	0.050589	1	0	*PRSS35*	3	-0.003912	1	0
GJA4	4	0.050699	1	0	*CDHR3*	4	-0.0039	1	0
FIGLA	3	0.050707	1	0	*ACBD7*	1	-0.003856	1	0
HSD17B8	4	0.050748	1	0	*CPD*	3	-0.003826	1	0
SQOR	3	0.050875	1	0	*KCNQ1*	3	-0.003813	1	0
GIN1	3	0.050893	1	0	*HMOX1*	4	-0.003805	1	0
DKK3	4	0.050908	1	0	*LSAMP*	3	-0.003714	1	0
TKFC	4	0.050986	1	0	*GPR173*	3	-0.003695	1	0
ZNF691	4	0.050997	1	0	*PFKL*	3	-0.00364	1	0
MROH1	3	0.051082	1	0	*ZFPM2*	4	-0.003611	1	0
HEATR5A	3	0.051092	1	0	*B9D1*	4	-0.003597	1	0
RAD18	4	0.051208	1	0	*ATP4B*	4	-0.003591	1	0
RGS18	3	0.051294	1	0	*ECH1*	4	-0.003471	1	0
ATP11B	4	0.051346	1	0	*ZNF575*	3	-0.003442	1	0
SETD3	4	0.051355	1	0	*ANAPC15*	4	-0.003409	1	0
PKIG	4	0.0516	1	0	*SLC51B*	4	-0.00333	1	0
SLC37A2	3	0.051611	1	0	*TPCN2*	4	-0.00333	1	0
VDR	3	0.051618	1	0	*ACE2*	4	-0.003323	1	0
ENY2	4	0.051657	1	0	*CATSPER3*	4	-0.003305	1	0
KCNH7	4	0.05173	1	0	*NR2E3*	2	-0.00326	1	0
CFAP53	4	0.051778	1	0	*ACTA2*	3	-0.003202	1	0
MAPK8	2	0.051915	1	0	*SLC35F3*	3	-0.003122	1	0
BPTF	4	0.051923	1	0	*LRRC34*	4	-0.003105	1	0
ZNF662	2	0.052006	1	0	*TFF1*	4	-0.003085	1	0
MTRF1	4	0.052008	1	0	*RILPL2*	4	-0.003083	1	0
BATF2	4	0.052059	1	0	*KCNJ14*	4	-0.003066	1	0
SP140	4	0.052064	1	0	*ART1*	2	-0.003044	1	0
SYCE1	4	0.052066	1	0	*TSNARE1*	2	-0.003032	1	0
PTPRK	3	0.052088	1	0	*TMEM134*	4	-0.003024	1	0
ZFPM1	4	0.052129	1	0	*NCOA2*	3	-0.002973	1	0
TBC1D22B	4	0.052235	1	0	*SUCLG2*	3	-0.002904	1	0
NMRK1	4	0.052263	1	0	*CCDC159*	4	-0.002832	1	0
MRPS35	3	0.052292	1	0	*IGFBP2*	3	-0.002815	1	0
RSL1D1	4	0.052354	1	0	*ATP5IF1*	4	-0.002742	1	0
H2AZ1	4	0.052419	1	0	*MROH8*	3	-0.002618	1	0
BLNK	3	0.052452	1	0	*TBC1D2*	4	-0.002497	1	0
SLC30A5	3	0.052461	1	0	*RNPS1*	3	-0.002477	1	0
WNK3	4	0.052476	1	0	*CD63*	4	-0.002461	1	0
MAP3K3	3	0.052484	1	0	*RNF144A*	4	-0.002388	1	0
BCL2L12	4	0.052575	1	0	*KLF4*	2	-0.00233	1	0
MSL3	3	0.052652	1	0	*GRIPAP1*	4	-0.002156	1	0
ZNF334	4	0.052653	1	0	*GADL1*	4	-0.002122	1	0
KCTD21	4	0.052725	1	0	*CNDP1*	4	-0.002122	1	0
JAKMIP3	3	0.052727	1	0	*MDM1*	3	-0.00211	1	0
CLK3	4	0.052761	1	0	*SLC44A5*	4	-0.002015	1	0
STKLD1	4	0.052766	1	0	*GHRHR*	4	-0.001982	1	0
ZNF395	3	0.052775	1	0	*MARVELD2*	3	-0.001964	1	0
NINL	2	0.052787	1	0	*VN1R1*	4	-0.001928	1	0
SHCBP1	3	0.05279	1	0	*GPR15*	4	-0.001885	1	0
MALSU1	4	0.052921	1	0	*MAPK14*	4	-0.001862	1	0

KLF16	1	0.052925	1	0	*CNR1*	3	-0.001862	1	0
NFS1	4	0.053021	1	0	*FKTN*	4	-0.001776	1	0
UCP1	3	0.053101	1	0	*WSCD2*	3	-0.001704	1	0
XKR8	4	0.053138	1	0	*CREB3L1*	4	-0.001691	1	0
DOC2A	4	0.05314	1	0	*ABCB10*	4	-0.001689	1	0
DPY19L2	4	0.053194	1	0	*HS3ST6*	4	-0.001654	1	0
ABCG4	4	0.053209	1	0	*CT45A1*	1	-0.001611	1	0
FAM122C	4	0.053356	1	0	*SLC5A7*	4	-0.00161	1	0
CITED1	3	0.053383	1	0	*CLDN1*	4	-0.001593	1	0
ADGRG5	3	0.053477	1	0	*SRM*	3	-0.001492	1	0
PKP4	4	0.053627	1	0	*RCOR3*	4	-0.001461	1	0
CSH2	1	0.05363	1	0	*MCM9*	4	-0.001385	1	0
FAM111B	4	0.05363	1	0	*ZNF512*	4	-0.00132	1	0
ACBD4	4	0.053682	1	0	*PDCD6IP*	4	-0.001271	1	0
CDK17	2	0.053725	1	0	*GPRIN3*	4	-0.001269	1	0
RIPOR3	4	0.053804	1	0	*INAVA*	4	-0.001236	1	0
FAM131C	4	0.053831	1	0	*FAM111B*	4	-0.001186	1	0
POP7	2	0.053926	1	0	*REPIN1*	3	-0.001106	1	0
TPSG1	2	0.054067	1	0	*ALDH7A1*	3	-9.09E-04	1	0
FAM170A	4	0.054154	1	0	*FOXN3*	4	-6.43E-04	1	0
ARL6	4	0.054165	1	0	*NRIP3*	3	-5.79E-04	1	0
CROCC	4	0.0542	1	0	*DNAL4*	3	-5.73E-04	1	0
TGM3	4	0.054224	1	0	*CTPS2*	4	-4.04E-04	1	0
ZNF223	3	0.054273	1	0	*BRD1*	3	-4.01E-04	1	0
NKX1-2	4	0.05429	1	0	*ARHGEF33*	4	-3.45E-04	1	0
ST8SIA4	4	0.054318	1	0	*HRNR*	4	-2.86E-04	1	0
NPAS1	4	0.054404	1	0	*ABCG8*	4	-2.81E-04	1	0
LIPG	3	0.054416	1	0	*TMEM184C*	4	-1.74E-04	1	0
ERCC4	4	0.054429	1	0	*ZNF500*	4	-1.41E-04	1	0
GALM	4	0.054456	1	0	*CA3*	4	-1.41E-04	1	0
MAPK15	2	0.054467	1	0	*C15orf41*	2	-1.19E-04	1	0
PBXIP1	4	0.054479	1	0	*CREBL2*	4	-6.94E-05	1	0
POGLUT3	3	0.054522	1	0	*C11orf24*	4	2.02E-05	1	0
PGPEP1	4	0.054526	1	0	*MTSS1*	3	1.35E-04	1	0
GOLGA2	4	0.054537	1	0	*PROS1*	3	1.90E-04	1	0
MEN1	3	0.054538	1	0	*DCAF17*	4	1.99E-04	1	0
ARHGAP6	4	0.054567	1	0	*SREK1*	4	2.98E-04	1	0
IGF2R	3	0.054578	1	0	*RNPC3*	4	3.30E-04	1	0
ASPH	4	0.054665	1	0	*ZNRD2*	4	3.51E-04	1	0
DBF4B	3	0.054679	1	0	*FOXJ1*	4	4.59E-04	1	0
SCUBE1	3	0.054743	1	0	*WFDC10A*	3	4.93E-04	1	0
CCR6	4	0.054769	1	0	*IRF2BP1*	3	5.02E-04	1	0
WDR76	4	0.054776	1	0	*DYNLT1*	4	5.07E-04	1	0
DHCR24	4	0.05486	1	0	*SIK3*	4	6.75E-04	1	0
ATG101	4	0.054893	1	0	*DEFA5*	4	7.44E-04	1	0
ERLEC1	4	0.054901	1	0	*H2BC14*	3	8.07E-04	1	0
ZNF296	4	0.054905	1	0	*RNF121*	3	8.42E-04	1	0
CREB3L3	4	0.054926	1	0	*GUCY2C*	4	9.42E-04	1	0
IGSF8	4	0.054964	1	0	*RARRES2*	4	0.001104	1	0
KMO	4	0.05503	1	0	*NKX6-2*	3	0.001206	1	0
SOX4	3	0.055114	1	0	*TACC1*	3	0.001207	1	0
CENPN	4	0.055123	1	0	*ULK4*	4	0.001267	1	0
LAMB4	3	0.055129	1	0	*COX8C*	4	0.00128	1	0
KDELR1	4	0.055145	1	0	*PAX9*	3	0.001288	1	0
SRP19	4	0.055186	1	0	*SENP7*	3	0.001384	1	0
CYP2J2	4	0.055205	1	0	*ALK*	2	0.001465	1	0
DNAJB6	4	0.055205	1	0	*EXTL2*	4	0.001486	1	0
GXYLT2	4	0.055259	1	0	*TNFRSF17*	4	0.001548	1	0

KRT13	4	0.055265	1	0	*NSG1*	3	0.001633	1	0
SHB	4	0.055311	1	0	*EIF4E1B*	3	0.001651	1	0
SLC25A36	3	0.055483	1	0	*AIMP2*	4	0.001652	1	0
PSMD9	4	0.055494	1	0	*HADHB*	3	0.001709	1	0
GHDC	3	0.055742	1	0	*CEP68*	4	0.001713	1	0
FBF1	3	0.055762	1	0	*KIF21A*	4	0.001726	1	0
TUBB2A	2	0.055808	1	0	*TUBA3E*	3	0.001737	1	0
LOC728485	2	0.055809	1	0	*OAT*	3	0.001742	1	0
CLIC4	4	0.055819	1	0	*DPYSL5*	4	0.001743	1	0
DNAJC21	3	0.055837	1	0	*CHD7*	4	0.001769	1	0
ELF3	4	0.055881	1	0	*LBX1*	4	0.001773	1	0
KIAA1586	2	0.055918	1	0	*FGF3*	3	0.001826	1	0
CATSPER4	2	0.055924	1	0	*DNAJC30*	4	0.001827	1	0
USP16	4	0.055925	1	0	*ZC3HAV1L*	3	0.001932	1	0
FRK	4	0.055961	1	0	*RGL4*	2	0.002056	1	0
MRPS33	4	0.056057	1	0	*CLINT1*	3	0.002099	1	0
HIP1R	3	0.056178	1	0	*DUOX1*	4	0.00212	1	0
PIGX	4	0.056286	1	0	*VWDE*	3	0.002124	1	0
REPS1	4	0.05634	1	0	*TMEM109*	3	0.002134	1	0
DPEP2	4	0.056488	1	0	*CNTN2*	3	0.002156	1	0
PLAC8	4	0.056493	1	0	*PCDHA5*	3	0.00217	1	0
FAM241B	4	0.056498	1	0	*UGT1A4*	4	0.002267	1	0
SUSD2	4	0.056552	1	0	*TMEM186*	4	0.002282	1	0
CELF2	4	0.056651	1	0	*AGBL3*	4	0.002428	1	0
RLF	4	0.056681	1	0	*ZZEF1*	4	0.002494	1	0
TRABD2A	3	0.056686	1	0	*CBY3*	4	0.002505	1	0
WFDC8	4	0.056728	1	0	*GFI1*	4	0.002537	1	0
DIO3	4	0.056778	1	0	*ERMN*	4	0.002537	1	0
C8G	3	0.056856	1	0	*CAMSAP2*	3	0.002549	1	0
OMG	4	0.056964	1	0	*MAN2A1*	4	0.002614	1	0
BAAT	3	0.057013	1	0	*BMP2*	3	0.002725	1	0
PTGER1	3	0.057066	1	0	*BDH1*	3	0.002796	1	0
ZNF74	4	0.057159	1	0	*SDK1*	4	0.002818	1	0
MAP4K2	4	0.057222	1	0	*CALML4*	4	0.002822	1	0
RAMP1	4	0.057239	1	0	*HNF1A*	4	0.002894	1	0
PDIK1L	4	0.057242	1	0	*CRADD*	4	0.002913	1	0
APOBEC3F	3	0.057261	1	0	*GYG1*	4	0.002959	1	0
TIGIT	4	0.057272	1	0	*MYO1B*	4	0.003184	1	0
CIB4	4	0.057291	1	0	*SLC11A1*	4	0.003263	1	0
CPNE1	4	0.057377	1	0	*LINC01621*	1	0.003336	1	0
PRSS3	4	0.057398	1	0	*QTRT2*	4	0.003383	1	0
NACC1	4	0.057425	1	0	*ANKRD61*	4	0.003387	1	0
NSMCE2	3	0.057435	1	0	*HEPH*	3	0.003596	1	0
CNKSR1	4	0.057475	1	0	*POP5*	3	0.003627	1	0
BTBD1	3	0.057488	1	0	*STAG2*	4	0.00373	1	0
SLC4A11	4	0.057508	1	0	*SERPINA3*	3	0.003765	1	0
RALY	4	0.057516	1	0	*KLHDC1*	4	0.003766	1	0
ALPK3	4	0.057534	1	0	*CCNDBP1*	3	0.00384	1	0
ZNF451	3	0.057636	1	0	*AHDC1*	3	0.003909	1	0
DUSP14	4	0.057845	1	0	*OR10C1*	4	0.00392	1	0
IAH1	4	0.057954	1	0	*AK1*	4	0.004006	1	0
CHMP2B	3	0.058079	1	0	*RTCA*	4	0.004018	1	0
ARHGAP9	4	0.058106	1	0	*FBXO38*	4	0.004021	1	0
ZFP36L1	4	0.058131	1	0	*GLI3*	4	0.004142	1	0
EHHADH	4	0.058183	1	0	*ZHX2*	4	0.004216	1	0
GEMIN5	3	0.058202	1	0	*CYP17A1*	4	0.004308	1	0
REEP4	3	0.058243	1	0	*FRMD8*	4	0.004329	1	0
PAOX	3	0.05836	1	0	*PIK3C2B*	4	0.004331	1	0

PUS7	3	0.058437	1	0	*ARV1*	3	0.004372	1	0
GFRA1	2	0.058456	1	0	*REM1*	4	0.004405	1	0
AKR1B15	4	0.05847	1	0	*LATS1*	3	0.00446	1	0
ZBBX	4	0.058492	1	0	*POLDIP3*	4	0.004617	1	0
NDUFAF6	4	0.058527	1	0	*CXCL2*	3	0.004934	1	0
ALCAM	4	0.058573	1	0	*SLC37A3*	3	0.004995	1	0
FAM120C	3	0.058653	1	0	*ZNF792*	2	0.005033	1	0
TRABD2B	3	0.058671	1	0	*KCNJ13*	4	0.005062	1	0
EIF2AK4	4	0.058847	1	0	*CELF2*	4	0.005148	1	0
ZNF516	2	0.058858	1	0	*TDRD7*	4	0.005236	1	0
AOC3	4	0.058944	1	0	*TOX*	4	0.005236	1	0
SMIM10L1	2	0.059027	1	0	*MAP2K3*	3	0.005359	1	0
NETO1	4	0.059029	1	0	*COL4A4*	4	0.005387	1	0
GPR108	4	0.059098	1	0	*ADGRF3*	3	0.005398	1	0
HRK	3	0.059112	1	0	*ZSCAN16*	3	0.005412	1	0
SLC35A3	4	0.059117	1	0	*COL6A6*	3	0.005427	1	0
NOTCH4	4	0.059157	1	0	*CHAT*	4	0.005436	1	0
PDK2	4	0.059173	1	0	*MARCHF10*	1	0.005503	1	0
TNNT2	2	0.059296	1	0	*AP4S1*	2	0.005524	1	0
PATE2	4	0.059326	1	0	*PRMT9*	3	0.005581	1	0
TK2	4	0.05942	1	0	*USP6*	2	0.005589	1	0
CEP44	3	0.059504	1	0	*SPATA33*	4	0.005686	1	0
MROH6	4	0.059582	1	0	*CAPS*	3	0.005715	1	0
PCDHGB4	3	0.059597	1	0	*EXD3*	4	0.005761	1	0
METTL2A	1	0.059654	1	0	*PIWIL2*	3	0.005782	1	0
CARMIL3	3	0.059675	1	0	*CHRNA10*	3	0.005898	1	0
PRKCD	4	0.0597	1	0	*FBXW7*	3	0.0059	1	0
TTC14	4	0.059797	1	0	*HELB*	4	0.005919	1	0
REV1	3	0.059822	1	0	*ANKRD13C*	4	0.005999	1	0
CIAO3	4	0.060003	1	0	*TBC1D3B*	1	0.006017	1	0
UQCRC1	4	0.060035	1	0	*LYZ*	4	0.006088	1	0
ZNF582	3	0.060164	1	0	*NEK11*	4	0.006139	1	0
TMEM178B	4	0.06019	1	0	*TRADD*	4	0.006274	1	0
ALG14	3	0.060199	1	0	*VNN3*	4	0.006381	1	0
PRR18	3	0.060205	1	0	*DBN1*	4	0.006391	1	0
C20orf197	3	0.060296	1	0	*UNG*	3	0.006423	1	0
ZNF394	4	0.060329	1	0	*ZNF766*	4	0.006445	1	0
FLNC	3	0.060331	1	0	*CFAP46*	2	0.006458	1	0
PRADC1	4	0.060357	1	0	*ARHGDIB*	4	0.006536	1	0
ZNF230	3	0.060379	1	0	*UBQLNL*	3	0.006551	1	0
OR2K2	3	0.060415	1	0	*PDAP1*	2	0.006617	1	0
CENPO	4	0.060506	1	0	*ZNF16*	3	0.006668	1	0
DNAJB14	3	0.060722	1	0	*DLG5*	4	0.00669	1	0
EIPR1	3	0.060755	1	0	*TOP2B*	4	0.006783	1	0
SURF2	4	0.060802	1	0	*CD1E*	4	0.006788	1	0
SLC10A5	4	0.060818	1	0	*ZNRF3*	4	0.006873	1	0
KRT10	4	0.060884	1	0	*OPN1SW*	4	0.006903	1	0
MAGED2	4	0.060896	1	0	*LTC4S*	2	0.006906	1	0
LPP	4	0.060904	1	0	*NUSAP1*	4	0.006967	1	0
HADH	2	0.060956	1	0	*FAM98B*	3	0.007001	1	0
TMBIM6	3	0.060994	1	0	*FLNB*	4	0.007022	1	0
ATF7IP2	4	0.061024	1	0	*VWA3A*	4	0.007126	1	0
FANCF	3	0.061223	1	0	*HYKK*	4	0.007141	1	0
MYC	4	0.061316	1	0	*SLC6A11*	1	0.007281	1	0
ATF7IP	4	0.061356	1	0	*ACBD5*	4	0.007298	1	0
TREM1	2	0.061377	1	0	*BHLHE41*	4	0.007334	1	0
PIGK	4	0.061409	1	0	*PTGER4*	3	0.007462	1	0
SMG7	4	0.061531	1	0	*SLC35G5*	4	0.007651	1	0

C19orf33	4	0.061612	1	0	*FANCG*	4	0.007711	1	0
GREB1	4	0.061763	1	0	*PCDHGA7*	3	0.007817	1	0
GNG2	4	0.061776	1	0	*ALDH1L2*	4	0.007918	1	0
IFI30	2	0.06182	1	0	*ZNF687*	4	0.007923	1	0
BACE2	4	0.061828	1	0	*NUDT9*	4	0.007946	1	0
PLEKHA1	3	0.061847	1	0	*STRA8*	4	0.008035	1	0
ZBTB17	3	0.061882	1	0	*FOXK1*	3	0.008051	1	0
ANAPC16	4	0.061923	1	0	*CR1L*	3	0.008125	1	0
RNF19A	4	0.061972	1	0	*SLC1A4*	4	0.008131	1	0
OR2AG2	4	0.062101	1	0	*ARHGAP21*	3	0.008247	1	0
RARA	4	0.062128	1	0	*FAM204A*	4	0.00825	1	0
ZCWPW1	4	0.06218	1	0	*YIPF4*	4	0.008273	1	0
TMEM156	3	0.062281	1	0	*CPS1*	4	0.008324	1	0
ZYX	4	0.062397	1	0	*PDE8A*	4	0.008338	1	0
DCTN5	4	0.062399	1	0	*HBS1L*	4	0.008378	1	0
LYSMD1	4	0.062462	1	0	*UGCG*	4	0.008445	1	0
FAM50A	3	0.06259	1	0	*ACAP1*	2	0.008493	1	0
ANKRD53	4	0.062617	1	0	*CAMTA2*	4	0.008501	1	0
SH3BP1	2	0.062642	1	0	*FOXC1*	4	0.008664	1	0
SEPTIN3	4	0.062648	1	0	*UBE2R2*	3	0.008671	1	0
MVB12B	4	0.062741	1	0	*F2RL3*	3	0.008758	1	0
COQ5	3	0.06283	1	0	*CMC2*	4	0.008786	1	0
HDLBP	4	0.062861	1	0	*GDAP1*	4	0.008824	1	0
TMEM220	3	0.062873	1	0	*IPO4*	4	0.008938	1	0
DMTN	4	0.062898	1	0	*MGLL*	4	0.009063	1	0
EYS	3	0.062948	1	0	*RNF207*	3	0.009117	1	0
SNAP47	4	0.062998	1	0	*TRAM1L1*	3	0.009135	1	0
STX11	4	0.063051	1	0	*GHDC*	3	0.009182	1	0
C20orf202	3	0.063052	1	0	*USH1C*	4	0.009224	1	0
RELL1	4	0.063072	1	0	*GALM*	4	0.00925	1	0
FBXO46	2	0.063123	1	0	*SYNGR3*	4	0.009275	1	0
ZNF439	4	0.063125	1	0	*OAZ2*	4	0.009279	1	0
TMPRSS6	4	0.06313	1	0	*GRB14*	3	0.009298	1	0
BTBD11	3	0.063275	1	0	*KIF5B*	4	0.009336	1	0
TMEM270	3	0.063378	1	0	*CYTH2*	3	0.00934	1	0
IL2RB	2	0.063444	1	0	*PTPRC*	3	0.00934	1	0
IDH1	4	0.063477	1	0	*RALY*	4	0.009366	1	0
CD70	4	0.063499	1	0	*SERINC5*	4	0.009383	1	0
NUP107	4	0.063499	1	0	*IMPG2*	3	0.009501	1	0
TRMT44	3	0.063563	1	0	*SRMS*	4	0.009501	1	0
LSM8	4	0.063652	1	0	*IFFO1*	2	0.009507	1	0
THTPA	4	0.063686	1	0	*HYOU1*	4	0.009559	1	0
DNAJC24	4	0.063692	1	0	*GALNT8*	4	0.009559	1	0
TULP1	4	0.063726	1	0	*SHISA5*	4	0.009636	1	0
KCNK2	4	0.063747	1	0	*TACC2*	4	0.00968	1	0
TCTEX1D4	2	0.063769	1	0	*THBS4*	3	0.009731	1	0
EPOR	3	0.063797	1	0	*TM6SF1*	3	0.009894	1	0
DZIP3	4	0.063846	1	0	*CDK14*	4	0.00994	1	0
GJA3	3	0.063863	1	0	*ZNF316*	4	0.009954	1	0
SHISAL2B	4	0.063872	1	0	*CEMIP*	3	0.010091	1	0
TMEM59L	4	0.063889	1	0	*PBX1*	3	0.010125	1	0
RHOA	4	0.0639	1	0	*CDC42SE1*	3	0.010163	1	0
TNFAIP3	4	0.0639	1	0	*NOS3*	4	0.010185	1	0
ZSCAN2	4	0.06391	1	0	*PTPN14*	4	0.010244	1	0
C14orf93	4	0.063926	1	0	*PCDHA9*	4	0.010315	1	0
TMED3	4	0.063987	1	0	*NLRP14*	4	0.010496	1	0
TMEM132E	3	0.064053	1	0	*GLIS2*	2	0.010535	1	0
WDR5B	2	0.06407	1	0	*TRIP11*	4	0.010634	1	0

LINGO3	2	0.064084	1	0	*PTH1R*	2	0.010635	1	0
LTBP4	4	0.064085	1	0	*APH1A*	3	0.010657	1	0
UCK1	4	0.06416	1	0	*HSD17B8*	4	0.010688	1	0
ZNF598	4	0.064303	1	0	*LDLRAP1*	4	0.010703	1	0
TRIM14	3	0.064359	1	0	*CILP*	4	0.010866	1	0
KNL1	4	0.064369	1	0	*TCF24*	3	0.010899	1	0
TKTL1	4	0.064381	1	0	*MPC1L*	1	0.010959	1	0
CXCL11	4	0.064511	1	0	*FLT3*	4	0.01098	1	0
ZNF697	4	0.06455	1	0	*SCP2*	4	0.010986	1	0
C8orf86	4	0.064602	1	0	*TNXB*	3	0.011085	1	0
RIOX2	3	0.06463	1	0	*AKAP13*	2	0.011094	1	0
CD9	3	0.064823	1	0	*TCEANC2*	4	0.011124	1	0
CEBPB	2	0.064825	1	0	*ZNF451*	3	0.011154	1	0
TUBA3E	3	0.064898	1	0	*MYH7*	4	0.01117	1	0
EDC4	4	0.065007	1	0	*TIMM8B*	4	0.011297	1	0
CARD11	4	0.065023	1	0	*CIC*	4	0.011359	1	0
FAM71F2	4	0.065084	1	0	*SLCO1B7*	3	0.011364	1	0
TFEC	3	0.065156	1	0	*GKAP1*	4	0.01139	1	0
GADD45A	3	0.065187	1	0	*CEP78*	4	0.011391	1	0
NBPF12	4	0.065216	1	0	*ZNF354C*	4	0.011439	1	0
MTHFD1	4	0.065303	1	0	*GPAM*	3	0.011448	1	0
ZNRF2	2	0.065316	1	0	*DSCAML1*	4	0.011547	1	0
ATP1A2	4	0.065369	1	0	*TWISTNB*	1	0.011624	1	0
GNAI3	4	0.06538	1	0	*RGPD8*	1	0.011644	1	0
SH2B3	4	0.065409	1	0	*LENEP*	4	0.011668	1	0
RYK	4	0.065417	1	0	*MAP3K12*	3	0.011715	1	0
ZNF239	4	0.065504	1	0	*CHRNB4*	3	0.011743	1	0
CXCR6	3	0.065545	1	0	*KRT40*	4	0.011788	1	0
RASAL1	4	0.065596	1	0	*FAM71F2*	4	0.011789	1	0
FSTL1	4	0.065603	1	0	*FLRT1*	4	0.011848	1	0
CRLS1	3	0.06567	1	0	*LPCAT1*	3	0.011859	1	0
PROX1	4	0.065802	1	0	*DLC1*	3	0.011883	1	0
C1orf174	4	0.065916	1	0	*CDKL3*	2	0.011918	1	0
MAD2L1	3	0.066084	1	0	*ZNF653*	4	0.011968	1	0
DONSON	3	0.066125	1	0	*PARP3*	2	0.011987	1	0
MCM2	3	0.066177	1	0	*DAB2*	4	0.011994	1	0
MSX2	2	0.066187	1	0	*TBXAS1*	4	0.012001	1	0
MEF2D	4	0.06627	1	0	*SCN3A*	4	0.012013	1	0
BRI3	4	0.066281	1	0	*SLC16A14*	4	0.012233	1	0
RPS12	3	0.066305	1	0	*ANAPC16*	4	0.012261	1	0
GBGT1	3	0.066312	1	0	*CYP4X1*	4	0.012266	1	0
NSA2	2	0.06633	1	0	*TTC23*	4	0.012267	1	0
ABHD1	4	0.066407	1	0	*AFAP1*	2	0.012295	1	0
TMEM163	3	0.066494	1	0	*DCUN1D1*	4	0.012666	1	0
CASK	4	0.066567	1	0	*FAM114A1*	4	0.012678	1	0
GSTZ1	4	0.066574	1	0	*ZFYVE9*	3	0.012826	1	0
TSPO	3	0.066611	1	0	*PRDX4*	4	0.01283	1	0
IRF9	3	0.066636	1	0	*PCDH19*	3	0.012852	1	0
TFAP2C	4	0.066691	1	0	*NELL2*	2	0.012924	1	0
CCNI	4	0.066986	1	0	*NKD2*	4	0.012953	1	0
FBXL20	3	0.067037	1	0	*C10orf105*	4	0.013016	1	0
DPY19L1	2	0.067158	1	0	*PCDHB8*	4	0.013074	1	0
F10	3	0.067243	1	0	*PSD2*	4	0.013082	1	0
CEP19	3	0.067294	1	0	*TXNDC9*	3	0.013089	1	0
INSM1	3	0.067324	1	0	*KLK1*	3	0.013139	1	0
KCNMA1	3	0.067332	1	0	*NAALADL2*	4	0.013161	1	0
TOMM5	3	0.067387	1	0	*SNAI3*	4	0.013164	1	0
TTC39A	3	0.067432	1	0	*PLA2R1*	4	0.013387	1	0

GLIS1	4	0.067536	1	0	*RHOXF1*	3	0.013411	1	0
EAF2	3	0.067595	1	0	*PDE3B*	3	0.013457	1	0
WEE1	4	0.067596	1	0	*TAGLN*	4	0.013569	1	0
TMEM68	4	0.067654	1	0	*COL9A1*	4	0.013657	1	0
TDRD12	3	0.067683	1	0	*INHBB*	4	0.013783	1	0
APOBEC3C	4	0.067731	1	0	*RBM38*	1	0.013973	1	0
BCL6B	3	0.067872	1	0	*CYP2C18*	4	0.014021	1	0
TIFA	4	0.067956	1	0	*ITGBL1*	4	0.014093	1	0
SCAMP4	3	0.067983	1	0	*PRRT3*	4	0.014125	1	0
MAP3K11	4	0.068103	1	0	*RPS12*	3	0.014135	1	0
TICAM1	4	0.068161	1	0	*PRDM1*	2	0.014202	1	0
CYP1A2	4	0.068203	1	0	*ZFP82*	3	0.014203	1	0
PSMD8	4	0.068287	1	0	*IFNLR1*	3	0.014207	1	0
PPP2R2C	4	0.068294	1	0	*SMAD1*	3	0.014256	1	0
DCPS	3	0.068356	1	0	*ERMP1*	4	0.014424	1	0
KCNG2	4	0.068415	1	0	*HOXB8*	3	0.014429	1	0
TACR1	4	0.068503	1	0	*BOD1*	4	0.014457	1	0
RC3H2	4	0.068555	1	0	*PCYT2*	4	0.014516	1	0
C17orf58	4	0.068603	1	0	*PPTC7*	3	0.014587	1	0
NDUFS3	4	0.068667	1	0	*ANKK1*	4	0.01463	1	0
ABCG1	3	0.068692	1	0	*PDIA3*	4	0.014676	1	0
TCHP	3	0.068793	1	0	*AGBL5*	2	0.014738	1	0
R3HCC1	3	0.068798	1	0	*OR52B6*	4	0.014741	1	0
KITLG	3	0.068939	1	0	*MUC16*	4	0.014814	1	0
RBL2	3	0.068955	1	0	*FBH1*	4	0.014832	1	0
ZNHIT6	4	0.068961	1	0	*NOXA1*	4	0.014879	1	0
ITGAX	4	0.069077	1	0	*ZNF592*	3	0.014963	1	0
SLC34A3	4	0.069094	1	0	*PNPLA6*	4	0.015049	1	0
DHDH	4	0.069203	1	0	*AP1M2*	4	0.01517	1	0
CST1	3	0.06921	1	0	*LMO1*	4	0.01527	1	0
DLG1	4	0.069271	1	0	*BAGE2*	2	0.015407	1	0
BTBD19	4	0.069272	1	0	*SFN*	3	0.015532	1	0
LPAR1	3	0.069388	1	0	*GPBP1L1*	3	0.015547	1	0
ASF1B	2	0.069416	1	0	*GJA1*	4	0.015594	1	0
FAN1	4	0.069476	1	0	*TEX46*	3	0.015626	1	0
PCP4L1	4	0.069521	1	0	*SAGE1*	4	0.015647	1	0
FAT4	3	0.06953	1	0	*C4orf46*	3	0.015674	1	0
SBF1	3	0.069532	1	0	*OXER1*	4	0.015711	1	0
PIF1	3	0.069576	1	0	*SLC4A11*	4	0.015715	1	0
MANEAL	3	0.069599	1	0	*NCDN*	4	0.015723	1	0
TLX3	4	0.06961	1	0	*S100G*	4	0.015738	1	0
LARS1	2	0.069696	1	0	*ARHGAP28*	4	0.015738	1	0
LRRC7	2	0.069809	1	0	*RAB19*	4	0.01574	1	0
BFSP2	4	0.069903	1	0	*C5AR2*	3	0.015789	1	0
ATAD2B	4	0.069953	1	0	*MCM10*	4	0.015886	1	0
IRX2	4	0.069969	1	0	*C2CD6*	4	0.015899	1	0
PPP2R3B	4	0.070033	1	0	*JADE2*	2	0.016001	1	0
CDC45	4	0.070092	1	0	*PCSK4*	4	0.016006	1	0
FGD5	3	0.070149	1	0	*GNB3*	4	0.016046	1	0
DOP1A	4	0.070193	1	0	*SAYSD1*	3	0.01606	1	0
NR1H2	2	0.070196	1	0	*HOXA7*	4	0.016199	1	0
FAM131B	3	0.070211	1	0	*NFIL3*	4	0.016318	1	0
MS4A1	3	0.070495	1	0	*SH3KBP1*	4	0.016333	1	0
C1QTNF9B	1	0.070556	1	0	*FAM122C*	4	0.016356	1	0
NHS	3	0.070595	1	0	*RGS3*	4	0.016387	1	0
FLAD1	2	0.070608	1	0	*SLC15A4*	3	0.016435	1	0
MMACHC	4	0.070647	1	0	*ZNF32*	4	0.016475	1	0
VENTX	1	0.070655	1	0	*CHPF2*	4	0.016492	1	0

ADK	4	0.070668	1	0	*PCDHGA3*	4	0.016546	1	0
CCNG2	4	0.070694	1	0	*CPEB4*	4	0.016651	1	0
SNX13	4	0.070825	1	0	*HIC1*	4	0.016792	1	0
MBLAC1	4	0.070827	1	0	*RECQL*	2	0.01694	1	0
TRIB2	4	0.070869	1	0	*PRDM2*	4	0.017124	1	0
MAGEB17	4	0.070943	1	0	*RUNX3*	4	0.017126	1	0
PSMD10	2	0.071019	1	0	*LZTFL1*	4	0.017244	1	0
LRFN1	4	0.071045	1	0	*SNX12*	4	0.017284	1	0
SLC41A2	4	0.071115	1	0	*HASPIN*	3	0.017414	1	0
CCDC149	4	0.071257	1	0	*TSPAN9*	3	0.017476	1	0
MAP3K21	4	0.071401	1	0	*STXBP1*	4	0.017511	1	0
UCP2	4	0.07143	1	0	*PRICKLE1*	4	0.0176	1	0
FBXW5	2	0.071541	1	0	*H4C8*	4	0.017663	1	0
FDFT1	4	0.071547	1	0	*FAM200B*	1	0.017712	1	0
MYOF	4	0.071657	1	0	*ZACN*	4	0.017748	1	0
SLC25A1	3	0.071758	1	0	*JAM3*	4	0.017824	1	0
RAB5A	3	0.071759	1	0	*CDH24*	4	0.017839	1	0
PGLS	3	0.071804	1	0	*WDPCP*	3	0.017863	1	0
PIK3CB	3	0.07181	1	0	*IGF1*	4	0.017867	1	0
ARHGAP4	4	0.071861	1	0	*OXSR1*	3	0.017881	1	0
CD83	4	0.072016	1	0	*TDP2*	4	0.017973	1	0
LYRM7	4	0.072067	1	0	*KLRG1*	2	0.017997	1	0
TMEM128	4	0.072152	1	0	*CMBL*	3	0.018041	1	0
MCF2L	2	0.072167	1	0	*ZBTB26*	2	0.018187	1	0
MED8	4	0.072172	1	0	*CCDC154*	4	0.018434	1	0
AKAP8	4	0.072174	1	0	*CST6*	3	0.018508	1	0
CHEK1	3	0.072231	1	0	*CYP27B1*	4	0.018535	1	0
ABCD1	4	0.072258	1	0	*ZSWIM1*	3	0.018542	1	0
WDR47	3	0.072286	1	0	*CTSD*	4	0.018565	1	0
RILP	4	0.072308	1	0	*SMIM8*	4	0.018576	1	0
TRIOBP	4	0.072316	1	0	*MAPKBP1*	4	0.018584	1	0
COG1	4	0.072417	1	0	*HOMER1*	4	0.018764	1	0
NCOA4	4	0.072474	1	0	*CRAT*	4	0.018766	1	0
EFHD1	3	0.072539	1	0	*ZC3H7A*	3	0.018768	1	0
ADAM28	4	0.072655	1	0	*SLC25A21*	4	0.018823	1	0
MARK3	4	0.072765	1	0	*LST1*	3	0.018831	1	0
TMEM88	3	0.072883	1	0	*LAD1*	4	0.018857	1	0
WDR73	4	0.072885	1	0	*EHBP1*	4	0.01887	1	0
IFNGR2	3	0.072962	1	0	*NFKBIA*	4	0.018927	1	0
FASTKD3	4	0.073048	1	0	*CDH17*	4	0.018944	1	0
SYVN1	4	0.073208	1	0	*FAM199X*	3	0.018952	1	0
SLC35D3	4	0.073364	1	0	*FOXG1*	4	0.018997	1	0
FAF1	4	0.073419	1	0	*TOR4A*	4	0.01901	1	0
PEPD	3	0.073453	1	0	*CHAC1*	1	0.019147	1	0
LONP2	3	0.073469	1	0	*ADAM19*	4	0.019153	1	0
ISL1	3	0.07352	1	0	*FOXF2*	4	0.019158	1	0
ZRANB3	4	0.073563	1	0	*LYRM9*	4	0.019189	1	0
GGCT	3	0.073648	1	0	*TMEM9B*	1	0.019285	1	0
CLUL1	4	0.073692	1	0	*PIK3CB*	3	0.019302	1	0
LRRC63	4	0.073763	1	0	*ARID3B*	3	0.019355	1	0
CLCN5	3	0.073771	1	0	*PSTPIP1*	4	0.019413	1	0
CRADD	4	0.073917	1	0	*GRB7*	4	0.019436	1	0
LEAP2	4	0.073984	1	0	*LRATD2*	4	0.019534	1	0
TIGD7	3	0.07415	1	0	*SLX4IP*	4	0.01957	1	0
TMC6	4	0.074151	1	0	*MOK*	4	0.019579	1	0
LIG4	4	0.074206	1	0	*PCTP*	4	0.019727	1	0
UBE2D3	4	0.07422	1	0	*EFR3B*	4	0.019779	1	0
TMEM9B	1	0.074403	1	0	*MEN1*	3	0.019806	1	0

MBD3	3	0.074701	1	0	*SPINK1*	4	0.01983	1	0
PIMREG	2	0.07477	1	0	*CUX2*	4	0.019948	1	0
DOK3	4	0.074775	1	0	*SLC22A18AS*	4	0.019996	1	0
HNRNPCL2	1	0.074874	1	0	*SLC22A20P*	4	0.020005	1	0
CCL2	3	0.074952	1	0	*USP30*	4	0.020091	1	0
ZBED6CL	4	0.075113	1	0	*FLVCR1*	3	0.020126	1	0
BRD4	4	0.07513	1	0	*NIPAL2*	4	0.020284	1	0
RASGEF1C	2	0.075194	1	0	*C19orf73*	4	0.020453	1	0
DDX60L	4	0.075428	1	0	*CDCP1*	3	0.020469	1	0
ALAS1	4	0.07548	1	0	*KRTDAP*	4	0.020683	1	0
PGA3	1	0.075632	1	0	*BRAP*	3	0.020714	1	0
NCAPH2	2	0.075639	1	0	*HRC*	4	0.020777	1	0
CADM1	3	0.075649	1	0	*AKR1C1*	1	0.020841	1	0
PHYH	3	0.075663	1	0	*VGLL3*	4	0.020869	1	0
DGCR2	3	0.07576	1	0	*CX3CL1*	3	0.020883	1	0
TLCD2	2	0.075811	1	0	*LYPD5*	4	0.020956	1	0
HERPUD2	3	0.075812	1	0	*ZNF189*	3	0.020963	1	0
TPPP	4	0.075835	1	0	*NAP1L3*	4	0.020965	1	0
ACAD10	3	0.075878	1	0	*RRAGB*	3	0.020988	1	0
SLC25A27	4	0.076032	1	0	*RAB39A*	4	0.021018	1	0
CRABP2	1	0.076137	1	0	*DLGAP3*	4	0.021079	1	0
GAMT	4	0.076164	1	0	*NSMCE2*	3	0.021133	1	0
FGD3	4	0.076182	1	0	*PITPNM3*	4	0.021316	1	0
MCC	4	0.076215	1	0	*KCNK5*	3	0.021418	1	0
PRCD	2	0.076337	1	0	*AXDND1*	4	0.021453	1	0
MSS51	4	0.07646	1	0	*FBXL19*	2	0.021531	1	0
UBE2E2	3	0.07647	1	0	*FAM185A*	2	0.021644	1	0
CWF19L1	4	0.076499	1	0	*MYD88*	4	0.021655	1	0
SPPL3	4	0.076537	1	0	*RELA*	4	0.021664	1	0
SLC26A4	3	0.07664	1	0	*HOXA2*	4	0.021733	1	0
WDR41	3	0.076671	1	0	*TRIB3*	4	0.021834	1	0
FAM199X	3	0.076794	1	0	*EFHD1*	3	0.021915	1	0
WFDC5	3	0.076811	1	0	*ACTG2*	4	0.021999	1	0
TRPM2	4	0.07692	1	0	*HDAC4*	4	0.022076	1	0
CEP70	4	0.076985	1	0	*CCDC89*	3	0.022099	1	0
AKAP10	4	0.077008	1	0	*ANO10*	3	0.022119	1	0
HIGD1A	3	0.077041	1	0	*DNAH1*	4	0.022141	1	0
FAM163B	4	0.077145	1	0	*DISP2*	4	0.022528	1	0
ADPRH	4	0.077165	1	0	*PTPN9*	3	0.022565	1	0
CNNM4	4	0.077199	1	0	*FAM107A*	4	0.022566	1	0
SV2A	4	0.077237	1	0	*MEIS3*	4	0.022608	1	0
PPP1R8	3	0.077266	1	0	*DENND4B*	4	0.022653	1	0
RSRP1	4	0.077316	1	0	*DVL2*	4	0.022689	1	0
UGT1A8	1	0.077343	1	0	*CCDC14*	2	0.0227	1	0
GRAP2	2	0.07735	1	0	*TNFRSF9*	4	0.02274	1	0
PIP5K1A	3	0.077368	1	0	*GOLGA6L2*	3	0.022771	1	0
GPX3	4	0.077507	1	0	*PGAP4*	3	0.022773	1	0
ANAPC4	4	0.077541	1	0	*ZNF615*	2	0.022852	1	0
LRRC61	4	0.077553	1	0	*TNFRSF8*	4	0.022925	1	0
COPA	3	0.077608	1	0	*ZIC2*	3	0.023018	1	0
SS18L2	4	0.077715	1	0	*GPR161*	3	0.023117	1	0
WIPF3	2	0.077742	1	0	*ATP6V1B1*	4	0.02326	1	0
TBPL2	4	0.077771	1	0	*MED22*	4	0.023261	1	0
TBC1D2	4	0.077782	1	0	*TRPV3*	3	0.023277	1	0
CYTL1	4	0.077791	1	0	*ESYT1*	4	0.023319	1	0
PRR29	3	0.077883	1	0	*FITM1*	3	0.02343	1	0
ARMC1	4	0.078047	1	0	*RCAN1*	4	0.023526	1	0
SNX21	4	0.078053	1	0	*TAB3*	3	0.023608	1	0

DCP1B	2	0.078075	1	0	*SMIM10L1*	2	0.023622	1	0
ZBTB22	3	0.078126	1	0	*AKAP7*	4	0.023695	1	0
PLEKHD1	3	0.07813	1	0	*MBLAC1*	4	0.023719	1	0
ANO10	3	0.07825	1	0	*ULK3*	3	0.02375	1	0
PEX5L	3	0.078388	1	0	*LDHA*	4	0.023817	1	0
PCSK1	4	0.078424	1	0	*LRRC8A*	4	0.023853	1	0
C12orf80	3	0.078434	1	0	*DTWD1*	4	0.023883	1	0
BRD3	2	0.078445	1	0	*QPRT*	3	0.023911	1	0
IDH3B	3	0.078488	1	0	*STARD9*	4	0.023949	1	0
RIBC1	3	0.078509	1	0	*RBM26*	2	0.023952	1	0
SLC25A25	3	0.078574	1	0	*BEAN1*	4	0.023969	1	0
PLPP4	4	0.078713	1	0	*SPRY1*	3	0.024001	1	0
NAV2	4	0.078773	1	0	*FBXL7*	4	0.024037	1	0
TMEM41A	4	0.078785	1	0	*NUCB2*	4	0.024039	1	0
CT55	4	0.078887	1	0	*PRKCD*	4	0.024044	1	0
MORN1	4	0.078915	1	0	*ENTPD8*	2	0.024317	1	0
TULP3	3	0.078955	1	0	*ZNF561*	2	0.02432	1	0
QSOX1	3	0.078962	1	0	*ATP6V0E1*	2	0.024365	1	0
YIF1A	4	0.079075	1	0	*MAPK8IP2*	4	0.024406	1	0
HOXB7	4	0.079095	1	0	*SETD3*	4	0.02444	1	0
ZNF606	4	0.079214	1	0	*ZNF782*	4	0.024467	1	0
KDM5B	4	0.079216	1	0	*CXCR4*	4	0.024471	1	0
ARHGEF3	3	0.079225	1	0	*JMJD1C*	3	0.024517	1	0
SLC1A6	4	0.079334	1	0	*LRRC23*	4	0.024677	1	0
ROBO1	4	0.079424	1	0	*ARMC6*	4	0.02468	1	0
TNF	4	0.079469	1	0	*FAM172A*	3	0.024702	1	0
LDHC	4	0.079543	1	0	*MCFD2*	4	0.024743	1	0
PKD1L2	3	0.079547	1	0	*IRF4*	2	0.0248	1	0
C1orf127	4	0.079609	1	0	*UGT3A2*	4	0.025035	1	0
MARCHF2	4	0.07961	1	0	*PPP4R1*	4	0.025107	1	0
BATF3	3	0.079611	1	0	*THRA*	4	0.025231	1	0
CUEDC2	3	0.079647	1	0	*SPNS3*	3	0.025386	1	0
NEMF	4	0.079796	1	0	*CCDC28A*	4	0.025392	1	0
ST7	4	0.079831	1	0	*TNNI1*	4	0.025413	1	0
FAM216A	3	0.079914	1	0	*KRTAP5-9*	3	0.025497	1	0
USP40	2	0.080013	1	0	*DNMT3B*	3	0.025507	1	0
STC1	4	0.080032	1	0	*UBE2Q2*	4	0.025588	1	0
F3	4	0.080063	1	0	*EFHC1*	3	0.025602	1	0
SPTAN1	4	0.080168	1	0	*IGBP1*	4	0.025647	1	0
GOLGA8N	1	0.080184	1	0	*NUDT18*	4	0.025658	1	0
ST6GALNAC2	4	0.080228	1	0	*FAM171B*	4	0.025711	1	0
CPN1	4	0.08024	1	0	*NKAIN2*	4	0.025758	1	0
AIM2	4	0.080242	1	0	*OXNAD1*	4	0.025826	1	0
MYOM2	3	0.080278	1	0	*ZNF488*	3	0.025998	1	0
SKIV2L	4	0.080324	1	0	*SLC1A5*	4	0.026064	1	0
C11orf74	3	0.080357	1	0	*STK16*	4	0.026093	1	0
IGFL1	4	0.080359	1	0	*ABCA7*	4	0.026131	1	0
TRIP12	4	0.080417	1	0	*KCNAB3*	3	0.026164	1	0
CDKAL1	4	0.080462	1	0	*SNED1*	3	0.026185	1	0
ZNF746	4	0.080534	1	0	*MAP4K3*	3	0.026204	1	0
PM20D2	4	0.08055	1	0	*PPP5C*	3	0.026337	1	0
XAF1	3	0.080587	1	0	*PLEKHA3*	1	0.026424	1	0
AP1S3	4	0.080615	1	0	*ZNF557*	3	0.026458	1	0
MCEE	3	0.080617	1	0	*DOCK7*	4	0.026498	1	0
SBK2	4	0.080624	1	0	*PACC1*	4	0.026517	1	0
MEAK7	4	0.080781	1	0	*SMARCB1*	4	0.026526	1	0
FAM81A	3	0.080795	1	0	*GSTP1*	4	0.026531	1	0
ZNF561	2	0.08084	1	0	*E2F7*	4	0.026542	1	0

CCM2L	3	0.080858	1	0	*ZNF423*	4	0.0266	1	0
MPG	4	0.081049	1	0	*ZNF398*	3	0.026872	1	0
ZNF583	4	0.081065	1	0	*ZNF254*	4	0.026893	1	0
MOGAT1	3	0.081135	1	0	*ARR3*	4	0.026985	1	0
SMOC1	4	0.081249	1	0	*ZNF91*	4	0.027003	1	0
NODAL	4	0.081475	1	0	*AMZ2*	4	0.027038	1	0
ZNF569	4	0.081478	1	0	*KCTD12*	4	0.027061	1	0
CSGALNACT2	2	0.081487	1	0	*DOP1A*	4	0.027095	1	0
SIVA1	3	0.081579	1	0	*PLCXD2*	4	0.027208	1	0
OR5P2	3	0.081633	1	0	*SLC39A14*	2	0.027209	1	0
PDE4DIP	2	0.081698	1	0	*IKBKB*	3	0.027239	1	0
C22orf39	1	0.081722	1	0	*GBX2*	4	0.027349	1	0
FZD6	4	0.081921	1	0	*ADAMTS18*	4	0.027376	1	0
LFNG	4	0.081938	1	0	*SH3GL3*	3	0.027414	1	0
GRIN3A	4	0.081951	1	0	*ATE1*	4	0.027528	1	0
GZF1	4	0.082012	1	0	*IGFBP3*	3	0.027535	1	0
TLNRD1	4	0.082017	1	0	*MYLIP*	3	0.027536	1	0
CRTC2	2	0.082059	1	0	*WEE1*	4	0.027583	1	0
TYRP1	4	0.082131	1	0	*CLK3*	4	0.027601	1	0
IQGAP2	3	0.082161	1	0	*ZNF232*	3	0.027677	1	0
SLC38A8	4	0.082261	1	0	*PLEKHO2*	4	0.027694	1	0
ZNF365	4	0.08229	1	0	*NOXRED1*	4	0.027699	1	0
ZNF25	4	0.082311	1	0	*SLC4A5*	4	0.027729	1	0
PPFIA4	4	0.082313	1	0	*SEMA6C*	4	0.02774	1	0
MATK	4	0.082317	1	0	*BRAF*	4	0.027762	1	0
KDELR3	2	0.082449	1	0	*HTRA2*	3	0.027772	1	0
KRT86	2	0.082487	1	0	*ERAP1*	4	0.0278	1	0
MOB1B	4	0.082515	1	0	*RAD54L*	4	0.027811	1	0
TOMM20L	3	0.082535	1	0	*VENTX*	1	0.027829	1	0
KPNA2	3	0.082546	1	0	*MYL6B*	4	0.02783	1	0
RHOT1	3	0.082556	1	0	*TGOLN2*	4	0.028085	1	0
NFIC	4	0.082611	1	0	*PLEKHB2*	4	0.028095	1	0
CYP3A43	3	0.082625	1	0	*ARL6IP4*	4	0.028285	1	0
SF3A3	3	0.082694	1	0	*RBM20*	3	0.02829	1	0
NGFR	3	0.082778	1	0	*APLN*	3	0.028334	1	0
NEXN	4	0.082816	1	0	*SIRT3*	3	0.02836	1	0
TSPYL1	3	0.082897	1	0	*CLDN4*	4	0.028381	1	0
KBTBD3	4	0.08293	1	0	*NRTN*	3	0.02839	1	0
COL10A1	3	0.082948	1	0	*STK19*	4	0.028428	1	0
CPNE4	4	0.083009	1	0	*NIN*	4	0.028478	1	0
GLDC	3	0.083043	1	0	*SPEF2*	3	0.028503	1	0
TOMM6	3	0.083097	1	0	*FAM217A*	3	0.02852	1	0
RPSA	2	0.083166	1	0	*ASCL5*	3	0.028523	1	0
PLPPR2	2	0.083186	1	0	*DENND1B*	4	0.028589	1	0
MBP	4	0.083304	1	0	*CENPU*	3	0.028643	1	0
DSCAML1	4	0.083325	1	0	*CHI3L2*	4	0.028656	1	0
ZFP37	4	0.083331	1	0	*TMEM65*	3	0.02867	1	0
FOXL2	4	0.083483	1	0	*TERF2IP*	4	0.028683	1	0
EXT1	4	0.083532	1	0	*RRP1B*	4	0.028701	1	0
RAB39B	4	0.08355	1	0	*KDM5B*	4	0.028766	1	0
TRIM6-TRIM3[illegible]	1	0.083576	1	0	*MSRA*	4	0.028941	1	0
FUT6	3	0.083792	1	0	*PCDHA12*	4	0.028946	1	0
SERPINB5	3	0.083969	1	0	*LHFPL3*	1	0.029052	1	0
IL1RN	3	0.083976	1	0	*PLA2G4A*	4	0.029152	1	0
IFI16	4	0.083989	1	0	*DMGDH*	2	0.029292	1	0
KCTD12	4	0.084005	1	0	*LRRC27*	4	0.029435	1	0
SLC25A6	4	0.084038	1	0	*LRFN4*	3	0.029529	1	0
PABPN1L	3	0.084154	1	0	*ODF3*	4	0.029531	1	0

IL1R1	4	0.084176	1	0	*LZTS2*	4	0.029543	1	0
CYGB	3	0.084268	1	0	*DLL4*	4	0.0296	1	0
COL1A2	4	0.084279	1	0	*THSD7B*	2	0.029679	1	0
KLHL4	4	0.084284	1	0	*MAP1B*	4	0.029705	1	0
ZNF7	2	0.084288	1	0	*SFXN2*	3	0.02981	1	0
PRMT2	3	0.084337	1	0	*PGGHG*	4	0.02985	1	0
CCDC63	4	0.084419	1	0	*LINC02843*	4	0.029859	1	0
SET	1	0.084472	1	0	*ZNF253*	4	0.029864	1	0
OSCP1	4	0.084581	1	0	*PRR5*	4	0.029883	1	0
FBXL3	4	0.084699	1	0	*CNPY3*	2	0.030002	1	0
ZNF160	4	0.084729	1	0	*ZFYVE19*	4	0.030287	1	0
ELFN1	4	0.08482	1	0	*SLC32A1*	4	0.030299	1	0
HECA	4	0.084917	1	0	*EXOC6B*	4	0.030381	1	0
CRNKL1	4	0.084933	1	0	*NASP*	4	0.030396	1	0
ANKRD61	4	0.084936	1	0	*THSD4*	4	0.03047	1	0
SLC5A6	4	0.084987	1	0	*STAU2*	4	0.030557	1	0
RPRD1A	4	0.085015	1	0	*ARID3A*	3	0.030715	1	0
ZFP3	3	0.085031	1	0	*RBM43*	4	0.030797	1	0
TBC1D20	3	0.085143	1	0	*FAM222A*	3	0.030989	1	0
AP5S1	4	0.085242	1	0	*BAZ1A*	3	0.031079	1	0
ZNF502	2	0.085258	1	0	*UBTD2*	3	0.031163	1	0
RARB	3	0.085308	1	0	*TIMM13*	3	0.031268	1	0
SH3BP5L	4	0.085356	1	0	*STX4*	4	0.031277	1	0
USB1	4	0.08537	1	0	*CAPN6*	4	0.031342	1	0
MRPL53	4	0.085451	1	0	*EFHC2*	4	0.031365	1	0
NUP54	4	0.085523	1	0	*MANBA*	4	0.031398	1	0
DEGS2	4	0.085526	1	0	*BAX*	4	0.031512	1	0
C12orf71	3	0.085576	1	0	*PPP1R10*	4	0.031589	1	0
LAMP1	4	0.085619	1	0	*PI4K2A*	3	0.031685	1	0
DNAJC13	4	0.085644	1	0	*FKBP11*	4	0.031707	1	0
ECM2	3	0.085649	1	0	*EPN1*	4	0.031767	1	0
NOXRED1	4	0.085651	1	0	*HMGB2*	4	0.031782	1	0
CKAP2L	4	0.085749	1	0	*VSTM4*	4	0.031812	1	0
FGF9	1	0.085849	1	0	*DEPP1*	3	0.031826	1	0
CHDH	4	0.085889	1	0	*ALPL*	4	0.031855	1	0
CROT	2	0.085921	1	0	*HHAT*	4	0.031862	1	0
POLB	4	0.085966	1	0	*UBE3D*	3	0.031877	1	0
C20orf141	4	0.086026	1	0	*PPDPF*	4	0.031972	1	0
PDE3B	3	0.086061	1	0	*ACOT7*	4	0.032037	1	0
CRACR2B	3	0.086065	1	0	*ST3GAL3*	4	0.032091	1	0
DLX3	2	0.086065	1	0	*DPCD*	4	0.032183	1	0
AKR1B10	3	0.086158	1	0	*PPM1B*	4	0.032336	1	0
RABL6	4	0.086234	1	0	*NFIC*	4	0.032353	1	0
EPC2	4	0.086264	1	0	*SPN*	4	0.032517	1	0
HEBP1	3	0.086275	1	0	*TPD52*	4	0.032568	1	0
TRIM40	2	0.086374	1	0	*GNB5*	2	0.032594	1	0
PHF20L1	3	0.086454	1	0	*GUCA2B*	4	0.032619	1	0
AMPD3	4	0.08646	1	0	*DHRS2*	4	0.032724	1	0
C5orf63	4	0.086462	1	0	*ZNF532*	4	0.032737	1	0
ZNF98	3	0.086491	1	0	*PC*	3	0.032846	1	0
SP1	3	0.086535	1	0	*DNAJB2*	4	0.032916	1	0
ZNF629	4	0.086675	1	0	*FKBP7*	3	0.033007	1	0
DHFR	1	0.086748	1	0	*WDR54*	4	0.033013	1	0
RAB9B	3	0.086757	1	0	*TSPYL4*	4	0.03303	1	0
RMDN3	3	0.086786	1	0	*ATG7*	4	0.033219	1	0
UBE3A	2	0.087022	1	0	*GBP1*	3	0.033342	1	0
VRTN	4	0.08704	1	0	*FAM71E2*	4	0.033392	1	0
ARHGAP28	4	0.087046	1	0	*IL36RN*	4	0.033558	1	0

SPATA7	4	0.087232	1	0	*KIFC3*	4	0.033714	1	0
NDST3	3	0.087247	1	0	*INO80E*	4	0.033743	1	0
KCTD2	4	0.087259	1	0	*SLC43A2*	4	0.033794	1	0
ZNF362	4	0.087277	1	0	*WNT7B*	4	0.033847	1	0
MIA2	7	0.087304	1	0	*CSF2RB*	4	0.034069	1	0
TSPAN10	4	0.087308	1	0	*TMEM40*	4	0.034075	1	0
PPP3CB	4	0.08731	1	0	*SIGMAR1*	4	0.034249	1	0
TMEM50B	4	0.087393	1	0	*PTPN21*	3	0.0343	1	0
ICE1	2	0.087398	1	0	*AGK*	2	0.034314	1	0
KLF3	3	0.087516	1	0	*FUZ*	4	0.034322	1	0
CASP8	4	0.087535	1	0	*CAPN13*	4	0.034421	1	0
MRPS26	3	0.087613	1	0	*POC1B*	3	0.034492	1	0
SGCA	4	0.087699	1	0	*FHDC1*	4	0.034632	1	0
TUBB4A	4	0.087767	1	0	*CDHR4*	4	0.034668	1	0
TMX4	4	0.087815	1	0	*SLC66A1*	2	0.034687	1	0
DCST1	4	0.087988	1	0	*HOMER3*	3	0.034867	1	0
CDKL2	3	0.088028	1	0	*EXT1*	4	0.034912	1	0
G6PC3	4	0.08807	1	0	*RFX1*	4	0.035042	1	0
RNF14	4	0.08816	1	0	*PKP1*	3	0.035071	1	0
NR2C2	4	0.088237	1	0	*TF*	4	0.035077	1	0
LRRN4CL	4	0.088287	1	0	*BRAT1*	3	0.035133	1	0
KCNMB3	4	0.088379	1	0	*PAAF1*	3	0.0352	1	0
CCDC134	4	0.088517	1	0	*TFDP1*	4	0.035287	1	0
CHPT1	4	0.088543	1	0	*PER3*	4	0.035391	1	0
ALG1L	2	0.08855	1	0	*TMEM119*	2	0.035483	1	0
FAIM	4	0.088646	1	0	*STX1B*	3	0.035503	1	0
HLA-DPB1	3	0.088746	1	0	*FAM118A*	4	0.03553	1	0
TUBB3	4	0.088976	1	0	*EPAS1*	3	0.035676	1	0
BCL6	4	0.089101	1	0	*DEFB124*	4	0.035763	1	0
NRIP3	3	0.089128	1	0	*CARD16*	4	0.035793	1	0
TMEM205	3	0.089169	1	0	*RSKR*	2	0.035827	1	0
RNASET2	4	0.089215	1	0	*SLC10A6*	4	0.035858	1	0
PDIA5	3	0.089248	1	0	*RPS6KL1*	4	0.035913	1	0
SLC30A4	3	0.089248	1	0	*MAGEA1*	4	0.03596	1	0
RXYLT1	4	0.089303	1	0	*RGS20*	3	0.035962	1	0
DNAJC30	4	0.089346	1	0	*OLFML3*	4	0.035963	1	0
IGFL4	4	0.089351	1	0	*BAMBI*	4	0.036022	1	0
CLIC6	4	0.089374	1	0	*NUDT6*	3	0.036145	1	0
KLHL18	3	0.089375	1	0	*ATG14*	4	0.036199	1	0
NYX	4	0.089504	1	0	*LMNB2*	3	0.036249	1	0
PVRIG	4	0.089528	1	0	*ZNF292*	4	0.036499	1	0
RAB38	3	0.089577	1	0	*MBNL2*	1	0.036514	1	0
DAAM1	4	0.089616	1	0	*USF1*	4	0.036554	1	0
SAP130	4	0.089822	1	0	*ZNF496*	4	0.036558	1	0
SLC2A13	2	0.089935	1	0	*CRHBP*	3	0.036584	1	0
LRIG2	4	0.089941	1	0	*GATA2*	3	0.036626	1	0
BTG2	4	0.090091	1	0	*CHMP6*	4	0.036643	1	0
CXCL14	3	0.090103	1	0	*SVIP*	4	0.036751	1	0
FOXK2	4	0.090172	1	0	*VPS50*	4	0.036764	1	0
TECPR1	4	0.090192	1	0	*SLC34A1*	3	0.036809	1	0
SMARCC2	4	0.09025	1	0	*SEC22A*	4	0.036855	1	0
RWDD1	4	0.0904	1	0	*SSNA1*	3	0.036919	1	0
LGI4	4	0.090419	1	0	*HCN2*	4	0.037047	1	0
PRRC2C	4	0.090469	1	0	*DOCK1*	4	0.0372	1	0
TPI1	4	0.090487	1	0	*IDH3G*	4	0.037326	1	0
CAPNS2	3	0.090524	1	0	*WWC1*	4	0.037499	1	0
A1CF	4	0.090559	1	0	*NTN3*	4	0.037542	1	0
ELMO3	3	0.090585	1	0	*FAM227B*	3	0.037558	1	0

FYCO1	4	0.090651	1	0	*SPPL2A*	4	0.037565	1	0
KIFBP	1	0.090743	1	0	*TMEM254*	3	0.037661	1	0
FOXR1	4	0.090752	1	0	*C20orf203*	4	0.037676	1	0
TYW5	3	0.090756	1	0	*MARK4*	4	0.03769	1	0
TTC9	4	0.090793	1	0	*POC5*	4	0.037706	1	0
IDH3G	4	0.09084	1	0	*ROS1*	2	0.037811	1	0
NCALD	4	0.090883	1	0	*PRSS21*	4	0.037837	1	0
ATP8A2	4	0.090905	1	0	*FILIP1L*	4	0.037853	1	0
GUSB	4	0.090908	1	0	*NDRG1*	4	0.037872	1	0
PDLIM3	4	0.090919	1	0	*GIGYF2*	4	0.037909	1	0
RNF26	4	0.090956	1	0	*ITGB1*	4	0.03791	1	0
AGPAT3	4	0.091002	1	0	*DKKL1*	3	0.038028	1	0
DOCK1	4	0.091033	1	0	*LRRN2*	2	0.038031	1	0
LILRB5	4	0.091049	1	0	*TEKT4*	4	0.038252	1	0
HBP1	4	0.091051	1	0	*ECHDC3*	4	0.038286	1	0
GABRD	3	0.091122	1	0	*FN3KRP*	4	0.038295	1	0
ZC3H12C	4	0.091193	1	0	*NAALAD2*	4	0.038298	1	0
MAMDC2	4	0.091197	1	0	*DCAF12*	4	0.038364	1	0
H2AZ2	2	0.091225	1	0	*PINK1*	4	0.038493	1	0
HBQ1	2	0.091241	1	0	*DCAF4*	3	0.038504	1	0
MYL12A	2	0.091439	1	0	*LARGE2*	4	0.038506	1	0
LATS2	2	0.09146	1	0	*CWC27*	3	0.038605	1	0
NGB	4	0.091502	1	0	*SYT12*	2	0.038614	1	0
CPLX3	4	0.091579	1	0	*DGUOK*	4	0.038643	1	0
NKIRAS2	4	0.091582	1	0	*PLA2G4C*	4	0.038671	1	0
CUTA	3	0.091585	1	0	*METTL1*	4	0.038785	1	0
B3GALT4	3	0.091592	1	0	*TNFSF13*	2	0.038858	1	0
NDUFA6	4	0.091611	1	0	*FBXW4*	4	0.03902	1	0
COLEC12	4	0.091685	1	0	*ZC3HC1*	4	0.039127	1	0
LEMD1	1	0.091764	1	0	*CMTM7*	3	0.039177	1	0
DNLZ	4	0.091779	1	0	*TM9SF3*	4	0.039203	1	0
CPSF4L	4	0.091822	1	0	*CTBS*	4	0.039215	1	0
RHNO1	3	0.091853	1	0	*BMERB1*	4	0.039231	1	0
CLSTN1	2	0.091877	1	0	*B3GAT2*	2	0.039303	1	0
ZWINT	3	0.092185	1	0	*AZI2*	3	0.039396	1	0
MFSD14A	4	0.092188	1	0	*TCIM*	4	0.039408	1	0
XKRX	4	0.09223	1	0	*ESCO2*	3	0.039442	1	0
ZFP36L2	4	0.092251	1	0	*ETV5*	4	0.039488	1	0
CMPK2	3	0.092401	1	0	*H1-4*	3	0.039573	1	0
RPA4	3	0.092426	1	0	*EDAR*	4	0.039639	1	0
MON1A	3	0.09247	1	0	*RGL3*	3	0.03966	1	0
GABBR2	4	0.092522	1	0	*LRSAM1*	4	0.039889	1	0
KIF26A	4	0.092581	1	0	*AMH*	4	0.039904	1	0
STXBP2	4	0.092618	1	0	*SLC38A8*	4	0.039985	1	0
MYO16	4	0.092655	1	0	*XKR7*	4	0.04001	1	0
AMPD2	3	0.092722	1	0	*SMG8*	4	0.040027	1	0
TSLP	4	0.092731	1	0	*DIP2B*	4	0.040078	1	0
SEC23A	4	0.092894	1	0	*MIR205HG*	2	0.040092	1	0
C9orf40	4	0.092959	1	0	*TMEM216*	3	0.040148	1	0
IGLL1	4	0.093019	1	0	*MFSD14A*	4	0.040164	1	0
CBLC	4	0.093032	1	0	*SDF2L1*	2	0.04022	1	0
C1orf194	3	0.0932	1	0	*PELI3*	4	0.040292	1	0
C10orf91	4	0.093291	1	0	*GDPD4*	4	0.040395	1	0
YTHDF3	4	0.093321	1	0	*NFASC*	4	0.0406	1	0
PRR11	4	0.093336	1	0	*CFAP97*	4	0.040614	1	0
HLA-DOB	2	0.093352	1	0	*C3orf38*	4	0.04068	1	0
PLIN5	3	0.09337	1	0	*CRTAP*	4	0.040812	1	0
IGHMBP2	3	0.093485	1	0	*CBLIF*	3	0.040827	1	0

PPIL3	4	0.093556	1	0	*SH3RF1*	4	0.04095	1	0
DAP3	4	0.093558	1	0	*FYB1*	2	0.040952	1	0
CRYBB3	4	0.093616	1	0	*TTC17*	3	0.040957	1	0
NR4A1	3	0.093709	1	0	*LNPK*	3	0.040994	1	0
THAP9	4	0.093722	1	0	*SFTA3*	3	0.041011	1	0
TMEM9	4	0.093773	1	0	*RBP4*	4	0.041035	1	0
ATF3	4	0.093831	1	0	*SLC39A3*	4	0.041128	1	0
TLL2	4	0.093897	1	0	*SSTR1*	3	0.041177	1	0
FGR	3	0.093915	1	0	*TATDN2*	4	0.041221	1	0
KIAA0825	4	0.094055	1	0	*C1QTNF3*	1	0.041249	1	0
SMPD3	4	0.094071	1	0	*SNX9*	4	0.041265	1	0
ALG9	4	0.094177	1	0	*XPNPEP1*	3	0.041353	1	0
TENT5C	4	0.094228	1	0	*ALDH6A1*	4	0.041381	1	0
CHST14	3	0.094243	1	0	*HCFC1R1*	4	0.041394	1	0
NUBP1	3	0.094339	1	0	*SLFN14*	3	0.041667	1	0
NAV3	4	0.094405	1	0	*IL33*	2	0.041793	1	0
SMIM19	4	0.094487	1	0	*SETD6*	3	0.041811	1	0
VASN	2	0.094538	1	0	*TOP3A*	4	0.04184	1	0
HLA-DRA	3	0.094579	1	0	*WFDC12*	4	0.041851	1	0
TONSL	4	0.094586	1	0	*UBR7*	4	0.041853	1	0
REN	4	0.094781	1	0	*GULP1*	3	0.042023	1	0
WWP1	4	0.094925	1	0	*PCDHGA8*	4	0.042136	1	0
SULT2B1	4	0.095	1	0	*NOSTRIN*	4	0.04215	1	0
EIF4E3	4	0.095014	1	0	*C2orf15*	1	0.04219	1	0
EPB41	4	0.095026	1	0	*H1-7*	4	0.042247	1	0
COL17A1	1	0.095144	1	0	*ZNF675*	4	0.042303	1	0
SORD	2	0.095154	1	0	*TMEM231*	3	0.042343	1	0
NLRC3	2	0.095207	1	0	*FRAT1*	4	0.042346	1	0
SUPT20HL2	3	0.095252	1	0	*GALNT6*	4	0.042479	1	0
CYP4F12	4	0.095271	1	0	*ACER3*	4	0.042505	1	0
EMC10	3	0.095305	1	0	*RFESD*	2	0.042567	1	0
CLPSL2	3	0.095379	1	0	*HMGN4*	3	0.042665	1	0
NPDC1	4	0.095518	1	0	*TMEM14B*	3	0.042666	1	0
C16orf97	4	0.095571	1	0	*CRTC2*	2	0.042714	1	0
TRRAP	3	0.095606	1	0	*STMN2*	4	0.042749	1	0
SYCE1L	4	0.095684	1	0	*SLC46A1*	3	0.042873	1	0
TRIM62	4	0.0957	1	0	*CELF5*	4	0.042914	1	0
F12	4	0.095742	1	0	*IL7*	4	0.043	1	0
ABHD12	4	0.095748	1	0	*SAFB*	4	0.043028	1	0
NRN1L	4	0.095761	1	0	*SHOC1*	3	0.043058	1	0
ZNF713	2	0.095905	1	0	*FAM49B*	2	0.043158	1	0
MUC13	3	0.095932	1	0	*ZBTB9*	4	0.043458	1	0
HIF1A	4	0.095943	1	0	*GTF2IRD1*	4	0.04347	1	0
WASF1	2	0.096121	1	0	*SLC45A3*	4	0.043472	1	0
SLC1A1	4	0.096191	1	0	*ACADM*	4	0.043684	1	0
STX17	4	0.096193	1	0	*APEX2*	4	0.043877	1	0
GRK5	4	0.09621	1	0	*NPC1L1*	3	0.04389	1	0
MARVELD2	3	0.09621	1	0	*PPIB*	4	0.043953	1	0
HOXC11	4	0.096299	1	0	*GATAD2A*	4	0.044054	1	0
DEGS1	3	0.096432	1	0	*EFCAB9*	4	0.044254	1	0
CHEK2	4	0.096469	1	0	*PDGFRL*	4	0.044258	1	0
RAVER2	3	0.09647	1	0	*IDH3B*	3	0.044276	1	0
ACOT2	4	0.096481	1	0	*SEPTIN12*	4	0.044382	1	0
KLHL21	3	0.096518	1	0	*SCN1B*	3	0.044401	1	0
DOCK3	4	0.096548	1	0	*IL27*	3	0.044507	1	0
GPR173	3	0.09662	1	0	*C19orf48*	4	0.044544	1	0
SGSM3	3	0.096626	1	0	*TDRD10*	4	0.044572	1	0
NTN1	4	0.096678	1	0	*TCTN3*	3	0.044592	1	0

PLEKHJ1	4	0.096693	1	0	*ZSCAN32*	3	0.044621	1	0
PHKA1	3	0.096864	1	0	*P2RX6*	4	0.044645	1	0
HIPK2	4	0.0969	1	0	*BAG3*	3	0.044677	1	0
PLP2	4	0.096928	1	0	*GPALPP1*	4	0.044678	1	0
NDUFAF3	4	0.097008	1	0	*MYEOV*	3	0.044713	1	0
SPOP	2	0.097138	1	0	*RNF26*	4	0.04476	1	0
SERINC2	4	0.097313	1	0	*EHHADH*	4	0.044813	1	0
DNPEP	2	0.097356	1	0	*RTL9*	3	0.044819	1	0
ZAP70	4	0.09737	1	0	*ATG16L1*	4	0.044827	1	0
GRID1	3	0.097393	1	0	*GTDC1*	3	0.044855	1	0
ADGRG6	4	0.097416	1	0	*SEPSECS*	3	0.044908	1	0
ZNF232	3	0.097643	1	0	*PPM1N*	3	0.045	1	0
CCR8	4	0.097839	1	0	*STK39*	3	0.045005	1	0
ZBTB43	3	0.097857	1	0	*ING5*	4	0.045128	1	0
CCDC38	4	0.097886	1	0	*KIF16B*	4	0.045197	1	0
HMCN2	4	0.098029	1	0	*CACYBP*	2	0.045355	1	0
PIP	4	0.098062	1	0	*CCDC86*	3	0.045491	1	0
CCDC24	3	0.09817	1	0	*WAS*	4	0.045492	1	0
LLGL1	4	0.098187	1	0	*CCDC169*	4	0.045548	1	0
ERGIC2	3	0.098226	1	0	*ZMAT1*	4	0.045583	1	0
GNPAT	3	0.098528	1	0	*PEX5L*	3	0.045675	1	0
SGK1	3	0.098577	1	0	*C11orf1*	4	0.04571	1	0
RAB23	3	0.098652	1	0	*CES3*	4	0.045855	1	0
HACL1	4	0.098779	1	0	*GPRC5C*	4	0.045966	1	0
GALR2	2	0.098946	1	0	*REST*	3	0.04597	1	0
CTSS	2	0.098966	1	0	*MAP3K3*	3	0.046045	1	0
SLC22A5	4	0.099152	1	0	*KIAA0513*	4	0.046057	1	0
MRPL30	4	0.099189	1	0	*COL9A3*	4	0.046095	1	0
APOBEC3H	4	0.099215	1	0	*TMPRSS5*	4	0.046159	1	0
RPGRIP1L	3	0.09934	1	0	*VWC2L*	4	0.046331	1	0
SERPINA7	4	0.099443	1	0	*NEURL1*	3	0.046378	1	0
LYPD2	3	0.099445	1	0	*HLX*	1	0.046436	1	0
NPR2	3	0.099457	1	0	*FANCL*	3	0.046444	1	0
NCAPG2	4	0.099578	1	0	*LOC388813*	4	0.046525	1	0
CDC34	4	0.099596	1	0	*LRRC8C*	4	0.046539	1	0
FAM117B	4	0.099708	1	0	*POU5F1*	3	0.04654	1	0
KIRREL1	4	0.099876	1	0	*ZNF587*	1	0.046603	1	0
FLNB	4	0.10007	1	0	*RAD9B*	2	0.046735	1	0
S100A10	4	0.10008	1	0	*AKAP14*	4	0.046769	1	0
ELMO1	4	0.10009	1	0	*FHL2*	4	0.046905	1	0
BRF1	4	0.10012	1	0	*C11orf74*	3	0.046971	1	0
ARNTL	4	0.10013	1	0	*SERPINA7*	4	0.047157	1	0
NDUFS4	4	0.10023	1	0	*TNFRSF10A*	4	0.04719	1	0
ORC6	4	0.1003	1	0	*ACTL8*	4	0.047206	1	0
TAL1	4	0.10034	1	0	*CD200*	4	0.047235	1	0
ITGB5	4	0.10038	1	0	*USP5*	4	0.047285	1	0
ARHGEF12	4	0.10039	1	0	*TBC1D31*	4	0.047308	1	0
SYCE3	4	0.10049	1	0	*PAPOLG*	4	0.047346	1	0
CERS4	2	0.10064	1	0	*PNMA6A*	4	0.047381	1	0
USP35	4	0.10064	1	0	*TPM2*	4	0.04742	1	0
FBXL7	4	0.10074	1	0	*EMC9*	4	0.047433	1	0
ESYT1	4	0.10089	1	0	*RAET1L*	3	0.047471	1	0
SNRNP48	3	0.10089	1	0	*C12orf71*	3	0.047688	1	0
IFITM1	2	0.10122	1	0	*MAGEE1*	4	0.047693	1	0
N6AMT1	4	0.10139	1	0	*TTPAL*	4	0.047776	1	0
NOS2	4	0.10141	1	0	*LRATD1*	8	0.047814	1	0
CCNO	3	0.10148	1	0	*EPB41*	4	0.04785	1	0
VSNL1	2	0.10154	1	0	*GPR35*	4	0.047854	1	0

APOD	3	0.10159	1	0	*DDIT3*	4	0.04795	1	0
APOLD1	2	0.1017	1	0	*TTC31*	4	0.047969	1	0
TM2D3	4	0.10176	1	0	*TUSC1*	4	0.047984	1	0
FOLR3	2	0.10191	1	0	*MED26*	4	0.047998	1	0
TGOLN2	4	0.10195	1	0	*SRSF6*	4	0.048024	1	0
BIRC7	4	0.10197	1	0	*ADGRA2*	3	0.048076	1	0
CLDND2	4	0.10199	1	0	*CHRDL2*	4	0.048121	1	0
VSIG2	3	0.102	1	0	*UCHL1*	4	0.048145	1	0
ATF2	3	0.10201	1	0	*SNAP25*	4	0.048155	1	0
RGS12	4	0.10203	1	0	*GLP2R*	4	0.048218	1	0
CFD	3	0.10209	1	0	*PAIP2B*	3	0.048219	1	0
LOC388780	4	0.10213	1	0	*VEGFA*	4	0.048225	1	0
CGN	4	0.10228	1	0	*PMEPA1*	4	0.048242	1	0
SSUH2	4	0.10234	1	0	*MBD3L1*	4	0.048338	1	0
SLC35C1	4	0.10237	1	0	*C12orf73*	4	0.048444	1	0
RAC1	2	0.1024	1	0	*NKAPL*	4	0.048449	1	0
PROP1	4	0.10241	1	0	*NFKBIB*	2	0.048479	1	0
AQP5	4	0.10244	1	0	*BTBD8*	1	0.048524	1	0
RIPK2	4	0.10249	1	0	*HERC1*	4	0.048556	1	0
BIK	3	0.10253	1	0	*ZDHHC21*	3	0.048574	1	0
MAF1	3	0.10253	1	0	*MTHFR*	3	0.048633	1	0
LRCH1	4	0.10255	1	0	*PRCD*	2	0.048698	1	0
TYW1B	3	0.10273	1	0	*HOXD12*	4	0.048707	1	0
C17orf98	2	0.10285	1	0	*PLEK2*	4	0.048738	1	0
SLC39A9	3	0.10294	1	0	*CYBRD1*	4	0.048773	1	0
EGR2	3	0.10298	1	0	*ZNF644*	1	0.048936	1	0
PIH1D1	3	0.10308	1	0	*IPCEF1*	3	0.049145	1	0
S100A12	4	0.10309	1	0	*ALG8*	2	0.049151	1	0
TMEM72	2	0.1031	1	0	*GLTPD2*	4	0.049175	1	0
CRYBG2	4	0.10314	1	0	*GALNT3*	4	0.049226	1	0
C21orf62	3	0.10325	1	0	*ZSCAN10*	4	0.049234	1	0
CCDC77	4	0.10329	1	0	*PCDHA3*	4	0.049264	1	0
COL18A1	4	0.10333	1	0	*C1RL*	3	0.049302	1	0
RNF113A	4	0.10336	1	0	*CLEC4M*	2	0.04934	1	0
ZNF605	4	0.10341	1	0	*RPH3AL*	4	0.049444	1	0
C11orf24	4	0.10354	1	0	*SLC6A20*	4	0.049448	1	0
CDX1	3	0.10368	1	0	*XPO7*	3	0.049464	1	0
SGPL1	3	0.10373	1	0	*SYT15*	1	0.049515	1	0
AP1AR	3	0.10376	1	0	*NEK2*	3	0.049638	1	0
NEB	3	0.10384	1	0	*ESS2*	4	0.049647	1	0
ACTB	3	0.10389	1	0	*FANCI*	4	0.049757	1	0
FAM114A2	4	0.10395	1	0	*ELMO1*	4	0.049902	1	0
ZC3H10	3	0.10397	1	0	*ZSCAN20*	1	0.049966	1	0
LHX4	2	0.10418	1	0	*HSD17B14*	4	0.050165	1	0
SLC6A15	3	0.10428	1	0	*CSRNP3*	3	0.050205	1	0
ZFAND1	4	0.10433	1	0	*PRAM1*	2	0.050375	1	0
PLCL1	4	0.10449	1	0	*USP42*	3	0.050385	1	0
SNRPA	3	0.1045	1	0	*ADK*	4	0.050552	1	0
QRSL1	4	0.10452	1	0	*MOAP1*	4	0.050593	1	0
DBNDD1	4	0.10456	1	0	*GNG2*	4	0.050655	1	0
H2BW2	4	0.1048	1	0	*CERCAM*	4	0.050668	1	0
BOK	4	0.10487	1	0	*RNF141*	4	0.050669	1	0
CALM1	3	0.10492	1	0	*CAPN12*	4	0.050694	1	0
SLC22A15	3	0.10496	1	0	*UBXN11*	3	0.050703	1	0
ANOS1	3	0.10516	1	0	*LYPLA2*	2	0.050711	1	0
C18orf25	4	0.10534	1	0	*BCL10*	3	0.050931	1	0
FBXO17	3	0.1054	1	0	*CUX1*	4	0.050977	1	0
BRINP1	3	0.10543	1	0	*SLC35C2*	3	0.05104	1	0

IL12RB1	4	0.10543	1	0	*ENGASE*	3	0.051183	1	0
IL11RA	4	0.10551	1	0	*FOXO6*	4	0.051233	1	0
BTBD8	1	0.10556	1	0	*SERPINF2*	4	0.0513	1	0
IWS1	3	0.10563	1	0	*GDF11*	4	0.051431	1	0
ABCB4	3	0.10564	1	0	*PRDM14*	3	0.051555	1	0
NXNL2	4	0.10574	1	0	*FOXF1*	1	0.051625	1	0
RAD51B	4	0.10575	1	0	*ST3GAL1*	3	0.051662	1	0
UTS2R	4	0.10576	1	0	*KLF13*	3	0.051782	1	0
SLCO1C1	3	0.1058	1	0	*CTSE*	3	0.051819	1	0
MYO3B	4	0.10585	1	0	*UFM1*	3	0.051827	1	0
LYL1	3	0.10596	1	0	*NEU1*	4	0.051836	1	0
CCDC13	4	0.10618	1	0	*IL15RA*	3	0.051906	1	0
BDP1	4	0.1062	1	0	*CRMP1*	4	0.051915	1	0
ODF3	4	0.1062	1	0	*LARGE1*	4	0.051918	1	0
SLC25A21	4	0.10624	1	0	*MTRNR2L8*	1	0.051961	1	0
ARL14	4	0.10645	1	0	*RCN2*	3	0.052025	1	0
PPM1B	4	0.10656	1	0	*ANP32C*	3	0.052063	1	0
SPEN	4	0.10659	1	0	*MSLN*	4	0.052185	1	0
WNT8A	3	0.10659	1	0	*CYP2D6*	2	0.052216	1	0
NEDD1	4	0.10665	1	0	*SSH3*	4	0.052262	1	0
VNN2	4	0.10668	1	0	*GSK3A*	3	0.052645	1	0
EMC7	3	0.10695	1	0	*RBM3*	4	0.052708	1	0
MYO19	3	0.10695	1	0	*ANKRD45*	4	0.052778	1	0
RDH12	3	0.10697	1	0	*GGTLC2*	2	0.052782	1	0
PRR14L	3	0.10705	1	0	*CRIPT*	3	0.052797	1	0
KIAA0319L	3	0.10714	1	0	*INHBA*	4	0.052841	1	0
DHRS4L2	2	0.10715	1	0	*SULF1*	4	0.052881	1	0
EPS15	4	0.10719	1	0	*PGM2*	3	0.052883	1	0
MBD6	4	0.1072	1	0	*CLDN6*	2	0.052941	1	0
PARL	4	0.10723	1	0	*KIF15*	3	0.052972	1	0
MPDZ	3	0.10726	1	0	*MID1IP1*	4	0.053076	1	0
BLZF1	4	0.10727	1	0	*RPS6KB2*	4	0.053122	1	0
RAB15	3	0.10742	1	0	*GABPB2*	4	0.053212	1	0
FXYD3	3	0.10752	1	0	*GPCPD1*	4	0.053228	1	0
PLD3	4	0.10756	1	0	*HAAO*	4	0.053264	1	0
SNCB	4	0.10763	1	0	*TSPAN32*	2	0.053288	1	0
SLC66A2	2	0.10767	1	0	*FBXL16*	4	0.053305	1	0
SCD5	4	0.10771	1	0	*UGDH*	3	0.053323	1	0
MTCP1	4	0.10772	1	0	*TMEM191C*	1	0.053343	1	0
SCRIB	4	0.10778	1	0	*NPAP1L*	4	0.053403	1	0
ADA	4	0.1079	1	0	*RABGEF1*	2	0.053416	1	0
LIPJ	4	0.10795	1	0	*CEP192*	4	0.05358	1	0
CEP89	3	0.10796	1	0	*ZNF215*	4	0.053749	1	0
PLXNA1	4	0.10796	1	0	*FREM2*	3	0.053807	1	0
B3GAT1	4	0.10802	1	0	*PIP*	4	0.053831	1	0
MON1B	4	0.10811	1	0	*XPOT*	4	0.053862	1	0
MAP1LC3C	3	0.10812	1	0	*TTYH1*	4	0.053898	1	0
MIA3	3	0.10825	1	0	*PPP1R3E*	3	0.053938	1	0
CHI3L2	4	0.10826	1	0	*ITM2B*	4	0.054062	1	0
ZNF530	4	0.10826	1	0	*HAO2*	4	0.054103	1	0
ZNF611	4	0.10829	1	0	*TMEM74*	4	0.054113	1	0
CBX8	4	0.10831	1	0	*ZMYND10*	2	0.054126	1	0
DRC3	3	0.10837	1	0	*HS3ST1*	3	0.054133	1	0
ZNF470	3	0.10844	1	0	*AFP*	3	0.054163	1	0
STARD10	3	0.10852	1	0	*ZFP2*	4	0.054163	1	0
TTC17	3	0.1087	1	0	*TAS2R10*	4	0.054186	1	0
RWDD4	1	0.10873	1	0	*ADCK1*	3	0.054201	1	0
SLC22A17	4	0.10873	1	0	*NRG4*	4	0.054284	1	0

NHLRC4	3	0.10893	1	0	*SMARCD3*	2	0.054315	1	0
PSMA1	4	0.10894	1	0	*AASS*	4	0.054338	1	0
VTI1B	4	0.10895	1	0	*PAQR7*	4	0.05439	1	0
NUDT22	4	0.109	1	0	*MSL3*	3	0.054531	1	0
KLHL14	3	0.10907	1	0	*SLC4A1AP*	4	0.054538	1	0
NDUFS8	4	0.10909	1	0	*ANO4*	4	0.054552	1	0
SLC46A3	3	0.10915	1	0	*OSBPL8*	2	0.054787	1	0
FPR2	4	0.10922	1	0	*THOP1*	1	0.054822	1	0
DMD	3	0.10932	1	0	*PTGDR2*	4	0.054872	1	0
AMIGO2	4	0.10937	1	0	*TM9SF1*	3	0.054973	1	0
KIF1C	3	0.1094	1	0	*OR7E154P*	1	0.054994	1	0
TRIM17	4	0.10942	1	0	*GLIS3*	4	0.055056	1	0
FNDC11	4	0.10945	1	0	*MCC*	4	0.055218	1	0
PALM3	3	0.10946	1	0	*ASAP2*	1	0.055401	1	0
MBD5	3	0.10953	1	0	*PIGH*	4	0.055404	1	0
RHOXF1	3	0.10955	1	0	*PSORS1C1*	3	0.055426	1	0
RNASE3	3	0.10959	1	0	*ABCA10*	3	0.055439	1	0
SIDT1	3	0.10965	1	0	*MOGAT1*	3	0.055488	1	0
YJEFN3	4	0.10966	1	0	*NRN1*	4	0.055544	1	0
ZNF467	4	0.1097	1	0	*DYNC1H1*	4	0.055723	1	0
MAGI1	4	0.10981	1	0	*HELLS*	2	0.055834	1	0
GIMAP2	4	0.10992	1	0	*ARHGEF17*	2	0.055858	1	0
CREBBP	4	0.11004	1	0	*SYT17*	4	0.056079	1	0
COL7A1	4	0.11006	1	0	*MMP1*	2	0.056118	1	0
INSYN2A	4	0.11008	1	0	*HOXB6*	3	0.056228	1	0
ANKRD36B	4	0.11014	1	0	*SPDYE5*	2	0.056482	1	0
CYP3A5	3	0.11026	1	0	*LY6E*	3	0.056545	1	0
TRHR	4	0.11032	1	0	*VRTN*	4	0.056625	1	0
KLHDC4	3	0.11041	1	0	*GFRA3*	4	0.05666	1	0
PARP10	2	0.11057	1	0	*ASGR1*	4	0.056666	1	0
ATP6AP2	3	0.11061	1	0	*PPFIA1*	3	0.056732	1	0
STRN4	3	0.11063	1	0	*LETM2*	2	0.056735	1	0
VPS50	4	0.11064	1	0	*CFLAR*	4	0.056738	1	0
FAM25A	2	0.11066	1	0	*KLHL26*	2	0.056896	1	0
DACH1	4	0.11075	1	0	*PLBD1*	2	0.056951	1	0
MTIF3	4	0.11075	1	0	*LCP1*	4	0.056973	1	0
UGT3A2	4	0.11076	1	0	*S100P*	4	0.057081	1	0
SGTA	3	0.11081	1	0	*CEP126*	3	0.057126	1	0
DXO	4	0.11096	1	0	*FARP1*	3	0.057135	1	0
FYTTD1	4	0.11101	1	0	*MPZL3*	2	0.057164	1	0
IGF2BP2	4	0.11117	1	0	*ALAS2*	2	0.05731	1	0
CCDC22	3	0.11122	1	0	*MEOX2*	2	0.057351	1	0
XCR1	4	0.11123	1	0	*TRPC6*	4	0.057394	1	0
VSIG8	2	0.1113	1	0	*RASD1*	4	0.057571	1	0
PNMA6A	4	0.11134	1	0	*ZNF518A*	4	0.057604	1	0
GMDS	4	0.11137	1	0	*CAVIN2*	4	0.057605	1	0
SULT4A1	4	0.11154	1	0	*UBE3B*	4	0.057641	1	0
FUNDC1	4	0.11168	1	0	*PKD1L1*	3	0.057683	1	0
IRX6	3	0.11168	1	0	*IGDCC4*	3	0.057686	1	0
UCP3	2	0.11169	1	0	*GOLT1A*	4	0.057723	1	0
VCL	4	0.11169	1	0	*MCUB*	3	0.057761	1	0
PAPSS2	4	0.11179	1	0	*KCTD15*	4	0.057859	1	0
ZNF660	4	0.1121	1	0	*H2AC8*	4	0.057957	1	0
TSFM	4	0.11214	1	0	*P2RY8*	4	0.057958	1	0
WNT5B	3	0.11217	1	0	*C12orf60*	4	0.057975	1	0
POLN	4	0.11222	1	0	*C16orf46*	4	0.05803	1	0
POLE	4	0.1124	1	0	*HACL1*	4	0.058137	1	0
CCL17	2	0.11248	1	0	*SLC12A6*	3	0.058249	1	0

LGALS1	4	0.11253	1	0	*TFAP2C*	4	0.058452	1	0
RIT1	3	0.11258	1	0	*ZNF257*	4	0.05889	1	0
RBKS	3	0.11272	1	0	*SP8*	4	0.058937	1	0
PDXP	3	0.11288	1	0	*PRSS57*	3	0.058946	1	0
ASIC4	4	0.11292	1	0	*UNC5C*	4	0.058951	1	0
RPP21	1	0.11294	1	0	*IFT22*	4	0.059069	1	0
WARS1	3	0.11304	1	0	*VPS35L*	4	0.05908	1	0
CHAMP1	4	0.11306	1	0	*APPBP2*	4	0.059082	1	0
AK3	4	0.11307	1	0	*RPS6KA6*	3	0.059106	1	0
FBXL6	3	0.11315	1	0	*MUL1*	4	0.05913	1	0
BBIP1	3	0.11356	1	0	*GFY*	4	0.059204	1	0
FCRL1	4	0.1138	1	0	*FAM186B*	3	0.059277	1	0
RASA4	1	0.11388	1	0	*SLC18A2*	4	0.059451	1	0
MEOX1	3	0.11397	1	0	*ZFR2*	3	0.059468	1	0
NME6	4	0.11403	1	0	*TAGAP*	1	0.059492	1	0
ABTB2	4	0.11425	1	0	*RIPPLY1*	4	0.059553	1	0
RPH3AL	4	0.11431	1	0	*PLEKHG7*	4	0.059553	1	0
KAT7	3	0.11433	1	0	*SLC43A3*	4	0.05966	1	0
ZNF615	2	0.11433	1	0	*DRAM1*	3	0.059707	1	0
MLF2	3	0.11446	1	0	*ICAM4*	4	0.059773	1	0
PCDHA9	4	0.11448	1	0	*CCDC43*	2	0.059984	1	0
APC2	3	0.11451	1	0	*GPRIN1*	1	0.06001	1	0
TNNT3	3	0.11461	1	0	*UBR3*	4	0.060093	1	0
BANK1	4	0.11487	1	0	*PLAAT4*	4	0.060167	1	0
LGALS4	4	0.1149	1	0	*C9orf131*	3	0.060168	1	0
ISOC1	4	0.1151	1	0	*TK2*	4	0.060172	1	0
FRYL	4	0.11513	1	0	*FAM110C*	2	0.060267	1	0
SAXO1	4	0.11519	1	0	*TMEM222*	4	0.060318	1	0
FUT11	3	0.11521	1	0	*UPP2*	2	0.060449	1	0
FILIP1L	4	0.11528	1	0	*H1-3*	3	0.060581	1	0
MYCL	3	0.11528	1	0	*MARCHF1*	3	0.060663	1	0
ZNF627	4	0.11531	1	0	*MINAR2*	4	0.060731	1	0
COL11A2	4	0.11532	1	0	*HS6ST1*	2	0.060768	1	0
KLHL2	4	0.11539	1	0	*WNK2*	4	0.060862	1	0
TFCP2	4	0.11539	1	0	*R3HDM4*	4	0.060875	1	0
GRASP	4	0.11543	1	0	*FCRL1*	4	0.061092	1	0
FSTL3	4	0.1155	1	0	*ZNF630*	4	0.061213	1	0
PRKAG2	4	0.11551	1	0	*PCMTD2*	4	0.061353	1	0
EBF4	4	0.11557	1	0	*OSMR*	4	0.061389	1	0
GNPNAT1	3	0.11562	1	0	*ACSL4*	4	0.061404	1	0
ATP2C2	3	0.11565	1	0	*ZNF628*	4	0.061538	1	0
TBC1D10B	4	0.11568	1	0	*REXO1*	4	0.061554	1	0
SMTN	4	0.11579	1	0	*HSPA8*	4	0.061575	1	0
RAB12	4	0.11598	1	0	*NBPF10*	2	0.061592	1	0
HADHA	4	0.11599	1	0	*KIAA0040*	4	0.061617	1	0
AMMECR1L	4	0.11603	1	0	*TRNP1*	4	0.061632	1	0
CEP295NL	4	0.11612	1	0	*PDLIM3*	4	0.061633	1	0
C12orf49	4	0.11621	1	0	*SLC37A1*	3	0.061739	1	0
MR1	3	0.11622	1	0	*KLHDC9*	3	0.061826	1	0
PUS1	4	0.11643	1	0	*KLHL21*	3	0.06193	1	0
SSBP4	4	0.11663	1	0	*FDXACB1*	3	0.061958	1	0
CLEC4D	4	0.11676	1	0	*MEGF8*	4	0.061964	1	0
PALM2AKAP2	11	0.11676	1	0	*SMKR1*	3	0.062045	1	0
NYNRIN	4	0.11679	1	0	*RABGAP1L*	4	0.062121	1	0
TMPRSS4	4	0.11679	1	0	*SHISA4*	4	0.062128	1	0
ZUP1	3	0.11679	1	0	*AGBL2*	4	0.062206	1	0
LHB	4	0.11681	1	0	*MSRB3*	4	0.062349	1	0
FAH	2	0.11682	1	0	*TENM1*	4	0.062498	1	0

FAM189A1	4	0.11682	1	0	*ATP11B*	4	0.062563	1	0
PEAK1	4	0.11683	1	0	*CHST5*	4	0.062582	1	0
PRDM5	4	0.11713	1	0	*IFNB1*	3	0.062618	1	0
TGM1	4	0.11721	1	0	*SCN9A*	2	0.062675	1	0
XIAP	4	0.11721	1	0	*MYH7B*	3	0.062807	1	0
ZNF491	4	0.1173	1	0	*LAMA1*	2	0.062823	1	0
CAPN3	4	0.11734	1	0	*XYLB*	4	0.06307	1	0
ALG3	4	0.11735	1	0	*EXT2*	4	0.06309	1	0
TACR2	4	0.11742	1	0	*PDLIM2*	4	0.063139	1	0
ENO2	4	0.11751	1	0	*BNC1*	4	0.063188	1	0
FSCN3	4	0.11756	1	0	*RMI2*	4	0.063189	1	0
WASHC2A	1	0.11764	1	0	*MAP7D3*	4	0.063283	1	0
METTL21A	4	0.11769	1	0	*CHST4*	4	0.063309	1	0
TRO	4	0.11771	1	0	*MFSD2A*	4	0.063375	1	0
RASL11B	4	0.11793	1	0	*ZNF483*	4	0.06339	1	0
SAMSN1	2	0.11801	1	0	*NACC2*	3	0.063404	1	0
SELENOH	3	0.11811	1	0	*IGLON5*	4	0.063611	1	0
GDAP2	4	0.11829	1	0	*VTA1*	3	0.063626	1	0
CTAGE9	1	0.11834	1	0	*BBIP1*	3	0.063805	1	0
GNPDA2	4	0.11834	1	0	*SLC35B3*	3	0.06401	1	0
GOLGA4	3	0.11834	1	0	*TMED4*	4	0.064057	1	0
IER3IP1	4	0.11843	1	0	*ABCC4*	3	0.064147	1	0
ACSM3	4	0.11845	1	0	*KIF7*	4	0.064247	1	0
DDX58	3	0.11854	1	0	*TRPS1*	1	0.064357	1	0
ZC3H14	4	0.11854	1	0	*LRRC42*	4	0.064398	1	0
INO80D	4	0.11859	1	0	*PCM1*	4	0.064423	1	0
IFITM2	4	0.11869	1	0	*MICALCL*	3	0.064467	1	0
SLCO1A2	3	0.1187	1	0	*OSBPL5*	4	0.064531	1	0
ATP4B	4	0.11879	1	0	*SNX19*	3	0.064722	1	0
SLA2	4	0.1188	1	0	*STARD3*	4	0.064846	1	0
SLC25A43	3	0.11882	1	0	*CACNB4*	3	0.064852	1	0
MAP4	2	0.11885	1	0	*PBK*	4	0.065008	1	0
C4orf3	2	0.11886	1	0	*TENM2*	4	0.065041	1	0
IFI27L1	4	0.11886	1	0	*OXT*	4	0.065076	1	0
PIGV	4	0.1189	1	0	*RBM11*	4	0.0651	1	0
GALK2	3	0.11904	1	0	*AQP11*	3	0.065139	1	0
PRRT2	3	0.11914	1	0	*SPSB3*	4	0.065154	1	0
TMEM126B	3	0.11917	1	0	*HRK*	3	0.065215	1	0
RHCE	4	0.11931	1	0	*TBC1D16*	4	0.065362	1	0
M1AP	3	0.11956	1	0	*NT5M*	4	0.065454	1	0
CDC42EP2	4	0.11959	1	0	*PCMT1*	3	0.065517	1	0
DHDDS	4	0.11959	1	0	*ITIH6*	4	0.065604	1	0
ZBTB37	3	0.1196	1	0	*GPR135*	4	0.065664	1	0
ST6GALNAC4	4	0.11962	1	0	*ZNF426*	2	0.065668	1	0
APLP1	4	0.11967	1	0	*CDIPT*	3	0.065728	1	0
HTATSF1	3	0.11975	1	0	*TNNT1*	4	0.06577	1	0
CYFIP2	4	0.11977	1	0	*IGFL2*	3	0.065826	1	0
HMCN1	4	0.11984	1	0	*COMMD9*	3	0.065857	1	0
WDR46	3	0.1199	1	0	*MYL3*	4	0.065928	1	0
BRCA2	3	0.11993	1	0	*UBE2E1*	4	0.066008	1	0
BOLL	4	0.11998	1	0	*CDK15*	4	0.066033	1	0
RAB4A	6	0.12005	1	0	*LTBP2*	2	0.066109	1	0
APPL2	3	0.1201	1	0	*EPC1*	3	0.066131	1	0
RNF146	4	0.12012	1	0	*TMEM185A*	3	0.06619	1	0
HBS1L	4	0.12014	1	0	*IQCF1*	4	0.066238	1	0
C16orf90	4	0.12019	1	0	*SUN3*	3	0.066408	1	0
NUAK2	4	0.12031	1	0	*DEF6*	4	0.066419	1	0
FAAP100	3	0.12034	1	0	*WDR60*	3	0.06653	1	0

MUC15	4	0.12048	1	0	*CARF*	3	0.066602	1	0
PGM3	3	0.1205	1	0	*HPRT1*	4	0.066612	1	0
RSPH6A	3	0.12066	1	0	*HDAC1*	4	0.066633	1	0
SLC7A6OS	4	0.12069	1	0	*ZNF251*	3	0.066654	1	0
RASGRP4	4	0.12073	1	0	*GRID2*	4	0.06667	1	0
DAW1	3	0.12074	1	0	*CYP2A7*	4	0.066729	1	0
MGME1	1	0.12074	1	0	*PSMD5*	4	0.066801	1	0
MFSD6	4	0.12085	1	0	*ZNF263*	4	0.066832	1	0
GAS2L3	4	0.12087	1	0	*CD24*	3	0.06688	1	0
HBEGF	3	0.12095	1	0	*CABCOCO1*	4	0.066904	1	0
IGF1	4	0.12117	1	0	*NFE2L3*	4	0.066952	1	0
C3orf38	4	0.1212	1	0	*SMARCAL1*	4	0.067007	1	0
C6orf62	4	0.12133	1	0	*PABPC1*	4	0.067015	1	0
CD274	4	0.12133	1	0	*FAIM*	4	0.06726	1	0
GRK2	4	0.12148	1	0	*SEZ6L2*	4	0.067283	1	0
LOC643802	2	0.12149	1	0	*PLXND1*	4	0.067297	1	0
METTL3	4	0.12151	1	0	*LAPTM4B*	3	0.067329	1	0
CADPS2	4	0.12157	1	0	*DAPK1*	4	0.067344	1	0
GPR156	3	0.12168	1	0	*TMEM200A*	4	0.067345	1	0
AJUBA	4	0.12173	1	0	*HCAR3*	3	0.067391	1	0
NAE1	4	0.12179	1	0	*IRS2*	4	0.067571	1	0
LAMC3	4	0.12182	1	0	*S1PR2*	4	0.067628	1	0
PITPNC1	2	0.12183	1	0	*TMEM239*	4	0.067914	1	0
HNRNPA3	2	0.12199	1	0	*RAF1*	4	0.068035	1	0
IGLON5	4	0.12208	1	0	*GPR142*	2	0.068062	1	0
DPH6	4	0.12215	1	0	*CALU*	3	0.068121	1	0
CLGN	2	0.12221	1	0	*DPM2*	4	0.06815	1	0
GRIA4	2	0.12227	1	0	*THSD7A*	2	0.06816	1	0
RTTN	4	0.12228	1	0	*NFATC1*	4	0.068167	1	0
ADGRL1	4	0.12229	1	0	*CCT6B*	4	0.068185	1	0
LINS1	3	0.12232	1	0	*SMTN*	4	0.068225	1	0
UTF1	1	0.12232	1	0	*PSG8*	1	0.068257	1	0
TGFB1I1	2	0.12256	1	0	*NUDT16*	3	0.068334	1	0
GP6	4	0.12257	1	0	*CNTNAP3*	2	0.06864	1	0
CCDC85A	4	0.1226	1	0	*H2AC15*	4	0.068728	1	0
SMARCD2	3	0.12263	1	0	*MAML1*	3	0.068784	1	0
FRMPD3	4	0.12267	1	0	*DNASE2*	4	0.068936	1	0
GPR137	4	0.12278	1	0	*ACAA2*	4	0.068985	1	0
ZNF680	3	0.12281	1	0	*ZNF674*	4	0.069012	1	0
SAT2	4	0.1229	1	0	*DOC2B*	3	0.06902	1	0
CALU	3	0.12307	1	0	*FAM122B*	4	0.069074	1	0
YTHDC2	3	0.12309	1	0	*LAMB2*	4	0.069134	1	0
NR5A1	4	0.12311	1	0	*CYB5R3*	4	0.069163	1	0
CLINT1	3	0.12312	1	0	*OR52W1*	3	0.069173	1	0
SLC5A1	3	0.12334	1	0	*AP1M1*	4	0.069187	1	0
ARHGAP21	3	0.12335	1	0	*FUS*	4	0.06927	1	0
REM2	3	0.12346	1	0	*USP35*	4	0.069271	1	0
INPP4B	4	0.12352	1	0	*TRIM45*	4	0.069337	1	0
LY6E	3	0.12352	1	0	*WFDC8*	4	0.06942	1	0
STK26	4	0.12353	1	0	*ZNF84*	4	0.069564	1	0
IGFBP5	4	0.12357	1	0	*QRICH2*	4	0.069591	1	0
INF2	3	0.12359	1	0	*SDC3*	4	0.069719	1	0
CYB5R3	4	0.12361	1	0	*NIPSNAP2*	3	0.069734	1	0
PTGES2	4	0.12361	1	0	*KHSRP*	3	0.06977	1	0
MAP6	4	0.12367	1	0	*SFR1*	3	0.069893	1	0
PPARGC1B	4	0.12376	1	0	*VDAC3*	4	0.069941	1	0
RIOK2	4	0.12377	1	0	*KIAA0556*	4	0.069981	1	0
MMP13	4	0.12378	1	0	*THAP5*	3	0.070083	1	0

CATSPERD	3	0.12404	1	0	*TOM1L2*	4	0.070116	1	0
SIGLEC9	4	0.1241	1	0	*TTBK2*	3	0.070189	1	0
RHBDF2	2	0.12416	1	0	*DLST*	4	0.070521	1	0
OC10192884	4	0.12424	1	0	*MMP20*	4	0.070529	1	0
NDFIP2	2	0.12429	1	0	*FAS*	4	0.070645	1	0
EXOC3L2	4	0.1243	1	0	*SELENOP*	4	0.070794	1	0
KCNE5	4	0.12436	1	0	*COL6A1*	4	0.070988	1	0
ZNF77	4	0.12437	1	0	*TOB1*	4	0.071082	1	0
CDC25A	3	0.12441	1	0	*BMPER*	3	0.071179	1	0
SEPTIN1	3	0.12444	1	0	*DMP1*	3	0.071256	1	0
GLCE	4	0.12472	1	0	*DYRK1B*	4	0.071615	1	0
SFXN5	3	0.12473	1	0	*CHRNA2*	2	0.07172	1	0
COL6A2	3	0.12476	1	0	*TMEM71*	3	0.07173	1	0
KIAA0753	3	0.12476	1	0	*FXYD1*	3	0.071769	1	0
TEX12	4	0.12481	1	0	*ADAM15*	4	0.071815	1	0
STAP2	3	0.12484	1	0	*RILP*	4	0.071822	1	0
TMTC2	4	0.12494	1	0	*TSPAN4*	4	0.07192	1	0
SEMA4F	3	0.12505	1	0	*C20orf197*	3	0.072061	1	0
TYW1	4	0.12505	1	0	*ANKRD13A*	1	0.07208	1	0
HGC6.3	1	0.12514	1	0	*FICD*	4	0.072201	1	0
SNCG	3	0.1252	1	0	*FTSJ3*	3	0.072238	1	0
RASSF9	4	0.12522	1	0	*DHRS13*	4	0.07224	1	0
PIEZO2	4	0.12524	1	0	*CRACR2A*	2	0.072424	1	0
GAK	4	0.12528	1	0	*F2RL1*	4	0.07245	1	0
HSPBP1	4	0.12536	1	0	*PRR14L*	3	0.072525	1	0
AFF3	3	0.12543	1	0	*MCU*	3	0.072572	1	0
PIK3R5	4	0.12548	1	0	*STARD5*	3	0.072617	1	0
TAFA1	4	0.12548	1	0	*BMP2K*	4	0.072619	1	0
SAMD14	3	0.12561	1	0	*BCL2L11*	3	0.073083	1	0
CACNG6	3	0.12568	1	0	*TUBGCP6*	3	0.073165	1	0
CHI3L1	4	0.12569	1	0	*ALDH16A1*	3	0.073176	1	0
PPIL4	4	0.1259	1	0	*SLC6A6*	3	0.073226	1	0
SYNPO	4	0.126	1	0	*C15orf39*	4	0.073237	1	0
ARL5A	3	0.12605	1	0	*TANC2*	4	0.07333	1	0
NLE1	3	0.12609	1	0	*SMCO2*	4	0.073394	1	0
SELL	3	0.12611	1	0	*DNPEP*	2	0.073442	1	0
TMEM131	1	0.12616	1	0	*PCDHGA12*	2	0.073465	1	0
CHRNA7	3	0.12622	1	0	*GPC3*	3	0.073478	1	0
ZNF236	4	0.12622	1	0	*ARHGAP32*	3	0.073558	1	0
MOSPD1	4	0.12624	1	0	*RHOA*	4	0.073578	1	0
SHFL	4	0.12657	1	0	*GGA2*	4	0.073649	1	0
ARMC12	3	0.12667	1	0	*IGSF8*	4	0.073661	1	0
MAML1	3	0.1269	1	0	*NEMF*	4	0.073678	1	0
PRSS2	2	0.12706	1	0	*ITGB6*	4	0.073732	1	0
FAM110D	4	0.12724	1	0	*CAMKV*	4	0.073775	1	0
KLF11	4	0.12737	1	0	*ZNF771*	3	0.073898	1	0
ZFPM2	4	0.12742	1	0	*NEIL1*	3	0.073942	1	0
DPEP1	4	0.12752	1	0	*LOC10050584*	3	0.074003	1	0
DPP8	3	0.12782	1	0	*IKBIP*	4	0.074184	1	0
ZNF568	4	0.12785	1	0	*FOXK2*	4	0.074198	1	0
TRIM41	4	0.12786	1	0	*ZNF470*	3	0.07449	1	0
ARPC1B	3	0.12791	1	0	*NUP50*	2	0.074524	1	0
LTBP2	2	0.12791	1	0	*RPL32*	1	0.07466	1	0
TESMIN	1	0.12795	1	0	*TASP1*	3	0.074718	1	0
MAP4K5	4	0.12804	1	0	*GAMT*	4	0.074786	1	0
CEP112	3	0.12806	1	0	*CADM1*	3	0.074815	1	0
ANAPC15	4	0.1281	1	0	*DHRS3*	4	0.074861	1	0
POMGNT2	3	0.12817	1	0	*USP16*	4	0.07498	1	0

FAM83G	3	0.12818	1	0	*ZBTB8A*	4	0.075007	1	0
RNF182	4	0.12836	1	0	*SNX17*	4	0.075028	1	0
WAPL	3	0.1284	1	0	*ZNF132*	4	0.075062	1	0
NENF	4	0.12856	1	0	*PPARG*	4	0.075091	1	0
MINDY2	4	0.1286	1	0	*TRPM5*	4	0.075163	1	0
NECAP2	2	0.12863	1	0	*ABHD17C*	4	0.075198	1	0
ATG10	4	0.12866	1	0	*CA1*	3	0.075208	1	0
BCL11A	4	0.12868	1	0	*THOC6*	4	0.075292	1	0
TREH	3	0.12883	1	0	*CASP6*	4	0.075323	1	0
STXBP5L	3	0.12884	1	0	*FAM241B*	4	0.075381	1	0
CCDC80	4	0.12887	1	0	*FAM13C*	4	0.075446	1	0
ADGRL2	4	0.12894	1	0	*IRX2*	4	0.075456	1	0
MCOLN1	2	0.12896	1	0	*TBC1D1*	4	0.075495	1	0
NMNAT1	3	0.12901	1	0	*UBXN6*	4	0.075581	1	0
GLI1	4	0.12902	1	0	*NPLOC4*	3	0.075844	1	0
ZNF471	2	0.12902	1	0	*PNMA5*	4	0.075844	1	0
PHGDH	4	0.12906	1	0	*EIF5A2*	4	0.075908	1	0
LAMA3	3	0.12914	1	0	*SORCS2*	3	0.075931	1	0
GPSM3	4	0.12918	1	0	*UBFD1*	3	0.075932	1	0
COPS7B	4	0.12925	1	0	*CXCL8*	4	0.075976	1	0
KAT14	3	0.12933	1	0	*ITIH2*	3	0.076071	1	0
COL13A1	4	0.12945	1	0	*NEMP2*	4	0.076106	1	0
LMBR1	4	0.12945	1	0	*SARS1*	3	0.076182	1	0
CLTC	4	0.12948	1	0	*ZNRF1*	4	0.076187	1	0
ZNF573	4	0.12957	1	0	*LEO1*	3	0.076289	1	0
ATP10A	3	0.12964	1	0	*WNK4*	3	0.076301	1	0
FAM155B	3	0.12978	1	0	*FAM91A1*	4	0.07636	1	0
CRLF3	4	0.12981	1	0	*GALK1*	3	0.076362	1	0
GNL3L	4	0.12983	1	0	*SETD7*	3	0.076451	1	0
WFDC2	4	0.12986	1	0	*DCDC1*	7	0.076462	1	0
UCK2	4	0.13007	1	0	*BCLAF3*	4	0.076478	1	0
LDLRAD3	3	0.13014	1	0	*SS18L1*	4	0.076564	1	0
LONRF1	4	0.13016	1	0	*CNTN1*	3	0.076595	1	0
UPRT	4	0.13016	1	0	*SLC27A5*	1	0.076597	1	0
ANXA5	4	0.13022	1	0	*RHBG*	4	0.076623	1	0
TRIM8	2	0.13028	1	0	*CLCN6*	4	0.076635	1	0
PEBP1	2	0.13061	1	0	*CRYM*	2	0.076662	1	0
DUSP1	4	0.13063	1	0	*IL17RD*	3	0.076666	1	0
C16orf46	4	0.13075	1	0	*SERPINA5*	4	0.07671	1	0
HOXB3	3	0.13078	1	0	*PCED1A*	4	0.07677	1	0
KDM3B	1	0.13078	1	0	*CLMN*	4	0.076774	1	0
AGFG1	3	0.13085	1	0	*MATR3*	4	0.076799	1	0
CCN4	4	0.13085	1	0	*PPP2CA*	4	0.076821	1	0
SH2D3C	3	0.13086	1	0	*LIMK2*	4	0.076831	1	0
PELO	4	0.13087	1	0	*EDIL3*	3	0.076863	1	0
CDK4	4	0.13126	1	0	*CCDC85B*	2	0.077027	1	0
LDHAL6B	3	0.13129	1	0	*PRKCE*	4	0.077106	1	0
NACC2	3	0.13137	1	0	*ALOX5*	4	0.077277	1	0
VMO1	4	0.1315	1	0	*IRAK1BP1*	4	0.077555	1	0
FANCG	4	0.13153	1	0	*ITGAD*	4	0.077556	1	0
SLC35E4	2	0.13153	1	0	*RNF125*	3	0.077623	1	0
ATF1	2	0.13159	1	0	*WNT6*	4	0.077654	1	0
RBM19	4	0.13164	1	0	*ZBTB43*	3	0.077689	1	0
PDF	4	0.1317	1	0	*ARMC10*	2	0.07784	1	0
KRBA2	4	0.13198	1	0	*SC5D*	4	0.078005	1	0
PAXX	3	0.13219	1	0	*BRPF3*	3	0.078037	1	0
ROR1	1	0.13256	1	0	*SLC35C1*	4	0.078051	1	0
CFAP300	4	0.13262	1	0	*SERPING1*	4	0.0782	1	0

MAGI2	4	0.13265	1	0	*MAN1A1*	1	0.078216	1	0
VN1R1	4	0.13266	1	0	*POLR2J2*	4	0.078265	1	0
TIMP3	4	0.13276	1	0	*SLC5A4*	4	0.078297	1	0
CFP	4	0.13295	1	0	*ABHD3*	4	0.07831	1	0
SND1	4	0.13299	1	0	*MYBPH*	3	0.078342	1	0
PSMC5	4	0.1331	1	0	*FBXO21*	4	0.078355	1	0
RHCG	4	0.13316	1	0	*NINJ1*	4	0.078389	1	0
UTP6	2	0.13316	1	0	*OMA1*	4	0.078438	1	0
HYKK	4	0.1333	1	0	*ITGAL*	3	0.078515	1	0
R3HDM1	4	0.13342	1	0	*DUSP10*	4	0.078864	1	0
HCK	4	0.13343	1	0	*ZNF233*	4	0.07892	1	0
SAP30BP	3	0.13345	1	0	*AP4B1*	4	0.079043	1	0
TSG101	4	0.13365	1	0	*FXYD7*	4	0.079131	1	0
SSR1	3	0.13395	1	0	*B3GALNT2*	4	0.079142	1	0
TNFRSF11B	4	0.13397	1	0	*FAM102A*	4	0.079295	1	0
ITPRIPL2	4	0.13408	1	0	*KCNRG*	3	0.079312	1	0
CENPL	4	0.1341	1	0	*RCAN2*	3	0.079352	1	0
ZBTB45	2	0.13422	1	0	*MPV17L*	4	0.079422	1	0
USP5	4	0.13425	1	0	*CD53*	4	0.079531	1	0
ANKAR	4	0.13427	1	0	*DTX4*	4	0.079613	1	0
FHDC1	4	0.1343	1	0	*GYS1*	3	0.079629	1	0
PPP1R12B	4	0.1343	1	0	*ARHGAP26*	4	0.079677	1	0
ZNF514	4	0.13434	1	0	*SIPA1L3*	4	0.07979	1	0
TMEM102	4	0.13441	1	0	*FASTK*	4	0.079798	1	0
FANCC	2	0.13442	1	0	*CXorf56*	4	0.07982	1	0
TXNDC9	3	0.1345	1	0	*NDFIP2*	2	0.079943	1	0
IDI1	3	0.13452	1	0	*UBL7*	4	0.080023	1	0
FAM76B	4	0.13457	1	0	*ITPKC*	4	0.080084	1	0
ACBD6	4	0.13461	1	0	*PCYT1A*	2	0.080122	1	0
PYGM	4	0.13466	1	0	*ATP2C2*	3	0.080174	1	0
LIMA1	3	0.13467	1	0	*MOB1A*	4	0.080221	1	0
KCNA7	4	0.13468	1	0	*GBX1*	4	0.080237	1	0
EFNA1	3	0.13475	1	0	*LIPE*	4	0.08062	1	0
ITGA11	2	0.13482	1	0	*GPATCH1*	2	0.080846	1	0
RASSF3	4	0.13499	1	0	*LZTS3*	3	0.080907	1	0
CACYBP	2	0.135	1	0	*CNGA1*	3	0.08093	1	0
RRS1	2	0.13509	1	0	*POLB*	4	0.08097	1	0
ENGASE	3	0.13512	1	0	*ACE*	2	0.081024	1	0
TFB2M	4	0.13532	1	0	*GPRC6A*	2	0.081057	1	0
LYPLA2	2	0.13555	1	0	*ADM2*	4	0.08106	1	0
IQCB1	1	0.1357	1	0	*DNMT1*	4	0.081116	1	0
EGF	3	0.13571	1	0	*RPEL1*	4	0.081157	1	0
FOXD2	3	0.13573	1	0	*SUPT7L*	3	0.081196	1	0
PLCB1	4	0.13574	1	0	*PCBP4*	3	0.08131	1	0
FAM227B	3	0.13576	1	0	*EDRF1*	4	0.081645	1	0
QTRT1	3	0.13579	1	0	*LRRC6*	4	0.081746	1	0
RBM3	4	0.13584	1	0	*NUP42*	3	0.081799	1	0
ISLR	4	0.13585	1	0	*DHX34*	3	0.081873	1	0
PHLDA1	4	0.13599	1	0	*AOC1*	1	0.081926	1	0
HNF1A	4	0.13601	1	0	*CTNNB1*	3	0.081948	1	0
PRKD1	4	0.13611	1	0	*SERPIND1*	4	0.081997	1	0
TTYH2	4	0.13612	1	0	*GAL3ST2*	4	0.082083	1	0
HSP90AB1	4	0.13616	1	0	*ZNF12*	4	0.082109	1	0
FREM1	3	0.13626	1	0	*ADGRV1*	4	0.082267	1	0
SOGA1	3	0.13638	1	0	*MEX3C*	4	0.082368	1	0
SLC6A4	4	0.13641	1	0	*SECISBP2*	2	0.082416	1	0
ARL2BP	3	0.13643	1	0	*TSPAN10*	4	0.082486	1	0
CYFIP1	4	0.13653	1	0	*ABCB8*	4	0.082538	1	0

EFR3B	4	0.13653	1	0	*CXorf38*	4	0.082653	1	0
ZBTB8A	4	0.13654	1	0	*PRKCSH*	2	0.082654	1	0
TOGARAM2	4	0.13665	1	0	*PCDHGB5*	3	0.08266	1	0
OSMR	4	0.13667	1	0	*H2AC21*	3	0.082693	1	0
MMADHC	4	0.13671	1	0	*NUCKS1*	4	0.082712	1	0
C16orf71	3	0.13694	1	0	*CEBPG*	2	0.082785	1	0
NEPRO	3	0.13705	1	0	*RPUSD1*	4	0.082785	1	0
OSTN	3	0.13708	1	0	*CPNE5*	3	0.082865	1	0
PIGF	3	0.13717	1	0	*CCNI*	4	0.082935	1	0
ADRA1B	3	0.13741	1	0	*MTR*	4	0.083156	1	0
PTDSS2	4	0.13747	1	0	*JKAMP*	4	0.083179	1	0
ICAM5	3	0.13762	1	0	*ATN1*	4	0.08323	1	0
MGA	2	0.13764	1	0	*PDZD3*	4	0.083346	1	0
PCSK4	4	0.13765	1	0	*KCTD7*	4	0.083378	1	0
HDX	4	0.13766	1	0	*GCK*	3	0.083428	1	0
C21orf91	4	0.13775	1	0	*ZNF420*	4	0.083456	1	0
MOK	4	0.13798	1	0	*CCDC122*	4	0.083602	1	0
DIPK1B	4	0.13805	1	0	*PSPH*	4	0.083607	1	0
SVOP	4	0.13809	1	0	*SIX5*	4	0.083646	1	0
CD109	3	0.1381	1	0	*FREM1*	3	0.0838	1	0
ATP6V1F	4	0.13815	1	0	*EID1*	4	0.083892	1	0
ADPGK	3	0.13822	1	0	*SRGAP3*	4	0.084002	1	0
KCNE3	3	0.13837	1	0	*GTF2E1*	3	0.084314	1	0
DLL3	2	0.1384	1	0	*RBPMS*	4	0.084361	1	0
UGT1A4	4	0.13842	1	0	*C18orf25*	4	0.084363	1	0
NCS1	3	0.13843	1	0	*ZNF830*	3	0.084395	1	0
DSCC1	3	0.13855	1	0	*MAP2K6*	4	0.084495	1	0
RNF167	4	0.13884	1	0	*DIPK1B*	4	0.084552	1	0
RAB3A	3	0.13896	1	0	*AK9*	3	0.084619	1	0
UNC93B1	4	0.139	1	0	*SHPK*	4	0.084802	1	0
C2orf76	4	0.13909	1	0	*PRTFDC1*	4	0.084818	1	0
TMED2	4	0.13911	1	0	*GAS2L3*	4	0.084931	1	0
NOXA1	4	0.13932	1	0	*WNT5A*	3	0.085118	1	0
STK33	2	0.13938	1	0	*BRD7*	4	0.085154	1	0
TMEM38B	3	0.13938	1	0	*RNASEH2A*	4	0.0852	1	0
CCDC106	4	0.13962	1	0	*PACS1*	3	0.085283	1	0
ATP9B	3	0.14001	1	0	*TTC21B*	4	0.085376	1	0
ZCCHC7	4	0.14012	1	0	*MSH6*	4	0.085446	1	0
TMEM263	3	0.14019	1	0	*BAZ2A*	2	0.085635	1	0
PACS1	3	0.14027	1	0	*ZNF598*	4	0.085793	1	0
DNAJC1	3	0.14028	1	0	*RAB40AL*	1	0.085956	1	0
RGCC	3	0.14029	1	0	*WNT11*	2	0.08601	1	0
SPAG8	4	0.14041	1	0	*KCNIP3*	3	0.086273	1	0
SYPL1	4	0.14042	1	0	*PPARA*	2	0.086532	1	0
CXADR	4	0.14047	1	0	*DESI2*	3	0.0866	1	0
TBCD	4	0.14059	1	0	*SMAP1*	4	0.086628	1	0
DPH5	4	0.14061	1	0	*RASIP1*	3	0.086664	1	0
ACOXL	3	0.14074	1	0	*DDI2*	3	0.086853	1	0
DUSP23	4	0.14079	1	0	*ACAP3*	4	0.08699	1	0
ABCA1	3	0.1408	1	0	*SH2D5*	4	0.087023	1	0
XRN1	4	0.14082	1	0	*SEPTIN4*	8	0.087037	1	0
CREB5	4	0.14087	1	0	*RNF139*	4	0.087125	1	0
GCH1	4	0.14088	1	0	*NAT16*	4	0.08713	1	0
PARK7	4	0.14088	1	0	*RFFL*	4	0.087138	1	0
ZNF550	4	0.14133	1	0	*PICK1*	4	0.087243	1	0
CYB5D2	3	0.14138	1	0	*NDST3*	3	0.087263	1	0
SLC43A3	4	0.14151	1	0	*NRARP*	4	0.087274	1	0
MRPL43	2	0.14159	1	0	*CCK*	4	0.08728	1	0

HOXB4	4	0.1416	1	0	*AKR1B15*	4	0.087575	1	0
ALAD	3	0.14172	1	0	*DELE1*	4	0.087597	1	0
WDFY2	3	0.1418	1	0	*POM121C*	2	0.087792	1	0
CEACAM16	4	0.14181	1	0	*MROH2A*	3	0.087804	1	0
C5orf30	3	0.14182	1	0	*TSLP*	4	0.087902	1	0
ERVFRD-1	4	0.14187	1	0	*TRPM2*	4	0.087951	1	0
AMDHD1	4	0.1419	1	0	*DDX60L*	4	0.088004	1	0
H2AC21	3	0.14199	1	0	*ANO1*	4	0.088031	1	0
CPSF1	3	0.14201	1	0	*CIITA*	4	0.088064	1	0
GNG5	2	0.14211	1	0	*RRP9*	3	0.088214	1	0
ST14	4	0.14216	1	0	*GNAI2*	4	0.088238	1	0
FEN1	3	0.1422	1	0	*EXOSC3*	2	0.088276	1	0
STPG3	1	0.14222	1	0	*TSN*	4	0.088317	1	0
COMMD8	4	0.14224	1	0	*BICC1*	3	0.088326	1	0
LRRC46	4	0.14235	1	0	*ZFYVE26*	4	0.08854	1	0
PODXL2	3	0.14236	1	0	*H2BC10*	3	0.088604	1	0
PHC1	1	0.14246	1	0	*MCOLN2*	4	0.088618	1	0
UCN	3	0.14248	1	0	*SMIM7*	4	0.08863	1	0
FOLH1B	2	0.14253	1	0	*H2AW*	4	0.088764	1	0
WDR62	3	0.14257	1	0	*DCLRE1A*	4	0.088768	1	0
ODF3B	4	0.14271	1	0	*NTN5*	4	0.088801	1	0
OGA	4	0.14295	1	0	*FBXO47*	4	0.08883	1	0
RNF17	3	0.14296	1	0	*HOXD3*	3	0.088968	1	0
CNP	3	0.14307	1	0	*SLC52A1*	2	0.08905	1	0
SIX5	4	0.14311	1	0	*VWA5B2*	4	0.089173	1	0
ERICH3	2	0.14321	1	0	*HIPK4*	4	0.089413	1	0
MMP3	2	0.14321	1	0	*SLC30A6*	4	0.089497	1	0
SPATA31C2	3	0.14323	1	0	*ERCC8*	3	0.089682	1	0
DEPDC1B	4	0.14327	1	0	*SPTSSB*	2	0.089711	1	0
HRC	4	0.14329	1	0	*SIMC1*	4	0.0899	1	0
OLFM1	4	0.14347	1	0	*TWSG1*	3	0.089905	1	0
CDADC1	4	0.14367	1	0	*LOXL1*	3	0.089911	1	0
DUS1L	4	0.14373	1	0	*ABCC10*	1	0.090113	1	0
PACRGL	4	0.14376	1	0	*HOOK1*	3	0.090182	1	0
SFPQ	4	0.14386	1	0	*MIR1915HG*	1	0.090204	1	0
COPS9	2	0.14394	1	0	*ZC3H10*	3	0.090216	1	0
TPO	4	0.14411	1	0	*H2AB1*	1	0.090262	1	0
FBXO22	3	0.14419	1	0	*HOXB13*	4	0.090475	1	0
POMP	4	0.1443	1	0	*ZNF680*	3	0.090516	1	0
AAMP	4	0.14432	1	0	*ARHGEF10*	4	0.090579	1	0
EZR	3	0.14449	1	0	*MRTFB*	4	0.090758	1	0
TRAPPC2	1	0.1445	1	0	*FARP2*	2	0.090771	1	0
PNPLA1	3	0.14484	1	0	*GAST*	4	0.090961	1	0
RGN	4	0.14493	1	0	*SPATA6L*	3	0.091006	1	0
NEDD4	4	0.14506	1	0	*FAM43A*	4	0.091015	1	0
ATG12	4	0.1451	1	0	*CYTIP*	3	0.091022	1	0
PEX2	4	0.14511	1	0	*BABAM2*	3	0.091053	1	0
CUX2	4	0.14518	1	0	*NPY4R*	2	0.091083	1	0
MAPK8IP2	4	0.14535	1	0	*CLOCK*	4	0.091158	1	0
PPFIA1	3	0.14549	1	0	*ZNF785*	3	0.091365	1	0
CLEC4M	2	0.14569	1	0	*ZCCHC7*	4	0.091424	1	0
KCNIP3	3	0.14572	1	0	*LRFN3*	3	0.091427	1	0
ACKR3	4	0.14578	1	0	*NUP62*	2	0.091431	1	0
C9orf131	3	0.14597	1	0	*SERTAD1*	2	0.091479	1	0
CTTNBP2NL	4	0.14598	1	0	*NSUN7*	4	0.091529	1	0
SDHD	1	0.14616	1	0	*MTOR*	3	0.091563	1	0
RPLP2	3	0.14649	1	0	*LHX1*	3	0.091593	1	0
TMEM67	3	0.14664	1	0	*MYO1H*	3	0.091643	1	0

GPM6A	4	0.14686	1	0	*SAMHD1*	4	0.091788	1	0
IL17REL	4	0.14696	1	0	*LRP8*	4	0.091834	1	0
UBE2N	2	0.14723	1	0	*CBLN3*	3	0.091956	1	0
ABCA13	4	0.14728	1	0	*PADI1*	3	0.09201	1	0
ADGRB1	4	0.14732	1	0	*UNC119*	3	0.092088	1	0
IPPK	4	0.14736	1	0	*TCEA2*	4	0.092124	1	0
PLPP3	4	0.14742	1	0	*SEMA4G*	2	0.092188	1	0
NR1D1	4	0.14749	1	0	*RAB14*	4	0.092206	1	0
TAS2R3	4	0.1476	1	0	*ANKFN1*	4	0.092431	1	0
ATP8B4	2	0.14782	1	0	*ALLC*	4	0.092606	1	0
ALK	2	0.14794	1	0	*HLF*	4	0.092613	1	0
SEC11A	4	0.14796	1	0	*TENT5A*	3	0.092812	1	0
EVI5	4	0.14801	1	0	*PABPC4L*	4	0.093185	1	0
RASA4B	1	0.14812	1	0	*ANKRA2*	2	0.093193	1	0
XCL1	1	0.14815	1	0	*FOXI3*	1	0.093233	1	0
DNAJC5B	3	0.1482	1	0	*ZBED3*	4	0.093243	1	0
HTRA4	4	0.14825	1	0	*ATP6AP1L*	4	0.093302	1	0
TCF7	4	0.14849	1	0	*TNFSF15*	2	0.093394	1	0
ENDOU	1	0.1485	1	0	*EML3*	2	0.093409	1	0
TM9SF3	4	0.1485	1	0	*PRLR*	3	0.093458	1	0
BNIP3L	3	0.14867	1	0	*SLC9B1*	4	0.093475	1	0
CHAT	4	0.14873	1	0	*PGAP3*	3	0.093571	1	0
NBPF20	2	0.14873	1	0	*RGS16*	1	0.093728	1	0
MAB21L2	4	0.14892	1	0	*ZNF30*	4	0.093733	1	0
GPATCH4	4	0.149	1	0	*CUEDC2*	3	0.093832	1	0
HMGN1	1	0.14905	1	0	*GPR153*	4	0.093837	1	0
NTN3	4	0.14911	1	0	*CENPV*	3	0.093852	1	0
ASB6	4	0.14919	1	0	*MORC4*	3	0.093898	1	0
KIAA1217	3	0.14927	1	0	*MARK3*	4	0.093933	1	0
SYNGR3	4	0.14932	1	0	*QSOX2*	4	0.094048	1	0
C1orf100	4	0.14933	1	0	*WARS1*	3	0.094187	1	0
NELFB	3	0.14934	1	0	*ZNF558*	4	0.094273	1	0
SLC2A8	2	0.14936	1	0	*ELOVL6*	3	0.094494	1	0
CYTH3	2	0.14941	1	0	*GRAMD4*	3	0.094715	1	0
HPCA	4	0.14941	1	0	*MUC5AC*	4	0.094725	1	0
CFAP99	4	0.14942	1	0	*CFAP58*	4	0.094733	1	0
C17orf97	2	0.14951	1	0	*GRIN2A*	4	0.094965	1	0
RPS6KA1	4	0.14951	1	0	*PRKAB1*	4	0.095007	1	0
KLRG2	4	0.14957	1	0	*TTLL12*	4	0.095072	1	0
SOCS2	4	0.14976	1	0	*WDR62*	3	0.095078	1	0
RAB9A	2	0.14984	1	0	*SUSD3*	3	0.095234	1	0
TLR2	2	0.14987	1	0	*KIAA1257*	4	0.09541	1	0
EIF6	4	0.14994	1	0	*ZC3HAV1*	4	0.095502	1	0
TMEM183A	4	0.15001	1	0	*ZNF430*	4	0.095609	1	0
YOD1	3	0.15003	1	0	*TESPA1*	4	0.095775	1	0
COPS3	4	0.15008	1	0	*WDR74*	4	0.095893	1	0
CEBPA	4	0.15013	1	0	*RD3*	3	0.095932	1	0
SLC9A4	3	0.15019	1	0	*MESD*	4	0.095958	1	0
C12orf43	2	0.15037	1	0	*HOOK3*	3	0.095992	1	0
OR2AG1	2	0.15037	1	0	*ISLR*	4	0.096033	1	0
CNKSR2	4	0.15046	1	0	*IBTK*	4	0.096109	1	0
MSANTD2	3	0.1505	1	0	*ZNF320*	3	0.096167	1	0
KCNMB1	4	0.15062	1	0	*SOWAHA*	4	0.096243	1	0
ZNF81	3	0.15081	1	0	*LDHD*	4	0.09632	1	0
NOS1AP	3	0.15082	1	0	*NUPR1*	4	0.096405	1	0
MPP7	4	0.15084	1	0	*ATG13*	4	0.096438	1	0
DUS2	3	0.15087	1	0	*TYRO3*	4	0.096457	1	0
DAG1	4	0.15109	1	0	*SLC12A3*	1	0.096691	1	0

UROD	4	0.15109	1	0	*HEPHL1*	4	0.096734	1	0
TUBGCP6	3	0.15131	1	0	*LIMK1*	3	0.096753	1	0
PTGES3	3	0.15134	1	0	*CLPB*	2	0.096781	1	0
C6orf15	3	0.15147	1	0	*PPP1R21*	4	0.097004	1	0
HLA-DRB1	2	0.15151	1	0	*ORAI2*	3	0.097034	1	0
MRPL21	4	0.15152	1	0	*LAYN*	4	0.097067	1	0
ARHGAP35	3	0.15156	1	0	*COTL1*	4	0.09712	1	0
RABGGTB	3	0.15169	1	0	*ITGB3*	4	0.097147	1	0
HPGD	4	0.15173	1	0	*CCKBR*	3	0.097149	1	0
ZMYND15	4	0.15186	1	0	*PPME1*	3	0.09718	1	0
ZBTB49	4	0.15193	1	0	*ARFIP2*	4	0.097445	1	0
MRPL27	4	0.15202	1	0	*ZNF710*	3	0.097553	1	0
SELPLG	4	0.15226	1	0	*ZNF223*	3	0.097667	1	0
EIF1	2	0.15228	1	0	*ISG15*	3	0.097667	1	0
MACROH2A1	2	0.15228	1	0	*SIRT6*	3	0.097832	1	0
RGS9BP	3	0.1525	1	0	*TMEM205*	3	0.097884	1	0
STXBP6	4	0.15254	1	0	*TSEN34*	3	0.098008	1	0
ARF4	3	0.15256	1	0	*REEP6*	3	0.098098	1	0
MEX3D	3	0.15272	1	0	*CD207*	4	0.098181	1	0
OGFOD2	4	0.15291	1	0	*SEMA4F*	3	0.09837	1	0
NECTIN3	3	0.15292	1	0	*NPSR1*	4	0.098562	1	0
AGBL5	2	0.15301	1	0	*RNF6*	4	0.098626	1	0
ZGLP1	4	0.15303	1	0	*KRT8*	2	0.098769	1	0
FAM83F	3	0.15329	1	0	*ZPLD1*	4	0.098986	1	0
B4GALNT1	4	0.15354	1	0	*LOC10050554*	4	0.099032	1	0
FABP4	3	0.1536	1	0	*RRM2*	8	0.099062	1	0
HEY2	4	0.15391	1	0	*NCS1*	3	0.099196	1	0
L3HYPDH	3	0.15393	1	0	*ACTR3B*	4	0.099653	1	0
SYCP2	4	0.15399	1	0	*VGLL4*	3	0.099774	1	0
SLC25A47	2	0.15445	1	0	*SLA2*	4	0.099823	1	0
DHX9	3	0.15455	1	0	*SUOX*	3	0.10001	1	0
GPR4	3	0.15492	1	0	*CALCB*	4	0.10008	1	0
C8orf82	3	0.15494	1	0	*CAPN5*	3	0.10016	1	0
P2RY1	4	0.15502	1	0	*PIGT*	3	0.1004	1	0
CCNT1	3	0.15525	1	0	*RANBP6*	4	0.10045	1	0
VSTM1	3	0.15532	1	0	*TESK2*	4	0.10047	1	0
ALKBH3	3	0.15533	1	0	*CD300LG*	4	0.1005	1	0
SFN	3	0.1554	1	0	*BRD2*	2	0.10053	1	0
MICAL3	4	0.15549	1	0	*ARHGAP12*	3	0.10065	1	0
OR4D1	4	0.15558	1	0	*KLHL7*	3	0.1007	1	0
SLC5A7	4	0.15565	1	0	*MVB12B*	4	0.10071	1	0
FZD7	2	0.15571	1	0	*FXN*	4	0.10073	1	0
PRSS23	4	0.15578	1	0	*SPTLC3*	4	0.10089	1	0
COPS7A	4	0.15579	1	0	*TLR9*	4	0.10104	1	0
B3GNT8	3	0.15581	1	0	*SMIM4*	2	0.10116	1	0
NTN5	4	0.15587	1	0	*HOXA11*	4	0.10118	1	0
ARMC2	4	0.15607	1	0	*H2BC8*	4	0.10129	1	0
LAPTM4B	3	0.15616	1	0	*ZNF541*	4	0.10134	1	0
SLC25A2	2	0.15616	1	0	*STX5*	4	0.10141	1	0
CDKN2C	3	0.15617	1	0	*CNR2*	3	0.10153	1	0
GRIN2D	2	0.15634	1	0	*OSER1*	3	0.10154	1	0
OXSR1	3	0.15634	1	0	*RESF1*	4	0.10156	1	0
PEX19	4	0.15642	1	0	*HOOK2*	4	0.10162	1	0
CXCL3	3	0.15646	1	0	*TMX4*	4	0.10171	1	0
FAM98C	4	0.15646	1	0	*CYTL1*	4	0.10177	1	0
GDF1	2	0.15681	1	0	*PWWP3A*	3	0.10182	1	0
IZUMO1	3	0.15683	1	0	*CACHD1*	4	0.10187	1	0
PTPRE	3	0.15687	1	0	*PLEKHS1*	3	0.10191	1	0

RBM4B	4	0.15696	1	0	*PLEKHA1*	3	0.10235	1	0
DOC2B	3	0.15698	1	0	*BAG6*	4	0.10237	1	0
LRP2	4	0.157	1	0	*SPTSSA*	4	0.10255	1	0
ADIRF	4	0.15705	1	0	*ATP11AUN*	3	0.10261	1	0
KLF2	3	0.15729	1	0	*CAV1*	4	0.10277	1	0
PLAC4	3	0.15737	1	0	*RBM44*	4	0.10285	1	0
FGF3	3	0.15745	1	0	*SP5*	1	0.10287	1	0
RAB11A	3	0.1575	1	0	*CMIP*	2	0.1029	1	0
ALDH3B2	4	0.15755	1	0	*LTBR*	3	0.10306	1	0
SPTLC3	4	0.15763	1	0	*ADGRG3*	4	0.1031	1	0
RUNDC1	4	0.15781	1	0	*DNALI1*	4	0.1031	1	0
ALG8	2	0.15782	1	0	*GEN1*	3	0.10311	1	0
CXorf38	4	0.15784	1	0	*EYA3*	4	0.10318	1	0
SPHK2	4	0.15786	1	0	*MYO1D*	4	0.10325	1	0
HNRNPA0	4	0.15793	1	0	*DOCK10*	3	0.10334	1	0
JDP2	4	0.15794	1	0	*FAM120A*	4	0.10338	1	0
ABCF1	4	0.15797	1	0	*RAB2B*	3	0.10341	1	0
FAM214A	3	0.15824	1	0	*SLC18A3*	2	0.1035	1	0
METTL1	4	0.1583	1	0	*SWI5*	4	0.1035	1	0
BCO2	3	0.15835	1	0	*PGAM4*	2	0.10353	1	0
CYP20A1	4	0.15835	1	0	*GPR17*	4	0.10358	1	0
ORMDL2	4	0.15837	1	0	*HOXC8*	3	0.10358	1	0
P4HB	2	0.15837	1	0	*MBOAT1*	3	0.10366	1	0
TBC1D3B	1	0.1584	1	0	*CDH11*	4	0.10367	1	0
SEL1L3	2	0.15843	1	0	*ARHGEF3*	3	0.1037	1	0
SLC30A1	1	0.15853	1	0	*FOXD4L4*	1	0.10382	1	0
RAD9B	2	0.15863	1	0	*CTAGE9*	1	0.1042	1	0
GOLGB1	3	0.15866	1	0	*S100A2*	4	0.1042	1	0
ROBO3	4	0.15894	1	0	*CXCL11*	4	0.10424	1	0
CR2	3	0.15903	1	0	*TFEC*	3	0.1043	1	0
SUPV3L1	2	0.15908	1	0	*SERINC2*	4	0.10443	1	0
PLA2G2A	4	0.15923	1	0	*CCDC57*	3	0.10446	1	0
HRNR	4	0.15924	1	0	*GABRD*	3	0.10453	1	0
RANBP17	4	0.15924	1	0	*KHDC4*	4	0.10455	1	0
CASP6	4	0.15963	1	0	*ZFP14*	4	0.10456	1	0
SLC45A2	4	0.15973	1	0	*SMAD7*	1	0.10485	1	0
TM4SF18	1	0.15977	1	0	*DTX1*	2	0.10489	1	0
VAPB	3	0.15982	1	0	*NPTXR*	4	0.10493	1	0
DEF8	4	0.1603	1	0	*TMUB1*	3	0.10493	1	0
TRPC6	4	0.1603	1	0	*EPHB1*	4	0.1051	1	0
HELB	4	0.16034	1	0	*ZNF836*	4	0.10519	1	0
ZNF286B	3	0.16036	1	0	*ESPN*	4	0.1052	1	0
BBS1	2	0.16043	1	0	*ZC3H13*	4	0.10522	1	0
MMP24	4	0.16062	1	0	*APPL1*	4	0.10528	1	0
TMEM184B	3	0.16075	1	0	*ARHGEF38*	4	0.1053	1	0
CES4A	4	0.16101	1	0	*PEX11A*	4	0.1053	1	0
ATN1	4	0.16105	1	0	*ADAMTS5*	3	0.1054	1	0
KIF9	4	0.16107	1	0	*B3GALT6*	4	0.10544	1	0
MFNG	3	0.16111	1	0	*SLC1A6*	4	0.10566	1	0
ZFP82	3	0.16118	1	0	*NBEAL1*	4	0.10567	1	0
ZNF319	3	0.16137	1	0	*MRGPRD*	4	0.10568	1	0
B9D2	4	0.16152	1	0	*PCNX4*	2	0.10577	1	0
GRIN2A	4	0.16159	1	0	*SH3GL1*	3	0.10605	1	0
SEMA3G	4	0.16166	1	0	*GMDS*	4	0.10609	1	0
BCAS3	4	0.16176	1	0	*FAM209A*	1	0.10615	1	0
USP17L15	1	0.16179	1	0	*ATP6AP1*	4	0.10633	1	0
MCIDAS	4	0.1619	1	0	*SPIN1*	4	0.10638	1	0
H6PD	4	0.16197	1	0	*ZKSCAN3*	4	0.10638	1	0

PCOLCE	4	0.16198	1	0	*HTR3A*	4	0.10656	1	0
FFAR3	1	0.16209	1	0	*COX6A1*	2	0.10657	1	0
IRF2BPL	4	0.16215	1	0	*STAC3*	4	0.10664	1	0
SLC12A3	1	0.16223	1	0	*ZBTB38*	3	0.10672	1	0
LARGE1	4	0.16242	1	0	*PPP1R3C*	3	0.10683	1	0
UAP1	4	0.16246	1	0	*SPRY4*	3	0.10686	1	0
SURF4	2	0.16256	1	0	*CCSAP*	4	0.1071	1	0
TTLL7	4	0.16256	1	0	*FNDC9*	3	0.10742	1	0
BET1L	3	0.16258	1	0	*SYNJ1*	4	0.10749	1	0
REX1BD	2	0.16261	1	0	*LRRC8D*	4	0.1076	1	0
CEP126	3	0.16266	1	0	*DUSP14*	4	0.1078	1	0
HVCN1	4	0.16268	1	0	*RAB39B*	4	0.10781	1	0
MEGF8	4	0.16271	1	0	*CADPS*	4	0.10782	1	0
PRF1	3	0.16275	1	0	*EMC8*	4	0.10785	1	0
SKA3	3	0.16278	1	0	*PSMD13*	4	0.10805	1	0
PIGU	3	0.16282	1	0	*TNFRSF11B*	4	0.10805	1	0
ANO1	4	0.16307	1	0	*EIF3B*	3	0.10812	1	0
CD248	4	0.16315	1	0	*MAP3K13*	2	0.10836	1	0
FAM151A	3	0.16319	1	0	*DNAJC27*	3	0.10859	1	0
INAFM2	4	0.16322	1	0	*FBXO33*	4	0.10859	1	0
AIF1L	2	0.16329	1	0	*BTC*	3	0.10869	1	0
CCN1	4	0.16338	1	0	*RSF1*	4	0.10892	1	0
CCDC89	3	0.16342	1	0	*SYTL5*	3	0.1091	1	0
NTM	3	0.16354	1	0	*PHIP*	3	0.10911	1	0
TSPAN33	3	0.16356	1	0	*ESAM*	4	0.10912	1	0
SNRNP40	4	0.16367	1	0	*TINAG*	4	0.10914	1	0
DDX55	3	0.16378	1	0	*F8*	4	0.10934	1	0
C4orf46	3	0.16419	1	0	*TAP2*	4	0.10942	1	0
AK4	3	0.1642	1	0	*RGCC*	3	0.10948	1	0
SLC30A7	4	0.16434	1	0	*PRKCZ*	2	0.10956	1	0
MIDN	4	0.16438	1	0	*NME9*	3	0.10965	1	0
UBE2Q2L	1	0.16438	1	0	*ORMDL2*	4	0.10981	1	0
CTSZ	4	0.16442	1	0	*EFNA3*	4	0.11006	1	0
SAMD10	3	0.16443	1	0	*CXorf40A*	2	0.11021	1	0
NAALAD2	4	0.16457	1	0	*TPRG1L*	4	0.11024	1	0
MPZ	4	0.16458	1	0	*TMEM106B*	3	0.11034	1	0
TMUB2	4	0.16476	1	0	*ZAR1L*	4	0.1104	1	0
CNTNAP3	2	0.16484	1	0	*TBX2*	2	0.11043	1	0
POU2F2	2	0.16484	1	0	*ADAP2*	4	0.11046	1	0
ZNF454	4	0.16491	1	0	*SMIM25*	3	0.11058	1	0
TEX46	3	0.16492	1	0	*S1PR3*	4	0.11067	1	0
NACA	3	0.16519	1	0	*SFTPA2*	3	0.11068	1	0
LRP1	4	0.16528	1	0	*C5orf15*	4	0.11094	1	0
SHROOM1	3	0.1653	1	0	*HEATR4*	4	0.11102	1	0
ZNF251	3	0.1656	1	0	*RTKN*	4	0.11109	1	0
IL27RA	4	0.16567	1	0	*BCL7A*	4	0.11132	1	0
FBXO9	4	0.16573	1	0	*BCL7B*	3	0.11161	1	0
DENND11	2	0.16588	1	0	*EIF2AK4*	4	0.11177	1	0
DPM1	4	0.16591	1	0	*PNRC2*	2	0.11181	1	0
KMT2D	4	0.16595	1	0	*TMEM176A*	4	0.11197	1	0
USP19	4	0.16596	1	0	*SORBS2*	4	0.11198	1	0
AGAP3	4	0.16602	1	0	*ITGA2B*	4	0.11203	1	0
MISP3	3	0.16614	1	0	*IL1F10*	3	0.11249	1	0
LEFTY1	3	0.16619	1	0	*LUC7L*	3	0.11254	1	0
ZNF524	4	0.16619	1	0	*GPSM1*	4	0.11255	1	0
THAP2	3	0.16631	1	0	*HACE1*	4	0.11257	1	0
KIF21A	4	0.16633	1	0	*TMEM45B*	3	0.11258	1	0
F11R	4	0.16637	1	0	*C18orf21*	3	0.1126	1	0

MOXD1	4	0.16638	1	0	*ETV3*	4	0.1126	1	0
EXOSC5	4	0.16643	1	0	*NSD2*	3	0.11265	1	0
METTL15	2	0.16657	1	0	*TRMT61B*	3	0.11278	1	0
CREM	4	0.16659	1	0	*CDC42EP4*	4	0.11291	1	0
TIMM13	3	0.16684	1	0	*CXADR*	4	0.11292	1	0
TSPAN5	4	0.16684	1	0	*NPC2*	3	0.11307	1	0
ZNF267	2	0.1669	1	0	*SIAH1*	4	0.11316	1	0
MTA3	1	0.16715	1	0	*PPP1R35*	1	0.11339	1	0
DPM3	4	0.16717	1	0	*THSD1*	3	0.1134	1	0
SCG2	2	0.16719	1	0	*HNRNPD*	3	0.1135	1	0
SP2	3	0.16736	1	0	*QTRT1*	3	0.11372	1	0
SPRY3	4	0.16737	1	0	*FOXL1*	2	0.11382	1	0
HSPB1	2	0.1674	1	0	*ZNF260*	3	0.11398	1	0
DDX56	4	0.1676	1	0	*RELL1*	4	0.11401	1	0
TRAK1	2	0.16798	1	0	*AIM2*	4	0.11408	1	0
CBWD6	2	0.16807	1	0	*IMAP1-GIMA*	2	0.11412	1	0
GDF11	4	0.16808	1	0	*CALML6*	3	0.11416	1	0
TMEM106B	3	0.16823	1	0	*LEPROTL1*	4	0.11435	1	0
MAPRE1	2	0.16843	1	0	*OR2A1*	1	0.11438	1	0
COTL1	4	0.16874	1	0	*DNAH12*	4	0.11449	1	0
SHLD2	2	0.16894	1	0	*ZEB1*	4	0.11457	1	0
CFLAR	4	0.16904	1	0	*CRTAC1*	4	0.11461	1	0
ENOSF1	4	0.16905	1	0	*LIX1L*	4	0.11465	1	0
KIFAP3	4	0.16918	1	0	*POPDC2*	4	0.11466	1	0
THBD	4	0.16921	1	0	*KIAA1324*	3	0.11469	1	0
ZNF410	4	0.16961	1	0	*C3*	4	0.1149	1	0
GDI1	4	0.16981	1	0	*NEDD4*	4	0.115	1	0
PLEKHA6	4	0.16987	1	0	*CHST1*	3	0.11517	1	0
HDAC10	4	0.16992	1	0	*HLA-F*	4	0.11551	1	0
GPR17	4	0.16995	1	0	*SRP14*	3	0.11554	1	0
RCCD1	3	0.17022	1	0	*HLA-E*	4	0.11557	1	0
RPL36AP37	1	0.17068	1	0	*MARK2*	4	0.11558	1	0
SLC25A16	4	0.1709	1	0	*C1orf105*	3	0.11559	1	0
SLC12A9	3	0.17091	1	0	*TSPAN15*	3	0.1156	1	0
N4BP3	4	0.17095	1	0	*RPA4*	3	0.11561	1	0
HMCES	4	0.17101	1	0	*UQCRH*	1	0.11565	1	0
ZNF839	4	0.17101	1	0	*DUSP19*	3	0.11565	1	0
SNHG28	2	0.17139	1	0	*NRBP2*	4	0.11566	1	0
KAT6B	3	0.17145	1	0	*LYG2*	4	0.11566	1	0
PDCD2	3	0.17147	1	0	*C1orf162*	4	0.11582	1	0
FAM178B	4	0.17161	1	0	*EDEM1*	3	0.11586	1	0
BSN	4	0.17164	1	0	*HSPB11*	4	0.11623	1	0
PEX7	4	0.17175	1	0	*FAXC*	3	0.11635	1	0
DEPDC1	3	0.17188	1	0	*ADNP2*	3	0.11706	1	0
PDE4C	4	0.17202	1	0	*AKT3*	3	0.11709	1	0
WDCP	4	0.17202	1	0	*NTNG2*	4	0.11711	1	0
SLC35E2B	3	0.17203	1	0	*TRIM14*	3	0.11713	1	0
ADAMTSL5	4	0.17226	1	0	*SOX9*	3	0.11719	1	0
LAMTOR5	3	0.17236	1	0	*GPSM3*	4	0.11725	1	0
RGS4	4	0.17237	1	0	*ENTPD2*	3	0.11729	1	0
CHST5	4	0.17244	1	0	*CTH*	3	0.11755	1	0
MFGE8	1	0.17263	1	0	*RASSF2*	4	0.11772	1	0
CKS1B	2	0.17273	1	0	*MRGPRX3*	4	0.11788	1	0
CISD3	4	0.17275	1	0	*SLC38A10*	4	0.11804	1	0
NELFCD	4	0.17291	1	0	*FAM161B*	4	0.11816	1	0
CD7	3	0.17294	1	0	*ISCA2*	4	0.11816	1	0
RBBP9	3	0.17332	1	0	*CFAP20*	3	0.11829	1	0
ZDHHC18	3	0.17334	1	0	*SNHG28*	2	0.11829	1	0

C11orf80	4	0.17351	1	0	*LOC643802*	2	0.11863	1	0
PTGER4	3	0.17364	1	0	*PHACTR1*	2	0.11873	1	0
TEDC2	2	0.17381	1	0	*CEACAM6*	2	0.11887	1	0
TIE1	2	0.17391	1	0	*TMEM198*	3	0.11895	1	0
SLC35F6	2	0.17401	1	0	*ZBTB1*	3	0.119	1	0
ZNF621	4	0.1741	1	0	*HELZ2*	4	0.11904	1	0
NOM1	2	0.17415	1	0	*GJB4*	4	0.11914	1	0
MPZL3	2	0.17439	1	0	*HPS4*	3	0.11924	1	0
RMDN1	2	0.17455	1	0	*PARL*	4	0.11924	1	0
SAG	4	0.17457	1	0	*SRA1*	3	0.11952	1	0
CANX	3	0.17496	1	0	*GSK3B*	4	0.11957	1	0
ZNF703	3	0.17496	1	0	*TMEM135*	3	0.12017	1	0
TRIM22	3	0.17507	1	0	*FAXDC2*	1	0.12034	1	0
CD37	2	0.17511	1	0	*CYP4F11*	4	0.12036	1	0
SHH	4	0.17528	1	0	*BCL3*	3	0.1206	1	0
COBLL1	4	0.17534	1	0	*MBOAT4*	4	0.1206	1	0
PKNOX1	4	0.17539	1	0	*SOCS3*	3	0.12062	1	0
SEM1	8	0.1755	1	0	*PKP2*	4	0.12069	1	0
FOS	1	0.17554	1	0	*FAM178B*	4	0.12069	1	0
UBQLN1	3	0.17554	1	0	*GBP2*	3	0.12076	1	0
AK1	4	0.17562	1	0	*AP4M1*	3	0.12091	1	0
TMEM239	4	0.17564	1	0	*PSAP*	4	0.12102	1	0
GRB14	3	0.17565	1	0	*DTWD2*	3	0.12103	1	0
TRAIP	4	0.17565	1	0	*WDFY1*	3	0.12115	1	0
CYCS	3	0.17595	1	0	*ARL6*	4	0.12119	1	0
DCLRE1A	4	0.17601	1	0	*AKAP6*	4	0.12123	1	0
IFT88	4	0.17604	1	0	*TSPYL6*	4	0.12135	1	0
ARFGEF1	4	0.17605	1	0	*XAF1*	3	0.12184	1	0
DHTKD1	4	0.17606	1	0	*GPR157*	4	0.12194	1	0
DLGAP4	2	0.17628	1	0	*PXDNL*	3	0.12195	1	0
AMH	4	0.17644	1	0	*TP63*	4	0.12199	1	0
LUZP1	4	0.17652	1	0	*CREM*	4	0.12203	1	0
PURB	3	0.17654	1	0	*PANK2*	4	0.12205	1	0
TNFSF9	2	0.17682	1	0	*TBX10*	1	0.12218	1	0
SREBF2	3	0.17693	1	0	*STIP1*	3	0.12261	1	0
HDAC8	4	0.17696	1	0	*RFX2*	4	0.12265	1	0
FGFR4	3	0.17697	1	0	*TMEM144*	4	0.12277	1	0
PAQR3	2	0.1771	1	0	*ST6GALNAC4*	4	0.12285	1	0
TRMT10C	3	0.17718	1	0	*CEP250*	4	0.12325	1	0
CFAP70	3	0.17739	1	0	*SEPTIN10*	3	0.12328	1	0
ARAP1	3	0.17759	1	0	*HEPN1*	4	0.12335	1	0
SELENOT	3	0.1776	1	0	*PRRG2*	4	0.12348	1	0
DNTTIP2	4	0.17772	1	0	*BBS2*	4	0.12377	1	0
PCDHB11	3	0.17776	1	0	*PITX3*	4	0.12388	1	0
VIRMA	4	0.17822	1	0	*FAM227A*	3	0.12399	1	0
CCDC102A	3	0.17829	1	0	*NDC80*	2	0.12407	1	0
MAPK11	4	0.17832	1	0	*ZMIZ1*	4	0.12413	1	0
MAT2B	3	0.17841	1	0	*MOBP*	4	0.12415	1	0
AKIRIN1	3	0.17856	1	0	*P4HA3*	3	0.12421	1	0
POP5	3	0.17865	1	0	*CERKL*	4	0.12421	1	0
PREP	4	0.17869	1	0	*KDM5A*	3	0.12435	1	0
OSBPL3	3	0.17879	1	0	*DTL*	3	0.12439	1	0
RMI1	4	0.17893	1	0	*BPIFB2*	4	0.12449	1	0
HES1	4	0.17909	1	0	*CCZ1B*	1	0.12453	1	0
KLHDC7A	3	0.17909	1	0	*GNAS*	4	0.12463	1	0
INTS6L	3	0.17923	1	0	*RAVER2*	3	0.12466	1	0
ALG11	3	0.17959	1	0	*HARBI1*	4	0.12494	1	0
ZNF835	3	0.17959	1	0	*TRIM33*	2	0.12497	1	0

LATS1	3	0.17999	1	0	*CPE*	3	0.12498	1	0
MESD	4	0.18008	1	0	*DHODH*	4	0.12508	1	0
FUZ	4	0.18018	1	0	*MAN2A2*	4	0.12525	1	0
TMEM8B	2	0.18019	1	0	*SLIT2*	4	0.12525	1	0
TYRO3	4	0.18021	1	0	*CERS6*	4	0.12537	1	0
RGL1	3	0.18029	1	0	*NOS2*	4	0.12541	1	0
FAM189B	3	0.18031	1	0	*C5orf64*	2	0.12555	1	0
URGCP	4	0.18033	1	0	*CNNM4*	4	0.12558	1	0
APRT	4	0.18035	1	0	*OPA3*	4	0.12561	1	0
LARP1B	4	0.18035	1	0	*MAN2B1*	4	0.12572	1	0
MTA1	2	0.18043	1	0	*ENPP5*	2	0.12585	1	0
TAS2R46	3	0.18067	1	0	*MGAM*	4	0.12619	1	0
CERS6	4	0.1807	1	0	*LOC10272495*	4	0.12622	1	0
MRI1	3	0.18082	1	0	*JUP*	4	0.12623	1	0
ARHGAP22	3	0.18095	1	0	*CHST14*	3	0.12629	1	0
CLK4	4	0.18106	1	0	*SLC35B2*	2	0.12641	1	0
CCDC121	4	0.18107	1	0	*NUGGC*	3	0.12644	1	0
ZNF429	4	0.18134	1	0	*TRIM50*	4	0.12644	1	0
PLLP	4	0.18146	1	0	*ZNF165*	2	0.12656	1	0
POLQ	4	0.1815	1	0	*METTL21A*	4	0.12659	1	0
MAPKBP1	4	0.18152	1	0	*CDKL4*	3	0.12679	1	0
MTR	4	0.18161	1	0	*CLEC6A*	3	0.12681	1	0
OC10192937.	3	0.18162	1	0	*TSPAN33*	3	0.12694	1	0
TTC37	4	0.18166	1	0	*PDCD11*	3	0.127	1	0
PDCD6IP	4	0.18174	1	0	*UBA7*	2	0.12702	1	0
GFOD1	3	0.18182	1	0	*TAF4B*	4	0.12743	1	0
SLC7A2	2	0.18191	1	0	*GPC1*	3	0.12773	1	0
STRA8	4	0.18195	1	0	*ATG4A*	4	0.12787	1	0
SARM1	4	0.18199	1	0	*NECTIN2*	4	0.12796	1	0
UBTD2	3	0.18201	1	0	*CXCR5*	3	0.12804	1	0
FBN3	4	0.18205	1	0	*GRIN2B*	4	0.12865	1	0
ZNF519	3	0.18224	1	0	*ANKS1A*	4	0.12883	1	0
B3GALT1	4	0.18225	1	0	*TIGD7*	3	0.12891	1	0
RNASE2	3	0.1825	1	0	*DUS2*	3	0.12897	1	0
TRIM31	2	0.1826	1	0	*CD84*	4	0.12907	1	0
DMAC1	3	0.18269	1	0	*PRR18*	3	0.12929	1	0
NAA30	4	0.18299	1	0	*FES*	4	0.12945	1	0
BZW2	3	0.18304	1	0	*PSG9*	3	0.12948	1	0
CYBB	3	0.18309	1	0	*NUDT22*	4	0.12952	1	0
ZNF541	4	0.18321	1	0	*RNF114*	3	0.12963	1	0
AGPAT1	3	0.18324	1	0	*SNRPC*	2	0.12966	1	0
GRN	4	0.18337	1	0	*SPR*	4	0.12972	1	0
SPOUT1	4	0.18357	1	0	*PPIL4*	4	0.12976	1	0
RGL2	4	0.18395	1	0	*SLC16A3*	4	0.12984	1	0
PAAF1	3	0.18398	1	0	*OGDHL*	4	0.13001	1	0
SNX9	4	0.18409	1	0	*HSPB1*	2	0.1301	1	0
EVL	4	0.1841	1	0	*MICAL3*	4	0.13031	1	0
RAC3	3	0.18411	1	0	*PPP1R14D*	4	0.13035	1	0
DBN1	4	0.18453	1	0	*ANGPT4*	4	0.13036	1	0
MRPL49	3	0.18462	1	0	*NOP9*	3	0.1304	1	0
CDT1	4	0.18467	1	0	*LAMTOR5*	3	0.1306	1	0
GFER	3	0.18473	1	0	*AFDN*	3	0.13094	1	0
SMCO4	3	0.18522	1	0	*NCF1*	2	0.13107	1	0
ZNF26	3	0.18527	1	0	*PTPRZ1*	3	0.13126	1	0
KAT2A	4	0.18536	1	0	*SH3GLB2*	4	0.13167	1	0
PARG	4	0.18537	1	0	*LRRC43*	2	0.13178	1	0
MYO9A	4	0.18544	1	0	*SPON2*	4	0.13179	1	0
CD1E	4	0.18545	1	0	*PCSK7*	3	0.13185	1	0

GALNT16	4	0.18568	1	0	*PRSS12*	3	0.13186	1	0
ZNF426	2	0.18576	1	0	*MTFR2*	4	0.13188	1	0
ZDHHC19	4	0.18577	1	0	*SCAMP4*	3	0.13188	1	0
PPP1R32	3	0.18587	1	0	*RIT1*	3	0.13199	1	0
LIN54	4	0.18593	1	0	*MTA3*	1	0.13201	1	0
RAET1L	3	0.18602	1	0	*TRMT13*	4	0.13212	1	0
ANXA10	4	0.18604	1	0	*CBR1*	2	0.13228	1	0
TSHZ2	3	0.18605	1	0	*ZNF300*	4	0.13231	1	0
CSAD	4	0.18607	1	0	*COMMD3*	1	0.13241	1	0
IQCG	3	0.1862	1	0	*SGSM3*	3	0.13246	1	0
SCNN1D	4	0.1864	1	0	*SLC39A11*	4	0.1325	1	0
ADAMTS3	3	0.18642	1	0	*RICTOR*	3	0.1325	1	0
EXOC2	4	0.1866	1	0	*LSM1*	3	0.13272	1	0
ENTR1	3	0.18662	1	0	*HCK*	4	0.13285	1	0
VTI1A	4	0.18676	1	0	*TAFA2*	3	0.13351	1	0
RTN3	3	0.18685	1	0	*FAM133B*	3	0.13374	1	0
FCMR	2	0.18687	1	0	*VSIG2*	3	0.13375	1	0
AKR1B1	3	0.18697	1	0	*ARFIP1*	3	0.13384	1	0
RNF38	3	0.18701	1	0	*AMPH*	3	0.13394	1	0
PASK	4	0.18709	1	0	*TUBG2*	3	0.13414	1	0
ZBTB7B	4	0.18715	1	0	*C12orf74*	4	0.13418	1	0
ESS2	4	0.18716	1	0	*KAT14*	3	0.13426	1	0
WDYHV1	4	0.18729	1	0	*MAP3K5*	4	0.13426	1	0
SCN5A	2	0.18755	1	0	*CLCF1*	3	0.13434	1	0
EXOSC1	4	0.18765	1	0	*CYBB*	3	0.13435	1	0
CBX6	3	0.18775	1	0	*SPEM2*	3	0.13456	1	0
COLCA1	3	0.18795	1	0	*SAP130*	4	0.13457	1	0
PPP1R3G	3	0.18811	1	0	*PTPRS*	4	0.1346	1	0
HIPK4	4	0.18818	1	0	*SLITRK5*	4	0.13482	1	0
ZNF682	4	0.18823	1	0	*ASXL1*	4	0.13492	1	0
STX18	4	0.18824	1	0	*FAM83C*	3	0.13505	1	0
HYAL4	4	0.18828	1	0	*C1GALT1C1*	3	0.13509	1	0
ARL8B	3	0.18844	1	0	*FAM133A*	3	0.13521	1	0
INHBE	3	0.18847	1	0	*DCAF16*	4	0.13551	1	0
AKAP3	3	0.18863	1	0	*DIRAS1*	2	0.1356	1	0
RHOG	4	0.18867	1	0	*ZNF780A*	4	0.13611	1	0
CIR1	3	0.18872	1	0	*ST6GAL2*	3	0.13637	1	0
AAK1	2	0.1889	1	0	*FRMPD1*	3	0.13659	1	0
NOP9	3	0.18895	1	0	*DBP*	1	0.13663	1	0
ANKRD13D	4	0.18937	1	0	*BRD4*	4	0.13691	1	0
FAM89B	3	0.18944	1	0	*ATF3*	4	0.13691	1	0
RARS1	4	0.19007	1	0	*LIG3*	2	0.13703	1	0
NFKB2	4	0.19009	1	0	*ROPN1*	3	0.13709	1	0
SOX3	4	0.19034	1	0	*OSBPL7*	4	0.13712	1	0
EXOC3L4	3	0.19036	1	0	*EAPP*	3	0.1372	1	0
EXO5	4	0.19082	1	0	*SLC14A2*	4	0.1372	1	0
H1-1	4	0.19085	1	0	*FAM50A*	3	0.13724	1	0
DNM1L	4	0.19112	1	0	*NMU*	3	0.13726	1	0
REV3L	2	0.19153	1	0	*P4HA1*	3	0.13726	1	0
TMEM14A	3	0.19156	1	0	*ZMYND19*	4	0.13758	1	0
INTS10	4	0.1917	1	0	*ELL3*	3	0.13763	1	0
NOA1	4	0.19185	1	0	*CCNT2*	4	0.13781	1	0
LCORL	4	0.19202	1	0	*GAL3ST4*	3	0.13782	1	0
C12orf42	3	0.19205	1	0	*CLEC2L*	3	0.13799	1	0
MAFA	2	0.19221	1	0	*LRP2BP*	3	0.13816	1	0
TDO2	4	0.19241	1	0	*SLC22A5*	4	0.13855	1	0
CYP4F22	4	0.19242	1	0	*ADTRP*	4	0.13856	1	0
KCTD7	4	0.1925	1	0	*IL37*	4	0.13871	1	0

SLC6A9	4	0.19259	1	0	*NKIRAS1*	4	0.13886	1	0
EIF3A	4	0.1927	1	0	*SIL1*	1	0.13901	1	0
ATP1B3	2	0.19287	1	0	*M6PR*	4	0.13908	1	0
HGS	4	0.19314	1	0	*KMT5C*	3	0.13917	1	0
SCAMP2	3	0.19315	1	0	*ZNF235*	3	0.13922	1	0
NUPR2	2	0.19326	1	0	*VPS41*	3	0.13933	1	0
TNKS1BP1	3	0.19342	1	0	*SPSB2*	3	0.13934	1	0
NDUFB4	3	0.19343	1	0	*ACTB*	3	0.13946	1	0
P2RX1	2	0.19363	1	0	*HPD*	4	0.13951	1	0
PLA2G4F	3	0.1937	1	0	*TMEM70*	4	0.13998	1	0
FSTL4	2	0.1938	1	0	*GGT1*	3	0.14011	1	0
GTF2IRD2B	1	0.19402	1	0	*TBX4*	4	0.14013	1	0
CACNB3	2	0.19497	1	0	*CSH2*	1	0.14052	1	0
NUP50	2	0.19498	1	0	*HAUS7*	4	0.14059	1	0
GULP1	3	0.19537	1	0	*GATAD2B*	3	0.14062	1	0
FBXL8	4	0.19538	1	0	*MAPK1IP1L*	3	0.1407	1	0
PDK3	3	0.19569	1	0	*MPLKIP*	3	0.14098	1	0
EXOC5	3	0.19576	1	0	*TMEM129*	4	0.14128	1	0
LINGO2	3	0.19605	1	0	*CAVIN1*	3	0.14129	1	0
NDUFS6	4	0.19607	1	0	*DIAPH1*	4	0.14147	1	0
CHD4	4	0.19609	1	0	*C6orf47*	2	0.14147	1	0
RRP36	2	0.19622	1	0	*B3GALNT1*	4	0.14159	1	0
GABRG3	2	0.1964	1	0	*CD200R1*	4	0.14165	1	0
TPP2	4	0.19649	1	0	*C1orf158*	3	0.14181	1	0
BBS5	4	0.19655	1	0	*SIGLEC6*	3	0.14212	1	0
ZNF783	3	0.19656	1	0	*RPL41*	3	0.14234	1	0
CEP63	3	0.19668	1	0	*ALDOB*	2	0.14237	1	0
POF1B	4	0.19671	1	0	*RMDN3*	3	0.1425	1	0
AQR	4	0.19682	1	0	*PTPN12*	4	0.14263	1	0
KCNE1	1	0.19692	1	0	*C8orf76*	1	0.14282	1	0
SHBG	4	0.19713	1	0	*HEG1*	3	0.14287	1	0
LINC01619	3	0.19725	1	0	*ATL1*	4	0.14292	1	0
MFAP4	4	0.1974	1	0	*S100A10*	4	0.1431	1	0
PLD6	4	0.1975	1	0	*CILP2*	4	0.14312	1	0
CXorf56	4	0.19756	1	0	*UFSP1*	4	0.14344	1	0
DOK1	4	0.19757	1	0	*PCDHGB1*	3	0.14346	1	0
ENPP4	4	0.19759	1	0	*KCNH7*	4	0.14358	1	0
MLLT10	2	0.19806	1	0	*TKTL2*	3	0.14362	1	0
KDM3A	4	0.19823	1	0	*GGACT*	2	0.14368	1	0
TAFA5	4	0.19864	1	0	*BMP6*	3	0.14374	1	0
IGSF5	4	0.19932	1	0	*IL11RA*	4	0.14378	1	0
INPP5D	2	0.1996	1	0	*NXNL2*	4	0.14386	1	0
STK17A	3	0.19971	1	0	*C3orf67*	4	0.14389	1	0
AKIRIN2	2	0.20014	1	0	*ATP1B2*	4	0.14395	1	0
TOMM22	3	0.20026	1	0	*DCUN1D2*	3	0.14419	1	0
CDK11B	3	0.20033	1	0	*LINGO3*	2	0.1442	1	0
TRIM63	4	0.20035	1	0	*NABP1*	4	0.14441	1	0
TECPR2	4	0.2004	1	0	*SEL1L3*	2	0.14475	1	0
ARV1	3	0.20069	1	0	*LILRB1*	3	0.14481	1	0
ZNF383	3	0.2007	1	0	*LYPD2*	3	0.1449	1	0
RHOF	4	0.20101	1	0	*NEB*	3	0.145	1	0
XBP1	4	0.20102	1	0	*NSMAF*	2	0.14525	1	0
CUL4A	2	0.20132	1	0	*ADCY3*	3	0.14558	1	0
MSH2	4	0.20143	1	0	*ZNF264*	4	0.14575	1	0
TCERG1	4	0.20145	1	0	*CAB39L*	2	0.14594	1	0
CPO	4	0.20151	1	0	*HIPK1*	3	0.14601	1	0
STRADA	4	0.20169	1	0	*BRMS1*	2	0.1463	1	0
PIGO	4	0.20183	1	0	*WWTR1*	4	0.14636	1	0

SCAMP3	3	0.20208	1	0	*FGD6*	4	0.1464	1	0
UNC119	3	0.2021	1	0	*TIAF1*	4	0.1469	1	0
TIAM2	3	0.20212	1	0	*STYX*	4	0.14695	1	0
HS3ST3B1	4	0.20226	1	0	*CYP2U1*	3	0.14696	1	0
UBTF	3	0.2023	1	0	*CPNE8*	3	0.14702	1	0
PRTN3	4	0.20273	1	0	*INPPL1*	3	0.14708	1	0
CCDC43	2	0.20274	1	0	*IFI27*	4	0.14722	1	0
LRRC45	2	0.20281	1	0	*RASGRP4*	4	0.14724	1	0
NOC2L	4	0.20291	1	0	*CPTP*	3	0.1473	1	0
POLR2L	4	0.20295	1	0	*TAS2R20*	4	0.14744	1	0
SIKE1	4	0.203	1	0	*ATF1*	2	0.14829	1	0
TRIM9	3	0.20302	1	0	*PKLR*	4	0.14876	1	0
SERTAD2	2	0.20314	1	0	*FOXL2*	4	0.14898	1	0
CHD6	2	0.2032	1	0	*DBNDD1*	4	0.14905	1	0
CLN5	1	0.20324	1	0	*SRRT*	4	0.1491	1	0
FAM126A	3	0.20331	1	0	*LAGE3*	2	0.1492	1	0
EXOC4	2	0.20333	1	0	*TSACC*	3	0.1493	1	0
SPPL2B	2	0.20338	1	0	*CRABP1*	4	0.14946	1	0
GALT	3	0.20342	1	0	*CD44*	3	0.1495	1	0
AFTPH	4	0.2036	1	0	*TULP1*	4	0.14957	1	0
HMGB2	4	0.20381	1	0	*JAGN1*	4	0.1496	1	0
SLC6A12	4	0.20386	1	0	*GPR87*	4	0.14961	1	0
VMAC	2	0.20386	1	0	*OR7G2*	4	0.14967	1	0
OTUB1	4	0.20393	1	0	*RRP15*	4	0.14974	1	0
ARHGEF5	2	0.20422	1	0	*C11orf95*	3	0.14984	1	0
GEMIN2	2	0.20433	1	0	*FEM1B*	3	0.15014	1	0
ANGEL2	3	0.20435	1	0	*TMEM258*	4	0.15016	1	0
MTMR14	4	0.20452	1	0	*RXRA*	3	0.15017	1	0
MZT2A	2	0.20502	1	0	*CRK*	3	0.15053	1	0
RCN3	4	0.20507	1	0	*MED4*	4	0.15064	1	0
GOSR1	2	0.20512	1	0	*TP53AIP1*	3	0.15075	1	0
ERBB4	3	0.2054	1	0	*DOLPP1*	4	0.15083	1	0
DERA	3	0.20547	1	0	*HIP1*	4	0.15103	1	0
SCN8A	3	0.20547	1	0	*YTHDC2*	3	0.15117	1	0
NEK7	2	0.20573	1	0	*TIGD2*	4	0.15135	1	0
NDUFA12	4	0.20591	1	0	*EPSTI1*	3	0.15169	1	0
CEP85L	4	0.20599	1	0	*DDX24*	4	0.15184	1	0
SLC25A32	4	0.20607	1	0	*RTN1*	2	0.15195	1	0
ZNF665	4	0.20613	1	0	*KLK2*	4	0.1521	1	0
PTPDC1	4	0.20641	1	0	*APOE*	4	0.15211	1	0
ZNF177	4	0.2066	1	0	*HDGFL3*	3	0.15224	1	0
NLRP3	3	0.20665	1	0	*TMEM150A*	3	0.15229	1	0
PRKCA	4	0.20672	1	0	*PGBD4*	4	0.15237	1	0
TIMP2	3	0.20679	1	0	*TBC1D14*	3	0.15252	1	0
CCZ1	1	0.20691	1	0	*TBC1D10C*	4	0.15284	1	0
SLC25A23	4	0.20692	1	0	*HSD17B11*	3	0.15292	1	0
GNAI1	3	0.20696	1	0	*SPATA13*	3	0.15317	1	0
TTC9C	3	0.20723	1	0	*UMAD1*	3	0.15386	1	0
NR6A1	4	0.20726	1	0	*CLIC2*	4	0.15391	1	0
ZSCAN20	1	0.20766	1	0	*RBM23*	4	0.15407	1	0
POLR1B	3	0.2077	1	0	*TMC8*	3	0.15412	1	0
CCNK	4	0.20784	1	0	*CEACAM1*	4	0.15414	1	0
MTG2	3	0.2079	1	0	*GOLGA5*	4	0.15473	1	0
XRCC3	3	0.20791	1	0	*EMD*	4	0.15517	1	0
SMIM3	4	0.20798	1	0	*CEP85*	4	0.15524	1	0
ILF3	4	0.20822	1	0	*MRNIP*	3	0.15556	1	0
NBPF9	2	0.20832	1	0	*ATP10B*	3	0.15557	1	0
GPRC5D	4	0.20842	1	0	*COBL*	4	0.15636	1	0

TSPOAP1	2	0.20842	1	0	*CSTA*	3	0.1566	1	0
FLVCR2	3	0.20868	1	0	*CHRNA6*	4	0.15687	1	0
RPN1	3	0.2087	1	0	*MGAM2*	3	0.15702	1	0
HSPA9	4	0.20897	1	0	*MSX2*	2	0.15743	1	0
DYRK4	4	0.20906	1	0	*PHKA2*	3	0.15782	1	0
CGREF1	4	0.20926	1	0	*TMX2*	2	0.15785	1	0
NFE2L2	3	0.20937	1	0	*HMSD*	2	0.15788	1	0
SDR9C7	3	0.20961	1	0	*CCDC114*	3	0.15791	1	0
AIFM2	3	0.20975	1	0	*CLTA*	2	0.15824	1	0
MTRF1L	2	0.20984	1	0	*DUSP6*	3	0.15833	1	0
SLC24A3	3	0.20991	1	0	*CTSH*	4	0.15837	1	0
FBXO3	3	0.21001	1	0	*L3MBTL3*	1	0.15856	1	0
JUND	4	0.21001	1	0	*RAB5C*	2	0.15864	1	0
ATP5MC2	4	0.2101	1	0	*ADAMTSL5*	4	0.15894	1	0
PLSCR1	2	0.21075	1	0	*PTK2*	4	0.15897	1	0
DBI	1	0.21078	1	0	*CHAF1A*	2	0.15899	1	0
BCCIP	3	0.21094	1	0	*SEPTIN3*	4	0.15906	1	0
PPP1R14B	2	0.21133	1	0	*SEC14L3*	3	0.15946	1	0
ZNF420	4	0.21147	1	0	*XAGE2*	3	0.1596	1	0
SPRED3	4	0.21153	1	0	*KCTD18*	4	0.16004	1	0
YIF1B	4	0.21163	1	0	*DENND6A*	4	0.16009	1	0
PPP6C	4	0.21168	1	0	*MKNK1*	1	0.16048	1	0
GPN3	3	0.21172	1	0	*CLK2*	4	0.16052	1	0
CATSPERG	2	0.212	1	0	*EBAG9*	3	0.16059	1	0
HCST	2	0.21238	1	0	*PECR*	4	0.16065	1	0
EIF2B4	4	0.2127	1	0	*PSORS1C2*	2	0.16066	1	0
TMEM87A	4	0.21286	1	0	*TPRN*	3	0.1611	1	0
LRATD2	4	0.21327	1	0	*LTBP4*	4	0.16126	1	0
ALKBH6	4	0.21353	1	0	*SGPP2*	3	0.16127	1	0
IPO5	4	0.21393	1	0	*EMC10*	3	0.16136	1	0
POP1	4	0.21396	1	0	*SERTAD3*	3	0.16183	1	0
CLDN2	3	0.21425	1	0	*ZNF799*	3	0.16203	1	0
FBXL15	2	0.21457	1	0	*CHMP1B*	2	0.16229	1	0
KIF23	4	0.21461	1	0	*FAAH2*	2	0.16251	1	0
POLA2	4	0.2148	1	0	*KHDRBS1*	2	0.16252	1	0
C12orf4	4	0.21486	1	0	*SCGB2B2*	4	0.16255	1	0
VMA21	2	0.21501	1	0	*SIGLEC1*	3	0.16282	1	0
LAMB2	4	0.21561	1	0	*PRCC*	4	0.16358	1	0
WDR74	4	0.21623	1	0	*EMILIN2*	3	0.16372	1	0
CALY	3	0.21627	1	0	*STK24*	2	0.1638	1	0
PIP4K2C	3	0.21637	1	0	*FAM71A*	4	0.16415	1	0
RPS13	3	0.21649	1	0	*C5orf63*	4	0.16424	1	0
RMDN2	3	0.21651	1	0	*HADH*	2	0.16452	1	0
FXYD5	4	0.21665	1	0	*GMPS*	3	0.16453	1	0
HABP4	3	0.21697	1	0	*RNASE2*	3	0.16479	1	0
PTPN1	2	0.21705	1	0	*RGS9BP*	3	0.16482	1	0
WDR33	4	0.21708	1	0	*PIGU*	3	0.16493	1	0
CDK12	4	0.21718	1	0	*MEPCE*	4	0.16501	1	0
XCL2	2	0.21757	1	0	*ADAM32*	4	0.16503	1	0
CC2D2B	5	0.21759	1	0	*P4HB*	2	0.16506	1	0
ADGRG1	3	0.21779	1	0	*HS3ST5*	3	0.16517	1	0
C3AR1	3	0.21795	1	0	*RHD*	3	0.1652	1	0
HNRNPD	3	0.21801	1	0	*ALDH5A1*	3	0.16538	1	0
RPL23	3	0.2181	1	0	*ACACB*	3	0.16546	1	0
CCDC12	3	0.21821	1	0	*PROC*	2	0.16585	1	0
VCAN	3	0.21825	1	0	*ZNF304*	1	0.16587	1	0
ELOA2	3	0.21831	1	0	*ZNF812P*	4	0.16622	1	0
RAB6C	1	0.21863	1	0	*DCLK1*	4	0.16634	1	0

UXS1	4	0.21869	1	0	*RIOK2*	4	0.16673	1	0
MTMR2	4	0.21877	1	0	*GPHA2*	2	0.16679	1	0
RBM43	4	0.21889	1	0	*SLC38A6*	3	0.16687	1	0
SLC25A40	4	0.21891	1	0	*RPS14*	1	0.16701	1	0
FGF22	1	0.21925	1	0	*C19orf18*	3	0.16748	1	0
ZBTB2	4	0.21943	1	0	*HNRNPA1*	3	0.16766	1	0
CLCF1	3	0.21978	1	0	*NUP205*	1	0.16768	1	0
BAALC	2	0.21982	1	0	*GATAD1*	3	0.1681	1	0
NFE2L3	4	0.21999	1	0	*TMEM45A*	3	0.16811	1	0
ZNF354B	4	0.22002	1	0	*PAWR*	4	0.16844	1	0
GPANK1	4	0.22016	1	0	*NTMT1*	4	0.16862	1	0
ZBTB38	3	0.22016	1	0	*SLC6A9*	4	0.16866	1	0
HES3	3	0.22025	1	0	*BTG4*	3	0.16876	1	0
METAP2	3	0.22074	1	0	*RTN4RL1*	4	0.16877	1	0
ECHDC1	3	0.22082	1	0	*CD6*	4	0.16922	1	0
RANBP9	2	0.22098	1	0	*NMT2*	2	0.16925	1	0
MTFMT	4	0.221	1	0	*NREP*	3	0.16936	1	0
PRICKLE4	4	0.22123	1	0	*NGFR*	3	0.16961	1	0
OCEL1	3	0.22164	1	0	*TPBG*	2	0.16966	1	0
LSM3	2	0.22176	1	0	*KAT6B*	3	0.16989	1	0
CCDC116	3	0.22178	1	0	*SCYL1*	3	0.1699	1	0
ACTR1B	2	0.22187	1	0	*ARPC5*	3	0.17012	1	0
ASB13	4	0.22199	1	0	*NSUN3*	4	0.17078	1	0
PAQR9	3	0.22206	1	0	*LOC149373*	2	0.1712	1	0
CASTOR2	3	0.22208	1	0	*NIPA2*	2	0.17134	1	0
THOP1	1	0.2222	1	0	*PTTG2*	2	0.1714	1	0
PFKL	3	0.22232	1	0	*LRRC7*	2	0.17141	1	0
MAFK	3	0.22247	1	0	*TAF15*	4	0.17161	1	0
TRAPPC4	4	0.2227	1	0	*PFKFB4*	4	0.17172	1	0
HPN	3	0.22274	1	0	*PPFIA2*	3	0.17187	1	0
ZNF69	2	0.22275	1	0	*KIAA2026*	3	0.17193	1	0
SETD6	3	0.22303	1	0	*FCGR1B*	1	0.17203	1	0
MT1X	2	0.22306	1	0	*RAB3B*	4	0.17248	1	0
NELFA	3	0.22341	1	0	*DERA*	3	0.17257	1	0
CINP	4	0.22356	1	0	*USP46*	3	0.17265	1	0
MIOS	4	0.22363	1	0	*RGS1*	3	0.17338	1	0
ZNF607	4	0.22376	1	0	*FSCN2*	3	0.17388	1	0
FTL	2	0.2241	1	0	*RUSC1-AS1*	4	0.174	1	0
CUL4B	2	0.2242	1	0	*MCPH1*	3	0.17409	1	0
CHORDC1	3	0.22423	1	0	*MUTYH*	3	0.17416	1	0
DHX29	2	0.22425	1	0	*INSM1*	3	0.17435	1	0
MTRNR2L9	2	0.22462	1	0	*CD14*	3	0.17444	1	0
SNAP23	3	0.22473	1	0	*POLR1B*	3	0.17459	1	0
FITM2	3	0.22475	1	0	*ALKBH3*	3	0.1749	1	0
AP2M1	4	0.22555	1	0	*BFSP1*	3	0.1751	1	0
SKI	3	0.22577	1	0	*ECPAS*	4	0.17525	1	0
HACD4	4	0.22581	1	0	*ZNF484*	3	0.17534	1	0
TXNL4B	4	0.22621	1	0	*PBX3*	4	0.17551	1	0
RNF138	2	0.22694	1	0	*SPINT2*	4	0.17572	1	0
PPIE	4	0.22789	1	0	*ZBED8*	4	0.17598	1	0
ZNF567	4	0.22808	1	0	*CFAP52*	4	0.17604	1	0
PWP1	3	0.22814	1	0	*MORN5*	4	0.17628	1	0
TMPRSS5	4	0.2284	1	0	*HAUS1*	4	0.17646	1	0
CYP4F11	4	0.22877	1	0	*WDR48*	3	0.17647	1	0
FBXO47	4	0.22889	1	0	*C1orf56*	3	0.17655	1	0
XPC	3	0.22896	1	0	*CLDN18*	3	0.17673	1	0
DROSHA	3	0.22916	1	0	*ZNF658*	2	0.17699	1	0
C2orf80	2	0.2293	1	0	*BLNK*	3	0.17699	1	0

C9orf106	2	0.22935	1	0	*PPFIBP2*	4	0.17738	1	0
MRPL38	4	0.2295	1	0	*MYOM2*	3	0.1775	1	0
TPM1	2	0.22962	1	0	*C7orf69*	3	0.17762	1	0
ZFP30	3	0.22976	1	0	*SPRYD3*	3	0.17781	1	0
PGBD1	3	0.2302	1	0	*DCAF1*	3	0.17791	1	0
PRCC	4	0.23026	1	0	*KRT25*	4	0.17792	1	0
TTC1	4	0.2304	1	0	*CDT1*	4	0.17798	1	0
LRAT	4	0.23062	1	0	*KRTAP5-7*	2	0.17818	1	0
SFXN1	4	0.23138	1	0	*ZCRB1*	4	0.17855	1	0
DST	3	0.23164	1	0	*CCDC126*	3	0.17856	1	0
AATF	3	0.23231	1	0	*CEP131*	4	0.17926	1	0
ZNF799	3	0.23231	1	0	*IGSF1*	4	0.17927	1	0
BMPR1A	3	0.23265	1	0	*P4HTM*	3	0.17963	1	0
TMEM170B	3	0.23275	1	0	*TTC5*	4	0.17968	1	0
HSPA12A	3	0.23291	1	0	*EFCAB2*	3	0.17977	1	0
RAB5IF	2	0.23292	1	0	*NFAT5*	4	0.18042	1	0
CYP2B6	4	0.23334	1	0	*TRIM44*	3	0.18081	1	0
AGXT	3	0.23337	1	0	*PHAX*	4	0.18118	1	0
ZNF566	3	0.23359	1	0	*SEMA4D*	4	0.18151	1	0
ZNF668	4	0.23367	1	0	*HLA-DPB1*	3	0.18177	1	0
TSBP1	4	0.23397	1	0	*WDR34*	3	0.18191	1	0
CBLIF	3	0.23432	1	0	*KIAA0408*	4	0.18194	1	0
DCTN2	4	0.23436	1	0	*PLEKHM3*	4	0.18205	1	0
COL8A2	3	0.23462	1	0	*KPNA3*	4	0.18275	1	0
RTP4	4	0.2351	1	0	*MAGEB2*	3	0.18281	1	0
CCDC103	3	0.23569	1	0	*SAC3D1*	3	0.18322	1	0
MTFP1	2	0.23598	1	0	*POM121*	3	0.18407	1	0
SLC25A29	2	0.23618	1	0	*ITPRID2*	3	0.18407	1	0
CENPF	3	0.23626	1	0	*C2orf88*	3	0.18409	1	0
CAMTA2	4	0.23627	1	0	*MAPK7*	3	0.18434	1	0
POLR1D	4	0.23627	1	0	*RSRC2*	3	0.18464	1	0
HGH1	3	0.23629	1	0	*ZDHHC4*	4	0.18515	1	0
MRPL22	3	0.23642	1	0	*MRGPRF*	3	0.18543	1	0
CDIPT	3	0.23685	1	0	*C2CD3*	3	0.18546	1	0
HAUS4	4	0.23685	1	0	*TNKS1BP1*	3	0.1855	1	0
TTC27	4	0.23738	1	0	*GNG7*	3	0.18625	1	0
H2AX	4	0.23747	1	0	*TXLNA*	4	0.1868	1	0
ATP1A3	4	0.23773	1	0	*GALNT13*	4	0.1868	1	0
RASAL3	3	0.23792	1	0	*ZBTB24*	3	0.18695	1	0
DLL1	3	0.23793	1	0	*ALG14*	3	0.18714	1	0
SPICE1	4	0.23826	1	0	*SQLE*	2	0.18716	1	0
SLC25A48	4	0.23833	1	0	*EVI5L*	3	0.18737	1	0
COPS4	3	0.23865	1	0	*KIF18B*	4	0.18765	1	0
LENG8	3	0.23942	1	0	*CHSY1*	3	0.18783	1	0
EXT2	4	0.23952	1	0	*CYP2S1*	3	0.18828	1	0
TOP2A	4	0.23965	1	0	*LRP4*	3	0.18846	1	0
PLXNB1	4	0.23982	1	0	*CCDC138*	4	0.18929	1	0
CIP2A	4	0.24006	1	0	*PSMD1*	4	0.1894	1	0
SLTM	4	0.2403	1	0	*PAPPA*	4	0.18957	1	0
ZNF543	3	0.24053	1	0	*SDF4*	4	0.18974	1	0
LGMN	2	0.24057	1	0	*RMDN2*	3	0.18996	1	0
CFAP77	1	0.24088	1	0	*SMIM13*	3	0.19022	1	0
DMPK	3	0.24183	1	0	*SLC30A3*	4	0.19026	1	0
DNAJC9	2	0.24189	1	0	*CAPN3*	4	0.19064	1	0
MPZL1	4	0.24222	1	0	*ATP6V1F*	4	0.19087	1	0
EXOSC10	4	0.24224	1	0	*DCN*	3	0.19114	1	0
PDE6D	3	0.24224	1	0	*SLC27A4*	4	0.19129	1	0
DDX28	4	0.24255	1	0	*TAF3*	4	0.19136	1	0

MIS12	4	0.24256	1	0	*FGA*	3	0.19141	1	0
ETF1	3	0.24268	1	0	*GRIN2D*	2	0.19208	1	0
HMSD	2	0.24288	1	0	*PDCD2*	3	0.19227	1	0
RNPEPL1	4	0.24331	1	0	*LINC02860*	3	0.19269	1	0
RMND5A	3	0.24348	1	0	*TTC34*	2	0.19367	1	0
YY1	3	0.24351	1	0	*ATP13A1*	3	0.19372	1	0
CDH26	3	0.24457	1	0	*RNF166*	2	0.19397	1	0
PLEKHM2	3	0.24459	1	0	*DND1*	4	0.19451	1	0
SLC35A2	2	0.24501	1	0	*TTC29*	3	0.19456	1	0
KHK	2	0.2462	1	0	*RPN1*	3	0.1948	1	0
FAM32A	3	0.24663	1	0	*YBX2*	3	0.19545	1	0
NCK1	2	0.24667	1	0	*CDRT1*	3	0.1955	1	0
NAA38	3	0.24683	1	0	*STK25*	3	0.19555	1	0
UTP14A	4	0.24714	1	0	*CCDC184*	3	0.19559	1	0
SLX4	4	0.2474	1	0	*FBN2*	3	0.19569	1	0
SKIL	4	0.24742	1	0	*COQ10B*	3	0.19602	1	0
RPA1	4	0.24815	1	0	*SF3A3*	3	0.19666	1	0
PFDN1	4	0.24844	1	0	*TOP3B*	3	0.19687	1	0
ELL	4	0.24851	1	0	*ZNF169*	3	0.19722	1	0
DENND3	4	0.24861	1	0	*TOMM20L*	3	0.19723	1	0
PTRHD1	3	0.24899	1	0	*FBXL15*	2	0.19761	1	0
ASTN2	4	0.2493	1	0	*TIMM29*	3	0.19765	1	0
KARS1	4	0.24943	1	0	*TCEA1*	2	0.19802	1	0
JRKL	4	0.24956	1	0	*ARSI*	2	0.19829	1	0
FBXO5	4	0.24985	1	0	*MAP1S*	2	0.19853	1	0
PRPF38A	4	0.25005	1	0	*CPSF1*	3	0.19905	1	0
SNX14	4	0.25014	1	0	*AP5Z1*	4	0.19913	1	0
KLHDC1	4	0.25015	1	0	*NUP188*	4	0.19924	1	0
SNAPIN	4	0.25036	1	0	*RNF128*	3	0.19953	1	0
ZNF407	3	0.25037	1	0	*SNAPC3*	3	0.19955	1	0
ACTL6A	4	0.25078	1	0	*BATF3*	3	0.20003	1	0
G3BP2	2	0.25104	1	0	*P2RY2*	3	0.20009	1	0
CCNYL1	4	0.25147	1	0	*PSME4*	4	0.20065	1	0
TAC3	3	0.25184	1	0	*SRP19*	4	0.20066	1	0
RABGGTA	3	0.25193	1	0	*PTPN6*	3	0.20067	1	0
NOXO1	4	0.25244	1	0	*WNT3*	2	0.20096	1	0
DCK	4	0.25249	1	0	*TMOD2*	3	0.20118	1	0
CEP85	4	0.25251	1	0	*DCP2*	4	0.20146	1	0
SPN	4	0.25267	1	0	*PLA2G4F*	3	0.20171	1	0
SLPI	2	0.25326	1	0	*WDR17*	4	0.20222	1	0
KMT5B	3	0.25327	1	0	*TEDDM1*	3	0.20272	1	0
POLDIP3	4	0.25365	1	0	*USP36*	4	0.20353	1	0
GTDC1	3	0.25386	1	0	*HOXB4*	4	0.20374	1	0
MSTN	4	0.25413	1	0	*ZNF790*	3	0.20377	1	0
DRG1	2	0.25481	1	0	*ADSS2*	3	0.20443	1	0
PRPF40A	4	0.25545	1	0	*BMS1*	2	0.20457	1	0
GNE	4	0.25602	1	0	*HAPLN1*	3	0.20521	1	0
TGS1	2	0.25702	1	0	*RBM25*	4	0.20561	1	0
EGLN3	2	0.25747	1	0	*AIFM2*	3	0.20608	1	0
TRMT61B	3	0.2578	1	0	*PCDHGA4*	3	0.20625	1	0
PGS1	3	0.25832	1	0	*HMGCR*	4	0.20631	1	0
THG1L	4	0.25841	1	0	*FAM219A*	3	0.20654	1	0
ZNF34	4	0.25887	1	0	*SLC16A5*	4	0.20679	1	0
IDNK	4	0.25961	1	0	*REXO4*	2	0.20719	1	0
UBE2M	2	0.2599	1	0	*APBB1IP*	3	0.20771	1	0
EPRS1	4	0.26043	1	0	*CSTF2*	4	0.20818	1	0
MSTO1	2	0.26092	1	0	*DHRS4L2*	2	0.20831	1	0
NDUFV3	4	0.26095	1	0	*CCDC88A*	4	0.20866	1	0

ARF1	2	0.26103	1	0	*RAB5IF*	2	0.2091	1	0
SMG5	3	0.26206	1	0	*TDP1*	3	0.20931	1	0
NCKIPSD	2	0.26265	1	0	*NFATC2*	3	0.20956	1	0
DNAJA4	2	0.2629	1	0	*CDK7*	3	0.21041	1	0
PKLR	4	0.26312	1	0	*PAK4*	4	0.21076	1	0
ELP1	4	0.26394	1	0	*ASXL2*	3	0.21142	1	0
MED11	3	0.2641	1	0	*SKI*	3	0.21145	1	0
PPA1	4	0.2641	1	0	*PTCH1*	3	0.21237	1	0
GTF2B	3	0.26414	1	0	*GIMAP8*	4	0.21277	1	0
PITX1	4	0.26477	1	0	*DNAJC8*	4	0.21293	1	0
NOL12	4	0.26493	1	0	*SLMAP*	4	0.21328	1	0
TMEM258	4	0.26505	1	0	*HECTD1*	3	0.21406	1	0
ONECUT2	2	0.26511	1	0	*PDGFA*	4	0.21536	1	0
HAVCR2	3	0.26519	1	0	*ZMYM6*	3	0.21538	1	0
PAX2	3	0.26542	1	0	*WASF1*	2	0.21545	1	0
SRP68	3	0.26567	1	0	*LBR*	3	0.21553	1	0
MOGS	3	0.26588	1	0	*MCM7*	3	0.2159	1	0
RBP7	3	0.26588	1	0	*NAAA*	4	0.21625	1	0
TAF8	3	0.26588	1	0	*IPO13*	4	0.21645	1	0
CCDC127	4	0.26603	1	0	*PHF21B*	4	0.2171	1	0
HYAL3	4	0.26634	1	0	*MANEAL*	3	0.2173	1	0
VARS	3	0.26675	1	0	*USP47*	2	0.21742	1	0
NATD1	2	0.26711	1	0	*NIP7*	4	0.2179	1	0
RSBN1L	4	0.26732	1	0	*SMAP2*	3	0.21897	1	0
DIDO1	4	0.26812	1	0	*RMND5A*	3	0.21965	1	0
PDGFRA	2	0.26823	1	0	*MRLN*	3	0.21972	1	0
MVK	4	0.26832	1	0	*DNAJB13*	4	0.2201	1	0
PITPNM2	3	0.2685	1	0	*CDH26*	3	0.22074	1	0
FAM167B	3	0.26889	1	0	*KCNJ4*	3	0.22079	1	0
TFF3	4	0.26891	1	0	*APCDD1L*	3	0.22081	1	0
ATP5PD	2	0.26929	1	0	*PAFAH1B2*	3	0.22144	1	0
SRSF6	4	0.27086	1	0	*GTSE1*	2	0.22183	1	0
HAUS1	4	0.27089	1	0	*TP53TG5*	3	0.22235	1	0
LEF1	2	0.27127	1	0	*NUDT1*	3	0.22313	1	0
CCNG1	3	0.27179	1	0	*CPSF2*	2	0.22334	1	0
ANLN	4	0.27274	1	0	*VAPB*	3	0.22398	1	0
CCDC27	3	0.27345	1	0	*TIMP1*	4	0.22449	1	0
RTN4RL1	4	0.27373	1	0	*CSNK1A1*	2	0.22574	1	0
PSMD11	4	0.27383	1	0	*CDKN2C*	3	0.22636	1	0
RR5-ARHGAF	1	0.27397	1	0	*PARN*	3	0.22704	1	0
RNPC3	4	0.2741	1	0	*UBC*	4	0.22724	1	0
RAB3B	4	0.27447	1	0	*FRMD4A*	2	0.22747	1	0
PRPSAP1	4	0.27575	1	0	*SLC6A8*	2	0.22809	1	0
WDR78	3	0.27575	1	0	*HNRNPA1L2*	3	0.2282	1	0
THAP6	4	0.27593	1	0	*OBSCN*	3	0.22863	1	0
CALCA	3	0.27598	1	0	*SNX18*	3	0.22904	1	0
EPN3	4	0.27619	1	0	*FLII*	4	0.22952	1	0
BNIP1	3	0.27711	1	0	*NISCH*	4	0.22981	1	0
GINS3	4	0.27809	1	0	*DFFA*	2	0.22985	1	0
UBA1	3	0.27809	1	0	*PRAP1*	3	0.23059	1	0
RHOB	3	0.27824	1	0	*PPP2R5C*	3	0.23092	1	0
AUNIP	3	0.27851	1	0	*RRS1*	2	0.23194	1	0
NANOS3	4	0.27981	1	0	*DMXL1*	3	0.23219	1	0
NIT2	2	0.28005	1	0	*SLC22A15*	3	0.23229	1	0
ATP6V0C	4	0.28016	1	0	*ADIRF*	4	0.23252	1	0
PKN1	4	0.28072	1	0	*SLF1*	3	0.23262	1	0
TBL3	4	0.28154	1	0	*DUS3L*	3	0.23274	1	0
ADSS2	3	0.28156	1	0	*SSC4D*	2	0.23284	1	0

NUDT13	2	0.2816	1	0	*ZNF446*	3	0.23301	1	0
MLXIP	4	0.28168	1	0	*IARS2*	3	0.23327	1	0
POLD2	3	0.28358	1	0	*SERPINB6*	2	0.23395	1	0
FBN2	3	0.28361	1	0	*RHBDL2*	4	0.23451	1	0
NDUFC1	4	0.28416	1	0	*TGS1*	2	0.23459	1	0
LRIT3	2	0.28437	1	0	*POTEE*	2	0.23539	1	0
RPS19BP1	4	0.28549	1	0	*ORC6*	4	0.23543	1	0
SEPSECS	3	0.2864	1	0	*GRINA*	4	0.23556	1	0
HCFC1R1	4	0.28664	1	0	*C11orf58*	3	0.23561	1	0
ACVR1B	4	0.28788	1	0	*MAGEA12*	2	0.23652	1	0
FAM98B	3	0.2879	1	0	*CDC42BPB*	3	0.23679	1	0
NLRP14	4	0.28823	1	0	*CD22*	3	0.23688	1	0
UBA5	3	0.28852	1	0	*KDM1A*	4	0.237	1	0
THOC7	4	0.28859	1	0	*NEIL3*	4	0.23715	1	0
MEMO1	4	0.28905	1	0	*LATS2*	2	0.23755	1	0
MYH14	3	0.28965	1	0	*TAS2R5*	3	0.23793	1	0
GPR142	2	0.29426	1	0	*CPNE6*	2	0.23856	1	0
ANKRD10	2	0.29449	1	0	*SFXN5*	3	0.23954	1	0
PNPLA7	2	0.29504	1	0	*GNPNAT1*	3	0.24126	1	0
ATIC	3	0.29527	1	0	*CTDSPL2*	4	0.24139	1	0
PITPNB	3	0.2957	1	0	*SPCS3*	4	0.24195	1	0
RNF157	4	0.29629	1	0	*MKS1*	3	0.24382	1	0
INTS4	4	0.29772	1	0	*ATP10A*	3	0.24386	1	0
CNOT10	3	0.29808	1	0	*SUSD4*	2	0.24386	1	0
CSE1L	4	0.29922	1	0	*PDLIM7*	3	0.24553	1	0
JUN	3	0.30162	1	0	*IFITM5*	3	0.24918	1	0
GGTLC2	2	0.3022	1	0	*SPACA9*	3	0.24943	1	0
GPSM2	3	0.30235	1	0	*RHOT2*	3	0.25074	1	0
DNAJC25	3	0.30372	1	0	*KREMEN2*	3	0.25146	1	0
AIMP2	4	0.3053	1	0	*ILF3*	4	0.25295	1	0
SNRPG	1	0.30697	1	0	*ZSWIM3*	2	0.25304	1	0
FARSB	4	0.31026	1	0	*KAT7*	3	0.25337	1	0
MARS1	4	0.3104	1	0	*RPS28*	2	0.25362	1	0
RHBDF1	3	0.31084	1	0	*CFAP70*	3	0.2553	1	0
EPB41L5	3	0.31176	1	0	*MAFF*	3	0.25665	1	0
RPL8	4	0.31272	1	0	*GAA*	3	0.25742	1	0
H2BC14	3	0.31477	1	0	*FBXO5*	4	0.25747	1	0
PELP1	4	0.31568	1	0	*ERBIN*	3	0.2579	1	0
WDR4	3	0.31693	1	0	*FANCC*	2	0.25974	1	0
MMS19	2	0.31719	1	0	*AMOTL1*	3	0.26135	1	0
PSMB3	4	0.3177	1	0	*ETF1*	3	0.2641	1	0
WDR61	4	0.31841	1	0	*PLXNB1*	4	0.26538	1	0
SUSD4	2	0.31989	1	0	*TBCB*	2	0.26741	1	0
METTL16	3	0.32081	1	0	*ECD*	4	0.26837	1	0
INCENP	4	0.32092	1	0	*NBAS*	2	0.26929	1	0
RPS7	2	0.32202	1	0	*CIAO2B*	3	0.27006	1	0
MTPAP	3	0.32305	1	0	*VPS4B*	2	0.27136	1	0
DPH7	2	0.32748	1	0	*AHSP*	3	0.27404	1	0
GTF3C1	4	0.33324	1	0	*TMEM88B*	3	0.2754	1	0
NDC80	2	0.33452	1	0	*ATAD5*	2	0.27659	1	0
ING3	2	0.33456	1	0	*RCL1*	3	0.27888	1	0
RAD51C	4	0.33687	1	0	*FAM181B*	2	0.2798	1	0
EIF5	3	0.33951	1	0	*DHDDS*	4	0.28497	1	0
SF3B5	3	0.34541	1	0	*LRPAP1*	3	0.285	1	0
TSR1	4	0.34673	1	0	*SMIM1*	2	0.28516	1	0
H2AC6	3	0.34898	1	0	*EPOP*	3	0.28569	1	0
WDR1	3	0.34963	1	0	*PSMA4*	3	0.2869	1	0
EEF1A1	1	0.35117	1	0	*DPM3*	4	0.28729	1	0

CHAF1B	4	0.35512	1	0	*ZNF75A*	3	0.29002	1	0
SAE1	4	0.35522	1	0	*UPB1*	4	0.29129	1	0
ANAPC11	4	0.35587	1	0	*C1orf122*	2	0.29755	1	0
MRPS15	2	0.36116	1	0	*LAMTOR3*	3	0.29785	1	0
RTCB	4	0.36176	1	0	*ANKRD10*	2	0.30211	1	0
ERH	4	0.36806	1	0	*PLEKHN1*	3	0.30354	1	0
RPL21	2	0.36944	1	0	*KPNB1*	4	0.30638	1	0
SDHC	2	0.37003	1	0	*RPL27A*	1	0.30811	1	0
MED21	2	0.37049	1	0	*RAB3GAP1*	2	0.30902	1	0
KMT5A	2	0.37862	1	0	*INHA*	3	0.31006	1	0
PSMB1	4	0.39794	1	0	*EIF3D*	3	0.31237	1	0
PARD6B	2	0.40047	1	0	*RNF224*	4	0.32058	1	0
PPP1R7	2	0.40354	1	0	*ARFRP1*	3	0.32538	1	0
CTDP1	3	0.43375	1	0	*SF3A2*	3	0.3272	1	0
ATP6V0B	4	0.43676	1	0	*NOL11*	3	0.332	1	0
RGP1	3	0.44329	1	0	*AK6*	4	0.34545	1	0
SNRPF	3	0.47306	1	0	*NADK2*	3	0.34668	1	0
COPB2	3	0.48321	1	0	*SNRNP48*	3	0.34894	1	0
CCT5	3	0.49319	1	0	*RPL23*	3	0.37595	1	0
RPL36	2	0.50324	1	0	*PCF11*	4	0.37646	1	0
RUVBL1	3	0.50375	1	0	*NOP56*	3	0.48113	1	0
WDR77	4	0.5502	1	0	*RPS2*	3	0.56525	1	0

Table S2. Summary of 27 cuproptosis-related genes.

**Gene**	**Type**
MTF1	Cuproptosis
CDKN2A	Cuproptosis
REXO2	Cuproptosis
PDHA1	Cuproptosis
LIPT1	Cuproptosis
DLAT	Cuproptosis
PDHB	Cuproptosis
LIAS	Cuproptosis
GLS	Cuproptosis
BRPF1	Cuproptosis
DLD	Cuproptosis
YEATS2	Cuproptosis
AFG3L2	Cuproptosis
MPC1	Cuproptosis
SLFN11	Cuproptosis
FDX1	Cuproptosis
COQ7	Cuproptosis
HAUS5	Cuproptosis
OXA1L	Cuproptosis
MCUR1	Cuproptosis
RPL3	Cuproptosis
CAPRIN1	Cuproptosis
EGLN1	Cuproptosis
MBTPS1	Cuproptosis
SOX2	Cuproptosis
SCAP	Cuproptosis
AHR	Cuproptosis

Table S3. The prognostic value of 27 cuproptosis-related genes in CRC patients.

Gene	HR	HR.95L	HR.95H	Cox *P* -value	Kaplan-meier *P* -value
MTF1	0.876	0.608	1.260	0.475	0.016
CDKN2A	1.140	0.995	1.306	0.059	0.016
REXO2	0.680	0.456	1.015	0.059	0.000
PDHA1	0.783	0.596	1.030	0.081	0.015
LIPT1	0.879	0.687	1.124	0.303	0.040
DLAT	0.741	0.564	0.972	0.031	0.001
PDHB	0.803	0.543	1.189	0.274	0.011
LIAS	0.749	0.621	0.902	0.002	0.000
GLS	1.536	1.098	2.149	0.012	0.000
BRPF1	0.911	0.604	1.375	0.658	0.213
DLD	0.819	0.603	1.112	0.201	0.025
YEATS2	1.544	0.991	2.406	0.055	0.020
AFG3L2	0.616	0.439	0.864	0.005	0.001
MPC1	0.753	0.531	1.067	0.111	0.000
SLFN11	1.077	0.944	1.230	0.271	0.003
FDX1	0.811	0.560	1.173	0.266	0.047
COQ7	0.527	0.368	0.754	0.000	0.000
HAUS5	0.789	0.540	1.152	0.220	0.004
OXA1L	0.880	0.635	1.219	0.442	0.018
MCUR1	0.914	0.695	1.201	0.519	0.041
RPL3	1.087	0.602	1.963	0.781	0.105
CAPRIN1	0.716	0.449	1.143	0.162	0.006
EGLN1	1.085	0.767	1.535	0.644	0.173
MBTPS1	1.047	0.691	1.586	0.828	0.195
SOX2	1.084	1.003	1.172	0.041	0.000
SCAP	0.992	0.735	1.340	0.960	0.432
AHR	1.357	1.074	1.715	0.011	0.002

Table S4. The subtype of 27 cuproptosis-related genes in CRC patients.

ID	cluster
TCGA_TCGA-DM-A288	A
TCGA_TCGA-QL-A97D	B
TCGA_TCGA-CM-6164	A
TCGA_TCGA-G4-6299	B
TCGA_TCGA-AZ-4615	C
TCGA_TCGA-AA-3549	A
TCGA_TCGA-CM-4752	A
TCGA_TCGA-DM-A1D9	A
TCGA_TCGA-AA-3688	A
TCGA_TCGA-AA-3854	A
TCGA_TCGA-A6-3809	C
TCGA_TCGA-CM-6165	C
TCGA_TCGA-CM-4751	B
TCGA_TCGA-A6-5659	C
TCGA_TCGA-CM-4750	A
TCGA_TCGA-AZ-4682	A
TCGA_TCGA-G4-6625	B
TCGA_TCGA-DM-A0XF	A
TCGA_TCGA-AA-3949	C
TCGA_TCGA-AA-3848	A
TCGA_TCGA-CA-6715	A
TCGA_TCGA-AA-3710	C
TCGA_TCGA-AA-3950	B
TCGA_TCGA-AA-A00N	B
TCGA_TCGA-A6-6138	C
TCGA_TCGA-DM-A28E	A
TCGA_TCGA-D5-6538	B
TCGA_TCGA-AA-A00K	B
TCGA_TCGA-AA-3715	B
TCGA_TCGA-AA-3506	C
TCGA_TCGA-CK-5916	C
TCGA_TCGA-QG-A5Z1	C
TCGA_TCGA-AD-6890	C
TCGA_TCGA-A6-2685	C
TCGA_TCGA-AA-A00W	A
TCGA_TCGA-CK-5914	A
TCGA_TCGA-A6-2686	C
TCGA_TCGA-CA-5255	B
TCGA_TCGA-A6-A5ZU	C
TCGA_TCGA-NH-A50V	C
TCGA_TCGA-QG-A5YX	A
TCGA_TCGA-A6-2677	C
TCGA_TCGA-CA-6717	C
TCGA_TCGA-NH-A6GC	C
TCGA_TCGA-AA-3562	A
TCGA_TCGA-A6-4105	C
TCGA_TCGA-A6-6141	B
TCGA_TCGA-AZ-4684	A
TCGA_TCGA-CK-6747	B
TCGA_TCGA-AU-6004	C
TCGA_TCGA-A6-2672	C
TCGA_TCGA-QG-A5Z2	C
TCGA_TCGA-A6-6780	C
TCGA_TCGA-F4-6459	C
TCGA_TCGA-AA-3970	C
TCGA_TCGA-NH-A8F7	A

TCGA_TCGA-G4-6321	A
TCGA_TCGA-AA-3524	A
TCGA_TCGA-DM-A1D0	A
TCGA_TCGA-G4-6307	A
TCGA_TCGA-DM-A1HB	A
TCGA_TCGA-AA-3697	A
TCGA_TCGA-CM-6679	C
TCGA_TCGA-AY-6197	A
TCGA_TCGA-A6-2684	C
TCGA_TCGA-AZ-4323	C
TCGA_TCGA-AA-3971	A
TCGA_TCGA-AA-3989	A
TCGA_TCGA-AA-A00D	C
TCGA_TCGA-AD-6548	B
TCGA_TCGA-D5-6539	B
TCGA_TCGA-A6-2679	A
TCGA_TCGA-A6-2674	B
TCGA_TCGA-AZ-4614	B
TCGA_TCGA-NH-A5IV	C
TCGA_TCGA-A6-5664	C
TCGA_TCGA-AZ-4315	A
TCGA_TCGA-AA-3510	A
TCGA_TCGA-AY-A71X	A
TCGA_TCGA-AA-3488	A
TCGA_TCGA-DM-A28F	A
TCGA_TCGA-G4-6293	C
TCGA_TCGA-D5-6533	A
TCGA_TCGA-AA-3941	B
TCGA_TCGA-DM-A28K	B
TCGA_TCGA-CM-5344	C
TCGA_TCGA-AA-3517	C
TCGA_TCGA-G4-6309	C
TCGA_TCGA-AY-6386	B
TCGA_TCGA-AA-3678	A
TCGA_TCGA-AA-A01X	A
TCGA_TCGA-CM-6677	B
TCGA_TCGA-CM-5861	A
TCGA_TCGA-D5-6923	C
TCGA_TCGA-AZ-6605	C
TCGA_TCGA-NH-A6GB	A
TCGA_TCGA-CM-5349	C
TCGA_TCGA-NH-A50U	A
TCGA_TCGA-AA-3530	C
TCGA_TCGA-G4-6627	C
TCGA_TCGA-DM-A28G	A
TCGA_TCGA-D5-6537	C
TCGA_TCGA-D5-5539	C
TCGA_TCGA-CM-6163	C
TCGA_TCGA-CM-4744	A
TCGA_TCGA-AA-A004	A
TCGA_TCGA-A6-3808	C
TCGA_TCGA-AA-A02J	A
TCGA_TCGA-AA-3837	C
TCGA_TCGA-CK-4947	C
TCGA_TCGA-CA-5256	A
TCGA_TCGA-CM-6167	C
TCGA_TCGA-4N-A93T	A
TCGA_TCGA-CA-5797	C

TCGA_TCGA-CM-6166	A
TCGA_TCGA-CK-4951	C
TCGA_TCGA-CA-6719	C
TCGA_TCGA-AA-A02R	A
TCGA_TCGA-A6-5666	C
TCGA_TCGA-AY-5543	A
TCGA_TCGA-F4-6704	B
TCGA_TCGA-AA-A00Q	A
TCGA_TCGA-A6-6781	C
TCGA_TCGA-AA-A00F	C
TCGA_TCGA-D5-6920	C
TCGA_TCGA-AA-3815	C
TCGA_TCGA-D5-5538	B
TCGA_TCGA-AA-3867	C
TCGA_TCGA-A6-6782	C
TCGA_TCGA-A6-6654	C
TCGA_TCGA-AA-A01D	B
TCGA_TCGA-G4-6311	C
TCGA_TCGA-A6-3807	C
TCGA_TCGA-AZ-5403	C
TCGA_TCGA-AD-6888	A
TCGA_TCGA-G4-6628	C
TCGA_TCGA-DM-A1D6	C
TCGA_TCGA-CM-5341	C
TCGA_TCGA-AA-3862	A
TCGA_TCGA-AA-3939	B
TCGA_TCGA-AA-3660	C
TCGA_TCGA-AA-3833	C
TCGA_TCGA-AA-A01P	B
TCGA_TCGA-AA-A00Z	A
TCGA_TCGA-CM-5863	B
TCGA_TCGA-AZ-6598	A
TCGA_TCGA-AA-3679	A
TCGA_TCGA-G4-6304	B
TCGA_TCGA-DM-A285	C
TCGA_TCGA-G4-6294	A
TCGA_TCGA-A6-4107	C
TCGA_TCGA-DM-A1D4	B
TCGA_TCGA-CA-6716	A
TCGA_TCGA-CK-4952	B
TCGA_TCGA-AA-3844	B
TCGA_TCGA-D5-6541	C
TCGA_TCGA-AA-A01G	A
TCGA_TCGA-AA-3870	C
TCGA_TCGA-D5-6532	A
TCGA_TCGA-CM-6169	C
TCGA_TCGA-D5-5541	C
TCGA_TCGA-DM-A1DB	A
TCGA_TCGA-AA-A00A	A
TCGA_TCGA-D5-6530	C
TCGA_TCGA-CM-5868	A
TCGA_TCGA-AA-3519	C
TCGA_TCGA-AA-3526	C
TCGA_TCGA-DM-A28M	A
TCGA_TCGA-D5-6926	C
TCGA_TCGA-AA-3534	C
TCGA_TCGA-G4-6317	A
TCGA_TCGA-AA-A03J	A

TCGA_TCGA-CM-6680	C
TCGA_TCGA-D5-6536	C
TCGA_TCGA-AD-A5EK	C
TCGA_TCGA-AA-3663	C
TCGA_TCGA-AZ-6607	C
TCGA_TCGA-AZ-5407	A
TCGA_TCGA-G4-6314	B
TCGA_TCGA-CM-5348	C
TCGA_TCGA-CK-6751	A
TCGA_TCGA-D5-6929	C
TCGA_TCGA-AA-3869	C
TCGA_TCGA-DM-A282	A
TCGA_TCGA-D5-7000	C
TCGA_TCGA-G4-6298	A
TCGA_TCGA-NH-A8F8	A
TCGA_TCGA-AA-3966	B
TCGA_TCGA-AA-3516	C
TCGA_TCGA-AA-3846	C
TCGA_TCGA-AA-3544	A
TCGA_TCGA-AA-3956	A
TCGA_TCGA-AA-3532	C
TCGA_TCGA-AA-3955	A
TCGA_TCGA-AD-6895	C
TCGA_TCGA-AA-3556	C
TCGA_TCGA-CM-6678	A
TCGA_TCGA-AA-3851	A
TCGA_TCGA-CM-5860	C
TCGA_TCGA-AA-A02F	A
TCGA_TCGA-DM-A1DA	A
TCGA_TCGA-CM-6162	C
TCGA_TCGA-AA-3522	A
TCGA_TCGA-AA-A00U	A
TCGA_TCGA-AA-3930	C
TCGA_TCGA-AZ-6599	C
TCGA_TCGA-AA-3947	C
TCGA_TCGA-CM-6171	B
TCGA_TCGA-CM-4743	B
TCGA_TCGA-A6-2675	C
TCGA_TCGA-G4-6297	C
TCGA_TCGA-A6-5656	C
TCGA_TCGA-DM-A0X9	B
TCGA_TCGA-AA-3553	C
TCGA_TCGA-DM-A0XD	A
TCGA_TCGA-AA-3511	C
TCGA_TCGA-A6-5667	A
TCGA_TCGA-AA-3866	C
TCGA_TCGA-AA-A017	A
TCGA_TCGA-CM-6674	C
TCGA_TCGA-DM-A1D8	B
TCGA_TCGA-CM-6170	A
TCGA_TCGA-AZ-4616	B
TCGA_TCGA-AA-3994	C
TCGA_TCGA-AA-3877	C
TCGA_TCGA-WS-AB45	C
TCGA_TCGA-AA-A00L	A
TCGA_TCGA-CM-5864	B
TCGA_TCGA-CA-5254	A
TCGA_TCGA-D5-6898	C

TCGA_TCGA-AA-3696	A
TCGA_TCGA-DM-A280	A
TCGA_TCGA-QG-A5YW	A
TCGA_TCGA-CK-4948	C
TCGA_TCGA-DM-A1HA	A
TCGA_TCGA-F4-6809	B
TCGA_TCGA-CM-4748	C
TCGA_TCGA-AA-3858	A
TCGA_TCGA-D5-6531	C
TCGA_TCGA-AA-3662	C
TCGA_TCGA-AA-A02K	B
TCGA_TCGA-AD-6889	B
TCGA_TCGA-AA-A01R	A
TCGA_TCGA-AA-3975	A
TCGA_TCGA-5M-AAT6	C
TCGA_TCGA-D5-6931	C
TCGA_TCGA-D5-6924	C
TCGA_TCGA-AA-3860	C
TCGA_TCGA-A6-5665	C
TCGA_TCGA-D5-6540	C
TCGA_TCGA-CA-6718	B
TCGA_TCGA-G4-6323	A
TCGA_TCGA-A6-A566	C
TCGA_TCGA-AZ-6608	A
TCGA_TCGA-A6-5662	A
TCGA_TCGA-AA-3831	C
TCGA_TCGA-AA-A02H	A
TCGA_TCGA-CM-6676	A
TCGA_TCGA-AD-6899	B
TCGA_TCGA-AA-3685	C
TCGA_TCGA-AA-3548	A
TCGA_TCGA-F4-6569	C
TCGA_TCGA-F4-6461	C
TCGA_TCGA-F4-6856	C
TCGA_TCGA-AY-A54L	A
TCGA_TCGA-AA-3538	C
TCGA_TCGA-AA-3842	C
TCGA_TCGA-AZ-4308	A
TCGA_TCGA-AA-3509	A
TCGA_TCGA-AA-3680	C
TCGA_TCGA-AA-3542	A
TCGA_TCGA-AD-6964	C
TCGA_TCGA-A6-6650	C
TCGA_TCGA-A6-6140	A
TCGA_TCGA-AA-A024	A
TCGA_TCGA-A6-2682	B
TCGA_TCGA-CM-6675	A
TCGA_TCGA-DM-A1D7	B
TCGA_TCGA-4T-AA8H	A
TCGA_TCGA-A6-6653	A
TCGA_TCGA-AA-3666	B
TCGA_TCGA-F4-6460	C
TCGA_TCGA-AA-3655	C
TCGA_TCGA-AA-3952	C
TCGA_TCGA-T9-A92H	A
TCGA_TCGA-AY-4070	B
TCGA_TCGA-AY-A8YK	A
TCGA_TCGA-CK-4950	A

TCGA_TCGA-F4-6570	B
TCGA_TCGA-AA-A00J	C
TCGA_TCGA-AA-3667	A
TCGA_TCGA-AA-3554	C
TCGA_TCGA-AZ-6606	A
TCGA_TCGA-D5-6922	C
TCGA_TCGA-A6-6137	A
TCGA_TCGA-AA-3495	C
TCGA_TCGA-AA-3489	C
TCGA_TCGA-G4-6322	A
TCGA_TCGA-SS-A7HO	B
TCGA_TCGA-AA-3976	C
TCGA_TCGA-AA-3811	C
TCGA_TCGA-AA-3819	A
TCGA_TCGA-AA-A02Y	A
TCGA_TCGA-AZ-6603	C
TCGA_TCGA-G4-6626	A
TCGA_TCGA-AA-3875	C
TCGA_TCGA-G4-6306	A
TCGA_TCGA-G4-6320	A
TCGA_TCGA-AA-3864	C
TCGA_TCGA-A6-6142	C
TCGA_TCGA-A6-2678	C
TCGA_TCGA-CA-5796	A
TCGA_TCGA-A6-A567	A
TCGA_TCGA-AA-3713	C
TCGA_TCGA-A6-6648	A
TCGA_TCGA-A6-A565	C
TCGA_TCGA-A6-5660	A
TCGA_TCGA-AZ-6601	C
TCGA_TCGA-5M-AATE	A
TCGA_TCGA-CM-6161	C
TCGA_TCGA-AZ-4313	A
TCGA_TCGA-A6-6652	A
TCGA_TCGA-AA-A00E	C
TCGA_TCGA-F4-6808	A
TCGA_TCGA-AA-A02W	A
TCGA_TCGA-DM-A28C	A
TCGA_TCGA-AA-3681	C
TCGA_TCGA-AA-3492	A
TCGA_TCGA-D5-6535	C
TCGA_TCGA-AD-6963	A
TCGA_TCGA-CM-4747	A
TCGA_TCGA-NH-A50T	A
TCGA_TCGA-CM-6168	C
TCGA_TCGA-AA-A01T	A
TCGA_TCGA-RU-A8FL	A
TCGA_TCGA-AA-3812	B
TCGA_TCGA-AY-A69D	B
TCGA_TCGA-A6-3810	C
TCGA_TCGA-DM-A28H	B
TCGA_TCGA-G4-6586	A
TCGA_TCGA-D5-6928	C
TCGA_TCGA-G4-6315	A
TCGA_TCGA-AA-3664	C
TCGA_TCGA-G4-6303	C
TCGA_TCGA-AA-3692	C
TCGA_TCGA-AA-3968	C

TCGA_TCGA-AA-3560	A
TCGA_TCGA-D5-5537	B
TCGA_TCGA-D5-6932	A
TCGA_TCGA-AA-3982	C
TCGA_TCGA-DM-A28A	B
TCGA_TCGA-CM-6172	A
TCGA_TCGA-D5-6930	B
TCGA_TCGA-D5-6529	C
TCGA_TCGA-A6-2683	A
TCGA_TCGA-CK-5913	C
TCGA_TCGA-D5-5540	A
TCGA_TCGA-AD-6901	C
TCGA_TCGA-AA-A01F	A
TCGA_TCGA-AA-3552	C
TCGA_TCGA-F4-6855	C
TCGA_TCGA-AD-5900	C
TCGA_TCGA-AA-3979	A
TCGA_TCGA-AA-A01I	A
TCGA_TCGA-AA-A03F	A
TCGA_TCGA-3L-AA1B	C
TCGA_TCGA-AA-3502	A
TCGA_TCGA-AA-A02E	A
TCGA_TCGA-CM-4746	A
TCGA_TCGA-G4-6295	B
TCGA_TCGA-AA-A01C	A
TCGA_TCGA-G4-6588	A
TCGA_TCGA-A6-2680	A
TCGA_TCGA-QG-A5YV	A
TCGA_TCGA-AA-3561	A
TCGA_TCGA-AA-3525	A
TCGA_TCGA-AA-3531	A
TCGA_TCGA-AA-3973	A
TCGA_TCGA-AA-3555	C
TCGA_TCGA-EI-6884	C
TCGA_TCGA-AG-3582	A
TCGA_TCGA-DC-6154	A
TCGA_TCGA-AG-3611	A
TCGA_TCGA-AG-3609	C
TCGA_TCGA-AG-4022	C
TCGA_TCGA-AG-A02N	A
TCGA_TCGA-AH-6549	C
TCGA_TCGA-AH-6903	C
TCGA_TCGA-F5-6863	A
TCGA_TCGA-AG-3887	A
TCGA_TCGA-AF-5654	A
TCGA_TCGA-DY-A1DE	A
TCGA_TCGA-AF-3913	A
TCGA_TCGA-AG-A014	A
TCGA_TCGA-G5-6572	C
TCGA_TCGA-AG-A011	A
TCGA_TCGA-EI-6511	C
TCGA_TCGA-AG-4008	C
TCGA_TCGA-AG-3731	C
TCGA_TCGA-AH-6544	A
TCGA_TCGA-AG-A026	A
TCGA_TCGA-EI-7004	C
TCGA_TCGA-DC-4745	A
TCGA_TCGA-EI-6883	A

TCGA_TCGA-EI-6917	C
TCGA_TCGA-F5-6861	A
TCGA_TCGA-F5-6465	C
TCGA_TCGA-AF-6672	A
TCGA_TCGA-AG-3885	A
TCGA_TCGA-EI-6509	C
TCGA_TCGA-DC-5337	A
TCGA_TCGA-AG-3893	A
TCGA_TCGA-AG-3901	C
TCGA_TCGA-AG-3726	A
TCGA_TCGA-DC-6160	A
TCGA_TCGA-AG-3581	C
TCGA_TCGA-F5-6812	C
TCGA_TCGA-AG-4001	C
TCGA_TCGA-AG-3909	A
TCGA_TCGA-AG-3592	A
TCGA_TCGA-EI-6513	A
TCGA_TCGA-DC-6158	C
TCGA_TCGA-EI-6512	C
TCGA_TCGA-CI-6622	A
TCGA_TCGA-BM-6198	C
TCGA_TCGA-DY-A1DC	B
TCGA_TCGA-AG-3600	A
TCGA_TCGA-AG-3599	A
TCGA_TCGA-AG-3584	C
TCGA_TCGA-DT-5265	C
TCGA_TCGA-AG-A036	B
TCGA_TCGA-AG-3593	C
TCGA_TCGA-AG-3580	C
TCGA_TCGA-DC-6683	A
TCGA_TCGA-AF-6655	C
TCGA_TCGA-AG-3728	A
TCGA_TCGA-AF-3400	C
TCGA_TCGA-AF-2691	C
TCGA_TCGA-CL-5918	A
TCGA_TCGA-F5-6864	C
TCGA_TCGA-AG-A01N	A
TCGA_TCGA-DC-5869	A
TCGA_TCGA-AG-3608	A
TCGA_TCGA-DY-A0XA	A
TCGA_TCGA-AG-3594	B
TCGA_TCGA-EF-5831	A
TCGA_TCGA-AG-3725	A
TCGA_TCGA-EI-6514	A
TCGA_TCGA-CI-6624	C
TCGA_TCGA-CI-6620	B
TCGA_TCGA-CI-6621	C
TCGA_TCGA-EI-6510	A
TCGA_TCGA-AG-A02X	A
TCGA_TCGA-AG-3894	A
TCGA_TCGA-AF-A56N	A
TCGA_TCGA-AG-A00C	A
TCGA_TCGA-EI-6885	C
TCGA_TCGA-AG-3732	C
TCGA_TCGA-AG-A016	A
TCGA_TCGA-EI-6882	C
TCGA_TCGA-AG-3575	C
TCGA_TCGA-AF-2693	C

TCGA_TCGA-DC-6157	A
TCGA_TCGA-AF-4110	C
TCGA_TCGA-AG-3591	C
TCGA_TCGA-EI-6881	A
TCGA_TCGA-AG-A008	C
TCGA_TCGA-AG-A015	A
TCGA_TCGA-AG-3892	C
TCGA_TCGA-G5-6233	C
TCGA_TCGA-AG-3898	A
TCGA_TCGA-AG-3578	C
TCGA_TCGA-DC-6682	A
TCGA_TCGA-F5-6813	C
TCGA_TCGA-F5-6702	C
TCGA_TCGA-CL-5917	A
TCGA_TCGA-CL-4957	A
TCGA_TCGA-EI-6507	C
TCGA_TCGA-DC-4749	A
TCGA_TCGA-AF-2690	C
TCGA_TCGA-EF-5830	A
TCGA_TCGA-AF-3911	C
TCGA_TCGA-DC-6155	A
TCGA_TCGA-AF-A56L	A
TCGA_TCGA-CI-6623	A
TCGA_TCGA-AH-6897	A
TCGA_TCGA-AG-3574	A
TCGA_TCGA-G5-6235	A
TCGA_TCGA-AG-4005	A
TCGA_TCGA-AG-3583	C
TCGA_TCGA-AH-6547	C
TCGA_TCGA-EI-7002	A
TCGA_TCGA-AG-A00Y	A
TCGA_TCGA-EI-6506	A
TCGA_TCGA-AH-6643	B
TCGA_TCGA-AG-3890	C
TCGA_TCGA-F5-6571	B
TCGA_TCGA-AG-3882	A
TCGA_TCGA-AG-3598	A
TCGA_TCGA-F5-6811	C
TCGA_TCGA-F5-6464	B
TCGA_TCGA-CI-6619	B
TCGA_TCGA-AF-2692	C
GSE17536_GSM437093	A
GSE17536_GSM437094	A
GSE17536_GSM437095	A
GSE17536_GSM437096	A
GSE17536_GSM437097	A
GSE17536_GSM437098	C
GSE17536_GSM437099	A
GSE17536_GSM437100	C
GSE17536_GSM437101	B
GSE17536_GSM437102	C
GSE17536_GSM437103	A
GSE17536_GSM437104	A
GSE17536_GSM437105	A
GSE17536_GSM437106	A
GSE17536_GSM437107	A
GSE17536_GSM437108	A
GSE17536_GSM437109	C

GSE17536_GSM437110	A
GSE17536_GSM437111	C
GSE17536_GSM437112	A
GSE17536_GSM437113	A
GSE17536_GSM437114	B
GSE17536_GSM437115	A
GSE17536_GSM437116	A
GSE17536_GSM437117	A
GSE17536_GSM437118	B
GSE17536_GSM437119	C
GSE17536_GSM437120	C
GSE17536_GSM437121	C
GSE17536_GSM437122	A
GSE17536_GSM437124	C
GSE17536_GSM437125	C
GSE17536_GSM437126	A
GSE17536_GSM437127	A
GSE17536_GSM437128	A
GSE17536_GSM437129	A
GSE17536_GSM437130	B
GSE17536_GSM437131	A
GSE17536_GSM437132	A
GSE17536_GSM437133	C
GSE17536_GSM437134	A
GSE17536_GSM437135	C
GSE17536_GSM437136	A
GSE17536_GSM437137	B
GSE17536_GSM437138	B
GSE17536_GSM437139	A
GSE17536_GSM437140	A
GSE17536_GSM437141	C
GSE17536_GSM437142	A
GSE17536_GSM437143	A
GSE17536_GSM437144	A
GSE17536_GSM437145	B
GSE17536_GSM437146	A
GSE17536_GSM437147	B
GSE17536_GSM437148	B
GSE17536_GSM437149	C
GSE17536_GSM437150	A
GSE17536_GSM437151	A
GSE17536_GSM437152	A
GSE17536_GSM437153	A
GSE17536_GSM437154	A
GSE17536_GSM437155	C
GSE17536_GSM437156	A
GSE17536_GSM437157	B
GSE17536_GSM437158	A
GSE17536_GSM437159	B
GSE17536_GSM437160	C
GSE17536_GSM437161	C
GSE17536_GSM437162	A
GSE17536_GSM437163	A
GSE17536_GSM437164	A
GSE17536_GSM437166	C
GSE17536_GSM437167	A
GSE17536_GSM437168	A
GSE17536_GSM437169	A

GSE17536_GSM437170	A
GSE17536_GSM437171	C
GSE17536_GSM437172	A
GSE17536_GSM437173	C
GSE17536_GSM437174	C
GSE17536_GSM437175	C
GSE17536_GSM437176	A
GSE17536_GSM437177	A
GSE17536_GSM437178	C
GSE17536_GSM437179	A
GSE17536_GSM437180	C
GSE17536_GSM437181	C
GSE17536_GSM437182	A
GSE17536_GSM437183	B
GSE17536_GSM437184	B
GSE17536_GSM437185	A
GSE17536_GSM437186	A
GSE17536_GSM437187	A
GSE17536_GSM437188	A
GSE17536_GSM437189	A
GSE17536_GSM437190	C
GSE17536_GSM437191	A
GSE17536_GSM437192	A
GSE17536_GSM437193	A
GSE17536_GSM437194	A
GSE17536_GSM437195	A
GSE17536_GSM437196	A
GSE17536_GSM437197	C
GSE17536_GSM437198	C
GSE17536_GSM437199	C
GSE17536_GSM437200	C
GSE17536_GSM437201	C
GSE17536_GSM437202	C
GSE17536_GSM437203	C
GSE17536_GSM437205	A
GSE17536_GSM437206	A
GSE17536_GSM437207	C
GSE17536_GSM437208	A
GSE17536_GSM437209	C
GSE17536_GSM437210	C
GSE17536_GSM437211	B
GSE17536_GSM437212	C
GSE17536_GSM437213	A
GSE17536_GSM437214	A
GSE17536_GSM437215	A
GSE17536_GSM437216	A
GSE17536_GSM437217	C
GSE17536_GSM437218	B
GSE17536_GSM437219	A
GSE17536_GSM437220	A
GSE17536_GSM437221	A
GSE17536_GSM437222	C
GSE17536_GSM437223	C
GSE17536_GSM437224	B
GSE17536_GSM437225	C
GSE17536_GSM437226	C
GSE17536_GSM437227	C
GSE17536_GSM437228	A

GSE17536_GSM437229	B
GSE17536_GSM437230	A
GSE17536_GSM437232	C
GSE17536_GSM437238	B
GSE17536_GSM437243	B
GSE17536_GSM437248	A
GSE17536_GSM437249	C
GSE17536_GSM437255	A
GSE17536_GSM437261	A
GSE17536_GSM437264	A
GSE17536_GSM437265	C
GSE17536_GSM437266	C
GSE17537_GSM437270	C
GSE17537_GSM437271	C
GSE17537_GSM437272	B
GSE17537_GSM437273	B
GSE17537_GSM437274	A
GSE17537_GSM437275	A
GSE17537_GSM437276	C
GSE17537_GSM437277	A
GSE17537_GSM437278	A
GSE17537_GSM437279	A
GSE17537_GSM437280	C
GSE17537_GSM437281	A
GSE17537_GSM437282	B
GSE17537_GSM437283	A
GSE17537_GSM437284	C
GSE17537_GSM437285	A
GSE17537_GSM437287	A
GSE17537_GSM437288	A
GSE17537_GSM437289	A
GSE17537_GSM437290	C
GSE17537_GSM437291	A
GSE17537_GSM437292	A
GSE17537_GSM437294	A
GSE17537_GSM437296	A
GSE17537_GSM437297	C
GSE17537_GSM437298	A
GSE17537_GSM437299	B
GSE17537_GSM437300	A
GSE17537_GSM437301	C
GSE17537_GSM437303	C
GSE17537_GSM437304	A
GSE17537_GSM437306	C
GSE17537_GSM437307	C
GSE17537_GSM437309	A
GSE17537_GSM437310	A
GSE17537_GSM437311	B
GSE17537_GSM437312	A
GSE17537_GSM437313	B
GSE17537_GSM437314	B
GSE17537_GSM437315	C
GSE17537_GSM437323	A
GSE17537_GSM437324	A
GSE39582_GSM971957	B
GSE39582_GSM971958	C
GSE39582_GSM971959	C
GSE39582_GSM971960	A

GSE39582_GSM971961	C
GSE39582_GSM971962	C
GSE39582_GSM971963	C
GSE39582_GSM971964	A
GSE39582_GSM971965	A
GSE39582_GSM971966	B
GSE39582_GSM971968	B
GSE39582_GSM971969	C
GSE39582_GSM971970	C
GSE39582_GSM971971	A
GSE39582_GSM971972	C
GSE39582_GSM971973	A
GSE39582_GSM971974	A
GSE39582_GSM971975	A
GSE39582_GSM971976	A
GSE39582_GSM971977	C
GSE39582_GSM971978	C
GSE39582_GSM971979	C
GSE39582_GSM971980	C
GSE39582_GSM971981	C
GSE39582_GSM971982	C
GSE39582_GSM971983	C
GSE39582_GSM971984	A
GSE39582_GSM971985	A
GSE39582_GSM971986	A
GSE39582_GSM971987	B
GSE39582_GSM971988	C
GSE39582_GSM971989	A
GSE39582_GSM971990	A
GSE39582_GSM971991	A
GSE39582_GSM971992	A
GSE39582_GSM971993	A
GSE39582_GSM971994	B
GSE39582_GSM971995	A
GSE39582_GSM971996	C
GSE39582_GSM971997	B
GSE39582_GSM971998	A
GSE39582_GSM971999	C
GSE39582_GSM972000	A
GSE39582_GSM972001	A
GSE39582_GSM972002	A
GSE39582_GSM972003	A
GSE39582_GSM972004	A
GSE39582_GSM972005	A
GSE39582_GSM972006	B
GSE39582_GSM972007	C
GSE39582_GSM972008	C
GSE39582_GSM972009	C
GSE39582_GSM972010	C
GSE39582_GSM972011	C
GSE39582_GSM972012	A
GSE39582_GSM972013	C
GSE39582_GSM972014	A
GSE39582_GSM972015	A
GSE39582_GSM972016	A
GSE39582_GSM972017	A
GSE39582_GSM972018	C
GSE39582_GSM972019	C

GSE39582_GSM972020	C
GSE39582_GSM972021	C
GSE39582_GSM972022	B
GSE39582_GSM972023	A
GSE39582_GSM972024	A
GSE39582_GSM972025	A
GSE39582_GSM972026	C
GSE39582_GSM972027	A
GSE39582_GSM972028	A
GSE39582_GSM972029	A
GSE39582_GSM972030	B
GSE39582_GSM972031	C
GSE39582_GSM972032	A
GSE39582_GSM972033	A
GSE39582_GSM972034	A
GSE39582_GSM972035	A
GSE39582_GSM972036	B
GSE39582_GSM972037	A
GSE39582_GSM972038	B
GSE39582_GSM972039	C
GSE39582_GSM972040	A
GSE39582_GSM972041	A
GSE39582_GSM972042	A
GSE39582_GSM972043	A
GSE39582_GSM972044	B
GSE39582_GSM972045	A
GSE39582_GSM972046	C
GSE39582_GSM972047	B
GSE39582_GSM972048	A
GSE39582_GSM972049	A
GSE39582_GSM972050	A
GSE39582_GSM972051	C
GSE39582_GSM972052	A
GSE39582_GSM972053	A
GSE39582_GSM972054	B
GSE39582_GSM972055	B
GSE39582_GSM972056	C
GSE39582_GSM972057	A
GSE39582_GSM972058	C
GSE39582_GSM972059	C
GSE39582_GSM972060	A
GSE39582_GSM972061	B
GSE39582_GSM972062	A
GSE39582_GSM972063	C
GSE39582_GSM972064	C
GSE39582_GSM972065	C
GSE39582_GSM972066	A
GSE39582_GSM972067	C
GSE39582_GSM972068	C
GSE39582_GSM972069	A
GSE39582_GSM972070	A
GSE39582_GSM972071	A
GSE39582_GSM972072	C
GSE39582_GSM972073	C
GSE39582_GSM972074	A
GSE39582_GSM972075	A
GSE39582_GSM972076	C
GSE39582_GSM972077	C

GSE39582_GSM972078	C
GSE39582_GSM972079	C
GSE39582_GSM972080	C
GSE39582_GSM972081	A
GSE39582_GSM972082	C
GSE39582_GSM972083	C
GSE39582_GSM972084	C
GSE39582_GSM972085	C
GSE39582_GSM972086	A
GSE39582_GSM972087	A
GSE39582_GSM972088	C
GSE39582_GSM972089	C
GSE39582_GSM972090	A
GSE39582_GSM972091	A
GSE39582_GSM972092	C
GSE39582_GSM972093	A
GSE39582_GSM972094	C
GSE39582_GSM972095	C
GSE39582_GSM972096	A
GSE39582_GSM972097	C
GSE39582_GSM972098	A
GSE39582_GSM972099	A
GSE39582_GSM972100	A
GSE39582_GSM972101	A
GSE39582_GSM972102	A
GSE39582_GSM972103	A
GSE39582_GSM972104	C
GSE39582_GSM972105	A
GSE39582_GSM972106	B
GSE39582_GSM972107	A
GSE39582_GSM972108	A
GSE39582_GSM972109	A
GSE39582_GSM972110	A
GSE39582_GSM972111	A
GSE39582_GSM972112	C
GSE39582_GSM972113	C
GSE39582_GSM972114	A
GSE39582_GSM972115	A
GSE39582_GSM972116	B
GSE39582_GSM972117	B
GSE39582_GSM972118	A
GSE39582_GSM972119	A
GSE39582_GSM972120	B
GSE39582_GSM972121	B
GSE39582_GSM972122	A
GSE39582_GSM972123	A
GSE39582_GSM972124	A
GSE39582_GSM972125	A
GSE39582_GSM972126	A
GSE39582_GSM972127	A
GSE39582_GSM972128	A
GSE39582_GSM972129	B
GSE39582_GSM972130	A
GSE39582_GSM972131	B
GSE39582_GSM972132	C
GSE39582_GSM972133	C
GSE39582_GSM972134	B
GSE39582_GSM972135	A

GSE39582_GSM972136	C
GSE39582_GSM972137	A
GSE39582_GSM972138	A
GSE39582_GSM972139	A
GSE39582_GSM972140	B
GSE39582_GSM972141	C
GSE39582_GSM972142	C
GSE39582_GSM972143	A
GSE39582_GSM972144	A
GSE39582_GSM972145	A
GSE39582_GSM972146	C
GSE39582_GSM972147	C
GSE39582_GSM972148	B
GSE39582_GSM972149	A
GSE39582_GSM972150	A
GSE39582_GSM972151	A
GSE39582_GSM972152	B
GSE39582_GSM972153	A
GSE39582_GSM972154	A
GSE39582_GSM972155	C
GSE39582_GSM972156	A
GSE39582_GSM972157	A
GSE39582_GSM972158	C
GSE39582_GSM972159	A
GSE39582_GSM972160	A
GSE39582_GSM972161	B
GSE39582_GSM972162	C
GSE39582_GSM972163	A
GSE39582_GSM972164	A
GSE39582_GSM972165	C
GSE39582_GSM972166	A
GSE39582_GSM972167	A
GSE39582_GSM972168	C
GSE39582_GSM972169	A
GSE39582_GSM972170	C
GSE39582_GSM972171	C
GSE39582_GSM972172	B
GSE39582_GSM972173	A
GSE39582_GSM972174	A
GSE39582_GSM972175	A
GSE39582_GSM972176	B
GSE39582_GSM972177	A
GSE39582_GSM972178	A
GSE39582_GSM972179	C
GSE39582_GSM972180	C
GSE39582_GSM972181	C
GSE39582_GSM972182	A
GSE39582_GSM972183	A
GSE39582_GSM972184	C
GSE39582_GSM972185	A
GSE39582_GSM972186	B
GSE39582_GSM972187	C
GSE39582_GSM972188	A
GSE39582_GSM972189	B
GSE39582_GSM972190	A
GSE39582_GSM972191	A
GSE39582_GSM972192	C
GSE39582_GSM972193	A

GSE39582_GSM972194	A
GSE39582_GSM972195	A
GSE39582_GSM972196	A
GSE39582_GSM972197	A
GSE39582_GSM972198	A
GSE39582_GSM972199	B
GSE39582_GSM972200	A
GSE39582_GSM972201	C
GSE39582_GSM972202	A
GSE39582_GSM972203	A
GSE39582_GSM972204	C
GSE39582_GSM972205	A
GSE39582_GSM972206	B
GSE39582_GSM972207	A
GSE39582_GSM972208	C
GSE39582_GSM972209	C
GSE39582_GSM972210	A
GSE39582_GSM972211	A
GSE39582_GSM972212	A
GSE39582_GSM972213	C
GSE39582_GSM972214	A
GSE39582_GSM972215	A
GSE39582_GSM972216	A
GSE39582_GSM972217	A
GSE39582_GSM972218	A
GSE39582_GSM972219	A
GSE39582_GSM972220	C
GSE39582_GSM972221	A
GSE39582_GSM972222	C
GSE39582_GSM972223	C
GSE39582_GSM972224	C
GSE39582_GSM972225	C
GSE39582_GSM972226	C
GSE39582_GSM972227	C
GSE39582_GSM972228	A
GSE39582_GSM972229	C
GSE39582_GSM972230	A
GSE39582_GSM972231	C
GSE39582_GSM972232	C
GSE39582_GSM972233	A
GSE39582_GSM972234	C
GSE39582_GSM972235	C
GSE39582_GSM972236	A
GSE39582_GSM972237	A
GSE39582_GSM972238	C
GSE39582_GSM972239	C
GSE39582_GSM972240	B
GSE39582_GSM972241	A
GSE39582_GSM972242	B
GSE39582_GSM972243	A
GSE39582_GSM972244	A
GSE39582_GSM972245	A
GSE39582_GSM972246	C
GSE39582_GSM972247	A
GSE39582_GSM972248	A
GSE39582_GSM972249	A
GSE39582_GSM972250	A
GSE39582_GSM972251	C

GSE39582_GSM972252	C
GSE39582_GSM972253	A
GSE39582_GSM972254	C
GSE39582_GSM972255	C
GSE39582_GSM972256	A
GSE39582_GSM972257	A
GSE39582_GSM972258	A
GSE39582_GSM972259	C
GSE39582_GSM972260	A
GSE39582_GSM972261	C
GSE39582_GSM972262	C
GSE39582_GSM972263	A
GSE39582_GSM972264	A
GSE39582_GSM972265	C
GSE39582_GSM972266	A
GSE39582_GSM972267	A
GSE39582_GSM972268	A
GSE39582_GSM972269	A
GSE39582_GSM972270	C
GSE39582_GSM972271	B
GSE39582_GSM972272	A
GSE39582_GSM972273	A
GSE39582_GSM972274	B
GSE39582_GSM972275	C
GSE39582_GSM972276	C
GSE39582_GSM972277	B
GSE39582_GSM972278	B
GSE39582_GSM972279	B
GSE39582_GSM972280	C
GSE39582_GSM972281	C
GSE39582_GSM972282	C
GSE39582_GSM972283	B
GSE39582_GSM972284	A
GSE39582_GSM972285	C
GSE39582_GSM972286	C
GSE39582_GSM972287	C
GSE39582_GSM972288	A
GSE39582_GSM972289	A
GSE39582_GSM972290	C
GSE39582_GSM972291	A
GSE39582_GSM972292	C
GSE39582_GSM972293	C
GSE39582_GSM972294	B
GSE39582_GSM972295	B
GSE39582_GSM972296	C
GSE39582_GSM972297	C
GSE39582_GSM972298	A
GSE39582_GSM972299	A
GSE39582_GSM972300	C
GSE39582_GSM972301	C
GSE39582_GSM972302	A
GSE39582_GSM972303	C
GSE39582_GSM972304	A
GSE39582_GSM972305	B
GSE39582_GSM972306	B
GSE39582_GSM972307	A
GSE39582_GSM972308	B
GSE39582_GSM972309	A

GSE39582_GSM972310	A
GSE39582_GSM972311	C
GSE39582_GSM972312	A
GSE39582_GSM972313	A
GSE39582_GSM972314	A
GSE39582_GSM972315	A
GSE39582_GSM972316	C
GSE39582_GSM972317	A
GSE39582_GSM972318	A
GSE39582_GSM972319	A
GSE39582_GSM972320	A
GSE39582_GSM972321	C
GSE39582_GSM972322	C
GSE39582_GSM972323	A
GSE39582_GSM972324	A
GSE39582_GSM972325	A
GSE39582_GSM972326	A
GSE39582_GSM972327	C
GSE39582_GSM972328	A
GSE39582_GSM972329	A
GSE39582_GSM972330	C
GSE39582_GSM972331	A
GSE39582_GSM972332	C
GSE39582_GSM972333	A
GSE39582_GSM972334	B
GSE39582_GSM972335	A
GSE39582_GSM972336	A
GSE39582_GSM972337	C
GSE39582_GSM972338	A
GSE39582_GSM972339	C
GSE39582_GSM972340	A
GSE39582_GSM972341	A
GSE39582_GSM972342	A
GSE39582_GSM972343	A
GSE39582_GSM972344	B
GSE39582_GSM972345	C
GSE39582_GSM972346	A
GSE39582_GSM972347	A
GSE39582_GSM972348	A
GSE39582_GSM972349	C
GSE39582_GSM972351	A
GSE39582_GSM972352	C
GSE39582_GSM972353	C
GSE39582_GSM972354	C
GSE39582_GSM972355	C
GSE39582_GSM972357	A
GSE39582_GSM972358	C
GSE39582_GSM972359	B
GSE39582_GSM972360	B
GSE39582_GSM972361	C
GSE39582_GSM972362	A
GSE39582_GSM972363	C
GSE39582_GSM972364	A
GSE39582_GSM972365	C
GSE39582_GSM972366	C
GSE39582_GSM972367	C
GSE39582_GSM972368	A
GSE39582_GSM972369	C

GSE39582_GSM972370	A
GSE39582_GSM972371	A
GSE39582_GSM972372	A
GSE39582_GSM972373	A
GSE39582_GSM972374	A
GSE39582_GSM972375	A
GSE39582_GSM972376	A
GSE39582_GSM972377	C
GSE39582_GSM972378	A
GSE39582_GSM972379	A
GSE39582_GSM972380	B
GSE39582_GSM972381	A
GSE39582_GSM972382	A
GSE39582_GSM972383	A
GSE39582_GSM972384	A
GSE39582_GSM972385	B
GSE39582_GSM972386	A
GSE39582_GSM972387	A
GSE39582_GSM972388	A
GSE39582_GSM972389	B
GSE39582_GSM972390	A
GSE39582_GSM972391	C
GSE39582_GSM972392	A
GSE39582_GSM972393	A
GSE39582_GSM972394	A
GSE39582_GSM972395	C
GSE39582_GSM972396	A
GSE39582_GSM972397	A
GSE39582_GSM972398	A
GSE39582_GSM972399	A
GSE39582_GSM972400	C
GSE39582_GSM972401	C
GSE39582_GSM972402	B
GSE39582_GSM972403	A
GSE39582_GSM972404	A
GSE39582_GSM972405	A
GSE39582_GSM972406	C
GSE39582_GSM972407	C
GSE39582_GSM972408	A
GSE39582_GSM972409	C
GSE39582_GSM972410	C
GSE39582_GSM972411	A
GSE39582_GSM972412	B
GSE39582_GSM972413	C
GSE39582_GSM972414	A
GSE39582_GSM972415	A
GSE39582_GSM972416	C
GSE39582_GSM972417	A
GSE39582_GSM972418	A
GSE39582_GSM972419	A
GSE39582_GSM972420	A
GSE39582_GSM972421	A
GSE39582_GSM972422	C
GSE39582_GSM972423	A
GSE39582_GSM972424	A
GSE39582_GSM972425	C
GSE39582_GSM972426	A
GSE39582_GSM972427	A

GSE39582_GSM972428	C
GSE39582_GSM972429	B
GSE39582_GSM972430	B
GSE39582_GSM972431	C
GSE39582_GSM972432	A
GSE39582_GSM972433	C
GSE39582_GSM972434	A
GSE39582_GSM972435	A
GSE39582_GSM972436	A
GSE39582_GSM972437	C
GSE39582_GSM972438	C
GSE39582_GSM972439	A
GSE39582_GSM972440	C
GSE39582_GSM972441	C
GSE39582_GSM972442	C
GSE39582_GSM972443	A
GSE39582_GSM972444	C
GSE39582_GSM972445	C
GSE39582_GSM972447	C
GSE39582_GSM972449	C
GSE39582_GSM972450	A
GSE39582_GSM972451	B
GSE39582_GSM972452	B
GSE39582_GSM972453	A
GSE39582_GSM972454	A
GSE39582_GSM972455	C
GSE39582_GSM972456	A
GSE39582_GSM972457	A
GSE39582_GSM972458	C
GSE39582_GSM972459	B
GSE39582_GSM972460	A
GSE39582_GSM972461	A
GSE39582_GSM972462	A
GSE39582_GSM972464	A
GSE39582_GSM972465	A
GSE39582_GSM972466	B
GSE39582_GSM972467	C
GSE39582_GSM972468	A
GSE39582_GSM972469	A
GSE39582_GSM972470	C
GSE39582_GSM972472	A
GSE39582_GSM972473	A
GSE39582_GSM972474	C
GSE39582_GSM972475	B
GSE39582_GSM972476	C
GSE39582_GSM972477	A
GSE39582_GSM972478	A
GSE39582_GSM972479	C
GSE39582_GSM972480	C
GSE39582_GSM972481	C
GSE39582_GSM972482	C
GSE39582_GSM972483	A
GSE39582_GSM972484	A
GSE39582_GSM972485	C
GSE39582_GSM972486	B
GSE39582_GSM972487	C
GSE39582_GSM972488	C
GSE39582_GSM972489	B

GSE39582_GSM972490	A
GSE39582_GSM972491	A
GSE39582_GSM972492	C
GSE39582_GSM972493	A
GSE39582_GSM972494	B
GSE39582_GSM972495	A
GSE39582_GSM972496	A
GSE39582_GSM972497	A
GSE39582_GSM972498	A
GSE39582_GSM972499	A
GSE39582_GSM972501	A
GSE39582_GSM972502	A
GSE39582_GSM972503	C
GSE39582_GSM972504	A
GSE39582_GSM972505	A
GSE39582_GSM972506	A
GSE39582_GSM972507	A
GSE39582_GSM972508	A
GSE39582_GSM972509	A
GSE39582_GSM972510	A
GSE39582_GSM972511	C
GSE39582_GSM972512	B
GSE39582_GSM972513	A
GSE39582_GSM972515	A
GSE39582_GSM972516	B
GSE39582_GSM972517	C
GSE39582_GSM972518	A
GSE39582_GSM972519	A
GSE39582_GSM972520	C
GSE39582_GSM972521	C
GSE39582_GSM972522	C
GSE38832_GSM950417	A
GSE38832_GSM950418	A
GSE38832_GSM950419	A
GSE38832_GSM950420	B
GSE38832_GSM950421	B
GSE38832_GSM950422	C
GSE38832_GSM950423	B
GSE38832_GSM950424	C
GSE38832_GSM950425	C
GSE38832_GSM950426	A
GSE38832_GSM950428	B
GSE38832_GSM950429	C
GSE38832_GSM950430	B
GSE38832_GSM950431	A
GSE38832_GSM950432	B
GSE38832_GSM950433	C
GSE38832_GSM950434	A
GSE38832_GSM950435	A
GSE38832_GSM950436	A
GSE38832_GSM950437	A
GSE38832_GSM950438	A
GSE38832_GSM950439	B
GSE38832_GSM950440	A
GSE38832_GSM950441	A
GSE38832_GSM950442	C
GSE38832_GSM950443	A
GSE38832_GSM950444	C

GSE38832_GSM950445	A
GSE38832_GSM950446	C
GSE38832_GSM950447	C
GSE38832_GSM950448	A
GSE38832_GSM950449	C
GSE38832_GSM950450	C
GSE38832_GSM950452	C
GSE38832_GSM950453	C
GSE38832_GSM950454	C
GSE38832_GSM950455	C
GSE38832_GSM950457	C
GSE38832_GSM950459	C
GSE38832_GSM950460	A
GSE38832_GSM950461	C
GSE38832_GSM950462	C
GSE38832_GSM950463	A
GSE38832_GSM950465	C
GSE38832_GSM950467	B
GSE38832_GSM950468	C
GSE38832_GSM950469	B
GSE38832_GSM950470	C
GSE38832_GSM950471	A
GSE38832_GSM950472	C
GSE38832_GSM950474	A
GSE38832_GSM950475	C
GSE38832_GSM950476	C
GSE38832_GSM950477	C
GSE38832_GSM950478	C
GSE38832_GSM950479	C
GSE38832_GSM950480	C
GSE38832_GSM950481	A
GSE38832_GSM950483	C
GSE38832_GSM950484	C
GSE38832_GSM950485	C
GSE38832_GSM950486	C
GSE38832_GSM950487	C
GSE38832_GSM950489	C
GSE38832_GSM950490	C
GSE38832_GSM950492	C
GSE38832_GSM950495	C
GSE38832_GSM950496	C
GSE38832_GSM950497	A
GSE38832_GSM950499	C
GSE38832_GSM950500	C
GSE38832_GSM950503	C
GSE38832_GSM950504	C
GSE38832_GSM950505	B
GSE38832_GSM950506	A
GSE38832_GSM950507	B
GSE38832_GSM950509	B
GSE38832_GSM950512	A
GSE38832_GSM950513	A
GSE38832_GSM950514	A
GSE38832_GSM950516	A
GSE38832_GSM950517	A
GSE38832_GSM950519	A
GSE38832_GSM950520	A
GSE38832_GSM950521	C

GSE38832_GSM950522	A
GSE38832_GSM950523	B
GSE38832_GSM950525	A
GSE38832_GSM950528	A
GSE38832_GSM950529	A
GSE38832_GSM950531	C
GSE38832_GSM950532	C

Table S5. The differential states of biological pathways in distinct cuproptosis subtypes by GSVA enrichment analysis.

Type	ID	logFC	AveExpr	t	P.Value	adj.P.Val	B
B-A	KEGG_SELENOAMINO	-0.168068	0.050225	-10.51776	1.43E-24	2.63E-22	44.96833
B-A	KEGG_SYSTEMIC_LUP	0.221573	-0.105013	10.09056	7.82E-23	7.19E-21	41.04581
B-A	KEGG_GLYCOSAMINC	0.204739	-0.111718	9.961353	2.56E-22	1.57E-20	39.88521
B-A	KEGG_PEROXISOME	-0.151031	0.065655	-9.412663	3.42E-20	1.57E-18	35.09327
B-A	KEGG_ECM_RECEPTO	0.183982	-0.113516	9.237774	1.55E-19	5.59E-18	33.61314
B-A	KEGG_CELL_ADHESIC	0.168475	-0.086769	9.219139	1.82E-19	5.59E-18	33.4568
B-A	KEGG_LEUKOCYTE_T	0.123841	-0.063096	8.704315	1.36E-17	3.57E-16	29.24292
B-A	KEGG_FOCAL_ADHES	0.129142	-0.077091	8.637803	2.34E-17	5.37E-16	28.71352
B-A	KEGG_NATURAL_KILI	0.128888	-0.061607	8.593389	3.35E-17	6.84E-16	28.36194
B-A	KEGG_CHEMOKINE_SI	0.120453	-0.0674	8.380629	1.84E-16	3.38E-15	26.69936
B-A	KEGG_VIRAL_MYOCA	0.151012	-0.080129	8.361519	2.14E-16	3.58E-15	26.55178
B-A	KEGG_HEMATOPOIET	0.167604	-0.097083	8.128	1.33E-15	2.03E-14	24.77205
B-A	KEGG_CYTOKINE_CY	0.127649	-0.075753	8.026954	2.88E-15	3.79E-14	24.01554
B-A	KEGG_LEISHMANIA_II	0.162852	-0.100146	7.927322	6.14E-15	7.54E-14	23.27772
B-A	KEGG_GRAFT_VERSU	0.222319	-0.107369	7.775847	1.91E-14	2.07E-13	22.17149
B-A	KEGG_GLYCOSYLPHO	-0.139238	0.05023	-7.467559	1.82E-13	1.76E-12	19.97831
B-A	KEGG_TYPE_I_DIABET	0.178933	-0.079221	7.41898	2.58E-13	2.37E-12	19.63991
B-A	KEGG_AMINOACYL_T	-0.171541	0.102165	-7.258843	8.01E-13	6.70E-12	18.53835
B-A	KEGG_ANTIGEN_PROC	0.149995	-0.063214	7.145006	1.77E-12	1.42E-11	17.76837
B-A	KEGG_PENTOSE_PHOS	-0.135606	0.053823	-7.047874	3.45E-12	2.64E-11	17.12003
B-A	KEGG_PRION_DISEASI	0.110596	-0.042782	6.952303	6.60E-12	4.64E-11	16.48993
B-A	KEGG_ALLOGRAFT_R	0.197448	-0.103106	6.947894	6.80E-12	4.64E-11	16.46105
B-A	KEGG_COMPLEMENT_	0.120516	-0.070883	6.921825	8.11E-12	5.33E-11	16.29062
B-A	KEGG_INTESTINAL_IN	0.172512	-0.098211	6.879984	1.07E-11	6.81E-11	16.0183
B-A	KEGG_TOLL_LIKE_RE	0.103233	-0.061847	6.767092	2.27E-11	1.30E-10	15.29101
B-A	KEGG_MATURITY_ON	-0.119044	0.067121	-6.763281	2.33E-11	1.30E-10	15.26664
B-A	KEGG_AUTOIMMUNE_	0.160184	-0.065324	6.690393	3.76E-11	1.98E-10	14.80312
B-A	KEGG_PYRUVATE_ME	-0.107764	0.068349	-6.323536	3.90E-10	1.75E-09	12.53988
B-A	KEGG_ASTHMA	0.161171	-0.099543	6.237122	6.65E-10	2.91E-09	12.02382
B-A	KEGG_PRIMARY_IMM	0.126165	-0.062714	5.543928	3.81E-08	1.43E-07	8.122924
B-A	KEGG_CITRATE_CYCI	-0.126623	0.085965	-5.522289	4.30E-08	1.58E-07	8.008055
B-A	KEGG_GLYCOSPHING	0.103822	-0.079357	5.321762	1.28E-07	4.61E-07	6.963658
B-A	KEGG_RIBOSOME	-0.141158	0.084123	-5.280983	1.59E-07	5.62E-07	6.755722
C-A	KEGG_ECM_RECEPTO	0.323543	-0.014355	22.09954	2.57E-92	4.72E-90	199.6861
C-A	KEGG_CELL_ADHESIC	0.270925	-0.0072	21.23299	2.72E-86	2.50E-84	185.8786
C-A	KEGG_CYTOKINE_CY	0.237348	-0.00125	21.16381	8.14E-86	4.99E-84	184.7877
C-A	KEGG_HEMATOPOIET	0.302964	-0.003106	21.14226	1.14E-85	5.26E-84	184.4482
C-A	KEGG_SYSTEMIC_LUP	0.325178	-0.014164	20.80873	2.20E-83	8.08E-82	179.2164
C-A	KEGG_GLYCOSAMINC	0.310599	-0.023283	20.17232	4.43E-79	1.36E-77	169.3516
C-A	KEGG_LEISHMANIA_II	0.295808	-0.008197	20.14484	6.78E-79	1.78E-77	168.9293
C-A	KEGG_CHEMOKINE_SI	0.20312	-0.006336	19.5298	8.35E-75	1.92E-73	159.557
C-A	KEGG_FOCAL_ADHES	0.217684	-0.011662	19.08562	6.81E-72	1.39E-70	152.8879
C-A	KEGG_VIRAL_MYOCA	0.244092	-0.008253	18.69782	2.21E-69	4.06E-68	147.1365
C-A	KEGG_LEUKOCYTE_T	0.191743	-0.007889	18.52231	2.95E-68	4.94E-67	144.5561
C-A	KEGG_COMPLEMENT_	0.223874	-0.000637	17.9683	9.67E-65	1.48E-63	136.5053
C-A	KEGG_TOLL_LIKE_RE	0.192388	-0.001401	17.46964	1.24E-61	1.76E-60	129.3859
C-A	KEGG_PEROXISOME	-0.205491	0.010891	-16.99156	1.06E-58	1.39E-57	122.6782
C-A	KEGG_ASTHMA	0.309235	-0.00124	16.97958	1.25E-58	1.53E-57	122.5116
C-A	KEGG_JAK_STAT_SIGI	0.156124	0.000402	16.90921	3.33E-58	3.84E-57	121.5347
C-A	KEGG_INTESTINAL_IN	0.297687	-0.007751	16.87827	5.13E-58	5.55E-57	121.106
C-A	KEGG_PATHWAYS_IN_	0.119884	-0.006298	16.84224	8.47E-58	8.66E-57	120.6074
C-A	KEGG_PYRUVATE_ME	-0.206938	0.002543	-16.56987	3.67E-56	3.55E-55	116.8607
C-A	KEGG_GLYCOSPHING	0.23743	0.000909	16.47987	1.26E-55	1.16E-54	115.6314
C-A	KEGG_NATURAL_KILI	0.182128	-0.011874	16.26269	2.45E-54	2.15E-53	112.6833
C-A	KEGG_AMINOACYL_T	-0.285566	0.016844	-16.11573	1.80E-53	1.50E-52	110.7032

C-A	KEGG_NOD_LIKE_REC	0.184254	0.004028	15.79071	1.41E-51	1.13E-50	106.3669
C-A	KEGG_HUNTINGTONS_	-0.180763	-0.005504	-15.43014	1.66E-49	1.28E-48	101.6268
C-A	KEGG_GRAFT_VERSU	0.317238	-0.020218	15.40954	2.18E-49	1.60E-48	101.3583
C-A	KEGG_REGULATION_(	0.129437	-0.008799	15.40066	2.45E-49	1.73E-48	101.2426
C-A	KEGG_ALLOGRAFT_R	0.305955	-0.014976	14.94389	9.16E-47	6.25E-46	95.35562
C-A	KEGG_B_CELL_RECEP	0.156296	-0.007759	14.90725	1.47E-46	9.64E-46	94.88876
C-A	KEGG_CITRATE_CYCL	-0.250874	0.005222	-14.86948	2.38E-46	1.51E-45	94.40838
C-A	KEGG_HYPERTROPHIC	0.160859	-0.00441	14.7471	1.13E-45	6.95E-45	92.85803
C-A	KEGG_SELENOAMINO_	-0.17628	0.012502	-14.36055	1.48E-43	8.76E-43	88.0214
C-A	KEGG_TYPE_I_DIABET	0.248323	-0.012182	14.27747	4.15E-43	2.39E-42	86.99407
C-A	KEGG_T_CELL_RECEP	0.141568	-0.007295	14.23241	7.26E-43	4.05E-42	86.43875
C-A	KEGG_MELANOMA	0.12086	-0.005209	14.19802	1.11E-42	6.01E-42	86.01582
C-A	KEGG_MATURITY_ON	-0.184067	0.014162	-14.14851	2.05E-42	1.08E-41	85.40808
C-A	KEGG_RIBOSOME	-0.275675	-0.004118	-13.99746	1.31E-41	6.71E-41	83.56396
C-A	KEGG_BUTANOATE_M	-0.200378	0.004138	-13.73747	3.10E-40	1.50E-39	80.42418
C-A	KEGG_DILATED_CARI	0.154479	-0.006977	13.55699	2.72E-39	1.28E-38	78.27057
C-A	KEGG_RENAL_CELL_(	0.139295	-0.008294	13.50353	5.14E-39	2.37E-38	77.63672
C-A	KEGG_RNA_POLYMER	-0.21083	-0.002913	-13.47449	7.27E-39	3.26E-38	77.29327
C-A	KEGG_SMALL_CELL_I	0.121075	-0.006735	13.36932	2.53E-38	1.11E-37	76.0539
C-A	KEGG_TGF_BETA_SIG	0.133744	-0.001538	13.32599	4.23E-38	1.81E-37	75.54545
C-A	KEGG_PENTOSE_PHOS	-0.18845	0.002903	-13.31403	4.87E-38	2.04E-37	75.40541
C-A	KEGG_AUTOIMMUNE_	0.223519	-0.004771	13.0822	7.38E-37	3.02E-36	72.70832
C-A	KEGG_BASE_EXCISIOI	-0.20181	-0.000854	-12.99257	2.09E-36	8.36E-36	71.67534
C-A	KEGG_FC_GAMMA_R_	0.124819	-0.011072	12.9441	3.66E-36	1.43E-35	71.11909
C-A	KEGG_GLYOXYLATE_	-0.204568	-0.008146	-12.69804	6.15E-35	2.36E-34	68.31985
C-A	KEGG_LYSINE_DEGRA	-0.149647	-0.004773	-12.54126	3.64E-34	1.37E-33	66.55812
C-A	KEGG_PROPANOATE_	-0.173839	0.004365	-12.15484	2.69E-32	9.90E-32	62.29006
C-A	KEGG_OXIDATIVE_PH	-0.185538	-0.005756	-12.00725	1.35E-31	4.89E-31	60.68792
C-A	KEGG_ANTIGEN_PROC	0.184491	-0.017508	12.00193	1.44E-31	5.08E-31	60.63051
C-A	KEGG_PRION_DISEASI	0.139447	-0.007567	11.82637	9.61E-31	3.34E-30	58.74591
C-A	KEGG_SPLICEOSOME	-0.174973	-0.002942	-11.74516	2.30E-30	7.83E-30	57.88178
C-A	KEGG_GLYCOSYLPHO	-0.163263	0.011345	-11.6336	7.56E-30	2.53E-29	56.70249
C-A	KEGG_PRIMARY_IMM	0.191872	-0.008009	11.49314	3.34E-29	1.10E-28	55.23081
C-A	KEGG_FC_EPSILON_RI	0.100163	-0.006301	11.36439	1.29E-28	4.15E-28	53.89463
C-A	KEGG_ALZHEIMERS_I	-0.127017	-0.005432	-11.10239	1.93E-27	6.11E-27	51.21391
C-A	KEGG_ARRHYTHMOG	0.129774	-0.006241	11.0997	1.98E-27	6.18E-27	51.18673
C-A	KEGG_HISTIDINE_ME1	-0.123334	-3.23E-05	-11.01471	4.71E-27	1.44E-26	50.32842
C-A	KEGG_PARKINSONS_I	-0.172416	-0.005789	-10.82532	3.19E-26	9.62E-26	48.43553
C-A	KEGG_PANCREATIC_C	0.103862	-0.007494	10.70055	1.11E-25	3.29E-25	47.20355
C-A	KEGG_PYRIMIDINE_M	-0.131827	-0.00291	-10.68268	1.32E-25	3.86E-25	47.02803
C-A	KEGG_DRUG_METABC	-0.118553	0.003407	-10.60081	2.97E-25	8.54E-25	46.22724
C-A	KEGG_TERPENOID_BA	-0.191687	-0.007519	-10.00862	8.87E-23	2.44E-22	40.59104
C-A	KEGG_ARGININE_ANI	-0.108562	-0.000846	-9.857102	3.65E-22	9.87E-22	39.1935
C-A	KEGG_N_GLYCAN_BIC	-0.11775	-0.000875	-9.667574	2.08E-21	5.48E-21	37.47131
C-A	KEGG_CARDIAC_MUS	-0.100596	-0.001896	-9.603515	3.73E-21	9.68E-21	36.89577
C-A	KEGG_RNA_DEGRADA	-0.135732	0.004162	-9.36473	3.18E-20	8.13E-20	34.77979
C-A	KEGG_GLYCOSPHING(	0.134311	0.002481	9.328242	4.40E-20	1.11E-19	34.46056
C-A	KEGG_ALANINE_ASPA	-0.104931	0.004185	-9.286347	6.37E-20	1.58E-19	34.09537
C-A	KEGG_GLYCOSAMINC	0.130055	-0.006817	9.276774	6.93E-20	1.70E-19	34.01212
C-A	KEGG_FATTY_ACID_M	-0.135795	0.000997	-8.990337	8.35E-19	2.02E-18	31.5563
C-A	KEGG_DNA_REPLICAT	-0.184322	0.003072	-8.962161	1.06E-18	2.54E-18	31.3184
C-A	KEGG_GLUTATHIONE_	-0.107313	-0.002797	-8.936481	1.32E-18	3.12E-18	31.10215
C-A	KEGG_PROXIMAL_TUI	-0.109307	0.004299	-8.634975	1.67E-17	3.79E-17	28.60444
C-A	KEGG_MISMATCH_RE	-0.154714	-0.00088	-8.482798	5.82E-17	1.29E-16	27.37285
C-A	KEGG_GLYCOSAMINC	0.13297	-0.011084	8.465104	6.73E-17	1.47E-16	27.23092
C-A	KEGG_GLYCOLYSIS_C	-0.10142	-0.005343	-8.295044	2.65E-16	5.67E-16	25.8804
C-A	KEGG_OTHER_GLYCA	-0.127833	0.003525	-8.120257	1.06E-15	2.21E-15	24.51809
C-A	KEGG_VALINE_LEUCI	-0.128716	-0.002537	-8.083417	1.41E-15	2.92E-15	24.2343

C-A	KEGG_NON_HOMOLO(	-0.137898	0.004513	-8.000123	2.70E-15	5.46E-15	23.59697
C-A	KEGG_STEROID_BIOS'	-0.148367	0.000856	-7.924226	4.85E-15	9.70E-15	23.02145
C-A	KEGG_HOMOLOGOUS_	-0.128897	0.00085	-7.705942	2.54E-14	5.03E-14	21.3941
C-A	KEGG_NUCLEOTIDE_E	-0.114719	-0.00136	-7.564732	7.26E-14	1.41E-13	20.36347
C-A	KEGG_ONE_CARBON_	-0.116404	-0.001309	-6.921954	6.94E-12	1.25E-11	15.89429
C-A	KEGG_CIRCADIAN_RH	0.107443	-0.013479	6.669964	3.75E-11	6.34E-11	14.24276
C-A	KEGG_ASCORBATE_A	-0.10997	0.004436	-6.028087	2.15E-09	3.38E-09	10.29578
C-A	KEGG_PROTEASOME	-0.102713	-0.013447	-5.334925	1.12E-07	1.63E-07	6.458881
C-B	KEGG_NOD_LIKE_REC	0.131071	0.069339	7.561875	1.07E-13	1.96E-11	20.54247
C-B	KEGG_GLYOXYLATE_	-0.162528	-0.075819	-6.738564	3.02E-11	2.77E-09	15.12029
C-B	KEGG_CYTOKINE_CY	0.109699	0.099704	6.402318	2.58E-10	1.55E-08	13.06589
C-B	KEGG_GLYCOSPHING(	0.133609	0.095107	6.358767	3.38E-10	1.55E-08	12.80672
C-B	KEGG_HEMATOPOIET	0.135361	0.127085	6.323409	4.20E-10	1.55E-08	12.59749
C-B	KEGG_ECM_RECEPTO	0.139562	0.1261	6.071973	1.93E-09	5.92E-08	11.14017
C-B	KEGG_LYSINE_DEGRA	-0.104419	-0.058395	-6.007877	2.83E-09	7.43E-08	10.77729
C-B	KEGG_LEISHMANIA_II	0.132955	0.118693	5.970436	3.52E-09	8.10E-08	10.56694
C-B	KEGG_ASTHMA	0.148063	0.128829	5.654847	2.15E-08	4.29E-07	8.841934
C-B	KEGG_COMPLEMENT_	0.103358	0.094619	5.53506	4.19E-08	6.42E-07	8.209804
C-B	KEGG_RNA_POLYMER	-0.125505	-0.084605	-5.416268	8.00E-08	1.13E-06	7.595318
C-B	KEGG_CELL_ADHESIC	0.10245	0.114513	5.250686	1.93E-07	2.37E-06	6.759491
C-B	KEGG_BUTANOATE_M	-0.112425	-0.075455	-5.180073	2.79E-07	2.86E-06	6.410414
C-B	KEGG_BASE_EXCISIOI	-0.11939	-0.079262	-5.116537	3.88E-07	3.76E-06	6.100103
C-B	KEGG_CITRATE_CYCL	-0.124251	-0.099124	-4.877371	1.29E-06	1.10E-05	4.964264
C-B	KEGG_INTESTINAL_IM	0.125175	0.122399	4.874089	1.31E-06	1.10E-05	4.949034
C-B	KEGG_TERPENOID_BA	-0.13048	-0.077136	-4.749247	2.41E-06	1.76E-05	4.376856
C-B	KEGG_SPLICEOSOME	-0.102874	-0.071106	-4.60826	4.71E-06	3.09E-05	3.747567
C-B	KEGG_GLYCOSAMINC	0.10586	0.119552	4.538421	6.52E-06	4.13E-05	3.442504
C-B	KEGG_RIBOSOME	-0.134517	-0.119353	-4.450906	9.73E-06	5.97E-05	3.066484
C-B	KEGG_SYSTEMIC_LUP	0.103605	0.13743	4.431426	1.06E-05	6.31E-05	2.983735
C-B	KEGG_AMINOACYL_T	-0.114025	-0.109728	-4.20393	2.91E-05	0.000153	2.042976
C-B	KEGG_MISMATCH_RE	-0.107874	-0.05634	-4.100869	4.53E-05	0.000225	1.632402
C-B	KEGG_DNA_REPLICAT	-0.116739	-0.066353	-3.910137	9.99E-05	0.000448	0.89837
C-B	KEGG_ALLOGRAFT_R	0.108507	0.124519	3.628282	0.000303	0.001115	-0.124643

Table S6. Relative fractions of tumor-infiltrating immune cells of CRC patients by CIBERSORT algorithm.

Mixture	C1	C2	C3	C4	C5	C6	C7	C8	C9	C10	C11	C12	C13	C14	C15	C16	C17	C18	C19	C20	C21	C22
TCGA-DM-A288	0.00	0.00	0.09	0.00	0.00	0.00	0.00	0.00	0.08	0.00	0.02	0.00	0.00	0.46	0.00	0.09	0.00	0.01	0.00	0.21	0.02	0.01
TCGA-QL-A97D	0.05	0.00	0.06	0.17	0.00	0.15	0.08	0.08	0.00	0.02	0.06	0.00	0.00	0.00	0.12	0.07	0.04	0.01	0.00	0.09	0.00	0.00
TCGA-CM-6164	0.01	0.04	0.02	0.19	0.00	0.00	0.07	0.05	0.01	0.00	0.04	0.00	0.00	0.20	0.15	0.08	0.01	0.00	0.00	0.10	0.00	0.02
TCGA-G4-6299	0.01	0.00	0.07	0.21	0.00	0.01	0.05	0.03	0.01	0.06	0.00	0.03	0.00	0.16	0.12	0.21	0.04	0.00	0.00	0.00	0.00	0.00
TCGA-AZ-4615	0.02	0.00	0.01	0.25	0.00	0.03	0.12	0.06	0.00	0.03	0.00	0.04	0.00	0.06	0.15	0.11	0.06	0.01	0.04	0.00	0.00	0.00
TCGA-AA-3549	0.03	0.00	0.11	0.07	0.00	0.11	0.05	0.02	0.02	0.00	0.07	0.00	0.00	0.16	0.05	0.03	0.00	0.03	0.00	0.21	0.00	0.05
TCGA-CM-4752	0.07	0.00	0.06	0.07	0.00	0.18	0.10	0.06	0.01	0.00	0.00	0.02	0.00	0.02	0.15	0.09	0.13	0.00	0.04	0.00	0.00	0.02
TCGA-DM-A1D9	0.00	0.07	0.02	0.05	0.00	0.27	0.07	0.02	0.00	0.00	0.07	0.00	0.00	0.08	0.06	0.11	0.07	0.01	0.00	0.09	0.00	0.00
TCGA-AA-3688	0.03	0.00	0.08	0.11	0.00	0.08	0.15	0.00	0.00	0.00	0.05	0.00	0.00	0.09	0.08	0.17	0.04	0.00	0.00	0.10	0.04	0.00
TCGA-AA-3854	0.03	0.00	0.02	0.07	0.00	0.24	0.00	0.00	0.04	0.00	0.04	0.00	0.01	0.05	0.04	0.20	0.03	0.04	0.00	0.16	0.01	0.01
TCGA-A6-3809	0.00	0.02	0.02	0.08	0.00	0.17	0.06	0.06	0.00	0.02	0.01	0.01	0.00	0.10	0.10	0.09	0.01	0.03	0.00	0.14	0.02	0.07
TCGA-CM-6165	0.02	0.01	0.02	0.07	0.00	0.09	0.05	0.02	0.00	0.02	0.00	0.01	0.00	0.31	0.11	0.16	0.05	0.00	0.05	0.01	0.00	0.02
TCGA-CM-4751	0.03	0.01	0.07	0.18	0.00	0.05	0.09	0.08	0.01	0.01	0.02	0.00	0.00	0.04	0.12	0.12	0.06	0.04	0.00	0.05	0.00	0.02
TCGA-A6-5659	0.09	0.00	0.03	0.00	0.00	0.18	0.03	0.05	0.00	0.00	0.01	0.00	0.03	0.23	0.07	0.11	0.02	0.02	0.00	0.11	0.02	0.00
TCGA-CM-4750	0.11	0.00	0.09	0.27	0.00	0.00	0.06	0.05	0.01	0.00	0.00	0.00	0.02	0.09	0.08	0.09	0.06	0.02	0.00	0.05	0.00	0.00
TCGA-AZ-4682	0.00	0.01	0.11	0.09	0.00	0.25	0.06	0.00	0.00	0.00	0.05	0.00	0.00	0.05	0.05	0.13	0.05	0.00	0.00	0.15	0.00	0.02
TCGA-G4-6625	0.02	0.03	0.04	0.14	0.00	0.03	0.13	0.07	0.00	0.00	0.04	0.00	0.00	0.15	0.07	0.08	0.08	0.04	0.00	0.06	0.02	0.00
TCGA-DM-A0XI	0.00	0.00	0.09	0.09	0.00	0.07	0.08	0.06	0.02	0.00	0.00	0.04	0.01	0.15	0.17	0.11	0.04	0.00	0.06	0.00	0.00	0.00
TCGA-AA-3949	0.08	0.00	0.01	0.11	0.00	0.04	0.11	0.04	0.00	0.07	0.00	0.00	0.00	0.10	0.12	0.13	0.05	0.01	0.00	0.05	0.02	0.06
TCGA-AA-3848	0.04	0.00	0.11	0.16	0.00	0.02	0.12	0.01	0.01	0.00	0.05	0.00	0.00	0.15	0.07	0.12	0.03	0.00	0.00	0.08	0.02	0.00
TCGA-CA-6715	0.00	0.01	0.05	0.10	0.00	0.03	0.06	0.00	0.03	0.00	0.09	0.00	0.00	0.39	0.01	0.09	0.00	0.01	0.00	0.14	0.00	0.00
TCGA-AA-3710	0.02	0.00	0.01	0.16	0.00	0.00	0.11	0.08	0.00	0.06	0.00	0.01	0.00	0.03	0.15	0.11	0.07	0.02	0.00	0.05	0.01	0.11
TCGA-AA-3950	0.00	0.00	0.01	0.10	0.00	0.16	0.05	0.01	0.00	0.02	0.00	0.02	0.00	0.13	0.10	0.16	0.06	0.00	0.00	0.08	0.00	0.09
TCGA-AA-A00N	0.03	0.00	0.01	0.04	0.00	0.18	0.00	0.00	0.00	0.00	0.01	0.00	0.00	0.27	0.05	0.18	0.01	0.02	0.00	0.15	0.00	0.05
TCGA-A6-6138	0.03	0.00	0.05	0.13	0.00	0.07	0.04	0.06	0.00	0.02	0.00	0.02	0.00	0.18	0.12	0.14	0.05	0.04	0.00	0.04	0.00	0.00
TCGA-DM-A28E	0.00	0.01	0.09	0.03	0.00	0.35	0.14	0.05	0.00	0.00	0.03	0.00	0.00	0.03	0.09	0.06	0.03	0.03	0.04	0.03	0.00	0.00
TCGA-D5-6538	0.05	0.00	0.00	0.03	0.00	0.15	0.10	0.00	0.00	0.00	0.14	0.00	0.00	0.19	0.00	0.15	0.05	0.04	0.00	0.07	0.00	0.02
TCGA-AA-A00K	0.02	0.02	0.05	0.06	0.00	0.13	0.18	0.01	0.03	0.00	0.07	0.00	0.00	0.21	0.01	0.06	0.02	0.03	0.00	0.10	0.00	0.00
TCGA-AA-3715	0.04	0.00	0.02	0.15	0.00	0.00	0.08	0.04	0.03	0.00	0.06	0.00	0.01	0.13	0.11	0.08	0.07	0.00	0.00	0.14	0.00	0.04
TCGA-AA-3506	0.07	0.00	0.10	0.17	0.00	0.05	0.00	0.03	0.02	0.00	0.05	0.00	0.00	0.16	0.06	0.12	0.03	0.00	0.00	0.10	0.02	0.00
TCGA-CK-5916	0.03	0.00	0.04	0.11	0.00	0.04	0.05	0.08	0.00	0.09	0.00	0.04	0.00	0.04	0.17	0.09	0.13	0.00	0.01	0.02	0.00	0.05
TCGA-QG-A5Z1	0.00	0.02	0.03	0.01	0.00	0.10	0.00	0.00	0.02	0.00	0.01	0.00	0.00	0.49	0.03	0.16	0.08	0.00	0.02	0.01	0.00	0.00
TCGA-AD-6890	0.03	0.00	0.10	0.14	0.00	0.08	0.17	0.02	0.00	0.00	0.04	0.00	0.00	0.15	0.09	0.11	0.03	0.00	0.00	0.02	0.00	0.00
TCGA-A6-2685	0.03	0.00	0.07	0.09	0.00	0.04	0.03	0.05	0.02	0.00	0.00	0.00	0.00	0.18	0.05	0.17	0.10	0.00	0.00	0.06	0.07	0.04
TCGA-AA-A00W	0.00	0.06	0.09	0.09	0.00	0.03	0.15	0.09	0.00	0.02	0.09	0.00	0.00	0.12	0.00	0.00	0.00	0.08	0.00	0.16	0.00	0.00
TCGA-CK-5914	0.02	0.00	0.00	0.14	0.00	0.00	0.16	0.04	0.00	0.00	0.00	0.07	0.00	0.11	0.12	0.13	0.01	0.01	0.00	0.17	0.01	0.00

TCGA-A6-2686	0.02	0.00	0.06	0.15	0.00	0.02	0.09	0.04	0.00	0.06	0.02	0.00	0.00	0.08	0.09	0.15	0.08	0.03	0.00	0.05	0.04	0.03
TCGA-CA-5255	0.00	0.02	0.10	0.10	0.00	0.15	0.00	0.03	0.06	0.04	0.00	0.00	0.00	0.04	0.11	0.08	0.08	0.06	0.00	0.14	0.00	0.00
TCGA-A6-A5ZU	0.07	0.00	0.04	0.10	0.00	0.01	0.08	0.03	0.00	0.00	0.02	0.02	0.00	0.27	0.14	0.15	0.02	0.00	0.05	0.00	0.00	0.01
TCGA-NH-A50V	0.05	0.00	0.05	0.05	0.00	0.15	0.00	0.11	0.06	0.00	0.06	0.00	0.00	0.03	0.09	0.11	0.01	0.03	0.00	0.16	0.00	0.03
TCGA-QG-A5YX	0.00	0.06	0.06	0.13	0.00	0.17	0.00	0.03	0.04	0.00	0.01	0.00	0.00	0.16	0.03	0.10	0.08	0.03	0.00	0.10	0.00	0.00
TCGA-A6-2677	0.00	0.03	0.05	0.00	0.00	0.32	0.07	0.00	0.00	0.04	0.03	0.00	0.00	0.12	0.06	0.11	0.07	0.00	0.03	0.02	0.04	0.00
TCGA-CA-6717	0.08	0.00	0.03	0.13	0.00	0.09	0.07	0.03	0.00	0.03	0.02	0.00	0.00	0.11	0.11	0.14	0.05	0.00	0.00	0.10	0.00	0.01
TCGA-NH-A6GC	0.00	0.01	0.18	0.11	0.00	0.11	0.07	0.02	0.02	0.00	0.00	0.06	0.00	0.19	0.01	0.17	0.03	0.00	0.03	0.00	0.00	0.00
TCGA-AA-3562	0.04	0.00	0.15	0.06	0.00	0.14	0.06	0.03	0.00	0.00	0.00	0.01	0.00	0.11	0.06	0.15	0.09	0.00	0.03	0.02	0.03	0.02
TCGA-A6-4105	0.04	0.00	0.02	0.17	0.00	0.01	0.13	0.07	0.00	0.00	0.00	0.00	0.00	0.10	0.11	0.16	0.02	0.04	0.00	0.09	0.00	0.04
TCGA-A6-6141	0.07	0.03	0.03	0.17	0.00	0.12	0.01	0.09	0.03	0.06	0.00	0.00	0.01	0.09	0.13	0.04	0.09	0.00	0.00	0.03	0.00	0.00
TCGA-AZ-4684	0.03	0.00	0.09	0.11	0.00	0.10	0.03	0.06	0.00	0.00	0.05	0.00	0.00	0.15	0.05	0.07	0.06	0.01	0.00	0.14	0.00	0.04
TCGA-CK-6747	0.08	0.00	0.09	0.14	0.00	0.22	0.06	0.00	0.03	0.00	0.00	0.00	0.01	0.09	0.11	0.05	0.10	0.00	0.00	0.00	0.01	0.00
TCGA-AU-6004	0.08	0.00	0.02	0.08	0.00	0.07	0.07	0.00	0.01	0.02	0.04	0.00	0.00	0.18	0.12	0.16	0.04	0.01	0.00	0.07	0.00	0.03
TCGA-A6-2672	0.02	0.00	0.05	0.16	0.00	0.04	0.05	0.07	0.01	0.00	0.01	0.02	0.00	0.17	0.14	0.09	0.04	0.02	0.00	0.06	0.00	0.05
TCGA-QG-A5Z2	0.14	0.00	0.12	0.22	0.00	0.06	0.07	0.07	0.01	0.01	0.03	0.00	0.00	0.06	0.07	0.07	0.02	0.02	0.00	0.02	0.00	0.00
TCGA-A6-6780	0.00	0.05	0.02	0.06	0.00	0.07	0.07	0.05	0.00	0.04	0.00	0.00	0.00	0.10	0.15	0.18	0.01	0.00	0.00	0.13	0.00	0.06
TCGA-F4-6459	0.02	0.02	0.00	0.19	0.00	0.02	0.12	0.02	0.01	0.00	0.00	0.00	0.01	0.20	0.08	0.14	0.06	0.00	0.00	0.06	0.04	0.00
TCGA-AA-3970	0.06	0.00	0.06	0.02	0.00	0.20	0.09	0.05	0.00	0.02	0.01	0.00	0.00	0.09	0.07	0.16	0.03	0.02	0.00	0.07	0.04	0.00
TCGA-NH-A8F7	0.00	0.06	0.05	0.00	0.14	0.00	0.00	0.00	0.00	0.00	0.07	0.00	0.00	0.24	0.00	0.19	0.00	0.00	0.00	0.17	0.03	0.06
TCGA-G4-6321	0.14	0.00	0.02	0.24	0.00	0.13	0.07	0.04	0.00	0.00	0.07	0.00	0.00	0.05	0.03	0.08	0.04	0.03	0.02	0.05	0.00	0.00
TCGA-AA-3524	0.08	0.00	0.02	0.18	0.00	0.04	0.18	0.03	0.00	0.00	0.00	0.01	0.00	0.02	0.08	0.16	0.04	0.00	0.00	0.06	0.08	0.01
TCGA-DM-A1D0	0.00	0.10	0.01	0.00	0.00	0.05	0.00	0.02	0.03	0.06	0.00	0.00	0.00	0.53	0.00	0.14	0.00	0.00	0.00	0.05	0.00	0.00
TCGA-G4-6307	0.18	0.00	0.09	0.10	0.00	0.09	0.10	0.07	0.00	0.00	0.00	0.00	0.00	0.18	0.04	0.00	0.11	0.00	0.00	0.03	0.00	0.00
TCGA-DM-A1HB	0.03	0.00	0.03	0.03	0.00	0.19	0.00	0.03	0.06	0.00	0.03	0.00	0.00	0.16	0.01	0.06	0.01	0.02	0.00	0.31	0.00	0.03
TCGA-AA-3697	0.02	0.05	0.06	0.04	0.00	0.09	0.08	0.05	0.00	0.06	0.03	0.00	0.00	0.12	0.06	0.11	0.03	0.04	0.00	0.14	0.00	0.00
TCGA-CM-6679	0.02	0.01	0.11	0.08	0.00	0.13	0.04	0.02	0.02	0.00	0.02	0.00	0.00	0.20	0.09	0.11	0.07	0.00	0.00	0.07	0.00	0.02
TCGA-AY-6197	0.02	0.00	0.01	0.08	0.00	0.19	0.06	0.01	0.04	0.00	0.04	0.00	0.00	0.19	0.04	0.08	0.00	0.02	0.00	0.20	0.01	0.00
TCGA-A6-2684	0.00	0.00	0.01	0.07	0.00	0.13	0.03	0.03	0.00	0.00	0.05	0.00	0.02	0.08	0.15	0.12	0.12	0.02	0.00	0.07	0.08	0.02
TCGA-AZ-4323	0.17	0.00	0.05	0.19	0.00	0.11	0.00	0.08	0.02	0.05	0.00	0.00	0.00	0.09	0.08	0.07	0.01	0.02	0.00	0.06	0.00	0.00
TCGA-AA-3971	0.11	0.00	0.09	0.13	0.00	0.02	0.09	0.01	0.00	0.02	0.00	0.00	0.00	0.12	0.08	0.15	0.05	0.00	0.01	0.06	0.05	0.01
TCGA-AA-3989	0.00	0.02	0.11	0.15	0.00	0.08	0.08	0.05	0.00	0.04	0.00	0.00	0.00	0.08	0.09	0.16	0.06	0.00	0.00	0.04	0.00	0.04
TCGA-AA-A00D	0.01	0.00	0.11	0.14	0.00	0.04	0.10	0.05	0.01	0.00	0.06	0.00	0.00	0.06	0.13	0.10	0.10	0.02	0.00	0.02	0.00	0.02
TCGA-AD-6548	0.04	0.00	0.04	0.09	0.00	0.06	0.16	0.04	0.00	0.00	0.00	0.01	0.00	0.17	0.13	0.12	0.02	0.02	0.00	0.06	0.00	0.04
TCGA-D5-6539	0.03	0.00	0.16	0.00	0.00	0.20	0.00	0.02	0.03	0.05	0.07	0.00	0.03	0.04	0.06	0.10	0.07	0.06	0.00	0.08	0.00	0.00
TCGA-A6-2679	0.01	0.00	0.14	0.21	0.00	0.05	0.00	0.07	0.07	0.03	0.00	0.00	0.00	0.13	0.07	0.10	0.04	0.00	0.00	0.08	0.00	0.00
TCGA-A6-2674	0.00	0.03	0.06	0.00	0.00	0.11	0.00	0.02	0.00	0.03	0.00	0.01	0.00	0.16	0.04	0.14	0.00	0.00	0.00	0.20	0.03	0.17
TCGA-AZ-4614	0.00	0.02	0.00	0.39	0.00	0.00	0.11	0.10	0.00	0.00	0.05	0.00	0.02	0.04	0.12	0.07	0.00	0.02	0.00	0.05	0.01	0.02

TCGA-NH-A5IV	0.04	0.00	0.08	0.13	0.00	0.00	0.02	0.09	0.02	0.08	0.00	0.08	0.00	0.13	0.08	0.20	0.02	0.00	0.00	0.03	0.00	0.00
TCGA-A6-5664	0.02	0.01	0.04	0.15	0.00	0.10	0.03	0.01	0.00	0.00	0.07	0.00	0.02	0.06	0.05	0.12	0.13	0.02	0.00	0.14	0.01	0.03
TCGA-AZ-4315	0.00	0.10	0.07	0.07	0.00	0.06	0.03	0.10	0.00	0.00	0.01	0.05	0.00	0.08	0.17	0.16	0.08	0.00	0.00	0.01	0.00	0.01
TCGA-AA-3510	0.03	0.00	0.07	0.17	0.00	0.02	0.08	0.06	0.00	0.00	0.01	0.00	0.00	0.12	0.13	0.17	0.03	0.00	0.00	0.08	0.02	0.01
TCGA-AY-A71X	0.01	0.00	0.10	0.20	0.00	0.19	0.00	0.02	0.03	0.00	0.00	0.01	0.00	0.22	0.01	0.09	0.00	0.00	0.00	0.14	0.00	0.00
TCGA-AA-3488	0.00	0.00	0.10	0.11	0.00	0.11	0.08	0.00	0.03	0.00	0.03	0.03	0.02	0.11	0.06	0.12	0.06	0.00	0.00	0.08	0.00	0.06
TCGA-DM-A28F	0.04	0.06	0.02	0.12	0.00	0.13	0.00	0.06	0.02	0.00	0.11	0.00	0.00	0.11	0.13	0.02	0.04	0.00	0.00	0.14	0.00	0.00
TCGA-G4-6293	0.05	0.00	0.09	0.06	0.00	0.20	0.22	0.00	0.00	0.03	0.05	0.00	0.00	0.11	0.07	0.06	0.02	0.01	0.00	0.02	0.00	0.01
TCGA-D5-6533	0.00	0.03	0.07	0.07	0.00	0.08	0.07	0.07	0.00	0.00	0.00	0.01	0.00	0.19	0.13	0.11	0.04	0.04	0.00	0.07	0.00	0.03
TCGA-AA-3941	0.08	0.00	0.02	0.08	0.00	0.30	0.00	0.01	0.03	0.00	0.00	0.00	0.00	0.00	0.07	0.16	0.15	0.01	0.00	0.04	0.05	0.01
TCGA-DM-A28K	0.00	0.05	0.02	0.07	0.00	0.16	0.00	0.04	0.00	0.00	0.00	0.03	0.00	0.28	0.08	0.14	0.00	0.00	0.00	0.12	0.01	0.00
TCGA-CM-5344	0.08	0.00	0.06	0.14	0.00	0.04	0.00	0.00	0.06	0.00	0.04	0.00	0.00	0.32	0.06	0.09	0.02	0.00	0.00	0.06	0.03	0.00
TCGA-AA-3517	0.01	0.00	0.14	0.06	0.00	0.13	0.10	0.00	0.00	0.00	0.08	0.00	0.00	0.13	0.03	0.14	0.04	0.00	0.00	0.10	0.00	0.03
TCGA-G4-6309	0.03	0.01	0.05	0.21	0.00	0.15	0.07	0.04	0.04	0.00	0.00	0.00	0.00	0.06	0.03	0.11	0.03	0.03	0.00	0.13	0.00	0.00
TCGA-AY-6386	0.00	0.02	0.10	0.11	0.00	0.09	0.03	0.03	0.02	0.01	0.06	0.00	0.00	0.20	0.06	0.06	0.06	0.01	0.00	0.12	0.00	0.02
TCGA-AA-3678	0.02	0.03	0.04	0.08	0.00	0.20	0.08	0.00	0.03	0.00	0.01	0.00	0.00	0.13	0.09	0.10	0.02	0.05	0.00	0.07	0.01	0.05
TCGA-AA-A01X	0.19	0.00	0.01	0.10	0.00	0.13	0.05	0.09	0.01	0.00	0.00	0.04	0.01	0.08	0.04	0.06	0.15	0.00	0.05	0.00	0.00	0.00
TCGA-CM-6677	0.00	0.03	0.08	0.12	0.00	0.10	0.12	0.06	0.00	0.00	0.03	0.00	0.00	0.12	0.10	0.11	0.04	0.00	0.02	0.05	0.00	0.00
TCGA-CM-5861	0.01	0.00	0.01	0.23	0.00	0.04	0.06	0.07	0.02	0.00	0.00	0.04	0.00	0.13	0.20	0.07	0.01	0.00	0.00	0.07	0.00	0.04
TCGA-D5-6923	0.00	0.03	0.04	0.01	0.00	0.13	0.03	0.00	0.00	0.00	0.05	0.00	0.00	0.30	0.07	0.09	0.03	0.02	0.00	0.14	0.00	0.05
TCGA-AZ-6605	0.01	0.01	0.10	0.00	0.00	0.16	0.04	0.04	0.01	0.04	0.02	0.00	0.00	0.19	0.11	0.11	0.06	0.00	0.00	0.08	0.00	0.02
TCGA-NH-A6GB	0.00	0.01	0.11	0.00	0.00	0.09	0.06	0.08	0.03	0.08	0.05	0.00	0.00	0.16	0.04	0.09	0.00	0.03	0.00	0.11	0.00	0.06
TCGA-CM-5349	0.11	0.00	0.00	0.05	0.00	0.09	0.02	0.00	0.02	0.00	0.00	0.01	0.01	0.42	0.10	0.10	0.06	0.00	0.00	0.00	0.01	0.00
TCGA-NH-A50U	0.00	0.09	0.01	0.02	0.00	0.05	0.00	0.06	0.05	0.00	0.00	0.01	0.00	0.34	0.05	0.17	0.00	0.00	0.00	0.14	0.00	0.01
TCGA-AA-3530	0.01	0.05	0.10	0.11	0.00	0.23	0.04	0.04	0.00	0.00	0.00	0.00	0.00	0.04	0.06	0.13	0.11	0.00	0.05	0.00	0.02	0.00
TCGA-G4-6627	0.08	0.00	0.06	0.08	0.00	0.17	0.06	0.05	0.00	0.02	0.01	0.00	0.00	0.04	0.12	0.12	0.03	0.04	0.00	0.07	0.01	0.03
TCGA-DM-A28G	0.00	0.04	0.09	0.15	0.00	0.26	0.05	0.02	0.00	0.00	0.01	0.00	0.00	0.14	0.04	0.08	0.06	0.00	0.00	0.05	0.00	0.00
TCGA-D5-6537	0.02	0.00	0.00	0.13	0.00	0.09	0.17	0.00	0.00	0.00	0.00	0.00	0.00	0.15	0.07	0.04	0.00	0.05	0.00	0.15	0.00	0.11
TCGA-D5-5539	0.04	0.00	0.09	0.00	0.00	0.16	0.00	0.01	0.00	0.00	0.00	0.00	0.00	0.26	0.08	0.13	0.04	0.00	0.00	0.11	0.02	0.05
TCGA-CM-6163	0.00	0.01	0.11	0.10	0.00	0.13	0.04	0.02	0.03	0.06	0.00	0.00	0.00	0.10	0.08	0.13	0.09	0.02	0.01	0.00	0.04	0.04
TCGA-CM-4744	0.03	0.00	0.02	0.30	0.00	0.00	0.08	0.11	0.01	0.04	0.00	0.07	0.00	0.00	0.07	0.10	0.10	0.01	0.00	0.05	0.00	0.00
TCGA-AA-A004	0.11	0.00	0.16	0.18	0.00	0.02	0.02	0.06	0.09	0.00	0.05	0.02	0.00	0.18	0.06	0.05	0.00	0.00	0.00	0.01	0.00	0.00
TCGA-A6-3808	0.06	0.00	0.03	0.08	0.00	0.09	0.04	0.06	0.00	0.00	0.00	0.01	0.00	0.10	0.08	0.13	0.07	0.00	0.00	0.13	0.04	0.06
TCGA-AA-A02J	0.00	0.02	0.15	0.00	0.00	0.20	0.18	0.01	0.03	0.00	0.05	0.00	0.00	0.22	0.02	0.05	0.02	0.03	0.00	0.02	0.00	0.00
TCGA-AA-3837	0.06	0.00	0.16	0.17	0.00	0.16	0.00	0.00	0.07	0.00	0.02	0.00	0.00	0.01	0.07	0.13	0.06	0.03	0.00	0.05	0.02	0.00
TCGA-CK-4947	0.04	0.01	0.06	0.11	0.00	0.21	0.04	0.08	0.00	0.00	0.00	0.00	0.00	0.07	0.09	0.10	0.12	0.02	0.00	0.04	0.00	0.00
TCGA-CA-5256	0.00	0.03	0.08	0.18	0.00	0.13	0.11	0.00	0.00	0.00	0.01	0.00	0.00	0.01	0.06	0.10	0.16	0.01	0.00	0.09	0.02	0.00
TCGA-CM-6167	0.11	0.00	0.02	0.07	0.00	0.17	0.00	0.07	0.00	0.01	0.00	0.00	0.00	0.03	0.13	0.17	0.08	0.00	0.12	0.00	0.02	0.00

TCGA-4N-A93T	0.13	0.00	0.20	0.17	0.00	0.17	0.00	0.06	0.01	0.00	0.04	0.00	0.02	0.03	0.03	0.00	0.00	0.00	0.00	0.15	0.00	0.00
TCGA-CA-5797	0.02	0.00	0.07	0.13	0.00	0.03	0.05	0.03	0.04	0.00	0.03	0.00	0.00	0.21	0.14	0.12	0.06	0.00	0.07	0.00	0.00	0.00
TCGA-CM-6166	0.03	0.00	0.01	0.00	0.00	0.16	0.11	0.03	0.04	0.00	0.11	0.00	0.00	0.31	0.01	0.05	0.00	0.03	0.00	0.09	0.00	0.00
TCGA-CK-4951	0.02	0.00	0.06	0.09	0.00	0.03	0.04	0.11	0.01	0.00	0.00	0.02	0.00	0.12	0.13	0.16	0.01	0.05	0.00	0.09	0.00	0.06
TCGA-CA-6719	0.01	0.02	0.04	0.13	0.00	0.02	0.02	0.02	0.05	0.02	0.00	0.02	0.00	0.29	0.14	0.16	0.02	0.00	0.00	0.05	0.00	0.00
TCGA-AA-A02R	0.01	0.01	0.00	0.18	0.00	0.00	0.12	0.11	0.00	0.00	0.00	0.06	0.00	0.12	0.19	0.11	0.03	0.00	0.04	0.00	0.00	0.00
TCGA-A6-5666	0.00	0.00	0.06	0.00	0.00	0.17	0.00	0.00	0.00	0.04	0.02	0.00	0.00	0.28	0.12	0.10	0.00	0.00	0.00	0.10	0.01	0.08
TCGA-AY-5543	0.07	0.00	0.06	0.16	0.00	0.09	0.09	0.09	0.00	0.00	0.00	0.00	0.00	0.11	0.12	0.08	0.04	0.04	0.00	0.05	0.00	0.00
TCGA-F4-6704	0.00	0.00	0.07	0.11	0.00	0.23	0.04	0.00	0.00	0.00	0.00	0.00	0.00	0.20	0.09	0.14	0.06	0.00	0.05	0.00	0.00	0.01
TCGA-AA-A00Q	0.06	0.00	0.02	0.14	0.00	0.07	0.01	0.02	0.05	0.01	0.00	0.00	0.00	0.15	0.17	0.14	0.10	0.00	0.00	0.05	0.00	0.00
TCGA-A6-6781	0.00	0.03	0.02	0.03	0.00	0.17	0.00	0.06	0.00	0.03	0.00	0.01	0.00	0.15	0.10	0.10	0.01	0.03	0.00	0.15	0.00	0.12
TCGA-AA-A00F	0.03	0.00	0.15	0.08	0.00	0.22	0.00	0.05	0.00	0.00	0.08	0.00	0.00	0.09	0.04	0.11	0.02	0.04	0.00	0.09	0.00	0.00
TCGA-D5-6920	0.00	0.01	0.12	0.00	0.00	0.21	0.15	0.00	0.01	0.03	0.00	0.00	0.00	0.15	0.04	0.09	0.00	0.06	0.00	0.09	0.00	0.03
TCGA-AA-3815	0.06	0.00	0.01	0.24	0.00	0.00	0.10	0.05	0.03	0.00	0.03	0.02	0.00	0.10	0.14	0.11	0.04	0.03	0.02	0.00	0.00	0.03
TCGA-D5-5538	0.05	0.00	0.04	0.00	0.00	0.11	0.00	0.03	0.01	0.07	0.00	0.00	0.00	0.16	0.11	0.16	0.08	0.00	0.00	0.11	0.02	0.05
TCGA-AA-3867	0.10	0.00	0.07	0.14	0.00	0.02	0.10	0.00	0.01	0.00	0.06	0.00	0.00	0.04	0.07	0.21	0.10	0.00	0.01	0.03	0.04	0.00
TCGA-A6-6782	0.05	0.00	0.06	0.12	0.00	0.05	0.06	0.03	0.00	0.06	0.00	0.00	0.00	0.13	0.14	0.17	0.01	0.00	0.00	0.10	0.00	0.01
TCGA-A6-6654	0.06	0.00	0.02	0.08	0.00	0.09	0.05	0.03	0.00	0.00	0.04	0.00	0.00	0.21	0.11	0.14	0.04	0.02	0.00	0.09	0.01	0.02
TCGA-AA-A01D	0.00	0.01	0.01	0.02	0.00	0.09	0.00	0.07	0.03	0.00	0.02	0.00	0.01	0.38	0.09	0.11	0.06	0.00	0.00	0.08	0.00	0.01
TCGA-G4-6311	0.13	0.00	0.04	0.05	0.00	0.03	0.04	0.02	0.02	0.00	0.05	0.00	0.00	0.27	0.06	0.08	0.04	0.00	0.00	0.13	0.01	0.03
TCGA-A6-3807	0.03	0.00	0.01	0.00	0.00	0.14	0.04	0.11	0.00	0.03	0.00	0.02	0.00	0.10	0.13	0.11	0.07	0.00	0.00	0.03	0.05	0.13
TCGA-AZ-5403	0.00	0.01	0.02	0.03	0.00	0.16	0.05	0.00	0.01	0.00	0.01	0.00	0.00	0.21	0.03	0.09	0.12	0.00	0.00	0.19	0.02	0.04
TCGA-AD-6888	0.02	0.00	0.11	0.16	0.00	0.14	0.08	0.00	0.02	0.00	0.03	0.03	0.01	0.22	0.00	0.00	0.06	0.11	0.00	0.02	0.00	0.00
TCGA-G4-6628	0.03	0.00	0.02	0.22	0.00	0.00	0.17	0.05	0.02	0.01	0.03	0.00	0.00	0.05	0.16	0.12	0.08	0.02	0.00	0.02	0.00	0.01
TCGA-DM-A1D6	0.00	0.09	0.10	0.12	0.00	0.23	0.00	0.11	0.00	0.00	0.00	0.03	0.00	0.16	0.00	0.06	0.00	0.06	0.00	0.04	0.00	0.00
TCGA-CM-5341	0.00	0.00	0.03	0.07	0.00	0.12	0.04	0.03	0.01	0.01	0.02	0.00	0.00	0.17	0.10	0.15	0.09	0.00	0.00	0.05	0.08	0.03
TCGA-AA-3862	0.07	0.00	0.01	0.15	0.00	0.04	0.08	0.11	0.02	0.01	0.00	0.00	0.00	0.01	0.14	0.11	0.05	0.04	0.00	0.09	0.03	0.04
TCGA-AA-3939	0.05	0.00	0.07	0.08	0.00	0.15	0.10	0.05	0.00	0.03	0.00	0.00	0.02	0.00	0.10	0.06	0.09	0.00	0.00	0.16	0.02	0.01
TCGA-AA-3660	0.03	0.02	0.05	0.06	0.00	0.09	0.06	0.00	0.00	0.00	0.10	0.00	0.00	0.00	0.01	0.18	0.14	0.07	0.01	0.09	0.04	0.04
TCGA-AA-3833	0.05	0.00	0.06	0.15	0.00	0.04	0.08	0.05	0.00	0.00	0.04	0.00	0.00	0.12	0.06	0.09	0.02	0.02	0.00	0.16	0.01	0.05
TCGA-AA-A01P	0.02	0.00	0.05	0.23	0.00	0.00	0.09	0.05	0.05	0.00	0.02	0.02	0.00	0.10	0.18	0.11	0.03	0.01	0.00	0.01	0.00	0.03
TCGA-AA-A00Z	0.00	0.07	0.03	0.11	0.00	0.08	0.14	0.03	0.00	0.00	0.03	0.00	0.00	0.12	0.12	0.11	0.02	0.03	0.09	0.00	0.00	0.01
TCGA-CM-5863	0.00	0.02	0.06	0.11	0.00	0.18	0.00	0.00	0.00	0.00	0.02	0.00	0.02	0.18	0.10	0.13	0.02	0.00	0.00	0.12	0.00	0.03
TCGA-AZ-6598	0.04	0.00	0.02	0.09	0.00	0.11	0.05	0.05	0.00	0.00	0.05	0.05	0.01	0.15	0.13	0.12	0.09	0.00	0.04	0.00	0.00	0.01
TCGA-AA-3679	0.00	0.02	0.10	0.08	0.00	0.16	0.10	0.00	0.00	0.00	0.07	0.00	0.02	0.09	0.08	0.16	0.07	0.00	0.01	0.01	0.01	0.02
TCGA-G4-6304	0.01	0.00	0.02	0.22	0.00	0.00	0.10	0.07	0.00	0.01	0.00	0.07	0.00	0.11	0.18	0.16	0.04	0.00	0.00	0.00	0.00	0.01
TCGA-DM-A285	0.00	0.04	0.07	0.08	0.00	0.06	0.00	0.04	0.07	0.00	0.00	0.02	0.00	0.35	0.08	0.13	0.03	0.00	0.00	0.00	0.03	0.00
TCGA-G4-6294	0.01	0.00	0.06	0.09	0.00	0.20	0.16	0.03	0.00	0.00	0.05	0.00	0.01	0.14	0.01	0.09	0.09	0.00	0.00	0.06	0.01	0.00

TCGA-A6-4107	0.06	0.00	0.10	0.05	0.00	0.16	0.00	0.03	0.00	0.00	0.07	0.00	0.01	0.04	0.08	0.14	0.10	0.00	0.00	0.16	0.01	0.00
TCGA-DM-A1D4	0.00	0.00	0.07	0.17	0.00	0.18	0.21	0.04	0.01	0.00	0.05	0.00	0.00	0.03	0.03	0.06	0.02	0.02	0.00	0.12	0.00	0.00
TCGA-CA-6716	0.08	0.00	0.09	0.01	0.00	0.23	0.07	0.00	0.03	0.00	0.04	0.00	0.02	0.23	0.03	0.07	0.05	0.00	0.00	0.05	0.00	0.00
TCGA-CK-4952	0.00	0.00	0.05	0.00	0.00	0.24	0.00	0.00	0.00	0.00	0.01	0.00	0.00	0.19	0.03	0.11	0.00	0.04	0.00	0.16	0.06	0.11
TCGA-AA-3844	0.03	0.00	0.02	0.05	0.00	0.24	0.07	0.10	0.00	0.00	0.01	0.00	0.00	0.09	0.07	0.09	0.06	0.06	0.00	0.08	0.00	0.01
TCGA-D5-6541	0.01	0.00	0.04	0.09	0.00	0.08	0.04	0.05	0.02	0.01	0.00	0.02	0.00	0.14	0.17	0.17	0.05	0.00	0.00	0.06	0.00	0.05
TCGA-AA-A01G	0.01	0.06	0.05	0.19	0.00	0.08	0.00	0.07	0.01	0.00	0.00	0.08	0.03	0.12	0.06	0.14	0.02	0.03	0.00	0.07	0.00	0.00
TCGA-AA-3870	0.05	0.00	0.05	0.16	0.00	0.00	0.05	0.03	0.05	0.00	0.02	0.00	0.00	0.13	0.08	0.13	0.14	0.00	0.00	0.04	0.05	0.03
TCGA-D5-6532	0.00	0.10	0.18	0.13	0.00	0.05	0.16	0.00	0.00	0.00	0.01	0.00	0.01	0.08	0.11	0.08	0.00	0.01	0.07	0.00	0.00	0.01
TCGA-CM-6169	0.00	0.01	0.09	0.12	0.00	0.04	0.11	0.01	0.01	0.00	0.02	0.00	0.00	0.17	0.11	0.10	0.07	0.01	0.05	0.00	0.04	0.03
TCGA-D5-5541	0.06	0.00	0.03	0.11	0.00	0.01	0.00	0.04	0.03	0.00	0.00	0.00	0.00	0.33	0.12	0.14	0.04	0.00	0.00	0.06	0.00	0.02
TCGA-DM-A1DE	0.05	0.00	0.03	0.35	0.00	0.00	0.16	0.04	0.00	0.00	0.03	0.00	0.00	0.10	0.08	0.04	0.07	0.00	0.00	0.05	0.00	0.00
TCGA-AA-A00A	0.05	0.00	0.03	0.11	0.00	0.07	0.03	0.09	0.00	0.00	0.00	0.04	0.00	0.15	0.15	0.16	0.01	0.00	0.00	0.09	0.00	0.00
TCGA-D5-6530	0.05	0.00	0.03	0.12	0.00	0.08	0.08	0.06	0.00	0.03	0.01	0.01	0.00	0.15	0.15	0.12	0.01	0.00	0.00	0.07	0.00	0.04
TCGA-CM-5868	0.02	0.04	0.03	0.08	0.00	0.09	0.01	0.00	0.07	0.03	0.00	0.00	0.00	0.34	0.09	0.15	0.03	0.01	0.01	0.00	0.00	0.01
TCGA-AA-3519	0.00	0.01	0.09	0.07	0.00	0.07	0.12	0.06	0.00	0.00	0.04	0.00	0.00	0.10	0.05	0.12	0.02	0.06	0.00	0.12	0.06	0.03
TCGA-AA-3526	0.05	0.00	0.03	0.13	0.00	0.05	0.12	0.10	0.00	0.01	0.00	0.00	0.00	0.06	0.15	0.15	0.02	0.02	0.00	0.05	0.04	0.02
TCGA-DM-A28M	0.00	0.03	0.02	0.06	0.00	0.35	0.09	0.02	0.05	0.00	0.06	0.00	0.00	0.00	0.09	0.02	0.08	0.02	0.00	0.11	0.00	0.00
TCGA-D5-6926	0.01	0.00	0.04	0.00	0.00	0.05	0.09	0.03	0.00	0.00	0.04	0.00	0.00	0.26	0.18	0.10	0.04	0.00	0.00	0.11	0.00	0.07
TCGA-AA-3534	0.05	0.00	0.02	0.04	0.00	0.21	0.13	0.02	0.00	0.00	0.04	0.00	0.00	0.12	0.04	0.15	0.06	0.02	0.00	0.06	0.01	0.03
TCGA-G4-6317	0.00	0.06	0.04	0.24	0.00	0.05	0.05	0.06	0.04	0.00	0.05	0.00	0.01	0.20	0.02	0.06	0.02	0.00	0.00	0.12	0.00	0.00
TCGA-AA-A03J	0.00	0.07	0.01	0.19	0.00	0.00	0.16	0.01	0.02	0.00	0.02	0.01	0.00	0.11	0.09	0.16	0.05	0.02	0.00	0.06	0.00	0.01
TCGA-CM-6680	0.01	0.00	0.07	0.09	0.00	0.20	0.03	0.02	0.06	0.00	0.04	0.00	0.00	0.16	0.08	0.12	0.05	0.01	0.00	0.06	0.00	0.00
TCGA-D5-6536	0.00	0.00	0.02	0.02	0.00	0.18	0.00	0.00	0.00	0.00	0.03	0.00	0.00	0.28	0.00	0.12	0.01	0.02	0.00	0.25	0.00	0.08
TCGA-AD-A5EK	0.00	0.00	0.03	0.05	0.00	0.29	0.07	0.03	0.04	0.00	0.08	0.00	0.01	0.15	0.03	0.11	0.00	0.07	0.01	0.02	0.00	0.00
TCGA-AA-3663	0.04	0.00	0.01	0.09	0.00	0.09	0.00	0.08	0.00	0.00	0.00	0.00	0.00	0.10	0.08	0.19	0.06	0.00	0.00	0.19	0.03	0.02
TCGA-AZ-6607	0.00	0.01	0.03	0.14	0.00	0.15	0.06	0.01	0.01	0.00	0.04	0.00	0.01	0.09	0.12	0.08	0.09	0.02	0.00	0.13	0.00	0.01
TCGA-AZ-5407	0.08	0.00	0.08	0.18	0.00	0.31	0.00	0.03	0.01	0.00	0.03	0.00	0.02	0.04	0.05	0.10	0.03	0.00	0.00	0.04	0.00	0.00
TCGA-G4-6314	0.03	0.00	0.04	0.12	0.00	0.12	0.03	0.05	0.00	0.00	0.03	0.00	0.01	0.17	0.12	0.15	0.07	0.00	0.00	0.06	0.00	0.00
TCGA-CM-5348	0.00	0.03	0.00	0.04	0.00	0.10	0.00	0.01	0.03	0.00	0.00	0.00	0.00	0.40	0.08	0.11	0.09	0.02	0.03	0.03	0.00	0.03
TCGA-CK-6751	0.03	0.00	0.06	0.15	0.00	0.09	0.11	0.06	0.00	0.00	0.01	0.01	0.00	0.15	0.09	0.12	0.04	0.00	0.00	0.10	0.00	0.00
TCGA-D5-6929	0.01	0.02	0.12	0.14	0.00	0.02	0.09	0.03	0.00	0.02	0.00	0.00	0.00	0.23	0.10	0.13	0.02	0.00	0.00	0.04	0.03	0.00
TCGA-AA-3869	0.02	0.00	0.01	0.11	0.00	0.09	0.12	0.02	0.00	0.00	0.04	0.00	0.00	0.14	0.11	0.14	0.05	0.00	0.00	0.05	0.01	0.08
TCGA-DM-A282	0.00	0.07	0.06	0.10	0.00	0.15	0.00	0.05	0.05	0.00	0.00	0.05	0.00	0.15	0.11	0.12	0.07	0.00	0.01	0.00	0.00	0.00
TCGA-D5-7000	0.01	0.01	0.06	0.02	0.00	0.12	0.00	0.06	0.02	0.04	0.00	0.00	0.00	0.17	0.08	0.09	0.03	0.03	0.00	0.14	0.04	0.08
TCGA-G4-6298	0.00	0.10	0.01	0.06	0.00	0.07	0.00	0.08	0.00	0.00	0.04	0.00	0.00	0.32	0.06	0.07	0.01	0.00	0.00	0.19	0.00	0.00
TCGA-NH-A8F8	0.01	0.03	0.07	0.14	0.00	0.00	0.00	0.06	0.01	0.02	0.00	0.00	0.00	0.15	0.12	0.22	0.00	0.01	0.00	0.08	0.05	0.03
TCGA-AA-3966	0.00	0.00	0.05	0.13	0.00	0.07	0.07	0.02	0.00	0.00	0.03	0.00	0.01	0.13	0.08	0.07	0.09	0.02	0.00	0.13	0.03	0.05

TCGA-AA-3516	0.00	0.05	0.01	0.15	0.00	0.01	0.11	0.05	0.00	0.04	0.00	0.03	0.00	0.06	0.13	0.10	0.02	0.10	0.00	0.08	0.00	0.04
TCGA-AA-3846	0.00	0.00	0.12	0.03	0.00	0.18	0.14	0.03	0.00	0.00	0.04	0.00	0.00	0.09	0.04	0.11	0.10	0.03	0.00	0.03	0.04	0.01
TCGA-AA-3544	0.05	0.00	0.02	0.19	0.00	0.00	0.03	0.04	0.02	0.00	0.00	0.03	0.00	0.19	0.13	0.17	0.05	0.00	0.00	0.05	0.00	0.00
TCGA-AA-3956	0.03	0.00	0.20	0.03	0.00	0.22	0.10	0.00	0.01	0.00	0.01	0.00	0.00	0.04	0.06	0.10	0.05	0.02	0.02	0.06	0.00	0.05
TCGA-AA-3532	0.04	0.01	0.09	0.02	0.00	0.16	0.07	0.05	0.00	0.00	0.03	0.00	0.00	0.06	0.13	0.13	0.10	0.00	0.00	0.04	0.02	0.03
TCGA-AA-3955	0.03	0.00	0.03	0.16	0.00	0.06	0.12	0.00	0.00	0.00	0.09	0.00	0.04	0.03	0.04	0.18	0.06	0.01	0.00	0.16	0.00	0.00
TCGA-AD-6895	0.00	0.02	0.03	0.20	0.00	0.06	0.12	0.08	0.00	0.00	0.06	0.00	0.00	0.05	0.15	0.10	0.06	0.02	0.04	0.02	0.00	0.00
TCGA-AA-3556	0.07	0.00	0.05	0.06	0.00	0.13	0.06	0.00	0.03	0.00	0.01	0.00	0.00	0.07	0.11	0.19	0.04	0.05	0.00	0.06	0.00	0.07
TCGA-CM-6678	0.00	0.04	0.06	0.04	0.00	0.20	0.00	0.00	0.05	0.00	0.03	0.00	0.00	0.26	0.02	0.04	0.01	0.03	0.00	0.21	0.00	0.02
TCGA-AA-3851	0.05	0.00	0.06	0.13	0.00	0.11	0.00	0.09	0.03	0.01	0.00	0.00	0.02	0.05	0.10	0.13	0.11	0.00	0.00	0.12	0.00	0.00
TCGA-CM-5860	0.00	0.00	0.08	0.02	0.00	0.21	0.00	0.02	0.03	0.00	0.00	0.00	0.00	0.18	0.14	0.17	0.06	0.00	0.00	0.09	0.00	0.02
TCGA-AA-A02F	0.02	0.00	0.05	0.08	0.00	0.21	0.00	0.00	0.06	0.00	0.02	0.00	0.00	0.25	0.10	0.06	0.02	0.00	0.00	0.13	0.00	0.00
TCGA-DM-A1DA	0.01	0.01	0.07	0.23	0.00	0.03	0.00	0.06	0.00	0.00	0.03	0.00	0.03	0.10	0.13	0.15	0.05	0.00	0.00	0.08	0.03	0.00
TCGA-CM-6162	0.04	0.00	0.01	0.07	0.00	0.17	0.05	0.05	0.00	0.00	0.00	0.00	0.00	0.13	0.13	0.16	0.04	0.02	0.00	0.05	0.01	0.08
TCGA-AA-3522	0.15	0.00	0.06	0.10	0.00	0.25	0.00	0.10	0.00	0.00	0.00	0.00	0.01	0.02	0.05	0.09	0.04	0.04	0.00	0.09	0.00	0.00
TCGA-AA-A00U	0.04	0.00	0.03	0.17	0.00	0.20	0.04	0.05	0.01	0.04	0.00	0.00	0.00	0.03	0.05	0.11	0.02	0.06	0.00	0.12	0.00	0.00
TCGA-AA-3930	0.05	0.00	0.01	0.05	0.00	0.21	0.00	0.04	0.00	0.00	0.03	0.00	0.00	0.07	0.08	0.11	0.06	0.04	0.00	0.13	0.02	0.11
TCGA-AZ-6599	0.10	0.00	0.08	0.29	0.00	0.23	0.00	0.04	0.02	0.00	0.04	0.00	0.03	0.00	0.00	0.06	0.00	0.04	0.00	0.07	0.00	0.00
TCGA-AA-3947	0.00	0.04	0.01	0.00	0.00	0.15	0.04	0.04	0.00	0.10	0.00	0.00	0.00	0.00	0.10	0.14	0.08	0.04	0.00	0.15	0.04	0.07
TCGA-CM-6171	0.00	0.00	0.03	0.08	0.00	0.18	0.03	0.03	0.00	0.00	0.00	0.02	0.00	0.07	0.09	0.15	0.02	0.02	0.00	0.14	0.00	0.15
TCGA-CM-4743	0.02	0.00	0.07	0.18	0.00	0.00	0.15	0.01	0.00	0.00	0.04	0.05	0.00	0.02	0.20	0.07	0.05	0.03	0.00	0.08	0.00	0.04
TCGA-A6-2675	0.05	0.00	0.06	0.03	0.00	0.05	0.00	0.02	0.05	0.01	0.02	0.00	0.00	0.18	0.11	0.22	0.03	0.00	0.00	0.12	0.00	0.05
TCGA-G4-6297	0.07	0.00	0.05	0.12	0.00	0.11	0.00	0.02	0.01	0.00	0.04	0.00	0.00	0.16	0.12	0.08	0.13	0.01	0.00	0.04	0.00	0.03
TCGA-A6-5656	0.00	0.02	0.04	0.00	0.03	0.18	0.13	0.04	0.00	0.00	0.03	0.00	0.00	0.29	0.04	0.05	0.03	0.04	0.00	0.04	0.02	0.02
TCGA-DM-A0X9	0.00	0.09	0.03	0.19	0.00	0.05	0.03	0.13	0.01	0.07	0.00	0.00	0.00	0.00	0.14	0.08	0.08	0.00	0.00	0.09	0.00	0.00
TCGA-AA-3553	0.02	0.00	0.09	0.12	0.00	0.05	0.17	0.03	0.00	0.00	0.01	0.00	0.00	0.10	0.09	0.10	0.10	0.02	0.02	0.00	0.02	0.04
TCGA-DM-A0XI	0.00	0.04	0.11	0.10	0.00	0.00	0.00	0.02	0.04	0.00	0.07	0.00	0.00	0.21	0.07	0.17	0.01	0.03	0.02	0.10	0.00	0.02
TCGA-AA-3511	0.03	0.00	0.04	0.12	0.00	0.10	0.04	0.02	0.05	0.00	0.02	0.00	0.00	0.19	0.08	0.20	0.01	0.03	0.02	0.04	0.00	0.02
TCGA-A6-5667	0.01	0.00	0.02	0.00	0.00	0.11	0.00	0.06	0.07	0.00	0.06	0.00	0.00	0.18	0.11	0.11	0.05	0.00	0.00	0.19	0.02	0.00
TCGA-AA-3866	0.02	0.00	0.07	0.03	0.00	0.12	0.09	0.02	0.01	0.04	0.00	0.00	0.00	0.13	0.09	0.11	0.11	0.03	0.00	0.06	0.01	0.05
TCGA-AA-A017	0.03	0.00	0.12	0.12	0.00	0.12	0.03	0.04	0.02	0.00	0.00	0.01	0.00	0.18	0.03	0.05	0.01	0.06	0.00	0.10	0.00	0.08
TCGA-CM-6674	0.01	0.01	0.01	0.19	0.00	0.05	0.09	0.02	0.00	0.00	0.00	0.00	0.00	0.16	0.21	0.16	0.04	0.00	0.00	0.05	0.00	0.00
TCGA-DM-A1D8	0.02	0.00	0.00	0.21	0.00	0.00	0.02	0.02	0.14	0.00	0.04	0.02	0.00	0.31	0.04	0.11	0.00	0.00	0.00	0.08	0.00	0.00
TCGA-CM-6170	0.02	0.05	0.01	0.08	0.00	0.10	0.00	0.07	0.01	0.00	0.05	0.00	0.00	0.16	0.11	0.16	0.02	0.00	0.00	0.11	0.00	0.04
TCGA-AZ-4616	0.02	0.01	0.05	0.26	0.00	0.00	0.11	0.08	0.00	0.00	0.00	0.02	0.00	0.07	0.19	0.12	0.01	0.03	0.00	0.02	0.00	0.00
TCGA-AA-3994	0.00	0.05	0.06	0.08	0.00	0.17	0.00	0.00	0.03	0.00	0.02	0.00	0.02	0.09	0.04	0.12	0.08	0.02	0.00	0.16	0.08	0.00
TCGA-AA-3877	0.03	0.00	0.01	0.07	0.00	0.12	0.03	0.09	0.00	0.00	0.01	0.01	0.00	0.06	0.14	0.13	0.06	0.00	0.00	0.07	0.02	0.13
TCGA-WS-AB45	0.03	0.00	0.02	0.07	0.00	0.14	0.07	0.00	0.00	0.00	0.07	0.00	0.02	0.14	0.07	0.17	0.00	0.01	0.00	0.04	0.02	0.13

TCGA-AA-A00L	0.00	0.01	0.08	0.00	0.00	0.15	0.11	0.03	0.05	0.00	0.08	0.00	0.00	0.22	0.04	0.04	0.00	0.05	0.00	0.14	0.00	0.00
TCGA-CM-5864	0.07	0.00	0.04	0.18	0.00	0.21	0.09	0.00	0.00	0.00	0.07	0.00	0.00	0.07	0.06	0.07	0.03	0.00	0.00	0.08	0.00	0.01
TCGA-CA-5254	0.01	0.06	0.08	0.03	0.00	0.21	0.00	0.00	0.07	0.03	0.00	0.02	0.00	0.19	0.05	0.11	0.00	0.01	0.06	0.05	0.00	0.00
TCGA-D5-6898	0.02	0.01	0.03	0.04	0.00	0.07	0.09	0.00	0.03	0.00	0.08	0.00	0.00	0.23	0.11	0.13	0.04	0.01	0.00	0.08	0.00	0.03
TCGA-AA-3696	0.02	0.00	0.07	0.02	0.00	0.33	0.00	0.01	0.00	0.00	0.02	0.00	0.02	0.12	0.04	0.17	0.00	0.04	0.00	0.15	0.00	0.00
TCGA-DM-A280	0.00	0.00	0.03	0.03	0.00	0.17	0.00	0.00	0.04	0.00	0.00	0.02	0.03	0.27	0.00	0.08	0.00	0.04	0.00	0.23	0.00	0.07
TCGA-QG-A5YV	0.01	0.01	0.16	0.04	0.00	0.24	0.00	0.06	0.02	0.00	0.01	0.00	0.00	0.12	0.07	0.09	0.01	0.02	0.00	0.13	0.01	0.00
TCGA-CK-4948	0.00	0.01	0.04	0.03	0.00	0.12	0.09	0.03	0.02	0.00	0.05	0.00	0.00	0.27	0.01	0.08	0.01	0.06	0.00	0.12	0.00	0.04
TCGA-DM-A1HA	0.01	0.00	0.02	0.30	0.00	0.00	0.05	0.19	0.00	0.01	0.00	0.12	0.00	0.01	0.16	0.09	0.00	0.00	0.03	0.00	0.00	0.00
TCGA-F4-6809	0.08	0.02	0.02	0.12	0.00	0.09	0.01	0.07	0.02	0.03	0.02	0.00	0.00	0.12	0.13	0.14	0.06	0.00	0.00	0.09	0.00	0.00
TCGA-CM-4748	0.12	0.00	0.01	0.08	0.00	0.00	0.04	0.11	0.00	0.00	0.00	0.01	0.00	0.14	0.07	0.10	0.07	0.00	0.00	0.24	0.00	0.01
TCGA-AA-3858	0.04	0.00	0.14	0.07	0.00	0.16	0.05	0.06	0.02	0.00	0.00	0.00	0.00	0.04	0.11	0.15	0.06	0.00	0.00	0.09	0.02	0.00
TCGA-D5-6531	0.01	0.01	0.01	0.16	0.00	0.00	0.08	0.05	0.07	0.02	0.00	0.01	0.00	0.13	0.19	0.14	0.04	0.01	0.00	0.03	0.03	0.00
TCGA-AA-3662	0.00	0.01	0.10	0.11	0.00	0.01	0.06	0.01	0.00	0.00	0.06	0.00	0.00	0.17	0.13	0.14	0.02	0.00	0.00	0.11	0.02	0.05
TCGA-AA-A02K	0.00	0.02	0.09	0.19	0.01	0.00	0.08	0.00	0.02	0.00	0.04	0.00	0.00	0.11	0.01	0.23	0.00	0.04	0.00	0.07	0.00	0.09
TCGA-AD-6889	0.00	0.05	0.06	0.03	0.00	0.25	0.03	0.05	0.00	0.00	0.02	0.00	0.00	0.11	0.04	0.08	0.01	0.03	0.00	0.19	0.01	0.06
TCGA-AA-A01R	0.02	0.00	0.01	0.30	0.00	0.00	0.07	0.10	0.02	0.01	0.00	0.04	0.00	0.15	0.11	0.11	0.03	0.01	0.00	0.03	0.00	0.00
TCGA-AA-3975	0.00	0.00	0.14	0.05	0.00	0.11	0.05	0.02	0.01	0.00	0.09	0.00	0.00	0.16	0.07	0.14	0.05	0.00	0.00	0.08	0.01	0.00
TCGA-5M-AAT6	0.01	0.04	0.05	0.19	0.00	0.00	0.10	0.07	0.00	0.02	0.00	0.01	0.00	0.13	0.16	0.12	0.06	0.01	0.03	0.00	0.00	0.01
TCGA-D5-6931	0.03	0.01	0.09	0.10	0.00	0.10	0.01	0.08	0.00	0.01	0.03	0.00	0.00	0.02	0.11	0.14	0.13	0.00	0.00	0.12	0.00	0.02
TCGA-D5-6924	0.00	0.03	0.11	0.14	0.00	0.00	0.04	0.05	0.00	0.01	0.01	0.00	0.00	0.19	0.13	0.12	0.04	0.00	0.06	0.01	0.00	0.05
TCGA-AA-3860	0.09	0.00	0.06	0.07	0.00	0.14	0.06	0.06	0.00	0.01	0.00	0.00	0.00	0.15	0.08	0.12	0.02	0.03	0.00	0.08	0.01	0.01
TCGA-A6-5665	0.06	0.00	0.05	0.10	0.00	0.10	0.14	0.07	0.00	0.02	0.03	0.00	0.00	0.06	0.08	0.08	0.00	0.04	0.00	0.13	0.00	0.02
TCGA-D5-6540	0.00	0.00	0.02	0.17	0.00	0.00	0.04	0.05	0.02	0.00	0.08	0.00	0.00	0.18	0.11	0.07	0.01	0.01	0.00	0.17	0.00	0.08
TCGA-CA-6718	0.06	0.00	0.05	0.09	0.00	0.00	0.10	0.06	0.00	0.09	0.00	0.04	0.00	0.09	0.22	0.13	0.03	0.01	0.00	0.00	0.00	0.04
TCGA-G4-6323	0.24	0.00	0.14	0.06	0.00	0.26	0.01	0.04	0.00	0.01	0.03	0.00	0.00	0.03	0.04	0.08	0.01	0.03	0.00	0.02	0.00	0.00
TCGA-A6-A566	0.01	0.00	0.02	0.08	0.00	0.11	0.00	0.05	0.01	0.00	0.03	0.00	0.01	0.27	0.07	0.22	0.02	0.02	0.00	0.09	0.00	0.01
TCGA-AZ-6608	0.02	0.00	0.06	0.05	0.00	0.13	0.30	0.00	0.07	0.00	0.10	0.00	0.01	0.15	0.00	0.00	0.02	0.03	0.00	0.03	0.03	0.00
TCGA-A6-5662	0.00	0.04	0.02	0.04	0.00	0.17	0.00	0.00	0.03	0.00	0.00	0.00	0.00	0.09	0.08	0.19	0.23	0.00	0.07	0.00	0.02	0.02
TCGA-AA-3831	0.01	0.00	0.13	0.10	0.00	0.18	0.08	0.03	0.00	0.00	0.02	0.00	0.01	0.04	0.04	0.17	0.08	0.01	0.00	0.07	0.01	0.03
TCGA-AA-A02H	0.00	0.07	0.15	0.13	0.00	0.17	0.07	0.03	0.00	0.00	0.03	0.00	0.00	0.07	0.05	0.14	0.06	0.00	0.00	0.03	0.00	0.00
TCGA-CM-6676	0.00	0.02	0.06	0.05	0.00	0.16	0.12	0.00	0.01	0.00	0.05	0.00	0.00	0.26	0.04	0.09	0.04	0.00	0.00	0.11	0.00	0.00
TCGA-AD-6899	0.05	0.00	0.04	0.11	0.00	0.05	0.03	0.00	0.04	0.06	0.00	0.00	0.00	0.20	0.15	0.16	0.00	0.00	0.00	0.06	0.03	0.00
TCGA-AA-3685	0.02	0.02	0.05	0.05	0.00	0.13	0.11	0.07	0.00	0.04	0.00	0.02	0.00	0.06	0.14	0.19	0.04	0.00	0.03	0.00	0.02	0.00
TCGA-AA-3548	0.01	0.03	0.06	0.16	0.00	0.11	0.02	0.06	0.04	0.03	0.00	0.00	0.00	0.07	0.09	0.14	0.12	0.00	0.00	0.04	0.00	0.00
TCGA-F4-6569	0.10	0.00	0.03	0.07	0.00	0.14	0.02	0.00	0.02	0.01	0.02	0.00	0.00	0.29	0.09	0.12	0.01	0.00	0.00	0.07	0.00	0.02
TCGA-F4-6461	0.27	0.00	0.03	0.06	0.00	0.12	0.01	0.06	0.02	0.00	0.02	0.00	0.00	0.15	0.10	0.13	0.00	0.00	0.02	0.02	0.00	0.00
TCGA-F4-6856	0.14	0.00	0.04	0.20	0.00	0.07	0.04	0.06	0.07	0.02	0.00	0.00	0.03	0.09	0.10	0.07	0.04	0.01	0.00	0.04	0.00	0.00

TCGA-AY-A54L	0.00	0.04	0.11	0.09	0.00	0.34	0.11	0.00	0.00	0.00	0.00	0.00	0.03	0.04	0.00	0.00	0.00	0.04	0.00	0.15	0.04	0.00
TCGA-AA-3538	0.03	0.02	0.04	0.03	0.00	0.13	0.08	0.00	0.00	0.00	0.05	0.00	0.00	0.17	0.04	0.13	0.08	0.02	0.00	0.10	0.00	0.08
TCGA-AA-3842	0.01	0.00	0.00	0.14	0.00	0.10	0.02	0.05	0.00	0.00	0.00	0.01	0.00	0.22	0.12	0.17	0.03	0.00	0.00	0.10	0.00	0.02
TCGA-AZ-4308	0.02	0.00	0.02	0.17	0.00	0.00	0.06	0.05	0.06	0.00	0.03	0.00	0.00	0.20	0.07	0.15	0.03	0.00	0.00	0.14	0.00	0.00
TCGA-AA-3509	0.02	0.03	0.01	0.17	0.00	0.10	0.10	0.02	0.00	0.00	0.00	0.00	0.00	0.08	0.09	0.17	0.06	0.03	0.00	0.05	0.03	0.05
TCGA-AA-3680	0.12	0.00	0.06	0.15	0.00	0.18	0.00	0.05	0.00	0.00	0.01	0.02	0.00	0.06	0.07	0.18	0.02	0.00	0.00	0.06	0.02	0.00
TCGA-AA-3542	0.00	0.03	0.11	0.15	0.01	0.00	0.04	0.00	0.02	0.00	0.05	0.00	0.00	0.15	0.05	0.11	0.07	0.00	0.00	0.18	0.01	0.02
TCGA-AD-6964	0.03	0.00	0.03	0.15	0.00	0.01	0.05	0.06	0.01	0.10	0.00	0.02	0.00	0.10	0.15	0.15	0.08	0.00	0.00	0.03	0.00	0.05
TCGA-A6-6650	0.00	0.03	0.12	0.00	0.00	0.41	0.00	0.02	0.00	0.00	0.03	0.00	0.02	0.07	0.09	0.05	0.00	0.02	0.00	0.14	0.01	0.00
TCGA-A6-6140	0.01	0.00	0.07	0.14	0.00	0.13	0.11	0.03	0.01	0.00	0.10	0.00	0.00	0.10	0.03	0.05	0.02	0.02	0.00	0.13	0.00	0.04
TCGA-AA-A024	0.04	0.00	0.11	0.07	0.00	0.20	0.00	0.06	0.02	0.00	0.01	0.03	0.02	0.20	0.05	0.08	0.03	0.00	0.00	0.07	0.00	0.00
TCGA-A6-2682	0.01	0.00	0.02	0.08	0.01	0.05	0.06	0.00	0.01	0.00	0.03	0.00	0.00	0.29	0.03	0.07	0.03	0.00	0.00	0.22	0.02	0.07
TCGA-CM-6675	0.00	0.06	0.07	0.09	0.00	0.18	0.02	0.13	0.02	0.00	0.01	0.01	0.00	0.02	0.11	0.11	0.09	0.02	0.02	0.04	0.00	0.01
TCGA-DM-A1D7	0.00	0.02	0.10	0.22	0.00	0.13	0.01	0.01	0.02	0.00	0.06	0.00	0.03	0.03	0.06	0.14	0.03	0.00	0.00	0.12	0.01	0.00
TCGA-4T-AA8H	0.00	0.02	0.24	0.16	0.00	0.23	0.01	0.00	0.01	0.00	0.07	0.00	0.00	0.02	0.00	0.06	0.05	0.04	0.00	0.06	0.05	0.00
TCGA-A6-6653	0.00	0.00	0.04	0.07	0.00	0.20	0.01	0.00	0.00	0.00	0.03	0.03	0.00	0.27	0.08	0.11	0.04	0.00	0.00	0.08	0.00	0.04
TCGA-AA-3666	0.00	0.00	0.07	0.04	0.00	0.17	0.07	0.03	0.00	0.00	0.05	0.00	0.00	0.14	0.10	0.15	0.02	0.01	0.00	0.09	0.00	0.05
TCGA-F4-6460	0.09	0.00	0.04	0.10	0.00	0.20	0.06	0.01	0.01	0.00	0.06	0.00	0.00	0.16	0.10	0.12	0.04	0.00	0.00	0.01	0.00	0.00
TCGA-AA-3655	0.02	0.01	0.04	0.04	0.00	0.15	0.00	0.00	0.09	0.00	0.00	0.00	0.00	0.18	0.15	0.17	0.04	0.04	0.00	0.07	0.00	0.00
TCGA-AA-3952	0.00	0.00	0.07	0.05	0.00	0.21	0.00	0.05	0.00	0.00	0.00	0.00	0.00	0.14	0.08	0.13	0.00	0.00	0.00	0.17	0.02	0.07
TCGA-T9-A92H	0.02	0.07	0.03	0.08	0.00	0.13	0.00	0.20	0.04	0.00	0.00	0.02	0.00	0.00	0.04	0.05	0.09	0.04	0.00	0.11	0.04	0.03
TCGA-AY-4070	0.11	0.00	0.12	0.12	0.00	0.15	0.02	0.09	0.02	0.00	0.03	0.00	0.00	0.08	0.07	0.06	0.03	0.02	0.00	0.05	0.00	0.00
TCGA-AY-A8YK	0.00	0.05	0.11	0.05	0.05	0.10	0.14	0.01	0.00	0.00	0.05	0.00	0.00	0.19	0.02	0.09	0.05	0.04	0.05	0.00	0.00	0.01
TCGA-CK-4950	0.02	0.00	0.11	0.17	0.00	0.10	0.08	0.04	0.00	0.01	0.02	0.00	0.00	0.10	0.04	0.09	0.08	0.01	0.01	0.05	0.04	0.03
TCGA-F4-6570	0.01	0.01	0.02	0.20	0.00	0.04	0.04	0.05	0.03	0.00	0.02	0.01	0.00	0.18	0.17	0.14	0.04	0.00	0.00	0.03	0.00	0.02
TCGA-AA-A00J	0.00	0.02	0.05	0.03	0.00	0.23	0.00	0.03	0.01	0.00	0.03	0.01	0.00	0.14	0.11	0.17	0.05	0.00	0.00	0.10	0.00	0.02
TCGA-AA-3667	0.02	0.00	0.04	0.15	0.00	0.00	0.11	0.02	0.01	0.00	0.10	0.00	0.00	0.14	0.06	0.12	0.03	0.00	0.00	0.14	0.01	0.06
TCGA-AA-3554	0.02	0.00	0.07	0.13	0.00	0.07	0.02	0.03	0.00	0.00	0.02	0.00	0.00	0.20	0.07	0.08	0.08	0.02	0.00	0.08	0.06	0.06
TCGA-AZ-6606	0.06	0.00	0.07	0.05	0.00	0.23	0.07	0.03	0.04	0.00	0.07	0.00	0.00	0.17	0.04	0.12	0.00	0.04	0.00	0.03	0.00	0.00
TCGA-D5-6922	0.00	0.05	0.04	0.04	0.00	0.05	0.01	0.01	0.03	0.00	0.02	0.00	0.00	0.20	0.18	0.24	0.04	0.00	0.00	0.08	0.00	0.00
TCGA-A6-6137	0.00	0.02	0.06	0.03	0.00	0.20	0.10	0.01	0.07	0.01	0.06	0.00	0.00	0.13	0.05	0.06	0.07	0.07	0.00	0.04	0.01	0.01
TCGA-AA-3495	0.00	0.00	0.22	0.11	0.00	0.11	0.12	0.00	0.00	0.00	0.00	0.04	0.00	0.02	0.06	0.20	0.03	0.00	0.02	0.04	0.00	0.04
TCGA-AA-3489	0.07	0.00	0.11	0.10	0.00	0.02	0.13	0.03	0.00	0.03	0.01	0.00	0.00	0.12	0.11	0.14	0.02	0.00	0.00	0.05	0.00	0.05
TCGA-G4-6322	0.04	0.00	0.07	0.09	0.00	0.20	0.01	0.02	0.03	0.00	0.06	0.00	0.00	0.15	0.05	0.09	0.01	0.02	0.00	0.15	0.03	0.00
TCGA-SS-A7HO	0.00	0.04	0.07	0.21	0.01	0.00	0.08	0.01	0.00	0.00	0.09	0.00	0.06	0.03	0.02	0.17	0.00	0.00	0.00	0.18	0.00	0.04
TCGA-AA-3976	0.07	0.00	0.02	0.11	0.00	0.14	0.19	0.01	0.00	0.01	0.04	0.00	0.00	0.00	0.11	0.17	0.06	0.00	0.02	0.02	0.00	0.03
TCGA-AA-3811	0.00	0.03	0.11	0.07	0.00	0.16	0.01	0.11	0.00	0.00	0.00	0.02	0.00	0.06	0.12	0.14	0.07	0.00	0.00	0.06	0.05	0.00
TCGA-AA-3819	0.07	0.00	0.12	0.06	0.00	0.13	0.06	0.04	0.01	0.00	0.02	0.00	0.00	0.04	0.01	0.12	0.12	0.05	0.05	0.01	0.04	0.03

TCGA-AA-A02Y	0.01	0.00	0.03	0.15	0.00	0.20	0.07	0.10	0.01	0.02	0.00	0.05	0.01	0.05	0.12	0.06	0.06	0.00	0.00	0.06	0.00	0.00
TCGA-AZ-6603	0.02	0.00	0.04	0.05	0.00	0.11	0.09	0.04	0.00	0.00	0.02	0.00	0.02	0.10	0.19	0.19	0.06	0.00	0.04	0.00	0.00	0.01
TCGA-G4-6626	0.05	0.00	0.03	0.17	0.00	0.10	0.12	0.06	0.00	0.00	0.02	0.00	0.03	0.11	0.10	0.02	0.04	0.00	0.00	0.10	0.05	0.00
TCGA-AA-3875	0.05	0.00	0.01	0.08	0.00	0.14	0.03	0.08	0.00	0.01	0.01	0.00	0.00	0.04	0.09	0.14	0.03	0.02	0.00	0.11	0.04	0.11
TCGA-G4-6306	0.01	0.06	0.02	0.22	0.00	0.17	0.03	0.05	0.01	0.04	0.00	0.00	0.00	0.11	0.10	0.00	0.07	0.04	0.00	0.08	0.00	0.00
TCGA-G4-6320	0.23	0.00	0.02	0.15	0.00	0.19	0.00	0.04	0.01	0.00	0.02	0.00	0.00	0.10	0.10	0.01	0.09	0.00	0.00	0.03	0.00	0.00
TCGA-AA-3864	0.05	0.00	0.02	0.03	0.00	0.18	0.00	0.10	0.00	0.00	0.00	0.00	0.00	0.12	0.09	0.14	0.03	0.03	0.00	0.14	0.01	0.05
TCGA-A6-6142	0.04	0.00	0.04	0.02	0.00	0.09	0.00	0.03	0.01	0.00	0.00	0.00	0.00	0.30	0.10	0.11	0.00	0.02	0.00	0.18	0.00	0.05
TCGA-A6-2678	0.00	0.00	0.06	0.11	0.00	0.17	0.04	0.03	0.02	0.00	0.00	0.03	0.00	0.10	0.11	0.18	0.05	0.00	0.00	0.08	0.00	0.02
TCGA-CA-5796	0.00	0.02	0.11	0.14	0.00	0.34	0.00	0.00	0.03	0.00	0.00	0.00	0.00	0.08	0.04	0.10	0.08	0.03	0.00	0.03	0.00	0.00
TCGA-A6-A567	0.09	0.00	0.08	0.10	0.00	0.12	0.00	0.12	0.00	0.00	0.00	0.00	0.00	0.16	0.09	0.11	0.08	0.00	0.02	0.03	0.00	0.00
TCGA-AA-3713	0.08	0.00	0.05	0.10	0.00	0.13	0.07	0.06	0.00	0.00	0.06	0.00	0.00	0.10	0.11	0.13	0.03	0.00	0.00	0.04	0.04	0.01
TCGA-A6-6648	0.00	0.00	0.14	0.15	0.00	0.19	0.08	0.00	0.00	0.00	0.00	0.00	0.00	0.06	0.08	0.07	0.10	0.01	0.07	0.03	0.02	0.00
TCGA-A6-A565	0.12	0.00	0.09	0.09	0.04	0.12	0.01	0.07	0.00	0.00	0.04	0.00	0.00	0.09	0.08	0.12	0.03	0.00	0.02	0.03	0.01	0.03
TCGA-A6-5660	0.00	0.02	0.02	0.09	0.00	0.10	0.10	0.00	0.00	0.00	0.04	0.00	0.00	0.24	0.02	0.17	0.06	0.03	0.00	0.07	0.03	0.02
TCGA-AZ-6601	0.09	0.00	0.02	0.11	0.00	0.02	0.05	0.06	0.02	0.02	0.02	0.00	0.00	0.18	0.14	0.15	0.04	0.00	0.00	0.08	0.00	0.01
TCGA-5M-AATE	0.02	0.00	0.08	0.10	0.00	0.19	0.01	0.00	0.05	0.00	0.03	0.00	0.00	0.18	0.04	0.15	0.04	0.00	0.00	0.08	0.00	0.00
TCGA-CM-6161	0.01	0.01	0.03	0.18	0.00	0.10	0.14	0.05	0.00	0.00	0.04	0.00	0.00	0.08	0.04	0.15	0.09	0.02	0.01	0.02	0.04	0.01
TCGA-AZ-4313	0.00	0.00	0.02	0.25	0.00	0.05	0.01	0.07	0.05	0.00	0.06	0.00	0.00	0.13	0.06	0.03	0.05	0.02	0.00	0.19	0.00	0.01
TCGA-A6-6652	0.00	0.00	0.02	0.08	0.00	0.14	0.07	0.00	0.03	0.00	0.08	0.00	0.00	0.46	0.00	0.02	0.00	0.04	0.00	0.05	0.00	0.00
TCGA-AA-A00E	0.08	0.00	0.01	0.11	0.00	0.08	0.09	0.05	0.00	0.00	0.06	0.01	0.00	0.16	0.11	0.12	0.09	0.00	0.00	0.03	0.00	0.00
TCGA-F4-6808	0.05	0.00	0.05	0.11	0.00	0.18	0.13	0.00	0.01	0.00	0.04	0.00	0.00	0.22	0.04	0.03	0.00	0.02	0.00	0.08	0.01	0.03
TCGA-AA-A02W	0.01	0.03	0.02	0.18	0.00	0.12	0.10	0.11	0.00	0.00	0.00	0.02	0.00	0.05	0.09	0.12	0.05	0.03	0.06	0.00	0.00	0.00
TCGA-DM-A28C	0.00	0.11	0.05	0.04	0.00	0.25	0.00	0.00	0.01	0.00	0.05	0.00	0.00	0.29	0.06	0.06	0.02	0.00	0.00	0.06	0.00	0.00
TCGA-AA-3681	0.02	0.00	0.05	0.16	0.00	0.09	0.09	0.05	0.00	0.00	0.02	0.00	0.00	0.12	0.09	0.13	0.06	0.00	0.00	0.05	0.04	0.03
TCGA-AA-3492	0.01	0.00	0.08	0.12	0.00	0.07	0.01	0.04	0.01	0.00	0.00	0.03	0.03	0.06	0.10	0.12	0.14	0.00	0.00	0.18	0.00	0.00
TCGA-D5-6535	0.00	0.03	0.09	0.10	0.00	0.09	0.04	0.07	0.04	0.02	0.01	0.00	0.00	0.16	0.12	0.10	0.04	0.02	0.00	0.05	0.00	0.03
TCGA-AD-6963	0.03	0.00	0.03	0.23	0.00	0.03	0.16	0.03	0.00	0.00	0.01	0.00	0.00	0.15	0.10	0.07	0.02	0.01	0.00	0.07	0.00	0.06
TCGA-CM-4747	0.01	0.00	0.11	0.16	0.00	0.03	0.00	0.01	0.11	0.00	0.03	0.00	0.00	0.23	0.04	0.09	0.03	0.00	0.00	0.13	0.00	0.01
TCGA-NH-A50T	0.00	0.04	0.08	0.04	0.00	0.09	0.00	0.00	0.06	0.00	0.09	0.00	0.00	0.16	0.04	0.11	0.00	0.05	0.00	0.13	0.03	0.09
TCGA-CM-6168	0.06	0.00	0.02	0.00	0.00	0.14	0.00	0.06	0.00	0.00	0.05	0.00	0.00	0.13	0.10	0.15	0.08	0.01	0.00	0.12	0.03	0.06
TCGA-AA-A01T	0.02	0.00	0.07	0.21	0.00	0.00	0.10	0.03	0.05	0.00	0.05	0.00	0.01	0.15	0.12	0.02	0.01	0.00	0.00	0.15	0.02	0.00
TCGA-RU-A8FL	0.00	0.16	0.06	0.14	0.00	0.11	0.14	0.05	0.02	0.00	0.04	0.00	0.02	0.15	0.01	0.01	0.04	0.00	0.00	0.03	0.01	0.00
TCGA-AA-3812	0.02	0.00	0.06	0.05	0.00	0.03	0.00	0.03	0.04	0.00	0.03	0.00	0.00	0.33	0.03	0.10	0.09	0.00	0.00	0.17	0.00	0.02
TCGA-AY-A69D	0.13	0.00	0.11	0.00	0.00	0.22	0.10	0.05	0.00	0.00	0.05	0.00	0.00	0.08	0.04	0.05	0.00	0.06	0.00	0.10	0.02	0.00
TCGA-A6-3810	0.00	0.03	0.03	0.00	0.00	0.26	0.01	0.05	0.00	0.01	0.00	0.00	0.00	0.13	0.14	0.09	0.01	0.00	0.00	0.14	0.02	0.08
TCGA-DM-A28H	0.00	0.04	0.12	0.13	0.00	0.09	0.10	0.02	0.00	0.00	0.05	0.00	0.02	0.06	0.06	0.18	0.03	0.00	0.00	0.09	0.00	0.00
TCGA-G4-6586	0.05	0.00	0.01	0.26	0.00	0.00	0.16	0.11	0.01	0.01	0.04	0.02	0.00	0.08	0.08	0.10	0.02	0.00	0.00	0.06	0.00	0.00

TCGA-D5-6928	0.02	0.02	0.10	0.13	0.00	0.00	0.14	0.03	0.02	0.04	0.06	0.00	0.00	0.12	0.13	0.08	0.07	0.00	0.00	0.02	0.00	0.03
TCGA-G4-6315	0.20	0.00	0.09	0.11	0.00	0.15	0.02	0.12	0.00	0.01	0.00	0.05	0.00	0.06	0.04	0.07	0.00	0.01	0.06	0.00	0.00	0.00
TCGA-AA-3664	0.00	0.00	0.08	0.05	0.00	0.20	0.00	0.13	0.02	0.00	0.00	0.01	0.01	0.06	0.09	0.15	0.00	0.03	0.00	0.15	0.01	0.00
TCGA-G4-6303	0.00	0.01	0.08	0.09	0.00	0.13	0.08	0.00	0.00	0.00	0.02	0.00	0.00	0.21	0.05	0.10	0.05	0.05	0.00	0.10	0.00	0.03
TCGA-AA-3692	0.01	0.01	0.02	0.10	0.00	0.08	0.09	0.02	0.00	0.00	0.01	0.00	0.02	0.07	0.03	0.13	0.13	0.00	0.00	0.19	0.05	0.04
TCGA-AA-3968	0.04	0.00	0.07	0.08	0.00	0.11	0.09	0.04	0.01	0.00	0.02	0.00	0.00	0.10	0.09	0.16	0.08	0.00	0.00	0.07	0.03	0.00
TCGA-AA-3560	0.08	0.00	0.02	0.13	0.00	0.19	0.05	0.02	0.03	0.00	0.01	0.00	0.02	0.06	0.06	0.11	0.08	0.00	0.00	0.08	0.04	0.01
TCGA-D5-5537	0.00	0.00	0.10	0.08	0.00	0.24	0.09	0.01	0.03	0.00	0.06	0.00	0.01	0.06	0.05	0.14	0.07	0.00	0.00	0.08	0.00	0.00
TCGA-D5-6932	0.01	0.00	0.01	0.13	0.00	0.07	0.06	0.03	0.01	0.00	0.03	0.00	0.00	0.22	0.09	0.13	0.04	0.02	0.00	0.10	0.02	0.03
TCGA-AA-3982	0.01	0.02	0.03	0.10	0.00	0.10	0.05	0.03	0.02	0.07	0.00	0.00	0.00	0.07	0.11	0.18	0.11	0.00	0.01	0.03	0.04	0.00
TCGA-DM-A28A	0.00	0.02	0.02	0.19	0.00	0.03	0.04	0.03	0.02	0.00	0.00	0.02	0.00	0.14	0.17	0.23	0.00	0.00	0.00	0.08	0.00	0.01
TCGA-CM-6172	0.00	0.00	0.09	0.04	0.00	0.21	0.02	0.00	0.01	0.00	0.03	0.00	0.00	0.17	0.02	0.15	0.05	0.02	0.10	0.02	0.03	0.03
TCGA-D5-6930	0.13	0.00	0.04	0.14	0.00	0.07	0.05	0.06	0.00	0.00	0.01	0.02	0.00	0.16	0.12	0.11	0.03	0.00	0.00	0.05	0.00	0.00
TCGA-D5-6529	0.00	0.01	0.16	0.10	0.00	0.07	0.06	0.05	0.02	0.00	0.03	0.00	0.00	0.11	0.10	0.09	0.07	0.00	0.00	0.07	0.01	0.05
TCGA-A6-2683	0.00	0.01	0.06	0.05	0.00	0.09	0.00	0.10	0.12	0.00	0.04	0.00	0.00	0.12	0.02	0.14	0.13	0.00	0.00	0.12	0.00	0.00
TCGA-CK-5913	0.00	0.00	0.01	0.10	0.00	0.17	0.02	0.07	0.00	0.00	0.04	0.01	0.00	0.12	0.11	0.05	0.10	0.01	0.00	0.13	0.00	0.07
TCGA-D5-5540	0.02	0.00	0.00	0.12	0.00	0.00	0.18	0.08	0.00	0.06	0.00	0.04	0.00	0.07	0.12	0.12	0.03	0.00	0.00	0.13	0.02	0.02
TCGA-AD-6901	0.01	0.00	0.02	0.11	0.00	0.02	0.06	0.03	0.00	0.06	0.00	0.02	0.00	0.19	0.15	0.20	0.06	0.00	0.00	0.04	0.00	0.01
TCGA-AA-A01F	0.00	0.03	0.05	0.00	0.00	0.34	0.16	0.00	0.02	0.04	0.00	0.06	0.00	0.13	0.05	0.09	0.01	0.00	0.00	0.01	0.00	0.00
TCGA-AA-3552	0.09	0.00	0.08	0.07	0.00	0.10	0.07	0.05	0.01	0.00	0.08	0.00	0.00	0.07	0.08	0.13	0.05	0.00	0.00	0.08	0.01	0.02
TCGA-F4-6855	0.00	0.00	0.06	0.05	0.00	0.12	0.00	0.03	0.03	0.00	0.00	0.04	0.01	0.21	0.07	0.20	0.10	0.00	0.00	0.05	0.02	0.02
TCGA-AD-5900	0.13	0.00	0.02	0.18	0.00	0.03	0.06	0.05	0.01	0.00	0.01	0.02	0.00	0.12	0.13	0.14	0.03	0.01	0.04	0.00	0.00	0.00
TCGA-AA-3979	0.02	0.00	0.05	0.08	0.00	0.11	0.12	0.03	0.00	0.00	0.03	0.00	0.00	0.14	0.05	0.14	0.01	0.02	0.00	0.09	0.00	0.09
TCGA-AA-A01I	0.00	0.02	0.11	0.12	0.00	0.18	0.11	0.06	0.00	0.00	0.00	0.01	0.00	0.06	0.13	0.08	0.06	0.02	0.00	0.05	0.00	0.00
TCGA-AA-A03F	0.00	0.01	0.21	0.12	0.00	0.06	0.00	0.01	0.11	0.00	0.09	0.00	0.00	0.15	0.02	0.04	0.05	0.01	0.00	0.12	0.00	0.00
TCGA-3L-AA1B	0.08	0.00	0.13	0.07	0.00	0.11	0.07	0.09	0.00	0.00	0.02	0.00	0.01	0.12	0.00	0.10	0.05	0.03	0.08	0.02	0.00	0.00
TCGA-AA-3502	0.01	0.00	0.11	0.19	0.00	0.15	0.00	0.04	0.03	0.00	0.03	0.00	0.01	0.00	0.04	0.13	0.12	0.01	0.00	0.09	0.06	0.00
TCGA-AA-A02E	0.00	0.00	0.10	0.16	0.00	0.17	0.00	0.01	0.07	0.00	0.05	0.00	0.02	0.16	0.06	0.04	0.06	0.00	0.00	0.08	0.02	0.00
TCGA-CM-4746	0.00	0.01	0.02	0.34	0.00	0.00	0.09	0.04	0.06	0.00	0.00	0.04	0.00	0.12	0.05	0.10	0.00	0.05	0.00	0.07	0.00	0.01
TCGA-G4-6295	0.06	0.00	0.04	0.23	0.00	0.00	0.12	0.08	0.00	0.02	0.04	0.00	0.00	0.05	0.10	0.07	0.11	0.02	0.00	0.04	0.00	0.00
TCGA-AA-A01C	0.00	0.02	0.02	0.11	0.00	0.11	0.16	0.08	0.00	0.00	0.00	0.02	0.00	0.14	0.12	0.14	0.04	0.00	0.06	0.00	0.00	0.00
TCGA-G4-6588	0.00	0.00	0.05	0.06	0.00	0.16	0.02	0.01	0.00	0.00	0.05	0.01	0.00	0.18	0.10	0.08	0.00	0.01	0.00	0.22	0.00	0.06
TCGA-A6-2680	0.02	0.00	0.10	0.02	0.00	0.08	0.00	0.05	0.01	0.00	0.09	0.00	0.00	0.18	0.08	0.13	0.05	0.04	0.00	0.12	0.03	0.00
TCGA-QG-A5YV	0.01	0.07	0.03	0.04	0.00	0.20	0.15	0.00	0.05	0.00	0.06	0.00	0.00	0.16	0.07	0.00	0.04	0.01	0.00	0.10	0.00	0.01
TCGA-AA-3561	0.02	0.03	0.02	0.10	0.00	0.22	0.12	0.00	0.00	0.00	0.03	0.00	0.03	0.01	0.10	0.18	0.02	0.00	0.00	0.07	0.00	0.06
TCGA-AA-3525	0.10	0.00	0.13	0.06	0.00	0.14	0.00	0.05	0.00	0.00	0.00	0.00	0.00	0.12	0.11	0.10	0.00	0.00	0.00	0.09	0.05	0.05
TCGA-AA-3531	0.00	0.01	0.08	0.25	0.00	0.00	0.03	0.01	0.08	0.00	0.05	0.00	0.02	0.22	0.09	0.10	0.05	0.00	0.00	0.02	0.00	0.00
TCGA-AA-3973	0.07	0.01	0.01	0.16	0.00	0.08	0.08	0.04	0.03	0.00	0.02	0.00	0.01	0.25	0.07	0.11	0.03	0.00	0.04	0.00	0.00	0.00

TCGA-AA-3555	0.01	0.00	0.00	0.00	0.00	0.18	0.00	0.02	0.00	0.00	0.03	0.00	0.00	0.18	0.15	0.10	0.01	0.01	0.00	0.14	0.01	0.17
TCGA-EI-6884	0.00	0.04	0.09	0.15	0.00	0.15	0.02	0.03	0.00	0.00	0.03	0.00	0.00	0.11	0.09	0.10	0.10	0.00	0.02	0.03	0.02	0.02
TCGA-AG-3582	0.03	0.00	0.08	0.07	0.00	0.09	0.09	0.00	0.01	0.00	0.02	0.00	0.00	0.15	0.13	0.15	0.01	0.00	0.00	0.14	0.00	0.03
TCGA-DC-6154	0.01	0.00	0.08	0.08	0.00	0.11	0.05	0.00	0.03	0.00	0.08	0.00	0.00	0.15	0.03	0.20	0.10	0.00	0.00	0.03	0.00	0.04
TCGA-AG-3611	0.00	0.00	0.16	0.06	0.00	0.18	0.02	0.01	0.01	0.00	0.05	0.00	0.00	0.11	0.04	0.12	0.11	0.00	0.00	0.03	0.06	0.03
TCGA-AG-3609	0.00	0.05	0.02	0.17	0.00	0.04	0.09	0.01	0.02	0.00	0.02	0.00	0.00	0.17	0.05	0.12	0.06	0.03	0.00	0.12	0.00	0.04
TCGA-AG-4022	0.04	0.00	0.05	0.12	0.00	0.12	0.07	0.02	0.00	0.00	0.02	0.00	0.00	0.16	0.10	0.09	0.09	0.03	0.02	0.00	0.05	0.02
TCGA-AG-A02N	0.00	0.04	0.03	0.13	0.00	0.15	0.12	0.05	0.00	0.02	0.00	0.06	0.00	0.06	0.09	0.08	0.04	0.00	0.13	0.00	0.00	0.00
TCGA-AH-6549	0.03	0.00	0.03	0.05	0.00	0.33	0.06	0.00	0.00	0.00	0.04	0.00	0.03	0.04	0.16	0.15	0.03	0.00	0.01	0.00	0.00	0.02
TCGA-AH-6903	0.00	0.03	0.13	0.09	0.00	0.32	0.09	0.00	0.00	0.00	0.02	0.00	0.02	0.04	0.02	0.06	0.04	0.05	0.00	0.08	0.00	0.00
TCGA-F5-6863	0.00	0.05	0.10	0.04	0.00	0.17	0.03	0.00	0.03	0.00	0.04	0.00	0.00	0.30	0.04	0.12	0.04	0.03	0.00	0.00	0.00	0.00
TCGA-AG-3887	0.03	0.00	0.06	0.25	0.00	0.09	0.15	0.03	0.01	0.00	0.00	0.02	0.00	0.09	0.08	0.11	0.03	0.00	0.00	0.04	0.00	0.00
TCGA-AF-5654	0.00	0.01	0.07	0.00	0.00	0.19	0.20	0.02	0.00	0.00	0.07	0.00	0.01	0.08	0.05	0.07	0.00	0.05	0.00	0.16	0.01	0.00
TCGA-DY-A1DE	0.01	0.01	0.05	0.11	0.00	0.00	0.04	0.06	0.05	0.01	0.01	0.00	0.00	0.24	0.12	0.17	0.02	0.01	0.00	0.08	0.00	0.00
TCGA-AF-3913	0.03	0.00	0.02	0.03	0.00	0.16	0.08	0.00	0.00	0.00	0.06	0.00	0.02	0.14	0.02	0.13	0.07	0.03	0.00	0.13	0.02	0.06
TCGA-AG-A014	0.00	0.08	0.08	0.00	0.04	0.19	0.00	0.00	0.00	0.00	0.07	0.00	0.03	0.14	0.03	0.00	0.03	0.06	0.00	0.20	0.00	0.04
TCGA-G5-6572	0.06	0.00	0.02	0.11	0.00	0.15	0.08	0.00	0.00	0.01	0.00	0.00	0.03	0.17	0.08	0.15	0.08	0.00	0.06	0.00	0.00	0.01
TCGA-AG-A011	0.10	0.00	0.17	0.10	0.00	0.10	0.00	0.04	0.00	0.00	0.00	0.04	0.02	0.15	0.06	0.14	0.01	0.00	0.00	0.06	0.00	0.00
TCGA-EI-6511	0.00	0.00	0.14	0.15	0.00	0.03	0.11	0.00	0.02	0.01	0.04	0.00	0.00	0.16	0.14	0.12	0.00	0.01	0.00	0.03	0.00	0.03
TCGA-AG-4008	0.05	0.02	0.02	0.10	0.00	0.00	0.09	0.04	0.00	0.00	0.09	0.00	0.00	0.11	0.15	0.20	0.06	0.00	0.01	0.05	0.00	0.04
TCGA-AG-3731	0.05	0.00	0.06	0.10	0.00	0.14	0.06	0.06	0.00	0.00	0.00	0.01	0.00	0.10	0.16	0.15	0.01	0.01	0.00	0.03	0.00	0.05
TCGA-AH-6544	0.19	0.07	0.12	0.02	0.00	0.19	0.00	0.08	0.00	0.00	0.00	0.02	0.00	0.19	0.03	0.01	0.00	0.06	0.00	0.02	0.00	0.00
TCGA-AG-A026	0.02	0.00	0.04	0.25	0.00	0.05	0.05	0.09	0.00	0.00	0.00	0.00	0.00	0.11	0.08	0.16	0.00	0.00	0.00	0.09	0.04	0.02
TCGA-EI-7004	0.08	0.00	0.00	0.07	0.00	0.17	0.00	0.01	0.00	0.00	0.02	0.00	0.00	0.21	0.06	0.22	0.02	0.01	0.00	0.07	0.00	0.05
TCGA-DC-4745	0.00	0.01	0.06	0.16	0.00	0.15	0.15	0.02	0.00	0.00	0.00	0.00	0.00	0.11	0.06	0.14	0.05	0.01	0.00	0.05	0.01	0.00
TCGA-EI-6883	0.08	0.00	0.06	0.13	0.00	0.05	0.12	0.03	0.00	0.03	0.03	0.00	0.00	0.22	0.10	0.10	0.03	0.00	0.00	0.03	0.00	0.00
TCGA-EI-6917	0.06	0.00	0.03	0.08	0.00	0.02	0.05	0.05	0.03	0.00	0.01	0.00	0.00	0.20	0.19	0.16	0.08	0.00	0.00	0.02	0.01	0.03
TCGA-F5-6861	0.00	0.04	0.03	0.10	0.00	0.18	0.02	0.04	0.00	0.00	0.02	0.00	0.00	0.22	0.10	0.14	0.04	0.00	0.00	0.06	0.00	0.00
TCGA-F5-6465	0.03	0.00	0.04	0.09	0.00	0.13	0.11	0.04	0.00	0.02	0.02	0.00	0.00	0.14	0.08	0.09	0.05	0.02	0.00	0.07	0.03	0.04
TCGA-AF-6672	0.14	0.00	0.01	0.14	0.00	0.28	0.00	0.08	0.00	0.00	0.00	0.00	0.00	0.01	0.03	0.09	0.09	0.03	0.00	0.09	0.00	0.00
TCGA-AG-3885	0.05	0.00	0.09	0.11	0.00	0.05	0.01	0.05	0.06	0.00	0.09	0.00	0.00	0.15	0.05	0.10	0.04	0.00	0.00	0.11	0.00	0.02
TCGA-EI-6509	0.00	0.03	0.02	0.00	0.00	0.02	0.00	0.00	0.11	0.00	0.02	0.00	0.00	0.59	0.05	0.12	0.00	0.00	0.00	0.05	0.00	0.00
TCGA-DC-5337	0.01	0.00	0.02	0.08	0.00	0.24	0.16	0.04	0.00	0.05	0.02	0.00	0.00	0.06	0.10	0.10	0.05	0.02	0.00	0.05	0.01	0.00
TCGA-AG-3893	0.01	0.00	0.01	0.09	0.00	0.03	0.06	0.02	0.00	0.00	0.09	0.00	0.00	0.19	0.16	0.15	0.08	0.00	0.00	0.07	0.02	0.02
TCGA-AG-3901	0.01	0.00	0.06	0.00	0.00	0.18	0.05	0.01	0.01	0.01	0.04	0.00	0.00	0.19	0.13	0.15	0.04	0.00	0.00	0.06	0.00	0.04
TCGA-AG-3726	0.06	0.00	0.05	0.09	0.04	0.00	0.16	0.00	0.02	0.00	0.10	0.00	0.00	0.20	0.03	0.02	0.07	0.05	0.00	0.11	0.00	0.00
TCGA-DC-6160	0.03	0.00	0.10	0.09	0.00	0.22	0.02	0.03	0.03	0.00	0.05	0.00	0.00	0.12	0.06	0.11	0.00	0.05	0.00	0.08	0.00	0.00
TCGA-AG-3581	0.01	0.00	0.18	0.05	0.00	0.10	0.04	0.00	0.03	0.00	0.08	0.00	0.00	0.12	0.06	0.13	0.03	0.00	0.00	0.10	0.01	0.05

TCGA-F5-6812	0.04	0.05	0.02	0.00	0.00	0.15	0.04	0.07	0.02	0.08	0.00	0.00	0.00	0.14	0.18	0.17	0.01	0.00	0.00	0.04	0.00	0.00
TCGA-AG-4001	0.00	0.00	0.06	0.12	0.00	0.09	0.03	0.01	0.04	0.00	0.02	0.00	0.00	0.23	0.11	0.17	0.05	0.00	0.00	0.04	0.00	0.03
TCGA-AG-3909	0.08	0.00	0.09	0.09	0.00	0.02	0.01	0.03	0.05	0.02	0.06	0.00	0.00	0.13	0.07	0.15	0.07	0.00	0.00	0.10	0.04	0.00
TCGA-AG-3592	0.01	0.01	0.09	0.04	0.00	0.19	0.01	0.02	0.01	0.02	0.04	0.00	0.00	0.11	0.12	0.14	0.02	0.00	0.00	0.14	0.00	0.02
TCGA-EI-6513	0.00	0.00	0.07	0.20	0.00	0.03	0.05	0.09	0.02	0.00	0.00	0.01	0.00	0.21	0.11	0.11	0.04	0.01	0.00	0.03	0.00	0.01
TCGA-DC-6158	0.04	0.00	0.03	0.09	0.00	0.10	0.15	0.04	0.00	0.00	0.04	0.00	0.00	0.16	0.11	0.12	0.04	0.01	0.00	0.04	0.00	0.03
TCGA-EI-6512	0.00	0.05	0.02	0.06	0.00	0.14	0.02	0.07	0.00	0.04	0.00	0.00	0.00	0.11	0.14	0.18	0.04	0.01	0.00	0.07	0.00	0.06
TCGA-CI-6622	0.00	0.02	0.08	0.08	0.00	0.22	0.11	0.00	0.00	0.00	0.02	0.00	0.00	0.14	0.05	0.16	0.04	0.00	0.02	0.01	0.01	0.03
TCGA-BM-6198	0.06	0.00	0.08	0.08	0.00	0.10	0.09	0.05	0.00	0.02	0.02	0.00	0.00	0.12	0.12	0.08	0.00	0.01	0.00	0.14	0.00	0.04
TCGA-DY-A1DC	0.02	0.03	0.10	0.14	0.00	0.09	0.14	0.03	0.01	0.00	0.00	0.00	0.00	0.09	0.10	0.10	0.12	0.00	0.00	0.04	0.00	0.00
TCGA-AG-3600	0.01	0.00	0.08	0.14	0.00	0.08	0.01	0.05	0.01	0.03	0.00	0.00	0.00	0.12	0.05	0.18	0.09	0.01	0.00	0.08	0.01	0.05
TCGA-AG-3599	0.05	0.00	0.01	0.23	0.00	0.06	0.01	0.04	0.02	0.00	0.00	0.05	0.00	0.11	0.12	0.17	0.06	0.00	0.00	0.05	0.01	0.00
TCGA-AG-3584	0.04	0.02	0.06	0.08	0.00	0.07	0.01	0.00	0.06	0.00	0.02	0.00	0.00	0.25	0.06	0.19	0.06	0.00	0.02	0.04	0.00	0.00
TCGA-DT-5265	0.00	0.03	0.03	0.10	0.00	0.02	0.00	0.03	0.02	0.00	0.00	0.01	0.00	0.27	0.02	0.23	0.10	0.02	0.00	0.07	0.00	0.03
TCGA-AG-A036	0.07	0.00	0.07	0.13	0.00	0.01	0.09	0.07	0.00	0.00	0.02	0.00	0.00	0.23	0.11	0.11	0.02	0.01	0.00	0.05	0.00	0.00
TCGA-AG-3593	0.00	0.00	0.06	0.06	0.00	0.16	0.09	0.03	0.03	0.00	0.07	0.00	0.00	0.08	0.05	0.12	0.11	0.02	0.00	0.07	0.02	0.03
TCGA-AG-3580	0.00	0.00	0.12	0.13	0.00	0.07	0.08	0.06	0.00	0.00	0.07	0.00	0.00	0.09	0.05	0.08	0.02	0.03	0.00	0.11	0.02	0.06
TCGA-DC-6683	0.06	0.00	0.01	0.05	0.00	0.15	0.01	0.09	0.03	0.00	0.01	0.00	0.00	0.33	0.11	0.09	0.04	0.00	0.02	0.00	0.00	0.00
TCGA-AF-6655	0.03	0.00	0.04	0.00	0.00	0.14	0.03	0.04	0.00	0.00	0.05	0.00	0.00	0.20	0.02	0.11	0.00	0.08	0.00	0.20	0.00	0.04
TCGA-AG-3728	0.07	0.00	0.05	0.18	0.00	0.01	0.10	0.01	0.04	0.00	0.09	0.00	0.00	0.11	0.08	0.14	0.06	0.00	0.00	0.06	0.00	0.00
TCGA-AF-3400	0.05	0.00	0.01	0.02	0.00	0.11	0.03	0.01	0.00	0.00	0.03	0.01	0.01	0.19	0.06	0.10	0.06	0.02	0.00	0.13	0.04	0.12
TCGA-AF-2691	0.07	0.00	0.02	0.11	0.00	0.17	0.02	0.06	0.00	0.04	0.00	0.03	0.00	0.15	0.08	0.14	0.03	0.00	0.00	0.05	0.00	0.02
TCGA-CL-5918	0.03	0.00	0.01	0.00	0.00	0.27	0.14	0.03	0.00	0.01	0.15	0.04	0.01	0.11	0.08	0.01	0.05	0.02	0.02	0.00	0.00	0.03
TCGA-F5-6864	0.01	0.00	0.07	0.18	0.00	0.13	0.01	0.05	0.00	0.00	0.00	0.00	0.00	0.13	0.12	0.18	0.05	0.00	0.00	0.07	0.00	0.00
TCGA-AG-A01N	0.04	0.00	0.04	0.10	0.00	0.08	0.08	0.00	0.02	0.00	0.05	0.00	0.00	0.35	0.02	0.02	0.07	0.00	0.00	0.11	0.00	0.02
TCGA-DC-5869	0.00	0.03	0.04	0.10	0.00	0.14	0.10	0.06	0.00	0.00	0.03	0.00	0.00	0.04	0.05	0.11	0.10	0.05	0.00	0.07	0.07	0.00
TCGA-AG-3608	0.00	0.02	0.03	0.14	0.00	0.06	0.11	0.06	0.01	0.05	0.00	0.00	0.00	0.05	0.08	0.15	0.12	0.01	0.00	0.05	0.03	0.03
TCGA-DY-A0XA	0.00	0.05	0.07	0.18	0.00	0.15	0.03	0.06	0.00	0.00	0.00	0.01	0.00	0.21	0.05	0.12	0.00	0.00	0.00	0.05	0.00	0.01
TCGA-AG-3594	0.05	0.00	0.01	0.17	0.00	0.00	0.07	0.13	0.01	0.09	0.00	0.00	0.00	0.03	0.14	0.12	0.02	0.01	0.00	0.05	0.01	0.10
TCGA-EF-5831	0.05	0.00	0.05	0.09	0.00	0.13	0.09	0.05	0.00	0.00	0.00	0.00	0.00	0.22	0.09	0.15	0.07	0.00	0.00	0.02	0.00	0.01
TCGA-AG-3725	0.02	0.04	0.06	0.04	0.00	0.19	0.00	0.04	0.03	0.04	0.02	0.00	0.01	0.14	0.10	0.09	0.02	0.00	0.00	0.17	0.00	0.00
TCGA-EI-6514	0.00	0.01	0.02	0.05	0.00	0.11	0.11	0.00	0.00	0.00	0.04	0.00	0.00	0.38	0.05	0.14	0.07	0.00	0.02	0.01	0.00	0.00
TCGA-CI-6624	0.02	0.02	0.07	0.10	0.00	0.13	0.03	0.03	0.03	0.01	0.03	0.00	0.00	0.13	0.08	0.11	0.06	0.03	0.07	0.01	0.00	0.02
TCGA-CI-6620	0.02	0.04	0.07	0.16	0.00	0.14	0.00	0.03	0.00	0.00	0.04	0.00	0.00	0.09	0.02	0.12	0.09	0.02	0.02	0.06	0.01	0.06
TCGA-CI-6621	0.01	0.00	0.03	0.11	0.00	0.04	0.15	0.01	0.00	0.00	0.03	0.00	0.00	0.21	0.08	0.14	0.03	0.02	0.00	0.05	0.00	0.07
TCGA-EI-6510	0.02	0.00	0.19	0.17	0.00	0.16	0.05	0.06	0.05	0.00	0.01	0.00	0.00	0.08	0.04	0.04	0.05	0.03	0.00	0.07	0.00	0.00
TCGA-AG-A02X	0.11	0.00	0.05	0.03	0.00	0.17	0.00	0.07	0.05	0.00	0.00	0.00	0.00	0.21	0.03	0.12	0.00	0.04	0.00	0.11	0.00	0.00
TCGA-AG-3894	0.00	0.01	0.09	0.13	0.00	0.15	0.06	0.00	0.00	0.00	0.07	0.00	0.00	0.08	0.05	0.15	0.05	0.01	0.07	0.01	0.03	0.02

TCGA-AF-A56N	0.03	0.00	0.10	0.04	0.00	0.16	0.16	0.03	0.00	0.00	0.00	0.00	0.00	0.18	0.02	0.10	0.03	0.04	0.00	0.08	0.00	0.01
TCGA-AG-A00C	0.01	0.01	0.16	0.18	0.00	0.13	0.20	0.01	0.00	0.00	0.06	0.00	0.00	0.12	0.04	0.00	0.04	0.04	0.01	0.00	0.00	0.00
TCGA-EI-6885	0.04	0.00	0.02	0.07	0.00	0.03	0.07	0.05	0.01	0.02	0.02	0.00	0.00	0.25	0.07	0.08	0.03	0.03	0.00	0.20	0.01	0.03
TCGA-AG-3732	0.12	0.00	0.11	0.12	0.00	0.15	0.03	0.07	0.01	0.03	0.02	0.00	0.00	0.05	0.10	0.09	0.00	0.01	0.04	0.04	0.00	0.00
TCGA-AG-A016	0.03	0.00	0.08	0.16	0.05	0.09	0.00	0.00	0.00	0.00	0.07	0.02	0.07	0.12	0.01	0.07	0.05	0.02	0.00	0.15	0.00	0.00
TCGA-EI-6882	0.01	0.02	0.10	0.00	0.00	0.23	0.02	0.01	0.00	0.07	0.02	0.00	0.00	0.15	0.10	0.11	0.00	0.01	0.00	0.11	0.00	0.04
TCGA-AG-3575	0.00	0.01	0.01	0.14	0.00	0.08	0.13	0.05	0.00	0.00	0.00	0.02	0.02	0.01	0.12	0.11	0.09	0.05	0.00	0.05	0.06	0.05
TCGA-AF-2693	0.03	0.00	0.08	0.09	0.00	0.20	0.02	0.04	0.00	0.01	0.01	0.00	0.00	0.08	0.08	0.11	0.06	0.04	0.00	0.06	0.01	0.05
TCGA-DC-6157	0.01	0.02	0.05	0.07	0.00	0.13	0.12	0.07	0.00	0.00	0.01	0.00	0.00	0.17	0.12	0.09	0.08	0.02	0.06	0.00	0.00	0.00
TCGA-AF-4110	0.03	0.00	0.11	0.08	0.00	0.10	0.05	0.03	0.00	0.01	0.06	0.00	0.00	0.16	0.09	0.13	0.03	0.00	0.00	0.06	0.03	0.02
TCGA-AG-3591	0.01	0.00	0.03	0.08	0.00	0.30	0.00	0.02	0.00	0.04	0.00	0.00	0.00	0.02	0.03	0.17	0.12	0.02	0.00	0.08	0.07	0.01
TCGA-EI-6881	0.01	0.01	0.03	0.00	0.00	0.25	0.06	0.05	0.00	0.00	0.09	0.00	0.00	0.11	0.03	0.10	0.00	0.06	0.00	0.12	0.00	0.08
TCGA-AG-A008	0.01	0.00	0.07	0.06	0.00	0.34	0.00	0.00	0.08	0.00	0.02	0.00	0.00	0.11	0.00	0.07	0.02	0.01	0.00	0.21	0.00	0.00
TCGA-AG-A015	0.00	0.08	0.01	0.05	0.00	0.23	0.00	0.00	0.05	0.00	0.09	0.00	0.02	0.15	0.00	0.00	0.06	0.10	0.00	0.18	0.00	0.00
TCGA-AG-3892	0.00	0.03	0.11	0.12	0.00	0.10	0.09	0.05	0.00	0.01	0.02	0.00	0.03	0.07	0.09	0.05	0.04	0.04	0.00	0.09	0.03	0.04
TCGA-G5-6233	0.00	0.08	0.04	0.03	0.00	0.19	0.00	0.04	0.04	0.00	0.00	0.00	0.00	0.17	0.01	0.13	0.03	0.07	0.00	0.13	0.03	0.01
TCGA-AG-3898	0.00	0.01	0.02	0.03	0.00	0.01	0.07	0.03	0.00	0.03	0.03	0.00	0.00	0.10	0.18	0.14	0.04	0.00	0.00	0.13	0.02	0.15
TCGA-AG-3578	0.00	0.02	0.04	0.05	0.00	0.23	0.00	0.00	0.00	0.00	0.03	0.00	0.00	0.14	0.05	0.14	0.03	0.03	0.00	0.10	0.00	0.13
TCGA-DC-6682	0.05	0.00	0.07	0.20	0.00	0.12	0.04	0.01	0.07	0.00	0.00	0.00	0.00	0.18	0.08	0.06	0.03	0.00	0.00	0.06	0.03	0.00
TCGA-F5-6813	0.04	0.03	0.07	0.15	0.00	0.06	0.03	0.06	0.00	0.05	0.00	0.00	0.00	0.17	0.14	0.12	0.02	0.00	0.00	0.08	0.00	0.00
TCGA-F5-6702	0.02	0.02	0.02	0.10	0.00	0.16	0.06	0.02	0.01	0.00	0.02	0.00	0.00	0.19	0.11	0.14	0.09	0.00	0.00	0.04	0.00	0.00
TCGA-CL-5917	0.00	0.02	0.03	0.05	0.00	0.14	0.10	0.00	0.01	0.00	0.05	0.00	0.03	0.27	0.00	0.01	0.04	0.05	0.00	0.12	0.00	0.08
TCGA-CL-4957	0.04	0.00	0.09	0.06	0.00	0.23	0.19	0.02	0.00	0.00	0.03	0.00	0.00	0.10	0.01	0.10	0.05	0.04	0.03	0.00	0.00	0.01
TCGA-EI-6507	0.05	0.00	0.03	0.13	0.00	0.07	0.08	0.03	0.00	0.05	0.03	0.00	0.00	0.17	0.08	0.08	0.00	0.01	0.00	0.15	0.00	0.06
TCGA-DC-4749	0.05	0.00	0.18	0.04	0.00	0.05	0.11	0.08	0.03	0.00	0.05	0.00	0.00	0.14	0.04	0.06	0.02	0.04	0.00	0.09	0.00	0.00
TCGA-AF-2690	0.00	0.02	0.11	0.08	0.00	0.12	0.05	0.04	0.01	0.01	0.02	0.00	0.00	0.11	0.11	0.15	0.06	0.00	0.00	0.08	0.00	0.04
TCGA-EF-5830	0.01	0.00	0.00	0.09	0.00	0.16	0.03	0.00	0.01	0.02	0.00	0.13	0.00	0.23	0.07	0.17	0.03	0.00	0.06	0.00	0.00	0.00
TCGA-AF-3911	0.01	0.01	0.04	0.15	0.00	0.13	0.06	0.04	0.00	0.00	0.02	0.00	0.00	0.10	0.07	0.11	0.10	0.01	0.00	0.06	0.06	0.04
TCGA-DC-6155	0.10	0.00	0.03	0.06	0.00	0.16	0.00	0.01	0.02	0.00	0.08	0.00	0.05	0.24	0.03	0.08	0.03	0.01	0.00	0.12	0.00	0.00
TCGA-AF-A56L	0.01	0.04	0.01	0.18	0.00	0.00	0.02	0.06	0.02	0.00	0.03	0.00	0.00	0.18	0.13	0.17	0.01	0.01	0.00	0.12	0.00	0.00
TCGA-CI-6623	0.01	0.00	0.05	0.07	0.00	0.08	0.19	0.00	0.00	0.00	0.05	0.00	0.00	0.16	0.04	0.05	0.01	0.02	0.00	0.11	0.00	0.16
TCGA-AH-6897	0.00	0.03	0.05	0.11	0.00	0.28	0.09	0.00	0.04	0.00	0.10	0.00	0.02	0.08	0.04	0.04	0.01	0.05	0.00	0.07	0.00	0.00
TCGA-AG-3574	0.00	0.00	0.13	0.19	0.00	0.05	0.11	0.02	0.01	0.00	0.04	0.00	0.02	0.07	0.05	0.15	0.11	0.00	0.00	0.01	0.03	0.02
TCGA-G5-6235	0.17	0.00	0.05	0.12	0.00	0.01	0.07	0.20	0.05	0.02	0.00	0.04	0.00	0.10	0.04	0.00	0.00	0.09	0.00	0.03	0.01	0.02
TCGA-AG-4005	0.00	0.02	0.10	0.10	0.00	0.15	0.00	0.00	0.06	0.00	0.01	0.00	0.00	0.20	0.03	0.14	0.05	0.01	0.00	0.07	0.00	0.06
TCGA-AG-3583	0.03	0.00	0.16	0.08	0.00	0.13	0.08	0.00	0.00	0.01	0.05	0.00	0.00	0.06	0.05	0.11	0.03	0.03	0.00	0.10	0.07	0.02
TCGA-AH-6547	0.06	0.00	0.12	0.09	0.00	0.15	0.00	0.01	0.04	0.05	0.00	0.00	0.00	0.14	0.08	0.13	0.04	0.01	0.00	0.05	0.02	0.01
TCGA-EI-7002	0.00	0.01	0.03	0.05	0.00	0.10	0.09	0.02	0.01	0.00	0.11	0.00	0.00	0.21	0.08	0.11	0.07	0.00	0.00	0.10	0.00	0.00

TCGA-AG-A00Y	0.07	0.01	0.03	0.19	0.00	0.04	0.12	0.02	0.04	0.00	0.00	0.00	0.00	0.17	0.14	0.05	0.02	0.04	0.02	0.05	0.00	0.00
TCGA-EI-6506	0.00	0.00	0.17	0.09	0.00	0.10	0.05	0.01	0.02	0.00	0.03	0.00	0.00	0.11	0.08	0.11	0.07	0.02	0.06	0.03	0.04	0.02
TCGA-AH-6643	0.00	0.02	0.00	0.07	0.00	0.06	0.00	0.08	0.01	0.00	0.05	0.00	0.00	0.27	0.11	0.09	0.00	0.00	0.00	0.12	0.00	0.12
TCGA-AG-3890	0.07	0.00	0.13	0.08	0.00	0.22	0.02	0.06	0.01	0.00	0.02	0.00	0.00	0.10	0.03	0.08	0.06	0.01	0.00	0.09	0.00	0.00
TCGA-F5-6571	0.02	0.00	0.05	0.16	0.00	0.10	0.05	0.02	0.03	0.00	0.01	0.00	0.00	0.14	0.11	0.16	0.09	0.00	0.00	0.02	0.03	0.01
TCGA-AG-3882	0.08	0.00	0.07	0.18	0.00	0.04	0.09	0.04	0.03	0.00	0.08	0.00	0.00	0.14	0.08	0.06	0.06	0.00	0.00	0.05	0.00	0.01
TCGA-AG-3598	0.02	0.00	0.02	0.18	0.00	0.00	0.12	0.10	0.00	0.00	0.00	0.07	0.00	0.04	0.17	0.15	0.05	0.00	0.00	0.03	0.03	0.02
TCGA-F5-6811	0.01	0.01	0.01	0.09	0.00	0.12	0.05	0.06	0.03	0.00	0.02	0.00	0.00	0.25	0.10	0.16	0.02	0.02	0.01	0.03	0.00	0.01
TCGA-F5-6464	0.02	0.01	0.00	0.16	0.00	0.08	0.02	0.04	0.05	0.01	0.00	0.00	0.00	0.15	0.12	0.14	0.08	0.00	0.00	0.06	0.02	0.03
TCGA-CI-6619	0.02	0.00	0.02	0.05	0.00	0.19	0.08	0.02	0.00	0.00	0.05	0.00	0.00	0.15	0.08	0.11	0.01	0.05	0.08	0.02	0.00	0.06
TCGA-AF-2692	0.01	0.00	0.03	0.16	0.00	0.13	0.00	0.03	0.00	0.00	0.04	0.00	0.02	0.14	0.01	0.03	0.08	0.06	0.00	0.17	0.07	0.02
GSM437093	0.00	0.02	0.07	0.22	0.00	0.13	0.06	0.06	0.01	0.00	0.01	0.01	0.00	0.12	0.11	0.12	0.03	0.03	0.01	0.00	0.00	0.00
GSM437094	0.04	0.03	0.16	0.10	0.00	0.29	0.00	0.04	0.06	0.00	0.05	0.00	0.00	0.06	0.03	0.08	0.04	0.01	0.00	0.02	0.00	0.00
GSM437095	0.02	0.01	0.18	0.19	0.00	0.25	0.00	0.01	0.01	0.00	0.02	0.00	0.01	0.06	0.01	0.01	0.08	0.00	0.00	0.08	0.00	0.07
GSM437096	0.02	0.03	0.03	0.14	0.00	0.17	0.02	0.12	0.02	0.00	0.04	0.00	0.04	0.14	0.05	0.07	0.00	0.03	0.00	0.07	0.00	0.00
GSM437097	0.03	0.00	0.02	0.21	0.00	0.15	0.00	0.03	0.03	0.00	0.06	0.00	0.00	0.14	0.04	0.16	0.02	0.02	0.00	0.10	0.00	0.00
GSM437098	0.00	0.00	0.11	0.18	0.00	0.09	0.11	0.06	0.01	0.00	0.05	0.00	0.00	0.11	0.12	0.08	0.02	0.02	0.05	0.00	0.00	0.00
GSM437099	0.00	0.06	0.16	0.23	0.00	0.27	0.03	0.02	0.01	0.03	0.00	0.00	0.00	0.01	0.05	0.07	0.01	0.02	0.00	0.04	0.00	0.00
GSM437100	0.00	0.00	0.10	0.13	0.00	0.18	0.07	0.07	0.00	0.00	0.06	0.00	0.00	0.05	0.05	0.08	0.05	0.08	0.00	0.05	0.00	0.04
GSM437101	0.00	0.03	0.02	0.18	0.00	0.12	0.10	0.06	0.00	0.00	0.07	0.00	0.00	0.11	0.03	0.08	0.06	0.00	0.00	0.14	0.00	0.00
GSM437102	0.00	0.00	0.08	0.03	0.00	0.20	0.01	0.00	0.00	0.00	0.03	0.00	0.03	0.17	0.02	0.23	0.00	0.04	0.00	0.11	0.01	0.05
GSM437103	0.00	0.00	0.07	0.15	0.00	0.10	0.07	0.08	0.00	0.00	0.04	0.00	0.01	0.15	0.11	0.08	0.08	0.00	0.00	0.04	0.00	0.00
GSM437104	0.03	0.00	0.01	0.40	0.00	0.00	0.02	0.10	0.07	0.00	0.11	0.00	0.02	0.04	0.03	0.04	0.03	0.03	0.00	0.07	0.00	0.00
GSM437105	0.17	0.00	0.09	0.10	0.00	0.17	0.02	0.08	0.00	0.00	0.04	0.00	0.00	0.07	0.11	0.05	0.04	0.00	0.00	0.07	0.00	0.00
GSM437106	0.01	0.03	0.09	0.04	0.00	0.15	0.15	0.08	0.02	0.02	0.09	0.00	0.00	0.03	0.05	0.08	0.07	0.02	0.00	0.05	0.00	0.02
GSM437107	0.04	0.00	0.11	0.08	0.00	0.28	0.00	0.05	0.06	0.00	0.00	0.02	0.00	0.07	0.13	0.07	0.05	0.00	0.00	0.05	0.00	0.00
GSM437108	0.00	0.02	0.05	0.08	0.00	0.24	0.00	0.07	0.03	0.00	0.10	0.00	0.00	0.06	0.09	0.03	0.06	0.00	0.00	0.11	0.00	0.04
GSM437109	0.01	0.01	0.05	0.10	0.00	0.14	0.06	0.07	0.05	0.00	0.01	0.00	0.00	0.04	0.17	0.11	0.14	0.01	0.02	0.00	0.00	0.01
GSM437110	0.05	0.03	0.12	0.14	0.00	0.13	0.07	0.05	0.05	0.00	0.05	0.00	0.03	0.09	0.11	0.00	0.06	0.00	0.00	0.03	0.00	0.00
GSM437111	0.06	0.00	0.01	0.09	0.00	0.05	0.00	0.07	0.01	0.00	0.04	0.00	0.00	0.12	0.06	0.16	0.01	0.07	0.00	0.21	0.00	0.04
GSM437112	0.05	0.00	0.01	0.07	0.00	0.13	0.05	0.11	0.00	0.00	0.01	0.00	0.01	0.09	0.14	0.04	0.03	0.03	0.00	0.14	0.04	0.05
GSM437113	0.01	0.01	0.00	0.14	0.00	0.06	0.14	0.02	0.01	0.00	0.02	0.00	0.00	0.31	0.02	0.14	0.08	0.00	0.02	0.01	0.00	0.00
GSM437114	0.01	0.02	0.10	0.11	0.00	0.21	0.05	0.04	0.04	0.00	0.05	0.00	0.01	0.08	0.09	0.06	0.08	0.00	0.00	0.06	0.01	0.00
GSM437115	0.04	0.00	0.10	0.22	0.00	0.09	0.17	0.00	0.01	0.00	0.06	0.00	0.00	0.08	0.01	0.13	0.08	0.00	0.00	0.01	0.00	0.00
GSM437116	0.03	0.00	0.09	0.19	0.00	0.00	0.10	0.09	0.00	0.03	0.00	0.00	0.00	0.14	0.12	0.11	0.03	0.00	0.00	0.02	0.01	0.02
GSM437117	0.00	0.01	0.00	0.12	0.00	0.12	0.08	0.06	0.00	0.00	0.04	0.00	0.00	0.31	0.08	0.10	0.00	0.00	0.00	0.08	0.00	0.00
GSM437118	0.00	0.00	0.00	0.14	0.00	0.00	0.12	0.06	0.00	0.07	0.01	0.00	0.00	0.04	0.22	0.09	0.00	0.13	0.00	0.00	0.00	0.12
GSM437119	0.04	0.00	0.00	0.06	0.00	0.17	0.00	0.00	0.02	0.00	0.03	0.00	0.00	0.35	0.03	0.19	0.05	0.00	0.06	0.00	0.00	0.00

GSM437120	0.00	0.00	0.17	0.07	0.00	0.18	0.10	0.01	0.00	0.00	0.07	0.00	0.01	0.05	0.02	0.11	0.01	0.02	0.00	0.07	0.11	0.00
GSM437121	0.05	0.00	0.05	0.15	0.00	0.04	0.06	0.07	0.02	0.05	0.00	0.04	0.00	0.14	0.13	0.15	0.05	0.02	0.00	0.00	0.00	0.00
GSM437122	0.00	0.05	0.02	0.12	0.00	0.22	0.05	0.02	0.03	0.00	0.03	0.00	0.02	0.16	0.06	0.09	0.01	0.03	0.00	0.07	0.01	0.00
GSM437124	0.00	0.04	0.01	0.16	0.00	0.07	0.00	0.09	0.01	0.00	0.04	0.01	0.00	0.18	0.15	0.11	0.07	0.00	0.00	0.03	0.00	0.03
GSM437125	0.00	0.00	0.04	0.06	0.00	0.12	0.00	0.03	0.02	0.00	0.01	0.00	0.00	0.16	0.03	0.12	0.10	0.01	0.00	0.09	0.09	0.11
GSM437126	0.00	0.00	0.01	0.32	0.00	0.00	0.20	0.03	0.00	0.00	0.06	0.02	0.00	0.06	0.13	0.07	0.04	0.03	0.00	0.00	0.00	0.03
GSM437127	0.04	0.05	0.03	0.06	0.00	0.21	0.02	0.03	0.04	0.00	0.01	0.00	0.00	0.17	0.03	0.09	0.08	0.04	0.00	0.07	0.00	0.01
GSM437128	0.01	0.02	0.07	0.24	0.00	0.15	0.02	0.07	0.02	0.00	0.00	0.01	0.02	0.10	0.05	0.10	0.04	0.02	0.00	0.07	0.00	0.00
GSM437129	0.02	0.01	0.10	0.20	0.00	0.15	0.08	0.01	0.00	0.00	0.05	0.00	0.03	0.04	0.10	0.11	0.05	0.03	0.00	0.00	0.00	0.00
GSM437130	0.00	0.05	0.00	0.09	0.00	0.13	0.06	0.10	0.03	0.00	0.00	0.02	0.00	0.14	0.15	0.08	0.09	0.00	0.00	0.06	0.00	0.01
GSM437131	0.03	0.04	0.12	0.15	0.00	0.20	0.04	0.02	0.02	0.00	0.02	0.00	0.00	0.03	0.07	0.10	0.02	0.02	0.13	0.00	0.00	0.00
GSM437132	0.01	0.03	0.01	0.17	0.00	0.18	0.04	0.00	0.04	0.00	0.09	0.00	0.02	0.14	0.08	0.08	0.07	0.00	0.00	0.06	0.00	0.00
GSM437133	0.00	0.01	0.04	0.19	0.00	0.05	0.09	0.05	0.00	0.02	0.02	0.02	0.00	0.03	0.20	0.18	0.01	0.07	0.00	0.02	0.00	0.00
GSM437134	0.00	0.08	0.01	0.19	0.00	0.07	0.00	0.07	0.00	0.00	0.04	0.00	0.00	0.13	0.07	0.09	0.06	0.03	0.00	0.07	0.00	0.10
GSM437135	0.00	0.03	0.03	0.10	0.00	0.17	0.00	0.00	0.00	0.00	0.04	0.00	0.00	0.30	0.03	0.09	0.02	0.02	0.00	0.15	0.00	0.01
GSM437136	0.00	0.06	0.01	0.11	0.00	0.06	0.09	0.04	0.00	0.00	0.03	0.04	0.00	0.25	0.05	0.15	0.06	0.03	0.02	0.00	0.00	0.00
GSM437137	0.01	0.00	0.00	0.19	0.00	0.04	0.11	0.05	0.00	0.00	0.04	0.01	0.00	0.19	0.06	0.19	0.01	0.07	0.00	0.04	0.00	0.00
GSM437138	0.00	0.02	0.03	0.04	0.00	0.38	0.00	0.00	0.02	0.00	0.10	0.00	0.03	0.13	0.02	0.07	0.00	0.03	0.00	0.04	0.00	0.09
GSM437139	0.01	0.00	0.00	0.12	0.00	0.27	0.03	0.00	0.00	0.00	0.02	0.00	0.00	0.31	0.04	0.18	0.00	0.02	0.00	0.00	0.00	0.00
GSM437140	0.03	0.00	0.10	0.16	0.00	0.17	0.00	0.11	0.01	0.00	0.06	0.00	0.03	0.07	0.05	0.00	0.02	0.00	0.00	0.16	0.03	0.00
GSM437141	0.00	0.00	0.02	0.16	0.00	0.17	0.05	0.11	0.01	0.04	0.00	0.02	0.00	0.10	0.20	0.07	0.05	0.00	0.00	0.00	0.00	0.00
GSM437142	0.00	0.08	0.01	0.04	0.00	0.10	0.07	0.06	0.00	0.00	0.06	0.00	0.00	0.11	0.08	0.08	0.00	0.02	0.00	0.12	0.00	0.16
GSM437143	0.00	0.00	0.05	0.24	0.00	0.00	0.10	0.06	0.03	0.04	0.04	0.00	0.00	0.08	0.13	0.08	0.07	0.00	0.00	0.04	0.00	0.02
GSM437144	0.02	0.00	0.12	0.07	0.00	0.10	0.04	0.10	0.02	0.00	0.05	0.00	0.00	0.19	0.06	0.13	0.00	0.01	0.00	0.09	0.00	0.00
GSM437145	0.02	0.00	0.13	0.16	0.00	0.05	0.04	0.10	0.00	0.02	0.02	0.00	0.00	0.11	0.16	0.09	0.00	0.00	0.00	0.05	0.00	0.04
GSM437146	0.04	0.00	0.07	0.02	0.00	0.32	0.07	0.03	0.01	0.12	0.00	0.00	0.00	0.08	0.06	0.02	0.02	0.04	0.00	0.07	0.00	0.00
GSM437147	0.01	0.00	0.11	0.01	0.00	0.28	0.00	0.05	0.00	0.00	0.03	0.09	0.01	0.02	0.12	0.12	0.10	0.00	0.00	0.06	0.00	0.00
GSM437148	0.01	0.00	0.02	0.16	0.00	0.03	0.19	0.05	0.01	0.00	0.14	0.00	0.00	0.10	0.08	0.04	0.06	0.00	0.00	0.07	0.00	0.04
GSM437149	0.02	0.00	0.09	0.10	0.00	0.15	0.00	0.05	0.01	0.00	0.02	0.00	0.00	0.23	0.06	0.07	0.01	0.00	0.00	0.17	0.00	0.02
GSM437150	0.00	0.02	0.03	0.23	0.00	0.04	0.00	0.04	0.04	0.00	0.04	0.00	0.00	0.08	0.00	0.07	0.07	0.04	0.00	0.20	0.00	0.08
GSM437151	0.01	0.01	0.12	0.07	0.00	0.21	0.04	0.05	0.03	0.00	0.07	0.00	0.00	0.13	0.05	0.07	0.03	0.01	0.00	0.09	0.00	0.00
GSM437152	0.01	0.00	0.01	0.24	0.00	0.00	0.11	0.08	0.03	0.00	0.04	0.00	0.00	0.06	0.11	0.15	0.10	0.00	0.00	0.05	0.00	0.01
GSM437153	0.10	0.00	0.01	0.25	0.00	0.03	0.01	0.02	0.11	0.00	0.14	0.00	0.00	0.02	0.00	0.00	0.07	0.05	0.00	0.19	0.00	0.00
GSM437154	0.04	0.01	0.10	0.24	0.00	0.18	0.00	0.04	0.01	0.00	0.02	0.00	0.03	0.06	0.05	0.05	0.09	0.00	0.00	0.09	0.00	0.00
GSM437155	0.10	0.00	0.06	0.12	0.00	0.14	0.07	0.02	0.01	0.00	0.07	0.00	0.00	0.09	0.11	0.06	0.10	0.01	0.00	0.03	0.00	0.03
GSM437156	0.02	0.00	0.00	0.11	0.00	0.03	0.11	0.05	0.02	0.02	0.12	0.01	0.00	0.05	0.20	0.08	0.07	0.01	0.01	0.00	0.00	0.08
GSM437157	0.05	0.00	0.03	0.05	0.00	0.12	0.02	0.06	0.00	0.00	0.05	0.00	0.01	0.37	0.06	0.09	0.05	0.00	0.00	0.03	0.01	0.00
GSM437158	0.00	0.00	0.02	0.07	0.00	0.31	0.00	0.00	0.02	0.00	0.11	0.00	0.03	0.05	0.01	0.12	0.11	0.02	0.00	0.13	0.00	0.00

GSM437159	0.04	0.00	0.06	0.04	0.00	0.15	0.06	0.04	0.00	0.00	0.03	0.00	0.00	0.18	0.11	0.05	0.07	0.00	0.00	0.12	0.00	0.04
GSM437160	0.03	0.00	0.01	0.15	0.00	0.21	0.00	0.05	0.04	0.00	0.01	0.01	0.00	0.12	0.07	0.03	0.03	0.02	0.00	0.14	0.00	0.07
GSM437161	0.06	0.00	0.04	0.02	0.00	0.12	0.02	0.08	0.01	0.01	0.00	0.00	0.00	0.08	0.13	0.06	0.08	0.00	0.00	0.14	0.10	0.06
GSM437162	0.03	0.00	0.11	0.08	0.00	0.25	0.04	0.06	0.00	0.00	0.00	0.00	0.00	0.05	0.07	0.15	0.02	0.02	0.03	0.06	0.01	0.01
GSM437163	0.00	0.04	0.03	0.08	0.00	0.06	0.06	0.03	0.02	0.00	0.01	0.00	0.00	0.09	0.01	0.15	0.17	0.05	0.13	0.03	0.03	0.01
GSM437164	0.01	0.01	0.03	0.20	0.00	0.16	0.03	0.02	0.02	0.00	0.00	0.01	0.00	0.15	0.14	0.10	0.08	0.00	0.01	0.02	0.00	0.00
GSM437166	0.03	0.00	0.07	0.09	0.00	0.09	0.12	0.01	0.00	0.00	0.03	0.00	0.06	0.05	0.06	0.10	0.03	0.00	0.00	0.13	0.06	0.08
GSM437167	0.07	0.00	0.03	0.20	0.00	0.02	0.02	0.05	0.02	0.00	0.01	0.01	0.00	0.09	0.12	0.12	0.06	0.00	0.00	0.06	0.01	0.11
GSM437168	0.01	0.01	0.01	0.27	0.00	0.00	0.07	0.03	0.03	0.00	0.04	0.00	0.04	0.17	0.09	0.11	0.04	0.03	0.00	0.03	0.00	0.01
GSM437169	0.00	0.04	0.00	0.09	0.00	0.15	0.08	0.00	0.09	0.00	0.06	0.00	0.00	0.28	0.02	0.09	0.01	0.02	0.00	0.05	0.00	0.00
GSM437170	0.00	0.04	0.02	0.07	0.00	0.20	0.02	0.00	0.05	0.00	0.04	0.00	0.03	0.26	0.02	0.06	0.08	0.01	0.00	0.07	0.03	0.00
GSM437171	0.02	0.00	0.02	0.07	0.00	0.12	0.00	0.05	0.04	0.01	0.00	0.07	0.03	0.06	0.15	0.14	0.08	0.00	0.00	0.12	0.01	0.01
GSM437172	0.03	0.01	0.01	0.16	0.00	0.10	0.13	0.05	0.00	0.00	0.01	0.00	0.03	0.07	0.03	0.11	0.10	0.03	0.00	0.08	0.05	0.00
GSM437173	0.00	0.03	0.02	0.03	0.00	0.12	0.00	0.11	0.01	0.00	0.03	0.00	0.00	0.08	0.04	0.26	0.15	0.01	0.00	0.09	0.00	0.04
GSM437174	0.00	0.07	0.01	0.11	0.00	0.12	0.09	0.05	0.00	0.00	0.07	0.00	0.00	0.18	0.10	0.12	0.03	0.00	0.06	0.00	0.00	0.01
GSM437175	0.04	0.00	0.02	0.17	0.00	0.04	0.03	0.07	0.02	0.02	0.03	0.00	0.00	0.08	0.12	0.10	0.05	0.00	0.00	0.10	0.00	0.10
GSM437176	0.15	0.00	0.00	0.04	0.00	0.16	0.00	0.01	0.00	0.00	0.10	0.00	0.00	0.10	0.10	0.02	0.00	0.03	0.00	0.20	0.00	0.07
GSM437177	0.02	0.07	0.02	0.15	0.00	0.10	0.10	0.08	0.00	0.00	0.02	0.01	0.01	0.12	0.06	0.11	0.07	0.01	0.02	0.01	0.00	0.01
GSM437178	0.00	0.02	0.03	0.16	0.00	0.09	0.05	0.09	0.03	0.00	0.04	0.00	0.01	0.11	0.08	0.09	0.08	0.00	0.00	0.09	0.02	0.01
GSM437179	0.00	0.08	0.00	0.15	0.00	0.15	0.08	0.03	0.06	0.00	0.03	0.00	0.00	0.11	0.07	0.07	0.10	0.03	0.00	0.04	0.00	0.00
GSM437180	0.00	0.01	0.01	0.03	0.00	0.24	0.00	0.01	0.00	0.00	0.06	0.00	0.05	0.19	0.02	0.09	0.03	0.06	0.00	0.16	0.00	0.05
GSM437181	0.02	0.02	0.04	0.04	0.00	0.17	0.00	0.06	0.00	0.01	0.02	0.00	0.01	0.11	0.03	0.07	0.08	0.07	0.00	0.22	0.00	0.02
GSM437182	0.04	0.00	0.00	0.22	0.00	0.04	0.08	0.03	0.01	0.00	0.07	0.01	0.00	0.07	0.18	0.15	0.06	0.00	0.00	0.00	0.04	0.00
GSM437183	0.00	0.02	0.07	0.16	0.00	0.13	0.18	0.00	0.01	0.00	0.04	0.00	0.00	0.12	0.08	0.08	0.09	0.01	0.00	0.00	0.00	0.01
GSM437184	0.07	0.00	0.00	0.12	0.00	0.10	0.10	0.13	0.00	0.00	0.01	0.00	0.00	0.01	0.16	0.06	0.03	0.04	0.00	0.09	0.00	0.06
GSM437185	0.00	0.07	0.05	0.23	0.00	0.00	0.05	0.03	0.07	0.00	0.09	0.00	0.00	0.07	0.00	0.08	0.13	0.03	0.00	0.09	0.00	0.01
GSM437186	0.00	0.01	0.01	0.12	0.00	0.23	0.08	0.00	0.02	0.00	0.03	0.00	0.00	0.21	0.10	0.11	0.01	0.00	0.00	0.05	0.00	0.00
GSM437187	0.02	0.00	0.09	0.09	0.00	0.12	0.00	0.02	0.00	0.00	0.01	0.00	0.00	0.12	0.05	0.06	0.02	0.00	0.00	0.21	0.13	0.06
GSM437188	0.00	0.04	0.07	0.19	0.00	0.04	0.12	0.08	0.01	0.00	0.03	0.00	0.00	0.10	0.09	0.10	0.10	0.02	0.01	0.00	0.00	0.00
GSM437189	0.01	0.01	0.00	0.01	0.00	0.09	0.00	0.01	0.04	0.00	0.03	0.00	0.00	0.50	0.03	0.14	0.02	0.01	0.00	0.04	0.00	0.06
GSM437190	0.01	0.00	0.03	0.06	0.00	0.09	0.06	0.02	0.00	0.00	0.05	0.00	0.02	0.29	0.03	0.06	0.00	0.01	0.00	0.14	0.02	0.10
GSM437191	0.02	0.02	0.09	0.18	0.00	0.12	0.07	0.08	0.01	0.01	0.00	0.00	0.00	0.03	0.08	0.13	0.10	0.01	0.00	0.05	0.00	0.01
GSM437192	0.00	0.04	0.04	0.09	0.00	0.21	0.00	0.08	0.00	0.00	0.01	0.01	0.01	0.14	0.05	0.14	0.03	0.04	0.02	0.07	0.00	0.01
GSM437193	0.01	0.00	0.00	0.14	0.00	0.23	0.00	0.06	0.00	0.00	0.03	0.03	0.00	0.14	0.12	0.07	0.01	0.03	0.00	0.12	0.00	0.02
GSM437194	0.00	0.03	0.16	0.17	0.00	0.18	0.00	0.03	0.07	0.00	0.03	0.01	0.01	0.07	0.02	0.08	0.05	0.02	0.00	0.07	0.00	0.00
GSM437195	0.02	0.01	0.04	0.10	0.00	0.13	0.02	0.04	0.01	0.00	0.04	0.00	0.00	0.24	0.08	0.10	0.03	0.00	0.00	0.15	0.00	0.00
GSM437196	0.00	0.05	0.04	0.16	0.00	0.17	0.11	0.02	0.00	0.00	0.08	0.00	0.00	0.14	0.08	0.07	0.05	0.00	0.01	0.00	0.02	0.00
GSM437197	0.00	0.04	0.00	0.09	0.00	0.16	0.00	0.00	0.00	0.00	0.09	0.00	0.03	0.36	0.02	0.03	0.00	0.02	0.00	0.16	0.00	0.01

GSM437198	0.01	0.00	0.01	0.12	0.00	0.07	0.10	0.02	0.00	0.00	0.02	0.02	0.00	0.04	0.09	0.10	0.04	0.00	0.00	0.23	0.08	0.06
GSM437199	0.03	0.00	0.01	0.24	0.00	0.17	0.00	0.03	0.00	0.00	0.00	0.00	0.01	0.10	0.09	0.21	0.06	0.00	0.01	0.03	0.00	0.00
GSM437200	0.03	0.00	0.00	0.04	0.00	0.23	0.00	0.00	0.04	0.00	0.06	0.00	0.01	0.21	0.03	0.12	0.05	0.02	0.00	0.14	0.00	0.03
GSM437201	0.03	0.00	0.05	0.03	0.00	0.17	0.00	0.01	0.01	0.00	0.03	0.00	0.01	0.30	0.03	0.12	0.06	0.00	0.00	0.03	0.00	0.10
GSM437202	0.02	0.00	0.04	0.10	0.00	0.16	0.01	0.06	0.00	0.00	0.01	0.02	0.00	0.05	0.08	0.17	0.06	0.12	0.08	0.00	0.00	0.02
GSM437203	0.00	0.00	0.04	0.17	0.00	0.03	0.10	0.04	0.02	0.09	0.00	0.00	0.01	0.20	0.16	0.08	0.00	0.00	0.00	0.05	0.01	0.01
GSM437205	0.00	0.02	0.13	0.08	0.00	0.13	0.02	0.01	0.02	0.00	0.04	0.00	0.00	0.09	0.04	0.16	0.05	0.05	0.02	0.10	0.02	0.00
GSM437206	0.00	0.04	0.09	0.09	0.00	0.19	0.02	0.01	0.03	0.00	0.04	0.00	0.00	0.28	0.07	0.06	0.02	0.00	0.00	0.07	0.00	0.00
GSM437207	0.03	0.00	0.01	0.03	0.00	0.18	0.00	0.07	0.03	0.00	0.04	0.00	0.01	0.21	0.07	0.05	0.04	0.00	0.00	0.16	0.04	0.05
GSM437208	0.00	0.10	0.06	0.15	0.00	0.21	0.01	0.00	0.08	0.00	0.00	0.03	0.04	0.11	0.03	0.12	0.00	0.02	0.00	0.03	0.00	0.00
GSM437209	0.00	0.00	0.02	0.42	0.00	0.00	0.15	0.04	0.03	0.00	0.03	0.01	0.00	0.03	0.11	0.06	0.03	0.00	0.02	0.00	0.00	0.04
GSM437210	0.01	0.03	0.03	0.06	0.00	0.15	0.00	0.02	0.03	0.00	0.05	0.00	0.00	0.19	0.07	0.10	0.02	0.00	0.00	0.14	0.00	0.10
GSM437211	0.02	0.00	0.04	0.24	0.00	0.00	0.17	0.03	0.00	0.01	0.04	0.00	0.00	0.10	0.15	0.09	0.06	0.00	0.00	0.01	0.00	0.03
GSM437212	0.01	0.02	0.01	0.14	0.00	0.00	0.19	0.07	0.00	0.00	0.04	0.04	0.00	0.00	0.24	0.07	0.06	0.01	0.01	0.00	0.00	0.09
GSM437213	0.02	0.00	0.17	0.14	0.00	0.15	0.00	0.01	0.05	0.00	0.03	0.00	0.01	0.05	0.05	0.08	0.06	0.06	0.00	0.06	0.04	0.01
GSM437214	0.00	0.02	0.08	0.03	0.00	0.25	0.00	0.02	0.06	0.00	0.04	0.00	0.00	0.07	0.03	0.12	0.15	0.03	0.02	0.09	0.00	0.01
GSM437215	0.02	0.00	0.11	0.19	0.00	0.08	0.00	0.00	0.01	0.00	0.03	0.00	0.00	0.15	0.03	0.09	0.05	0.02	0.00	0.15	0.05	0.03
GSM437216	0.11	0.00	0.18	0.08	0.00	0.20	0.06	0.06	0.00	0.00	0.03	0.01	0.00	0.02	0.05	0.12	0.03	0.01	0.04	0.00	0.00	0.00
GSM437217	0.26	0.00	0.08	0.06	0.00	0.14	0.15	0.04	0.00	0.00	0.09	0.00	0.00	0.10	0.02	0.01	0.00	0.02	0.00	0.03	0.00	0.00
GSM437218	0.00	0.04	0.09	0.09	0.00	0.27	0.00	0.02	0.02	0.00	0.01	0.02	0.01	0.06	0.03	0.11	0.03	0.03	0.04	0.04	0.08	0.00
GSM437219	0.03	0.00	0.08	0.04	0.00	0.34	0.06	0.01	0.01	0.00	0.06	0.00	0.00	0.07	0.01	0.11	0.07	0.02	0.00	0.03	0.01	0.05
GSM437220	0.01	0.01	0.02	0.10	0.00	0.07	0.02	0.09	0.07	0.05	0.00	0.01	0.00	0.20	0.13	0.11	0.07	0.00	0.00	0.02	0.00	0.01
GSM437221	0.04	0.00	0.03	0.04	0.00	0.24	0.05	0.03	0.00	0.00	0.08	0.00	0.00	0.18	0.07	0.08	0.00	0.01	0.00	0.11	0.00	0.03
GSM437222	0.00	0.01	0.02	0.04	0.00	0.16	0.00	0.01	0.01	0.01	0.01	0.00	0.00	0.39	0.05	0.23	0.00	0.00	0.00	0.04	0.03	0.01
GSM437223	0.03	0.00	0.06	0.07	0.00	0.16	0.03	0.03	0.00	0.00	0.02	0.00	0.00	0.20	0.07	0.15	0.09	0.00	0.02	0.02	0.02	0.03
GSM437224	0.00	0.04	0.06	0.11	0.00	0.11	0.02	0.13	0.02	0.00	0.03	0.00	0.00	0.10	0.10	0.11	0.07	0.02	0.00	0.06	0.01	0.00
GSM437225	0.01	0.00	0.03	0.13	0.00	0.14	0.08	0.04	0.01	0.01	0.00	0.01	0.03	0.05	0.08	0.12	0.12	0.08	0.04	0.00	0.02	0.00
GSM437226	0.41	0.00	0.03	0.02	0.00	0.12	0.05	0.01	0.00	0.05	0.04	0.00	0.05	0.03	0.04	0.05	0.04	0.00	0.00	0.03	0.02	0.00
GSM437227	0.02	0.00	0.03	0.04	0.00	0.03	0.05	0.02	0.01	0.03	0.07	0.00	0.00	0.34	0.08	0.12	0.00	0.00	0.00	0.09	0.04	0.03
GSM437228	0.02	0.00	0.00	0.14	0.00	0.00	0.00	0.00	0.08	0.00	0.00	0.01	0.00	0.44	0.04	0.23	0.04	0.00	0.00	0.00	0.00	0.00
GSM437229	0.02	0.00	0.04	0.19	0.00	0.00	0.09	0.06	0.00	0.03	0.01	0.00	0.01	0.14	0.10	0.05	0.05	0.00	0.00	0.09	0.01	0.11
GSM437230	0.03	0.00	0.12	0.23	0.00	0.07	0.14	0.06	0.01	0.00	0.00	0.02	0.00	0.04	0.04	0.10	0.07	0.00	0.07	0.00	0.00	0.00
GSM437232	0.00	0.11	0.14	0.16	0.00	0.18	0.01	0.00	0.00	0.00	0.01	0.00	0.01	0.13	0.06	0.05	0.07	0.00	0.00	0.06	0.00	0.00
GSM437238	0.00	0.05	0.02	0.19	0.00	0.14	0.00	0.03	0.04	0.00	0.00	0.08	0.03	0.08	0.14	0.11	0.09	0.00	0.01	0.01	0.00	0.00
GSM437243	0.01	0.03	0.00	0.08	0.00	0.09	0.00	0.03	0.03	0.00	0.04	0.00	0.00	0.25	0.05	0.23	0.01	0.00	0.00	0.10	0.00	0.05
GSM437248	0.00	0.11	0.05	0.14	0.00	0.13	0.03	0.08	0.01	0.00	0.03	0.00	0.06	0.09	0.07	0.09	0.00	0.03	0.00	0.06	0.00	0.00
GSM437249	0.04	0.02	0.06	0.15	0.00	0.16	0.10	0.05	0.00	0.00	0.06	0.00	0.00	0.06	0.04	0.10	0.04	0.03	0.01	0.07	0.00	0.01
GSM437255	0.05	0.02	0.08	0.15	0.00	0.32	0.00	0.02	0.04	0.00	0.03	0.00	0.00	0.03	0.09	0.04	0.04	0.00	0.00	0.07	0.02	0.00

GSM437261	0.01	0.01	0.10	0.12	0.00	0.22	0.17	0.00	0.00	0.00	0.00	0.00	0.00	0.12	0.06	0.09	0.06	0.00	0.00	0.05	0.00	0.00
GSM437264	0.07	0.00	0.08	0.14	0.00	0.08	0.05	0.01	0.03	0.00	0.08	0.00	0.00	0.17	0.02	0.08	0.00	0.00	0.00	0.17	0.00	0.00
GSM437265	0.01	0.02	0.03	0.11	0.00	0.08	0.03	0.02	0.00	0.00	0.02	0.00	0.00	0.37	0.04	0.11	0.02	0.01	0.00	0.10	0.00	0.01
GSM437266	0.00	0.05	0.11	0.30	0.00	0.00	0.01	0.07	0.02	0.09	0.00	0.00	0.00	0.09	0.13	0.07	0.03	0.00	0.01	0.01	0.00	0.00
GSM437270	0.00	0.02	0.02	0.06	0.00	0.12	0.00	0.04	0.00	0.00	0.04	0.00	0.09	0.08	0.03	0.10	0.03	0.02	0.00	0.16	0.01	0.18
GSM437271	0.01	0.03	0.03	0.13	0.00	0.08	0.15	0.02	0.00	0.00	0.07	0.00	0.00	0.15	0.16	0.09	0.00	0.00	0.00	0.07	0.00	0.00
GSM437272	0.03	0.00	0.04	0.16	0.00	0.07	0.01	0.02	0.00	0.00	0.02	0.00	0.00	0.14	0.07	0.03	0.03	0.03	0.00	0.19	0.00	0.15
GSM437273	0.06	0.00	0.00	0.24	0.00	0.20	0.00	0.06	0.02	0.00	0.01	0.04	0.00	0.11	0.04	0.10	0.03	0.03	0.00	0.06	0.01	0.00
GSM437274	0.00	0.02	0.08	0.17	0.00	0.25	0.00	0.00	0.04	0.00	0.02	0.00	0.03	0.08	0.03	0.14	0.04	0.02	0.00	0.07	0.00	0.00
GSM437275	0.00	0.00	0.14	0.18	0.00	0.24	0.04	0.01	0.01	0.00	0.01	0.01	0.01	0.12	0.03	0.10	0.01	0.02	0.00	0.04	0.00	0.01
GSM437276	0.02	0.00	0.01	0.04	0.04	0.14	0.04	0.01	0.00	0.00	0.06	0.00	0.01	0.30	0.08	0.07	0.00	0.03	0.02	0.02	0.00	0.10
GSM437277	0.02	0.00	0.09	0.07	0.00	0.26	0.13	0.00	0.00	0.00	0.05	0.00	0.00	0.13	0.03	0.08	0.05	0.05	0.01	0.01	0.00	0.01
GSM437278	0.00	0.02	0.02	0.10	0.00	0.16	0.09	0.02	0.00	0.00	0.05	0.00	0.00	0.19	0.04	0.07	0.01	0.05	0.00	0.16	0.00	0.03
GSM437279	0.05	0.04	0.00	0.19	0.00	0.20	0.00	0.00	0.01	0.00	0.00	0.04	0.00	0.12	0.08	0.14	0.10	0.00	0.00	0.04	0.00	0.00
GSM437280	0.00	0.00	0.00	0.01	0.00	0.23	0.06	0.03	0.01	0.00	0.02	0.01	0.00	0.16	0.10	0.02	0.04	0.00	0.00	0.16	0.00	0.15
GSM437281	0.01	0.05	0.05	0.16	0.00	0.20	0.07	0.00	0.02	0.00	0.02	0.00	0.00	0.03	0.04	0.09	0.06	0.03	0.00	0.10	0.00	0.06
GSM437282	0.08	0.00	0.17	0.08	0.00	0.14	0.11	0.00	0.02	0.00	0.00	0.01	0.00	0.06	0.07	0.07	0.05	0.02	0.10	0.00	0.00	0.02
GSM437283	0.05	0.00	0.11	0.18	0.00	0.27	0.00	0.02	0.00	0.00	0.00	0.06	0.01	0.01	0.03	0.11	0.10	0.00	0.05	0.00	0.00	0.00
GSM437284	0.03	0.00	0.02	0.08	0.00	0.22	0.00	0.09	0.03	0.00	0.04	0.00	0.00	0.17	0.18	0.06	0.02	0.00	0.00	0.04	0.00	0.01
GSM437285	0.03	0.10	0.04	0.24	0.00	0.20	0.02	0.00	0.05	0.00	0.00	0.00	0.00	0.06	0.04	0.04	0.11	0.00	0.00	0.04	0.00	0.03
GSM437287	0.00	0.04	0.04	0.19	0.00	0.13	0.11	0.04	0.01	0.00	0.02	0.00	0.00	0.14	0.07	0.09	0.07	0.02	0.00	0.01	0.00	0.01
GSM437288	0.13	0.00	0.06	0.10	0.00	0.19	0.02	0.05	0.00	0.00	0.06	0.00	0.00	0.16	0.09	0.13	0.00	0.00	0.00	0.01	0.00	0.00
GSM437289	0.00	0.01	0.03	0.18	0.00	0.14	0.05	0.06	0.00	0.00	0.02	0.03	0.00	0.17	0.13	0.12	0.04	0.00	0.00	0.03	0.00	0.00
GSM437290	0.01	0.01	0.02	0.13	0.00	0.17	0.12	0.04	0.00	0.00	0.09	0.00	0.03	0.13	0.04	0.07	0.00	0.05	0.00	0.11	0.00	0.00
GSM437291	0.02	0.03	0.01	0.18	0.00	0.07	0.02	0.13	0.10	0.05	0.00	0.00	0.00	0.08	0.07	0.04	0.07	0.07	0.00	0.06	0.00	0.00
GSM437292	0.00	0.00	0.05	0.16	0.00	0.19	0.06	0.03	0.01	0.00	0.05	0.00	0.01	0.03	0.05	0.11	0.14	0.04	0.00	0.06	0.00	0.00
GSM437294	0.00	0.03	0.01	0.10	0.00	0.07	0.16	0.09	0.00	0.01	0.07	0.00	0.00	0.13	0.18	0.09	0.03	0.02	0.00	0.01	0.00	0.00
GSM437296	0.02	0.05	0.01	0.15	0.00	0.08	0.14	0.07	0.00	0.00	0.02	0.00	0.00	0.05	0.10	0.10	0.14	0.00	0.06	0.00	0.00	0.00
GSM437297	0.01	0.00	0.02	0.37	0.00	0.00	0.20	0.03	0.00	0.02	0.09	0.00	0.00	0.04	0.07	0.08	0.02	0.00	0.04	0.00	0.00	0.00
GSM437298	0.00	0.03	0.02	0.23	0.00	0.11	0.19	0.03	0.00	0.00	0.05	0.00	0.02	0.07	0.04	0.09	0.07	0.00	0.00	0.04	0.00	0.00
GSM437299	0.02	0.00	0.09	0.07	0.00	0.31	0.03	0.03	0.00	0.00	0.07	0.00	0.00	0.09	0.09	0.04	0.01	0.01	0.00	0.11	0.03	0.00
GSM437300	0.00	0.00	0.06	0.10	0.00	0.16	0.04	0.03	0.03	0.00	0.06	0.00	0.00	0.26	0.06	0.15	0.04	0.00	0.00	0.01	0.00	0.01
GSM437301	0.00	0.04	0.10	0.06	0.00	0.11	0.02	0.04	0.01	0.00	0.03	0.00	0.00	0.28	0.11	0.15	0.00	0.00	0.00	0.04	0.00	0.01
GSM437303	0.00	0.09	0.08	0.09	0.00	0.17	0.03	0.05	0.05	0.00	0.09	0.00	0.00	0.11	0.03	0.09	0.01	0.02	0.00	0.08	0.00	0.00
GSM437304	0.00	0.01	0.08	0.09	0.00	0.24	0.01	0.00	0.02	0.00	0.05	0.00	0.02	0.00	0.03	0.10	0.08	0.00	0.17	0.05	0.05	0.00
GSM437306	0.02	0.00	0.10	0.03	0.00	0.20	0.01	0.01	0.00	0.04	0.05	0.00	0.00	0.16	0.05	0.12	0.00	0.02	0.00	0.06	0.05	0.08
GSM437307	0.02	0.00	0.03	0.27	0.00	0.00	0.22	0.08	0.05	0.00	0.00	0.00	0.00	0.04	0.12	0.11	0.04	0.00	0.00	0.00	0.00	0.02
GSM437309	0.05	0.00	0.11	0.20	0.00	0.01	0.01	0.12	0.05	0.00	0.05	0.00	0.00	0.06	0.17	0.09	0.07	0.00	0.00	0.00	0.00	0.00

GSM437310	0.00	0.06	0.02	0.17	0.00	0.10	0.10	0.00	0.08	0.00	0.09	0.00	0.00	0.10	0.05	0.13	0.01	0.00	0.00	0.08	0.00	0.01
GSM437311	0.00	0.07	0.01	0.18	0.00	0.15	0.02	0.04	0.08	0.00	0.05	0.00	0.03	0.05	0.09	0.05	0.07	0.03	0.00	0.07	0.00	0.01
GSM437312	0.01	0.02	0.02	0.15	0.00	0.26	0.03	0.05	0.03	0.00	0.07	0.00	0.01	0.09	0.05	0.09	0.05	0.02	0.00	0.04	0.00	0.00
GSM437313	0.01	0.00	0.03	0.16	0.00	0.06	0.12	0.07	0.00	0.00	0.06	0.02	0.00	0.05	0.17	0.08	0.08	0.00	0.00	0.04	0.02	0.03
GSM437314	0.00	0.05	0.00	0.10	0.00	0.05	0.02	0.05	0.02	0.01	0.05	0.00	0.00	0.23	0.06	0.10	0.04	0.00	0.00	0.16	0.00	0.06
GSM437315	0.03	0.00	0.04	0.07	0.00	0.14	0.06	0.00	0.00	0.01	0.04	0.00	0.00	0.08	0.02	0.16	0.02	0.02	0.00	0.17	0.05	0.10
GSM437323	0.00	0.01	0.24	0.09	0.00	0.15	0.00	0.05	0.04	0.00	0.02	0.00	0.00	0.13	0.04	0.12	0.05	0.01	0.00	0.04	0.01	0.01
GSM437324	0.02	0.03	0.06	0.22	0.00	0.22	0.00	0.03	0.04	0.00	0.00	0.00	0.00	0.11	0.05	0.11	0.04	0.00	0.00	0.07	0.00	0.00
GSM971957	0.02	0.00	0.02	0.09	0.00	0.09	0.12	0.02	0.03	0.00	0.04	0.04	0.00	0.18	0.08	0.05	0.03	0.00	0.00	0.13	0.02	0.05
GSM971958	0.00	0.01	0.07	0.10	0.00	0.07	0.02	0.00	0.01	0.01	0.04	0.00	0.00	0.35	0.03	0.16	0.00	0.02	0.00	0.05	0.00	0.06
GSM971959	0.01	0.03	0.05	0.11	0.00	0.06	0.00	0.05	0.02	0.00	0.00	0.03	0.02	0.11	0.03	0.12	0.00	0.02	0.00	0.33	0.01	0.00
GSM971960	0.07	0.00	0.14	0.10	0.00	0.19	0.14	0.03	0.00	0.00	0.04	0.00	0.03	0.09	0.04	0.09	0.02	0.00	0.02	0.00	0.00	0.00
GSM971961	0.00	0.00	0.05	0.04	0.00	0.05	0.02	0.01	0.01	0.01	0.00	0.00	0.13	0.44	0.03	0.15	0.00	0.00	0.00	0.05	0.00	0.01
GSM971962	0.08	0.00	0.06	0.01	0.00	0.10	0.03	0.05	0.02	0.01	0.02	0.00	0.00	0.27	0.10	0.17	0.01	0.00	0.00	0.04	0.00	0.02
GSM971963	0.05	0.00	0.03	0.29	0.00	0.00	0.15	0.04	0.01	0.02	0.05	0.00	0.00	0.08	0.14	0.09	0.02	0.00	0.03	0.00	0.00	0.01
GSM971964	0.00	0.04	0.12	0.07	0.00	0.26	0.08	0.03	0.00	0.00	0.06	0.00	0.01	0.06	0.03	0.08	0.10	0.03	0.00	0.03	0.02	0.00
GSM971965	0.01	0.01	0.04	0.08	0.00	0.16	0.05	0.00	0.04	0.00	0.07	0.00	0.00	0.13	0.05	0.16	0.08	0.02	0.00	0.09	0.00	0.00
GSM971966	0.01	0.00	0.03	0.11	0.00	0.16	0.07	0.05	0.00	0.00	0.02	0.02	0.00	0.11	0.07	0.16	0.03	0.03	0.01	0.02	0.00	0.11
GSM971968	0.00	0.00	0.01	0.03	0.00	0.13	0.03	0.01	0.03	0.00	0.01	0.00	0.13	0.19	0.05	0.12	0.17	0.03	0.06	0.00	0.01	0.00
GSM971969	0.03	0.01	0.05	0.14	0.00	0.00	0.06	0.08	0.05	0.09	0.00	0.00	0.00	0.11	0.14	0.13	0.10	0.00	0.00	0.00	0.00	0.00
GSM971970	0.00	0.02	0.00	0.18	0.00	0.15	0.09	0.07	0.01	0.00	0.04	0.02	0.03	0.16	0.08	0.06	0.01	0.02	0.00	0.04	0.00	0.03
GSM971971	0.00	0.00	0.04	0.27	0.00	0.01	0.12	0.05	0.01	0.00	0.03	0.00	0.00	0.10	0.15	0.06	0.13	0.00	0.00	0.02	0.01	0.00
GSM971972	0.00	0.05	0.04	0.30	0.00	0.00	0.07	0.09	0.01	0.00	0.05	0.01	0.00	0.07	0.11	0.16	0.05	0.00	0.00	0.00	0.00	0.00
GSM971973	0.00	0.04	0.00	0.13	0.00	0.03	0.01	0.12	0.01	0.00	0.02	0.03	0.00	0.20	0.07	0.14	0.07	0.00	0.00	0.07	0.00	0.06
GSM971974	0.02	0.00	0.06	0.12	0.00	0.34	0.00	0.02	0.02	0.00	0.07	0.00	0.02	0.11	0.02	0.05	0.06	0.01	0.00	0.07	0.00	0.00
GSM971975	0.00	0.07	0.08	0.08	0.00	0.16	0.08	0.01	0.00	0.00	0.07	0.00	0.01	0.20	0.02	0.05	0.00	0.03	0.00	0.10	0.00	0.04
GSM971976	0.00	0.07	0.02	0.16	0.00	0.22	0.17	0.00	0.00	0.00	0.06	0.00	0.03	0.08	0.06	0.08	0.02	0.01	0.01	0.00	0.00	0.00
GSM971977	0.01	0.01	0.02	0.04	0.00	0.15	0.01	0.09	0.05	0.00	0.02	0.00	0.00	0.13	0.16	0.12	0.05	0.00	0.00	0.05	0.00	0.08
GSM971978	0.03	0.00	0.02	0.18	0.00	0.00	0.16	0.08	0.00	0.00	0.07	0.04	0.00	0.04	0.14	0.12	0.07	0.00	0.04	0.00	0.01	0.00
GSM971979	0.00	0.00	0.00	0.00	0.00	0.14	0.00	0.02	0.00	0.00	0.01	0.00	0.00	0.32	0.03	0.02	0.00	0.02	0.00	0.32	0.01	0.10
GSM971980	0.01	0.02	0.06	0.09	0.00	0.25	0.05	0.00	0.00	0.06	0.00	0.00	0.00	0.09	0.15	0.08	0.00	0.02	0.00	0.03	0.06	0.04
GSM971981	0.00	0.00	0.05	0.30	0.00	0.00	0.09	0.05	0.04	0.01	0.00	0.01	0.00	0.06	0.14	0.22	0.01	0.00	0.00	0.01	0.00	0.02
GSM971982	0.00	0.00	0.02	0.08	0.00	0.03	0.07	0.05	0.01	0.00	0.07	0.00	0.00	0.13	0.19	0.14	0.03	0.00	0.00	0.10	0.00	0.07
GSM971983	0.06	0.00	0.03	0.10	0.00	0.12	0.07	0.06	0.02	0.00	0.01	0.02	0.00	0.18	0.11	0.07	0.03	0.00	0.00	0.02	0.03	0.07
GSM971984	0.00	0.00	0.05	0.19	0.00	0.05	0.16	0.03	0.00	0.01	0.05	0.00	0.00	0.10	0.17	0.10	0.07	0.00	0.00	0.00	0.00	0.00
GSM971985	0.02	0.00	0.12	0.16	0.00	0.29	0.00	0.04	0.04	0.00	0.04	0.00	0.02	0.07	0.03	0.04	0.04	0.00	0.00	0.09	0.00	0.00
GSM971986	0.03	0.02	0.05	0.14	0.00	0.03	0.06	0.06	0.07	0.00	0.11	0.00	0.01	0.13	0.08	0.07	0.01	0.00	0.00	0.13	0.00	0.00
GSM971987	0.00	0.04	0.03	0.10	0.00	0.18	0.01	0.00	0.00	0.05	0.00	0.00	0.00	0.01	0.05	0.10	0.12	0.00	0.21	0.00	0.10	0.00

GSM971988	0.01	0.12	0.01	0.13	0.00	0.25	0.00	0.00	0.07	0.00	0.00	0.02	0.02	0.14	0.08	0.07	0.00	0.06	0.00	0.04	0.00	0.00
GSM971989	0.02	0.00	0.08	0.13	0.00	0.32	0.01	0.07	0.00	0.00	0.00	0.05	0.00	0.05	0.06	0.05	0.08	0.03	0.05	0.00	0.00	0.00
GSM971990	0.00	0.04	0.08	0.09	0.00	0.19	0.12	0.00	0.00	0.00	0.06	0.00	0.00	0.08	0.08	0.09	0.06	0.00	0.06	0.00	0.02	0.03
GSM971991	0.00	0.00	0.06	0.12	0.00	0.09	0.11	0.04	0.00	0.00	0.00	0.00	0.00	0.31	0.09	0.04	0.00	0.00	0.00	0.11	0.00	0.03
GSM971992	0.01	0.00	0.15	0.10	0.00	0.18	0.17	0.00	0.01	0.00	0.01	0.00	0.00	0.15	0.08	0.07	0.03	0.03	0.00	0.01	0.00	0.00
GSM971993	0.03	0.00	0.07	0.18	0.00	0.13	0.03	0.08	0.00	0.00	0.02	0.00	0.00	0.09	0.16	0.05	0.07	0.00	0.00	0.09	0.00	0.01
GSM971994	0.00	0.00	0.05	0.06	0.00	0.32	0.01	0.01	0.02	0.00	0.06	0.01	0.00	0.13	0.02	0.12	0.08	0.05	0.00	0.05	0.00	0.01
GSM971995	0.00	0.00	0.07	0.13	0.00	0.13	0.03	0.03	0.05	0.00	0.03	0.00	0.00	0.03	0.08	0.14	0.19	0.00	0.04	0.03	0.03	0.00
GSM971996	0.00	0.00	0.04	0.17	0.00	0.00	0.08	0.02	0.03	0.00	0.04	0.00	0.00	0.15	0.09	0.15	0.10	0.03	0.00	0.07	0.01	0.02
GSM971997	0.17	0.00	0.01	0.26	0.00	0.00	0.14	0.05	0.00	0.01	0.01	0.00	0.00	0.05	0.16	0.06	0.03	0.00	0.03	0.00	0.01	0.00
GSM971998	0.05	0.00	0.10	0.14	0.00	0.11	0.06	0.00	0.00	0.00	0.07	0.00	0.00	0.03	0.11	0.17	0.06	0.00	0.05	0.00	0.05	0.00
GSM971999	0.03	0.01	0.06	0.09	0.00	0.16	0.09	0.08	0.00	0.03	0.04	0.00	0.00	0.10	0.07	0.10	0.03	0.00	0.00	0.05	0.06	0.00
GSM972000	0.00	0.04	0.01	0.23	0.00	0.05	0.00	0.04	0.01	0.00	0.00	0.02	0.00	0.17	0.10	0.25	0.06	0.00	0.00	0.03	0.00	0.00
GSM972001	0.12	0.00	0.12	0.09	0.00	0.15	0.17	0.03	0.00	0.00	0.05	0.00	0.01	0.10	0.06	0.03	0.04	0.00	0.00	0.04	0.00	0.00
GSM972002	0.05	0.00	0.02	0.11	0.00	0.16	0.00	0.00	0.00	0.00	0.03	0.00	0.00	0.15	0.04	0.06	0.01	0.02	0.00	0.25	0.00	0.09
GSM972003	0.00	0.02	0.17	0.14	0.00	0.18	0.07	0.06	0.01	0.00	0.06	0.00	0.01	0.06	0.10	0.07	0.02	0.00	0.00	0.06	0.00	0.00
GSM972004	0.02	0.00	0.11	0.17	0.00	0.20	0.05	0.04	0.00	0.00	0.04	0.00	0.00	0.09	0.06	0.10	0.02	0.01	0.00	0.09	0.00	0.00
GSM972005	0.00	0.06	0.00	0.11	0.00	0.18	0.00	0.04	0.05	0.00	0.05	0.00	0.00	0.04	0.01	0.04	0.03	0.00	0.00	0.37	0.00	0.00
GSM972006	0.03	0.00	0.01	0.15	0.00	0.10	0.04	0.06	0.00	0.00	0.03	0.00	0.00	0.18	0.09	0.09	0.06	0.00	0.00	0.09	0.09	0.00
GSM972007	0.02	0.00	0.06	0.13	0.00	0.13	0.01	0.03	0.02	0.00	0.00	0.02	0.07	0.12	0.06	0.13	0.12	0.00	0.05	0.00	0.00	0.00
GSM972008	0.01	0.00	0.00	0.04	0.00	0.05	0.01	0.00	0.02	0.00	0.05	0.00	0.08	0.14	0.04	0.12	0.06	0.01	0.00	0.09	0.00	0.29
GSM972009	0.00	0.01	0.11	0.14	0.00	0.18	0.02	0.01	0.02	0.00	0.00	0.02	0.00	0.07	0.04	0.09	0.09	0.01	0.17	0.02	0.00	0.00
GSM972010	0.02	0.02	0.02	0.02	0.00	0.09	0.09	0.03	0.03	0.00	0.03	0.00	0.00	0.24	0.02	0.08	0.02	0.04	0.00	0.20	0.02	0.03
GSM972011	0.08	0.00	0.08	0.23	0.00	0.02	0.04	0.03	0.03	0.00	0.01	0.00	0.03	0.02	0.17	0.15	0.04	0.00	0.02	0.05	0.00	0.00
GSM972012	0.00	0.10	0.05	0.00	0.00	0.20	0.00	0.07	0.01	0.00	0.05	0.00	0.00	0.13	0.04	0.04	0.00	0.08	0.00	0.21	0.00	0.00
GSM972013	0.00	0.04	0.01	0.07	0.00	0.20	0.09	0.00	0.00	0.00	0.05	0.00	0.00	0.16	0.05	0.10	0.00	0.03	0.00	0.13	0.00	0.06
GSM972014	0.03	0.00	0.01	0.07	0.00	0.14	0.00	0.03	0.03	0.00	0.02	0.00	0.00	0.14	0.07	0.09	0.04	0.02	0.00	0.22	0.05	0.03
GSM972015	0.02	0.00	0.00	0.13	0.00	0.19	0.01	0.04	0.02	0.00	0.11	0.00	0.00	0.16	0.12	0.06	0.01	0.00	0.00	0.07	0.00	0.05
GSM972016	0.03	0.00	0.07	0.07	0.00	0.08	0.00	0.06	0.01	0.00	0.03	0.00	0.00	0.24	0.03	0.08	0.00	0.04	0.00	0.21	0.00	0.06
GSM972017	0.01	0.01	0.04	0.19	0.00	0.01	0.06	0.07	0.05	0.00	0.04	0.00	0.00	0.26	0.04	0.11	0.02	0.00	0.00	0.08	0.00	0.00
GSM972018	0.01	0.00	0.04	0.04	0.00	0.16	0.02	0.06	0.00	0.00	0.00	0.04	0.00	0.05	0.21	0.16	0.07	0.00	0.00	0.06	0.01	0.07
GSM972019	0.08	0.00	0.02	0.14	0.00	0.07	0.05	0.10	0.02	0.14	0.00	0.00	0.00	0.09	0.12	0.07	0.07	0.00	0.01	0.02	0.00	0.01
GSM972020	0.04	0.04	0.13	0.06	0.00	0.26	0.07	0.00	0.01	0.00	0.03	0.00	0.00	0.10	0.09	0.03	0.06	0.03	0.00	0.04	0.00	0.02
GSM972021	0.00	0.04	0.00	0.09	0.00	0.21	0.00	0.08	0.00	0.00	0.06	0.00	0.00	0.22	0.11	0.11	0.02	0.00	0.00	0.05	0.00	0.00
GSM972022	0.03	0.06	0.03	0.13	0.00	0.20	0.04	0.02	0.00	0.00	0.01	0.00	0.00	0.13	0.14	0.08	0.06	0.00	0.00	0.06	0.00	0.00
GSM972023	0.13	0.00	0.03	0.04	0.00	0.14	0.00	0.01	0.00	0.00	0.06	0.00	0.00	0.25	0.03	0.08	0.00	0.01	0.00	0.22	0.00	0.00
GSM972024	0.00	0.05	0.02	0.09	0.00	0.24	0.16	0.00	0.01	0.00	0.06	0.00	0.02	0.12	0.03	0.08	0.02	0.03	0.00	0.10	0.00	0.00
GSM972025	0.00	0.01	0.14	0.24	0.00	0.14	0.00	0.00	0.01	0.00	0.00	0.02	0.00	0.00	0.06	0.13	0.11	0.00	0.00	0.10	0.05	0.00

GSM972026	0.07	0.00	0.01	0.17	0.00	0.03	0.12	0.00	0.00	0.00	0.00	0.00	0.02	0.05	0.08	0.21	0.14	0.00	0.00	0.06	0.01	0.02
GSM972027	0.02	0.03	0.01	0.13	0.00	0.12	0.17	0.00	0.03	0.00	0.03	0.03	0.00	0.25	0.03	0.13	0.03	0.00	0.00	0.01	0.00	0.00
GSM972028	0.04	0.00	0.10	0.18	0.00	0.16	0.01	0.01	0.02	0.00	0.06	0.00	0.00	0.02	0.05	0.10	0.15	0.01	0.00	0.04	0.00	0.05
GSM972029	0.02	0.00	0.06	0.16	0.00	0.07	0.06	0.02	0.03	0.00	0.08	0.00	0.00	0.15	0.03	0.15	0.02	0.01	0.02	0.11	0.00	0.00
GSM972030	0.00	0.01	0.16	0.09	0.00	0.18	0.00	0.00	0.00	0.00	0.02	0.00	0.04	0.18	0.06	0.13	0.00	0.06	0.01	0.02	0.00	0.03
GSM972031	0.00	0.01	0.14	0.05	0.00	0.20	0.05	0.04	0.00	0.00	0.05	0.00	0.00	0.18	0.08	0.09	0.05	0.00	0.00	0.07	0.00	0.00
GSM972032	0.03	0.00	0.06	0.03	0.00	0.25	0.13	0.04	0.00	0.00	0.08	0.00	0.00	0.16	0.04	0.11	0.03	0.02	0.00	0.03	0.00	0.00
GSM972033	0.00	0.08	0.02	0.15	0.00	0.26	0.13	0.00	0.02	0.00	0.02	0.03	0.01	0.08	0.03	0.11	0.02	0.02	0.03	0.00	0.00	0.00
GSM972034	0.00	0.12	0.06	0.08	0.00	0.24	0.04	0.00	0.04	0.00	0.05	0.00	0.00	0.07	0.06	0.05	0.09	0.05	0.00	0.06	0.00	0.00
GSM972035	0.04	0.00	0.02	0.10	0.00	0.17	0.20	0.01	0.00	0.00	0.08	0.00	0.04	0.05	0.04	0.15	0.01	0.05	0.00	0.03	0.00	0.00
GSM972036	0.00	0.08	0.07	0.17	0.00	0.18	0.05	0.03	0.02	0.00	0.05	0.00	0.00	0.10	0.08	0.09	0.04	0.01	0.00	0.02	0.00	0.01
GSM972037	0.04	0.03	0.05	0.18	0.00	0.11	0.00	0.07	0.02	0.00	0.01	0.00	0.00	0.12	0.05	0.08	0.05	0.05	0.00	0.13	0.01	0.00
GSM972038	0.01	0.02	0.00	0.11	0.00	0.09	0.06	0.06	0.01	0.00	0.07	0.00	0.01	0.12	0.06	0.10	0.04	0.11	0.00	0.11	0.01	0.00
GSM972039	0.06	0.00	0.14	0.13	0.00	0.08	0.00	0.08	0.00	0.05	0.00	0.01	0.12	0.04	0.04	0.02	0.03	0.01	0.09	0.04	0.02	0.02
GSM972040	0.03	0.00	0.03	0.06	0.00	0.20	0.07	0.02	0.01	0.00	0.05	0.00	0.00	0.16	0.02	0.07	0.18	0.07	0.00	0.02	0.00	0.01
GSM972041	0.00	0.03	0.01	0.11	0.00	0.14	0.07	0.02	0.00	0.00	0.03	0.00	0.00	0.22	0.08	0.11	0.04	0.01	0.00	0.10	0.00	0.03
GSM972042	0.03	0.00	0.17	0.07	0.00	0.27	0.00	0.00	0.07	0.00	0.03	0.00	0.02	0.12	0.08	0.00	0.07	0.03	0.00	0.03	0.00	0.00
GSM972043	0.02	0.00	0.00	0.03	0.00	0.30	0.17	0.00	0.00	0.00	0.14	0.00	0.03	0.17	0.03	0.04	0.03	0.02	0.00	0.02	0.00	0.00
GSM972044	0.00	0.03	0.01	0.10	0.00	0.25	0.00	0.00	0.07	0.00	0.06	0.00	0.00	0.13	0.03	0.09	0.03	0.02	0.00	0.13	0.00	0.05
GSM972045	0.15	0.00	0.00	0.10	0.00	0.20	0.00	0.11	0.02	0.00	0.02	0.00	0.00	0.12	0.03	0.12	0.00	0.04	0.00	0.08	0.01	0.00
GSM972046	0.03	0.00	0.03	0.05	0.00	0.23	0.08	0.00	0.00	0.09	0.02	0.00	0.00	0.11	0.06	0.19	0.08	0.00	0.00	0.01	0.00	0.02
GSM972047	0.03	0.00	0.01	0.16	0.00	0.09	0.18	0.01	0.00	0.00	0.04	0.01	0.00	0.21	0.03	0.12	0.06	0.00	0.04	0.00	0.00	0.01
GSM972048	0.04	0.00	0.07	0.10	0.00	0.24	0.07	0.01	0.00	0.00	0.03	0.00	0.03	0.16	0.04	0.11	0.01	0.02	0.00	0.08	0.00	0.00
GSM972049	0.00	0.06	0.00	0.12	0.00	0.23	0.00	0.02	0.04	0.00	0.07	0.00	0.00	0.16	0.02	0.09	0.02	0.05	0.00	0.10	0.00	0.01
GSM972050	0.00	0.01	0.21	0.15	0.00	0.31	0.00	0.05	0.00	0.00	0.02	0.00	0.00	0.02	0.02	0.06	0.07	0.04	0.00	0.04	0.00	0.00
GSM972051	0.00	0.06	0.00	0.05	0.00	0.17	0.03	0.03	0.00	0.00	0.03	0.00	0.01	0.18	0.07	0.19	0.02	0.05	0.02	0.07	0.00	0.02
GSM972052	0.01	0.00	0.11	0.09	0.00	0.16	0.10	0.07	0.00	0.00	0.05	0.00	0.00	0.12	0.11	0.11	0.01	0.01	0.05	0.00	0.00	0.00
GSM972053	0.00	0.05	0.02	0.08	0.00	0.16	0.14	0.05	0.00	0.00	0.05	0.00	0.00	0.11	0.16	0.09	0.05	0.05	0.00	0.00	0.00	0.00
GSM972054	0.02	0.01	0.00	0.09	0.00	0.11	0.00	0.05	0.01	0.00	0.06	0.00	0.00	0.26	0.13	0.15	0.03	0.00	0.00	0.05	0.00	0.03
GSM972055	0.01	0.00	0.04	0.24	0.00	0.03	0.06	0.06	0.01	0.00	0.04	0.00	0.00	0.12	0.11	0.16	0.04	0.00	0.00	0.07	0.00	0.02
GSM972056	0.01	0.00	0.03	0.29	0.00	0.10	0.00	0.03	0.03	0.00	0.02	0.00	0.00	0.10	0.04	0.13	0.01	0.04	0.00	0.16	0.01	0.00
GSM972057	0.00	0.02	0.02	0.26	0.00	0.03	0.07	0.03	0.01	0.00	0.05	0.00	0.00	0.00	0.04	0.20	0.05	0.00	0.00	0.16	0.01	0.06
GSM972058	0.03	0.00	0.02	0.06	0.00	0.15	0.02	0.03	0.00	0.00	0.03	0.03	0.00	0.13	0.09	0.09	0.01	0.03	0.00	0.10	0.00	0.19
GSM972059	0.10	0.00	0.07	0.22	0.00	0.00	0.05	0.02	0.01	0.00	0.11	0.00	0.00	0.10	0.07	0.09	0.03	0.00	0.00	0.12	0.00	0.02
GSM972060	0.00	0.00	0.01	0.14	0.00	0.06	0.11	0.07	0.01	0.00	0.00	0.05	0.00	0.14	0.14	0.10	0.12	0.00	0.04	0.00	0.00	0.00
GSM972061	0.00	0.05	0.09	0.26	0.00	0.00	0.12	0.02	0.03	0.00	0.01	0.00	0.00	0.14	0.06	0.14	0.00	0.00	0.00	0.06	0.00	0.01
GSM972062	0.00	0.01	0.09	0.15	0.00	0.10	0.04	0.02	0.01	0.00	0.01	0.00	0.01	0.09	0.03	0.12	0.19	0.01	0.00	0.12	0.01	0.01
GSM972063	0.01	0.01	0.06	0.12	0.00	0.10	0.04	0.03	0.03	0.00	0.01	0.00	0.00	0.14	0.09	0.09	0.13	0.00	0.00	0.11	0.04	0.00

GSM972064	0.02	0.00	0.09	0.07	0.00	0.06	0.01	0.02	0.00	0.01	0.03	0.00	0.00	0.15	0.06	0.10	0.02	0.00	0.00	0.28	0.00	0.07
GSM972065	0.03	0.00	0.08	0.15	0.00	0.14	0.08	0.03	0.00	0.00	0.02	0.01	0.00	0.13	0.09	0.14	0.04	0.00	0.00	0.07	0.00	0.00
GSM972066	0.09	0.00	0.12	0.07	0.00	0.13	0.07	0.00	0.00	0.00	0.05	0.00	0.00	0.19	0.05	0.09	0.01	0.00	0.00	0.07	0.00	0.04
GSM972067	0.04	0.00	0.08	0.19	0.00	0.06	0.06	0.06	0.02	0.00	0.01	0.02	0.01	0.12	0.12	0.11	0.09	0.00	0.01	0.02	0.00	0.00
GSM972068	0.00	0.02	0.02	0.10	0.00	0.03	0.00	0.00	0.04	0.01	0.00	0.00	0.01	0.31	0.05	0.16	0.09	0.00	0.00	0.10	0.02	0.03
GSM972069	0.01	0.00	0.13	0.12	0.00	0.16	0.07	0.04	0.01	0.00	0.04	0.00	0.00	0.16	0.04	0.09	0.01	0.02	0.00	0.04	0.03	0.03
GSM972070	0.02	0.00	0.17	0.07	0.00	0.18	0.00	0.00	0.01	0.04	0.00	0.00	0.00	0.05	0.04	0.13	0.11	0.00	0.09	0.02	0.05	0.02
GSM972071	0.00	0.02	0.02	0.07	0.00	0.17	0.00	0.00	0.06	0.00	0.00	0.00	0.00	0.37	0.04	0.09	0.04	0.01	0.00	0.10	0.02	0.00
GSM972072	0.02	0.00	0.02	0.17	0.00	0.00	0.11	0.06	0.00	0.18	0.00	0.03	0.00	0.10	0.12	0.10	0.02	0.01	0.05	0.00	0.00	0.01
GSM972073	0.01	0.01	0.07	0.05	0.00	0.15	0.07	0.04	0.00	0.03	0.00	0.00	0.00	0.21	0.10	0.08	0.06	0.01	0.00	0.07	0.00	0.03
GSM972074	0.00	0.01	0.09	0.10	0.00	0.15	0.11	0.02	0.00	0.00	0.02	0.00	0.00	0.15	0.07	0.08	0.12	0.00	0.00	0.04	0.00	0.03
GSM972075	0.03	0.00	0.06	0.14	0.00	0.09	0.08	0.04	0.00	0.00	0.00	0.02	0.00	0.19	0.15	0.13	0.03	0.00	0.00	0.05	0.00	0.00
GSM972076	0.04	0.00	0.04	0.17	0.00	0.06	0.10	0.07	0.00	0.00	0.03	0.04	0.00	0.15	0.12	0.09	0.02	0.00	0.00	0.03	0.00	0.04
GSM972077	0.05	0.00	0.10	0.19	0.00	0.02	0.01	0.05	0.08	0.00	0.05	0.00	0.00	0.06	0.03	0.08	0.15	0.00	0.00	0.13	0.00	0.00
GSM972078	0.01	0.00	0.02	0.06	0.00	0.09	0.10	0.03	0.01	0.00	0.06	0.00	0.00	0.21	0.09	0.12	0.00	0.00	0.00	0.17	0.00	0.04
GSM972079	0.12	0.00	0.04	0.20	0.00	0.00	0.05	0.03	0.05	0.00	0.09	0.00	0.03	0.06	0.03	0.13	0.08	0.01	0.00	0.03	0.00	0.04
GSM972080	0.01	0.03	0.02	0.02	0.00	0.18	0.00	0.01	0.03	0.00	0.06	0.00	0.00	0.25	0.10	0.05	0.02	0.00	0.00	0.17	0.00	0.06
GSM972081	0.02	0.01	0.11	0.12	0.00	0.12	0.15	0.00	0.03	0.00	0.07	0.00	0.03	0.01	0.07	0.08	0.06	0.00	0.00	0.08	0.01	0.02
GSM972082	0.05	0.00	0.07	0.13	0.00	0.20	0.01	0.00	0.02	0.03	0.02	0.00	0.00	0.11	0.05	0.10	0.02	0.02	0.00	0.13	0.00	0.02
GSM972083	0.02	0.02	0.04	0.16	0.00	0.19	0.02	0.06	0.01	0.00	0.00	0.00	0.00	0.02	0.11	0.08	0.11	0.00	0.00	0.07	0.03	0.05
GSM972084	0.04	0.07	0.08	0.02	0.00	0.29	0.00	0.04	0.01	0.08	0.00	0.00	0.00	0.00	0.04	0.16	0.03	0.00	0.05	0.05	0.03	0.00
GSM972085	0.02	0.00	0.14	0.15	0.00	0.16	0.03	0.01	0.00	0.00	0.09	0.00	0.00	0.09	0.04	0.07	0.00	0.03	0.00	0.12	0.00	0.05
GSM972086	0.00	0.09	0.03	0.17	0.00	0.08	0.03	0.16	0.00	0.00	0.01	0.01	0.00	0.10	0.13	0.10	0.03	0.04	0.00	0.01	0.00	0.00
GSM972087	0.03	0.00	0.12	0.14	0.00	0.19	0.07	0.06	0.00	0.00	0.00	0.00	0.00	0.11	0.08	0.04	0.01	0.03	0.00	0.12	0.00	0.00
GSM972088	0.31	0.00	0.06	0.08	0.00	0.24	0.00	0.05	0.00	0.02	0.00	0.00	0.00	0.00	0.04	0.12	0.00	0.01	0.00	0.05	0.00	0.01
GSM972089	0.12	0.00	0.00	0.06	0.00	0.17	0.00	0.05	0.01	0.00	0.00	0.00	0.02	0.00	0.11	0.22	0.11	0.00	0.09	0.02	0.00	0.01
GSM972090	0.01	0.04	0.00	0.13	0.00	0.25	0.05	0.04	0.03	0.00	0.04	0.02	0.01	0.04	0.04	0.16	0.07	0.03	0.00	0.03	0.00	0.00
GSM972091	0.00	0.08	0.02	0.16	0.00	0.20	0.17	0.04	0.01	0.00	0.03	0.00	0.00	0.07	0.06	0.05	0.03	0.02	0.00	0.05	0.00	0.01
GSM972092	0.00	0.01	0.02	0.07	0.00	0.10	0.03	0.09	0.03	0.00	0.00	0.01	0.00	0.18	0.18	0.12	0.12	0.00	0.02	0.01	0.00	0.02
GSM972093	0.02	0.01	0.13	0.02	0.00	0.22	0.08	0.04	0.00	0.00	0.03	0.00	0.00	0.21	0.06	0.11	0.01	0.02	0.04	0.00	0.01	0.02
GSM972094	0.00	0.00	0.14	0.26	0.00	0.05	0.09	0.01	0.00	0.00	0.04	0.00	0.00	0.16	0.09	0.07	0.00	0.02	0.00	0.05	0.00	0.02
GSM972095	0.00	0.00	0.01	0.04	0.00	0.15	0.04	0.06	0.01	0.00	0.03	0.00	0.00	0.24	0.15	0.11	0.03	0.02	0.00	0.08	0.01	0.02
GSM972096	0.00	0.07	0.01	0.32	0.00	0.10	0.06	0.04	0.01	0.00	0.03	0.00	0.06	0.06	0.01	0.04	0.04	0.02	0.00	0.13	0.00	0.00
GSM972097	0.00	0.08	0.02	0.23	0.00	0.12	0.00	0.05	0.00	0.00	0.00	0.03	0.01	0.10	0.04	0.21	0.03	0.00	0.08	0.00	0.00	0.00
GSM972098	0.00	0.10	0.01	0.22	0.00	0.22	0.00	0.06	0.07	0.00	0.00	0.05	0.01	0.04	0.02	0.11	0.05	0.03	0.01	0.00	0.00	0.00
GSM972099	0.01	0.00	0.04	0.18	0.00	0.19	0.19	0.00	0.01	0.00	0.08	0.00	0.01	0.05	0.03	0.10	0.05	0.00	0.00	0.07	0.00	0.00
GSM972100	0.00	0.07	0.02	0.11	0.00	0.17	0.02	0.02	0.04	0.00	0.06	0.00	0.00	0.16	0.06	0.07	0.03	0.02	0.00	0.16	0.00	0.00
GSM972101	0.05	0.00	0.08	0.18	0.00	0.23	0.00	0.01	0.00	0.00	0.04	0.00	0.00	0.00	0.04	0.12	0.09	0.01	0.03	0.02	0.09	0.00

GSM972102	0.01	0.04	0.06	0.14	0.00	0.22	0.00	0.04	0.00	0.10	0.00	0.00	0.00	0.00	0.05	0.12	0.05	0.00	0.10	0.00	0.05	0.01
GSM972103	0.00	0.05	0.05	0.28	0.00	0.00	0.04	0.11	0.04	0.00	0.00	0.02	0.00	0.05	0.16	0.10	0.06	0.00	0.03	0.00	0.00	0.00
GSM972104	0.05	0.00	0.10	0.06	0.00	0.16	0.04	0.03	0.00	0.00	0.03	0.00	0.00	0.06	0.06	0.09	0.10	0.00	0.10	0.02	0.10	0.00
GSM972105	0.00	0.04	0.02	0.18	0.00	0.05	0.11	0.07	0.07	0.00	0.06	0.00	0.00	0.14	0.06	0.12	0.02	0.02	0.00	0.04	0.00	0.00
GSM972106	0.01	0.04	0.00	0.17	0.00	0.20	0.05	0.02	0.06	0.00	0.07	0.00	0.01	0.08	0.09	0.10	0.08	0.03	0.00	0.01	0.00	0.00
GSM972107	0.00	0.06	0.07	0.14	0.00	0.00	0.00	0.04	0.06	0.01	0.05	0.00	0.00	0.17	0.08	0.07	0.02	0.01	0.00	0.16	0.02	0.02
GSM972108	0.02	0.00	0.07	0.14	0.00	0.19	0.14	0.03	0.00	0.00	0.02	0.00	0.00	0.13	0.10	0.08	0.06	0.00	0.00	0.03	0.00	0.00
GSM972109	0.00	0.01	0.02	0.14	0.00	0.09	0.02	0.07	0.01	0.00	0.02	0.04	0.00	0.19	0.10	0.12	0.01	0.00	0.00	0.13	0.00	0.02
GSM972110	0.03	0.00	0.12	0.15	0.00	0.27	0.01	0.06	0.00	0.00	0.03	0.00	0.03	0.02	0.07	0.09	0.03	0.03	0.00	0.06	0.01	0.00
GSM972111	0.00	0.03	0.17	0.24	0.00	0.14	0.00	0.01	0.07	0.00	0.03	0.00	0.03	0.05	0.03	0.05	0.04	0.01	0.00	0.10	0.00	0.00
GSM972112	0.01	0.01	0.01	0.17	0.00	0.09	0.00	0.01	0.00	0.06	0.00	0.00	0.00	0.21	0.08	0.13	0.06	0.00	0.00	0.10	0.02	0.03
GSM972113	0.00	0.01	0.08	0.20	0.00	0.21	0.00	0.00	0.00	0.00	0.00	0.00	0.04	0.11	0.03	0.19	0.07	0.00	0.00	0.05	0.00	0.01
GSM972114	0.02	0.00	0.08	0.08	0.00	0.12	0.00	0.02	0.01	0.00	0.01	0.00	0.00	0.14	0.02	0.06	0.02	0.05	0.00	0.23	0.02	0.11
GSM972115	0.04	0.00	0.02	0.16	0.00	0.22	0.03	0.03	0.00	0.00	0.06	0.02	0.00	0.08	0.03	0.12	0.05	0.02	0.00	0.06	0.00	0.05
GSM972116	0.00	0.03	0.13	0.21	0.00	0.13	0.01	0.02	0.04	0.00	0.00	0.00	0.01	0.00	0.02	0.14	0.12	0.00	0.12	0.01	0.00	0.00
GSM972117	0.03	0.00	0.02	0.26	0.00	0.01	0.19	0.05	0.00	0.01	0.04	0.00	0.00	0.09	0.13	0.08	0.03	0.01	0.04	0.00	0.00	0.00
GSM972118	0.00	0.04	0.03	0.15	0.00	0.20	0.00	0.02	0.05	0.00	0.00	0.09	0.01	0.12	0.03	0.07	0.06	0.01	0.08	0.00	0.03	0.01
GSM972119	0.00	0.05	0.02	0.09	0.00	0.22	0.20	0.02	0.01	0.00	0.04	0.00	0.00	0.07	0.07	0.14	0.03	0.02	0.03	0.00	0.00	0.01
GSM972120	0.02	0.00	0.06	0.15	0.00	0.06	0.13	0.06	0.03	0.00	0.01	0.00	0.01	0.15	0.15	0.07	0.03	0.03	0.00	0.02	0.00	0.00
GSM972121	0.05	0.00	0.13	0.07	0.00	0.17	0.00	0.06	0.05	0.00	0.10	0.00	0.00	0.09	0.04	0.04	0.02	0.00	0.00	0.15	0.02	0.00
GSM972122	0.00	0.08	0.01	0.23	0.00	0.13	0.00	0.01	0.07	0.00	0.03	0.00	0.04	0.10	0.04	0.06	0.04	0.01	0.00	0.13	0.02	0.00
GSM972123	0.07	0.00	0.10	0.14	0.00	0.12	0.12	0.09	0.00	0.00	0.02	0.01	0.02	0.13	0.06	0.00	0.07	0.04	0.01	0.00	0.00	0.00
GSM972124	0.00	0.06	0.07	0.11	0.01	0.08	0.07	0.00	0.00	0.00	0.03	0.00	0.00	0.11	0.08	0.18	0.06	0.00	0.12	0.03	0.00	0.00
GSM972125	0.05	0.00	0.09	0.11	0.00	0.18	0.01	0.01	0.00	0.00	0.05	0.00	0.00	0.09	0.04	0.18	0.03	0.01	0.08	0.02	0.04	0.01
GSM972126	0.00	0.18	0.01	0.17	0.00	0.01	0.20	0.02	0.00	0.00	0.02	0.00	0.01	0.12	0.14	0.06	0.02	0.03	0.00	0.00	0.00	0.00
GSM972127	0.00	0.03	0.08	0.18	0.00	0.00	0.04	0.07	0.04	0.00	0.06	0.00	0.00	0.10	0.09	0.19	0.03	0.00	0.00	0.10	0.00	0.00
GSM972128	0.00	0.00	0.05	0.14	0.00	0.14	0.01	0.00	0.01	0.00	0.06	0.00	0.01	0.25	0.07	0.08	0.03	0.00	0.00	0.09	0.00	0.06
GSM972129	0.05	0.00	0.12	0.17	0.00	0.04	0.10	0.10	0.00	0.00	0.00	0.02	0.02	0.07	0.08	0.11	0.04	0.01	0.00	0.05	0.01	0.00
GSM972130	0.05	0.00	0.00	0.14	0.00	0.22	0.04	0.01	0.00	0.00	0.09	0.00	0.00	0.18	0.09	0.06	0.06	0.00	0.00	0.07	0.00	0.00
GSM972131	0.06	0.06	0.04	0.08	0.00	0.10	0.00	0.05	0.02	0.01	0.00	0.00	0.00	0.32	0.08	0.11	0.00	0.01	0.00	0.05	0.00	0.00
GSM972132	0.03	0.00	0.04	0.09	0.00	0.17	0.05	0.01	0.01	0.00	0.02	0.00	0.00	0.28	0.09	0.10	0.02	0.00	0.01	0.06	0.00	0.01
GSM972133	0.02	0.00	0.06	0.07	0.00	0.20	0.00	0.05	0.02	0.00	0.00	0.01	0.00	0.15	0.07	0.20	0.05	0.01	0.00	0.06	0.01	0.02
GSM972134	0.15	0.00	0.05	0.03	0.00	0.14	0.00	0.02	0.00	0.00	0.01	0.00	0.00	0.11	0.06	0.08	0.02	0.03	0.00	0.11	0.11	0.08
GSM972135	0.00	0.05	0.01	0.16	0.00	0.20	0.00	0.02	0.06	0.00	0.05	0.00	0.01	0.06	0.04	0.10	0.09	0.00	0.00	0.16	0.00	0.00
GSM972136	0.02	0.03	0.05	0.07	0.00	0.27	0.01	0.00	0.01	0.00	0.04	0.00	0.00	0.18	0.04	0.09	0.05	0.03	0.00	0.11	0.00	0.01
GSM972137	0.02	0.00	0.08	0.11	0.00	0.23	0.09	0.00	0.00	0.00	0.07	0.00	0.00	0.12	0.08	0.08	0.05	0.00	0.00	0.07	0.00	0.00
GSM972138	0.06	0.00	0.13	0.15	0.00	0.15	0.01	0.00	0.05	0.00	0.05	0.00	0.02	0.06	0.03	0.05	0.02	0.03	0.00	0.18	0.00	0.00
GSM972139	0.03	0.00	0.07	0.07	0.02	0.15	0.13	0.00	0.00	0.00	0.10	0.00	0.00	0.14	0.03	0.11	0.03	0.00	0.00	0.10	0.00	0.01

GSM972140	0.01	0.00	0.00	0.04	0.00	0.15	0.00	0.01	0.03	0.00	0.00	0.00	0.00	0.40	0.09	0.19	0.00	0.00	0.00	0.06	0.00	0.02
GSM972141	0.01	0.00	0.00	0.00	0.00	0.22	0.09	0.00	0.00	0.00	0.09	0.00	0.00	0.18	0.06	0.02	0.02	0.00	0.00	0.16	0.02	0.14
GSM972142	0.00	0.05	0.04	0.06	0.00	0.31	0.04	0.00	0.02	0.00	0.05	0.00	0.01	0.05	0.10	0.09	0.11	0.01	0.01	0.02	0.01	0.02
GSM972143	0.01	0.00	0.04	0.07	0.00	0.18	0.09	0.10	0.00	0.00	0.02	0.03	0.00	0.12	0.19	0.05	0.05	0.00	0.00	0.06	0.00	0.00
GSM972144	0.02	0.00	0.16	0.24	0.00	0.13	0.08	0.01	0.01	0.00	0.06	0.03	0.03	0.05	0.03	0.11	0.03	0.00	0.00	0.01	0.00	0.00
GSM972145	0.08	0.04	0.06	0.01	0.00	0.31	0.00	0.08	0.00	0.00	0.06	0.00	0.00	0.08	0.08	0.13	0.00	0.02	0.00	0.04	0.00	0.00
GSM972146	0.03	0.01	0.08	0.14	0.00	0.16	0.00	0.04	0.03	0.01	0.00	0.00	0.00	0.12	0.12	0.08	0.03	0.00	0.00	0.12	0.00	0.04
GSM972147	0.00	0.02	0.00	0.09	0.00	0.14	0.15	0.07	0.00	0.00	0.01	0.00	0.00	0.13	0.19	0.12	0.02	0.01	0.04	0.00	0.00	0.01
GSM972148	0.03	0.00	0.02	0.17	0.00	0.12	0.14	0.08	0.01	0.00	0.02	0.00	0.00	0.07	0.09	0.14	0.03	0.01	0.03	0.00	0.02	0.01
GSM972149	0.03	0.00	0.08	0.20	0.00	0.00	0.11	0.00	0.05	0.00	0.06	0.00	0.00	0.07	0.04	0.13	0.01	0.03	0.00	0.19	0.00	0.02
GSM972150	0.00	0.04	0.16	0.10	0.00	0.23	0.01	0.00	0.01	0.00	0.03	0.00	0.00	0.08	0.05	0.12	0.02	0.05	0.06	0.00	0.04	0.02
GSM972151	0.02	0.04	0.12	0.11	0.00	0.22	0.05	0.01	0.01	0.00	0.00	0.00	0.00	0.10	0.09	0.09	0.05	0.01	0.00	0.08	0.00	0.00
GSM972152	0.00	0.02	0.10	0.12	0.00	0.15	0.12	0.01	0.00	0.00	0.04	0.00	0.00	0.16	0.03	0.09	0.01	0.01	0.00	0.09	0.02	0.00
GSM972153	0.00	0.03	0.11	0.06	0.00	0.16	0.07	0.09	0.00	0.00	0.01	0.01	0.00	0.09	0.06	0.08	0.01	0.01	0.00	0.03	0.15	0.01
GSM972154	0.00	0.06	0.02	0.11	0.00	0.34	0.00	0.04	0.00	0.00	0.00	0.00	0.00	0.09	0.11	0.09	0.03	0.00	0.00	0.09	0.02	0.00
GSM972155	0.03	0.01	0.04	0.18	0.00	0.15	0.13	0.00	0.00	0.00	0.10	0.00	0.00	0.03	0.00	0.08	0.05	0.05	0.00	0.12	0.03	0.01
GSM972156	0.00	0.00	0.10	0.13	0.00	0.24	0.00	0.00	0.00	0.00	0.00	0.02	0.00	0.09	0.03	0.11	0.07	0.02	0.00	0.12	0.06	0.00
GSM972157	0.00	0.05	0.08	0.11	0.00	0.12	0.11	0.03	0.00	0.00	0.00	0.01	0.00	0.09	0.10	0.15	0.02	0.00	0.09	0.00	0.03	0.02
GSM972158	0.01	0.00	0.07	0.15	0.00	0.00	0.02	0.10	0.01	0.01	0.00	0.01	0.00	0.27	0.13	0.09	0.04	0.00	0.00	0.05	0.00	0.04
GSM972159	0.03	0.05	0.05	0.06	0.00	0.28	0.03	0.10	0.00	0.00	0.05	0.00	0.00	0.08	0.11	0.04	0.04	0.01	0.00	0.07	0.00	0.00
GSM972160	0.00	0.02	0.02	0.00	0.00	0.18	0.02	0.04	0.10	0.02	0.05	0.04	0.00	0.15	0.13	0.10	0.07	0.00	0.03	0.00	0.04	0.00
GSM972161	0.00	0.03	0.02	0.15	0.00	0.25	0.03	0.00	0.03	0.00	0.02	0.01	0.01	0.14	0.03	0.11	0.07	0.03	0.00	0.05	0.00	0.01
GSM972162	0.00	0.02	0.02	0.01	0.00	0.15	0.00	0.03	0.04	0.00	0.06	0.00	0.00	0.23	0.14	0.07	0.01	0.01	0.00	0.18	0.01	0.05
GSM972163	0.01	0.02	0.14	0.08	0.00	0.32	0.02	0.00	0.00	0.00	0.08	0.00	0.00	0.06	0.02	0.10	0.03	0.02	0.00	0.10	0.00	0.00
GSM972164	0.03	0.00	0.05	0.02	0.00	0.14	0.08	0.02	0.00	0.00	0.04	0.00	0.00	0.25	0.07	0.04	0.03	0.01	0.00	0.14	0.00	0.09
GSM972165	0.04	0.00	0.07	0.06	0.00	0.16	0.00	0.06	0.00	0.00	0.03	0.00	0.00	0.10	0.08	0.14	0.07	0.00	0.00	0.13	0.05	0.00
GSM972166	0.02	0.04	0.02	0.03	0.00	0.28	0.00	0.05	0.00	0.00	0.06	0.00	0.00	0.08	0.03	0.05	0.11	0.00	0.00	0.15	0.02	0.04
GSM972167	0.00	0.02	0.13	0.06	0.00	0.30	0.04	0.02	0.01	0.00	0.02	0.00	0.01	0.08	0.06	0.08	0.11	0.00	0.01	0.00	0.03	0.02
GSM972168	0.00	0.00	0.01	0.03	0.00	0.24	0.00	0.00	0.00	0.00	0.05	0.00	0.00	0.11	0.08	0.05	0.01	0.01	0.00	0.22	0.00	0.19
GSM972169	0.01	0.03	0.03	0.04	0.00	0.18	0.11	0.05	0.00	0.00	0.09	0.00	0.00	0.07	0.07	0.06	0.04	0.01	0.00	0.18	0.02	0.00
GSM972170	0.05	0.00	0.09	0.14	0.00	0.08	0.16	0.05	0.00	0.00	0.02	0.00	0.00	0.12	0.06	0.11	0.05	0.02	0.01	0.03	0.00	0.00
GSM972171	0.06	0.00	0.04	0.21	0.00	0.02	0.22	0.00	0.00	0.02	0.01	0.00	0.01	0.02	0.18	0.13	0.03	0.03	0.01	0.00	0.01	0.00
GSM972172	0.00	0.02	0.03	0.17	0.00	0.05	0.00	0.08	0.06	0.00	0.07	0.00	0.01	0.05	0.02	0.09	0.07	0.11	0.00	0.18	0.00	0.00
GSM972173	0.02	0.00	0.01	0.22	0.00	0.08	0.19	0.00	0.00	0.00	0.02	0.01	0.00	0.11	0.05	0.20	0.09	0.00	0.00	0.00	0.00	0.01
GSM972174	0.00	0.06	0.11	0.25	0.00	0.04	0.13	0.05	0.02	0.00	0.01	0.00	0.00	0.10	0.08	0.09	0.00	0.00	0.06	0.00	0.00	0.00
GSM972175	0.00	0.06	0.10	0.34	0.00	0.00	0.14	0.03	0.02	0.00	0.00	0.00	0.03	0.06	0.05	0.07	0.06	0.00	0.00	0.04	0.00	0.00
GSM972176	0.03	0.00	0.03	0.22	0.00	0.07	0.01	0.06	0.05	0.00	0.01	0.03	0.01	0.11	0.17	0.10	0.08	0.00	0.00	0.01	0.00	0.00
GSM972177	0.00	0.08	0.07	0.15	0.00	0.12	0.07	0.00	0.08	0.00	0.05	0.00	0.01	0.13	0.04	0.09	0.06	0.00	0.00	0.04	0.00	0.00

GSM972178	0.00	0.05	0.05	0.23	0.00	0.16	0.00	0.02	0.09	0.00	0.07	0.00	0.03	0.00	0.01	0.10	0.07	0.00	0.00	0.10	0.01	0.00
GSM972179	0.12	0.00	0.15	0.14	0.00	0.03	0.01	0.06	0.01	0.00	0.02	0.00	0.00	0.17	0.07	0.07	0.03	0.00	0.00	0.10	0.00	0.03
GSM972180	0.00	0.00	0.01	0.53	0.00	0.00	0.18	0.04	0.00	0.00	0.06	0.00	0.00	0.00	0.07	0.08	0.02	0.00	0.00	0.01	0.00	0.00
GSM972181	0.00	0.00	0.00	0.01	0.00	0.14	0.00	0.00	0.00	0.00	0.06	0.00	0.19	0.25	0.02	0.02	0.00	0.04	0.00	0.17	0.00	0.10
GSM972182	0.01	0.04	0.08	0.14	0.00	0.16	0.01	0.03	0.03	0.00	0.09	0.00	0.01	0.06	0.02	0.07	0.07	0.01	0.00	0.17	0.00	0.00
GSM972183	0.02	0.03	0.09	0.16	0.00	0.24	0.00	0.06	0.00	0.00	0.02	0.00	0.05	0.09	0.02	0.03	0.04	0.02	0.00	0.13	0.00	0.00
GSM972184	0.00	0.00	0.00	0.13	0.00	0.23	0.03	0.00	0.00	0.00	0.05	0.01	0.00	0.11	0.07	0.17	0.08	0.00	0.00	0.06	0.00	0.06
GSM972185	0.00	0.03	0.04	0.19	0.00	0.12	0.00	0.00	0.09	0.00	0.00	0.02	0.00	0.03	0.05	0.15	0.17	0.00	0.00	0.10	0.00	0.00
GSM972186	0.01	0.02	0.00	0.19	0.00	0.05	0.00	0.04	0.05	0.00	0.00	0.03	0.04	0.00	0.03	0.27	0.07	0.05	0.11	0.01	0.01	0.01
GSM972187	0.02	0.00	0.16	0.05	0.00	0.05	0.00	0.03	0.05	0.00	0.00	0.04	0.00	0.26	0.08	0.15	0.02	0.00	0.00	0.03	0.00	0.04
GSM972188	0.02	0.00	0.03	0.14	0.00	0.05	0.04	0.01	0.07	0.00	0.03	0.00	0.00	0.05	0.03	0.15	0.21	0.04	0.00	0.11	0.00	0.02
GSM972189	0.02	0.00	0.01	0.30	0.00	0.00	0.12	0.04	0.05	0.06	0.02	0.00	0.00	0.07	0.14	0.10	0.06	0.00	0.01	0.00	0.00	0.00
GSM972190	0.09	0.00	0.07	0.08	0.00	0.24	0.04	0.02	0.07	0.00	0.04	0.01	0.00	0.16	0.04	0.04	0.02	0.03	0.00	0.04	0.00	0.00
GSM972191	0.06	0.00	0.06	0.08	0.00	0.27	0.05	0.06	0.00	0.00	0.03	0.00	0.00	0.13	0.08	0.08	0.04	0.00	0.00	0.04	0.00	0.00
GSM972192	0.02	0.00	0.12	0.12	0.00	0.02	0.00	0.09	0.00	0.00	0.03	0.00	0.00	0.13	0.09	0.13	0.01	0.07	0.00	0.15	0.00	0.02
GSM972193	0.17	0.00	0.08	0.25	0.00	0.07	0.04	0.06	0.06	0.00	0.00	0.00	0.00	0.10	0.03	0.02	0.03	0.01	0.00	0.08	0.00	0.00
GSM972194	0.05	0.00	0.07	0.17	0.00	0.03	0.14	0.08	0.04	0.00	0.04	0.00	0.03	0.12	0.03	0.05	0.08	0.04	0.00	0.04	0.00	0.00
GSM972195	0.03	0.00	0.05	0.21	0.00	0.01	0.02	0.09	0.01	0.00	0.02	0.02	0.00	0.08	0.07	0.18	0.03	0.00	0.00	0.17	0.01	0.00
GSM972196	0.00	0.00	0.03	0.25	0.00	0.00	0.14	0.06	0.00	0.00	0.08	0.00	0.00	0.10	0.12	0.05	0.02	0.00	0.00	0.06	0.00	0.08
GSM972197	0.00	0.07	0.12	0.25	0.00	0.02	0.02	0.06	0.05	0.00	0.01	0.00	0.00	0.16	0.03	0.03	0.04	0.03	0.00	0.11	0.00	0.00
GSM972198	0.00	0.00	0.04	0.17	0.00	0.17	0.08	0.00	0.02	0.00	0.06	0.01	0.02	0.12	0.03	0.09	0.11	0.03	0.04	0.00	0.01	0.00
GSM972199	0.00	0.03	0.05	0.22	0.00	0.10	0.00	0.04	0.05	0.00	0.05	0.00	0.05	0.10	0.06	0.06	0.10	0.00	0.00	0.09	0.01	0.00
GSM972200	0.03	0.00	0.10	0.25	0.00	0.01	0.07	0.04	0.02	0.00	0.00	0.01	0.02	0.09	0.07	0.13	0.06	0.00	0.00	0.11	0.00	0.00
GSM972201	0.01	0.00	0.15	0.12	0.00	0.00	0.02	0.08	0.04	0.02	0.00	0.02	0.00	0.14	0.13	0.13	0.07	0.00	0.00	0.07	0.01	0.00
GSM972202	0.02	0.00	0.07	0.14	0.00	0.07	0.08	0.03	0.01	0.00	0.06	0.00	0.00	0.10	0.06	0.11	0.09	0.02	0.00	0.11	0.01	0.03
GSM972203	0.02	0.01	0.04	0.22	0.00	0.00	0.13	0.05	0.02	0.02	0.01	0.00	0.00	0.00	0.11	0.11	0.23	0.00	0.02	0.01	0.00	0.00
GSM972204	0.02	0.00	0.13	0.02	0.00	0.31	0.07	0.04	0.02	0.00	0.06	0.00	0.00	0.06	0.04	0.12	0.04	0.00	0.00	0.06	0.00	0.01
GSM972205	0.02	0.00	0.04	0.27	0.00	0.00	0.18	0.09	0.00	0.00	0.02	0.02	0.00	0.03	0.12	0.09	0.10	0.00	0.00	0.03	0.00	0.00
GSM972206	0.01	0.01	0.05	0.13	0.00	0.14	0.06	0.01	0.04	0.00	0.06	0.00	0.00	0.07	0.05	0.12	0.15	0.00	0.06	0.00	0.05	0.00
GSM972207	0.03	0.00	0.14	0.10	0.00	0.11	0.00	0.08	0.05	0.00	0.02	0.00	0.00	0.09	0.15	0.08	0.03	0.00	0.00	0.08	0.04	0.00
GSM972208	0.01	0.00	0.10	0.09	0.00	0.07	0.03	0.06	0.02	0.00	0.00	0.00	0.00	0.17	0.21	0.17	0.01	0.00	0.00	0.02	0.00	0.04
GSM972209	0.12	0.00	0.00	0.06	0.00	0.12	0.11	0.05	0.00	0.10	0.00	0.00	0.00	0.13	0.14	0.11	0.01	0.00	0.02	0.00	0.00	0.02
GSM972210	0.00	0.01	0.01	0.12	0.00	0.05	0.11	0.03	0.01	0.00	0.07	0.00	0.00	0.20	0.08	0.14	0.05	0.00	0.00	0.07	0.04	0.00
GSM972211	0.00	0.02	0.03	0.08	0.00	0.14	0.00	0.06	0.06	0.00	0.06	0.00	0.00	0.27	0.07	0.10	0.05	0.01	0.00	0.05	0.00	0.02
GSM972212	0.02	0.00	0.03	0.08	0.00	0.19	0.11	0.02	0.00	0.00	0.07	0.00	0.00	0.11	0.07	0.08	0.12	0.02	0.00	0.06	0.00	0.01
GSM972213	0.01	0.00	0.02	0.15	0.00	0.02	0.03	0.06	0.04	0.00	0.00	0.05	0.00	0.41	0.02	0.19	0.00	0.01	0.00	0.00	0.00	0.00
GSM972214	0.02	0.01	0.03	0.09	0.00	0.22	0.00	0.01	0.00	0.00	0.04	0.00	0.00	0.12	0.01	0.15	0.03	0.06	0.00	0.01	0.15	0.03
GSM972215	0.02	0.02	0.01	0.00	0.00	0.15	0.11	0.04	0.00	0.00	0.06	0.00	0.00	0.14	0.11	0.09	0.04	0.04	0.00	0.07	0.08	0.02

GSM972216	0.03	0.00	0.05	0.13	0.00	0.11	0.17	0.04	0.00	0.00	0.05	0.00	0.00	0.15	0.04	0.09	0.07	0.00	0.00	0.04	0.00	0.01
GSM972217	0.00	0.03	0.02	0.04	0.00	0.13	0.04	0.06	0.00	0.00	0.08	0.00	0.02	0.19	0.07	0.15	0.10	0.01	0.00	0.05	0.00	0.00
GSM972218	0.02	0.02	0.03	0.08	0.00	0.29	0.04	0.01	0.00	0.00	0.08	0.00	0.01	0.04	0.04	0.09	0.11	0.02	0.00	0.08	0.02	0.00
GSM972219	0.02	0.00	0.07	0.05	0.00	0.19	0.06	0.00	0.03	0.00	0.06	0.00	0.00	0.21	0.05	0.09	0.00	0.01	0.00	0.15	0.00	0.00
GSM972220	0.06	0.01	0.02	0.07	0.00	0.01	0.07	0.10	0.03	0.09	0.05	0.00	0.00	0.10	0.16	0.08	0.01	0.00	0.00	0.09	0.01	0.04
GSM972221	0.00	0.00	0.07	0.11	0.00	0.20	0.11	0.01	0.00	0.03	0.04	0.00	0.00	0.14	0.10	0.09	0.03	0.01	0.00	0.05	0.00	0.01
GSM972222	0.02	0.00	0.02	0.12	0.00	0.20	0.00	0.06	0.00	0.00	0.00	0.03	0.00	0.06	0.08	0.16	0.09	0.02	0.00	0.06	0.06	0.00
GSM972223	0.03	0.00	0.04	0.02	0.00	0.23	0.02	0.00	0.00	0.01	0.04	0.00	0.00	0.15	0.10	0.10	0.04	0.00	0.00	0.07	0.00	0.14
GSM972224	0.00	0.00	0.02	0.17	0.00	0.09	0.00	0.08	0.02	0.00	0.01	0.02	0.00	0.15	0.12	0.12	0.04	0.00	0.00	0.08	0.01	0.09
GSM972225	0.03	0.00	0.03	0.16	0.00	0.14	0.09	0.14	0.00	0.00	0.00	0.00	0.03	0.05	0.08	0.05	0.02	0.03	0.00	0.13	0.01	0.00
GSM972226	0.00	0.03	0.05	0.07	0.00	0.26	0.00	0.02	0.00	0.00	0.08	0.00	0.00	0.11	0.05	0.11	0.01	0.01	0.00	0.14	0.02	0.06
GSM972227	0.00	0.07	0.06	0.14	0.00	0.24	0.00	0.00	0.07	0.00	0.07	0.00	0.02	0.00	0.04	0.11	0.05	0.01	0.00	0.10	0.00	0.01
GSM972228	0.00	0.01	0.04	0.23	0.00	0.14	0.03	0.06	0.08	0.00	0.00	0.00	0.00	0.03	0.06	0.11	0.12	0.00	0.00	0.07	0.00	0.01
GSM972229	0.00	0.00	0.00	0.04	0.00	0.08	0.03	0.03	0.00	0.04	0.00	0.00	0.00	0.22	0.05	0.05	0.01	0.00	0.00	0.32	0.03	0.08
GSM972230	0.00	0.07	0.02	0.16	0.00	0.13	0.00	0.09	0.04	0.00	0.01	0.01	0.00	0.26	0.09	0.08	0.00	0.02	0.00	0.03	0.00	0.00
GSM972231	0.02	0.00	0.10	0.00	0.01	0.11	0.06	0.02	0.00	0.03	0.03	0.00	0.00	0.18	0.03	0.09	0.02	0.04	0.01	0.10	0.00	0.15
GSM972232	0.00	0.04	0.01	0.19	0.00	0.06	0.00	0.06	0.04	0.00	0.05	0.00	0.00	0.26	0.07	0.16	0.01	0.00	0.00	0.03	0.01	0.02
GSM972233	0.00	0.01	0.01	0.08	0.00	0.10	0.03	0.02	0.00	0.00	0.04	0.00	0.00	0.37	0.04	0.15	0.03	0.00	0.00	0.10	0.00	0.02
GSM972234	0.00	0.02	0.03	0.05	0.00	0.19	0.01	0.02	0.02	0.00	0.03	0.00	0.00	0.20	0.05	0.20	0.04	0.00	0.00	0.07	0.03	0.02
GSM972235	0.02	0.02	0.02	0.06	0.00	0.17	0.09	0.04	0.00	0.00	0.06	0.00	0.00	0.15	0.09	0.17	0.04	0.00	0.00	0.06	0.00	0.00
GSM972236	0.00	0.08	0.02	0.20	0.00	0.04	0.11	0.00	0.00	0.00	0.03	0.00	0.00	0.18	0.06	0.12	0.05	0.00	0.00	0.10	0.00	0.00
GSM972237	0.02	0.00	0.02	0.07	0.00	0.30	0.11	0.01	0.00	0.00	0.04	0.00	0.02	0.12	0.03	0.05	0.05	0.06	0.08	0.02	0.00	0.00
GSM972238	0.00	0.05	0.01	0.06	0.00	0.09	0.07	0.06	0.04	0.00	0.02	0.00	0.00	0.19	0.08	0.10	0.12	0.03	0.01	0.02	0.00	0.03
GSM972239	0.02	0.00	0.13	0.13	0.00	0.05	0.09	0.07	0.02	0.00	0.00	0.02	0.01	0.10	0.10	0.11	0.09	0.00	0.05	0.00	0.01	0.00
GSM972240	0.01	0.00	0.00	0.19	0.00	0.00	0.06	0.05	0.00	0.00	0.00	0.04	0.00	0.18	0.17	0.13	0.10	0.00	0.05	0.00	0.00	0.02
GSM972241	0.03	0.00	0.02	0.00	0.00	0.21	0.01	0.04	0.04	0.10	0.03	0.08	0.01	0.17	0.17	0.04	0.05	0.00	0.03	0.00	0.00	0.00
GSM972242	0.01	0.00	0.01	0.19	0.00	0.12	0.04	0.05	0.04	0.00	0.00	0.00	0.00	0.07	0.12	0.17	0.07	0.00	0.00	0.07	0.00	0.03
GSM972243	0.01	0.00	0.11	0.19	0.00	0.16	0.03	0.01	0.00	0.05	0.00	0.04	0.00	0.04	0.07	0.10	0.14	0.00	0.00	0.03	0.01	0.02
GSM972244	0.15	0.00	0.11	0.16	0.00	0.05	0.09	0.04	0.04	0.00	0.03	0.00	0.00	0.02	0.07	0.07	0.02	0.00	0.00	0.11	0.00	0.05
GSM972245	0.06	0.04	0.11	0.15	0.00	0.00	0.00	0.05	0.10	0.00	0.03	0.00	0.04	0.00	0.08	0.13	0.04	0.01	0.00	0.16	0.00	0.00
GSM972246	0.02	0.02	0.02	0.04	0.00	0.28	0.09	0.00	0.04	0.00	0.00	0.00	0.00	0.15	0.11	0.08	0.05	0.00	0.00	0.08	0.00	0.02
GSM972247	0.03	0.00	0.02	0.13	0.00	0.14	0.00	0.06	0.02	0.00	0.04	0.00	0.00	0.22	0.07	0.11	0.03	0.00	0.00	0.14	0.00	0.00
GSM972248	0.01	0.07	0.08	0.16	0.00	0.09	0.00	0.02	0.10	0.00	0.00	0.00	0.05	0.05	0.08	0.13	0.03	0.00	0.00	0.12	0.00	0.00
GSM972249	0.04	0.00	0.07	0.18	0.00	0.16	0.05	0.05	0.00	0.00	0.02	0.01	0.02	0.10	0.11	0.10	0.07	0.00	0.02	0.00	0.00	0.00
GSM972250	0.03	0.00	0.06	0.12	0.00	0.13	0.10	0.04	0.00	0.00	0.04	0.00	0.01	0.15	0.04	0.18	0.07	0.01	0.01	0.01	0.00	0.01
GSM972251	0.06	0.00	0.01	0.14	0.00	0.00	0.19	0.05	0.00	0.00	0.02	0.00	0.00	0.12	0.13	0.09	0.05	0.00	0.11	0.00	0.03	0.00
GSM972252	0.03	0.00	0.03	0.15	0.00	0.12	0.07	0.06	0.04	0.00	0.03	0.00	0.01	0.04	0.12	0.17	0.08	0.00	0.00	0.04	0.00	0.00
GSM972253	0.03	0.03	0.11	0.01	0.00	0.24	0.11	0.00	0.01	0.00	0.03	0.00	0.00	0.16	0.02	0.06	0.10	0.00	0.00	0.08	0.00	0.01

GSM972254	0.02	0.00	0.01	0.07	0.00	0.00	0.00	0.07	0.04	0.00	0.08	0.00	0.00	0.43	0.05	0.07	0.04	0.01	0.00	0.09	0.00	0.01
GSM972255	0.01	0.02	0.01	0.12	0.00	0.15	0.00	0.07	0.00	0.00	0.03	0.00	0.03	0.22	0.06	0.12	0.02	0.00	0.00	0.09	0.01	0.02
GSM972256	0.03	0.03	0.05	0.20	0.00	0.07	0.05	0.08	0.00	0.00	0.07	0.00	0.00	0.14	0.04	0.10	0.01	0.01	0.00	0.11	0.00	0.01
GSM972257	0.00	0.07	0.04	0.17	0.00	0.03	0.08	0.05	0.02	0.00	0.00	0.06	0.00	0.15	0.10	0.16	0.04	0.00	0.00	0.03	0.00	0.00
GSM972258	0.11	0.00	0.02	0.15	0.00	0.00	0.19	0.02	0.00	0.00	0.09	0.00	0.00	0.05	0.02	0.19	0.05	0.02	0.00	0.09	0.00	0.01
GSM972259	0.06	0.00	0.15	0.15	0.00	0.04	0.00	0.09	0.09	0.00	0.03	0.00	0.00	0.01	0.09	0.11	0.05	0.00	0.00	0.11	0.00	0.01
GSM972260	0.06	0.00	0.01	0.16	0.00	0.16	0.05	0.03	0.02	0.00	0.09	0.00	0.03	0.10	0.02	0.05	0.03	0.05	0.00	0.14	0.00	0.00
GSM972261	0.07	0.00	0.12	0.00	0.00	0.21	0.00	0.06	0.00	0.04	0.00	0.04	0.00	0.08	0.08	0.04	0.04	0.01	0.00	0.13	0.00	0.06
GSM972262	0.01	0.00	0.00	0.17	0.00	0.00	0.02	0.06	0.01	0.06	0.00	0.00	0.00	0.03	0.16	0.23	0.16	0.00	0.04	0.00	0.00	0.05
GSM972263	0.07	0.00	0.11	0.00	0.00	0.12	0.08	0.09	0.02	0.01	0.03	0.00	0.00	0.11	0.08	0.02	0.02	0.10	0.00	0.07	0.00	0.06
GSM972264	0.00	0.01	0.01	0.06	0.00	0.10	0.12	0.03	0.00	0.00	0.05	0.00	0.00	0.17	0.19	0.12	0.03	0.00	0.00	0.09	0.00	0.02
GSM972265	0.09	0.00	0.01	0.16	0.00	0.17	0.00	0.07	0.00	0.00	0.00	0.04	0.01	0.09	0.06	0.16	0.05	0.03	0.00	0.04	0.01	0.01
GSM972266	0.00	0.05	0.08	0.12	0.00	0.17	0.09	0.03	0.00	0.00	0.07	0.00	0.00	0.04	0.04	0.11	0.06	0.08	0.05	0.00	0.00	0.01
GSM972267	0.00	0.03	0.12	0.26	0.00	0.03	0.13	0.03	0.04	0.00	0.00	0.01	0.00	0.02	0.05	0.12	0.10	0.01	0.00	0.03	0.00	0.02
GSM972268	0.06	0.00	0.05	0.24	0.00	0.21	0.00	0.01	0.04	0.00	0.01	0.03	0.04	0.04	0.04	0.12	0.05	0.00	0.00	0.05	0.01	0.00
GSM972269	0.08	0.00	0.17	0.11	0.00	0.18	0.00	0.00	0.05	0.00	0.02	0.00	0.00	0.18	0.02	0.05	0.03	0.02	0.00	0.09	0.00	0.00
GSM972270	0.03	0.00	0.16	0.08	0.00	0.14	0.11	0.03	0.01	0.07	0.02	0.00	0.00	0.11	0.07	0.09	0.02	0.01	0.00	0.04	0.00	0.01
GSM972271	0.07	0.00	0.01	0.26	0.00	0.09	0.00	0.00	0.00	0.00	0.03	0.01	0.00	0.06	0.11	0.18	0.04	0.01	0.00	0.09	0.00	0.04
GSM972272	0.13	0.00	0.02	0.24	0.00	0.09	0.00	0.02	0.11	0.00	0.02	0.00	0.02	0.04	0.05	0.10	0.07	0.02	0.00	0.07	0.00	0.01
GSM972273	0.03	0.00	0.18	0.11	0.00	0.29	0.01	0.05	0.00	0.00	0.00	0.03	0.00	0.01	0.04	0.17	0.05	0.00	0.00	0.02	0.00	0.01
GSM972274	0.01	0.00	0.09	0.16	0.00	0.00	0.04	0.03	0.00	0.08	0.06	0.00	0.00	0.14	0.21	0.09	0.02	0.01	0.00	0.05	0.00	0.01
GSM972275	0.03	0.00	0.02	0.27	0.00	0.00	0.18	0.01	0.00	0.00	0.07	0.00	0.00	0.01	0.08	0.17	0.05	0.01	0.00	0.00	0.08	0.03
GSM972276	0.00	0.06	0.04	0.19	0.00	0.03	0.15	0.04	0.02	0.00	0.03	0.00	0.00	0.08	0.16	0.07	0.01	0.06	0.02	0.00	0.01	0.02
GSM972277	0.01	0.00	0.02	0.05	0.00	0.28	0.02	0.02	0.02	0.00	0.05	0.00	0.00	0.16	0.05	0.08	0.04	0.08	0.00	0.10	0.00	0.02
GSM972278	0.00	0.04	0.02	0.00	0.00	0.15	0.11	0.06	0.01	0.05	0.11	0.02	0.00	0.13	0.14	0.06	0.03	0.02	0.01	0.00	0.00	0.03
GSM972279	0.03	0.00	0.16	0.05	0.00	0.06	0.10	0.04	0.01	0.01	0.03	0.00	0.00	0.11	0.06	0.17	0.00	0.04	0.00	0.09	0.01	0.05
GSM972280	0.03	0.00	0.05	0.07	0.00	0.04	0.02	0.07	0.03	0.00	0.01	0.00	0.00	0.33	0.05	0.07	0.00	0.00	0.00	0.17	0.00	0.08
GSM972281	0.02	0.00	0.10	0.09	0.00	0.21	0.18	0.00	0.00	0.07	0.00	0.00	0.00	0.15	0.08	0.06	0.00	0.00	0.00	0.03	0.00	0.00
GSM972282	0.04	0.00	0.02	0.04	0.00	0.08	0.05	0.05	0.00	0.00	0.09	0.00	0.00	0.24	0.07	0.16	0.01	0.04	0.00	0.10	0.00	0.00
GSM972283	0.00	0.00	0.06	0.28	0.00	0.00	0.10	0.04	0.03	0.00	0.07	0.00	0.00	0.12	0.12	0.07	0.00	0.03	0.00	0.09	0.00	0.00
GSM972284	0.00	0.11	0.00	0.16	0.00	0.10	0.12	0.05	0.02	0.00	0.00	0.05	0.02	0.08	0.03	0.16	0.00	0.07	0.02	0.00	0.00	0.00
GSM972285	0.01	0.01	0.03	0.06	0.00	0.23	0.00	0.00	0.00	0.00	0.04	0.00	0.00	0.17	0.03	0.06	0.02	0.02	0.00	0.23	0.06	0.04
GSM972286	0.01	0.00	0.01	0.15	0.00	0.17	0.07	0.04	0.01	0.00	0.10	0.00	0.00	0.17	0.10	0.08	0.03	0.01	0.00	0.06	0.00	0.01
GSM972287	0.02	0.00	0.01	0.04	0.00	0.04	0.00	0.03	0.00	0.00	0.00	0.04	0.04	0.09	0.04	0.04	0.00	0.09	0.00	0.37	0.02	0.12
GSM972288	0.12	0.00	0.01	0.29	0.00	0.06	0.00	0.04	0.02	0.00	0.00	0.04	0.00	0.14	0.06	0.12	0.00	0.00	0.00	0.07	0.00	0.03
GSM972289	0.01	0.02	0.10	0.02	0.00	0.41	0.00	0.00	0.05	0.00	0.03	0.00	0.05	0.08	0.04	0.06	0.04	0.00	0.00	0.07	0.00	0.01
GSM972290	0.04	0.00	0.10	0.05	0.00	0.06	0.05	0.10	0.00	0.02	0.02	0.00	0.00	0.17	0.10	0.05	0.02	0.03	0.00	0.11	0.05	0.04
GSM972291	0.00	0.01	0.03	0.17	0.00	0.20	0.01	0.01	0.00	0.00	0.00	0.00	0.03	0.08	0.04	0.14	0.15	0.00	0.02	0.00	0.09	0.00

GSM972292	0.04	0.00	0.00	0.05	0.00	0.19	0.04	0.01	0.00	0.00	0.07	0.02	0.00	0.18	0.02	0.06	0.00	0.08	0.00	0.19	0.00	0.04
GSM972293	0.07	0.04	0.01	0.13	0.00	0.12	0.00	0.14	0.02	0.00	0.01	0.00	0.00	0.07	0.13	0.06	0.03	0.00	0.00	0.14	0.02	0.00
GSM972294	0.00	0.00	0.00	0.40	0.00	0.00	0.17	0.05	0.00	0.11	0.00	0.02	0.00	0.04	0.09	0.06	0.01	0.00	0.00	0.01	0.02	0.01
GSM972295	0.10	0.00	0.02	0.20	0.00	0.12	0.00	0.00	0.12	0.00	0.00	0.09	0.00	0.13	0.02	0.13	0.00	0.01	0.00	0.04	0.00	0.01
GSM972296	0.06	0.00	0.17	0.16	0.00	0.18	0.02	0.05	0.05	0.00	0.02	0.00	0.01	0.10	0.05	0.00	0.05	0.02	0.00	0.06	0.01	0.00
GSM972297	0.01	0.00	0.07	0.06	0.00	0.12	0.04	0.03	0.00	0.00	0.03	0.00	0.03	0.11	0.08	0.13	0.00	0.00	0.00	0.11	0.00	0.17
GSM972298	0.02	0.01	0.08	0.06	0.00	0.16	0.05	0.11	0.00	0.00	0.00	0.00	0.00	0.16	0.11	0.14	0.00	0.03	0.00	0.06	0.00	0.02
GSM972299	0.01	0.00	0.01	0.18	0.00	0.16	0.06	0.02	0.02	0.00	0.05	0.02	0.00	0.12	0.18	0.08	0.06	0.00	0.03	0.00	0.00	0.00
GSM972300	0.03	0.00	0.04	0.11	0.00	0.37	0.00	0.00	0.00	0.00	0.02	0.00	0.02	0.03	0.05	0.12	0.15	0.00	0.00	0.04	0.02	0.00
GSM972301	0.05	0.00	0.10	0.08	0.00	0.09	0.02	0.03	0.02	0.04	0.01	0.00	0.00	0.06	0.06	0.12	0.16	0.02	0.03	0.01	0.07	0.03
GSM972302	0.02	0.02	0.05	0.15	0.00	0.19	0.00	0.01	0.06	0.00	0.04	0.00	0.01	0.07	0.03	0.13	0.12	0.00	0.01	0.09	0.00	0.00
GSM972303	0.01	0.00	0.01	0.08	0.00	0.26	0.14	0.00	0.04	0.00	0.08	0.00	0.01	0.11	0.01	0.02	0.00	0.08	0.00	0.13	0.00	0.01
GSM972304	0.04	0.01	0.09	0.11	0.00	0.18	0.12	0.03	0.00	0.01	0.00	0.00	0.00	0.18	0.06	0.04	0.03	0.04	0.00	0.07	0.00	0.00
GSM972305	0.13	0.00	0.05	0.11	0.00	0.06	0.03	0.07	0.00	0.01	0.04	0.00	0.00	0.13	0.01	0.09	0.02	0.09	0.00	0.14	0.01	0.01
GSM972306	0.02	0.01	0.04	0.06	0.00	0.21	0.03	0.07	0.01	0.00	0.04	0.01	0.00	0.18	0.07	0.07	0.09	0.02	0.00	0.09	0.00	0.00
GSM972307	0.00	0.09	0.01	0.16	0.00	0.17	0.08	0.07	0.01	0.00	0.05	0.00	0.00	0.02	0.10	0.07	0.07	0.03	0.00	0.06	0.00	0.01
GSM972308	0.04	0.00	0.03	0.21	0.00	0.00	0.01	0.14	0.00	0.04	0.00	0.08	0.00	0.04	0.15	0.14	0.08	0.00	0.00	0.04	0.00	0.00
GSM972309	0.05	0.00	0.01	0.21	0.00	0.03	0.18	0.07	0.00	0.00	0.12	0.00	0.07	0.09	0.04	0.01	0.01	0.06	0.00	0.03	0.04	0.00
GSM972310	0.01	0.00	0.03	0.06	0.00	0.28	0.09	0.02	0.04	0.00	0.08	0.00	0.01	0.15	0.08	0.06	0.03	0.00	0.00	0.07	0.00	0.00
GSM972311	0.05	0.00	0.00	0.03	0.00	0.31	0.00	0.02	0.03	0.00	0.01	0.07	0.03	0.12	0.07	0.08	0.04	0.00	0.14	0.00	0.00	0.00
GSM972312	0.03	0.03	0.00	0.24	0.00	0.03	0.16	0.00	0.02	0.00	0.06	0.00	0.00	0.17	0.03	0.06	0.03	0.00	0.00	0.10	0.02	0.02
GSM972313	0.04	0.00	0.00	0.00	0.00	0.29	0.15	0.01	0.05	0.00	0.03	0.07	0.02	0.11	0.06	0.05	0.05	0.03	0.05	0.00	0.00	0.00
GSM972314	0.00	0.01	0.01	0.28	0.00	0.00	0.07	0.07	0.05	0.00	0.00	0.07	0.00	0.10	0.15	0.12	0.06	0.00	0.01	0.00	0.00	0.02
GSM972315	0.01	0.00	0.08	0.17	0.00	0.18	0.06	0.00	0.03	0.00	0.00	0.03	0.00	0.04	0.07	0.12	0.09	0.03	0.08	0.00	0.00	0.01
GSM972316	0.03	0.00	0.07	0.12	0.00	0.16	0.00	0.05	0.00	0.00	0.00	0.02	0.00	0.12	0.05	0.12	0.05	0.02	0.00	0.12	0.01	0.06
GSM972317	0.03	0.00	0.00	0.15	0.00	0.04	0.00	0.04	0.03	0.00	0.01	0.03	0.03	0.12	0.24	0.09	0.03	0.00	0.00	0.12	0.03	0.00
GSM972318	0.00	0.04	0.13	0.10	0.00	0.12	0.10	0.02	0.00	0.00	0.06	0.00	0.03	0.05	0.04	0.09	0.00	0.01	0.00	0.14	0.00	0.05
GSM972319	0.00	0.09	0.01	0.19	0.00	0.08	0.11	0.04	0.00	0.00	0.04	0.00	0.00	0.04	0.15	0.11	0.04	0.02	0.06	0.00	0.00	0.01
GSM972320	0.00	0.05	0.04	0.19	0.00	0.16	0.06	0.00	0.00	0.00	0.01	0.01	0.00	0.05	0.05	0.19	0.10	0.02	0.02	0.04	0.00	0.00
GSM972321	0.07	0.02	0.15	0.07	0.00	0.14	0.02	0.06	0.02	0.00	0.08	0.00	0.04	0.07	0.10	0.08	0.03	0.00	0.03	0.03	0.00	0.01
GSM972322	0.15	0.00	0.11	0.06	0.00	0.21	0.08	0.07	0.01	0.00	0.06	0.00	0.00	0.03	0.09	0.02	0.07	0.00	0.01	0.03	0.00	0.02
GSM972323	0.00	0.01	0.17	0.08	0.00	0.14	0.03	0.01	0.03	0.00	0.04	0.00	0.00	0.13	0.04	0.14	0.09	0.00	0.05	0.02	0.03	0.00
GSM972324	0.04	0.00	0.00	0.10	0.00	0.16	0.07	0.00	0.04	0.00	0.03	0.09	0.03	0.16	0.05	0.10	0.05	0.00	0.06	0.00	0.00	0.00
GSM972325	0.00	0.05	0.04	0.09	0.00	0.14	0.04	0.01	0.03	0.00	0.04	0.00	0.00	0.16	0.03	0.05	0.04	0.03	0.00	0.19	0.00	0.06
GSM972326	0.02	0.00	0.04	0.04	0.00	0.39	0.00	0.00	0.07	0.00	0.03	0.05	0.02	0.08	0.04	0.13	0.03	0.00	0.06	0.00	0.00	0.00
GSM972327	0.02	0.00	0.07	0.21	0.00	0.11	0.01	0.02	0.00	0.00	0.00	0.03	0.02	0.08	0.11	0.18	0.05	0.01	0.00	0.02	0.04	0.01
GSM972328	0.04	0.00	0.00	0.11	0.00	0.08	0.07	0.00	0.04	0.00	0.11	0.00	0.00	0.17	0.05	0.07	0.06	0.03	0.00	0.17	0.01	0.00
GSM972329	0.02	0.03	0.06	0.09	0.00	0.06	0.04	0.06	0.02	0.03	0.02	0.00	0.00	0.08	0.16	0.12	0.07	0.00	0.00	0.08	0.04	0.01

GSM972330	0.02	0.00	0.09	0.07	0.00	0.16	0.04	0.03	0.00	0.00	0.04	0.00	0.00	0.18	0.15	0.04	0.01	0.00	0.00	0.14	0.00	0.04
GSM972331	0.01	0.00	0.07	0.02	0.00	0.16	0.07	0.03	0.03	0.00	0.06	0.00	0.00	0.22	0.03	0.08	0.01	0.04	0.00	0.13	0.02	0.00
GSM972332	0.03	0.02	0.01	0.19	0.00	0.05	0.00	0.04	0.06	0.00	0.00	0.00	0.00	0.12	0.02	0.16	0.01	0.03	0.00	0.20	0.01	0.04
GSM972333	0.02	0.00	0.08	0.12	0.00	0.18	0.04	0.00	0.03	0.00	0.05	0.00	0.02	0.13	0.04	0.13	0.06	0.03	0.00	0.04	0.02	0.00
GSM972334	0.00	0.00	0.03	0.17	0.00	0.05	0.14	0.05	0.02	0.00	0.05	0.00	0.00	0.09	0.19	0.11	0.02	0.01	0.00	0.04	0.00	0.02
GSM972335	0.02	0.00	0.14	0.11	0.00	0.11	0.10	0.02	0.00	0.00	0.03	0.00	0.00	0.00	0.05	0.14	0.05	0.00	0.00	0.17	0.01	0.04
GSM972336	0.01	0.00	0.10	0.12	0.00	0.09	0.00	0.00	0.04	0.00	0.02	0.00	0.00	0.10	0.05	0.19	0.10	0.00	0.00	0.10	0.04	0.03
GSM972337	0.10	0.00	0.00	0.27	0.00	0.00	0.09	0.07	0.01	0.10	0.00	0.00	0.00	0.06	0.17	0.07	0.02	0.00	0.01	0.01	0.01	0.00
GSM972338	0.05	0.02	0.14	0.19	0.00	0.05	0.10	0.02	0.05	0.00	0.00	0.00	0.04	0.05	0.06	0.10	0.09	0.00	0.00	0.04	0.00	0.00
GSM972339	0.00	0.04	0.10	0.04	0.00	0.28	0.03	0.04	0.01	0.00	0.04	0.00	0.02	0.06	0.09	0.15	0.05	0.02	0.00	0.01	0.01	0.00
GSM972340	0.00	0.02	0.01	0.07	0.00	0.07	0.05	0.01	0.00	0.00	0.04	0.00	0.00	0.28	0.02	0.10	0.00	0.00	0.00	0.22	0.02	0.09
GSM972341	0.03	0.00	0.00	0.24	0.00	0.00	0.00	0.06	0.02	0.00	0.02	0.05	0.00	0.20	0.08	0.16	0.07	0.00	0.00	0.04	0.03	0.00
GSM972342	0.02	0.01	0.09	0.09	0.00	0.13	0.06	0.05	0.00	0.01	0.02	0.00	0.00	0.24	0.09	0.13	0.02	0.00	0.03	0.01	0.00	0.00
GSM972343	0.00	0.02	0.02	0.07	0.00	0.30	0.00	0.01	0.01	0.00	0.00	0.00	0.03	0.05	0.12	0.06	0.21	0.00	0.00	0.09	0.00	0.00
GSM972344	0.00	0.02	0.01	0.09	0.00	0.17	0.13	0.05	0.00	0.00	0.10	0.00	0.03	0.12	0.03	0.10	0.01	0.03	0.00	0.11	0.00	0.00
GSM972345	0.00	0.01	0.01	0.08	0.00	0.08	0.00	0.05	0.07	0.00	0.00	0.03	0.00	0.11	0.16	0.28	0.05	0.00	0.04	0.00	0.02	0.01
GSM972346	0.02	0.04	0.00	0.13	0.00	0.31	0.01	0.01	0.02	0.00	0.09	0.00	0.00	0.06	0.02	0.09	0.00	0.06	0.00	0.06	0.00	0.06
GSM972347	0.02	0.02	0.11	0.19	0.00	0.03	0.12	0.06	0.00	0.00	0.01	0.01	0.00	0.11	0.15	0.12	0.05	0.00	0.00	0.01	0.00	0.00
GSM972348	0.00	0.04	0.09	0.05	0.00	0.24	0.17	0.01	0.00	0.00	0.07	0.00	0.00	0.04	0.00	0.09	0.04	0.11	0.03	0.00	0.02	0.00
GSM972349	0.11	0.00	0.19	0.16	0.00	0.03	0.02	0.02	0.00	0.00	0.00	0.00	0.00	0.20	0.08	0.12	0.00	0.00	0.01	0.03	0.00	0.02
GSM972351	0.03	0.00	0.18	0.08	0.00	0.23	0.00	0.01	0.05	0.00	0.00	0.00	0.00	0.13	0.05	0.09	0.04	0.00	0.00	0.08	0.00	0.02
GSM972352	0.02	0.00	0.09	0.06	0.00	0.18	0.00	0.04	0.01	0.00	0.00	0.02	0.00	0.08	0.09	0.25	0.09	0.00	0.00	0.02	0.02	0.02
GSM972353	0.06	0.03	0.08	0.05	0.00	0.03	0.07	0.11	0.00	0.01	0.00	0.06	0.00	0.10	0.20	0.09	0.05	0.01	0.04	0.00	0.00	0.01
GSM972354	0.02	0.00	0.00	0.20	0.00	0.07	0.06	0.10	0.01	0.00	0.00	0.12	0.03	0.14	0.21	0.00	0.02	0.02	0.00	0.02	0.00	0.00
GSM972355	0.00	0.03	0.03	0.16	0.00	0.11	0.02	0.09	0.02	0.00	0.01	0.00	0.00	0.15	0.12	0.10	0.06	0.00	0.05	0.00	0.01	0.03
GSM972357	0.02	0.00	0.08	0.03	0.00	0.29	0.00	0.10	0.00	0.05	0.00	0.10	0.01	0.05	0.05	0.11	0.05	0.00	0.00	0.03	0.02	0.00
GSM972358	0.01	0.00	0.09	0.08	0.00	0.17	0.01	0.07	0.00	0.00	0.04	0.00	0.01	0.10	0.09	0.10	0.12	0.01	0.00	0.07	0.03	0.00
GSM972359	0.08	0.00	0.05	0.06	0.00	0.25	0.00	0.04	0.00	0.02	0.00	0.00	0.00	0.00	0.06	0.15	0.06	0.00	0.19	0.02	0.01	0.01
GSM972360	0.01	0.00	0.01	0.23	0.00	0.06	0.05	0.07	0.06	0.00	0.05	0.02	0.02	0.04	0.13	0.12	0.06	0.02	0.04	0.00	0.00	0.00
GSM972361	0.00	0.00	0.04	0.33	0.00	0.00	0.21	0.01	0.00	0.09	0.00	0.00	0.02	0.01	0.11	0.15	0.01	0.00	0.01	0.00	0.00	0.00
GSM972362	0.01	0.00	0.16	0.13	0.00	0.20	0.12	0.07	0.00	0.00	0.00	0.00	0.01	0.06	0.04	0.05	0.03	0.02	0.07	0.03	0.00	0.00
GSM972363	0.01	0.00	0.03	0.19	0.00	0.00	0.00	0.02	0.02	0.00	0.05	0.00	0.00	0.31	0.13	0.10	0.02	0.00	0.00	0.06	0.00	0.05
GSM972364	0.01	0.01	0.00	0.15	0.00	0.24	0.00	0.00	0.01	0.00	0.05	0.00	0.00	0.14	0.02	0.17	0.02	0.00	0.00	0.18	0.00	0.00
GSM972365	0.09	0.00	0.02	0.06	0.00	0.13	0.05	0.00	0.00	0.00	0.03	0.00	0.05	0.13	0.05	0.05	0.10	0.04	0.00	0.12	0.06	0.03
GSM972366	0.02	0.03	0.04	0.14	0.00	0.29	0.00	0.03	0.01	0.00	0.03	0.00	0.00	0.02	0.04	0.16	0.02	0.00	0.00	0.08	0.09	0.00
GSM972367	0.01	0.00	0.11	0.14	0.00	0.08	0.00	0.02	0.05	0.00	0.03	0.00	0.00	0.25	0.07	0.08	0.08	0.00	0.00	0.07	0.00	0.01
GSM972368	0.01	0.00	0.07	0.23	0.00	0.01	0.11	0.06	0.03	0.00	0.02	0.03	0.00	0.18	0.02	0.08	0.02	0.00	0.00	0.10	0.00	0.03
GSM972369	0.00	0.00	0.01	0.06	0.00	0.05	0.06	0.01	0.00	0.00	0.02	0.01	0.00	0.24	0.05	0.11	0.02	0.00	0.00	0.18	0.01	0.18

GSM972370	0.05	0.00	0.13	0.17	0.00	0.15	0.06	0.01	0.03	0.00	0.03	0.00	0.00	0.08	0.05	0.09	0.06	0.00	0.00	0.07	0.00	0.01
GSM972371	0.12	0.00	0.00	0.08	0.00	0.12	0.02	0.09	0.00	0.00	0.03	0.00	0.00	0.25	0.02	0.09	0.06	0.01	0.01	0.04	0.06	0.00
GSM972372	0.09	0.00	0.10	0.06	0.00	0.13	0.04	0.03	0.03	0.00	0.02	0.00	0.01	0.13	0.07	0.16	0.00	0.00	0.00	0.07	0.01	0.03
GSM972373	0.00	0.06	0.00	0.14	0.00	0.20	0.14	0.00	0.00	0.00	0.02	0.00	0.01	0.19	0.04	0.13	0.02	0.02	0.00	0.03	0.00	0.00
GSM972374	0.05	0.00	0.06	0.17	0.00	0.05	0.13	0.11	0.01	0.00	0.01	0.01	0.01	0.08	0.13	0.09	0.05	0.00	0.00	0.05	0.00	0.00
GSM972375	0.01	0.00	0.02	0.12	0.00	0.11	0.03	0.04	0.03	0.00	0.01	0.01	0.00	0.04	0.06	0.18	0.17	0.02	0.00	0.05	0.01	0.09
GSM972376	0.00	0.02	0.00	0.12	0.00	0.05	0.03	0.08	0.02	0.00	0.00	0.03	0.00	0.41	0.09	0.06	0.04	0.00	0.00	0.06	0.00	0.00
GSM972377	0.04	0.00	0.04	0.05	0.01	0.05	0.07	0.04	0.02	0.00	0.05	0.00	0.00	0.36	0.07	0.09	0.01	0.02	0.00	0.05	0.00	0.02
GSM972378	0.03	0.00	0.12	0.05	0.00	0.02	0.01	0.00	0.01	0.00	0.05	0.00	0.00	0.34	0.01	0.04	0.00	0.03	0.00	0.23	0.00	0.07
GSM972379	0.00	0.03	0.14	0.15	0.00	0.26	0.00	0.05	0.01	0.00	0.03	0.00	0.00	0.08	0.04	0.10	0.03	0.00	0.00	0.08	0.00	0.00
GSM972380	0.10	0.00	0.10	0.04	0.00	0.17	0.05	0.08	0.00	0.00	0.04	0.00	0.00	0.10	0.13	0.07	0.02	0.00	0.04	0.04	0.00	0.03
GSM972381	0.00	0.05	0.00	0.07	0.00	0.06	0.00	0.00	0.05	0.00	0.00	0.02	0.00	0.49	0.02	0.19	0.00	0.00	0.00	0.05	0.00	0.01
GSM972382	0.01	0.00	0.01	0.15	0.00	0.04	0.12	0.03	0.00	0.00	0.03	0.00	0.00	0.39	0.06	0.05	0.05	0.02	0.00	0.04	0.00	0.00
GSM972383	0.03	0.01	0.17	0.05	0.00	0.22	0.09	0.00	0.02	0.00	0.00	0.02	0.00	0.14	0.03	0.12	0.01	0.00	0.08	0.00	0.00	0.00
GSM972384	0.03	0.00	0.00	0.07	0.00	0.08	0.00	0.03	0.00	0.00	0.00	0.00	0.00	0.04	0.03	0.01	0.05	0.10	0.00	0.31	0.10	0.15
GSM972385	0.07	0.00	0.03	0.12	0.00	0.00	0.14	0.03	0.02	0.00	0.04	0.00	0.00	0.22	0.08	0.13	0.04	0.00	0.00	0.02	0.06	0.00
GSM972386	0.02	0.00	0.10	0.11	0.00	0.21	0.08	0.00	0.00	0.00	0.05	0.00	0.00	0.06	0.02	0.13	0.03	0.01	0.00	0.14	0.04	0.00
GSM972387	0.00	0.01	0.06	0.08	0.00	0.18	0.18	0.00	0.00	0.00	0.04	0.00	0.00	0.16	0.06	0.10	0.00	0.03	0.08	0.00	0.00	0.03
GSM972388	0.01	0.04	0.00	0.12	0.00	0.12	0.04	0.00	0.00	0.00	0.09	0.00	0.06	0.18	0.12	0.06	0.05	0.02	0.00	0.08	0.00	0.00
GSM972389	0.01	0.00	0.02	0.04	0.00	0.37	0.10	0.00	0.00	0.00	0.03	0.00	0.00	0.14	0.14	0.08	0.05	0.00	0.00	0.01	0.00	0.00
GSM972390	0.24	0.00	0.14	0.28	0.00	0.00	0.00	0.04	0.04	0.00	0.00	0.00	0.00	0.06	0.02	0.08	0.03	0.00	0.00	0.06	0.00	0.00
GSM972391	0.01	0.00	0.01	0.10	0.00	0.18	0.00	0.00	0.04	0.00	0.04	0.02	0.00	0.15	0.06	0.08	0.02	0.03	0.00	0.20	0.00	0.05
GSM972392	0.02	0.02	0.03	0.15	0.00	0.09	0.03	0.07	0.03	0.00	0.02	0.00	0.00	0.17	0.07	0.16	0.01	0.02	0.00	0.09	0.00	0.01
GSM972393	0.05	0.00	0.05	0.15	0.00	0.00	0.10	0.07	0.02	0.00	0.05	0.00	0.00	0.15	0.05	0.08	0.01	0.00	0.00	0.21	0.00	0.00
GSM972394	0.01	0.03	0.09	0.13	0.00	0.04	0.00	0.06	0.05	0.00	0.07	0.00	0.00	0.11	0.07	0.15	0.07	0.00	0.00	0.12	0.00	0.00
GSM972395	0.03	0.00	0.02	0.09	0.00	0.12	0.05	0.03	0.00	0.00	0.00	0.00	0.00	0.10	0.15	0.15	0.08	0.00	0.05	0.00	0.11	0.01
GSM972396	0.00	0.06	0.01	0.10	0.00	0.35	0.00	0.00	0.03	0.00	0.05	0.00	0.04	0.06	0.04	0.13	0.02	0.02	0.00	0.09	0.01	0.00
GSM972397	0.00	0.04	0.06	0.26	0.00	0.03	0.20	0.02	0.00	0.00	0.03	0.00	0.01	0.06	0.09	0.06	0.03	0.01	0.00	0.01	0.06	0.00
GSM972398	0.00	0.03	0.06	0.00	0.00	0.28	0.17	0.00	0.00	0.03	0.03	0.00	0.00	0.05	0.07	0.07	0.08	0.03	0.00	0.02	0.00	0.07
GSM972399	0.00	0.03	0.03	0.00	0.00	0.29	0.00	0.00	0.05	0.00	0.11	0.00	0.00	0.20	0.02	0.02	0.09	0.06	0.00	0.10	0.00	0.00
GSM972400	0.00	0.01	0.06	0.10	0.00	0.25	0.00	0.00	0.00	0.00	0.05	0.00	0.00	0.09	0.04	0.15	0.03	0.00	0.00	0.13	0.05	0.03
GSM972401	0.05	0.00	0.07	0.03	0.00	0.05	0.00	0.02	0.01	0.00	0.01	0.00	0.22	0.09	0.03	0.03	0.00	0.01	0.00	0.15	0.05	0.18
GSM972402	0.00	0.02	0.07	0.11	0.00	0.12	0.02	0.01	0.01	0.00	0.03	0.00	0.01	0.23	0.05	0.11	0.02	0.00	0.00	0.15	0.00	0.02
GSM972403	0.00	0.01	0.02	0.07	0.00	0.09	0.00	0.04	0.03	0.00	0.00	0.00	0.00	0.21	0.14	0.09	0.02	0.00	0.00	0.19	0.00	0.09
GSM972404	0.00	0.02	0.06	0.12	0.00	0.05	0.00	0.03	0.03	0.00	0.08	0.00	0.03	0.07	0.09	0.08	0.12	0.00	0.00	0.15	0.07	0.00
GSM972405	0.00	0.03	0.04	0.08	0.00	0.15	0.01	0.01	0.00	0.00	0.05	0.00	0.00	0.19	0.04	0.13	0.00	0.02	0.00	0.14	0.01	0.10
GSM972406	0.01	0.00	0.03	0.13	0.00	0.08	0.04	0.02	0.00	0.00	0.03	0.00	0.00	0.24	0.11	0.15	0.02	0.02	0.00	0.09	0.00	0.03
GSM972407	0.00	0.03	0.03	0.09	0.00	0.02	0.09	0.04	0.04	0.00	0.04	0.00	0.00	0.39	0.02	0.12	0.03	0.02	0.00	0.05	0.00	0.00

GSM972408	0.00	0.02	0.00	0.00	0.00	0.16	0.00	0.01	0.00	0.01	0.01	0.00	0.00	0.14	0.01	0.05	0.03	0.04	0.00	0.25	0.03	0.24
GSM972409	0.01	0.00	0.01	0.17	0.00	0.00	0.13	0.07	0.01	0.04	0.01	0.00	0.00	0.10	0.17	0.17	0.10	0.00	0.02	0.00	0.00	0.00
GSM972410	0.01	0.00	0.01	0.10	0.00	0.06	0.01	0.01	0.02	0.00	0.07	0.00	0.00	0.39	0.04	0.06	0.10	0.00	0.00	0.12	0.00	0.00
GSM972411	0.03	0.00	0.04	0.06	0.00	0.23	0.15	0.00	0.01	0.00	0.10	0.00	0.00	0.12	0.03	0.04	0.02	0.04	0.00	0.10	0.03	0.00
GSM972412	0.08	0.00	0.12	0.14	0.00	0.20	0.00	0.02	0.03	0.00	0.00	0.00	0.00	0.05	0.08	0.12	0.03	0.00	0.05	0.01	0.07	0.00
GSM972413	0.01	0.02	0.01	0.11	0.00	0.08	0.01	0.04	0.01	0.00	0.00	0.00	0.01	0.32	0.05	0.16	0.05	0.00	0.00	0.09	0.01	0.02
GSM972414	0.02	0.00	0.11	0.07	0.00	0.20	0.04	0.09	0.00	0.00	0.00	0.01	0.00	0.08	0.08	0.08	0.05	0.01	0.00	0.10	0.07	0.01
GSM972415	0.00	0.04	0.14	0.09	0.00	0.20	0.08	0.00	0.00	0.00	0.10	0.00	0.02	0.05	0.02	0.05	0.04	0.05	0.00	0.11	0.00	0.00
GSM972416	0.00	0.06	0.03	0.10	0.00	0.02	0.00	0.02	0.03	0.00	0.04	0.00	0.00	0.41	0.05	0.11	0.00	0.00	0.00	0.15	0.00	0.00
GSM972417	0.00	0.03	0.00	0.05	0.00	0.06	0.06	0.02	0.01	0.00	0.01	0.00	0.00	0.59	0.02	0.13	0.01	0.01	0.01	0.00	0.00	0.00
GSM972418	0.00	0.05	0.01	0.13	0.00	0.04	0.08	0.10	0.03	0.00	0.06	0.00	0.00	0.25	0.08	0.12	0.04	0.00	0.00	0.00	0.00	0.00
GSM972419	0.00	0.02	0.01	0.10	0.00	0.17	0.00	0.01	0.04	0.00	0.02	0.00	0.05	0.11	0.03	0.10	0.12	0.03	0.00	0.17	0.00	0.03
GSM972420	0.10	0.00	0.09	0.31	0.00	0.00	0.02	0.08	0.04	0.03	0.00	0.00	0.00	0.01	0.06	0.12	0.02	0.04	0.00	0.07	0.00	0.02
GSM972421	0.00	0.00	0.00	0.07	0.00	0.17	0.03	0.04	0.00	0.00	0.02	0.00	0.02	0.22	0.07	0.20	0.03	0.00	0.00	0.10	0.00	0.03
GSM972422	0.04	0.00	0.03	0.20	0.00	0.00	0.02	0.08	0.01	0.00	0.00	0.03	0.05	0.16	0.11	0.12	0.07	0.00	0.00	0.07	0.00	0.01
GSM972423	0.04	0.00	0.04	0.13	0.00	0.19	0.00	0.04	0.10	0.00	0.08	0.00	0.01	0.13	0.06	0.04	0.04	0.03	0.01	0.04	0.00	0.00
GSM972424	0.00	0.02	0.01	0.23	0.00	0.03	0.11	0.00	0.06	0.00	0.04	0.01	0.00	0.21	0.01	0.09	0.02	0.02	0.00	0.13	0.00	0.00
GSM972425	0.02	0.00	0.02	0.08	0.00	0.10	0.00	0.05	0.00	0.00	0.06	0.00	0.01	0.34	0.03	0.04	0.00	0.01	0.00	0.15	0.00	0.10
GSM972426	0.07	0.00	0.08	0.22	0.00	0.00	0.01	0.05	0.07	0.00	0.03	0.00	0.02	0.08	0.07	0.09	0.06	0.00	0.05	0.08	0.00	0.01
GSM972427	0.01	0.01	0.04	0.16	0.00	0.17	0.06	0.01	0.01	0.00	0.07	0.00	0.02	0.23	0.03	0.07	0.00	0.02	0.00	0.08	0.00	0.00
GSM972428	0.00	0.02	0.02	0.16	0.00	0.06	0.03	0.04	0.00	0.00	0.08	0.00	0.00	0.16	0.02	0.09	0.02	0.02	0.00	0.23	0.01	0.06
GSM972429	0.04	0.00	0.13	0.18	0.00	0.09	0.01	0.07	0.06	0.00	0.04	0.00	0.00	0.00	0.07	0.07	0.10	0.00	0.00	0.12	0.01	0.01
GSM972430	0.12	0.00	0.05	0.07	0.00	0.09	0.06	0.07	0.00	0.00	0.02	0.01	0.00	0.08	0.05	0.06	0.07	0.07	0.01	0.08	0.03	0.08
GSM972431	0.07	0.00	0.01	0.07	0.00	0.24	0.06	0.07	0.00	0.05	0.01	0.00	0.00	0.02	0.03	0.11	0.09	0.03	0.05	0.02	0.07	0.00
GSM972432	0.00	0.02	0.00	0.16	0.00	0.07	0.06	0.05	0.00	0.00	0.04	0.00	0.00	0.06	0.05	0.25	0.12	0.00	0.05	0.05	0.01	0.01
GSM972433	0.00	0.04	0.02	0.00	0.00	0.20	0.00	0.06	0.00	0.02	0.04	0.00	0.03	0.08	0.02	0.16	0.02	0.03	0.00	0.17	0.04	0.04
GSM972434	0.00	0.03	0.19	0.04	0.00	0.18	0.14	0.00	0.00	0.00	0.04	0.00	0.00	0.01	0.05	0.11	0.09	0.00	0.07	0.00	0.03	0.01
GSM972435	0.00	0.05	0.11	0.11	0.00	0.17	0.14	0.00	0.01	0.00	0.00	0.01	0.01	0.07	0.02	0.16	0.04	0.03	0.06	0.00	0.00	0.00
GSM972436	0.00	0.05	0.00	0.08	0.00	0.02	0.02	0.00	0.03	0.00	0.08	0.00	0.02	0.32	0.02	0.17	0.07	0.00	0.00	0.10	0.00	0.03
GSM972437	0.05	0.00	0.10	0.11	0.00	0.13	0.00	0.03	0.00	0.00	0.04	0.00	0.00	0.21	0.06	0.12	0.02	0.00	0.00	0.08	0.01	0.03
GSM972438	0.02	0.00	0.01	0.06	0.00	0.15	0.02	0.02	0.01	0.00	0.02	0.00	0.12	0.14	0.11	0.10	0.00	0.00	0.00	0.05	0.00	0.17
GSM972439	0.02	0.00	0.11	0.12	0.00	0.18	0.13	0.00	0.01	0.00	0.06	0.00	0.03	0.00	0.05	0.16	0.04	0.00	0.05	0.00	0.02	0.00
GSM972440	0.00	0.01	0.07	0.13	0.00	0.06	0.05	0.02	0.01	0.00	0.00	0.02	0.00	0.10	0.13	0.12	0.00	0.06	0.00	0.19	0.00	0.04
GSM972441	0.03	0.00	0.02	0.09	0.00	0.27	0.02	0.00	0.00	0.04	0.00	0.00	0.00	0.06	0.05	0.17	0.10	0.02	0.01	0.05	0.07	0.00
GSM972442	0.20	0.00	0.00	0.13	0.00	0.14	0.05	0.02	0.01	0.08	0.00	0.00	0.00	0.07	0.14	0.07	0.07	0.00	0.02	0.00	0.00	0.00
GSM972443	0.07	0.00	0.00	0.09	0.00	0.11	0.00	0.08	0.04	0.01	0.00	0.03	0.00	0.27	0.04	0.10	0.02	0.02	0.00	0.13	0.00	0.00
GSM972444	0.02	0.00	0.11	0.04	0.00	0.05	0.04	0.01	0.00	0.05	0.08	0.00	0.00	0.20	0.14	0.06	0.00	0.02	0.00	0.12	0.00	0.03
GSM972445	0.03	0.00	0.01	0.35	0.00	0.00	0.15	0.04	0.01	0.00	0.02	0.00	0.00	0.08	0.14	0.05	0.02	0.03	0.00	0.04	0.01	0.01

GSM972447	0.02	0.00	0.06	0.09	0.00	0.17	0.04	0.00	0.01	0.00	0.06	0.00	0.00	0.11	0.05	0.10	0.05	0.02	0.02	0.08	0.11	0.03
GSM972449	0.01	0.04	0.05	0.09	0.00	0.16	0.00	0.03	0.04	0.00	0.04	0.00	0.00	0.12	0.02	0.17	0.07	0.03	0.07	0.06	0.00	0.00
GSM972450	0.01	0.00	0.05	0.05	0.00	0.37	0.03	0.03	0.02	0.00	0.06	0.00	0.00	0.12	0.06	0.11	0.03	0.01	0.00	0.05	0.00	0.01
GSM972451	0.00	0.07	0.01	0.06	0.00	0.17	0.16	0.00	0.00	0.00	0.10	0.00	0.01	0.11	0.05	0.08	0.07	0.00	0.00	0.10	0.01	0.01
GSM972452	0.00	0.00	0.04	0.30	0.00	0.00	0.15	0.03	0.00	0.00	0.07	0.00	0.01	0.13	0.12	0.12	0.01	0.00	0.00	0.01	0.00	0.01
GSM972453	0.00	0.05	0.01	0.05	0.00	0.23	0.00	0.01	0.02	0.00	0.08	0.00	0.00	0.23	0.03	0.08	0.05	0.00	0.00	0.17	0.00	0.00
GSM972454	0.08	0.00	0.16	0.11	0.00	0.10	0.10	0.07	0.00	0.01	0.00	0.01	0.00	0.12	0.09	0.07	0.04	0.00	0.02	0.01	0.01	0.00
GSM972455	0.01	0.00	0.01	0.07	0.00	0.06	0.00	0.02	0.00	0.00	0.02	0.00	0.12	0.15	0.08	0.02	0.00	0.02	0.00	0.21	0.00	0.20
GSM972456	0.01	0.00	0.14	0.09	0.00	0.21	0.03	0.00	0.01	0.00	0.05	0.00	0.00	0.07	0.09	0.20	0.04	0.00	0.00	0.03	0.00	0.03
GSM972457	0.01	0.01	0.11	0.13	0.00	0.30	0.00	0.05	0.02	0.00	0.05	0.00	0.02	0.06	0.04	0.07	0.06	0.02	0.00	0.04	0.01	0.00
GSM972458	0.01	0.00	0.01	0.19	0.00	0.00	0.17	0.11	0.01	0.00	0.12	0.00	0.00	0.12	0.10	0.08	0.02	0.01	0.00	0.03	0.00	0.03
GSM972459	0.11	0.00	0.12	0.10	0.00	0.17	0.11	0.00	0.00	0.00	0.03	0.00	0.00	0.07	0.10	0.09	0.01	0.00	0.05	0.00	0.00	0.04
GSM972460	0.00	0.09	0.03	0.17	0.00	0.11	0.04	0.06	0.01	0.00	0.00	0.02	0.01	0.07	0.09	0.13	0.10	0.05	0.00	0.01	0.00	0.00
GSM972461	0.02	0.00	0.02	0.23	0.00	0.07	0.00	0.02	0.02	0.00	0.00	0.02	0.03	0.14	0.18	0.10	0.05	0.00	0.00	0.07	0.02	0.00
GSM972462	0.02	0.00	0.03	0.25	0.00	0.04	0.13	0.03	0.00	0.00	0.09	0.00	0.00	0.16	0.11	0.04	0.05	0.03	0.00	0.01	0.01	0.00
GSM972464	0.30	0.00	0.00	0.06	0.00	0.05	0.06	0.09	0.01	0.01	0.02	0.00	0.00	0.05	0.03	0.09	0.11	0.01	0.00	0.05	0.07	0.00
GSM972465	0.00	0.06	0.00	0.08	0.00	0.24	0.00	0.00	0.02	0.00	0.04	0.00	0.00	0.28	0.05	0.08	0.00	0.00	0.00	0.13	0.00	0.02
GSM972466	0.00	0.06	0.00	0.22	0.00	0.12	0.04	0.04	0.00	0.00	0.08	0.00	0.00	0.07	0.05	0.05	0.00	0.07	0.00	0.10	0.00	0.08
GSM972467	0.03	0.01	0.10	0.16	0.00	0.00	0.04	0.06	0.01	0.00	0.00	0.00	0.00	0.09	0.14	0.16	0.08	0.00	0.10	0.00	0.00	0.01
GSM972468	0.00	0.04	0.01	0.05	0.00	0.14	0.07	0.07	0.00	0.00	0.04	0.01	0.03	0.22	0.15	0.01	0.04	0.00	0.00	0.05	0.05	0.00
GSM972469	0.00	0.03	0.09	0.08	0.00	0.19	0.02	0.03	0.04	0.00	0.04	0.00	0.00	0.11	0.05	0.10	0.08	0.04	0.00	0.07	0.01	0.02
GSM972470	0.00	0.00	0.05	0.18	0.00	0.00	0.08	0.06	0.00	0.11	0.00	0.00	0.00	0.06	0.12	0.19	0.00	0.00	0.00	0.07	0.00	0.08
GSM972472	0.03	0.00	0.01	0.02	0.00	0.17	0.00	0.00	0.02	0.00	0.06	0.00	0.01	0.16	0.02	0.10	0.00	0.00	0.00	0.28	0.03	0.08
GSM972473	0.01	0.00	0.06	0.08	0.00	0.18	0.09	0.04	0.00	0.00	0.06	0.00	0.02	0.06	0.04	0.07	0.05	0.08	0.00	0.12	0.02	0.03
GSM972474	0.01	0.02	0.09	0.14	0.00	0.11	0.05	0.04	0.03	0.00	0.00	0.02	0.01	0.12	0.11	0.05	0.01	0.06	0.06	0.00	0.04	0.03
GSM972475	0.00	0.00	0.07	0.10	0.00	0.29	0.00	0.00	0.06	0.00	0.00	0.00	0.00	0.21	0.04	0.12	0.03	0.00	0.00	0.07	0.00	0.00
GSM972476	0.01	0.00	0.01	0.09	0.00	0.09	0.00	0.06	0.01	0.00	0.00	0.00	0.00	0.20	0.09	0.15	0.06	0.00	0.00	0.08	0.10	0.05
GSM972477	0.02	0.08	0.04	0.23	0.00	0.07	0.00	0.09	0.05	0.01	0.00	0.00	0.01	0.00	0.10	0.09	0.04	0.03	0.00	0.10	0.02	0.01
GSM972478	0.01	0.01	0.16	0.09	0.00	0.19	0.00	0.07	0.02	0.00	0.00	0.01	0.00	0.08	0.05	0.10	0.04	0.05	0.00	0.09	0.02	0.00
GSM972479	0.00	0.01	0.03	0.03	0.00	0.05	0.00	0.02	0.01	0.00	0.00	0.00	0.00	0.38	0.02	0.22	0.00	0.00	0.00	0.17	0.00	0.07
GSM972480	0.00	0.00	0.08	0.23	0.00	0.17	0.04	0.03	0.03	0.00	0.05	0.00	0.00	0.00	0.07	0.12	0.05	0.03	0.05	0.03	0.01	0.01
GSM972481	0.04	0.00	0.16	0.06	0.00	0.14	0.06	0.03	0.02	0.00	0.04	0.00	0.00	0.20	0.05	0.09	0.07	0.00	0.00	0.02	0.03	0.00
GSM972482	0.00	0.01	0.01	0.07	0.00	0.26	0.00	0.01	0.01	0.00	0.02	0.01	0.02	0.21	0.07	0.17	0.07	0.00	0.00	0.03	0.00	0.03
GSM972483	0.01	0.03	0.01	0.13	0.00	0.20	0.04	0.01	0.00	0.00	0.01	0.00	0.03	0.15	0.09	0.19	0.11	0.00	0.00	0.00	0.00	0.00
GSM972484	0.00	0.00	0.01	0.17	0.00	0.02	0.01	0.11	0.00	0.00	0.00	0.00	0.00	0.31	0.14	0.11	0.04	0.00	0.00	0.06	0.00	0.01
GSM972485	0.05	0.00	0.04	0.12	0.00	0.08	0.14	0.07	0.03	0.00	0.04	0.00	0.00	0.17	0.03	0.08	0.02	0.10	0.03	0.00	0.00	0.00
GSM972486	0.00	0.02	0.02	0.05	0.00	0.11	0.02	0.03	0.03	0.00	0.02	0.00	0.00	0.48	0.06	0.04	0.03	0.00	0.00	0.07	0.00	0.02
GSM972487	0.02	0.02	0.11	0.04	0.00	0.33	0.00	0.00	0.01	0.00	0.01	0.00	0.00	0.13	0.03	0.09	0.08	0.00	0.00	0.12	0.00	0.00

GSM972488	0.05	0.00	0.21	0.07	0.00	0.27	0.00	0.03	0.02	0.00	0.05	0.00	0.01	0.07	0.03	0.07	0.03	0.01	0.00	0.10	0.00	0.00
GSM972489	0.01	0.01	0.10	0.10	0.00	0.18	0.04	0.05	0.01	0.04	0.05	0.00	0.00	0.10	0.05	0.12	0.01	0.02	0.01	0.10	0.00	0.00
GSM972490	0.03	0.00	0.03	0.05	0.00	0.15	0.11	0.06	0.00	0.00	0.07	0.00	0.00	0.24	0.03	0.07	0.00	0.03	0.00	0.12	0.00	0.02
GSM972491	0.03	0.00	0.00	0.17	0.00	0.04	0.00	0.06	0.02	0.00	0.02	0.00	0.00	0.23	0.06	0.10	0.03	0.00	0.00	0.20	0.00	0.03
GSM972492	0.05	0.00	0.01	0.13	0.00	0.05	0.11	0.04	0.01	0.00	0.06	0.07	0.00	0.09	0.08	0.18	0.09	0.00	0.00	0.00	0.00	0.03
GSM972493	0.04	0.00	0.03	0.14	0.00	0.16	0.00	0.07	0.04	0.00	0.05	0.00	0.00	0.13	0.16	0.06	0.06	0.00	0.00	0.04	0.02	0.00
GSM972494	0.02	0.00	0.02	0.22	0.00	0.00	0.03	0.12	0.08	0.06	0.00	0.00	0.00	0.08	0.18	0.02	0.14	0.01	0.00	0.03	0.00	0.00
GSM972495	0.00	0.02	0.02	0.09	0.00	0.15	0.17	0.00	0.00	0.00	0.00	0.04	0.00	0.06	0.09	0.01	0.33	0.00	0.04	0.00	0.00	0.00
GSM972496	0.13	0.00	0.06	0.19	0.00	0.12	0.01	0.11	0.02	0.01	0.06	0.00	0.00	0.03	0.04	0.03	0.01	0.05	0.00	0.10	0.00	0.02
GSM972497	0.01	0.04	0.06	0.07	0.00	0.08	0.08	0.03	0.00	0.00	0.08	0.00	0.00	0.24	0.04	0.05	0.10	0.00	0.00	0.10	0.00	0.00
GSM972498	0.00	0.01	0.00	0.04	0.00	0.31	0.07	0.00	0.02	0.00	0.09	0.00	0.00	0.12	0.05	0.04	0.17	0.04	0.01	0.01	0.00	0.03
GSM972499	0.05	0.00	0.08	0.00	0.00	0.04	0.07	0.09	0.00	0.07	0.00	0.06	0.00	0.03	0.13	0.04	0.00	0.12	0.00	0.10	0.00	0.12
GSM972501	0.02	0.00	0.15	0.14	0.00	0.03	0.01	0.08	0.02	0.02	0.02	0.00	0.00	0.16	0.06	0.09	0.02	0.00	0.00	0.14	0.00	0.04
GSM972502	0.02	0.02	0.06	0.16	0.00	0.19	0.00	0.02	0.08	0.00	0.00	0.02	0.02	0.12	0.04	0.12	0.04	0.02	0.00	0.09	0.00	0.00
GSM972503	0.24	0.00	0.04	0.11	0.00	0.12	0.01	0.06	0.04	0.00	0.05	0.00	0.00	0.04	0.05	0.13	0.03	0.00	0.00	0.09	0.00	0.00
GSM972504	0.00	0.06	0.05	0.17	0.00	0.00	0.06	0.09	0.01	0.00	0.05	0.00	0.00	0.22	0.07	0.14	0.01	0.00	0.00	0.07	0.00	0.00
GSM972505	0.02	0.02	0.08	0.14	0.00	0.00	0.11	0.05	0.04	0.00	0.09	0.00	0.00	0.19	0.02	0.06	0.00	0.07	0.00	0.09	0.00	0.00
GSM972506	0.02	0.02	0.07	0.27	0.00	0.01	0.09	0.00	0.01	0.00	0.00	0.00	0.00	0.08	0.06	0.16	0.06	0.00	0.00	0.13	0.00	0.02
GSM972507	0.07	0.00	0.10	0.13	0.00	0.02	0.03	0.07	0.00	0.05	0.00	0.03	0.00	0.04	0.20	0.17	0.05	0.02	0.03	0.00	0.00	0.00
GSM972508	0.00	0.01	0.07	0.19	0.00	0.11	0.14	0.05	0.01	0.02	0.00	0.00	0.00	0.08	0.10	0.12	0.02	0.02	0.03	0.00	0.02	0.01
GSM972509	0.00	0.03	0.02	0.09	0.00	0.22	0.17	0.04	0.00	0.01	0.01	0.00	0.00	0.14	0.07	0.04	0.08	0.03	0.00	0.00	0.06	0.00
GSM972510	0.00	0.04	0.13	0.06	0.00	0.40	0.00	0.00	0.02	0.01	0.00	0.00	0.00	0.00	0.04	0.21	0.02	0.02	0.00	0.04	0.00	0.00
GSM972511	0.00	0.04	0.04	0.00	0.00	0.24	0.06	0.00	0.00	0.00	0.00	0.00	0.00	0.11	0.09	0.11	0.13	0.00	0.00	0.11	0.04	0.04
GSM972512	0.10	0.00	0.12	0.07	0.00	0.15	0.02	0.11	0.00	0.00	0.03	0.01	0.00	0.15	0.02	0.12	0.04	0.04	0.00	0.02	0.00	0.01
GSM972513	0.00	0.04	0.00	0.10	0.00	0.20	0.04	0.03	0.01	0.00	0.03	0.00	0.00	0.18	0.06	0.17	0.00	0.06	0.00	0.06	0.00	0.01
GSM972515	0.03	0.00	0.05	0.07	0.00	0.28	0.07	0.00	0.01	0.00	0.05	0.00	0.01	0.11	0.06	0.12	0.02	0.05	0.00	0.03	0.00	0.03
GSM972516	0.03	0.00	0.00	0.24	0.00	0.12	0.10	0.00	0.07	0.00	0.00	0.02	0.03	0.15	0.11	0.01	0.09	0.00	0.00	0.01	0.00	0.00
GSM972517	0.02	0.04	0.03	0.00	0.00	0.14	0.06	0.06	0.03	0.00	0.02	0.01	0.00	0.17	0.14	0.09	0.11	0.00	0.00	0.04	0.03	0.00
GSM972518	0.05	0.01	0.09	0.13	0.00	0.00	0.00	0.12	0.00	0.01	0.00	0.01	0.00	0.15	0.07	0.17	0.03	0.00	0.00	0.06	0.00	0.08
GSM972519	0.00	0.03	0.03	0.16	0.00	0.07	0.00	0.03	0.03	0.00	0.10	0.00	0.00	0.12	0.01	0.08	0.01	0.03	0.00	0.24	0.01	0.04
GSM972520	0.00	0.02	0.08	0.02	0.00	0.20	0.05	0.05	0.00	0.03	0.00	0.00	0.07	0.10	0.10	0.07	0.04	0.01	0.04	0.01	0.08	0.04
GSM972521	0.01	0.01	0.01	0.19	0.00	0.00	0.13	0.07	0.00	0.00	0.01	0.00	0.00	0.14	0.14	0.18	0.04	0.01	0.02	0.02	0.00	0.01
GSM972522	0.08	0.00	0.07	0.14	0.00	0.11	0.02	0.04	0.06	0.00	0.03	0.00	0.00	0.11	0.07	0.11	0.10	0.02	0.00	0.04	0.00	0.01
GSM950417	0.00	0.04	0.16	0.13	0.00	0.10	0.08	0.01	0.06	0.00	0.03	0.00	0.00	0.09	0.03	0.10	0.04	0.02	0.00	0.09	0.00	0.00
GSM950418	0.06	0.00	0.05	0.17	0.00	0.23	0.00	0.03	0.05	0.00	0.07	0.00	0.02	0.11	0.04	0.01	0.05	0.00	0.00	0.11	0.00	0.00
GSM950419	0.00	0.05	0.02	0.18	0.00	0.22	0.00	0.05	0.03	0.00	0.07	0.00	0.06	0.10	0.04	0.00	0.01	0.02	0.00	0.15	0.02	0.00
GSM950420	0.04	0.00	0.04	0.21	0.00	0.04	0.11	0.07	0.01	0.00	0.01	0.02	0.02	0.06	0.14	0.08	0.07	0.00	0.00	0.06	0.00	0.02
GSM950421	0.01	0.01	0.00	0.13	0.00	0.04	0.05	0.02	0.04	0.00	0.04	0.00	0.00	0.24	0.08	0.07	0.08	0.00	0.00	0.13	0.00	0.07

GSM950422	0.00	0.01	0.06	0.06	0.00	0.11	0.04	0.00	0.00	0.01	0.07	0.00	0.01	0.13	0.04	0.14	0.01	0.01	0.00	0.17	0.06	0.08
GSM950423	0.01	0.00	0.03	0.08	0.00	0.07	0.00	0.01	0.07	0.00	0.00	0.00	0.00	0.29	0.12	0.19	0.04	0.00	0.00	0.07	0.00	0.01
GSM950424	0.04	0.00	0.06	0.07	0.00	0.04	0.02	0.02	0.02	0.00	0.02	0.01	0.00	0.36	0.03	0.14	0.00	0.00	0.00	0.15	0.00	0.01
GSM950425	0.03	0.00	0.18	0.08	0.00	0.18	0.01	0.04	0.00	0.01	0.00	0.00	0.00	0.09	0.10	0.11	0.12	0.00	0.05	0.00	0.00	0.00
GSM950426	0.00	0.00	0.04	0.14	0.00	0.16	0.14	0.01	0.00	0.00	0.04	0.00	0.04	0.14	0.03	0.20	0.01	0.05	0.00	0.00	0.00	0.00
GSM950428	0.23	0.00	0.03	0.07	0.01	0.08	0.14	0.03	0.00	0.00	0.09	0.00	0.01	0.13	0.04	0.11	0.01	0.00	0.00	0.00	0.01	0.00
GSM950429	0.05	0.00	0.03	0.12	0.00	0.10	0.10	0.03	0.00	0.00	0.06	0.02	0.00	0.13	0.16	0.07	0.03	0.00	0.00	0.07	0.00	0.04
GSM950430	0.02	0.01	0.03	0.17	0.00	0.10	0.03	0.02	0.00	0.00	0.03	0.00	0.00	0.17	0.08	0.07	0.00	0.00	0.00	0.16	0.00	0.10
GSM950431	0.04	0.00	0.08	0.15	0.00	0.26	0.00	0.00	0.08	0.00	0.06	0.00	0.00	0.12	0.03	0.00	0.02	0.00	0.00	0.17	0.00	0.00
GSM950432	0.09	0.00	0.03	0.13	0.00	0.29	0.00	0.07	0.02	0.00	0.00	0.05	0.00	0.12	0.05	0.06	0.03	0.00	0.00	0.05	0.00	0.00
GSM950433	0.05	0.00	0.01	0.11	0.00	0.15	0.06	0.00	0.00	0.00	0.08	0.00	0.02	0.22	0.06	0.07	0.04	0.01	0.00	0.06	0.00	0.06
GSM950434	0.05	0.00	0.04	0.10	0.00	0.26	0.13	0.00	0.02	0.00	0.07	0.00	0.00	0.14	0.03	0.04	0.03	0.00	0.00	0.09	0.00	0.00
GSM950435	0.02	0.01	0.02	0.03	0.00	0.17	0.05	0.03	0.00	0.00	0.05	0.00	0.00	0.16	0.09	0.06	0.02	0.00	0.00	0.23	0.00	0.05
GSM950436	0.02	0.00	0.01	0.03	0.00	0.15	0.06	0.01	0.03	0.00	0.03	0.00	0.00	0.14	0.10	0.04	0.02	0.00	0.00	0.18	0.00	0.18
GSM950437	0.04	0.00	0.05	0.21	0.00	0.14	0.08	0.00	0.01	0.00	0.06	0.00	0.01	0.09	0.04	0.05	0.03	0.02	0.02	0.09	0.00	0.07
GSM950438	0.04	0.01	0.06	0.18	0.00	0.25	0.00	0.00	0.05	0.00	0.02	0.00	0.01	0.07	0.04	0.10	0.05	0.02	0.03	0.07	0.00	0.00
GSM950439	0.17	0.00	0.13	0.08	0.00	0.19	0.04	0.01	0.03	0.00	0.06	0.00	0.00	0.06	0.06	0.01	0.03	0.02	0.00	0.08	0.00	0.02
GSM950440	0.09	0.00	0.03	0.16	0.00	0.17	0.00	0.00	0.09	0.00	0.01	0.00	0.00	0.10	0.06	0.08	0.02	0.01	0.04	0.10	0.00	0.01
GSM950441	0.02	0.07	0.08	0.15	0.00	0.21	0.00	0.00	0.06	0.00	0.07	0.00	0.02	0.00	0.04	0.11	0.09	0.00	0.03	0.05	0.00	0.00
GSM950442	0.02	0.00	0.03	0.11	0.00	0.23	0.02	0.03	0.06	0.00	0.01	0.00	0.00	0.13	0.12	0.11	0.03	0.00	0.00	0.10	0.00	0.02
GSM950443	0.05	0.05	0.04	0.08	0.00	0.25	0.00	0.01	0.07	0.00	0.04	0.00	0.00	0.12	0.05	0.05	0.01	0.00	0.00	0.12	0.00	0.03
GSM950444	0.04	0.00	0.02	0.06	0.00	0.16	0.01	0.01	0.00	0.00	0.06	0.00	0.03	0.10	0.03	0.10	0.00	0.04	0.00	0.17	0.00	0.16
GSM950445	0.01	0.00	0.06	0.04	0.00	0.08	0.05	0.04	0.00	0.01	0.00	0.05	0.00	0.33	0.12	0.17	0.02	0.01	0.04	0.00	0.00	0.00
GSM950446	0.01	0.00	0.12	0.11	0.00	0.11	0.01	0.07	0.00	0.00	0.01	0.00	0.01	0.16	0.08	0.10	0.10	0.02	0.00	0.05	0.02	0.01
GSM950447	0.00	0.00	0.04	0.17	0.00	0.00	0.08	0.05	0.00	0.00	0.06	0.00	0.00	0.27	0.13	0.09	0.02	0.02	0.00	0.06	0.00	0.00
GSM950448	0.00	0.00	0.01	0.06	0.00	0.15	0.08	0.07	0.00	0.00	0.06	0.04	0.04	0.26	0.05	0.10	0.03	0.00	0.00	0.03	0.01	0.00
GSM950449	0.00	0.02	0.02	0.09	0.00	0.02	0.09	0.08	0.00	0.00	0.05	0.00	0.00	0.17	0.14	0.11	0.09	0.06	0.00	0.04	0.00	0.01
GSM950450	0.01	0.00	0.12	0.12	0.00	0.06	0.13	0.09	0.00	0.00	0.06	0.00	0.00	0.06	0.10	0.07	0.12	0.04	0.00	0.01	0.00	0.01
GSM950452	0.02	0.00	0.02	0.19	0.00	0.04	0.05	0.08	0.04	0.00	0.01	0.00	0.00	0.04	0.06	0.15	0.05	0.19	0.00	0.02	0.02	0.00
GSM950453	0.07	0.00	0.19	0.12	0.00	0.13	0.08	0.01	0.00	0.05	0.01	0.00	0.00	0.09	0.05	0.09	0.00	0.02	0.00	0.05	0.00	0.02
GSM950454	0.00	0.00	0.17	0.08	0.00	0.17	0.03	0.04	0.00	0.03	0.05	0.00	0.00	0.08	0.05	0.13	0.07	0.05	0.00	0.04	0.00	0.00
GSM950455	0.05	0.00	0.12	0.07	0.00	0.12	0.14	0.02	0.00	0.00	0.03	0.03	0.00	0.03	0.14	0.09	0.01	0.03	0.00	0.06	0.04	0.02
GSM950457	0.02	0.00	0.01	0.08	0.00	0.07	0.05	0.10	0.00	0.04	0.00	0.01	0.00	0.16	0.10	0.12	0.04	0.04	0.00	0.04	0.07	0.04
GSM950459	0.03	0.00	0.04	0.01	0.00	0.04	0.14	0.02	0.02	0.05	0.00	0.00	0.01	0.23	0.15	0.10	0.04	0.06	0.05	0.00	0.01	0.01
GSM950460	0.04	0.00	0.01	0.22	0.00	0.10	0.08	0.08	0.04	0.00	0.00	0.06	0.00	0.08	0.04	0.16	0.07	0.01	0.00	0.00	0.00	0.00
GSM950461	0.02	0.00	0.01	0.09	0.00	0.05	0.00	0.05	0.06	0.00	0.00	0.00	0.00	0.40	0.05	0.17	0.01	0.04	0.00	0.06	0.00	0.00
GSM950462	0.02	0.00	0.03	0.08	0.00	0.15	0.03	0.08	0.00	0.05	0.02	0.00	0.00	0.19	0.11	0.07	0.10	0.04	0.00	0.03	0.00	0.00
GSM950463	0.00	0.02	0.16	0.13	0.00	0.17	0.05	0.03	0.00	0.01	0.06	0.00	0.00	0.06	0.06	0.15	0.01	0.00	0.00	0.08	0.00	0.00

GSM950465	0.00	0.00	0.10	0.08	0.00	0.11	0.04	0.06	0.02	0.05	0.01	0.00	0.00	0.16	0.12	0.12	0.05	0.00	0.00	0.04	0.01	0.03
GSM950467	0.00	0.03	0.02	0.10	0.00	0.07	0.08	0.05	0.00	0.00	0.02	0.00	0.00	0.24	0.10	0.08	0.11	0.07	0.00	0.02	0.00	0.02
GSM950468	0.03	0.01	0.09	0.06	0.00	0.15	0.07	0.02	0.00	0.04	0.00	0.04	0.00	0.14	0.12	0.15	0.03	0.00	0.01	0.03	0.00	0.00
GSM950469	0.01	0.06	0.07	0.06	0.00	0.18	0.04	0.06	0.00	0.03	0.00	0.08	0.00	0.09	0.10	0.12	0.02	0.04	0.05	0.00	0.00	0.00
GSM950470	0.03	0.00	0.04	0.14	0.00	0.06	0.07	0.01	0.05	0.01	0.00	0.00	0.00	0.18	0.06	0.16	0.16	0.00	0.00	0.02	0.00	0.01
GSM950471	0.08	0.00	0.23	0.10	0.00	0.08	0.06	0.00	0.03	0.00	0.02	0.00	0.00	0.22	0.02	0.10	0.01	0.02	0.00	0.02	0.00	0.00
GSM950472	0.00	0.00	0.14	0.12	0.00	0.09	0.06	0.07	0.04	0.03	0.05	0.00	0.00	0.04	0.13	0.08	0.12	0.02	0.00	0.02	0.00	0.00
GSM950474	0.19	0.00	0.01	0.08	0.00	0.22	0.01	0.09	0.02	0.04	0.06	0.00	0.00	0.03	0.01	0.09	0.01	0.04	0.00	0.08	0.00	0.00
GSM950475	0.00	0.03	0.06	0.10	0.00	0.14	0.02	0.08	0.00	0.05	0.01	0.03	0.00	0.10	0.09	0.19	0.02	0.01	0.00	0.06	0.00	0.00
GSM950476	0.05	0.00	0.05	0.09	0.00	0.13	0.05	0.06	0.00	0.00	0.00	0.00	0.00	0.14	0.14	0.08	0.00	0.02	0.00	0.08	0.00	0.09
GSM950477	0.10	0.00	0.08	0.09	0.00	0.13	0.13	0.01	0.00	0.00	0.05	0.00	0.00	0.13	0.02	0.09	0.13	0.00	0.01	0.03	0.00	0.00
GSM950478	0.00	0.00	0.03	0.04	0.00	0.25	0.04	0.05	0.00	0.00	0.02	0.00	0.00	0.12	0.02	0.15	0.07	0.10	0.11	0.00	0.00	0.01
GSM950479	0.00	0.09	0.09	0.07	0.00	0.20	0.05	0.05	0.01	0.04	0.01	0.00	0.00	0.08	0.04	0.12	0.05	0.03	0.00	0.06	0.00	0.00
GSM950480	0.02	0.01	0.08	0.08	0.00	0.13	0.12	0.12	0.00	0.00	0.02	0.01	0.04	0.13	0.06	0.07	0.07	0.02	0.00	0.00	0.00	0.00
GSM950481	0.00	0.01	0.04	0.16	0.00	0.00	0.19	0.07	0.00	0.02	0.04	0.00	0.00	0.11	0.12	0.07	0.07	0.03	0.00	0.05	0.00	0.00
GSM950483	0.13	0.00	0.06	0.08	0.00	0.18	0.06	0.12	0.00	0.00	0.00	0.00	0.00	0.06	0.07	0.08	0.06	0.02	0.02	0.02	0.02	0.02
GSM950484	0.00	0.00	0.12	0.09	0.00	0.04	0.10	0.05	0.00	0.06	0.00	0.00	0.00	0.21	0.13	0.08	0.07	0.00	0.00	0.03	0.00	0.01
GSM950485	0.00	0.00	0.11	0.11	0.00	0.11	0.20	0.03	0.00	0.00	0.02	0.00	0.00	0.08	0.06	0.13	0.09	0.03	0.03	0.00	0.00	0.00
GSM950486	0.00	0.06	0.07	0.09	0.00	0.07	0.18	0.02	0.00	0.00	0.00	0.01	0.00	0.04	0.00	0.18	0.07	0.11	0.05	0.00	0.06	0.00
GSM950487	0.00	0.00	0.01	0.01	0.01	0.06	0.08	0.00	0.00	0.00	0.05	0.00	0.00	0.39	0.03	0.19	0.05	0.04	0.00	0.07	0.00	0.00
GSM950489	0.01	0.00	0.08	0.05	0.00	0.32	0.00	0.01	0.00	0.02	0.01	0.00	0.00	0.00	0.04	0.21	0.14	0.00	0.09	0.01	0.02	0.00
GSM950490	0.04	0.00	0.11	0.05	0.00	0.08	0.06	0.06	0.00	0.07	0.00	0.00	0.00	0.08	0.14	0.15	0.04	0.00	0.00	0.05	0.05	0.02
GSM950492	0.05	0.00	0.13	0.02	0.00	0.15	0.00	0.07	0.01	0.01	0.03	0.00	0.00	0.08	0.07	0.14	0.03	0.01	0.00	0.15	0.03	0.03
GSM950495	0.00	0.00	0.08	0.02	0.00	0.13	0.05	0.02	0.00	0.03	0.05	0.00	0.00	0.15	0.11	0.27	0.00	0.00	0.00	0.05	0.01	0.03
GSM950496	0.02	0.00	0.07	0.11	0.00	0.12	0.13	0.07	0.00	0.01	0.01	0.04	0.01	0.09	0.14	0.10	0.03	0.00	0.05	0.00	0.00	0.00
GSM950497	0.04	0.00	0.02	0.05	0.00	0.06	0.09	0.01	0.00	0.00	0.09	0.00	0.00	0.34	0.01	0.16	0.00	0.02	0.00	0.11	0.00	0.00
GSM950499	0.01	0.00	0.05	0.18	0.00	0.16	0.05	0.00	0.01	0.00	0.01	0.00	0.03	0.05	0.09	0.21	0.06	0.02	0.00	0.08	0.00	0.00
GSM950500	0.03	0.00	0.05	0.14	0.00	0.00	0.12	0.07	0.00	0.02	0.00	0.03	0.00	0.12	0.12	0.08	0.05	0.00	0.00	0.08	0.03	0.03
GSM950503	0.14	0.00	0.05	0.08	0.00	0.13	0.05	0.04	0.00	0.02	0.02	0.00	0.00	0.05	0.05	0.12	0.06	0.04	0.00	0.06	0.07	0.03
GSM950504	0.03	0.00	0.03	0.11	0.00	0.36	0.04	0.02	0.00	0.00	0.02	0.00	0.01	0.09	0.02	0.10	0.07	0.06	0.00	0.04	0.01	0.00
GSM950505	0.01	0.00	0.02	0.12	0.00	0.05	0.08	0.00	0.00	0.00	0.03	0.00	0.02	0.22	0.09	0.07	0.00	0.01	0.00	0.10	0.00	0.16
GSM950506	0.00	0.02	0.03	0.21	0.00	0.14	0.00	0.02	0.00	0.00	0.00	0.00	0.01	0.02	0.06	0.12	0.20	0.01	0.00	0.11	0.04	0.00
GSM950507	0.01	0.01	0.05	0.13	0.00	0.03	0.00	0.08	0.01	0.00	0.00	0.00	0.00	0.23	0.13	0.14	0.06	0.00	0.00	0.12	0.00	0.00
GSM950509	0.06	0.00	0.02	0.30	0.00	0.01	0.03	0.06	0.09	0.00	0.00	0.03	0.02	0.09	0.13	0.03	0.05	0.00	0.00	0.07	0.01	0.00
GSM950512	0.00	0.07	0.05	0.26	0.00	0.11	0.08	0.02	0.03	0.00	0.04	0.00	0.00	0.05	0.04	0.06	0.13	0.01	0.00	0.05	0.00	0.00
GSM950513	0.11	0.00	0.05	0.17	0.00	0.10	0.00	0.07	0.06	0.00	0.08	0.00	0.00	0.10	0.08	0.09	0.00	0.00	0.00	0.08	0.00	0.00
GSM950514	0.07	0.00	0.03	0.14	0.00	0.16	0.03	0.05	0.03	0.00	0.07	0.00	0.00	0.10	0.08	0.09	0.06	0.00	0.00	0.09	0.00	0.00
GSM950516	0.05	0.02	0.00	0.23	0.00	0.08	0.06	0.06	0.09	0.00	0.05	0.00	0.00	0.05	0.07	0.09	0.04	0.04	0.00	0.07	0.00	0.00

GSM950517	0.03	0.00	0.00	0.15	0.00	0.08	0.08	0.10	0.00	0.00	0.01	0.03	0.00	0.14	0.16	0.08	0.04	0.00	0.00	0.07	0.00	0.01
GSM950519	0.00	0.04	0.03	0.20	0.00	0.09	0.11	0.01	0.09	0.00	0.05	0.00	0.01	0.10	0.04	0.11	0.04	0.00	0.00	0.08	0.01	0.00
GSM950520	0.09	0.00	0.01	0.20	0.00	0.11	0.08	0.06	0.03	0.00	0.02	0.00	0.00	0.09	0.09	0.08	0.07	0.00	0.06	0.00	0.00	0.00
GSM950521	0.02	0.00	0.03	0.28	0.00	0.02	0.11	0.05	0.03	0.00	0.10	0.00	0.00	0.07	0.11	0.13	0.02	0.00	0.00	0.03	0.00	0.01
GSM950522	0.04	0.00	0.01	0.18	0.00	0.09	0.26	0.00	0.00	0.00	0.09	0.00	0.00	0.10	0.03	0.08	0.02	0.03	0.00	0.06	0.00	0.00
GSM950523	0.01	0.02	0.09	0.13	0.00	0.22	0.04	0.01	0.04	0.00	0.07	0.00	0.00	0.07	0.08	0.07	0.01	0.01	0.00	0.10	0.01	0.02
GSM950525	0.07	0.00	0.05	0.07	0.00	0.19	0.02	0.00	0.07	0.00	0.05	0.00	0.00	0.22	0.06	0.07	0.03	0.00	0.00	0.08	0.00	0.00
GSM950528	0.10	0.00	0.10	0.19	0.00	0.01	0.06	0.08	0.01	0.00	0.06	0.01	0.00	0.08	0.11	0.10	0.03	0.01	0.01	0.01	0.00	0.00
GSM950529	0.04	0.06	0.05	0.13	0.00	0.08	0.07	0.05	0.08	0.00	0.10	0.00	0.02	0.06	0.01	0.04	0.02	0.02	0.00	0.17	0.00	0.00
GSM950531	0.03	0.00	0.08	0.09	0.00	0.18	0.01	0.00	0.00	0.00	0.07	0.00	0.00	0.12	0.05	0.13	0.02	0.01	0.00	0.09	0.05	0.06
GSM950532	0.00	0.00	0.06	0.21	0.00	0.00	0.09	0.11	0.04	0.04	0.00	0.02	0.00	0.04	0.14	0.14	0.04	0.00	0.01	0.00	0.02	0.03

Note:from C1 to C22 indicates B cells na ïve, B cells memory, Plasma cells, T cells CD8, T cells CD4 na ïve, T cells CD4 memory resting, T cells CD4 memory activated,T cells follicular helper,T cells regulatory (Tregs),T cells gamma delta,NK cells resting,NK cells activated,Monocytes,Macrophages M0 Macrophages M1,Macrophages M2,DC resting,DC activated,Mast cells resting,Mast cells activated,Eosinophils,Neutrophils

Table S7. Functional analysis of the 702 differentially expressed genes between the cuproptosis subtypes .

Type	ONTO	ID	Description	Ratio	BgRatio	pvalue	p.adjust	qvalue	geneID	Count
GO	BP	GO:0060326	cell chemotaxis	58/647	310/18723	2.07E-26	6.1E-23	4.27E-23	NRP1/VCAM1/C3	58
GO	BP	GO:0050900	leukocyte migration	63/647	369/18723	2.6E-26	6.1E-23	4.27E-23	IL1R1/VCAM1/C3	63
GO	BP	GO:0097529	myeloid leukocyte migration	49/647	220/18723	5.13E-26	8.02E-23	5.62E-23	IL1R1/C3AR1/SL	49
GO	BP	GO:0030595	leukocyte chemotaxis	48/647	230/18723	3.48E-24	4.08E-21	2.86E-21	C3AR1/SLAMF8/	48
GO	BP	GO:0097530	granulocyte migration	37/647	148/18723	8.88E-22	7.08E-19	4.96E-19	IL1R1/C3AR1/SL	37
GO	BP	GO:1990266	neutrophil migration	34/647	122/18723	9.06E-22	7.08E-19	4.96E-19	IL1R1/C3AR1/SL	34
GO	BP	GO:0071621	granulocyte chemotaxis	34/647	125/18723	2.15E-21	1.44E-18	1.01E-18	C3AR1/NCKAP1I	34
GO	BP	GO:0032103	positive regulation of response to external	61/647	427/18723	2.46E-21	1.44E-18	1.01E-18	NRP1/CLEC7A/M	61
GO	BP	GO:0030593	neutrophil chemotaxis	31/647	103/18723	4.64E-21	2.42E-18	1.69E-18	C3AR1/NCKAP1I	31
GO	BP	GO:0030198	extracellular matrix organization	48/647	301/18723	5.39E-19	2.53E-16	1.77E-16	FAP/PHLDB2/LO	48
GO	BP	GO:0043062	extracellular structure organization	48/647	302/18723	6.2E-19	2.64E-16	1.85E-16	FAP/PHLDB2/LO	48
GO	BP	GO:0045229	external encapsulating structure organizati	48/647	304/18723	8.2E-19	3.21E-16	2.25E-16	FAP/PHLDB2/LO	48
GO	BP	GO:0001819	positive regulation of cytokine production	59/647	467/18723	4.88E-18	1.76E-15	1.23E-15	TLR1/P2RX7/CLI	59
GO	BP	GO:1990868	response to chemokine	27/647	97/18723	1.55E-17	4.83E-15	3.38E-15	LOX/DOCK8/CCI	27
GO	BP	GO:1990869	cellular response to chemokine	27/647	97/18723	1.55E-17	4.83E-15	3.38E-15	LOX/DOCK8/CCI	27
GO	BP	GO:0002683	negative regulation of immune system pro	55/647	434/18723	6.09E-17	1.78E-14	1.25E-14	MAFB/GPNMB/C	55
GO	BP	GO:0070098	chemokine-mediated signaling pathway	25/647	88/18723	1.44E-16	3.75E-14	2.63E-14	CCR1/CXCR4/CC	25
GO	BP	GO:0070661	leukocyte proliferation	46/647	318/18723	1.48E-16	3.75E-14	2.63E-14	GPNMB/CD86/V(	46
GO	BP	GO:0071674	mononuclear cell migration	36/647	196/18723	1.52E-16	3.75E-14	2.63E-14	C3AR1/DOCK8/S	36
GO	BP	GO:0050921	positive regulation of chemotaxis	30/647	141/18723	8.16E-16	1.91E-13	1.34E-13	NRP1/C3AR1/VE	30
GO	BP	GO:0002548	monocyte chemotaxis	22/647	70/18723	8.97E-16	2E-13	1.4E-13	SLAMF8/CCR1/C	22
GO	BP	GO:0002237	response to molecule of bacterial origin	48/647	363/18723	1.19E-15	2.53E-13	1.78E-13	TLR1/CD86/VCA	48
GO	BP	GO:0002685	regulation of leukocyte migration	36/647	210/18723	1.47E-15	3E-13	2.11E-13	IL1R1/C3AR1/DC	36
GO	BP	GO:0032496	response to lipopolysaccharide	46/647	343/18723	2.79E-15	5.28E-13	3.7E-13	CD86/VCAM1/MI	46
GO	BP	GO:0007159	leukocyte cell-cell adhesion	48/647	371/18723	2.81E-15	5.28E-13	3.7E-13	GPNMB/CD86/V(	48
GO	BP	GO:0045785	positive regulation of cell adhesion	52/647	437/18723	6.21E-15	1.12E-12	7.85E-13	NRP1/CD86/VCA	52
GO	BP	GO:0070663	regulation of leukocyte proliferation	38/647	245/18723	6.98E-15	1.21E-12	8.5E-13	GPNMB/CD86/V(	38
GO	BP	GO:0042110	T cell activation	55/647	487/18723	9.38E-15	1.57E-12	1.1E-12	CLEC7A/MAFB/(	55
GO	BP	GO:0001503	ossification	49/647	408/18723	2.72E-14	4.26E-12	2.98E-12	P2RX7/RASSF2/(	49
GO	BP	GO:1903037	regulation of leukocyte cell-cell adhesion	44/647	336/18723	2.72E-14	4.26E-12	2.98E-12	GPNMB/CD86/V(	44
GO	BP	GO:0050866	negative regulation of cell activation	34/647	210/18723	5.22E-14	7.9E-12	5.53E-12	GPNMB/CD86/M	34
GO	BP	GO:0002274	myeloid leukocyte activation	35/647	223/18723	5.76E-14	8.17E-12	5.72E-12	TLR1/ITGAM/LC	35
GO	BP	GO:0050920	regulation of chemotaxis	35/647	223/18723	5.76E-14	8.17E-12	5.72E-12	NRP1/C3AR1/SL/	35
GO	BP	GO:0046651	lymphocyte proliferation	40/647	288/18723	5.92E-14	8.17E-12	5.72E-12	GPNMB/CD86/V(	40
GO	BP	GO:0002253	activation of immune response	46/647	375/18723	7.95E-14	1.06E-11	7.46E-12	CLEC7A/CFH/MI	46
GO	BP	GO:0032943	mononuclear cell proliferation	40/647	291/18723	8.39E-14	1.09E-11	7.65E-12	GPNMB/CD86/V(	40

GO	BP	GO:0031349	positive regulation of defense response	39/647	278/18723	8.85E-14	1.12E-11	7.86E-12	CLEC7A/MNDA/	39
GO	BP	GO:1903039	positive regulation of leukocyte cell-cell a	36/647	239/18723	9.1E-14	1.12E-11	7.87E-12	CD86/VCAM1/TN	36
GO	BP	GO:0030199	collagen fibril organization	19/647	61/18723	1E-13	1.21E-11	8.46E-12	LOX/DDR2/COL5	19
GO	BP	GO:0002687	positive regulation of leukocyte migration	27/647	135/18723	1.14E-13	1.33E-11	9.33E-12	IL1R1/C3AR1/DC	27
GO	BP	GO:0019221	cytokine-mediated signaling pathway	52/647	472/18723	1.41E-13	1.61E-11	1.13E-11	IL1R1/OSMR/TN	52
GO	BP	GO:0002697	regulation of immune effector process	43/647	339/18723	1.66E-13	1.85E-11	1.29E-11	ITGAM/CLEC7A/	43
GO	BP	GO:0050863	regulation of T cell activation	42/647	329/18723	2.57E-13	2.8E-11	1.96E-11	GPNMB/CD86/V(	42
GO	BP	GO:0050867	positive regulation of cell activation	48/647	420/18723	3.27E-13	3.49E-11	2.44E-11	ITGAM/CLEC7A/	48
GO	BP	GO:0002695	negative regulation of leukocyte activation	31/647	187/18723	3.59E-13	3.74E-11	2.62E-11	GPNMB/CD86/M	31
GO	BP	GO:0002764	immune response-regulating signaling path	51/647	468/18723	3.74E-13	3.81E-11	2.67E-11	TLR1/CLEC7A/C	51
GO	BP	GO:0050670	regulation of lymphocyte proliferation	34/647	225/18723	4.08E-13	4.08E-11	2.86E-11	GPNMB/CD86/V(	34
GO	BP	GO:0002696	positive regulation of leukocyte activation	47/647	409/18723	4.77E-13	4.66E-11	3.27E-11	ITGAM/CLEC7A/	47
GO	BP	GO:0002688	regulation of leukocyte chemotaxis	25/647	122/18723	5.22E-13	4.97E-11	3.48E-11	C3AR1/SLAMF8/	25
GO	BP	GO:0032944	regulation of mononuclear cell proliferatic	34/647	227/18723	5.3E-13	4.97E-11	3.48E-11	GPNMB/CD86/V(	34
GO	BP	GO:0050870	positive regulation of T cell activation	33/647	216/18723	6.64E-13	6.1E-11	4.28E-11	CD86/VCAM1/TN	33
GO	BP	GO:0022409	positive regulation of cell-cell adhesion	38/647	284/18723	8.35E-13	7.53E-11	5.27E-11	CD86/VCAM1/TN	38
GO	BP	GO:0042098	T cell proliferation	31/647	199/18723	1.98E-12	1.75E-10	1.23E-10	GPNMB/CD86/V(	31
GO	BP	GO:1902105	regulation of leukocyte differentiation	37/647	279/18723	2.2E-12	1.91E-10	1.34E-10	MAFB/RASSF2/M	37
GO	BP	GO:1903706	regulation of hemopoiesis	43/647	367/18723	2.53E-12	2.16E-10	1.51E-10	MAFB/RASSF2/M	43
GO	BP	GO:0050727	regulation of inflammatory response	44/647	386/18723	3.59E-12	3E-10	2.1E-10	IL1R1/OSMR/FC(	44
GO	BP	GO:0002460	adaptive immune response based on somat	42/647	356/18723	3.65E-12	3E-10	2.1E-10	CLEC7A/IL1R1/T	42
GO	BP	GO:0071216	cellular response to biotic stimulus	34/647	246/18723	5.43E-12	4.39E-10	3.07E-10	TLR1/CLEC7A/C	34
GO	BP	GO:0071219	cellular response to molecule of bacterial (	32/647	221/18723	6.54E-12	5.17E-10	3.62E-10	TLR1/CD86/MRC	32
GO	BP	GO:0002690	positive regulation of leukocyte chemotax	21/647	94/18723	6.62E-12	5.17E-10	3.62E-10	C3AR1/VEGFC/C	21
GO	BP	GO:0071675	regulation of mononuclear cell migration	23/647	115/18723	7.61E-12	5.85E-10	4.1E-10	C3AR1/DOCK8/S	23
GO	BP	GO:0055074	calcium ion homeostasis	48/647	460/18723	9.09E-12	6.88E-10	4.82E-10	P2RX7/PKD2/C3/	48
GO	BP	GO:0006874	cellular calcium ion homeostasis	47/647	448/18723	1.25E-11	9.16E-10	6.42E-10	P2RX7/PKD2/C3/	47
GO	BP	GO:0022407	regulation of cell-cell adhesion	47/647	448/18723	1.25E-11	9.16E-10	6.42E-10	GPNMB/CD86/V(	47
GO	BP	GO:0019722	calcium-mediated signaling	30/647	202/18723	1.56E-11	1.13E-09	7.9E-10	P2RX7/CLEC7A/	30
GO	BP	GO:0072503	cellular divalent inorganic cation homeost	49/647	486/18723	1.9E-11	1.35E-09	9.44E-10	P2RX7/PKD2/C3/	49
GO	BP	GO:0002443	leukocyte mediated immunity	46/647	440/18723	2.35E-11	1.64E-09	1.15E-09	ITGAM/CLEC7A/	46
GO	BP	GO:0051251	positive regulation of lymphocyte activatic	41/647	362/18723	2.45E-11	1.69E-09	1.18E-09	CLEC7A/CD86/V	41
GO	BP	GO:0050777	negative regulation of immune response	29/647	194/18723	2.91E-11	1.97E-09	1.38E-09	FCGR2B/CD84/SI	29
GO	BP	GO:0007204	positive regulation of cytosolic calcium io	38/647	319/18723	2.98E-11	1.97E-09	1.38E-09	P2RX7/PKD2/C3/	38
GO	BP	GO:0051250	negative regulation of lymphocyte activati	26/647	157/18723	2.98E-11	1.97E-09	1.38E-09	GPNMB/CD86/M	26
GO	BP	GO:0048247	lymphocyte chemotaxis	17/647	64/18723	3.52E-11	2.29E-09	1.61E-09	CCL2/PIK3CG/GI	17
GO	BP	GO:0043410	positive regulation of MAPK cascade	48/647	480/18723	4.09E-11	2.63E-09	1.84E-09	NRP1/CLEC7A/R	48
GO	BP	GO:0072676	lymphocyte migration	22/647	117/18723	7.78E-11	4.93E-09	3.45E-09	DOCK8/CCL2/PII	22

GO	BP	GO:0006898	receptor-mediated endocytosis	32/647	244/18723	9.29E-11	5.81E-09	4.07E-09	ITGAM/RAB31/M	32
GO	BP	GO:1903131	mononuclear cell differentiation	44/647	426/18723	9.62E-11	5.94E-09	4.16E-09	MAFB/DOCK10/(	44
GO	BP	GO:0030282	bone mineralization	22/647	119/18723	1.1E-10	6.69E-09	4.69E-09	P2RX7/GPNMB/I	22
GO	BP	GO:0002822	regulation of adaptive immune response ba	26/647	168/18723	1.41E-10	8.49E-09	5.95E-09	CLEC7A/IL1R1/T	26
GO	BP	GO:0051480	regulation of cytosolic calcium ion concen	39/647	353/18723	1.6E-10	9.48E-09	6.64E-09	P2RX7/PKD2/C3/	39
GO	BP	GO:0006909	phagocytosis	36/647	308/18723	1.71E-10	1E-08	7.01E-09	ITGAM/CLEC7A/	36
GO	BP	GO:0071222	cellular response to lipopolysaccharide	29/647	209/18723	1.83E-10	1.06E-08	7.41E-09	CD86/MRC1/LY9	29
GO	BP	GO:0002819	regulation of adaptive immune response	27/647	183/18723	1.9E-10	1.09E-08	7.63E-09	CLEC7A/IL1R1/T	27
GO	BP	GO:0042129	regulation of T cell proliferation	26/647	171/18723	2.11E-10	1.19E-08	8.34E-09	GPNMB/CD86/V(	26
GO	BP	GO:0019932	second-messenger-mediated signaling	36/647	312/18723	2.45E-10	1.37E-08	9.57E-09	P2RX7/CLEC7A/	36
GO	BP	GO:0030098	lymphocyte differentiation	40/647	374/18723	2.48E-10	1.37E-08	9.57E-09	MAFB/DOCK10/(	40
GO	BP	GO:0002703	regulation of leukocyte mediated immunit	30/647	226/18723	2.68E-10	1.46E-08	1.02E-08	ITGAM/CLEC7A/	30
GO	BP	GO:1901342	regulation of vasculature development	38/647	348/18723	3.87E-10	2.08E-08	1.46E-08	NRP1/GPNMB/C3	38
GO	BP	GO:0051216	cartilage development	27/647	190/18723	4.52E-10	2.39E-08	1.67E-08	PRRX1/CHST11/S	27
GO	BP	GO:0070374	positive regulation of ERK1 and ERK2 ca	29/647	217/18723	4.53E-10	2.39E-08	1.67E-08	NRP1/GPNMB/FI	29
GO	BP	GO:0002366	leukocyte activation involved in immune r	33/647	275/18723	5.02E-10	2.61E-08	1.83E-08	ITGAM/CLEC7A/	33
GO	BP	GO:0050764	regulation of phagocytosis	19/647	95/18723	5.07E-10	2.61E-08	1.83E-08	CLEC7A/RAB31/	19
GO	BP	GO:0032102	negative regulation of response to external	42/647	420/18723	7.04E-10	3.59E-08	2.51E-08	NRP1/FAP/FCGR	42
GO	BP	GO:0070372	regulation of ERK1 and ERK2 cascade	35/647	309/18723	7.23E-10	3.64E-08	2.55E-08	NRP1/GPNMB/FI	35
GO	BP	GO:0002263	cell activation involved in immune respon	33/647	279/18723	7.31E-10	3.65E-08	2.55E-08	ITGAM/CLEC7A/	33
GO	BP	GO:0042060	wound healing	42/647	422/18723	8.14E-10	4.02E-08	2.81E-08	CLEC7A/ENTPD1	42
GO	BP	GO:0045765	regulation of angiogenesis	37/647	342/18723	8.57E-10	4.19E-08	2.93E-08	NRP1/GPNMB/C3	37
GO	BP	GO:0002831	regulation of response to biotic stimulus	36/647	327/18723	9.03E-10	4.36E-08	3.06E-08	CLEC7A/MNDA/	36
GO	BP	GO:0002698	negative regulation of immune effector pro	20/647	110/18723	1.05E-09	5.04E-08	3.53E-08	FCGR2B/CD84/SI	20
GO	BP	GO:0002768	immune response-regulating cell surface re	35/647	315/18723	1.21E-09	5.74E-08	4.02E-08	CLEC7A/MNDA/	35
GO	BP	GO:0006959	humoral immune response	35/647	317/18723	1.44E-09	6.73E-08	4.72E-08	CFH/FCGR2B/PT	35
GO	BP	GO:0090025	regulation of monocyte chemotaxis	11/647	29/18723	1.51E-09	7.03E-08	4.92E-08	SLAMF8/CCR1/S	11
GO	BP	GO:0070665	positive regulation of leukocyte proliferati	23/647	150/18723	1.97E-09	9.06E-08	6.35E-08	CD86/VCAM1/TN	23
GO	BP	GO:0045088	regulation of innate immune response	28/647	218/18723	2.26E-09	1.03E-07	7.2E-08	CLEC7A/MNDA/	28
GO	BP	GO:0006968	cellular defense response	14/647	54/18723	2.72E-09	1.23E-07	8.6E-08	MNDA/LY96/NC1	14
GO	BP	GO:0042116	macrophage activation	19/647	106/18723	3.53E-09	1.58E-07	1.1E-07	TLR1/ITGAM/FC	19
GO	BP	GO:0061448	connective tissue development	30/647	252/18723	3.74E-09	1.66E-07	1.16E-07	PRRX1/CHST11/S	30
GO	BP	GO:0070555	response to interleukin-1	22/647	143/18723	4.15E-09	1.8E-07	1.26E-07	IL1R1/CCL2/AKA	22
GO	BP	GO:0070371	ERK1 and ERK2 cascade	35/647	330/18723	4.16E-09	1.8E-07	1.26E-07	NRP1/GPNMB/FI	35
GO	BP	GO:0001774	microglial cell activation	13/647	47/18723	4.24E-09	1.8E-07	1.26E-07	TLR1/ITGAM/TL	13
GO	BP	GO:0071677	positive regulation of mononuclear cell mi	15/647	65/18723	4.25E-09	1.8E-07	1.26E-07	DOCK8/CCR1/IT(	15
GO	BP	GO:0031214	biomineral tissue development	24/647	169/18723	4.26E-09	1.8E-07	1.26E-07	P2RX7/GPNMB/I	24
GO	BP	GO:1902107	positive regulation of leukocyte differentia	23/647	157/18723	4.87E-09	2.02E-07	1.42E-07	CD86/CCR1/NCK	23

GO	BP	GO:1903708	positive regulation of hemopoiesis	23/647	157/18723	4.87E-09	2.02E-07	1.42E-07	CD86/CCR1/NCK	23
GO	BP	GO:0110148	biomineralization	24/647	171/18723	5.41E-09	2.23E-07	1.56E-07	P2RX7/GPNMB/I	24
GO	BP	GO:0030217	T cell differentiation	30/647	257/18723	5.95E-09	2.43E-07	1.7E-07	MAFB/CD86/DO(	30
GO	BP	GO:0071706	tumor necrosis factor superfamily cytokin	25/647	186/18723	6.42E-09	2.57E-07	1.8E-07	TLR1/CLEC7A/G	25
GO	BP	GO:1903555	regulation of tumor necrosis factor superfa	25/647	186/18723	6.42E-09	2.57E-07	1.8E-07	TLR1/CLEC7A/G	25
GO	BP	GO:0050673	epithelial cell proliferation	41/647	437/18723	7.32E-09	2.91E-07	2.04E-07	NRP1/SNAI2/FAI	41
GO	BP	GO:0002429	immune response-activating cell surface re	32/647	291/18723	8.07E-09	3.16E-07	2.21E-07	CLEC7A/MNDA/	32
GO	BP	GO:0002757	immune response-activating signal transdu	32/647	291/18723	8.07E-09	3.16E-07	2.21E-07	CLEC7A/MNDA/	32
GO	BP	GO:0050671	positive regulation of lymphocyte prolifera	21/647	137/18723	9.98E-09	3.87E-07	2.71E-07	CD86/VCAM1/TN	21
GO	BP	GO:0070664	negative regulation of leukocyte proliferat	17/647	90/18723	1.03E-08	3.97E-07	2.78E-07	GPNMB/CD86/M	17
GO	BP	GO:0071347	cellular response to interleukin-1	19/647	113/18723	1.06E-08	4.05E-07	2.84E-07	IL1R1/CCL2/AKA	19
GO	BP	GO:0032946	positive regulation of mononuclear cell pr	21/647	138/18723	1.14E-08	4.31E-07	3.02E-07	CD86/VCAM1/TN	21
GO	BP	GO:0018212	peptidyl-tyrosine modification	37/647	378/18723	1.32E-08	4.94E-07	3.46E-07	NRP1/CLEC7A/P	37
GO	BP	GO:2000403	positive regulation of lymphocyte migratic	11/647	35/18723	1.51E-08	5.62E-07	3.94E-07	DOCK8/ITGA4/JA	11
GO	BP	GO:1903038	negative regulation of leukocyte cell-cell a	21/647	141/18723	1.68E-08	6.14E-07	4.3E-07	GPNMB/CD86/FC	21
GO	BP	GO:0032640	tumor necrosis factor production	24/647	181/18723	1.69E-08	6.14E-07	4.3E-07	TLR1/CLEC7A/G	24
GO	BP	GO:0032680	regulation of tumor necrosis factor produc	24/647	181/18723	1.69E-08	6.14E-07	4.3E-07	TLR1/CLEC7A/G	24
GO	BP	GO:0002449	lymphocyte mediated immunity	35/647	350/18723	1.9E-08	6.86E-07	4.8E-07	IL1R1/TLR8/FCG	35
GO	BP	GO:0051924	regulation of calcium ion transport	29/647	255/18723	1.92E-08	6.88E-07	4.82E-07	P2RX7/PKD2/CD	29
GO	BP	GO:0007162	negative regulation of cell adhesion	32/647	303/18723	2.14E-08	7.61E-07	5.33E-07	PLXNC1/GPNME	32
GO	BP	GO:1903557	positive regulation of tumor necrosis facto	18/647	107/18723	2.57E-08	9.05E-07	6.34E-07	TLR1/CLEC7A/C	18
GO	BP	GO:0006816	calcium ion transport	39/647	422/18723	2.59E-08	9.05E-07	6.34E-07	P2RX7/PKD2/CD	39
GO	BP	GO:0035987	endodermal cell differentiation	12/647	45/18723	2.65E-08	9.2E-07	6.45E-07	COL5A2/ITGB2/I	12
GO	BP	GO:0001706	endoderm formation	13/647	54/18723	2.67E-08	9.21E-07	6.45E-07	COL5A2/ITGB2/I	13
GO	BP	GO:0018108	peptidyl-tyrosine phosphorylation	36/647	375/18723	3.42E-08	1.17E-06	8.2E-07	NRP1/CLEC7A/P	36
GO	BP	GO:0045619	regulation of lymphocyte differentiation	23/647	174/18723	3.55E-08	1.21E-06	8.46E-07	CD86/SLAMF8/N	23
GO	BP	GO:0050868	negative regulation of T cell activation	19/647	122/18723	3.86E-08	1.3E-06	9.13E-07	GPNMB/CD86/FC	19
GO	BP	GO:1904645	response to amyloid-beta	13/647	56/18723	4.28E-08	1.43E-06	1E-06	VCAM1/FCGR2E	13
GO	BP	GO:0050766	positive regulation of phagocytosis	14/647	66/18723	4.43E-08	1.47E-06	1.03E-06	CLEC7A/RAB31/	14
GO	BP	GO:0031341	regulation of cell killing	17/647	99/18723	4.57E-08	1.5E-06	1.05E-06	P2RX7/ITGAM/C	17
GO	BP	GO:0032602	chemokine production	17/647	99/18723	4.57E-08	1.5E-06	1.05E-06	CLEC7A/SNAI2/T	17
GO	BP	GO:0002699	positive regulation of immune effector prc	27/647	235/18723	4.79E-08	1.56E-06	1.09E-06	ITGAM/CLEC7A/	27
GO	BP	GO:0050678	regulation of epithelial cell proliferation	36/647	381/18723	5.12E-08	1.66E-06	1.16E-06	NRP1/SNAI2/MC	36
GO	BP	GO:0032609	interferon-gamma production	18/647	112/18723	5.34E-08	1.7E-06	1.19E-06	CLEC7A/IL1R1/T	18
GO	BP	GO:0032649	regulation of interferon-gamma productior	18/647	112/18723	5.34E-08	1.7E-06	1.19E-06	CLEC7A/IL1R1/T	18
GO	BP	GO:0051051	negative regulation of transport	41/647	470/18723	5.7E-08	1.8E-06	1.26E-06	PKD2/FCGR2B/T	41
GO	BP	GO:0042102	positive regulation of T cell proliferation	17/647	101/18723	6.21E-08	1.95E-06	1.37E-06	CD86/VCAM1/TN	17
GO	BP	GO:0045582	positive regulation of T cell differentiatior	16/647	91/18723	8.05E-08	2.52E-06	1.76E-06	CD86/NCKAP1L/	16

GO	BP	GO:0002706	regulation of lymphocyte mediated immur	22/647	168/18723	8.34E-08	2.58E-06	1.81E-06	IL1R1/FCGR2B/F	22
GO	BP	GO:0032760	positive regulation of tumor necrosis facto	17/647	103/18723	8.36E-08	2.58E-06	1.81E-06	TLR1/CLEC7A/L	17
GO	BP	GO:1902622	regulation of neutrophil migration	11/647	41/18723	9.47E-08	2.9E-06	2.03E-06	IL1R1/C3AR1/SL	11
GO	BP	GO:0050729	positive regulation of inflammatory respor	20/647	142/18723	9.56E-08	2.91E-06	2.04E-06	OSMR/TLR2/PIK	20
GO	BP	GO:0045621	positive regulation of lymphocyte differen	17/647	104/18723	9.68E-08	2.93E-06	2.05E-06	CD86/NCKAP1L/	17
GO	BP	GO:0046631	alpha-beta T cell activation	21/647	156/18723	1.01E-07	3.05E-06	2.14E-06	CD86/DOCK2/CL	21
GO	BP	GO:0036336	dendritic cell migration	10/647	33/18723	1.02E-07	3.05E-06	2.14E-06	DOCK8/SLAMF8	10
GO	BP	GO:0048245	eosinophil chemotaxis	8/647	19/18723	1.05E-07	3.12E-06	2.18E-06	CCL2/CCL8/CCL	8
GO	BP	GO:0001649	osteoblast differentiation	26/647	229/18723	1.08E-07	3.18E-06	2.23E-06	RASSF2/GPNMB	26
GO	BP	GO:0002407	dendritic cell chemotaxis	9/647	26/18723	1.23E-07	3.59E-06	2.52E-06	SLAMF8/CCR1/C	9
GO	BP	GO:2000401	regulation of lymphocyte migration	13/647	61/18723	1.27E-07	3.69E-06	2.59E-06	DOCK8/CCL2/IT	13
GO	BP	GO:0030336	negative regulation of cell migration	33/647	344/18723	1.3E-07	3.77E-06	2.64E-06	MITF/PHLDB2/M	33
GO	BP	GO:0050672	negative regulation of lymphocyte prolifer	15/647	83/18723	1.4E-07	4.03E-06	2.83E-06	GPNMB/CD86/M	15
GO	BP	GO:0033627	cell adhesion mediated by integrin	14/647	72/18723	1.41E-07	4.04E-06	2.83E-06	SNAI2/FBN1/NCI	14
GO	BP	GO:0050730	regulation of peptidyl-tyrosine phosphoryl	28/647	264/18723	1.49E-07	4.25E-06	2.98E-06	NRP1/CLEC7A/P	28
GO	BP	GO:0001906	cell killing	23/647	188/18723	1.51E-07	4.26E-06	2.98E-06	P2RX7/ITGAM/C	23
GO	BP	GO:0060071	Wnt signaling pathway, planar cell polarit	12/647	52/18723	1.52E-07	4.28E-06	3E-06	GPC6/PRICKLE1	12
GO	BP	GO:0032945	negative regulation of mononuclear cell pr	15/647	84/18723	1.65E-07	4.62E-06	3.23E-06	GPNMB/CD86/M	15
GO	BP	GO:0002704	negative regulation of leukocyte mediated	13/647	63/18723	1.9E-07	5.26E-06	3.69E-06	FCGR2B/CD84/H	13
GO	BP	GO:0009615	response to virus	34/647	367/18723	1.93E-07	5.33E-06	3.73E-06	SLFN11/TLR8/TL	34
GO	BP	GO:0002709	regulation of T cell mediated immunity	15/647	85/18723	1.95E-07	5.33E-06	3.74E-06	IL1R1/FCGR2B/N	15
GO	BP	GO:0032642	regulation of chemokine production	16/647	98/18723	2.34E-07	6.38E-06	4.47E-06	CLEC7A/SNAI2/T	16
GO	BP	GO:0010959	regulation of metal ion transport	36/647	406/18723	2.49E-07	6.76E-06	4.74E-06	P2RX7/PKD2/CD	36
GO	BP	GO:0090026	positive regulation of monocyte chemotax	8/647	21/18723	2.66E-07	7.17E-06	5.02E-06	CCR1/PLA2G7/C	8
GO	BP	GO:0050808	synapse organization	37/647	426/18723	2.84E-07	7.62E-06	5.34E-06	NRP1/ITGAM/GP	37
GO	BP	GO:0001915	negative regulation of T cell mediated cyto	6/647	10/18723	3.1E-07	8.27E-06	5.79E-06	FCGR2B/NCKAP	6
GO	BP	GO:0031348	negative regulation of defense response	27/647	258/18723	3.26E-07	8.64E-06	6.05E-06	FCGR2B/TNFAIP	27
GO	BP	GO:0072678	T cell migration	13/647	66/18723	3.37E-07	8.87E-06	6.21E-06	DOCK8/CCL2/PII	13
GO	BP	GO:2000146	negative regulation of cell motility	33/647	359/18723	3.5E-07	9.16E-06	6.42E-06	MITF/PHLDB2/M	33
GO	BP	GO:0090175	regulation of establishment of planar polai	12/647	56/18723	3.63E-07	9.46E-06	6.63E-06	GPC6/PRICKLE1	12
GO	BP	GO:0002275	myeloid cell activation involved in immun	15/647	91/18723	4.9E-07	1.27E-05	8.89E-06	ITGAM/DOCK2/C	15
GO	BP	GO:0007160	cell-matrix adhesion	25/647	233/18723	5.56E-07	1.43E-05	1E-05	NRP1/VCAM1/FI	25
GO	BP	GO:0051271	negative regulation of cellular component	33/647	367/18723	5.77E-07	1.48E-05	1.04E-05	MITF/PHLDB2/M	33
GO	BP	GO:0072677	eosinophil migration	8/647	23/18723	6.03E-07	1.54E-05	1.08E-05	CCL2/CCL8/CCL	8
GO	BP	GO:0002820	negative regulation of adaptive immune re	12/647	59/18723	6.62E-07	1.67E-05	1.17E-05	FCGR2B/HAVCR	12
GO	BP	GO:0098883	synapse pruning	6/647	11/18723	6.63E-07	1.67E-05	1.17E-05	ITGAM/C1QB/C3	6
GO	BP	GO:0045667	regulation of osteoblast differentiation	18/647	132/18723	6.79E-07	1.7E-05	1.19E-05	RASSF2/SNAI2/F	18
GO	BP	GO:0045580	regulation of T cell differentiation	19/647	146/18723	6.97E-07	1.74E-05	1.22E-05	CD86/NCKAP1L/	19

GO	BP	GO:0071887	leukocyte apoptotic process	16/647	106/18723	7.05E-07	1.75E-05	1.23E-05	DOCK8/HCLS1/C	16
GO	BP	GO:0034612	response to tumor necrosis factor	26/647	253/18723	7.64E-07	1.88E-05	1.32E-05	VCAM1/TNFSF1:	26
GO	BP	GO:0016055	Wnt signaling pathway	37/647	444/18723	7.83E-07	1.92E-05	1.35E-05	ZEB2/PKD2/SNA	37
GO	BP	GO:0007229	integrin-mediated signaling pathway	16/647	107/18723	8.03E-07	1.96E-05	1.37E-05	NRP1/ITGAM/FE	16
GO	BP	GO:0040013	negative regulation of locomotion	34/647	391/18723	8.43E-07	2.05E-05	1.43E-05	NRP1/MITF/PHLI	34
GO	BP	GO:0198738	cell-cell signaling by wnt	37/647	446/18723	8.73E-07	2.11E-05	1.48E-05	ZEB2/PKD2/SNA	37
GO	BP	GO:0002573	myeloid leukocyte differentiation	23/647	208/18723	9.28E-07	2.23E-05	1.56E-05	MAFB/RASSF2/M	23
GO	BP	GO:0048015	phosphatidylinositol-mediated signaling	21/647	178/18723	9.54E-07	2.28E-05	1.6E-05	PDGFC/FYN/HCI	21
GO	BP	GO:0032729	positive regulation of interferon-gamma pı	13/647	72/18723	9.65E-07	2.29E-05	1.6E-05	CLEC7A/IL1R1/T	13
GO	BP	GO:0035567	non-canonical Wnt signaling pathway	13/647	72/18723	9.65E-07	2.29E-05	1.6E-05	GPC6/PRICKLE1.	13
GO	BP	GO:0098742	cell-cell adhesion via plasma-membrane ad	27/647	273/18723	9.99E-07	2.35E-05	1.65E-05	ITGAM/GPC6/VC	27
GO	BP	GO:0045637	regulation of myeloid cell differentiation	23/647	210/18723	1.1E-06	2.57E-05	1.8E-05	MAFB/RASSF2/M	23
GO	BP	GO:0032613	interleukin-10 production	12/647	62/18723	1.16E-06	2.67E-05	1.87E-05	CLEC7A/FCGR2I	12
GO	BP	GO:0032623	interleukin-2 production	12/647	62/18723	1.16E-06	2.67E-05	1.87E-05	CLEC7A/CD86/H	12
GO	BP	GO:0032653	regulation of interleukin-10 production	12/647	62/18723	1.16E-06	2.67E-05	1.87E-05	CLEC7A/FCGR2I	12
GO	BP	GO:0032663	regulation of interleukin-2 production	12/647	62/18723	1.16E-06	2.67E-05	1.87E-05	CLEC7A/CD86/H	12
GO	BP	GO:0030100	regulation of endocytosis	23/647	211/18723	1.19E-06	2.73E-05	1.91E-05	RAB31/NCKAP1I	23
GO	BP	GO:0022408	negative regulation of cell-cell adhesion	22/647	196/18723	1.24E-06	2.82E-05	1.98E-05	GPNMB/CD86/FC	22
GO	BP	GO:1901623	regulation of lymphocyte chemotaxis	8/647	25/18723	1.25E-06	2.83E-05	1.99E-05	CCL2/CCR2/CCL	8
GO	BP	GO:0043277	apoptotic cell clearance	11/647	52/18723	1.28E-06	2.89E-05	2.02E-05	CCL2/CD300LF/T	11
GO	BP	GO:0002367	cytokine production involved in immune r	15/647	98/18723	1.3E-06	2.93E-05	2.05E-05	CLEC7A/IL1R1/L	15
GO	BP	GO:0030111	regulation of Wnt signaling pathway	30/647	328/18723	1.32E-06	2.95E-05	2.07E-05	ZEB2/SNAI2/CDI	30
GO	BP	GO:0048017	inositol lipid-mediated signaling	21/647	182/18723	1.38E-06	3.06E-05	2.14E-05	PDGFC/FYN/HCI	21
GO	BP	GO:2000027	regulation of animal organ morphogenesis	17/647	125/18723	1.43E-06	3.17E-05	2.22E-05	GPC6/SULF1/PRI	17
GO	BP	GO:0050864	regulation of B cell activation	22/647	198/18723	1.47E-06	3.24E-05	2.27E-05	MNDA/TNFSF13I	22
GO	BP	GO:0002444	myeloid leukocyte mediated immunity	15/647	99/18723	1.49E-06	3.26E-05	2.29E-05	ITGAM/FCGR2B/	15
GO	BP	GO:0001667	ameboidal-type cell migration	38/647	475/18723	1.51E-06	3.28E-05	2.3E-05	ZEB2/NRP1/FAP/	38
GO	BP	GO:0050869	negative regulation of B cell activation	9/647	34/18723	1.61E-06	3.49E-05	2.44E-05	MNDA/FCGR2B/	9
GO	BP	GO:0045807	positive regulation of endocytosis	15/647	100/18723	1.7E-06	3.67E-05	2.57E-05	RAB31/NCKAP1I	15
GO	BP	GO:0002710	negative regulation of T cell mediated imr	8/647	26/18723	1.75E-06	3.77E-05	2.64E-05	FCGR2B/NCKAP	8
GO	BP	GO:0030178	negative regulation of Wnt signaling pathv	20/647	170/18723	1.8E-06	3.85E-05	2.7E-05	SNAI2/MCC/PRI(	20
GO	BP	GO:0034341	response to interferon-gamma	18/647	141/18723	1.81E-06	3.85E-05	2.7E-05	MRC1/TLR2/VIM	18
GO	BP	GO:0002823	negative regulation of adaptive immune re	11/647	54/18723	1.91E-06	4.05E-05	2.83E-05	FCGR2B/HAVCR	11
GO	BP	GO:0042113	B cell activation	30/647	334/18723	1.92E-06	4.06E-05	2.84E-05	DOCK10/CD86/V	30
GO	BP	GO:0031295	T cell costimulation	10/647	44/18723	1.94E-06	4.08E-05	2.86E-05	CD86/TNFSF13B.	10
GO	BP	GO:0032612	interleukin-1 production	17/647	128/18723	2.01E-06	4.18E-05	2.93E-05	P2RX7/CLEC7A/I	17
GO	BP	GO:0032652	regulation of interleukin-1 production	17/647	128/18723	2.01E-06	4.18E-05	2.93E-05	P2RX7/CLEC7A/I	17
GO	BP	GO:0007492	endoderm development	13/647	77/18723	2.13E-06	4.43E-05	3.1E-05	COL5A2/ITGB2/I	13

GO	BP	GO:0002430	complement receptor mediated signaling p	6/647	13/18723	2.32E-06	4.79E-05	3.36E-05	C3AR1/FPR3/C5A	6
GO	BP	GO:1904646	cellular response to amyloid-beta	10/647	45/18723	2.42E-06	4.98E-05	3.49E-05	VCAM1/FCGR2B	10
GO	BP	GO:0001818	negative regulation of cytokine production	31/647	357/18723	2.64E-06	5.41E-05	3.79E-05	GPNMB/TLR8/FC	31
GO	BP	GO:0045089	positive regulation of innate immune respo	17/647	131/18723	2.77E-06	5.66E-05	3.96E-05	CLEC7A/MNDA/	17
GO	BP	GO:0048771	tissue remodeling	20/647	175/18723	2.84E-06	5.76E-05	4.03E-05	P2RX7/RASSF2/C	20
GO	BP	GO:0007599	hemostasis	23/647	222/18723	2.87E-06	5.79E-05	4.06E-05	ENTPD1/FAP/FL	23
GO	BP	GO:0014068	positive regulation of phosphatidylinositol	13/647	79/18723	2.88E-06	5.79E-05	4.06E-05	PDGFC/FYN/HCI	13
GO	BP	GO:0031294	lymphocyte costimulation	10/647	46/18723	3E-06	6.01E-05	4.21E-05	CD86/TNFSF13B/	10
GO	BP	GO:0071346	cellular response to interferon-gamma	16/647	118/18723	3.03E-06	6.05E-05	4.24E-05	MRC1/TLR2/VIM	16
GO	BP	GO:0050679	positive regulation of epithelial cell prolife	22/647	207/18723	3.09E-06	6.14E-05	4.3E-05	NRP1/VEGFC/C5	22
GO	BP	GO:0010575	positive regulation of vascular endothelial	8/647	28/18723	3.28E-06	6.41E-05	4.49E-05	C3AR1/SULF1/CY	8
GO	BP	GO:0010818	T cell chemotaxis	8/647	28/18723	3.28E-06	6.41E-05	4.49E-05	PIK3CG/GPR183/	8
GO	BP	GO:0031342	negative regulation of cell killing	8/647	28/18723	3.28E-06	6.41E-05	4.49E-05	FCGR2B/HAVCR	8
GO	BP	GO:1902624	positive regulation of neutrophil migration	8/647	28/18723	3.28E-06	6.41E-05	4.49E-05	IL1R1/C3AR1/NC	8
GO	BP	GO:0051346	negative regulation of hydrolase activity	32/647	379/18723	3.31E-06	6.44E-05	4.51E-05	TIMP2/NCKAP1L	32
GO	BP	GO:0045730	respiratory burst	9/647	37/18723	3.47E-06	6.73E-05	4.71E-05	CLEC7A/NCF2/S	9
GO	BP	GO:0001935	endothelial cell proliferation	21/647	193/18723	3.55E-06	6.85E-05	4.8E-05	NRP1/PTPRM/SU	21
GO	BP	GO:0002062	chondrocyte differentiation	15/647	106/18723	3.59E-06	6.89E-05	4.83E-05	CHST11/SNAI2/T	15
GO	BP	GO:0051235	maintenance of location	29/647	327/18723	3.66E-06	7E-05	4.91E-05	CCDC88A/PKD2/	29
GO	BP	GO:0031589	cell-substrate adhesion	31/647	363/18723	3.73E-06	7.1E-05	4.98E-05	NRP1/VCAM1/FE	31
GO	BP	GO:0002761	regulation of myeloid leukocyte differentia	16/647	120/18723	3.79E-06	7.2E-05	5.04E-05	MAFB/RASSF2/M	16
GO	BP	GO:0001936	regulation of endothelial cell proliferation	20/647	179/18723	4.02E-06	7.61E-05	5.33E-05	NRP1/PTPRM/SU	20
GO	BP	GO:0006936	muscle contraction	30/647	347/18723	4.17E-06	7.86E-05	5.51E-05	DOCK4/SULF1/C	30
GO	BP	GO:0150146	cell junction disassembly	7/647	21/18723	4.34E-06	8.14E-05	5.7E-05	ITGAM/SNAI2/C	7
GO	BP	GO:2000406	positive regulation of T cell migration	8/647	29/18723	4.39E-06	8.2E-05	5.74E-05	DOCK8/ITGA4/C	8
GO	BP	GO:0097242	amyloid-beta clearance	9/647	38/18723	4.41E-06	8.2E-05	5.74E-05	ITGAM/MSR1/C5	9
GO	BP	GO:0001910	regulation of leukocyte mediated cytotoxic	13/647	82/18723	4.42E-06	8.2E-05	5.74E-05	ITGAM/FCGR2B/	13
GO	BP	GO:0002456	T cell mediated immunity	15/647	109/18723	5.11E-06	9.42E-05	6.6E-05	IL1R1/FCGR2B/N	15
GO	BP	GO:0090090	negative regulation of canonical Wnt signa	17/647	137/18723	5.15E-06	9.47E-05	6.64E-05	SNAI2/MCC/PRIC	17
GO	BP	GO:0002718	regulation of cytokine production involved	14/647	96/18723	5.27E-06	9.65E-05	6.76E-05	CLEC7A/IL1R1/L	14
GO	BP	GO:0051651	maintenance of location in cell	22/647	214/18723	5.33E-06	9.72E-05	6.81E-05	CCDC88A/PKD2/	22
GO	BP	GO:0032611	interleukin-1 beta production	15/647	110/18723	5.73E-06	0.000104	7.26E-05	P2RX7/CLEC7A/I	15
GO	BP	GO:0032651	regulation of interleukin-1 beta production	15/647	110/18723	5.73E-06	0.000104	7.26E-05	P2RX7/CLEC7A/I	15
GO	BP	GO:0002833	positive regulation of response to biotic st	19/647	168/18723	5.84E-06	0.000105	7.38E-05	CLEC7A/MNDA/	19
GO	BP	GO:0001736	establishment of planar polarity	12/647	72/18723	6.03E-06	0.000108	7.56E-05	GPC6/PRICKLE1/	12
GO	BP	GO:0007164	establishment of tissue polarity	12/647	72/18723	6.03E-06	0.000108	7.56E-05	GPC6/PRICKLE1/	12
GO	BP	GO:0042430	indole-containing compound metabolic pro	7/647	22/18723	6.17E-06	0.00011	7.71E-05	RNF180/KMO/KY	7
GO	BP	GO:0002468	dendritic cell antigen processing and prese	6/647	15/18723	6.38E-06	0.000113	7.94E-05	FCGR2B/CLEC4A	6

GO	BP	GO:0014066	regulation of phosphatidylinositol 3-kinas	15/647	111/18723	6.41E-06	0.000113	7.95E-05	PDGFC/FYN/HCI	15
GO	BP	GO:0008360	regulation of cell shape	18/647	154/18723	6.44E-06	0.000113	7.95E-05	PLXNC1/FERMT	18
GO	BP	GO:0033674	positive regulation of kinase activity	36/647	467/18723	6.56E-06	0.000115	8.07E-05	CCDC88A/PKD2/	36
GO	BP	GO:0007596	blood coagulation	22/647	217/18723	6.67E-06	0.000117	8.18E-05	ENTPD1/FAP/FC	22
GO	BP	GO:0032732	positive regulation of interleukin-1 produc	12/647	73/18723	6.99E-06	0.000121	8.51E-05	P2RX7/CLEC7A/	12
GO	BP	GO:0043299	leukocyte degranulation	12/647	73/18723	6.99E-06	0.000121	8.51E-05	ITGAM/FCGR2B/	12
GO	BP	GO:0046632	alpha-beta T cell differentiation	15/647	112/18723	7.17E-06	0.000124	8.69E-05	CD86/NCKAP1L/	15
GO	BP	GO:0032731	positive regulation of interleukin-1 beta pr	11/647	62/18723	7.88E-06	0.000136	9.52E-05	P2RX7/CLEC7A/	11
GO	BP	GO:0071622	regulation of granulocyte chemotaxis	10/647	51/18723	8.04E-06	0.000138	9.68E-05	C3AR1/NCKAP1	10
GO	BP	GO:0002286	T cell activation involved in immune resp	15/647	114/18723	8.94E-06	0.000153	0.000107	CD86/FCGR2B/H	15
GO	BP	GO:0060348	bone development	21/647	205/18723	9.17E-06	0.000156	0.00011	FBN1/FLI1/LOX/	21
GO	BP	GO:0044403	biological process involved in symbiotic i	26/647	290/18723	9.53E-06	0.000162	0.000113	NRP1/CD86/MRC	26
GO	BP	GO:0050817	coagulation	22/647	222/18723	9.62E-06	0.000163	0.000114	ENTPD1/FAP/FC	22
GO	BP	GO:0090022	regulation of neutrophil chemotaxis	8/647	32/18723	9.82E-06	0.000166	0.000116	C3AR1/NCKAP1	8
GO	BP	GO:0030099	myeloid cell differentiation	31/647	381/18723	9.93E-06	0.000167	0.000117	MAFB/RASSF2/M	31
GO	BP	GO:0014065	phosphatidylinositol 3-kinase signaling	17/647	144/18723	1.01E-05	0.000169	0.000119	PDGFC/FYN/HCI	17
GO	BP	GO:0035747	natural killer cell chemotaxis	5/647	10/18723	1.06E-05	0.000177	0.000124	CCL2/PIK3CG/C	5
GO	BP	GO:0016064	immunoglobulin mediated immune respon	21/647	207/18723	1.07E-05	0.000177	0.000124	TLR8/FCGR2B/P	21
GO	BP	GO:2000404	regulation of T cell migration	9/647	42/18723	1.07E-05	0.000177	0.000124	DOCK8/ITGA4/C	9
GO	BP	GO:1902106	negative regulation of leukocyte differenti	14/647	102/18723	1.08E-05	0.000178	0.000125	MAFB/FBN1/FC	14
GO	BP	GO:0030888	regulation of B cell proliferation	11/647	64/18723	1.08E-05	0.000178	0.000125	MNDA/TNFSF13	11
GO	BP	GO:0043271	negative regulation of ion transport	18/647	160/18723	1.1E-05	0.00018	0.000126	PKD2/KCNE4/CL	18
GO	BP	GO:0060402	calcium ion transport into cytosol	18/647	160/18723	1.1E-05	0.00018	0.000126	P2RX7/PKD2/CA	18
GO	BP	GO:0002707	negative regulation of lymphocyte mediat	10/647	53/18723	1.15E-05	0.000188	0.000132	FCGR2B/HAVCR	10
GO	BP	GO:0001911	negative regulation of leukocyte mediated	7/647	24/18723	1.18E-05	0.000191	0.000134	FCGR2B/HAVCR	7
GO	BP	GO:0090023	positive regulation of neutrophil chemota	7/647	24/18723	1.18E-05	0.000191	0.000134	C3AR1/NCKAP1	7
GO	BP	GO:0052547	regulation of peptidase activity	35/647	461/18723	1.22E-05	0.000197	0.000138	CLEC7A/TIMP2/	35
GO	BP	GO:0043405	regulation of MAP kinase activity	19/647	177/18723	1.25E-05	0.0002	0.00014	PDGFC/TRIB2/PI	19
GO	BP	GO:0050731	positive regulation of peptidyl-tyrosine ph	20/647	193/18723	1.26E-05	0.0002	0.00014	NRP1/CLEC7A/N	20
GO	BP	GO:0050901	leukocyte tethering or rolling	8/647	33/18723	1.26E-05	0.0002	0.00014	VCAM1/ITGA4/J	8
GO	BP	GO:0014002	astrocyte development	9/647	43/18723	1.31E-05	0.000207	0.000145	VIM/C5AR1/ROR	9
GO	BP	GO:0036230	granulocyte activation	9/647	43/18723	1.31E-05	0.000207	0.000145	ITGAM/FCGR2B/	9
GO	BP	GO:0019724	B cell mediated immunity	21/647	210/18723	1.33E-05	0.00021	0.000147	TLR8/FCGR2B/P	21
GO	BP	GO:0032963	collagen metabolic process	14/647	104/18723	1.36E-05	0.000214	0.00015	FAP/VIM/CTSL/	14
GO	BP	GO:0060191	regulation of lipase activity	13/647	91/18723	1.43E-05	0.000224	0.000157	CD86/C5AR1/RG	13
GO	BP	GO:0010466	negative regulation of peptidase activity	24/647	262/18723	1.46E-05	0.000228	0.00016	TIMP2/SPOCK1/	24
GO	BP	GO:0050918	positive chemotaxis	11/647	66/18723	1.47E-05	0.000229	0.00016	NRP1/GPNMB/V	11
GO	BP	GO:0002700	regulation of production of molecular med	18/647	164/18723	1.55E-05	0.00024	0.000168	CLEC7A/IL1R1/C	18

GO	BP	GO:0071356	cellular response to tumor necrosis factor	22/647	229/18723	1.57E-05	0.000243	0.00017	VCAM1/TNFSF13	22
GO	BP	GO:0002705	positive regulation of leukocyte mediated	16/647	134/18723	1.59E-05	0.000243	0.00017	ITGAM/CLEC7A/	16
GO	BP	GO:0061041	regulation of wound healing	16/647	134/18723	1.59E-05	0.000243	0.00017	CLEC7A/FERMT	16
GO	BP	GO:0045834	positive regulation of lipid metabolic proc	17/647	149/18723	1.59E-05	0.000243	0.00017	FGR/AVPR1A/TW	17
GO	BP	GO:0042554	superoxide anion generation	9/647	44/18723	1.59E-05	0.000243	0.00017	ITGAM/CLEC7A/	9
GO	BP	GO:0002495	antigen processing and presentation of pep	8/647	34/18723	1.59E-05	0.000243	0.00017	FCGR2B/FCER1G	8
GO	BP	GO:0006029	proteoglycan metabolic process	12/647	79/18723	1.61E-05	0.000245	0.000171	DSE/CHST11/SUL	12
GO	BP	GO:1905517	macrophage migration	10/647	55/18723	1.63E-05	0.000246	0.000172	C3AR1/SLAMF8/	10
GO	BP	GO:1903707	negative regulation of hemopoiesis	14/647	106/18723	1.69E-05	0.000255	0.000179	MAFB/FBN1/FCG	14
GO	BP	GO:0010517	regulation of phospholipase activity	11/647	67/18723	1.7E-05	0.000255	0.000179	CD86/C5AR1/RG	11
GO	BP	GO:0042130	negative regulation of T cell proliferation	11/647	67/18723	1.7E-05	0.000255	0.000179	GPNMB/CD86/HA	11
GO	BP	GO:0045766	positive regulation of angiogenesis	19/647	181/18723	1.71E-05	0.000255	0.000179	NRP1/C3AR1/AD	19
GO	BP	GO:1904018	positive regulation of vasculature develop	19/647	181/18723	1.71E-05	0.000255	0.000179	NRP1/C3AR1/AD	19
GO	BP	GO:0001704	formation of primary germ layer	15/647	121/18723	1.85E-05	0.000273	0.000192	COL5A2/GJA1/IT	15
GO	BP	GO:0002224	toll-like receptor signaling pathway	15/647	121/18723	1.85E-05	0.000273	0.000192	TLR1/COLEC12/I	15
GO	BP	GO:0060401	cytosolic calcium ion transport	19/647	182/18723	1.85E-05	0.000273	0.000192	P2RX7/PKD2/CA	19
GO	BP	GO:0006568	tryptophan metabolic process	5/647	11/18723	1.89E-05	0.000277	0.000194	KMO/KYNU/IL4I	5
GO	BP	GO:0044409	entry into host	17/647	151/18723	1.9E-05	0.000279	0.000195	NRP1/CD86/MRC	17
GO	BP	GO:0002886	regulation of myeloid leukocyte mediated	10/647	56/18723	1.92E-05	0.00028	0.000196	ITGAM/FCGR2B/	10
GO	BP	GO:0002920	regulation of humoral immune response	9/647	45/18723	1.93E-05	0.000281	0.000197	CFH/FCGR2B/PT	9
GO	BP	GO:0010631	epithelial cell migration	29/647	357/18723	1.97E-05	0.000285	0.0002	ZEB2/NRP1/FAP/	29
GO	BP	GO:1904062	regulation of cation transmembrane transp	29/647	357/18723	1.97E-05	0.000285	0.0002	PKD2/CACNA2D	29
GO	BP	GO:0050654	chondroitin sulfate proteoglycan metaboli	8/647	35/18723	2E-05	0.000289	0.000203	DSE/CHST11/CH	8
GO	BP	GO:0030316	osteoclast differentiation	13/647	94/18723	2.04E-05	0.000294	0.000206	MAFB/RASSF2/M	13
GO	BP	GO:2000106	regulation of leukocyte apoptotic process	12/647	81/18723	2.09E-05	0.0003	0.00021	DOCK8/HCLS1/M	12
GO	BP	GO:2000050	regulation of non-canonical Wnt signaling	7/647	26/18723	2.11E-05	0.000302	0.000211	SFRP2/RSPO3/DA	7
GO	BP	GO:0061756	leukocyte adhesion to vascular endothelial	10/647	57/18723	2.26E-05	0.000321	0.000225	VCAM1/ITGB2/IT	10
GO	BP	GO:0045669	positive regulation of osteoblast differenti	11/647	69/18723	2.27E-05	0.000323	0.000226	FERMT2/DDR2/W	11
GO	BP	GO:0090132	epithelium migration	29/647	360/18723	2.3E-05	0.000326	0.000228	ZEB2/NRP1/FAP/	29
GO	BP	GO:0010951	negative regulation of endopeptidase activ	23/647	252/18723	2.32E-05	0.000328	0.00023	TIMP2/SPOCK1/C	23
GO	BP	GO:0050878	regulation of body fluid levels	30/647	379/18723	2.33E-05	0.000328	0.00023	ENTPD1/FAP/HE	30
GO	BP	GO:0007369	gastrulation	19/647	185/18723	2.33E-05	0.000328	0.00023	PHLDB2/COL5A2	19
GO	BP	GO:0002504	antigen processing and presentation of pep	8/647	36/18723	2.5E-05	0.000349	0.000244	FCGR2B/FCER1G	8
GO	BP	GO:0042119	neutrophil activation	8/647	36/18723	2.5E-05	0.000349	0.000244	ITGAM/FCGR2B/	8
GO	BP	GO:0014812	muscle cell migration	14/647	110/18723	2.59E-05	0.000361	0.000253	NRP1/DOCK4/NO	14
GO	BP	GO:0045123	cellular extravasation	11/647	70/18723	2.61E-05	0.000362	0.000254	IL1R1/VCAM1/C	11
GO	BP	GO:0050804	modulation of chemical synaptic transmiss	33/647	439/18723	2.64E-05	0.000365	0.000255	CDH11/TSHZ3/SI	33
GO	BP	GO:0099177	regulation of trans-synaptic signaling	33/647	440/18723	2.76E-05	0.000377	0.000264	CDH11/TSHZ3/SI	33

GO	BP	GO:0002825	regulation of T-helper 1 type immune resp	7/647	27/18723	2.77E-05	0.000377	0.000264	IL1R1/HAVCR2/T	7
GO	BP	GO:0032703	negative regulation of interleukin-2 produ	7/647	27/18723	2.77E-05	0.000377	0.000264	HAVCR2/PTPRC/	7
GO	BP	GO:0036037	CD8-positive, alpha-beta T cell activation	7/647	27/18723	2.77E-05	0.000377	0.000264	CLEC4A/NCKAP	7
GO	BP	GO:0071624	positive regulation of granulocyte chemota	7/647	27/18723	2.77E-05	0.000377	0.000264	C3AR1/NCKAP1I	7
GO	BP	GO:0090288	negative regulation of cellular response to	14/647	111/18723	2.87E-05	0.000391	0.000274	FBN1/SULF1/DC	14
GO	BP	GO:0002221	pattern recognition receptor signaling path	18/647	172/18723	2.96E-05	0.000401	0.000281	TLR1/COLEC12/I	18
GO	BP	GO:0090130	tissue migration	29/647	365/18723	2.96E-05	0.000401	0.000281	ZEB2/NRP1/FAP/	29
GO	BP	GO:0032722	positive regulation of chemokine producti	11/647	71/18723	2.99E-05	0.000404	0.000283	CLEC7A/TLR2/C	11
GO	BP	GO:0030183	B cell differentiation	16/647	141/18723	3.01E-05	0.000405	0.000283	DOCK10/VCAM1	16
GO	BP	GO:0140131	positive regulation of lymphocyte chemota	6/647	19/18723	3.07E-05	0.000411	0.000288	CCR2/CCL4/CCL	6
GO	BP	GO:0001990	regulation of systemic arterial blood press	8/647	37/18723	3.09E-05	0.000413	0.00029	SUCNR1/AVPR1.	8
GO	BP	GO:0016049	cell growth	35/647	482/18723	3.11E-05	0.000415	0.000291	NRP1/SPOCK1/C	35
GO	BP	GO:0002526	acute inflammatory response	14/647	112/18723	3.18E-05	0.000423	0.000296	CD163/VCAM1/C	14
GO	BP	GO:0045860	positive regulation of protein kinase activi	30/647	386/18723	3.28E-05	0.000434	0.000304	CCDC88A/PKD2/	30
GO	BP	GO:0097553	calcium ion transmembrane import into cy	16/647	142/18723	3.29E-05	0.000434	0.000304	PKD2/CACNA2D	16
GO	BP	GO:0033628	regulation of cell adhesion mediated by in	9/647	48/18723	3.33E-05	0.000439	0.000308	SNAI2/NCKAP1L	9
GO	BP	GO:0031623	receptor internalization	14/647	113/18723	3.52E-05	0.000462	0.000324	RAB31/FCER1G/	14
GO	BP	GO:0002675	positive regulation of acute inflammatory	7/647	28/18723	3.58E-05	0.000467	0.000327	OSMR/PIK3CG/A	7
GO	BP	GO:0038094	Fc-gamma receptor signaling pathway	7/647	28/18723	3.58E-05	0.000467	0.000327	FCGR2B/FYN/PT	7
GO	BP	GO:0042100	B cell proliferation	13/647	99/18723	3.58E-05	0.000467	0.000327	MNDA/TNFSF13	13
GO	BP	GO:0002478	antigen processing and presentation of exo	8/647	38/18723	3.8E-05	0.000492	0.000345	FCGR2B/CLEC4/	8
GO	BP	GO:0009595	detection of biotic stimulus	8/647	38/18723	3.8E-05	0.000492	0.000345	TLR1/CLEC7A/F.	8
GO	BP	GO:0035924	cellular response to vascular endothelial g	11/647	73/18723	3.91E-05	0.000505	0.000354	NRP1/VCAM1/G/	11
GO	BP	GO:0043542	endothelial cell migration	24/647	279/18723	4.08E-05	0.000526	0.000368	NRP1/FAP/PTPRI	24
GO	BP	GO:0043030	regulation of macrophage activation	10/647	61/18723	4.16E-05	0.000534	0.000374	FCGR2B/CD84/H	10
GO	BP	GO:0002577	regulation of antigen processing and prese	6/647	20/18723	4.26E-05	0.000546	0.000382	FCGR2B/LILRB2	6
GO	BP	GO:0051209	release of sequestered calcium ion into cyt	14/647	115/18723	4.29E-05	0.000548	0.000384	PKD2/CLIC2/PTF	14
GO	BP	GO:0006956	complement activation	15/647	130/18723	4.36E-05	0.000555	0.000389	CFH/CR1/SERPIN	15
GO	BP	GO:0006801	superoxide metabolic process	11/647	74/18723	4.45E-05	0.000565	0.000396	ITGAM/CLEC7A/	11
GO	BP	GO:0019058	viral life cycle	26/647	317/18723	4.45E-05	0.000565	0.000396	NRP1/CD86/MRC	26
GO	BP	GO:0002285	lymphocyte activation involved in immune	19/647	194/18723	4.52E-05	0.000571	0.0004	DOCK10/CD86/F	19
GO	BP	GO:0038093	Fc receptor signaling pathway	9/647	50/18723	4.68E-05	0.00059	0.000413	FCGR2B/FCGR1I	9
GO	BP	GO:0003012	muscle system process	33/647	452/18723	4.69E-05	0.00059	0.000413	DOCK4/SULF1/C	33
GO	BP	GO:0060759	regulation of response to cytokine stimulu	17/647	162/18723	4.72E-05	0.000591	0.000414	IL1R1/TLR2/PTP	17
GO	BP	GO:0051283	negative regulation of sequestering of calc	14/647	116/18723	4.72E-05	0.000591	0.000414	PKD2/CLIC2/PTF	14
GO	BP	GO:0032615	interleukin-12 production	10/647	62/18723	4.81E-05	0.000595	0.000417	CLEC7A/TLR8/T	10
GO	BP	GO:0032655	regulation of interleukin-12 production	10/647	62/18723	4.81E-05	0.000595	0.000417	CLEC7A/TLR8/T	10
GO	BP	GO:0032757	positive regulation of interleukin-8 produc	10/647	62/18723	4.81E-05	0.000595	0.000417	TLR1/CLEC7A/T	10

GO	BP	GO:0045576	mast cell activation	10/647	62/18723	4.81E-05	0.000595	0.000417	LCP2/CD84/PIK3	10
GO	BP	GO:0001768	establishment of T cell polarity	5/647	13/18723	4.96E-05	0.000607	0.000425	DOCK8/DOCK2/(	5
GO	BP	GO:0006586	indolalkylamine metabolic process	5/647	13/18723	4.96E-05	0.000607	0.000425	KMO/KYNU/IL4I	5
GO	BP	GO:0060100	positive regulation of phagocytosis, engulf	5/647	13/18723	4.96E-05	0.000607	0.000425	RAB31/NCKAP1I	5
GO	BP	GO:1905155	positive regulation of membrane invaginat	5/647	13/18723	4.96E-05	0.000607	0.000425	RAB31/NCKAP1I	5
GO	BP	GO:0042063	gliogenesis	25/647	301/18723	5.06E-05	0.000618	0.000433	TLR2/VIM/GLI3/(	25
GO	BP	GO:0051607	defense response to virus	23/647	265/18723	5.11E-05	0.000621	0.000435	SLFN11/TLR8/TL	23
GO	BP	GO:0140546	defense response to symbiont	23/647	265/18723	5.11E-05	0.000621	0.000435	SLFN11/TLR8/TL	23
GO	BP	GO:0043393	regulation of protein binding	19/647	196/18723	5.2E-05	0.00063	0.000441	NRP1/LOX/DZIP1	19
GO	BP	GO:0048660	regulation of smooth muscle cell prolifera	18/647	180/18723	5.41E-05	0.000654	0.000458	GJA1/CALCRL/M	18
GO	BP	GO:0150077	regulation of neuroinflammatory response	8/647	40/18723	5.62E-05	0.000678	0.000475	PTPRC/LRRK2/S	8
GO	BP	GO:0051282	regulation of sequestering of calcium ion	14/647	118/18723	5.72E-05	0.000687	0.000481	PKD2/CLIC2/PTP	14
GO	BP	GO:0030449	regulation of complement activation	6/647	21/18723	5.79E-05	0.000689	0.000483	CFH/CR1/SERPIN	6
GO	BP	GO:0019886	antigen processing and presentation of exo	7/647	30/18723	5.79E-05	0.000689	0.000483	FCGR2B/FCER1C	7
GO	BP	GO:0030204	chondroitin sulfate metabolic process	7/647	30/18723	5.79E-05	0.000689	0.000483	DSE/CHST11/CH	7
GO	BP	GO:0045577	regulation of B cell differentiation	7/647	30/18723	5.79E-05	0.000689	0.000483	SLAMF8/NCKAP	7
GO	BP	GO:0090287	regulation of cellular response to growth f	25/647	304/18723	5.95E-05	0.000703	0.000493	ZEB2/CHST11/FE	25
GO	BP	GO:0032635	interleukin-6 production	17/647	165/18723	5.96E-05	0.000703	0.000493	TLR1/CLEC7A/T	17
GO	BP	GO:0032675	regulation of interleukin-6 production	17/647	165/18723	5.96E-05	0.000703	0.000493	TLR1/CLEC7A/T	17
GO	BP	GO:0046849	bone remodeling	12/647	90/18723	6.11E-05	0.000719	0.000504	P2RX7/RASSF2/N	12
GO	BP	GO:0045670	regulation of osteoclast differentiation	10/647	64/18723	6.37E-05	0.000748	0.000524	MAFB/RASSF2/M	10
GO	BP	GO:0001959	regulation of cytokine-mediated signaling	16/647	150/18723	6.43E-05	0.000752	0.000527	IL1R1/PTPRC/CX	16
GO	BP	GO:0043112	receptor metabolic process	17/647	166/18723	6.43E-05	0.000752	0.000527	RAB31/FCER1G/	17
GO	BP	GO:0002218	activation of innate immune response	9/647	52/18723	6.47E-05	0.000754	0.000529	CLEC7A/MNDA/	9
GO	BP	GO:0051090	regulation of DNA-binding transcription f	32/647	440/18723	6.56E-05	0.000763	0.000534	CLEC7A/PKD2/T	32
GO	BP	GO:1903034	regulation of response to wounding	17/647	167/18723	6.93E-05	0.000804	0.000564	CLEC7A/FERMT	17
GO	BP	GO:0060828	regulation of canonical Wnt signaling path	22/647	253/18723	7.19E-05	0.000833	0.000584	ZEB2/SNAI2/CDI	22
GO	BP	GO:0048659	smooth muscle cell proliferation	18/647	184/18723	7.22E-05	0.000833	0.000584	GJA1/CALCRL/M	18
GO	BP	GO:0019882	antigen processing and presentation	13/647	106/18723	7.39E-05	0.000852	0.000597	FCGR2B/CLEC4A	13
GO	BP	GO:0001767	establishment of lymphocyte polarity	5/647	14/18723	7.49E-05	0.000859	0.000602	DOCK8/DOCK2/(	5
GO	BP	GO:0001771	immunological synapse formation	5/647	14/18723	7.49E-05	0.000859	0.000602	DOCK8/DOCK2/I	5
GO	BP	GO:0032693	negative regulation of interleukin-10 produ	6/647	22/18723	7.73E-05	0.000884	0.000619	FCGR2B/TRIB2/T	6
GO	BP	GO:0030574	collagen catabolic process	8/647	42/18723	8.12E-05	0.000924	0.000648	FAP/CTSL/ADAM	8
GO	BP	GO:0032689	negative regulation of interferon-gamma p	8/647	42/18723	8.12E-05	0.000924	0.000648	HAVCR2/LAPTM	8
GO	BP	GO:0002824	positive regulation of adaptive immune res	13/647	107/18723	8.16E-05	0.000926	0.000649	CLEC7A/IL1R1/T	13
GO	BP	GO:0061844	antimicrobial humoral immune response m	11/647	79/18723	8.23E-05	0.000933	0.000653	CXCL13/CXCL9/	11
GO	BP	GO:0051208	sequestering of calcium ion	14/647	122/18723	8.25E-05	0.000933	0.000653	PKD2/CLIC2/PTP	14
GO	BP	GO:0033273	response to vitamin	12/647	93/18723	8.45E-05	0.000953	0.000667	SNAI2/POSTN/M	12

GO	BP	GO:0071559	response to transforming growth factor bet	22/647	256/18723	8.56E-05	0.000963	0.000675	ZEB2/NR3C1/CH	22
GO	BP	GO:0002832	negative regulation of response to biotic st	13/647	108/18723	8.98E-05	0.001008	0.000706	SLAMF8/HAVCR	13
GO	BP	GO:0061037	negative regulation of cartilage developme	7/647	32/18723	9.02E-05	0.001009	0.000707	SNAI2/CTSK/EFE	7
GO	BP	GO:0042742	defense response to bacterium	27/647	350/18723	9.12E-05	0.001018	0.000713	COLEC12/TLR2/S	27
GO	BP	GO:0001738	morphogenesis of a polarized epithelium	12/647	94/18723	9.39E-05	0.001046	0.000733	GPC6/PRICKLE1.	12
GO	BP	GO:0046635	positive regulation of alpha-beta T cell act	10/647	67/18723	9.5E-05	0.001055	0.000739	CD86/NCKAP1L/	10
GO	BP	GO:0002861	regulation of inflammatory response to ant	8/647	43/18723	9.68E-05	0.001071	0.00075	FCGR2B/FYN/HC	8
GO	BP	GO:0031952	regulation of protein autophosphorylation	8/647	43/18723	9.68E-05	0.001071	0.00075	RASSF2/GPNMB.	8
GO	BP	GO:0001909	leukocyte mediated cytotoxicity	14/647	124/18723	9.86E-05	0.001087	0.000762	ITGAM/FCGR2B/	14
GO	BP	GO:0002758	innate immune response-activating signal	6/647	23/18723	0.000102	0.001109	0.000777	CLEC7A/FYN/HC	6
GO	BP	GO:0003081	regulation of systemic arterial blood press	6/647	23/18723	0.000102	0.001109	0.000777	SUCNR1/PCSK5/	6
GO	BP	GO:0006929	substrate-dependent cell migration	6/647	23/18723	0.000102	0.001109	0.000777	NRP1/VEGFC/PT	6
GO	BP	GO:0045932	negative regulation of muscle contraction	6/647	23/18723	0.000102	0.001109	0.000777	DOCK4/PIK3CG/	6
GO	BP	GO:0000768	syncytium formation by plasma membrane	9/647	55/18723	0.000102	0.001109	0.000777	ADAM12/PLEKH	9
GO	BP	GO:0140253	cell-cell fusion	9/647	55/18723	0.000102	0.001109	0.000777	ADAM12/PLEKH	9
GO	BP	GO:0034765	regulation of ion transmembrane transport	34/647	491/18723	0.000102	0.001112	0.000779	P2RX7/PKD2/CA	34
GO	BP	GO:0001960	negative regulation of cytokine-mediated s	11/647	81/18723	0.000104	0.001124	0.000787	PTPRC/SLIT2/PX	11
GO	BP	GO:0002679	respiratory burst involved in defense respo	5/647	15/18723	0.000109	0.001174	0.000823	SLAMF8/PIK3CG	5
GO	BP	GO:0034638	phosphatidylcholine catabolic process	5/647	15/18723	0.000109	0.001174	0.000823	PLA2G7/ENPP2/A	5
GO	BP	GO:0060099	regulation of phagocytosis, engulfment	5/647	15/18723	0.000109	0.001174	0.000823	RAB31/NCKAP1I	5
GO	BP	GO:0002335	mature B cell differentiation	7/647	33/18723	0.000111	0.001187	0.000831	DOCK10/FCGR2I	7
GO	BP	GO:0002431	Fc receptor mediated stimulatory signaling	7/647	33/18723	0.000111	0.001187	0.000831	FCGR2B/FYN/PT	7
GO	BP	GO:0043552	positive regulation of phosphatidylinositol	7/647	33/18723	0.000111	0.001187	0.000831	FGR/PRKD1/FGF	7
GO	BP	GO:0008037	cell recognition	20/647	225/18723	0.000112	0.001195	0.000837	NRP1/CLEC7A/C	20
GO	BP	GO:0150076	neuroinflammatory response	8/647	44/18723	0.000115	0.00122	0.000855	PTPRC/LRRK2/S	8
GO	BP	GO:0007178	transmembrane receptor protein serine/thr	27/647	355/18723	0.000115	0.001225	0.000858	ZEB2/CHST11/FE	27
GO	BP	GO:0001558	regulation of cell growth	30/647	414/18723	0.000117	0.001242	0.00087	NRP1/SPOCK1/C	30
GO	BP	GO:0010518	positive regulation of phospholipase activi	9/647	56/18723	0.000118	0.001244	0.000871	CD86/C5AR1/AV	9
GO	BP	GO:1901224	positive regulation of NIK/NF-kappaB sig	10/647	69/18723	0.000122	0.00129	0.000904	CD86/TLR2/HAV	10
GO	BP	GO:0052126	movement in host environment	17/647	175/18723	0.000124	0.001302	0.000912	NRP1/CD86/MRC	17
GO	BP	GO:0003018	vascular process in circulatory system	22/647	263/18723	0.000127	0.001331	0.000933	FERMT2/DOCK4	22
GO	BP	GO:0001655	urogenital system development	26/647	338/18723	0.000128	0.00134	0.000939	NRP1/PKD2/FBN	26
GO	BP	GO:0002821	positive regulation of adaptive immune res	13/647	112/18723	0.000131	0.001365	0.000956	CLEC7A/IL1R1/T	13
GO	BP	GO:0002755	MyD88-dependent toll-like receptor signal	6/647	24/18723	0.000131	0.00137	0.00096	TLR1/TLR8/TLR2	6
GO	BP	GO:0006949	syncytium formation	9/647	57/18723	0.000136	0.00141	0.000988	ADAM12/PLEKH	9
GO	BP	GO:0046718	viral entry into host cell	15/647	144/18723	0.000141	0.001463	0.001025	NRP1/CD86/MRC	15
GO	BP	GO:0033002	muscle cell proliferation	21/647	248/18723	0.000151	0.001567	0.001098	ZFPM2/MEIS1/G.	21
GO	BP	GO:0035265	organ growth	17/647	178/18723	0.000152	0.001573	0.001102	ZFPM2/HEG1/DD	17

GO	BP	GO:0009074	aromatic amino acid family catabolic proc	5/647	16/18723	0.000154	0.001577	0.001105	KMO/KYNU/IL4I	5
GO	BP	GO:0010819	regulation of T cell chemotaxis	5/647	16/18723	0.000154	0.001577	0.001105	CCR2/CXCL13/C	5
GO	BP	GO:1903236	regulation of leukocyte tethering or rolling	5/647	16/18723	0.000154	0.001577	0.001105	ITGA4/CXCL12/C	5
GO	BP	GO:1905153	regulation of membrane invagination	5/647	16/18723	0.000154	0.001577	0.001105	RAB31/NCKAP1L	5
GO	BP	GO:2000095	regulation of Wnt signaling pathway, plan	5/647	16/18723	0.000154	0.001577	0.001105	SFRP2/RSPO3/DA	5
GO	BP	GO:0010574	regulation of vascular endothelial growth	9/647	58/18723	0.000156	0.001587	0.001112	C3AR1/SULF1/CY	9
GO	BP	GO:0045824	negative regulation of innate immune resp	10/647	71/18723	0.000156	0.001589	0.001113	SLAMF8/HAVCR	10
GO	BP	GO:2000107	negative regulation of leukocyte apoptotic	8/647	46/18723	0.000159	0.001614	0.001131	DOCK8/HCLS1/L	8
GO	BP	GO:0032092	positive regulation of protein binding	11/647	85/18723	0.000161	0.001633	0.001144	NRP1/LRRK2/CT	11
GO	BP	GO:0006691	leukotriene metabolic process	7/647	35/18723	0.000165	0.001661	0.001164	TLR2/ALOX5AP/	7
GO	BP	GO:0033280	response to vitamin D	7/647	35/18723	0.000165	0.001661	0.001164	SNAI2/TNC/SPP1	7
GO	BP	GO:0051098	regulation of binding	27/647	363/18723	0.000166	0.001675	0.001173	NRP1/LOX/DZIP1	27
GO	BP	GO:0003071	renal system process involved in regulatio	6/647	25/18723	0.000168	0.001683	0.001179	GJA1/SUCNR1/P	6
GO	BP	GO:0032331	negative regulation of chondrocyte differe	6/647	25/18723	0.000168	0.001683	0.001179	SNAI2/EFEMP1/A	6
GO	BP	GO:0071560	cellular response to transforming growth f	21/647	250/18723	0.000169	0.001689	0.001184	ZEB2/NR3C1/CH	21
GO	BP	GO:1902903	regulation of supramolecular fiber organiz	28/647	383/18723	0.000169	0.001689	0.001184	NRP1/CCDC88A/	28
GO	BP	GO:0003015	heart process	21/647	251/18723	0.000179	0.00178	0.001247	CACNA2D1/KCN	21
GO	BP	GO:0060761	negative regulation of response to cytokin	11/647	86/18723	0.000179	0.00178	0.001247	PTPRC/SLIT2/PX	11
GO	BP	GO:0010975	regulation of neuron projection developme	31/647	445/18723	0.000184	0.001827	0.00128	NRP1/PLXNC1/P	31
GO	BP	GO:0050680	negative regulation of epithelial cell prolif	16/647	164/18723	0.000185	0.001827	0.00128	SNAI2/MCC/PTP	16
GO	BP	GO:0003044	regulation of systemic arterial blood press	8/647	47/18723	0.000186	0.001831	0.001283	SUCNR1/AVPR1	8
GO	BP	GO:0019884	antigen processing and presentation of exo	8/647	47/18723	0.000186	0.001831	0.001283	FCGR2B/CLEC4A	8
GO	BP	GO:0050830	defense response to Gram-positive bacteri	12/647	101/18723	0.000188	0.00185	0.001296	TLR2/HAVCR2/C	12
GO	BP	GO:0050848	regulation of calcium-mediated signaling	10/647	73/18723	0.000198	0.001939	0.001358	CLEC7A/LRRK2/	10
GO	BP	GO:0090322	regulation of superoxide metabolic proces	7/647	36/18723	0.000198	0.00194	0.001359	ITGAM/CLEC7A/	7
GO	BP	GO:0001776	leukocyte homeostasis	11/647	87/18723	0.000199	0.00194	0.001359	DOCK10/TNFSF1	11
GO	BP	GO:0022604	regulation of cell morphogenesis	24/647	309/18723	0.000199	0.00194	0.001359	NRP1/PLXNC1/F	24
GO	BP	GO:0010522	regulation of calcium ion transport into cy	12/647	102/18723	0.000207	0.002007	0.001406	P2RX7/PKD2/FY	12
GO	BP	GO:0032677	regulation of interleukin-8 production	12/647	102/18723	0.000207	0.002007	0.001406	TLR1/CLEC7A/T	12
GO	BP	GO:0045591	positive regulation of regulatory T cell dif	5/647	17/18723	0.000212	0.002058	0.001442	LILRB2/CR1/LIL	5
GO	BP	GO:0002673	regulation of acute inflammatory response	8/647	48/18723	0.000216	0.002092	0.001466	OSMR/FCGR2B/I	8
GO	BP	GO:0019233	sensory perception of pain	12/647	103/18723	0.000227	0.002182	0.001529	P2RX7/FYN/CXC	12
GO	BP	GO:0032637	interleukin-8 production	12/647	103/18723	0.000227	0.002182	0.001529	TLR1/CLEC7A/T	12
GO	BP	GO:0007156	homophilic cell adhesion via plasma mem	16/647	167/18723	0.000228	0.002182	0.001529	CDH11/PTPRM/C	16
GO	BP	GO:0007254	JNK cascade	16/647	167/18723	0.000228	0.002182	0.001529	RASSF2/ARHGEI	16
GO	BP	GO:0001822	kidney development	23/647	293/18723	0.00023	0.002203	0.001543	NRP1/PKD2/FBN	23
GO	BP	GO:0045861	negative regulation of proteolysis	26/647	351/18723	0.000233	0.002226	0.00156	GAS1/TIMP2/SP	26
GO	BP	GO:0002369	T cell cytokine production	7/647	37/18723	0.000237	0.002254	0.001579	IL1R1/TNFSF4/L	7

GO	BP	GO:0002724	regulation of T cell cytokine production	7/647	37/18723	0.000237	0.002254	0.001579	IL1R1/TNFSF4/LI	7
GO	BP	GO:0090218	positive regulation of lipid kinase activity	7/647	37/18723	0.000237	0.002254	0.001579	FGR/PRKD1/FGF	7
GO	BP	GO:0043270	positive regulation of ion transport	22/647	275/18723	0.00024	0.002272	0.001592	P2RX7/PKD2/CA	22
GO	BP	GO:0014910	regulation of smooth muscle cell migratio	11/647	89/18723	0.000244	0.002304	0.001614	NRP1/DOCK4/NO	11
GO	BP	GO:0052548	regulation of endopeptidase activity	30/647	432/18723	0.000246	0.002319	0.001625	CLEC7A/TIMP2/I	30
GO	BP	GO:0002292	T cell differentiation involved in immune	10/647	75/18723	0.000248	0.002319	0.001625	CD86/FCER1G/TI	10
GO	BP	GO:0046634	regulation of alpha-beta T cell activation	12/647	104/18723	0.000249	0.002319	0.001625	CD86/NCKAP1L/	12
GO	BP	GO:0051701	biological process involved in interaction	18/647	203/18723	0.000251	0.002319	0.001625	NRP1/CD86/MRC	18
GO	BP	GO:0002762	negative regulation of myeloid leukocyte c	8/647	49/18723	0.000251	0.002319	0.001625	MAFB/FBN1/INH	8
GO	BP	GO:0030225	macrophage differentiation	8/647	49/18723	0.000251	0.002319	0.001625	TLR2/HCLS1/INH	8
GO	BP	GO:0002291	T cell activation via T cell receptor contac	4/647	10/18723	0.000251	0.002319	0.001625	HAVCR2/LILRB1	4
GO	BP	GO:0048251	elastic fiber assembly	4/647	10/18723	0.000251	0.002319	0.001625	LOX/FBLN5/EMI	4
GO	BP	GO:0070099	regulation of chemokine-mediated signalir	4/647	10/18723	0.000251	0.002319	0.001625	SLIT2/TREM2/RO	4
GO	BP	GO:0070391	response to lipoteichoic acid	4/647	10/18723	0.000251	0.002319	0.001625	TLR2/CD14/CD36	4
GO	BP	GO:0071223	cellular response to lipoteichoic acid	4/647	10/18723	0.000251	0.002319	0.001625	TLR2/CD14/CD36	4
GO	BP	GO:2000425	regulation of apoptotic cell clearance	4/647	10/18723	0.000251	0.002319	0.001625	CCL2/CD300LF/C	4
GO	BP	GO:0051403	stress-activated MAPK cascade	20/647	239/18723	0.000252	0.002324	0.001628	CLEC7A/RASSF2	20
GO	BP	GO:0008217	regulation of blood pressure	17/647	186/18723	0.000258	0.002373	0.001663	VEGFC/POSTN/C	17
GO	BP	GO:0010573	vascular endothelial growth factor product	9/647	62/18723	0.000262	0.002403	0.001683	C3AR1/SULF1/CY	9
GO	BP	GO:0048002	antigen processing and presentation of pep	9/647	62/18723	0.000262	0.002403	0.001683	FCGR2B/CLEC4A	9
GO	BP	GO:0002433	immune response-regulating cell surface r	6/647	27/18723	0.000265	0.00241	0.001688	FCGR2B/FYN/PT	6
GO	BP	GO:0038096	Fc-gamma receptor signaling pathway inv	6/647	27/18723	0.000265	0.00241	0.001688	FCGR2B/FYN/PT	6
GO	BP	GO:0042402	cellular biogenic amine catabolic process	6/647	27/18723	0.000265	0.00241	0.001688	KMO/MOXD1/KY	6
GO	BP	GO:0045638	negative regulation of myeloid cell differe	11/647	90/18723	0.000269	0.002445	0.001713	MAFB/FBN1/ME	11
GO	BP	GO:0002455	humoral immune response mediated by cir	13/647	121/18723	0.000284	0.002568	0.001799	FCGR2B/PTPRC/	13
GO	BP	GO:0002544	chronic inflammatory response	5/647	18/18723	0.000286	0.002568	0.001799	VCAM1/GJA1/S1	5
GO	BP	GO:0002643	regulation of tolerance induction	5/647	18/18723	0.000286	0.002568	0.001799	HAVCR2/LILRB2	5
GO	BP	GO:0006957	complement activation, alternative pathwa	5/647	18/18723	0.000286	0.002568	0.001799	CFH/CR1/VSIG4/	5
GO	BP	GO:0030728	ovulation	5/647	18/18723	0.000286	0.002568	0.001799	TNFAIP6/INHBA	5
GO	BP	GO:0046851	negative regulation of bone remodeling	5/647	18/18723	0.000286	0.002568	0.001799	P2RX7/GREM1/II	5
GO	BP	GO:0046638	positive regulation of alpha-beta T cell dif	8/647	50/18723	0.00029	0.002598	0.00182	CD86/NCKAP1L/	8
GO	BP	GO:0022617	extracellular matrix disassembly	9/647	63/18723	0.000297	0.002651	0.001858	FAP/DDR2/CTSK	9
GO	BP	GO:0032233	positive regulation of actin filament bundl	9/647	63/18723	0.000297	0.002651	0.001858	NRP1/CCDC88A/	9
GO	BP	GO:0051928	positive regulation of calcium ion transpor	13/647	122/18723	0.000308	0.00275	0.001927	P2RX7/PKD2/CA	13
GO	BP	GO:0002313	mature B cell differentiation involved in ir	6/647	28/18723	0.000327	0.002905	0.002035	DOCK10/FCGR2I	6
GO	BP	GO:0045589	regulation of regulatory T cell differentiat	6/647	28/18723	0.000327	0.002905	0.002035	LILRB2/CR1/TNF	6
GO	BP	GO:0001914	regulation of T cell mediated cytotoxicity	7/647	39/18723	0.000334	0.002962	0.002075	FCGR2B/NCKAP	7
GO	BP	GO:0051495	positive regulation of cytoskeleton organiz	19/647	226/18723	0.000337	0.002979	0.002087	NRP1/P2RX7/CCI	19

GO	BP	GO:0030500	regulation of bone mineralization	10/647	78/18723	0.000343	0.003023	0.002118	P2RX7/DDR2/SR	10
GO	BP	GO:0061045	negative regulation of wound healing	10/647	78/18723	0.000343	0.003023	0.002118	FAP/PHLDB2/GJ.	10
GO	BP	GO:0072001	renal system development	23/647	302/18723	0.000355	0.003124	0.002188	NRP1/PKD2/FBN	23
GO	BP	GO:0046777	protein autophosphorylation	19/647	227/18723	0.000356	0.003125	0.002189	RASSF2/GPNMB.	19
GO	BP	GO:1902905	positive regulation of supramolecular fiber	18/647	209/18723	0.000358	0.003134	0.002196	NRP1/CCDC88A/	18
GO	BP	GO:0032755	positive regulation of interleukin-6 produc	11/647	93/18723	0.000359	0.003142	0.002202	TLR1/CLEC7A/T	11
GO	BP	GO:0060070	canonical Wnt signaling pathway	23/647	303/18723	0.000372	0.003249	0.002276	ZEB2/SNAI2/MIT	23
GO	BP	GO:0002888	positive regulation of myeloid leukocyte m	5/647	19/18723	0.000377	0.003273	0.002293	ITGAM/ITGB2/B	5
GO	BP	GO:0035313	wound healing, spreading of epidermal cel	5/647	19/18723	0.000377	0.003273	0.002293	FERMT2/PHLDB	5
GO	BP	GO:1903978	regulation of microglial cell activation	5/647	19/18723	0.000377	0.003273	0.002293	PTPRC/LRRK2/S	5
GO	BP	GO:0048260	positive regulation of receptor-mediated e	8/647	52/18723	0.000382	0.003292	0.002306	SGIP1/GREM1/C	8
GO	BP	GO:0002604	regulation of dendritic cell antigen process	4/647	11/18723	0.000384	0.003292	0.002306	FCGR2B/FGL2/C	4
GO	BP	GO:0032493	response to bacterial lipoprotein	4/647	11/18723	0.000384	0.003292	0.002306	TLR1/TLR2/CD1	4
GO	BP	GO:0046643	regulation of gamma-delta T cell activatio	4/647	11/18723	0.000384	0.003292	0.002306	NCKAP1L/PTPR	4
GO	BP	GO:0070189	kynurenine metabolic process	4/647	11/18723	0.000384	0.003292	0.002306	KMO/KYNU/TD	4
GO	BP	GO:0097048	dendritic cell apoptotic process	4/647	11/18723	0.000384	0.003292	0.002306	LILRB1/CXCL12/	4
GO	BP	GO:2000668	regulation of dendritic cell apoptotic proce	4/647	11/18723	0.000384	0.003292	0.002306	LILRB1/CXCL12/	4
GO	BP	GO:0031098	stress-activated protein kinase signaling ca	20/647	247/18723	0.000388	0.003317	0.002324	CLEC7A/RASSF2	20
GO	BP	GO:0010863	positive regulation of phospholipase C act	7/647	40/18723	0.000393	0.003352	0.002348	CD86/C5AR1/AV	7
GO	BP	GO:1904994	regulation of leukocyte adhesion to vascul	7/647	40/18723	0.000393	0.003352	0.002348	ITGB2/ITGA4/CX	7
GO	BP	GO:1903035	negative regulation of response to woundi	11/647	94/18723	0.000394	0.003356	0.002351	FAP/PHLDB2/GJ.	11
GO	BP	GO:0002862	negative regulation of inflammatory respo	6/647	29/18723	0.0004	0.003396	0.002379	FCGR2B/FYN/H	6
GO	BP	GO:0009310	amine catabolic process	6/647	29/18723	0.0004	0.003396	0.002379	KMO/MOXD1/K	6
GO	BP	GO:0050807	regulation of synapse organization	18/647	211/18723	0.000401	0.003396	0.002379	GPC6/FCGR2B/T	18
GO	BP	GO:0050728	negative regulation of inflammatory respo	16/647	176/18723	0.000412	0.003479	0.002438	FCGR2B/TNFAIP	16
GO	BP	GO:0003014	renal system process	12/647	110/18723	0.000419	0.003529	0.002472	SULF1/GJA1/SU	12
GO	BP	GO:0006939	smooth muscle contraction	12/647	110/18723	0.000419	0.003529	0.002472	DOCK4/SULF1/C	12
GO	BP	GO:1901654	response to ketone	17/647	194/18723	0.000423	0.003553	0.002489	NR3C1/TLR2/PO	17
GO	BP	GO:0002532	production of molecular mediator involved	11/647	95/18723	0.000432	0.003627	0.002541	CLEC7A/SLAMF	11
GO	BP	GO:0008347	glial cell migration	8/647	53/18723	0.000437	0.003658	0.002563	GLI3/CCL2/GPR1	8
GO	BP	GO:0001938	positive regulation of endothelial cell prol	12/647	111/18723	0.000456	0.003808	0.002668	NRP1/VEGFC/IT	12
GO	BP	GO:0006911	phagocytosis, engulfment	13/647	127/18723	0.000457	0.00381	0.002669	ITGAM/RAB31/F	13
GO	BP	GO:1905521	regulation of macrophage migration	7/647	41/18723	0.00046	0.003832	0.002685	C3AR1/SLAMF8/	7
GO	BP	GO:0002440	production of molecular mediator of immu	23/647	308/18723	0.000468	0.003893	0.002728	CLEC7A/IL1R1/C	23
GO	BP	GO:0003073	regulation of systemic arterial blood press	11/647	96/18723	0.000473	0.003929	0.002753	POSTN/GJA1/SU	11
GO	BP	GO:0007202	activation of phospholipase C activity	6/647	30/18723	0.000486	0.004011	0.00281	CD86/C5AR1/AV	6
GO	BP	GO:0034694	response to prostaglandin	6/647	30/18723	0.000486	0.004011	0.00281	TNFSF4/PTGFR/	6
GO	BP	GO:0051968	positive regulation of synaptic transmissio	6/647	30/18723	0.000486	0.004011	0.00281	TSHZ3/CCL2/KM	6

GO	BP	GO:0032930	positive regulation of superoxide anion ge	5/647	20/18723	0.000488	0.004011	0.00281	ITGAM/CLEC7A/	5
GO	BP	GO:0034104	negative regulation of tissue remodeling	5/647	20/18723	0.000488	0.004011	0.00281	P2RX7/GREM1/II	5
GO	BP	GO:0046629	gamma-delta T cell activation	5/647	20/18723	0.000488	0.004011	0.00281	NCKAP1L/PTPR(	5
GO	BP	GO:0006941	striated muscle contraction	16/647	179/18723	0.000497	0.004071	0.002852	CACNA2D1/KCN	16
GO	BP	GO:0032330	regulation of chondrocyte differentiation	8/647	54/18723	0.000498	0.004071	0.002852	SNAI2/TRPS1/GL	8
GO	BP	GO:0014909	smooth muscle cell migration	11/647	97/18723	0.000518	0.004221	0.002958	NRP1/DOCK4/N(	11
GO	BP	GO:0070167	regulation of biomineral tissue developme	11/647	97/18723	0.000518	0.004221	0.002958	P2RX7/DDR2/SR(	11
GO	BP	GO:0031640	killing of cells of other organism	9/647	68/18723	0.00053	0.004292	0.003007	P2RX7/CLEC7A/'	9
GO	BP	GO:0051926	negative regulation of calcium ion transpo	9/647	68/18723	0.00053	0.004292	0.003007	PKD2/CLIC2/LIL	9
GO	BP	GO:1900046	regulation of hemostasis	9/647	68/18723	0.00053	0.004292	0.003007	FAP/SERPING1/(	9
GO	BP	GO:0009612	response to mechanical stimulus	18/647	216/18723	0.000531	0.004292	0.003007	PKD2/TLR8/SLC	18
GO	BP	GO:0006022	aminoglycan metabolic process	13/647	129/18723	0.000531	0.004292	0.003007	DSE/CHST11/ST3	13
GO	BP	GO:0030890	positive regulation of B cell proliferation	7/647	42/18723	0.000536	0.004313	0.003022	TNFSF13B/NCKA	7
GO	BP	GO:1900274	regulation of phospholipase C activity	7/647	42/18723	0.000536	0.004313	0.003022	CD86/C5AR1/AV	7
GO	BP	GO:0002708	positive regulation of lymphocyte mediate	12/647	113/18723	0.000536	0.004313	0.003022	IL1R1/PTPRC/KL	12
GO	BP	GO:0032490	detection of molecule of bacterial origin	4/647	12/18723	0.00056	0.004483	0.003141	TLR1/LY96/TLR2	4
GO	BP	GO:0043301	negative regulation of leukocyte degranul	4/647	12/18723	0.00056	0.004483	0.003141	FCGR2B/CD84/N	4
GO	BP	GO:0043383	negative T cell selection	4/647	12/18723	0.00056	0.004483	0.003141	DOCK2/PTPRC/C	4
GO	BP	GO:0045620	negative regulation of lymphocyte differen	8/647	55/18723	0.000565	0.004512	0.003161	GLI3/INHBA/CR1	8
GO	BP	GO:0043367	CD4-positive, alpha-beta T cell differentia	10/647	83/18723	0.000568	0.004523	0.003169	CD86/NCKAP1L/	10
GO	BP	GO:0050886	endocrine process	10/647	83/18723	0.000568	0.004523	0.003169	GJA1/INHBA/SU(	10
GO	BP	GO:0043547	positive regulation of GTPase activity	20/647	255/18723	0.000582	0.004628	0.003242	DOCK10/FERMT	20
GO	BP	GO:0002063	chondrocyte development	6/647	31/18723	0.000585	0.004628	0.003243	CHST11/SULF1/S	6
GO	BP	GO:0002828	regulation of type 2 immune response	6/647	31/18723	0.000585	0.004628	0.003243	CD86/TNFSF4/AI	6
GO	BP	GO:0045066	regulatory T cell differentiation	6/647	31/18723	0.000585	0.004628	0.003243	LILRB2/CR1/TNI	6
GO	BP	GO:0050805	negative regulation of synaptic transmissio	9/647	69/18723	0.000592	0.004649	0.003258	LILRB2/LRRK2/I	9
GO	BP	GO:0051966	regulation of synaptic transmission, glutan	9/647	69/18723	0.000592	0.004649	0.003258	TSHZ3/CCL2/LRI	9
GO	BP	GO:0060193	positive regulation of lipase activity	9/647	69/18723	0.000592	0.004649	0.003258	CD86/C5AR1/AV	9
GO	BP	GO:0050803	regulation of synapse structure or activity	18/647	218/18723	0.000592	0.004649	0.003258	GPC6/FCGR2B/T	18
GO	BP	GO:0016032	viral process	28/647	415/18723	0.000616	0.004826	0.003381	NRP1/CD86/MRC	28
GO	BP	GO:0110149	regulation of biomineralization	11/647	99/18723	0.000617	0.004826	0.003381	P2RX7/DDR2/SR(	11
GO	BP	GO:0042088	T-helper 1 type immune response	7/647	43/18723	0.000621	0.004844	0.003394	IL1R1/HAVCR2/T	7
GO	BP	GO:0033630	positive regulation of cell adhesion mediat	5/647	21/18723	0.000623	0.004844	0.003394	NCKAP1L/SFRP2	5
GO	BP	GO:0044766	multi-organism transport	5/647	21/18723	0.000623	0.004844	0.003394	CTSL/SIGLEC1/(	5
GO	BP	GO:1902579	multi-organism localization	5/647	21/18723	0.000623	0.004844	0.003394	CTSL/SIGLEC1/(	5
GO	BP	GO:0042310	vasoconstriction	10/647	84/18723	0.000625	0.004855	0.003402	DOCK4/GJA1/AV	10
GO	BP	GO:0030278	regulation of ossification	12/647	115/18723	0.000629	0.004873	0.003414	P2RX7/DDR2/SR(	12
GO	BP	GO:0150063	visual system development	26/647	375/18723	0.000638	0.004939	0.003461	NRP1/ITGAM/MI	26

GO	BP	GO:0016525	negative regulation of angiogenesis	14/647	149/18723	0.000669	0.005166	0.003619	PTPRM/SULF1/D	14
GO	BP	GO:0043372	positive regulation of CD4-positive, alpha	6/647	32/18723	0.0007	0.005386	0.003774	CD86/NCKAP1L/	6
GO	BP	GO:0050850	positive regulation of calcium-mediated si	6/647	32/18723	0.0007	0.005386	0.003774	CLEC7A/TREM2/	6
GO	BP	GO:0050851	antigen receptor-mediated signaling pathw	19/647	240/18723	0.000707	0.005433	0.003806	MNDA/LCP2/FC(	19
GO	BP	GO:0034329	cell junction assembly	28/647	420/18723	0.000742	0.005694	0.003989	NRP1/SNAI2/GP(	28
GO	BP	GO:0099173	postsynapse organization	15/647	168/18723	0.000745	0.005706	0.003997	NRP1/DOCK10/F	15
GO	BP	GO:0034103	regulation of tissue remodeling	10/647	86/18723	0.000754	0.005769	0.004042	P2RX7/GPNMB/I	10
GO	BP	GO:0071900	regulation of protein serine/threonine kina	25/647	359/18723	0.000756	0.005775	0.004046	PKD2/FERMT2/H	25
GO	BP	GO:2000181	negative regulation of blood vessel morph	14/647	151/18723	0.000763	0.00582	0.004078	PTPRM/SULF1/D	14
GO	BP	GO:0002220	innate immune response activating cell sur	5/647	22/18723	0.000783	0.005963	0.004178	CLEC7A/FYN/KI	5
GO	BP	GO:0032494	response to peptidoglycan	4/647	13/18723	0.000787	0.005973	0.004185	C5AR1/IRAK3/TI	4
GO	BP	GO:0051709	regulation of killing of cells of other orgar	4/647	13/18723	0.000787	0.005973	0.004185	P2RX7/CLEC7A/(	4
GO	BP	GO:0006937	regulation of muscle contraction	15/647	169/18723	0.000792	0.005982	0.004191	DOCK4/CLIC2/PI	15
GO	BP	GO:0009308	amine metabolic process	12/647	118/18723	0.000792	0.005982	0.004191	ITGAM/VCAM1/	12
GO	BP	GO:0030203	glycosaminoglycan metabolic process	12/647	118/18723	0.000792	0.005982	0.004191	DSE/CHST11/ST3	12
GO	BP	GO:1904063	negative regulation of cation transmembra	11/647	102/18723	0.000794	0.005985	0.004193	PKD2/KCNE4/CL	11
GO	BP	GO:0007249	I-kappaB kinase/NF-kappaB signaling	21/647	281/18723	0.000804	0.006055	0.004242	CLEC7A/TLR8/T	21
GO	BP	GO:0048880	sensory system development	26/647	381/18723	0.000806	0.006059	0.004245	NRP1/ITGAM/MI	26
GO	BP	GO:0051092	positive regulation of NF-kappaB transcrip	14/647	152/18723	0.000815	0.006103	0.004276	CLEC7A/TLR2/B	14
GO	BP	GO:1901343	negative regulation of vasculature develop	14/647	152/18723	0.000815	0.006103	0.004276	PTPRM/SULF1/D	14
GO	BP	GO:0030850	prostate gland development	7/647	45/18723	0.000824	0.006154	0.004312	SULF1/GLI3/SER	7
GO	BP	GO:0085029	extracellular matrix assembly	7/647	45/18723	0.000824	0.006154	0.004312	PHLDB2/LOX/PX	7
GO	BP	GO:0099024	plasma membrane invagination	13/647	136/18723	0.000876	0.00652	0.004568	ITGAM/RAB31/F	13
GO	BP	GO:1900180	regulation of protein localization to nucleu	13/647	136/18723	0.000876	0.00652	0.004568	FERMT2/MDFIC/	13
GO	BP	GO:0061035	regulation of cartilage development	9/647	73/18723	0.000897	0.006665	0.00467	SNAI2/TRPS1/GL	9
GO	BP	GO:0071774	response to fibroblast growth factor	12/647	120/18723	0.00092	0.006824	0.004781	SULF1/CCL2/POS	12
GO	BP	GO:0007200	phospholipase C-activating G protein-coup	11/647	104/18723	0.000934	0.006921	0.004849	C3AR1/FPR3/GPI	11
GO	BP	GO:0060048	cardiac muscle contraction	13/647	137/18723	0.000938	0.006937	0.00486	CACNA2D1/KCN	13
GO	BP	GO:0007157	heterophilic cell-cell adhesion via plasma	7/647	46/18723	0.000944	0.006957	0.004874	VCAM1/HMCN1/	7
GO	BP	GO:0043300	regulation of leukocyte degranulation	7/647	46/18723	0.000944	0.006957	0.004874	ITGAM/FCGR2B/	7
GO	BP	GO:0050650	chondroitin sulfate proteoglycan biosynthe	5/647	23/18723	0.000973	0.007161	0.005017	DSE/CHST11/CH	5
GO	BP	GO:0032743	positive regulation of interleukin-2 produc	6/647	34/18723	0.000979	0.007197	0.005042	CLEC7A/CD86/PT	6
GO	BP	GO:0002437	inflammatory response to antigenic stimul	9/647	74/18723	0.00099	0.007268	0.005092	FCGR2B/FYN/HC	9
GO	BP	GO:0051099	positive regulation of binding	15/647	173/18723	0.001007	0.007374	0.005167	NRP1/CLIC2/LRF	15
GO	BP	GO:0009100	glycoprotein metabolic process	26/647	387/18723	0.001012	0.0074	0.005185	DSE/CHST11/ST8	26
GO	BP	GO:0009620	response to fungus	8/647	60/18723	0.001021	0.007442	0.005214	CLEC7A/BTK/CL	8
GO	BP	GO:0031663	lipopolysaccharide-mediated signaling pat	8/647	60/18723	0.001021	0.007442	0.005214	LY96/TLR2/CCL2	8
GO	BP	GO:0007517	muscle organ development	23/647	327/18723	0.00106	0.007715	0.005406	ZFPM2/HEG1/LC	23

GO	BP	GO:0019730	antimicrobial humoral response	12/647	122/18723	0.001064	0.007723	0.005411	CXCL13/CXCL9/	12
GO	BP	GO:0007584	response to nutrient	15/647	174/18723	0.001067	0.007723	0.005411	SNAI2/VCAM1/P	15
GO	BP	GO:0002467	germinal center formation	4/647	14/18723	0.001072	0.007723	0.005411	TNFSF13B/KLHL	4
GO	BP	GO:0035589	G protein-coupled purinergic nucleotide re	4/647	14/18723	0.001072	0.007723	0.005411	GPR34/P2RY13/C	4
GO	BP	GO:0043374	CD8-positive, alpha-beta T cell differentia	4/647	14/18723	0.001072	0.007723	0.005411	NCKAP1L/TNFSI	4
GO	BP	GO:0050930	induction of positive chemotaxis	4/647	14/18723	0.001072	0.007723	0.005411	VEGFC/CXCL12/	4
GO	BP	GO:0086103	G protein-coupled receptor signaling pathw	4/647	14/18723	0.001072	0.007723	0.005411	RGS2/CAV1/PLN	4
GO	BP	GO:0014911	positive regulation of smooth muscle cell r	7/647	47/18723	0.001076	0.00774	0.005423	NRP1/DOCK4/NC	7
GO	BP	GO:0006690	icosanoid metabolic process	12/647	123/18723	0.001143	0.008207	0.00575	TLR2/CYP1B1/AI	12
GO	BP	GO:0030177	positive regulation of Wnt signaling pathw	13/647	140/18723	0.001146	0.008216	0.005757	ZEB2/TLR2/SULI	13
GO	BP	GO:0018149	peptide cross-linking	6/647	35/18723	0.001148	0.008216	0.005757	DCN/COL3A1/AN	6
GO	BP	GO:0002922	positive regulation of humoral immune res	5/647	24/18723	0.001194	0.008504	0.005958	FCGR2B/PTPRC/	5
GO	BP	GO:0032928	regulation of superoxide anion generation	5/647	24/18723	0.001194	0.008504	0.005958	ITGAM/CLEC7A/	5
GO	BP	GO:0045649	regulation of macrophage differentiation	5/647	24/18723	0.001194	0.008504	0.005958	HCLS1/INHBA/C	5
GO	BP	GO:0043534	blood vessel endothelial cell migration	15/647	176/18723	0.001198	0.008504	0.005958	NRP1/VEGFC/MI	15
GO	BP	GO:0001937	negative regulation of endothelial cell prol	9/647	76/18723	0.001201	0.008504	0.005958	PTPRM/SULF1/C	9
GO	BP	GO:0032720	negative regulation of tumor necrosis facto	9/647	76/18723	0.001201	0.008504	0.005958	GPNMB/HAVCR	9
GO	BP	GO:2000379	positive regulation of reactive oxygen spec	9/647	76/18723	0.001201	0.008504	0.005958	ITGAM/CLEC7A/	9
GO	BP	GO:0032409	regulation of transporter activity	22/647	310/18723	0.001202	0.008504	0.005958	PKD2/CACNA2D	22
GO	BP	GO:0002686	negative regulation of leukocyte migration	7/647	48/18723	0.001223	0.008618	0.006038	SLAMF8/CCL2/S	7
GO	BP	GO:0035296	regulation of tube diameter	13/647	141/18723	0.001224	0.008618	0.006038	DOCK4/GJA1/RG	13
GO	BP	GO:0097746	blood vessel diameter maintenance	13/647	141/18723	0.001224	0.008618	0.006038	DOCK4/GJA1/RG	13
GO	BP	GO:0019216	regulation of lipid metabolic process	23/647	331/18723	0.001245	0.008751	0.006131	SNAI2/PIK3CG/F	23
GO	BP	GO:0060485	mesenchyme development	21/647	291/18723	0.001251	0.00878	0.006151	NRP1/PKD2/SNA	21
GO	BP	GO:1903169	regulation of calcium ion transmembrane t	14/647	159/18723	0.001263	0.008852	0.006202	PKD2/CACNA2D	14
GO	BP	GO:0007188	adenylate cyclase-modulating G protein-co	18/647	233/18723	0.001276	0.008922	0.006251	GPR65/CALCRL/	18
GO	BP	GO:0006958	complement activation, classical pathway	11/647	108/18723	0.001277	0.008922	0.006251	CR1/SERPING1/C	11
GO	BP	GO:0010038	response to metal ion	25/647	373/18723	0.001296	0.009044	0.006336	PKD2/VCAM1/LI	25
GO	BP	GO:0010632	regulation of epithelial cell migration	21/647	292/18723	0.001305	0.009072	0.006356	NRP1/MCC/PTPR	21
GO	BP	GO:0070588	calcium ion transmembrane transport	22/647	312/18723	0.001306	0.009072	0.006356	P2RX7/PKD2/CA	22
GO	BP	GO:0035150	regulation of tube size	13/647	142/18723	0.001306	0.009072	0.006356	DOCK4/GJA1/RG	13
GO	BP	GO:0021675	nerve development	9/647	77/18723	0.001319	0.009152	0.006412	NRP1/MAFB/VCA	9
GO	BP	GO:0097305	response to alcohol	19/647	253/18723	0.001322	0.009158	0.006416	VCAM1/FYN/INI	19
GO	BP	GO:0002691	regulation of cellular extravasation	6/647	36/18723	0.001338	0.009237	0.006472	IL1R1/ITGA4/CX	6
GO	BP	GO:0042092	type 2 immune response	6/647	36/18723	0.001338	0.009237	0.006472	CD86/TNFSF4/AI	6
GO	BP	GO:0034766	negative regulation of ion transmembrane	11/647	109/18723	0.001377	0.009494	0.006652	PKD2/KCNE4/CL	11
GO	BP	GO:0001913	T cell mediated cytotoxicity	7/647	49/18723	0.001385	0.009537	0.006682	FCGR2B/NCKAP	7
GO	BP	GO:0031343	positive regulation of cell killing	8/647	63/18723	0.001411	0.009702	0.006798	ITGAM/CLEC7A/	8

GO	BP	GO:0010820	positive regulation of T cell chemotaxis	4/647	15/18723	0.001422	0.009737	0.006822	CCR2/CXCL13/C	4
GO	BP	GO:0030852	regulation of granulocyte differentiation	4/647	15/18723	0.001422	0.009737	0.006822	HCLS1/EVI2B/C1	4
GO	BP	GO:0045986	negative regulation of smooth muscle cont	4/647	15/18723	0.001422	0.009737	0.006822	DOCK4/CALCRL	4
GO	BP	GO:1903556	negative regulation of tumor necrosis facto	9/647	78/18723	0.001447	0.009859	0.006908	GPNMB/HAVCR:	9
GO	BP	GO:0033622	integrin activation	5/647	25/18723	0.001451	0.009859	0.006908	FERMT2/PLEK/C	5
GO	BP	GO:0048143	astrocyte activation	5/647	25/18723	0.001451	0.009859	0.006908	C5AR1/TREM2/C	5
GO	BP	GO:0098581	detection of external biotic stimulus	5/647	25/18723	0.001451	0.009859	0.006908	TLR1/CLEC7A/L	5
GO	BP	GO:1904996	positive regulation of leukocyte adhesion t	5/647	25/18723	0.001451	0.009859	0.006908	ITGB2/ITGA4/CC	5
GO	BP	GO:0032970	regulation of actin filament-based process	26/647	397/18723	0.001455	0.009873	0.006918	NRP1/CCDC88A/	26
GO	BP	GO:1905954	positive regulation of lipid localization	11/647	110/18723	0.001483	0.010037	0.007032	MSR1/CAV1/CD3	11
GO	BP	GO:0010324	membrane invagination	13/647	144/18723	0.001483	0.010037	0.007032	ITGAM/RAB31/F	13
GO	BP	GO:0043409	negative regulation of MAPK cascade	15/647	180/18723	0.001501	0.010139	0.007103	P2RX7/PTPRC/R(	15
GO	BP	GO:0002042	cell migration involved in sprouting angio	10/647	94/18723	0.001506	0.01016	0.007118	NRP1/MEOX2/SL	10
GO	BP	GO:0090596	sensory organ morphogenesis	19/647	256/18723	0.001516	0.010199	0.007145	PRRX1/MAFB/FE	19
GO	BP	GO:1903522	regulation of blood circulation	19/647	256/18723	0.001516	0.010199	0.007145	DOCK4/CACNA2	19
GO	BP	GO:0009410	response to xenobiotic stimulus	29/647	462/18723	0.001526	0.010249	0.007181	LOX/PTPRM/SLA	29
GO	BP	GO:1900047	negative regulation of hemostasis	7/647	50/18723	0.001563	0.010483	0.007345	FAP/SERPING1/T	7
GO	BP	GO:0006940	regulation of smooth muscle contraction	8/647	64/18723	0.001565	0.010483	0.007345	DOCK4/CALCRL	8
GO	BP	GO:0051279	regulation of release of sequestered calciu	9/647	79/18723	0.001584	0.010594	0.007423	PKD2/CLIC2/PRK	9
GO	BP	GO:0071902	positive regulation of protein serine/threor	16/647	200/18723	0.001624	0.01085	0.007602	PKD2/FERMT2/P	16
GO	BP	GO:0022898	regulation of transmembrane transporter a	20/647	278/18723	0.001676	0.011166	0.007823	PKD2/CACNA2D	20
GO	BP	GO:0110053	regulation of actin filament organization	20/647	278/18723	0.001676	0.011166	0.007823	NRP1/CCDC88A/	20
GO	BP	GO:0072593	reactive oxygen species metabolic process	18/647	239/18723	0.001696	0.01128	0.007903	ITGAM/CLEC7A/	18
GO	BP	GO:0048705	skeletal system morphogenesis	17/647	220/18723	0.001715	0.011367	0.007964	PRRX1/CHST11/(	17
GO	BP	GO:0043406	positive regulation of MAP kinase activity	11/647	112/18723	0.001716	0.011367	0.007964	PDGFC/PIK3CG/I	11
GO	BP	GO:1901222	regulation of NIK/NF-kappaB signaling	11/647	112/18723	0.001716	0.011367	0.007964	RASSF2/CD86/TI	11
GO	BP	GO:0002720	positive regulation of cytokine production	8/647	65/18723	0.001732	0.011436	0.008013	CLEC7A/IL1R1/L	8
GO	BP	GO:1905330	regulation of morphogenesis of an epitheli	8/647	65/18723	0.001732	0.011436	0.008013	SULF1/GJA1/FGI	8
GO	BP	GO:0003416	endochondral bone growth	5/647	26/18723	0.001746	0.011465	0.008033	DDR2/BNC2/EV(	5
GO	BP	GO:0006706	steroid catabolic process	5/647	26/18723	0.001746	0.011465	0.008033	HSD11B1/SPP1/A	5
GO	BP	GO:0009435	NAD biosynthetic process	5/647	26/18723	0.001746	0.011465	0.008033	KMO/KYNU/PTC	5
GO	BP	GO:1905523	positive regulation of macrophage migrati	5/647	26/18723	0.001746	0.011465	0.008033	C3AR1/C5AR1/C:	5
GO	BP	GO:0046850	regulation of bone remodeling	7/647	51/18723	0.001759	0.011536	0.008083	P2RX7/CSF1R/GI	7
GO	BP	GO:0055017	cardiac muscle tissue growth	10/647	96/18723	0.001767	0.011571	0.008107	ZFPM2/HEG1/MI	10
GO	BP	GO:0048246	macrophage chemotaxis	6/647	38/18723	0.001788	0.011695	0.008194	C3AR1/SLAMF8/	6
GO	BP	GO:0010810	regulation of cell-substrate adhesion	17/647	221/18723	0.0018	0.011756	0.008237	NRP1/FERMT2/P	17
GO	BP	GO:0044344	cellular response to fibroblast growth facto	11/647	113/18723	0.001844	0.011917	0.008349	SULF1/CCL2/POS	11
GO	BP	GO:0001991	regulation of systemic arterial blood press	4/647	16/18723	0.001845	0.011917	0.008349	SUCNR1/PCSK5/	4

GO	BP	GO:0002399	MHC class II protein complex assembly	4/647	16/18723	0.001845	0.011917	0.008349	HLA-DPA1/HLA-	4
GO	BP	GO:0002503	peptide antigen assembly with MHC class	4/647	16/18723	0.001845	0.011917	0.008349	HLA-DPA1/HLA-	4
GO	BP	GO:0002830	positive regulation of type 2 immune respo	4/647	16/18723	0.001845	0.011917	0.008349	CD86/TNFSF4/RS	4
GO	BP	GO:0002921	negative regulation of humoral immune re	4/647	16/18723	0.001845	0.011917	0.008349	FCGR2B/CR1/SE	4
GO	BP	GO:0051770	positive regulation of nitric-oxide synthase	4/647	16/18723	0.001845	0.011917	0.008349	TLR2/CCL2/LRR	4
GO	BP	GO:0071639	positive regulation of monocyte chemotac	4/647	16/18723	0.001845	0.011917	0.008349	CLEC7A/CD84/T	4
GO	BP	GO:0060047	heart contraction	18/647	241/18723	0.00186	0.011994	0.008403	CACNA2D1/KCN	18
GO	BP	GO:2001257	regulation of cation channel activity	15/647	184/18723	0.001865	0.012009	0.008414	PKD2/CACNA2D	15
GO	BP	GO:0046209	nitric oxide metabolic process	9/647	81/18723	0.001889	0.012135	0.008502	CLEC7A/PKD2/T	9
GO	BP	GO:0048708	astrocyte differentiation	9/647	81/18723	0.001889	0.012135	0.008502	VIM/C5AR1/ROR	9
GO	BP	GO:0051384	response to glucocorticoid	13/647	148/18723	0.001899	0.012179	0.008533	NR3C1/SERPINF	13
GO	BP	GO:0001933	negative regulation of protein phosphoryla	23/647	342/18723	0.001904	0.012197	0.008546	RASSF2/HEG1/S	23
GO	BP	GO:0019229	regulation of vasoconstriction	8/647	66/18723	0.001912	0.012215	0.008558	DOCK4/GJA1/AV	8
GO	BP	GO:0030193	regulation of blood coagulation	8/647	66/18723	0.001912	0.012215	0.008558	FAP/SERPING1/C	8
GO	BP	GO:0090101	negative regulation of transmembrane rece	12/647	131/18723	0.001966	0.012542	0.008787	CHST11/FBN1/C	12
GO	BP	GO:0002701	negative regulation of production of mole	6/647	39/18723	0.002053	0.013042	0.009137	FCGR2B/CR1/IR	6
GO	BP	GO:0045923	positive regulation of fatty acid metabolic	6/647	39/18723	0.002053	0.013042	0.009137	AVPR1A/TWIST	6
GO	BP	GO:2000516	positive regulation of CD4-positive, alpha	6/647	39/18723	0.002053	0.013042	0.009137	CD86/NCKAP1L/	6
GO	BP	GO:2001057	reactive nitrogen species metabolic proces	9/647	82/18723	0.002059	0.013062	0.009151	CLEC7A/PKD2/T	9
GO	BP	GO:0010758	regulation of macrophage chemotaxis	5/647	27/18723	0.002082	0.013142	0.009208	C3AR1/SLAMF8/	5
GO	BP	GO:0031954	positive regulation of protein autophospho	5/647	27/18723	0.002082	0.013142	0.009208	RASSF2/GPNMB	5
GO	BP	GO:0033688	regulation of osteoblast proliferation	5/647	27/18723	0.002082	0.013142	0.009208	CTHRC1/GREM1	5
GO	BP	GO:0060142	regulation of syncytium formation by plas	5/647	27/18723	0.002082	0.013142	0.009208	PLEKHO1/TYRO	5
GO	BP	GO:0010001	glial cell differentiation	17/647	225/18723	0.002176	0.013717	0.00961	TLR2/VIM/GLI3/	17
GO	BP	GO:0045744	negative regulation of G protein-coupled r	7/647	53/18723	0.002207	0.013891	0.009733	RGS2/PLEK/RGS	7
GO	BP	GO:0046328	regulation of JNK cascade	12/647	133/18723	0.002234	0.014041	0.009837	RASSF2/FCGR2E	12
GO	BP	GO:0048592	eye morphogenesis	13/647	151/18723	0.002269	0.014225	0.009966	FBN1/PTPRM/CC	13
GO	BP	GO:2001236	regulation of extrinsic apoptotic signaling	13/647	151/18723	0.002269	0.014225	0.009966	NRP1/SNAI2/FYN	13
GO	BP	GO:0021782	glial cell development	11/647	116/18723	0.002272	0.014225	0.009966	TLR2/VIM/C5AR	11
GO	BP	GO:0046637	regulation of alpha-beta T cell differentiat	8/647	68/18723	0.002317	0.014487	0.01015	CD86/NCKAP1L/	8
GO	BP	GO:0001780	neutrophil homeostasis	4/647	17/18723	0.002347	0.014598	0.010228	ANXA1/PDE4B/C	4
GO	BP	GO:0010934	macrophage cytokine production	4/647	17/18723	0.002347	0.014598	0.010228	LAPTM5/IRAK3/	4
GO	BP	GO:0010935	regulation of macrophage cytokine produc	4/647	17/18723	0.002347	0.014598	0.010228	LAPTM5/IRAK3/	4
GO	BP	GO:2000811	negative regulation of anoikis	4/647	17/18723	0.002347	0.014598	0.010228	SNAI2/CAV1/ITC	4
GO	BP	GO:0043087	regulation of GTPase activity	23/647	348/18723	0.002375	0.014753	0.010336	PLXNC1/DOCK1	23
GO	BP	GO:0008277	regulation of G protein-coupled receptor s	12/647	134/18723	0.002379	0.014753	0.010336	RGS2/PLEK/RGS	12
GO	BP	GO:0060840	artery development	10/647	100/18723	0.002399	0.014857	0.010409	NRP1/PRRX1/PK	10
GO	BP	GO:0010524	positive regulation of calcium ion transpo	7/647	54/18723	0.002462	0.015202	0.010651	P2RX7/PKD2/CA	7

GO	BP	GO:0002507	tolerance induction	5/647	28/18723	0.002464	0.015202	0.010651	HAVCR2/LILRB2	5
GO	BP	GO:0045671	negative regulation of osteoclast differenti	5/647	28/18723	0.002464	0.015202	0.010651	MAFB/FBN1/LIL	5
GO	BP	GO:0002287	alpha-beta T cell activation involved in im	8/647	69/18723	0.002544	0.015652	0.010966	CD86/TNFSF4/GI	8
GO	BP	GO:0002293	alpha-beta T cell differentiation involved i	8/647	69/18723	0.002544	0.015652	0.010966	CD86/TNFSF4/GI	8
GO	BP	GO:0006576	cellular biogenic amine metabolic process	10/647	101/18723	0.002582	0.015846	0.011102	ITGAM/ITGB2/R	10
GO	BP	GO:0098869	cellular oxidant detoxification	10/647	101/18723	0.002582	0.015846	0.011102	GPX8/CLIC2/AL(	10
GO	BP	GO:0032733	positive regulation of interleukin-10 produ	6/647	41/18723	0.002669	0.016336	0.011446	CLEC7A/TLR2/T	6
GO	BP	GO:0032735	positive regulation of interleukin-12 produ	6/647	41/18723	0.002669	0.016336	0.011446	CLEC7A/TLR2/L	6
GO	BP	GO:0035710	CD4-positive, alpha-beta T cell activation	10/647	102/18723	0.002776	0.016972	0.011891	CD86/NCKAP1L/	10
GO	BP	GO:0032872	regulation of stress-activated MAPK casca	15/647	192/18723	0.002815	0.017186	0.012041	CLEC7A/RASSF2	15
GO	BP	GO:0031346	positive regulation of cell projection orgar	23/647	353/18723	0.002841	0.01732	0.012135	NRP1/PLXNC1/P.	23
GO	BP	GO:1903510	mucopolysaccharide metabolic process	9/647	86/18723	0.002861	0.017421	0.012206	DSE/CHST11/ST3	9
GO	BP	GO:0019359	nicotinamide nucleotide biosynthetic proc	5/647	29/18723	0.002894	0.017509	0.012267	KMO/KYNU/PTC	5
GO	BP	GO:0019363	pyridine nucleotide biosynthetic process	5/647	29/18723	0.002894	0.017509	0.012267	KMO/KYNU/PTC	5
GO	BP	GO:0021602	cranial nerve morphogenesis	5/647	29/18723	0.002894	0.017509	0.012267	NRP1/MAFB/GLI	5
GO	BP	GO:0033028	myeloid cell apoptotic process	5/647	29/18723	0.002894	0.017509	0.012267	CTSL/MEF2C/AN	5
GO	BP	GO:0098868	bone growth	5/647	29/18723	0.002894	0.017509	0.012267	DDR2/BNC2/EV(	5
GO	BP	GO:0002223	stimulatory C-type lectin receptor signalin	4/647	18/18723	0.002936	0.017585	0.01232	CLEC7A/FYN/KI	4
GO	BP	GO:0002283	neutrophil activation involved in immune	4/647	18/18723	0.002936	0.017585	0.01232	ITGAM/FCER1G/	4
GO	BP	GO:0002501	peptide antigen assembly with MHC prote	4/647	18/18723	0.002936	0.017585	0.01232	HLA-DPA1/HLA-	4
GO	BP	GO:0002923	regulation of humoral immune response m	4/647	18/18723	0.002936	0.017585	0.01232	FCGR2B/PTPRC/	4
GO	BP	GO:0030206	chondroitin sulfate biosynthetic process	4/647	18/18723	0.002936	0.017585	0.01232	DSE/CHST11/CH	4
GO	BP	GO:0030889	negative regulation of B cell proliferation	4/647	18/18723	0.002936	0.017585	0.01232	MNDA/FCGR2B/	4
GO	BP	GO:1990840	response to lectin	4/647	18/18723	0.002936	0.017585	0.01232	CLEC7A/FYN/KI	4
GO	BP	GO:1990858	cellular response to lectin	4/647	18/18723	0.002936	0.017585	0.01232	CLEC7A/FYN/KI	4
GO	BP	GO:0007409	axonogenesis	26/647	418/18723	0.002953	0.017662	0.012374	NRP1/PLXNC1/C	26
GO	BP	GO:0010594	regulation of endothelial cell migration	17/647	232/18723	0.002993	0.017878	0.012526	NRP1/PTPRM/VE	17
GO	BP	GO:0048872	homeostasis of number of cells	19/647	272/18723	0.003005	0.017905	0.012545	MAFB/RASSF2/D	19
GO	BP	GO:0051962	positive regulation of nervous system deve	19/647	272/18723	0.003005	0.017905	0.012545	NRP1/PLXNC1/T	19
GO	BP	GO:0001764	neuron migration	13/647	156/18723	0.003018	0.017942	0.012571	NRP1/FYN/SPOC	13
GO	BP	GO:0007520	myoblast fusion	6/647	42/18723	0.003024	0.017942	0.012571	ADAM12/PLEKH	6
GO	BP	GO:0001912	positive regulation of leukocyte mediated	7/647	56/18723	0.003038	0.017942	0.012571	ITGAM/PTPRC/K	7
GO	BP	GO:0022029	telencephalon cell migration	7/647	56/18723	0.003038	0.017942	0.012571	GLI3/CXCR4/LRI	7
GO	BP	GO:0030166	proteoglycan biosynthetic process	7/647	56/18723	0.003038	0.017942	0.012571	DSE/CHST11/CH	7
GO	BP	GO:0045599	negative regulation of fat cell differentiati	7/647	56/18723	0.003038	0.017942	0.012571	ZFPM2/FERMT2/	7
GO	BP	GO:0046456	icosanoid biosynthetic process	7/647	56/18723	0.003038	0.017942	0.012571	ALOX5AP/AVPR	7
GO	BP	GO:0050818	regulation of coagulation	8/647	71/18723	0.003048	0.017953	0.012579	FAP/SERPING1/(	8
GO	BP	GO:0061515	myeloid cell development	8/647	71/18723	0.003048	0.017953	0.012579	FBN1/FLI1/TLR2.	8

GO	BP	GO:0045936	negative regulation of phosphate metaboli	27/647	441/18723	0.003082	0.018134	0.012705	RASSF2/HEG1/S/	27
GO	BP	GO:1900182	positive regulation of protein localization	9/647	87/18723	0.003096	0.018185	0.012741	FERMT2/FYN/HC	9
GO	BP	GO:0006869	lipid transport	25/647	398/18723	0.003099	0.018185	0.012741	P2RX7/ATP8B2//	25
GO	BP	GO:0010563	negative regulation of phosphorus metabo	27/647	442/18723	0.003179	0.018632	0.013054	RASSF2/HEG1/S/	27
GO	BP	GO:2000377	regulation of reactive oxygen species meta	13/647	157/18723	0.00319	0.018672	0.013082	ITGAM/CLEC7A/	13
GO	BP	GO:0048661	positive regulation of smooth muscle cell	10/647	104/18723	0.0032	0.018685	0.013092	GJA1/CALCRL/A	10
GO	BP	GO:0060419	heart growth	10/647	104/18723	0.0032	0.018685	0.013092	ZFPM2/HEG1/MI	10
GO	BP	GO:0070302	regulation of stress-activated protein kinas	15/647	195/18723	0.003261	0.01902	0.013326	CLEC7A/RASSF2	15
GO	BP	GO:0060560	developmental growth involved in morpho	17/647	234/18723	0.003268	0.019038	0.013338	NRP1/CXCR4/PO	17
GO	BP	GO:0055021	regulation of cardiac muscle tissue growth	8/647	72/18723	0.003327	0.019357	0.013562	ZFPM2/MEIS1/GJ	8
GO	BP	GO:0010232	vascular transport	9/647	88/18723	0.003345	0.019437	0.013618	SLC2A3/SLC1A3	9
GO	BP	GO:0060760	positive regulation of response to cytokine	7/647	57/18723	0.003362	0.019489	0.013654	IL1R1/TLR2/CXC	7
GO	BP	GO:0061005	cell differentiation involved in kidney dev	7/647	57/18723	0.003362	0.019489	0.013654	WWTR1/GLI3/MI	7
GO	BP	GO:0009072	aromatic amino acid family metabolic proc	5/647	30/18723	0.003375	0.01954	0.013691	KMO/KYNU/IL4I	5
GO	BP	GO:0032956	regulation of actin cytoskeleton organizati	23/647	358/18723	0.00338	0.019544	0.013693	NRP1/CCDC88A/	23
GO	BP	GO:0032231	regulation of actin filament bundle assemb	10/647	105/18723	0.00343	0.019808	0.013878	NRP1/CCDC88A/	10
GO	BP	GO:0090092	regulation of transmembrane receptor prot	18/647	256/18723	0.003563	0.020532	0.014385	ZEB2/CHST11/FE	18
GO	BP	GO:0048762	mesenchymal cell differentiation	17/647	236/18723	0.003564	0.020532	0.014385	NRP1/SNAI2/FEF	17
GO	BP	GO:0010811	positive regulation of cell-substrate adhesi	11/647	123/18723	0.00359	0.020594	0.014429	NRP1/FERMT2/C	11
GO	BP	GO:0002281	macrophage activation involved in immun	4/647	19/18723	0.003619	0.020594	0.014429	HAVCR2/SUCNR	4
GO	BP	GO:0002396	MHC protein complex assembly	4/647	19/18723	0.003619	0.020594	0.014429	HLA-DPA1/HLA-	4
GO	BP	GO:0010759	positive regulation of macrophage chemot	4/647	19/18723	0.003619	0.020594	0.014429	C3AR1/C5AR1/C	4
GO	BP	GO:0032695	negative regulation of interleukin-12 prod	4/647	19/18723	0.003619	0.020594	0.014429	TLR8/IRAK3/LIL	4
GO	BP	GO:0033194	response to hydroperoxide	4/647	19/18723	0.003619	0.020594	0.014429	PRKD1/CD36/DA	4
GO	BP	GO:0044546	NLRP3 inflammasome complex assembly	4/647	19/18723	0.003619	0.020594	0.014429	GBP5/CD36/TREI	4
GO	BP	GO:0046794	transport of virus	4/647	19/18723	0.003619	0.020594	0.014429	CTSL/SIGLEC1/C	4
GO	BP	GO:1903975	regulation of glial cell migration	4/647	19/18723	0.003619	0.020594	0.014429	GPR183/TIAM1/T	4
GO	BP	GO:2001185	regulation of CD8-positive, alpha-beta T c	4/647	19/18723	0.003619	0.020594	0.014429	NCKAP1L/LILRE	4
GO	BP	GO:0035821	modulation of process of other organism	10/647	106/18723	0.003674	0.020854	0.014611	P2RX7/CLEC7A/	10
GO	BP	GO:0090263	positive regulation of canonical Wnt signa	10/647	106/18723	0.003674	0.020854	0.014611	ZEB2/LRRK2/CA	10
GO	BP	GO:0032387	negative regulation of intracellular transpo	7/647	58/18723	0.003712	0.021049	0.014748	MDFIC/LRRK2/M	7
GO	BP	GO:0010720	positive regulation of cell development	20/647	298/18723	0.003733	0.021139	0.01481	NRP1/PLXNC1/C	20
GO	BP	GO:0007416	synapse assembly	14/647	179/18723	0.003793	0.021456	0.015033	GPC6/TLR2/PCD	14
GO	BP	GO:0010876	lipid localization	27/647	448/18723	0.003814	0.021549	0.015098	P2RX7/ATP8B2//	27
GO	BP	GO:0099175	regulation of postsynapse organization	9/647	90/18723	0.003892	0.021961	0.015386	FCGR2B/SRGN/F	9
GO	BP	GO:0001516	prostaglandin biosynthetic process	5/647	31/18723	0.003912	0.021995	0.01541	AVPR1A/ANXA1	5
GO	BP	GO:0008045	motor neuron axon guidance	5/647	31/18723	0.003912	0.021995	0.01541	NRP1/CHN1/SLIT	5
GO	BP	GO:0046457	prostanoid biosynthetic process	5/647	31/18723	0.003912	0.021995	0.01541	AVPR1A/ANXA1	5

GO	BP	GO:0097191	extrinsic apoptotic signaling pathway	16/647	219/18723	0.004026	0.022611	0.015842	NRP1/P2RX7/SN.	16
GO	BP	GO:0042326	negative regulation of phosphorylation	24/647	385/18723	0.00408	0.022723	0.01592	RASSF2/HEG1/S.	24
GO	BP	GO:0021885	forebrain cell migration	7/647	59/18723	0.00409	0.022723	0.01592	GLI3/CXCR4/LRI	7
GO	BP	GO:0043407	negative regulation of MAP kinase activity	7/647	59/18723	0.00409	0.022723	0.01592	RGS2/CAV1/SFR	7
GO	BP	GO:0043551	regulation of phosphatidylinositol 3-kinase	7/647	59/18723	0.00409	0.022723	0.01592	FGR/PRKD1/FGF	7
GO	BP	GO:0048010	vascular endothelial growth factor recepto	7/647	59/18723	0.00409	0.022723	0.01592	NRP1/SULF1/VE(	7
GO	BP	GO:0050732	negative regulation of peptidyl-tyrosine ph	7/647	59/18723	0.00409	0.022723	0.01592	SAMSN1/PTPRC/	7
GO	BP	GO:2001258	negative regulation of cation channel activ	7/647	59/18723	0.00409	0.022723	0.01592	PKD2/KCNE4/CL	7
GO	BP	GO:0002524	hypersensitivity	3/647	10/18723	0.004109	0.022723	0.01592	FCGR2B/BTK/C3	3
GO	BP	GO:0002765	immune response-inhibiting signal transdu	3/647	10/18723	0.004109	0.022723	0.01592	LILRB2/LILRB1/	3
GO	BP	GO:0002887	negative regulation of myeloid leukocyte r	3/647	10/18723	0.004109	0.022723	0.01592	FCGR2B/CD84/C	3
GO	BP	GO:0033089	positive regulation of T cell differentiation	3/647	10/18723	0.004109	0.022723	0.01592	RASGRP1/IL7R/E	3
GO	BP	GO:0045625	regulation of T-helper 1 cell differentiation	3/647	10/18723	0.004109	0.022723	0.01592	TNFSF4/ANXA1/	3
GO	BP	GO:1904338	regulation of dopaminergic neuron differe	3/647	10/18723	0.004109	0.022723	0.01592	SFRP2/TIAM1/SF	3
GO	BP	GO:0097696	receptor signaling pathway via STAT	14/647	181/18723	0.004188	0.023088	0.016176	PKD2/IL10RA/FY	14
GO	BP	GO:1905952	regulation of lipid localization	14/647	181/18723	0.004188	0.023088	0.016176	MSR1/CAV1/CD3	14
GO	BP	GO:0051091	positive regulation of DNA-binding transc	18/647	260/18723	0.00419	0.023088	0.016176	CLEC7A/TLR2/D	18
GO	BP	GO:0006024	glycosaminoglycan biosynthetic process	8/647	75/18723	0.004287	0.023534	0.016489	DSE/CHST11/ST3	8
GO	BP	GO:0034121	regulation of toll-like receptor signaling pa	8/647	75/18723	0.004287	0.023534	0.016489	TLR1/TLR2/CD3(	8
GO	BP	GO:0106106	cold-induced thermogenesis	12/647	144/18723	0.004291	0.023534	0.016489	PDGFC/CXCR4/C	12
GO	BP	GO:0120161	regulation of cold-induced thermogenesis	12/647	144/18723	0.004291	0.023534	0.016489	PDGFC/CXCR4/C	12
GO	BP	GO:0071379	cellular response to prostaglandin stimulus	4/647	20/18723	0.004402	0.024083	0.016873	TNFSF4/PTGFR/T	4
GO	BP	GO:0140632	inflammasome complex assembly	4/647	20/18723	0.004402	0.024083	0.016873	GBP5/CD36/TREI	4
GO	BP	GO:0002712	regulation of B cell mediated immunity	7/647	60/18723	0.004495	0.024375	0.017078	FCGR2B/PTPRC/	7
GO	BP	GO:0002889	regulation of immunoglobulin mediated in	7/647	60/18723	0.004495	0.024375	0.017078	FCGR2B/PTPRC/	7
GO	BP	GO:0042306	regulation of protein import into nucleus	7/647	60/18723	0.004495	0.024375	0.017078	MDFIC/HCLS1/G	7
GO	BP	GO:0035249	synaptic transmission, glutamatergic	9/647	92/18723	0.004506	0.024375	0.017078	TSHZ3/CCL2/LRI	9
GO	BP	GO:0003180	aortic valve morphogenesis	5/647	32/18723	0.004507	0.024375	0.017078	SNAI2/SLIT2/TW	5
GO	BP	GO:0019835	cytolysis	5/647	32/18723	0.004507	0.024375	0.017078	CR1/C7/GZMA/L	5
GO	BP	GO:0033687	osteoblast proliferation	5/647	32/18723	0.004507	0.024375	0.017078	CTHRC1/GREM1	5
GO	BP	GO:0035767	endothelial cell chemotaxis	5/647	32/18723	0.004507	0.024375	0.017078	NRP1/PRKD1/FG	5
GO	BP	GO:0046475	glycerophospholipid catabolic process	5/647	32/18723	0.004507	0.024375	0.017078	PLA2G7/ENPP2/A	5
GO	BP	GO:0072525	pyridine-containing compound biosyntheti	5/647	32/18723	0.004507	0.024375	0.017078	KMO/KYNU/PTG	5
GO	BP	GO:0050806	positive regulation of synaptic transmissio	13/647	164/18723	0.004625	0.024986	0.017506	TSHZ3/SLC1A3/I	13
GO	BP	GO:0006809	nitric oxide biosynthetic process	8/647	76/18723	0.00465	0.02509	0.017579	CLEC7A/PKD2/C	8
GO	BP	GO:0048259	regulation of receptor-mediated endocytos	10/647	110/18723	0.004787	0.025798	0.018075	SGIP1/GREM1/C	10
GO	BP	GO:1902807	negative regulation of cell cycle G1/S pha	9/647	93/18723	0.00484	0.026058	0.018257	SLFN11/PKD2/GI	9
GO	BP	GO:0071772	response to BMP	13/647	165/18723	0.004867	0.026143	0.018317	FBN1/SULF1/SFI	13

GO	BP	GO:0071773	cellular response to BMP stimulus	13/647	165/18723	0.004867	0.026143	0.018317	FBN1/SULF1/SFI	13
GO	BP	GO:0034113	heterotypic cell-cell adhesion	7/647	61/18723	0.00493	0.026418	0.018509	VCAM1/PTPRC/I	7
GO	BP	GO:0060998	regulation of dendritic spine development	7/647	61/18723	0.00493	0.026418	0.018509	MEF2C/PLK2/SR	7
GO	BP	GO:1901888	regulation of cell junction assembly	15/647	204/18723	0.004961	0.026555	0.018606	NRP1/SNAI2/GP(	15
GO	BP	GO:0055117	regulation of cardiac muscle contraction	8/647	77/18723	0.005036	0.026925	0.018865	CLIC2/PIK3CG/R	8
GO	BP	GO:1903531	negative regulation of secretion by cell	12/647	147/18723	0.005059	0.027016	0.018928	FCGR2B/CD84/N	12
GO	BP	GO:0002040	sprouting angiogenesis	14/647	185/18723	0.005077	0.027055	0.018955	NRP1/VEGFC/MI	14
GO	BP	GO:0043433	negative regulation of DNA-binding transc	14/647	185/18723	0.005077	0.027055	0.018955	PKD2/HAVCR2/(	14
GO	BP	GO:0019362	pyridine nucleotide metabolic process	5/647	33/18723	0.005163	0.027389	0.019189	KMO/KYNU/PTC	5
GO	BP	GO:0045920	negative regulation of exocytosis	5/647	33/18723	0.005163	0.027389	0.019189	FCGR2B/CD84/N	5
GO	BP	GO:0046496	nicotinamide nucleotide metabolic process	5/647	33/18723	0.005163	0.027389	0.019189	KMO/KYNU/PTC	5
GO	BP	GO:0071295	cellular response to vitamin	5/647	33/18723	0.005163	0.027389	0.019189	SNAI2/POSTN/TI	5
GO	BP	GO:0001666	response to hypoxia	20/647	307/18723	0.005185	0.02747	0.019246	VCAM1/TLR2/VI	20
GO	BP	GO:0051702	biological process involved in interaction	9/647	94/18723	0.005193	0.027485	0.019257	TREM1/CCL8/CC	9
GO	BP	GO:0001654	eye development	23/647	371/18723	0.0052	0.02749	0.01926	NRP1/MITF/FBN	23
GO	BP	GO:0050769	positive regulation of neurogenesis	16/647	225/18723	0.005217	0.027549	0.019302	NRP1/PLXNC1/T	16
GO	BP	GO:0002689	negative regulation of leukocyte chemotax	4/647	21/18723	0.005291	0.027687	0.019399	SLAMF8/CCL2/S	4
GO	BP	GO:0006925	inflammatory cell apoptotic process	4/647	21/18723	0.005291	0.027687	0.019399	CTSL/MEF2C/AN	4
GO	BP	GO:0051767	nitric-oxide synthase biosynthetic process	4/647	21/18723	0.005291	0.027687	0.019399	TLR2/CCL2/LRR	4
GO	BP	GO:0051769	regulation of nitric-oxide synthase biosynt	4/647	21/18723	0.005291	0.027687	0.019399	TLR2/CCL2/LRR	4
GO	BP	GO:0071498	cellular response to fluid shear stress	4/647	21/18723	0.005291	0.027687	0.019399	PKD2/MEF2C/PT	4
GO	BP	GO:0071605	monocyte chemotactic protein-1 productio	4/647	21/18723	0.005291	0.027687	0.019399	CLEC7A/CD84/T	4
GO	BP	GO:0071637	regulation of monocyte chemotactic protei	4/647	21/18723	0.005291	0.027687	0.019399	CLEC7A/CD84/T	4
GO	BP	GO:0097062	dendritic spine maintenance	4/647	21/18723	0.005291	0.027687	0.019399	FCGR2B/FYN/TF	4
GO	BP	GO:0043123	positive regulation of I-kappaB kinase/NF	14/647	186/18723	0.005322	0.027821	0.019492	CLEC7A/FYN/GJ	14
GO	BP	GO:0010212	response to ionizing radiation	12/647	148/18723	0.005338	0.027861	0.01952	VCAM1/TSPYL5/	12
GO	BP	GO:0048483	autonomic nervous system development	6/647	47/18723	0.005354	0.027861	0.01952	NRP1/VCAM1/E(	6
GO	BP	GO:0050798	activated T cell proliferation	6/647	47/18723	0.005354	0.027861	0.01952	FYN/PDCD1LG2/	6
GO	BP	GO:0090279	regulation of calcium ion import	6/647	47/18723	0.005354	0.027861	0.01952	PKD2/FYN/CCL2	6
GO	BP	GO:0045862	positive regulation of proteolysis	23/647	372/18723	0.005369	0.027908	0.019554	CLEC7A/FYN/PR	23
GO	BP	GO:0031960	response to corticosteroid	13/647	167/18723	0.005382	0.027945	0.019579	NR3C1/SERPINF	13
GO	BP	GO:0003007	heart morphogenesis	17/647	246/18723	0.005395	0.027956	0.019587	NRP1/PKD2/SNA	17
GO	BP	GO:0045428	regulation of nitric oxide biosynthetic pro(	7/647	62/18723	0.005396	0.027956	0.019587	CLEC7A/PKD2/C	7
GO	BP	GO:0008016	regulation of heart contraction	15/647	206/18723	0.005423	0.028064	0.019663	CACNA2D1/KCN	15
GO	BP	GO:0010633	negative regulation of epithelial cell migra	10/647	112/18723	0.005435	0.028065	0.019663	MCC/PTPRM/DC	10
GO	BP	GO:0042476	odontogenesis	11/647	130/18723	0.00546	0.028065	0.019663	GLI3/FAM20A/IN	11
GO	BP	GO:0001867	complement activation, lectin pathway	3/647	11/18723	0.005506	0.028065	0.019663	SERPING1/FCN1	3
GO	BP	GO:0002645	positive regulation of tolerance induction	3/647	11/18723	0.005506	0.028065	0.019663	LILRB2/IRAK3/L	3

GO	BP	GO:0021561	facial nerve development	3/647	11/18723	0.005506	0.028065	0.019663	NRP1/EGR2/HOX	3
GO	BP	GO:0021604	cranial nerve structural organization	3/647	11/18723	0.005506	0.028065	0.019663	NRP1/EGR2/HOX	3
GO	BP	GO:0021610	facial nerve morphogenesis	3/647	11/18723	0.005506	0.028065	0.019663	NRP1/EGR2/HOX	3
GO	BP	GO:0033625	positive regulation of integrin activation	3/647	11/18723	0.005506	0.028065	0.019663	FERMT2/PLEK/C	3
GO	BP	GO:0034350	regulation of glial cell apoptotic process	3/647	11/18723	0.005506	0.028065	0.019663	CCL2/AKAP12/T	3
GO	BP	GO:0045060	negative thymic T cell selection	3/647	11/18723	0.005506	0.028065	0.019663	DOCK2/PTPRC/C	3
GO	BP	GO:0045628	regulation of T-helper 2 cell differentiatio	3/647	11/18723	0.005506	0.028065	0.019663	CD86/TNFSF4/AI	3
GO	BP	GO:0060315	negative regulation of ryanodine-sensitive	3/647	11/18723	0.005506	0.028065	0.019663	PKD2/CLIC2/CAS	3
GO	BP	GO:0097104	postsynaptic membrane assembly	3/647	11/18723	0.005506	0.028065	0.019663	NLGN4X/CDH2/(	3
GO	BP	GO:1903238	positive regulation of leukocyte tethering	3/647	11/18723	0.005506	0.028065	0.019663	ITGA4/CCR2/SEI	3
GO	BP	GO:1902806	regulation of cell cycle G1/S phase transit	13/647	168/18723	0.005655	0.028789	0.020171	SLFN11/PKD2/GI	13
GO	BP	GO:0007411	axon guidance	16/647	227/18723	0.005672	0.028817	0.02019	NRP1/PLXNC1/P	16
GO	BP	GO:0098657	import into cell	16/647	227/18723	0.005672	0.028817	0.02019	IL10RA/SLC2A3/	16
GO	BP	GO:0051047	positive regulation of secretion	20/647	310/18723	0.005761	0.029237	0.020484	ITGAM/TLR2/VE	20
GO	BP	GO:0050853	B cell receptor signaling pathway	11/647	131/18723	0.00578	0.029284	0.020517	MNDA/FCGR2B/	11
GO	BP	GO:0044106	cellular amine metabolic process	10/647	113/18723	0.005783	0.029284	0.020517	ITGAM/ITGB2/R	10
GO	BP	GO:0030308	negative regulation of cell growth	14/647	188/18723	0.00584	0.029481	0.020655	NRP1/GJA1/INHI	14
GO	BP	GO:0006023	aminoglycan biosynthetic process	8/647	79/18723	0.005881	0.029481	0.020655	DSE/CHST11/ST	8
GO	BP	GO:0050772	positive regulation of axonogenesis	8/647	79/18723	0.005881	0.029481	0.020655	NRP1/PLXNC1/S	8
GO	BP	GO:0060420	regulation of heart growth	8/647	79/18723	0.005881	0.029481	0.020655	ZFPM2/MEIS1/G	8
GO	BP	GO:0002446	neutrophil mediated immunity	5/647	34/18723	0.005885	0.029481	0.020655	ITGAM/ITGB2/TI	5
GO	BP	GO:0035590	purinergic nucleotide receptor signaling pa	5/647	34/18723	0.005885	0.029481	0.020655	P2RX7/GPR34/P2	5
GO	BP	GO:0044319	wound healing, spreading of cells	5/647	34/18723	0.005885	0.029481	0.020655	FERMT2/PHLDB	5
GO	BP	GO:0055094	response to lipoprotein particle	5/647	34/18723	0.005885	0.029481	0.020655	FCER1G/ITGB2/(	5
GO	BP	GO:0090505	epiboly involved in wound healing	5/647	34/18723	0.005885	0.029481	0.020655	FERMT2/PHLDB	5
GO	BP	GO:0098751	bone cell development	5/647	34/18723	0.005885	0.029481	0.020655	FBN1/FLI1/MEIS	5
GO	BP	GO:1904589	regulation of protein import	7/647	63/18723	0.005894	0.029493	0.020664	MDFIC/HCLS1/G	7
GO	BP	GO:0097485	neuron projection guidance	16/647	228/18723	0.005912	0.029553	0.020706	NRP1/PLXNC1/P	16
GO	BP	GO:0043303	mast cell degranulation	6/647	48/18723	0.005944	0.029617	0.02075	CD84/PIK3CG/FC	6
GO	BP	GO:1903053	regulation of extracellular matrix organiza	6/647	48/18723	0.005944	0.029617	0.02075	FAP/PHLDB2/DD	6
GO	BP	GO:2001238	positive regulation of extrinsic apoptotic s	6/647	48/18723	0.005944	0.029617	0.02075	PTPRC/INHBA/C	6
GO	BP	GO:0043122	regulation of I-kappaB kinase/NF-kappaB	17/647	249/18723	0.006074	0.030232	0.021182	CLEC7A/FYN/GJ	17
GO	BP	GO:0045444	fat cell differentiation	16/647	229/18723	0.006159	0.030627	0.021459	MAFB/ZFPM2/FE	16
GO	BP	GO:0007259	receptor signaling pathway via JAK-STAT	13/647	170/18723	0.006234	0.030965	0.021695	PKD2/IL10RA/FY	13
GO	BP	GO:0035584	calcium-mediated signaling using intracell	4/647	22/18723	0.006292	0.031156	0.021829	VCAM1/PTPRC/I	4
GO	BP	GO:0045624	positive regulation of T-helper cell differe	4/647	22/18723	0.006292	0.031156	0.021829	CD86/TNFSF4/AI	4
GO	BP	GO:1900120	regulation of receptor binding	4/647	22/18723	0.006292	0.031156	0.021829	NRP1/LOX/MMP	4
GO	BP	GO:0010596	negative regulation of endothelial cell mig	9/647	97/18723	0.006373	0.03149	0.022063	PTPRM/DCN/SEI	9

GO	BP	GO:0120162	positive regulation of cold-induced thermo	9/647	97/18723	0.006373	0.03149	0.022063	PDGFC/CXCR4/C	9
GO	BP	GO:0045055	regulated exocytosis	16/647	230/18723	0.006415	0.031665	0.022185	ITGAM/RAB31/F	16
GO	BP	GO:0080164	regulation of nitric oxide metabolic proces	7/647	64/18723	0.006425	0.03168	0.022196	CLEC7A/PKD2/C	7
GO	BP	GO:0051048	negative regulation of secretion	13/647	171/18723	0.006541	0.032171	0.02254	FCGR2B/CD84/N	13
GO	BP	GO:0061564	axon development	27/647	467/18723	0.006575	0.032171	0.02254	NRP1/PLXNC1/C	27
GO	BP	GO:0030509	BMP signaling pathway	12/647	152/18723	0.006577	0.032171	0.02254	FBN1/SULF1/SFI	12
GO	BP	GO:0002279	mast cell activation involved in immune re	6/647	49/18723	0.00658	0.032171	0.02254	CD84/PIK3CG/FC	6
GO	BP	GO:0008038	neuron recognition	6/647	49/18723	0.00658	0.032171	0.02254	NRP1/CXCR4/NE	6
GO	BP	GO:0030195	negative regulation of blood coagulation	6/647	49/18723	0.00658	0.032171	0.02254	FAP/SERPING1/T	6
GO	BP	GO:0050832	defense response to fungus	6/647	49/18723	0.00658	0.032171	0.02254	CLEC7A/CLEC4E	6
GO	BP	GO:0052372	modulation by symbiont of entry into host	6/647	49/18723	0.00658	0.032171	0.02254	TRIM22/CAV1/F(	6
GO	BP	GO:0034405	response to fluid shear stress	5/647	35/18723	0.006674	0.032465	0.022746	PKD2/GJA1/MEF	5
GO	BP	GO:0035633	maintenance of blood-brain barrier	5/647	35/18723	0.006674	0.032465	0.022746	GJA1/LAMA2/JA	5
GO	BP	GO:0045907	positive regulation of vasoconstriction	5/647	35/18723	0.006674	0.032465	0.022746	GJA1/AVPR1A/C	5
GO	BP	GO:0090050	positive regulation of cell migration involv	5/647	35/18723	0.006674	0.032465	0.022746	NRP1/PLK2/ANX	5
GO	BP	GO:0090504	epiboly	5/647	35/18723	0.006674	0.032465	0.022746	FERMT2/PHLDB	5
GO	BP	GO:0090257	regulation of muscle system process	17/647	252/18723	0.00682	0.033139	0.023218	DOCK4/CLIC2/PI	17
GO	BP	GO:0006979	response to oxidative stress	26/647	446/18723	0.006828	0.033144	0.023222	PKD2/GPX8/FYN	26
GO	BP	GO:1990748	cellular detoxification	10/647	116/18723	0.006934	0.033626	0.023559	GPX8/CLIC2/AL(	10
GO	BP	GO:0062197	cellular response to chemical stress	21/647	337/18723	0.006973	0.033775	0.023664	PKD2/GPX8/FYN	21
GO	BP	GO:0015909	long-chain fatty acid transport	7/647	65/18723	0.006992	0.033833	0.023704	CD36/ANXA1/AF	7
GO	BP	GO:0060537	muscle tissue development	24/647	403/18723	0.007103	0.034061	0.023864	PKD2/ZFPM2/HE	24
GO	BP	GO:0002664	regulation of T cell tolerance induction	3/647	12/18723	0.007155	0.034061	0.023864	LILRB2/LILRB4/	3
GO	BP	GO:0031953	negative regulation of protein autophospho	3/647	12/18723	0.007155	0.034061	0.023864	PTPRC/CAV1/AD	3
GO	BP	GO:0032621	interleukin-18 production	3/647	12/18723	0.007155	0.034061	0.023864	TLR2/CD84/GBP	3
GO	BP	GO:0032661	regulation of interleukin-18 production	3/647	12/18723	0.007155	0.034061	0.023864	TLR2/CD84/GBP	3
GO	BP	GO:0033004	negative regulation of mast cell activation	3/647	12/18723	0.007155	0.034061	0.023864	CD84/CD300LF/M	3
GO	BP	GO:0033629	negative regulation of cell adhesion media	3/647	12/18723	0.007155	0.034061	0.023864	SNAI2/CYP1B1/S	3
GO	BP	GO:0034154	toll-like receptor 7 signaling pathway	3/647	12/18723	0.007155	0.034061	0.023864	HAVCR2/TLR7/F	3
GO	BP	GO:0043380	regulation of memory T cell differentiation	3/647	12/18723	0.007155	0.034061	0.023864	TNFSF4/FGL2/HI	3
GO	BP	GO:0045916	negative regulation of complement activat	3/647	12/18723	0.007155	0.034061	0.023864	CR1/SERPING1/V	3
GO	BP	GO:0060907	positive regulation of macrophage cytokin	3/647	12/18723	0.007155	0.034061	0.023864	LAPTM5/LILRB1	3
GO	BP	GO:0071635	negative regulation of transforming growtl	3/647	12/18723	0.007155	0.034061	0.023864	TYROBP/LILRB1	3
GO	BP	GO:0090177	establishment of planar polarity involved i	3/647	12/18723	0.007155	0.034061	0.023864	CTHRC1/SFRP2/	3
GO	BP	GO:1902563	regulation of neutrophil activation	3/647	12/18723	0.007155	0.034061	0.023864	ITGAM/FCGR2B/	3
GO	BP	GO:1902950	regulation of dendritic spine maintenance	3/647	12/18723	0.007155	0.034061	0.023864	FCGR2B/FYN/AF	3
GO	BP	GO:2001204	regulation of osteoclast development	3/647	12/18723	0.007155	0.034061	0.023864	FBN1/TYROBP/L	3
GO	BP	GO:0001961	positive regulation of cytokine-mediated s	6/647	50/18723	0.007264	0.034351	0.024067	IL1R1/CXCR4/LA	6

GO	BP	GO:0002448	mast cell mediated immunity	6/647	50/18723	0.007264	0.034351	0.024067	CD84/PIK3CG/FC	6
GO	BP	GO:0045058	T cell selection	6/647	50/18723	0.007264	0.034351	0.024067	DOCK2/PTPRC/C	6
GO	BP	GO:0070231	T cell apoptotic process	6/647	50/18723	0.007264	0.034351	0.024067	DOCK8/GLI3/IL7	6
GO	BP	GO:0010717	regulation of epithelial to mesenchymal tra	9/647	99/18723	0.007267	0.034351	0.024067	FERMT2/PHLDB	9
GO	BP	GO:0015837	amine transport	9/647	99/18723	0.007267	0.034351	0.024067	AQP9/RGS2/AVP	9
GO	BP	GO:0060996	dendritic spine development	9/647	99/18723	0.007267	0.034351	0.024067	DOCK10/LRRK2/	9
GO	BP	GO:0002312	B cell activation involved in immune respo	8/647	82/18723	0.007346	0.03469	0.024305	DOCK10/FCGR2I	8
GO	BP	GO:0002702	positive regulation of production of molec	10/647	117/18723	0.007355	0.034697	0.02431	CLEC7A/IL1R1/C	10
GO	BP	GO:0034695	response to prostaglandin E	4/647	23/18723	0.007412	0.03479	0.024375	TNFSF4/SFRP1/C	4
GO	BP	GO:0050765	negative regulation of phagocytosis	4/647	23/18723	0.007412	0.03479	0.024375	FCGR2B/TLR2/C	4
GO	BP	GO:0060384	innervation	4/647	23/18723	0.007412	0.03479	0.024375	NRP1/VCAM1/SU	4
GO	BP	GO:0071305	cellular response to vitamin D	4/647	23/18723	0.007412	0.03479	0.024375	SNAI2/TNC/MN1	4
GO	BP	GO:1903306	negative regulation of regulated secretory	4/647	23/18723	0.007412	0.03479	0.024375	FCGR2B/CD84/N	4
GO	BP	GO:0007568	aging	21/647	339/18723	0.007437	0.034839	0.024409	VCAM1/NOX4/T.	21
GO	BP	GO:0048545	response to steroid hormone	21/647	339/18723	0.007437	0.034839	0.024409	NR3C1/LOX/TLR	21
GO	BP	GO:0048588	developmental cell growth	16/647	234/18723	0.007526	0.035122	0.024608	NRP1/CXCR4/PO	16
GO	BP	GO:0001659	temperature homeostasis	13/647	174/18723	0.007534	0.035122	0.024608	PDGFC/CXCR4/C	13
GO	BP	GO:0046466	membrane lipid catabolic process	5/647	36/18723	0.007535	0.035122	0.024608	CYP1B1/SPHK1/I	5
GO	BP	GO:0051955	regulation of amino acid transport	5/647	36/18723	0.007535	0.035122	0.024608	RGS2/AVPR1A/K	5
GO	BP	GO:0071402	cellular response to lipoprotein particle sti	5/647	36/18723	0.007535	0.035122	0.024608	FCER1G/ITGB2/C	5
GO	BP	GO:0042093	T-helper cell differentiation	7/647	66/18723	0.007594	0.035327	0.024752	CD86/TNFSF4/GI	7
GO	BP	GO:0050922	negative regulation of chemotaxis	7/647	66/18723	0.007594	0.035327	0.024752	NRP1/SLAMF8/C	7
GO	BP	GO:0034614	cellular response to reactive oxygen specie	12/647	155/18723	0.007648	0.035543	0.024903	PKD2/CYP1B1/LI	12
GO	BP	GO:0014706	striated muscle tissue development	23/647	384/18723	0.007768	0.036028	0.025242	ZFPM2/HEG1/LC	23
GO	BP	GO:0042692	muscle cell differentiation	23/647	384/18723	0.007768	0.036028	0.025242	QKI/LOX/ADAM	23
GO	BP	GO:0022612	gland morphogenesis	10/647	118/18723	0.007796	0.03612	0.025307	NRP1/SULF1/GLI	10
GO	BP	GO:0048678	response to axon injury	8/647	83/18723	0.007891	0.036526	0.025591	DPYSL3/MAP1B/	8
GO	BP	GO:0006636	unsaturated fatty acid biosynthetic process	6/647	51/18723	0.007998	0.03691	0.025861	AVPR1A/ANXA1	6
GO	BP	GO:0043370	regulation of CD4-positive, alpha-beta T c	6/647	51/18723	0.007998	0.03691	0.025861	CD86/NCKAP1L/	6
GO	BP	GO:0050982	detection of mechanical stimulus	6/647	51/18723	0.007998	0.03691	0.025861	PKD2/FYN/CXCI	6
GO	BP	GO:0045471	response to ethanol	11/647	137/18723	0.008024	0.036996	0.02592	VCAM1/FYN/CD	11
GO	BP	GO:0048738	cardiac muscle tissue development	16/647	236/18723	0.008136	0.037477	0.026257	ZFPM2/HEG1/AL	16
GO	BP	GO:0032890	regulation of organic acid transport	7/647	67/18723	0.008235	0.037892	0.026548	RGS2/AVPR1A/K	7
GO	BP	GO:0001763	morphogenesis of a branching structure	14/647	196/18723	0.008328	0.038247	0.026797	NRP1/PKD2/SUL	14
GO	BP	GO:0031032	actomyosin structure organization	14/647	196/18723	0.008328	0.038247	0.026797	FRMD6/NRP1/CC	14
GO	BP	GO:0001837	epithelial to mesenchymal transition	12/647	157/18723	0.008435	0.038598	0.027043	SNAI2/FERMT2/I	12
GO	BP	GO:1990845	adaptive thermogenesis	12/647	157/18723	0.008435	0.038598	0.027043	PDGFC/CXCR4/C	12
GO	BP	GO:0032370	positive regulation of lipid transport	8/647	84/18723	0.008465	0.038598	0.027043	CAV1/TREM2/SI	8

GO	BP	GO:0097061	dendritic spine organization	8/647	84/18723	0.008465	0.038598	0.027043	DOCK10/FCGR2I	8
GO	BP	GO:2000134	negative regulation of G1/S transition of n	8/647	84/18723	0.008465	0.038598	0.027043	SLFN11/PKD2/GI	8
GO	BP	GO:0003176	aortic valve development	5/647	37/18723	0.00847	0.038598	0.027043	SNAI2/SLIT2/TW	5
GO	BP	GO:0043368	positive T cell selection	5/647	37/18723	0.00847	0.038598	0.027043	DOCK2/PTPRC/C	5
GO	BP	GO:0051954	positive regulation of amine transport	5/647	37/18723	0.00847	0.038598	0.027043	AVPR1A/KMO/C	5
GO	BP	GO:0045165	cell fate commitment	17/647	258/18723	0.008535	0.038855	0.027223	NRP1/PRRX1/MI	17
GO	BP	GO:0036293	response to decreased oxygen levels	20/647	322/18723	0.008619	0.039176	0.027448	VCAM1/TLR2/VI	20
GO	BP	GO:0002726	positive regulation of T cell cytokine prod	4/647	24/18723	0.008656	0.039176	0.027448	IL1R1/TNFSF4/R	4
GO	BP	GO:0016486	peptide hormone processing	4/647	24/18723	0.008656	0.039176	0.027448	CTSL/PCSK5/CP	4
GO	BP	GO:0071676	negative regulation of mononuclear cell m	4/647	24/18723	0.008656	0.039176	0.027448	SLAMF8/CCL2/S	4
GO	BP	GO:2000209	regulation of anoikis	4/647	24/18723	0.008656	0.039176	0.027448	SNAI2/CAV1/ITC	4
GO	BP	GO:2000310	regulation of NMDA receptor activity	4/647	24/18723	0.008656	0.039176	0.027448	CCL2/MEF2C/DA	4
GO	BP	GO:0010952	positive regulation of peptidase activity	14/647	197/18723	0.00869	0.039296	0.027532	CLEC7A/FYN/LA	14
GO	BP	GO:0021545	cranial nerve development	6/647	52/18723	0.008783	0.039601	0.027746	NRP1/MAFB/SLC	6
GO	BP	GO:0030514	negative regulation of BMP signaling path	6/647	52/18723	0.008783	0.039601	0.027746	FBN1/SFRP2/GRI	6
GO	BP	GO:0051496	positive regulation of stress fiber assembly	6/647	52/18723	0.008783	0.039601	0.027746	NRP1/CCDC88A/	6
GO	BP	GO:0002294	CD4-positive, alpha-beta T cell differentia	7/647	68/18723	0.008914	0.04015	0.02813	CD86/TNFSF4/GI	7
GO	BP	GO:0007179	transforming growth factor beta receptor s	14/647	198/18723	0.009065	0.040239	0.028193	ZEB2/CHST11/FI	14
GO	BP	GO:0010958	regulation of amino acid import across pla	3/647	13/18723	0.009065	0.040239	0.028193	RGS2/RGS4/ACE	3
GO	BP	GO:0031392	regulation of prostaglandin biosynthetic pr	3/647	13/18723	0.009065	0.040239	0.028193	AVPR1A/ANXA1	3
GO	BP	GO:0032252	secretory granule localization	3/647	13/18723	0.009065	0.040239	0.028193	PIK3CG/RASGRI	3
GO	BP	GO:0033689	negative regulation of osteoblast prolifera	3/647	13/18723	0.009065	0.040239	0.028193	GREM1/MN1/SFI	3
GO	BP	GO:0036005	response to macrophage colony-stimulatin	3/647	13/18723	0.009065	0.040239	0.028193	TLR2/CSF1R/TRI	3
GO	BP	GO:0036006	cellular response to macrophage colony-st	3/647	13/18723	0.009065	0.040239	0.028193	TLR2/CSF1R/TRI	3
GO	BP	GO:0042492	gamma-delta T cell differentiation	3/647	13/18723	0.009065	0.040239	0.028193	NCKAP1L/PTPR	3
GO	BP	GO:0043379	memory T cell differentiation	3/647	13/18723	0.009065	0.040239	0.028193	TNFSF4/FGL2/HI	3
GO	BP	GO:0051280	negative regulation of release of sequester	3/647	13/18723	0.009065	0.040239	0.028193	PKD2/CLIC2/CAS	3
GO	BP	GO:0051451	myoblast migration	3/647	13/18723	0.009065	0.040239	0.028193	PLEKHO1/ANXA	3
GO	BP	GO:1903789	regulation of amino acid transmembrane t	3/647	13/18723	0.009065	0.040239	0.028193	RGS2/RGS4/ACE	3
GO	BP	GO:1903977	positive regulation of glial cell migration	3/647	13/18723	0.009065	0.040239	0.028193	TIAM1/TREM2/C	3
GO	BP	GO:1905907	negative regulation of amyloid fibril forma	3/647	13/18723	0.009065	0.040239	0.028193	TREM2/APOE/CI	3
GO	BP	GO:2001198	regulation of dendritic cell differentiation	3/647	13/18723	0.009065	0.040239	0.028193	FCGR2B/LILRB2	3
GO	BP	GO:0097006	regulation of plasma lipoprotein particle le	8/647	85/18723	0.009071	0.040239	0.028193	MSR1/PLA2G7/C	8
GO	BP	GO:1903532	positive regulation of secretion by cell	18/647	282/18723	0.009468	0.041948	0.02939	ITGAM/TLR2/VE	18
GO	BP	GO:0030279	negative regulation of ossification	5/647	38/18723	0.009483	0.041948	0.02939	SRGN/CCR1/MEI	5
GO	BP	GO:0072524	pyridine-containing compound metabolic p	5/647	38/18723	0.009483	0.041948	0.02939	KMO/KYNU/PTC	5
GO	BP	GO:0070482	response to oxygen levels	21/647	347/18723	0.009556	0.042231	0.029588	VCAM1/TLR2/VI	21
GO	BP	GO:0007566	embryo implantation	6/647	53/18723	0.009623	0.042328	0.029657	GJA1/SPP1/PCSK	6

GO	BP	GO:0034381	plasma lipoprotein particle clearance	6/647	53/18723	0.009623	0.042328	0.029657	MSR1/CD36/TRE	6
GO	BP	GO:0045806	negative regulation of endocytosis	6/647	53/18723	0.009623	0.042328	0.029657	CAV1/PRKD1/LI	6
GO	BP	GO:0050819	negative regulation of coagulation	6/647	53/18723	0.009623	0.042328	0.029657	FAP/SERPING1/T	6
GO	BP	GO:0071320	cellular response to cAMP	6/647	53/18723	0.009623	0.042328	0.029657	PKD2/NOX4/PIK	6
GO	BP	GO:0015908	fatty acid transport	8/647	86/18723	0.009708	0.042548	0.029811	CD36/ANXA1/AI	8
GO	BP	GO:0032413	negative regulation of ion transmembrane	8/647	86/18723	0.009708	0.042548	0.029811	PKD2/KCNE4/CL	8
GO	BP	GO:0048864	stem cell development	8/647	86/18723	0.009708	0.042548	0.029811	NRP1/SNAI2/PTF	8
GO	BP	GO:0044706	multi-multicellular organism process	15/647	220/18723	0.009709	0.042548	0.029811	HAVCR2/GJA1/R	15
GO	BP	GO:0051146	striated muscle cell differentiation	18/647	283/18723	0.009797	0.042895	0.030053	ADAM12/ALPK2	18
GO	BP	GO:0044843	cell cycle G1/S phase transition	16/647	241/18723	0.009837	0.043026	0.030145	SLFN11/BCAT1/I	16
GO	BP	GO:0032355	response to estradiol	11/647	141/18723	0.009861	0.043092	0.030192	POSTN/GJA1/MA	11
GO	BP	GO:0010560	positive regulation of glycoprotein biosyn	4/647	25/18723	0.010028	0.043458	0.030448	IGF1/CCL21/RAN	4
GO	BP	GO:0030318	melanocyte differentiation	4/647	25/18723	0.010028	0.043458	0.030448	ZEB2/MITF/GLI3	4
GO	BP	GO:0032753	positive regulation of interleukin-4 produc	4/647	25/18723	0.010028	0.043458	0.030448	CD86/HAVCR2/F	4
GO	BP	GO:0042730	fibrinolysis	4/647	25/18723	0.010028	0.043458	0.030448	FAP/SERPING1/S	4
GO	BP	GO:0050927	positive regulation of positive chemotaxis	4/647	25/18723	0.010028	0.043458	0.030448	VEGFC/CXCL12/	4
GO	BP	GO:0060740	prostate gland epithelium morphogenesis	4/647	25/18723	0.010028	0.043458	0.030448	SULF1/TNC/CYP	4
GO	BP	GO:0140448	signaling receptor ligand precursor proces	4/647	25/18723	0.010028	0.043458	0.030448	CTSL/PCSK5/CP	4
GO	BP	GO:1903055	positive regulation of extracellular matrix	4/647	25/18723	0.010028	0.043458	0.030448	PHLDB2/DDR2/E	4
GO	BP	GO:1903792	negative regulation of anion transport	4/647	25/18723	0.010028	0.043458	0.030448	RGS2/RGS4/SFRI	4
GO	BP	GO:0030168	platelet activation	10/647	123/18723	0.010309	0.044576	0.031232	FCER1G/PIK3CG	10
GO	BP	GO:0050852	T cell receptor signaling pathway	10/647	123/18723	0.010309	0.044576	0.031232	LCP2/FYN/PTPR	10
GO	BP	GO:0030323	respiratory tube development	13/647	181/18723	0.010315	0.044576	0.031232	ZFPM2/HEG1/LC	13
GO	BP	GO:2000045	regulation of G1/S transition of mitotic ce	11/647	142/18723	0.010367	0.044723	0.031334	SLFN11/PKD2/GI	11
GO	BP	GO:0044070	regulation of anion transport	8/647	87/18723	0.010377	0.044723	0.031334	RGS2/AVPR1A/K	8
GO	BP	GO:0150104	transport across blood-brain barrier	8/647	87/18723	0.010377	0.044723	0.031334	SLC2A3/SLC1A3	8
GO	BP	GO:0002381	immunoglobulin production involved in in	7/647	70/18723	0.010394	0.044752	0.031355	PTPRC/BTK/TNF	7
GO	BP	GO:0000302	response to reactive oxygen species	15/647	222/18723	0.010495	0.045148	0.031632	PKD2/FYN/CYP1	15
GO	BP	GO:0062207	regulation of pattern recognition receptor	9/647	105/18723	0.01053	0.045258	0.031709	TLR1/TLR2/CD3	9
GO	BP	GO:0001941	postsynaptic membrane organization	5/647	39/18723	0.010575	0.045286	0.031729	NLGN4X/CDH2/	5
GO	BP	GO:0002347	response to tumor cell	5/647	39/18723	0.010575	0.045286	0.031729	HAVCR2/LAPTM	5
GO	BP	GO:0043267	negative regulation of potassium ion trans	5/647	39/18723	0.010575	0.045286	0.031729	KCNE4/CAV1/RC	5
GO	BP	GO:0045622	regulation of T-helper cell differentiation	5/647	39/18723	0.010575	0.045286	0.031729	CD86/TNFSF4/AI	5
GO	BP	GO:0051100	negative regulation of binding	12/647	162/18723	0.01068	0.04569	0.032012	LRRK2/ITGA4/C	12
GO	BP	GO:0097237	cellular response to toxic substance	10/647	124/18723	0.010878	0.046476	0.032562	GPX8/CLIC2/AL	10
GO	BP	GO:0034763	negative regulation of transmembrane tran	11/647	143/18723	0.010893	0.046476	0.032562	PKD2/KCNE4/CL	11
GO	BP	GO:0038061	NIK/NF-kappaB signaling	11/647	143/18723	0.010893	0.046476	0.032562	RASSF2/CD86/TI	11
GO	BP	GO:0050829	defense response to Gram-negative bacter	8/647	88/18723	0.01108	0.047233	0.033093	TREM1/PRKD1/N	8

GO	BP	GO:0043550	regulation of lipid kinase activity	7/647	71/18723	0.011197	0.047504	0.033283	FGR/PRKD1/FGF	7
GO	BP	GO:0002517	T cell tolerance induction	3/647	14/18723	0.011245	0.047504	0.033283	LILRB2/LILRB4/(	3
GO	BP	GO:0002864	regulation of acute inflammatory response	3/647	14/18723	0.011245	0.047504	0.033283	FCGR2B/BTK/C3	3
GO	BP	GO:0009214	cyclic nucleotide catabolic process	3/647	14/18723	0.011245	0.047504	0.033283	PDE1A/PDE4B/PI	3
GO	BP	GO:0010872	regulation of cholesterol esterification	3/647	14/18723	0.011245	0.047504	0.033283	APOE/APOC1/AC	3
GO	BP	GO:0044764	multi-organism cellular process	3/647	14/18723	0.011245	0.047504	0.033283	CTSL/SIGLEC1/C	3
GO	BP	GO:0050966	detection of mechanical stimulus involved	3/647	14/18723	0.011245	0.047504	0.033283	FYN/CXCR4/CX(	3
GO	BP	GO:0071481	cellular response to X-ray	3/647	14/18723	0.011245	0.047504	0.033283	SFRP2/SFRP1/PR	3
GO	BP	GO:0090715	immunological memory formation process	3/647	14/18723	0.011245	0.047504	0.033283	TNFSF4/FGL2/HI	3
GO	BP	GO:0097091	synaptic vesicle clustering	3/647	14/18723	0.011245	0.047504	0.033283	PCDH17/SYNDIG	3
GO	BP	GO:0007015	actin filament organization	25/647	442/18723	0.011269	0.04756	0.033322	MYO5A/NRP1/W	25
GO	BP	GO:0003179	heart valve morphogenesis	6/647	55/18723	0.011471	0.048369	0.033889	SNAI2/MEF2C/SI	6
GO	BP	GO:0010875	positive regulation of cholesterol efflux	4/647	26/18723	0.011534	0.048418	0.033923	CAV1/TREM2/AI	4
GO	BP	GO:0042537	benzene-containing compound metabolic p	4/647	26/18723	0.011534	0.048418	0.033923	KMO/KYNU/TD(	4
GO	BP	GO:0050926	regulation of positive chemotaxis	4/647	26/18723	0.011534	0.048418	0.033923	VEGFC/CXCL12/	4
GO	BP	GO:0060314	regulation of ryanodine-sensitive calcium-	4/647	26/18723	0.011534	0.048418	0.033923	PKD2/CLIC2/PLN	4
GO	BP	GO:2000108	positive regulation of leukocyte apoptotic	4/647	26/18723	0.011534	0.048418	0.033923	MEF2C/ANXA1/(	4
GO	BP	GO:0034599	cellular response to oxidative stress	18/647	288/18723	0.011584	0.048586	0.034041	PKD2/GPX8/FYN	18
GO	BP	GO:0032412	regulation of ion transmembrane transport	17/647	267/18723	0.011738	0.048935	0.034286	PKD2/CACNA2D	17
GO	BP	GO:0002714	positive regulation of B cell mediated imm	5/647	40/18723	0.011751	0.048935	0.034286	PTPRC/BTK/TNF	5
GO	BP	GO:0002891	positive regulation of immunoglobulin me	5/647	40/18723	0.011751	0.048935	0.034286	PTPRC/BTK/TNF	5
GO	BP	GO:0010543	regulation of platelet activation	5/647	40/18723	0.011751	0.048935	0.034286	FCER1G/PLEK/A	5
GO	BP	GO:0032892	positive regulation of organic acid transpo	5/647	40/18723	0.011751	0.048935	0.034286	AVPR1A/KMO/P	5
GO	BP	GO:0045429	positive regulation of nitric oxide biosynth	5/647	40/18723	0.011751	0.048935	0.034286	CLEC7A/PKD2/C	5
GO	BP	GO:0051281	positive regulation of release of sequestere	5/647	40/18723	0.011751	0.048935	0.034286	PKD2/TRPC1/CX	5
GO	BP	GO:1901020	negative regulation of calcium ion transme	5/647	40/18723	0.011751	0.048935	0.034286	PKD2/CLIC2/PLN	5
GO	BP	GO:0016485	protein processing	15/647	225/18723	0.011767	0.048957	0.034301	GAS1/SRGN/GLI	15
GO	BP	GO:0046330	positive regulation of JNK cascade	8/647	89/18723	0.011818	0.049093	0.034396	RASSF2/FCGR2E	8
GO	BP	GO:0031532	actin cytoskeleton reorganization	9/647	107/18723	0.011831	0.049093	0.034396	NRP1/GPR65/HC	9
GO	BP	GO:0051341	regulation of oxidoreductase activity	9/647	107/18723	0.011831	0.049093	0.034396	SLAMF8/LRRK2/	9
GO	BP	GO:0043903	regulation of biological process involved i	7/647	72/18723	0.012044	0.049847	0.034924	TRIM22/CAV1/F(	7
GO	BP	GO:0070227	lymphocyte apoptotic process	7/647	72/18723	0.012044	0.049847	0.034924	DOCK8/GLI3/BT	7
GO	BP	GO:0071479	cellular response to ionizing radiation	7/647	72/18723	0.012044	0.049847	0.034924	TSPYL5/NOX4/II	7
GO	BP	GO:0032874	positive regulation of stress-activated MA	10/647	126/18723	0.012088	0.049982	0.035019	CLEC7A/RASSF2	10
GO	BP	GO:0002711	positive regulation of T cell mediated imm	6/647	56/18723	0.012483	0.051524	0.036099	IL1R1/PTPRC/TN	6
GO	BP	GO:0010332	response to gamma radiation	6/647	56/18723	0.012483	0.051524	0.036099	TSPYL5/NOX4/P	6
GO	BP	GO:0061097	regulation of protein tyrosine kinase activi	8/647	90/18723	0.012591	0.051844	0.036323	NOX4/PTPRC/CS	8
GO	BP	GO:1904705	regulation of vascular associated smooth r	8/647	90/18723	0.012591	0.051844	0.036323	GJA1/MEF2C/AD	8

GO	BP	GO:0007189	adenylate cyclase-activating G protein-cou	11/647	146/18723	0.012593	0.051844	0.036323	GPR65/CALCRL/	11
GO	BP	GO:0030048	actin filament-based movement	10/647	127/18723	0.012729	0.052345	0.036674	MYO5A/WIPF1/C	10
GO	BP	GO:0048608	reproductive structure development	24/647	424/18723	0.012742	0.052345	0.036674	PKD2/ZFPM2/SU	24
GO	BP	GO:0031345	negative regulation of cell projection orga	13/647	186/18723	0.012748	0.052345	0.036674	NRP1/VIM/FYN/S	13
GO	BP	GO:0030307	positive regulation of cell growth	12/647	166/18723	0.012786	0.052454	0.036751	NRP1/CXCR4/AV	12
GO	BP	GO:0086003	cardiac muscle cell contraction	7/647	73/18723	0.012937	0.053027	0.037152	CACNA2D1/KCN	7
GO	BP	GO:0042307	positive regulation of protein import into r	5/647	41/18723	0.013013	0.05315	0.037239	HCLS1/GLI3/PRK	5
GO	BP	GO:0071634	regulation of transforming growth factor b	5/647	41/18723	0.013013	0.05315	0.037239	LUM/TYROBP/LI	5
GO	BP	GO:1904407	positive regulation of nitric oxide metabol	5/647	41/18723	0.013013	0.05315	0.037239	CLEC7A/PKD2/C	5
GO	BP	GO:1905314	semi-lunar valve development	5/647	41/18723	0.013013	0.05315	0.037239	SNAI2/SLIT2/TW	5
GO	BP	GO:0002719	negative regulation of cytokine productior	4/647	27/18723	0.013178	0.053684	0.037613	IRAK3/LILRB1/L	4
GO	BP	GO:0007263	nitric oxide mediated signal transduction	4/647	27/18723	0.013178	0.053684	0.037613	FPR1/CD36/APOI	4
GO	BP	GO:0060512	prostate gland morphogenesis	4/647	27/18723	0.013178	0.053684	0.037613	SULF1/TNC/CYP	4
GO	BP	GO:0032410	negative regulation of transporter activity	9/647	109/18723	0.013248	0.053923	0.03778	PKD2/KCNE4/CL	9
GO	BP	GO:0001952	regulation of cell-matrix adhesion	10/647	128/18723	0.013396	0.054358	0.038085	NRP1/FERMT2/P	10
GO	BP	GO:0070304	positive regulation of stress-activated prot	10/647	128/18723	0.013396	0.054358	0.038085	CLEC7A/RASSF2	10
GO	BP	GO:0014033	neural crest cell differentiation	8/647	91/18723	0.013401	0.054358	0.038085	NRP1/SNAI2/ME	8
GO	BP	GO:1990874	vascular associated smooth muscle cell pro	8/647	91/18723	0.013401	0.054358	0.038085	GJA1/MEF2C/AD	8
GO	BP	GO:0002011	morphogenesis of an epithelial sheet	6/647	57/18723	0.013556	0.054937	0.038491	FERMT2/PHLDB	6
GO	BP	GO:0007265	Ras protein signal transduction	20/647	337/18723	0.013697	0.055051	0.038571	NRP1/HEG1/MRA	20
GO	BP	GO:0023035	CD40 signaling pathway	3/647	15/18723	0.013701	0.055051	0.038571	CD86/ITGA5/TRI	3
GO	BP	GO:0042182	ketone catabolic process	3/647	15/18723	0.013701	0.055051	0.038571	KYNU/HSD17B6/	3
GO	BP	GO:0042249	establishment of planar polarity of embryo	3/647	15/18723	0.013701	0.055051	0.038571	CTHRC1/SFRP2/S	3
GO	BP	GO:0045064	T-helper 2 cell differentiation	3/647	15/18723	0.013701	0.055051	0.038571	CD86/TNFSF4/AI	3
GO	BP	GO:0046007	negative regulation of activated T cell pro	3/647	15/18723	0.013701	0.055051	0.038571	PDCD1LG2/CRT/	3
GO	BP	GO:0051284	positive regulation of sequestering of calci	3/647	15/18723	0.013701	0.055051	0.038571	PKD2/CLIC2/CAS	3
GO	BP	GO:1901841	regulation of high voltage-gated calcium c	3/647	15/18723	0.013701	0.055051	0.038571	CACNA2D1/PDE	3
GO	BP	GO:2000052	positive regulation of non-canonical Wnt s	3/647	15/18723	0.013701	0.055051	0.038571	RSPO3/ANKRD6/	3
GO	BP	GO:2001279	regulation of unsaturated fatty acid biosyn	3/647	15/18723	0.013701	0.055051	0.038571	AVPR1A/ANXA1	3
GO	BP	GO:0061458	reproductive system development	24/647	427/18723	0.013781	0.055323	0.038761	PKD2/ZFPM2/SU	24
GO	BP	GO:0014074	response to purine-containing compound	11/647	148/18723	0.013834	0.055491	0.038879	P2RX7/PKD2/NO	11
GO	BP	GO:0070509	calcium ion import	8/647	92/18723	0.014248	0.057103	0.040008	PKD2/CACNA2D	8
GO	BP	GO:0002763	positive regulation of myeloid leukocyte d	6/647	58/18723	0.014691	0.058828	0.041217	CCR1/HCLS1/EV	6
GO	BP	GO:0033077	T cell differentiation in thymus	7/647	75/18723	0.014864	0.059417	0.04163	MAFB/DOCK2/PT	7
GO	BP	GO:0048662	negative regulation of smooth muscle cell	7/647	75/18723	0.014864	0.059417	0.04163	MEF2C/APOE/NI	7
GO	BP	GO:0032607	interferon-alpha production	4/647	28/18723	0.014963	0.059492	0.041682	TLR8/HAVCR2/T	4
GO	BP	GO:0032647	regulation of interferon-alpha production	4/647	28/18723	0.014963	0.059492	0.041682	TLR8/HAVCR2/T	4
GO	BP	GO:0034123	positive regulation of toll-like receptor sig	4/647	28/18723	0.014963	0.059492	0.041682	TLR1/TLR2/CAV	4

GO	BP	GO:0061082	myeloid leukocyte cytokine production	4/647	28/18723	0.014963	0.059492	0.041682	LAPTM5/IRAK3/	4
GO	BP	GO:0086011	membrane repolarization during action po	4/647	28/18723	0.014963	0.059492	0.041682	CACNA2D1/KCN	4
GO	BP	GO:1903020	positive regulation of glycoprotein metabo	4/647	28/18723	0.014963	0.059492	0.041682	IGF1/CCL21/RAM	4
GO	BP	GO:0050890	cognition	18/647	296/18723	0.014971	0.059492	0.041682	TLR2/FYN/LILRI	18
GO	BP	GO:0019217	regulation of fatty acid metabolic process	8/647	93/18723	0.015134	0.059985	0.042027	AVPR1A/CAV1/T	8
GO	BP	GO:0051591	response to cAMP	8/647	93/18723	0.015134	0.059985	0.042027	PKD2/NOX4/PIK	8
GO	BP	GO:0106027	neuron projection organization	8/647	93/18723	0.015134	0.059985	0.042027	DOCK10/FCGR2I	8
GO	BP	GO:0042180	cellular ketone metabolic process	14/647	211/18723	0.015192	0.060164	0.042153	AVPR1A/CAV1/T	14
GO	BP	GO:0046683	response to organophosphorus	10/647	131/18723	0.015554	0.061546	0.043121	P2RX7/PKD2/NO	10
GO	BP	GO:0006469	negative regulation of protein kinase activ	14/647	212/18723	0.01577	0.062269	0.043628	HEG1/PTPRC/WV	14
GO	BP	GO:0016339	calcium-dependent cell-cell adhesion via p	5/647	43/18723	0.015803	0.062269	0.043628	CDH11/SELL/CD	5
GO	BP	GO:0071604	transforming growth factor beta productio	5/647	43/18723	0.015803	0.062269	0.043628	LUM/TYROBP/L	5
GO	BP	GO:1903793	positive regulation of anion transport	5/647	43/18723	0.015803	0.062269	0.043628	AVPR1A/KMO/P	5
GO	BP	GO:1904591	positive regulation of protein import	5/647	43/18723	0.015803	0.062269	0.043628	HCLS1/GLI3/PRK	5
GO	BP	GO:0090303	positive regulation of wound healing	6/647	59/18723	0.015892	0.062545	0.043821	CLEC7A/FERMT	6
GO	BP	GO:0043536	positive regulation of blood vessel endoth	7/647	76/18723	0.0159	0.062545	0.043821	NRP1/VEGFC/PR	7
GO	BP	GO:0016042	lipid catabolic process	19/647	320/18723	0.015926	0.062545	0.043821	CYP1B1/HSD11B	19
GO	BP	GO:0071496	cellular response to external stimulus	19/647	320/18723	0.015926	0.062545	0.043821	SNAI2/VCAM1/T	19
GO	BP	GO:0032091	negative regulation of protein binding	8/647	94/18723	0.016058	0.062958	0.044111	LRRK2/ITGA4/C	8
GO	BP	GO:0045185	maintenance of protein location	8/647	94/18723	0.016058	0.062958	0.044111	CCDC88A/PKD2/	8
GO	BP	GO:0007611	learning or memory	16/647	255/18723	0.016124	0.063161	0.044253	TLR2/FYN/LILRI	16
GO	BP	GO:0001977	renal system process involved in regulatio	3/647	16/18723	0.016437	0.063909	0.044776	GJA1/HSD11B2/C	3
GO	BP	GO:0002827	positive regulation of T-helper 1 type imm	3/647	16/18723	0.016437	0.063909	0.044776	IL1R1/CCR2/PLA	3
GO	BP	GO:0010649	regulation of cell communication by electr	3/647	16/18723	0.016437	0.063909	0.044776	GJA1/CAV1/CAS	3
GO	BP	GO:0021783	preganglionic parasympathetic fiber devel	3/647	16/18723	0.016437	0.063909	0.044776	NRP1/EGR2/HOX	3
GO	BP	GO:0033631	cell-cell adhesion mediated by integrin	3/647	16/18723	0.016437	0.063909	0.044776	ITGA4/CXCL13/C	3
GO	BP	GO:0034138	toll-like receptor 3 signaling pathway	3/647	16/18723	0.016437	0.063909	0.044776	COLEC12/HAVC	3
GO	BP	GO:0034374	low-density lipoprotein particle remodelin	3/647	16/18723	0.016437	0.063909	0.044776	PLA2G7/APOE/A	3
GO	BP	GO:0045779	negative regulation of bone resorption	3/647	16/18723	0.016437	0.063909	0.044776	P2RX7/IL6/CD38	3
GO	BP	GO:1905906	regulation of amyloid fibril formation	3/647	16/18723	0.016437	0.063909	0.044776	TREM2/APOE/CI	3
GO	BP	GO:1990138	neuron projection extension	12/647	172/18723	0.016525	0.064198	0.044979	NRP1/CXCR4/PO	12
GO	BP	GO:0050871	positive regulation of B cell activation	11/647	152/18723	0.016591	0.064399	0.04512	TNFSF13B/NCK	11
GO	BP	GO:0007565	female pregnancy	13/647	193/18723	0.016872	0.065436	0.045846	HAVCR2/GJA1/R	13
GO	BP	GO:1902430	negative regulation of amyloid-beta forma	4/647	29/18723	0.016894	0.06547	0.04587	RTN1/APOE/IGF	4
GO	BP	GO:0000082	G1/S transition of mitotic cell cycle	14/647	214/18723	0.016976	0.065731	0.046053	SLFN11/BCAT1/I	14
GO	BP	GO:0006942	regulation of striated muscle contraction	8/647	95/18723	0.017023	0.06586	0.046144	CLIC2/PIK3CG/R	8
GO	BP	GO:0060038	cardiac muscle cell proliferation	6/647	60/18723	0.017158	0.066326	0.04647	ZFPM2/MEIS1/G	6
GO	BP	GO:0010469	regulation of signaling receptor activity	12/647	173/18723	0.017221	0.066515	0.046603	NRP1/FCGR2B/C	12

GO	BP	GO:0007389	pattern specification process	24/647	436/18723	0.017309	0.066633	0.046685	NRP1/ITGAM/PK	24
GO	BP	GO:0042269	regulation of natural killer cell mediated c	5/647	44/18723	0.017337	0.066633	0.046685	HAVCR2/KLRD1	5
GO	BP	GO:0045124	regulation of bone resorption	5/647	44/18723	0.017337	0.066633	0.046685	P2RX7/CSF1R/SF	5
GO	BP	GO:0046006	regulation of activated T cell proliferation	5/647	44/18723	0.017337	0.066633	0.046685	PDCD1LG2/CRT	5
GO	BP	GO:0048066	developmental pigmentation	5/647	44/18723	0.017337	0.066633	0.046685	ZEB2/MITF/GLI3	5
GO	BP	GO:0062208	positive regulation of pattern recognition r	5/647	44/18723	0.017337	0.066633	0.046685	TLR1/TLR2/CAV	5
GO	BP	GO:0071692	protein localization to extracellular region	21/647	368/18723	0.017489	0.067162	0.047056	FBN1/TLR2/VEG	21
GO	BP	GO:0030510	regulation of BMP signaling pathway	8/647	96/18723	0.018029	0.069124	0.04843	FBN1/SULF1/SFI	8
GO	BP	GO:1901655	cellular response to ketone	8/647	96/18723	0.018029	0.069124	0.04843	NR3C1/POSTN/S	8
GO	BP	GO:0050770	regulation of axonogenesis	11/647	154/18723	0.018114	0.069392	0.048619	NRP1/PLXNC1/C	11
GO	BP	GO:0045927	positive regulation of growth	16/647	259/18723	0.018395	0.070411	0.049332	NRP1/ZFPM2/CX	16
GO	BP	GO:0043954	cellular component maintenance	6/647	61/18723	0.018492	0.070724	0.049552	FERMT2/FCGR2I	6
GO	BP	GO:0015849	organic acid transport	18/647	303/18723	0.01853	0.070814	0.049615	SLC1A3/GJA1/A	18
GO	CC	GO:0062023	collagen-containing extracellular matrix	86/682	425/19550	2.5E-41	1.04E-38	8.82E-39	GPC6/FBN1/CCD	86
GO	CC	GO:0005581	collagen trimer	25/682	87/19550	1.35E-16	2.8E-14	2.37E-14	COLEC12/LOX/C	25
GO	CC	GO:0005788	endoplasmic reticulum lumen	36/682	313/19550	3.55E-10	4.93E-08	4.17E-08	GPX8/FBN1/PDG	36
GO	CC	GO:0030667	secretory granule membrane	34/682	311/19550	4.13E-09	4.3E-07	3.64E-07	ITGAM/RAB31/C	34
GO	CC	GO:0005604	basement membrane	17/682	96/19550	3.28E-08	2.73E-06	2.31E-06	FBN1/CCDC80/C	17
GO	CC	GO:0070820	tertiary granule	22/682	164/19550	6.41E-08	4.44E-06	3.77E-06	ITGAM/OLR1/TN	22
GO	CC	GO:0070821	tertiary granule membrane	13/682	73/19550	1.27E-06	6.29E-05	5.33E-05	ITGAM/OLR1/SL	13
GO	CC	GO:0005583	fibrillar collagen trimer	6/682	12/19550	1.36E-06	6.29E-05	5.33E-05	COL5A2/LUM/C	6
GO	CC	GO:0098643	banded collagen fibril	6/682	12/19550	1.36E-06	6.29E-05	5.33E-05	COL5A2/LUM/C	6
GO	CC	GO:0009897	external side of plasma membrane	35/682	421/19550	2.06E-06	8.59E-05	7.28E-05	ITGAM/CD163/II	35
GO	CC	GO:0030139	endocytic vesicle	30/682	336/19550	2.66E-06	9.49E-05	8.04E-05	TLR1/RAB31/CD	30
GO	CC	GO:0044853	plasma membrane raft	16/682	116/19550	2.74E-06	9.49E-05	8.04E-05	ITGAM/LCP2/MS	16
GO	CC	GO:0031091	platelet alpha granule	14/682	91/19550	3.09E-06	9.88E-05	8.37E-05	VEGFC/SRGN/P	14
GO	CC	GO:0098644	complex of collagen trimers	7/682	21/19550	4.62E-06	0.000137	0.000116	COL5A2/LUM/C	7
GO	CC	GO:0045121	membrane raft	29/682	335/19550	7.11E-06	0.000185	0.000157	TLR1/ITGAM/LC	29
GO	CC	GO:0098857	membrane microdomain	29/682	335/19550	7.11E-06	0.000185	0.000157	TLR1/ITGAM/LC	29
GO	CC	GO:0042581	specific granule	18/682	160/19550	1.26E-05	0.000307	0.000261	ITGAM/C3AR1/C	18
GO	CC	GO:0030666	endocytic vesicle membrane	20/682	193/19550	1.45E-05	0.000335	0.000284	TLR1/RAB31/CD	20
GO	CC	GO:0035579	specific granule membrane	13/682	91/19550	1.59E-05	0.000347	0.000294	ITGAM/C3AR1/C	13
GO	CC	GO:0042383	sarcolemma	15/682	138/19550	9.7E-05	0.002018	0.001711	VCAM1/CACNA	15
GO	CC	GO:0031093	platelet alpha granule lumen	10/682	67/19550	0.000103	0.002038	0.001727	VEGFC/SRGN/SI	10
GO	CC	GO:0034774	secretory granule lumen	25/682	322/19550	0.000173	0.00327	0.002772	MNDA/DOCK2/V	25
GO	CC	GO:0060205	cytoplasmic vesicle lumen	25/682	325/19550	0.0002	0.003608	0.003059	MNDA/DOCK2/V	25
GO	CC	GO:0098636	protein complex involved in cell adhesion	7/682	36/19550	0.00021	0.003647	0.003092	ITGAM/ITGB2/IT	7
GO	CC	GO:0031983	vesicle lumen	25/682	327/19550	0.000219	0.003647	0.003092	MNDA/DOCK2/V	25

GO	CC	GO:0043202	lysosomal lumen	11/682	97/19550	0.000561	0.008977	0.007609	GPC6/VCAN/CTS	11
GO	CC	GO:0008305	integrin complex	6/682	31/19550	0.000616	0.009486	0.008041	ITGAM/ITGB2/IT	6
GO	CC	GO:0001772	immunological synapse	7/682	44/19550	0.000759	0.011275	0.009557	HAVCR2/CRTAM	7
GO	CC	GO:0005796	Golgi lumen	11/682	104/19550	0.00101	0.014495	0.012287	GPC6/VCAN/DCI	11
GO	CC	GO:0043020	NADPH oxidase complex	4/682	14/19550	0.001111	0.015406	0.013059	NCF2/NOX4/NCF	4
GO	CC	GO:0005775	vacuolar lumen	15/682	174/19550	0.001176	0.015756	0.013356	GPC6/MNDA/VC	15
GO	CC	GO:0101003	ficolin-1-rich granule membrane	8/682	61/19550	0.001212	0.015756	0.013356	SLC2A3/NCKAP1	8
GO	CC	GO:0101002	ficolin-1-rich granule	15/682	185/19550	0.002158	0.027207	0.023062	MNDA/TNFAIP6/	15
GO	CC	GO:0031092	platelet alpha granule membrane	4/682	17/19550	0.00243	0.028887	0.024487	PCDH7/CD109/SI	4
GO	CC	GO:0042613	MHC class II protein complex	4/682	17/19550	0.00243	0.028887	0.024487	HLA-DPA1/HLA-	4
GO	CC	GO:0005769	early endosome	25/682	394/19550	0.003084	0.035642	0.030213	MYO5A/NRP1/R/	25
GO	CC	GO:0030669	clathrin-coated endocytic vesicle membrai	8/682	72/19550	0.003527	0.039654	0.033614	FCGR1B/SGIP1/H	8
GO	MF	GO:0005201	extracellular matrix structural constituent	50/653	172/18368	6.99E-32	5.13E-29	4.37E-29	FBN1/COL5A2/V	50
GO	MF	GO:0005539	glycosaminoglycan binding	44/653	230/18368	3.31E-20	1.21E-17	1.03E-17	NRP1/CFH/GPNM	44
GO	MF	GO:0008201	heparin binding	33/653	166/18368	5.58E-16	1.36E-13	1.16E-13	NRP1/CFH/GPNM	33
GO	MF	GO:0008009	chemokine activity	18/653	49/18368	2.69E-14	4.94E-12	4.21E-12	CCL2/CCL8/CCL	18
GO	MF	GO:0005518	collagen binding	20/653	69/18368	1.76E-13	2.59E-11	2.2E-11	LOX/DDR2/ECM	20
GO	MF	GO:0140375	immune receptor activity	27/653	144/18368	1.13E-12	1.38E-10	1.17E-10	IL1R1/OSMR/C3/	27
GO	MF	GO:0042379	chemokine receptor binding	19/653	72/18368	4.56E-12	4.77E-10	4.07E-10	CCL2/CCL8/CCL	19
GO	MF	GO:0005178	integrin binding	26/653	144/18368	7.3E-12	6.69E-10	5.7E-10	ITGAM/GPNMB/	26
GO	MF	GO:1901681	sulfur compound binding	35/653	265/18368	2.15E-11	1.75E-09	1.49E-09	NRP1/CFH/GPNM	35
GO	MF	GO:0038187	pattern recognition receptor activity	11/653	26/18368	5.01E-10	3.67E-08	3.13E-08	CLEC7A/COLEC	11
GO	MF	GO:0005126	cytokine receptor binding	32/653	271/18368	2.74E-09	1.83E-07	1.56E-07	CCDC88A/FERM	32
GO	MF	GO:0005125	cytokine activity	29/653	235/18368	5.59E-09	3.41E-07	2.91E-07	TNFSF13B/CCL2	29
GO	MF	GO:0030020	extracellular matrix structural constituent	12/653	41/18368	1.13E-08	6.38E-07	5.43E-07	COL5A2/COL6A3	12
GO	MF	GO:0001664	G protein-coupled receptor binding	32/653	295/18368	2.18E-08	1.14E-06	9.72E-07	FYN/CNRIP1/CCI	32
GO	MF	GO:0043394	proteoglycan binding	11/653	36/18368	2.82E-08	1.38E-06	1.17E-06	CFH/GPNMB/PTI	11
GO	MF	GO:0045236	CXCR chemokine receptor binding	8/653	18/18368	7.8E-08	3.57E-06	3.04E-06	CXCL12/CXCL13	8
GO	MF	GO:0048018	receptor ligand activity	42/653	487/18368	1.17E-07	5.04E-06	4.29E-06	GPNMB/FBN1/TI	42
GO	MF	GO:0030546	signaling receptor activator activity	42/653	495/18368	1.83E-07	7.44E-06	6.34E-06	GPNMB/FBN1/TI	42
GO	MF	GO:0001540	amyloid-beta binding	15/653	84/18368	2.38E-07	9.17E-06	7.8E-06	ITGAM/FCGR2B/	15
GO	MF	GO:0048020	CCR chemokine receptor binding	11/653	48/18368	7.18E-07	2.63E-05	2.24E-05	CCL2/CCL8/CCL	11
GO	MF	GO:0019864	IgG binding	6/653	11/18368	7.83E-07	2.73E-05	2.33E-05	FCGR2B/FCGR1I	6
GO	MF	GO:0004875	complement receptor activity	6/653	12/18368	1.52E-06	5.06E-05	4.31E-05	C3AR1/FPR3/C5/	6
GO	MF	GO:0019955	cytokine binding	18/653	139/18368	2.18E-06	6.96E-05	5.93E-05	NRP1/IL1R1/OSM	18
GO	MF	GO:0050840	extracellular matrix binding	11/653	56/18368	3.65E-06	0.000112	9.5E-05	DCN/OLFML2B/S	11
GO	MF	GO:0030021	extracellular matrix structural constituent	7/653	22/18368	7.43E-06	0.000218	0.000186	VCAN/DCN/LUM	7
GO	MF	GO:0030246	carbohydrate binding	25/653	271/18368	1.39E-05	0.000392	0.000333	CLEC7A/COLEC	25

GO	MF	GO:0038024	cargo receptor activity	12/653	77/18368	1.63E-05	0.000443	0.000378	ITGAM/CD163/C	12
GO	MF	GO:0061134	peptidase regulator activity	22/653	230/18368	2.59E-05	0.000677	0.000576	TIMP2/SPOCK1/(	22
GO	MF	GO:0035325	Toll-like receptor binding	5/653	12/18368	3.6E-05	0.000909	0.000774	TLR1/LY96/TLR2	5
GO	MF	GO:0015026	coreceptor activity	9/653	48/18368	4.16E-05	0.001016	0.000865	NRP1/GPC6/CD8	9
GO	MF	GO:0001848	complement binding	6/653	21/18368	6.78E-05	0.001603	0.001365	ITGAM/C5AR1/IT	6
GO	MF	GO:0042277	peptide binding	26/653	321/18368	8.71E-05	0.001995	0.001699	TLR1/ITGAM/FC	26
GO	MF	GO:0019865	immunoglobulin binding	6/653	23/18368	0.000119	0.002614	0.002226	FCGR2B/FCGR1I	6
GO	MF	GO:0001846	opsonin binding	5/653	15/18368	0.000125	0.002614	0.002226	ITGAM/ITGB2/C	5
GO	MF	GO:0050664	oxidoreductase activity, acting on NAD(P	5/653	15/18368	0.000125	0.002614	0.002226	NCF2/NOX4/KM	5
GO	MF	GO:0019956	chemokine binding	7/653	33/18368	0.000132	0.002697	0.002297	CCR1/CXCR4/IT(	7
GO	MF	GO:0008191	metalloendopeptidase inhibitor activity	5/653	16/18368	0.000176	0.0034	0.002895	TIMP2/SPOCK1/	5
GO	MF	GO:0016641	oxidoreductase activity, acting on the CH-	5/653	16/18368	0.000176	0.0034	0.002895	VCAM1/LOX/LO	5
GO	MF	GO:0023023	MHC protein complex binding	7/653	36/18368	0.000236	0.004435	0.003777	LILRB2/KLRD1/I	7
GO	MF	GO:0016175	superoxide-generating NAD(P)H oxidase	4/653	10/18368	0.00028	0.005046	0.004297	NCF2/NOX4/NCF	4
GO	MF	GO:0042056	chemoattractant activity	7/653	37/18368	0.000282	0.005046	0.004297	GPNMB/VEGFC/	7
GO	MF	GO:0001968	fibronectin binding	6/653	28/18368	0.000381	0.006341	0.0054	CCDC80/CTSL/IT	6
GO	MF	GO:0003953	NAD+ nucleosidase activity	6/653	28/18368	0.000381	0.006341	0.0054	TLR1/IL1R1/TLR	6
GO	MF	GO:0030414	peptidase inhibitor activity	17/653	187/18368	0.000381	0.006341	0.0054	TIMP2/SPOCK1/(	17
GO	MF	GO:0097493	structural molecule activity conferring ela	4/653	11/18368	0.000428	0.006968	0.005933	FBN1/FBLN5/FBI	4
GO	MF	GO:0033218	amide binding	28/653	400/18368	0.000537	0.008561	0.00729	TLR1/ITGAM/FC	28
GO	MF	GO:0016638	oxidoreductase activity, acting on the CH-	5/653	20/18368	0.000556	0.008623	0.007343	VCAM1/LOX/LO	5
GO	MF	GO:0017147	Wnt-protein binding	6/653	30/18368	0.000565	0.008623	0.007343	CTHRC1/ROR1/S	6
GO	MF	GO:0061135	endopeptidase regulator activity	17/653	194/18368	0.000581	0.008696	0.007406	TIMP2/SPOCK1/(	17
GO	MF	GO:0004896	cytokine receptor activity	11/653	97/18368	0.000654	0.009594	0.00817	IL1R1/OSMR/CSI	11
GO	MF	GO:0001614	purinergic nucleotide receptor activity	5/653	21/18368	0.000708	0.009887	0.00842	P2RX7/GPR34/P2	5
GO	MF	GO:0016502	nucleotide receptor activity	5/653	21/18368	0.000708	0.009887	0.00842	P2RX7/GPR34/P2	5
GO	MF	GO:0004866	endopeptidase inhibitor activity	16/653	180/18368	0.000715	0.009887	0.00842	TIMP2/SPOCK1/(	16
GO	MF	GO:0008528	G protein-coupled peptide receptor activit	14/653	147/18368	0.000769	0.010444	0.008894	FPR3/CCR1/CXC	14
GO	MF	GO:0016702	oxidoreductase activity, acting on single d	5/653	23/18368	0.001105	0.01472	0.012536	ALOX5AP/CDO1	5
GO	MF	GO:0001653	peptide receptor activity	14/653	153/18368	0.001138	0.014901	0.01269	FPR3/CCR1/CXC	14
GO	MF	GO:0005172	vascular endothelial growth factor recepto	4/653	14/18368	0.001192	0.015058	0.012823	CCDC88A/VEGF	4
GO	MF	GO:0045028	G protein-coupled purinergic nucleotide re	4/653	14/18368	0.001192	0.015058	0.012823	GPR34/P2RY13/C	4
GO	MF	GO:0005044	scavenger receptor activity	7/653	47/18368	0.001268	0.015754	0.013416	CD163/COLEC12	7
GO	MF	GO:0001965	G-protein alpha-subunit binding	5/653	24/18368	0.001355	0.016017	0.01364	CCDC88A/RGS2/	5
GO	MF	GO:0016701	oxidoreductase activity, acting on single d	5/653	24/18368	0.001355	0.016017	0.01364	ALOX5AP/CDO1	5
GO	MF	GO:0019957	C-C chemokine binding	5/653	24/18368	0.001355	0.016017	0.01364	CCR1/CXCR4/CC	5
GO	MF	GO:0070851	growth factor receptor binding	13/653	141/18368	0.001575	0.018329	0.015609	CCDC88A/IL1R1/	13
GO	MF	GO:0030247	polysaccharide binding	5/653	25/18368	0.001645	0.018835	0.01604	CLEC7A/ENPP2/I	5

GO	MF	GO:0016004	phospholipase activator activity	4/653	16/18368	0.002047	0.022396	0.019072	FYN/BTK/CCL8/(	4
GO	MF	GO:0050135	NAD(P)+ nucleosidase activity	4/653	16/18368	0.002047	0.022396	0.019072	TLR1/IL1R1/TLR	4
GO	MF	GO:0061809	NAD+ nucleotidase, cyclic ADP-ribose ge	4/653	16/18368	0.002047	0.022396	0.019072	TLR1/IL1R1/TLR	4
GO	MF	GO:0004867	serine-type endopeptidase inhibitor activit	10/653	98/18368	0.002533	0.027251	0.023207	SPOCK1/COL6A3	10
GO	MF	GO:0030169	low-density lipoprotein particle binding	4/653	17/18368	0.002602	0.027251	0.023207	COLEC12/MSR1/	4
GO	MF	GO:0043395	heparan sulfate proteoglycan binding	4/653	17/18368	0.002602	0.027251	0.023207	CFH/PTPRC/APC	4
GO	MF	GO:0060229	lipase activator activity	4/653	18/18368	0.003253	0.033583	0.028599	FYN/BTK/CCL8/(	4
GO	MF	GO:0071813	lipoprotein particle binding	5/653	31/18368	0.004413	0.042929	0.036558	COLEC12/MSR1/	5
GO	MF	GO:0071814	protein-lipid complex binding	5/653	31/18368	0.004413	0.042929	0.036558	COLEC12/MSR1/	5
GO	MF	GO:0032395	MHC class II receptor activity	3/653	10/18368	0.004451	0.042929	0.036558	HLA-DPA1/HLA-	3
GO	MF	GO:0050786	RAGE receptor binding	3/653	10/18368	0.004451	0.042929	0.036558	FPR1/S100A8/S10	3
GO	MF	GO:0071723	lipopeptide binding	3/653	10/18368	0.004451	0.042929	0.036558	TLR1/TLR2/CD14	3
GO	MF	GO:0019838	growth factor binding	12/653	141/18368	0.004529	0.043117	0.036717	NRP1/IL1R1/OSM	12
GO	MF	GO:0004714	transmembrane receptor protein tyrosine k	11/653	124/18368	0.004717	0.044328	0.037749	NRP1/DDR2/FYN	11
GO	MF	GO:0001530	lipopolysaccharide binding	5/653	33/18368	0.005815	0.053933	0.045928	P2RX7/LY96/TLI	5
GO	MF	GO:0042608	T cell receptor binding	3/653	11/18368	0.00596	0.053933	0.045928	DOCK2/FYN/HLA	3
GO	MF	GO:0048407	platelet-derived growth factor binding	3/653	11/18368	0.00596	0.053933	0.045928	COL3A1/COL5A1	3
GO	MF	GO:0004222	metalloendopeptidase activity	10/653	111/18368	0.006195	0.055376	0.047157	ADAM12/ADAM	10
KEGG	KEGG	hsa04061	Viral protein interaction with cytokine anc	24/345	100/8142	2.14E-12	5.85E-10	4.85E-10	IL10RA/CCR1/CX	24
KEGG	KEGG	hsa04145	Phagosome	29/345	152/8142	5.12E-12	5.93E-10	4.92E-10	ITGAM/CLEC7A/	29
KEGG	KEGG	hsa05150	Staphylococcus aureus infection	23/345	96/8142	6.52E-12	5.93E-10	4.92E-10	ITGAM/CFH/C3A	23
KEGG	KEGG	hsa04062	Chemokine signaling pathway	30/345	192/8142	3.96E-10	2.7E-08	2.24E-08	GNB4/DOCK2/CC	30
KEGG	KEGG	hsa04610	Complement and coagulation cascades	19/345	85/8142	1.66E-09	9.04E-08	7.49E-08	ITGAM/CFH/C3A	19
KEGG	KEGG	hsa05144	Malaria	14/345	50/8142	1.08E-08	4.69E-07	3.89E-07	VCAM1/TLR2/CC	14
KEGG	KEGG	hsa05133	Pertussis	17/345	76/8142	1.2E-08	4.69E-07	3.89E-07	ITGAM/LY96/ITC	17
KEGG	KEGG	hsa04060	Cytokine-cytokine receptor interaction	35/345	295/8142	2.49E-08	8.51E-07	7.05E-07	IL1R1/OSMR/TN	35
KEGG	KEGG	hsa05323	Rheumatoid arthritis	18/345	93/8142	4.97E-08	1.51E-06	1.25E-06	CD86/TNFSF13B/	18
KEGG	KEGG	hsa05140	Leishmaniasis	16/345	77/8142	9.86E-08	2.69E-06	2.23E-06	ITGAM/NCF2/TL	16
KEGG	KEGG	hsa04514	Cell adhesion molecules	22/345	149/8142	2.62E-07	6.51E-06	5.39E-06	ITGAM/CD86/VC	22
KEGG	KEGG	hsa04380	Osteoclast differentiation	20/345	128/8142	3.65E-07	8.3E-06	6.88E-06	MITF/IL1R1/NCF	20
KEGG	KEGG	hsa04640	Hematopoietic cell lineage	16/345	99/8142	3.48E-06	7.31E-05	6.06E-05	ITGAM/IL1R1/CI	16
KEGG	KEGG	hsa04672	Intestinal immune network for IgA produc	11/345	49/8142	4.61E-06	9E-05	7.46E-05	CD86/TNFSF13B/	11
KEGG	KEGG	hsa05152	Tuberculosis	22/345	180/8142	6.73E-06	0.000115	9.52E-05	TLR1/ITGAM/CL	22
KEGG	KEGG	hsa04620	Toll-like receptor signaling pathway	16/345	104/8142	6.73E-06	0.000115	9.52E-05	TLR1/CD86/LY96	16
KEGG	KEGG	hsa04974	Protein digestion and absorption	15/345	103/8142	2.57E-05	0.000412	0.000342	COL5A2/COL6A3	15
KEGG	KEGG	hsa04933	AGE-RAGE signaling pathway in diabetic	14/345	100/8142	7.44E-05	0.001096	0.000909	VCAM1/VEGFC/	14
KEGG	KEGG	hsa04512	ECM-receptor interaction	13/345	88/8142	7.63E-05	0.001096	0.000909	COL6A3/ITGA4/I	13
KEGG	KEGG	hsa04064	NF-kappa B signaling pathway	14/345	104/8142	0.000115	0.001567	0.001299	IL1R1/VCAM1/L	14

KEGG	KEGG	hsa05416	Viral myocarditis	10/345	60/8142	0.000186	0.002419	0.002005	CD86/FYN/ITGB[illegible]	10
KEGG	KEGG	hsa05171	Coronavirus disease - COVID-19	22/345	232/8142	0.000323	0.004011	0.003325	NRP1/TLR8/C3A[illegible]	22
KEGG	KEGG	hsa04657	IL-17 signaling pathway	12/345	94/8142	0.000575	0.00683	0.005662	CCL2/S100A8/CC[illegible]	12
KEGG	KEGG	hsa04670	Leukocyte transendothelial migration	13/345	114/8142	0.001021	0.011611	0.009625	ITGAM/VCAM1/[illegible]	13
KEGG	KEGG	hsa05205	Proteoglycans in cancer	19/345	205/8142	0.001079	0.011779	0.009765	TLR2/MRAS/HC[illegible]	19
KEGG	KEGG	hsa04613	Neutrophil extracellular trap formation	18/345	190/8142	0.001128	0.011845	0.009819	ITGAM/CLEC7A[illegible]	18
KEGG	KEGG	hsa05146	Amoebiasis	12/345	102/8142	0.001202	0.012152	0.010074	ITGAM/IL1R1/T[illegible]	12
KEGG	KEGG	hsa00380	Tryptophan metabolism	7/345	42/8142	0.001728	0.016265	0.013484	CYP1B1/KMO/K[illegible]	7
KEGG	KEGG	hsa05332	Graft-versus-host disease	7/345	42/8142	0.001728	0.016265	0.013484	CD86/KLRD1/HL[illegible]	7
KEGG	KEGG	hsa05322	Systemic lupus erythematosus	14/345	136/8142	0.001804	0.01642	0.013612	CD86/FCGR2A/C[illegible]	14
KEGG	KEGG	hsa04510	Focal adhesion	18/345	201/8142	0.002131	0.018766	0.015557	PDGFC/VEGFC/[illegible]	18
KEGG	KEGG	hsa05134	Legionellosis	8/345	57/8142	0.002574	0.021956	0.018201	ITGAM/TLR2/IT[illegible]	8
KEGG	KEGG	hsa04936	Alcoholic liver disease	14/345	142/8142	0.002708	0.022402	0.018571	LY96/C3AR1/NO[illegible]	14
KEGG	KEGG	hsa05142	Chagas disease	11/345	102/8142	0.0038	0.030509	0.025292	TLR2/CCL2/C1Q[illegible]	11
KEGG	KEGG	hsa04151	PI3K-Akt signaling pathway	26/345	354/8142	0.004264	0.033261	0.027573	GNB4/OSMR/TL[illegible]	26
KEGG	KEGG	hsa05417	Lipid and atherosclerosis	18/345	215/8142	0.004404	0.033396	0.027685	VCAM1/LY96/N[illegible]	18
KEGG	KEGG	hsa04668	TNF signaling pathway	11/345	112/8142	0.007697	0.054446	0.045136	VCAM1/VEGFC/[illegible]	11
KEGG	KEGG	hsa04015	Rap1 signaling pathway	17/345	210/8142	0.00775	0.054446	0.045136	ITGAM/LCP2/DC[illegible]	17
KEGG	KEGG	hsa04666	Fc gamma R-mediated phagocytosis	10/345	97/8142	0.007778	0.054446	0.045136	FCGR2B/PTPRC/[illegible]	10

Table S8. The gene subtype of cuproptosis subgroups related genes in CRC patients.

ID	geneCluster
TCGA_TCGA-DM-A288	I
TCGA_TCGA-CM-6164	II
TCGA_TCGA-AZ-4615	II
TCGA_TCGA-AA-3549	I
TCGA_TCGA-AY-4071	I
TCGA_TCGA-CM-4752	II
TCGA_TCGA-DM-A1D9	I
TCGA_TCGA-AA-3688	I
TCGA_TCGA-AA-3854	I
TCGA_TCGA-A6-3809	II
TCGA_TCGA-CM-6165	II
TCGA_TCGA-A6-5659	II
TCGA_TCGA-AA-3494	I
TCGA_TCGA-CM-4750	I
TCGA_TCGA-AZ-4682	I
TCGA_TCGA-DM-A0XF	I
TCGA_TCGA-AA-3529	I
TCGA_TCGA-AA-3949	II
TCGA_TCGA-AA-3848	I
TCGA_TCGA-CA-6715	I
TCGA_TCGA-AA-3710	II
TCGA_TCGA-A6-6138	II
TCGA_TCGA-DM-A28E	I
TCGA_TCGA-AA-3506	I
TCGA_TCGA-AU-3779	II
TCGA_TCGA-CK-5916	II
TCGA_TCGA-QG-A5Z1	II
TCGA_TCGA-AD-6890	I
TCGA_TCGA-A6-2685	II
TCGA_TCGA-AA-A00W	I
TCGA_TCGA-CK-5914	I
TCGA_TCGA-A6-2686	II
TCGA_TCGA-A6-A5ZU	II
TCGA_TCGA-NH-A50V	II
TCGA_TCGA-QG-A5YX	I
TCGA_TCGA-A6-2677	I
TCGA_TCGA-CA-6717	II
TCGA_TCGA-NH-A6GC	II
TCGA_TCGA-AA-3562	I
TCGA_TCGA-A6-4105	II
TCGA_TCGA-AA-A00R	I
TCGA_TCGA-AZ-4684	II
TCGA_TCGA-AU-6004	II
TCGA_TCGA-A6-2672	II
TCGA_TCGA-QG-A5Z2	I
TCGA_TCGA-A6-6780	II
TCGA_TCGA-F4-6459	II
TCGA_TCGA-AA-3970	I
TCGA_TCGA-NH-A8F7	I
TCGA_TCGA-G4-6321	I
TCGA_TCGA-AA-3524	I
TCGA_TCGA-DM-A1D0	I
TCGA_TCGA-G4-6307	I
TCGA_TCGA-DM-A1HB	I
TCGA_TCGA-AA-3697	I
TCGA_TCGA-CM-6679	II

TCGA_TCGA-AY-6197	I
TCGA_TCGA-A6-2684	II
TCGA_TCGA-AZ-4323	II
TCGA_TCGA-AA-3971	I
TCGA_TCGA-AA-3989	I
TCGA_TCGA-AA-A00D	II
TCGA_TCGA-A6-2679	I
TCGA_TCGA-NH-A5IV	II
TCGA_TCGA-A6-5664	II
TCGA_TCGA-AZ-4315	I
TCGA_TCGA-F4-6806	II
TCGA_TCGA-AA-3510	I
TCGA_TCGA-AY-A71X	I
TCGA_TCGA-AA-3488	I
TCGA_TCGA-DM-A28F	I
TCGA_TCGA-G4-6293	I
TCGA_TCGA-5M-AAT4	I
TCGA_TCGA-D5-6533	I
TCGA_TCGA-CM-5344	II
TCGA_TCGA-AA-3517	I
TCGA_TCGA-G4-6309	I
TCGA_TCGA-AA-3678	I
TCGA_TCGA-AA-A01X	I
TCGA_TCGA-AA-3821	II
TCGA_TCGA-CM-5861	I
TCGA_TCGA-D5-6923	II
TCGA_TCGA-AZ-6605	II
TCGA_TCGA-NH-A6GB	I
TCGA_TCGA-CM-5349	II
TCGA_TCGA-NH-A50U	I
TCGA_TCGA-AA-3530	I
TCGA_TCGA-G4-6627	II
TCGA_TCGA-DM-A28G	I
TCGA_TCGA-D5-6537	I
TCGA_TCGA-D5-5539	II
TCGA_TCGA-CM-6163	II
TCGA_TCGA-CM-4744	I
TCGA_TCGA-AA-A004	I
TCGA_TCGA-A6-3808	II
TCGA_TCGA-AA-A02J	I
TCGA_TCGA-AA-3837	II
TCGA_TCGA-CK-4947	II
TCGA_TCGA-CA-5256	I
TCGA_TCGA-CM-6167	II
TCGA_TCGA-4N-A93T	I
TCGA_TCGA-AA-3521	II
TCGA_TCGA-CA-5797	II
TCGA_TCGA-CM-6166	II
TCGA_TCGA-CK-4951	II
TCGA_TCGA-CA-6719	II
TCGA_TCGA-AA-A02R	II
TCGA_TCGA-AA-A02O	I
TCGA_TCGA-A6-5666	I
TCGA_TCGA-AY-5543	I
TCGA_TCGA-AA-3845	II
TCGA_TCGA-AA-3856	I
TCGA_TCGA-AA-A00Q	I
TCGA_TCGA-A6-6781	II

TCGA_TCGA-AA-A00F	II
TCGA_TCGA-D5-6920	I
TCGA_TCGA-AA-3815	I
TCGA_TCGA-AA-3867	II
TCGA_TCGA-A6-6782	II
TCGA_TCGA-A6-6654	II
TCGA_TCGA-G4-6311	II
TCGA_TCGA-A6-3807	II
TCGA_TCGA-AZ-5403	II
TCGA_TCGA-AD-6888	I
TCGA_TCGA-G4-6628	II
TCGA_TCGA-DM-A1D6	I
TCGA_TCGA-CM-5341	II
TCGA_TCGA-AA-3862	I
TCGA_TCGA-AA-3660	I
TCGA_TCGA-AA-3833	II
TCGA_TCGA-AA-A00Z	I
TCGA_TCGA-AZ-6598	I
TCGA_TCGA-AA-3679	I
TCGA_TCGA-DM-A285	II
TCGA_TCGA-G4-6294	I
TCGA_TCGA-A6-4107	II
TCGA_TCGA-CA-6716	II
TCGA_TCGA-AA-A01V	I
TCGA_TCGA-D5-6541	II
TCGA_TCGA-AA-A01G	I
TCGA_TCGA-AA-3870	II
TCGA_TCGA-D5-6532	I
TCGA_TCGA-CM-6169	II
TCGA_TCGA-D5-5541	II
TCGA_TCGA-DM-A1DB	I
TCGA_TCGA-AA-A00A	I
TCGA_TCGA-D5-6530	I
TCGA_TCGA-CM-5868	II
TCGA_TCGA-AA-3519	I
TCGA_TCGA-AA-3526	I
TCGA_TCGA-DM-A28M	I
TCGA_TCGA-D5-6926	II
TCGA_TCGA-AA-3534	II
TCGA_TCGA-G4-6317	I
TCGA_TCGA-AA-A03J	I
TCGA_TCGA-CM-6680	II
TCGA_TCGA-AA-3984	II
TCGA_TCGA-D5-6536	II
TCGA_TCGA-AD-A5EK	I
TCGA_TCGA-AA-3663	I
TCGA_TCGA-AZ-6607	II
TCGA_TCGA-AZ-5407	I
TCGA_TCGA-CM-5348	II
TCGA_TCGA-CK-6751	II
TCGA_TCGA-D5-6929	II
TCGA_TCGA-A6-2681	II
TCGA_TCGA-AA-3869	II
TCGA_TCGA-DM-A282	I
TCGA_TCGA-D5-7000	II
TCGA_TCGA-AM-5821	II
TCGA_TCGA-G4-6298	II
TCGA_TCGA-AA-A01S	I

TCGA_TCGA-NH-A8F8	II
TCGA_TCGA-AA-3516	I
TCGA_TCGA-AA-3846	I
TCGA_TCGA-AA-3544	I
TCGA_TCGA-AA-3956	II
TCGA_TCGA-AA-3532	II
TCGA_TCGA-AA-3955	I
TCGA_TCGA-AD-6895	II
TCGA_TCGA-AA-3556	I
TCGA_TCGA-CM-6678	I
TCGA_TCGA-AA-3851	I
TCGA_TCGA-CM-5860	II
TCGA_TCGA-AA-A02F	I
TCGA_TCGA-DM-A1DA	I
TCGA_TCGA-CM-6162	II
TCGA_TCGA-AA-3522	I
TCGA_TCGA-AA-A00U	I
TCGA_TCGA-AA-3930	II
TCGA_TCGA-AZ-6599	I
TCGA_TCGA-AA-3947	II
TCGA_TCGA-AA-3814	II
TCGA_TCGA-NH-A6GA	I
TCGA_TCGA-A6-2675	II
TCGA_TCGA-G4-6297	II
TCGA_TCGA-A6-5656	II
TCGA_TCGA-AA-3553	II
TCGA_TCGA-DM-A0XD	I
TCGA_TCGA-AA-3511	II
TCGA_TCGA-A6-A56B	II
TCGA_TCGA-A6-5667	II
TCGA_TCGA-AA-3866	II
TCGA_TCGA-AA-A017	I
TCGA_TCGA-CM-6674	II
TCGA_TCGA-CM-6170	II
TCGA_TCGA-AA-3994	II
TCGA_TCGA-AA-3877	II
TCGA_TCGA-WS-AB45	II
TCGA_TCGA-AA-A00L	I
TCGA_TCGA-CA-5254	II
TCGA_TCGA-D5-6898	II
TCGA_TCGA-AA-3696	I
TCGA_TCGA-DM-A280	I
TCGA_TCGA-QG-A5YW	I
TCGA_TCGA-AD-6965	I
TCGA_TCGA-CK-4948	II
TCGA_TCGA-DM-A1HA	I
TCGA_TCGA-5M-AAT5	I
TCGA_TCGA-CM-4748	II
TCGA_TCGA-AA-3858	I
TCGA_TCGA-D5-6531	II
TCGA_TCGA-AA-3662	II
TCGA_TCGA-AM-5820	II
TCGA_TCGA-AA-A01R	I
TCGA_TCGA-AA-3975	I
TCGA_TCGA-5M-AAT6	II
TCGA_TCGA-D5-6931	II
TCGA_TCGA-D5-6924	II
TCGA_TCGA-AA-3860	II

TCGA_TCGA-A6-5665	I
TCGA_TCGA-5M-AATA	I
TCGA_TCGA-D5-6540	II
TCGA_TCGA-G4-6323	I
TCGA_TCGA-A6-A566	II
TCGA_TCGA-AZ-6608	I
TCGA_TCGA-AA-3712	II
TCGA_TCGA-A6-5662	II
TCGA_TCGA-AA-3831	I
TCGA_TCGA-AA-A02H	I
TCGA_TCGA-CM-6676	II
TCGA_TCGA-AA-3685	I
TCGA_TCGA-CK-5915	I
TCGA_TCGA-AA-3548	I
TCGA_TCGA-F4-6569	II
TCGA_TCGA-F4-6461	II
TCGA_TCGA-F4-6854	II
TCGA_TCGA-F4-6856	I
TCGA_TCGA-AY-A54L	I
TCGA_TCGA-AA-3538	II
TCGA_TCGA-AA-3842	II
TCGA_TCGA-AZ-4308	II
TCGA_TCGA-CK-6746	I
TCGA_TCGA-AA-3509	I
TCGA_TCGA-AA-3680	I
TCGA_TCGA-AA-3542	I
TCGA_TCGA-AD-6964	II
TCGA_TCGA-A6-6650	I
TCGA_TCGA-A6-6140	I
TCGA_TCGA-AA-A024	I
TCGA_TCGA-CM-6675	I
TCGA_TCGA-4T-AA8H	I
TCGA_TCGA-A6-6653	I
TCGA_TCGA-F4-6460	II
TCGA_TCGA-AA-3655	II
TCGA_TCGA-AA-3952	II
TCGA_TCGA-T9-A92H	I
TCGA_TCGA-AY-A8YK	I
TCGA_TCGA-CK-4950	I
TCGA_TCGA-AZ-6600	II
TCGA_TCGA-AA-A00J	II
TCGA_TCGA-AA-3667	I
TCGA_TCGA-AA-3554	II
TCGA_TCGA-AZ-6606	I
TCGA_TCGA-AA-3852	II
TCGA_TCGA-D5-6922	II
TCGA_TCGA-A6-6137	I
TCGA_TCGA-AA-3495	I
TCGA_TCGA-AA-3489	II
TCGA_TCGA-G4-6322	I
TCGA_TCGA-AA-3976	I
TCGA_TCGA-AA-3811	II
TCGA_TCGA-AD-A5EJ	I
TCGA_TCGA-AA-3819	I
TCGA_TCGA-AA-A02Y	I
TCGA_TCGA-AZ-6603	II
TCGA_TCGA-AA-A022	I
TCGA_TCGA-G4-6626	I

TCGA_TCGA-AA-3875	II
TCGA_TCGA-G4-6306	I
TCGA_TCGA-G4-6320	I
TCGA_TCGA-CK-6748	II
TCGA_TCGA-AA-3864	II
TCGA_TCGA-A6-6142	II
TCGA_TCGA-A6-2678	II
TCGA_TCGA-CA-5796	I
TCGA_TCGA-A6-A567	II
TCGA_TCGA-AA-3713	I
TCGA_TCGA-A6-6648	I
TCGA_TCGA-A6-A565	II
TCGA_TCGA-A6-5660	II
TCGA_TCGA-AZ-6601	II
TCGA_TCGA-5M-AATE	I
TCGA_TCGA-CM-6161	II
TCGA_TCGA-AA-A01Q	I
TCGA_TCGA-AZ-4313	I
TCGA_TCGA-A6-6652	I
TCGA_TCGA-AA-A00E	I
TCGA_TCGA-F4-6808	I
TCGA_TCGA-AA-A02W	I
TCGA_TCGA-DM-A28C	I
TCGA_TCGA-D5-6927	II
TCGA_TCGA-AA-3681	I
TCGA_TCGA-AA-3492	I
TCGA_TCGA-A6-2671	II
TCGA_TCGA-D5-6535	II
TCGA_TCGA-AA-A029	I
TCGA_TCGA-AD-6963	I
TCGA_TCGA-CM-4747	I
TCGA_TCGA-NH-A50T	I
TCGA_TCGA-CM-6168	II
TCGA_TCGA-AA-3518	I
TCGA_TCGA-AA-A01T	I
TCGA_TCGA-RU-A8FL	I
TCGA_TCGA-AA-3972	I
TCGA_TCGA-A6-3810	II
TCGA_TCGA-G4-6586	I
TCGA_TCGA-D5-6928	II
TCGA_TCGA-G4-6315	I
TCGA_TCGA-AA-3664	I
TCGA_TCGA-G4-6303	II
TCGA_TCGA-AA-3692	I
TCGA_TCGA-AA-3968	II
TCGA_TCGA-AA-3560	I
TCGA_TCGA-CM-5862	II
TCGA_TCGA-D5-6932	II
TCGA_TCGA-AA-3982	II
TCGA_TCGA-CM-6172	II
TCGA_TCGA-D5-6529	II
TCGA_TCGA-A6-2683	I
TCGA_TCGA-CK-5913	II
TCGA_TCGA-D5-5540	I
TCGA_TCGA-AD-6901	II
TCGA_TCGA-AA-A01F	I
TCGA_TCGA-AA-3552	I
TCGA_TCGA-F4-6855	II

TCGA_TCGA-AD-5900	II
TCGA_TCGA-AA-3979	I
TCGA_TCGA-AA-A01I	I
TCGA_TCGA-AA-A03F	I
TCGA_TCGA-3L-AA1B	II
TCGA_TCGA-AA-3693	I
TCGA_TCGA-AA-3502	I
TCGA_TCGA-AA-A02E	I
TCGA_TCGA-CM-4746	I
TCGA_TCGA-AA-A01C	II
TCGA_TCGA-G4-6588	II
TCGA_TCGA-A6-2680	I
TCGA_TCGA-QG-A5YV	I
TCGA_TCGA-AA-3561	I
TCGA_TCGA-AA-3525	I
TCGA_TCGA-AA-3531	I
TCGA_TCGA-AA-3973	II
TCGA_TCGA-AA-3555	II
TCGA_TCGA-EI-6884	II
TCGA_TCGA-AG-3582	I
TCGA_TCGA-DC-6154	I
TCGA_TCGA-AG-3611	I
TCGA_TCGA-AG-3609	II
TCGA_TCGA-AG-4022	II
TCGA_TCGA-AG-A02N	I
TCGA_TCGA-AH-6549	II
TCGA_TCGA-AH-6903	I
TCGA_TCGA-AG-3727	I
TCGA_TCGA-F5-6863	II
TCGA_TCGA-AG-3887	I
TCGA_TCGA-AG-A01J	I
TCGA_TCGA-AF-5654	I
TCGA_TCGA-DY-A1DE	II
TCGA_TCGA-AF-3913	II
TCGA_TCGA-AG-A014	I
TCGA_TCGA-AG-4015	I
TCGA_TCGA-G5-6572	II
TCGA_TCGA-AG-A011	I
TCGA_TCGA-EI-6511	II
TCGA_TCGA-AG-4008	II
TCGA_TCGA-AG-3731	II
TCGA_TCGA-AG-A032	I
TCGA_TCGA-AH-6544	I
TCGA_TCGA-AG-A020	I
TCGA_TCGA-AG-A026	II
TCGA_TCGA-AG-3883	II
TCGA_TCGA-EI-7004	II
TCGA_TCGA-AF-6136	I
TCGA_TCGA-DC-4745	I
TCGA_TCGA-EI-6883	I
TCGA_TCGA-EI-6917	II
TCGA_TCGA-F5-6861	II
TCGA_TCGA-F5-6465	II
TCGA_TCGA-AF-6672	I
TCGA_TCGA-AG-3885	I
TCGA_TCGA-EI-6509	II
TCGA_TCGA-DC-5337	I
TCGA_TCGA-AG-3893	II

TCGA_TCGA-AG-A02G	I
TCGA_TCGA-AG-3901	II
TCGA_TCGA-AG-3726	I
TCGA_TCGA-AG-3896	I
TCGA_TCGA-DC-6160	I
TCGA_TCGA-AG-3581	I
TCGA_TCGA-F5-6812	II
TCGA_TCGA-AG-4001	II
TCGA_TCGA-AF-2687	II
TCGA_TCGA-AG-3909	I
TCGA_TCGA-AG-3878	II
TCGA_TCGA-AG-3592	I
TCGA_TCGA-EI-6513	I
TCGA_TCGA-DC-6158	II
TCGA_TCGA-EI-6512	I
TCGA_TCGA-AG-3601	I
TCGA_TCGA-CI-6622	I
TCGA_TCGA-BM-6198	II
TCGA_TCGA-AG-3600	I
TCGA_TCGA-AG-3599	I
TCGA_TCGA-AG-3584	I
TCGA_TCGA-DC-6156	II
TCGA_TCGA-DT-5265	II
TCGA_TCGA-AG-3593	I
TCGA_TCGA-AG-A01Y	II
TCGA_TCGA-AG-3580	I
TCGA_TCGA-AG-3742	II
TCGA_TCGA-DC-6683	II
TCGA_TCGA-AF-6655	II
TCGA_TCGA-AG-A023	II
TCGA_TCGA-AG-3728	II
TCGA_TCGA-AF-3400	II
TCGA_TCGA-AF-2691	II
TCGA_TCGA-CL-5918	I
TCGA_TCGA-F5-6864	II
TCGA_TCGA-AF-A56K	II
TCGA_TCGA-AG-4021	II
TCGA_TCGA-AG-A01N	I
TCGA_TCGA-DC-5869	II
TCGA_TCGA-AG-3608	I
TCGA_TCGA-DY-A0XA	I
TCGA_TCGA-EF-5831	II
TCGA_TCGA-AG-3725	I
TCGA_TCGA-EI-6514	II
TCGA_TCGA-CI-6624	II
TCGA_TCGA-CI-6621	II
TCGA_TCGA-EI-6510	I
TCGA_TCGA-DY-A1DG	I
TCGA_TCGA-AG-A02X	I
TCGA_TCGA-AG-3587	I
TCGA_TCGA-AG-3894	I
TCGA_TCGA-AG-A00H	II
TCGA_TCGA-AF-A56N	II
TCGA_TCGA-AG-A00C	I
TCGA_TCGA-EI-6885	II
TCGA_TCGA-AG-3732	I
TCGA_TCGA-AG-4007	I
TCGA_TCGA-AG-A016	I

TCGA_TCGA-EI-6882	I
TCGA_TCGA-AG-3575	II
TCGA_TCGA-AF-2693	II
TCGA_TCGA-DC-6157	I
TCGA_TCGA-AF-4110	II
TCGA_TCGA-AG-3591	I
TCGA_TCGA-EI-6881	I
TCGA_TCGA-AG-A008	I
TCGA_TCGA-AG-A015	I
TCGA_TCGA-AG-3892	I
TCGA_TCGA-G5-6233	I
TCGA_TCGA-AG-3898	II
TCGA_TCGA-AG-3578	I
TCGA_TCGA-DC-6682	I
TCGA_TCGA-F5-6813	II
TCGA_TCGA-F5-6702	II
TCGA_TCGA-CL-5917	II
TCGA_TCGA-CL-4957	I
TCGA_TCGA-EI-6507	II
TCGA_TCGA-DC-4749	I
TCGA_TCGA-AF-2690	II
TCGA_TCGA-EF-5830	I
TCGA_TCGA-AF-3911	II
TCGA_TCGA-DC-6155	I
TCGA_TCGA-AF-A56L	II
TCGA_TCGA-CI-6623	I
TCGA_TCGA-AH-6897	I
TCGA_TCGA-AG-3574	I
TCGA_TCGA-G5-6235	I
TCGA_TCGA-AG-4005	II
TCGA_TCGA-AG-3583	I
TCGA_TCGA-AH-6547	II
TCGA_TCGA-EI-7002	II
TCGA_TCGA-AG-A00Y	I
TCGA_TCGA-EI-6506	I
TCGA_TCGA-AG-3890	I
TCGA_TCGA-AG-3882	I
TCGA_TCGA-AG-3598	I
TCGA_TCGA-F5-6811	II
TCGA_TCGA-F5-6810	II
TCGA_TCGA-AG-3602	I
TCGA_TCGA-AF-2692	I
GSE17536_GSM437093	I
GSE17536_GSM437094	I
GSE17536_GSM437095	I
GSE17536_GSM437096	I
GSE17536_GSM437097	I
GSE17536_GSM437098	II
GSE17536_GSM437099	I
GSE17536_GSM437100	I
GSE17536_GSM437102	II
GSE17536_GSM437103	I
GSE17536_GSM437104	I
GSE17536_GSM437105	I
GSE17536_GSM437106	I
GSE17536_GSM437107	I
GSE17536_GSM437108	I
GSE17536_GSM437109	II

GSE17536_GSM437110	I
GSE17536_GSM437111	II
GSE17536_GSM437112	I
GSE17536_GSM437113	I
GSE17536_GSM437115	I
GSE17536_GSM437116	II
GSE17536_GSM437117	II
GSE17536_GSM437119	II
GSE17536_GSM437120	II
GSE17536_GSM437121	I
GSE17536_GSM437122	I
GSE17536_GSM437123	I
GSE17536_GSM437124	II
GSE17536_GSM437125	II
GSE17536_GSM437126	I
GSE17536_GSM437127	II
GSE17536_GSM437128	I
GSE17536_GSM437129	I
GSE17536_GSM437131	I
GSE17536_GSM437132	I
GSE17536_GSM437133	II
GSE17536_GSM437134	II
GSE17536_GSM437135	II
GSE17536_GSM437136	I
GSE17536_GSM437139	I
GSE17536_GSM437140	I
GSE17536_GSM437141	I
GSE17536_GSM437142	I
GSE17536_GSM437143	I
GSE17536_GSM437144	II
GSE17536_GSM437146	I
GSE17536_GSM437149	II
GSE17536_GSM437150	I
GSE17536_GSM437151	I
GSE17536_GSM437152	II
GSE17536_GSM437153	I
GSE17536_GSM437154	I
GSE17536_GSM437155	II
GSE17536_GSM437156	I
GSE17536_GSM437158	I
GSE17536_GSM437160	I
GSE17536_GSM437161	II
GSE17536_GSM437162	II
GSE17536_GSM437163	II
GSE17536_GSM437164	I
GSE17536_GSM437166	II
GSE17536_GSM437167	I
GSE17536_GSM437168	I
GSE17536_GSM437169	I
GSE17536_GSM437170	II
GSE17536_GSM437171	II
GSE17536_GSM437172	I
GSE17536_GSM437173	II
GSE17536_GSM437174	II
GSE17536_GSM437175	II
GSE17536_GSM437176	I
GSE17536_GSM437177	I
GSE17536_GSM437178	II

GSE17536_GSM437179	I
GSE17536_GSM437180	II
GSE17536_GSM437181	II
GSE17536_GSM437182	II
GSE17536_GSM437185	I
GSE17536_GSM437186	I
GSE17536_GSM437187	I
GSE17536_GSM437188	II
GSE17536_GSM437189	II
GSE17536_GSM437190	II
GSE17536_GSM437191	I
GSE17536_GSM437192	II
GSE17536_GSM437193	I
GSE17536_GSM437194	I
GSE17536_GSM437195	II
GSE17536_GSM437196	I
GSE17536_GSM437197	II
GSE17536_GSM437198	II
GSE17536_GSM437199	II
GSE17536_GSM437200	II
GSE17536_GSM437201	II
GSE17536_GSM437202	II
GSE17536_GSM437203	II
GSE17536_GSM437204	II
GSE17536_GSM437205	II
GSE17536_GSM437206	I
GSE17536_GSM437207	II
GSE17536_GSM437208	I
GSE17536_GSM437209	II
GSE17536_GSM437210	II
GSE17536_GSM437212	I
GSE17536_GSM437213	I
GSE17536_GSM437214	II
GSE17536_GSM437215	I
GSE17536_GSM437216	I
GSE17536_GSM437217	I
GSE17536_GSM437219	I
GSE17536_GSM437220	I
GSE17536_GSM437221	I
GSE17536_GSM437222	II
GSE17536_GSM437223	II
GSE17536_GSM437225	II
GSE17536_GSM437226	II
GSE17536_GSM437227	II
GSE17536_GSM437228	I
GSE17536_GSM437230	I
GSE17536_GSM437231	II
GSE17536_GSM437232	I
GSE17536_GSM437233	II
GSE17536_GSM437235	II
GSE17536_GSM437237	I
GSE17536_GSM437239	II
GSE17536_GSM437240	I
GSE17536_GSM437242	II
GSE17536_GSM437244	I
GSE17536_GSM437245	II
GSE17536_GSM437246	I
GSE17536_GSM437247	II

GSE17536_GSM437248	I
GSE17536_GSM437249	I
GSE17536_GSM437250	II
GSE17536_GSM437251	II
GSE17536_GSM437252	I
GSE17536_GSM437253	I
GSE17536_GSM437254	I
GSE17536_GSM437255	I
GSE17536_GSM437256	I
GSE17536_GSM437257	II
GSE17536_GSM437258	I
GSE17536_GSM437259	I
GSE17536_GSM437260	I
GSE17536_GSM437261	I
GSE17536_GSM437262	II
GSE17536_GSM437263	II
GSE17536_GSM437264	I
GSE17536_GSM437265	II
GSE17536_GSM437266	I
GSE17536_GSM437267	I
GSE17536_GSM437268	I
GSE17536_GSM437269	I
GSE17537_GSM437270	II
GSE17537_GSM437271	I
GSE17537_GSM437274	I
GSE17537_GSM437275	I
GSE17537_GSM437276	II
GSE17537_GSM437277	I
GSE17537_GSM437278	I
GSE17537_GSM437279	II
GSE17537_GSM437280	II
GSE17537_GSM437281	I
GSE17537_GSM437283	I
GSE17537_GSM437284	II
GSE17537_GSM437285	I
GSE17537_GSM437286	I
GSE17537_GSM437287	I
GSE17537_GSM437288	I
GSE17537_GSM437289	I
GSE17537_GSM437290	I
GSE17537_GSM437291	I
GSE17537_GSM437292	I
GSE17537_GSM437293	II
GSE17537_GSM437294	II
GSE17537_GSM437295	I
GSE17537_GSM437296	I
GSE17537_GSM437297	I
GSE17537_GSM437298	I
GSE17537_GSM437300	I
GSE17537_GSM437301	II
GSE17537_GSM437302	I
GSE17537_GSM437303	I
GSE17537_GSM437304	II
GSE17537_GSM437305	II
GSE17537_GSM437306	II
GSE17537_GSM437307	II
GSE17537_GSM437308	II
GSE17537_GSM437309	I

GSE17537_GSM437310	I
GSE17537_GSM437312	I
GSE17537_GSM437315	II
GSE17537_GSM437316	II
GSE17537_GSM437317	II
GSE17537_GSM437318	II
GSE17537_GSM437319	II
GSE17537_GSM437320	I
GSE17537_GSM437321	I
GSE17537_GSM437322	I
GSE17537_GSM437323	I
GSE17537_GSM437324	I
GSE39582_GSM971958	II
GSE39582_GSM971959	I
GSE39582_GSM971960	I
GSE39582_GSM971961	II
GSE39582_GSM971962	II
GSE39582_GSM971963	II
GSE39582_GSM971964	I
GSE39582_GSM971965	II
GSE39582_GSM971967	I
GSE39582_GSM971969	I
GSE39582_GSM971970	I
GSE39582_GSM971971	I
GSE39582_GSM971972	I
GSE39582_GSM971973	I
GSE39582_GSM971974	I
GSE39582_GSM971975	I
GSE39582_GSM971976	I
GSE39582_GSM971977	II
GSE39582_GSM971978	I
GSE39582_GSM971979	I
GSE39582_GSM971980	I
GSE39582_GSM971981	II
GSE39582_GSM971982	II
GSE39582_GSM971983	II
GSE39582_GSM971984	II
GSE39582_GSM971985	I
GSE39582_GSM971986	II
GSE39582_GSM971988	I
GSE39582_GSM971989	I
GSE39582_GSM971990	I
GSE39582_GSM971991	II
GSE39582_GSM971992	I
GSE39582_GSM971993	I
GSE39582_GSM971995	I
GSE39582_GSM971996	II
GSE39582_GSM971998	I
GSE39582_GSM971999	II
GSE39582_GSM972000	I
GSE39582_GSM972001	I
GSE39582_GSM972002	I
GSE39582_GSM972003	I
GSE39582_GSM972004	I
GSE39582_GSM972005	I
GSE39582_GSM972007	II
GSE39582_GSM972008	II
GSE39582_GSM972009	II

GSE39582_GSM972010	II
GSE39582_GSM972011	II
GSE39582_GSM972012	I
GSE39582_GSM972013	I
GSE39582_GSM972014	I
GSE39582_GSM972015	I
GSE39582_GSM972016	II
GSE39582_GSM972017	I
GSE39582_GSM972018	II
GSE39582_GSM972019	II
GSE39582_GSM972020	I
GSE39582_GSM972021	I
GSE39582_GSM972023	I
GSE39582_GSM972024	I
GSE39582_GSM972025	I
GSE39582_GSM972026	II
GSE39582_GSM972027	I
GSE39582_GSM972028	I
GSE39582_GSM972029	II
GSE39582_GSM972031	I
GSE39582_GSM972032	I
GSE39582_GSM972033	I
GSE39582_GSM972034	I
GSE39582_GSM972035	I
GSE39582_GSM972037	I
GSE39582_GSM972039	II
GSE39582_GSM972040	I
GSE39582_GSM972041	I
GSE39582_GSM972042	I
GSE39582_GSM972043	I
GSE39582_GSM972045	I
GSE39582_GSM972046	II
GSE39582_GSM972048	I
GSE39582_GSM972049	I
GSE39582_GSM972050	I
GSE39582_GSM972051	II
GSE39582_GSM972052	I
GSE39582_GSM972053	I
GSE39582_GSM972056	I
GSE39582_GSM972057	I
GSE39582_GSM972058	II
GSE39582_GSM972059	II
GSE39582_GSM972060	I
GSE39582_GSM972062	I
GSE39582_GSM972063	II
GSE39582_GSM972064	II
GSE39582_GSM972065	I
GSE39582_GSM972066	I
GSE39582_GSM972067	II
GSE39582_GSM972068	II
GSE39582_GSM972069	II
GSE39582_GSM972070	I
GSE39582_GSM972071	II
GSE39582_GSM972072	II
GSE39582_GSM972073	II
GSE39582_GSM972074	I
GSE39582_GSM972075	I
GSE39582_GSM972076	II

GSE39582_GSM972077	II
GSE39582_GSM972078	II
GSE39582_GSM972079	II
GSE39582_GSM972080	II
GSE39582_GSM972081	I
GSE39582_GSM972082	II
GSE39582_GSM972083	II
GSE39582_GSM972084	II
GSE39582_GSM972085	I
GSE39582_GSM972086	I
GSE39582_GSM972087	I
GSE39582_GSM972088	II
GSE39582_GSM972089	II
GSE39582_GSM972090	I
GSE39582_GSM972091	I
GSE39582_GSM972092	II
GSE39582_GSM972093	II
GSE39582_GSM972094	II
GSE39582_GSM972095	II
GSE39582_GSM972096	I
GSE39582_GSM972097	II
GSE39582_GSM972098	I
GSE39582_GSM972099	I
GSE39582_GSM972100	I
GSE39582_GSM972101	I
GSE39582_GSM972102	II
GSE39582_GSM972103	II
GSE39582_GSM972104	II
GSE39582_GSM972105	I
GSE39582_GSM972107	I
GSE39582_GSM972108	I
GSE39582_GSM972109	I
GSE39582_GSM972110	I
GSE39582_GSM972111	I
GSE39582_GSM972112	II
GSE39582_GSM972113	II
GSE39582_GSM972114	I
GSE39582_GSM972115	I
GSE39582_GSM972118	I
GSE39582_GSM972119	I
GSE39582_GSM972122	I
GSE39582_GSM972123	I
GSE39582_GSM972124	I
GSE39582_GSM972125	II
GSE39582_GSM972126	I
GSE39582_GSM972127	II
GSE39582_GSM972128	II
GSE39582_GSM972130	I
GSE39582_GSM972132	II
GSE39582_GSM972133	II
GSE39582_GSM972135	I
GSE39582_GSM972136	I
GSE39582_GSM972137	I
GSE39582_GSM972138	I
GSE39582_GSM972139	I
GSE39582_GSM972141	II
GSE39582_GSM972142	I
GSE39582_GSM972143	I

GSE39582_GSM972144	I
GSE39582_GSM972145	I
GSE39582_GSM972146	II
GSE39582_GSM972147	I
GSE39582_GSM972149	I
GSE39582_GSM972150	I
GSE39582_GSM972151	I
GSE39582_GSM972153	I
GSE39582_GSM972154	I
GSE39582_GSM972155	I
GSE39582_GSM972156	I
GSE39582_GSM972157	II
GSE39582_GSM972158	II
GSE39582_GSM972159	I
GSE39582_GSM972160	I
GSE39582_GSM972162	II
GSE39582_GSM972163	I
GSE39582_GSM972164	II
GSE39582_GSM972165	II
GSE39582_GSM972166	I
GSE39582_GSM972167	I
GSE39582_GSM972168	II
GSE39582_GSM972169	I
GSE39582_GSM972170	II
GSE39582_GSM972171	II
GSE39582_GSM972173	I
GSE39582_GSM972174	I
GSE39582_GSM972175	I
GSE39582_GSM972177	I
GSE39582_GSM972178	I
GSE39582_GSM972179	II
GSE39582_GSM972180	I
GSE39582_GSM972181	II
GSE39582_GSM972182	I
GSE39582_GSM972183	I
GSE39582_GSM972184	II
GSE39582_GSM972185	I
GSE39582_GSM972187	II
GSE39582_GSM972188	I
GSE39582_GSM972190	I
GSE39582_GSM972191	I
GSE39582_GSM972192	I
GSE39582_GSM972193	I
GSE39582_GSM972194	I
GSE39582_GSM972195	I
GSE39582_GSM972196	I
GSE39582_GSM972197	I
GSE39582_GSM972198	I
GSE39582_GSM972200	I
GSE39582_GSM972201	II
GSE39582_GSM972202	I
GSE39582_GSM972203	I
GSE39582_GSM972204	II
GSE39582_GSM972205	I
GSE39582_GSM972207	II
GSE39582_GSM972208	II
GSE39582_GSM972209	II
GSE39582_GSM972210	II

GSE39582_GSM972211	II
GSE39582_GSM972212	I
GSE39582_GSM972213	II
GSE39582_GSM972214	I
GSE39582_GSM972215	I
GSE39582_GSM972216	I
GSE39582_GSM972217	I
GSE39582_GSM972218	I
GSE39582_GSM972219	I
GSE39582_GSM972220	II
GSE39582_GSM972221	I
GSE39582_GSM972222	I
GSE39582_GSM972223	II
GSE39582_GSM972224	II
GSE39582_GSM972225	I
GSE39582_GSM972226	I
GSE39582_GSM972227	I
GSE39582_GSM972228	I
GSE39582_GSM972229	II
GSE39582_GSM972230	I
GSE39582_GSM972231	II
GSE39582_GSM972232	II
GSE39582_GSM972233	I
GSE39582_GSM972234	II
GSE39582_GSM972235	II
GSE39582_GSM972236	I
GSE39582_GSM972237	I
GSE39582_GSM972238	II
GSE39582_GSM972239	II
GSE39582_GSM972241	I
GSE39582_GSM972243	I
GSE39582_GSM972244	I
GSE39582_GSM972245	I
GSE39582_GSM972246	I
GSE39582_GSM972247	I
GSE39582_GSM972248	I
GSE39582_GSM972249	I
GSE39582_GSM972250	I
GSE39582_GSM972251	II
GSE39582_GSM972252	II
GSE39582_GSM972253	I
GSE39582_GSM972254	II
GSE39582_GSM972255	II
GSE39582_GSM972256	I
GSE39582_GSM972257	I
GSE39582_GSM972258	I
GSE39582_GSM972259	I
GSE39582_GSM972260	I
GSE39582_GSM972261	I
GSE39582_GSM972262	II
GSE39582_GSM972263	I
GSE39582_GSM972264	I
GSE39582_GSM972265	I
GSE39582_GSM972266	I
GSE39582_GSM972267	I
GSE39582_GSM972268	I
GSE39582_GSM972269	I
GSE39582_GSM972270	I

GSE39582_GSM972272	I
GSE39582_GSM972273	I
GSE39582_GSM972275	II
GSE39582_GSM972276	I
GSE39582_GSM972280	II
GSE39582_GSM972281	I
GSE39582_GSM972282	I
GSE39582_GSM972284	I
GSE39582_GSM972285	I
GSE39582_GSM972286	II
GSE39582_GSM972287	II
GSE39582_GSM972288	I
GSE39582_GSM972289	I
GSE39582_GSM972290	II
GSE39582_GSM972291	II
GSE39582_GSM972292	I
GSE39582_GSM972293	II
GSE39582_GSM972296	I
GSE39582_GSM972297	II
GSE39582_GSM972298	I
GSE39582_GSM972299	II
GSE39582_GSM972300	I
GSE39582_GSM972301	II
GSE39582_GSM972302	I
GSE39582_GSM972303	I
GSE39582_GSM972304	I
GSE39582_GSM972307	I
GSE39582_GSM972309	I
GSE39582_GSM972310	I
GSE39582_GSM972311	II
GSE39582_GSM972312	I
GSE39582_GSM972313	I
GSE39582_GSM972314	I
GSE39582_GSM972315	I
GSE39582_GSM972316	I
GSE39582_GSM972317	II
GSE39582_GSM972318	I
GSE39582_GSM972319	I
GSE39582_GSM972320	I
GSE39582_GSM972321	I
GSE39582_GSM972322	I
GSE39582_GSM972323	I
GSE39582_GSM972324	I
GSE39582_GSM972325	I
GSE39582_GSM972326	I
GSE39582_GSM972327	II
GSE39582_GSM972328	II
GSE39582_GSM972329	II
GSE39582_GSM972330	II
GSE39582_GSM972331	I
GSE39582_GSM972332	II
GSE39582_GSM972333	I
GSE39582_GSM972335	I
GSE39582_GSM972336	I
GSE39582_GSM972337	II
GSE39582_GSM972338	I
GSE39582_GSM972339	I
GSE39582_GSM972340	I

GSE39582_GSM972341	I
GSE39582_GSM972342	II
GSE39582_GSM972343	I
GSE39582_GSM972345	II
GSE39582_GSM972346	I
GSE39582_GSM972347	I
GSE39582_GSM972348	I
GSE39582_GSM972349	II
GSE39582_GSM972350	I
GSE39582_GSM972351	I
GSE39582_GSM972352	II
GSE39582_GSM972353	II
GSE39582_GSM972354	I
GSE39582_GSM972355	II
GSE39582_GSM972356	I
GSE39582_GSM972357	I
GSE39582_GSM972358	I
GSE39582_GSM972361	II
GSE39582_GSM972362	I
GSE39582_GSM972363	I
GSE39582_GSM972364	I
GSE39582_GSM972365	II
GSE39582_GSM972366	II
GSE39582_GSM972367	II
GSE39582_GSM972368	I
GSE39582_GSM972369	II
GSE39582_GSM972370	I
GSE39582_GSM972371	I
GSE39582_GSM972372	I
GSE39582_GSM972373	I
GSE39582_GSM972374	I
GSE39582_GSM972375	II
GSE39582_GSM972376	II
GSE39582_GSM972377	II
GSE39582_GSM972378	II
GSE39582_GSM972379	I
GSE39582_GSM972381	II
GSE39582_GSM972382	I
GSE39582_GSM972383	I
GSE39582_GSM972384	II
GSE39582_GSM972386	I
GSE39582_GSM972387	I
GSE39582_GSM972388	I
GSE39582_GSM972390	I
GSE39582_GSM972391	I
GSE39582_GSM972392	I
GSE39582_GSM972393	I
GSE39582_GSM972394	II
GSE39582_GSM972395	II
GSE39582_GSM972396	I
GSE39582_GSM972397	I
GSE39582_GSM972398	I
GSE39582_GSM972399	I
GSE39582_GSM972400	I
GSE39582_GSM972401	II
GSE39582_GSM972403	I
GSE39582_GSM972404	I
GSE39582_GSM972405	I

GSE39582_GSM972406	II
GSE39582_GSM972407	II
GSE39582_GSM972408	I
GSE39582_GSM972409	II
GSE39582_GSM972410	II
GSE39582_GSM972411	I
GSE39582_GSM972413	II
GSE39582_GSM972414	I
GSE39582_GSM972415	I
GSE39582_GSM972416	II
GSE39582_GSM972417	II
GSE39582_GSM972418	I
GSE39582_GSM972419	II
GSE39582_GSM972420	II
GSE39582_GSM972421	II
GSE39582_GSM972422	II
GSE39582_GSM972423	I
GSE39582_GSM972424	I
GSE39582_GSM972425	II
GSE39582_GSM972426	II
GSE39582_GSM972427	I
GSE39582_GSM972428	II
GSE39582_GSM972431	II
GSE39582_GSM972432	II
GSE39582_GSM972433	II
GSE39582_GSM972434	I
GSE39582_GSM972435	I
GSE39582_GSM972436	I
GSE39582_GSM972437	II
GSE39582_GSM972438	II
GSE39582_GSM972439	II
GSE39582_GSM972440	II
GSE39582_GSM972441	II
GSE39582_GSM972442	I
GSE39582_GSM972443	I
GSE39582_GSM972444	II
GSE39582_GSM972445	II
GSE39582_GSM972446	I
GSE39582_GSM972447	I
GSE39582_GSM972448	I
GSE39582_GSM972449	II
GSE39582_GSM972450	I
GSE39582_GSM972453	I
GSE39582_GSM972454	I
GSE39582_GSM972455	II
GSE39582_GSM972456	I
GSE39582_GSM972457	I
GSE39582_GSM972458	II
GSE39582_GSM972460	II
GSE39582_GSM972461	I
GSE39582_GSM972462	I
GSE39582_GSM972463	I
GSE39582_GSM972464	II
GSE39582_GSM972465	I
GSE39582_GSM972467	II
GSE39582_GSM972468	I
GSE39582_GSM972469	I
GSE39582_GSM972470	II

GSE39582_GSM972471	I
GSE39582_GSM972472	II
GSE39582_GSM972473	I
GSE39582_GSM972474	II
GSE39582_GSM972476	II
GSE39582_GSM972477	I
GSE39582_GSM972478	I
GSE39582_GSM972479	II
GSE39582_GSM972480	I
GSE39582_GSM972481	II
GSE39582_GSM972482	II
GSE39582_GSM972483	II
GSE39582_GSM972484	II
GSE39582_GSM972485	I
GSE39582_GSM972487	I
GSE39582_GSM972488	I
GSE39582_GSM972490	I
GSE39582_GSM972491	I
GSE39582_GSM972492	I
GSE39582_GSM972493	II
GSE39582_GSM972495	I
GSE39582_GSM972496	I
GSE39582_GSM972497	I
GSE39582_GSM972498	I
GSE39582_GSM972499	I
GSE39582_GSM972500	II
GSE39582_GSM972501	I
GSE39582_GSM972502	I
GSE39582_GSM972503	II
GSE39582_GSM972504	II
GSE39582_GSM972505	I
GSE39582_GSM972506	II
GSE39582_GSM972507	I
GSE39582_GSM972508	I
GSE39582_GSM972509	I
GSE39582_GSM972510	I
GSE39582_GSM972511	I
GSE39582_GSM972513	I
GSE39582_GSM972514	I
GSE39582_GSM972515	I
GSE39582_GSM972517	II
GSE39582_GSM972518	II
GSE39582_GSM972519	II
GSE39582_GSM972520	I
GSE39582_GSM972521	II
GSE39582_GSM972522	II
GSE38832_GSM950411	II
GSE38832_GSM950412	II
GSE38832_GSM950413	II
GSE38832_GSM950414	I
GSE38832_GSM950415	I
GSE38832_GSM950416	I
GSE38832_GSM950417	I
GSE38832_GSM950418	I
GSE38832_GSM950419	I
GSE38832_GSM950422	II
GSE38832_GSM950424	II
GSE38832_GSM950425	II

GSE38832_GSM950426	I
GSE38832_GSM950429	I
GSE38832_GSM950431	I
GSE38832_GSM950433	II
GSE38832_GSM950434	I
GSE38832_GSM950435	I
GSE38832_GSM950436	I
GSE38832_GSM950437	I
GSE38832_GSM950438	I
GSE38832_GSM950440	I
GSE38832_GSM950441	I
GSE38832_GSM950442	II
GSE38832_GSM950443	I
GSE38832_GSM950444	II
GSE38832_GSM950445	II
GSE38832_GSM950446	II
GSE38832_GSM950447	II
GSE38832_GSM950448	I
GSE38832_GSM950449	II
GSE38832_GSM950450	I
GSE38832_GSM950451	II
GSE38832_GSM950452	II
GSE38832_GSM950453	I
GSE38832_GSM950454	I
GSE38832_GSM950455	II
GSE38832_GSM950456	I
GSE38832_GSM950457	II
GSE38832_GSM950458	I
GSE38832_GSM950459	II
GSE38832_GSM950460	I
GSE38832_GSM950461	II
GSE38832_GSM950462	II
GSE38832_GSM950463	II
GSE38832_GSM950464	II
GSE38832_GSM950465	II
GSE38832_GSM950466	II
GSE38832_GSM950468	II
GSE38832_GSM950470	II
GSE38832_GSM950471	II
GSE38832_GSM950472	I
GSE38832_GSM950473	II
GSE38832_GSM950474	I
GSE38832_GSM950475	I
GSE38832_GSM950476	II
GSE38832_GSM950477	II
GSE38832_GSM950478	II
GSE38832_GSM950479	II
GSE38832_GSM950480	I
GSE38832_GSM950481	I
GSE38832_GSM950482	I
GSE38832_GSM950483	I
GSE38832_GSM950484	II
GSE38832_GSM950485	II
GSE38832_GSM950486	II
GSE38832_GSM950487	II
GSE38832_GSM950488	II
GSE38832_GSM950489	II
GSE38832_GSM950490	II

GSE38832_GSM950491	II
GSE38832_GSM950492	II
GSE38832_GSM950493	II
GSE38832_GSM950494	II
GSE38832_GSM950495	II
GSE38832_GSM950496	II
GSE38832_GSM950497	I
GSE38832_GSM950498	I
GSE38832_GSM950499	II
GSE38832_GSM950500	II
GSE38832_GSM950502	I
GSE38832_GSM950503	II
GSE38832_GSM950504	I
GSE38832_GSM950506	I
GSE38832_GSM950508	I
GSE38832_GSM950510	II
GSE38832_GSM950511	I
GSE38832_GSM950512	I
GSE38832_GSM950513	I
GSE38832_GSM950514	I
GSE38832_GSM950515	I
GSE38832_GSM950516	I
GSE38832_GSM950517	I
GSE38832_GSM950518	I
GSE38832_GSM950519	I
GSE38832_GSM950520	I
GSE38832_GSM950521	I
GSE38832_GSM950522	I
GSE38832_GSM950524	I
GSE38832_GSM950525	I
GSE38832_GSM950526	II
GSE38832_GSM950527	I
GSE38832_GSM950528	I
GSE38832_GSM950529	I
GSE38832_GSM950530	II
GSE38832_GSM950531	II
GSE38832_GSM950532	II

Table S9. The prognostic values of 428 cuproptosis subtype related genes in CRC patients.

Gene	HR	HR.95L	HR.95H	Cox *P* -value
ZEB2	1.292	1.081	1.546	0.005
GNB4	1.249	1.053	1.482	0.011
FRMD6	1.297	1.149	1.463	0.000
MYO5A	1.274	1.042	1.557	0.018
QKI	1.457	1.199	1.771	0.000
NRP1	1.489	1.199	1.850	0.000
BCAT1	1.227	1.083	1.392	0.001
NR3C1	1.205	1.028	1.412	0.022
PRRX1	1.222	1.090	1.371	0.001
DPYD	1.244	1.049	1.474	0.012
ITGAM	1.184	1.043	1.345	0.009
CHST11	1.222	1.019	1.466	0.031
VGLL3	1.286	1.149	1.439	0.000
PLXDC2	1.208	1.050	1.391	0.008
CCDC88A	1.261	1.065	1.493	0.007
C20orf194	1.424	1.210	1.676	0.000
PKD2	1.308	1.108	1.543	0.001
MAFB	1.186	1.027	1.370	0.020
RAB31	1.219	1.065	1.396	0.004
CFH	1.203	1.059	1.367	0.005
GPNMB	1.169	1.039	1.316	0.009
SNAI2	1.205	1.052	1.382	0.007
MITF	1.389	1.184	1.629	0.000
IL1R1	1.459	1.186	1.795	0.000
COLEC12	1.268	1.145	1.404	0.000
GPC6	1.203	1.051	1.377	0.007
JAZF1	1.438	1.208	1.713	0.000
TM6SF1	1.162	1.009	1.338	0.037
CLMP	1.190	1.040	1.360	0.011
ST3GAL6	1.323	1.095	1.598	0.004
TRPS1	1.386	1.182	1.625	0.000
MAGI2-AS	1.403	1.184	1.663	0.000
ZFPM2	1.373	1.223	1.540	0.000
FERMT2	1.337	1.164	1.537	0.000
GPX8	1.295	1.119	1.499	0.001
TMEM45A	1.199	1.074	1.338	0.001
OSMR	1.397	1.180	1.653	0.000
FAP	1.178	1.071	1.296	0.001
FBN1	1.310	1.148	1.495	0.000
HEG1	1.431	1.190	1.722	0.000
CLIP4	1.288	1.109	1.496	0.001
MPDZ	1.397	1.224	1.595	0.000
LINC0109	1.171	1.028	1.333	0.017
OLR1	1.196	1.088	1.314	0.000
GFPT2	1.213	1.079	1.365	0.001
MIR100HG	1.277	1.158	1.408	0.000
GAS1	1.212	1.104	1.330	0.000
PHLDB2	1.369	1.143	1.639	0.001
LOX	1.257	1.100	1.436	0.001
CDK14	1.411	1.186	1.679	0.000
ZNF521	1.297	1.129	1.491	0.000
DOCK4	1.238	1.011	1.517	0.039
GLT8D2	1.217	1.057	1.401	0.006
ADAM12	1.263	1.112	1.434	0.000
CDH11	1.371	1.188	1.583	0.000
ATP8B2	1.308	1.078	1.587	0.007

MCC	1.445	1.208	1.728	0.000
SYNE1	1.357	1.116	1.649	0.002
CCDC80	1.289	1.143	1.454	0.000
TCF4	1.415	1.191	1.680	0.000
TSHZ3	1.359	1.139	1.622	0.001
ZNF532	1.577	1.338	1.857	0.000
PTPRM	1.481	1.217	1.801	0.000
PDGFC	1.284	1.127	1.464	0.000
DZIP1	1.431	1.211	1.692	0.000
TNFAIP6	1.136	1.031	1.251	0.010
SULF1	1.212	1.076	1.366	0.002
TSPYL5	1.357	1.170	1.575	0.000
MDFIC	1.278	1.066	1.532	0.008
MRAS	1.359	1.117	1.654	0.002
SLC2A3	1.338	1.178	1.519	0.000
DDR2	1.270	1.108	1.455	0.001
UBE2E2	1.193	1.040	1.369	0.012
TPST1	1.337	1.111	1.607	0.002
CACNA2D	1.300	1.122	1.506	0.000
BNC2	1.301	1.133	1.493	0.000
VIM	1.527	1.246	1.871	0.000
KCNE4	1.445	1.246	1.677	0.000
VEGFC	1.451	1.231	1.709	0.000
CHST15	1.382	1.126	1.696	0.002
NOX4	1.306	1.153	1.479	0.000
TIMP2	1.391	1.174	1.649	0.000
OLFML1	1.267	1.077	1.490	0.004
FYN	1.413	1.186	1.685	0.000
COL5A2	1.354	1.178	1.558	0.000
SPOCK1	1.192	1.088	1.305	0.000
CNRIP1	1.304	1.104	1.540	0.002
PRICKLE1	1.465	1.277	1.681	0.000
WWTR1	1.482	1.247	1.760	0.000
SYNC	1.212	1.077	1.364	0.001
MSRB3	1.388	1.193	1.616	0.000
VCAN	1.297	1.141	1.473	0.000
GLI3	1.372	1.175	1.601	0.000
ARL4C	1.579	1.307	1.907	0.000
AMOTL1	1.528	1.264	1.846	0.000
ECM2	1.367	1.192	1.568	0.000
CTSL	1.250	1.030	1.517	0.024
CYP1B1	1.249	1.139	1.369	0.000
MEIS2	1.216	1.092	1.353	0.000
MSR1	1.172	1.016	1.352	0.029
ARMCX1	1.333	1.163	1.529	0.000
COL6A3	1.299	1.111	1.518	0.001
DSEL	1.301	1.079	1.569	0.006
DCN	1.275	1.062	1.532	0.009
NUAK1	1.331	1.137	1.559	0.000
DOK5	1.277	1.098	1.484	0.001
C5AR1	1.176	1.030	1.341	0.016
TRIB2	1.204	1.021	1.420	0.028
LUM	1.254	1.075	1.464	0.004
NEXN	1.191	1.064	1.334	0.002
POSTN	1.293	1.114	1.501	0.001
MEIS1	1.264	1.108	1.442	0.000
DNM3OS	1.233	1.061	1.432	0.006
NAP1L3	1.303	1.163	1.460	0.000

GJA1	1.362	1.165	1.593	0.000
FAM20A	1.186	1.009	1.393	0.038
PCDH7	1.363	1.172	1.585	0.000
DPYSL3	1.417	1.195	1.680	0.000
INHBA	1.346	1.172	1.546	0.000
RAI14	1.493	1.251	1.782	0.000
STON1	1.469	1.260	1.712	0.000
SERPING1	1.195	1.038	1.375	0.013
COL8A1	1.344	1.172	1.541	0.000
BICC1	1.327	1.111	1.585	0.002
NXN	1.298	1.130	1.491	0.000
SDC2	1.321	1.118	1.560	0.001
CALCRL	1.272	1.072	1.508	0.006
BEND6	1.429	1.218	1.676	0.000
TSHZ2	1.333	1.165	1.525	0.000
FAM110B	1.355	1.138	1.612	0.001
CFL2	1.256	1.083	1.456	0.003
CTHRC1	1.220	1.096	1.358	0.000
FBXL7	1.453	1.208	1.747	0.000
TREM1	1.100	1.005	1.205	0.039
PABPC4L	1.493	1.267	1.759	0.000
RNF180	1.454	1.270	1.666	0.000
VSIG4	1.150	1.015	1.302	0.028
PCDH17	1.280	1.077	1.521	0.005
RGS2	1.154	1.016	1.311	0.028
C3orf80	1.214	1.070	1.378	0.003
CYBRD1	1.305	1.141	1.492	0.000
EVC	1.233	1.038	1.464	0.017
OLFML2B	1.311	1.135	1.515	0.000
EBF1	1.430	1.195	1.712	0.000
ZNF677	1.353	1.117	1.639	0.002
KLHL5	1.245	1.035	1.497	0.020
CHSY3	1.413	1.214	1.645	0.000
RUNX1T1	1.296	1.090	1.542	0.003
AKAP12	1.412	1.247	1.598	0.000
SERPINF1	1.180	1.025	1.359	0.021
MEOX2	1.254	1.131	1.391	0.000
MLLT11	1.630	1.384	1.920	0.000
ADAMTS2	1.195	1.020	1.400	0.028
CD109	1.294	1.144	1.464	0.000
KLRD1	0.802	0.682	0.944	0.008
NID2	1.242	1.066	1.446	0.005
MEF2C	1.218	1.014	1.464	0.035
TCEAL7	1.301	1.145	1.478	0.000
SGCE	1.312	1.137	1.515	0.000
FNDC1	1.169	1.070	1.278	0.001
FAM126A	1.249	1.056	1.478	0.010
PDE1A	1.347	1.147	1.582	0.000
STEAP4	1.213	1.057	1.393	0.006
NLGN4X	1.250	1.070	1.460	0.005
AEBP1	1.301	1.140	1.484	0.000
TNFSF4	1.167	1.029	1.324	0.017
GBP5	0.850	0.749	0.964	0.012
PALMD	1.423	1.221	1.660	0.000
MICU3	1.301	1.142	1.482	0.000
TUBB6	1.354	1.153	1.592	0.000
COL10A1	1.149	1.071	1.233	0.000
BACH2	1.345	1.142	1.583	0.000

C1R	1.214	1.033	1.427	0.018
COL15A1	1.194	1.042	1.367	0.011
DCLK1	1.284	1.114	1.480	0.001
LAMA2	1.230	1.054	1.434	0.009
ANGPTL2	1.294	1.092	1.534	0.003
COL3A1	1.419	1.198	1.679	0.000
SMARCA1	1.628	1.381	1.920	0.000
MXRA5	1.191	1.050	1.351	0.007
GALNT15	1.297	1.138	1.478	0.000
SLIT2	1.265	1.140	1.403	0.000
UBE2QL1	1.269	1.115	1.445	0.000
LRCH2	1.250	1.092	1.432	0.001
AVPR1A	1.230	1.060	1.427	0.006
PXDN	1.461	1.242	1.718	0.000
RTN1	1.166	1.020	1.333	0.024
EGR2	1.267	1.113	1.443	0.000
SSPN	1.316	1.100	1.575	0.003
C14orf132	1.340	1.162	1.546	0.000
EFEMP1	1.309	1.122	1.527	0.001
CAV1	1.281	1.080	1.520	0.004
TWIST1	1.236	1.103	1.384	0.000
FGF7	1.205	1.064	1.365	0.003
JAM2	1.292	1.111	1.503	0.001
PRKD1	1.499	1.273	1.765	0.000
ROR1	1.245	1.083	1.432	0.002
MFAP5	1.145	1.051	1.248	0.002
KCNJ8	1.410	1.205	1.650	0.000
MAP1B	1.532	1.327	1.768	0.000
SGIP1	1.294	1.111	1.507	0.001
SFRP2	1.130	1.057	1.207	0.000
ENOX1	1.311	1.095	1.571	0.003
THBS2	1.239	1.119	1.371	0.000
THSD7A	1.397	1.215	1.607	0.000
ADAMTS1	1.307	1.098	1.556	0.003
CCDC102	1.271	1.104	1.463	0.001
SRPX	1.274	1.133	1.432	0.000
RASGRP1	0.807	0.704	0.925	0.002
TRPC1	1.389	1.202	1.606	0.000
HOPX	1.348	1.144	1.588	0.000
DACT1	1.219	1.071	1.388	0.003
MGP	1.332	1.188	1.494	0.000
FIBIN	1.344	1.158	1.559	0.000
TMEM47	1.281	1.120	1.465	0.000
COL8A2	1.314	1.141	1.513	0.000
SPARC	1.416	1.195	1.677	0.000
PLK2	1.288	1.117	1.484	0.000
PDLIM3	1.351	1.154	1.580	0.000
DNAJB4	1.359	1.139	1.622	0.001
ZNF542P	1.320	1.127	1.547	0.001
COL5A1	1.329	1.151	1.535	0.000
ADAMTS1	1.278	1.114	1.466	0.000
IL21R	0.827	0.694	0.986	0.034
SRGAP2C	1.468	1.266	1.701	0.000
CRISPLD1	1.380	1.233	1.545	0.000
HMCN1	1.296	1.170	1.437	0.000
ADAMTS5	1.459	1.230	1.731	0.000
COL12A1	1.309	1.105	1.550	0.002
CD36	1.384	1.212	1.581	0.000

SPARCL1	1.327	1.155	1.525	0.000
COL11A1	1.181	1.080	1.291	0.000
SGCD	1.374	1.179	1.601	0.000
TFPI	1.233	1.043	1.458	0.014
SYNDIG1	1.227	1.060	1.421	0.006
MYH10	1.383	1.171	1.634	0.000
PDGFRL	1.175	1.069	1.291	0.001
RGS4	1.249	1.109	1.406	0.000
COL1A2	1.327	1.144	1.539	0.000
TENM4	1.306	1.141	1.496	0.000
SGK1	1.206	1.020	1.426	0.028
PLN	1.163	1.066	1.268	0.001
GLIS2	1.497	1.289	1.740	0.000
ASPN	1.226	1.122	1.340	0.000
LOXL1	1.307	1.143	1.494	0.000
CNTN4	1.362	1.173	1.581	0.000
KIF26B	1.452	1.257	1.678	0.000
RASSF8	1.267	1.072	1.498	0.005
COL24A1	1.220	1.062	1.402	0.005
HTRA1	1.454	1.239	1.706	0.000
FBLN5	1.293	1.114	1.499	0.001
SETBP1	1.193	1.032	1.380	0.017
EML1	1.179	1.012	1.374	0.035
ITGA5	1.364	1.164	1.599	0.000
SPHK1	1.223	1.055	1.417	0.008
ANKRD6	1.478	1.275	1.714	0.000
TMEM255	1.206	1.070	1.360	0.002
NAV3	1.446	1.263	1.655	0.000
KCND2	1.256	1.117	1.413	0.000
PCOLCE	1.200	1.040	1.384	0.013
RNF150	1.237	1.087	1.408	0.001
CDH2	1.314	1.179	1.463	0.000
GLIS3	1.327	1.168	1.507	0.000
PABPC5	1.423	1.207	1.679	0.000
ANXA1	1.341	1.126	1.596	0.001
MARCO	1.189	1.092	1.295	0.000
PTHLH	1.246	1.080	1.436	0.002
GLRB	1.388	1.196	1.610	0.000
PTGIS	1.246	1.106	1.404	0.000
ZFHX4	1.332	1.164	1.525	0.000
TMEM200	1.287	1.100	1.505	0.002
BGN	1.379	1.185	1.605	0.000
EBF2	1.448	1.261	1.663	0.000
FBLN2	1.191	1.043	1.361	0.010
TREM2	1.208	1.058	1.381	0.005
ARMCX2	1.412	1.223	1.630	0.000
ABCA6	1.209	1.070	1.367	0.002
TANC2	1.319	1.121	1.552	0.001
F13A1	1.106	1.001	1.221	0.048
RHOH	0.804	0.680	0.949	0.010
CXCL12	1.158	1.011	1.328	0.035
GGT5	1.372	1.166	1.614	0.000
AOC3	1.279	1.129	1.450	0.000
DAPK1	1.226	1.040	1.446	0.015
ELOVL4	1.294	1.124	1.490	0.000
PCDHB7	1.375	1.196	1.582	0.000
ZNF667-A	1.490	1.271	1.746	0.000
FILIP1	1.349	1.176	1.547	0.000

GEM	1.331	1.145	1.547	0.000
PRDM6	1.443	1.250	1.665	0.000
PEG3	1.302	1.127	1.504	0.000
C8orf88	1.250	1.116	1.400	0.000
ERMN	1.253	1.103	1.422	0.001
SPP1	1.189	1.076	1.313	0.001
EMILIN1	1.278	1.087	1.503	0.003
ENPP2	1.192	1.040	1.367	0.012
FN1	1.293	1.114	1.500	0.001
NDN	1.289	1.103	1.506	0.001
HOXB2	1.315	1.129	1.532	0.000
P2RY13	0.834	0.739	0.941	0.003
SCG2	1.398	1.259	1.552	0.000
FGF2	1.189	1.035	1.367	0.015
SERPINE1	1.413	1.239	1.611	0.000
HSPB8	1.241	1.058	1.455	0.008
RBMS1	1.318	1.137	1.529	0.000
PRICKLE2	1.421	1.219	1.656	0.000
HLA-DMB	0.853	0.737	0.988	0.034
PRR15	0.818	0.699	0.958	0.013
PCSK5	1.335	1.160	1.536	0.000
ZNF469	1.230	1.044	1.449	0.013
ANGPTL1	1.158	1.055	1.271	0.002
HTR2B	1.258	1.166	1.356	0.000
RERG	1.225	1.087	1.381	0.001
MRGPRF	1.156	1.020	1.311	0.024
CYS1	1.314	1.146	1.506	0.000
BOC	1.350	1.171	1.557	0.000
APOE	1.147	1.001	1.314	0.048
SLC24A3	1.173	1.017	1.352	0.028
HSD17B6	1.287	1.148	1.443	0.000
ISM1	1.396	1.257	1.551	0.000
LAMP5	1.280	1.151	1.424	0.000
SFRP4	1.157	1.066	1.255	0.000
IGF1	1.231	1.073	1.411	0.003
IL7R	0.850	0.734	0.984	0.029
CNTN1	1.271	1.131	1.428	0.000
ITGBL1	1.229	1.119	1.350	0.000
ARSI	1.269	1.101	1.463	0.001
CILP	1.192	1.082	1.313	0.000
MCEMP1	1.165	1.046	1.298	0.006
SLC43A3	1.198	1.033	1.390	0.017
MN1	1.309	1.162	1.475	0.000
PI15	1.143	1.020	1.280	0.021
TAGLN	1.330	1.150	1.538	0.000
FABP4	1.245	1.140	1.360	0.000
GULP1	1.350	1.168	1.561	0.000
HLA-DRA	0.880	0.775	0.999	0.048
PRELP	1.325	1.184	1.483	0.000
EPYC	1.144	1.076	1.216	0.000
APOC1	1.156	1.010	1.323	0.035
PPP1R3C	1.244	1.077	1.437	0.003
THEMIS	0.843	0.721	0.985	0.031
TIGIT	0.787	0.674	0.920	0.003
ISLR	1.312	1.147	1.502	0.000
ROR2	1.183	1.041	1.343	0.010
SUSD5	1.329	1.163	1.519	0.000
FXYD6	1.289	1.128	1.474	0.000

ZBED2	1.152	1.033	1.284	0.011
CRYAB	1.455	1.265	1.674	0.000
HSD11B2	0.829	0.728	0.945	0.005
OMD	1.191	1.095	1.295	0.000
LRRC15	1.193	1.038	1.372	0.013
CXCL13	0.873	0.812	0.938	0.000
PTX3	1.176	1.039	1.331	0.010
TIMP3	1.200	1.062	1.355	0.003
PDZRN4	1.209	1.097	1.334	0.000
CDO1	1.190	1.072	1.321	0.001
CXCL9	0.810	0.740	0.886	0.000
C11orf96	1.228	1.064	1.418	0.005
PCDHB5	1.309	1.168	1.467	0.000
HS3ST2	1.220	1.081	1.376	0.001
CCL5	0.831	0.729	0.948	0.006
LMOD1	1.225	1.081	1.388	0.001
NPR3	1.382	1.246	1.533	0.000
NKX3-2	1.317	1.153	1.505	0.000
BEX4	1.246	1.096	1.416	0.001
NBEA	1.176	1.033	1.339	0.014
SPON1	1.156	1.028	1.301	0.016
COMP	1.196	1.113	1.285	0.000
MATN3	1.266	1.154	1.389	0.000
CXCL10	0.796	0.725	0.873	0.000
LINC0111[illegible]	1.198	1.058	1.358	0.004
C10orf99	0.934	0.875	0.997	0.042
THBS4	1.170	1.075	1.274	0.000
ZNF331	1.224	1.062	1.411	0.005
C5orf46	1.178	1.080	1.284	0.000
SFRP1	1.120	1.005	1.249	0.041
SYNPO2	1.174	1.056	1.306	0.003
RRAGD	1.237	1.093	1.401	0.001
TRAT1	0.843	0.732	0.969	0.017
HOXC6	1.112	1.039	1.190	0.002
DDC	0.833	0.746	0.929	0.001
ZNF304	1.210	1.061	1.380	0.004
FHL1	1.181	1.035	1.348	0.013
SELENBP1	0.824	0.738	0.921	0.001
CNN1	1.150	1.042	1.269	0.006
NKG7	0.867	0.766	0.980	0.023
BCHE	1.176	1.084	1.277	0.000
OGN	1.114	1.033	1.200	0.005
CDHR1	0.881	0.808	0.961	0.004
AHNAK2	1.349	1.190	1.528	0.000
C7	1.236	1.111	1.375	0.000
ID1	0.879	0.785	0.985	0.026
CD38	0.809	0.717	0.913	0.001
AGTR1	1.230	1.122	1.349	0.000
MAMDC2	1.143	1.039	1.257	0.006
PCOLCE2	1.210	1.108	1.321	0.000
RAMP1	1.158	1.028	1.306	0.016
TRBC1	0.807	0.713	0.913	0.001
AMIGO2	1.281	1.132	1.450	0.000
GXYLT2	1.177	1.043	1.329	0.008
GZMA	0.809	0.721	0.908	0.000
TCEAL2	1.180	1.084	1.284	0.000
EGR3	1.201	1.069	1.349	0.002
IDO1	0.794	0.714	0.884	0.000

KANK4	1.351	1.226	1.488	0.000
MAB21L2	1.126	1.014	1.249	0.026
MMP12	0.853	0.775	0.938	0.001
CALB2	1.175	1.061	1.300	0.002
DUSP4	1.148	1.034	1.273	0.009
GNLY	0.803	0.714	0.903	0.000
PCDHB16	1.236	1.123	1.362	0.000
IGFL2	1.125	1.035	1.223	0.005
SHISA2	1.169	1.048	1.304	0.005
CHIT1	1.105	1.007	1.211	0.035
AIM2	0.884	0.796	0.981	0.020
CXCL11	0.895	0.831	0.964	0.003
ADIPOQ	1.193	1.105	1.288	0.000
SPINK1	0.854	0.786	0.927	0.000
CASQ2	1.132	1.039	1.234	0.005
FABP1	0.909	0.842	0.983	0.017
MMP1	0.909	0.847	0.975	0.008
CKMT2	0.905	0.839	0.977	0.011
SLC26A3	0.931	0.872	0.994	0.032
CHP2	0.916	0.849	0.987	0.022
GRP	1.181	1.088	1.281	0.000
VIP	1.086	1.007	1.171	0.032
TNFRSF17	0.862	0.796	0.933	0.000
KRT6A	1.159	1.089	1.234	0.000

Table S10. Multivariate Cox regression analysis of 8 CRGs associated with DFS in CRC patients.

Gene	coef	HR	HR.95L	HR.95H	*P* -value
KLRD1	-0.469	0.626	0.451	0.867	0.005
SMARCA1	0.281	1.325	1.010	1.738	0.042
SCG2	0.185	1.203	1.011	1.432	0.037
SERPINE1	0.263	1.301	1.036	1.633	0.024
HS3ST2	0.227	1.255	1.051	1.498	0.012
CXCL10	-0.230	0.795	0.667	0.947	0.010
SELENBP1	-0.217	0.805	0.667	0.971	0.023
MMP12	-0.148	0.863	0.731	1.018	0.080
