# Supplementary material for: Molecular Subtypes Based on Cuproptosis-Related Genes and Tumor Microenvironment Infiltration Characterization in Colorectal Cancer
Source: J Oncol. 2022 Oct 11;2022:5034092. doi: 10.1155/2022/5034092 (PMC9579866; doi:10.1155/2022/5034092)
Supplement: Supplementary 2 — Figure S1-S9. Figure S1: Flowchart of the research. Figure S2: Unsupervised clustering of cuproptosis cluster and consensus matrix heatmaps for k = 2, 4-9. Figure S3: Unsupervised clustering of cuproptosis-related gene cluster and Consensus matrix heatmaps for k = 3 − 9. Figure S4: The identification of candidate prognostic genes by LASSO regression analysis. Figure S5: Construction of CRG_score model in the training dataset. Figure S6: Validation of CRG_score model in the testing dataset. Figure S7: Validation of CRG_score model in the GSE161158 dataset. Figure S8: Development and evaluation of a prognostic nomogram in the training and testing sets. Figure S9: Relationships between CRG_score and chemotherapeutic sensitivity. [file 5034092.f2.pdf]

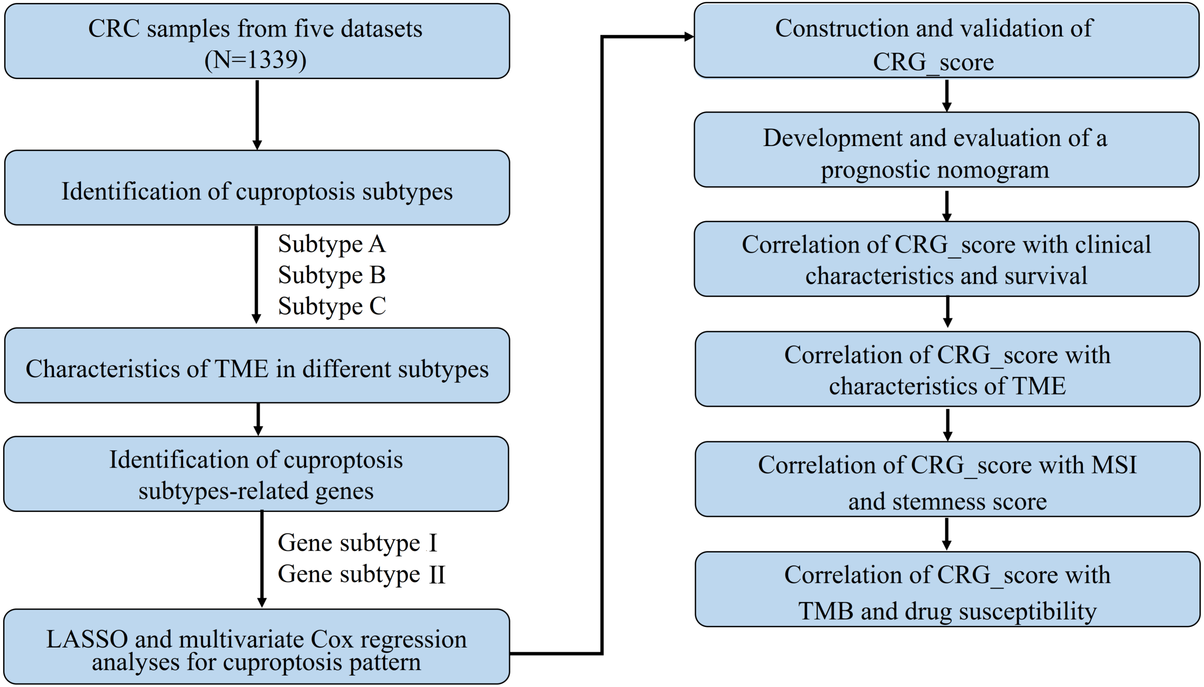

Supplementary Fig. S1 Flowchart of the research. TME, tumor microenvironment; CRG, cuproptosis related genes; TMB, tumor mutation burden; CRC, colorectal cancer

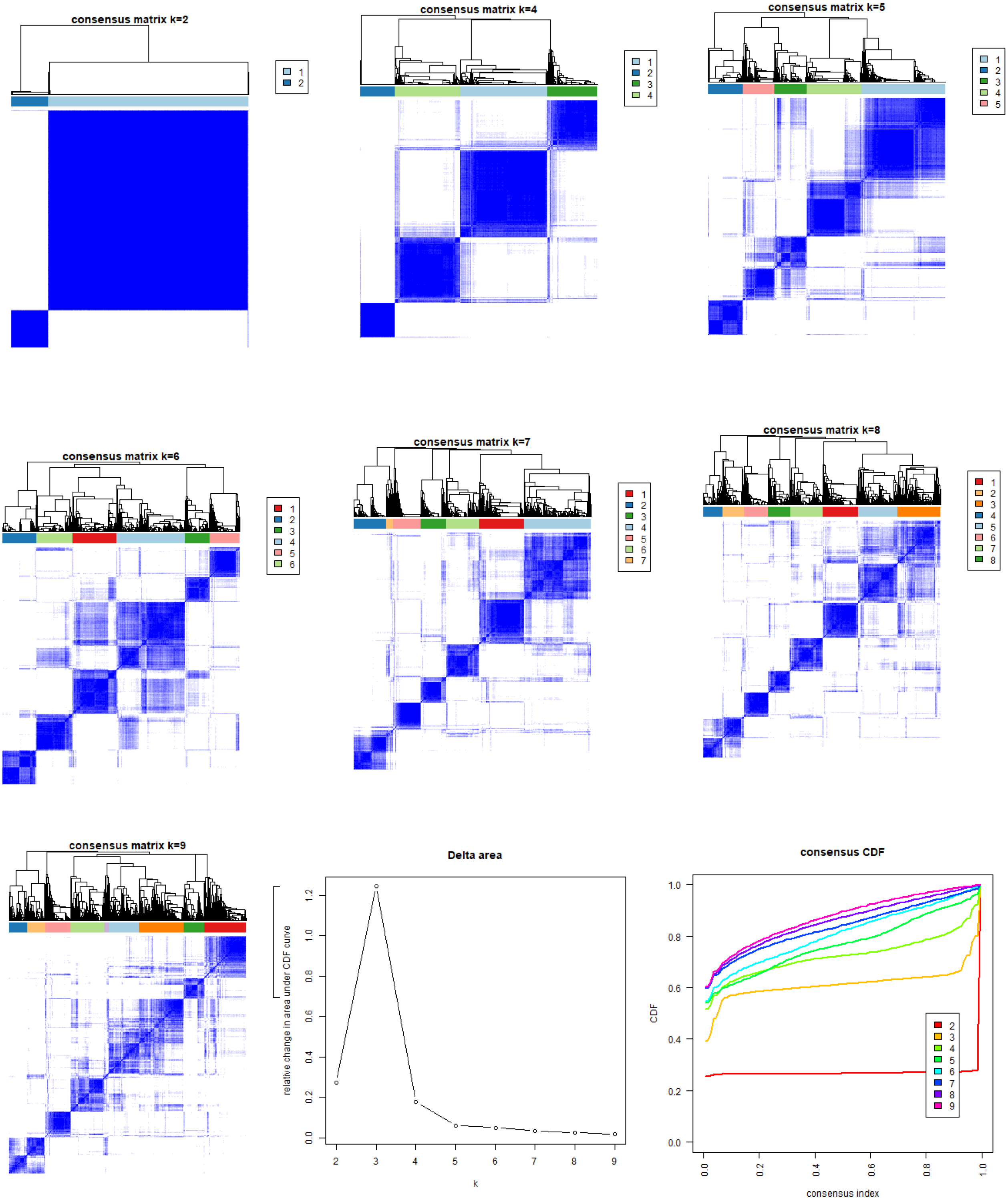

Supplementary Fig. S2 Unsupervised clustering of cuproptosis cluster and consensus matrix heatmaps for k = 2, 4-9.

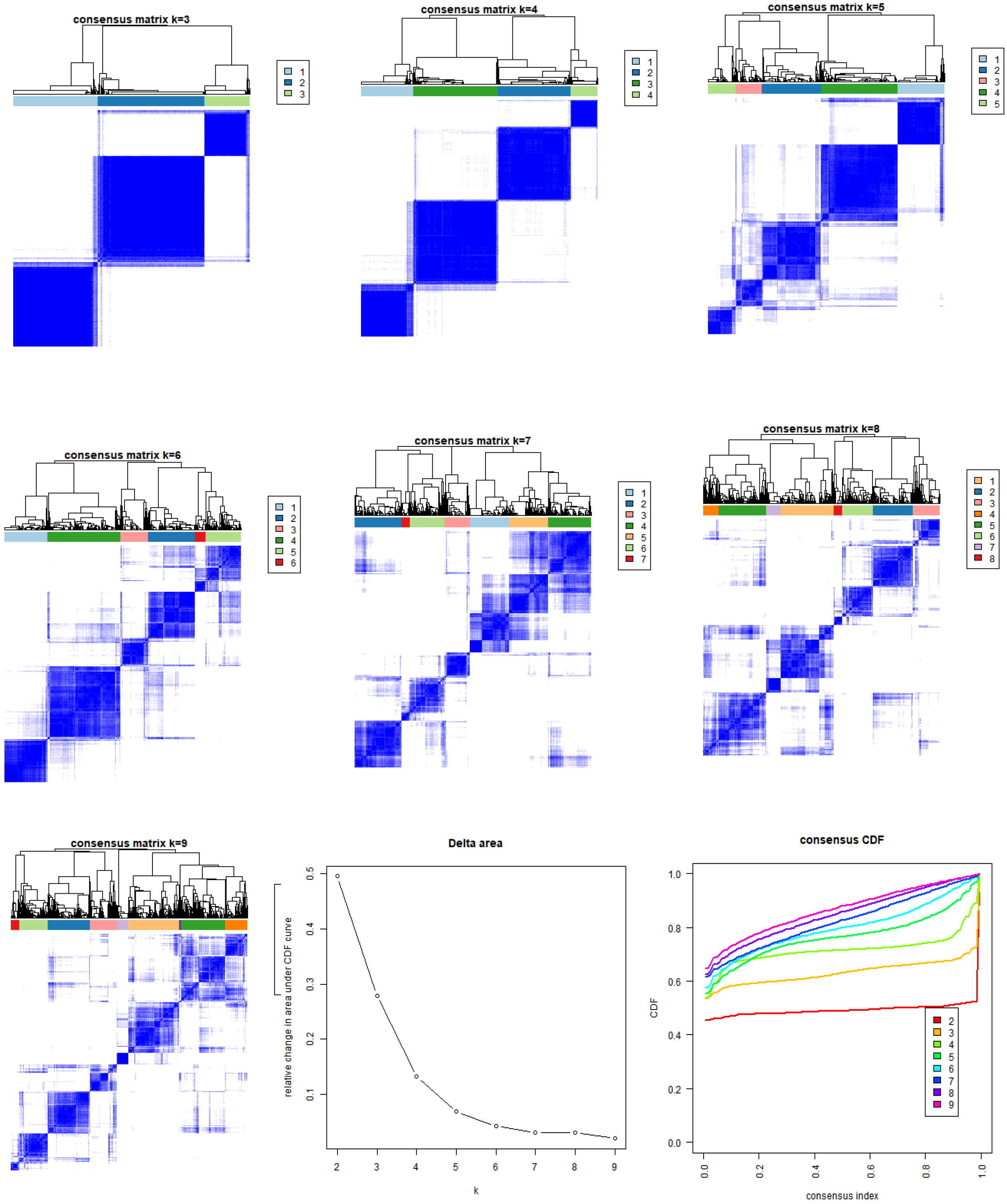

Supplementary Fig. S3 Unsupervised clustering of cuproptosis-related gene cluster and Consensus matrix heatmaps for k = 3-9.

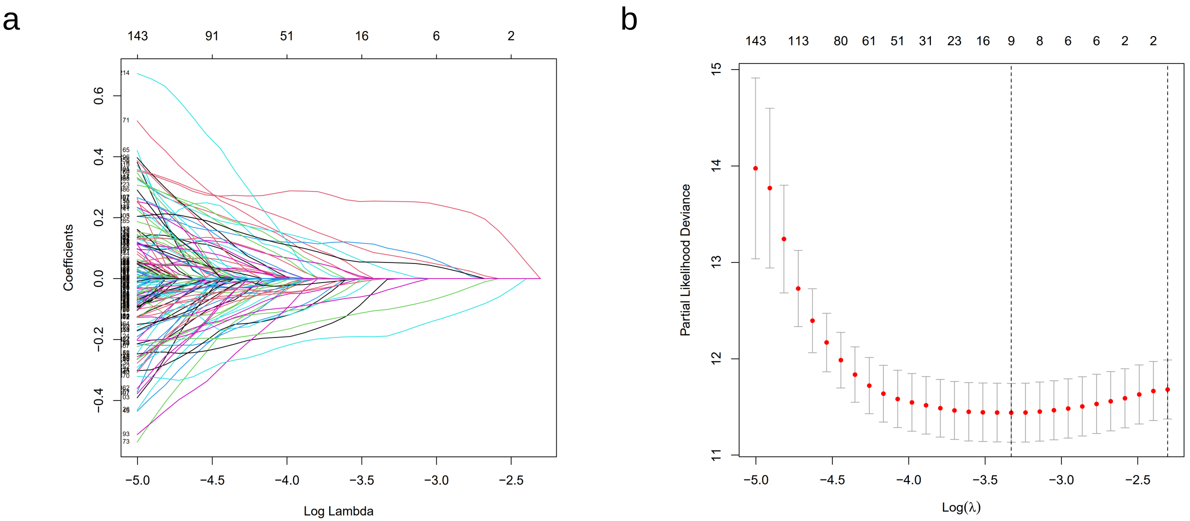

Supplementary Fig. S4 The identification of candidate prognostic genes by LASSO regression analysis. (a-b) The LASSO regression analysis and partial likelihood deviance on the prognostic genes.

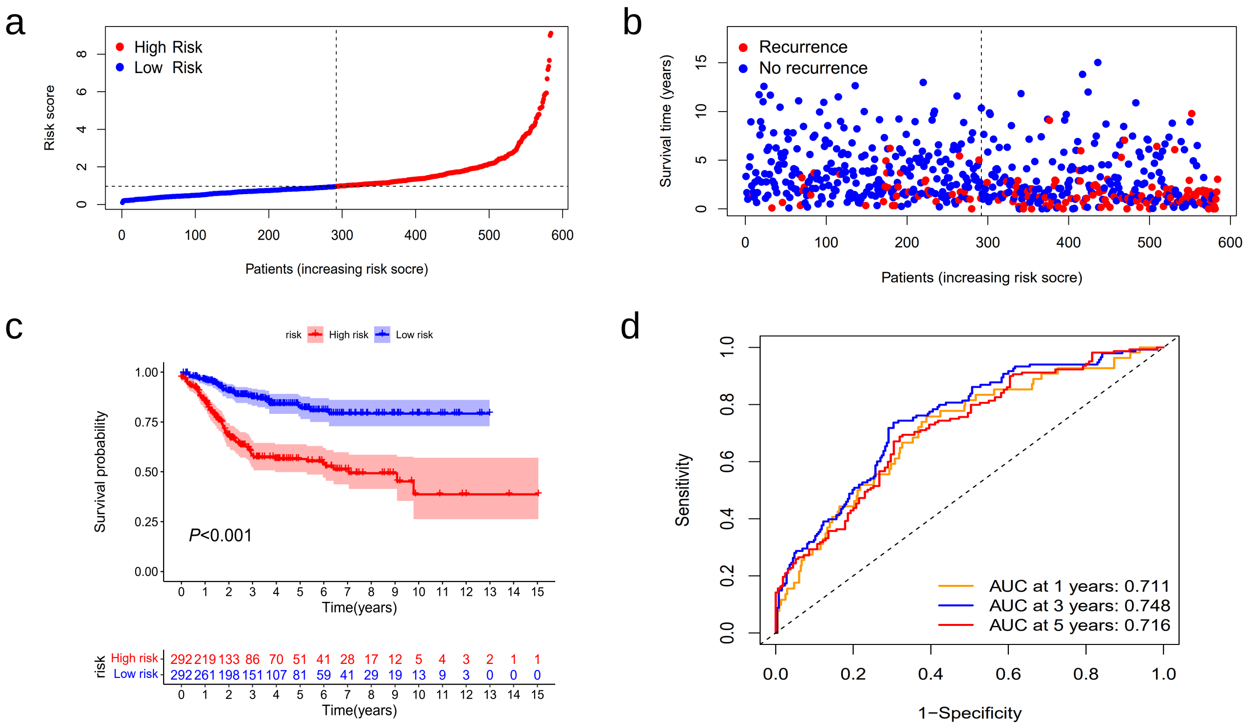

Supplementary Fig. S5 Construction of CRG_score model in the training dataset. (a) Distribution of risk scores between the high-risk and low-risk subgroups. (b) Survival overview in two high-risk and low-risk groups. (c) Comparison of DFS between the high-risk score and low-risk score groups. (d) ROC curves of the CRG_score to demonstrate the sensitivity and specificity in predicting the DFS of CRC patients. CRC, colorectal cancer; CRG, curproptosis related genes; DFS, disease-free survival.

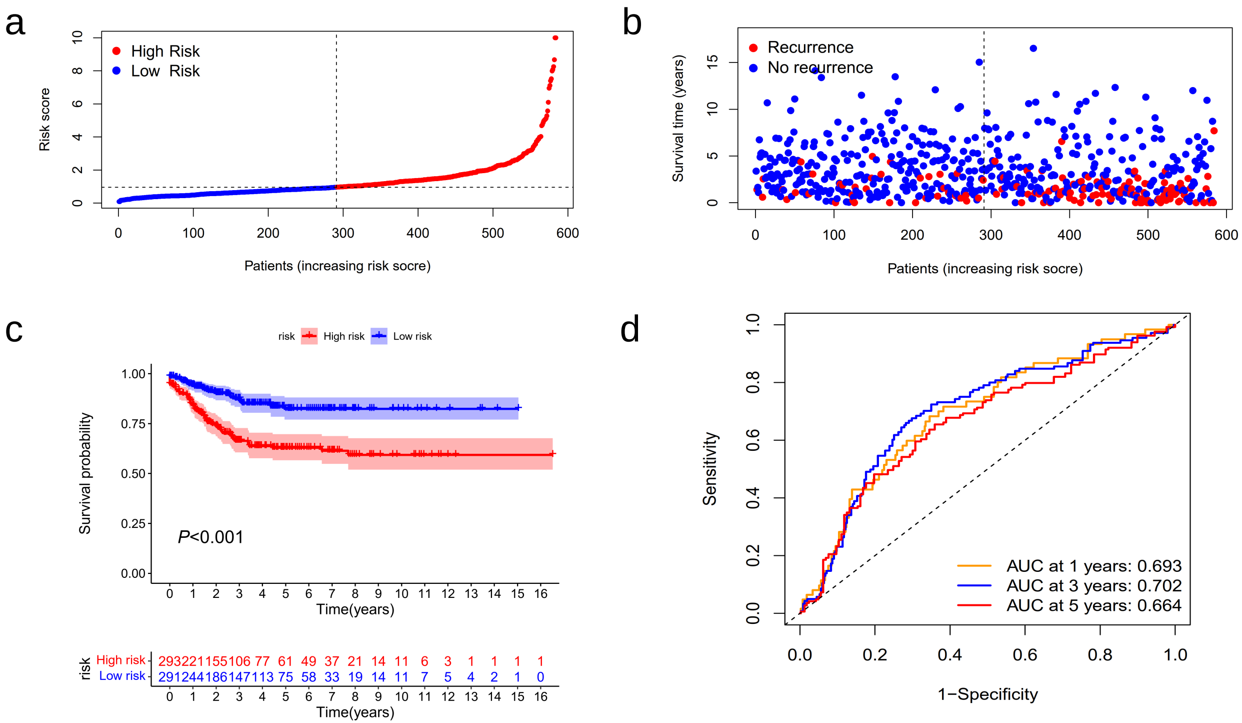

Supplementary Fig. S6 Validation of CRG_score model in the testing dataset. (a) Distribution of risk scores between the high-risk and low-risk subgroups. (b) Survival overview in two high-risk and low-risk groups. (c) Comparison of DFS between the high-risk score and low-risk score groups. (d) ROC curves of the CRG_score to demonstrate the sensitivity and specificity in predicting the DFS of CRC patients. CRC, colorectal cancer; CRG, curproptosis related genes; DFS, disease-free survival.

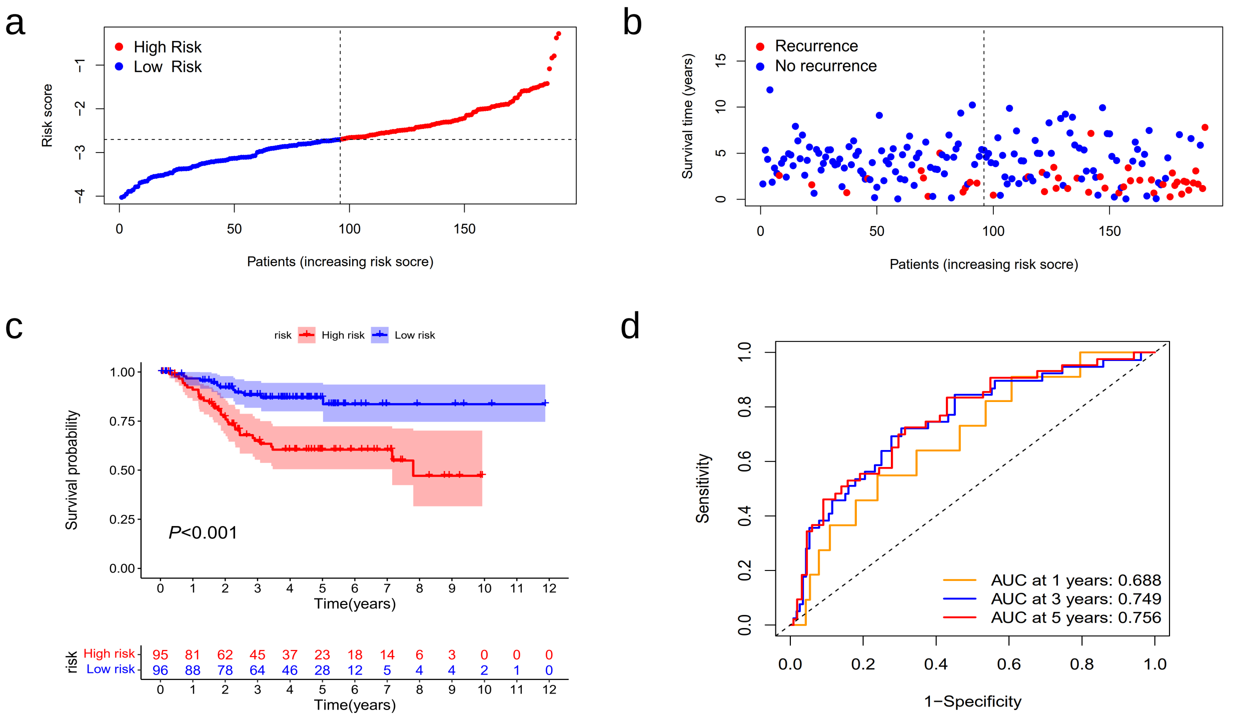

Supplementary Fig. S7 Validation of CRG_score model in the GSE161158 dataset. (a) Distribution of risk scores between the high-risk and low-risk subgroups. (b) Survival overview in two high-risk and low-risk groups. (c) Comparison of DFS between the high-risk score and low-risk score groups. (d) ROC curves of the CRG_score to demonstrate the sensitivity and specificity in predicting the DFS of glioma patients. CRC, colorectal cancer; CRG, curproptosis related genes; DFS, disease-free survival.

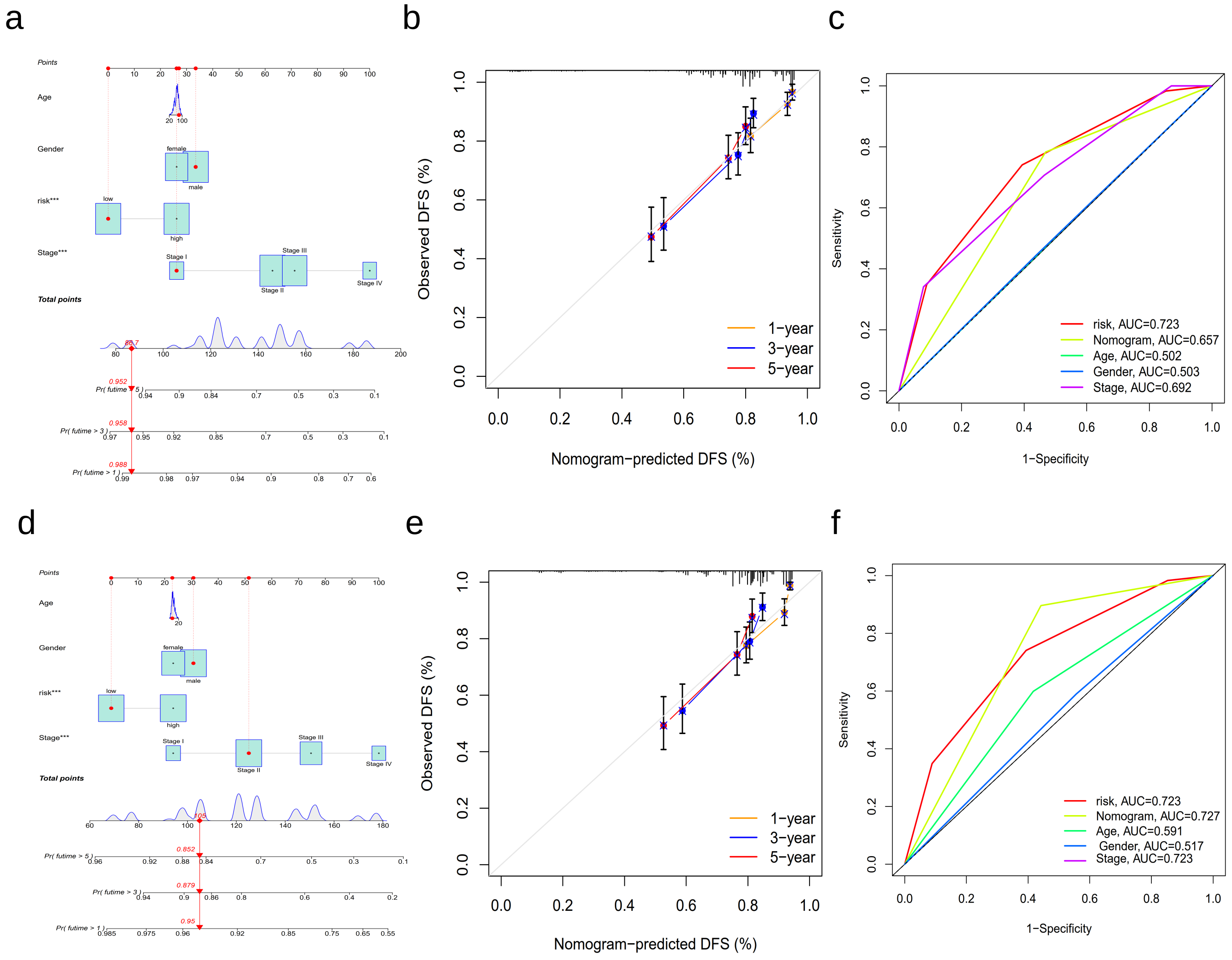

Supplementary Fig. S8 Development and evaluation of a prognostic nomogram in the training and testing sets. Nomogram for predicting the 1-, 3-, and 5-year DFS of CRC patients in the training (a) and testing (d) sets. Calibration curves of the nomogram for predicting of 1-, 3-, and 5-year DFS of CRC patients in the training (b) and testing (e) sets. Comparison of ROC curves between the Nomogram and other clinical characteristics in predicting the DFS of CRC patients in the training (c) and testing (f) sets. CRC, colorectal cancer; DFS, disease-free survival.

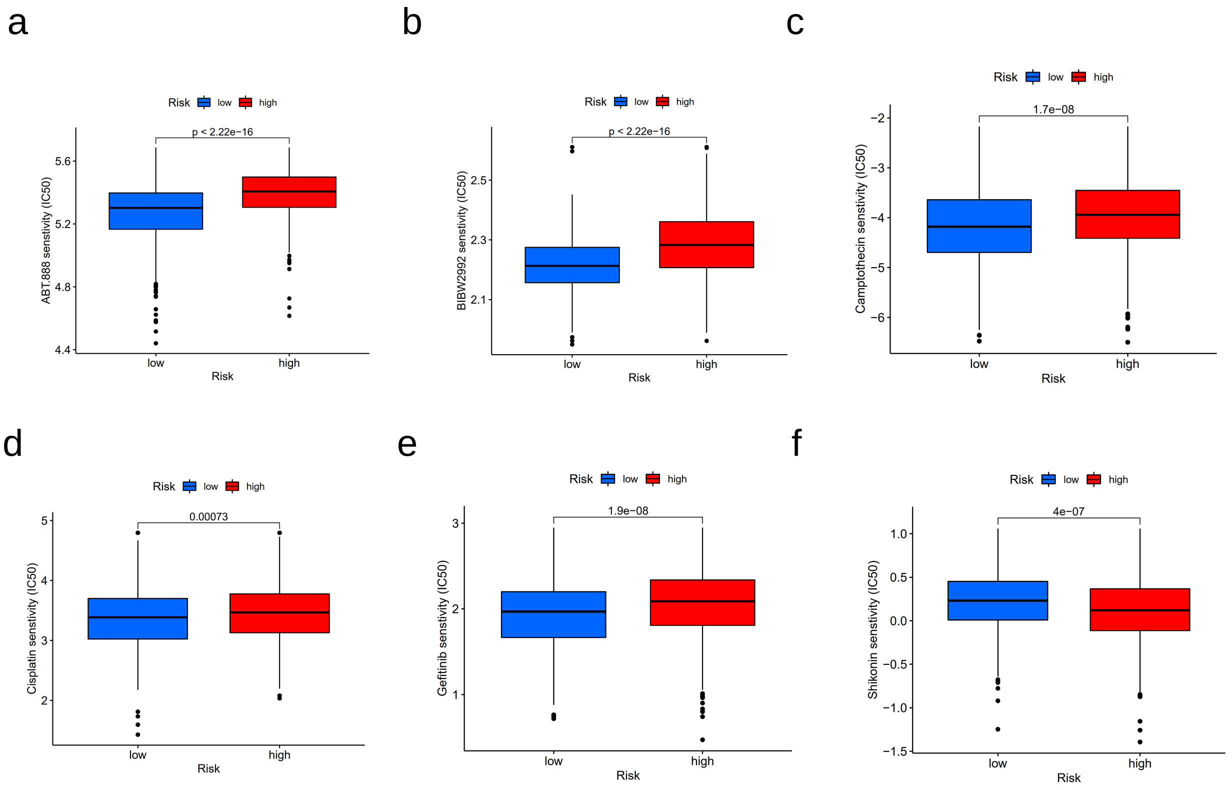

Supplementary Fig. S9 Relationships between CRG_score and chemotherapeutic sensitivity. CRG, curproptosis related genes
